# Supplementary material for: Comparative Genomics Reveal the Utilization Ability of Variable Carbohydrates as Key Genetic Features of Listeria Pathogens in Their Pathogenic Lifestyles
Source: Pathogens. 2022 Nov 28;11(12):1430. doi: 10.3390/pathogens11121430 (PMC9784484; doi:10.3390/pathogens11121430)
Supplement: Supplementary file 1 [file Supplymentary Tables S2.pdf › pathogens-1990707-supplementary/Supplymentary Tables S2.pdf]

Table S2. The ratio of homologous between genomes

Strain A	Strain B	Percentage of homologous genes
Listeria_welshimeri_1643	Listeria_grayi_1641	0.7021
Listeria_welshimeri_serovar_6b_str_SLCC5334_386043	Listeria_grayi_1641	0.7019
Listeria_seeligeri_serovar_1_2b_str_SLCC3954_683837	Listeria_grayi_1641	0.7092
Listeria_seeligeri_FSL_S4-171_702452	Listeria_grayi_1641	0.6579
Listeria_seeligeri_FSL_N1-067_702453	Listeria_grayi_1641	0.6717
Listeria_seeligeri_1640_v1	Listeria_grayi_1641	0.7081
Listeria_seeligeri_1640	Listeria_grayi_1641	0.6948
Listeria_monocytogenes_WSLC1042_1457188	Listeria_grayi_1641	0.7077
Listeria_monocytogenes_WSLC1001_1457187	Listeria_grayi_1641	0.7108
Listeria_monocytogenes_SLCC7179_879090	Listeria_grayi_1641	0.7125
Listeria_monocytogenes_SLCC5850_882096	Listeria_grayi_1641	0.7138
Listeria_monocytogenes_SLCC2755_932919	Listeria_grayi_1641	0.7053
Listeria_monocytogenes_SLCC2540_879089	Listeria_grayi_1641	0.7087
Listeria_monocytogenes_SLCC2479_882020	Listeria_grayi_1641	0.7032
Listeria_monocytogenes_SLCC2378_879088	Listeria_grayi_1641	0.7088
Listeria_monocytogenes_SLCC2376_882097	Listeria_grayi_1641	0.7212
Listeria_monocytogenes_SLCC2372_932920	Listeria_grayi_1641	0.7017
Listeria_monocytogenes_serotype_4b_str_LL195_1230340	Listeria_grayi_1641	0.7121
Listeria_monocytogenes_serotype_4b_str_LIS0087_1134669	Listeria_grayi_1641	0.6927
Listeria_monocytogenes_serotype_4b_str_F2365_265669	Listeria_grayi_1641	0.7121
Listeria_monocytogenes_serotype_4b_str_CLIP_80459_568819	Listeria_grayi_1641	0.7185
Listeria_monocytogenes_serotype_4b_str_81-0861_1196161	Listeria_grayi_1641	0.7005
Listeria_monocytogenes_serotype_4b_str_81-0592_1196160	Listeria_grayi_1641	0.7014
Listeria_monocytogenes_serotype_4b_str_81-0558_1196159	Listeria_grayi_1641	0.702
Listeria_monocytogenes_serotype_4b_str_10-0809_1196162	Listeria_grayi_1641	0.7014
Listeria_monocytogenes_serotype_4b_str_02-6680_1196175	Listeria_grayi_1641	0.7128
Listeria_monocytogenes_serotype_4b_str_02-6679_1196174	Listeria_grayi_1641	0.7128
Listeria_monocytogenes_serotype_4b_str_02-1792_1196173	Listeria_grayi_1641	0.7025
Listeria_monocytogenes_serotype_4b_str_02-1289_1196172	Listeria_grayi_1641	0.7025
Listeria_monocytogenes_serotype_4b_str_02-1103_1196171	Listeria_grayi_1641	0.7025
Listeria_monocytogenes_serotype_3c_str_10-5027_1196195	Listeria_grayi_1641	0.7027
Listeria_monocytogenes_serotype_1_2c_str_10-5026_1196194	Listeria_grayi_1641	0.7035
Listeria_monocytogenes_serotype_1_2c_str_10-5025_1196193	Listeria_grayi_1641	0.7035
Listeria_monocytogenes_serotype_1_2b_str_10-0811_1196164	Listeria_grayi_1641	0.7002
Listeria_monocytogenes_serotype_1_2b_str_10-0810_1196163	Listeria_grayi_1641	0.7002
Listeria_monocytogenes_serotype_1_2a_str_99-6370_1196179	Listeria_grayi_1641	0.6952
Listeria_monocytogenes_serotype_1_2a_str_98-2035_1196178	Listeria_grayi_1641	0.6952
Listeria_monocytogenes_serotype_1_2a_str_95-0093_1196177	Listeria_grayi_1641	0.6994
Listeria_monocytogenes_serotype_1_2a_str_88-0478_1196176	Listeria_grayi_1641	0.6984
Listeria_monocytogenes_serotype_1_2a_str_10-5024_1196192	Listeria_grayi_1641	0.6997
Listeria_monocytogenes_serotype_1_2a_str_10-4758_1196168	Listeria_grayi_1641	0.7131
Listeria_monocytogenes_serotype_1_2a_str_10-4754_1196167	Listeria_grayi_1641	0.7131
Listeria_monocytogenes_serotype_1_2a_str_10-1321_1196191	Listeria_grayi_1641	0.6997
Listeria_monocytogenes_serotype_1_2a_str_10-1047_1196190	Listeria_grayi_1641	0.7
Listeria_monocytogenes_serotype_1_2a_str_10-1046_1196189	Listeria_grayi_1641	0.7
Listeria_monocytogenes_serotype_1_2a_str_10-0934_1196170	Listeria_grayi_1641	0.7208
Listeria_monocytogenes_serotype_1_2a_str_10-0933_1196169	Listeria_grayi_1641	0.7208
Listeria_monocytogenes_serotype_1_2a_str_10-0815_1196188	Listeria_grayi_1641	0.6963
Listeria_monocytogenes_serotype_1_2a_str_10-0814_1196187	Listeria_grayi_1641	0.6998
Listeria_monocytogenes_serotype_1_2a_str_10-0813_1196166	Listeria_grayi_1641	0.7075
Listeria_monocytogenes_serotype_1_2a_str_10-0812_1196165	Listeria_grayi_1641	0.7075
Listeria_monocytogenes_serotype_1_2a_str_08-7669_1196186	Listeria_grayi_1641	0.7067
Listeria_monocytogenes_serotype_1_2a_str_08-7374_1196185	Listeria_grayi_1641	0.6997
Listeria_monocytogenes_serotype_1_2a_str_08-6997_1196184	Listeria_grayi_1641	0.6963
Listeria_monocytogenes_serotype_1_2a_str_08-6569_1196183	Listeria_grayi_1641	0.6963
Listeria_monocytogenes_serotype_1_2a_str_08-6056_1196182	Listeria_grayi_1641	0.6963
Listeria_monocytogenes_serotype_1_2a_str_04-5457_1196181	Listeria_grayi_1641	0.6997
Listeria_monocytogenes_serotype_1_2a_str_02-5993_1196180	Listeria_grayi_1641	0.6994
Listeria_monocytogenes_R479a_1437838	Listeria_grayi_1641	0.7071
Listeria_monocytogenes_N53-1_1234142	Listeria_grayi_1641	0.6811
Listeria_monocytogenes_M7_1030009	Listeria_grayi_1641	0.6933
Listeria_monocytogenes_LO28_393132	Listeria_grayi_1641	0.7021
Listeria_monocytogenes_L99_563174	Listeria_grayi_1641	0.6938
Listeria_monocytogenes_L312_882094	Listeria_grayi_1641	0.7185
Listeria_monocytogenes_J1816_930781	Listeria_grayi_1641	0.6987
Listeria_monocytogenes_J1-220_930782	Listeria_grayi_1641	0.698
Listeria_monocytogenes_J0161_393130	Listeria_grayi_1641	0.7012
Listeria_monocytogenes_HCC23_552536	Listeria_grayi_1641	0.6935
Listeria_monocytogenes_FSL_R2-561_393126	Listeria_grayi_1641	0.702
Listeria_monocytogenes_FSL_J1-208_393119	Listeria_grayi_1641	0.7082
Listeria_monocytogenes_FSL_J1-175_393118	Listeria_grayi_1641	0.7076
Listeria_monocytogenes_Finland_1998_393127	Listeria_grayi_1641	0.7167
Listeria_monocytogenes_EGD-e_169963_v1	Listeria_grayi_1641	0.7096
Listeria_monocytogenes_EGD-e_169963	Listeria_grayi_1641	0.71
Listeria_monocytogenes_EGD_1334565	Listeria_grayi_1641	0.7145
Listeria_monocytogenes_CFSAN002349_1299895	Listeria_grayi_1641	0.7147
Listeria_monocytogenes_ATCC_19117_882095_v1	Listeria_grayi_1641	0.7124
Listeria_monocytogenes_ATCC_19117_882095	Listeria_grayi_1641	0.7123
Listeria_monocytogenes_6179_1288295	Listeria_grayi_1641	0.6994
Listeria_monocytogenes_1639_v9	Listeria_grayi_1641	0.6861
Listeria_monocytogenes_1639_v99	Listeria_grayi_1641	0.7047
Listeria_monocytogenes_1639_v98	Listeria_grayi_1641	0.704
Listeria_monocytogenes_1639_v97	Listeria_grayi_1641	0.705
Listeria_monocytogenes_1639_v96	Listeria_grayi_1641	0.7022
Listeria_monocytogenes_1639_v95	Listeria_grayi_1641	0.7023
Listeria_monocytogenes_1639_v94	Listeria_grayi_1641	0.7025
Listeria_monocytogenes_1639_v93	Listeria_grayi_1641	0.7066
Listeria_monocytogenes_1639_v92	Listeria_grayi_1641	0.7036

Listeria_monocytogenes_1639_v91	Listeria_grayi_1641	0.6991
Listeria_monocytogenes_1639_v90	Listeria_grayi_1641	0.704
Listeria_monocytogenes_1639_v8	Listeria_grayi_1641	0.7012
Listeria_monocytogenes_1639_v89	Listeria_grayi_1641	0.7092
Listeria_monocytogenes_1639_v88	Listeria_grayi_1641	0.7071
Listeria_monocytogenes_1639_v87	Listeria_grayi_1641	0.7043
Listeria_monocytogenes_1639_v86	Listeria_grayi_1641	0.704
Listeria_monocytogenes_1639_v85	Listeria_grayi_1641	0.7004
Listeria_monocytogenes_1639_v84	Listeria_grayi_1641	0.7083
Listeria_monocytogenes_1639_v83	Listeria_grayi_1641	0.7045
Listeria_monocytogenes_1639_v82	Listeria_grayi_1641	0.7039
Listeria_monocytogenes_1639_v81	Listeria_grayi_1641	0.703
Listeria_monocytogenes_1639_v80	Listeria_grayi_1641	0.7067
Listeria_monocytogenes_1639_v7	Listeria_grayi_1641	0.7012
Listeria_monocytogenes_1639_v79	Listeria_grayi_1641	0.7014
Listeria_monocytogenes_1639_v78	Listeria_grayi_1641	0.7033
Listeria_monocytogenes_1639_v77	Listeria_grayi_1641	0.7056
Listeria_monocytogenes_1639_v76	Listeria_grayi_1641	0.7001
Listeria_monocytogenes_1639_v75	Listeria_grayi_1641	0.6938
Listeria_monocytogenes_1639_v74	Listeria_grayi_1641	0.7225
Listeria_monocytogenes_1639_v73	Listeria_grayi_1641	0.7014
Listeria_monocytogenes_1639_v72	Listeria_grayi_1641	0.7026
Listeria_monocytogenes_1639_v71	Listeria_grayi_1641	0.713
Listeria_monocytogenes_1639_v70	Listeria_grayi_1641	0.702
Listeria_monocytogenes_1639_v6	Listeria_grayi_1641	0.7007
Listeria_monocytogenes_1639_v69	Listeria_grayi_1641	0.7017
Listeria_monocytogenes_1639_v68	Listeria_grayi_1641	0.7061
Listeria_monocytogenes_1639_v67	Listeria_grayi_1641	0.7011
Listeria_monocytogenes_1639_v66	Listeria_grayi_1641	0.7091
Listeria_monocytogenes_1639_v65	Listeria_grayi_1641	0.7007
Listeria_monocytogenes_1639_v64	Listeria_grayi_1641	0.7055
Listeria_monocytogenes_1639_v63	Listeria_grayi_1641	0.708
Listeria_monocytogenes_1639_v62	Listeria_grayi_1641	0.6989
Listeria_monocytogenes_1639_v61	Listeria_grayi_1641	0.701
Listeria_monocytogenes_1639_v60	Listeria_grayi_1641	0.7017
Listeria_monocytogenes_1639_v5	Listeria_grayi_1641	0.6952
Listeria_monocytogenes_1639_v59	Listeria_grayi_1641	0.705
Listeria_monocytogenes_1639_v58	Listeria_grayi_1641	0.7032
Listeria_monocytogenes_1639_v57	Listeria_grayi_1641	0.6976
Listeria_monocytogenes_1639_v56	Listeria_grayi_1641	0.7044
Listeria_monocytogenes_1639_v55	Listeria_grayi_1641	0.6991
Listeria_monocytogenes_1639_v54	Listeria_grayi_1641	0.7086
Listeria_monocytogenes_1639_v53	Listeria_grayi_1641	0.7065
Listeria_monocytogenes_1639_v52	Listeria_grayi_1641	0.7011
Listeria_monocytogenes_1639_v51	Listeria_grayi_1641	0.7022
Listeria_monocytogenes_1639_v50	Listeria_grayi_1641	0.6989
Listeria_monocytogenes_1639_v4	Listeria_grayi_1641	0.6887
Listeria_monocytogenes_1639_v49	Listeria_grayi_1641	0.7015
Listeria_monocytogenes_1639_v48	Listeria_grayi_1641	0.6996
Listeria_monocytogenes_1639_v47	Listeria_grayi_1641	0.6994
Listeria_monocytogenes_1639_v46	Listeria_grayi_1641	0.7051
Listeria_monocytogenes_1639_v45	Listeria_grayi_1641	0.7015
Listeria_monocytogenes_1639_v44	Listeria_grayi_1641	0.6997
Listeria_monocytogenes_1639_v43	Listeria_grayi_1641	0.6997
Listeria_monocytogenes_1639_v42	Listeria_grayi_1641	0.6953
Listeria_monocytogenes_1639_v41	Listeria_grayi_1641	0.6928
Listeria_monocytogenes_1639_v40	Listeria_grayi_1641	0.7051
Listeria_monocytogenes_1639_v3	Listeria_grayi_1641	0.689
Listeria_monocytogenes_1639_v39	Listeria_grayi_1641	0.6992
Listeria_monocytogenes_1639_v38	Listeria_grayi_1641	0.7002
Listeria_monocytogenes_1639_v37	Listeria_grayi_1641	0.6967
Listeria_monocytogenes_1639_v36	Listeria_grayi_1641	0.6997
Listeria_monocytogenes_1639_v35	Listeria_grayi_1641	0.7044
Listeria_monocytogenes_1639_v34	Listeria_grayi_1641	0.6997
Listeria_monocytogenes_1639_v33	Listeria_grayi_1641	0.7002
Listeria_monocytogenes_1639_v32	Listeria_grayi_1641	0.6955
Listeria_monocytogenes_1639_v31	Listeria_grayi_1641	0.7001
Listeria_monocytogenes_1639_v30	Listeria_grayi_1641	0.7023
Listeria_monocytogenes_1639_v2	Listeria_grayi_1641	0.6926
Listeria_monocytogenes_1639_v29	Listeria_grayi_1641	0.6949
Listeria_monocytogenes_1639_v28	Listeria_grayi_1641	0.6955
Listeria_monocytogenes_1639_v27	Listeria_grayi_1641	0.6927
Listeria_monocytogenes_1639_v26	Listeria_grayi_1641	0.6928
Listeria_monocytogenes_1639_v25	Listeria_grayi_1641	0.6925
Listeria_monocytogenes_1639_v24	Listeria_grayi_1641	0.6988
Listeria_monocytogenes_1639_v23	Listeria_grayi_1641	0.6988
Listeria_monocytogenes_1639_v22	Listeria_grayi_1641	0.7013
Listeria_monocytogenes_1639_v21	Listeria_grayi_1641	0.6981
Listeria_monocytogenes_1639_v20	Listeria_grayi_1641	0.697
Listeria_monocytogenes_1639_v1	Listeria_grayi_1641	0.6932
Listeria_monocytogenes_1639_v19	Listeria_grayi_1641	0.6967
Listeria_monocytogenes_1639_v198	Listeria_grayi_1641	0.6828
Listeria_monocytogenes_1639_v197	Listeria_grayi_1641	0.7002
Listeria_monocytogenes_1639_v196	Listeria_grayi_1641	0.6966
Listeria_monocytogenes_1639_v195	Listeria_grayi_1641	0.7225
Listeria_monocytogenes_1639_v194	Listeria_grayi_1641	0.722
Listeria_monocytogenes_1639_v193	Listeria_grayi_1641	0.7216
Listeria_monocytogenes_1639_v192	Listeria_grayi_1641	0.7027
Listeria_monocytogenes_1639_v191	Listeria_grayi_1641	0.7207
Listeria_monocytogenes_1639_v190	Listeria_grayi_1641	0.7219
Listeria_monocytogenes_1639_v18	Listeria_grayi_1641	0.6926

Listeria_monocytogenes_1639_v189	Listeria_grayi_1641	0.7216
Listeria_monocytogenes_1639_v188	Listeria_grayi_1641	0.7213
Listeria_monocytogenes_1639_v187	Listeria_grayi_1641	0.7219
Listeria_monocytogenes_1639_v186	Listeria_grayi_1641	0.7222
Listeria_monocytogenes_1639_v185	Listeria_grayi_1641	0.7219
Listeria_monocytogenes_1639_v184	Listeria_grayi_1641	0.7218
Listeria_monocytogenes_1639_v183	Listeria_grayi_1641	0.7183
Listeria_monocytogenes_1639_v182	Listeria_grayi_1641	0.7201
Listeria_monocytogenes_1639_v181	Listeria_grayi_1641	0.7067
Listeria_monocytogenes_1639_v180	Listeria_grayi_1641	0.7067
Listeria_monocytogenes_1639_v17	Listeria_grayi_1641	0.6926
Listeria_monocytogenes_1639_v179	Listeria_grayi_1641	0.7124
Listeria_monocytogenes_1639_v178	Listeria_grayi_1641	0.7187
Listeria_monocytogenes_1639_v177	Listeria_grayi_1641	0.713
Listeria_monocytogenes_1639_v176	Listeria_grayi_1641	0.7189
Listeria_monocytogenes_1639_v175	Listeria_grayi_1641	0.7118
Listeria_monocytogenes_1639_v174	Listeria_grayi_1641	0.7183
Listeria_monocytogenes_1639_v173	Listeria_grayi_1641	0.7163
Listeria_monocytogenes_1639_v172	Listeria_grayi_1641	0.7131
Listeria_monocytogenes_1639_v171	Listeria_grayi_1641	0.715
Listeria_monocytogenes_1639_v170	Listeria_grayi_1641	0.7172
Listeria_monocytogenes_1639_v16	Listeria_grayi_1641	0.6983
Listeria_monocytogenes_1639_v169	Listeria_grayi_1641	0.7151
Listeria_monocytogenes_1639_v168	Listeria_grayi_1641	0.7191
Listeria_monocytogenes_1639_v167	Listeria_grayi_1641	0.7151
Listeria_monocytogenes_1639_v166	Listeria_grayi_1641	0.7193
Listeria_monocytogenes_1639_v165	Listeria_grayi_1641	0.7149
Listeria_monocytogenes_1639_v164	Listeria_grayi_1641	0.7129
Listeria_monocytogenes_1639_v163	Listeria_grayi_1641	0.7181
Listeria_monocytogenes_1639_v162	Listeria_grayi_1641	0.7129
Listeria_monocytogenes_1639_v161	Listeria_grayi_1641	0.7122
Listeria_monocytogenes_1639_v160	Listeria_grayi_1641	0.7123
Listeria_monocytogenes_1639_v15	Listeria_grayi_1641	0.6938
Listeria_monocytogenes_1639_v159	Listeria_grayi_1641	0.7134
Listeria_monocytogenes_1639_v158	Listeria_grayi_1641	0.7121
Listeria_monocytogenes_1639_v157	Listeria_grayi_1641	0.7121
Listeria_monocytogenes_1639_v156	Listeria_grayi_1641	0.7242
Listeria_monocytogenes_1639_v155	Listeria_grayi_1641	0.7126
Listeria_monocytogenes_1639_v154	Listeria_grayi_1641	0.7125
Listeria_monocytogenes_1639_v153	Listeria_grayi_1641	0.7129
Listeria_monocytogenes_1639_v152	Listeria_grayi_1641	0.7137
Listeria_monocytogenes_1639_v151	Listeria_grayi_1641	0.7137
Listeria_monocytogenes_1639_v150	Listeria_grayi_1641	0.7154
Listeria_monocytogenes_1639_v14	Listeria_grayi_1641	0.7014
Listeria_monocytogenes_1639_v149	Listeria_grayi_1641	0.7145
Listeria_monocytogenes_1639_v148	Listeria_grayi_1641	0.7125
Listeria_monocytogenes_1639_v147	Listeria_grayi_1641	0.7212
Listeria_monocytogenes_1639_v146	Listeria_grayi_1641	0.7164
Listeria_monocytogenes_1639_v145	Listeria_grayi_1641	0.7167
Listeria_monocytogenes_1639_v144	Listeria_grayi_1641	0.7182
Listeria_monocytogenes_1639_v143	Listeria_grayi_1641	0.7163
Listeria_monocytogenes_1639_v142	Listeria_grayi_1641	0.7182
Listeria_monocytogenes_1639_v141	Listeria_grayi_1641	0.7166
Listeria_monocytogenes_1639_v140	Listeria_grayi_1641	0.7185
Listeria_monocytogenes_1639_v13	Listeria_grayi_1641	0.6941
Listeria_monocytogenes_1639_v139	Listeria_grayi_1641	0.7122
Listeria_monocytogenes_1639_v138	Listeria_grayi_1641	0.7183
Listeria_monocytogenes_1639_v137	Listeria_grayi_1641	0.7179
Listeria_monocytogenes_1639_v136	Listeria_grayi_1641	0.7113
Listeria_monocytogenes_1639_v135	Listeria_grayi_1641	0.7026
Listeria_monocytogenes_1639_v134	Listeria_grayi_1641	0.7084
Listeria_monocytogenes_1639_v133	Listeria_grayi_1641	0.7114
Listeria_monocytogenes_1639_v132	Listeria_grayi_1641	0.7019
Listeria_monocytogenes_1639_v131	Listeria_grayi_1641	0.706
Listeria_monocytogenes_1639_v130	Listeria_grayi_1641	0.7075
Listeria_monocytogenes_1639_v12	Listeria_grayi_1641	0.6906
Listeria_monocytogenes_1639_v129	Listeria_grayi_1641	0.7174
Listeria_monocytogenes_1639_v128	Listeria_grayi_1641	0.7139
Listeria_monocytogenes_1639_v127	Listeria_grayi_1641	0.7088
Listeria_monocytogenes_1639_v126	Listeria_grayi_1641	0.7135
Listeria_monocytogenes_1639_v125	Listeria_grayi_1641	0.713
Listeria_monocytogenes_1639_v124	Listeria_grayi_1641	0.706
Listeria_monocytogenes_1639_v123	Listeria_grayi_1641	0.7147
Listeria_monocytogenes_1639_v122	Listeria_grayi_1641	0.715
Listeria_monocytogenes_1639_v121	Listeria_grayi_1641	0.715
Listeria_monocytogenes_1639_v120	Listeria_grayi_1641	0.7147
Listeria_monocytogenes_1639_v11	Listeria_grayi_1641	0.6952
Listeria_monocytogenes_1639_v119	Listeria_grayi_1641	0.715
Listeria_monocytogenes_1639_v118	Listeria_grayi_1641	0.715
Listeria_monocytogenes_1639_v117	Listeria_grayi_1641	0.7092
Listeria_monocytogenes_1639_v116	Listeria_grayi_1641	0.7083
Listeria_monocytogenes_1639_v115	Listeria_grayi_1641	0.7085
Listeria_monocytogenes_1639_v114	Listeria_grayi_1641	0.7083
Listeria_monocytogenes_1639_v113	Listeria_grayi_1641	0.7134
Listeria_monocytogenes_1639_v112	Listeria_grayi_1641	0.7055
Listeria_monocytogenes_1639_v111	Listeria_grayi_1641	0.7085
Listeria_monocytogenes_1639_v110	Listeria_grayi_1641	0.7089
Listeria_monocytogenes_1639_v10	Listeria_grayi_1641	0.6895
Listeria_monocytogenes_1639_v109	Listeria_grayi_1641	0.7077
Listeria_monocytogenes_1639_v108	Listeria_grayi_1641	0.7067
Listeria_monocytogenes_1639_v107	Listeria_grayi_1641	0.7112

Listeria_monocytogenes_1639_v106	Listeria_grayi_1641	0.7116
Listeria_monocytogenes_1639_v105	Listeria_grayi_1641	0.7116
Listeria_monocytogenes_1639_v104	Listeria_grayi_1641	0.7118
Listeria_monocytogenes_1639_v103	Listeria_grayi_1641	0.7118
Listeria_monocytogenes_1639_v102	Listeria_grayi_1641	0.7059
Listeria_monocytogenes_1639_v101	Listeria_grayi_1641	0.7071
Listeria_monocytogenes_1639_v100	Listeria_grayi_1641	0.7066
Listeria_monocytogenes_1639	Listeria_grayi_1641	0.7216
Listeria_monocytogenes_10403S_393133	Listeria_grayi_1641	0.7159
Listeria_monocytogenes_08-5923_637381	Listeria_grayi_1641	0.6997
Listeria_monocytogenes_08-5578_653938	Listeria_grayi_1641	0.698
Listeria_monocytogenes_07PF0776_1126011	Listeria_grayi_1641	0.7114
Listeria_marthii_FSL_S4-120_702457	Listeria_grayi_1641	0.675
Listeria_marthii_529731_v1	Listeria_grayi_1641	0.6993
Listeria_marthii_529731	Listeria_grayi_1641	0.7076
Listeria_ivanovii_WSLC3009_1457190	Listeria_grayi_1641	0.7074
Listeria_ivanovii_subsp_londoniensis_202752_v2	Listeria_grayi_1641	0.7062
Listeria_ivanovii_subsp_londoniensis_202752_v1	Listeria_grayi_1641	0.6972
Listeria_ivanovii_subsp_londoniensis_202752	Listeria_grayi_1641	0.7017
Listeria_ivanovii_subsp_ivanovii_PAM_55_881621	Listeria_grayi_1641	0.7067
Listeria_ivanovii_subsp_ivanovii_202751_v2	Listeria_grayi_1641	0.708
Listeria_ivanovii_subsp_ivanovii_202751_v1	Listeria_grayi_1641	0.708
Listeria_ivanovii_subsp_ivanovii_202751	Listeria_grayi_1641	0.708
Listeria_ivanovii_FSL_F6-596_702454	Listeria_grayi_1641	0.6529
Listeria_ivanovii_1638	Listeria_grayi_1641	0.7077
Listeria_innocua_FSL_S4-378_702456	Listeria_grayi_1641	0.648
Listeria_innocua_FSL_J1-023_702455	Listeria_grayi_1641	0.6805
Listeria_innocua_Clip11262_272626	Listeria_grayi_1641	0.6909
Listeria_innocua_1642_v4	Listeria_grayi_1641	0.6922
Listeria_innocua_1642_v3	Listeria_grayi_1641	0.7061
Listeria_innocua_1642_v2	Listeria_grayi_1641	0.7071
Listeria_innocua_1642_v1	Listeria_grayi_1641	0.71
Listeria_innocua_1642	Listeria_grayi_1641	0.706
Listeria_grayi_DSM_20601_525367	Listeria_grayi_1641	1
Listeria_grayi_1641_v2	Listeria_grayi_1641	0.9062
Listeria_grayi_1641_v1	Listeria_grayi_1641	0.8932
Listeria_grayi_1641	Listeria_grayi_1641	0.0533
Listeria_welshimeri_1643	Listeria_grayi_1641_v1	0.6674
Listeria_welshimeri_serovar_6b_str_SLCC5334_386043	Listeria_grayi_1641_v1	0.6673
Listeria_seeligeri_serovar_1_2b_str_SLCC3954_683837	Listeria_grayi_1641_v1	0.6731
Listeria_seeligeri_FSL_S4-171_702452	Listeria_grayi_1641_v1	0.6219
Listeria_seeligeri_FSL_N1-067_702453	Listeria_grayi_1641_v1	0.6286
Listeria_seeligeri_1640_v1	Listeria_grayi_1641_v1	0.6705
Listeria_seeligeri_1640	Listeria_grayi_1641_v1	0.6609
Listeria_monocytogenes_WSLC1042_1457188	Listeria_grayi_1641_v1	0.675
Listeria_monocytogenes_WSLC1001_1457187	Listeria_grayi_1641_v1	0.6728
Listeria_monocytogenes_SLCC7179_879090	Listeria_grayi_1641_v1	0.6759
Listeria_monocytogenes_SLCC5850_882096	Listeria_grayi_1641_v1	0.6755
Listeria_monocytogenes_SLCC2755_932919	Listeria_grayi_1641_v1	0.6718
Listeria_monocytogenes_SLCC2540_879089	Listeria_grayi_1641_v1	0.6776
Listeria_monocytogenes_SLCC2479_882020	Listeria_grayi_1641_v1	0.6699
Listeria_monocytogenes_SLCC2378_879088	Listeria_grayi_1641_v1	0.6756
Listeria_monocytogenes_SLCC2376_882097	Listeria_grayi_1641_v1	0.6837
Listeria_monocytogenes_SLCC2372_932920	Listeria_grayi_1641_v1	0.6688
Listeria_monocytogenes_serotype_4b_str_LL195_1230340	Listeria_grayi_1641_v1	0.6773
Listeria_monocytogenes_serotype_4b_str_LIS0087_1134669	Listeria_grayi_1641_v1	0.6563
Listeria_monocytogenes_serotype_4b_str_F2365_265669	Listeria_grayi_1641_v1	0.6769
Listeria_monocytogenes_serotype_4b_str_CLIP_80459_568819	Listeria_grayi_1641_v1	0.6804
Listeria_monocytogenes_serotype_4b_str_81-0861_1196161	Listeria_grayi_1641_v1	0.6695
Listeria_monocytogenes_serotype_4b_str_81-0592_1196160	Listeria_grayi_1641_v1	0.6704
Listeria_monocytogenes_serotype_4b_str_81-0558_1196159	Listeria_grayi_1641_v1	0.6709
Listeria_monocytogenes_serotype_4b_str_10-0809_1196162	Listeria_grayi_1641_v1	0.6704
Listeria_monocytogenes_serotype_4b_str_02-6680_1196175	Listeria_grayi_1641_v1	0.6746
Listeria_monocytogenes_serotype_4b_str_02-6679_1196174	Listeria_grayi_1641_v1	0.6746
Listeria_monocytogenes_serotype_4b_str_02-1792_1196173	Listeria_grayi_1641_v1	0.6695
Listeria_monocytogenes_serotype_4b_str_02-1289_1196172	Listeria_grayi_1641_v1	0.6695
Listeria_monocytogenes_serotype_4b_str_02-1103_1196171	Listeria_grayi_1641_v1	0.6695
Listeria_monocytogenes_serotype_3c_str_10-5027_1196195	Listeria_grayi_1641_v1	0.6687
Listeria_monocytogenes_serotype_1_2c_str_10-5026_1196194	Listeria_grayi_1641_v1	0.6694
Listeria_monocytogenes_serotype_1_2c_str_10-5025_1196193	Listeria_grayi_1641_v1	0.6694
Listeria_monocytogenes_serotype_1_2b_str_10-0811_1196164	Listeria_grayi_1641_v1	0.6657
Listeria_monocytogenes_serotype_1_2b_str_10-0810_1196163	Listeria_grayi_1641_v1	0.6657
Listeria_monocytogenes_serotype_1_2a_str_99-6370_1196179	Listeria_grayi_1641_v1	0.6594
Listeria_monocytogenes_serotype_1_2a_str_98-2035_1196178	Listeria_grayi_1641_v1	0.6594
Listeria_monocytogenes_serotype_1_2a_str_95-0093_1196177	Listeria_grayi_1641_v1	0.6628
Listeria_monocytogenes_serotype_1_2a_str_88-0478_1196176	Listeria_grayi_1641_v1	0.6618
Listeria_monocytogenes_serotype_1_2a_str_10-5024_1196192	Listeria_grayi_1641_v1	0.6631
Listeria_monocytogenes_serotype_1_2a_str_10-4758_1196168	Listeria_grayi_1641_v1	0.6745
Listeria_monocytogenes_serotype_1_2a_str_10-4754_1196167	Listeria_grayi_1641_v1	0.6745
Listeria_monocytogenes_serotype_1_2a_str_10-1321_1196191	Listeria_grayi_1641_v1	0.6631
Listeria_monocytogenes_serotype_1_2a_str_10-1047_1196190	Listeria_grayi_1641_v1	0.6633
Listeria_monocytogenes_serotype_1_2a_str_10-1046_1196189	Listeria_grayi_1641_v1	0.6633
Listeria_monocytogenes_serotype_1_2a_str_10-0934_1196170	Listeria_grayi_1641_v1	0.6817
Listeria_monocytogenes_serotype_1_2a_str_10-0933_1196169	Listeria_grayi_1641_v1	0.6817
Listeria_monocytogenes_serotype_1_2a_str_10-0815_1196188	Listeria_grayi_1641_v1	0.66
Listeria_monocytogenes_serotype_1_2a_str_10-0814_1196187	Listeria_grayi_1641_v1	0.6632
Listeria_monocytogenes_serotype_1_2a_str_10-0813_1196166	Listeria_grayi_1641_v1	0.6713
Listeria_monocytogenes_serotype_1_2a_str_10-0812_1196165	Listeria_grayi_1641_v1	0.6713
Listeria_monocytogenes_serotype_1_2a_str_08-7669_1196186	Listeria_grayi_1641_v1	0.6694
Listeria_monocytogenes_serotype_1_2a_str_08-7374_1196185	Listeria_grayi_1641_v1	0.6631
Listeria_monocytogenes_serotype_1_2a_str_08-6997_1196184	Listeria_grayi_1641_v1	0.66

Listeria_monocytogenes_serotype_1_2a_str_08-6569_1196183	Listeria_grayi_1641_v1	0.66
Listeria_monocytogenes_serotype_1_2a_str_08-6056_1196182	Listeria_grayi_1641_v1	0.66
Listeria_monocytogenes_serotype_1_2a_str_04-5457_1196181	Listeria_grayi_1641_v1	0.6631
Listeria_monocytogenes_serotype_1_2a_str_02-5993_1196180	Listeria_grayi_1641_v1	0.6628
Listeria_monocytogenes_R479a_1437838	Listeria_grayi_1641_v1	0.666
Listeria_monocytogenes_N53-1_1234142	Listeria_grayi_1641_v1	0.6504
Listeria_monocytogenes_M7_1030009	Listeria_grayi_1641_v1	0.66
Listeria_monocytogenes_LO28_393132	Listeria_grayi_1641_v1	0.6678
Listeria_monocytogenes_L99_563174	Listeria_grayi_1641_v1	0.6605
Listeria_monocytogenes_L312_882094	Listeria_grayi_1641_v1	0.6804
Listeria_monocytogenes_J1816_930781	Listeria_grayi_1641_v1	0.6672
Listeria_monocytogenes_J1-220_930782	Listeria_grayi_1641_v1	0.6641
Listeria_monocytogenes_J0161_393130	Listeria_grayi_1641_v1	0.6669
Listeria_monocytogenes_HCC23_552536	Listeria_grayi_1641_v1	0.6603
Listeria_monocytogenes_FSL_R2-561_393126	Listeria_grayi_1641_v1	0.6684
Listeria_monocytogenes_FSL_J1-208_393119	Listeria_grayi_1641_v1	0.6686
Listeria_monocytogenes_FSL_J1-175_393118	Listeria_grayi_1641_v1	0.6737
Listeria_monocytogenes_Finland_1998_393127	Listeria_grayi_1641_v1	0.6789
Listeria_monocytogenes_EGD-e_169963_v1	Listeria_grayi_1641_v1	0.6757
Listeria_monocytogenes_EGD-e_169963	Listeria_grayi_1641_v1	0.6761
Listeria_monocytogenes_EGD_1334565	Listeria_grayi_1641_v1	0.6761
Listeria_monocytogenes_CFSAN002349_1299895	Listeria_grayi_1641_v1	0.6773
Listeria_monocytogenes_ATCC_19117_882095_v1	Listeria_grayi_1641_v1	0.6766
Listeria_monocytogenes_ATCC_19117_882095	Listeria_grayi_1641_v1	0.6765
Listeria_monocytogenes_6179_1288295	Listeria_grayi_1641_v1	0.6627
Listeria_monocytogenes_1639_v9	Listeria_grayi_1641_v1	0.6549
Listeria_monocytogenes_1639_v99	Listeria_grayi_1641_v1	0.6684
Listeria_monocytogenes_1639_v98	Listeria_grayi_1641_v1	0.668
Listeria_monocytogenes_1639_v97	Listeria_grayi_1641_v1	0.6699
Listeria_monocytogenes_1639_v96	Listeria_grayi_1641_v1	0.6672
Listeria_monocytogenes_1639_v95	Listeria_grayi_1641_v1	0.6677
Listeria_monocytogenes_1639_v94	Listeria_grayi_1641_v1	0.6675
Listeria_monocytogenes_1639_v93	Listeria_grayi_1641_v1	0.6693
Listeria_monocytogenes_1639_v92	Listeria_grayi_1641_v1	0.6678
Listeria_monocytogenes_1639_v91	Listeria_grayi_1641_v1	0.6653
Listeria_monocytogenes_1639_v90	Listeria_grayi_1641_v1	0.668
Listeria_monocytogenes_1639_v8	Listeria_grayi_1641_v1	0.6631
Listeria_monocytogenes_1639_v89	Listeria_grayi_1641_v1	0.6742
Listeria_monocytogenes_1639_v88	Listeria_grayi_1641_v1	0.6717
Listeria_monocytogenes_1639_v87	Listeria_grayi_1641_v1	0.6677
Listeria_monocytogenes_1639_v86	Listeria_grayi_1641_v1	0.6674
Listeria_monocytogenes_1639_v85	Listeria_grayi_1641_v1	0.6688
Listeria_monocytogenes_1639_v84	Listeria_grayi_1641_v1	0.6719
Listeria_monocytogenes_1639_v83	Listeria_grayi_1641_v1	0.6695
Listeria_monocytogenes_1639_v82	Listeria_grayi_1641_v1	0.669
Listeria_monocytogenes_1639_v81	Listeria_grayi_1641_v1	0.6678
Listeria_monocytogenes_1639_v80	Listeria_grayi_1641_v1	0.67
Listeria_monocytogenes_1639_v7	Listeria_grayi_1641_v1	0.6631
Listeria_monocytogenes_1639_v79	Listeria_grayi_1641_v1	0.6663
Listeria_monocytogenes_1639_v78	Listeria_grayi_1641_v1	0.6673
Listeria_monocytogenes_1639_v77	Listeria_grayi_1641_v1	0.6717
Listeria_monocytogenes_1639_v76	Listeria_grayi_1641_v1	0.6623
Listeria_monocytogenes_1639_v75	Listeria_grayi_1641_v1	0.6605
Listeria_monocytogenes_1639_v74	Listeria_grayi_1641_v1	0.6719
Listeria_monocytogenes_1639_v73	Listeria_grayi_1641_v1	0.668
Listeria_monocytogenes_1639_v72	Listeria_grayi_1641_v1	0.668
Listeria_monocytogenes_1639_v71	Listeria_grayi_1641_v1	0.6636
Listeria_monocytogenes_1639_v70	Listeria_grayi_1641_v1	0.6685
Listeria_monocytogenes_1639_v6	Listeria_grayi_1641_v1	0.6626
Listeria_monocytogenes_1639_v69	Listeria_grayi_1641_v1	0.6707
Listeria_monocytogenes_1639_v68	Listeria_grayi_1641_v1	0.6729
Listeria_monocytogenes_1639_v67	Listeria_grayi_1641_v1	0.6685
Listeria_monocytogenes_1639_v66	Listeria_grayi_1641_v1	0.6733
Listeria_monocytogenes_1639_v65	Listeria_grayi_1641_v1	0.6658
Listeria_monocytogenes_1639_v64	Listeria_grayi_1641_v1	0.6735
Listeria_monocytogenes_1639_v63	Listeria_grayi_1641_v1	0.6713
Listeria_monocytogenes_1639_v62	Listeria_grayi_1641_v1	0.6652
Listeria_monocytogenes_1639_v61	Listeria_grayi_1641_v1	0.6653
Listeria_monocytogenes_1639_v60	Listeria_grayi_1641_v1	0.668
Listeria_monocytogenes_1639_v5	Listeria_grayi_1641_v1	0.6512
Listeria_monocytogenes_1639_v59	Listeria_grayi_1641_v1	0.6697
Listeria_monocytogenes_1639_v58	Listeria_grayi_1641_v1	0.6717
Listeria_monocytogenes_1639_v57	Listeria_grayi_1641_v1	0.6637
Listeria_monocytogenes_1639_v56	Listeria_grayi_1641_v1	0.6709
Listeria_monocytogenes_1639_v55	Listeria_grayi_1641_v1	0.6635
Listeria_monocytogenes_1639_v54	Listeria_grayi_1641_v1	0.6736
Listeria_monocytogenes_1639_v53	Listeria_grayi_1641_v1	0.6748
Listeria_monocytogenes_1639_v52	Listeria_grayi_1641_v1	0.666
Listeria_monocytogenes_1639_v51	Listeria_grayi_1641_v1	0.669
Listeria_monocytogenes_1639_v50	Listeria_grayi_1641_v1	0.6623
Listeria_monocytogenes_1639_v4	Listeria_grayi_1641_v1	0.6559
Listeria_monocytogenes_1639_v49	Listeria_grayi_1641_v1	0.669
Listeria_monocytogenes_1639_v48	Listeria_grayi_1641_v1	0.6659
Listeria_monocytogenes_1639_v47	Listeria_grayi_1641_v1	0.6628
Listeria_monocytogenes_1639_v46	Listeria_grayi_1641_v1	0.6728
Listeria_monocytogenes_1639_v45	Listeria_grayi_1641_v1	0.6608
Listeria_monocytogenes_1639_v44	Listeria_grayi_1641_v1	0.6631
Listeria_monocytogenes_1639_v43	Listeria_grayi_1641_v1	0.6631
Listeria_monocytogenes_1639_v42	Listeria_grayi_1641_v1	0.6658
Listeria_monocytogenes_1639_v41	Listeria_grayi_1641_v1	0.6587
Listeria_monocytogenes_1639_v40	Listeria_grayi_1641_v1	0.6699

Listeria_monocytogenes_1639_v3	Listeria_grayi_1641_v1	0.6563
Listeria_monocytogenes_1639_v39	Listeria_grayi_1641_v1	0.6669
Listeria_monocytogenes_1639_v38	Listeria_grayi_1641_v1	0.6675
Listeria_monocytogenes_1639_v37	Listeria_grayi_1641_v1	0.6633
Listeria_monocytogenes_1639_v36	Listeria_grayi_1641_v1	0.6658
Listeria_monocytogenes_1639_v35	Listeria_grayi_1641_v1	0.6692
Listeria_monocytogenes_1639_v34	Listeria_grayi_1641_v1	0.6668
Listeria_monocytogenes_1639_v33	Listeria_grayi_1641_v1	0.6657
Listeria_monocytogenes_1639_v32	Listeria_grayi_1641_v1	0.6622
Listeria_monocytogenes_1639_v31	Listeria_grayi_1641_v1	0.6672
Listeria_monocytogenes_1639_v30	Listeria_grayi_1641_v1	0.6703
Listeria_monocytogenes_1639_v2	Listeria_grayi_1641_v1	0.6497
Listeria_monocytogenes_1639_v29	Listeria_grayi_1641_v1	0.6614
Listeria_monocytogenes_1639_v28	Listeria_grayi_1641_v1	0.6626
Listeria_monocytogenes_1639_v27	Listeria_grayi_1641_v1	0.6606
Listeria_monocytogenes_1639_v26	Listeria_grayi_1641_v1	0.6607
Listeria_monocytogenes_1639_v25	Listeria_grayi_1641_v1	0.6608
Listeria_monocytogenes_1639_v24	Listeria_grayi_1641_v1	0.6652
Listeria_monocytogenes_1639_v23	Listeria_grayi_1641_v1	0.6656
Listeria_monocytogenes_1639_v22	Listeria_grayi_1641_v1	0.6667
Listeria_monocytogenes_1639_v21	Listeria_grayi_1641_v1	0.6642
Listeria_monocytogenes_1639_v20	Listeria_grayi_1641_v1	0.6636
Listeria_monocytogenes_1639_v1	Listeria_grayi_1641_v1	0.6576
Listeria_monocytogenes_1639_v19	Listeria_grayi_1641_v1	0.6633
Listeria_monocytogenes_1639_v198	Listeria_grayi_1641_v1	0.6526
Listeria_monocytogenes_1639_v197	Listeria_grayi_1641_v1	0.6683
Listeria_monocytogenes_1639_v196	Listeria_grayi_1641_v1	0.6585
Listeria_monocytogenes_1639_v195	Listeria_grayi_1641_v1	0.6845
Listeria_monocytogenes_1639_v194	Listeria_grayi_1641_v1	0.6845
Listeria_monocytogenes_1639_v193	Listeria_grayi_1641_v1	0.6841
Listeria_monocytogenes_1639_v192	Listeria_grayi_1641_v1	0.6627
Listeria_monocytogenes_1639_v191	Listeria_grayi_1641_v1	0.6826
Listeria_monocytogenes_1639_v190	Listeria_grayi_1641_v1	0.6826
Listeria_monocytogenes_1639_v18	Listeria_grayi_1641_v1	0.6603
Listeria_monocytogenes_1639_v189	Listeria_grayi_1641_v1	0.6823
Listeria_monocytogenes_1639_v188	Listeria_grayi_1641_v1	0.6828
Listeria_monocytogenes_1639_v187	Listeria_grayi_1641_v1	0.6826
Listeria_monocytogenes_1639_v186	Listeria_grayi_1641_v1	0.6829
Listeria_monocytogenes_1639_v185	Listeria_grayi_1641_v1	0.6826
Listeria_monocytogenes_1639_v184	Listeria_grayi_1641_v1	0.6825
Listeria_monocytogenes_1639_v183	Listeria_grayi_1641_v1	0.6818
Listeria_monocytogenes_1639_v182	Listeria_grayi_1641_v1	0.6835
Listeria_monocytogenes_1639_v181	Listeria_grayi_1641_v1	0.6689
Listeria_monocytogenes_1639_v180	Listeria_grayi_1641_v1	0.6689
Listeria_monocytogenes_1639_v17	Listeria_grayi_1641_v1	0.6603
Listeria_monocytogenes_1639_v179	Listeria_grayi_1641_v1	0.677
Listeria_monocytogenes_1639_v178	Listeria_grayi_1641_v1	0.6822
Listeria_monocytogenes_1639_v177	Listeria_grayi_1641_v1	0.676
Listeria_monocytogenes_1639_v176	Listeria_grayi_1641_v1	0.68
Listeria_monocytogenes_1639_v175	Listeria_grayi_1641_v1	0.6753
Listeria_monocytogenes_1639_v174	Listeria_grayi_1641_v1	0.6819
Listeria_monocytogenes_1639_v173	Listeria_grayi_1641_v1	0.6796
Listeria_monocytogenes_1639_v172	Listeria_grayi_1641_v1	0.676
Listeria_monocytogenes_1639_v171	Listeria_grayi_1641_v1	0.6767
Listeria_monocytogenes_1639_v170	Listeria_grayi_1641_v1	0.6777
Listeria_monocytogenes_1639_v16	Listeria_grayi_1641_v1	0.6579
Listeria_monocytogenes_1639_v169	Listeria_grayi_1641_v1	0.6769
Listeria_monocytogenes_1639_v168	Listeria_grayi_1641_v1	0.6819
Listeria_monocytogenes_1639_v167	Listeria_grayi_1641_v1	0.6787
Listeria_monocytogenes_1639_v166	Listeria_grayi_1641_v1	0.6826
Listeria_monocytogenes_1639_v165	Listeria_grayi_1641_v1	0.6766
Listeria_monocytogenes_1639_v164	Listeria_grayi_1641_v1	0.6777
Listeria_monocytogenes_1639_v163	Listeria_grayi_1641_v1	0.6819
Listeria_monocytogenes_1639_v162	Listeria_grayi_1641_v1	0.6777
Listeria_monocytogenes_1639_v161	Listeria_grayi_1641_v1	0.6771
Listeria_monocytogenes_1639_v160	Listeria_grayi_1641_v1	0.6772
Listeria_monocytogenes_1639_v15	Listeria_grayi_1641_v1	0.6606
Listeria_monocytogenes_1639_v159	Listeria_grayi_1641_v1	0.6764
Listeria_monocytogenes_1639_v158	Listeria_grayi_1641_v1	0.6769
Listeria_monocytogenes_1639_v157	Listeria_grayi_1641_v1	0.6773
Listeria_monocytogenes_1639_v156	Listeria_grayi_1641_v1	0.6734
Listeria_monocytogenes_1639_v155	Listeria_grayi_1641_v1	0.6757
Listeria_monocytogenes_1639_v154	Listeria_grayi_1641_v1	0.6752
Listeria_monocytogenes_1639_v153	Listeria_grayi_1641_v1	0.676
Listeria_monocytogenes_1639_v152	Listeria_grayi_1641_v1	0.6767
Listeria_monocytogenes_1639_v151	Listeria_grayi_1641_v1	0.6767
Listeria_monocytogenes_1639_v150	Listeria_grayi_1641_v1	0.6765
Listeria_monocytogenes_1639_v14	Listeria_grayi_1641_v1	0.6675
Listeria_monocytogenes_1639_v149	Listeria_grayi_1641_v1	0.6761
Listeria_monocytogenes_1639_v148	Listeria_grayi_1641_v1	0.6759
Listeria_monocytogenes_1639_v147	Listeria_grayi_1641_v1	0.6716
Listeria_monocytogenes_1639_v146	Listeria_grayi_1641_v1	0.6805
Listeria_monocytogenes_1639_v145	Listeria_grayi_1641_v1	0.6837
Listeria_monocytogenes_1639_v144	Listeria_grayi_1641_v1	0.6801
Listeria_monocytogenes_1639_v143	Listeria_grayi_1641_v1	0.6786
Listeria_monocytogenes_1639_v142	Listeria_grayi_1641_v1	0.6801
Listeria_monocytogenes_1639_v141	Listeria_grayi_1641_v1	0.6808
Listeria_monocytogenes_1639_v140	Listeria_grayi_1641_v1	0.6804
Listeria_monocytogenes_1639_v13	Listeria_grayi_1641_v1	0.6608
Listeria_monocytogenes_1639_v139	Listeria_grayi_1641_v1	0.674
Listeria_monocytogenes_1639_v138	Listeria_grayi_1641_v1	0.6802

Listeria_monocytogenes_1639_v137	Listeria_grayi_1641_v1	0.6798
Listeria_monocytogenes_1639_v136	Listeria_grayi_1641_v1	0.6724
Listeria_monocytogenes_1639_v135	Listeria_grayi_1641_v1	0.6714
Listeria_monocytogenes_1639_v134	Listeria_grayi_1641_v1	0.6751
Listeria_monocytogenes_1639_v133	Listeria_grayi_1641_v1	0.6746
Listeria_monocytogenes_1639_v132	Listeria_grayi_1641_v1	0.6646
Listeria_monocytogenes_1639_v131	Listeria_grayi_1641_v1	0.6704
Listeria_monocytogenes_1639_v130	Listeria_grayi_1641_v1	0.6733
Listeria_monocytogenes_1639_v12	Listeria_grayi_1641_v1	0.6583
Listeria_monocytogenes_1639_v129	Listeria_grayi_1641_v1	0.6809
Listeria_monocytogenes_1639_v128	Listeria_grayi_1641_v1	0.6796
Listeria_monocytogenes_1639_v127	Listeria_grayi_1641_v1	0.6738
Listeria_monocytogenes_1639_v126	Listeria_grayi_1641_v1	0.6792
Listeria_monocytogenes_1639_v125	Listeria_grayi_1641_v1	0.6769
Listeria_monocytogenes_1639_v124	Listeria_grayi_1641_v1	0.6718
Listeria_monocytogenes_1639_v123	Listeria_grayi_1641_v1	0.6773
Listeria_monocytogenes_1639_v122	Listeria_grayi_1641_v1	0.6775
Listeria_monocytogenes_1639_v121	Listeria_grayi_1641_v1	0.6775
Listeria_monocytogenes_1639_v120	Listeria_grayi_1641_v1	0.6773
Listeria_monocytogenes_1639_v11	Listeria_grayi_1641_v1	0.659
Listeria_monocytogenes_1639_v119	Listeria_grayi_1641_v1	0.6775
Listeria_monocytogenes_1639_v118	Listeria_grayi_1641_v1	0.6775
Listeria_monocytogenes_1639_v117	Listeria_grayi_1641_v1	0.6738
Listeria_monocytogenes_1639_v116	Listeria_grayi_1641_v1	0.6751
Listeria_monocytogenes_1639_v115	Listeria_grayi_1641_v1	0.6753
Listeria_monocytogenes_1639_v114	Listeria_grayi_1641_v1	0.6712
Listeria_monocytogenes_1639_v113	Listeria_grayi_1641_v1	0.6765
Listeria_monocytogenes_1639_v112	Listeria_grayi_1641_v1	0.6726
Listeria_monocytogenes_1639_v111	Listeria_grayi_1641_v1	0.6739
Listeria_monocytogenes_1639_v110	Listeria_grayi_1641_v1	0.6722
Listeria_monocytogenes_1639_v10	Listeria_grayi_1641_v1	0.6564
Listeria_monocytogenes_1639_v109	Listeria_grayi_1641_v1	0.6714
Listeria_monocytogenes_1639_v108	Listeria_grayi_1641_v1	0.6712
Listeria_monocytogenes_1639_v107	Listeria_grayi_1641_v1	0.6759
Listeria_monocytogenes_1639_v106	Listeria_grayi_1641_v1	0.6759
Listeria_monocytogenes_1639_v105	Listeria_grayi_1641_v1	0.6759
Listeria_monocytogenes_1639_v104	Listeria_grayi_1641_v1	0.6761
Listeria_monocytogenes_1639_v103	Listeria_grayi_1641_v1	0.6761
Listeria_monocytogenes_1639_v102	Listeria_grayi_1641_v1	0.67
Listeria_monocytogenes_1639_v101	Listeria_grayi_1641_v1	0.666
Listeria_monocytogenes_1639_v100	Listeria_grayi_1641_v1	0.6657
Listeria_monocytogenes_1639	Listeria_grayi_1641_v1	0.6823
Listeria_monocytogenes_10403S_393133	Listeria_grayi_1641_v1	0.678
Listeria_monocytogenes_08-5923_637381	Listeria_grayi_1641_v1	0.6631
Listeria_monocytogenes_08-5578_653938	Listeria_grayi_1641_v1	0.6577
Listeria_monocytogenes_07PF0776_1126011	Listeria_grayi_1641_v1	0.6763
Listeria_marthii_FSL_S4-120_702457	Listeria_grayi_1641_v1	0.6379
Listeria_marthii_529731_v1	Listeria_grayi_1641_v1	0.6619
Listeria_marthii_529731	Listeria_grayi_1641_v1	0.6677
Listeria_ivanovii_WSLC3009_1457190	Listeria_grayi_1641_v1	0.6711
Listeria_ivanovii_subsp_londoniensis_202752_v2	Listeria_grayi_1641_v1	0.6711
Listeria_ivanovii_subsp_londoniensis_202752_v1	Listeria_grayi_1641_v1	0.6619
Listeria_ivanovii_subsp_londoniensis_202752	Listeria_grayi_1641_v1	0.6674
Listeria_ivanovii_subsp_ivanovii_PAM_55_881621	Listeria_grayi_1641_v1	0.6701
Listeria_ivanovii_subsp_ivanovii_202751_v2	Listeria_grayi_1641_v1	0.6712
Listeria_ivanovii_subsp_ivanovii_202751_v1	Listeria_grayi_1641_v1	0.6712
Listeria_ivanovii_subsp_ivanovii_202751	Listeria_grayi_1641_v1	0.6712
Listeria_ivanovii_FSL_F6-596_702454	Listeria_grayi_1641_v1	0.6204
Listeria_ivanovii_1638	Listeria_grayi_1641_v1	0.6717
Listeria_innocua_FSL_S4-378_702456	Listeria_grayi_1641_v1	0.6145
Listeria_innocua_FSL_J1-023_702455	Listeria_grayi_1641_v1	0.6442
Listeria_innocua_Clip11262_272626	Listeria_grayi_1641_v1	0.6568
Listeria_innocua_1642_v4	Listeria_grayi_1641_v1	0.6573
Listeria_innocua_1642_v3	Listeria_grayi_1641_v1	0.6729
Listeria_innocua_1642_v2	Listeria_grayi_1641_v1	0.6715
Listeria_innocua_1642_v1	Listeria_grayi_1641_v1	0.6748
Listeria_innocua_1642	Listeria_grayi_1641_v1	0.6724
Listeria_grayi_DSM_20601_525367	Listeria_grayi_1641_v1	0.8932
Listeria_grayi_1641_v2	Listeria_grayi_1641_v1	0.8612
Listeria_grayi_1641_v1	Listeria_grayi_1641_v1	0.0951
Listeria_welshimeri_1643	Listeria_grayi_1641_v2	0.7038
Listeria_welshimeri_serovar_6b_str_SLCC5334_386043	Listeria_grayi_1641_v2	0.7036
Listeria_seeligeri_serovar_1_2b_str_SLCC3954_683837	Listeria_grayi_1641_v2	0.7089
Listeria_seeligeri_FSL_S4-171_702452	Listeria_grayi_1641_v2	0.6549
Listeria_seeligeri_FSL_N1-067_702453	Listeria_grayi_1641_v2	0.6549
Listeria_seeligeri_1640_v1	Listeria_grayi_1641_v2	0.7094
Listeria_seeligeri_1640	Listeria_grayi_1641_v2	0.6934
Listeria_monocytogenes_WSLC1042_1457188	Listeria_grayi_1641_v2	0.7125
Listeria_monocytogenes_WSLC1001_1457187	Listeria_grayi_1641_v2	0.7124
Listeria_monocytogenes_SLCC7179_879090	Listeria_grayi_1641_v2	0.7164
Listeria_monocytogenes_SLCC5850_882096	Listeria_grayi_1641_v2	0.7154
Listeria_monocytogenes_SLCC2755_932919	Listeria_grayi_1641_v2	0.7077
Listeria_monocytogenes_SLCC2540_879089	Listeria_grayi_1641_v2	0.7151
Listeria_monocytogenes_SLCC2479_882020	Listeria_grayi_1641_v2	0.7056
Listeria_monocytogenes_SLCC2378_879088	Listeria_grayi_1641_v2	0.7136
Listeria_monocytogenes_SLCC2376_882097	Listeria_grayi_1641_v2	0.722
Listeria_monocytogenes_SLCC2372_932920	Listeria_grayi_1641_v2	0.7022
Listeria_monocytogenes_serotype_4b_str_LL195_1230340	Listeria_grayi_1641_v2	0.7156
Listeria_monocytogenes_serotype_4b_str_LIS0087_1134669	Listeria_grayi_1641_v2	0.6946
Listeria_monocytogenes_serotype_4b_str_F2365_265669	Listeria_grayi_1641_v2	0.7156
Listeria_monocytogenes_serotype_4b_str_CLIP_80459_568819	Listeria_grayi_1641_v2	0.7216

Listeria_monocytogenes_serotype_4b_str_81-0861_1196161	Listeria_grayi_1641_v2	0.7053
Listeria_monocytogenes_serotype_4b_str_81-0592_1196160	Listeria_grayi_1641_v2	0.7062
Listeria_monocytogenes_serotype_4b_str_81-0558_1196159	Listeria_grayi_1641_v2	0.7068
Listeria_monocytogenes_serotype_4b_str_10-0809_1196162	Listeria_grayi_1641_v2	0.7062
Listeria_monocytogenes_serotype_4b_str_02-6680_1196175	Listeria_grayi_1641_v2	0.7168
Listeria_monocytogenes_serotype_4b_str_02-6679_1196174	Listeria_grayi_1641_v2	0.7168
Listeria_monocytogenes_serotype_4b_str_02-1792_1196173	Listeria_grayi_1641_v2	0.7065
Listeria_monocytogenes_serotype_4b_str_02-1289_1196172	Listeria_grayi_1641_v2	0.7065
Listeria_monocytogenes_serotype_4b_str_02-1103_1196171	Listeria_grayi_1641_v2	0.7065
Listeria_monocytogenes_serotype_3c_str_10-5027_1196195	Listeria_grayi_1641_v2	0.7047
Listeria_monocytogenes_serotype_1_2c_str_10-5026_1196194	Listeria_grayi_1641_v2	0.7055
Listeria_monocytogenes_serotype_1_2c_str_10-5025_1196193	Listeria_grayi_1641_v2	0.7055
Listeria_monocytogenes_serotype_1_2b_str_10-0811_1196164	Listeria_grayi_1641_v2	0.7019
Listeria_monocytogenes_serotype_1_2b_str_10-0810_1196163	Listeria_grayi_1641_v2	0.7019
Listeria_monocytogenes_serotype_1_2a_str_99-6370_1196179	Listeria_grayi_1641_v2	0.6981
Listeria_monocytogenes_serotype_1_2a_str_98-2035_1196178	Listeria_grayi_1641_v2	0.6981
Listeria_monocytogenes_serotype_1_2a_str_95-0093_1196177	Listeria_grayi_1641_v2	0.7019
Listeria_monocytogenes_serotype_1_2a_str_88-0478_1196176	Listeria_grayi_1641_v2	0.7009
Listeria_monocytogenes_serotype_1_2a_str_10-5024_1196192	Listeria_grayi_1641_v2	0.7025
Listeria_monocytogenes_serotype_1_2a_str_10-4758_1196168	Listeria_grayi_1641_v2	0.7155
Listeria_monocytogenes_serotype_1_2a_str_10-4754_1196167	Listeria_grayi_1641_v2	0.7155
Listeria_monocytogenes_serotype_1_2a_str_10-1321_1196191	Listeria_grayi_1641_v2	0.7025
Listeria_monocytogenes_serotype_1_2a_str_10-1047_1196190	Listeria_grayi_1641_v2	0.7028
Listeria_monocytogenes_serotype_1_2a_str_10-1046_1196189	Listeria_grayi_1641_v2	0.7028
Listeria_monocytogenes_serotype_1_2a_str_10-0934_1196170	Listeria_grayi_1641_v2	0.7247
Listeria_monocytogenes_serotype_1_2a_str_10-0933_1196169	Listeria_grayi_1641_v2	0.7247
Listeria_monocytogenes_serotype_1_2a_str_10-0815_1196188	Listeria_grayi_1641_v2	0.6992
Listeria_monocytogenes_serotype_1_2a_str_10-0814_1196187	Listeria_grayi_1641_v2	0.7027
Listeria_monocytogenes_serotype_1_2a_str_10-0813_1196166	Listeria_grayi_1641_v2	0.7099
Listeria_monocytogenes_serotype_1_2a_str_10-0812_1196165	Listeria_grayi_1641_v2	0.7099
Listeria_monocytogenes_serotype_1_2a_str_08-7669_1196186	Listeria_grayi_1641_v2	0.7092
Listeria_monocytogenes_serotype_1_2a_str_08-7374_1196185	Listeria_grayi_1641_v2	0.7025
Listeria_monocytogenes_serotype_1_2a_str_08-6997_1196184	Listeria_grayi_1641_v2	0.6992
Listeria_monocytogenes_serotype_1_2a_str_08-6569_1196183	Listeria_grayi_1641_v2	0.6992
Listeria_monocytogenes_serotype_1_2a_str_08-6056_1196182	Listeria_grayi_1641_v2	0.6992
Listeria_monocytogenes_serotype_1_2a_str_04-5457_1196181	Listeria_grayi_1641_v2	0.7025
Listeria_monocytogenes_serotype_1_2a_str_02-5993_1196180	Listeria_grayi_1641_v2	0.7023
Listeria_monocytogenes_R479a_1437838	Listeria_grayi_1641_v2	0.7021
Listeria_monocytogenes_N53-1_1234142	Listeria_grayi_1641_v2	0.6841
Listeria_monocytogenes_M7_1030009	Listeria_grayi_1641_v2	0.6939
Listeria_monocytogenes_LO28_393132	Listeria_grayi_1641_v2	0.7045
Listeria_monocytogenes_L99_563174	Listeria_grayi_1641_v2	0.6944
Listeria_monocytogenes_L312_882094	Listeria_grayi_1641_v2	0.7216
Listeria_monocytogenes_J1816_930781	Listeria_grayi_1641_v2	0.7016
Listeria_monocytogenes_J1-220_930782	Listeria_grayi_1641_v2	0.702
Listeria_monocytogenes_J0161_393130	Listeria_grayi_1641_v2	0.7056
Listeria_monocytogenes_HCC23_552536	Listeria_grayi_1641_v2	0.6941
Listeria_monocytogenes_FSL_R2-561_393126	Listeria_grayi_1641_v2	0.704
Listeria_monocytogenes_FSL_J1-208_393119	Listeria_grayi_1641_v2	0.707
Listeria_monocytogenes_FSL_J1-175_393118	Listeria_grayi_1641_v2	0.7104
Listeria_monocytogenes_Finland_1998_393127	Listeria_grayi_1641_v2	0.7211
Listeria_monocytogenes_EGD-e_169963_v1	Listeria_grayi_1641_v2	0.7139
Listeria_monocytogenes_EGD-e_169963	Listeria_grayi_1641_v2	0.7143
Listeria_monocytogenes_EGD_1334565	Listeria_grayi_1641_v2	0.7161
Listeria_monocytogenes_CFSAN002349_1299895	Listeria_grayi_1641_v2	0.7179
Listeria_monocytogenes_ATCC_19117_882095_v1	Listeria_grayi_1641_v2	0.7164
Listeria_monocytogenes_ATCC_19117_882095	Listeria_grayi_1641_v2	0.7163
Listeria_monocytogenes_6179_1288295	Listeria_grayi_1641_v2	0.6965
Listeria_monocytogenes_1639_v9	Listeria_grayi_1641_v2	0.6898
Listeria_monocytogenes_1639_v99	Listeria_grayi_1641_v2	0.7036
Listeria_monocytogenes_1639_v98	Listeria_grayi_1641_v2	0.7064
Listeria_monocytogenes_1639_v97	Listeria_grayi_1641_v2	0.7078
Listeria_monocytogenes_1639_v96	Listeria_grayi_1641_v2	0.7027
Listeria_monocytogenes_1639_v95	Listeria_grayi_1641_v2	0.7028
Listeria_monocytogenes_1639_v94	Listeria_grayi_1641_v2	0.7031
Listeria_monocytogenes_1639_v93	Listeria_grayi_1641_v2	0.7051
Listeria_monocytogenes_1639_v92	Listeria_grayi_1641_v2	0.7033
Listeria_monocytogenes_1639_v91	Listeria_grayi_1641_v2	0.7024
Listeria_monocytogenes_1639_v90	Listeria_grayi_1641_v2	0.7064
Listeria_monocytogenes_1639_v8	Listeria_grayi_1641_v2	0.6933
Listeria_monocytogenes_1639_v89	Listeria_grayi_1641_v2	0.7117
Listeria_monocytogenes_1639_v88	Listeria_grayi_1641_v2	0.7095
Listeria_monocytogenes_1639_v87	Listeria_grayi_1641_v2	0.7056
Listeria_monocytogenes_1639_v86	Listeria_grayi_1641_v2	0.7057
Listeria_monocytogenes_1639_v85	Listeria_grayi_1641_v2	0.7052
Listeria_monocytogenes_1639_v84	Listeria_grayi_1641_v2	0.7099
Listeria_monocytogenes_1639_v83	Listeria_grayi_1641_v2	0.7085
Listeria_monocytogenes_1639_v82	Listeria_grayi_1641_v2	0.7079
Listeria_monocytogenes_1639_v81	Listeria_grayi_1641_v2	0.7063
Listeria_monocytogenes_1639_v80	Listeria_grayi_1641_v2	0.7079
Listeria_monocytogenes_1639_v7	Listeria_grayi_1641_v2	0.6933
Listeria_monocytogenes_1639_v79	Listeria_grayi_1641_v2	0.705
Listeria_monocytogenes_1639_v78	Listeria_grayi_1641_v2	0.7065
Listeria_monocytogenes_1639_v77	Listeria_grayi_1641_v2	0.7112
Listeria_monocytogenes_1639_v76	Listeria_grayi_1641_v2	0.7026
Listeria_monocytogenes_1639_v75	Listeria_grayi_1641_v2	0.6944
Listeria_monocytogenes_1639_v74	Listeria_grayi_1641_v2	0.7088
Listeria_monocytogenes_1639_v73	Listeria_grayi_1641_v2	0.7011
Listeria_monocytogenes_1639_v72	Listeria_grayi_1641_v2	0.7074
Listeria_monocytogenes_1639_v71	Listeria_grayi_1641_v2	0.6991
Listeria_monocytogenes_1639_v70	Listeria_grayi_1641_v2	0.7033

Listeria_monocytogenes_1639_v6	Listeria_grayi_1641_v2	0.6928
Listeria_monocytogenes_1639_v69	Listeria_grayi_1641_v2	0.7065
Listeria_monocytogenes_1639_v68	Listeria_grayi_1641_v2	0.712
Listeria_monocytogenes_1639_v67	Listeria_grayi_1641_v2	0.7043
Listeria_monocytogenes_1639_v66	Listeria_grayi_1641_v2	0.7131
Listeria_monocytogenes_1639_v65	Listeria_grayi_1641_v2	0.7047
Listeria_monocytogenes_1639_v64	Listeria_grayi_1641_v2	0.7126
Listeria_monocytogenes_1639_v63	Listeria_grayi_1641_v2	0.7042
Listeria_monocytogenes_1639_v62	Listeria_grayi_1641_v2	0.703
Listeria_monocytogenes_1639_v61	Listeria_grayi_1641_v2	0.7046
Listeria_monocytogenes_1639_v60	Listeria_grayi_1641_v2	0.7062
Listeria_monocytogenes_1639_v5	Listeria_grayi_1641_v2	0.6824
Listeria_monocytogenes_1639_v59	Listeria_grayi_1641_v2	0.709
Listeria_monocytogenes_1639_v58	Listeria_grayi_1641_v2	0.7072
Listeria_monocytogenes_1639_v57	Listeria_grayi_1641_v2	0.7008
Listeria_monocytogenes_1639_v56	Listeria_grayi_1641_v2	0.7088
Listeria_monocytogenes_1639_v55	Listeria_grayi_1641_v2	0.6978
Listeria_monocytogenes_1639_v54	Listeria_grayi_1641_v2	0.7118
Listeria_monocytogenes_1639_v53	Listeria_grayi_1641_v2	0.7151
Listeria_monocytogenes_1639_v52	Listeria_grayi_1641_v2	0.7047
Listeria_monocytogenes_1639_v51	Listeria_grayi_1641_v2	0.7074
Listeria_monocytogenes_1639_v50	Listeria_grayi_1641_v2	0.7014
Listeria_monocytogenes_1639_v4	Listeria_grayi_1641_v2	0.6908
Listeria_monocytogenes_1639_v49	Listeria_grayi_1641_v2	0.7067
Listeria_monocytogenes_1639_v48	Listeria_grayi_1641_v2	0.7024
Listeria_monocytogenes_1639_v47	Listeria_grayi_1641_v2	0.7023
Listeria_monocytogenes_1639_v46	Listeria_grayi_1641_v2	0.7114
Listeria_monocytogenes_1639_v45	Listeria_grayi_1641_v2	0.6959
Listeria_monocytogenes_1639_v44	Listeria_grayi_1641_v2	0.7025
Listeria_monocytogenes_1639_v43	Listeria_grayi_1641_v2	0.7025
Listeria_monocytogenes_1639_v42	Listeria_grayi_1641_v2	0.7021
Listeria_monocytogenes_1639_v41	Listeria_grayi_1641_v2	0.6912
Listeria_monocytogenes_1639_v40	Listeria_grayi_1641_v2	0.706
Listeria_monocytogenes_1639_v3	Listeria_grayi_1641_v2	0.6912
Listeria_monocytogenes_1639_v39	Listeria_grayi_1641_v2	0.7001
Listeria_monocytogenes_1639_v38	Listeria_grayi_1641_v2	0.7046
Listeria_monocytogenes_1639_v37	Listeria_grayi_1641_v2	0.7004
Listeria_monocytogenes_1639_v36	Listeria_grayi_1641_v2	0.7017
Listeria_monocytogenes_1639_v35	Listeria_grayi_1641_v2	0.7068
Listeria_monocytogenes_1639_v34	Listeria_grayi_1641_v2	0.7061
Listeria_monocytogenes_1639_v33	Listeria_grayi_1641_v2	0.7019
Listeria_monocytogenes_1639_v32	Listeria_grayi_1641_v2	0.6984
Listeria_monocytogenes_1639_v31	Listeria_grayi_1641_v2	0.7065
Listeria_monocytogenes_1639_v30	Listeria_grayi_1641_v2	0.7086
Listeria_monocytogenes_1639_v2	Listeria_grayi_1641_v2	0.6809
Listeria_monocytogenes_1639_v29	Listeria_grayi_1641_v2	0.6947
Listeria_monocytogenes_1639_v28	Listeria_grayi_1641_v2	0.6977
Listeria_monocytogenes_1639_v27	Listeria_grayi_1641_v2	0.6937
Listeria_monocytogenes_1639_v26	Listeria_grayi_1641_v2	0.6938
Listeria_monocytogenes_1639_v25	Listeria_grayi_1641_v2	0.6935
Listeria_monocytogenes_1639_v24	Listeria_grayi_1641_v2	0.7017
Listeria_monocytogenes_1639_v23	Listeria_grayi_1641_v2	0.7017
Listeria_monocytogenes_1639_v22	Listeria_grayi_1641_v2	0.7029
Listeria_monocytogenes_1639_v21	Listeria_grayi_1641_v2	0.7022
Listeria_monocytogenes_1639_v20	Listeria_grayi_1641_v2	0.6998
Listeria_monocytogenes_1639_v1	Listeria_grayi_1641_v2	0.69
Listeria_monocytogenes_1639_v19	Listeria_grayi_1641_v2	0.6996
Listeria_monocytogenes_1639_v198	Listeria_grayi_1641_v2	0.6858
Listeria_monocytogenes_1639_v197	Listeria_grayi_1641_v2	0.7042
Listeria_monocytogenes_1639_v196	Listeria_grayi_1641_v2	0.6984
Listeria_monocytogenes_1639_v195	Listeria_grayi_1641_v2	0.7232
Listeria_monocytogenes_1639_v194	Listeria_grayi_1641_v2	0.7228
Listeria_monocytogenes_1639_v193	Listeria_grayi_1641_v2	0.7224
Listeria_monocytogenes_1639_v192	Listeria_grayi_1641_v2	0.6997
Listeria_monocytogenes_1639_v191	Listeria_grayi_1641_v2	0.7251
Listeria_monocytogenes_1639_v190	Listeria_grayi_1641_v2	0.7242
Listeria_monocytogenes_1639_v18	Listeria_grayi_1641_v2	0.6963
Listeria_monocytogenes_1639_v189	Listeria_grayi_1641_v2	0.7239
Listeria_monocytogenes_1639_v188	Listeria_grayi_1641_v2	0.7248
Listeria_monocytogenes_1639_v187	Listeria_grayi_1641_v2	0.7242
Listeria_monocytogenes_1639_v186	Listeria_grayi_1641_v2	0.7245
Listeria_monocytogenes_1639_v185	Listeria_grayi_1641_v2	0.7242
Listeria_monocytogenes_1639_v184	Listeria_grayi_1641_v2	0.7241
Listeria_monocytogenes_1639_v183	Listeria_grayi_1641_v2	0.7218
Listeria_monocytogenes_1639_v182	Listeria_grayi_1641_v2	0.7236
Listeria_monocytogenes_1639_v181	Listeria_grayi_1641_v2	0.7087
Listeria_monocytogenes_1639_v180	Listeria_grayi_1641_v2	0.7087
Listeria_monocytogenes_1639_v17	Listeria_grayi_1641_v2	0.6963
Listeria_monocytogenes_1639_v179	Listeria_grayi_1641_v2	0.7172
Listeria_monocytogenes_1639_v178	Listeria_grayi_1641_v2	0.7238
Listeria_monocytogenes_1639_v177	Listeria_grayi_1641_v2	0.7158
Listeria_monocytogenes_1639_v176	Listeria_grayi_1641_v2	0.7228
Listeria_monocytogenes_1639_v175	Listeria_grayi_1641_v2	0.7165
Listeria_monocytogenes_1639_v174	Listeria_grayi_1641_v2	0.7234
Listeria_monocytogenes_1639_v173	Listeria_grayi_1641_v2	0.7179
Listeria_monocytogenes_1639_v172	Listeria_grayi_1641_v2	0.7147
Listeria_monocytogenes_1639_v171	Listeria_grayi_1641_v2	0.7154
Listeria_monocytogenes_1639_v170	Listeria_grayi_1641_v2	0.7195
Listeria_monocytogenes_1639_v16	Listeria_grayi_1641_v2	0.6927
Listeria_monocytogenes_1639_v169	Listeria_grayi_1641_v2	0.7183
Listeria_monocytogenes_1639_v168	Listeria_grayi_1641_v2	0.7238

Listeria_monocytogenes_1639_v167	Listeria_grayi_1641_v2	0.7206
Listeria_monocytogenes_1639_v166	Listeria_grayi_1641_v2	0.7241
Listeria_monocytogenes_1639_v165	Listeria_grayi_1641_v2	0.718
Listeria_monocytogenes_1639_v164	Listeria_grayi_1641_v2	0.7165
Listeria_monocytogenes_1639_v163	Listeria_grayi_1641_v2	0.7172
Listeria_monocytogenes_1639_v162	Listeria_grayi_1641_v2	0.7165
Listeria_monocytogenes_1639_v161	Listeria_grayi_1641_v2	0.7158
Listeria_monocytogenes_1639_v160	Listeria_grayi_1641_v2	0.7159
Listeria_monocytogenes_1639_v15	Listeria_grayi_1641_v2	0.6956
Listeria_monocytogenes_1639_v159	Listeria_grayi_1641_v2	0.7162
Listeria_monocytogenes_1639_v158	Listeria_grayi_1641_v2	0.7156
Listeria_monocytogenes_1639_v157	Listeria_grayi_1641_v2	0.7156
Listeria_monocytogenes_1639_v156	Listeria_grayi_1641_v2	0.7097
Listeria_monocytogenes_1639_v155	Listeria_grayi_1641_v2	0.7158
Listeria_monocytogenes_1639_v154	Listeria_grayi_1641_v2	0.7164
Listeria_monocytogenes_1639_v153	Listeria_grayi_1641_v2	0.7165
Listeria_monocytogenes_1639_v152	Listeria_grayi_1641_v2	0.7165
Listeria_monocytogenes_1639_v151	Listeria_grayi_1641_v2	0.7165
Listeria_monocytogenes_1639_v150	Listeria_grayi_1641_v2	0.717
Listeria_monocytogenes_1639_v14	Listeria_grayi_1641_v2	0.6966
Listeria_monocytogenes_1639_v149	Listeria_grayi_1641_v2	0.7161
Listeria_monocytogenes_1639_v148	Listeria_grayi_1641_v2	0.7152
Listeria_monocytogenes_1639_v147	Listeria_grayi_1641_v2	0.7079
Listeria_monocytogenes_1639_v146	Listeria_grayi_1641_v2	0.7207
Listeria_monocytogenes_1639_v145	Listeria_grayi_1641_v2	0.7246
Listeria_monocytogenes_1639_v144	Listeria_grayi_1641_v2	0.7214
Listeria_monocytogenes_1639_v143	Listeria_grayi_1641_v2	0.719
Listeria_monocytogenes_1639_v142	Listeria_grayi_1641_v2	0.7214
Listeria_monocytogenes_1639_v141	Listeria_grayi_1641_v2	0.7185
Listeria_monocytogenes_1639_v140	Listeria_grayi_1641_v2	0.7216
Listeria_monocytogenes_1639_v13	Listeria_grayi_1641_v2	0.6958
Listeria_monocytogenes_1639_v139	Listeria_grayi_1641_v2	0.715
Listeria_monocytogenes_1639_v138	Listeria_grayi_1641_v2	0.7215
Listeria_monocytogenes_1639_v137	Listeria_grayi_1641_v2	0.7211
Listeria_monocytogenes_1639_v136	Listeria_grayi_1641_v2	0.7149
Listeria_monocytogenes_1639_v135	Listeria_grayi_1641_v2	0.7078
Listeria_monocytogenes_1639_v134	Listeria_grayi_1641_v2	0.7132
Listeria_monocytogenes_1639_v133	Listeria_grayi_1641_v2	0.7138
Listeria_monocytogenes_1639_v132	Listeria_grayi_1641_v2	0.7024
Listeria_monocytogenes_1639_v131	Listeria_grayi_1641_v2	0.7072
Listeria_monocytogenes_1639_v130	Listeria_grayi_1641_v2	0.708
Listeria_monocytogenes_1639_v12	Listeria_grayi_1641_v2	0.6904
Listeria_monocytogenes_1639_v129	Listeria_grayi_1641_v2	0.7214
Listeria_monocytogenes_1639_v128	Listeria_grayi_1641_v2	0.7179
Listeria_monocytogenes_1639_v127	Listeria_grayi_1641_v2	0.7124
Listeria_monocytogenes_1639_v126	Listeria_grayi_1641_v2	0.7171
Listeria_monocytogenes_1639_v125	Listeria_grayi_1641_v2	0.7181
Listeria_monocytogenes_1639_v124	Listeria_grayi_1641_v2	0.7101
Listeria_monocytogenes_1639_v123	Listeria_grayi_1641_v2	0.7179
Listeria_monocytogenes_1639_v122	Listeria_grayi_1641_v2	0.7178
Listeria_monocytogenes_1639_v121	Listeria_grayi_1641_v2	0.7178
Listeria_monocytogenes_1639_v120	Listeria_grayi_1641_v2	0.7175
Listeria_monocytogenes_1639_v11	Listeria_grayi_1641_v2	0.6928
Listeria_monocytogenes_1639_v119	Listeria_grayi_1641_v2	0.7178
Listeria_monocytogenes_1639_v118	Listeria_grayi_1641_v2	0.7178
Listeria_monocytogenes_1639_v117	Listeria_grayi_1641_v2	0.7144
Listeria_monocytogenes_1639_v116	Listeria_grayi_1641_v2	0.7131
Listeria_monocytogenes_1639_v115	Listeria_grayi_1641_v2	0.7133
Listeria_monocytogenes_1639_v114	Listeria_grayi_1641_v2	0.7103
Listeria_monocytogenes_1639_v113	Listeria_grayi_1641_v2	0.7146
Listeria_monocytogenes_1639_v112	Listeria_grayi_1641_v2	0.7075
Listeria_monocytogenes_1639_v111	Listeria_grayi_1641_v2	0.7109
Listeria_monocytogenes_1639_v110	Listeria_grayi_1641_v2	0.7105
Listeria_monocytogenes_1639_v10	Listeria_grayi_1641_v2	0.6932
Listeria_monocytogenes_1639_v109	Listeria_grayi_1641_v2	0.7093
Listeria_monocytogenes_1639_v108	Listeria_grayi_1641_v2	0.7075
Listeria_monocytogenes_1639_v107	Listeria_grayi_1641_v2	0.7151
Listeria_monocytogenes_1639_v106	Listeria_grayi_1641_v2	0.7155
Listeria_monocytogenes_1639_v105	Listeria_grayi_1641_v2	0.7155
Listeria_monocytogenes_1639_v104	Listeria_grayi_1641_v2	0.7158
Listeria_monocytogenes_1639_v103	Listeria_grayi_1641_v2	0.7158
Listeria_monocytogenes_1639_v102	Listeria_grayi_1641_v2	0.7103
Listeria_monocytogenes_1639_v101	Listeria_grayi_1641_v2	0.7021
Listeria_monocytogenes_1639_v100	Listeria_grayi_1641_v2	0.7013
Listeria_monocytogenes_1639	Listeria_grayi_1641_v2	0.7239
Listeria_monocytogenes_10403S_393133	Listeria_grayi_1641_v2	0.7183
Listeria_monocytogenes_08-5923_637381	Listeria_grayi_1641_v2	0.7025
Listeria_monocytogenes_08-5578_653938	Listeria_grayi_1641_v2	0.6925
Listeria_monocytogenes_07PF0776_1126011	Listeria_grayi_1641_v2	0.7154
Listeria_marthii_FSL_S4-120_702457	Listeria_grayi_1641_v2	0.6759
Listeria_marthii_529731_v1	Listeria_grayi_1641_v2	0.7003
Listeria_marthii_529731	Listeria_grayi_1641_v2	0.7033
Listeria_ivanovii_WSLC3009_1457190	Listeria_grayi_1641_v2	0.7099
Listeria_ivanovii_subsp_londoniensis_202752_v2	Listeria_grayi_1641_v2	0.7051
Listeria_ivanovii_subsp_londoniensis_202752_v1	Listeria_grayi_1641_v2	0.695
Listeria_ivanovii_subsp_londoniensis_202752	Listeria_grayi_1641_v2	0.7018
Listeria_ivanovii_subsp_ivanovii_PAM_55_881621	Listeria_grayi_1641_v2	0.7088
Listeria_ivanovii_subsp_ivanovii_202751_v2	Listeria_grayi_1641_v2	0.7104
Listeria_ivanovii_subsp_ivanovii_202751_v1	Listeria_grayi_1641_v2	0.7104
Listeria_ivanovii_subsp_ivanovii_202751	Listeria_grayi_1641_v2	0.7104
Listeria_ivanovii_FSL_F6-596_702454	Listeria_grayi_1641_v2	0.6524

Listeria_ivanovii_1638	Listeria_grayi_1641_v2	0.7106
Listeria_innocua_FSL_S4-378_702456	Listeria_grayi_1641_v2	0.6523
Listeria_innocua_FSL_J1-023_702455	Listeria_grayi_1641_v2	0.6809
Listeria_innocua_Clip11262_272626	Listeria_grayi_1641_v2	0.6889
Listeria_innocua_1642_v4	Listeria_grayi_1641_v2	0.6906
Listeria_innocua_1642_v3	Listeria_grayi_1641_v2	0.7058
Listeria_innocua_1642_v2	Listeria_grayi_1641_v2	0.7064
Listeria_innocua_1642_v1	Listeria_grayi_1641_v2	0.7097
Listeria_innocua_1642	Listeria_grayi_1641_v2	0.7057
Listeria_grayi_DSM_20601_525367	Listeria_grayi_1641_v2	0.9062
Listeria_grayi_1641_v2	Listeria_grayi_1641_v2	0.0667
Listeria_welshimeri_1643	Listeria_grayi_DSM_20601_525367	0.7021
Listeria_welshimeri_serovar_6b_str_SLCC5334_386043	Listeria_grayi_DSM_20601_525367	0.7019
Listeria_seeligeri_serovar_1_2b_str_SLCC3954_683837	Listeria_grayi_DSM_20601_525367	0.7092
Listeria_seeligeri_FSL_S4-171_702452	Listeria_grayi_DSM_20601_525367	0.6579
Listeria_seeligeri_FSL_N1-067_702453	Listeria_grayi_DSM_20601_525367	0.6717
Listeria_seeligeri_1640_v1	Listeria_grayi_DSM_20601_525367	0.7081
Listeria_seeligeri_1640	Listeria_grayi_DSM_20601_525367	0.6948
Listeria_monocytogenes_WSLC1042_1457188	Listeria_grayi_DSM_20601_525367	0.7077
Listeria_monocytogenes_WSLC1001_1457187	Listeria_grayi_DSM_20601_525367	0.7108
Listeria_monocytogenes_SLCC7179_879090	Listeria_grayi_DSM_20601_525367	0.7125
Listeria_monocytogenes_SLCC5850_882096	Listeria_grayi_DSM_20601_525367	0.7138
Listeria_monocytogenes_SLCC2755_932919	Listeria_grayi_DSM_20601_525367	0.7053
Listeria_monocytogenes_SLCC2540_879089	Listeria_grayi_DSM_20601_525367	0.7087
Listeria_monocytogenes_SLCC2479_882020	Listeria_grayi_DSM_20601_525367	0.7032
Listeria_monocytogenes_SLCC2378_879088	Listeria_grayi_DSM_20601_525367	0.7088
Listeria_monocytogenes_SLCC2376_882097	Listeria_grayi_DSM_20601_525367	0.7212
Listeria_monocytogenes_SLCC2372_932920	Listeria_grayi_DSM_20601_525367	0.7017
Listeria_monocytogenes_serotype_4b_str_LL195_1230340	Listeria_grayi_DSM_20601_525367	0.7121
Listeria_monocytogenes_serotype_4b_str_LIS0087_1134669	Listeria_grayi_DSM_20601_525367	0.6927
Listeria_monocytogenes_serotype_4b_str_F2365_265669	Listeria_grayi_DSM_20601_525367	0.7121
Listeria_monocytogenes_serotype_4b_str_CLIP_80459_568819	Listeria_grayi_DSM_20601_525367	0.7185
Listeria_monocytogenes_serotype_4b_str_81-0861_1196161	Listeria_grayi_DSM_20601_525367	0.7005
Listeria_monocytogenes_serotype_4b_str_81-0592_1196160	Listeria_grayi_DSM_20601_525367	0.7014
Listeria_monocytogenes_serotype_4b_str_81-0558_1196159	Listeria_grayi_DSM_20601_525367	0.702
Listeria_monocytogenes_serotype_4b_str_10-0809_1196162	Listeria_grayi_DSM_20601_525367	0.7014
Listeria_monocytogenes_serotype_4b_str_02-6680_1196175	Listeria_grayi_DSM_20601_525367	0.7128
Listeria_monocytogenes_serotype_4b_str_02-6679_1196174	Listeria_grayi_DSM_20601_525367	0.7128
Listeria_monocytogenes_serotype_4b_str_02-1792_1196173	Listeria_grayi_DSM_20601_525367	0.7025
Listeria_monocytogenes_serotype_4b_str_02-1289_1196172	Listeria_grayi_DSM_20601_525367	0.7025
Listeria_monocytogenes_serotype_4b_str_02-1103_1196171	Listeria_grayi_DSM_20601_525367	0.7025
Listeria_monocytogenes_serotype_3c_str_10-5027_1196195	Listeria_grayi_DSM_20601_525367	0.7027
Listeria_monocytogenes_serotype_1_2c_str_10-5026_1196194	Listeria_grayi_DSM_20601_525367	0.7035
Listeria_monocytogenes_serotype_1_2c_str_10-5025_1196193	Listeria_grayi_DSM_20601_525367	0.7035
Listeria_monocytogenes_serotype_1_2b_str_10-0811_1196164	Listeria_grayi_DSM_20601_525367	0.7002
Listeria_monocytogenes_serotype_1_2b_str_10-0810_1196163	Listeria_grayi_DSM_20601_525367	0.7002
Listeria_monocytogenes_serotype_1_2a_str_99-6370_1196179	Listeria_grayi_DSM_20601_525367	0.6952
Listeria_monocytogenes_serotype_1_2a_str_98-2035_1196178	Listeria_grayi_DSM_20601_525367	0.6952
Listeria_monocytogenes_serotype_1_2a_str_95-0093_1196177	Listeria_grayi_DSM_20601_525367	0.6994
Listeria_monocytogenes_serotype_1_2a_str_88-0478_1196176	Listeria_grayi_DSM_20601_525367	0.6984
Listeria_monocytogenes_serotype_1_2a_str_10-5024_1196192	Listeria_grayi_DSM_20601_525367	0.6997
Listeria_monocytogenes_serotype_1_2a_str_10-4758_1196168	Listeria_grayi_DSM_20601_525367	0.7131
Listeria_monocytogenes_serotype_1_2a_str_10-4754_1196167	Listeria_grayi_DSM_20601_525367	0.7131
Listeria_monocytogenes_serotype_1_2a_str_10-1321_1196191	Listeria_grayi_DSM_20601_525367	0.6997
Listeria_monocytogenes_serotype_1_2a_str_10-1047_1196190	Listeria_grayi_DSM_20601_525367	0.7
Listeria_monocytogenes_serotype_1_2a_str_10-1046_1196189	Listeria_grayi_DSM_20601_525367	0.7
Listeria_monocytogenes_serotype_1_2a_str_10-0934_1196170	Listeria_grayi_DSM_20601_525367	0.7208
Listeria_monocytogenes_serotype_1_2a_str_10-0933_1196169	Listeria_grayi_DSM_20601_525367	0.7208
Listeria_monocytogenes_serotype_1_2a_str_10-0815_1196188	Listeria_grayi_DSM_20601_525367	0.6963
Listeria_monocytogenes_serotype_1_2a_str_10-0814_1196187	Listeria_grayi_DSM_20601_525367	0.6998
Listeria_monocytogenes_serotype_1_2a_str_10-0813_1196166	Listeria_grayi_DSM_20601_525367	0.7075
Listeria_monocytogenes_serotype_1_2a_str_10-0812_1196165	Listeria_grayi_DSM_20601_525367	0.7075
Listeria_monocytogenes_serotype_1_2a_str_08-7669_1196186	Listeria_grayi_DSM_20601_525367	0.7067
Listeria_monocytogenes_serotype_1_2a_str_08-7374_1196185	Listeria_grayi_DSM_20601_525367	0.6997
Listeria_monocytogenes_serotype_1_2a_str_08-6997_1196184	Listeria_grayi_DSM_20601_525367	0.6963
Listeria_monocytogenes_serotype_1_2a_str_08-6569_1196183	Listeria_grayi_DSM_20601_525367	0.6963
Listeria_monocytogenes_serotype_1_2a_str_08-6056_1196182	Listeria_grayi_DSM_20601_525367	0.6963
Listeria_monocytogenes_serotype_1_2a_str_04-5457_1196181	Listeria_grayi_DSM_20601_525367	0.6997
Listeria_monocytogenes_serotype_1_2a_str_02-5993_1196180	Listeria_grayi_DSM_20601_525367	0.6994
Listeria_monocytogenes_R479a_1437838	Listeria_grayi_DSM_20601_525367	0.7071
Listeria_monocytogenes_N53-1_1234142	Listeria_grayi_DSM_20601_525367	0.6811
Listeria_monocytogenes_M7_1030009	Listeria_grayi_DSM_20601_525367	0.6933
Listeria_monocytogenes_LO28_393132	Listeria_grayi_DSM_20601_525367	0.7021
Listeria_monocytogenes_L99_563174	Listeria_grayi_DSM_20601_525367	0.6938
Listeria_monocytogenes_L312_882094	Listeria_grayi_DSM_20601_525367	0.7185
Listeria_monocytogenes_J1816_930781	Listeria_grayi_DSM_20601_525367	0.6987
Listeria_monocytogenes_J1-220_930782	Listeria_grayi_DSM_20601_525367	0.698
Listeria_monocytogenes_J0161_393130	Listeria_grayi_DSM_20601_525367	0.7012
Listeria_monocytogenes_HCC23_552536	Listeria_grayi_DSM_20601_525367	0.6935
Listeria_monocytogenes_FSL_R2-561_393126	Listeria_grayi_DSM_20601_525367	0.702
Listeria_monocytogenes_FSL_J1-208_393119	Listeria_grayi_DSM_20601_525367	0.7082
Listeria_monocytogenes_FSL_J1-175_393118	Listeria_grayi_DSM_20601_525367	0.7076
Listeria_monocytogenes_Finland_1998_393127	Listeria_grayi_DSM_20601_525367	0.7167
Listeria_monocytogenes_EGD-e_169963_v1	Listeria_grayi_DSM_20601_525367	0.7096
Listeria_monocytogenes_EGD-e_169963	Listeria_grayi_DSM_20601_525367	0.71
Listeria_monocytogenes_EGD_1334565	Listeria_grayi_DSM_20601_525367	0.7145
Listeria_monocytogenes_CFSAN002349_1299895	Listeria_grayi_DSM_20601_525367	0.7147
Listeria_monocytogenes_ATCC_19117_882095_v1	Listeria_grayi_DSM_20601_525367	0.7124
Listeria_monocytogenes_ATCC_19117_882095	Listeria_grayi_DSM_20601_525367	0.7123
Listeria_monocytogenes_6179_1288295	Listeria_grayi_DSM_20601_525367	0.6994
Listeria_monocytogenes_1639_v9	Listeria_grayi_DSM_20601_525367	0.6861

Listeria_monocytogenes_1639_v99	Listeria_grayi_DSM_20601_525367	0.7047
Listeria_monocytogenes_1639_v98	Listeria_grayi_DSM_20601_525367	0.704
Listeria_monocytogenes_1639_v97	Listeria_grayi_DSM_20601_525367	0.705
Listeria_monocytogenes_1639_v96	Listeria_grayi_DSM_20601_525367	0.7022
Listeria_monocytogenes_1639_v95	Listeria_grayi_DSM_20601_525367	0.7023
Listeria_monocytogenes_1639_v94	Listeria_grayi_DSM_20601_525367	0.7025
Listeria_monocytogenes_1639_v93	Listeria_grayi_DSM_20601_525367	0.7066
Listeria_monocytogenes_1639_v92	Listeria_grayi_DSM_20601_525367	0.7036
Listeria_monocytogenes_1639_v91	Listeria_grayi_DSM_20601_525367	0.6991
Listeria_monocytogenes_1639_v90	Listeria_grayi_DSM_20601_525367	0.704
Listeria_monocytogenes_1639_v8	Listeria_grayi_DSM_20601_525367	0.7012
Listeria_monocytogenes_1639_v89	Listeria_grayi_DSM_20601_525367	0.7092
Listeria_monocytogenes_1639_v88	Listeria_grayi_DSM_20601_525367	0.7071
Listeria_monocytogenes_1639_v87	Listeria_grayi_DSM_20601_525367	0.7043
Listeria_monocytogenes_1639_v86	Listeria_grayi_DSM_20601_525367	0.704
Listeria_monocytogenes_1639_v85	Listeria_grayi_DSM_20601_525367	0.7004
Listeria_monocytogenes_1639_v84	Listeria_grayi_DSM_20601_525367	0.7083
Listeria_monocytogenes_1639_v83	Listeria_grayi_DSM_20601_525367	0.7045
Listeria_monocytogenes_1639_v82	Listeria_grayi_DSM_20601_525367	0.7039
Listeria_monocytogenes_1639_v81	Listeria_grayi_DSM_20601_525367	0.703
Listeria_monocytogenes_1639_v80	Listeria_grayi_DSM_20601_525367	0.7067
Listeria_monocytogenes_1639_v7	Listeria_grayi_DSM_20601_525367	0.7012
Listeria_monocytogenes_1639_v79	Listeria_grayi_DSM_20601_525367	0.7014
Listeria_monocytogenes_1639_v78	Listeria_grayi_DSM_20601_525367	0.7033
Listeria_monocytogenes_1639_v77	Listeria_grayi_DSM_20601_525367	0.7056
Listeria_monocytogenes_1639_v76	Listeria_grayi_DSM_20601_525367	0.7001
Listeria_monocytogenes_1639_v75	Listeria_grayi_DSM_20601_525367	0.6938
Listeria_monocytogenes_1639_v74	Listeria_grayi_DSM_20601_525367	0.7225
Listeria_monocytogenes_1639_v73	Listeria_grayi_DSM_20601_525367	0.7014
Listeria_monocytogenes_1639_v72	Listeria_grayi_DSM_20601_525367	0.7026
Listeria_monocytogenes_1639_v71	Listeria_grayi_DSM_20601_525367	0.713
Listeria_monocytogenes_1639_v70	Listeria_grayi_DSM_20601_525367	0.702
Listeria_monocytogenes_1639_v6	Listeria_grayi_DSM_20601_525367	0.7007
Listeria_monocytogenes_1639_v69	Listeria_grayi_DSM_20601_525367	0.7017
Listeria_monocytogenes_1639_v68	Listeria_grayi_DSM_20601_525367	0.7061
Listeria_monocytogenes_1639_v67	Listeria_grayi_DSM_20601_525367	0.7011
Listeria_monocytogenes_1639_v66	Listeria_grayi_DSM_20601_525367	0.7091
Listeria_monocytogenes_1639_v65	Listeria_grayi_DSM_20601_525367	0.7007
Listeria_monocytogenes_1639_v64	Listeria_grayi_DSM_20601_525367	0.7055
Listeria_monocytogenes_1639_v63	Listeria_grayi_DSM_20601_525367	0.708
Listeria_monocytogenes_1639_v62	Listeria_grayi_DSM_20601_525367	0.6989
Listeria_monocytogenes_1639_v61	Listeria_grayi_DSM_20601_525367	0.701
Listeria_monocytogenes_1639_v60	Listeria_grayi_DSM_20601_525367	0.7017
Listeria_monocytogenes_1639_v5	Listeria_grayi_DSM_20601_525367	0.6952
Listeria_monocytogenes_1639_v59	Listeria_grayi_DSM_20601_525367	0.705
Listeria_monocytogenes_1639_v58	Listeria_grayi_DSM_20601_525367	0.7032
Listeria_monocytogenes_1639_v57	Listeria_grayi_DSM_20601_525367	0.6976
Listeria_monocytogenes_1639_v56	Listeria_grayi_DSM_20601_525367	0.7044
Listeria_monocytogenes_1639_v55	Listeria_grayi_DSM_20601_525367	0.6991
Listeria_monocytogenes_1639_v54	Listeria_grayi_DSM_20601_525367	0.7086
Listeria_monocytogenes_1639_v53	Listeria_grayi_DSM_20601_525367	0.7065
Listeria_monocytogenes_1639_v52	Listeria_grayi_DSM_20601_525367	0.7011
Listeria_monocytogenes_1639_v51	Listeria_grayi_DSM_20601_525367	0.7022
Listeria_monocytogenes_1639_v50	Listeria_grayi_DSM_20601_525367	0.6989
Listeria_monocytogenes_1639_v4	Listeria_grayi_DSM_20601_525367	0.6887
Listeria_monocytogenes_1639_v49	Listeria_grayi_DSM_20601_525367	0.7015
Listeria_monocytogenes_1639_v48	Listeria_grayi_DSM_20601_525367	0.6996
Listeria_monocytogenes_1639_v47	Listeria_grayi_DSM_20601_525367	0.6994
Listeria_monocytogenes_1639_v46	Listeria_grayi_DSM_20601_525367	0.7051
Listeria_monocytogenes_1639_v45	Listeria_grayi_DSM_20601_525367	0.7015
Listeria_monocytogenes_1639_v44	Listeria_grayi_DSM_20601_525367	0.6997
Listeria_monocytogenes_1639_v43	Listeria_grayi_DSM_20601_525367	0.6997
Listeria_monocytogenes_1639_v42	Listeria_grayi_DSM_20601_525367	0.6953
Listeria_monocytogenes_1639_v41	Listeria_grayi_DSM_20601_525367	0.6928
Listeria_monocytogenes_1639_v40	Listeria_grayi_DSM_20601_525367	0.7051
Listeria_monocytogenes_1639_v3	Listeria_grayi_DSM_20601_525367	0.689
Listeria_monocytogenes_1639_v39	Listeria_grayi_DSM_20601_525367	0.6992
Listeria_monocytogenes_1639_v38	Listeria_grayi_DSM_20601_525367	0.7002
Listeria_monocytogenes_1639_v37	Listeria_grayi_DSM_20601_525367	0.6967
Listeria_monocytogenes_1639_v36	Listeria_grayi_DSM_20601_525367	0.6997
Listeria_monocytogenes_1639_v35	Listeria_grayi_DSM_20601_525367	0.7044
Listeria_monocytogenes_1639_v34	Listeria_grayi_DSM_20601_525367	0.6997
Listeria_monocytogenes_1639_v33	Listeria_grayi_DSM_20601_525367	0.7002
Listeria_monocytogenes_1639_v32	Listeria_grayi_DSM_20601_525367	0.6955
Listeria_monocytogenes_1639_v31	Listeria_grayi_DSM_20601_525367	0.7001
Listeria_monocytogenes_1639_v30	Listeria_grayi_DSM_20601_525367	0.7023
Listeria_monocytogenes_1639_v2	Listeria_grayi_DSM_20601_525367	0.6926
Listeria_monocytogenes_1639_v29	Listeria_grayi_DSM_20601_525367	0.6949
Listeria_monocytogenes_1639_v28	Listeria_grayi_DSM_20601_525367	0.6955
Listeria_monocytogenes_1639_v27	Listeria_grayi_DSM_20601_525367	0.6927
Listeria_monocytogenes_1639_v26	Listeria_grayi_DSM_20601_525367	0.6928
Listeria_monocytogenes_1639_v25	Listeria_grayi_DSM_20601_525367	0.6925
Listeria_monocytogenes_1639_v24	Listeria_grayi_DSM_20601_525367	0.6988
Listeria_monocytogenes_1639_v23	Listeria_grayi_DSM_20601_525367	0.6988
Listeria_monocytogenes_1639_v22	Listeria_grayi_DSM_20601_525367	0.7013
Listeria_monocytogenes_1639_v21	Listeria_grayi_DSM_20601_525367	0.6981
Listeria_monocytogenes_1639_v20	Listeria_grayi_DSM_20601_525367	0.697
Listeria_monocytogenes_1639_v1	Listeria_grayi_DSM_20601_525367	0.6932
Listeria_monocytogenes_1639_v19	Listeria_grayi_DSM_20601_525367	0.6967
Listeria_monocytogenes_1639_v198	Listeria_grayi_DSM_20601_525367	0.6828
Listeria_monocytogenes_1639_v197	Listeria_grayi_DSM_20601_525367	0.7002

Listeria_monocytogenes_1639_v196	Listeria_grayi_DSM_20601_525367	0.6966
Listeria_monocytogenes_1639_v195	Listeria_grayi_DSM_20601_525367	0.7225
Listeria_monocytogenes_1639_v194	Listeria_grayi_DSM_20601_525367	0.722
Listeria_monocytogenes_1639_v193	Listeria_grayi_DSM_20601_525367	0.7216
Listeria_monocytogenes_1639_v192	Listeria_grayi_DSM_20601_525367	0.7027
Listeria_monocytogenes_1639_v191	Listeria_grayi_DSM_20601_525367	0.7207
Listeria_monocytogenes_1639_v190	Listeria_grayi_DSM_20601_525367	0.7219
Listeria_monocytogenes_1639_v18	Listeria_grayi_DSM_20601_525367	0.6926
Listeria_monocytogenes_1639_v189	Listeria_grayi_DSM_20601_525367	0.7216
Listeria_monocytogenes_1639_v188	Listeria_grayi_DSM_20601_525367	0.7213
Listeria_monocytogenes_1639_v187	Listeria_grayi_DSM_20601_525367	0.7219
Listeria_monocytogenes_1639_v186	Listeria_grayi_DSM_20601_525367	0.7222
Listeria_monocytogenes_1639_v185	Listeria_grayi_DSM_20601_525367	0.7219
Listeria_monocytogenes_1639_v184	Listeria_grayi_DSM_20601_525367	0.7218
Listeria_monocytogenes_1639_v183	Listeria_grayi_DSM_20601_525367	0.7183
Listeria_monocytogenes_1639_v182	Listeria_grayi_DSM_20601_525367	0.7201
Listeria_monocytogenes_1639_v181	Listeria_grayi_DSM_20601_525367	0.7067
Listeria_monocytogenes_1639_v180	Listeria_grayi_DSM_20601_525367	0.7067
Listeria_monocytogenes_1639_v17	Listeria_grayi_DSM_20601_525367	0.6926
Listeria_monocytogenes_1639_v179	Listeria_grayi_DSM_20601_525367	0.7124
Listeria_monocytogenes_1639_v178	Listeria_grayi_DSM_20601_525367	0.7187
Listeria_monocytogenes_1639_v177	Listeria_grayi_DSM_20601_525367	0.713
Listeria_monocytogenes_1639_v176	Listeria_grayi_DSM_20601_525367	0.7189
Listeria_monocytogenes_1639_v175	Listeria_grayi_DSM_20601_525367	0.7118
Listeria_monocytogenes_1639_v174	Listeria_grayi_DSM_20601_525367	0.7183
Listeria_monocytogenes_1639_v173	Listeria_grayi_DSM_20601_525367	0.7163
Listeria_monocytogenes_1639_v172	Listeria_grayi_DSM_20601_525367	0.7131
Listeria_monocytogenes_1639_v171	Listeria_grayi_DSM_20601_525367	0.715
Listeria_monocytogenes_1639_v170	Listeria_grayi_DSM_20601_525367	0.7172
Listeria_monocytogenes_1639_v16	Listeria_grayi_DSM_20601_525367	0.6983
Listeria_monocytogenes_1639_v169	Listeria_grayi_DSM_20601_525367	0.7151
Listeria_monocytogenes_1639_v168	Listeria_grayi_DSM_20601_525367	0.7191
Listeria_monocytogenes_1639_v167	Listeria_grayi_DSM_20601_525367	0.7151
Listeria_monocytogenes_1639_v166	Listeria_grayi_DSM_20601_525367	0.7193
Listeria_monocytogenes_1639_v165	Listeria_grayi_DSM_20601_525367	0.7149
Listeria_monocytogenes_1639_v164	Listeria_grayi_DSM_20601_525367	0.7129
Listeria_monocytogenes_1639_v163	Listeria_grayi_DSM_20601_525367	0.7181
Listeria_monocytogenes_1639_v162	Listeria_grayi_DSM_20601_525367	0.7129
Listeria_monocytogenes_1639_v161	Listeria_grayi_DSM_20601_525367	0.7122
Listeria_monocytogenes_1639_v160	Listeria_grayi_DSM_20601_525367	0.7123
Listeria_monocytogenes_1639_v15	Listeria_grayi_DSM_20601_525367	0.6938
Listeria_monocytogenes_1639_v159	Listeria_grayi_DSM_20601_525367	0.7134
Listeria_monocytogenes_1639_v158	Listeria_grayi_DSM_20601_525367	0.7121
Listeria_monocytogenes_1639_v157	Listeria_grayi_DSM_20601_525367	0.7121
Listeria_monocytogenes_1639_v156	Listeria_grayi_DSM_20601_525367	0.7242
Listeria_monocytogenes_1639_v155	Listeria_grayi_DSM_20601_525367	0.7126
Listeria_monocytogenes_1639_v154	Listeria_grayi_DSM_20601_525367	0.7125
Listeria_monocytogenes_1639_v153	Listeria_grayi_DSM_20601_525367	0.7129
Listeria_monocytogenes_1639_v152	Listeria_grayi_DSM_20601_525367	0.7137
Listeria_monocytogenes_1639_v151	Listeria_grayi_DSM_20601_525367	0.7137
Listeria_monocytogenes_1639_v150	Listeria_grayi_DSM_20601_525367	0.7154
Listeria_monocytogenes_1639_v14	Listeria_grayi_DSM_20601_525367	0.7014
Listeria_monocytogenes_1639_v149	Listeria_grayi_DSM_20601_525367	0.7145
Listeria_monocytogenes_1639_v148	Listeria_grayi_DSM_20601_525367	0.7125
Listeria_monocytogenes_1639_v147	Listeria_grayi_DSM_20601_525367	0.7212
Listeria_monocytogenes_1639_v146	Listeria_grayi_DSM_20601_525367	0.7164
Listeria_monocytogenes_1639_v145	Listeria_grayi_DSM_20601_525367	0.7167
Listeria_monocytogenes_1639_v144	Listeria_grayi_DSM_20601_525367	0.7182
Listeria_monocytogenes_1639_v143	Listeria_grayi_DSM_20601_525367	0.7163
Listeria_monocytogenes_1639_v142	Listeria_grayi_DSM_20601_525367	0.7182
Listeria_monocytogenes_1639_v141	Listeria_grayi_DSM_20601_525367	0.7166
Listeria_monocytogenes_1639_v140	Listeria_grayi_DSM_20601_525367	0.7185
Listeria_monocytogenes_1639_v13	Listeria_grayi_DSM_20601_525367	0.6941
Listeria_monocytogenes_1639_v139	Listeria_grayi_DSM_20601_525367	0.7122
Listeria_monocytogenes_1639_v138	Listeria_grayi_DSM_20601_525367	0.7183
Listeria_monocytogenes_1639_v137	Listeria_grayi_DSM_20601_525367	0.7179
Listeria_monocytogenes_1639_v136	Listeria_grayi_DSM_20601_525367	0.7113
Listeria_monocytogenes_1639_v135	Listeria_grayi_DSM_20601_525367	0.7026
Listeria_monocytogenes_1639_v134	Listeria_grayi_DSM_20601_525367	0.7084
Listeria_monocytogenes_1639_v133	Listeria_grayi_DSM_20601_525367	0.7114
Listeria_monocytogenes_1639_v132	Listeria_grayi_DSM_20601_525367	0.7019
Listeria_monocytogenes_1639_v131	Listeria_grayi_DSM_20601_525367	0.706
Listeria_monocytogenes_1639_v130	Listeria_grayi_DSM_20601_525367	0.7075
Listeria_monocytogenes_1639_v12	Listeria_grayi_DSM_20601_525367	0.6906
Listeria_monocytogenes_1639_v129	Listeria_grayi_DSM_20601_525367	0.7174
Listeria_monocytogenes_1639_v128	Listeria_grayi_DSM_20601_525367	0.7139
Listeria_monocytogenes_1639_v127	Listeria_grayi_DSM_20601_525367	0.7088
Listeria_monocytogenes_1639_v126	Listeria_grayi_DSM_20601_525367	0.7135
Listeria_monocytogenes_1639_v125	Listeria_grayi_DSM_20601_525367	0.713
Listeria_monocytogenes_1639_v124	Listeria_grayi_DSM_20601_525367	0.706
Listeria_monocytogenes_1639_v123	Listeria_grayi_DSM_20601_525367	0.7147
Listeria_monocytogenes_1639_v122	Listeria_grayi_DSM_20601_525367	0.715
Listeria_monocytogenes_1639_v121	Listeria_grayi_DSM_20601_525367	0.715
Listeria_monocytogenes_1639_v120	Listeria_grayi_DSM_20601_525367	0.7147
Listeria_monocytogenes_1639_v11	Listeria_grayi_DSM_20601_525367	0.6952
Listeria_monocytogenes_1639_v119	Listeria_grayi_DSM_20601_525367	0.715
Listeria_monocytogenes_1639_v118	Listeria_grayi_DSM_20601_525367	0.715
Listeria_monocytogenes_1639_v117	Listeria_grayi_DSM_20601_525367	0.7092
Listeria_monocytogenes_1639_v116	Listeria_grayi_DSM_20601_525367	0.7083
Listeria_monocytogenes_1639_v115	Listeria_grayi_DSM_20601_525367	0.7085
Listeria_monocytogenes_1639_v114	Listeria_grayi_DSM_20601_525367	0.7083

Listeria_monocytogenes_1639_v113	Listeria_grayi_DSM_20601_525367	0.7134
Listeria_monocytogenes_1639_v112	Listeria_grayi_DSM_20601_525367	0.7055
Listeria_monocytogenes_1639_v111	Listeria_grayi_DSM_20601_525367	0.7085
Listeria_monocytogenes_1639_v110	Listeria_grayi_DSM_20601_525367	0.7089
Listeria_monocytogenes_1639_v10	Listeria_grayi_DSM_20601_525367	0.6895
Listeria_monocytogenes_1639_v109	Listeria_grayi_DSM_20601_525367	0.7077
Listeria_monocytogenes_1639_v108	Listeria_grayi_DSM_20601_525367	0.7067
Listeria_monocytogenes_1639_v107	Listeria_grayi_DSM_20601_525367	0.7112
Listeria_monocytogenes_1639_v106	Listeria_grayi_DSM_20601_525367	0.7116
Listeria_monocytogenes_1639_v105	Listeria_grayi_DSM_20601_525367	0.7116
Listeria_monocytogenes_1639_v104	Listeria_grayi_DSM_20601_525367	0.7118
Listeria_monocytogenes_1639_v103	Listeria_grayi_DSM_20601_525367	0.7118
Listeria_monocytogenes_1639_v102	Listeria_grayi_DSM_20601_525367	0.7059
Listeria_monocytogenes_1639_v101	Listeria_grayi_DSM_20601_525367	0.7071
Listeria_monocytogenes_1639_v100	Listeria_grayi_DSM_20601_525367	0.7066
Listeria_monocytogenes_1639	Listeria_grayi_DSM_20601_525367	0.7216
Listeria_monocytogenes_10403S_393133	Listeria_grayi_DSM_20601_525367	0.7159
Listeria_monocytogenes_08-5923_637381	Listeria_grayi_DSM_20601_525367	0.6997
Listeria_monocytogenes_08-5578_653938	Listeria_grayi_DSM_20601_525367	0.698
Listeria_monocytogenes_07PF0776_1126011	Listeria_grayi_DSM_20601_525367	0.7114
Listeria_marthii_FSL_S4-120_702457	Listeria_grayi_DSM_20601_525367	0.675
Listeria_marthii_529731_v1	Listeria_grayi_DSM_20601_525367	0.6993
Listeria_marthii_529731	Listeria_grayi_DSM_20601_525367	0.7076
Listeria_ivanovii_WSLC3009_1457190	Listeria_grayi_DSM_20601_525367	0.7074
Listeria_ivanovii_subsp_londoniensis_202752_v2	Listeria_grayi_DSM_20601_525367	0.7062
Listeria_ivanovii_subsp_londoniensis_202752_v1	Listeria_grayi_DSM_20601_525367	0.6972
Listeria_ivanovii_subsp_londoniensis_202752	Listeria_grayi_DSM_20601_525367	0.7017
Listeria_ivanovii_subsp_ivanovii_PAM_55_881621	Listeria_grayi_DSM_20601_525367	0.7067
Listeria_ivanovii_subsp_ivanovii_202751_v2	Listeria_grayi_DSM_20601_525367	0.708
Listeria_ivanovii_subsp_ivanovii_202751_v1	Listeria_grayi_DSM_20601_525367	0.708
Listeria_ivanovii_subsp_ivanovii_202751	Listeria_grayi_DSM_20601_525367	0.708
Listeria_ivanovii_FSL_F6-596_702454	Listeria_grayi_DSM_20601_525367	0.6529
Listeria_ivanovii_1638	Listeria_grayi_DSM_20601_525367	0.7077
Listeria_innocua_FSL_S4-378_702456	Listeria_grayi_DSM_20601_525367	0.648
Listeria_innocua_FSL_J1-023_702455	Listeria_grayi_DSM_20601_525367	0.6805
Listeria_innocua_Clip11262_272626	Listeria_grayi_DSM_20601_525367	0.6909
Listeria_innocua_1642_v4	Listeria_grayi_DSM_20601_525367	0.6922
Listeria_innocua_1642_v3	Listeria_grayi_DSM_20601_525367	0.7061
Listeria_innocua_1642_v2	Listeria_grayi_DSM_20601_525367	0.7071
Listeria_innocua_1642_v1	Listeria_grayi_DSM_20601_525367	0.71
Listeria_innocua_1642	Listeria_grayi_DSM_20601_525367	0.706
Listeria_grayi_DSM_20601_525367	Listeria_grayi_DSM_20601_525367	0.0533
Listeria_welshimeri_1643	Listeria_innocua_1642	0.8745
Listeria_welshimeri_serovar_6b_str_SLCC5334_386043	Listeria_innocua_1642	0.8742
Listeria_seeligeri_serovar_1_2b_str_SLCC3954_683837	Listeria_innocua_1642	0.8685
Listeria_seeligeri_FSL_S4-171_702452	Listeria_innocua_1642	0.8244
Listeria_seeligeri_FSL_N1-067_702453	Listeria_innocua_1642	0.8205
Listeria_seeligeri_1640_v1	Listeria_innocua_1642	0.8705
Listeria_seeligeri_1640	Listeria_innocua_1642	0.87
Listeria_monocytogenes_WSLC1042_1457188	Listeria_innocua_1642	0.91
Listeria_monocytogenes_WSLC1001_1457187	Listeria_innocua_1642	0.9051
Listeria_monocytogenes_SLCC7179_879090	Listeria_innocua_1642	0.9134
Listeria_monocytogenes_SLCC5850_882096	Listeria_innocua_1642	0.9092
Listeria_monocytogenes_SLCC2755_932919	Listeria_innocua_1642	0.895
Listeria_monocytogenes_SLCC2540_879089	Listeria_innocua_1642	0.9066
Listeria_monocytogenes_SLCC2479_882020	Listeria_innocua_1642	0.9102
Listeria_monocytogenes_SLCC2378_879088	Listeria_innocua_1642	0.9107
Listeria_monocytogenes_SLCC2376_882097	Listeria_innocua_1642	0.9241
Listeria_monocytogenes_SLCC2372_932920	Listeria_innocua_1642	0.9028
Listeria_monocytogenes_serotype_4b_str_LL195_1230340	Listeria_innocua_1642	0.9162
Listeria_monocytogenes_serotype_4b_str_LIS0087_1134669	Listeria_innocua_1642	0.8908
Listeria_monocytogenes_serotype_4b_str_F2365_265669	Listeria_innocua_1642	0.9162
Listeria_monocytogenes_serotype_4b_str_CLIP_80459_568819	Listeria_innocua_1642	0.9172
Listeria_monocytogenes_serotype_4b_str_81-0861_1196161	Listeria_innocua_1642	0.9109
Listeria_monocytogenes_serotype_4b_str_81-0592_1196160	Listeria_innocua_1642	0.912
Listeria_monocytogenes_serotype_4b_str_81-0558_1196159	Listeria_innocua_1642	0.9123
Listeria_monocytogenes_serotype_4b_str_10-0809_1196162	Listeria_innocua_1642	0.9123
Listeria_monocytogenes_serotype_4b_str_02-6680_1196175	Listeria_innocua_1642	0.9159
Listeria_monocytogenes_serotype_4b_str_02-6679_1196174	Listeria_innocua_1642	0.9159
Listeria_monocytogenes_serotype_4b_str_02-1792_1196173	Listeria_innocua_1642	0.9127
Listeria_monocytogenes_serotype_4b_str_02-1289_1196172	Listeria_innocua_1642	0.9127
Listeria_monocytogenes_serotype_4b_str_02-1103_1196171	Listeria_innocua_1642	0.9127
Listeria_monocytogenes_serotype_3c_str_10-5027_1196195	Listeria_innocua_1642	0.9091
Listeria_monocytogenes_serotype_1_2c_str_10-5026_1196194	Listeria_innocua_1642	0.9095
Listeria_monocytogenes_serotype_1_2c_str_10-5025_1196193	Listeria_innocua_1642	0.9095
Listeria_monocytogenes_serotype_1_2b_str_10-0811_1196164	Listeria_innocua_1642	0.9065
Listeria_monocytogenes_serotype_1_2b_str_10-0810_1196163	Listeria_innocua_1642	0.9065
Listeria_monocytogenes_serotype_1_2a_str_99-6370_1196179	Listeria_innocua_1642	0.8963
Listeria_monocytogenes_serotype_1_2a_str_98-2035_1196178	Listeria_innocua_1642	0.8963
Listeria_monocytogenes_serotype_1_2a_str_95-0093_1196177	Listeria_innocua_1642	0.9001
Listeria_monocytogenes_serotype_1_2a_str_88-0478_1196176	Listeria_innocua_1642	0.8993
Listeria_monocytogenes_serotype_1_2a_str_10-5024_1196192	Listeria_innocua_1642	0.9001
Listeria_monocytogenes_serotype_1_2a_str_10-4758_1196168	Listeria_innocua_1642	0.9082
Listeria_monocytogenes_serotype_1_2a_str_10-4754_1196167	Listeria_innocua_1642	0.9086
Listeria_monocytogenes_serotype_1_2a_str_10-1321_1196191	Listeria_innocua_1642	0.9001
Listeria_monocytogenes_serotype_1_2a_str_10-1047_1196190	Listeria_innocua_1642	0.9007
Listeria_monocytogenes_serotype_1_2a_str_10-1046_1196189	Listeria_innocua_1642	0.9007
Listeria_monocytogenes_serotype_1_2a_str_10-0934_1196170	Listeria_innocua_1642	0.9185
Listeria_monocytogenes_serotype_1_2a_str_10-0933_1196169	Listeria_innocua_1642	0.9185
Listeria_monocytogenes_serotype_1_2a_str_10-0815_1196188	Listeria_innocua_1642	0.8965
Listeria_monocytogenes_serotype_1_2a_str_10-0814_1196187	Listeria_innocua_1642	0.9004

Listeria_monocytogenes_serotype_1_2a_str_10-0813_1196166	Listeria_innocua_1642	0.9095
Listeria_monocytogenes_serotype_1_2a_str_10-0812_1196165	Listeria_innocua_1642	0.9095
Listeria_monocytogenes_serotype_1_2a_str_08-7669_1196186	Listeria_innocua_1642	0.9088
Listeria_monocytogenes_serotype_1_2a_str_08-7374_1196185	Listeria_innocua_1642	0.9001
Listeria_monocytogenes_serotype_1_2a_str_08-6997_1196184	Listeria_innocua_1642	0.8965
Listeria_monocytogenes_serotype_1_2a_str_08-6569_1196183	Listeria_innocua_1642	0.8965
Listeria_monocytogenes_serotype_1_2a_str_08-6056_1196182	Listeria_innocua_1642	0.8965
Listeria_monocytogenes_serotype_1_2a_str_04-5457_1196181	Listeria_innocua_1642	0.9001
Listeria_monocytogenes_serotype_1_2a_str_02-5993_1196180	Listeria_innocua_1642	0.9001
Listeria_monocytogenes_R479a_1437838	Listeria_innocua_1642	0.8964
Listeria_monocytogenes_N53-1_1234142	Listeria_innocua_1642	0.8727
Listeria_monocytogenes_M7_1030009	Listeria_innocua_1642	0.8973
Listeria_monocytogenes_LO28_393132	Listeria_innocua_1642	0.9052
Listeria_monocytogenes_L99_563174	Listeria_innocua_1642	0.8976
Listeria_monocytogenes_L312_882094	Listeria_innocua_1642	0.9172
Listeria_monocytogenes_J1816_930781	Listeria_innocua_1642	0.9172
Listeria_monocytogenes_J1-220_930782	Listeria_innocua_1642	0.9063
Listeria_monocytogenes_J0161_393130	Listeria_innocua_1642	0.9104
Listeria_monocytogenes_HCC23_552536	Listeria_innocua_1642	0.8973
Listeria_monocytogenes_FSL_R2-561_393126	Listeria_innocua_1642	0.9074
Listeria_monocytogenes_FSL_J1-208_393119	Listeria_innocua_1642	0.9008
Listeria_monocytogenes_FSL_J1-175_393118	Listeria_innocua_1642	0.9104
Listeria_monocytogenes_Finland_1998_393127	Listeria_innocua_1642	0.9131
Listeria_monocytogenes_EGD-e_169963_v1	Listeria_innocua_1642	0.9155
Listeria_monocytogenes_EGD-e_169963	Listeria_innocua_1642	0.9159
Listeria_monocytogenes_EGD_1334565	Listeria_innocua_1642	0.91
Listeria_monocytogenes_CFSAN002349_1299895	Listeria_innocua_1642	0.9163
Listeria_monocytogenes_ATCC_19117_882095_v1	Listeria_innocua_1642	0.9142
Listeria_monocytogenes_ATCC_19117_882095	Listeria_innocua_1642	0.9138
Listeria_monocytogenes_6179_1288295	Listeria_innocua_1642	0.9013
Listeria_monocytogenes_1639_v9	Listeria_innocua_1642	0.8972
Listeria_monocytogenes_1639_v99	Listeria_innocua_1642	0.9179
Listeria_monocytogenes_1639_v98	Listeria_innocua_1642	0.9092
Listeria_monocytogenes_1639_v97	Listeria_innocua_1642	0.9206
Listeria_monocytogenes_1639_v96	Listeria_innocua_1642	0.9165
Listeria_monocytogenes_1639_v95	Listeria_innocua_1642	0.9165
Listeria_monocytogenes_1639_v94	Listeria_innocua_1642	0.9169
Listeria_monocytogenes_1639_v93	Listeria_innocua_1642	0.8929
Listeria_monocytogenes_1639_v92	Listeria_innocua_1642	0.9191
Listeria_monocytogenes_1639_v91	Listeria_innocua_1642	0.9081
Listeria_monocytogenes_1639_v90	Listeria_innocua_1642	0.9092
Listeria_monocytogenes_1639_v8	Listeria_innocua_1642	0.8888
Listeria_monocytogenes_1639_v89	Listeria_innocua_1642	0.9158
Listeria_monocytogenes_1639_v88	Listeria_innocua_1642	0.9134
Listeria_monocytogenes_1639_v87	Listeria_innocua_1642	0.904
Listeria_monocytogenes_1639_v86	Listeria_innocua_1642	0.9037
Listeria_monocytogenes_1639_v85	Listeria_innocua_1642	0.9114
Listeria_monocytogenes_1639_v84	Listeria_innocua_1642	0.9094
Listeria_monocytogenes_1639_v83	Listeria_innocua_1642	0.9129
Listeria_monocytogenes_1639_v82	Listeria_innocua_1642	0.9123
Listeria_monocytogenes_1639_v81	Listeria_innocua_1642	0.9066
Listeria_monocytogenes_1639_v80	Listeria_innocua_1642	0.9102
Listeria_monocytogenes_1639_v7	Listeria_innocua_1642	0.8888
Listeria_monocytogenes_1639_v79	Listeria_innocua_1642	0.9053
Listeria_monocytogenes_1639_v78	Listeria_innocua_1642	0.9067
Listeria_monocytogenes_1639_v77	Listeria_innocua_1642	0.9093
Listeria_monocytogenes_1639_v76	Listeria_innocua_1642	0.907
Listeria_monocytogenes_1639_v75	Listeria_innocua_1642	0.8976
Listeria_monocytogenes_1639_v74	Listeria_innocua_1642	0.9003
Listeria_monocytogenes_1639_v73	Listeria_innocua_1642	0.8993
Listeria_monocytogenes_1639_v72	Listeria_innocua_1642	0.9129
Listeria_monocytogenes_1639_v71	Listeria_innocua_1642	0.8947
Listeria_monocytogenes_1639_v70	Listeria_innocua_1642	0.9088
Listeria_monocytogenes_1639_v6	Listeria_innocua_1642	0.8885
Listeria_monocytogenes_1639_v69	Listeria_innocua_1642	0.912
Listeria_monocytogenes_1639_v68	Listeria_innocua_1642	0.9021
Listeria_monocytogenes_1639_v67	Listeria_innocua_1642	0.9138
Listeria_monocytogenes_1639_v66	Listeria_innocua_1642	0.9057
Listeria_monocytogenes_1639_v65	Listeria_innocua_1642	0.899
Listeria_monocytogenes_1639_v64	Listeria_innocua_1642	0.8989
Listeria_monocytogenes_1639_v63	Listeria_innocua_1642	0.9087
Listeria_monocytogenes_1639_v62	Listeria_innocua_1642	0.9067
Listeria_monocytogenes_1639_v61	Listeria_innocua_1642	0.9038
Listeria_monocytogenes_1639_v60	Listeria_innocua_1642	0.9107
Listeria_monocytogenes_1639_v5	Listeria_innocua_1642	0.8738
Listeria_monocytogenes_1639_v59	Listeria_innocua_1642	0.9077
Listeria_monocytogenes_1639_v58	Listeria_innocua_1642	0.9152
Listeria_monocytogenes_1639_v57	Listeria_innocua_1642	0.9143
Listeria_monocytogenes_1639_v56	Listeria_innocua_1642	0.9204
Listeria_monocytogenes_1639_v55	Listeria_innocua_1642	0.9138
Listeria_monocytogenes_1639_v54	Listeria_innocua_1642	0.9138
Listeria_monocytogenes_1639_v53	Listeria_innocua_1642	0.8991
Listeria_monocytogenes_1639_v52	Listeria_innocua_1642	0.9132
Listeria_monocytogenes_1639_v51	Listeria_innocua_1642	0.9075
Listeria_monocytogenes_1639_v50	Listeria_innocua_1642	0.8993
Listeria_monocytogenes_1639_v4	Listeria_innocua_1642	0.8861
Listeria_monocytogenes_1639_v49	Listeria_innocua_1642	0.9027
Listeria_monocytogenes_1639_v48	Listeria_innocua_1642	0.9063
Listeria_monocytogenes_1639_v47	Listeria_innocua_1642	0.8997
Listeria_monocytogenes_1639_v46	Listeria_innocua_1642	0.907
Listeria_monocytogenes_1639_v45	Listeria_innocua_1642	0.8912

Listeria_monocytogenes_1639_v44	Listeria_innocua_1642	0.9001
Listeria_monocytogenes_1639_v43	Listeria_innocua_1642	0.9001
Listeria_monocytogenes_1639_v42	Listeria_innocua_1642	0.8984
Listeria_monocytogenes_1639_v41	Listeria_innocua_1642	0.8824
Listeria_monocytogenes_1639_v40	Listeria_innocua_1642	0.9018
Listeria_monocytogenes_1639_v3	Listeria_innocua_1642	0.8865
Listeria_monocytogenes_1639_v39	Listeria_innocua_1642	0.9112
Listeria_monocytogenes_1639_v38	Listeria_innocua_1642	0.8976
Listeria_monocytogenes_1639_v37	Listeria_innocua_1642	0.8944
Listeria_monocytogenes_1639_v36	Listeria_innocua_1642	0.8916
Listeria_monocytogenes_1639_v35	Listeria_innocua_1642	0.9024
Listeria_monocytogenes_1639_v34	Listeria_innocua_1642	0.9026
Listeria_monocytogenes_1639_v33	Listeria_innocua_1642	0.9065
Listeria_monocytogenes_1639_v32	Listeria_innocua_1642	0.8968
Listeria_monocytogenes_1639_v31	Listeria_innocua_1642	0.9029
Listeria_monocytogenes_1639_v30	Listeria_innocua_1642	0.9004
Listeria_monocytogenes_1639_v2	Listeria_innocua_1642	0.8734
Listeria_monocytogenes_1639_v29	Listeria_innocua_1642	0.8958
Listeria_monocytogenes_1639_v28	Listeria_innocua_1642	0.9034
Listeria_monocytogenes_1639_v27	Listeria_innocua_1642	0.8864
Listeria_monocytogenes_1639_v26	Listeria_innocua_1642	0.8858
Listeria_monocytogenes_1639_v25	Listeria_innocua_1642	0.8865
Listeria_monocytogenes_1639_v24	Listeria_innocua_1642	0.9063
Listeria_monocytogenes_1639_v23	Listeria_innocua_1642	0.906
Listeria_monocytogenes_1639_v22	Listeria_innocua_1642	0.9128
Listeria_monocytogenes_1639_v21	Listeria_innocua_1642	0.9045
Listeria_monocytogenes_1639_v20	Listeria_innocua_1642	0.9004
Listeria_monocytogenes_1639_v1	Listeria_innocua_1642	0.8952
Listeria_monocytogenes_1639_v19	Listeria_innocua_1642	0.9001
Listeria_monocytogenes_1639_v198	Listeria_innocua_1642	0.8734
Listeria_monocytogenes_1639_v197	Listeria_innocua_1642	0.9046
Listeria_monocytogenes_1639_v196	Listeria_innocua_1642	0.8935
Listeria_monocytogenes_1639_v195	Listeria_innocua_1642	0.9205
Listeria_monocytogenes_1639_v194	Listeria_innocua_1642	0.9245
Listeria_monocytogenes_1639_v193	Listeria_innocua_1642	0.9241
Listeria_monocytogenes_1639_v192	Listeria_innocua_1642	0.8929
Listeria_monocytogenes_1639_v191	Listeria_innocua_1642	0.9156
Listeria_monocytogenes_1639_v190	Listeria_innocua_1642	0.9184
Listeria_monocytogenes_1639_v18	Listeria_innocua_1642	0.9118
Listeria_monocytogenes_1639_v189	Listeria_innocua_1642	0.9172
Listeria_monocytogenes_1639_v188	Listeria_innocua_1642	0.9151
Listeria_monocytogenes_1639_v187	Listeria_innocua_1642	0.9183
Listeria_monocytogenes_1639_v186	Listeria_innocua_1642	0.9183
Listeria_monocytogenes_1639_v185	Listeria_innocua_1642	0.9187
Listeria_monocytogenes_1639_v184	Listeria_innocua_1642	0.9176
Listeria_monocytogenes_1639_v183	Listeria_innocua_1642	0.9099
Listeria_monocytogenes_1639_v182	Listeria_innocua_1642	0.9113
Listeria_monocytogenes_1639_v181	Listeria_innocua_1642	0.912
Listeria_monocytogenes_1639_v180	Listeria_innocua_1642	0.912
Listeria_monocytogenes_1639_v17	Listeria_innocua_1642	0.9122
Listeria_monocytogenes_1639_v179	Listeria_innocua_1642	0.9123
Listeria_monocytogenes_1639_v178	Listeria_innocua_1642	0.9122
Listeria_monocytogenes_1639_v177	Listeria_innocua_1642	0.919
Listeria_monocytogenes_1639_v176	Listeria_innocua_1642	0.9131
Listeria_monocytogenes_1639_v175	Listeria_innocua_1642	0.9085
Listeria_monocytogenes_1639_v174	Listeria_innocua_1642	0.9166
Listeria_monocytogenes_1639_v173	Listeria_innocua_1642	0.9185
Listeria_monocytogenes_1639_v172	Listeria_innocua_1642	0.9193
Listeria_monocytogenes_1639_v171	Listeria_innocua_1642	0.9068
Listeria_monocytogenes_1639_v170	Listeria_innocua_1642	0.9147
Listeria_monocytogenes_1639_v16	Listeria_innocua_1642	0.8873
Listeria_monocytogenes_1639_v169	Listeria_innocua_1642	0.9099
Listeria_monocytogenes_1639_v168	Listeria_innocua_1642	0.9083
Listeria_monocytogenes_1639_v167	Listeria_innocua_1642	0.9178
Listeria_monocytogenes_1639_v166	Listeria_innocua_1642	0.9082
Listeria_monocytogenes_1639_v165	Listeria_innocua_1642	0.9095
Listeria_monocytogenes_1639_v164	Listeria_innocua_1642	0.9157
Listeria_monocytogenes_1639_v163	Listeria_innocua_1642	0.9116
Listeria_monocytogenes_1639_v162	Listeria_innocua_1642	0.9157
Listeria_monocytogenes_1639_v161	Listeria_innocua_1642	0.9165
Listeria_monocytogenes_1639_v160	Listeria_innocua_1642	0.9162
Listeria_monocytogenes_1639_v15	Listeria_innocua_1642	0.8919
Listeria_monocytogenes_1639_v159	Listeria_innocua_1642	0.919
Listeria_monocytogenes_1639_v158	Listeria_innocua_1642	0.9162
Listeria_monocytogenes_1639_v157	Listeria_innocua_1642	0.9162
Listeria_monocytogenes_1639_v156	Listeria_innocua_1642	0.9038
Listeria_monocytogenes_1639_v155	Listeria_innocua_1642	0.9173
Listeria_monocytogenes_1639_v154	Listeria_innocua_1642	0.906
Listeria_monocytogenes_1639_v153	Listeria_innocua_1642	0.9124
Listeria_monocytogenes_1639_v152	Listeria_innocua_1642	0.919
Listeria_monocytogenes_1639_v151	Listeria_innocua_1642	0.919
Listeria_monocytogenes_1639_v150	Listeria_innocua_1642	0.9101
Listeria_monocytogenes_1639_v14	Listeria_innocua_1642	0.8985
Listeria_monocytogenes_1639_v149	Listeria_innocua_1642	0.9103
Listeria_monocytogenes_1639_v148	Listeria_innocua_1642	0.9179
Listeria_monocytogenes_1639_v147	Listeria_innocua_1642	0.9056
Listeria_monocytogenes_1639_v146	Listeria_innocua_1642	0.9136
Listeria_monocytogenes_1639_v145	Listeria_innocua_1642	0.9104
Listeria_monocytogenes_1639_v144	Listeria_innocua_1642	0.9165
Listeria_monocytogenes_1639_v143	Listeria_innocua_1642	0.9149
Listeria_monocytogenes_1639_v142	Listeria_innocua_1642	0.9169

Listeria_monocytogenes_1639_v141	Listeria_innocua_1642	0.9156
Listeria_monocytogenes_1639_v140	Listeria_innocua_1642	0.9176
Listeria_monocytogenes_1639_v13	Listeria_innocua_1642	0.8919
Listeria_monocytogenes_1639_v139	Listeria_innocua_1642	0.9154
Listeria_monocytogenes_1639_v138	Listeria_innocua_1642	0.9184
Listeria_monocytogenes_1639_v137	Listeria_innocua_1642	0.9184
Listeria_monocytogenes_1639_v136	Listeria_innocua_1642	0.9086
Listeria_monocytogenes_1639_v135	Listeria_innocua_1642	0.9075
Listeria_monocytogenes_1639_v134	Listeria_innocua_1642	0.9104
Listeria_monocytogenes_1639_v133	Listeria_innocua_1642	0.9189
Listeria_monocytogenes_1639_v132	Listeria_innocua_1642	0.902
Listeria_monocytogenes_1639_v131	Listeria_innocua_1642	0.916
Listeria_monocytogenes_1639_v130	Listeria_innocua_1642	0.9002
Listeria_monocytogenes_1639_v12	Listeria_innocua_1642	0.8929
Listeria_monocytogenes_1639_v129	Listeria_innocua_1642	0.9176
Listeria_monocytogenes_1639_v128	Listeria_innocua_1642	0.9122
Listeria_monocytogenes_1639_v127	Listeria_innocua_1642	0.9136
Listeria_monocytogenes_1639_v126	Listeria_innocua_1642	0.9107
Listeria_monocytogenes_1639_v125	Listeria_innocua_1642	0.9121
Listeria_monocytogenes_1639_v124	Listeria_innocua_1642	0.9111
Listeria_monocytogenes_1639_v123	Listeria_innocua_1642	0.9163
Listeria_monocytogenes_1639_v122	Listeria_innocua_1642	0.9163
Listeria_monocytogenes_1639_v121	Listeria_innocua_1642	0.9163
Listeria_monocytogenes_1639_v120	Listeria_innocua_1642	0.9163
Listeria_monocytogenes_1639_v11	Listeria_innocua_1642	0.8971
Listeria_monocytogenes_1639_v119	Listeria_innocua_1642	0.9163
Listeria_monocytogenes_1639_v118	Listeria_innocua_1642	0.9163
Listeria_monocytogenes_1639_v117	Listeria_innocua_1642	0.9049
Listeria_monocytogenes_1639_v116	Listeria_innocua_1642	0.9103
Listeria_monocytogenes_1639_v115	Listeria_innocua_1642	0.9107
Listeria_monocytogenes_1639_v114	Listeria_innocua_1642	0.9124
Listeria_monocytogenes_1639_v113	Listeria_innocua_1642	0.9129
Listeria_monocytogenes_1639_v112	Listeria_innocua_1642	0.9212
Listeria_monocytogenes_1639_v111	Listeria_innocua_1642	0.9006
Listeria_monocytogenes_1639_v110	Listeria_innocua_1642	0.9084
Listeria_monocytogenes_1639_v10	Listeria_innocua_1642	0.8935
Listeria_monocytogenes_1639_v109	Listeria_innocua_1642	0.9132
Listeria_monocytogenes_1639_v108	Listeria_innocua_1642	0.9177
Listeria_monocytogenes_1639_v107	Listeria_innocua_1642	0.9128
Listeria_monocytogenes_1639_v106	Listeria_innocua_1642	0.9132
Listeria_monocytogenes_1639_v105	Listeria_innocua_1642	0.9132
Listeria_monocytogenes_1639_v104	Listeria_innocua_1642	0.9132
Listeria_monocytogenes_1639_v103	Listeria_innocua_1642	0.9135
Listeria_monocytogenes_1639_v102	Listeria_innocua_1642	0.9076
Listeria_monocytogenes_1639_v101	Listeria_innocua_1642	0.8965
Listeria_monocytogenes_1639_v100	Listeria_innocua_1642	0.8951
Listeria_monocytogenes_1639	Listeria_innocua_1642	0.918
Listeria_monocytogenes_10403S_393133	Listeria_innocua_1642	0.9167
Listeria_monocytogenes_08-5923_637381	Listeria_innocua_1642	0.9001
Listeria_monocytogenes_08-5578_653938	Listeria_innocua_1642	0.8873
Listeria_monocytogenes_07PF0776_1126011	Listeria_innocua_1642	0.9131
Listeria_marthii_FSL_S4-120_702457	Listeria_innocua_1642	0.8514
Listeria_marthii_529731_v1	Listeria_innocua_1642	0.8958
Listeria_marthii_529731	Listeria_innocua_1642	0.8958
Listeria_ivanovii_WSLC3009_1457190	Listeria_innocua_1642	0.8714
Listeria_ivanovii_subsp_londoniensis_202752_v2	Listeria_innocua_1642	0.869
Listeria_ivanovii_subsp_londoniensis_202752_v1	Listeria_innocua_1642	0.8616
Listeria_ivanovii_subsp_londoniensis_202752	Listeria_innocua_1642	0.874
Listeria_ivanovii_subsp_ivanovii_PAM_55_881621	Listeria_innocua_1642	0.8702
Listeria_ivanovii_subsp_ivanovii_202751_v2	Listeria_innocua_1642	0.8718
Listeria_ivanovii_subsp_ivanovii_202751_v1	Listeria_innocua_1642	0.8718
Listeria_ivanovii_subsp_ivanovii_202751	Listeria_innocua_1642	0.8718
Listeria_ivanovii_FSL_F6-596_702454	Listeria_innocua_1642	0.8145
Listeria_ivanovii_1638	Listeria_innocua_1642	0.8718
Listeria_innocua_FSL_S4-378_702456	Listeria_innocua_1642	0.8381
Listeria_innocua_FSL_J1-023_702455	Listeria_innocua_1642	0.8753
Listeria_innocua_Clip11262_272626	Listeria_innocua_1642	0.9222
Listeria_innocua_1642_v4	Listeria_innocua_1642	0.9054
Listeria_innocua_1642_v3	Listeria_innocua_1642	0.9181
Listeria_innocua_1642_v2	Listeria_innocua_1642	0.9113
Listeria_innocua_1642_v1	Listeria_innocua_1642	0.9422
Listeria_innocua_1642	Listeria_innocua_1642	0.0749
Listeria_welshimeri_1643	Listeria_innocua_1642_v1	0.8726
Listeria_welshimeri_serovar_6b_str_SLCC5334_386043	Listeria_innocua_1642_v1	0.8719
Listeria_seeligeri_serovar_1_2b_str_SLCC3954_683837	Listeria_innocua_1642_v1	0.8674
Listeria_seeligeri_FSL_S4-171_702452	Listeria_innocua_1642_v1	0.8261
Listeria_seeligeri_FSL_N1-067_702453	Listeria_innocua_1642_v1	0.821
Listeria_seeligeri_1640_v1	Listeria_innocua_1642_v1	0.872
Listeria_seeligeri_1640	Listeria_innocua_1642_v1	0.8711
Listeria_monocytogenes_WSLC1042_1457188	Listeria_innocua_1642_v1	0.9103
Listeria_monocytogenes_WSLC1001_1457187	Listeria_innocua_1642_v1	0.9016
Listeria_monocytogenes_SLCC7179_879090	Listeria_innocua_1642_v1	0.9102
Listeria_monocytogenes_SLCC5850_882096	Listeria_innocua_1642_v1	0.9049
Listeria_monocytogenes_SLCC2755_932919	Listeria_innocua_1642_v1	0.9125
Listeria_monocytogenes_SLCC2540_879089	Listeria_innocua_1642_v1	0.9031
Listeria_monocytogenes_SLCC2479_882020	Listeria_innocua_1642_v1	0.9068
Listeria_monocytogenes_SLCC2378_879088	Listeria_innocua_1642_v1	0.9117
Listeria_monocytogenes_SLCC2376_882097	Listeria_innocua_1642_v1	0.922
Listeria_monocytogenes_SLCC2372_932920	Listeria_innocua_1642_v1	0.9145
Listeria_monocytogenes_serotype_4b_str_LL195_1230340	Listeria_innocua_1642_v1	0.9149
Listeria_monocytogenes_serotype_4b_str_LIS0087_1134669	Listeria_innocua_1642_v1	0.8855

Listeria_monocytogenes_serotype_4b_str_F2365_265669	Listeria_innocua_1642_v1	0.9149
Listeria_monocytogenes_serotype_4b_str_CLIP_80459_568819	Listeria_innocua_1642_v1	0.9182
Listeria_monocytogenes_serotype_4b_str_81-0861_1196161	Listeria_innocua_1642_v1	0.9078
Listeria_monocytogenes_serotype_4b_str_81-0592_1196160	Listeria_innocua_1642_v1	0.9089
Listeria_monocytogenes_serotype_4b_str_81-0558_1196159	Listeria_innocua_1642_v1	0.9099
Listeria_monocytogenes_serotype_4b_str_10-0809_1196162	Listeria_innocua_1642_v1	0.9092
Listeria_monocytogenes_serotype_4b_str_02-6680_1196175	Listeria_innocua_1642_v1	0.9139
Listeria_monocytogenes_serotype_4b_str_02-6679_1196174	Listeria_innocua_1642_v1	0.9139
Listeria_monocytogenes_serotype_4b_str_02-1792_1196173	Listeria_innocua_1642_v1	0.9088
Listeria_monocytogenes_serotype_4b_str_02-1289_1196172	Listeria_innocua_1642_v1	0.9088
Listeria_monocytogenes_serotype_4b_str_02-1103_1196171	Listeria_innocua_1642_v1	0.9088
Listeria_monocytogenes_serotype_3c_str_10-5027_1196195	Listeria_innocua_1642_v1	0.9071
Listeria_monocytogenes_serotype_1_2c_str_10-5026_1196194	Listeria_innocua_1642_v1	0.9079
Listeria_monocytogenes_serotype_1_2c_str_10-5025_1196193	Listeria_innocua_1642_v1	0.9079
Listeria_monocytogenes_serotype_1_2b_str_10-0811_1196164	Listeria_innocua_1642_v1	0.8968
Listeria_monocytogenes_serotype_1_2b_str_10-0810_1196163	Listeria_innocua_1642_v1	0.8968
Listeria_monocytogenes_serotype_1_2a_str_99-6370_1196179	Listeria_innocua_1642_v1	0.8922
Listeria_monocytogenes_serotype_1_2a_str_98-2035_1196178	Listeria_innocua_1642_v1	0.8922
Listeria_monocytogenes_serotype_1_2a_str_95-0093_1196177	Listeria_innocua_1642_v1	0.897
Listeria_monocytogenes_serotype_1_2a_str_88-0478_1196176	Listeria_innocua_1642_v1	0.8962
Listeria_monocytogenes_serotype_1_2a_str_10-5024_1196192	Listeria_innocua_1642_v1	0.8974
Listeria_monocytogenes_serotype_1_2a_str_10-4758_1196168	Listeria_innocua_1642_v1	0.9032
Listeria_monocytogenes_serotype_1_2a_str_10-4754_1196167	Listeria_innocua_1642_v1	0.9032
Listeria_monocytogenes_serotype_1_2a_str_10-1321_1196191	Listeria_innocua_1642_v1	0.8974
Listeria_monocytogenes_serotype_1_2a_str_10-1047_1196190	Listeria_innocua_1642_v1	0.8973
Listeria_monocytogenes_serotype_1_2a_str_10-1046_1196189	Listeria_innocua_1642_v1	0.8973
Listeria_monocytogenes_serotype_1_2a_str_10-0934_1196170	Listeria_innocua_1642_v1	0.9126
Listeria_monocytogenes_serotype_1_2a_str_10-0933_1196169	Listeria_innocua_1642_v1	0.9126
Listeria_monocytogenes_serotype_1_2a_str_10-0815_1196188	Listeria_innocua_1642_v1	0.8931
Listeria_monocytogenes_serotype_1_2a_str_10-0814_1196187	Listeria_innocua_1642_v1	0.8974
Listeria_monocytogenes_serotype_1_2a_str_10-0813_1196166	Listeria_innocua_1642_v1	0.9046
Listeria_monocytogenes_serotype_1_2a_str_10-0812_1196165	Listeria_innocua_1642_v1	0.9046
Listeria_monocytogenes_serotype_1_2a_str_08-7669_1196186	Listeria_innocua_1642_v1	0.9049
Listeria_monocytogenes_serotype_1_2a_str_08-7374_1196185	Listeria_innocua_1642_v1	0.8974
Listeria_monocytogenes_serotype_1_2a_str_08-6997_1196184	Listeria_innocua_1642_v1	0.8931
Listeria_monocytogenes_serotype_1_2a_str_08-6569_1196183	Listeria_innocua_1642_v1	0.8931
Listeria_monocytogenes_serotype_1_2a_str_08-6056_1196182	Listeria_innocua_1642_v1	0.8931
Listeria_monocytogenes_serotype_1_2a_str_04-5457_1196181	Listeria_innocua_1642_v1	0.8974
Listeria_monocytogenes_serotype_1_2a_str_02-5993_1196180	Listeria_innocua_1642_v1	0.897
Listeria_monocytogenes_R479a_1437838	Listeria_innocua_1642_v1	0.9041
Listeria_monocytogenes_N53-1_1234142	Listeria_innocua_1642_v1	0.8669
Listeria_monocytogenes_M7_1030009	Listeria_innocua_1642_v1	0.8913
Listeria_monocytogenes_LO28_393132	Listeria_innocua_1642_v1	0.9036
Listeria_monocytogenes_L99_563174	Listeria_innocua_1642_v1	0.8919
Listeria_monocytogenes_L312_882094	Listeria_innocua_1642_v1	0.9182
Listeria_monocytogenes_J1816_930781	Listeria_innocua_1642_v1	0.9122
Listeria_monocytogenes_J1-220_930782	Listeria_innocua_1642_v1	0.9007
Listeria_monocytogenes_J0161_393130	Listeria_innocua_1642_v1	0.9058
Listeria_monocytogenes_HCC23_552536	Listeria_innocua_1642_v1	0.8916
Listeria_monocytogenes_FSL_R2-561_393126	Listeria_innocua_1642_v1	0.9043
Listeria_monocytogenes_FSL_J1-208_393119	Listeria_innocua_1642_v1	0.9034
Listeria_monocytogenes_FSL_J1-175_393118	Listeria_innocua_1642_v1	0.9012
Listeria_monocytogenes_Finland_1998_393127	Listeria_innocua_1642_v1	0.9115
Listeria_monocytogenes_EGD-e_169963_v1	Listeria_innocua_1642_v1	0.9135
Listeria_monocytogenes_EGD-e_169963	Listeria_innocua_1642_v1	0.9139
Listeria_monocytogenes_EGD_1334565	Listeria_innocua_1642_v1	0.9057
Listeria_monocytogenes_CFSAN002349_1299895	Listeria_innocua_1642_v1	0.9128
Listeria_monocytogenes_ATCC_19117_882095_v1	Listeria_innocua_1642_v1	0.9126
Listeria_monocytogenes_ATCC_19117_882095	Listeria_innocua_1642_v1	0.9125
Listeria_monocytogenes_6179_1288295	Listeria_innocua_1642_v1	0.9016
Listeria_monocytogenes_1639_v9	Listeria_innocua_1642_v1	0.8861
Listeria_monocytogenes_1639_v99	Listeria_innocua_1642_v1	0.9219
Listeria_monocytogenes_1639_v98	Listeria_innocua_1642_v1	0.9075
Listeria_monocytogenes_1639_v97	Listeria_innocua_1642_v1	0.9115
Listeria_monocytogenes_1639_v96	Listeria_innocua_1642_v1	0.9219
Listeria_monocytogenes_1639_v95	Listeria_innocua_1642_v1	0.922
Listeria_monocytogenes_1639_v94	Listeria_innocua_1642_v1	0.9223
Listeria_monocytogenes_1639_v93	Listeria_innocua_1642_v1	0.8973
Listeria_monocytogenes_1639_v92	Listeria_innocua_1642_v1	0.9189
Listeria_monocytogenes_1639_v91	Listeria_innocua_1642_v1	0.9039
Listeria_monocytogenes_1639_v90	Listeria_innocua_1642_v1	0.9075
Listeria_monocytogenes_1639_v8	Listeria_innocua_1642_v1	0.8979
Listeria_monocytogenes_1639_v89	Listeria_innocua_1642_v1	0.9104
Listeria_monocytogenes_1639_v88	Listeria_innocua_1642_v1	0.9062
Listeria_monocytogenes_1639_v87	Listeria_innocua_1642_v1	0.8991
Listeria_monocytogenes_1639_v86	Listeria_innocua_1642_v1	0.8987
Listeria_monocytogenes_1639_v85	Listeria_innocua_1642_v1	0.9072
Listeria_monocytogenes_1639_v84	Listeria_innocua_1642_v1	0.9074
Listeria_monocytogenes_1639_v83	Listeria_innocua_1642_v1	0.9091
Listeria_monocytogenes_1639_v82	Listeria_innocua_1642_v1	0.9084
Listeria_monocytogenes_1639_v81	Listeria_innocua_1642_v1	0.905
Listeria_monocytogenes_1639_v80	Listeria_innocua_1642_v1	0.8992
Listeria_monocytogenes_1639_v7	Listeria_innocua_1642_v1	0.8979
Listeria_monocytogenes_1639_v79	Listeria_innocua_1642_v1	0.9041
Listeria_monocytogenes_1639_v78	Listeria_innocua_1642_v1	0.9047
Listeria_monocytogenes_1639_v77	Listeria_innocua_1642_v1	0.9119
Listeria_monocytogenes_1639_v76	Listeria_innocua_1642_v1	0.8984
Listeria_monocytogenes_1639_v75	Listeria_innocua_1642_v1	0.8919
Listeria_monocytogenes_1639_v74	Listeria_innocua_1642_v1	0.9017
Listeria_monocytogenes_1639_v73	Listeria_innocua_1642_v1	0.9078
Listeria_monocytogenes_1639_v72	Listeria_innocua_1642_v1	0.9071

Listeria_monocytogenes_1639_v71	Listeria_innocua_1642_v1	0.8939
Listeria_monocytogenes_1639_v70	Listeria_innocua_1642_v1	0.9094
Listeria_monocytogenes_1639_v6	Listeria_innocua_1642_v1	0.8972
Listeria_monocytogenes_1639_v69	Listeria_innocua_1642_v1	0.9092
Listeria_monocytogenes_1639_v68	Listeria_innocua_1642_v1	0.9046
Listeria_monocytogenes_1639_v67	Listeria_innocua_1642_v1	0.9054
Listeria_monocytogenes_1639_v66	Listeria_innocua_1642_v1	0.9007
Listeria_monocytogenes_1639_v65	Listeria_innocua_1642_v1	0.8963
Listeria_monocytogenes_1639_v64	Listeria_innocua_1642_v1	0.8984
Listeria_monocytogenes_1639_v63	Listeria_innocua_1642_v1	0.9086
Listeria_monocytogenes_1639_v62	Listeria_innocua_1642_v1	0.9028
Listeria_monocytogenes_1639_v61	Listeria_innocua_1642_v1	0.8996
Listeria_monocytogenes_1639_v60	Listeria_innocua_1642_v1	0.9065
Listeria_monocytogenes_1639_v5	Listeria_innocua_1642_v1	0.8805
Listeria_monocytogenes_1639_v59	Listeria_innocua_1642_v1	0.9053
Listeria_monocytogenes_1639_v58	Listeria_innocua_1642_v1	0.9098
Listeria_monocytogenes_1639_v57	Listeria_innocua_1642_v1	0.9023
Listeria_monocytogenes_1639_v56	Listeria_innocua_1642_v1	0.9143
Listeria_monocytogenes_1639_v55	Listeria_innocua_1642_v1	0.9163
Listeria_monocytogenes_1639_v54	Listeria_innocua_1642_v1	0.9092
Listeria_monocytogenes_1639_v53	Listeria_innocua_1642_v1	0.9005
Listeria_monocytogenes_1639_v52	Listeria_innocua_1642_v1	0.9049
Listeria_monocytogenes_1639_v51	Listeria_innocua_1642_v1	0.9118
Listeria_monocytogenes_1639_v50	Listeria_innocua_1642_v1	0.8963
Listeria_monocytogenes_1639_v4	Listeria_innocua_1642_v1	0.8922
Listeria_monocytogenes_1639_v49	Listeria_innocua_1642_v1	0.9066
Listeria_monocytogenes_1639_v48	Listeria_innocua_1642_v1	0.9025
Listeria_monocytogenes_1639_v47	Listeria_innocua_1642_v1	0.8967
Listeria_monocytogenes_1639_v46	Listeria_innocua_1642_v1	0.9095
Listeria_monocytogenes_1639_v45	Listeria_innocua_1642_v1	0.8966
Listeria_monocytogenes_1639_v44	Listeria_innocua_1642_v1	0.8974
Listeria_monocytogenes_1639_v43	Listeria_innocua_1642_v1	0.8974
Listeria_monocytogenes_1639_v42	Listeria_innocua_1642_v1	0.8961
Listeria_monocytogenes_1639_v41	Listeria_innocua_1642_v1	0.894
Listeria_monocytogenes_1639_v40	Listeria_innocua_1642_v1	0.9039
Listeria_monocytogenes_1639_v3	Listeria_innocua_1642_v1	0.893
Listeria_monocytogenes_1639_v39	Listeria_innocua_1642_v1	0.901
Listeria_monocytogenes_1639_v38	Listeria_innocua_1642_v1	0.8957
Listeria_monocytogenes_1639_v37	Listeria_innocua_1642_v1	0.8899
Listeria_monocytogenes_1639_v36	Listeria_innocua_1642_v1	0.903
Listeria_monocytogenes_1639_v35	Listeria_innocua_1642_v1	0.9004
Listeria_monocytogenes_1639_v34	Listeria_innocua_1642_v1	0.9017
Listeria_monocytogenes_1639_v33	Listeria_innocua_1642_v1	0.8968
Listeria_monocytogenes_1639_v32	Listeria_innocua_1642_v1	0.8941
Listeria_monocytogenes_1639_v31	Listeria_innocua_1642_v1	0.9021
Listeria_monocytogenes_1639_v30	Listeria_innocua_1642_v1	0.8943
Listeria_monocytogenes_1639_v2	Listeria_innocua_1642_v1	0.8866
Listeria_monocytogenes_1639_v29	Listeria_innocua_1642_v1	0.9042
Listeria_monocytogenes_1639_v28	Listeria_innocua_1642_v1	0.8993
Listeria_monocytogenes_1639_v27	Listeria_innocua_1642_v1	0.9018
Listeria_monocytogenes_1639_v26	Listeria_innocua_1642_v1	0.9014
Listeria_monocytogenes_1639_v25	Listeria_innocua_1642_v1	0.9011
Listeria_monocytogenes_1639_v24	Listeria_innocua_1642_v1	0.9007
Listeria_monocytogenes_1639_v23	Listeria_innocua_1642_v1	0.9007
Listeria_monocytogenes_1639_v22	Listeria_innocua_1642_v1	0.9022
Listeria_monocytogenes_1639_v21	Listeria_innocua_1642_v1	0.9014
Listeria_monocytogenes_1639_v20	Listeria_innocua_1642_v1	0.9158
Listeria_monocytogenes_1639_v1	Listeria_innocua_1642_v1	0.8962
Listeria_monocytogenes_1639_v19	Listeria_innocua_1642_v1	0.9151
Listeria_monocytogenes_1639_v198	Listeria_innocua_1642_v1	0.869
Listeria_monocytogenes_1639_v197	Listeria_innocua_1642_v1	0.9011
Listeria_monocytogenes_1639_v196	Listeria_innocua_1642_v1	0.8888
Listeria_monocytogenes_1639_v195	Listeria_innocua_1642_v1	0.9212
Listeria_monocytogenes_1639_v194	Listeria_innocua_1642_v1	0.9228
Listeria_monocytogenes_1639_v193	Listeria_innocua_1642_v1	0.9224
Listeria_monocytogenes_1639_v192	Listeria_innocua_1642_v1	0.9007
Listeria_monocytogenes_1639_v191	Listeria_innocua_1642_v1	0.9147
Listeria_monocytogenes_1639_v190	Listeria_innocua_1642_v1	0.9151
Listeria_monocytogenes_1639_v18	Listeria_innocua_1642_v1	0.9003
Listeria_monocytogenes_1639_v189	Listeria_innocua_1642_v1	0.9144
Listeria_monocytogenes_1639_v188	Listeria_innocua_1642_v1	0.9165
Listeria_monocytogenes_1639_v187	Listeria_innocua_1642_v1	0.9148
Listeria_monocytogenes_1639_v186	Listeria_innocua_1642_v1	0.9155
Listeria_monocytogenes_1639_v185	Listeria_innocua_1642_v1	0.9151
Listeria_monocytogenes_1639_v184	Listeria_innocua_1642_v1	0.9143
Listeria_monocytogenes_1639_v183	Listeria_innocua_1642_v1	0.9094
Listeria_monocytogenes_1639_v182	Listeria_innocua_1642_v1	0.9111
Listeria_monocytogenes_1639_v181	Listeria_innocua_1642_v1	0.9138
Listeria_monocytogenes_1639_v180	Listeria_innocua_1642_v1	0.9138
Listeria_monocytogenes_1639_v17	Listeria_innocua_1642_v1	0.9003
Listeria_monocytogenes_1639_v179	Listeria_innocua_1642_v1	0.9103
Listeria_monocytogenes_1639_v178	Listeria_innocua_1642_v1	0.9094
Listeria_monocytogenes_1639_v177	Listeria_innocua_1642_v1	0.9128
Listeria_monocytogenes_1639_v176	Listeria_innocua_1642_v1	0.9152
Listeria_monocytogenes_1639_v175	Listeria_innocua_1642_v1	0.9072
Listeria_monocytogenes_1639_v174	Listeria_innocua_1642_v1	0.9145
Listeria_monocytogenes_1639_v173	Listeria_innocua_1642_v1	0.916
Listeria_monocytogenes_1639_v172	Listeria_innocua_1642_v1	0.9165
Listeria_monocytogenes_1639_v171	Listeria_innocua_1642_v1	0.9187
Listeria_monocytogenes_1639_v170	Listeria_innocua_1642_v1	0.9097
Listeria_monocytogenes_1639_v16	Listeria_innocua_1642_v1	0.8927

Listeria_monocytogenes_1639_v169	Listeria_innocua_1642_v1	0.9079
Listeria_monocytogenes_1639_v168	Listeria_innocua_1642_v1	0.9081
Listeria_monocytogenes_1639_v167	Listeria_innocua_1642_v1	0.9115
Listeria_monocytogenes_1639_v166	Listeria_innocua_1642_v1	0.9085
Listeria_monocytogenes_1639_v165	Listeria_innocua_1642_v1	0.9075
Listeria_monocytogenes_1639_v164	Listeria_innocua_1642_v1	0.914
Listeria_monocytogenes_1639_v163	Listeria_innocua_1642_v1	0.9149
Listeria_monocytogenes_1639_v162	Listeria_innocua_1642_v1	0.914
Listeria_monocytogenes_1639_v161	Listeria_innocua_1642_v1	0.9152
Listeria_monocytogenes_1639_v160	Listeria_innocua_1642_v1	0.9152
Listeria_monocytogenes_1639_v15	Listeria_innocua_1642_v1	0.8977
Listeria_monocytogenes_1639_v159	Listeria_innocua_1642_v1	0.9128
Listeria_monocytogenes_1639_v158	Listeria_innocua_1642_v1	0.9149
Listeria_monocytogenes_1639_v157	Listeria_innocua_1642_v1	0.9149
Listeria_monocytogenes_1639_v156	Listeria_innocua_1642_v1	0.9019
Listeria_monocytogenes_1639_v155	Listeria_innocua_1642_v1	0.9172
Listeria_monocytogenes_1639_v154	Listeria_innocua_1642_v1	0.9059
Listeria_monocytogenes_1639_v153	Listeria_innocua_1642_v1	0.9134
Listeria_monocytogenes_1639_v152	Listeria_innocua_1642_v1	0.9131
Listeria_monocytogenes_1639_v151	Listeria_innocua_1642_v1	0.9131
Listeria_monocytogenes_1639_v150	Listeria_innocua_1642_v1	0.9062
Listeria_monocytogenes_1639_v14	Listeria_innocua_1642_v1	0.8925
Listeria_monocytogenes_1639_v149	Listeria_innocua_1642_v1	0.9057
Listeria_monocytogenes_1639_v148	Listeria_innocua_1642_v1	0.9117
Listeria_monocytogenes_1639_v147	Listeria_innocua_1642_v1	0.9022
Listeria_monocytogenes_1639_v146	Listeria_innocua_1642_v1	0.9127
Listeria_monocytogenes_1639_v145	Listeria_innocua_1642_v1	0.9111
Listeria_monocytogenes_1639_v144	Listeria_innocua_1642_v1	0.9175
Listeria_monocytogenes_1639_v143	Listeria_innocua_1642_v1	0.9113
Listeria_monocytogenes_1639_v142	Listeria_innocua_1642_v1	0.9179
Listeria_monocytogenes_1639_v141	Listeria_innocua_1642_v1	0.9132
Listeria_monocytogenes_1639_v140	Listeria_innocua_1642_v1	0.9182
Listeria_monocytogenes_1639_v13	Listeria_innocua_1642_v1	0.8981
Listeria_monocytogenes_1639_v139	Listeria_innocua_1642_v1	0.9142
Listeria_monocytogenes_1639_v138	Listeria_innocua_1642_v1	0.9168
Listeria_monocytogenes_1639_v137	Listeria_innocua_1642_v1	0.9164
Listeria_monocytogenes_1639_v136	Listeria_innocua_1642_v1	0.9029
Listeria_monocytogenes_1639_v135	Listeria_innocua_1642_v1	0.9044
Listeria_monocytogenes_1639_v134	Listeria_innocua_1642_v1	0.9114
Listeria_monocytogenes_1639_v133	Listeria_innocua_1642_v1	0.9146
Listeria_monocytogenes_1639_v132	Listeria_innocua_1642_v1	0.9004
Listeria_monocytogenes_1639_v131	Listeria_innocua_1642_v1	0.908
Listeria_monocytogenes_1639_v130	Listeria_innocua_1642_v1	0.9142
Listeria_monocytogenes_1639_v12	Listeria_innocua_1642_v1	0.8994
Listeria_monocytogenes_1639_v129	Listeria_innocua_1642_v1	0.9133
Listeria_monocytogenes_1639_v128	Listeria_innocua_1642_v1	0.9143
Listeria_monocytogenes_1639_v127	Listeria_innocua_1642_v1	0.9079
Listeria_monocytogenes_1639_v126	Listeria_innocua_1642_v1	0.9128
Listeria_monocytogenes_1639_v125	Listeria_innocua_1642_v1	0.909
Listeria_monocytogenes_1639_v124	Listeria_innocua_1642_v1	0.9132
Listeria_monocytogenes_1639_v123	Listeria_innocua_1642_v1	0.9128
Listeria_monocytogenes_1639_v122	Listeria_innocua_1642_v1	0.9132
Listeria_monocytogenes_1639_v121	Listeria_innocua_1642_v1	0.9132
Listeria_monocytogenes_1639_v120	Listeria_innocua_1642_v1	0.9128
Listeria_monocytogenes_1639_v11	Listeria_innocua_1642_v1	0.8984
Listeria_monocytogenes_1639_v119	Listeria_innocua_1642_v1	0.9132
Listeria_monocytogenes_1639_v118	Listeria_innocua_1642_v1	0.9132
Listeria_monocytogenes_1639_v117	Listeria_innocua_1642_v1	0.9006
Listeria_monocytogenes_1639_v116	Listeria_innocua_1642_v1	0.911
Listeria_monocytogenes_1639_v115	Listeria_innocua_1642_v1	0.9113
Listeria_monocytogenes_1639_v114	Listeria_innocua_1642_v1	0.9097
Listeria_monocytogenes_1639_v113	Listeria_innocua_1642_v1	0.904
Listeria_monocytogenes_1639_v112	Listeria_innocua_1642_v1	0.9181
Listeria_monocytogenes_1639_v111	Listeria_innocua_1642_v1	0.9159
Listeria_monocytogenes_1639_v110	Listeria_innocua_1642_v1	0.9034
Listeria_monocytogenes_1639_v10	Listeria_innocua_1642_v1	0.8843
Listeria_monocytogenes_1639_v109	Listeria_innocua_1642_v1	0.9086
Listeria_monocytogenes_1639_v108	Listeria_innocua_1642_v1	0.9085
Listeria_monocytogenes_1639_v107	Listeria_innocua_1642_v1	0.9112
Listeria_monocytogenes_1639_v106	Listeria_innocua_1642_v1	0.9115
Listeria_monocytogenes_1639_v105	Listeria_innocua_1642_v1	0.9115
Listeria_monocytogenes_1639_v104	Listeria_innocua_1642_v1	0.9119
Listeria_monocytogenes_1639_v103	Listeria_innocua_1642_v1	0.9119
Listeria_monocytogenes_1639_v102	Listeria_innocua_1642_v1	0.9074
Listeria_monocytogenes_1639_v101	Listeria_innocua_1642_v1	0.9045
Listeria_monocytogenes_1639_v100	Listeria_innocua_1642_v1	0.9028
Listeria_monocytogenes_1639	Listeria_innocua_1642_v1	0.9148
Listeria_monocytogenes_10403S_393133	Listeria_innocua_1642_v1	0.9124
Listeria_monocytogenes_08-5923_637381	Listeria_innocua_1642_v1	0.8974
Listeria_monocytogenes_08-5578_653938	Listeria_innocua_1642_v1	0.8924
Listeria_monocytogenes_07PF0776_1126011	Listeria_innocua_1642_v1	0.9123
Listeria_marthii_FSL_S4-120_702457	Listeria_innocua_1642_v1	0.8561
Listeria_marthii_529731_v1	Listeria_innocua_1642_v1	0.8953
Listeria_marthii_529731	Listeria_innocua_1642_v1	0.8927
Listeria_ivanovii_WSLC3009_1457190	Listeria_innocua_1642_v1	0.868
Listeria_ivanovii_subsp_londoniensis_202752_v2	Listeria_innocua_1642_v1	0.8705
Listeria_ivanovii_subsp_londoniensis_202752_v1	Listeria_innocua_1642_v1	0.8612
Listeria_ivanovii_subsp_londoniensis_202752	Listeria_innocua_1642_v1	0.874
Listeria_ivanovii_subsp_ivanovii_PAM_55_881621	Listeria_innocua_1642_v1	0.8675
Listeria_ivanovii_subsp_ivanovii_202751_v2	Listeria_innocua_1642_v1	0.8687
Listeria_ivanovii_subsp_ivanovii_202751_v1	Listeria_innocua_1642_v1	0.8687

Listeria_ivanovii_subsp_ivanovii_202751	Listeria_innocua_1642_v1	0.8687
Listeria_ivanovii_FSL_F6-596_702454	Listeria_innocua_1642_v1	0.8143
Listeria_ivanovii_1638	Listeria_innocua_1642_v1	0.8684
Listeria_innocua_FSL_S4-378_702456	Listeria_innocua_1642_v1	0.8389
Listeria_innocua_FSL_J1-023_702455	Listeria_innocua_1642_v1	0.8699
Listeria_innocua_Clip11262_272626	Listeria_innocua_1642_v1	0.9159
Listeria_innocua_1642_v4	Listeria_innocua_1642_v1	0.9202
Listeria_innocua_1642_v3	Listeria_innocua_1642_v1	0.9313
Listeria_innocua_1642_v2	Listeria_innocua_1642_v1	0.9283
Listeria_innocua_1642_v1	Listeria_innocua_1642_v1	0.0632
Listeria_welshimeri_1643	Listeria_innocua_1642_v2	0.8788
Listeria_welshimeri_serovar_6b_str_SLCC5334_386043	Listeria_innocua_1642_v2	0.8784
Listeria_seeligeri_serovar_1_2b_str_SLCC3954_683837	Listeria_innocua_1642_v2	0.8606
Listeria_seeligeri_FSL_S4-171_702452	Listeria_innocua_1642_v2	0.8076
Listeria_seeligeri_FSL_N1-067_702453	Listeria_innocua_1642_v2	0.8102
Listeria_seeligeri_1640_v1	Listeria_innocua_1642_v2	0.8661
Listeria_seeligeri_1640	Listeria_innocua_1642_v2	0.8567
Listeria_monocytogenes_WSLC1042_1457188	Listeria_innocua_1642_v2	0.9019
Listeria_monocytogenes_WSLC1001_1457187	Listeria_innocua_1642_v2	0.8978
Listeria_monocytogenes_SLCC7179_879090	Listeria_innocua_1642_v2	0.9011
Listeria_monocytogenes_SLCC5850_882096	Listeria_innocua_1642_v2	0.9015
Listeria_monocytogenes_SLCC2755_932919	Listeria_innocua_1642_v2	0.9008
Listeria_monocytogenes_SLCC2540_879089	Listeria_innocua_1642_v2	0.8997
Listeria_monocytogenes_SLCC2479_882020	Listeria_innocua_1642_v2	0.8956
Listeria_monocytogenes_SLCC2378_879088	Listeria_innocua_1642_v2	0.9029
Listeria_monocytogenes_SLCC2376_882097	Listeria_innocua_1642_v2	0.9168
Listeria_monocytogenes_SLCC2372_932920	Listeria_innocua_1642_v2	0.8978
Listeria_monocytogenes_serotype_4b_str_LL195_1230340	Listeria_innocua_1642_v2	0.9068
Listeria_monocytogenes_serotype_4b_str_LIS0087_1134669	Listeria_innocua_1642_v2	0.8768
Listeria_monocytogenes_serotype_4b_str_F2365_265669	Listeria_innocua_1642_v2	0.9068
Listeria_monocytogenes_serotype_4b_str_CLIP_80459_568819	Listeria_innocua_1642_v2	0.9086
Listeria_monocytogenes_serotype_4b_str_81-0861_1196161	Listeria_innocua_1642_v2	0.894
Listeria_monocytogenes_serotype_4b_str_81-0592_1196160	Listeria_innocua_1642_v2	0.895
Listeria_monocytogenes_serotype_4b_str_81-0558_1196159	Listeria_innocua_1642_v2	0.8957
Listeria_monocytogenes_serotype_4b_str_10-0809_1196162	Listeria_innocua_1642_v2	0.8954
Listeria_monocytogenes_serotype_4b_str_02-6680_1196175	Listeria_innocua_1642_v2	0.9095
Listeria_monocytogenes_serotype_4b_str_02-6679_1196174	Listeria_innocua_1642_v2	0.9095
Listeria_monocytogenes_serotype_4b_str_02-1792_1196173	Listeria_innocua_1642_v2	0.8957
Listeria_monocytogenes_serotype_4b_str_02-1289_1196172	Listeria_innocua_1642_v2	0.8957
Listeria_monocytogenes_serotype_4b_str_02-1103_1196171	Listeria_innocua_1642_v2	0.8957
Listeria_monocytogenes_serotype_3c_str_10-5027_1196195	Listeria_innocua_1642_v2	0.8951
Listeria_monocytogenes_serotype_1_2c_str_10-5026_1196194	Listeria_innocua_1642_v2	0.8956
Listeria_monocytogenes_serotype_1_2c_str_10-5025_1196193	Listeria_innocua_1642_v2	0.8956
Listeria_monocytogenes_serotype_1_2b_str_10-0811_1196164	Listeria_innocua_1642_v2	0.8886
Listeria_monocytogenes_serotype_1_2b_str_10-0810_1196163	Listeria_innocua_1642_v2	0.8886
Listeria_monocytogenes_serotype_1_2a_str_99-6370_1196179	Listeria_innocua_1642_v2	0.8793
Listeria_monocytogenes_serotype_1_2a_str_98-2035_1196178	Listeria_innocua_1642_v2	0.8793
Listeria_monocytogenes_serotype_1_2a_str_95-0093_1196177	Listeria_innocua_1642_v2	0.8834
Listeria_monocytogenes_serotype_1_2a_str_88-0478_1196176	Listeria_innocua_1642_v2	0.8819
Listeria_monocytogenes_serotype_1_2a_str_10-5024_1196192	Listeria_innocua_1642_v2	0.883
Listeria_monocytogenes_serotype_1_2a_str_10-4758_1196168	Listeria_innocua_1642_v2	0.8972
Listeria_monocytogenes_serotype_1_2a_str_10-4754_1196167	Listeria_innocua_1642_v2	0.8975
Listeria_monocytogenes_serotype_1_2a_str_10-1321_1196191	Listeria_innocua_1642_v2	0.883
Listeria_monocytogenes_serotype_1_2a_str_10-1047_1196190	Listeria_innocua_1642_v2	0.8844
Listeria_monocytogenes_serotype_1_2a_str_10-1046_1196189	Listeria_innocua_1642_v2	0.8844
Listeria_monocytogenes_serotype_1_2a_str_10-0934_1196170	Listeria_innocua_1642_v2	0.906
Listeria_monocytogenes_serotype_1_2a_str_10-0933_1196169	Listeria_innocua_1642_v2	0.906
Listeria_monocytogenes_serotype_1_2a_str_10-0815_1196188	Listeria_innocua_1642_v2	0.8803
Listeria_monocytogenes_serotype_1_2a_str_10-0814_1196187	Listeria_innocua_1642_v2	0.8834
Listeria_monocytogenes_serotype_1_2a_str_10-0813_1196166	Listeria_innocua_1642_v2	0.8952
Listeria_monocytogenes_serotype_1_2a_str_10-0812_1196165	Listeria_innocua_1642_v2	0.8952
Listeria_monocytogenes_serotype_1_2a_str_08-7669_1196186	Listeria_innocua_1642_v2	0.8881
Listeria_monocytogenes_serotype_1_2a_str_08-7374_1196185	Listeria_innocua_1642_v2	0.883
Listeria_monocytogenes_serotype_1_2a_str_08-6997_1196184	Listeria_innocua_1642_v2	0.8803
Listeria_monocytogenes_serotype_1_2a_str_08-6569_1196183	Listeria_innocua_1642_v2	0.8803
Listeria_monocytogenes_serotype_1_2a_str_08-6056_1196182	Listeria_innocua_1642_v2	0.8803
Listeria_monocytogenes_serotype_1_2a_str_04-5457_1196181	Listeria_innocua_1642_v2	0.883
Listeria_monocytogenes_serotype_1_2a_str_02-5993_1196180	Listeria_innocua_1642_v2	0.883
Listeria_monocytogenes_R479a_1437838	Listeria_innocua_1642_v2	0.8875
Listeria_monocytogenes_N53-1_1234142	Listeria_innocua_1642_v2	0.8587
Listeria_monocytogenes_M7_1030009	Listeria_innocua_1642_v2	0.8894
Listeria_monocytogenes_LO28_393132	Listeria_innocua_1642_v2	0.8957
Listeria_monocytogenes_L99_563174	Listeria_innocua_1642_v2	0.8897
Listeria_monocytogenes_L312_882094	Listeria_innocua_1642_v2	0.9086
Listeria_monocytogenes_J1816_930781	Listeria_innocua_1642_v2	0.8895
Listeria_monocytogenes_J1-220_930782	Listeria_innocua_1642_v2	0.8921
Listeria_monocytogenes_J0161_393130	Listeria_innocua_1642_v2	0.8898
Listeria_monocytogenes_HCC23_552536	Listeria_innocua_1642_v2	0.8894
Listeria_monocytogenes_FSL_R2-561_393126	Listeria_innocua_1642_v2	0.8946
Listeria_monocytogenes_FSL_J1-208_393119	Listeria_innocua_1642_v2	0.8988
Listeria_monocytogenes_FSL_J1-175_393118	Listeria_innocua_1642_v2	0.8993
Listeria_monocytogenes_Finland_1998_393127	Listeria_innocua_1642_v2	0.9037
Listeria_monocytogenes_EGD-e_169963_v1	Listeria_innocua_1642_v2	0.9036
Listeria_monocytogenes_EGD-e_169963	Listeria_innocua_1642_v2	0.904
Listeria_monocytogenes_EGD_1334565	Listeria_innocua_1642_v2	0.9022
Listeria_monocytogenes_CFSAN002349_1299895	Listeria_innocua_1642_v2	0.9036
Listeria_monocytogenes_ATCC_19117_882095_v1	Listeria_innocua_1642_v2	0.9041
Listeria_monocytogenes_ATCC_19117_882095	Listeria_innocua_1642_v2	0.9038
Listeria_monocytogenes_6179_1288295	Listeria_innocua_1642_v2	0.8884
Listeria_monocytogenes_1639_v9	Listeria_innocua_1642_v2	0.8749
Listeria_monocytogenes_1639_v99	Listeria_innocua_1642_v2	0.8995

Listeria_monocytogenes_1639_v98	Listeria_innocua_1642_v2	0.8893
Listeria_monocytogenes_1639_v97	Listeria_innocua_1642_v2	0.8939
Listeria_monocytogenes_1639_v96	Listeria_innocua_1642_v2	0.8989
Listeria_monocytogenes_1639_v95	Listeria_innocua_1642_v2	0.8989
Listeria_monocytogenes_1639_v94	Listeria_innocua_1642_v2	0.8996
Listeria_monocytogenes_1639_v93	Listeria_innocua_1642_v2	0.8929
Listeria_monocytogenes_1639_v92	Listeria_innocua_1642_v2	0.8996
Listeria_monocytogenes_1639_v91	Listeria_innocua_1642_v2	0.8957
Listeria_monocytogenes_1639_v90	Listeria_innocua_1642_v2	0.8893
Listeria_monocytogenes_1639_v8	Listeria_innocua_1642_v2	0.8804
Listeria_monocytogenes_1639_v89	Listeria_innocua_1642_v2	0.9005
Listeria_monocytogenes_1639_v88	Listeria_innocua_1642_v2	0.8931
Listeria_monocytogenes_1639_v87	Listeria_innocua_1642_v2	0.8902
Listeria_monocytogenes_1639_v86	Listeria_innocua_1642_v2	0.8898
Listeria_monocytogenes_1639_v85	Listeria_innocua_1642_v2	0.8937
Listeria_monocytogenes_1639_v84	Listeria_innocua_1642_v2	0.8943
Listeria_monocytogenes_1639_v83	Listeria_innocua_1642_v2	0.8956
Listeria_monocytogenes_1639_v82	Listeria_innocua_1642_v2	0.8949
Listeria_monocytogenes_1639_v81	Listeria_innocua_1642_v2	0.8893
Listeria_monocytogenes_1639_v80	Listeria_innocua_1642_v2	0.8924
Listeria_monocytogenes_1639_v7	Listeria_innocua_1642_v2	0.8804
Listeria_monocytogenes_1639_v79	Listeria_innocua_1642_v2	0.8881
Listeria_monocytogenes_1639_v78	Listeria_innocua_1642_v2	0.8902
Listeria_monocytogenes_1639_v77	Listeria_innocua_1642_v2	0.8931
Listeria_monocytogenes_1639_v76	Listeria_innocua_1642_v2	0.8858
Listeria_monocytogenes_1639_v75	Listeria_innocua_1642_v2	0.8897
Listeria_monocytogenes_1639_v74	Listeria_innocua_1642_v2	0.8909
Listeria_monocytogenes_1639_v73	Listeria_innocua_1642_v2	0.8977
Listeria_monocytogenes_1639_v72	Listeria_innocua_1642_v2	0.894
Listeria_monocytogenes_1639_v71	Listeria_innocua_1642_v2	0.8829
Listeria_monocytogenes_1639_v70	Listeria_innocua_1642_v2	0.8956
Listeria_monocytogenes_1639_v6	Listeria_innocua_1642_v2	0.8801
Listeria_monocytogenes_1639_v69	Listeria_innocua_1642_v2	0.895
Listeria_monocytogenes_1639_v68	Listeria_innocua_1642_v2	0.8941
Listeria_monocytogenes_1639_v67	Listeria_innocua_1642_v2	0.8923
Listeria_monocytogenes_1639_v66	Listeria_innocua_1642_v2	0.8969
Listeria_monocytogenes_1639_v65	Listeria_innocua_1642_v2	0.8863
Listeria_monocytogenes_1639_v64	Listeria_innocua_1642_v2	0.8954
Listeria_monocytogenes_1639_v63	Listeria_innocua_1642_v2	0.8988
Listeria_monocytogenes_1639_v62	Listeria_innocua_1642_v2	0.8865
Listeria_monocytogenes_1639_v61	Listeria_innocua_1642_v2	0.8848
Listeria_monocytogenes_1639_v60	Listeria_innocua_1642_v2	0.8882
Listeria_monocytogenes_1639_v5	Listeria_innocua_1642_v2	0.8675
Listeria_monocytogenes_1639_v59	Listeria_innocua_1642_v2	0.8989
Listeria_monocytogenes_1639_v58	Listeria_innocua_1642_v2	0.8967
Listeria_monocytogenes_1639_v57	Listeria_innocua_1642_v2	0.8875
Listeria_monocytogenes_1639_v56	Listeria_innocua_1642_v2	0.8955
Listeria_monocytogenes_1639_v55	Listeria_innocua_1642_v2	0.8934
Listeria_monocytogenes_1639_v54	Listeria_innocua_1642_v2	0.8937
Listeria_monocytogenes_1639_v53	Listeria_innocua_1642_v2	0.8941
Listeria_monocytogenes_1639_v52	Listeria_innocua_1642_v2	0.8951
Listeria_monocytogenes_1639_v51	Listeria_innocua_1642_v2	0.8962
Listeria_monocytogenes_1639_v50	Listeria_innocua_1642_v2	0.8823
Listeria_monocytogenes_1639_v4	Listeria_innocua_1642_v2	0.8767
Listeria_monocytogenes_1639_v49	Listeria_innocua_1642_v2	0.9006
Listeria_monocytogenes_1639_v48	Listeria_innocua_1642_v2	0.8925
Listeria_monocytogenes_1639_v47	Listeria_innocua_1642_v2	0.8823
Listeria_monocytogenes_1639_v46	Listeria_innocua_1642_v2	0.8938
Listeria_monocytogenes_1639_v45	Listeria_innocua_1642_v2	0.8798
Listeria_monocytogenes_1639_v44	Listeria_innocua_1642_v2	0.883
Listeria_monocytogenes_1639_v43	Listeria_innocua_1642_v2	0.883
Listeria_monocytogenes_1639_v42	Listeria_innocua_1642_v2	0.8829
Listeria_monocytogenes_1639_v41	Listeria_innocua_1642_v2	0.8828
Listeria_monocytogenes_1639_v40	Listeria_innocua_1642_v2	0.8994
Listeria_monocytogenes_1639_v3	Listeria_innocua_1642_v2	0.8774
Listeria_monocytogenes_1639_v39	Listeria_innocua_1642_v2	0.8884
Listeria_monocytogenes_1639_v38	Listeria_innocua_1642_v2	0.8923
Listeria_monocytogenes_1639_v37	Listeria_innocua_1642_v2	0.8892
Listeria_monocytogenes_1639_v36	Listeria_innocua_1642_v2	0.8919
Listeria_monocytogenes_1639_v35	Listeria_innocua_1642_v2	0.8963
Listeria_monocytogenes_1639_v34	Listeria_innocua_1642_v2	0.8888
Listeria_monocytogenes_1639_v33	Listeria_innocua_1642_v2	0.8886
Listeria_monocytogenes_1639_v32	Listeria_innocua_1642_v2	0.8882
Listeria_monocytogenes_1639_v31	Listeria_innocua_1642_v2	0.8891
Listeria_monocytogenes_1639_v30	Listeria_innocua_1642_v2	0.8877
Listeria_monocytogenes_1639_v2	Listeria_innocua_1642_v2	0.87
Listeria_monocytogenes_1639_v29	Listeria_innocua_1642_v2	0.8913
Listeria_monocytogenes_1639_v28	Listeria_innocua_1642_v2	0.8875
Listeria_monocytogenes_1639_v27	Listeria_innocua_1642_v2	0.889
Listeria_monocytogenes_1639_v26	Listeria_innocua_1642_v2	0.8887
Listeria_monocytogenes_1639_v25	Listeria_innocua_1642_v2	0.8887
Listeria_monocytogenes_1639_v24	Listeria_innocua_1642_v2	0.8914
Listeria_monocytogenes_1639_v23	Listeria_innocua_1642_v2	0.8911
Listeria_monocytogenes_1639_v22	Listeria_innocua_1642_v2	0.8881
Listeria_monocytogenes_1639_v21	Listeria_innocua_1642_v2	0.8925
Listeria_monocytogenes_1639_v20	Listeria_innocua_1642_v2	0.8937
Listeria_monocytogenes_1639_v1	Listeria_innocua_1642_v2	0.8835
Listeria_monocytogenes_1639_v19	Listeria_innocua_1642_v2	0.893
Listeria_monocytogenes_1639_v198	Listeria_innocua_1642_v2	0.8605
Listeria_monocytogenes_1639_v197	Listeria_innocua_1642_v2	0.8911
Listeria_monocytogenes_1639_v196	Listeria_innocua_1642_v2	0.8854

Listeria_monocytogenes_1639_v195	Listeria_innocua_1642_v2	0.9171
Listeria_monocytogenes_1639_v194	Listeria_innocua_1642_v2	0.9176
Listeria_monocytogenes_1639_v193	Listeria_innocua_1642_v2	0.9169
Listeria_monocytogenes_1639_v192	Listeria_innocua_1642_v2	0.8913
Listeria_monocytogenes_1639_v191	Listeria_innocua_1642_v2	0.9077
Listeria_monocytogenes_1639_v190	Listeria_innocua_1642_v2	0.9082
Listeria_monocytogenes_1639_v18	Listeria_innocua_1642_v2	0.882
Listeria_monocytogenes_1639_v189	Listeria_innocua_1642_v2	0.9074
Listeria_monocytogenes_1639_v188	Listeria_innocua_1642_v2	0.908
Listeria_monocytogenes_1639_v187	Listeria_innocua_1642_v2	0.9081
Listeria_monocytogenes_1639_v186	Listeria_innocua_1642_v2	0.9085
Listeria_monocytogenes_1639_v185	Listeria_innocua_1642_v2	0.9085
Listeria_monocytogenes_1639_v184	Listeria_innocua_1642_v2	0.9074
Listeria_monocytogenes_1639_v183	Listeria_innocua_1642_v2	0.9058
Listeria_monocytogenes_1639_v182	Listeria_innocua_1642_v2	0.9076
Listeria_monocytogenes_1639_v181	Listeria_innocua_1642_v2	0.9151
Listeria_monocytogenes_1639_v180	Listeria_innocua_1642_v2	0.9151
Listeria_monocytogenes_1639_v17	Listeria_innocua_1642_v2	0.8823
Listeria_monocytogenes_1639_v179	Listeria_innocua_1642_v2	0.8967
Listeria_monocytogenes_1639_v178	Listeria_innocua_1642_v2	0.9101
Listeria_monocytogenes_1639_v177	Listeria_innocua_1642_v2	0.9058
Listeria_monocytogenes_1639_v176	Listeria_innocua_1642_v2	0.9117
Listeria_monocytogenes_1639_v175	Listeria_innocua_1642_v2	0.9011
Listeria_monocytogenes_1639_v174	Listeria_innocua_1642_v2	0.9056
Listeria_monocytogenes_1639_v173	Listeria_innocua_1642_v2	0.9068
Listeria_monocytogenes_1639_v172	Listeria_innocua_1642_v2	0.9133
Listeria_monocytogenes_1639_v171	Listeria_innocua_1642_v2	0.9058
Listeria_monocytogenes_1639_v170	Listeria_innocua_1642_v2	0.9035
Listeria_monocytogenes_1639_v16	Listeria_innocua_1642_v2	0.8768
Listeria_monocytogenes_1639_v169	Listeria_innocua_1642_v2	0.904
Listeria_monocytogenes_1639_v168	Listeria_innocua_1642_v2	0.9042
Listeria_monocytogenes_1639_v167	Listeria_innocua_1642_v2	0.902
Listeria_monocytogenes_1639_v166	Listeria_innocua_1642_v2	0.9046
Listeria_monocytogenes_1639_v165	Listeria_innocua_1642_v2	0.9037
Listeria_monocytogenes_1639_v164	Listeria_innocua_1642_v2	0.9082
Listeria_monocytogenes_1639_v163	Listeria_innocua_1642_v2	0.9057
Listeria_monocytogenes_1639_v162	Listeria_innocua_1642_v2	0.9082
Listeria_monocytogenes_1639_v161	Listeria_innocua_1642_v2	0.9075
Listeria_monocytogenes_1639_v160	Listeria_innocua_1642_v2	0.9072
Listeria_monocytogenes_1639_v15	Listeria_innocua_1642_v2	0.885
Listeria_monocytogenes_1639_v159	Listeria_innocua_1642_v2	0.9058
Listeria_monocytogenes_1639_v158	Listeria_innocua_1642_v2	0.9068
Listeria_monocytogenes_1639_v157	Listeria_innocua_1642_v2	0.9068
Listeria_monocytogenes_1639_v156	Listeria_innocua_1642_v2	0.8929
Listeria_monocytogenes_1639_v155	Listeria_innocua_1642_v2	0.8986
Listeria_monocytogenes_1639_v154	Listeria_innocua_1642_v2	0.8991
Listeria_monocytogenes_1639_v153	Listeria_innocua_1642_v2	0.8982
Listeria_monocytogenes_1639_v152	Listeria_innocua_1642_v2	0.9058
Listeria_monocytogenes_1639_v151	Listeria_innocua_1642_v2	0.9058
Listeria_monocytogenes_1639_v150	Listeria_innocua_1642_v2	0.9012
Listeria_monocytogenes_1639_v14	Listeria_innocua_1642_v2	0.8827
Listeria_monocytogenes_1639_v149	Listeria_innocua_1642_v2	0.9025
Listeria_monocytogenes_1639_v148	Listeria_innocua_1642_v2	0.9048
Listeria_monocytogenes_1639_v147	Listeria_innocua_1642_v2	0.8969
Listeria_monocytogenes_1639_v146	Listeria_innocua_1642_v2	0.9062
Listeria_monocytogenes_1639_v145	Listeria_innocua_1642_v2	0.9052
Listeria_monocytogenes_1639_v144	Listeria_innocua_1642_v2	0.9079
Listeria_monocytogenes_1639_v143	Listeria_innocua_1642_v2	0.9044
Listeria_monocytogenes_1639_v142	Listeria_innocua_1642_v2	0.9083
Listeria_monocytogenes_1639_v141	Listeria_innocua_1642_v2	0.9021
Listeria_monocytogenes_1639_v140	Listeria_innocua_1642_v2	0.909
Listeria_monocytogenes_1639_v13	Listeria_innocua_1642_v2	0.8853
Listeria_monocytogenes_1639_v139	Listeria_innocua_1642_v2	0.9065
Listeria_monocytogenes_1639_v138	Listeria_innocua_1642_v2	0.9105
Listeria_monocytogenes_1639_v137	Listeria_innocua_1642_v2	0.9102
Listeria_monocytogenes_1639_v136	Listeria_innocua_1642_v2	0.8968
Listeria_monocytogenes_1639_v135	Listeria_innocua_1642_v2	0.8983
Listeria_monocytogenes_1639_v134	Listeria_innocua_1642_v2	0.9027
Listeria_monocytogenes_1639_v133	Listeria_innocua_1642_v2	0.8983
Listeria_monocytogenes_1639_v132	Listeria_innocua_1642_v2	0.8941
Listeria_monocytogenes_1639_v131	Listeria_innocua_1642_v2	0.8971
Listeria_monocytogenes_1639_v130	Listeria_innocua_1642_v2	0.9049
Listeria_monocytogenes_1639_v12	Listeria_innocua_1642_v2	0.8863
Listeria_monocytogenes_1639_v129	Listeria_innocua_1642_v2	0.9075
Listeria_monocytogenes_1639_v128	Listeria_innocua_1642_v2	0.9036
Listeria_monocytogenes_1639_v127	Listeria_innocua_1642_v2	0.8969
Listeria_monocytogenes_1639_v126	Listeria_innocua_1642_v2	0.9018
Listeria_monocytogenes_1639_v125	Listeria_innocua_1642_v2	0.8968
Listeria_monocytogenes_1639_v124	Listeria_innocua_1642_v2	0.9104
Listeria_monocytogenes_1639_v123	Listeria_innocua_1642_v2	0.9036
Listeria_monocytogenes_1639_v122	Listeria_innocua_1642_v2	0.9036
Listeria_monocytogenes_1639_v121	Listeria_innocua_1642_v2	0.9036
Listeria_monocytogenes_1639_v120	Listeria_innocua_1642_v2	0.9036
Listeria_monocytogenes_1639_v11	Listeria_innocua_1642_v2	0.8853
Listeria_monocytogenes_1639_v119	Listeria_innocua_1642_v2	0.9036
Listeria_monocytogenes_1639_v118	Listeria_innocua_1642_v2	0.9036
Listeria_monocytogenes_1639_v117	Listeria_innocua_1642_v2	0.8979
Listeria_monocytogenes_1639_v116	Listeria_innocua_1642_v2	0.9026
Listeria_monocytogenes_1639_v115	Listeria_innocua_1642_v2	0.9029
Listeria_monocytogenes_1639_v114	Listeria_innocua_1642_v2	0.8951
Listeria_monocytogenes_1639_v113	Listeria_innocua_1642_v2	0.9002

Listeria_monocytogenes_1639_v112	Listeria_innocua_1642_v2	0.8981
Listeria_monocytogenes_1639_v111	Listeria_innocua_1642_v2	0.9049
Listeria_monocytogenes_1639_v110	Listeria_innocua_1642_v2	0.894
Listeria_monocytogenes_1639_v10	Listeria_innocua_1642_v2	0.8749
Listeria_monocytogenes_1639_v109	Listeria_innocua_1642_v2	0.8955
Listeria_monocytogenes_1639_v108	Listeria_innocua_1642_v2	0.898
Listeria_monocytogenes_1639_v107	Listeria_innocua_1642_v2	0.9027
Listeria_monocytogenes_1639_v106	Listeria_innocua_1642_v2	0.9031
Listeria_monocytogenes_1639_v105	Listeria_innocua_1642_v2	0.9031
Listeria_monocytogenes_1639_v104	Listeria_innocua_1642_v2	0.9031
Listeria_monocytogenes_1639_v103	Listeria_innocua_1642_v2	0.9035
Listeria_monocytogenes_1639_v102	Listeria_innocua_1642_v2	0.891
Listeria_monocytogenes_1639_v101	Listeria_innocua_1642_v2	0.8868
Listeria_monocytogenes_1639_v100	Listeria_innocua_1642_v2	0.8869
Listeria_monocytogenes_1639	Listeria_innocua_1642_v2	0.9082
Listeria_monocytogenes_10403S_393133	Listeria_innocua_1642_v2	0.9006
Listeria_monocytogenes_08-5923_637381	Listeria_innocua_1642_v2	0.883
Listeria_monocytogenes_08-5578_653938	Listeria_innocua_1642_v2	0.8768
Listeria_monocytogenes_07PF0776_1126011	Listeria_innocua_1642_v2	0.9034
Listeria_marthii_FSL_S4-120_702457	Listeria_innocua_1642_v2	0.8494
Listeria_marthii_529731_v1	Listeria_innocua_1642_v2	0.8845
Listeria_marthii_529731	Listeria_innocua_1642_v2	0.8908
Listeria_ivanovii_WSLC3009_1457190	Listeria_innocua_1642_v2	0.8659
Listeria_ivanovii_subsp_londoniensis_202752_v2	Listeria_innocua_1642_v2	0.8639
Listeria_ivanovii_subsp_londoniensis_202752_v1	Listeria_innocua_1642_v2	0.8611
Listeria_ivanovii_subsp_londoniensis_202752	Listeria_innocua_1642_v2	0.8595
Listeria_ivanovii_subsp_ivanovii_PAM_55_881621	Listeria_innocua_1642_v2	0.8647
Listeria_ivanovii_subsp_ivanovii_202751_v2	Listeria_innocua_1642_v2	0.8662
Listeria_ivanovii_subsp_ivanovii_202751_v1	Listeria_innocua_1642_v2	0.8662
Listeria_ivanovii_subsp_ivanovii_202751	Listeria_innocua_1642_v2	0.8662
Listeria_ivanovii_FSL_F6-596_702454	Listeria_innocua_1642_v2	0.7988
Listeria_ivanovii_1638	Listeria_innocua_1642_v2	0.8662
Listeria_innocua_FSL_S4-378_702456	Listeria_innocua_1642_v2	0.8344
Listeria_innocua_FSL_J1-023_702455	Listeria_innocua_1642_v2	0.8723
Listeria_innocua_Clip11262_272626	Listeria_innocua_1642_v2	0.903
Listeria_innocua_1642_v4	Listeria_innocua_1642_v2	0.9218
Listeria_innocua_1642_v3	Listeria_innocua_1642_v2	0.9786
Listeria_innocua_1642_v2	Listeria_innocua_1642_v2	0.0823
Listeria_welshimeri_1643	Listeria_innocua_1642_v3	0.8702
Listeria_welshimeri_serovar_6b_str_SLCC5334_386043	Listeria_innocua_1642_v3	0.8699
Listeria_seeligeri_serovar_1_2b_str_SLCC3954_683837	Listeria_innocua_1642_v3	0.86
Listeria_seeligeri_FSL_S4-171_702452	Listeria_innocua_1642_v3	0.8101
Listeria_seeligeri_FSL_N1-067_702453	Listeria_innocua_1642_v3	0.8122
Listeria_seeligeri_1640_v1	Listeria_innocua_1642_v3	0.8658
Listeria_seeligeri_1640	Listeria_innocua_1642_v3	0.8658
Listeria_monocytogenes_WSLC1042_1457188	Listeria_innocua_1642_v3	0.9024
Listeria_monocytogenes_WSLC1001_1457187	Listeria_innocua_1642_v3	0.8946
Listeria_monocytogenes_SLCC7179_879090	Listeria_innocua_1642_v3	0.9086
Listeria_monocytogenes_SLCC5850_882096	Listeria_innocua_1642_v3	0.8978
Listeria_monocytogenes_SLCC2755_932919	Listeria_innocua_1642_v3	0.9001
Listeria_monocytogenes_SLCC2540_879089	Listeria_innocua_1642_v3	0.8975
Listeria_monocytogenes_SLCC2479_882020	Listeria_innocua_1642_v3	0.8967
Listeria_monocytogenes_SLCC2378_879088	Listeria_innocua_1642_v3	0.9034
Listeria_monocytogenes_SLCC2376_882097	Listeria_innocua_1642_v3	0.9161
Listeria_monocytogenes_SLCC2372_932920	Listeria_innocua_1642_v3	0.899
Listeria_monocytogenes_serotype_4b_str_LL195_1230340	Listeria_innocua_1642_v3	0.9069
Listeria_monocytogenes_serotype_4b_str_LIS0087_1134669	Listeria_innocua_1642_v3	0.8833
Listeria_monocytogenes_serotype_4b_str_F2365_265669	Listeria_innocua_1642_v3	0.9069
Listeria_monocytogenes_serotype_4b_str_CLIP_80459_568819	Listeria_innocua_1642_v3	0.9083
Listeria_monocytogenes_serotype_4b_str_81-0861_1196161	Listeria_innocua_1642_v3	0.9063
Listeria_monocytogenes_serotype_4b_str_81-0592_1196160	Listeria_innocua_1642_v3	0.9073
Listeria_monocytogenes_serotype_4b_str_81-0558_1196159	Listeria_innocua_1642_v3	0.908
Listeria_monocytogenes_serotype_4b_str_10-0809_1196162	Listeria_innocua_1642_v3	0.9077
Listeria_monocytogenes_serotype_4b_str_02-6680_1196175	Listeria_innocua_1642_v3	0.9096
Listeria_monocytogenes_serotype_4b_str_02-6679_1196174	Listeria_innocua_1642_v3	0.9096
Listeria_monocytogenes_serotype_4b_str_02-1792_1196173	Listeria_innocua_1642_v3	0.908
Listeria_monocytogenes_serotype_4b_str_02-1289_1196172	Listeria_innocua_1642_v3	0.908
Listeria_monocytogenes_serotype_4b_str_02-1103_1196171	Listeria_innocua_1642_v3	0.908
Listeria_monocytogenes_serotype_3c_str_10-5027_1196195	Listeria_innocua_1642_v3	0.8956
Listeria_monocytogenes_serotype_1_2c_str_10-5026_1196194	Listeria_innocua_1642_v3	0.8964
Listeria_monocytogenes_serotype_1_2c_str_10-5025_1196193	Listeria_innocua_1642_v3	0.8964
Listeria_monocytogenes_serotype_1_2b_str_10-0811_1196164	Listeria_innocua_1642_v3	0.8953
Listeria_monocytogenes_serotype_1_2b_str_10-0810_1196163	Listeria_innocua_1642_v3	0.8953
Listeria_monocytogenes_serotype_1_2a_str_99-6370_1196179	Listeria_innocua_1642_v3	0.8798
Listeria_monocytogenes_serotype_1_2a_str_98-2035_1196178	Listeria_innocua_1642_v3	0.8798
Listeria_monocytogenes_serotype_1_2a_str_95-0093_1196177	Listeria_innocua_1642_v3	0.8846
Listeria_monocytogenes_serotype_1_2a_str_88-0478_1196176	Listeria_innocua_1642_v3	0.8838
Listeria_monocytogenes_serotype_1_2a_str_10-5024_1196192	Listeria_innocua_1642_v3	0.8846
Listeria_monocytogenes_serotype_1_2a_str_10-4758_1196168	Listeria_innocua_1642_v3	0.8954
Listeria_monocytogenes_serotype_1_2a_str_10-4754_1196167	Listeria_innocua_1642_v3	0.8957
Listeria_monocytogenes_serotype_1_2a_str_10-1321_1196191	Listeria_innocua_1642_v3	0.8846
Listeria_monocytogenes_serotype_1_2a_str_10-1047_1196190	Listeria_innocua_1642_v3	0.8852
Listeria_monocytogenes_serotype_1_2a_str_10-1046_1196189	Listeria_innocua_1642_v3	0.8852
Listeria_monocytogenes_serotype_1_2a_str_10-0934_1196170	Listeria_innocua_1642_v3	0.9065
Listeria_monocytogenes_serotype_1_2a_str_10-0933_1196169	Listeria_innocua_1642_v3	0.9065
Listeria_monocytogenes_serotype_1_2a_str_10-0815_1196188	Listeria_innocua_1642_v3	0.8807
Listeria_monocytogenes_serotype_1_2a_str_10-0814_1196187	Listeria_innocua_1642_v3	0.8849
Listeria_monocytogenes_serotype_1_2a_str_10-0813_1196166	Listeria_innocua_1642_v3	0.9019
Listeria_monocytogenes_serotype_1_2a_str_10-0812_1196165	Listeria_innocua_1642_v3	0.9019
Listeria_monocytogenes_serotype_1_2a_str_08-7669_1196186	Listeria_innocua_1642_v3	0.8934
Listeria_monocytogenes_serotype_1_2a_str_08-7374_1196185	Listeria_innocua_1642_v3	0.8846

Listeria_monocytogenes_serotype_1_2a_str_08-6997_1196184	Listeria_innocua_1642_v3	0.8807
Listeria_monocytogenes_serotype_1_2a_str_08-6569_1196183	Listeria_innocua_1642_v3	0.8807
Listeria_monocytogenes_serotype_1_2a_str_08-6056_1196182	Listeria_innocua_1642_v3	0.8807
Listeria_monocytogenes_serotype_1_2a_str_04-5457_1196181	Listeria_innocua_1642_v3	0.8846
Listeria_monocytogenes_serotype_1_2a_str_02-5993_1196180	Listeria_innocua_1642_v3	0.8846
Listeria_monocytogenes_R479a_1437838	Listeria_innocua_1642_v3	0.8872
Listeria_monocytogenes_N53-1_1234142	Listeria_innocua_1642_v3	0.865
Listeria_monocytogenes_M7_1030009	Listeria_innocua_1642_v3	0.8957
Listeria_monocytogenes_LO28_393132	Listeria_innocua_1642_v3	0.9028
Listeria_monocytogenes_L99_563174	Listeria_innocua_1642_v3	0.8961
Listeria_monocytogenes_L312_882094	Listeria_innocua_1642_v3	0.9083
Listeria_monocytogenes_J1816_930781	Listeria_innocua_1642_v3	0.8951
Listeria_monocytogenes_J1-220_930782	Listeria_innocua_1642_v3	0.8999
Listeria_monocytogenes_J0161_393130	Listeria_innocua_1642_v3	0.9013
Listeria_monocytogenes_HCC23_552536	Listeria_innocua_1642_v3	0.8957
Listeria_monocytogenes_FSL_R2-561_393126	Listeria_innocua_1642_v3	0.8943
Listeria_monocytogenes_FSL_J1-208_393119	Listeria_innocua_1642_v3	0.8989
Listeria_monocytogenes_FSL_J1-175_393118	Listeria_innocua_1642_v3	0.8971
Listeria_monocytogenes_Finland_1998_393127	Listeria_innocua_1642_v3	0.9042
Listeria_monocytogenes_EGD-e_169963_v1	Listeria_innocua_1642_v3	0.9138
Listeria_monocytogenes_EGD-e_169963	Listeria_innocua_1642_v3	0.9141
Listeria_monocytogenes_EGD_1334565	Listeria_innocua_1642_v3	0.8985
Listeria_monocytogenes_CFSAN002349_1299895	Listeria_innocua_1642_v3	0.9037
Listeria_monocytogenes_ATCC_19117_882095_v1	Listeria_innocua_1642_v3	0.9046
Listeria_monocytogenes_ATCC_19117_882095	Listeria_innocua_1642_v3	0.9042
Listeria_monocytogenes_6179_1288295	Listeria_innocua_1642_v3	0.8969
Listeria_monocytogenes_1639_v9	Listeria_innocua_1642_v3	0.8804
Listeria_monocytogenes_1639_v99	Listeria_innocua_1642_v3	0.9048
Listeria_monocytogenes_1639_v98	Listeria_innocua_1642_v3	0.8975
Listeria_monocytogenes_1639_v97	Listeria_innocua_1642_v3	0.8988
Listeria_monocytogenes_1639_v96	Listeria_innocua_1642_v3	0.9045
Listeria_monocytogenes_1639_v95	Listeria_innocua_1642_v3	0.9045
Listeria_monocytogenes_1639_v94	Listeria_innocua_1642_v3	0.9052
Listeria_monocytogenes_1639_v93	Listeria_innocua_1642_v3	0.8904
Listeria_monocytogenes_1639_v92	Listeria_innocua_1642_v3	0.9048
Listeria_monocytogenes_1639_v91	Listeria_innocua_1642_v3	0.9031
Listeria_monocytogenes_1639_v90	Listeria_innocua_1642_v3	0.8975
Listeria_monocytogenes_1639_v8	Listeria_innocua_1642_v3	0.8812
Listeria_monocytogenes_1639_v89	Listeria_innocua_1642_v3	0.9088
Listeria_monocytogenes_1639_v88	Listeria_innocua_1642_v3	0.8935
Listeria_monocytogenes_1639_v87	Listeria_innocua_1642_v3	0.8973
Listeria_monocytogenes_1639_v86	Listeria_innocua_1642_v3	0.8969
Listeria_monocytogenes_1639_v85	Listeria_innocua_1642_v3	0.9064
Listeria_monocytogenes_1639_v84	Listeria_innocua_1642_v3	0.8948
Listeria_monocytogenes_1639_v83	Listeria_innocua_1642_v3	0.9061
Listeria_monocytogenes_1639_v82	Listeria_innocua_1642_v3	0.9054
Listeria_monocytogenes_1639_v81	Listeria_innocua_1642_v3	0.892
Listeria_monocytogenes_1639_v80	Listeria_innocua_1642_v3	0.8977
Listeria_monocytogenes_1639_v7	Listeria_innocua_1642_v3	0.8812
Listeria_monocytogenes_1639_v79	Listeria_innocua_1642_v3	0.89
Listeria_monocytogenes_1639_v78	Listeria_innocua_1642_v3	0.8917
Listeria_monocytogenes_1639_v77	Listeria_innocua_1642_v3	0.898
Listeria_monocytogenes_1639_v76	Listeria_innocua_1642_v3	0.8844
Listeria_monocytogenes_1639_v75	Listeria_innocua_1642_v3	0.8961
Listeria_monocytogenes_1639_v74	Listeria_innocua_1642_v3	0.8999
Listeria_monocytogenes_1639_v73	Listeria_innocua_1642_v3	0.9044
Listeria_monocytogenes_1639_v72	Listeria_innocua_1642_v3	0.9045
Listeria_monocytogenes_1639_v71	Listeria_innocua_1642_v3	0.8852
Listeria_monocytogenes_1639_v70	Listeria_innocua_1642_v3	0.8975
Listeria_monocytogenes_1639_v6	Listeria_innocua_1642_v3	0.8809
Listeria_monocytogenes_1639_v69	Listeria_innocua_1642_v3	0.9073
Listeria_monocytogenes_1639_v68	Listeria_innocua_1642_v3	0.9035
Listeria_monocytogenes_1639_v67	Listeria_innocua_1642_v3	0.8931
Listeria_monocytogenes_1639_v66	Listeria_innocua_1642_v3	0.8951
Listeria_monocytogenes_1639_v65	Listeria_innocua_1642_v3	0.8923
Listeria_monocytogenes_1639_v64	Listeria_innocua_1642_v3	0.8918
Listeria_monocytogenes_1639_v63	Listeria_innocua_1642_v3	0.8996
Listeria_monocytogenes_1639_v62	Listeria_innocua_1642_v3	0.8884
Listeria_monocytogenes_1639_v61	Listeria_innocua_1642_v3	0.8868
Listeria_monocytogenes_1639_v60	Listeria_innocua_1642_v3	0.8961
Listeria_monocytogenes_1639_v5	Listeria_innocua_1642_v3	0.8747
Listeria_monocytogenes_1639_v59	Listeria_innocua_1642_v3	0.8975
Listeria_monocytogenes_1639_v58	Listeria_innocua_1642_v3	0.9086
Listeria_monocytogenes_1639_v57	Listeria_innocua_1642_v3	0.892
Listeria_monocytogenes_1639_v56	Listeria_innocua_1642_v3	0.9027
Listeria_monocytogenes_1639_v55	Listeria_innocua_1642_v3	0.9041
Listeria_monocytogenes_1639_v54	Listeria_innocua_1642_v3	0.8998
Listeria_monocytogenes_1639_v53	Listeria_innocua_1642_v3	0.8938
Listeria_monocytogenes_1639_v52	Listeria_innocua_1642_v3	0.9026
Listeria_monocytogenes_1639_v51	Listeria_innocua_1642_v3	0.9081
Listeria_monocytogenes_1639_v50	Listeria_innocua_1642_v3	0.8838
Listeria_monocytogenes_1639_v4	Listeria_innocua_1642_v3	0.8836
Listeria_monocytogenes_1639_v49	Listeria_innocua_1642_v3	0.9007
Listeria_monocytogenes_1639_v48	Listeria_innocua_1642_v3	0.8922
Listeria_monocytogenes_1639_v47	Listeria_innocua_1642_v3	0.8846
Listeria_monocytogenes_1639_v46	Listeria_innocua_1642_v3	0.8969
Listeria_monocytogenes_1639_v45	Listeria_innocua_1642_v3	0.8814
Listeria_monocytogenes_1639_v44	Listeria_innocua_1642_v3	0.8846
Listeria_monocytogenes_1639_v43	Listeria_innocua_1642_v3	0.8846
Listeria_monocytogenes_1639_v42	Listeria_innocua_1642_v3	0.8856
Listeria_monocytogenes_1639_v41	Listeria_innocua_1642_v3	0.8854

Listeria_monocytogenes_1639_v40	Listeria_innocua_1642_v3	0.8976
Listeria_monocytogenes_1639_v3	Listeria_innocua_1642_v3	0.8844
Listeria_monocytogenes_1639_v39	Listeria_innocua_1642_v3	0.8903
Listeria_monocytogenes_1639_v38	Listeria_innocua_1642_v3	0.8905
Listeria_monocytogenes_1639_v37	Listeria_innocua_1642_v3	0.8864
Listeria_monocytogenes_1639_v36	Listeria_innocua_1642_v3	0.8898
Listeria_monocytogenes_1639_v35	Listeria_innocua_1642_v3	0.8949
Listeria_monocytogenes_1639_v34	Listeria_innocua_1642_v3	0.8903
Listeria_monocytogenes_1639_v33	Listeria_innocua_1642_v3	0.8953
Listeria_monocytogenes_1639_v32	Listeria_innocua_1642_v3	0.8949
Listeria_monocytogenes_1639_v31	Listeria_innocua_1642_v3	0.8907
Listeria_monocytogenes_1639_v30	Listeria_innocua_1642_v3	0.8867
Listeria_monocytogenes_1639_v2	Listeria_innocua_1642_v3	0.88
Listeria_monocytogenes_1639_v29	Listeria_innocua_1642_v3	0.8918
Listeria_monocytogenes_1639_v28	Listeria_innocua_1642_v3	0.8897
Listeria_monocytogenes_1639_v27	Listeria_innocua_1642_v3	0.8949
Listeria_monocytogenes_1639_v26	Listeria_innocua_1642_v3	0.8946
Listeria_monocytogenes_1639_v25	Listeria_innocua_1642_v3	0.8949
Listeria_monocytogenes_1639_v24	Listeria_innocua_1642_v3	0.9003
Listeria_monocytogenes_1639_v23	Listeria_innocua_1642_v3	0.9
Listeria_monocytogenes_1639_v22	Listeria_innocua_1642_v3	0.8956
Listeria_monocytogenes_1639_v21	Listeria_innocua_1642_v3	0.8989
Listeria_monocytogenes_1639_v20	Listeria_innocua_1642_v3	0.8948
Listeria_monocytogenes_1639_v1	Listeria_innocua_1642_v3	0.8901
Listeria_monocytogenes_1639_v19	Listeria_innocua_1642_v3	0.8941
Listeria_monocytogenes_1639_v198	Listeria_innocua_1642_v3	0.866
Listeria_monocytogenes_1639_v197	Listeria_innocua_1642_v3	0.9011
Listeria_monocytogenes_1639_v196	Listeria_innocua_1642_v3	0.8817
Listeria_monocytogenes_1639_v195	Listeria_innocua_1642_v3	0.9164
Listeria_monocytogenes_1639_v194	Listeria_innocua_1642_v3	0.9165
Listeria_monocytogenes_1639_v193	Listeria_innocua_1642_v3	0.9162
Listeria_monocytogenes_1639_v192	Listeria_innocua_1642_v3	0.8918
Listeria_monocytogenes_1639_v191	Listeria_innocua_1642_v3	0.9077
Listeria_monocytogenes_1639_v190	Listeria_innocua_1642_v3	0.9082
Listeria_monocytogenes_1639_v18	Listeria_innocua_1642_v3	0.8927
Listeria_monocytogenes_1639_v189	Listeria_innocua_1642_v3	0.9075
Listeria_monocytogenes_1639_v188	Listeria_innocua_1642_v3	0.9081
Listeria_monocytogenes_1639_v187	Listeria_innocua_1642_v3	0.9082
Listeria_monocytogenes_1639_v186	Listeria_innocua_1642_v3	0.9086
Listeria_monocytogenes_1639_v185	Listeria_innocua_1642_v3	0.9086
Listeria_monocytogenes_1639_v184	Listeria_innocua_1642_v3	0.9074
Listeria_monocytogenes_1639_v183	Listeria_innocua_1642_v3	0.9052
Listeria_monocytogenes_1639_v182	Listeria_innocua_1642_v3	0.9069
Listeria_monocytogenes_1639_v181	Listeria_innocua_1642_v3	0.9152
Listeria_monocytogenes_1639_v180	Listeria_innocua_1642_v3	0.9152
Listeria_monocytogenes_1639_v17	Listeria_innocua_1642_v3	0.893
Listeria_monocytogenes_1639_v179	Listeria_innocua_1642_v3	0.8986
Listeria_monocytogenes_1639_v178	Listeria_innocua_1642_v3	0.9094
Listeria_monocytogenes_1639_v177	Listeria_innocua_1642_v3	0.9059
Listeria_monocytogenes_1639_v176	Listeria_innocua_1642_v3	0.911
Listeria_monocytogenes_1639_v175	Listeria_innocua_1642_v3	0.9004
Listeria_monocytogenes_1639_v174	Listeria_innocua_1642_v3	0.9061
Listeria_monocytogenes_1639_v173	Listeria_innocua_1642_v3	0.9065
Listeria_monocytogenes_1639_v172	Listeria_innocua_1642_v3	0.9133
Listeria_monocytogenes_1639_v171	Listeria_innocua_1642_v3	0.9055
Listeria_monocytogenes_1639_v170	Listeria_innocua_1642_v3	0.9032
Listeria_monocytogenes_1639_v16	Listeria_innocua_1642_v3	0.8772
Listeria_monocytogenes_1639_v169	Listeria_innocua_1642_v3	0.9018
Listeria_monocytogenes_1639_v168	Listeria_innocua_1642_v3	0.9036
Listeria_monocytogenes_1639_v167	Listeria_innocua_1642_v3	0.9024
Listeria_monocytogenes_1639_v166	Listeria_innocua_1642_v3	0.9039
Listeria_monocytogenes_1639_v165	Listeria_innocua_1642_v3	0.9015
Listeria_monocytogenes_1639_v164	Listeria_innocua_1642_v3	0.9079
Listeria_monocytogenes_1639_v163	Listeria_innocua_1642_v3	0.9058
Listeria_monocytogenes_1639_v162	Listeria_innocua_1642_v3	0.9079
Listeria_monocytogenes_1639_v161	Listeria_innocua_1642_v3	0.9075
Listeria_monocytogenes_1639_v160	Listeria_innocua_1642_v3	0.9072
Listeria_monocytogenes_1639_v15	Listeria_innocua_1642_v3	0.8909
Listeria_monocytogenes_1639_v159	Listeria_innocua_1642_v3	0.9059
Listeria_monocytogenes_1639_v158	Listeria_innocua_1642_v3	0.9069
Listeria_monocytogenes_1639_v157	Listeria_innocua_1642_v3	0.9069
Listeria_monocytogenes_1639_v156	Listeria_innocua_1642_v3	0.9026
Listeria_monocytogenes_1639_v155	Listeria_innocua_1642_v3	0.9028
Listeria_monocytogenes_1639_v154	Listeria_innocua_1642_v3	0.895
Listeria_monocytogenes_1639_v153	Listeria_innocua_1642_v3	0.9103
Listeria_monocytogenes_1639_v152	Listeria_innocua_1642_v3	0.9059
Listeria_monocytogenes_1639_v151	Listeria_innocua_1642_v3	0.9059
Listeria_monocytogenes_1639_v150	Listeria_innocua_1642_v3	0.8994
Listeria_monocytogenes_1639_v14	Listeria_innocua_1642_v3	0.8919
Listeria_monocytogenes_1639_v149	Listeria_innocua_1642_v3	0.8989
Listeria_monocytogenes_1639_v148	Listeria_innocua_1642_v3	0.9049
Listeria_monocytogenes_1639_v147	Listeria_innocua_1642_v3	0.8973
Listeria_monocytogenes_1639_v146	Listeria_innocua_1642_v3	0.9062
Listeria_monocytogenes_1639_v145	Listeria_innocua_1642_v3	0.9053
Listeria_monocytogenes_1639_v144	Listeria_innocua_1642_v3	0.9076
Listeria_monocytogenes_1639_v143	Listeria_innocua_1642_v3	0.9045
Listeria_monocytogenes_1639_v142	Listeria_innocua_1642_v3	0.908
Listeria_monocytogenes_1639_v141	Listeria_innocua_1642_v3	0.904
Listeria_monocytogenes_1639_v140	Listeria_innocua_1642_v3	0.9086
Listeria_monocytogenes_1639_v13	Listeria_innocua_1642_v3	0.8909
Listeria_monocytogenes_1639_v139	Listeria_innocua_1642_v3	0.9065

Listeria_monocytogenes_1639_v138	Listeria_innocua_1642_v3	0.9102
Listeria_monocytogenes_1639_v137	Listeria_innocua_1642_v3	0.9098
Listeria_monocytogenes_1639_v136	Listeria_innocua_1642_v3	0.895
Listeria_monocytogenes_1639_v135	Listeria_innocua_1642_v3	0.9084
Listeria_monocytogenes_1639_v134	Listeria_innocua_1642_v3	0.9031
Listeria_monocytogenes_1639_v133	Listeria_innocua_1642_v3	0.8988
Listeria_monocytogenes_1639_v132	Listeria_innocua_1642_v3	0.9023
Listeria_monocytogenes_1639_v131	Listeria_innocua_1642_v3	0.9053
Listeria_monocytogenes_1639_v130	Listeria_innocua_1642_v3	0.9031
Listeria_monocytogenes_1639_v12	Listeria_innocua_1642_v3	0.8874
Listeria_monocytogenes_1639_v129	Listeria_innocua_1642_v3	0.9075
Listeria_monocytogenes_1639_v128	Listeria_innocua_1642_v3	0.9052
Listeria_monocytogenes_1639_v127	Listeria_innocua_1642_v3	0.9093
Listeria_monocytogenes_1639_v126	Listeria_innocua_1642_v3	0.9033
Listeria_monocytogenes_1639_v125	Listeria_innocua_1642_v3	0.9014
Listeria_monocytogenes_1639_v124	Listeria_innocua_1642_v3	0.9101
Listeria_monocytogenes_1639_v123	Listeria_innocua_1642_v3	0.9037
Listeria_monocytogenes_1639_v122	Listeria_innocua_1642_v3	0.9037
Listeria_monocytogenes_1639_v121	Listeria_innocua_1642_v3	0.9037
Listeria_monocytogenes_1639_v120	Listeria_innocua_1642_v3	0.9037
Listeria_monocytogenes_1639_v11	Listeria_innocua_1642_v3	0.893
Listeria_monocytogenes_1639_v119	Listeria_innocua_1642_v3	0.9037
Listeria_monocytogenes_1639_v118	Listeria_innocua_1642_v3	0.9037
Listeria_monocytogenes_1639_v117	Listeria_innocua_1642_v3	0.8954
Listeria_monocytogenes_1639_v116	Listeria_innocua_1642_v3	0.903
Listeria_monocytogenes_1639_v115	Listeria_innocua_1642_v3	0.9034
Listeria_monocytogenes_1639_v114	Listeria_innocua_1642_v3	0.8955
Listeria_monocytogenes_1639_v113	Listeria_innocua_1642_v3	0.8977
Listeria_monocytogenes_1639_v112	Listeria_innocua_1642_v3	0.9045
Listeria_monocytogenes_1639_v111	Listeria_innocua_1642_v3	0.9042
Listeria_monocytogenes_1639_v110	Listeria_innocua_1642_v3	0.9008
Listeria_monocytogenes_1639_v10	Listeria_innocua_1642_v3	0.8746
Listeria_monocytogenes_1639_v109	Listeria_innocua_1642_v3	0.8959
Listeria_monocytogenes_1639_v108	Listeria_innocua_1642_v3	0.9062
Listeria_monocytogenes_1639_v107	Listeria_innocua_1642_v3	0.9032
Listeria_monocytogenes_1639_v106	Listeria_innocua_1642_v3	0.9036
Listeria_monocytogenes_1639_v105	Listeria_innocua_1642_v3	0.9036
Listeria_monocytogenes_1639_v104	Listeria_innocua_1642_v3	0.9036
Listeria_monocytogenes_1639_v103	Listeria_innocua_1642_v3	0.9039
Listeria_monocytogenes_1639_v102	Listeria_innocua_1642_v3	0.8914
Listeria_monocytogenes_1639_v101	Listeria_innocua_1642_v3	0.8869
Listeria_monocytogenes_1639_v100	Listeria_innocua_1642_v3	0.8866
Listeria_monocytogenes_1639	Listeria_innocua_1642_v3	0.9082
Listeria_monocytogenes_10403S_393133	Listeria_innocua_1642_v3	0.9108
Listeria_monocytogenes_08-5923_637381	Listeria_innocua_1642_v3	0.8846
Listeria_monocytogenes_08-5578_653938	Listeria_innocua_1642_v3	0.8772
Listeria_monocytogenes_07PF0776_1126011	Listeria_innocua_1642_v3	0.9035
Listeria_marthii_FSL_S4-120_702457	Listeria_innocua_1642_v3	0.8488
Listeria_marthii_529731_v1	Listeria_innocua_1642_v3	0.8951
Listeria_marthii_529731	Listeria_innocua_1642_v3	0.8901
Listeria_ivanovii_WSLC3009_1457190	Listeria_innocua_1642_v3	0.8649
Listeria_ivanovii_subsp_londoniensis_202752_v2	Listeria_innocua_1642_v3	0.864
Listeria_ivanovii_subsp_londoniensis_202752_v1	Listeria_innocua_1642_v3	0.8515
Listeria_ivanovii_subsp_londoniensis_202752	Listeria_innocua_1642_v3	0.8596
Listeria_ivanovii_subsp_ivanovii_PAM_55_881621	Listeria_innocua_1642_v3	0.8636
Listeria_ivanovii_subsp_ivanovii_202751_v2	Listeria_innocua_1642_v3	0.8652
Listeria_ivanovii_subsp_ivanovii_202751_v1	Listeria_innocua_1642_v3	0.8652
Listeria_ivanovii_subsp_ivanovii_202751	Listeria_innocua_1642_v3	0.8652
Listeria_ivanovii_FSL_F6-596_702454	Listeria_innocua_1642_v3	0.7993
Listeria_ivanovii_1638	Listeria_innocua_1642_v3	0.8652
Listeria_innocua_FSL_S4-378_702456	Listeria_innocua_1642_v3	0.8383
Listeria_innocua_FSL_J1-023_702455	Listeria_innocua_1642_v3	0.8717
Listeria_innocua_Clip11262_272626	Listeria_innocua_1642_v3	0.9078
Listeria_innocua_1642_v4	Listeria_innocua_1642_v3	0.9161
Listeria_innocua_1642_v3	Listeria_innocua_1642_v3	0.0847
Listeria_welshimeri_1643	Listeria_innocua_1642_v4	0.8589
Listeria_welshimeri_serovar_6b_str_SLCC5334_386043	Listeria_innocua_1642_v4	0.8583
Listeria_seeligeri_serovar_1_2b_str_SLCC3954_683837	Listeria_innocua_1642_v4	0.8451
Listeria_seeligeri_FSL_S4-171_702452	Listeria_innocua_1642_v4	0.7947
Listeria_seeligeri_FSL_N1-067_702453	Listeria_innocua_1642_v4	0.808
Listeria_seeligeri_1640_v1	Listeria_innocua_1642_v4	0.8558
Listeria_seeligeri_1640	Listeria_innocua_1642_v4	0.8638
Listeria_monocytogenes_WSLC1042_1457188	Listeria_innocua_1642_v4	0.8858
Listeria_monocytogenes_WSLC1001_1457187	Listeria_innocua_1642_v4	0.8884
Listeria_monocytogenes_SLCC7179_879090	Listeria_innocua_1642_v4	0.8908
Listeria_monocytogenes_SLCC5850_882096	Listeria_innocua_1642_v4	0.892
Listeria_monocytogenes_SLCC2755_932919	Listeria_innocua_1642_v4	0.8947
Listeria_monocytogenes_SLCC2540_879089	Listeria_innocua_1642_v4	0.8851
Listeria_monocytogenes_SLCC2479_882020	Listeria_innocua_1642_v4	0.8878
Listeria_monocytogenes_SLCC2378_879088	Listeria_innocua_1642_v4	0.8871
Listeria_monocytogenes_SLCC2376_882097	Listeria_innocua_1642_v4	0.8972
Listeria_monocytogenes_SLCC2372_932920	Listeria_innocua_1642_v4	0.8958
Listeria_monocytogenes_serotype_4b_str_LL195_1230340	Listeria_innocua_1642_v4	0.8905
Listeria_monocytogenes_serotype_4b_str_LIS0087_1134669	Listeria_innocua_1642_v4	0.8688
Listeria_monocytogenes_serotype_4b_str_F2365_265669	Listeria_innocua_1642_v4	0.8905
Listeria_monocytogenes_serotype_4b_str_CLIP_80459_568819	Listeria_innocua_1642_v4	0.8937
Listeria_monocytogenes_serotype_4b_str_81-0861_1196161	Listeria_innocua_1642_v4	0.8833
Listeria_monocytogenes_serotype_4b_str_81-0592_1196160	Listeria_innocua_1642_v4	0.8843
Listeria_monocytogenes_serotype_4b_str_81-0558_1196159	Listeria_innocua_1642_v4	0.8853
Listeria_monocytogenes_serotype_4b_str_10-0809_1196162	Listeria_innocua_1642_v4	0.8846
Listeria_monocytogenes_serotype_4b_str_02-6680_1196175	Listeria_innocua_1642_v4	0.8939

Listeria_monocytogenes_serotype_4b_str_02-6679_1196174	Listeria_innocua_1642_v4	0.8939
Listeria_monocytogenes_serotype_4b_str_02-1792_1196173	Listeria_innocua_1642_v4	0.885
Listeria_monocytogenes_serotype_4b_str_02-1289_1196172	Listeria_innocua_1642_v4	0.885
Listeria_monocytogenes_serotype_4b_str_02-1103_1196171	Listeria_innocua_1642_v4	0.885
Listeria_monocytogenes_serotype_3c_str_10-5027_1196195	Listeria_innocua_1642_v4	0.8874
Listeria_monocytogenes_serotype_1_2c_str_10-5026_1196194	Listeria_innocua_1642_v4	0.8884
Listeria_monocytogenes_serotype_1_2c_str_10-5025_1196193	Listeria_innocua_1642_v4	0.8884
Listeria_monocytogenes_serotype_1_2b_str_10-0811_1196164	Listeria_innocua_1642_v4	0.8813
Listeria_monocytogenes_serotype_1_2b_str_10-0810_1196163	Listeria_innocua_1642_v4	0.8813
Listeria_monocytogenes_serotype_1_2a_str_99-6370_1196179	Listeria_innocua_1642_v4	0.8759
Listeria_monocytogenes_serotype_1_2a_str_98-2035_1196178	Listeria_innocua_1642_v4	0.8759
Listeria_monocytogenes_serotype_1_2a_str_95-0093_1196177	Listeria_innocua_1642_v4	0.8777
Listeria_monocytogenes_serotype_1_2a_str_88-0478_1196176	Listeria_innocua_1642_v4	0.8772
Listeria_monocytogenes_serotype_1_2a_str_10-5024_1196192	Listeria_innocua_1642_v4	0.878
Listeria_monocytogenes_serotype_1_2a_str_10-4758_1196168	Listeria_innocua_1642_v4	0.8877
Listeria_monocytogenes_serotype_1_2a_str_10-4754_1196167	Listeria_innocua_1642_v4	0.8877
Listeria_monocytogenes_serotype_1_2a_str_10-1321_1196191	Listeria_innocua_1642_v4	0.878
Listeria_monocytogenes_serotype_1_2a_str_10-1047_1196190	Listeria_innocua_1642_v4	0.8779
Listeria_monocytogenes_serotype_1_2a_str_10-1046_1196189	Listeria_innocua_1642_v4	0.8779
Listeria_monocytogenes_serotype_1_2a_str_10-0934_1196170	Listeria_innocua_1642_v4	0.8923
Listeria_monocytogenes_serotype_1_2a_str_10-0933_1196169	Listeria_innocua_1642_v4	0.8923
Listeria_monocytogenes_serotype_1_2a_str_10-0815_1196188	Listeria_innocua_1642_v4	0.8757
Listeria_monocytogenes_serotype_1_2a_str_10-0814_1196187	Listeria_innocua_1642_v4	0.878
Listeria_monocytogenes_serotype_1_2a_str_10-0813_1196166	Listeria_innocua_1642_v4	0.8866
Listeria_monocytogenes_serotype_1_2a_str_10-0812_1196165	Listeria_innocua_1642_v4	0.8866
Listeria_monocytogenes_serotype_1_2a_str_08-7669_1196186	Listeria_innocua_1642_v4	0.8815
Listeria_monocytogenes_serotype_1_2a_str_08-7374_1196185	Listeria_innocua_1642_v4	0.878
Listeria_monocytogenes_serotype_1_2a_str_08-6997_1196184	Listeria_innocua_1642_v4	0.8757
Listeria_monocytogenes_serotype_1_2a_str_08-6569_1196183	Listeria_innocua_1642_v4	0.8757
Listeria_monocytogenes_serotype_1_2a_str_08-6056_1196182	Listeria_innocua_1642_v4	0.8757
Listeria_monocytogenes_serotype_1_2a_str_04-5457_1196181	Listeria_innocua_1642_v4	0.878
Listeria_monocytogenes_serotype_1_2a_str_02-5993_1196180	Listeria_innocua_1642_v4	0.8777
Listeria_monocytogenes_R479a_1437838	Listeria_innocua_1642_v4	0.8853
Listeria_monocytogenes_N53-1_1234142	Listeria_innocua_1642_v4	0.8483
Listeria_monocytogenes_M7_1030009	Listeria_innocua_1642_v4	0.8742
Listeria_monocytogenes_LO28_393132	Listeria_innocua_1642_v4	0.8861
Listeria_monocytogenes_L99_563174	Listeria_innocua_1642_v4	0.8749
Listeria_monocytogenes_L312_882094	Listeria_innocua_1642_v4	0.8937
Listeria_monocytogenes_J1816_930781	Listeria_innocua_1642_v4	0.8789
Listeria_monocytogenes_J1-220_930782	Listeria_innocua_1642_v4	0.8841
Listeria_monocytogenes_J0161_393130	Listeria_innocua_1642_v4	0.8813
Listeria_monocytogenes_HCC23_552536	Listeria_innocua_1642_v4	0.8745
Listeria_monocytogenes_FSL_R2-561_393126	Listeria_innocua_1642_v4	0.8868
Listeria_monocytogenes_FSL_J1-208_393119	Listeria_innocua_1642_v4	0.8853
Listeria_monocytogenes_FSL_J1-175_393118	Listeria_innocua_1642_v4	0.8873
Listeria_monocytogenes_Finland_1998_393127	Listeria_innocua_1642_v4	0.8907
Listeria_monocytogenes_EGD-e_169963_v1	Listeria_innocua_1642_v4	0.8879
Listeria_monocytogenes_EGD-e_169963	Listeria_innocua_1642_v4	0.8883
Listeria_monocytogenes_EGD_1334565	Listeria_innocua_1642_v4	0.8927
Listeria_monocytogenes_CFSAN002349_1299895	Listeria_innocua_1642_v4	0.8882
Listeria_monocytogenes_ATCC_19117_882095_v1	Listeria_innocua_1642_v4	0.8883
Listeria_monocytogenes_ATCC_19117_882095	Listeria_innocua_1642_v4	0.8883
Listeria_monocytogenes_6179_1288295	Listeria_innocua_1642_v4	0.8858
Listeria_monocytogenes_1639_v9	Listeria_innocua_1642_v4	0.8876
Listeria_monocytogenes_1639_v99	Listeria_innocua_1642_v4	0.8891
Listeria_monocytogenes_1639_v98	Listeria_innocua_1642_v4	0.8827
Listeria_monocytogenes_1639_v97	Listeria_innocua_1642_v4	0.8846
Listeria_monocytogenes_1639_v96	Listeria_innocua_1642_v4	0.8877
Listeria_monocytogenes_1639_v95	Listeria_innocua_1642_v4	0.8878
Listeria_monocytogenes_1639_v94	Listeria_innocua_1642_v4	0.8881
Listeria_monocytogenes_1639_v93	Listeria_innocua_1642_v4	0.8834
Listeria_monocytogenes_1639_v92	Listeria_innocua_1642_v4	0.8902
Listeria_monocytogenes_1639_v91	Listeria_innocua_1642_v4	0.8871
Listeria_monocytogenes_1639_v90	Listeria_innocua_1642_v4	0.8827
Listeria_monocytogenes_1639_v8	Listeria_innocua_1642_v4	0.8844
Listeria_monocytogenes_1639_v89	Listeria_innocua_1642_v4	0.89
Listeria_monocytogenes_1639_v88	Listeria_innocua_1642_v4	0.8835
Listeria_monocytogenes_1639_v87	Listeria_innocua_1642_v4	0.8853
Listeria_monocytogenes_1639_v86	Listeria_innocua_1642_v4	0.885
Listeria_monocytogenes_1639_v85	Listeria_innocua_1642_v4	0.8838
Listeria_monocytogenes_1639_v84	Listeria_innocua_1642_v4	0.8843
Listeria_monocytogenes_1639_v83	Listeria_innocua_1642_v4	0.8837
Listeria_monocytogenes_1639_v82	Listeria_innocua_1642_v4	0.8831
Listeria_monocytogenes_1639_v81	Listeria_innocua_1642_v4	0.8842
Listeria_monocytogenes_1639_v80	Listeria_innocua_1642_v4	0.8843
Listeria_monocytogenes_1639_v7	Listeria_innocua_1642_v4	0.8844
Listeria_monocytogenes_1639_v79	Listeria_innocua_1642_v4	0.8815
Listeria_monocytogenes_1639_v78	Listeria_innocua_1642_v4	0.8835
Listeria_monocytogenes_1639_v77	Listeria_innocua_1642_v4	0.882
Listeria_monocytogenes_1639_v76	Listeria_innocua_1642_v4	0.8793
Listeria_monocytogenes_1639_v75	Listeria_innocua_1642_v4	0.8749
Listeria_monocytogenes_1639_v74	Listeria_innocua_1642_v4	0.8857
Listeria_monocytogenes_1639_v73	Listeria_innocua_1642_v4	0.8957
Listeria_monocytogenes_1639_v72	Listeria_innocua_1642_v4	0.884
Listeria_monocytogenes_1639_v71	Listeria_innocua_1642_v4	0.8758
Listeria_monocytogenes_1639_v70	Listeria_innocua_1642_v4	0.8914
Listeria_monocytogenes_1639_v6	Listeria_innocua_1642_v4	0.8838
Listeria_monocytogenes_1639_v69	Listeria_innocua_1642_v4	0.8846
Listeria_monocytogenes_1639_v68	Listeria_innocua_1642_v4	0.883
Listeria_monocytogenes_1639_v67	Listeria_innocua_1642_v4	0.8838
Listeria_monocytogenes_1639_v66	Listeria_innocua_1642_v4	0.8868

Listeria_monocytogenes_1639_v65	Listeria_innocua_1642_v4	0.8809
Listeria_monocytogenes_1639_v64	Listeria_innocua_1642_v4	0.885
Listeria_monocytogenes_1639_v63	Listeria_innocua_1642_v4	0.8892
Listeria_monocytogenes_1639_v62	Listeria_innocua_1642_v4	0.8807
Listeria_monocytogenes_1639_v61	Listeria_innocua_1642_v4	0.8801
Listeria_monocytogenes_1639_v60	Listeria_innocua_1642_v4	0.8823
Listeria_monocytogenes_1639_v5	Listeria_innocua_1642_v4	0.8873
Listeria_monocytogenes_1639_v59	Listeria_innocua_1642_v4	0.8877
Listeria_monocytogenes_1639_v58	Listeria_innocua_1642_v4	0.8848
Listeria_monocytogenes_1639_v57	Listeria_innocua_1642_v4	0.8972
Listeria_monocytogenes_1639_v56	Listeria_innocua_1642_v4	0.8844
Listeria_monocytogenes_1639_v55	Listeria_innocua_1642_v4	0.8846
Listeria_monocytogenes_1639_v54	Listeria_innocua_1642_v4	0.8811
Listeria_monocytogenes_1639_v53	Listeria_innocua_1642_v4	0.8801
Listeria_monocytogenes_1639_v52	Listeria_innocua_1642_v4	0.8855
Listeria_monocytogenes_1639_v51	Listeria_innocua_1642_v4	0.8894
Listeria_monocytogenes_1639_v50	Listeria_innocua_1642_v4	0.8769
Listeria_monocytogenes_1639_v4	Listeria_innocua_1642_v4	0.896
Listeria_monocytogenes_1639_v49	Listeria_innocua_1642_v4	0.8829
Listeria_monocytogenes_1639_v48	Listeria_innocua_1642_v4	0.8852
Listeria_monocytogenes_1639_v47	Listeria_innocua_1642_v4	0.8787
Listeria_monocytogenes_1639_v46	Listeria_innocua_1642_v4	0.8806
Listeria_monocytogenes_1639_v45	Listeria_innocua_1642_v4	0.8796
Listeria_monocytogenes_1639_v44	Listeria_innocua_1642_v4	0.878
Listeria_monocytogenes_1639_v43	Listeria_innocua_1642_v4	0.878
Listeria_monocytogenes_1639_v42	Listeria_innocua_1642_v4	0.8729
Listeria_monocytogenes_1639_v41	Listeria_innocua_1642_v4	0.8736
Listeria_monocytogenes_1639_v40	Listeria_innocua_1642_v4	0.8926
Listeria_monocytogenes_1639_v3	Listeria_innocua_1642_v4	0.8967
Listeria_monocytogenes_1639_v39	Listeria_innocua_1642_v4	0.8793
Listeria_monocytogenes_1639_v38	Listeria_innocua_1642_v4	0.8795
Listeria_monocytogenes_1639_v37	Listeria_innocua_1642_v4	0.8931
Listeria_monocytogenes_1639_v36	Listeria_innocua_1642_v4	0.8904
Listeria_monocytogenes_1639_v35	Listeria_innocua_1642_v4	0.8867
Listeria_monocytogenes_1639_v34	Listeria_innocua_1642_v4	0.8833
Listeria_monocytogenes_1639_v33	Listeria_innocua_1642_v4	0.8813
Listeria_monocytogenes_1639_v32	Listeria_innocua_1642_v4	0.8781
Listeria_monocytogenes_1639_v31	Listeria_innocua_1642_v4	0.8836
Listeria_monocytogenes_1639_v30	Listeria_innocua_1642_v4	0.8942
Listeria_monocytogenes_1639_v2	Listeria_innocua_1642_v4	0.888
Listeria_monocytogenes_1639_v29	Listeria_innocua_1642_v4	0.8898
Listeria_monocytogenes_1639_v28	Listeria_innocua_1642_v4	0.8835
Listeria_monocytogenes_1639_v27	Listeria_innocua_1642_v4	0.89
Listeria_monocytogenes_1639_v26	Listeria_innocua_1642_v4	0.8897
Listeria_monocytogenes_1639_v25	Listeria_innocua_1642_v4	0.8894
Listeria_monocytogenes_1639_v24	Listeria_innocua_1642_v4	0.8859
Listeria_monocytogenes_1639_v23	Listeria_innocua_1642_v4	0.8862
Listeria_monocytogenes_1639_v22	Listeria_innocua_1642_v4	0.8815
Listeria_monocytogenes_1639_v21	Listeria_innocua_1642_v4	0.8838
Listeria_monocytogenes_1639_v20	Listeria_innocua_1642_v4	0.8921
Listeria_monocytogenes_1639_v1	Listeria_innocua_1642_v4	0.8811
Listeria_monocytogenes_1639_v19	Listeria_innocua_1642_v4	0.8914
Listeria_monocytogenes_1639_v198	Listeria_innocua_1642_v4	0.8499
Listeria_monocytogenes_1639_v197	Listeria_innocua_1642_v4	0.8769
Listeria_monocytogenes_1639_v196	Listeria_innocua_1642_v4	0.876
Listeria_monocytogenes_1639_v195	Listeria_innocua_1642_v4	0.8963
Listeria_monocytogenes_1639_v194	Listeria_innocua_1642_v4	0.8979
Listeria_monocytogenes_1639_v193	Listeria_innocua_1642_v4	0.8975
Listeria_monocytogenes_1639_v192	Listeria_innocua_1642_v4	0.8767
Listeria_monocytogenes_1639_v191	Listeria_innocua_1642_v4	0.8946
Listeria_monocytogenes_1639_v190	Listeria_innocua_1642_v4	0.8951
Listeria_monocytogenes_1639_v18	Listeria_innocua_1642_v4	0.8932
Listeria_monocytogenes_1639_v189	Listeria_innocua_1642_v4	0.8943
Listeria_monocytogenes_1639_v188	Listeria_innocua_1642_v4	0.893
Listeria_monocytogenes_1639_v187	Listeria_innocua_1642_v4	0.8947
Listeria_monocytogenes_1639_v186	Listeria_innocua_1642_v4	0.8954
Listeria_monocytogenes_1639_v185	Listeria_innocua_1642_v4	0.8951
Listeria_monocytogenes_1639_v184	Listeria_innocua_1642_v4	0.8943
Listeria_monocytogenes_1639_v183	Listeria_innocua_1642_v4	0.888
Listeria_monocytogenes_1639_v182	Listeria_innocua_1642_v4	0.8896
Listeria_monocytogenes_1639_v181	Listeria_innocua_1642_v4	0.8902
Listeria_monocytogenes_1639_v180	Listeria_innocua_1642_v4	0.8902
Listeria_monocytogenes_1639_v17	Listeria_innocua_1642_v4	0.8932
Listeria_monocytogenes_1639_v179	Listeria_innocua_1642_v4	0.8868
Listeria_monocytogenes_1639_v178	Listeria_innocua_1642_v4	0.891
Listeria_monocytogenes_1639_v177	Listeria_innocua_1642_v4	0.8896
Listeria_monocytogenes_1639_v176	Listeria_innocua_1642_v4	0.8902
Listeria_monocytogenes_1639_v175	Listeria_innocua_1642_v4	0.893
Listeria_monocytogenes_1639_v174	Listeria_innocua_1642_v4	0.893
Listeria_monocytogenes_1639_v173	Listeria_innocua_1642_v4	0.8919
Listeria_monocytogenes_1639_v172	Listeria_innocua_1642_v4	0.8943
Listeria_monocytogenes_1639_v171	Listeria_innocua_1642_v4	0.9011
Listeria_monocytogenes_1639_v170	Listeria_innocua_1642_v4	0.8921
Listeria_monocytogenes_1639_v16	Listeria_innocua_1642_v4	0.8773
Listeria_monocytogenes_1639_v169	Listeria_innocua_1642_v4	0.8929
Listeria_monocytogenes_1639_v168	Listeria_innocua_1642_v4	0.8875
Listeria_monocytogenes_1639_v167	Listeria_innocua_1642_v4	0.8869
Listeria_monocytogenes_1639_v166	Listeria_innocua_1642_v4	0.8879
Listeria_monocytogenes_1639_v165	Listeria_innocua_1642_v4	0.8934
Listeria_monocytogenes_1639_v164	Listeria_innocua_1642_v4	0.8896
Listeria_monocytogenes_1639_v163	Listeria_innocua_1642_v4	0.8935

Listeria_monocytogenes_1639_v162	Listeria_innocua_1642_v4	0.8896
Listeria_monocytogenes_1639_v161	Listeria_innocua_1642_v4	0.8908
Listeria_monocytogenes_1639_v160	Listeria_innocua_1642_v4	0.8908
Listeria_monocytogenes_1639_v15	Listeria_innocua_1642_v4	0.9
Listeria_monocytogenes_1639_v159	Listeria_innocua_1642_v4	0.8896
Listeria_monocytogenes_1639_v158	Listeria_innocua_1642_v4	0.8905
Listeria_monocytogenes_1639_v157	Listeria_innocua_1642_v4	0.8905
Listeria_monocytogenes_1639_v156	Listeria_innocua_1642_v4	0.8855
Listeria_monocytogenes_1639_v155	Listeria_innocua_1642_v4	0.8884
Listeria_monocytogenes_1639_v154	Listeria_innocua_1642_v4	0.8877
Listeria_monocytogenes_1639_v153	Listeria_innocua_1642_v4	0.8884
Listeria_monocytogenes_1639_v152	Listeria_innocua_1642_v4	0.8899
Listeria_monocytogenes_1639_v151	Listeria_innocua_1642_v4	0.8899
Listeria_monocytogenes_1639_v150	Listeria_innocua_1642_v4	0.8913
Listeria_monocytogenes_1639_v14	Listeria_innocua_1642_v4	0.8763
Listeria_monocytogenes_1639_v149	Listeria_innocua_1642_v4	0.8927
Listeria_monocytogenes_1639_v148	Listeria_innocua_1642_v4	0.8886
Listeria_monocytogenes_1639_v147	Listeria_innocua_1642_v4	0.8836
Listeria_monocytogenes_1639_v146	Listeria_innocua_1642_v4	0.892
Listeria_monocytogenes_1639_v145	Listeria_innocua_1642_v4	0.8893
Listeria_monocytogenes_1639_v144	Listeria_innocua_1642_v4	0.893
Listeria_monocytogenes_1639_v143	Listeria_innocua_1642_v4	0.8925
Listeria_monocytogenes_1639_v142	Listeria_innocua_1642_v4	0.8934
Listeria_monocytogenes_1639_v141	Listeria_innocua_1642_v4	0.888
Listeria_monocytogenes_1639_v140	Listeria_innocua_1642_v4	0.8937
Listeria_monocytogenes_1639_v13	Listeria_innocua_1642_v4	0.9003
Listeria_monocytogenes_1639_v139	Listeria_innocua_1642_v4	0.8905
Listeria_monocytogenes_1639_v138	Listeria_innocua_1642_v4	0.8949
Listeria_monocytogenes_1639_v137	Listeria_innocua_1642_v4	0.8945
Listeria_monocytogenes_1639_v136	Listeria_innocua_1642_v4	0.8871
Listeria_monocytogenes_1639_v135	Listeria_innocua_1642_v4	0.8795
Listeria_monocytogenes_1639_v134	Listeria_innocua_1642_v4	0.8868
Listeria_monocytogenes_1639_v133	Listeria_innocua_1642_v4	0.8896
Listeria_monocytogenes_1639_v132	Listeria_innocua_1642_v4	0.8953
Listeria_monocytogenes_1639_v131	Listeria_innocua_1642_v4	0.8866
Listeria_monocytogenes_1639_v130	Listeria_innocua_1642_v4	0.8997
Listeria_monocytogenes_1639_v12	Listeria_innocua_1642_v4	0.8884
Listeria_monocytogenes_1639_v129	Listeria_innocua_1642_v4	0.8893
Listeria_monocytogenes_1639_v128	Listeria_innocua_1642_v4	0.8848
Listeria_monocytogenes_1639_v127	Listeria_innocua_1642_v4	0.8846
Listeria_monocytogenes_1639_v126	Listeria_innocua_1642_v4	0.8837
Listeria_monocytogenes_1639_v125	Listeria_innocua_1642_v4	0.8826
Listeria_monocytogenes_1639_v124	Listeria_innocua_1642_v4	0.8897
Listeria_monocytogenes_1639_v123	Listeria_innocua_1642_v4	0.8882
Listeria_monocytogenes_1639_v122	Listeria_innocua_1642_v4	0.8885
Listeria_monocytogenes_1639_v121	Listeria_innocua_1642_v4	0.8885
Listeria_monocytogenes_1639_v120	Listeria_innocua_1642_v4	0.8882
Listeria_monocytogenes_1639_v11	Listeria_innocua_1642_v4	0.8832
Listeria_monocytogenes_1639_v119	Listeria_innocua_1642_v4	0.8885
Listeria_monocytogenes_1639_v118	Listeria_innocua_1642_v4	0.8885
Listeria_monocytogenes_1639_v117	Listeria_innocua_1642_v4	0.8838
Listeria_monocytogenes_1639_v116	Listeria_innocua_1642_v4	0.8865
Listeria_monocytogenes_1639_v115	Listeria_innocua_1642_v4	0.8868
Listeria_monocytogenes_1639_v114	Listeria_innocua_1642_v4	0.885
Listeria_monocytogenes_1639_v113	Listeria_innocua_1642_v4	0.8904
Listeria_monocytogenes_1639_v112	Listeria_innocua_1642_v4	0.8858
Listeria_monocytogenes_1639_v111	Listeria_innocua_1642_v4	0.8983
Listeria_monocytogenes_1639_v110	Listeria_innocua_1642_v4	0.8869
Listeria_monocytogenes_1639_v10	Listeria_innocua_1642_v4	0.8862
Listeria_monocytogenes_1639_v109	Listeria_innocua_1642_v4	0.8865
Listeria_monocytogenes_1639_v108	Listeria_innocua_1642_v4	0.8876
Listeria_monocytogenes_1639_v107	Listeria_innocua_1642_v4	0.887
Listeria_monocytogenes_1639_v106	Listeria_innocua_1642_v4	0.8873
Listeria_monocytogenes_1639_v105	Listeria_innocua_1642_v4	0.8873
Listeria_monocytogenes_1639_v104	Listeria_innocua_1642_v4	0.8877
Listeria_monocytogenes_1639_v103	Listeria_innocua_1642_v4	0.8877
Listeria_monocytogenes_1639_v102	Listeria_innocua_1642_v4	0.885
Listeria_monocytogenes_1639_v101	Listeria_innocua_1642_v4	0.8868
Listeria_monocytogenes_1639_v100	Listeria_innocua_1642_v4	0.8847
Listeria_monocytogenes_1639	Listeria_innocua_1642_v4	0.8947
Listeria_monocytogenes_10403S_393133	Listeria_innocua_1642_v4	0.8915
Listeria_monocytogenes_08-5923_637381	Listeria_innocua_1642_v4	0.878
Listeria_monocytogenes_08-5578_653938	Listeria_innocua_1642_v4	0.877
Listeria_monocytogenes_07PF0776_1126011	Listeria_innocua_1642_v4	0.8868
Listeria_marthii_FSL_S4-120_702457	Listeria_innocua_1642_v4	0.8286
Listeria_marthii_529731_v1	Listeria_innocua_1642_v4	0.8739
Listeria_marthii_529731	Listeria_innocua_1642_v4	0.8718
Listeria_ivanovii_WSLC3009_1457190	Listeria_innocua_1642_v4	0.8479
Listeria_ivanovii_subsp_londoniensis_202752_v2	Listeria_innocua_1642_v4	0.8532
Listeria_ivanovii_subsp_londoniensis_202752_v1	Listeria_innocua_1642_v4	0.8499
Listeria_ivanovii_subsp_londoniensis_202752	Listeria_innocua_1642_v4	0.8451
Listeria_ivanovii_subsp_ivanovii_PAM_55_881621	Listeria_innocua_1642_v4	0.8473
Listeria_ivanovii_subsp_ivanovii_202751_v2	Listeria_innocua_1642_v4	0.8485
Listeria_ivanovii_subsp_ivanovii_202751_v1	Listeria_innocua_1642_v4	0.8485
Listeria_ivanovii_subsp_ivanovii_202751	Listeria_innocua_1642_v4	0.8485
Listeria_ivanovii_FSL_F6-596_702454	Listeria_innocua_1642_v4	0.7848
Listeria_ivanovii_1638	Listeria_innocua_1642_v4	0.8482
Listeria_innocua_FSL_S4-378_702456	Listeria_innocua_1642_v4	0.8185
Listeria_innocua_FSL_J1-023_702455	Listeria_innocua_1642_v4	0.8453
Listeria_innocua_Clip11262_272626	Listeria_innocua_1642_v4	0.9219
Listeria_innocua_1642_v4	Listeria_innocua_1642_v4	0.0686

Listeria_welshimeri_1643	Listeria_innocua_Clip11262_272626	0.8557
Listeria_welshimeri_serovar_6b_str_SLCC5334_386043	Listeria_innocua_Clip11262_272626	0.8553
Listeria_seeligeri_serovar_1_2b_str_SLCC3954_683837	Listeria_innocua_Clip11262_272626	0.847
Listeria_seeligeri_FSL_S4-171_702452	Listeria_innocua_Clip11262_272626	0.8024
Listeria_seeligeri_FSL_N1-067_702453	Listeria_innocua_Clip11262_272626	0.8151
Listeria_seeligeri_1640_v1	Listeria_innocua_Clip11262_272626	0.8514
Listeria_seeligeri_1640	Listeria_innocua_Clip11262_272626	0.8716
Listeria_monocytogenes_WSLC1042_1457188	Listeria_innocua_Clip11262_272626	0.8826
Listeria_monocytogenes_WSLC1001_1457187	Listeria_innocua_Clip11262_272626	0.8922
Listeria_monocytogenes_SLCC7179_879090	Listeria_innocua_Clip11262_272626	0.8942
Listeria_monocytogenes_SLCC5850_882096	Listeria_innocua_Clip11262_272626	0.8943
Listeria_monocytogenes_SLCC2755_932919	Listeria_innocua_Clip11262_272626	0.8791
Listeria_monocytogenes_SLCC2540_879089	Listeria_innocua_Clip11262_272626	0.8907
Listeria_monocytogenes_SLCC2479_882020	Listeria_innocua_Clip11262_272626	0.8922
Listeria_monocytogenes_SLCC2378_879088	Listeria_innocua_Clip11262_272626	0.8835
Listeria_monocytogenes_SLCC2376_882097	Listeria_innocua_Clip11262_272626	0.8957
Listeria_monocytogenes_SLCC2372_932920	Listeria_innocua_Clip11262_272626	0.8905
Listeria_monocytogenes_serotype_4b_str_LL195_1230340	Listeria_innocua_Clip11262_272626	0.8861
Listeria_monocytogenes_serotype_4b_str_LIS0087_1134669	Listeria_innocua_Clip11262_272626	0.8785
Listeria_monocytogenes_serotype_4b_str_F2365_265669	Listeria_innocua_Clip11262_272626	0.8861
Listeria_monocytogenes_serotype_4b_str_CLIP_80459_568819	Listeria_innocua_Clip11262_272626	0.889
Listeria_monocytogenes_serotype_4b_str_81-0861_1196161	Listeria_innocua_Clip11262_272626	0.8913
Listeria_monocytogenes_serotype_4b_str_81-0592_1196160	Listeria_innocua_Clip11262_272626	0.8923
Listeria_monocytogenes_serotype_4b_str_81-0558_1196159	Listeria_innocua_Clip11262_272626	0.8923
Listeria_monocytogenes_serotype_4b_str_10-0809_1196162	Listeria_innocua_Clip11262_272626	0.8927
Listeria_monocytogenes_serotype_4b_str_02-6680_1196175	Listeria_innocua_Clip11262_272626	0.8873
Listeria_monocytogenes_serotype_4b_str_02-6679_1196174	Listeria_innocua_Clip11262_272626	0.8873
Listeria_monocytogenes_serotype_4b_str_02-1792_1196173	Listeria_innocua_Clip11262_272626	0.892
Listeria_monocytogenes_serotype_4b_str_02-1289_1196172	Listeria_innocua_Clip11262_272626	0.892
Listeria_monocytogenes_serotype_4b_str_02-1103_1196171	Listeria_innocua_Clip11262_272626	0.892
Listeria_monocytogenes_serotype_3c_str_10-5027_1196195	Listeria_innocua_Clip11262_272626	0.8954
Listeria_monocytogenes_serotype_1_2c_str_10-5026_1196194	Listeria_innocua_Clip11262_272626	0.8958
Listeria_monocytogenes_serotype_1_2c_str_10-5025_1196193	Listeria_innocua_Clip11262_272626	0.8958
Listeria_monocytogenes_serotype_1_2b_str_10-0811_1196164	Listeria_innocua_Clip11262_272626	0.898
Listeria_monocytogenes_serotype_1_2b_str_10-0810_1196163	Listeria_innocua_Clip11262_272626	0.898
Listeria_monocytogenes_serotype_1_2a_str_99-6370_1196179	Listeria_innocua_Clip11262_272626	0.8838
Listeria_monocytogenes_serotype_1_2a_str_98-2035_1196178	Listeria_innocua_Clip11262_272626	0.8838
Listeria_monocytogenes_serotype_1_2a_str_95-0093_1196177	Listeria_innocua_Clip11262_272626	0.886
Listeria_monocytogenes_serotype_1_2a_str_88-0478_1196176	Listeria_innocua_Clip11262_272626	0.8863
Listeria_monocytogenes_serotype_1_2a_str_10-5024_1196192	Listeria_innocua_Clip11262_272626	0.8864
Listeria_monocytogenes_serotype_1_2a_str_10-4758_1196168	Listeria_innocua_Clip11262_272626	0.8897
Listeria_monocytogenes_serotype_1_2a_str_10-4754_1196167	Listeria_innocua_Clip11262_272626	0.89
Listeria_monocytogenes_serotype_1_2a_str_10-1321_1196191	Listeria_innocua_Clip11262_272626	0.8864
Listeria_monocytogenes_serotype_1_2a_str_10-1047_1196190	Listeria_innocua_Clip11262_272626	0.8863
Listeria_monocytogenes_serotype_1_2a_str_10-1046_1196189	Listeria_innocua_Clip11262_272626	0.8863
Listeria_monocytogenes_serotype_1_2a_str_10-0934_1196170	Listeria_innocua_Clip11262_272626	0.8942
Listeria_monocytogenes_serotype_1_2a_str_10-0933_1196169	Listeria_innocua_Clip11262_272626	0.8942
Listeria_monocytogenes_serotype_1_2a_str_10-0815_1196188	Listeria_innocua_Clip11262_272626	0.8844
Listeria_monocytogenes_serotype_1_2a_str_10-0814_1196187	Listeria_innocua_Clip11262_272626	0.8867
Listeria_monocytogenes_serotype_1_2a_str_10-0813_1196166	Listeria_innocua_Clip11262_272626	0.8987
Listeria_monocytogenes_serotype_1_2a_str_10-0812_1196165	Listeria_innocua_Clip11262_272626	0.8987
Listeria_monocytogenes_serotype_1_2a_str_08-7669_1196186	Listeria_innocua_Clip11262_272626	0.8867
Listeria_monocytogenes_serotype_1_2a_str_08-7374_1196185	Listeria_innocua_Clip11262_272626	0.8864
Listeria_monocytogenes_serotype_1_2a_str_08-6997_1196184	Listeria_innocua_Clip11262_272626	0.8844
Listeria_monocytogenes_serotype_1_2a_str_08-6569_1196183	Listeria_innocua_Clip11262_272626	0.8844
Listeria_monocytogenes_serotype_1_2a_str_08-6056_1196182	Listeria_innocua_Clip11262_272626	0.8844
Listeria_monocytogenes_serotype_1_2a_str_04-5457_1196181	Listeria_innocua_Clip11262_272626	0.8864
Listeria_monocytogenes_serotype_1_2a_str_02-5993_1196180	Listeria_innocua_Clip11262_272626	0.8864
Listeria_monocytogenes_R479a_1437838	Listeria_innocua_Clip11262_272626	0.8901
Listeria_monocytogenes_N53-1_1234142	Listeria_innocua_Clip11262_272626	0.8519
Listeria_monocytogenes_M7_1030009	Listeria_innocua_Clip11262_272626	0.8876
Listeria_monocytogenes_LO28_393132	Listeria_innocua_Clip11262_272626	0.8945
Listeria_monocytogenes_L99_563174	Listeria_innocua_Clip11262_272626	0.8879
Listeria_monocytogenes_L312_882094	Listeria_innocua_Clip11262_272626	0.889
Listeria_monocytogenes_J1816_930781	Listeria_innocua_Clip11262_272626	0.8884
Listeria_monocytogenes_J1-220_930782	Listeria_innocua_Clip11262_272626	0.8949
Listeria_monocytogenes_J0161_393130	Listeria_innocua_Clip11262_272626	0.8934
Listeria_monocytogenes_HCC23_552536	Listeria_innocua_Clip11262_272626	0.8872
Listeria_monocytogenes_FSL_R2-561_393126	Listeria_innocua_Clip11262_272626	0.8923
Listeria_monocytogenes_FSL_J1-208_393119	Listeria_innocua_Clip11262_272626	0.8714
Listeria_monocytogenes_FSL_J1-175_393118	Listeria_innocua_Clip11262_272626	0.8939
Listeria_monocytogenes_Finland_1998_393127	Listeria_innocua_Clip11262_272626	0.8871
Listeria_monocytogenes_EGD-e_169963_v1	Listeria_innocua_Clip11262_272626	0.8956
Listeria_monocytogenes_EGD-e_169963	Listeria_innocua_Clip11262_272626	0.896
Listeria_monocytogenes_EGD_1334565	Listeria_innocua_Clip11262_272626	0.895
Listeria_monocytogenes_CFSAN002349_1299895	Listeria_innocua_Clip11262_272626	0.8889
Listeria_monocytogenes_ATCC_19117_882095_v1	Listeria_innocua_Clip11262_272626	0.8865
Listeria_monocytogenes_ATCC_19117_882095	Listeria_innocua_Clip11262_272626	0.8861
Listeria_monocytogenes_6179_1288295	Listeria_innocua_Clip11262_272626	0.8974
Listeria_monocytogenes_1639_v9	Listeria_innocua_Clip11262_272626	0.9087
Listeria_monocytogenes_1639_v99	Listeria_innocua_Clip11262_272626	0.8961
Listeria_monocytogenes_1639_v98	Listeria_innocua_Clip11262_272626	0.8955
Listeria_monocytogenes_1639_v97	Listeria_innocua_Clip11262_272626	0.8901
Listeria_monocytogenes_1639_v96	Listeria_innocua_Clip11262_272626	0.8954
Listeria_monocytogenes_1639_v95	Listeria_innocua_Clip11262_272626	0.8954
Listeria_monocytogenes_1639_v94	Listeria_innocua_Clip11262_272626	0.8957
Listeria_monocytogenes_1639_v93	Listeria_innocua_Clip11262_272626	0.8812
Listeria_monocytogenes_1639_v92	Listeria_innocua_Clip11262_272626	0.8961
Listeria_monocytogenes_1639_v91	Listeria_innocua_Clip11262_272626	0.8955
Listeria_monocytogenes_1639_v90	Listeria_innocua_Clip11262_272626	0.8955
Listeria_monocytogenes_1639_v8	Listeria_innocua_Clip11262_272626	0.8824

Listeria_monocytogenes_1639_v89	Listeria_innocua_Clip11262_272626	0.8966
Listeria_monocytogenes_1639_v88	Listeria_innocua_Clip11262_272626	0.897
Listeria_monocytogenes_1639_v87	Listeria_innocua_Clip11262_272626	0.8974
Listeria_monocytogenes_1639_v86	Listeria_innocua_Clip11262_272626	0.897
Listeria_monocytogenes_1639_v85	Listeria_innocua_Clip11262_272626	0.8914
Listeria_monocytogenes_1639_v84	Listeria_innocua_Clip11262_272626	0.8935
Listeria_monocytogenes_1639_v83	Listeria_innocua_Clip11262_272626	0.8929
Listeria_monocytogenes_1639_v82	Listeria_innocua_Clip11262_272626	0.8922
Listeria_monocytogenes_1639_v81	Listeria_innocua_Clip11262_272626	0.8904
Listeria_monocytogenes_1639_v80	Listeria_innocua_Clip11262_272626	0.8982
Listeria_monocytogenes_1639_v7	Listeria_innocua_Clip11262_272626	0.8824
Listeria_monocytogenes_1639_v79	Listeria_innocua_Clip11262_272626	0.8877
Listeria_monocytogenes_1639_v78	Listeria_innocua_Clip11262_272626	0.8909
Listeria_monocytogenes_1639_v77	Listeria_innocua_Clip11262_272626	0.8893
Listeria_monocytogenes_1639_v76	Listeria_innocua_Clip11262_272626	0.8891
Listeria_monocytogenes_1639_v75	Listeria_innocua_Clip11262_272626	0.8879
Listeria_monocytogenes_1639_v74	Listeria_innocua_Clip11262_272626	0.892
Listeria_monocytogenes_1639_v73	Listeria_innocua_Clip11262_272626	0.8965
Listeria_monocytogenes_1639_v72	Listeria_innocua_Clip11262_272626	0.8906
Listeria_monocytogenes_1639_v71	Listeria_innocua_Clip11262_272626	0.8891
Listeria_monocytogenes_1639_v70	Listeria_innocua_Clip11262_272626	0.8962
Listeria_monocytogenes_1639_v6	Listeria_innocua_Clip11262_272626	0.882
Listeria_monocytogenes_1639_v69	Listeria_innocua_Clip11262_272626	0.8923
Listeria_monocytogenes_1639_v68	Listeria_innocua_Clip11262_272626	0.8875
Listeria_monocytogenes_1639_v67	Listeria_innocua_Clip11262_272626	0.8955
Listeria_monocytogenes_1639_v66	Listeria_innocua_Clip11262_272626	0.8887
Listeria_monocytogenes_1639_v65	Listeria_innocua_Clip11262_272626	0.8922
Listeria_monocytogenes_1639_v64	Listeria_innocua_Clip11262_272626	0.8848
Listeria_monocytogenes_1639_v63	Listeria_innocua_Clip11262_272626	0.9022
Listeria_monocytogenes_1639_v62	Listeria_innocua_Clip11262_272626	0.8909
Listeria_monocytogenes_1639_v61	Listeria_innocua_Clip11262_272626	0.8903
Listeria_monocytogenes_1639_v60	Listeria_innocua_Clip11262_272626	0.8882
Listeria_monocytogenes_1639_v5	Listeria_innocua_Clip11262_272626	0.8937
Listeria_monocytogenes_1639_v59	Listeria_innocua_Clip11262_272626	0.8888
Listeria_monocytogenes_1639_v58	Listeria_innocua_Clip11262_272626	0.895
Listeria_monocytogenes_1639_v57	Listeria_innocua_Clip11262_272626	0.9059
Listeria_monocytogenes_1639_v56	Listeria_innocua_Clip11262_272626	0.8943
Listeria_monocytogenes_1639_v55	Listeria_innocua_Clip11262_272626	0.8976
Listeria_monocytogenes_1639_v54	Listeria_innocua_Clip11262_272626	0.8896
Listeria_monocytogenes_1639_v53	Listeria_innocua_Clip11262_272626	0.8769
Listeria_monocytogenes_1639_v52	Listeria_innocua_Clip11262_272626	0.8961
Listeria_monocytogenes_1639_v51	Listeria_innocua_Clip11262_272626	0.8928
Listeria_monocytogenes_1639_v50	Listeria_innocua_Clip11262_272626	0.8856
Listeria_monocytogenes_1639_v4	Listeria_innocua_Clip11262_272626	0.9003
Listeria_monocytogenes_1639_v49	Listeria_innocua_Clip11262_272626	0.8786
Listeria_monocytogenes_1639_v48	Listeria_innocua_Clip11262_272626	0.8942
Listeria_monocytogenes_1639_v47	Listeria_innocua_Clip11262_272626	0.8857
Listeria_monocytogenes_1639_v46	Listeria_innocua_Clip11262_272626	0.8865
Listeria_monocytogenes_1639_v45	Listeria_innocua_Clip11262_272626	0.8839
Listeria_monocytogenes_1639_v44	Listeria_innocua_Clip11262_272626	0.8864
Listeria_monocytogenes_1639_v43	Listeria_innocua_Clip11262_272626	0.8864
Listeria_monocytogenes_1639_v42	Listeria_innocua_Clip11262_272626	0.8802
Listeria_monocytogenes_1639_v41	Listeria_innocua_Clip11262_272626	0.8822
Listeria_monocytogenes_1639_v40	Listeria_innocua_Clip11262_272626	0.889
Listeria_monocytogenes_1639_v3	Listeria_innocua_Clip11262_272626	0.901
Listeria_monocytogenes_1639_v39	Listeria_innocua_Clip11262_272626	0.8975
Listeria_monocytogenes_1639_v38	Listeria_innocua_Clip11262_272626	0.8817
Listeria_monocytogenes_1639_v37	Listeria_innocua_Clip11262_272626	0.8971
Listeria_monocytogenes_1639_v36	Listeria_innocua_Clip11262_272626	0.88
Listeria_monocytogenes_1639_v35	Listeria_innocua_Clip11262_272626	0.8863
Listeria_monocytogenes_1639_v34	Listeria_innocua_Clip11262_272626	0.888
Listeria_monocytogenes_1639_v33	Listeria_innocua_Clip11262_272626	0.898
Listeria_monocytogenes_1639_v32	Listeria_innocua_Clip11262_272626	0.8893
Listeria_monocytogenes_1639_v31	Listeria_innocua_Clip11262_272626	0.8884
Listeria_monocytogenes_1639_v30	Listeria_innocua_Clip11262_272626	0.8993
Listeria_monocytogenes_1639_v2	Listeria_innocua_Clip11262_272626	0.893
Listeria_monocytogenes_1639_v29	Listeria_innocua_Clip11262_272626	0.8906
Listeria_monocytogenes_1639_v28	Listeria_innocua_Clip11262_272626	0.8896
Listeria_monocytogenes_1639_v27	Listeria_innocua_Clip11262_272626	0.8854
Listeria_monocytogenes_1639_v26	Listeria_innocua_Clip11262_272626	0.8862
Listeria_monocytogenes_1639_v25	Listeria_innocua_Clip11262_272626	0.8858
Listeria_monocytogenes_1639_v24	Listeria_innocua_Clip11262_272626	0.8946
Listeria_monocytogenes_1639_v23	Listeria_innocua_Clip11262_272626	0.8939
Listeria_monocytogenes_1639_v22	Listeria_innocua_Clip11262_272626	0.9044
Listeria_monocytogenes_1639_v21	Listeria_innocua_Clip11262_272626	0.8946
Listeria_monocytogenes_1639_v20	Listeria_innocua_Clip11262_272626	0.8928
Listeria_monocytogenes_1639_v1	Listeria_innocua_Clip11262_272626	0.8932
Listeria_monocytogenes_1639_v19	Listeria_innocua_Clip11262_272626	0.8922
Listeria_monocytogenes_1639_v198	Listeria_innocua_Clip11262_272626	0.8528
Listeria_monocytogenes_1639_v197	Listeria_innocua_Clip11262_272626	0.8838
Listeria_monocytogenes_1639_v196	Listeria_innocua_Clip11262_272626	0.8784
Listeria_monocytogenes_1639_v195	Listeria_innocua_Clip11262_272626	0.8926
Listeria_monocytogenes_1639_v194	Listeria_innocua_Clip11262_272626	0.8961
Listeria_monocytogenes_1639_v193	Listeria_innocua_Clip11262_272626	0.8957
Listeria_monocytogenes_1639_v192	Listeria_innocua_Clip11262_272626	0.8698
Listeria_monocytogenes_1639_v191	Listeria_innocua_Clip11262_272626	0.8898
Listeria_monocytogenes_1639_v190	Listeria_innocua_Clip11262_272626	0.8936
Listeria_monocytogenes_1639_v18	Listeria_innocua_Clip11262_272626	0.9094
Listeria_monocytogenes_1639_v189	Listeria_innocua_Clip11262_272626	0.8926
Listeria_monocytogenes_1639_v188	Listeria_innocua_Clip11262_272626	0.8897
Listeria_monocytogenes_1639_v187	Listeria_innocua_Clip11262_272626	0.8936

Listeria_monocytogenes_1639_v186	Listeria_innocua_Clip11262_272626	0.8936
Listeria_monocytogenes_1639_v185	Listeria_innocua_Clip11262_272626	0.894
Listeria_monocytogenes_1639_v184	Listeria_innocua_Clip11262_272626	0.8929
Listeria_monocytogenes_1639_v183	Listeria_innocua_Clip11262_272626	0.8839
Listeria_monocytogenes_1639_v182	Listeria_innocua_Clip11262_272626	0.8852
Listeria_monocytogenes_1639_v181	Listeria_innocua_Clip11262_272626	0.8833
Listeria_monocytogenes_1639_v180	Listeria_innocua_Clip11262_272626	0.8833
Listeria_monocytogenes_1639_v17	Listeria_innocua_Clip11262_272626	0.9097
Listeria_monocytogenes_1639_v179	Listeria_innocua_Clip11262_272626	0.8884
Listeria_monocytogenes_1639_v178	Listeria_innocua_Clip11262_272626	0.8877
Listeria_monocytogenes_1639_v177	Listeria_innocua_Clip11262_272626	0.8878
Listeria_monocytogenes_1639_v176	Listeria_innocua_Clip11262_272626	0.8896
Listeria_monocytogenes_1639_v175	Listeria_innocua_Clip11262_272626	0.892
Listeria_monocytogenes_1639_v174	Listeria_innocua_Clip11262_272626	0.8912
Listeria_monocytogenes_1639_v173	Listeria_innocua_Clip11262_272626	0.8923
Listeria_monocytogenes_1639_v172	Listeria_innocua_Clip11262_272626	0.8895
Listeria_monocytogenes_1639_v171	Listeria_innocua_Clip11262_272626	0.8901
Listeria_monocytogenes_1639_v170	Listeria_innocua_Clip11262_272626	0.8914
Listeria_monocytogenes_1639_v16	Listeria_innocua_Clip11262_272626	0.8813
Listeria_monocytogenes_1639_v169	Listeria_innocua_Clip11262_272626	0.8908
Listeria_monocytogenes_1639_v168	Listeria_innocua_Clip11262_272626	0.8835
Listeria_monocytogenes_1639_v167	Listeria_innocua_Clip11262_272626	0.8921
Listeria_monocytogenes_1639_v166	Listeria_innocua_Clip11262_272626	0.8839
Listeria_monocytogenes_1639_v165	Listeria_innocua_Clip11262_272626	0.8904
Listeria_monocytogenes_1639_v164	Listeria_innocua_Clip11262_272626	0.8856
Listeria_monocytogenes_1639_v163	Listeria_innocua_Clip11262_272626	0.8909
Listeria_monocytogenes_1639_v162	Listeria_innocua_Clip11262_272626	0.8856
Listeria_monocytogenes_1639_v161	Listeria_innocua_Clip11262_272626	0.886
Listeria_monocytogenes_1639_v160	Listeria_innocua_Clip11262_272626	0.8857
Listeria_monocytogenes_1639_v15	Listeria_innocua_Clip11262_272626	0.9089
Listeria_monocytogenes_1639_v159	Listeria_innocua_Clip11262_272626	0.8878
Listeria_monocytogenes_1639_v158	Listeria_innocua_Clip11262_272626	0.8861
Listeria_monocytogenes_1639_v157	Listeria_innocua_Clip11262_272626	0.8861
Listeria_monocytogenes_1639_v156	Listeria_innocua_Clip11262_272626	0.8924
Listeria_monocytogenes_1639_v155	Listeria_innocua_Clip11262_272626	0.8944
Listeria_monocytogenes_1639_v154	Listeria_innocua_Clip11262_272626	0.8904
Listeria_monocytogenes_1639_v153	Listeria_innocua_Clip11262_272626	0.8958
Listeria_monocytogenes_1639_v152	Listeria_innocua_Clip11262_272626	0.8878
Listeria_monocytogenes_1639_v151	Listeria_innocua_Clip11262_272626	0.8878
Listeria_monocytogenes_1639_v150	Listeria_innocua_Clip11262_272626	0.8925
Listeria_monocytogenes_1639_v14	Listeria_innocua_Clip11262_272626	0.8882
Listeria_monocytogenes_1639_v149	Listeria_innocua_Clip11262_272626	0.8953
Listeria_monocytogenes_1639_v148	Listeria_innocua_Clip11262_272626	0.8868
Listeria_monocytogenes_1639_v147	Listeria_innocua_Clip11262_272626	0.884
Listeria_monocytogenes_1639_v146	Listeria_innocua_Clip11262_272626	0.8899
Listeria_monocytogenes_1639_v145	Listeria_innocua_Clip11262_272626	0.8853
Listeria_monocytogenes_1639_v144	Listeria_innocua_Clip11262_272626	0.8883
Listeria_monocytogenes_1639_v143	Listeria_innocua_Clip11262_272626	0.8896
Listeria_monocytogenes_1639_v142	Listeria_innocua_Clip11262_272626	0.8886
Listeria_monocytogenes_1639_v141	Listeria_innocua_Clip11262_272626	0.8892
Listeria_monocytogenes_1639_v140	Listeria_innocua_Clip11262_272626	0.8893
Listeria_monocytogenes_1639_v13	Listeria_innocua_Clip11262_272626	0.9085
Listeria_monocytogenes_1639_v139	Listeria_innocua_Clip11262_272626	0.8858
Listeria_monocytogenes_1639_v138	Listeria_innocua_Clip11262_272626	0.8905
Listeria_monocytogenes_1639_v137	Listeria_innocua_Clip11262_272626	0.8901
Listeria_monocytogenes_1639_v136	Listeria_innocua_Clip11262_272626	0.8886
Listeria_monocytogenes_1639_v135	Listeria_innocua_Clip11262_272626	0.8886
Listeria_monocytogenes_1639_v134	Listeria_innocua_Clip11262_272626	0.8832
Listeria_monocytogenes_1639_v133	Listeria_innocua_Clip11262_272626	0.8941
Listeria_monocytogenes_1639_v132	Listeria_innocua_Clip11262_272626	0.8842
Listeria_monocytogenes_1639_v131	Listeria_innocua_Clip11262_272626	0.8914
Listeria_monocytogenes_1639_v130	Listeria_innocua_Clip11262_272626	0.8886
Listeria_monocytogenes_1639_v12	Listeria_innocua_Clip11262_272626	0.8863
Listeria_monocytogenes_1639_v129	Listeria_innocua_Clip11262_272626	0.8916
Listeria_monocytogenes_1639_v128	Listeria_innocua_Clip11262_272626	0.8863
Listeria_monocytogenes_1639_v127	Listeria_innocua_Clip11262_272626	0.8964
Listeria_monocytogenes_1639_v126	Listeria_innocua_Clip11262_272626	0.8849
Listeria_monocytogenes_1639_v125	Listeria_innocua_Clip11262_272626	0.8882
Listeria_monocytogenes_1639_v124	Listeria_innocua_Clip11262_272626	0.8824
Listeria_monocytogenes_1639_v123	Listeria_innocua_Clip11262_272626	0.8889
Listeria_monocytogenes_1639_v122	Listeria_innocua_Clip11262_272626	0.8889
Listeria_monocytogenes_1639_v121	Listeria_innocua_Clip11262_272626	0.8889
Listeria_monocytogenes_1639_v120	Listeria_innocua_Clip11262_272626	0.8889
Listeria_monocytogenes_1639_v11	Listeria_innocua_Clip11262_272626	0.8943
Listeria_monocytogenes_1639_v119	Listeria_innocua_Clip11262_272626	0.8889
Listeria_monocytogenes_1639_v118	Listeria_innocua_Clip11262_272626	0.8889
Listeria_monocytogenes_1639_v117	Listeria_innocua_Clip11262_272626	0.8879
Listeria_monocytogenes_1639_v116	Listeria_innocua_Clip11262_272626	0.8832
Listeria_monocytogenes_1639_v115	Listeria_innocua_Clip11262_272626	0.8835
Listeria_monocytogenes_1639_v114	Listeria_innocua_Clip11262_272626	0.8968
Listeria_monocytogenes_1639_v113	Listeria_innocua_Clip11262_272626	0.8938
Listeria_monocytogenes_1639_v112	Listeria_innocua_Clip11262_272626	0.8943
Listeria_monocytogenes_1639_v111	Listeria_innocua_Clip11262_272626	0.8856
Listeria_monocytogenes_1639_v110	Listeria_innocua_Clip11262_272626	0.8957
Listeria_monocytogenes_1639_v10	Listeria_innocua_Clip11262_272626	0.8994
Listeria_monocytogenes_1639_v109	Listeria_innocua_Clip11262_272626	0.8993
Listeria_monocytogenes_1639_v108	Listeria_innocua_Clip11262_272626	0.8931
Listeria_monocytogenes_1639_v107	Listeria_innocua_Clip11262_272626	0.8852
Listeria_monocytogenes_1639_v106	Listeria_innocua_Clip11262_272626	0.8855
Listeria_monocytogenes_1639_v105	Listeria_innocua_Clip11262_272626	0.8855
Listeria_monocytogenes_1639_v104	Listeria_innocua_Clip11262_272626	0.8855

Listeria_monocytogenes_1639_v103	Listeria_innocua_Clip11262_272626	0.8859
Listeria_monocytogenes_1639_v102	Listeria_innocua_Clip11262_272626	0.8957
Listeria_monocytogenes_1639_v101	Listeria_innocua_Clip11262_272626	0.8912
Listeria_monocytogenes_1639_v100	Listeria_innocua_Clip11262_272626	0.8894
Listeria_monocytogenes_1639	Listeria_innocua_Clip11262_272626	0.8933
Listeria_monocytogenes_10403S_393133	Listeria_innocua_Clip11262_272626	0.8974
Listeria_monocytogenes_08-5923_637381	Listeria_innocua_Clip11262_272626	0.8864
Listeria_monocytogenes_08-5578_653938	Listeria_innocua_Clip11262_272626	0.8813
Listeria_monocytogenes_07PF0776_1126011	Listeria_innocua_Clip11262_272626	0.8835
Listeria_marthii_FSL_S4-120_702457	Listeria_innocua_Clip11262_272626	0.8238
Listeria_marthii_529731_v1	Listeria_innocua_Clip11262_272626	0.8777
Listeria_marthii_529731	Listeria_innocua_Clip11262_272626	0.866
Listeria_ivanovii_WSLC3009_1457190	Listeria_innocua_Clip11262_272626	0.8464
Listeria_ivanovii_subsp_londoniensis_202752_v2	Listeria_innocua_Clip11262_272626	0.8456
Listeria_ivanovii_subsp_londoniensis_202752_v1	Listeria_innocua_Clip11262_272626	0.8507
Listeria_ivanovii_subsp_londoniensis_202752	Listeria_innocua_Clip11262_272626	0.8495
Listeria_ivanovii_subsp_ivanovii_PAM_55_881621	Listeria_innocua_Clip11262_272626	0.8452
Listeria_ivanovii_subsp_ivanovii_202751_v2	Listeria_innocua_Clip11262_272626	0.8468
Listeria_ivanovii_subsp_ivanovii_202751_v1	Listeria_innocua_Clip11262_272626	0.8468
Listeria_ivanovii_subsp_ivanovii_202751	Listeria_innocua_Clip11262_272626	0.8468
Listeria_ivanovii_FSL_F6-596_702454	Listeria_innocua_Clip11262_272626	0.7901
Listeria_ivanovii_1638	Listeria_innocua_Clip11262_272626	0.8468
Listeria_innocua_FSL_S4-378_702456	Listeria_innocua_Clip11262_272626	0.8175
Listeria_innocua_FSL_J1-023_702455	Listeria_innocua_Clip11262_272626	0.8439
Listeria_innocua_Clip11262_272626	Listeria_innocua_Clip11262_272626	0.0873
Listeria_welshimeri_1643	Listeria_innocua_FSL_J1-023_702455	0.8482
Listeria_welshimeri_serovar_6b_str_SLCC5334_386043	Listeria_innocua_FSL_J1-023_702455	0.8479
Listeria_seeligeri_serovar_1_2b_str_SLCC3954_683837	Listeria_innocua_FSL_J1-023_702455	0.8444
Listeria_seeligeri_FSL_S4-171_702452	Listeria_innocua_FSL_J1-023_702455	0.7958
Listeria_seeligeri_FSL_N1-067_702453	Listeria_innocua_FSL_J1-023_702455	0.795
Listeria_seeligeri_1640_v1	Listeria_innocua_FSL_J1-023_702455	0.8444
Listeria_seeligeri_1640	Listeria_innocua_FSL_J1-023_702455	0.8387
Listeria_monocytogenes_WSLC1042_1457188	Listeria_innocua_FSL_J1-023_702455	0.8802
Listeria_monocytogenes_WSLC1001_1457187	Listeria_innocua_FSL_J1-023_702455	0.869
Listeria_monocytogenes_SLCC7179_879090	Listeria_innocua_FSL_J1-023_702455	0.8764
Listeria_monocytogenes_SLCC5850_882096	Listeria_innocua_FSL_J1-023_702455	0.8727
Listeria_monocytogenes_SLCC2755_932919	Listeria_innocua_FSL_J1-023_702455	0.864
Listeria_monocytogenes_SLCC2540_879089	Listeria_innocua_FSL_J1-023_702455	0.8678
Listeria_monocytogenes_SLCC2479_882020	Listeria_innocua_FSL_J1-023_702455	0.8672
Listeria_monocytogenes_SLCC2378_879088	Listeria_innocua_FSL_J1-023_702455	0.8812
Listeria_monocytogenes_SLCC2376_882097	Listeria_innocua_FSL_J1-023_702455	0.8882
Listeria_monocytogenes_SLCC2372_932920	Listeria_innocua_FSL_J1-023_702455	0.8602
Listeria_monocytogenes_serotype_4b_str_LL195_1230340	Listeria_innocua_FSL_J1-023_702455	0.8864
Listeria_monocytogenes_serotype_4b_str_LIS0087_1134669	Listeria_innocua_FSL_J1-023_702455	0.8497
Listeria_monocytogenes_serotype_4b_str_F2365_265669	Listeria_innocua_FSL_J1-023_702455	0.8864
Listeria_monocytogenes_serotype_4b_str_CLIP_80459_568819	Listeria_innocua_FSL_J1-023_702455	0.8827
Listeria_monocytogenes_serotype_4b_str_81-0861_1196161	Listeria_innocua_FSL_J1-023_702455	0.8717
Listeria_monocytogenes_serotype_4b_str_81-0592_1196160	Listeria_innocua_FSL_J1-023_702455	0.8728
Listeria_monocytogenes_serotype_4b_str_81-0558_1196159	Listeria_innocua_FSL_J1-023_702455	0.8723
Listeria_monocytogenes_serotype_4b_str_10-0809_1196162	Listeria_innocua_FSL_J1-023_702455	0.8732
Listeria_monocytogenes_serotype_4b_str_02-6680_1196175	Listeria_innocua_FSL_J1-023_702455	0.8829
Listeria_monocytogenes_serotype_4b_str_02-6679_1196174	Listeria_innocua_FSL_J1-023_702455	0.8829
Listeria_monocytogenes_serotype_4b_str_02-1792_1196173	Listeria_innocua_FSL_J1-023_702455	0.8732
Listeria_monocytogenes_serotype_4b_str_02-1289_1196172	Listeria_innocua_FSL_J1-023_702455	0.8732
Listeria_monocytogenes_serotype_4b_str_02-1103_1196171	Listeria_innocua_FSL_J1-023_702455	0.8732
Listeria_monocytogenes_serotype_3c_str_10-5027_1196195	Listeria_innocua_FSL_J1-023_702455	0.8652
Listeria_monocytogenes_serotype_1_2c_str_10-5026_1196194	Listeria_innocua_FSL_J1-023_702455	0.8656
Listeria_monocytogenes_serotype_1_2c_str_10-5025_1196193	Listeria_innocua_FSL_J1-023_702455	0.8656
Listeria_monocytogenes_serotype_1_2b_str_10-0811_1196164	Listeria_innocua_FSL_J1-023_702455	0.8604
Listeria_monocytogenes_serotype_1_2b_str_10-0810_1196163	Listeria_innocua_FSL_J1-023_702455	0.8604
Listeria_monocytogenes_serotype_1_2a_str_99-6370_1196179	Listeria_innocua_FSL_J1-023_702455	0.8521
Listeria_monocytogenes_serotype_1_2a_str_98-2035_1196178	Listeria_innocua_FSL_J1-023_702455	0.8521
Listeria_monocytogenes_serotype_1_2a_str_95-0093_1196177	Listeria_innocua_FSL_J1-023_702455	0.8565
Listeria_monocytogenes_serotype_1_2a_str_88-0478_1196176	Listeria_innocua_FSL_J1-023_702455	0.8549
Listeria_monocytogenes_serotype_1_2a_str_10-5024_1196192	Listeria_innocua_FSL_J1-023_702455	0.8565
Listeria_monocytogenes_serotype_1_2a_str_10-4758_1196168	Listeria_innocua_FSL_J1-023_702455	0.8745
Listeria_monocytogenes_serotype_1_2a_str_10-4754_1196167	Listeria_innocua_FSL_J1-023_702455	0.8748
Listeria_monocytogenes_serotype_1_2a_str_10-1321_1196191	Listeria_innocua_FSL_J1-023_702455	0.8565
Listeria_monocytogenes_serotype_1_2a_str_10-1047_1196190	Listeria_innocua_FSL_J1-023_702455	0.8572
Listeria_monocytogenes_serotype_1_2a_str_10-1046_1196189	Listeria_innocua_FSL_J1-023_702455	0.8572
Listeria_monocytogenes_serotype_1_2a_str_10-0934_1196170	Listeria_innocua_FSL_J1-023_702455	0.8842
Listeria_monocytogenes_serotype_1_2a_str_10-0933_1196169	Listeria_innocua_FSL_J1-023_702455	0.8842
Listeria_monocytogenes_serotype_1_2a_str_10-0815_1196188	Listeria_innocua_FSL_J1-023_702455	0.853
Listeria_monocytogenes_serotype_1_2a_str_10-0814_1196187	Listeria_innocua_FSL_J1-023_702455	0.8569
Listeria_monocytogenes_serotype_1_2a_str_10-0813_1196166	Listeria_innocua_FSL_J1-023_702455	0.8671
Listeria_monocytogenes_serotype_1_2a_str_10-0812_1196165	Listeria_innocua_FSL_J1-023_702455	0.8671
Listeria_monocytogenes_serotype_1_2a_str_08-7669_1196186	Listeria_innocua_FSL_J1-023_702455	0.8639
Listeria_monocytogenes_serotype_1_2a_str_08-7374_1196185	Listeria_innocua_FSL_J1-023_702455	0.8565
Listeria_monocytogenes_serotype_1_2a_str_08-6997_1196184	Listeria_innocua_FSL_J1-023_702455	0.853
Listeria_monocytogenes_serotype_1_2a_str_08-6569_1196183	Listeria_innocua_FSL_J1-023_702455	0.853
Listeria_monocytogenes_serotype_1_2a_str_08-6056_1196182	Listeria_innocua_FSL_J1-023_702455	0.853
Listeria_monocytogenes_serotype_1_2a_str_04-5457_1196181	Listeria_innocua_FSL_J1-023_702455	0.8565
Listeria_monocytogenes_serotype_1_2a_str_02-5993_1196180	Listeria_innocua_FSL_J1-023_702455	0.8565
Listeria_monocytogenes_R479a_1437838	Listeria_innocua_FSL_J1-023_702455	0.8555
Listeria_monocytogenes_N53-1_1234142	Listeria_innocua_FSL_J1-023_702455	0.8377
Listeria_monocytogenes_M7_1030009	Listeria_innocua_FSL_J1-023_702455	0.8729
Listeria_monocytogenes_LO28_393132	Listeria_innocua_FSL_J1-023_702455	0.8655
Listeria_monocytogenes_L99_563174	Listeria_innocua_FSL_J1-023_702455	0.8733
Listeria_monocytogenes_L312_882094	Listeria_innocua_FSL_J1-023_702455	0.8827
Listeria_monocytogenes_J1816_930781	Listeria_innocua_FSL_J1-023_702455	0.8667
Listeria_monocytogenes_J1-220_930782	Listeria_innocua_FSL_J1-023_702455	0.8649

Listeria_monocytogenes_J0161_393130	Listeria_innocua_FSL_J1-023_702455	0.8623
Listeria_monocytogenes_HCC23_552536	Listeria_innocua_FSL_J1-023_702455	0.8729
Listeria_monocytogenes_FSL_R2-561_393126	Listeria_innocua_FSL_J1-023_702455	0.8654
Listeria_monocytogenes_FSL_J1-208_393119	Listeria_innocua_FSL_J1-023_702455	0.8706
Listeria_monocytogenes_FSL_J1-175_393118	Listeria_innocua_FSL_J1-023_702455	0.8739
Listeria_monocytogenes_Finland_1998_393127	Listeria_innocua_FSL_J1-023_702455	0.881
Listeria_monocytogenes_EGD-e_169963_v1	Listeria_innocua_FSL_J1-023_702455	0.8734
Listeria_monocytogenes_EGD-e_169963	Listeria_innocua_FSL_J1-023_702455	0.8738
Listeria_monocytogenes_EGD_1334565	Listeria_innocua_FSL_J1-023_702455	0.8734
Listeria_monocytogenes_CFSAN002349_1299895	Listeria_innocua_FSL_J1-023_702455	0.8819
Listeria_monocytogenes_ATCC_19117_882095_v1	Listeria_innocua_FSL_J1-023_702455	0.8793
Listeria_monocytogenes_ATCC_19117_882095	Listeria_innocua_FSL_J1-023_702455	0.8793
Listeria_monocytogenes_6179_1288295	Listeria_innocua_FSL_J1-023_702455	0.8517
Listeria_monocytogenes_1639_v9	Listeria_innocua_FSL_J1-023_702455	0.846
Listeria_monocytogenes_1639_v99	Listeria_innocua_FSL_J1-023_702455	0.8679
Listeria_monocytogenes_1639_v98	Listeria_innocua_FSL_J1-023_702455	0.8595
Listeria_monocytogenes_1639_v97	Listeria_innocua_FSL_J1-023_702455	0.8719
Listeria_monocytogenes_1639_v96	Listeria_innocua_FSL_J1-023_702455	0.8673
Listeria_monocytogenes_1639_v95	Listeria_innocua_FSL_J1-023_702455	0.8673
Listeria_monocytogenes_1639_v94	Listeria_innocua_FSL_J1-023_702455	0.8676
Listeria_monocytogenes_1639_v93	Listeria_innocua_FSL_J1-023_702455	0.8618
Listeria_monocytogenes_1639_v92	Listeria_innocua_FSL_J1-023_702455	0.8684
Listeria_monocytogenes_1639_v91	Listeria_innocua_FSL_J1-023_702455	0.8732
Listeria_monocytogenes_1639_v90	Listeria_innocua_FSL_J1-023_702455	0.8595
Listeria_monocytogenes_1639_v8	Listeria_innocua_FSL_J1-023_702455	0.8477
Listeria_monocytogenes_1639_v89	Listeria_innocua_FSL_J1-023_702455	0.8764
Listeria_monocytogenes_1639_v88	Listeria_innocua_FSL_J1-023_702455	0.8684
Listeria_monocytogenes_1639_v87	Listeria_innocua_FSL_J1-023_702455	0.8616
Listeria_monocytogenes_1639_v86	Listeria_innocua_FSL_J1-023_702455	0.8616
Listeria_monocytogenes_1639_v85	Listeria_innocua_FSL_J1-023_702455	0.8715
Listeria_monocytogenes_1639_v84	Listeria_innocua_FSL_J1-023_702455	0.8673
Listeria_monocytogenes_1639_v83	Listeria_innocua_FSL_J1-023_702455	0.8702
Listeria_monocytogenes_1639_v82	Listeria_innocua_FSL_J1-023_702455	0.8699
Listeria_monocytogenes_1639_v81	Listeria_innocua_FSL_J1-023_702455	0.8649
Listeria_monocytogenes_1639_v80	Listeria_innocua_FSL_J1-023_702455	0.8653
Listeria_monocytogenes_1639_v7	Listeria_innocua_FSL_J1-023_702455	0.8477
Listeria_monocytogenes_1639_v79	Listeria_innocua_FSL_J1-023_702455	0.8624
Listeria_monocytogenes_1639_v78	Listeria_innocua_FSL_J1-023_702455	0.8642
Listeria_monocytogenes_1639_v77	Listeria_innocua_FSL_J1-023_702455	0.8656
Listeria_monocytogenes_1639_v76	Listeria_innocua_FSL_J1-023_702455	0.859
Listeria_monocytogenes_1639_v75	Listeria_innocua_FSL_J1-023_702455	0.8733
Listeria_monocytogenes_1639_v74	Listeria_innocua_FSL_J1-023_702455	0.8596
Listeria_monocytogenes_1639_v73	Listeria_innocua_FSL_J1-023_702455	0.8604
Listeria_monocytogenes_1639_v72	Listeria_innocua_FSL_J1-023_702455	0.8686
Listeria_monocytogenes_1639_v71	Listeria_innocua_FSL_J1-023_702455	0.8511
Listeria_monocytogenes_1639_v70	Listeria_innocua_FSL_J1-023_702455	0.8639
Listeria_monocytogenes_1639_v6	Listeria_innocua_FSL_J1-023_702455	0.8474
Listeria_monocytogenes_1639_v69	Listeria_innocua_FSL_J1-023_702455	0.8728
Listeria_monocytogenes_1639_v68	Listeria_innocua_FSL_J1-023_702455	0.8668
Listeria_monocytogenes_1639_v67	Listeria_innocua_FSL_J1-023_702455	0.8641
Listeria_monocytogenes_1639_v66	Listeria_innocua_FSL_J1-023_702455	0.8738
Listeria_monocytogenes_1639_v65	Listeria_innocua_FSL_J1-023_702455	0.8588
Listeria_monocytogenes_1639_v64	Listeria_innocua_FSL_J1-023_702455	0.8686
Listeria_monocytogenes_1639_v63	Listeria_innocua_FSL_J1-023_702455	0.8614
Listeria_monocytogenes_1639_v62	Listeria_innocua_FSL_J1-023_702455	0.8601
Listeria_monocytogenes_1639_v61	Listeria_innocua_FSL_J1-023_702455	0.8615
Listeria_monocytogenes_1639_v60	Listeria_innocua_FSL_J1-023_702455	0.8637
Listeria_monocytogenes_1639_v5	Listeria_innocua_FSL_J1-023_702455	0.8294
Listeria_monocytogenes_1639_v59	Listeria_innocua_FSL_J1-023_702455	0.8737
Listeria_monocytogenes_1639_v58	Listeria_innocua_FSL_J1-023_702455	0.871
Listeria_monocytogenes_1639_v57	Listeria_innocua_FSL_J1-023_702455	0.8644
Listeria_monocytogenes_1639_v56	Listeria_innocua_FSL_J1-023_702455	0.8706
Listeria_monocytogenes_1639_v55	Listeria_innocua_FSL_J1-023_702455	0.8618
Listeria_monocytogenes_1639_v54	Listeria_innocua_FSL_J1-023_702455	0.87
Listeria_monocytogenes_1639_v53	Listeria_innocua_FSL_J1-023_702455	0.8698
Listeria_monocytogenes_1639_v52	Listeria_innocua_FSL_J1-023_702455	0.871
Listeria_monocytogenes_1639_v51	Listeria_innocua_FSL_J1-023_702455	0.8648
Listeria_monocytogenes_1639_v50	Listeria_innocua_FSL_J1-023_702455	0.8558
Listeria_monocytogenes_1639_v4	Listeria_innocua_FSL_J1-023_702455	0.8414
Listeria_monocytogenes_1639_v49	Listeria_innocua_FSL_J1-023_702455	0.8853
Listeria_monocytogenes_1639_v48	Listeria_innocua_FSL_J1-023_702455	0.8607
Listeria_monocytogenes_1639_v47	Listeria_innocua_FSL_J1-023_702455	0.8562
Listeria_monocytogenes_1639_v46	Listeria_innocua_FSL_J1-023_702455	0.8657
Listeria_monocytogenes_1639_v45	Listeria_innocua_FSL_J1-023_702455	0.8481
Listeria_monocytogenes_1639_v44	Listeria_innocua_FSL_J1-023_702455	0.8565
Listeria_monocytogenes_1639_v43	Listeria_innocua_FSL_J1-023_702455	0.8565
Listeria_monocytogenes_1639_v42	Listeria_innocua_FSL_J1-023_702455	0.8581
Listeria_monocytogenes_1639_v41	Listeria_innocua_FSL_J1-023_702455	0.8432
Listeria_monocytogenes_1639_v40	Listeria_innocua_FSL_J1-023_702455	0.8658
Listeria_monocytogenes_1639_v3	Listeria_innocua_FSL_J1-023_702455	0.8418
Listeria_monocytogenes_1639_v39	Listeria_innocua_FSL_J1-023_702455	0.8609
Listeria_monocytogenes_1639_v38	Listeria_innocua_FSL_J1-023_702455	0.8647
Listeria_monocytogenes_1639_v37	Listeria_innocua_FSL_J1-023_702455	0.8585
Listeria_monocytogenes_1639_v36	Listeria_innocua_FSL_J1-023_702455	0.856
Listeria_monocytogenes_1639_v35	Listeria_innocua_FSL_J1-023_702455	0.8672
Listeria_monocytogenes_1639_v34	Listeria_innocua_FSL_J1-023_702455	0.8598
Listeria_monocytogenes_1639_v33	Listeria_innocua_FSL_J1-023_702455	0.8604
Listeria_monocytogenes_1639_v32	Listeria_innocua_FSL_J1-023_702455	0.8583
Listeria_monocytogenes_1639_v31	Listeria_innocua_FSL_J1-023_702455	0.8602
Listeria_monocytogenes_1639_v30	Listeria_innocua_FSL_J1-023_702455	0.8579
Listeria_monocytogenes_1639_v2	Listeria_innocua_FSL_J1-023_702455	0.8271

Listeria_monocytogenes_1639_v29	Listeria_innocua_FSL_J1-023_702455	0.8544
Listeria_monocytogenes_1639_v28	Listeria_innocua_FSL_J1-023_702455	0.8586
Listeria_monocytogenes_1639_v27	Listeria_innocua_FSL_J1-023_702455	0.8502
Listeria_monocytogenes_1639_v26	Listeria_innocua_FSL_J1-023_702455	0.85
Listeria_monocytogenes_1639_v25	Listeria_innocua_FSL_J1-023_702455	0.85
Listeria_monocytogenes_1639_v24	Listeria_innocua_FSL_J1-023_702455	0.8653
Listeria_monocytogenes_1639_v23	Listeria_innocua_FSL_J1-023_702455	0.865
Listeria_monocytogenes_1639_v22	Listeria_innocua_FSL_J1-023_702455	0.8618
Listeria_monocytogenes_1639_v21	Listeria_innocua_FSL_J1-023_702455	0.865
Listeria_monocytogenes_1639_v20	Listeria_innocua_FSL_J1-023_702455	0.8532
Listeria_monocytogenes_1639_v1	Listeria_innocua_FSL_J1-023_702455	0.8477
Listeria_monocytogenes_1639_v19	Listeria_innocua_FSL_J1-023_702455	0.8529
Listeria_monocytogenes_1639_v198	Listeria_innocua_FSL_J1-023_702455	0.8397
Listeria_monocytogenes_1639_v197	Listeria_innocua_FSL_J1-023_702455	0.8615
Listeria_monocytogenes_1639_v196	Listeria_innocua_FSL_J1-023_702455	0.8585
Listeria_monocytogenes_1639_v195	Listeria_innocua_FSL_J1-023_702455	0.8901
Listeria_monocytogenes_1639_v194	Listeria_innocua_FSL_J1-023_702455	0.8891
Listeria_monocytogenes_1639_v193	Listeria_innocua_FSL_J1-023_702455	0.8887
Listeria_monocytogenes_1639_v192	Listeria_innocua_FSL_J1-023_702455	0.8608
Listeria_monocytogenes_1639_v191	Listeria_innocua_FSL_J1-023_702455	0.8851
Listeria_monocytogenes_1639_v190	Listeria_innocua_FSL_J1-023_702455	0.8829
Listeria_monocytogenes_1639_v18	Listeria_innocua_FSL_J1-023_702455	0.8591
Listeria_monocytogenes_1639_v189	Listeria_innocua_FSL_J1-023_702455	0.8817
Listeria_monocytogenes_1639_v188	Listeria_innocua_FSL_J1-023_702455	0.8814
Listeria_monocytogenes_1639_v187	Listeria_innocua_FSL_J1-023_702455	0.8828
Listeria_monocytogenes_1639_v186	Listeria_innocua_FSL_J1-023_702455	0.8828
Listeria_monocytogenes_1639_v185	Listeria_innocua_FSL_J1-023_702455	0.8832
Listeria_monocytogenes_1639_v184	Listeria_innocua_FSL_J1-023_702455	0.882
Listeria_monocytogenes_1639_v183	Listeria_innocua_FSL_J1-023_702455	0.8836
Listeria_monocytogenes_1639_v182	Listeria_innocua_FSL_J1-023_702455	0.885
Listeria_monocytogenes_1639_v181	Listeria_innocua_FSL_J1-023_702455	0.879
Listeria_monocytogenes_1639_v180	Listeria_innocua_FSL_J1-023_702455	0.879
Listeria_monocytogenes_1639_v17	Listeria_innocua_FSL_J1-023_702455	0.8594
Listeria_monocytogenes_1639_v179	Listeria_innocua_FSL_J1-023_702455	0.8746
Listeria_monocytogenes_1639_v178	Listeria_innocua_FSL_J1-023_702455	0.8845
Listeria_monocytogenes_1639_v177	Listeria_innocua_FSL_J1-023_702455	0.8842
Listeria_monocytogenes_1639_v176	Listeria_innocua_FSL_J1-023_702455	0.8884
Listeria_monocytogenes_1639_v175	Listeria_innocua_FSL_J1-023_702455	0.8777
Listeria_monocytogenes_1639_v174	Listeria_innocua_FSL_J1-023_702455	0.8815
Listeria_monocytogenes_1639_v173	Listeria_innocua_FSL_J1-023_702455	0.8827
Listeria_monocytogenes_1639_v172	Listeria_innocua_FSL_J1-023_702455	0.8868
Listeria_monocytogenes_1639_v171	Listeria_innocua_FSL_J1-023_702455	0.8695
Listeria_monocytogenes_1639_v170	Listeria_innocua_FSL_J1-023_702455	0.877
Listeria_monocytogenes_1639_v16	Listeria_innocua_FSL_J1-023_702455	0.8443
Listeria_monocytogenes_1639_v169	Listeria_innocua_FSL_J1-023_702455	0.8771
Listeria_monocytogenes_1639_v168	Listeria_innocua_FSL_J1-023_702455	0.8828
Listeria_monocytogenes_1639_v167	Listeria_innocua_FSL_J1-023_702455	0.8782
Listeria_monocytogenes_1639_v166	Listeria_innocua_FSL_J1-023_702455	0.8831
Listeria_monocytogenes_1639_v165	Listeria_innocua_FSL_J1-023_702455	0.8768
Listeria_monocytogenes_1639_v164	Listeria_innocua_FSL_J1-023_702455	0.8878
Listeria_monocytogenes_1639_v163	Listeria_innocua_FSL_J1-023_702455	0.8745
Listeria_monocytogenes_1639_v162	Listeria_innocua_FSL_J1-023_702455	0.8878
Listeria_monocytogenes_1639_v161	Listeria_innocua_FSL_J1-023_702455	0.8862
Listeria_monocytogenes_1639_v160	Listeria_innocua_FSL_J1-023_702455	0.8863
Listeria_monocytogenes_1639_v15	Listeria_innocua_FSL_J1-023_702455	0.848
Listeria_monocytogenes_1639_v159	Listeria_innocua_FSL_J1-023_702455	0.8842
Listeria_monocytogenes_1639_v158	Listeria_innocua_FSL_J1-023_702455	0.8864
Listeria_monocytogenes_1639_v157	Listeria_innocua_FSL_J1-023_702455	0.8864
Listeria_monocytogenes_1639_v156	Listeria_innocua_FSL_J1-023_702455	0.8616
Listeria_monocytogenes_1639_v155	Listeria_innocua_FSL_J1-023_702455	0.8704
Listeria_monocytogenes_1639_v154	Listeria_innocua_FSL_J1-023_702455	0.8728
Listeria_monocytogenes_1639_v153	Listeria_innocua_FSL_J1-023_702455	0.8711
Listeria_monocytogenes_1639_v152	Listeria_innocua_FSL_J1-023_702455	0.8842
Listeria_monocytogenes_1639_v151	Listeria_innocua_FSL_J1-023_702455	0.8842
Listeria_monocytogenes_1639_v150	Listeria_innocua_FSL_J1-023_702455	0.8747
Listeria_monocytogenes_1639_v14	Listeria_innocua_FSL_J1-023_702455	0.8548
Listeria_monocytogenes_1639_v149	Listeria_innocua_FSL_J1-023_702455	0.8738
Listeria_monocytogenes_1639_v148	Listeria_innocua_FSL_J1-023_702455	0.8831
Listeria_monocytogenes_1639_v147	Listeria_innocua_FSL_J1-023_702455	0.8709
Listeria_monocytogenes_1639_v146	Listeria_innocua_FSL_J1-023_702455	0.8824
Listeria_monocytogenes_1639_v145	Listeria_innocua_FSL_J1-023_702455	0.8827
Listeria_monocytogenes_1639_v144	Listeria_innocua_FSL_J1-023_702455	0.8823
Listeria_monocytogenes_1639_v143	Listeria_innocua_FSL_J1-023_702455	0.8842
Listeria_monocytogenes_1639_v142	Listeria_innocua_FSL_J1-023_702455	0.8827
Listeria_monocytogenes_1639_v141	Listeria_innocua_FSL_J1-023_702455	0.8786
Listeria_monocytogenes_1639_v140	Listeria_innocua_FSL_J1-023_702455	0.883
Listeria_monocytogenes_1639_v13	Listeria_innocua_FSL_J1-023_702455	0.8483
Listeria_monocytogenes_1639_v139	Listeria_innocua_FSL_J1-023_702455	0.8797
Listeria_monocytogenes_1639_v138	Listeria_innocua_FSL_J1-023_702455	0.8843
Listeria_monocytogenes_1639_v137	Listeria_innocua_FSL_J1-023_702455	0.8835
Listeria_monocytogenes_1639_v136	Listeria_innocua_FSL_J1-023_702455	0.8741
Listeria_monocytogenes_1639_v135	Listeria_innocua_FSL_J1-023_702455	0.8715
Listeria_monocytogenes_1639_v134	Listeria_innocua_FSL_J1-023_702455	0.8809
Listeria_monocytogenes_1639_v133	Listeria_innocua_FSL_J1-023_702455	0.8748
Listeria_monocytogenes_1639_v132	Listeria_innocua_FSL_J1-023_702455	0.8617
Listeria_monocytogenes_1639_v131	Listeria_innocua_FSL_J1-023_702455	0.874
Listeria_monocytogenes_1639_v130	Listeria_innocua_FSL_J1-023_702455	0.8665
Listeria_monocytogenes_1639_v12	Listeria_innocua_FSL_J1-023_702455	0.8502
Listeria_monocytogenes_1639_v129	Listeria_innocua_FSL_J1-023_702455	0.8795
Listeria_monocytogenes_1639_v128	Listeria_innocua_FSL_J1-023_702455	0.8748
Listeria_monocytogenes_1639_v127	Listeria_innocua_FSL_J1-023_702455	0.8715

Listeria_monocytogenes_1639_v126	Listeria_innocua_FSL_J1-023_702455	0.8748
Listeria_monocytogenes_1639_v125	Listeria_innocua_FSL_J1-023_702455	0.8756
Listeria_monocytogenes_1639_v124	Listeria_innocua_FSL_J1-023_702455	0.8722
Listeria_monocytogenes_1639_v123	Listeria_innocua_FSL_J1-023_702455	0.8819
Listeria_monocytogenes_1639_v122	Listeria_innocua_FSL_J1-023_702455	0.8819
Listeria_monocytogenes_1639_v121	Listeria_innocua_FSL_J1-023_702455	0.8819
Listeria_monocytogenes_1639_v120	Listeria_innocua_FSL_J1-023_702455	0.8823
Listeria_monocytogenes_1639_v11	Listeria_innocua_FSL_J1-023_702455	0.8491
Listeria_monocytogenes_1639_v119	Listeria_innocua_FSL_J1-023_702455	0.8819
Listeria_monocytogenes_1639_v118	Listeria_innocua_FSL_J1-023_702455	0.8819
Listeria_monocytogenes_1639_v117	Listeria_innocua_FSL_J1-023_702455	0.8737
Listeria_monocytogenes_1639_v116	Listeria_innocua_FSL_J1-023_702455	0.8813
Listeria_monocytogenes_1639_v115	Listeria_innocua_FSL_J1-023_702455	0.8812
Listeria_monocytogenes_1639_v114	Listeria_innocua_FSL_J1-023_702455	0.8681
Listeria_monocytogenes_1639_v113	Listeria_innocua_FSL_J1-023_702455	0.8749
Listeria_monocytogenes_1639_v112	Listeria_innocua_FSL_J1-023_702455	0.8778
Listeria_monocytogenes_1639_v111	Listeria_innocua_FSL_J1-023_702455	0.8662
Listeria_monocytogenes_1639_v110	Listeria_innocua_FSL_J1-023_702455	0.8666
Listeria_monocytogenes_1639_v10	Listeria_innocua_FSL_J1-023_702455	0.8464
Listeria_monocytogenes_1639_v109	Listeria_innocua_FSL_J1-023_702455	0.8666
Listeria_monocytogenes_1639_v108	Listeria_innocua_FSL_J1-023_702455	0.8754
Listeria_monocytogenes_1639_v107	Listeria_innocua_FSL_J1-023_702455	0.8783
Listeria_monocytogenes_1639_v106	Listeria_innocua_FSL_J1-023_702455	0.8783
Listeria_monocytogenes_1639_v105	Listeria_innocua_FSL_J1-023_702455	0.8786
Listeria_monocytogenes_1639_v104	Listeria_innocua_FSL_J1-023_702455	0.8783
Listeria_monocytogenes_1639_v103	Listeria_innocua_FSL_J1-023_702455	0.8786
Listeria_monocytogenes_1639_v102	Listeria_innocua_FSL_J1-023_702455	0.8653
Listeria_monocytogenes_1639_v101	Listeria_innocua_FSL_J1-023_702455	0.8548
Listeria_monocytogenes_1639_v100	Listeria_innocua_FSL_J1-023_702455	0.8545
Listeria_monocytogenes_1639	Listeria_innocua_FSL_J1-023_702455	0.8825
Listeria_monocytogenes_10403S_393133	Listeria_innocua_FSL_J1-023_702455	0.8751
Listeria_monocytogenes_08-5923_637381	Listeria_innocua_FSL_J1-023_702455	0.8565
Listeria_monocytogenes_08-5578_653938	Listeria_innocua_FSL_J1-023_702455	0.8443
Listeria_monocytogenes_07PF0776_1126011	Listeria_innocua_FSL_J1-023_702455	0.8832
Listeria_marthii_FSL_S4-120_702457	Listeria_innocua_FSL_J1-023_702455	0.8349
Listeria_marthii_529731_v1	Listeria_innocua_FSL_J1-023_702455	0.8512
Listeria_marthii_529731	Listeria_innocua_FSL_J1-023_702455	0.8588
Listeria_ivanovii_WSLC3009_1457190	Listeria_innocua_FSL_J1-023_702455	0.8447
Listeria_ivanovii_subsp_londoniensis_202752_v2	Listeria_innocua_FSL_J1-023_702455	0.8418
Listeria_ivanovii_subsp_londoniensis_202752_v1	Listeria_innocua_FSL_J1-023_702455	0.8423
Listeria_ivanovii_subsp_londoniensis_202752	Listeria_innocua_FSL_J1-023_702455	0.8366
Listeria_ivanovii_subsp_ivanovii_PAM_55_881621	Listeria_innocua_FSL_J1-023_702455	0.8438
Listeria_ivanovii_subsp_ivanovii_202751_v2	Listeria_innocua_FSL_J1-023_702455	0.845
Listeria_ivanovii_subsp_ivanovii_202751_v1	Listeria_innocua_FSL_J1-023_702455	0.845
Listeria_ivanovii_subsp_ivanovii_202751	Listeria_innocua_FSL_J1-023_702455	0.845
Listeria_ivanovii_FSL_F6-596_702454	Listeria_innocua_FSL_J1-023_702455	0.7859
Listeria_ivanovii_1638	Listeria_innocua_FSL_J1-023_702455	0.8458
Listeria_innocua_FSL_S4-378_702456	Listeria_innocua_FSL_J1-023_702455	0.8228
Listeria_innocua_FSL_J1-023_702455	Listeria_innocua_FSL_J1-023_702455	0.0577
Listeria_welshimeri_1643	Listeria_innocua_FSL_S4-378_702456	0.7969
Listeria_welshimeri_serovar_6b_str_SLCC5334_386043	Listeria_innocua_FSL_S4-378_702456	0.7963
Listeria_seeligeri_serovar_1_2b_str_SLCC3954_683837	Listeria_innocua_FSL_S4-378_702456	0.7919
Listeria_seeligeri_FSL_S4-171_702452	Listeria_innocua_FSL_S4-378_702456	0.757
Listeria_seeligeri_FSL_N1-067_702453	Listeria_innocua_FSL_S4-378_702456	0.7494
Listeria_seeligeri_1640_v1	Listeria_innocua_FSL_S4-378_702456	0.7967
Listeria_seeligeri_1640	Listeria_innocua_FSL_S4-378_702456	0.7913
Listeria_monocytogenes_WSLC1042_1457188	Listeria_innocua_FSL_S4-378_702456	0.8268
Listeria_monocytogenes_WSLC1001_1457187	Listeria_innocua_FSL_S4-378_702456	0.824
Listeria_monocytogenes_SLCC7179_879090	Listeria_innocua_FSL_S4-378_702456	0.8294
Listeria_monocytogenes_SLCC5850_882096	Listeria_innocua_FSL_S4-378_702456	0.8274
Listeria_monocytogenes_SLCC2755_932919	Listeria_innocua_FSL_S4-378_702456	0.8304
Listeria_monocytogenes_SLCC2540_879089	Listeria_innocua_FSL_S4-378_702456	0.8208
Listeria_monocytogenes_SLCC2479_882020	Listeria_innocua_FSL_S4-378_702456	0.8137
Listeria_monocytogenes_SLCC2378_879088	Listeria_innocua_FSL_S4-378_702456	0.8281
Listeria_monocytogenes_SLCC2376_882097	Listeria_innocua_FSL_S4-378_702456	0.8368
Listeria_monocytogenes_SLCC2372_932920	Listeria_innocua_FSL_S4-378_702456	0.8072
Listeria_monocytogenes_serotype_4b_str_LL195_1230340	Listeria_innocua_FSL_S4-378_702456	0.8332
Listeria_monocytogenes_serotype_4b_str_LIS0087_1134669	Listeria_innocua_FSL_S4-378_702456	0.8075
Listeria_monocytogenes_serotype_4b_str_F2365_265669	Listeria_innocua_FSL_S4-378_702456	0.8332
Listeria_monocytogenes_serotype_4b_str_CLIP_80459_568819	Listeria_innocua_FSL_S4-378_702456	0.8389
Listeria_monocytogenes_serotype_4b_str_81-0861_1196161	Listeria_innocua_FSL_S4-378_702456	0.8235
Listeria_monocytogenes_serotype_4b_str_81-0592_1196160	Listeria_innocua_FSL_S4-378_702456	0.8245
Listeria_monocytogenes_serotype_4b_str_81-0558_1196159	Listeria_innocua_FSL_S4-378_702456	0.8252
Listeria_monocytogenes_serotype_4b_str_10-0809_1196162	Listeria_innocua_FSL_S4-378_702456	0.8249
Listeria_monocytogenes_serotype_4b_str_02-6680_1196175	Listeria_innocua_FSL_S4-378_702456	0.835
Listeria_monocytogenes_serotype_4b_str_02-6679_1196174	Listeria_innocua_FSL_S4-378_702456	0.835
Listeria_monocytogenes_serotype_4b_str_02-1792_1196173	Listeria_innocua_FSL_S4-378_702456	0.8249
Listeria_monocytogenes_serotype_4b_str_02-1289_1196172	Listeria_innocua_FSL_S4-378_702456	0.8249
Listeria_monocytogenes_serotype_4b_str_02-1103_1196171	Listeria_innocua_FSL_S4-378_702456	0.8249
Listeria_monocytogenes_serotype_3c_str_10-5027_1196195	Listeria_innocua_FSL_S4-378_702456	0.8121
Listeria_monocytogenes_serotype_1_2c_str_10-5026_1196194	Listeria_innocua_FSL_S4-378_702456	0.8133
Listeria_monocytogenes_serotype_1_2c_str_10-5025_1196193	Listeria_innocua_FSL_S4-378_702456	0.8133
Listeria_monocytogenes_serotype_1_2b_str_10-0811_1196164	Listeria_innocua_FSL_S4-378_702456	0.8168
Listeria_monocytogenes_serotype_1_2b_str_10-0810_1196163	Listeria_innocua_FSL_S4-378_702456	0.8168
Listeria_monocytogenes_serotype_1_2a_str_99-6370_1196179	Listeria_innocua_FSL_S4-378_702456	0.806
Listeria_monocytogenes_serotype_1_2a_str_98-2035_1196178	Listeria_innocua_FSL_S4-378_702456	0.806
Listeria_monocytogenes_serotype_1_2a_str_95-0093_1196177	Listeria_innocua_FSL_S4-378_702456	0.8105
Listeria_monocytogenes_serotype_1_2a_str_88-0478_1196176	Listeria_innocua_FSL_S4-378_702456	0.8095
Listeria_monocytogenes_serotype_1_2a_str_10-5024_1196192	Listeria_innocua_FSL_S4-378_702456	0.8108
Listeria_monocytogenes_serotype_1_2a_str_10-4758_1196168	Listeria_innocua_FSL_S4-378_702456	0.8227
Listeria_monocytogenes_serotype_1_2a_str_10-4754_1196167	Listeria_innocua_FSL_S4-378_702456	0.8227

Listeria_monocytogenes_serotype_1_2a_str_10-1321_1196191	Listeria_innocua_FSL_S4-378_702456	0.8108
Listeria_monocytogenes_serotype_1_2a_str_10-1047_1196190	Listeria_innocua_FSL_S4-378_702456	0.8111
Listeria_monocytogenes_serotype_1_2a_str_10-1046_1196189	Listeria_innocua_FSL_S4-378_702456	0.8111
Listeria_monocytogenes_serotype_1_2a_str_10-0934_1196170	Listeria_innocua_FSL_S4-378_702456	0.8344
Listeria_monocytogenes_serotype_1_2a_str_10-0933_1196169	Listeria_innocua_FSL_S4-378_702456	0.8344
Listeria_monocytogenes_serotype_1_2a_str_10-0815_1196188	Listeria_innocua_FSL_S4-378_702456	0.8072
Listeria_monocytogenes_serotype_1_2a_str_10-0814_1196187	Listeria_innocua_FSL_S4-378_702456	0.8108
Listeria_monocytogenes_serotype_1_2a_str_10-0813_1196166	Listeria_innocua_FSL_S4-378_702456	0.825
Listeria_monocytogenes_serotype_1_2a_str_10-0812_1196165	Listeria_innocua_FSL_S4-378_702456	0.825
Listeria_monocytogenes_serotype_1_2a_str_08-7669_1196186	Listeria_innocua_FSL_S4-378_702456	0.8169
Listeria_monocytogenes_serotype_1_2a_str_08-7374_1196185	Listeria_innocua_FSL_S4-378_702456	0.8108
Listeria_monocytogenes_serotype_1_2a_str_08-6997_1196184	Listeria_innocua_FSL_S4-378_702456	0.8072
Listeria_monocytogenes_serotype_1_2a_str_08-6569_1196183	Listeria_innocua_FSL_S4-378_702456	0.8072
Listeria_monocytogenes_serotype_1_2a_str_08-6056_1196182	Listeria_innocua_FSL_S4-378_702456	0.8072
Listeria_monocytogenes_serotype_1_2a_str_04-5457_1196181	Listeria_innocua_FSL_S4-378_702456	0.8108
Listeria_monocytogenes_serotype_1_2a_str_02-5993_1196180	Listeria_innocua_FSL_S4-378_702456	0.8105
Listeria_monocytogenes_R479a_1437838	Listeria_innocua_FSL_S4-378_702456	0.8083
Listeria_monocytogenes_N53-1_1234142	Listeria_innocua_FSL_S4-378_702456	0.7874
Listeria_monocytogenes_M7_1030009	Listeria_innocua_FSL_S4-378_702456	0.8126
Listeria_monocytogenes_LO28_393132	Listeria_innocua_FSL_S4-378_702456	0.816
Listeria_monocytogenes_L99_563174	Listeria_innocua_FSL_S4-378_702456	0.8133
Listeria_monocytogenes_L312_882094	Listeria_innocua_FSL_S4-378_702456	0.8389
Listeria_monocytogenes_J1816_930781	Listeria_innocua_FSL_S4-378_702456	0.8172
Listeria_monocytogenes_J1-220_930782	Listeria_innocua_FSL_S4-378_702456	0.8191
Listeria_monocytogenes_J0161_393130	Listeria_innocua_FSL_S4-378_702456	0.817
Listeria_monocytogenes_HCC23_552536	Listeria_innocua_FSL_S4-378_702456	0.813
Listeria_monocytogenes_FSL_R2-561_393126	Listeria_innocua_FSL_S4-378_702456	0.8128
Listeria_monocytogenes_FSL_J1-208_393119	Listeria_innocua_FSL_S4-378_702456	0.8266
Listeria_monocytogenes_FSL_J1-175_393118	Listeria_innocua_FSL_S4-378_702456	0.8232
Listeria_monocytogenes_Finland_1998_393127	Listeria_innocua_FSL_S4-378_702456	0.8293
Listeria_monocytogenes_EGD-e_169963_v1	Listeria_innocua_FSL_S4-378_702456	0.8214
Listeria_monocytogenes_EGD-e_169963	Listeria_innocua_FSL_S4-378_702456	0.8218
Listeria_monocytogenes_EGD_1334565	Listeria_innocua_FSL_S4-378_702456	0.8281
Listeria_monocytogenes_CFSAN002349_1299895	Listeria_innocua_FSL_S4-378_702456	0.8328
Listeria_monocytogenes_ATCC_19117_882095_v1	Listeria_innocua_FSL_S4-378_702456	0.8292
Listeria_monocytogenes_ATCC_19117_882095	Listeria_innocua_FSL_S4-378_702456	0.8295
Listeria_monocytogenes_6179_1288295	Listeria_innocua_FSL_S4-378_702456	0.8087
Listeria_monocytogenes_1639_v9	Listeria_innocua_FSL_S4-378_702456	0.8036
Listeria_monocytogenes_1639_v99	Listeria_innocua_FSL_S4-378_702456	0.8176
Listeria_monocytogenes_1639_v98	Listeria_innocua_FSL_S4-378_702456	0.8195
Listeria_monocytogenes_1639_v97	Listeria_innocua_FSL_S4-378_702456	0.8235
Listeria_monocytogenes_1639_v96	Listeria_innocua_FSL_S4-378_702456	0.8166
Listeria_monocytogenes_1639_v95	Listeria_innocua_FSL_S4-378_702456	0.8167
Listeria_monocytogenes_1639_v94	Listeria_innocua_FSL_S4-378_702456	0.8166
Listeria_monocytogenes_1639_v93	Listeria_innocua_FSL_S4-378_702456	0.8076
Listeria_monocytogenes_1639_v92	Listeria_innocua_FSL_S4-378_702456	0.8188
Listeria_monocytogenes_1639_v91	Listeria_innocua_FSL_S4-378_702456	0.8243
Listeria_monocytogenes_1639_v90	Listeria_innocua_FSL_S4-378_702456	0.8195
Listeria_monocytogenes_1639_v8	Listeria_innocua_FSL_S4-378_702456	0.8093
Listeria_monocytogenes_1639_v89	Listeria_innocua_FSL_S4-378_702456	0.8305
Listeria_monocytogenes_1639_v88	Listeria_innocua_FSL_S4-378_702456	0.8217
Listeria_monocytogenes_1639_v87	Listeria_innocua_FSL_S4-378_702456	0.8216
Listeria_monocytogenes_1639_v86	Listeria_innocua_FSL_S4-378_702456	0.8217
Listeria_monocytogenes_1639_v85	Listeria_innocua_FSL_S4-378_702456	0.823
Listeria_monocytogenes_1639_v84	Listeria_innocua_FSL_S4-378_702456	0.8189
Listeria_monocytogenes_1639_v83	Listeria_innocua_FSL_S4-378_702456	0.8274
Listeria_monocytogenes_1639_v82	Listeria_innocua_FSL_S4-378_702456	0.8267
Listeria_monocytogenes_1639_v81	Listeria_innocua_FSL_S4-378_702456	0.8151
Listeria_monocytogenes_1639_v80	Listeria_innocua_FSL_S4-378_702456	0.8216
Listeria_monocytogenes_1639_v7	Listeria_innocua_FSL_S4-378_702456	0.8093
Listeria_monocytogenes_1639_v79	Listeria_innocua_FSL_S4-378_702456	0.8132
Listeria_monocytogenes_1639_v78	Listeria_innocua_FSL_S4-378_702456	0.8156
Listeria_monocytogenes_1639_v77	Listeria_innocua_FSL_S4-378_702456	0.8188
Listeria_monocytogenes_1639_v76	Listeria_innocua_FSL_S4-378_702456	0.8117
Listeria_monocytogenes_1639_v75	Listeria_innocua_FSL_S4-378_702456	0.8133
Listeria_monocytogenes_1639_v74	Listeria_innocua_FSL_S4-378_702456	0.8171
Listeria_monocytogenes_1639_v73	Listeria_innocua_FSL_S4-378_702456	0.8112
Listeria_monocytogenes_1639_v72	Listeria_innocua_FSL_S4-378_702456	0.8241
Listeria_monocytogenes_1639_v71	Listeria_innocua_FSL_S4-378_702456	0.8056
Listeria_monocytogenes_1639_v70	Listeria_innocua_FSL_S4-378_702456	0.8106
Listeria_monocytogenes_1639_v6	Listeria_innocua_FSL_S4-378_702456	0.8087
Listeria_monocytogenes_1639_v69	Listeria_innocua_FSL_S4-378_702456	0.8249
Listeria_monocytogenes_1639_v68	Listeria_innocua_FSL_S4-378_702456	0.8181
Listeria_monocytogenes_1639_v67	Listeria_innocua_FSL_S4-378_702456	0.817
Listeria_monocytogenes_1639_v66	Listeria_innocua_FSL_S4-378_702456	0.8213
Listeria_monocytogenes_1639_v65	Listeria_innocua_FSL_S4-378_702456	0.8155
Listeria_monocytogenes_1639_v64	Listeria_innocua_FSL_S4-378_702456	0.8162
Listeria_monocytogenes_1639_v63	Listeria_innocua_FSL_S4-378_702456	0.8147
Listeria_monocytogenes_1639_v62	Listeria_innocua_FSL_S4-378_702456	0.8113
Listeria_monocytogenes_1639_v61	Listeria_innocua_FSL_S4-378_702456	0.8155
Listeria_monocytogenes_1639_v60	Listeria_innocua_FSL_S4-378_702456	0.8139
Listeria_monocytogenes_1639_v5	Listeria_innocua_FSL_S4-378_702456	0.7976
Listeria_monocytogenes_1639_v59	Listeria_innocua_FSL_S4-378_702456	0.8222
Listeria_monocytogenes_1639_v58	Listeria_innocua_FSL_S4-378_702456	0.8242
Listeria_monocytogenes_1639_v57	Listeria_innocua_FSL_S4-378_702456	0.8147
Listeria_monocytogenes_1639_v56	Listeria_innocua_FSL_S4-378_702456	0.8227
Listeria_monocytogenes_1639_v55	Listeria_innocua_FSL_S4-378_702456	0.8169
Listeria_monocytogenes_1639_v54	Listeria_innocua_FSL_S4-378_702456	0.8207
Listeria_monocytogenes_1639_v53	Listeria_innocua_FSL_S4-378_702456	0.8162
Listeria_monocytogenes_1639_v52	Listeria_innocua_FSL_S4-378_702456	0.8237
Listeria_monocytogenes_1639_v51	Listeria_innocua_FSL_S4-378_702456	0.8202

Listeria_monocytogenes_1639_v50	Listeria_innocua_FSL_S4-378_702456	0.8096
Listeria_monocytogenes_1639_v4	Listeria_innocua_FSL_S4-378_702456	0.8088
Listeria_monocytogenes_1639_v49	Listeria_innocua_FSL_S4-378_702456	0.8235
Listeria_monocytogenes_1639_v48	Listeria_innocua_FSL_S4-378_702456	0.8095
Listeria_monocytogenes_1639_v47	Listeria_innocua_FSL_S4-378_702456	0.8105
Listeria_monocytogenes_1639_v46	Listeria_innocua_FSL_S4-378_702456	0.8179
Listeria_monocytogenes_1639_v45	Listeria_innocua_FSL_S4-378_702456	0.8023
Listeria_monocytogenes_1639_v44	Listeria_innocua_FSL_S4-378_702456	0.8108
Listeria_monocytogenes_1639_v43	Listeria_innocua_FSL_S4-378_702456	0.8108
Listeria_monocytogenes_1639_v42	Listeria_innocua_FSL_S4-378_702456	0.8047
Listeria_monocytogenes_1639_v41	Listeria_innocua_FSL_S4-378_702456	0.7999
Listeria_monocytogenes_1639_v40	Listeria_innocua_FSL_S4-378_702456	0.8124
Listeria_monocytogenes_1639_v3	Listeria_innocua_FSL_S4-378_702456	0.8092
Listeria_monocytogenes_1639_v39	Listeria_innocua_FSL_S4-378_702456	0.8138
Listeria_monocytogenes_1639_v38	Listeria_innocua_FSL_S4-378_702456	0.821
Listeria_monocytogenes_1639_v37	Listeria_innocua_FSL_S4-378_702456	0.8163
Listeria_monocytogenes_1639_v36	Listeria_innocua_FSL_S4-378_702456	0.817
Listeria_monocytogenes_1639_v35	Listeria_innocua_FSL_S4-378_702456	0.813
Listeria_monocytogenes_1639_v34	Listeria_innocua_FSL_S4-378_702456	0.8105
Listeria_monocytogenes_1639_v33	Listeria_innocua_FSL_S4-378_702456	0.8168
Listeria_monocytogenes_1639_v32	Listeria_innocua_FSL_S4-378_702456	0.8169
Listeria_monocytogenes_1639_v31	Listeria_innocua_FSL_S4-378_702456	0.8105
Listeria_monocytogenes_1639_v30	Listeria_innocua_FSL_S4-378_702456	0.8142
Listeria_monocytogenes_1639_v2	Listeria_innocua_FSL_S4-378_702456	0.801
Listeria_monocytogenes_1639_v29	Listeria_innocua_FSL_S4-378_702456	0.8107
Listeria_monocytogenes_1639_v28	Listeria_innocua_FSL_S4-378_702456	0.8146
Listeria_monocytogenes_1639_v27	Listeria_innocua_FSL_S4-378_702456	0.809
Listeria_monocytogenes_1639_v26	Listeria_innocua_FSL_S4-378_702456	0.8091
Listeria_monocytogenes_1639_v25	Listeria_innocua_FSL_S4-378_702456	0.8092
Listeria_monocytogenes_1639_v24	Listeria_innocua_FSL_S4-378_702456	0.8191
Listeria_monocytogenes_1639_v23	Listeria_innocua_FSL_S4-378_702456	0.8191
Listeria_monocytogenes_1639_v22	Listeria_innocua_FSL_S4-378_702456	0.8192
Listeria_monocytogenes_1639_v21	Listeria_innocua_FSL_S4-378_702456	0.8195
Listeria_monocytogenes_1639_v20	Listeria_innocua_FSL_S4-378_702456	0.8126
Listeria_monocytogenes_1639_v1	Listeria_innocua_FSL_S4-378_702456	0.8025
Listeria_monocytogenes_1639_v19	Listeria_innocua_FSL_S4-378_702456	0.8123
Listeria_monocytogenes_1639_v198	Listeria_innocua_FSL_S4-378_702456	0.7891
Listeria_monocytogenes_1639_v197	Listeria_innocua_FSL_S4-378_702456	0.8105
Listeria_monocytogenes_1639_v196	Listeria_innocua_FSL_S4-378_702456	0.8157
Listeria_monocytogenes_1639_v195	Listeria_innocua_FSL_S4-378_702456	0.8354
Listeria_monocytogenes_1639_v194	Listeria_innocua_FSL_S4-378_702456	0.8376
Listeria_monocytogenes_1639_v193	Listeria_innocua_FSL_S4-378_702456	0.8372
Listeria_monocytogenes_1639_v192	Listeria_innocua_FSL_S4-378_702456	0.8173
Listeria_monocytogenes_1639_v191	Listeria_innocua_FSL_S4-378_702456	0.8339
Listeria_monocytogenes_1639_v190	Listeria_innocua_FSL_S4-378_702456	0.8349
Listeria_monocytogenes_1639_v18	Listeria_innocua_FSL_S4-378_702456	0.814
Listeria_monocytogenes_1639_v189	Listeria_innocua_FSL_S4-378_702456	0.8346
Listeria_monocytogenes_1639_v188	Listeria_innocua_FSL_S4-378_702456	0.8326
Listeria_monocytogenes_1639_v187	Listeria_innocua_FSL_S4-378_702456	0.8349
Listeria_monocytogenes_1639_v186	Listeria_innocua_FSL_S4-378_702456	0.8352
Listeria_monocytogenes_1639_v185	Listeria_innocua_FSL_S4-378_702456	0.8349
Listeria_monocytogenes_1639_v184	Listeria_innocua_FSL_S4-378_702456	0.8345
Listeria_monocytogenes_1639_v183	Listeria_innocua_FSL_S4-378_702456	0.8258
Listeria_monocytogenes_1639_v182	Listeria_innocua_FSL_S4-378_702456	0.8275
Listeria_monocytogenes_1639_v181	Listeria_innocua_FSL_S4-378_702456	0.8324
Listeria_monocytogenes_1639_v180	Listeria_innocua_FSL_S4-378_702456	0.8324
Listeria_monocytogenes_1639_v17	Listeria_innocua_FSL_S4-378_702456	0.814
Listeria_monocytogenes_1639_v179	Listeria_innocua_FSL_S4-378_702456	0.8198
Listeria_monocytogenes_1639_v178	Listeria_innocua_FSL_S4-378_702456	0.829
Listeria_monocytogenes_1639_v177	Listeria_innocua_FSL_S4-378_702456	0.8308
Listeria_monocytogenes_1639_v176	Listeria_innocua_FSL_S4-378_702456	0.8323
Listeria_monocytogenes_1639_v175	Listeria_innocua_FSL_S4-378_702456	0.8247
Listeria_monocytogenes_1639_v174	Listeria_innocua_FSL_S4-378_702456	0.8308
Listeria_monocytogenes_1639_v173	Listeria_innocua_FSL_S4-378_702456	0.834
Listeria_monocytogenes_1639_v172	Listeria_innocua_FSL_S4-378_702456	0.8322
Listeria_monocytogenes_1639_v171	Listeria_innocua_FSL_S4-378_702456	0.8221
Listeria_monocytogenes_1639_v170	Listeria_innocua_FSL_S4-378_702456	0.8295
Listeria_monocytogenes_1639_v16	Listeria_innocua_FSL_S4-378_702456	0.799
Listeria_monocytogenes_1639_v169	Listeria_innocua_FSL_S4-378_702456	0.8285
Listeria_monocytogenes_1639_v168	Listeria_innocua_FSL_S4-378_702456	0.8255
Listeria_monocytogenes_1639_v167	Listeria_innocua_FSL_S4-378_702456	0.8243
Listeria_monocytogenes_1639_v166	Listeria_innocua_FSL_S4-378_702456	0.8259
Listeria_monocytogenes_1639_v165	Listeria_innocua_FSL_S4-378_702456	0.8282
Listeria_monocytogenes_1639_v164	Listeria_innocua_FSL_S4-378_702456	0.8322
Listeria_monocytogenes_1639_v163	Listeria_innocua_FSL_S4-378_702456	0.8269
Listeria_monocytogenes_1639_v162	Listeria_innocua_FSL_S4-378_702456	0.8322
Listeria_monocytogenes_1639_v161	Listeria_innocua_FSL_S4-378_702456	0.8331
Listeria_monocytogenes_1639_v160	Listeria_innocua_FSL_S4-378_702456	0.8331
Listeria_monocytogenes_1639_v15	Listeria_innocua_FSL_S4-378_702456	0.8049
Listeria_monocytogenes_1639_v159	Listeria_innocua_FSL_S4-378_702456	0.8312
Listeria_monocytogenes_1639_v158	Listeria_innocua_FSL_S4-378_702456	0.8332
Listeria_monocytogenes_1639_v157	Listeria_innocua_FSL_S4-378_702456	0.8332
Listeria_monocytogenes_1639_v156	Listeria_innocua_FSL_S4-378_702456	0.8201
Listeria_monocytogenes_1639_v155	Listeria_innocua_FSL_S4-378_702456	0.825
Listeria_monocytogenes_1639_v154	Listeria_innocua_FSL_S4-378_702456	0.8252
Listeria_monocytogenes_1639_v153	Listeria_innocua_FSL_S4-378_702456	0.8277
Listeria_monocytogenes_1639_v152	Listeria_innocua_FSL_S4-378_702456	0.8315
Listeria_monocytogenes_1639_v151	Listeria_innocua_FSL_S4-378_702456	0.8315
Listeria_monocytogenes_1639_v150	Listeria_innocua_FSL_S4-378_702456	0.8281
Listeria_monocytogenes_1639_v14	Listeria_innocua_FSL_S4-378_702456	0.8192
Listeria_monocytogenes_1639_v149	Listeria_innocua_FSL_S4-378_702456	0.8281

Listeria_monocytogenes_1639_v148	Listeria_innocua_FSL_S4-378_702456	0.8301
Listeria_monocytogenes_1639_v147	Listeria_innocua_FSL_S4-378_702456	0.8188
Listeria_monocytogenes_1639_v146	Listeria_innocua_FSL_S4-378_702456	0.8303
Listeria_monocytogenes_1639_v145	Listeria_innocua_FSL_S4-378_702456	0.8273
Listeria_monocytogenes_1639_v144	Listeria_innocua_FSL_S4-378_702456	0.8382
Listeria_monocytogenes_1639_v143	Listeria_innocua_FSL_S4-378_702456	0.8278
Listeria_monocytogenes_1639_v142	Listeria_innocua_FSL_S4-378_702456	0.8386
Listeria_monocytogenes_1639_v141	Listeria_innocua_FSL_S4-378_702456	0.8312
Listeria_monocytogenes_1639_v140	Listeria_innocua_FSL_S4-378_702456	0.8389
Listeria_monocytogenes_1639_v13	Listeria_innocua_FSL_S4-378_702456	0.8048
Listeria_monocytogenes_1639_v139	Listeria_innocua_FSL_S4-378_702456	0.8341
Listeria_monocytogenes_1639_v138	Listeria_innocua_FSL_S4-378_702456	0.8382
Listeria_monocytogenes_1639_v137	Listeria_innocua_FSL_S4-378_702456	0.8378
Listeria_monocytogenes_1639_v136	Listeria_innocua_FSL_S4-378_702456	0.8228
Listeria_monocytogenes_1639_v135	Listeria_innocua_FSL_S4-378_702456	0.8196
Listeria_monocytogenes_1639_v134	Listeria_innocua_FSL_S4-378_702456	0.8276
Listeria_monocytogenes_1639_v133	Listeria_innocua_FSL_S4-378_702456	0.8264
Listeria_monocytogenes_1639_v132	Listeria_innocua_FSL_S4-378_702456	0.8183
Listeria_monocytogenes_1639_v131	Listeria_innocua_FSL_S4-378_702456	0.827
Listeria_monocytogenes_1639_v130	Listeria_innocua_FSL_S4-378_702456	0.8134
Listeria_monocytogenes_1639_v12	Listeria_innocua_FSL_S4-378_702456	0.8053
Listeria_monocytogenes_1639_v129	Listeria_innocua_FSL_S4-378_702456	0.8292
Listeria_monocytogenes_1639_v128	Listeria_innocua_FSL_S4-378_702456	0.8277
Listeria_monocytogenes_1639_v127	Listeria_innocua_FSL_S4-378_702456	0.8245
Listeria_monocytogenes_1639_v126	Listeria_innocua_FSL_S4-378_702456	0.8273
Listeria_monocytogenes_1639_v125	Listeria_innocua_FSL_S4-378_702456	0.8194
Listeria_monocytogenes_1639_v124	Listeria_innocua_FSL_S4-378_702456	0.8312
Listeria_monocytogenes_1639_v123	Listeria_innocua_FSL_S4-378_702456	0.8328
Listeria_monocytogenes_1639_v122	Listeria_innocua_FSL_S4-378_702456	0.8331
Listeria_monocytogenes_1639_v121	Listeria_innocua_FSL_S4-378_702456	0.8331
Listeria_monocytogenes_1639_v120	Listeria_innocua_FSL_S4-378_702456	0.8328
Listeria_monocytogenes_1639_v11	Listeria_innocua_FSL_S4-378_702456	0.8044
Listeria_monocytogenes_1639_v119	Listeria_innocua_FSL_S4-378_702456	0.8331
Listeria_monocytogenes_1639_v118	Listeria_innocua_FSL_S4-378_702456	0.8331
Listeria_monocytogenes_1639_v117	Listeria_innocua_FSL_S4-378_702456	0.8192
Listeria_monocytogenes_1639_v116	Listeria_innocua_FSL_S4-378_702456	0.8274
Listeria_monocytogenes_1639_v115	Listeria_innocua_FSL_S4-378_702456	0.8278
Listeria_monocytogenes_1639_v114	Listeria_innocua_FSL_S4-378_702456	0.8232
Listeria_monocytogenes_1639_v113	Listeria_innocua_FSL_S4-378_702456	0.8256
Listeria_monocytogenes_1639_v112	Listeria_innocua_FSL_S4-378_702456	0.825
Listeria_monocytogenes_1639_v111	Listeria_innocua_FSL_S4-378_702456	0.8236
Listeria_monocytogenes_1639_v110	Listeria_innocua_FSL_S4-378_702456	0.8245
Listeria_monocytogenes_1639_v10	Listeria_innocua_FSL_S4-378_702456	0.8108
Listeria_monocytogenes_1639_v109	Listeria_innocua_FSL_S4-378_702456	0.8218
Listeria_monocytogenes_1639_v108	Listeria_innocua_FSL_S4-378_702456	0.8278
Listeria_monocytogenes_1639_v107	Listeria_innocua_FSL_S4-378_702456	0.8278
Listeria_monocytogenes_1639_v106	Listeria_innocua_FSL_S4-378_702456	0.8282
Listeria_monocytogenes_1639_v105	Listeria_innocua_FSL_S4-378_702456	0.8282
Listeria_monocytogenes_1639_v104	Listeria_innocua_FSL_S4-378_702456	0.8285
Listeria_monocytogenes_1639_v103	Listeria_innocua_FSL_S4-378_702456	0.8285
Listeria_monocytogenes_1639_v102	Listeria_innocua_FSL_S4-378_702456	0.8185
Listeria_monocytogenes_1639_v101	Listeria_innocua_FSL_S4-378_702456	0.8083
Listeria_monocytogenes_1639_v100	Listeria_innocua_FSL_S4-378_702456	0.8078
Listeria_monocytogenes_1639	Listeria_innocua_FSL_S4-378_702456	0.8346
Listeria_monocytogenes_10403S_393133	Listeria_innocua_FSL_S4-378_702456	0.8316
Listeria_monocytogenes_08-5923_637381	Listeria_innocua_FSL_S4-378_702456	0.8108
Listeria_monocytogenes_08-5578_653938	Listeria_innocua_FSL_S4-378_702456	0.7987
Listeria_monocytogenes_07PF0776_1126011	Listeria_innocua_FSL_S4-378_702456	0.8313
Listeria_marthii_FSL_S4-120_702457	Listeria_innocua_FSL_S4-378_702456	0.7963
Listeria_marthii_529731_v1	Listeria_innocua_FSL_S4-378_702456	0.8184
Listeria_marthii_529731	Listeria_innocua_FSL_S4-378_702456	0.8125
Listeria_ivanovii_WSLC3009_1457190	Listeria_innocua_FSL_S4-378_702456	0.7945
Listeria_ivanovii_subsp_londoniensis_202752_v2	Listeria_innocua_FSL_S4-378_702456	0.7908
Listeria_ivanovii_subsp_londoniensis_202752_v1	Listeria_innocua_FSL_S4-378_702456	0.7833
Listeria_ivanovii_subsp_londoniensis_202752	Listeria_innocua_FSL_S4-378_702456	0.7873
Listeria_ivanovii_subsp_ivanovii_PAM_55_881621	Listeria_innocua_FSL_S4-378_702456	0.7936
Listeria_ivanovii_subsp_ivanovii_202751_v2	Listeria_innocua_FSL_S4-378_702456	0.7951
Listeria_ivanovii_subsp_ivanovii_202751_v1	Listeria_innocua_FSL_S4-378_702456	0.7951
Listeria_ivanovii_subsp_ivanovii_202751	Listeria_innocua_FSL_S4-378_702456	0.7951
Listeria_ivanovii_FSL_F6-596_702454	Listeria_innocua_FSL_S4-378_702456	0.7471
Listeria_ivanovii_1638	Listeria_innocua_FSL_S4-378_702456	0.7952
Listeria_innocua_FSL_S4-378_702456	Listeria_innocua_FSL_S4-378_702456	0.0457
Listeria_welshimeri_1643	Listeria_ivanovii_1638	0.8619
Listeria_welshimeri_serovar_6b_str_SLCC5334_386043	Listeria_ivanovii_1638	0.8616
Listeria_seeligeri_serovar_1_2b_str_SLCC3954_683837	Listeria_ivanovii_1638	0.8833
Listeria_seeligeri_FSL_S4-171_702452	Listeria_ivanovii_1638	0.8195
Listeria_seeligeri_FSL_N1-067_702453	Listeria_ivanovii_1638	0.8261
Listeria_seeligeri_1640_v1	Listeria_ivanovii_1638	0.8918
Listeria_seeligeri_1640	Listeria_ivanovii_1638	0.8769
Listeria_monocytogenes_WSLC1042_1457188	Listeria_ivanovii_1638	0.8983
Listeria_monocytogenes_WSLC1001_1457187	Listeria_ivanovii_1638	0.8812
Listeria_monocytogenes_SLCC7179_879090	Listeria_ivanovii_1638	0.8885
Listeria_monocytogenes_SLCC5850_882096	Listeria_ivanovii_1638	0.886
Listeria_monocytogenes_SLCC2755_932919	Listeria_ivanovii_1638	0.8877
Listeria_monocytogenes_SLCC2540_879089	Listeria_ivanovii_1638	0.8907
Listeria_monocytogenes_SLCC2479_882020	Listeria_ivanovii_1638	0.8797
Listeria_monocytogenes_SLCC2378_879088	Listeria_ivanovii_1638	0.8993
Listeria_monocytogenes_SLCC2376_882097	Listeria_ivanovii_1638	0.8975
Listeria_monocytogenes_SLCC2372_932920	Listeria_ivanovii_1638	0.8724
Listeria_monocytogenes_serotype_4b_str_LL195_1230340	Listeria_ivanovii_1638	0.9052
Listeria_monocytogenes_serotype_4b_str_LIS0087_1134669	Listeria_ivanovii_1638	0.8622

Listeria_monocytogenes_serotype_4b_str_F2365_265669	Listeria_ivanovii_1638	0.9052
Listeria_monocytogenes_serotype_4b_str_CLIP_80459_568819	Listeria_ivanovii_1638	0.9039
Listeria_monocytogenes_serotype_4b_str_81-0861_1196161	Listeria_ivanovii_1638	0.8887
Listeria_monocytogenes_serotype_4b_str_81-0592_1196160	Listeria_ivanovii_1638	0.8897
Listeria_monocytogenes_serotype_4b_str_81-0558_1196159	Listeria_ivanovii_1638	0.8904
Listeria_monocytogenes_serotype_4b_str_10-0809_1196162	Listeria_ivanovii_1638	0.8901
Listeria_monocytogenes_serotype_4b_str_02-6680_1196175	Listeria_ivanovii_1638	0.903
Listeria_monocytogenes_serotype_4b_str_02-6679_1196174	Listeria_ivanovii_1638	0.903
Listeria_monocytogenes_serotype_4b_str_02-1792_1196173	Listeria_ivanovii_1638	0.8901
Listeria_monocytogenes_serotype_4b_str_02-1289_1196172	Listeria_ivanovii_1638	0.8901
Listeria_monocytogenes_serotype_4b_str_02-1103_1196171	Listeria_ivanovii_1638	0.8901
Listeria_monocytogenes_serotype_3c_str_10-5027_1196195	Listeria_ivanovii_1638	0.8785
Listeria_monocytogenes_serotype_1_2c_str_10-5026_1196194	Listeria_ivanovii_1638	0.8793
Listeria_monocytogenes_serotype_1_2c_str_10-5025_1196193	Listeria_ivanovii_1638	0.8793
Listeria_monocytogenes_serotype_1_2b_str_10-0811_1196164	Listeria_ivanovii_1638	0.8795
Listeria_monocytogenes_serotype_1_2b_str_10-0810_1196163	Listeria_ivanovii_1638	0.8795
Listeria_monocytogenes_serotype_1_2a_str_99-6370_1196179	Listeria_ivanovii_1638	0.866
Listeria_monocytogenes_serotype_1_2a_str_98-2035_1196178	Listeria_ivanovii_1638	0.866
Listeria_monocytogenes_serotype_1_2a_str_95-0093_1196177	Listeria_ivanovii_1638	0.8711
Listeria_monocytogenes_serotype_1_2a_str_88-0478_1196176	Listeria_ivanovii_1638	0.87
Listeria_monocytogenes_serotype_1_2a_str_10-5024_1196192	Listeria_ivanovii_1638	0.8711
Listeria_monocytogenes_serotype_1_2a_str_10-4758_1196168	Listeria_ivanovii_1638	0.8854
Listeria_monocytogenes_serotype_1_2a_str_10-4754_1196167	Listeria_ivanovii_1638	0.8857
Listeria_monocytogenes_serotype_1_2a_str_10-1321_1196191	Listeria_ivanovii_1638	0.8711
Listeria_monocytogenes_serotype_1_2a_str_10-1047_1196190	Listeria_ivanovii_1638	0.8718
Listeria_monocytogenes_serotype_1_2a_str_10-1046_1196189	Listeria_ivanovii_1638	0.8718
Listeria_monocytogenes_serotype_1_2a_str_10-0934_1196170	Listeria_ivanovii_1638	0.8959
Listeria_monocytogenes_serotype_1_2a_str_10-0933_1196169	Listeria_ivanovii_1638	0.8959
Listeria_monocytogenes_serotype_1_2a_str_10-0815_1196188	Listeria_ivanovii_1638	0.8673
Listeria_monocytogenes_serotype_1_2a_str_10-0814_1196187	Listeria_ivanovii_1638	0.8715
Listeria_monocytogenes_serotype_1_2a_str_10-0813_1196166	Listeria_ivanovii_1638	0.8789
Listeria_monocytogenes_serotype_1_2a_str_10-0812_1196165	Listeria_ivanovii_1638	0.8789
Listeria_monocytogenes_serotype_1_2a_str_08-7669_1196186	Listeria_ivanovii_1638	0.8797
Listeria_monocytogenes_serotype_1_2a_str_08-7374_1196185	Listeria_ivanovii_1638	0.8711
Listeria_monocytogenes_serotype_1_2a_str_08-6997_1196184	Listeria_ivanovii_1638	0.8673
Listeria_monocytogenes_serotype_1_2a_str_08-6569_1196183	Listeria_ivanovii_1638	0.8673
Listeria_monocytogenes_serotype_1_2a_str_08-6056_1196182	Listeria_ivanovii_1638	0.8673
Listeria_monocytogenes_serotype_1_2a_str_04-5457_1196181	Listeria_ivanovii_1638	0.8711
Listeria_monocytogenes_serotype_1_2a_str_02-5993_1196180	Listeria_ivanovii_1638	0.8711
Listeria_monocytogenes_R479a_1437838	Listeria_ivanovii_1638	0.8709
Listeria_monocytogenes_N53-1_1234142	Listeria_ivanovii_1638	0.8499
Listeria_monocytogenes_M7_1030009	Listeria_ivanovii_1638	0.8608
Listeria_monocytogenes_LO28_393132	Listeria_ivanovii_1638	0.8784
Listeria_monocytogenes_L99_563174	Listeria_ivanovii_1638	0.8612
Listeria_monocytogenes_L312_882094	Listeria_ivanovii_1638	0.9039
Listeria_monocytogenes_J1816_930781	Listeria_ivanovii_1638	0.8815
Listeria_monocytogenes_J1-220_930782	Listeria_ivanovii_1638	0.8789
Listeria_monocytogenes_J0161_393130	Listeria_ivanovii_1638	0.8784
Listeria_monocytogenes_HCC23_552536	Listeria_ivanovii_1638	0.8608
Listeria_monocytogenes_FSL_R2-561_393126	Listeria_ivanovii_1638	0.878
Listeria_monocytogenes_FSL_J1-208_393119	Listeria_ivanovii_1638	0.8744
Listeria_monocytogenes_FSL_J1-175_393118	Listeria_ivanovii_1638	0.8952
Listeria_monocytogenes_Finland_1998_393127	Listeria_ivanovii_1638	0.897
Listeria_monocytogenes_EGD-e_169963_v1	Listeria_ivanovii_1638	0.8863
Listeria_monocytogenes_EGD-e_169963	Listeria_ivanovii_1638	0.8867
Listeria_monocytogenes_EGD_1334565	Listeria_ivanovii_1638	0.8863
Listeria_monocytogenes_CFSAN002349_1299895	Listeria_ivanovii_1638	0.9004
Listeria_monocytogenes_ATCC_19117_882095_v1	Listeria_ivanovii_1638	0.8978
Listeria_monocytogenes_ATCC_19117_882095	Listeria_ivanovii_1638	0.8975
Listeria_monocytogenes_6179_1288295	Listeria_ivanovii_1638	0.863
Listeria_monocytogenes_1639_v9	Listeria_ivanovii_1638	0.8678
Listeria_monocytogenes_1639_v99	Listeria_ivanovii_1638	0.8812
Listeria_monocytogenes_1639_v98	Listeria_ivanovii_1638	0.8771
Listeria_monocytogenes_1639_v97	Listeria_ivanovii_1638	0.8931
Listeria_monocytogenes_1639_v96	Listeria_ivanovii_1638	0.8805
Listeria_monocytogenes_1639_v95	Listeria_ivanovii_1638	0.8806
Listeria_monocytogenes_1639_v94	Listeria_ivanovii_1638	0.8808
Listeria_monocytogenes_1639_v93	Listeria_ivanovii_1638	0.8737
Listeria_monocytogenes_1639_v92	Listeria_ivanovii_1638	0.8808
Listeria_monocytogenes_1639_v91	Listeria_ivanovii_1638	0.8874
Listeria_monocytogenes_1639_v90	Listeria_ivanovii_1638	0.8771
Listeria_monocytogenes_1639_v8	Listeria_ivanovii_1638	0.8605
Listeria_monocytogenes_1639_v89	Listeria_ivanovii_1638	0.8938
Listeria_monocytogenes_1639_v88	Listeria_ivanovii_1638	0.8779
Listeria_monocytogenes_1639_v87	Listeria_ivanovii_1638	0.8735
Listeria_monocytogenes_1639_v86	Listeria_ivanovii_1638	0.8739
Listeria_monocytogenes_1639_v85	Listeria_ivanovii_1638	0.8884
Listeria_monocytogenes_1639_v84	Listeria_ivanovii_1638	0.8799
Listeria_monocytogenes_1639_v83	Listeria_ivanovii_1638	0.8873
Listeria_monocytogenes_1639_v82	Listeria_ivanovii_1638	0.887
Listeria_monocytogenes_1639_v81	Listeria_ivanovii_1638	0.8783
Listeria_monocytogenes_1639_v80	Listeria_ivanovii_1638	0.8837
Listeria_monocytogenes_1639_v7	Listeria_ivanovii_1638	0.8605
Listeria_monocytogenes_1639_v79	Listeria_ivanovii_1638	0.8766
Listeria_monocytogenes_1639_v78	Listeria_ivanovii_1638	0.8776
Listeria_monocytogenes_1639_v77	Listeria_ivanovii_1638	0.8798
Listeria_monocytogenes_1639_v76	Listeria_ivanovii_1638	0.8702
Listeria_monocytogenes_1639_v75	Listeria_ivanovii_1638	0.8612
Listeria_monocytogenes_1639_v74	Listeria_ivanovii_1638	0.8746
Listeria_monocytogenes_1639_v73	Listeria_ivanovii_1638	0.8738
Listeria_monocytogenes_1639_v72	Listeria_ivanovii_1638	0.8861

Listeria_monocytogenes_1639_v71	Listeria_ivanovii_1638	0.8651
Listeria_monocytogenes_1639_v70	Listeria_ivanovii_1638	0.8761
Listeria_monocytogenes_1639_v6	Listeria_ivanovii_1638	0.8602
Listeria_monocytogenes_1639_v69	Listeria_ivanovii_1638	0.8897
Listeria_monocytogenes_1639_v68	Listeria_ivanovii_1638	0.8824
Listeria_monocytogenes_1639_v67	Listeria_ivanovii_1638	0.887
Listeria_monocytogenes_1639_v66	Listeria_ivanovii_1638	0.8852
Listeria_monocytogenes_1639_v65	Listeria_ivanovii_1638	0.8742
Listeria_monocytogenes_1639_v64	Listeria_ivanovii_1638	0.8773
Listeria_monocytogenes_1639_v63	Listeria_ivanovii_1638	0.8805
Listeria_monocytogenes_1639_v62	Listeria_ivanovii_1638	0.8743
Listeria_monocytogenes_1639_v61	Listeria_ivanovii_1638	0.8737
Listeria_monocytogenes_1639_v60	Listeria_ivanovii_1638	0.8797
Listeria_monocytogenes_1639_v5	Listeria_ivanovii_1638	0.8478
Listeria_monocytogenes_1639_v59	Listeria_ivanovii_1638	0.8876
Listeria_monocytogenes_1639_v58	Listeria_ivanovii_1638	0.8907
Listeria_monocytogenes_1639_v57	Listeria_ivanovii_1638	0.8776
Listeria_monocytogenes_1639_v56	Listeria_ivanovii_1638	0.8876
Listeria_monocytogenes_1639_v55	Listeria_ivanovii_1638	0.8736
Listeria_monocytogenes_1639_v54	Listeria_ivanovii_1638	0.8926
Listeria_monocytogenes_1639_v53	Listeria_ivanovii_1638	0.8808
Listeria_monocytogenes_1639_v52	Listeria_ivanovii_1638	0.8846
Listeria_monocytogenes_1639_v51	Listeria_ivanovii_1638	0.8826
Listeria_monocytogenes_1639_v50	Listeria_ivanovii_1638	0.87
Listeria_monocytogenes_1639_v4	Listeria_ivanovii_1638	0.8595
Listeria_monocytogenes_1639_v49	Listeria_ivanovii_1638	0.8898
Listeria_monocytogenes_1639_v48	Listeria_ivanovii_1638	0.8741
Listeria_monocytogenes_1639_v47	Listeria_ivanovii_1638	0.8708
Listeria_monocytogenes_1639_v46	Listeria_ivanovii_1638	0.8817
Listeria_monocytogenes_1639_v45	Listeria_ivanovii_1638	0.8625
Listeria_monocytogenes_1639_v44	Listeria_ivanovii_1638	0.8711
Listeria_monocytogenes_1639_v43	Listeria_ivanovii_1638	0.8711
Listeria_monocytogenes_1639_v42	Listeria_ivanovii_1638	0.8659
Listeria_monocytogenes_1639_v41	Listeria_ivanovii_1638	0.8549
Listeria_monocytogenes_1639_v40	Listeria_ivanovii_1638	0.8791
Listeria_monocytogenes_1639_v3	Listeria_ivanovii_1638	0.8598
Listeria_monocytogenes_1639_v39	Listeria_ivanovii_1638	0.883
Listeria_monocytogenes_1639_v38	Listeria_ivanovii_1638	0.8753
Listeria_monocytogenes_1639_v37	Listeria_ivanovii_1638	0.8712
Listeria_monocytogenes_1639_v36	Listeria_ivanovii_1638	0.8684
Listeria_monocytogenes_1639_v35	Listeria_ivanovii_1638	0.8797
Listeria_monocytogenes_1639_v34	Listeria_ivanovii_1638	0.8744
Listeria_monocytogenes_1639_v33	Listeria_ivanovii_1638	0.8795
Listeria_monocytogenes_1639_v32	Listeria_ivanovii_1638	0.8698
Listeria_monocytogenes_1639_v31	Listeria_ivanovii_1638	0.8748
Listeria_monocytogenes_1639_v30	Listeria_ivanovii_1638	0.8808
Listeria_monocytogenes_1639_v2	Listeria_ivanovii_1638	0.8496
Listeria_monocytogenes_1639_v29	Listeria_ivanovii_1638	0.866
Listeria_monocytogenes_1639_v28	Listeria_ivanovii_1638	0.8701
Listeria_monocytogenes_1639_v27	Listeria_ivanovii_1638	0.8619
Listeria_monocytogenes_1639_v26	Listeria_ivanovii_1638	0.8616
Listeria_monocytogenes_1639_v25	Listeria_ivanovii_1638	0.8616
Listeria_monocytogenes_1639_v24	Listeria_ivanovii_1638	0.8793
Listeria_monocytogenes_1639_v23	Listeria_ivanovii_1638	0.879
Listeria_monocytogenes_1639_v22	Listeria_ivanovii_1638	0.8801
Listeria_monocytogenes_1639_v21	Listeria_ivanovii_1638	0.879
Listeria_monocytogenes_1639_v20	Listeria_ivanovii_1638	0.8765
Listeria_monocytogenes_1639_v1	Listeria_ivanovii_1638	0.8571
Listeria_monocytogenes_1639_v19	Listeria_ivanovii_1638	0.8761
Listeria_monocytogenes_1639_v198	Listeria_ivanovii_1638	0.8531
Listeria_monocytogenes_1639_v197	Listeria_ivanovii_1638	0.8741
Listeria_monocytogenes_1639_v196	Listeria_ivanovii_1638	0.8685
Listeria_monocytogenes_1639_v195	Listeria_ivanovii_1638	0.8966
Listeria_monocytogenes_1639_v194	Listeria_ivanovii_1638	0.8975
Listeria_monocytogenes_1639_v193	Listeria_ivanovii_1638	0.8979
Listeria_monocytogenes_1639_v192	Listeria_ivanovii_1638	0.8667
Listeria_monocytogenes_1639_v191	Listeria_ivanovii_1638	0.8975
Listeria_monocytogenes_1639_v190	Listeria_ivanovii_1638	0.8969
Listeria_monocytogenes_1639_v18	Listeria_ivanovii_1638	0.8721
Listeria_monocytogenes_1639_v189	Listeria_ivanovii_1638	0.8965
Listeria_monocytogenes_1639_v188	Listeria_ivanovii_1638	0.8998
Listeria_monocytogenes_1639_v187	Listeria_ivanovii_1638	0.8972
Listeria_monocytogenes_1639_v186	Listeria_ivanovii_1638	0.8972
Listeria_monocytogenes_1639_v185	Listeria_ivanovii_1638	0.8972
Listeria_monocytogenes_1639_v184	Listeria_ivanovii_1638	0.8964
Listeria_monocytogenes_1639_v183	Listeria_ivanovii_1638	0.8984
Listeria_monocytogenes_1639_v182	Listeria_ivanovii_1638	0.9001
Listeria_monocytogenes_1639_v181	Listeria_ivanovii_1638	0.8845
Listeria_monocytogenes_1639_v180	Listeria_ivanovii_1638	0.8845
Listeria_monocytogenes_1639_v17	Listeria_ivanovii_1638	0.8724
Listeria_monocytogenes_1639_v179	Listeria_ivanovii_1638	0.8868
Listeria_monocytogenes_1639_v178	Listeria_ivanovii_1638	0.9011
Listeria_monocytogenes_1639_v177	Listeria_ivanovii_1638	0.8984
Listeria_monocytogenes_1639_v176	Listeria_ivanovii_1638	0.8965
Listeria_monocytogenes_1639_v175	Listeria_ivanovii_1638	0.889
Listeria_monocytogenes_1639_v174	Listeria_ivanovii_1638	0.8982
Listeria_monocytogenes_1639_v173	Listeria_ivanovii_1638	0.9048
Listeria_monocytogenes_1639_v172	Listeria_ivanovii_1638	0.8902
Listeria_monocytogenes_1639_v171	Listeria_ivanovii_1638	0.8828
Listeria_monocytogenes_1639_v170	Listeria_ivanovii_1638	0.8906
Listeria_monocytogenes_1639_v16	Listeria_ivanovii_1638	0.8583

Listeria_monocytogenes_1639_v169	Listeria_ivanovii_1638	0.8908
Listeria_monocytogenes_1639_v168	Listeria_ivanovii_1638	0.8964
Listeria_monocytogenes_1639_v167	Listeria_ivanovii_1638	0.8906
Listeria_monocytogenes_1639_v166	Listeria_ivanovii_1638	0.8967
Listeria_monocytogenes_1639_v165	Listeria_ivanovii_1638	0.8904
Listeria_monocytogenes_1639_v164	Listeria_ivanovii_1638	0.9066
Listeria_monocytogenes_1639_v163	Listeria_ivanovii_1638	0.8979
Listeria_monocytogenes_1639_v162	Listeria_ivanovii_1638	0.9066
Listeria_monocytogenes_1639_v161	Listeria_ivanovii_1638	0.9059
Listeria_monocytogenes_1639_v160	Listeria_ivanovii_1638	0.9059
Listeria_monocytogenes_1639_v15	Listeria_ivanovii_1638	0.8659
Listeria_monocytogenes_1639_v159	Listeria_ivanovii_1638	0.8984
Listeria_monocytogenes_1639_v158	Listeria_ivanovii_1638	0.9052
Listeria_monocytogenes_1639_v157	Listeria_ivanovii_1638	0.9052
Listeria_monocytogenes_1639_v156	Listeria_ivanovii_1638	0.8762
Listeria_monocytogenes_1639_v155	Listeria_ivanovii_1638	0.8888
Listeria_monocytogenes_1639_v154	Listeria_ivanovii_1638	0.8888
Listeria_monocytogenes_1639_v153	Listeria_ivanovii_1638	0.8891
Listeria_monocytogenes_1639_v152	Listeria_ivanovii_1638	0.8984
Listeria_monocytogenes_1639_v151	Listeria_ivanovii_1638	0.8984
Listeria_monocytogenes_1639_v150	Listeria_ivanovii_1638	0.888
Listeria_monocytogenes_1639_v14	Listeria_ivanovii_1638	0.8713
Listeria_monocytogenes_1639_v149	Listeria_ivanovii_1638	0.8867
Listeria_monocytogenes_1639_v148	Listeria_ivanovii_1638	0.8973
Listeria_monocytogenes_1639_v147	Listeria_ivanovii_1638	0.8852
Listeria_monocytogenes_1639_v146	Listeria_ivanovii_1638	0.9014
Listeria_monocytogenes_1639_v145	Listeria_ivanovii_1638	0.8932
Listeria_monocytogenes_1639_v144	Listeria_ivanovii_1638	0.904
Listeria_monocytogenes_1639_v143	Listeria_ivanovii_1638	0.8981
Listeria_monocytogenes_1639_v142	Listeria_ivanovii_1638	0.904
Listeria_monocytogenes_1639_v141	Listeria_ivanovii_1638	0.8992
Listeria_monocytogenes_1639_v140	Listeria_ivanovii_1638	0.9043
Listeria_monocytogenes_1639_v13	Listeria_ivanovii_1638	0.8659
Listeria_monocytogenes_1639_v139	Listeria_ivanovii_1638	0.8971
Listeria_monocytogenes_1639_v138	Listeria_ivanovii_1638	0.904
Listeria_monocytogenes_1639_v137	Listeria_ivanovii_1638	0.9032
Listeria_monocytogenes_1639_v136	Listeria_ivanovii_1638	0.8851
Listeria_monocytogenes_1639_v135	Listeria_ivanovii_1638	0.873
Listeria_monocytogenes_1639_v134	Listeria_ivanovii_1638	0.899
Listeria_monocytogenes_1639_v133	Listeria_ivanovii_1638	0.8919
Listeria_monocytogenes_1639_v132	Listeria_ivanovii_1638	0.8756
Listeria_monocytogenes_1639_v131	Listeria_ivanovii_1638	0.8872
Listeria_monocytogenes_1639_v130	Listeria_ivanovii_1638	0.879
Listeria_monocytogenes_1639_v12	Listeria_ivanovii_1638	0.8603
Listeria_monocytogenes_1639_v129	Listeria_ivanovii_1638	0.9001
Listeria_monocytogenes_1639_v128	Listeria_ivanovii_1638	0.8958
Listeria_monocytogenes_1639_v127	Listeria_ivanovii_1638	0.8867
Listeria_monocytogenes_1639_v126	Listeria_ivanovii_1638	0.8943
Listeria_monocytogenes_1639_v125	Listeria_ivanovii_1638	0.8881
Listeria_monocytogenes_1639_v124	Listeria_ivanovii_1638	0.8878
Listeria_monocytogenes_1639_v123	Listeria_ivanovii_1638	0.9004
Listeria_monocytogenes_1639_v122	Listeria_ivanovii_1638	0.9004
Listeria_monocytogenes_1639_v121	Listeria_ivanovii_1638	0.9004
Listeria_monocytogenes_1639_v120	Listeria_ivanovii_1638	0.9008
Listeria_monocytogenes_1639_v11	Listeria_ivanovii_1638	0.8593
Listeria_monocytogenes_1639_v119	Listeria_ivanovii_1638	0.9004
Listeria_monocytogenes_1639_v118	Listeria_ivanovii_1638	0.9004
Listeria_monocytogenes_1639_v117	Listeria_ivanovii_1638	0.8858
Listeria_monocytogenes_1639_v116	Listeria_ivanovii_1638	0.899
Listeria_monocytogenes_1639_v115	Listeria_ivanovii_1638	0.8993
Listeria_monocytogenes_1639_v114	Listeria_ivanovii_1638	0.8795
Listeria_monocytogenes_1639_v113	Listeria_ivanovii_1638	0.8943
Listeria_monocytogenes_1639_v112	Listeria_ivanovii_1638	0.8952
Listeria_monocytogenes_1639_v111	Listeria_ivanovii_1638	0.8911
Listeria_monocytogenes_1639_v110	Listeria_ivanovii_1638	0.8792
Listeria_monocytogenes_1639_v10	Listeria_ivanovii_1638	0.8641
Listeria_monocytogenes_1639_v109	Listeria_ivanovii_1638	0.8792
Listeria_monocytogenes_1639_v108	Listeria_ivanovii_1638	0.8886
Listeria_monocytogenes_1639_v107	Listeria_ivanovii_1638	0.8968
Listeria_monocytogenes_1639_v106	Listeria_ivanovii_1638	0.8968
Listeria_monocytogenes_1639_v105	Listeria_ivanovii_1638	0.8972
Listeria_monocytogenes_1639_v104	Listeria_ivanovii_1638	0.8968
Listeria_monocytogenes_1639_v103	Listeria_ivanovii_1638	0.8972
Listeria_monocytogenes_1639_v102	Listeria_ivanovii_1638	0.8814
Listeria_monocytogenes_1639_v101	Listeria_ivanovii_1638	0.8705
Listeria_monocytogenes_1639_v100	Listeria_ivanovii_1638	0.8703
Listeria_monocytogenes_1639	Listeria_ivanovii_1638	0.8969
Listeria_monocytogenes_10403S_393133	Listeria_ivanovii_1638	0.8892
Listeria_monocytogenes_08-5923_637381	Listeria_ivanovii_1638	0.8711
Listeria_monocytogenes_08-5578_653938	Listeria_ivanovii_1638	0.8583
Listeria_monocytogenes_07PF0776_1126011	Listeria_ivanovii_1638	0.9037
Listeria_marthii_FSL_S4-120_702457	Listeria_ivanovii_1638	0.8283
Listeria_marthii_529731_v1	Listeria_ivanovii_1638	0.8642
Listeria_marthii_529731	Listeria_ivanovii_1638	0.8738
Listeria_ivanovii_WSLC3009_1457190	Listeria_ivanovii_1638	0.9981
Listeria_ivanovii_subsp_londoniensis_202752_v2	Listeria_ivanovii_1638	0.9024
Listeria_ivanovii_subsp_londoniensis_202752_v1	Listeria_ivanovii_1638	0.8914
Listeria_ivanovii_subsp_londoniensis_202752	Listeria_ivanovii_1638	0.8981
Listeria_ivanovii_subsp_ivanovii_PAM_55_881621	Listeria_ivanovii_1638	0.9946
Listeria_ivanovii_subsp_ivanovii_202751_v2	Listeria_ivanovii_1638	0.9985
Listeria_ivanovii_subsp_ivanovii_202751_v1	Listeria_ivanovii_1638	0.9985

Listeria_ivanovii_subsp_ivanovii_202751	Listeria_ivanovii_1638	0.9985
Listeria_ivanovii_FSL_F6-596_702454	Listeria_ivanovii_1638	0.8338
Listeria_ivanovii_1638	Listeria_ivanovii_1638	0.062
Listeria_welshimeri_1643	Listeria_ivanovii_FSL_F6-596_702454	0.8025
Listeria_welshimeri_serovar_6b_str_SLCC5334_386043	Listeria_ivanovii_FSL_F6-596_702454	0.8022
Listeria_seeligeri_serovar_1_2b_str_SLCC3954_683837	Listeria_ivanovii_FSL_F6-596_702454	0.8169
Listeria_seeligeri_FSL_S4-171_702452	Listeria_ivanovii_FSL_F6-596_702454	0.7857
Listeria_seeligeri_FSL_N1-067_702453	Listeria_ivanovii_FSL_F6-596_702454	0.7872
Listeria_seeligeri_1640_v1	Listeria_ivanovii_FSL_F6-596_702454	0.828
Listeria_seeligeri_1640	Listeria_ivanovii_FSL_F6-596_702454	0.815
Listeria_monocytogenes_WSLC1042_1457188	Listeria_ivanovii_FSL_F6-596_702454	0.8209
Listeria_monocytogenes_WSLC1001_1457187	Listeria_ivanovii_FSL_F6-596_702454	0.8117
Listeria_monocytogenes_SLCC7179_879090	Listeria_ivanovii_FSL_F6-596_702454	0.8189
Listeria_monocytogenes_SLCC5850_882096	Listeria_ivanovii_FSL_F6-596_702454	0.8162
Listeria_monocytogenes_SLCC2755_932919	Listeria_ivanovii_FSL_F6-596_702454	0.8139
Listeria_monocytogenes_SLCC2540_879089	Listeria_ivanovii_FSL_F6-596_702454	0.8147
Listeria_monocytogenes_SLCC2479_882020	Listeria_ivanovii_FSL_F6-596_702454	0.8149
Listeria_monocytogenes_SLCC2378_879088	Listeria_ivanovii_FSL_F6-596_702454	0.8219
Listeria_monocytogenes_SLCC2376_882097	Listeria_ivanovii_FSL_F6-596_702454	0.8233
Listeria_monocytogenes_SLCC2372_932920	Listeria_ivanovii_FSL_F6-596_702454	0.8087
Listeria_monocytogenes_serotype_4b_str_LL195_1230340	Listeria_ivanovii_FSL_F6-596_702454	0.827
Listeria_monocytogenes_serotype_4b_str_LIS0087_1134669	Listeria_ivanovii_FSL_F6-596_702454	0.7945
Listeria_monocytogenes_serotype_4b_str_F2365_265669	Listeria_ivanovii_FSL_F6-596_702454	0.827
Listeria_monocytogenes_serotype_4b_str_CLIP_80459_568819	Listeria_ivanovii_FSL_F6-596_702454	0.8275
Listeria_monocytogenes_serotype_4b_str_81-0861_1196161	Listeria_ivanovii_FSL_F6-596_702454	0.8147
Listeria_monocytogenes_serotype_4b_str_81-0592_1196160	Listeria_ivanovii_FSL_F6-596_702454	0.8157
Listeria_monocytogenes_serotype_4b_str_81-0558_1196159	Listeria_ivanovii_FSL_F6-596_702454	0.8164
Listeria_monocytogenes_serotype_4b_str_10-0809_1196162	Listeria_ivanovii_FSL_F6-596_702454	0.8161
Listeria_monocytogenes_serotype_4b_str_02-6680_1196175	Listeria_ivanovii_FSL_F6-596_702454	0.8279
Listeria_monocytogenes_serotype_4b_str_02-6679_1196174	Listeria_ivanovii_FSL_F6-596_702454	0.8279
Listeria_monocytogenes_serotype_4b_str_02-1792_1196173	Listeria_ivanovii_FSL_F6-596_702454	0.8164
Listeria_monocytogenes_serotype_4b_str_02-1289_1196172	Listeria_ivanovii_FSL_F6-596_702454	0.8164
Listeria_monocytogenes_serotype_4b_str_02-1103_1196171	Listeria_ivanovii_FSL_F6-596_702454	0.8164
Listeria_monocytogenes_serotype_3c_str_10-5027_1196195	Listeria_ivanovii_FSL_F6-596_702454	0.8144
Listeria_monocytogenes_serotype_1_2c_str_10-5026_1196194	Listeria_ivanovii_FSL_F6-596_702454	0.8153
Listeria_monocytogenes_serotype_1_2c_str_10-5025_1196193	Listeria_ivanovii_FSL_F6-596_702454	0.8153
Listeria_monocytogenes_serotype_1_2b_str_10-0811_1196164	Listeria_ivanovii_FSL_F6-596_702454	0.8065
Listeria_monocytogenes_serotype_1_2b_str_10-0810_1196163	Listeria_ivanovii_FSL_F6-596_702454	0.8065
Listeria_monocytogenes_serotype_1_2a_str_99-6370_1196179	Listeria_ivanovii_FSL_F6-596_702454	0.803
Listeria_monocytogenes_serotype_1_2a_str_98-2035_1196178	Listeria_ivanovii_FSL_F6-596_702454	0.803
Listeria_monocytogenes_serotype_1_2a_str_95-0093_1196177	Listeria_ivanovii_FSL_F6-596_702454	0.8081
Listeria_monocytogenes_serotype_1_2a_str_88-0478_1196176	Listeria_ivanovii_FSL_F6-596_702454	0.8065
Listeria_monocytogenes_serotype_1_2a_str_10-5024_1196192	Listeria_ivanovii_FSL_F6-596_702454	0.8081
Listeria_monocytogenes_serotype_1_2a_str_10-4758_1196168	Listeria_ivanovii_FSL_F6-596_702454	0.8138
Listeria_monocytogenes_serotype_1_2a_str_10-4754_1196167	Listeria_ivanovii_FSL_F6-596_702454	0.8141
Listeria_monocytogenes_serotype_1_2a_str_10-1321_1196191	Listeria_ivanovii_FSL_F6-596_702454	0.8081
Listeria_monocytogenes_serotype_1_2a_str_10-1047_1196190	Listeria_ivanovii_FSL_F6-596_702454	0.8084
Listeria_monocytogenes_serotype_1_2a_str_10-1046_1196189	Listeria_ivanovii_FSL_F6-596_702454	0.8084
Listeria_monocytogenes_serotype_1_2a_str_10-0934_1196170	Listeria_ivanovii_FSL_F6-596_702454	0.8238
Listeria_monocytogenes_serotype_1_2a_str_10-0933_1196169	Listeria_ivanovii_FSL_F6-596_702454	0.8238
Listeria_monocytogenes_serotype_1_2a_str_10-0815_1196188	Listeria_ivanovii_FSL_F6-596_702454	0.8042
Listeria_monocytogenes_serotype_1_2a_str_10-0814_1196187	Listeria_ivanovii_FSL_F6-596_702454	0.8081
Listeria_monocytogenes_serotype_1_2a_str_10-0813_1196166	Listeria_ivanovii_FSL_F6-596_702454	0.8107
Listeria_monocytogenes_serotype_1_2a_str_10-0812_1196165	Listeria_ivanovii_FSL_F6-596_702454	0.8107
Listeria_monocytogenes_serotype_1_2a_str_08-7669_1196186	Listeria_ivanovii_FSL_F6-596_702454	0.8153
Listeria_monocytogenes_serotype_1_2a_str_08-7374_1196185	Listeria_ivanovii_FSL_F6-596_702454	0.8081
Listeria_monocytogenes_serotype_1_2a_str_08-6997_1196184	Listeria_ivanovii_FSL_F6-596_702454	0.8042
Listeria_monocytogenes_serotype_1_2a_str_08-6569_1196183	Listeria_ivanovii_FSL_F6-596_702454	0.8042
Listeria_monocytogenes_serotype_1_2a_str_08-6056_1196182	Listeria_ivanovii_FSL_F6-596_702454	0.8042
Listeria_monocytogenes_serotype_1_2a_str_04-5457_1196181	Listeria_ivanovii_FSL_F6-596_702454	0.8081
Listeria_monocytogenes_serotype_1_2a_str_02-5993_1196180	Listeria_ivanovii_FSL_F6-596_702454	0.8081
Listeria_monocytogenes_R479a_1437838	Listeria_ivanovii_FSL_F6-596_702454	0.8079
Listeria_monocytogenes_N53-1_1234142	Listeria_ivanovii_FSL_F6-596_702454	0.783
Listeria_monocytogenes_M7_1030009	Listeria_ivanovii_FSL_F6-596_702454	0.7963
Listeria_monocytogenes_LO28_393132	Listeria_ivanovii_FSL_F6-596_702454	0.8106
Listeria_monocytogenes_L99_563174	Listeria_ivanovii_FSL_F6-596_702454	0.7962
Listeria_monocytogenes_L312_882094	Listeria_ivanovii_FSL_F6-596_702454	0.8275
Listeria_monocytogenes_J1816_930781	Listeria_ivanovii_FSL_F6-596_702454	0.8184
Listeria_monocytogenes_J1-220_930782	Listeria_ivanovii_FSL_F6-596_702454	0.8084
Listeria_monocytogenes_J0161_393130	Listeria_ivanovii_FSL_F6-596_702454	0.8182
Listeria_monocytogenes_HCC23_552536	Listeria_ivanovii_FSL_F6-596_702454	0.7959
Listeria_monocytogenes_FSL_R2-561_393126	Listeria_ivanovii_FSL_F6-596_702454	0.8132
Listeria_monocytogenes_FSL_J1-208_393119	Listeria_ivanovii_FSL_F6-596_702454	0.8071
Listeria_monocytogenes_FSL_J1-175_393118	Listeria_ivanovii_FSL_F6-596_702454	0.8174
Listeria_monocytogenes_Finland_1998_393127	Listeria_ivanovii_FSL_F6-596_702454	0.8272
Listeria_monocytogenes_EGD-e_169963_v1	Listeria_ivanovii_FSL_F6-596_702454	0.8152
Listeria_monocytogenes_EGD-e_169963	Listeria_ivanovii_FSL_F6-596_702454	0.8152
Listeria_monocytogenes_EGD_1334565	Listeria_ivanovii_FSL_F6-596_702454	0.8165
Listeria_monocytogenes_CFSAN002349_1299895	Listeria_ivanovii_FSL_F6-596_702454	0.8281
Listeria_monocytogenes_ATCC_19117_882095_v1	Listeria_ivanovii_FSL_F6-596_702454	0.8233
Listeria_monocytogenes_ATCC_19117_882095	Listeria_ivanovii_FSL_F6-596_702454	0.8233
Listeria_monocytogenes_6179_1288295	Listeria_ivanovii_FSL_F6-596_702454	0.8061
Listeria_monocytogenes_1639_v9	Listeria_ivanovii_FSL_F6-596_702454	0.8006
Listeria_monocytogenes_1639_v99	Listeria_ivanovii_FSL_F6-596_702454	0.8196
Listeria_monocytogenes_1639_v98	Listeria_ivanovii_FSL_F6-596_702454	0.8138
Listeria_monocytogenes_1639_v97	Listeria_ivanovii_FSL_F6-596_702454	0.8273
Listeria_monocytogenes_1639_v96	Listeria_ivanovii_FSL_F6-596_702454	0.8186
Listeria_monocytogenes_1639_v95	Listeria_ivanovii_FSL_F6-596_702454	0.8186
Listeria_monocytogenes_1639_v94	Listeria_ivanovii_FSL_F6-596_702454	0.8193
Listeria_monocytogenes_1639_v93	Listeria_ivanovii_FSL_F6-596_702454	0.8038
Listeria_monocytogenes_1639_v92	Listeria_ivanovii_FSL_F6-596_702454	0.82

Listeria_monocytogenes_1639_v91	Listeria_ivanovii_FSL_F6-596_702454	0.8136
Listeria_monocytogenes_1639_v90	Listeria_ivanovii_FSL_F6-596_702454	0.8138
Listeria_monocytogenes_1639_v8	Listeria_ivanovii_FSL_F6-596_702454	0.7985
Listeria_monocytogenes_1639_v89	Listeria_ivanovii_FSL_F6-596_702454	0.8246
Listeria_monocytogenes_1639_v88	Listeria_ivanovii_FSL_F6-596_702454	0.8156
Listeria_monocytogenes_1639_v87	Listeria_ivanovii_FSL_F6-596_702454	0.8071
Listeria_monocytogenes_1639_v86	Listeria_ivanovii_FSL_F6-596_702454	0.8071
Listeria_monocytogenes_1639_v85	Listeria_ivanovii_FSL_F6-596_702454	0.8141
Listeria_monocytogenes_1639_v84	Listeria_ivanovii_FSL_F6-596_702454	0.8197
Listeria_monocytogenes_1639_v83	Listeria_ivanovii_FSL_F6-596_702454	0.8158
Listeria_monocytogenes_1639_v82	Listeria_ivanovii_FSL_F6-596_702454	0.8155
Listeria_monocytogenes_1639_v81	Listeria_ivanovii_FSL_F6-596_702454	0.819
Listeria_monocytogenes_1639_v80	Listeria_ivanovii_FSL_F6-596_702454	0.8109
Listeria_monocytogenes_1639_v7	Listeria_ivanovii_FSL_F6-596_702454	0.7985
Listeria_monocytogenes_1639_v79	Listeria_ivanovii_FSL_F6-596_702454	0.817
Listeria_monocytogenes_1639_v78	Listeria_ivanovii_FSL_F6-596_702454	0.818
Listeria_monocytogenes_1639_v77	Listeria_ivanovii_FSL_F6-596_702454	0.8211
Listeria_monocytogenes_1639_v76	Listeria_ivanovii_FSL_F6-596_702454	0.809
Listeria_monocytogenes_1639_v75	Listeria_ivanovii_FSL_F6-596_702454	0.7962
Listeria_monocytogenes_1639_v74	Listeria_ivanovii_FSL_F6-596_702454	0.806
Listeria_monocytogenes_1639_v73	Listeria_ivanovii_FSL_F6-596_702454	0.8055
Listeria_monocytogenes_1639_v72	Listeria_ivanovii_FSL_F6-596_702454	0.8149
Listeria_monocytogenes_1639_v71	Listeria_ivanovii_FSL_F6-596_702454	0.8071
Listeria_monocytogenes_1639_v70	Listeria_ivanovii_FSL_F6-596_702454	0.8137
Listeria_monocytogenes_1639_v6	Listeria_ivanovii_FSL_F6-596_702454	0.7986
Listeria_monocytogenes_1639_v69	Listeria_ivanovii_FSL_F6-596_702454	0.8157
Listeria_monocytogenes_1639_v68	Listeria_ivanovii_FSL_F6-596_702454	0.8155
Listeria_monocytogenes_1639_v67	Listeria_ivanovii_FSL_F6-596_702454	0.8186
Listeria_monocytogenes_1639_v66	Listeria_ivanovii_FSL_F6-596_702454	0.8128
Listeria_monocytogenes_1639_v65	Listeria_ivanovii_FSL_F6-596_702454	0.8053
Listeria_monocytogenes_1639_v64	Listeria_ivanovii_FSL_F6-596_702454	0.8089
Listeria_monocytogenes_1639_v63	Listeria_ivanovii_FSL_F6-596_702454	0.8135
Listeria_monocytogenes_1639_v62	Listeria_ivanovii_FSL_F6-596_702454	0.816
Listeria_monocytogenes_1639_v61	Listeria_ivanovii_FSL_F6-596_702454	0.8102
Listeria_monocytogenes_1639_v60	Listeria_ivanovii_FSL_F6-596_702454	0.8193
Listeria_monocytogenes_1639_v5	Listeria_ivanovii_FSL_F6-596_702454	0.7779
Listeria_monocytogenes_1639_v59	Listeria_ivanovii_FSL_F6-596_702454	0.8149
Listeria_monocytogenes_1639_v58	Listeria_ivanovii_FSL_F6-596_702454	0.8165
Listeria_monocytogenes_1639_v57	Listeria_ivanovii_FSL_F6-596_702454	0.8155
Listeria_monocytogenes_1639_v56	Listeria_ivanovii_FSL_F6-596_702454	0.8234
Listeria_monocytogenes_1639_v55	Listeria_ivanovii_FSL_F6-596_702454	0.8139
Listeria_monocytogenes_1639_v54	Listeria_ivanovii_FSL_F6-596_702454	0.8291
Listeria_monocytogenes_1639_v53	Listeria_ivanovii_FSL_F6-596_702454	0.81
Listeria_monocytogenes_1639_v52	Listeria_ivanovii_FSL_F6-596_702454	0.8133
Listeria_monocytogenes_1639_v51	Listeria_ivanovii_FSL_F6-596_702454	0.8115
Listeria_monocytogenes_1639_v50	Listeria_ivanovii_FSL_F6-596_702454	0.807
Listeria_monocytogenes_1639_v4	Listeria_ivanovii_FSL_F6-596_702454	0.7894
Listeria_monocytogenes_1639_v49	Listeria_ivanovii_FSL_F6-596_702454	0.815
Listeria_monocytogenes_1639_v48	Listeria_ivanovii_FSL_F6-596_702454	0.8107
Listeria_monocytogenes_1639_v47	Listeria_ivanovii_FSL_F6-596_702454	0.8075
Listeria_monocytogenes_1639_v46	Listeria_ivanovii_FSL_F6-596_702454	0.8221
Listeria_monocytogenes_1639_v45	Listeria_ivanovii_FSL_F6-596_702454	0.8004
Listeria_monocytogenes_1639_v44	Listeria_ivanovii_FSL_F6-596_702454	0.8081
Listeria_monocytogenes_1639_v43	Listeria_ivanovii_FSL_F6-596_702454	0.8081
Listeria_monocytogenes_1639_v42	Listeria_ivanovii_FSL_F6-596_702454	0.8062
Listeria_monocytogenes_1639_v41	Listeria_ivanovii_FSL_F6-596_702454	0.7902
Listeria_monocytogenes_1639_v40	Listeria_ivanovii_FSL_F6-596_702454	0.8086
Listeria_monocytogenes_1639_v3	Listeria_ivanovii_FSL_F6-596_702454	0.7898
Listeria_monocytogenes_1639_v39	Listeria_ivanovii_FSL_F6-596_702454	0.8145
Listeria_monocytogenes_1639_v38	Listeria_ivanovii_FSL_F6-596_702454	0.805
Listeria_monocytogenes_1639_v37	Listeria_ivanovii_FSL_F6-596_702454	0.8004
Listeria_monocytogenes_1639_v36	Listeria_ivanovii_FSL_F6-596_702454	0.8018
Listeria_monocytogenes_1639_v35	Listeria_ivanovii_FSL_F6-596_702454	0.8095
Listeria_monocytogenes_1639_v34	Listeria_ivanovii_FSL_F6-596_702454	0.8155
Listeria_monocytogenes_1639_v33	Listeria_ivanovii_FSL_F6-596_702454	0.8065
Listeria_monocytogenes_1639_v32	Listeria_ivanovii_FSL_F6-596_702454	0.8002
Listeria_monocytogenes_1639_v31	Listeria_ivanovii_FSL_F6-596_702454	0.8155
Listeria_monocytogenes_1639_v30	Listeria_ivanovii_FSL_F6-596_702454	0.8066
Listeria_monocytogenes_1639_v2	Listeria_ivanovii_FSL_F6-596_702454	0.7878
Listeria_monocytogenes_1639_v29	Listeria_ivanovii_FSL_F6-596_702454	0.8035
Listeria_monocytogenes_1639_v28	Listeria_ivanovii_FSL_F6-596_702454	0.8085
Listeria_monocytogenes_1639_v27	Listeria_ivanovii_FSL_F6-596_702454	0.7944
Listeria_monocytogenes_1639_v26	Listeria_ivanovii_FSL_F6-596_702454	0.7938
Listeria_monocytogenes_1639_v25	Listeria_ivanovii_FSL_F6-596_702454	0.7942
Listeria_monocytogenes_1639_v24	Listeria_ivanovii_FSL_F6-596_702454	0.8095
Listeria_monocytogenes_1639_v23	Listeria_ivanovii_FSL_F6-596_702454	0.8092
Listeria_monocytogenes_1639_v22	Listeria_ivanovii_FSL_F6-596_702454	0.8139
Listeria_monocytogenes_1639_v21	Listeria_ivanovii_FSL_F6-596_702454	0.8092
Listeria_monocytogenes_1639_v20	Listeria_ivanovii_FSL_F6-596_702454	0.8149
Listeria_monocytogenes_1639_v1	Listeria_ivanovii_FSL_F6-596_702454	0.801
Listeria_monocytogenes_1639_v19	Listeria_ivanovii_FSL_F6-596_702454	0.8146
Listeria_monocytogenes_1639_v198	Listeria_ivanovii_FSL_F6-596_702454	0.7865
Listeria_monocytogenes_1639_v197	Listeria_ivanovii_FSL_F6-596_702454	0.8029
Listeria_monocytogenes_1639_v196	Listeria_ivanovii_FSL_F6-596_702454	0.8008
Listeria_monocytogenes_1639_v195	Listeria_ivanovii_FSL_F6-596_702454	0.8247
Listeria_monocytogenes_1639_v194	Listeria_ivanovii_FSL_F6-596_702454	0.8242
Listeria_monocytogenes_1639_v193	Listeria_ivanovii_FSL_F6-596_702454	0.8234
Listeria_monocytogenes_1639_v192	Listeria_ivanovii_FSL_F6-596_702454	0.8019
Listeria_monocytogenes_1639_v191	Listeria_ivanovii_FSL_F6-596_702454	0.8264
Listeria_monocytogenes_1639_v190	Listeria_ivanovii_FSL_F6-596_702454	0.8251
Listeria_monocytogenes_1639_v18	Listeria_ivanovii_FSL_F6-596_702454	0.8114

Listeria_monocytogenes_1639_v189	Listeria_ivanovii_FSL_F6-596_702454	0.8248
Listeria_monocytogenes_1639_v188	Listeria_ivanovii_FSL_F6-596_702454	0.8255
Listeria_monocytogenes_1639_v187	Listeria_ivanovii_FSL_F6-596_702454	0.8254
Listeria_monocytogenes_1639_v186	Listeria_ivanovii_FSL_F6-596_702454	0.8254
Listeria_monocytogenes_1639_v185	Listeria_ivanovii_FSL_F6-596_702454	0.8254
Listeria_monocytogenes_1639_v184	Listeria_ivanovii_FSL_F6-596_702454	0.8246
Listeria_monocytogenes_1639_v183	Listeria_ivanovii_FSL_F6-596_702454	0.825
Listeria_monocytogenes_1639_v182	Listeria_ivanovii_FSL_F6-596_702454	0.8258
Listeria_monocytogenes_1639_v181	Listeria_ivanovii_FSL_F6-596_702454	0.811
Listeria_monocytogenes_1639_v180	Listeria_ivanovii_FSL_F6-596_702454	0.811
Listeria_monocytogenes_1639_v17	Listeria_ivanovii_FSL_F6-596_702454	0.8117
Listeria_monocytogenes_1639_v179	Listeria_ivanovii_FSL_F6-596_702454	0.8202
Listeria_monocytogenes_1639_v178	Listeria_ivanovii_FSL_F6-596_702454	0.8247
Listeria_monocytogenes_1639_v177	Listeria_ivanovii_FSL_F6-596_702454	0.8257
Listeria_monocytogenes_1639_v176	Listeria_ivanovii_FSL_F6-596_702454	0.8209
Listeria_monocytogenes_1639_v175	Listeria_ivanovii_FSL_F6-596_702454	0.817
Listeria_monocytogenes_1639_v174	Listeria_ivanovii_FSL_F6-596_702454	0.8265
Listeria_monocytogenes_1639_v173	Listeria_ivanovii_FSL_F6-596_702454	0.8277
Listeria_monocytogenes_1639_v172	Listeria_ivanovii_FSL_F6-596_702454	0.8192
Listeria_monocytogenes_1639_v171	Listeria_ivanovii_FSL_F6-596_702454	0.8139
Listeria_monocytogenes_1639_v170	Listeria_ivanovii_FSL_F6-596_702454	0.8201
Listeria_monocytogenes_1639_v16	Listeria_ivanovii_FSL_F6-596_702454	0.7965
Listeria_monocytogenes_1639_v169	Listeria_ivanovii_FSL_F6-596_702454	0.8218
Listeria_monocytogenes_1639_v168	Listeria_ivanovii_FSL_F6-596_702454	0.8216
Listeria_monocytogenes_1639_v167	Listeria_ivanovii_FSL_F6-596_702454	0.8255
Listeria_monocytogenes_1639_v166	Listeria_ivanovii_FSL_F6-596_702454	0.822
Listeria_monocytogenes_1639_v165	Listeria_ivanovii_FSL_F6-596_702454	0.8215
Listeria_monocytogenes_1639_v164	Listeria_ivanovii_FSL_F6-596_702454	0.8263
Listeria_monocytogenes_1639_v163	Listeria_ivanovii_FSL_F6-596_702454	0.823
Listeria_monocytogenes_1639_v162	Listeria_ivanovii_FSL_F6-596_702454	0.8263
Listeria_monocytogenes_1639_v161	Listeria_ivanovii_FSL_F6-596_702454	0.8279
Listeria_monocytogenes_1639_v160	Listeria_ivanovii_FSL_F6-596_702454	0.8277
Listeria_monocytogenes_1639_v15	Listeria_ivanovii_FSL_F6-596_702454	0.7944
Listeria_monocytogenes_1639_v159	Listeria_ivanovii_FSL_F6-596_702454	0.8257
Listeria_monocytogenes_1639_v158	Listeria_ivanovii_FSL_F6-596_702454	0.827
Listeria_monocytogenes_1639_v157	Listeria_ivanovii_FSL_F6-596_702454	0.827
Listeria_monocytogenes_1639_v156	Listeria_ivanovii_FSL_F6-596_702454	0.8067
Listeria_monocytogenes_1639_v155	Listeria_ivanovii_FSL_F6-596_702454	0.8235
Listeria_monocytogenes_1639_v154	Listeria_ivanovii_FSL_F6-596_702454	0.8166
Listeria_monocytogenes_1639_v153	Listeria_ivanovii_FSL_F6-596_702454	0.818
Listeria_monocytogenes_1639_v152	Listeria_ivanovii_FSL_F6-596_702454	0.8257
Listeria_monocytogenes_1639_v151	Listeria_ivanovii_FSL_F6-596_702454	0.8257
Listeria_monocytogenes_1639_v150	Listeria_ivanovii_FSL_F6-596_702454	0.8176
Listeria_monocytogenes_1639_v14	Listeria_ivanovii_FSL_F6-596_702454	0.7981
Listeria_monocytogenes_1639_v149	Listeria_ivanovii_FSL_F6-596_702454	0.8168
Listeria_monocytogenes_1639_v148	Listeria_ivanovii_FSL_F6-596_702454	0.8246
Listeria_monocytogenes_1639_v147	Listeria_ivanovii_FSL_F6-596_702454	0.8131
Listeria_monocytogenes_1639_v146	Listeria_ivanovii_FSL_F6-596_702454	0.8272
Listeria_monocytogenes_1639_v145	Listeria_ivanovii_FSL_F6-596_702454	0.8221
Listeria_monocytogenes_1639_v144	Listeria_ivanovii_FSL_F6-596_702454	0.8272
Listeria_monocytogenes_1639_v143	Listeria_ivanovii_FSL_F6-596_702454	0.8231
Listeria_monocytogenes_1639_v142	Listeria_ivanovii_FSL_F6-596_702454	0.8276
Listeria_monocytogenes_1639_v141	Listeria_ivanovii_FSL_F6-596_702454	0.8254
Listeria_monocytogenes_1639_v140	Listeria_ivanovii_FSL_F6-596_702454	0.8279
Listeria_monocytogenes_1639_v13	Listeria_ivanovii_FSL_F6-596_702454	0.7947
Listeria_monocytogenes_1639_v139	Listeria_ivanovii_FSL_F6-596_702454	0.8246
Listeria_monocytogenes_1639_v138	Listeria_ivanovii_FSL_F6-596_702454	0.8276
Listeria_monocytogenes_1639_v137	Listeria_ivanovii_FSL_F6-596_702454	0.8268
Listeria_monocytogenes_1639_v136	Listeria_ivanovii_FSL_F6-596_702454	0.8135
Listeria_monocytogenes_1639_v135	Listeria_ivanovii_FSL_F6-596_702454	0.8053
Listeria_monocytogenes_1639_v134	Listeria_ivanovii_FSL_F6-596_702454	0.8222
Listeria_monocytogenes_1639_v133	Listeria_ivanovii_FSL_F6-596_702454	0.8283
Listeria_monocytogenes_1639_v132	Listeria_ivanovii_FSL_F6-596_702454	0.8034
Listeria_monocytogenes_1639_v131	Listeria_ivanovii_FSL_F6-596_702454	0.817
Listeria_monocytogenes_1639_v130	Listeria_ivanovii_FSL_F6-596_702454	0.8088
Listeria_monocytogenes_1639_v12	Listeria_ivanovii_FSL_F6-596_702454	0.797
Listeria_monocytogenes_1639_v129	Listeria_ivanovii_FSL_F6-596_702454	0.8221
Listeria_monocytogenes_1639_v128	Listeria_ivanovii_FSL_F6-596_702454	0.8222
Listeria_monocytogenes_1639_v127	Listeria_ivanovii_FSL_F6-596_702454	0.8159
Listeria_monocytogenes_1639_v126	Listeria_ivanovii_FSL_F6-596_702454	0.8211
Listeria_monocytogenes_1639_v125	Listeria_ivanovii_FSL_F6-596_702454	0.8225
Listeria_monocytogenes_1639_v124	Listeria_ivanovii_FSL_F6-596_702454	0.8153
Listeria_monocytogenes_1639_v123	Listeria_ivanovii_FSL_F6-596_702454	0.8281
Listeria_monocytogenes_1639_v122	Listeria_ivanovii_FSL_F6-596_702454	0.8281
Listeria_monocytogenes_1639_v121	Listeria_ivanovii_FSL_F6-596_702454	0.8281
Listeria_monocytogenes_1639_v120	Listeria_ivanovii_FSL_F6-596_702454	0.8285
Listeria_monocytogenes_1639_v11	Listeria_ivanovii_FSL_F6-596_702454	0.803
Listeria_monocytogenes_1639_v119	Listeria_ivanovii_FSL_F6-596_702454	0.8281
Listeria_monocytogenes_1639_v118	Listeria_ivanovii_FSL_F6-596_702454	0.8281
Listeria_monocytogenes_1639_v117	Listeria_ivanovii_FSL_F6-596_702454	0.8142
Listeria_monocytogenes_1639_v116	Listeria_ivanovii_FSL_F6-596_702454	0.8216
Listeria_monocytogenes_1639_v115	Listeria_ivanovii_FSL_F6-596_702454	0.8219
Listeria_monocytogenes_1639_v114	Listeria_ivanovii_FSL_F6-596_702454	0.817
Listeria_monocytogenes_1639_v113	Listeria_ivanovii_FSL_F6-596_702454	0.8179
Listeria_monocytogenes_1639_v112	Listeria_ivanovii_FSL_F6-596_702454	0.8281
Listeria_monocytogenes_1639_v111	Listeria_ivanovii_FSL_F6-596_702454	0.8174
Listeria_monocytogenes_1639_v110	Listeria_ivanovii_FSL_F6-596_702454	0.8106
Listeria_monocytogenes_1639_v10	Listeria_ivanovii_FSL_F6-596_702454	0.798
Listeria_monocytogenes_1639_v109	Listeria_ivanovii_FSL_F6-596_702454	0.8179
Listeria_monocytogenes_1639_v108	Listeria_ivanovii_FSL_F6-596_702454	0.8181
Listeria_monocytogenes_1639_v107	Listeria_ivanovii_FSL_F6-596_702454	0.8223

Listeria_monocytogenes_1639_v106	Listeria_ivanovii_FSL_F6-596_702454	0.8223
Listeria_monocytogenes_1639_v105	Listeria_ivanovii_FSL_F6-596_702454	0.8227
Listeria_monocytogenes_1639_v104	Listeria_ivanovii_FSL_F6-596_702454	0.8223
Listeria_monocytogenes_1639_v103	Listeria_ivanovii_FSL_F6-596_702454	0.8226
Listeria_monocytogenes_1639_v102	Listeria_ivanovii_FSL_F6-596_702454	0.8174
Listeria_monocytogenes_1639_v101	Listeria_ivanovii_FSL_F6-596_702454	0.8076
Listeria_monocytogenes_1639_v100	Listeria_ivanovii_FSL_F6-596_702454	0.807
Listeria_monocytogenes_1639	Listeria_ivanovii_FSL_F6-596_702454	0.8251
Listeria_monocytogenes_10403S_393133	Listeria_ivanovii_FSL_F6-596_702454	0.8191
Listeria_monocytogenes_08-5923_637381	Listeria_ivanovii_FSL_F6-596_702454	0.8081
Listeria_monocytogenes_08-5578_653938	Listeria_ivanovii_FSL_F6-596_702454	0.7965
Listeria_monocytogenes_07PF0776_1126011	Listeria_ivanovii_FSL_F6-596_702454	0.827
Listeria_marthii_FSL_S4-120_702457	Listeria_ivanovii_FSL_F6-596_702454	0.7675
Listeria_marthii_529731_v1	Listeria_ivanovii_FSL_F6-596_702454	0.7941
Listeria_marthii_529731	Listeria_ivanovii_FSL_F6-596_702454	0.7989
Listeria_ivanovii_WSLC3009_1457190	Listeria_ivanovii_FSL_F6-596_702454	0.8331
Listeria_ivanovii_subsp_londoniensis_202752_v2	Listeria_ivanovii_FSL_F6-596_702454	0.9073
Listeria_ivanovii_subsp_londoniensis_202752_v1	Listeria_ivanovii_FSL_F6-596_702454	0.8719
Listeria_ivanovii_subsp_londoniensis_202752	Listeria_ivanovii_FSL_F6-596_702454	0.9234
Listeria_ivanovii_subsp_ivanovii_PAM_55_881621	Listeria_ivanovii_FSL_F6-596_702454	0.8326
Listeria_ivanovii_subsp_ivanovii_202751_v2	Listeria_ivanovii_FSL_F6-596_702454	0.8334
Listeria_ivanovii_subsp_ivanovii_202751_v1	Listeria_ivanovii_FSL_F6-596_702454	0.8334
Listeria_ivanovii_subsp_ivanovii_202751	Listeria_ivanovii_FSL_F6-596_702454	0.8334
Listeria_ivanovii_FSL_F6-596_702454	Listeria_ivanovii_FSL_F6-596_702454	0.0514
Listeria_welshimeri_1643	Listeria_ivanovii_subsp_ivanovii_202751	0.8618
Listeria_welshimeri_serovar_6b_str_SLCC5334_386043	Listeria_ivanovii_subsp_ivanovii_202751	0.8612
Listeria_seeligeri_serovar_1_2b_str_SLCC3954_683837	Listeria_ivanovii_subsp_ivanovii_202751	0.8826
Listeria_seeligeri_FSL_S4-171_702452	Listeria_ivanovii_subsp_ivanovii_202751	0.8191
Listeria_seeligeri_FSL_N1-067_702453	Listeria_ivanovii_subsp_ivanovii_202751	0.8253
Listeria_seeligeri_1640_v1	Listeria_ivanovii_subsp_ivanovii_202751	0.891
Listeria_seeligeri_1640	Listeria_ivanovii_subsp_ivanovii_202751	0.8765
Listeria_monocytogenes_WSLC1042_1457188	Listeria_ivanovii_subsp_ivanovii_202751	0.8983
Listeria_monocytogenes_WSLC1001_1457187	Listeria_ivanovii_subsp_ivanovii_202751	0.8815
Listeria_monocytogenes_SLCC7179_879090	Listeria_ivanovii_subsp_ivanovii_202751	0.8889
Listeria_monocytogenes_SLCC5850_882096	Listeria_ivanovii_subsp_ivanovii_202751	0.8863
Listeria_monocytogenes_SLCC2755_932919	Listeria_ivanovii_subsp_ivanovii_202751	0.888
Listeria_monocytogenes_SLCC2540_879089	Listeria_ivanovii_subsp_ivanovii_202751	0.8906
Listeria_monocytogenes_SLCC2479_882020	Listeria_ivanovii_subsp_ivanovii_202751	0.8797
Listeria_monocytogenes_SLCC2378_879088	Listeria_ivanovii_subsp_ivanovii_202751	0.8997
Listeria_monocytogenes_SLCC2376_882097	Listeria_ivanovii_subsp_ivanovii_202751	0.8975
Listeria_monocytogenes_SLCC2372_932920	Listeria_ivanovii_subsp_ivanovii_202751	0.8724
Listeria_monocytogenes_serotype_4b_str_LL195_1230340	Listeria_ivanovii_subsp_ivanovii_202751	0.9056
Listeria_monocytogenes_serotype_4b_str_LIS0087_1134669	Listeria_ivanovii_subsp_ivanovii_202751	0.8622
Listeria_monocytogenes_serotype_4b_str_F2365_265669	Listeria_ivanovii_subsp_ivanovii_202751	0.9056
Listeria_monocytogenes_serotype_4b_str_CLIP_80459_568819	Listeria_ivanovii_subsp_ivanovii_202751	0.9043
Listeria_monocytogenes_serotype_4b_str_81-0861_1196161	Listeria_ivanovii_subsp_ivanovii_202751	0.8887
Listeria_monocytogenes_serotype_4b_str_81-0592_1196160	Listeria_ivanovii_subsp_ivanovii_202751	0.8897
Listeria_monocytogenes_serotype_4b_str_81-0558_1196159	Listeria_ivanovii_subsp_ivanovii_202751	0.8908
Listeria_monocytogenes_serotype_4b_str_10-0809_1196162	Listeria_ivanovii_subsp_ivanovii_202751	0.8901
Listeria_monocytogenes_serotype_4b_str_02-6680_1196175	Listeria_ivanovii_subsp_ivanovii_202751	0.9026
Listeria_monocytogenes_serotype_4b_str_02-6679_1196174	Listeria_ivanovii_subsp_ivanovii_202751	0.9026
Listeria_monocytogenes_serotype_4b_str_02-1792_1196173	Listeria_ivanovii_subsp_ivanovii_202751	0.8904
Listeria_monocytogenes_serotype_4b_str_02-1289_1196172	Listeria_ivanovii_subsp_ivanovii_202751	0.8904
Listeria_monocytogenes_serotype_4b_str_02-1103_1196171	Listeria_ivanovii_subsp_ivanovii_202751	0.8904
Listeria_monocytogenes_serotype_3c_str_10-5027_1196195	Listeria_ivanovii_subsp_ivanovii_202751	0.8785
Listeria_monocytogenes_serotype_1_2c_str_10-5026_1196194	Listeria_ivanovii_subsp_ivanovii_202751	0.8797
Listeria_monocytogenes_serotype_1_2c_str_10-5025_1196193	Listeria_ivanovii_subsp_ivanovii_202751	0.8797
Listeria_monocytogenes_serotype_1_2b_str_10-0811_1196164	Listeria_ivanovii_subsp_ivanovii_202751	0.8795
Listeria_monocytogenes_serotype_1_2b_str_10-0810_1196163	Listeria_ivanovii_subsp_ivanovii_202751	0.8795
Listeria_monocytogenes_serotype_1_2a_str_99-6370_1196179	Listeria_ivanovii_subsp_ivanovii_202751	0.866
Listeria_monocytogenes_serotype_1_2a_str_98-2035_1196178	Listeria_ivanovii_subsp_ivanovii_202751	0.866
Listeria_monocytogenes_serotype_1_2a_str_95-0093_1196177	Listeria_ivanovii_subsp_ivanovii_202751	0.8711
Listeria_monocytogenes_serotype_1_2a_str_88-0478_1196176	Listeria_ivanovii_subsp_ivanovii_202751	0.8703
Listeria_monocytogenes_serotype_1_2a_str_10-5024_1196192	Listeria_ivanovii_subsp_ivanovii_202751	0.8715
Listeria_monocytogenes_serotype_1_2a_str_10-4758_1196168	Listeria_ivanovii_subsp_ivanovii_202751	0.8857
Listeria_monocytogenes_serotype_1_2a_str_10-4754_1196167	Listeria_ivanovii_subsp_ivanovii_202751	0.8857
Listeria_monocytogenes_serotype_1_2a_str_10-1321_1196191	Listeria_ivanovii_subsp_ivanovii_202751	0.8715
Listeria_monocytogenes_serotype_1_2a_str_10-1047_1196190	Listeria_ivanovii_subsp_ivanovii_202751	0.8718
Listeria_monocytogenes_serotype_1_2a_str_10-1046_1196189	Listeria_ivanovii_subsp_ivanovii_202751	0.8718
Listeria_monocytogenes_serotype_1_2a_str_10-0934_1196170	Listeria_ivanovii_subsp_ivanovii_202751	0.8959
Listeria_monocytogenes_serotype_1_2a_str_10-0933_1196169	Listeria_ivanovii_subsp_ivanovii_202751	0.8959
Listeria_monocytogenes_serotype_1_2a_str_10-0815_1196188	Listeria_ivanovii_subsp_ivanovii_202751	0.8673
Listeria_monocytogenes_serotype_1_2a_str_10-0814_1196187	Listeria_ivanovii_subsp_ivanovii_202751	0.8715
Listeria_monocytogenes_serotype_1_2a_str_10-0813_1196166	Listeria_ivanovii_subsp_ivanovii_202751	0.8789
Listeria_monocytogenes_serotype_1_2a_str_10-0812_1196165	Listeria_ivanovii_subsp_ivanovii_202751	0.8789
Listeria_monocytogenes_serotype_1_2a_str_08-7669_1196186	Listeria_ivanovii_subsp_ivanovii_202751	0.8797
Listeria_monocytogenes_serotype_1_2a_str_08-7374_1196185	Listeria_ivanovii_subsp_ivanovii_202751	0.8715
Listeria_monocytogenes_serotype_1_2a_str_08-6997_1196184	Listeria_ivanovii_subsp_ivanovii_202751	0.8673
Listeria_monocytogenes_serotype_1_2a_str_08-6569_1196183	Listeria_ivanovii_subsp_ivanovii_202751	0.8673
Listeria_monocytogenes_serotype_1_2a_str_08-6056_1196182	Listeria_ivanovii_subsp_ivanovii_202751	0.8673
Listeria_monocytogenes_serotype_1_2a_str_04-5457_1196181	Listeria_ivanovii_subsp_ivanovii_202751	0.8715
Listeria_monocytogenes_serotype_1_2a_str_02-5993_1196180	Listeria_ivanovii_subsp_ivanovii_202751	0.8711
Listeria_monocytogenes_R479a_1437838	Listeria_ivanovii_subsp_ivanovii_202751	0.8709
Listeria_monocytogenes_N53-1_1234142	Listeria_ivanovii_subsp_ivanovii_202751	0.8499
Listeria_monocytogenes_M7_1030009	Listeria_ivanovii_subsp_ivanovii_202751	0.8608
Listeria_monocytogenes_LO28_393132	Listeria_ivanovii_subsp_ivanovii_202751	0.8784
Listeria_monocytogenes_L99_563174	Listeria_ivanovii_subsp_ivanovii_202751	0.8615
Listeria_monocytogenes_L312_882094	Listeria_ivanovii_subsp_ivanovii_202751	0.9043
Listeria_monocytogenes_J1816_930781	Listeria_ivanovii_subsp_ivanovii_202751	0.8815
Listeria_monocytogenes_J1-220_930782	Listeria_ivanovii_subsp_ivanovii_202751	0.8789
Listeria_monocytogenes_J0161_393130	Listeria_ivanovii_subsp_ivanovii_202751	0.8784

Listeria_monocytogenes_HCC23_552536	Listeria_ivanovii_subsp_ivanovii_202751	0.8612
Listeria_monocytogenes_FSL_R2-561_393126	Listeria_ivanovii_subsp_ivanovii_202751	0.878
Listeria_monocytogenes_FSL_J1-208_393119	Listeria_ivanovii_subsp_ivanovii_202751	0.8744
Listeria_monocytogenes_FSL_J1-175_393118	Listeria_ivanovii_subsp_ivanovii_202751	0.8948
Listeria_monocytogenes_Finland_1998_393127	Listeria_ivanovii_subsp_ivanovii_202751	0.897
Listeria_monocytogenes_EGD-e_169963_v1	Listeria_ivanovii_subsp_ivanovii_202751	0.8863
Listeria_monocytogenes_EGD-e_169963	Listeria_ivanovii_subsp_ivanovii_202751	0.8867
Listeria_monocytogenes_EGD_1334565	Listeria_ivanovii_subsp_ivanovii_202751	0.8867
Listeria_monocytogenes_CFSAN002349_1299895	Listeria_ivanovii_subsp_ivanovii_202751	0.9
Listeria_monocytogenes_ATCC_19117_882095_v1	Listeria_ivanovii_subsp_ivanovii_202751	0.8978
Listeria_monocytogenes_ATCC_19117_882095	Listeria_ivanovii_subsp_ivanovii_202751	0.8978
Listeria_monocytogenes_6179_1288295	Listeria_ivanovii_subsp_ivanovii_202751	0.8629
Listeria_monocytogenes_1639_v9	Listeria_ivanovii_subsp_ivanovii_202751	0.8674
Listeria_monocytogenes_1639_v99	Listeria_ivanovii_subsp_ivanovii_202751	0.8815
Listeria_monocytogenes_1639_v98	Listeria_ivanovii_subsp_ivanovii_202751	0.8771
Listeria_monocytogenes_1639_v97	Listeria_ivanovii_subsp_ivanovii_202751	0.8931
Listeria_monocytogenes_1639_v96	Listeria_ivanovii_subsp_ivanovii_202751	0.8808
Listeria_monocytogenes_1639_v95	Listeria_ivanovii_subsp_ivanovii_202751	0.8809
Listeria_monocytogenes_1639_v94	Listeria_ivanovii_subsp_ivanovii_202751	0.8808
Listeria_monocytogenes_1639_v93	Listeria_ivanovii_subsp_ivanovii_202751	0.8737
Listeria_monocytogenes_1639_v92	Listeria_ivanovii_subsp_ivanovii_202751	0.8812
Listeria_monocytogenes_1639_v91	Listeria_ivanovii_subsp_ivanovii_202751	0.8878
Listeria_monocytogenes_1639_v90	Listeria_ivanovii_subsp_ivanovii_202751	0.8771
Listeria_monocytogenes_1639_v8	Listeria_ivanovii_subsp_ivanovii_202751	0.8608
Listeria_monocytogenes_1639_v89	Listeria_ivanovii_subsp_ivanovii_202751	0.8938
Listeria_monocytogenes_1639_v88	Listeria_ivanovii_subsp_ivanovii_202751	0.8783
Listeria_monocytogenes_1639_v87	Listeria_ivanovii_subsp_ivanovii_202751	0.8738
Listeria_monocytogenes_1639_v86	Listeria_ivanovii_subsp_ivanovii_202751	0.8739
Listeria_monocytogenes_1639_v85	Listeria_ivanovii_subsp_ivanovii_202751	0.888
Listeria_monocytogenes_1639_v84	Listeria_ivanovii_subsp_ivanovii_202751	0.8802
Listeria_monocytogenes_1639_v83	Listeria_ivanovii_subsp_ivanovii_202751	0.8873
Listeria_monocytogenes_1639_v82	Listeria_ivanovii_subsp_ivanovii_202751	0.8866
Listeria_monocytogenes_1639_v81	Listeria_ivanovii_subsp_ivanovii_202751	0.8786
Listeria_monocytogenes_1639_v80	Listeria_ivanovii_subsp_ivanovii_202751	0.884
Listeria_monocytogenes_1639_v7	Listeria_ivanovii_subsp_ivanovii_202751	0.8608
Listeria_monocytogenes_1639_v79	Listeria_ivanovii_subsp_ivanovii_202751	0.8766
Listeria_monocytogenes_1639_v78	Listeria_ivanovii_subsp_ivanovii_202751	0.8779
Listeria_monocytogenes_1639_v77	Listeria_ivanovii_subsp_ivanovii_202751	0.8798
Listeria_monocytogenes_1639_v76	Listeria_ivanovii_subsp_ivanovii_202751	0.8702
Listeria_monocytogenes_1639_v75	Listeria_ivanovii_subsp_ivanovii_202751	0.8615
Listeria_monocytogenes_1639_v74	Listeria_ivanovii_subsp_ivanovii_202751	0.8746
Listeria_monocytogenes_1639_v73	Listeria_ivanovii_subsp_ivanovii_202751	0.8738
Listeria_monocytogenes_1639_v72	Listeria_ivanovii_subsp_ivanovii_202751	0.8864
Listeria_monocytogenes_1639_v71	Listeria_ivanovii_subsp_ivanovii_202751	0.8647
Listeria_monocytogenes_1639_v70	Listeria_ivanovii_subsp_ivanovii_202751	0.8764
Listeria_monocytogenes_1639_v6	Listeria_ivanovii_subsp_ivanovii_202751	0.8602
Listeria_monocytogenes_1639_v69	Listeria_ivanovii_subsp_ivanovii_202751	0.8901
Listeria_monocytogenes_1639_v68	Listeria_ivanovii_subsp_ivanovii_202751	0.8824
Listeria_monocytogenes_1639_v67	Listeria_ivanovii_subsp_ivanovii_202751	0.8869
Listeria_monocytogenes_1639_v66	Listeria_ivanovii_subsp_ivanovii_202751	0.8855
Listeria_monocytogenes_1639_v65	Listeria_ivanovii_subsp_ivanovii_202751	0.8745
Listeria_monocytogenes_1639_v64	Listeria_ivanovii_subsp_ivanovii_202751	0.8773
Listeria_monocytogenes_1639_v63	Listeria_ivanovii_subsp_ivanovii_202751	0.8805
Listeria_monocytogenes_1639_v62	Listeria_ivanovii_subsp_ivanovii_202751	0.8746
Listeria_monocytogenes_1639_v61	Listeria_ivanovii_subsp_ivanovii_202751	0.8741
Listeria_monocytogenes_1639_v60	Listeria_ivanovii_subsp_ivanovii_202751	0.8797
Listeria_monocytogenes_1639_v5	Listeria_ivanovii_subsp_ivanovii_202751	0.8481
Listeria_monocytogenes_1639_v59	Listeria_ivanovii_subsp_ivanovii_202751	0.888
Listeria_monocytogenes_1639_v58	Listeria_ivanovii_subsp_ivanovii_202751	0.8907
Listeria_monocytogenes_1639_v57	Listeria_ivanovii_subsp_ivanovii_202751	0.878
Listeria_monocytogenes_1639_v56	Listeria_ivanovii_subsp_ivanovii_202751	0.8876
Listeria_monocytogenes_1639_v55	Listeria_ivanovii_subsp_ivanovii_202751	0.8739
Listeria_monocytogenes_1639_v54	Listeria_ivanovii_subsp_ivanovii_202751	0.8926
Listeria_monocytogenes_1639_v53	Listeria_ivanovii_subsp_ivanovii_202751	0.8805
Listeria_monocytogenes_1639_v52	Listeria_ivanovii_subsp_ivanovii_202751	0.8846
Listeria_monocytogenes_1639_v51	Listeria_ivanovii_subsp_ivanovii_202751	0.883
Listeria_monocytogenes_1639_v50	Listeria_ivanovii_subsp_ivanovii_202751	0.8703
Listeria_monocytogenes_1639_v4	Listeria_ivanovii_subsp_ivanovii_202751	0.8595
Listeria_monocytogenes_1639_v49	Listeria_ivanovii_subsp_ivanovii_202751	0.8902
Listeria_monocytogenes_1639_v48	Listeria_ivanovii_subsp_ivanovii_202751	0.8741
Listeria_monocytogenes_1639_v47	Listeria_ivanovii_subsp_ivanovii_202751	0.8711
Listeria_monocytogenes_1639_v46	Listeria_ivanovii_subsp_ivanovii_202751	0.8817
Listeria_monocytogenes_1639_v45	Listeria_ivanovii_subsp_ivanovii_202751	0.8625
Listeria_monocytogenes_1639_v44	Listeria_ivanovii_subsp_ivanovii_202751	0.8715
Listeria_monocytogenes_1639_v43	Listeria_ivanovii_subsp_ivanovii_202751	0.8715
Listeria_monocytogenes_1639_v42	Listeria_ivanovii_subsp_ivanovii_202751	0.8651
Listeria_monocytogenes_1639_v41	Listeria_ivanovii_subsp_ivanovii_202751	0.8545
Listeria_monocytogenes_1639_v40	Listeria_ivanovii_subsp_ivanovii_202751	0.8791
Listeria_monocytogenes_1639_v3	Listeria_ivanovii_subsp_ivanovii_202751	0.8598
Listeria_monocytogenes_1639_v39	Listeria_ivanovii_subsp_ivanovii_202751	0.8826
Listeria_monocytogenes_1639_v38	Listeria_ivanovii_subsp_ivanovii_202751	0.8753
Listeria_monocytogenes_1639_v37	Listeria_ivanovii_subsp_ivanovii_202751	0.8715
Listeria_monocytogenes_1639_v36	Listeria_ivanovii_subsp_ivanovii_202751	0.868
Listeria_monocytogenes_1639_v35	Listeria_ivanovii_subsp_ivanovii_202751	0.8797
Listeria_monocytogenes_1639_v34	Listeria_ivanovii_subsp_ivanovii_202751	0.8743
Listeria_monocytogenes_1639_v33	Listeria_ivanovii_subsp_ivanovii_202751	0.8795
Listeria_monocytogenes_1639_v32	Listeria_ivanovii_subsp_ivanovii_202751	0.8701
Listeria_monocytogenes_1639_v31	Listeria_ivanovii_subsp_ivanovii_202751	0.8747
Listeria_monocytogenes_1639_v30	Listeria_ivanovii_subsp_ivanovii_202751	0.8808
Listeria_monocytogenes_1639_v2	Listeria_ivanovii_subsp_ivanovii_202751	0.8499
Listeria_monocytogenes_1639_v29	Listeria_ivanovii_subsp_ivanovii_202751	0.866

Listeria_monocytogenes_1639_v28	Listeria_ivanovii_subsp_ivanovii_202751	0.8701
Listeria_monocytogenes_1639_v27	Listeria_ivanovii_subsp_ivanovii_202751	0.8619
Listeria_monocytogenes_1639_v26	Listeria_ivanovii_subsp_ivanovii_202751	0.8619
Listeria_monocytogenes_1639_v25	Listeria_ivanovii_subsp_ivanovii_202751	0.8616
Listeria_monocytogenes_1639_v24	Listeria_ivanovii_subsp_ivanovii_202751	0.8793
Listeria_monocytogenes_1639_v23	Listeria_ivanovii_subsp_ivanovii_202751	0.8793
Listeria_monocytogenes_1639_v22	Listeria_ivanovii_subsp_ivanovii_202751	0.8804
Listeria_monocytogenes_1639_v21	Listeria_ivanovii_subsp_ivanovii_202751	0.879
Listeria_monocytogenes_1639_v20	Listeria_ivanovii_subsp_ivanovii_202751	0.8765
Listeria_monocytogenes_1639_v1	Listeria_ivanovii_subsp_ivanovii_202751	0.8567
Listeria_monocytogenes_1639_v19	Listeria_ivanovii_subsp_ivanovii_202751	0.8761
Listeria_monocytogenes_1639_v198	Listeria_ivanovii_subsp_ivanovii_202751	0.8531
Listeria_monocytogenes_1639_v197	Listeria_ivanovii_subsp_ivanovii_202751	0.8744
Listeria_monocytogenes_1639_v196	Listeria_ivanovii_subsp_ivanovii_202751	0.8688
Listeria_monocytogenes_1639_v195	Listeria_ivanovii_subsp_ivanovii_202751	0.8966
Listeria_monocytogenes_1639_v194	Listeria_ivanovii_subsp_ivanovii_202751	0.8979
Listeria_monocytogenes_1639_v193	Listeria_ivanovii_subsp_ivanovii_202751	0.8982
Listeria_monocytogenes_1639_v192	Listeria_ivanovii_subsp_ivanovii_202751	0.8667
Listeria_monocytogenes_1639_v191	Listeria_ivanovii_subsp_ivanovii_202751	0.8975
Listeria_monocytogenes_1639_v190	Listeria_ivanovii_subsp_ivanovii_202751	0.8972
Listeria_monocytogenes_1639_v18	Listeria_ivanovii_subsp_ivanovii_202751	0.8724
Listeria_monocytogenes_1639_v189	Listeria_ivanovii_subsp_ivanovii_202751	0.8969
Listeria_monocytogenes_1639_v188	Listeria_ivanovii_subsp_ivanovii_202751	0.8997
Listeria_monocytogenes_1639_v187	Listeria_ivanovii_subsp_ivanovii_202751	0.8972
Listeria_monocytogenes_1639_v186	Listeria_ivanovii_subsp_ivanovii_202751	0.8976
Listeria_monocytogenes_1639_v185	Listeria_ivanovii_subsp_ivanovii_202751	0.8972
Listeria_monocytogenes_1639_v184	Listeria_ivanovii_subsp_ivanovii_202751	0.8968
Listeria_monocytogenes_1639_v183	Listeria_ivanovii_subsp_ivanovii_202751	0.898
Listeria_monocytogenes_1639_v182	Listeria_ivanovii_subsp_ivanovii_202751	0.9001
Listeria_monocytogenes_1639_v181	Listeria_ivanovii_subsp_ivanovii_202751	0.8845
Listeria_monocytogenes_1639_v180	Listeria_ivanovii_subsp_ivanovii_202751	0.8845
Listeria_monocytogenes_1639_v17	Listeria_ivanovii_subsp_ivanovii_202751	0.8724
Listeria_monocytogenes_1639_v179	Listeria_ivanovii_subsp_ivanovii_202751	0.8871
Listeria_monocytogenes_1639_v178	Listeria_ivanovii_subsp_ivanovii_202751	0.9015
Listeria_monocytogenes_1639_v177	Listeria_ivanovii_subsp_ivanovii_202751	0.898
Listeria_monocytogenes_1639_v176	Listeria_ivanovii_subsp_ivanovii_202751	0.8965
Listeria_monocytogenes_1639_v175	Listeria_ivanovii_subsp_ivanovii_202751	0.8893
Listeria_monocytogenes_1639_v174	Listeria_ivanovii_subsp_ivanovii_202751	0.8981
Listeria_monocytogenes_1639_v173	Listeria_ivanovii_subsp_ivanovii_202751	0.9051
Listeria_monocytogenes_1639_v172	Listeria_ivanovii_subsp_ivanovii_202751	0.8902
Listeria_monocytogenes_1639_v171	Listeria_ivanovii_subsp_ivanovii_202751	0.8828
Listeria_monocytogenes_1639_v170	Listeria_ivanovii_subsp_ivanovii_202751	0.8906
Listeria_monocytogenes_1639_v16	Listeria_ivanovii_subsp_ivanovii_202751	0.8586
Listeria_monocytogenes_1639_v169	Listeria_ivanovii_subsp_ivanovii_202751	0.8908
Listeria_monocytogenes_1639_v168	Listeria_ivanovii_subsp_ivanovii_202751	0.8967
Listeria_monocytogenes_1639_v167	Listeria_ivanovii_subsp_ivanovii_202751	0.8906
Listeria_monocytogenes_1639_v166	Listeria_ivanovii_subsp_ivanovii_202751	0.8971
Listeria_monocytogenes_1639_v165	Listeria_ivanovii_subsp_ivanovii_202751	0.8904
Listeria_monocytogenes_1639_v164	Listeria_ivanovii_subsp_ivanovii_202751	0.9066
Listeria_monocytogenes_1639_v163	Listeria_ivanovii_subsp_ivanovii_202751	0.8978
Listeria_monocytogenes_1639_v162	Listeria_ivanovii_subsp_ivanovii_202751	0.9066
Listeria_monocytogenes_1639_v161	Listeria_ivanovii_subsp_ivanovii_202751	0.9059
Listeria_monocytogenes_1639_v160	Listeria_ivanovii_subsp_ivanovii_202751	0.9059
Listeria_monocytogenes_1639_v15	Listeria_ivanovii_subsp_ivanovii_202751	0.8659
Listeria_monocytogenes_1639_v159	Listeria_ivanovii_subsp_ivanovii_202751	0.8984
Listeria_monocytogenes_1639_v158	Listeria_ivanovii_subsp_ivanovii_202751	0.9056
Listeria_monocytogenes_1639_v157	Listeria_ivanovii_subsp_ivanovii_202751	0.9056
Listeria_monocytogenes_1639_v156	Listeria_ivanovii_subsp_ivanovii_202751	0.8762
Listeria_monocytogenes_1639_v155	Listeria_ivanovii_subsp_ivanovii_202751	0.8887
Listeria_monocytogenes_1639_v154	Listeria_ivanovii_subsp_ivanovii_202751	0.8892
Listeria_monocytogenes_1639_v153	Listeria_ivanovii_subsp_ivanovii_202751	0.8891
Listeria_monocytogenes_1639_v152	Listeria_ivanovii_subsp_ivanovii_202751	0.8988
Listeria_monocytogenes_1639_v151	Listeria_ivanovii_subsp_ivanovii_202751	0.8988
Listeria_monocytogenes_1639_v150	Listeria_ivanovii_subsp_ivanovii_202751	0.888
Listeria_monocytogenes_1639_v14	Listeria_ivanovii_subsp_ivanovii_202751	0.8716
Listeria_monocytogenes_1639_v149	Listeria_ivanovii_subsp_ivanovii_202751	0.8867
Listeria_monocytogenes_1639_v148	Listeria_ivanovii_subsp_ivanovii_202751	0.8973
Listeria_monocytogenes_1639_v147	Listeria_ivanovii_subsp_ivanovii_202751	0.8852
Listeria_monocytogenes_1639_v146	Listeria_ivanovii_subsp_ivanovii_202751	0.9014
Listeria_monocytogenes_1639_v145	Listeria_ivanovii_subsp_ivanovii_202751	0.8924
Listeria_monocytogenes_1639_v144	Listeria_ivanovii_subsp_ivanovii_202751	0.9039
Listeria_monocytogenes_1639_v143	Listeria_ivanovii_subsp_ivanovii_202751	0.898
Listeria_monocytogenes_1639_v142	Listeria_ivanovii_subsp_ivanovii_202751	0.9039
Listeria_monocytogenes_1639_v141	Listeria_ivanovii_subsp_ivanovii_202751	0.8995
Listeria_monocytogenes_1639_v140	Listeria_ivanovii_subsp_ivanovii_202751	0.9043
Listeria_monocytogenes_1639_v13	Listeria_ivanovii_subsp_ivanovii_202751	0.8662
Listeria_monocytogenes_1639_v139	Listeria_ivanovii_subsp_ivanovii_202751	0.8971
Listeria_monocytogenes_1639_v138	Listeria_ivanovii_subsp_ivanovii_202751	0.9036
Listeria_monocytogenes_1639_v137	Listeria_ivanovii_subsp_ivanovii_202751	0.9032
Listeria_monocytogenes_1639_v136	Listeria_ivanovii_subsp_ivanovii_202751	0.8851
Listeria_monocytogenes_1639_v135	Listeria_ivanovii_subsp_ivanovii_202751	0.873
Listeria_monocytogenes_1639_v134	Listeria_ivanovii_subsp_ivanovii_202751	0.8994
Listeria_monocytogenes_1639_v133	Listeria_ivanovii_subsp_ivanovii_202751	0.8915
Listeria_monocytogenes_1639_v132	Listeria_ivanovii_subsp_ivanovii_202751	0.8759
Listeria_monocytogenes_1639_v131	Listeria_ivanovii_subsp_ivanovii_202751	0.8876
Listeria_monocytogenes_1639_v130	Listeria_ivanovii_subsp_ivanovii_202751	0.8793
Listeria_monocytogenes_1639_v12	Listeria_ivanovii_subsp_ivanovii_202751	0.8603
Listeria_monocytogenes_1639_v129	Listeria_ivanovii_subsp_ivanovii_202751	0.9001
Listeria_monocytogenes_1639_v128	Listeria_ivanovii_subsp_ivanovii_202751	0.8958
Listeria_monocytogenes_1639_v127	Listeria_ivanovii_subsp_ivanovii_202751	0.8867
Listeria_monocytogenes_1639_v126	Listeria_ivanovii_subsp_ivanovii_202751	0.8946

Listeria_monocytogenes_1639_v125	Listeria_ivanovii_subsp_ivanovii_202751	0.8885
Listeria_monocytogenes_1639_v124	Listeria_ivanovii_subsp_ivanovii_202751	0.8882
Listeria_monocytogenes_1639_v123	Listeria_ivanovii_subsp_ivanovii_202751	0.9
Listeria_monocytogenes_1639_v122	Listeria_ivanovii_subsp_ivanovii_202751	0.9003
Listeria_monocytogenes_1639_v121	Listeria_ivanovii_subsp_ivanovii_202751	0.9003
Listeria_monocytogenes_1639_v120	Listeria_ivanovii_subsp_ivanovii_202751	0.9
Listeria_monocytogenes_1639_v11	Listeria_ivanovii_subsp_ivanovii_202751	0.8592
Listeria_monocytogenes_1639_v119	Listeria_ivanovii_subsp_ivanovii_202751	0.9003
Listeria_monocytogenes_1639_v118	Listeria_ivanovii_subsp_ivanovii_202751	0.9003
Listeria_monocytogenes_1639_v117	Listeria_ivanovii_subsp_ivanovii_202751	0.8862
Listeria_monocytogenes_1639_v116	Listeria_ivanovii_subsp_ivanovii_202751	0.899
Listeria_monocytogenes_1639_v115	Listeria_ivanovii_subsp_ivanovii_202751	0.8993
Listeria_monocytogenes_1639_v114	Listeria_ivanovii_subsp_ivanovii_202751	0.8798
Listeria_monocytogenes_1639_v113	Listeria_ivanovii_subsp_ivanovii_202751	0.8946
Listeria_monocytogenes_1639_v112	Listeria_ivanovii_subsp_ivanovii_202751	0.8948
Listeria_monocytogenes_1639_v111	Listeria_ivanovii_subsp_ivanovii_202751	0.8914
Listeria_monocytogenes_1639_v110	Listeria_ivanovii_subsp_ivanovii_202751	0.8796
Listeria_monocytogenes_1639_v10	Listeria_ivanovii_subsp_ivanovii_202751	0.8641
Listeria_monocytogenes_1639_v109	Listeria_ivanovii_subsp_ivanovii_202751	0.8792
Listeria_monocytogenes_1639_v108	Listeria_ivanovii_subsp_ivanovii_202751	0.8886
Listeria_monocytogenes_1639_v107	Listeria_ivanovii_subsp_ivanovii_202751	0.8964
Listeria_monocytogenes_1639_v106	Listeria_ivanovii_subsp_ivanovii_202751	0.8968
Listeria_monocytogenes_1639_v105	Listeria_ivanovii_subsp_ivanovii_202751	0.8968
Listeria_monocytogenes_1639_v104	Listeria_ivanovii_subsp_ivanovii_202751	0.8972
Listeria_monocytogenes_1639_v103	Listeria_ivanovii_subsp_ivanovii_202751	0.8972
Listeria_monocytogenes_1639_v102	Listeria_ivanovii_subsp_ivanovii_202751	0.8817
Listeria_monocytogenes_1639_v101	Listeria_ivanovii_subsp_ivanovii_202751	0.8709
Listeria_monocytogenes_1639_v100	Listeria_ivanovii_subsp_ivanovii_202751	0.8703
Listeria_monocytogenes_1639	Listeria_ivanovii_subsp_ivanovii_202751	0.8969
Listeria_monocytogenes_10403S_393133	Listeria_ivanovii_subsp_ivanovii_202751	0.8892
Listeria_monocytogenes_08-5923_637381	Listeria_ivanovii_subsp_ivanovii_202751	0.8715
Listeria_monocytogenes_08-5578_653938	Listeria_ivanovii_subsp_ivanovii_202751	0.8583
Listeria_monocytogenes_07PF0776_1126011	Listeria_ivanovii_subsp_ivanovii_202751	0.9033
Listeria_marthii_FSL_S4-120_702457	Listeria_ivanovii_subsp_ivanovii_202751	0.8275
Listeria_marthii_529731_v1	Listeria_ivanovii_subsp_ivanovii_202751	0.8638
Listeria_marthii_529731	Listeria_ivanovii_subsp_ivanovii_202751	0.8734
Listeria_ivanovii_WSLC3009_1457190	Listeria_ivanovii_subsp_ivanovii_202751	0.9992
Listeria_ivanovii_subsp_londoniensis_202752_v2	Listeria_ivanovii_subsp_ivanovii_202751	0.902
Listeria_ivanovii_subsp_londoniensis_202752_v1	Listeria_ivanovii_subsp_ivanovii_202751	0.8907
Listeria_ivanovii_subsp_londoniensis_202752	Listeria_ivanovii_subsp_ivanovii_202751	0.8974
Listeria_ivanovii_subsp_ivanovii_PAM_55_881621	Listeria_ivanovii_subsp_ivanovii_202751	0.9954
Listeria_ivanovii_subsp_ivanovii_202751_v2	Listeria_ivanovii_subsp_ivanovii_202751	1
Listeria_ivanovii_subsp_ivanovii_202751_v1	Listeria_ivanovii_subsp_ivanovii_202751	1
Listeria_ivanovii_subsp_ivanovii_202751	Listeria_ivanovii_subsp_ivanovii_202751	0.0628
Listeria_welshimeri_1643	Listeria_ivanovii_subsp_ivanovii_202751_v1	0.8618
Listeria_welshimeri_serovar_6b_str_SLCC5334_386043	Listeria_ivanovii_subsp_ivanovii_202751_v1	0.8612
Listeria_seeligeri_serovar_1_2b_str_SLCC3954_683837	Listeria_ivanovii_subsp_ivanovii_202751_v1	0.8826
Listeria_seeligeri_FSL_S4-171_702452	Listeria_ivanovii_subsp_ivanovii_202751_v1	0.8191
Listeria_seeligeri_FSL_N1-067_702453	Listeria_ivanovii_subsp_ivanovii_202751_v1	0.8253
Listeria_seeligeri_1640_v1	Listeria_ivanovii_subsp_ivanovii_202751_v1	0.891
Listeria_seeligeri_1640	Listeria_ivanovii_subsp_ivanovii_202751_v1	0.8765
Listeria_monocytogenes_WSLC1042_1457188	Listeria_ivanovii_subsp_ivanovii_202751_v1	0.8983
Listeria_monocytogenes_WSLC1001_1457187	Listeria_ivanovii_subsp_ivanovii_202751_v1	0.8815
Listeria_monocytogenes_SLCC7179_879090	Listeria_ivanovii_subsp_ivanovii_202751_v1	0.8889
Listeria_monocytogenes_SLCC5850_882096	Listeria_ivanovii_subsp_ivanovii_202751_v1	0.8863
Listeria_monocytogenes_SLCC2755_932919	Listeria_ivanovii_subsp_ivanovii_202751_v1	0.888
Listeria_monocytogenes_SLCC2540_879089	Listeria_ivanovii_subsp_ivanovii_202751_v1	0.8906
Listeria_monocytogenes_SLCC2479_882020	Listeria_ivanovii_subsp_ivanovii_202751_v1	0.8797
Listeria_monocytogenes_SLCC2378_879088	Listeria_ivanovii_subsp_ivanovii_202751_v1	0.8997
Listeria_monocytogenes_SLCC2376_882097	Listeria_ivanovii_subsp_ivanovii_202751_v1	0.8975
Listeria_monocytogenes_SLCC2372_932920	Listeria_ivanovii_subsp_ivanovii_202751_v1	0.8724
Listeria_monocytogenes_serotype_4b_str_LL195_1230340	Listeria_ivanovii_subsp_ivanovii_202751_v1	0.9056
Listeria_monocytogenes_serotype_4b_str_LIS0087_1134669	Listeria_ivanovii_subsp_ivanovii_202751_v1	0.8622
Listeria_monocytogenes_serotype_4b_str_F2365_265669	Listeria_ivanovii_subsp_ivanovii_202751_v1	0.9056
Listeria_monocytogenes_serotype_4b_str_CLIP_80459_568819	Listeria_ivanovii_subsp_ivanovii_202751_v1	0.9043
Listeria_monocytogenes_serotype_4b_str_81-0861_1196161	Listeria_ivanovii_subsp_ivanovii_202751_v1	0.8887
Listeria_monocytogenes_serotype_4b_str_81-0592_1196160	Listeria_ivanovii_subsp_ivanovii_202751_v1	0.8897
Listeria_monocytogenes_serotype_4b_str_81-0558_1196159	Listeria_ivanovii_subsp_ivanovii_202751_v1	0.8908
Listeria_monocytogenes_serotype_4b_str_10-0809_1196162	Listeria_ivanovii_subsp_ivanovii_202751_v1	0.8901
Listeria_monocytogenes_serotype_4b_str_02-6680_1196175	Listeria_ivanovii_subsp_ivanovii_202751_v1	0.9026
Listeria_monocytogenes_serotype_4b_str_02-6679_1196174	Listeria_ivanovii_subsp_ivanovii_202751_v1	0.9026
Listeria_monocytogenes_serotype_4b_str_02-1792_1196173	Listeria_ivanovii_subsp_ivanovii_202751_v1	0.8904
Listeria_monocytogenes_serotype_4b_str_02-1289_1196172	Listeria_ivanovii_subsp_ivanovii_202751_v1	0.8904
Listeria_monocytogenes_serotype_4b_str_02-1103_1196171	Listeria_ivanovii_subsp_ivanovii_202751_v1	0.8904
Listeria_monocytogenes_serotype_3c_str_10-5027_1196195	Listeria_ivanovii_subsp_ivanovii_202751_v1	0.8785
Listeria_monocytogenes_serotype_1_2c_str_10-5026_1196194	Listeria_ivanovii_subsp_ivanovii_202751_v1	0.8797
Listeria_monocytogenes_serotype_1_2c_str_10-5025_1196193	Listeria_ivanovii_subsp_ivanovii_202751_v1	0.8797
Listeria_monocytogenes_serotype_1_2b_str_10-0811_1196164	Listeria_ivanovii_subsp_ivanovii_202751_v1	0.8795
Listeria_monocytogenes_serotype_1_2b_str_10-0810_1196163	Listeria_ivanovii_subsp_ivanovii_202751_v1	0.8795
Listeria_monocytogenes_serotype_1_2a_str_99-6370_1196179	Listeria_ivanovii_subsp_ivanovii_202751_v1	0.866
Listeria_monocytogenes_serotype_1_2a_str_98-2035_1196178	Listeria_ivanovii_subsp_ivanovii_202751_v1	0.866
Listeria_monocytogenes_serotype_1_2a_str_95-0093_1196177	Listeria_ivanovii_subsp_ivanovii_202751_v1	0.8711
Listeria_monocytogenes_serotype_1_2a_str_88-0478_1196176	Listeria_ivanovii_subsp_ivanovii_202751_v1	0.8703
Listeria_monocytogenes_serotype_1_2a_str_10-5024_1196192	Listeria_ivanovii_subsp_ivanovii_202751_v1	0.8715
Listeria_monocytogenes_serotype_1_2a_str_10-4758_1196168	Listeria_ivanovii_subsp_ivanovii_202751_v1	0.8857
Listeria_monocytogenes_serotype_1_2a_str_10-4754_1196167	Listeria_ivanovii_subsp_ivanovii_202751_v1	0.8857
Listeria_monocytogenes_serotype_1_2a_str_10-1321_1196191	Listeria_ivanovii_subsp_ivanovii_202751_v1	0.8715
Listeria_monocytogenes_serotype_1_2a_str_10-1047_1196190	Listeria_ivanovii_subsp_ivanovii_202751_v1	0.8718
Listeria_monocytogenes_serotype_1_2a_str_10-1046_1196189	Listeria_ivanovii_subsp_ivanovii_202751_v1	0.8718
Listeria_monocytogenes_serotype_1_2a_str_10-0934_1196170	Listeria_ivanovii_subsp_ivanovii_202751_v1	0.8959
Listeria_monocytogenes_serotype_1_2a_str_10-0933_1196169	Listeria_ivanovii_subsp_ivanovii_202751_v1	0.8959

Listeria_monocytogenes_serotype_1_2a_str_10-0815_1196188	Listeria_ivanovii_subsp_ivanovii_202751_v1	0.8673
Listeria_monocytogenes_serotype_1_2a_str_10-0814_1196187	Listeria_ivanovii_subsp_ivanovii_202751_v1	0.8715
Listeria_monocytogenes_serotype_1_2a_str_10-0813_1196166	Listeria_ivanovii_subsp_ivanovii_202751_v1	0.8789
Listeria_monocytogenes_serotype_1_2a_str_10-0812_1196165	Listeria_ivanovii_subsp_ivanovii_202751_v1	0.8789
Listeria_monocytogenes_serotype_1_2a_str_08-7669_1196186	Listeria_ivanovii_subsp_ivanovii_202751_v1	0.8797
Listeria_monocytogenes_serotype_1_2a_str_08-7374_1196185	Listeria_ivanovii_subsp_ivanovii_202751_v1	0.8715
Listeria_monocytogenes_serotype_1_2a_str_08-6997_1196184	Listeria_ivanovii_subsp_ivanovii_202751_v1	0.8673
Listeria_monocytogenes_serotype_1_2a_str_08-6569_1196183	Listeria_ivanovii_subsp_ivanovii_202751_v1	0.8673
Listeria_monocytogenes_serotype_1_2a_str_08-6056_1196182	Listeria_ivanovii_subsp_ivanovii_202751_v1	0.8673
Listeria_monocytogenes_serotype_1_2a_str_04-5457_1196181	Listeria_ivanovii_subsp_ivanovii_202751_v1	0.8715
Listeria_monocytogenes_serotype_1_2a_str_02-5993_1196180	Listeria_ivanovii_subsp_ivanovii_202751_v1	0.8711
Listeria_monocytogenes_R479a_1437838	Listeria_ivanovii_subsp_ivanovii_202751_v1	0.8709
Listeria_monocytogenes_N53-1_1234142	Listeria_ivanovii_subsp_ivanovii_202751_v1	0.8499
Listeria_monocytogenes_M7_1030009	Listeria_ivanovii_subsp_ivanovii_202751_v1	0.8608
Listeria_monocytogenes_LO28_393132	Listeria_ivanovii_subsp_ivanovii_202751_v1	0.8784
Listeria_monocytogenes_L99_563174	Listeria_ivanovii_subsp_ivanovii_202751_v1	0.8615
Listeria_monocytogenes_L312_882094	Listeria_ivanovii_subsp_ivanovii_202751_v1	0.9043
Listeria_monocytogenes_J1816_930781	Listeria_ivanovii_subsp_ivanovii_202751_v1	0.8815
Listeria_monocytogenes_J1-220_930782	Listeria_ivanovii_subsp_ivanovii_202751_v1	0.8789
Listeria_monocytogenes_J0161_393130	Listeria_ivanovii_subsp_ivanovii_202751_v1	0.8784
Listeria_monocytogenes_HCC23_552536	Listeria_ivanovii_subsp_ivanovii_202751_v1	0.8612
Listeria_monocytogenes_FSL_R2-561_393126	Listeria_ivanovii_subsp_ivanovii_202751_v1	0.878
Listeria_monocytogenes_FSL_J1-208_393119	Listeria_ivanovii_subsp_ivanovii_202751_v1	0.8744
Listeria_monocytogenes_FSL_J1-175_393118	Listeria_ivanovii_subsp_ivanovii_202751_v1	0.8948
Listeria_monocytogenes_Finland_1998_393127	Listeria_ivanovii_subsp_ivanovii_202751_v1	0.897
Listeria_monocytogenes_EGD-e_169963_v1	Listeria_ivanovii_subsp_ivanovii_202751_v1	0.8863
Listeria_monocytogenes_EGD-e_169963	Listeria_ivanovii_subsp_ivanovii_202751_v1	0.8867
Listeria_monocytogenes_EGD_1334565	Listeria_ivanovii_subsp_ivanovii_202751_v1	0.8867
Listeria_monocytogenes_CFSAN002349_1299895	Listeria_ivanovii_subsp_ivanovii_202751_v1	0.9
Listeria_monocytogenes_ATCC_19117_882095_v1	Listeria_ivanovii_subsp_ivanovii_202751_v1	0.8978
Listeria_monocytogenes_ATCC_19117_882095	Listeria_ivanovii_subsp_ivanovii_202751_v1	0.8978
Listeria_monocytogenes_6179_1288295	Listeria_ivanovii_subsp_ivanovii_202751_v1	0.8629
Listeria_monocytogenes_1639_v9	Listeria_ivanovii_subsp_ivanovii_202751_v1	0.8674
Listeria_monocytogenes_1639_v99	Listeria_ivanovii_subsp_ivanovii_202751_v1	0.8815
Listeria_monocytogenes_1639_v98	Listeria_ivanovii_subsp_ivanovii_202751_v1	0.8771
Listeria_monocytogenes_1639_v97	Listeria_ivanovii_subsp_ivanovii_202751_v1	0.8931
Listeria_monocytogenes_1639_v96	Listeria_ivanovii_subsp_ivanovii_202751_v1	0.8808
Listeria_monocytogenes_1639_v95	Listeria_ivanovii_subsp_ivanovii_202751_v1	0.8809
Listeria_monocytogenes_1639_v94	Listeria_ivanovii_subsp_ivanovii_202751_v1	0.8808
Listeria_monocytogenes_1639_v93	Listeria_ivanovii_subsp_ivanovii_202751_v1	0.8737
Listeria_monocytogenes_1639_v92	Listeria_ivanovii_subsp_ivanovii_202751_v1	0.8812
Listeria_monocytogenes_1639_v91	Listeria_ivanovii_subsp_ivanovii_202751_v1	0.8878
Listeria_monocytogenes_1639_v90	Listeria_ivanovii_subsp_ivanovii_202751_v1	0.8771
Listeria_monocytogenes_1639_v8	Listeria_ivanovii_subsp_ivanovii_202751_v1	0.8608
Listeria_monocytogenes_1639_v89	Listeria_ivanovii_subsp_ivanovii_202751_v1	0.8938
Listeria_monocytogenes_1639_v88	Listeria_ivanovii_subsp_ivanovii_202751_v1	0.8783
Listeria_monocytogenes_1639_v87	Listeria_ivanovii_subsp_ivanovii_202751_v1	0.8738
Listeria_monocytogenes_1639_v86	Listeria_ivanovii_subsp_ivanovii_202751_v1	0.8739
Listeria_monocytogenes_1639_v85	Listeria_ivanovii_subsp_ivanovii_202751_v1	0.888
Listeria_monocytogenes_1639_v84	Listeria_ivanovii_subsp_ivanovii_202751_v1	0.8802
Listeria_monocytogenes_1639_v83	Listeria_ivanovii_subsp_ivanovii_202751_v1	0.8873
Listeria_monocytogenes_1639_v82	Listeria_ivanovii_subsp_ivanovii_202751_v1	0.8866
Listeria_monocytogenes_1639_v81	Listeria_ivanovii_subsp_ivanovii_202751_v1	0.8786
Listeria_monocytogenes_1639_v80	Listeria_ivanovii_subsp_ivanovii_202751_v1	0.884
Listeria_monocytogenes_1639_v7	Listeria_ivanovii_subsp_ivanovii_202751_v1	0.8608
Listeria_monocytogenes_1639_v79	Listeria_ivanovii_subsp_ivanovii_202751_v1	0.8766
Listeria_monocytogenes_1639_v78	Listeria_ivanovii_subsp_ivanovii_202751_v1	0.8779
Listeria_monocytogenes_1639_v77	Listeria_ivanovii_subsp_ivanovii_202751_v1	0.8798
Listeria_monocytogenes_1639_v76	Listeria_ivanovii_subsp_ivanovii_202751_v1	0.8702
Listeria_monocytogenes_1639_v75	Listeria_ivanovii_subsp_ivanovii_202751_v1	0.8615
Listeria_monocytogenes_1639_v74	Listeria_ivanovii_subsp_ivanovii_202751_v1	0.8746
Listeria_monocytogenes_1639_v73	Listeria_ivanovii_subsp_ivanovii_202751_v1	0.8738
Listeria_monocytogenes_1639_v72	Listeria_ivanovii_subsp_ivanovii_202751_v1	0.8864
Listeria_monocytogenes_1639_v71	Listeria_ivanovii_subsp_ivanovii_202751_v1	0.8647
Listeria_monocytogenes_1639_v70	Listeria_ivanovii_subsp_ivanovii_202751_v1	0.8764
Listeria_monocytogenes_1639_v6	Listeria_ivanovii_subsp_ivanovii_202751_v1	0.8602
Listeria_monocytogenes_1639_v69	Listeria_ivanovii_subsp_ivanovii_202751_v1	0.8901
Listeria_monocytogenes_1639_v68	Listeria_ivanovii_subsp_ivanovii_202751_v1	0.8824
Listeria_monocytogenes_1639_v67	Listeria_ivanovii_subsp_ivanovii_202751_v1	0.8869
Listeria_monocytogenes_1639_v66	Listeria_ivanovii_subsp_ivanovii_202751_v1	0.8855
Listeria_monocytogenes_1639_v65	Listeria_ivanovii_subsp_ivanovii_202751_v1	0.8745
Listeria_monocytogenes_1639_v64	Listeria_ivanovii_subsp_ivanovii_202751_v1	0.8773
Listeria_monocytogenes_1639_v63	Listeria_ivanovii_subsp_ivanovii_202751_v1	0.8805
Listeria_monocytogenes_1639_v62	Listeria_ivanovii_subsp_ivanovii_202751_v1	0.8746
Listeria_monocytogenes_1639_v61	Listeria_ivanovii_subsp_ivanovii_202751_v1	0.8741
Listeria_monocytogenes_1639_v60	Listeria_ivanovii_subsp_ivanovii_202751_v1	0.8797
Listeria_monocytogenes_1639_v5	Listeria_ivanovii_subsp_ivanovii_202751_v1	0.8481
Listeria_monocytogenes_1639_v59	Listeria_ivanovii_subsp_ivanovii_202751_v1	0.888
Listeria_monocytogenes_1639_v58	Listeria_ivanovii_subsp_ivanovii_202751_v1	0.8907
Listeria_monocytogenes_1639_v57	Listeria_ivanovii_subsp_ivanovii_202751_v1	0.878
Listeria_monocytogenes_1639_v56	Listeria_ivanovii_subsp_ivanovii_202751_v1	0.8876
Listeria_monocytogenes_1639_v55	Listeria_ivanovii_subsp_ivanovii_202751_v1	0.8739
Listeria_monocytogenes_1639_v54	Listeria_ivanovii_subsp_ivanovii_202751_v1	0.8926
Listeria_monocytogenes_1639_v53	Listeria_ivanovii_subsp_ivanovii_202751_v1	0.8805
Listeria_monocytogenes_1639_v52	Listeria_ivanovii_subsp_ivanovii_202751_v1	0.8846
Listeria_monocytogenes_1639_v51	Listeria_ivanovii_subsp_ivanovii_202751_v1	0.883
Listeria_monocytogenes_1639_v50	Listeria_ivanovii_subsp_ivanovii_202751_v1	0.8703
Listeria_monocytogenes_1639_v4	Listeria_ivanovii_subsp_ivanovii_202751_v1	0.8595
Listeria_monocytogenes_1639_v49	Listeria_ivanovii_subsp_ivanovii_202751_v1	0.8902
Listeria_monocytogenes_1639_v48	Listeria_ivanovii_subsp_ivanovii_202751_v1	0.8741
Listeria_monocytogenes_1639_v47	Listeria_ivanovii_subsp_ivanovii_202751_v1	0.8711

Listeria_monocytogenes_1639_v46	Listeria_ivanovii_subsp_ivanovii_202751_v1	0.8817
Listeria_monocytogenes_1639_v45	Listeria_ivanovii_subsp_ivanovii_202751_v1	0.8625
Listeria_monocytogenes_1639_v44	Listeria_ivanovii_subsp_ivanovii_202751_v1	0.8715
Listeria_monocytogenes_1639_v43	Listeria_ivanovii_subsp_ivanovii_202751_v1	0.8715
Listeria_monocytogenes_1639_v42	Listeria_ivanovii_subsp_ivanovii_202751_v1	0.8651
Listeria_monocytogenes_1639_v41	Listeria_ivanovii_subsp_ivanovii_202751_v1	0.8545
Listeria_monocytogenes_1639_v40	Listeria_ivanovii_subsp_ivanovii_202751_v1	0.8791
Listeria_monocytogenes_1639_v3	Listeria_ivanovii_subsp_ivanovii_202751_v1	0.8598
Listeria_monocytogenes_1639_v39	Listeria_ivanovii_subsp_ivanovii_202751_v1	0.8826
Listeria_monocytogenes_1639_v38	Listeria_ivanovii_subsp_ivanovii_202751_v1	0.8753
Listeria_monocytogenes_1639_v37	Listeria_ivanovii_subsp_ivanovii_202751_v1	0.8715
Listeria_monocytogenes_1639_v36	Listeria_ivanovii_subsp_ivanovii_202751_v1	0.868
Listeria_monocytogenes_1639_v35	Listeria_ivanovii_subsp_ivanovii_202751_v1	0.8797
Listeria_monocytogenes_1639_v34	Listeria_ivanovii_subsp_ivanovii_202751_v1	0.8743
Listeria_monocytogenes_1639_v33	Listeria_ivanovii_subsp_ivanovii_202751_v1	0.8795
Listeria_monocytogenes_1639_v32	Listeria_ivanovii_subsp_ivanovii_202751_v1	0.8701
Listeria_monocytogenes_1639_v31	Listeria_ivanovii_subsp_ivanovii_202751_v1	0.8747
Listeria_monocytogenes_1639_v30	Listeria_ivanovii_subsp_ivanovii_202751_v1	0.8808
Listeria_monocytogenes_1639_v2	Listeria_ivanovii_subsp_ivanovii_202751_v1	0.8499
Listeria_monocytogenes_1639_v29	Listeria_ivanovii_subsp_ivanovii_202751_v1	0.866
Listeria_monocytogenes_1639_v28	Listeria_ivanovii_subsp_ivanovii_202751_v1	0.8701
Listeria_monocytogenes_1639_v27	Listeria_ivanovii_subsp_ivanovii_202751_v1	0.8619
Listeria_monocytogenes_1639_v26	Listeria_ivanovii_subsp_ivanovii_202751_v1	0.8619
Listeria_monocytogenes_1639_v25	Listeria_ivanovii_subsp_ivanovii_202751_v1	0.8616
Listeria_monocytogenes_1639_v24	Listeria_ivanovii_subsp_ivanovii_202751_v1	0.8793
Listeria_monocytogenes_1639_v23	Listeria_ivanovii_subsp_ivanovii_202751_v1	0.8793
Listeria_monocytogenes_1639_v22	Listeria_ivanovii_subsp_ivanovii_202751_v1	0.8804
Listeria_monocytogenes_1639_v21	Listeria_ivanovii_subsp_ivanovii_202751_v1	0.879
Listeria_monocytogenes_1639_v20	Listeria_ivanovii_subsp_ivanovii_202751_v1	0.8765
Listeria_monocytogenes_1639_v1	Listeria_ivanovii_subsp_ivanovii_202751_v1	0.8567
Listeria_monocytogenes_1639_v19	Listeria_ivanovii_subsp_ivanovii_202751_v1	0.8761
Listeria_monocytogenes_1639_v198	Listeria_ivanovii_subsp_ivanovii_202751_v1	0.8531
Listeria_monocytogenes_1639_v197	Listeria_ivanovii_subsp_ivanovii_202751_v1	0.8744
Listeria_monocytogenes_1639_v196	Listeria_ivanovii_subsp_ivanovii_202751_v1	0.8688
Listeria_monocytogenes_1639_v195	Listeria_ivanovii_subsp_ivanovii_202751_v1	0.8966
Listeria_monocytogenes_1639_v194	Listeria_ivanovii_subsp_ivanovii_202751_v1	0.8979
Listeria_monocytogenes_1639_v193	Listeria_ivanovii_subsp_ivanovii_202751_v1	0.8982
Listeria_monocytogenes_1639_v192	Listeria_ivanovii_subsp_ivanovii_202751_v1	0.8667
Listeria_monocytogenes_1639_v191	Listeria_ivanovii_subsp_ivanovii_202751_v1	0.8975
Listeria_monocytogenes_1639_v190	Listeria_ivanovii_subsp_ivanovii_202751_v1	0.8972
Listeria_monocytogenes_1639_v18	Listeria_ivanovii_subsp_ivanovii_202751_v1	0.8724
Listeria_monocytogenes_1639_v189	Listeria_ivanovii_subsp_ivanovii_202751_v1	0.8969
Listeria_monocytogenes_1639_v188	Listeria_ivanovii_subsp_ivanovii_202751_v1	0.8997
Listeria_monocytogenes_1639_v187	Listeria_ivanovii_subsp_ivanovii_202751_v1	0.8972
Listeria_monocytogenes_1639_v186	Listeria_ivanovii_subsp_ivanovii_202751_v1	0.8976
Listeria_monocytogenes_1639_v185	Listeria_ivanovii_subsp_ivanovii_202751_v1	0.8972
Listeria_monocytogenes_1639_v184	Listeria_ivanovii_subsp_ivanovii_202751_v1	0.8968
Listeria_monocytogenes_1639_v183	Listeria_ivanovii_subsp_ivanovii_202751_v1	0.898
Listeria_monocytogenes_1639_v182	Listeria_ivanovii_subsp_ivanovii_202751_v1	0.9001
Listeria_monocytogenes_1639_v181	Listeria_ivanovii_subsp_ivanovii_202751_v1	0.8845
Listeria_monocytogenes_1639_v180	Listeria_ivanovii_subsp_ivanovii_202751_v1	0.8845
Listeria_monocytogenes_1639_v17	Listeria_ivanovii_subsp_ivanovii_202751_v1	0.8724
Listeria_monocytogenes_1639_v179	Listeria_ivanovii_subsp_ivanovii_202751_v1	0.8871
Listeria_monocytogenes_1639_v178	Listeria_ivanovii_subsp_ivanovii_202751_v1	0.9015
Listeria_monocytogenes_1639_v177	Listeria_ivanovii_subsp_ivanovii_202751_v1	0.898
Listeria_monocytogenes_1639_v176	Listeria_ivanovii_subsp_ivanovii_202751_v1	0.8965
Listeria_monocytogenes_1639_v175	Listeria_ivanovii_subsp_ivanovii_202751_v1	0.8893
Listeria_monocytogenes_1639_v174	Listeria_ivanovii_subsp_ivanovii_202751_v1	0.8981
Listeria_monocytogenes_1639_v173	Listeria_ivanovii_subsp_ivanovii_202751_v1	0.9051
Listeria_monocytogenes_1639_v172	Listeria_ivanovii_subsp_ivanovii_202751_v1	0.8902
Listeria_monocytogenes_1639_v171	Listeria_ivanovii_subsp_ivanovii_202751_v1	0.8828
Listeria_monocytogenes_1639_v170	Listeria_ivanovii_subsp_ivanovii_202751_v1	0.8906
Listeria_monocytogenes_1639_v16	Listeria_ivanovii_subsp_ivanovii_202751_v1	0.8586
Listeria_monocytogenes_1639_v169	Listeria_ivanovii_subsp_ivanovii_202751_v1	0.8908
Listeria_monocytogenes_1639_v168	Listeria_ivanovii_subsp_ivanovii_202751_v1	0.8967
Listeria_monocytogenes_1639_v167	Listeria_ivanovii_subsp_ivanovii_202751_v1	0.8906
Listeria_monocytogenes_1639_v166	Listeria_ivanovii_subsp_ivanovii_202751_v1	0.8971
Listeria_monocytogenes_1639_v165	Listeria_ivanovii_subsp_ivanovii_202751_v1	0.8904
Listeria_monocytogenes_1639_v164	Listeria_ivanovii_subsp_ivanovii_202751_v1	0.9066
Listeria_monocytogenes_1639_v163	Listeria_ivanovii_subsp_ivanovii_202751_v1	0.8978
Listeria_monocytogenes_1639_v162	Listeria_ivanovii_subsp_ivanovii_202751_v1	0.9066
Listeria_monocytogenes_1639_v161	Listeria_ivanovii_subsp_ivanovii_202751_v1	0.9059
Listeria_monocytogenes_1639_v160	Listeria_ivanovii_subsp_ivanovii_202751_v1	0.9059
Listeria_monocytogenes_1639_v15	Listeria_ivanovii_subsp_ivanovii_202751_v1	0.8659
Listeria_monocytogenes_1639_v159	Listeria_ivanovii_subsp_ivanovii_202751_v1	0.8984
Listeria_monocytogenes_1639_v158	Listeria_ivanovii_subsp_ivanovii_202751_v1	0.9056
Listeria_monocytogenes_1639_v157	Listeria_ivanovii_subsp_ivanovii_202751_v1	0.9056
Listeria_monocytogenes_1639_v156	Listeria_ivanovii_subsp_ivanovii_202751_v1	0.8762
Listeria_monocytogenes_1639_v155	Listeria_ivanovii_subsp_ivanovii_202751_v1	0.8887
Listeria_monocytogenes_1639_v154	Listeria_ivanovii_subsp_ivanovii_202751_v1	0.8892
Listeria_monocytogenes_1639_v153	Listeria_ivanovii_subsp_ivanovii_202751_v1	0.8891
Listeria_monocytogenes_1639_v152	Listeria_ivanovii_subsp_ivanovii_202751_v1	0.8988
Listeria_monocytogenes_1639_v151	Listeria_ivanovii_subsp_ivanovii_202751_v1	0.8988
Listeria_monocytogenes_1639_v150	Listeria_ivanovii_subsp_ivanovii_202751_v1	0.888
Listeria_monocytogenes_1639_v14	Listeria_ivanovii_subsp_ivanovii_202751_v1	0.8716
Listeria_monocytogenes_1639_v149	Listeria_ivanovii_subsp_ivanovii_202751_v1	0.8867
Listeria_monocytogenes_1639_v148	Listeria_ivanovii_subsp_ivanovii_202751_v1	0.8973
Listeria_monocytogenes_1639_v147	Listeria_ivanovii_subsp_ivanovii_202751_v1	0.8852
Listeria_monocytogenes_1639_v146	Listeria_ivanovii_subsp_ivanovii_202751_v1	0.9014
Listeria_monocytogenes_1639_v145	Listeria_ivanovii_subsp_ivanovii_202751_v1	0.8924
Listeria_monocytogenes_1639_v144	Listeria_ivanovii_subsp_ivanovii_202751_v1	0.9039

Listeria_monocytogenes_1639_v143	Listeria_ivanovii_subsp_ivanovii_202751_v1	0.898
Listeria_monocytogenes_1639_v142	Listeria_ivanovii_subsp_ivanovii_202751_v1	0.9039
Listeria_monocytogenes_1639_v141	Listeria_ivanovii_subsp_ivanovii_202751_v1	0.8995
Listeria_monocytogenes_1639_v140	Listeria_ivanovii_subsp_ivanovii_202751_v1	0.9043
Listeria_monocytogenes_1639_v13	Listeria_ivanovii_subsp_ivanovii_202751_v1	0.8662
Listeria_monocytogenes_1639_v139	Listeria_ivanovii_subsp_ivanovii_202751_v1	0.8971
Listeria_monocytogenes_1639_v138	Listeria_ivanovii_subsp_ivanovii_202751_v1	0.9036
Listeria_monocytogenes_1639_v137	Listeria_ivanovii_subsp_ivanovii_202751_v1	0.9032
Listeria_monocytogenes_1639_v136	Listeria_ivanovii_subsp_ivanovii_202751_v1	0.8851
Listeria_monocytogenes_1639_v135	Listeria_ivanovii_subsp_ivanovii_202751_v1	0.873
Listeria_monocytogenes_1639_v134	Listeria_ivanovii_subsp_ivanovii_202751_v1	0.8994
Listeria_monocytogenes_1639_v133	Listeria_ivanovii_subsp_ivanovii_202751_v1	0.8915
Listeria_monocytogenes_1639_v132	Listeria_ivanovii_subsp_ivanovii_202751_v1	0.8759
Listeria_monocytogenes_1639_v131	Listeria_ivanovii_subsp_ivanovii_202751_v1	0.8876
Listeria_monocytogenes_1639_v130	Listeria_ivanovii_subsp_ivanovii_202751_v1	0.8793
Listeria_monocytogenes_1639_v12	Listeria_ivanovii_subsp_ivanovii_202751_v1	0.8603
Listeria_monocytogenes_1639_v129	Listeria_ivanovii_subsp_ivanovii_202751_v1	0.9001
Listeria_monocytogenes_1639_v128	Listeria_ivanovii_subsp_ivanovii_202751_v1	0.8958
Listeria_monocytogenes_1639_v127	Listeria_ivanovii_subsp_ivanovii_202751_v1	0.8867
Listeria_monocytogenes_1639_v126	Listeria_ivanovii_subsp_ivanovii_202751_v1	0.8946
Listeria_monocytogenes_1639_v125	Listeria_ivanovii_subsp_ivanovii_202751_v1	0.8885
Listeria_monocytogenes_1639_v124	Listeria_ivanovii_subsp_ivanovii_202751_v1	0.8882
Listeria_monocytogenes_1639_v123	Listeria_ivanovii_subsp_ivanovii_202751_v1	0.9
Listeria_monocytogenes_1639_v122	Listeria_ivanovii_subsp_ivanovii_202751_v1	0.9003
Listeria_monocytogenes_1639_v121	Listeria_ivanovii_subsp_ivanovii_202751_v1	0.9003
Listeria_monocytogenes_1639_v120	Listeria_ivanovii_subsp_ivanovii_202751_v1	0.9
Listeria_monocytogenes_1639_v11	Listeria_ivanovii_subsp_ivanovii_202751_v1	0.8592
Listeria_monocytogenes_1639_v119	Listeria_ivanovii_subsp_ivanovii_202751_v1	0.9003
Listeria_monocytogenes_1639_v118	Listeria_ivanovii_subsp_ivanovii_202751_v1	0.9003
Listeria_monocytogenes_1639_v117	Listeria_ivanovii_subsp_ivanovii_202751_v1	0.8862
Listeria_monocytogenes_1639_v116	Listeria_ivanovii_subsp_ivanovii_202751_v1	0.899
Listeria_monocytogenes_1639_v115	Listeria_ivanovii_subsp_ivanovii_202751_v1	0.8993
Listeria_monocytogenes_1639_v114	Listeria_ivanovii_subsp_ivanovii_202751_v1	0.8798
Listeria_monocytogenes_1639_v113	Listeria_ivanovii_subsp_ivanovii_202751_v1	0.8946
Listeria_monocytogenes_1639_v112	Listeria_ivanovii_subsp_ivanovii_202751_v1	0.8948
Listeria_monocytogenes_1639_v111	Listeria_ivanovii_subsp_ivanovii_202751_v1	0.8914
Listeria_monocytogenes_1639_v110	Listeria_ivanovii_subsp_ivanovii_202751_v1	0.8796
Listeria_monocytogenes_1639_v10	Listeria_ivanovii_subsp_ivanovii_202751_v1	0.8641
Listeria_monocytogenes_1639_v109	Listeria_ivanovii_subsp_ivanovii_202751_v1	0.8792
Listeria_monocytogenes_1639_v108	Listeria_ivanovii_subsp_ivanovii_202751_v1	0.8886
Listeria_monocytogenes_1639_v107	Listeria_ivanovii_subsp_ivanovii_202751_v1	0.8964
Listeria_monocytogenes_1639_v106	Listeria_ivanovii_subsp_ivanovii_202751_v1	0.8968
Listeria_monocytogenes_1639_v105	Listeria_ivanovii_subsp_ivanovii_202751_v1	0.8968
Listeria_monocytogenes_1639_v104	Listeria_ivanovii_subsp_ivanovii_202751_v1	0.8972
Listeria_monocytogenes_1639_v103	Listeria_ivanovii_subsp_ivanovii_202751_v1	0.8972
Listeria_monocytogenes_1639_v102	Listeria_ivanovii_subsp_ivanovii_202751_v1	0.8817
Listeria_monocytogenes_1639_v101	Listeria_ivanovii_subsp_ivanovii_202751_v1	0.8709
Listeria_monocytogenes_1639_v100	Listeria_ivanovii_subsp_ivanovii_202751_v1	0.8703
Listeria_monocytogenes_1639	Listeria_ivanovii_subsp_ivanovii_202751_v1	0.8969
Listeria_monocytogenes_10403S_393133	Listeria_ivanovii_subsp_ivanovii_202751_v1	0.8892
Listeria_monocytogenes_08-5923_637381	Listeria_ivanovii_subsp_ivanovii_202751_v1	0.8715
Listeria_monocytogenes_08-5578_653938	Listeria_ivanovii_subsp_ivanovii_202751_v1	0.8583
Listeria_monocytogenes_07PF0776_1126011	Listeria_ivanovii_subsp_ivanovii_202751_v1	0.9033
Listeria_marthii_FSL_S4-120_702457	Listeria_ivanovii_subsp_ivanovii_202751_v1	0.8275
Listeria_marthii_529731_v1	Listeria_ivanovii_subsp_ivanovii_202751_v1	0.8638
Listeria_marthii_529731	Listeria_ivanovii_subsp_ivanovii_202751_v1	0.8734
Listeria_ivanovii_WSLC3009_1457190	Listeria_ivanovii_subsp_ivanovii_202751_v1	0.9992
Listeria_ivanovii_subsp_londoniensis_202752_v2	Listeria_ivanovii_subsp_ivanovii_202751_v1	0.902
Listeria_ivanovii_subsp_londoniensis_202752_v1	Listeria_ivanovii_subsp_ivanovii_202751_v1	0.8907
Listeria_ivanovii_subsp_londoniensis_202752	Listeria_ivanovii_subsp_ivanovii_202751_v1	0.8974
Listeria_ivanovii_subsp_ivanovii_PAM_55_881621	Listeria_ivanovii_subsp_ivanovii_202751_v1	0.9954
Listeria_ivanovii_subsp_ivanovii_202751_v2	Listeria_ivanovii_subsp_ivanovii_202751_v1	1
Listeria_ivanovii_subsp_ivanovii_202751_v1	Listeria_ivanovii_subsp_ivanovii_202751_v1	0.0624
Listeria_welshimeri_1643	Listeria_ivanovii_subsp_ivanovii_202751_v2	0.8618
Listeria_welshimeri_serovar_6b_str_SLCC5334_386043	Listeria_ivanovii_subsp_ivanovii_202751_v2	0.8612
Listeria_seeligeri_serovar_1_2b_str_SLCC3954_683837	Listeria_ivanovii_subsp_ivanovii_202751_v2	0.8826
Listeria_seeligeri_FSL_S4-171_702452	Listeria_ivanovii_subsp_ivanovii_202751_v2	0.8191
Listeria_seeligeri_FSL_N1-067_702453	Listeria_ivanovii_subsp_ivanovii_202751_v2	0.8253
Listeria_seeligeri_1640_v1	Listeria_ivanovii_subsp_ivanovii_202751_v2	0.891
Listeria_seeligeri_1640	Listeria_ivanovii_subsp_ivanovii_202751_v2	0.8765
Listeria_monocytogenes_WSLC1042_1457188	Listeria_ivanovii_subsp_ivanovii_202751_v2	0.8983
Listeria_monocytogenes_WSLC1001_1457187	Listeria_ivanovii_subsp_ivanovii_202751_v2	0.8815
Listeria_monocytogenes_SLCC7179_879090	Listeria_ivanovii_subsp_ivanovii_202751_v2	0.8889
Listeria_monocytogenes_SLCC5850_882096	Listeria_ivanovii_subsp_ivanovii_202751_v2	0.8863
Listeria_monocytogenes_SLCC2755_932919	Listeria_ivanovii_subsp_ivanovii_202751_v2	0.888
Listeria_monocytogenes_SLCC2540_879089	Listeria_ivanovii_subsp_ivanovii_202751_v2	0.8906
Listeria_monocytogenes_SLCC2479_882020	Listeria_ivanovii_subsp_ivanovii_202751_v2	0.8797
Listeria_monocytogenes_SLCC2378_879088	Listeria_ivanovii_subsp_ivanovii_202751_v2	0.8997
Listeria_monocytogenes_SLCC2376_882097	Listeria_ivanovii_subsp_ivanovii_202751_v2	0.8975
Listeria_monocytogenes_SLCC2372_932920	Listeria_ivanovii_subsp_ivanovii_202751_v2	0.8724
Listeria_monocytogenes_serotype_4b_str_LL195_1230340	Listeria_ivanovii_subsp_ivanovii_202751_v2	0.9056
Listeria_monocytogenes_serotype_4b_str_LIS0087_1134669	Listeria_ivanovii_subsp_ivanovii_202751_v2	0.8622
Listeria_monocytogenes_serotype_4b_str_F2365_265669	Listeria_ivanovii_subsp_ivanovii_202751_v2	0.9056
Listeria_monocytogenes_serotype_4b_str_CLIP_80459_568819	Listeria_ivanovii_subsp_ivanovii_202751_v2	0.9043
Listeria_monocytogenes_serotype_4b_str_81-0861_1196161	Listeria_ivanovii_subsp_ivanovii_202751_v2	0.8887
Listeria_monocytogenes_serotype_4b_str_81-0592_1196160	Listeria_ivanovii_subsp_ivanovii_202751_v2	0.8897
Listeria_monocytogenes_serotype_4b_str_81-0558_1196159	Listeria_ivanovii_subsp_ivanovii_202751_v2	0.8908
Listeria_monocytogenes_serotype_4b_str_10-0809_1196162	Listeria_ivanovii_subsp_ivanovii_202751_v2	0.8901
Listeria_monocytogenes_serotype_4b_str_02-6680_1196175	Listeria_ivanovii_subsp_ivanovii_202751_v2	0.9026
Listeria_monocytogenes_serotype_4b_str_02-6679_1196174	Listeria_ivanovii_subsp_ivanovii_202751_v2	0.9026
Listeria_monocytogenes_serotype_4b_str_02-1792_1196173	Listeria_ivanovii_subsp_ivanovii_202751_v2	0.8904

Listeria_monocytogenes_serotype_4b_str_02-1289_1196172	Listeria_ivanovii_subsp_ivanovii_202751_v2	0.8904
Listeria_monocytogenes_serotype_4b_str_02-1103_1196171	Listeria_ivanovii_subsp_ivanovii_202751_v2	0.8904
Listeria_monocytogenes_serotype_3c_str_10-5027_1196195	Listeria_ivanovii_subsp_ivanovii_202751_v2	0.8785
Listeria_monocytogenes_serotype_1_2c_str_10-5026_1196194	Listeria_ivanovii_subsp_ivanovii_202751_v2	0.8797
Listeria_monocytogenes_serotype_1_2c_str_10-5025_1196193	Listeria_ivanovii_subsp_ivanovii_202751_v2	0.8797
Listeria_monocytogenes_serotype_1_2b_str_10-0811_1196164	Listeria_ivanovii_subsp_ivanovii_202751_v2	0.8795
Listeria_monocytogenes_serotype_1_2b_str_10-0810_1196163	Listeria_ivanovii_subsp_ivanovii_202751_v2	0.8795
Listeria_monocytogenes_serotype_1_2a_str_99-6370_1196179	Listeria_ivanovii_subsp_ivanovii_202751_v2	0.866
Listeria_monocytogenes_serotype_1_2a_str_98-2035_1196178	Listeria_ivanovii_subsp_ivanovii_202751_v2	0.866
Listeria_monocytogenes_serotype_1_2a_str_95-0093_1196177	Listeria_ivanovii_subsp_ivanovii_202751_v2	0.8711
Listeria_monocytogenes_serotype_1_2a_str_88-0478_1196176	Listeria_ivanovii_subsp_ivanovii_202751_v2	0.8703
Listeria_monocytogenes_serotype_1_2a_str_10-5024_1196192	Listeria_ivanovii_subsp_ivanovii_202751_v2	0.8715
Listeria_monocytogenes_serotype_1_2a_str_10-4758_1196168	Listeria_ivanovii_subsp_ivanovii_202751_v2	0.8857
Listeria_monocytogenes_serotype_1_2a_str_10-4754_1196167	Listeria_ivanovii_subsp_ivanovii_202751_v2	0.8857
Listeria_monocytogenes_serotype_1_2a_str_10-1321_1196191	Listeria_ivanovii_subsp_ivanovii_202751_v2	0.8715
Listeria_monocytogenes_serotype_1_2a_str_10-1047_1196190	Listeria_ivanovii_subsp_ivanovii_202751_v2	0.8718
Listeria_monocytogenes_serotype_1_2a_str_10-1046_1196189	Listeria_ivanovii_subsp_ivanovii_202751_v2	0.8718
Listeria_monocytogenes_serotype_1_2a_str_10-0934_1196170	Listeria_ivanovii_subsp_ivanovii_202751_v2	0.8959
Listeria_monocytogenes_serotype_1_2a_str_10-0933_1196169	Listeria_ivanovii_subsp_ivanovii_202751_v2	0.8959
Listeria_monocytogenes_serotype_1_2a_str_10-0815_1196188	Listeria_ivanovii_subsp_ivanovii_202751_v2	0.8673
Listeria_monocytogenes_serotype_1_2a_str_10-0814_1196187	Listeria_ivanovii_subsp_ivanovii_202751_v2	0.8715
Listeria_monocytogenes_serotype_1_2a_str_10-0813_1196166	Listeria_ivanovii_subsp_ivanovii_202751_v2	0.8789
Listeria_monocytogenes_serotype_1_2a_str_10-0812_1196165	Listeria_ivanovii_subsp_ivanovii_202751_v2	0.8789
Listeria_monocytogenes_serotype_1_2a_str_08-7669_1196186	Listeria_ivanovii_subsp_ivanovii_202751_v2	0.8797
Listeria_monocytogenes_serotype_1_2a_str_08-7374_1196185	Listeria_ivanovii_subsp_ivanovii_202751_v2	0.8715
Listeria_monocytogenes_serotype_1_2a_str_08-6997_1196184	Listeria_ivanovii_subsp_ivanovii_202751_v2	0.8673
Listeria_monocytogenes_serotype_1_2a_str_08-6569_1196183	Listeria_ivanovii_subsp_ivanovii_202751_v2	0.8673
Listeria_monocytogenes_serotype_1_2a_str_08-6056_1196182	Listeria_ivanovii_subsp_ivanovii_202751_v2	0.8673
Listeria_monocytogenes_serotype_1_2a_str_04-5457_1196181	Listeria_ivanovii_subsp_ivanovii_202751_v2	0.8715
Listeria_monocytogenes_serotype_1_2a_str_02-5993_1196180	Listeria_ivanovii_subsp_ivanovii_202751_v2	0.8711
Listeria_monocytogenes_R479a_1437838	Listeria_ivanovii_subsp_ivanovii_202751_v2	0.8709
Listeria_monocytogenes_N53-1_1234142	Listeria_ivanovii_subsp_ivanovii_202751_v2	0.8499
Listeria_monocytogenes_M7_1030009	Listeria_ivanovii_subsp_ivanovii_202751_v2	0.8608
Listeria_monocytogenes_LO28_393132	Listeria_ivanovii_subsp_ivanovii_202751_v2	0.8784
Listeria_monocytogenes_L99_563174	Listeria_ivanovii_subsp_ivanovii_202751_v2	0.8615
Listeria_monocytogenes_L312_882094	Listeria_ivanovii_subsp_ivanovii_202751_v2	0.9043
Listeria_monocytogenes_J1816_930781	Listeria_ivanovii_subsp_ivanovii_202751_v2	0.8815
Listeria_monocytogenes_J1-220_930782	Listeria_ivanovii_subsp_ivanovii_202751_v2	0.8789
Listeria_monocytogenes_J0161_393130	Listeria_ivanovii_subsp_ivanovii_202751_v2	0.8784
Listeria_monocytogenes_HCC23_552536	Listeria_ivanovii_subsp_ivanovii_202751_v2	0.8612
Listeria_monocytogenes_FSL_R2-561_393126	Listeria_ivanovii_subsp_ivanovii_202751_v2	0.878
Listeria_monocytogenes_FSL_J1-208_393119	Listeria_ivanovii_subsp_ivanovii_202751_v2	0.8744
Listeria_monocytogenes_FSL_J1-175_393118	Listeria_ivanovii_subsp_ivanovii_202751_v2	0.8948
Listeria_monocytogenes_Finland_1998_393127	Listeria_ivanovii_subsp_ivanovii_202751_v2	0.897
Listeria_monocytogenes_EGD-e_169963_v1	Listeria_ivanovii_subsp_ivanovii_202751_v2	0.8863
Listeria_monocytogenes_EGD-e_169963	Listeria_ivanovii_subsp_ivanovii_202751_v2	0.8867
Listeria_monocytogenes_EGD_1334565	Listeria_ivanovii_subsp_ivanovii_202751_v2	0.8867
Listeria_monocytogenes_CFSAN002349_1299895	Listeria_ivanovii_subsp_ivanovii_202751_v2	0.9
Listeria_monocytogenes_ATCC_19117_882095_v1	Listeria_ivanovii_subsp_ivanovii_202751_v2	0.8978
Listeria_monocytogenes_ATCC_19117_882095	Listeria_ivanovii_subsp_ivanovii_202751_v2	0.8978
Listeria_monocytogenes_6179_1288295	Listeria_ivanovii_subsp_ivanovii_202751_v2	0.8629
Listeria_monocytogenes_1639_v9	Listeria_ivanovii_subsp_ivanovii_202751_v2	0.8674
Listeria_monocytogenes_1639_v99	Listeria_ivanovii_subsp_ivanovii_202751_v2	0.8815
Listeria_monocytogenes_1639_v98	Listeria_ivanovii_subsp_ivanovii_202751_v2	0.8771
Listeria_monocytogenes_1639_v97	Listeria_ivanovii_subsp_ivanovii_202751_v2	0.8931
Listeria_monocytogenes_1639_v96	Listeria_ivanovii_subsp_ivanovii_202751_v2	0.8808
Listeria_monocytogenes_1639_v95	Listeria_ivanovii_subsp_ivanovii_202751_v2	0.8809
Listeria_monocytogenes_1639_v94	Listeria_ivanovii_subsp_ivanovii_202751_v2	0.8808
Listeria_monocytogenes_1639_v93	Listeria_ivanovii_subsp_ivanovii_202751_v2	0.8737
Listeria_monocytogenes_1639_v92	Listeria_ivanovii_subsp_ivanovii_202751_v2	0.8812
Listeria_monocytogenes_1639_v91	Listeria_ivanovii_subsp_ivanovii_202751_v2	0.8878
Listeria_monocytogenes_1639_v90	Listeria_ivanovii_subsp_ivanovii_202751_v2	0.8771
Listeria_monocytogenes_1639_v8	Listeria_ivanovii_subsp_ivanovii_202751_v2	0.8608
Listeria_monocytogenes_1639_v89	Listeria_ivanovii_subsp_ivanovii_202751_v2	0.8938
Listeria_monocytogenes_1639_v88	Listeria_ivanovii_subsp_ivanovii_202751_v2	0.8783
Listeria_monocytogenes_1639_v87	Listeria_ivanovii_subsp_ivanovii_202751_v2	0.8738
Listeria_monocytogenes_1639_v86	Listeria_ivanovii_subsp_ivanovii_202751_v2	0.8739
Listeria_monocytogenes_1639_v85	Listeria_ivanovii_subsp_ivanovii_202751_v2	0.888
Listeria_monocytogenes_1639_v84	Listeria_ivanovii_subsp_ivanovii_202751_v2	0.8802
Listeria_monocytogenes_1639_v83	Listeria_ivanovii_subsp_ivanovii_202751_v2	0.8873
Listeria_monocytogenes_1639_v82	Listeria_ivanovii_subsp_ivanovii_202751_v2	0.8866
Listeria_monocytogenes_1639_v81	Listeria_ivanovii_subsp_ivanovii_202751_v2	0.8786
Listeria_monocytogenes_1639_v80	Listeria_ivanovii_subsp_ivanovii_202751_v2	0.884
Listeria_monocytogenes_1639_v7	Listeria_ivanovii_subsp_ivanovii_202751_v2	0.8608
Listeria_monocytogenes_1639_v79	Listeria_ivanovii_subsp_ivanovii_202751_v2	0.8766
Listeria_monocytogenes_1639_v78	Listeria_ivanovii_subsp_ivanovii_202751_v2	0.8779
Listeria_monocytogenes_1639_v77	Listeria_ivanovii_subsp_ivanovii_202751_v2	0.8798
Listeria_monocytogenes_1639_v76	Listeria_ivanovii_subsp_ivanovii_202751_v2	0.8702
Listeria_monocytogenes_1639_v75	Listeria_ivanovii_subsp_ivanovii_202751_v2	0.8615
Listeria_monocytogenes_1639_v74	Listeria_ivanovii_subsp_ivanovii_202751_v2	0.8746
Listeria_monocytogenes_1639_v73	Listeria_ivanovii_subsp_ivanovii_202751_v2	0.8738
Listeria_monocytogenes_1639_v72	Listeria_ivanovii_subsp_ivanovii_202751_v2	0.8864
Listeria_monocytogenes_1639_v71	Listeria_ivanovii_subsp_ivanovii_202751_v2	0.8647
Listeria_monocytogenes_1639_v70	Listeria_ivanovii_subsp_ivanovii_202751_v2	0.8764
Listeria_monocytogenes_1639_v6	Listeria_ivanovii_subsp_ivanovii_202751_v2	0.8602
Listeria_monocytogenes_1639_v69	Listeria_ivanovii_subsp_ivanovii_202751_v2	0.8901
Listeria_monocytogenes_1639_v68	Listeria_ivanovii_subsp_ivanovii_202751_v2	0.8824
Listeria_monocytogenes_1639_v67	Listeria_ivanovii_subsp_ivanovii_202751_v2	0.8869
Listeria_monocytogenes_1639_v66	Listeria_ivanovii_subsp_ivanovii_202751_v2	0.8855
Listeria_monocytogenes_1639_v65	Listeria_ivanovii_subsp_ivanovii_202751_v2	0.8745
Listeria_monocytogenes_1639_v64	Listeria_ivanovii_subsp_ivanovii_202751_v2	0.8773

Listeria_monocytogenes_1639_v63	Listeria_ivanovii_subsp_ivanovii_202751_v2	0.8805
Listeria_monocytogenes_1639_v62	Listeria_ivanovii_subsp_ivanovii_202751_v2	0.8746
Listeria_monocytogenes_1639_v61	Listeria_ivanovii_subsp_ivanovii_202751_v2	0.8741
Listeria_monocytogenes_1639_v60	Listeria_ivanovii_subsp_ivanovii_202751_v2	0.8797
Listeria_monocytogenes_1639_v5	Listeria_ivanovii_subsp_ivanovii_202751_v2	0.8481
Listeria_monocytogenes_1639_v59	Listeria_ivanovii_subsp_ivanovii_202751_v2	0.888
Listeria_monocytogenes_1639_v58	Listeria_ivanovii_subsp_ivanovii_202751_v2	0.8907
Listeria_monocytogenes_1639_v57	Listeria_ivanovii_subsp_ivanovii_202751_v2	0.878
Listeria_monocytogenes_1639_v56	Listeria_ivanovii_subsp_ivanovii_202751_v2	0.8876
Listeria_monocytogenes_1639_v55	Listeria_ivanovii_subsp_ivanovii_202751_v2	0.8739
Listeria_monocytogenes_1639_v54	Listeria_ivanovii_subsp_ivanovii_202751_v2	0.8926
Listeria_monocytogenes_1639_v53	Listeria_ivanovii_subsp_ivanovii_202751_v2	0.8805
Listeria_monocytogenes_1639_v52	Listeria_ivanovii_subsp_ivanovii_202751_v2	0.8846
Listeria_monocytogenes_1639_v51	Listeria_ivanovii_subsp_ivanovii_202751_v2	0.883
Listeria_monocytogenes_1639_v50	Listeria_ivanovii_subsp_ivanovii_202751_v2	0.8703
Listeria_monocytogenes_1639_v4	Listeria_ivanovii_subsp_ivanovii_202751_v2	0.8595
Listeria_monocytogenes_1639_v49	Listeria_ivanovii_subsp_ivanovii_202751_v2	0.8902
Listeria_monocytogenes_1639_v48	Listeria_ivanovii_subsp_ivanovii_202751_v2	0.8741
Listeria_monocytogenes_1639_v47	Listeria_ivanovii_subsp_ivanovii_202751_v2	0.8711
Listeria_monocytogenes_1639_v46	Listeria_ivanovii_subsp_ivanovii_202751_v2	0.8817
Listeria_monocytogenes_1639_v45	Listeria_ivanovii_subsp_ivanovii_202751_v2	0.8625
Listeria_monocytogenes_1639_v44	Listeria_ivanovii_subsp_ivanovii_202751_v2	0.8715
Listeria_monocytogenes_1639_v43	Listeria_ivanovii_subsp_ivanovii_202751_v2	0.8715
Listeria_monocytogenes_1639_v42	Listeria_ivanovii_subsp_ivanovii_202751_v2	0.8651
Listeria_monocytogenes_1639_v41	Listeria_ivanovii_subsp_ivanovii_202751_v2	0.8545
Listeria_monocytogenes_1639_v40	Listeria_ivanovii_subsp_ivanovii_202751_v2	0.8791
Listeria_monocytogenes_1639_v3	Listeria_ivanovii_subsp_ivanovii_202751_v2	0.8598
Listeria_monocytogenes_1639_v39	Listeria_ivanovii_subsp_ivanovii_202751_v2	0.8826
Listeria_monocytogenes_1639_v38	Listeria_ivanovii_subsp_ivanovii_202751_v2	0.8753
Listeria_monocytogenes_1639_v37	Listeria_ivanovii_subsp_ivanovii_202751_v2	0.8715
Listeria_monocytogenes_1639_v36	Listeria_ivanovii_subsp_ivanovii_202751_v2	0.868
Listeria_monocytogenes_1639_v35	Listeria_ivanovii_subsp_ivanovii_202751_v2	0.8797
Listeria_monocytogenes_1639_v34	Listeria_ivanovii_subsp_ivanovii_202751_v2	0.8743
Listeria_monocytogenes_1639_v33	Listeria_ivanovii_subsp_ivanovii_202751_v2	0.8795
Listeria_monocytogenes_1639_v32	Listeria_ivanovii_subsp_ivanovii_202751_v2	0.8701
Listeria_monocytogenes_1639_v31	Listeria_ivanovii_subsp_ivanovii_202751_v2	0.8747
Listeria_monocytogenes_1639_v30	Listeria_ivanovii_subsp_ivanovii_202751_v2	0.8808
Listeria_monocytogenes_1639_v2	Listeria_ivanovii_subsp_ivanovii_202751_v2	0.8499
Listeria_monocytogenes_1639_v29	Listeria_ivanovii_subsp_ivanovii_202751_v2	0.866
Listeria_monocytogenes_1639_v28	Listeria_ivanovii_subsp_ivanovii_202751_v2	0.8701
Listeria_monocytogenes_1639_v27	Listeria_ivanovii_subsp_ivanovii_202751_v2	0.8619
Listeria_monocytogenes_1639_v26	Listeria_ivanovii_subsp_ivanovii_202751_v2	0.8619
Listeria_monocytogenes_1639_v25	Listeria_ivanovii_subsp_ivanovii_202751_v2	0.8616
Listeria_monocytogenes_1639_v24	Listeria_ivanovii_subsp_ivanovii_202751_v2	0.8793
Listeria_monocytogenes_1639_v23	Listeria_ivanovii_subsp_ivanovii_202751_v2	0.8793
Listeria_monocytogenes_1639_v22	Listeria_ivanovii_subsp_ivanovii_202751_v2	0.8804
Listeria_monocytogenes_1639_v21	Listeria_ivanovii_subsp_ivanovii_202751_v2	0.879
Listeria_monocytogenes_1639_v20	Listeria_ivanovii_subsp_ivanovii_202751_v2	0.8765
Listeria_monocytogenes_1639_v1	Listeria_ivanovii_subsp_ivanovii_202751_v2	0.8567
Listeria_monocytogenes_1639_v19	Listeria_ivanovii_subsp_ivanovii_202751_v2	0.8761
Listeria_monocytogenes_1639_v198	Listeria_ivanovii_subsp_ivanovii_202751_v2	0.8531
Listeria_monocytogenes_1639_v197	Listeria_ivanovii_subsp_ivanovii_202751_v2	0.8744
Listeria_monocytogenes_1639_v196	Listeria_ivanovii_subsp_ivanovii_202751_v2	0.8688
Listeria_monocytogenes_1639_v195	Listeria_ivanovii_subsp_ivanovii_202751_v2	0.8966
Listeria_monocytogenes_1639_v194	Listeria_ivanovii_subsp_ivanovii_202751_v2	0.8979
Listeria_monocytogenes_1639_v193	Listeria_ivanovii_subsp_ivanovii_202751_v2	0.8982
Listeria_monocytogenes_1639_v192	Listeria_ivanovii_subsp_ivanovii_202751_v2	0.8667
Listeria_monocytogenes_1639_v191	Listeria_ivanovii_subsp_ivanovii_202751_v2	0.8975
Listeria_monocytogenes_1639_v190	Listeria_ivanovii_subsp_ivanovii_202751_v2	0.8972
Listeria_monocytogenes_1639_v18	Listeria_ivanovii_subsp_ivanovii_202751_v2	0.8724
Listeria_monocytogenes_1639_v189	Listeria_ivanovii_subsp_ivanovii_202751_v2	0.8969
Listeria_monocytogenes_1639_v188	Listeria_ivanovii_subsp_ivanovii_202751_v2	0.8997
Listeria_monocytogenes_1639_v187	Listeria_ivanovii_subsp_ivanovii_202751_v2	0.8972
Listeria_monocytogenes_1639_v186	Listeria_ivanovii_subsp_ivanovii_202751_v2	0.8976
Listeria_monocytogenes_1639_v185	Listeria_ivanovii_subsp_ivanovii_202751_v2	0.8972
Listeria_monocytogenes_1639_v184	Listeria_ivanovii_subsp_ivanovii_202751_v2	0.8968
Listeria_monocytogenes_1639_v183	Listeria_ivanovii_subsp_ivanovii_202751_v2	0.898
Listeria_monocytogenes_1639_v182	Listeria_ivanovii_subsp_ivanovii_202751_v2	0.9001
Listeria_monocytogenes_1639_v181	Listeria_ivanovii_subsp_ivanovii_202751_v2	0.8845
Listeria_monocytogenes_1639_v180	Listeria_ivanovii_subsp_ivanovii_202751_v2	0.8845
Listeria_monocytogenes_1639_v17	Listeria_ivanovii_subsp_ivanovii_202751_v2	0.8724
Listeria_monocytogenes_1639_v179	Listeria_ivanovii_subsp_ivanovii_202751_v2	0.8871
Listeria_monocytogenes_1639_v178	Listeria_ivanovii_subsp_ivanovii_202751_v2	0.9015
Listeria_monocytogenes_1639_v177	Listeria_ivanovii_subsp_ivanovii_202751_v2	0.898
Listeria_monocytogenes_1639_v176	Listeria_ivanovii_subsp_ivanovii_202751_v2	0.8965
Listeria_monocytogenes_1639_v175	Listeria_ivanovii_subsp_ivanovii_202751_v2	0.8893
Listeria_monocytogenes_1639_v174	Listeria_ivanovii_subsp_ivanovii_202751_v2	0.8981
Listeria_monocytogenes_1639_v173	Listeria_ivanovii_subsp_ivanovii_202751_v2	0.9051
Listeria_monocytogenes_1639_v172	Listeria_ivanovii_subsp_ivanovii_202751_v2	0.8902
Listeria_monocytogenes_1639_v171	Listeria_ivanovii_subsp_ivanovii_202751_v2	0.8828
Listeria_monocytogenes_1639_v170	Listeria_ivanovii_subsp_ivanovii_202751_v2	0.8906
Listeria_monocytogenes_1639_v16	Listeria_ivanovii_subsp_ivanovii_202751_v2	0.8586
Listeria_monocytogenes_1639_v169	Listeria_ivanovii_subsp_ivanovii_202751_v2	0.8908
Listeria_monocytogenes_1639_v168	Listeria_ivanovii_subsp_ivanovii_202751_v2	0.8967
Listeria_monocytogenes_1639_v167	Listeria_ivanovii_subsp_ivanovii_202751_v2	0.8906
Listeria_monocytogenes_1639_v166	Listeria_ivanovii_subsp_ivanovii_202751_v2	0.8971
Listeria_monocytogenes_1639_v165	Listeria_ivanovii_subsp_ivanovii_202751_v2	0.8904
Listeria_monocytogenes_1639_v164	Listeria_ivanovii_subsp_ivanovii_202751_v2	0.9066
Listeria_monocytogenes_1639_v163	Listeria_ivanovii_subsp_ivanovii_202751_v2	0.8978
Listeria_monocytogenes_1639_v162	Listeria_ivanovii_subsp_ivanovii_202751_v2	0.9066
Listeria_monocytogenes_1639_v161	Listeria_ivanovii_subsp_ivanovii_202751_v2	0.9059

Listeria_monocytogenes_1639_v160	Listeria_ivanovii_subsp_ivanovii_202751_v2	0.9059
Listeria_monocytogenes_1639_v15	Listeria_ivanovii_subsp_ivanovii_202751_v2	0.8659
Listeria_monocytogenes_1639_v159	Listeria_ivanovii_subsp_ivanovii_202751_v2	0.8984
Listeria_monocytogenes_1639_v158	Listeria_ivanovii_subsp_ivanovii_202751_v2	0.9056
Listeria_monocytogenes_1639_v157	Listeria_ivanovii_subsp_ivanovii_202751_v2	0.9056
Listeria_monocytogenes_1639_v156	Listeria_ivanovii_subsp_ivanovii_202751_v2	0.8762
Listeria_monocytogenes_1639_v155	Listeria_ivanovii_subsp_ivanovii_202751_v2	0.8887
Listeria_monocytogenes_1639_v154	Listeria_ivanovii_subsp_ivanovii_202751_v2	0.8892
Listeria_monocytogenes_1639_v153	Listeria_ivanovii_subsp_ivanovii_202751_v2	0.8891
Listeria_monocytogenes_1639_v152	Listeria_ivanovii_subsp_ivanovii_202751_v2	0.8988
Listeria_monocytogenes_1639_v151	Listeria_ivanovii_subsp_ivanovii_202751_v2	0.8988
Listeria_monocytogenes_1639_v150	Listeria_ivanovii_subsp_ivanovii_202751_v2	0.888
Listeria_monocytogenes_1639_v14	Listeria_ivanovii_subsp_ivanovii_202751_v2	0.8716
Listeria_monocytogenes_1639_v149	Listeria_ivanovii_subsp_ivanovii_202751_v2	0.8867
Listeria_monocytogenes_1639_v148	Listeria_ivanovii_subsp_ivanovii_202751_v2	0.8973
Listeria_monocytogenes_1639_v147	Listeria_ivanovii_subsp_ivanovii_202751_v2	0.8852
Listeria_monocytogenes_1639_v146	Listeria_ivanovii_subsp_ivanovii_202751_v2	0.9014
Listeria_monocytogenes_1639_v145	Listeria_ivanovii_subsp_ivanovii_202751_v2	0.8924
Listeria_monocytogenes_1639_v144	Listeria_ivanovii_subsp_ivanovii_202751_v2	0.9039
Listeria_monocytogenes_1639_v143	Listeria_ivanovii_subsp_ivanovii_202751_v2	0.898
Listeria_monocytogenes_1639_v142	Listeria_ivanovii_subsp_ivanovii_202751_v2	0.9039
Listeria_monocytogenes_1639_v141	Listeria_ivanovii_subsp_ivanovii_202751_v2	0.8995
Listeria_monocytogenes_1639_v140	Listeria_ivanovii_subsp_ivanovii_202751_v2	0.9043
Listeria_monocytogenes_1639_v13	Listeria_ivanovii_subsp_ivanovii_202751_v2	0.8662
Listeria_monocytogenes_1639_v139	Listeria_ivanovii_subsp_ivanovii_202751_v2	0.8971
Listeria_monocytogenes_1639_v138	Listeria_ivanovii_subsp_ivanovii_202751_v2	0.9036
Listeria_monocytogenes_1639_v137	Listeria_ivanovii_subsp_ivanovii_202751_v2	0.9032
Listeria_monocytogenes_1639_v136	Listeria_ivanovii_subsp_ivanovii_202751_v2	0.8851
Listeria_monocytogenes_1639_v135	Listeria_ivanovii_subsp_ivanovii_202751_v2	0.873
Listeria_monocytogenes_1639_v134	Listeria_ivanovii_subsp_ivanovii_202751_v2	0.8994
Listeria_monocytogenes_1639_v133	Listeria_ivanovii_subsp_ivanovii_202751_v2	0.8915
Listeria_monocytogenes_1639_v132	Listeria_ivanovii_subsp_ivanovii_202751_v2	0.8759
Listeria_monocytogenes_1639_v131	Listeria_ivanovii_subsp_ivanovii_202751_v2	0.8876
Listeria_monocytogenes_1639_v130	Listeria_ivanovii_subsp_ivanovii_202751_v2	0.8793
Listeria_monocytogenes_1639_v12	Listeria_ivanovii_subsp_ivanovii_202751_v2	0.8603
Listeria_monocytogenes_1639_v129	Listeria_ivanovii_subsp_ivanovii_202751_v2	0.9001
Listeria_monocytogenes_1639_v128	Listeria_ivanovii_subsp_ivanovii_202751_v2	0.8958
Listeria_monocytogenes_1639_v127	Listeria_ivanovii_subsp_ivanovii_202751_v2	0.8867
Listeria_monocytogenes_1639_v126	Listeria_ivanovii_subsp_ivanovii_202751_v2	0.8946
Listeria_monocytogenes_1639_v125	Listeria_ivanovii_subsp_ivanovii_202751_v2	0.8885
Listeria_monocytogenes_1639_v124	Listeria_ivanovii_subsp_ivanovii_202751_v2	0.8882
Listeria_monocytogenes_1639_v123	Listeria_ivanovii_subsp_ivanovii_202751_v2	0.9
Listeria_monocytogenes_1639_v122	Listeria_ivanovii_subsp_ivanovii_202751_v2	0.9003
Listeria_monocytogenes_1639_v121	Listeria_ivanovii_subsp_ivanovii_202751_v2	0.9003
Listeria_monocytogenes_1639_v120	Listeria_ivanovii_subsp_ivanovii_202751_v2	0.9
Listeria_monocytogenes_1639_v11	Listeria_ivanovii_subsp_ivanovii_202751_v2	0.8592
Listeria_monocytogenes_1639_v119	Listeria_ivanovii_subsp_ivanovii_202751_v2	0.9003
Listeria_monocytogenes_1639_v118	Listeria_ivanovii_subsp_ivanovii_202751_v2	0.9003
Listeria_monocytogenes_1639_v117	Listeria_ivanovii_subsp_ivanovii_202751_v2	0.8862
Listeria_monocytogenes_1639_v116	Listeria_ivanovii_subsp_ivanovii_202751_v2	0.899
Listeria_monocytogenes_1639_v115	Listeria_ivanovii_subsp_ivanovii_202751_v2	0.8993
Listeria_monocytogenes_1639_v114	Listeria_ivanovii_subsp_ivanovii_202751_v2	0.8798
Listeria_monocytogenes_1639_v113	Listeria_ivanovii_subsp_ivanovii_202751_v2	0.8946
Listeria_monocytogenes_1639_v112	Listeria_ivanovii_subsp_ivanovii_202751_v2	0.8948
Listeria_monocytogenes_1639_v111	Listeria_ivanovii_subsp_ivanovii_202751_v2	0.8914
Listeria_monocytogenes_1639_v110	Listeria_ivanovii_subsp_ivanovii_202751_v2	0.8796
Listeria_monocytogenes_1639_v10	Listeria_ivanovii_subsp_ivanovii_202751_v2	0.8641
Listeria_monocytogenes_1639_v109	Listeria_ivanovii_subsp_ivanovii_202751_v2	0.8792
Listeria_monocytogenes_1639_v108	Listeria_ivanovii_subsp_ivanovii_202751_v2	0.8886
Listeria_monocytogenes_1639_v107	Listeria_ivanovii_subsp_ivanovii_202751_v2	0.8964
Listeria_monocytogenes_1639_v106	Listeria_ivanovii_subsp_ivanovii_202751_v2	0.8968
Listeria_monocytogenes_1639_v105	Listeria_ivanovii_subsp_ivanovii_202751_v2	0.8968
Listeria_monocytogenes_1639_v104	Listeria_ivanovii_subsp_ivanovii_202751_v2	0.8972
Listeria_monocytogenes_1639_v103	Listeria_ivanovii_subsp_ivanovii_202751_v2	0.8972
Listeria_monocytogenes_1639_v102	Listeria_ivanovii_subsp_ivanovii_202751_v2	0.8817
Listeria_monocytogenes_1639_v101	Listeria_ivanovii_subsp_ivanovii_202751_v2	0.8709
Listeria_monocytogenes_1639_v100	Listeria_ivanovii_subsp_ivanovii_202751_v2	0.8703
Listeria_monocytogenes_1639	Listeria_ivanovii_subsp_ivanovii_202751_v2	0.8969
Listeria_monocytogenes_10403S_393133	Listeria_ivanovii_subsp_ivanovii_202751_v2	0.8892
Listeria_monocytogenes_08-5923_637381	Listeria_ivanovii_subsp_ivanovii_202751_v2	0.8715
Listeria_monocytogenes_08-5578_653938	Listeria_ivanovii_subsp_ivanovii_202751_v2	0.8583
Listeria_monocytogenes_07PF0776_1126011	Listeria_ivanovii_subsp_ivanovii_202751_v2	0.9033
Listeria_marthii_FSL_S4-120_702457	Listeria_ivanovii_subsp_ivanovii_202751_v2	0.8275
Listeria_marthii_529731_v1	Listeria_ivanovii_subsp_ivanovii_202751_v2	0.8638
Listeria_marthii_529731	Listeria_ivanovii_subsp_ivanovii_202751_v2	0.8734
Listeria_ivanovii_WSLC3009_1457190	Listeria_ivanovii_subsp_ivanovii_202751_v2	0.9992
Listeria_ivanovii_subsp_londoniensis_202752_v2	Listeria_ivanovii_subsp_ivanovii_202751_v2	0.902
Listeria_ivanovii_subsp_londoniensis_202752_v1	Listeria_ivanovii_subsp_ivanovii_202751_v2	0.8907
Listeria_ivanovii_subsp_londoniensis_202752	Listeria_ivanovii_subsp_ivanovii_202751_v2	0.8974
Listeria_ivanovii_subsp_ivanovii_PAM_55_881621	Listeria_ivanovii_subsp_ivanovii_202751_v2	0.9954
Listeria_ivanovii_subsp_ivanovii_202751_v2	Listeria_ivanovii_subsp_ivanovii_202751_v2	0.0624
Listeria_welshimeri_1643	Listeria_ivanovii_subsp_ivanovii_PAM_55_881621	0.8606
Listeria_welshimeri_serovar_6b_str_SLCC5334_386043	Listeria_ivanovii_subsp_ivanovii_PAM_55_881621	0.8599
Listeria_seeligeri_serovar_1_2b_str_SLCC3954_683837	Listeria_ivanovii_subsp_ivanovii_PAM_55_881621	0.8844
Listeria_seeligeri_FSL_S4-171_702452	Listeria_ivanovii_subsp_ivanovii_PAM_55_881621	0.8179
Listeria_seeligeri_FSL_N1-067_702453	Listeria_ivanovii_subsp_ivanovii_PAM_55_881621	0.8272
Listeria_seeligeri_1640_v1	Listeria_ivanovii_subsp_ivanovii_PAM_55_881621	0.8897
Listeria_seeligeri_1640	Listeria_ivanovii_subsp_ivanovii_PAM_55_881621	0.8783
Listeria_monocytogenes_WSLC1042_1457188	Listeria_ivanovii_subsp_ivanovii_PAM_55_881621	0.897
Listeria_monocytogenes_WSLC1001_1457187	Listeria_ivanovii_subsp_ivanovii_PAM_55_881621	0.8803
Listeria_monocytogenes_SLCC7179_879090	Listeria_ivanovii_subsp_ivanovii_PAM_55_881621	0.8876

Listeria_monocytogenes_SLCC5850_882096	Listeria_ivanovii_subsp_ivanovii_PAM_55_881621	0.8851
Listeria_monocytogenes_SLCC2755_932919	Listeria_ivanovii_subsp_ivanovii_PAM_55_881621	0.8891
Listeria_monocytogenes_SLCC2540_879089	Listeria_ivanovii_subsp_ivanovii_PAM_55_881621	0.889
Listeria_monocytogenes_SLCC2479_882020	Listeria_ivanovii_subsp_ivanovii_PAM_55_881621	0.8785
Listeria_monocytogenes_SLCC2378_879088	Listeria_ivanovii_subsp_ivanovii_PAM_55_881621	0.898
Listeria_monocytogenes_SLCC2376_882097	Listeria_ivanovii_subsp_ivanovii_PAM_55_881621	0.8958
Listeria_monocytogenes_SLCC2372_932920	Listeria_ivanovii_subsp_ivanovii_PAM_55_881621	0.8716
Listeria_monocytogenes_serotype_4b_str_LL195_1230340	Listeria_ivanovii_subsp_ivanovii_PAM_55_881621	0.9038
Listeria_monocytogenes_serotype_4b_str_LIS0087_1134669	Listeria_ivanovii_subsp_ivanovii_PAM_55_881621	0.8609
Listeria_monocytogenes_serotype_4b_str_F2365_265669	Listeria_ivanovii_subsp_ivanovii_PAM_55_881621	0.9038
Listeria_monocytogenes_serotype_4b_str_CLIP_80459_568819	Listeria_ivanovii_subsp_ivanovii_PAM_55_881621	0.9026
Listeria_monocytogenes_serotype_4b_str_81-0861_1196161	Listeria_ivanovii_subsp_ivanovii_PAM_55_881621	0.887
Listeria_monocytogenes_serotype_4b_str_81-0592_1196160	Listeria_ivanovii_subsp_ivanovii_PAM_55_881621	0.8881
Listeria_monocytogenes_serotype_4b_str_81-0558_1196159	Listeria_ivanovii_subsp_ivanovii_PAM_55_881621	0.8891
Listeria_monocytogenes_serotype_4b_str_10-0809_1196162	Listeria_ivanovii_subsp_ivanovii_PAM_55_881621	0.8885
Listeria_monocytogenes_serotype_4b_str_02-6680_1196175	Listeria_ivanovii_subsp_ivanovii_PAM_55_881621	0.9013
Listeria_monocytogenes_serotype_4b_str_02-6679_1196174	Listeria_ivanovii_subsp_ivanovii_PAM_55_881621	0.9013
Listeria_monocytogenes_serotype_4b_str_02-1792_1196173	Listeria_ivanovii_subsp_ivanovii_PAM_55_881621	0.8888
Listeria_monocytogenes_serotype_4b_str_02-1289_1196172	Listeria_ivanovii_subsp_ivanovii_PAM_55_881621	0.8888
Listeria_monocytogenes_serotype_4b_str_02-1103_1196171	Listeria_ivanovii_subsp_ivanovii_PAM_55_881621	0.8888
Listeria_monocytogenes_serotype_3c_str_10-5027_1196195	Listeria_ivanovii_subsp_ivanovii_PAM_55_881621	0.8773
Listeria_monocytogenes_serotype_1_2c_str_10-5026_1196194	Listeria_ivanovii_subsp_ivanovii_PAM_55_881621	0.8784
Listeria_monocytogenes_serotype_1_2c_str_10-5025_1196193	Listeria_ivanovii_subsp_ivanovii_PAM_55_881621	0.8784
Listeria_monocytogenes_serotype_1_2b_str_10-0811_1196164	Listeria_ivanovii_subsp_ivanovii_PAM_55_881621	0.8779
Listeria_monocytogenes_serotype_1_2b_str_10-0810_1196163	Listeria_ivanovii_subsp_ivanovii_PAM_55_881621	0.8779
Listeria_monocytogenes_serotype_1_2a_str_99-6370_1196179	Listeria_ivanovii_subsp_ivanovii_PAM_55_881621	0.8648
Listeria_monocytogenes_serotype_1_2a_str_98-2035_1196178	Listeria_ivanovii_subsp_ivanovii_PAM_55_881621	0.8648
Listeria_monocytogenes_serotype_1_2a_str_95-0093_1196177	Listeria_ivanovii_subsp_ivanovii_PAM_55_881621	0.8699
Listeria_monocytogenes_serotype_1_2a_str_88-0478_1196176	Listeria_ivanovii_subsp_ivanovii_PAM_55_881621	0.8691
Listeria_monocytogenes_serotype_1_2a_str_10-5024_1196192	Listeria_ivanovii_subsp_ivanovii_PAM_55_881621	0.8703
Listeria_monocytogenes_serotype_1_2a_str_10-4758_1196168	Listeria_ivanovii_subsp_ivanovii_PAM_55_881621	0.8845
Listeria_monocytogenes_serotype_1_2a_str_10-4754_1196167	Listeria_ivanovii_subsp_ivanovii_PAM_55_881621	0.8845
Listeria_monocytogenes_serotype_1_2a_str_10-1321_1196191	Listeria_ivanovii_subsp_ivanovii_PAM_55_881621	0.8703
Listeria_monocytogenes_serotype_1_2a_str_10-1047_1196190	Listeria_ivanovii_subsp_ivanovii_PAM_55_881621	0.8706
Listeria_monocytogenes_serotype_1_2a_str_10-1046_1196189	Listeria_ivanovii_subsp_ivanovii_PAM_55_881621	0.8706
Listeria_monocytogenes_serotype_1_2a_str_10-0934_1196170	Listeria_ivanovii_subsp_ivanovii_PAM_55_881621	0.8946
Listeria_monocytogenes_serotype_1_2a_str_10-0933_1196169	Listeria_ivanovii_subsp_ivanovii_PAM_55_881621	0.8946
Listeria_monocytogenes_serotype_1_2a_str_10-0815_1196188	Listeria_ivanovii_subsp_ivanovii_PAM_55_881621	0.8661
Listeria_monocytogenes_serotype_1_2a_str_10-0814_1196187	Listeria_ivanovii_subsp_ivanovii_PAM_55_881621	0.8703
Listeria_monocytogenes_serotype_1_2a_str_10-0813_1196166	Listeria_ivanovii_subsp_ivanovii_PAM_55_881621	0.8777
Listeria_monocytogenes_serotype_1_2a_str_10-0812_1196165	Listeria_ivanovii_subsp_ivanovii_PAM_55_881621	0.8777
Listeria_monocytogenes_serotype_1_2a_str_08-7669_1196186	Listeria_ivanovii_subsp_ivanovii_PAM_55_881621	0.8784
Listeria_monocytogenes_serotype_1_2a_str_08-7374_1196185	Listeria_ivanovii_subsp_ivanovii_PAM_55_881621	0.8703
Listeria_monocytogenes_serotype_1_2a_str_08-6997_1196184	Listeria_ivanovii_subsp_ivanovii_PAM_55_881621	0.8661
Listeria_monocytogenes_serotype_1_2a_str_08-6569_1196183	Listeria_ivanovii_subsp_ivanovii_PAM_55_881621	0.8661
Listeria_monocytogenes_serotype_1_2a_str_08-6056_1196182	Listeria_ivanovii_subsp_ivanovii_PAM_55_881621	0.8661
Listeria_monocytogenes_serotype_1_2a_str_04-5457_1196181	Listeria_ivanovii_subsp_ivanovii_PAM_55_881621	0.8703
Listeria_monocytogenes_serotype_1_2a_str_02-5993_1196180	Listeria_ivanovii_subsp_ivanovii_PAM_55_881621	0.8699
Listeria_monocytogenes_R479a_1437838	Listeria_ivanovii_subsp_ivanovii_PAM_55_881621	0.8697
Listeria_monocytogenes_N53-1_1234142	Listeria_ivanovii_subsp_ivanovii_PAM_55_881621	0.8492
Listeria_monocytogenes_M7_1030009	Listeria_ivanovii_subsp_ivanovii_PAM_55_881621	0.8619
Listeria_monocytogenes_LO28_393132	Listeria_ivanovii_subsp_ivanovii_PAM_55_881621	0.8772
Listeria_monocytogenes_L99_563174	Listeria_ivanovii_subsp_ivanovii_PAM_55_881621	0.8625
Listeria_monocytogenes_L312_882094	Listeria_ivanovii_subsp_ivanovii_PAM_55_881621	0.9026
Listeria_monocytogenes_J1816_930781	Listeria_ivanovii_subsp_ivanovii_PAM_55_881621	0.8802
Listeria_monocytogenes_J1-220_930782	Listeria_ivanovii_subsp_ivanovii_PAM_55_881621	0.8773
Listeria_monocytogenes_J0161_393130	Listeria_ivanovii_subsp_ivanovii_PAM_55_881621	0.8798
Listeria_monocytogenes_HCC23_552536	Listeria_ivanovii_subsp_ivanovii_PAM_55_881621	0.8622
Listeria_monocytogenes_FSL_R2-561_393126	Listeria_ivanovii_subsp_ivanovii_PAM_55_881621	0.8767
Listeria_monocytogenes_FSL_J1-208_393119	Listeria_ivanovii_subsp_ivanovii_PAM_55_881621	0.8732
Listeria_monocytogenes_FSL_J1-175_393118	Listeria_ivanovii_subsp_ivanovii_PAM_55_881621	0.8931
Listeria_monocytogenes_Finland_1998_393127	Listeria_ivanovii_subsp_ivanovii_PAM_55_881621	0.8988
Listeria_monocytogenes_EGD-e_169963_v1	Listeria_ivanovii_subsp_ivanovii_PAM_55_881621	0.885
Listeria_monocytogenes_EGD-e_169963	Listeria_ivanovii_subsp_ivanovii_PAM_55_881621	0.8854
Listeria_monocytogenes_EGD_1334565	Listeria_ivanovii_subsp_ivanovii_PAM_55_881621	0.8854
Listeria_monocytogenes_CFSAN002349_1299895	Listeria_ivanovii_subsp_ivanovii_PAM_55_881621	0.8983
Listeria_monocytogenes_ATCC_19117_882095_v1	Listeria_ivanovii_subsp_ivanovii_PAM_55_881621	0.8962
Listeria_monocytogenes_ATCC_19117_882095	Listeria_ivanovii_subsp_ivanovii_PAM_55_881621	0.8961
Listeria_monocytogenes_6179_1288295	Listeria_ivanovii_subsp_ivanovii_PAM_55_881621	0.8644
Listeria_monocytogenes_1639_v9	Listeria_ivanovii_subsp_ivanovii_PAM_55_881621	0.8659
Listeria_monocytogenes_1639_v99	Listeria_ivanovii_subsp_ivanovii_PAM_55_881621	0.8799
Listeria_monocytogenes_1639_v98	Listeria_ivanovii_subsp_ivanovii_PAM_55_881621	0.8758
Listeria_monocytogenes_1639_v97	Listeria_ivanovii_subsp_ivanovii_PAM_55_881621	0.8915
Listeria_monocytogenes_1639_v96	Listeria_ivanovii_subsp_ivanovii_PAM_55_881621	0.8792
Listeria_monocytogenes_1639_v95	Listeria_ivanovii_subsp_ivanovii_PAM_55_881621	0.8793
Listeria_monocytogenes_1639_v94	Listeria_ivanovii_subsp_ivanovii_PAM_55_881621	0.8792
Listeria_monocytogenes_1639_v93	Listeria_ivanovii_subsp_ivanovii_PAM_55_881621	0.8751
Listeria_monocytogenes_1639_v92	Listeria_ivanovii_subsp_ivanovii_PAM_55_881621	0.8796
Listeria_monocytogenes_1639_v91	Listeria_ivanovii_subsp_ivanovii_PAM_55_881621	0.8861
Listeria_monocytogenes_1639_v90	Listeria_ivanovii_subsp_ivanovii_PAM_55_881621	0.8758
Listeria_monocytogenes_1639_v8	Listeria_ivanovii_subsp_ivanovii_PAM_55_881621	0.8596
Listeria_monocytogenes_1639_v89	Listeria_ivanovii_subsp_ivanovii_PAM_55_881621	0.8921
Listeria_monocytogenes_1639_v88	Listeria_ivanovii_subsp_ivanovii_PAM_55_881621	0.877
Listeria_monocytogenes_1639_v87	Listeria_ivanovii_subsp_ivanovii_PAM_55_881621	0.8726
Listeria_monocytogenes_1639_v86	Listeria_ivanovii_subsp_ivanovii_PAM_55_881621	0.8726
Listeria_monocytogenes_1639_v85	Listeria_ivanovii_subsp_ivanovii_PAM_55_881621	0.8868
Listeria_monocytogenes_1639_v84	Listeria_ivanovii_subsp_ivanovii_PAM_55_881621	0.879
Listeria_monocytogenes_1639_v83	Listeria_ivanovii_subsp_ivanovii_PAM_55_881621	0.8856
Listeria_monocytogenes_1639_v82	Listeria_ivanovii_subsp_ivanovii_PAM_55_881621	0.8854
Listeria_monocytogenes_1639_v81	Listeria_ivanovii_subsp_ivanovii_PAM_55_881621	0.8804
Listeria_monocytogenes_1639_v80	Listeria_ivanovii_subsp_ivanovii_PAM_55_881621	0.8824

Listeria_monocytogenes_1639_v7	Listeria_ivanovii_subsp_ivanovii_PAM_55_881621	0.8596
Listeria_monocytogenes_1639_v79	Listeria_ivanovii_subsp_ivanovii_PAM_55_881621	0.8784
Listeria_monocytogenes_1639_v78	Listeria_ivanovii_subsp_ivanovii_PAM_55_881621	0.8797
Listeria_monocytogenes_1639_v77	Listeria_ivanovii_subsp_ivanovii_PAM_55_881621	0.8785
Listeria_monocytogenes_1639_v76	Listeria_ivanovii_subsp_ivanovii_PAM_55_881621	0.869
Listeria_monocytogenes_1639_v75	Listeria_ivanovii_subsp_ivanovii_PAM_55_881621	0.8625
Listeria_monocytogenes_1639_v74	Listeria_ivanovii_subsp_ivanovii_PAM_55_881621	0.8734
Listeria_monocytogenes_1639_v73	Listeria_ivanovii_subsp_ivanovii_PAM_55_881621	0.8726
Listeria_monocytogenes_1639_v72	Listeria_ivanovii_subsp_ivanovii_PAM_55_881621	0.8848
Listeria_monocytogenes_1639_v71	Listeria_ivanovii_subsp_ivanovii_PAM_55_881621	0.8665
Listeria_monocytogenes_1639_v70	Listeria_ivanovii_subsp_ivanovii_PAM_55_881621	0.8752
Listeria_monocytogenes_1639_v6	Listeria_ivanovii_subsp_ivanovii_PAM_55_881621	0.859
Listeria_monocytogenes_1639_v69	Listeria_ivanovii_subsp_ivanovii_PAM_55_881621	0.8884
Listeria_monocytogenes_1639_v68	Listeria_ivanovii_subsp_ivanovii_PAM_55_881621	0.8838
Listeria_monocytogenes_1639_v67	Listeria_ivanovii_subsp_ivanovii_PAM_55_881621	0.8853
Listeria_monocytogenes_1639_v66	Listeria_ivanovii_subsp_ivanovii_PAM_55_881621	0.8869
Listeria_monocytogenes_1639_v65	Listeria_ivanovii_subsp_ivanovii_PAM_55_881621	0.8733
Listeria_monocytogenes_1639_v64	Listeria_ivanovii_subsp_ivanovii_PAM_55_881621	0.876
Listeria_monocytogenes_1639_v63	Listeria_ivanovii_subsp_ivanovii_PAM_55_881621	0.8789
Listeria_monocytogenes_1639_v62	Listeria_ivanovii_subsp_ivanovii_PAM_55_881621	0.8764
Listeria_monocytogenes_1639_v61	Listeria_ivanovii_subsp_ivanovii_PAM_55_881621	0.8728
Listeria_monocytogenes_1639_v60	Listeria_ivanovii_subsp_ivanovii_PAM_55_881621	0.8811
Listeria_monocytogenes_1639_v5	Listeria_ivanovii_subsp_ivanovii_PAM_55_881621	0.847
Listeria_monocytogenes_1639_v59	Listeria_ivanovii_subsp_ivanovii_PAM_55_881621	0.8863
Listeria_monocytogenes_1639_v58	Listeria_ivanovii_subsp_ivanovii_PAM_55_881621	0.8886
Listeria_monocytogenes_1639_v57	Listeria_ivanovii_subsp_ivanovii_PAM_55_881621	0.8764
Listeria_monocytogenes_1639_v56	Listeria_ivanovii_subsp_ivanovii_PAM_55_881621	0.886
Listeria_monocytogenes_1639_v55	Listeria_ivanovii_subsp_ivanovii_PAM_55_881621	0.8723
Listeria_monocytogenes_1639_v54	Listeria_ivanovii_subsp_ivanovii_PAM_55_881621	0.8913
Listeria_monocytogenes_1639_v53	Listeria_ivanovii_subsp_ivanovii_PAM_55_881621	0.8792
Listeria_monocytogenes_1639_v52	Listeria_ivanovii_subsp_ivanovii_PAM_55_881621	0.883
Listeria_monocytogenes_1639_v51	Listeria_ivanovii_subsp_ivanovii_PAM_55_881621	0.8814
Listeria_monocytogenes_1639_v50	Listeria_ivanovii_subsp_ivanovii_PAM_55_881621	0.8691
Listeria_monocytogenes_1639_v4	Listeria_ivanovii_subsp_ivanovii_PAM_55_881621	0.8583
Listeria_monocytogenes_1639_v49	Listeria_ivanovii_subsp_ivanovii_PAM_55_881621	0.8885
Listeria_monocytogenes_1639_v48	Listeria_ivanovii_subsp_ivanovii_PAM_55_881621	0.8728
Listeria_monocytogenes_1639_v47	Listeria_ivanovii_subsp_ivanovii_PAM_55_881621	0.8699
Listeria_monocytogenes_1639_v46	Listeria_ivanovii_subsp_ivanovii_PAM_55_881621	0.8831
Listeria_monocytogenes_1639_v45	Listeria_ivanovii_subsp_ivanovii_PAM_55_881621	0.8613
Listeria_monocytogenes_1639_v44	Listeria_ivanovii_subsp_ivanovii_PAM_55_881621	0.8703
Listeria_monocytogenes_1639_v43	Listeria_ivanovii_subsp_ivanovii_PAM_55_881621	0.8703
Listeria_monocytogenes_1639_v42	Listeria_ivanovii_subsp_ivanovii_PAM_55_881621	0.8643
Listeria_monocytogenes_1639_v41	Listeria_ivanovii_subsp_ivanovii_PAM_55_881621	0.8537
Listeria_monocytogenes_1639_v40	Listeria_ivanovii_subsp_ivanovii_PAM_55_881621	0.8779
Listeria_monocytogenes_1639_v3	Listeria_ivanovii_subsp_ivanovii_PAM_55_881621	0.8587
Listeria_monocytogenes_1639_v39	Listeria_ivanovii_subsp_ivanovii_PAM_55_881621	0.881
Listeria_monocytogenes_1639_v38	Listeria_ivanovii_subsp_ivanovii_PAM_55_881621	0.8741
Listeria_monocytogenes_1639_v37	Listeria_ivanovii_subsp_ivanovii_PAM_55_881621	0.8703
Listeria_monocytogenes_1639_v36	Listeria_ivanovii_subsp_ivanovii_PAM_55_881621	0.8698
Listeria_monocytogenes_1639_v35	Listeria_ivanovii_subsp_ivanovii_PAM_55_881621	0.8785
Listeria_monocytogenes_1639_v34	Listeria_ivanovii_subsp_ivanovii_PAM_55_881621	0.8758
Listeria_monocytogenes_1639_v33	Listeria_ivanovii_subsp_ivanovii_PAM_55_881621	0.8779
Listeria_monocytogenes_1639_v32	Listeria_ivanovii_subsp_ivanovii_PAM_55_881621	0.8689
Listeria_monocytogenes_1639_v31	Listeria_ivanovii_subsp_ivanovii_PAM_55_881621	0.8761
Listeria_monocytogenes_1639_v30	Listeria_ivanovii_subsp_ivanovii_PAM_55_881621	0.8818
Listeria_monocytogenes_1639_v2	Listeria_ivanovii_subsp_ivanovii_PAM_55_881621	0.8509
Listeria_monocytogenes_1639_v29	Listeria_ivanovii_subsp_ivanovii_PAM_55_881621	0.8648
Listeria_monocytogenes_1639_v28	Listeria_ivanovii_subsp_ivanovii_PAM_55_881621	0.8689
Listeria_monocytogenes_1639_v27	Listeria_ivanovii_subsp_ivanovii_PAM_55_881621	0.861
Listeria_monocytogenes_1639_v26	Listeria_ivanovii_subsp_ivanovii_PAM_55_881621	0.8611
Listeria_monocytogenes_1639_v25	Listeria_ivanovii_subsp_ivanovii_PAM_55_881621	0.8608
Listeria_monocytogenes_1639_v24	Listeria_ivanovii_subsp_ivanovii_PAM_55_881621	0.8777
Listeria_monocytogenes_1639_v23	Listeria_ivanovii_subsp_ivanovii_PAM_55_881621	0.8777
Listeria_monocytogenes_1639_v22	Listeria_ivanovii_subsp_ivanovii_PAM_55_881621	0.8788
Listeria_monocytogenes_1639_v21	Listeria_ivanovii_subsp_ivanovii_PAM_55_881621	0.8774
Listeria_monocytogenes_1639_v20	Listeria_ivanovii_subsp_ivanovii_PAM_55_881621	0.8775
Listeria_monocytogenes_1639_v1	Listeria_ivanovii_subsp_ivanovii_PAM_55_881621	0.8581
Listeria_monocytogenes_1639_v19	Listeria_ivanovii_subsp_ivanovii_PAM_55_881621	0.8771
Listeria_monocytogenes_1639_v198	Listeria_ivanovii_subsp_ivanovii_PAM_55_881621	0.8523
Listeria_monocytogenes_1639_v197	Listeria_ivanovii_subsp_ivanovii_PAM_55_881621	0.8736
Listeria_monocytogenes_1639_v196	Listeria_ivanovii_subsp_ivanovii_PAM_55_881621	0.8676
Listeria_monocytogenes_1639_v195	Listeria_ivanovii_subsp_ivanovii_PAM_55_881621	0.8949
Listeria_monocytogenes_1639_v194	Listeria_ivanovii_subsp_ivanovii_PAM_55_881621	0.8961
Listeria_monocytogenes_1639_v193	Listeria_ivanovii_subsp_ivanovii_PAM_55_881621	0.8965
Listeria_monocytogenes_1639_v192	Listeria_ivanovii_subsp_ivanovii_PAM_55_881621	0.8655
Listeria_monocytogenes_1639_v191	Listeria_ivanovii_subsp_ivanovii_PAM_55_881621	0.8997
Listeria_monocytogenes_1639_v190	Listeria_ivanovii_subsp_ivanovii_PAM_55_881621	0.8959
Listeria_monocytogenes_1639_v18	Listeria_ivanovii_subsp_ivanovii_PAM_55_881621	0.8708
Listeria_monocytogenes_1639_v189	Listeria_ivanovii_subsp_ivanovii_PAM_55_881621	0.8956
Listeria_monocytogenes_1639_v188	Listeria_ivanovii_subsp_ivanovii_PAM_55_881621	0.8984
Listeria_monocytogenes_1639_v187	Listeria_ivanovii_subsp_ivanovii_PAM_55_881621	0.8959
Listeria_monocytogenes_1639_v186	Listeria_ivanovii_subsp_ivanovii_PAM_55_881621	0.8962
Listeria_monocytogenes_1639_v185	Listeria_ivanovii_subsp_ivanovii_PAM_55_881621	0.8959
Listeria_monocytogenes_1639_v184	Listeria_ivanovii_subsp_ivanovii_PAM_55_881621	0.8955
Listeria_monocytogenes_1639_v183	Listeria_ivanovii_subsp_ivanovii_PAM_55_881621	0.8971
Listeria_monocytogenes_1639_v182	Listeria_ivanovii_subsp_ivanovii_PAM_55_881621	0.8988
Listeria_monocytogenes_1639_v181	Listeria_ivanovii_subsp_ivanovii_PAM_55_881621	0.8832
Listeria_monocytogenes_1639_v180	Listeria_ivanovii_subsp_ivanovii_PAM_55_881621	0.8832
Listeria_monocytogenes_1639_v17	Listeria_ivanovii_subsp_ivanovii_PAM_55_881621	0.8708
Listeria_monocytogenes_1639_v179	Listeria_ivanovii_subsp_ivanovii_PAM_55_881621	0.8889
Listeria_monocytogenes_1639_v178	Listeria_ivanovii_subsp_ivanovii_PAM_55_881621	0.9002

Listeria_monocytogenes_1639_v177	Listeria_ivanovii_subsp_ivanovii_PAM_55_881621	0.8967
Listeria_monocytogenes_1639_v176	Listeria_ivanovii_subsp_ivanovii_PAM_55_881621	0.8948
Listeria_monocytogenes_1639_v175	Listeria_ivanovii_subsp_ivanovii_PAM_55_881621	0.888
Listeria_monocytogenes_1639_v174	Listeria_ivanovii_subsp_ivanovii_PAM_55_881621	0.8999
Listeria_monocytogenes_1639_v173	Listeria_ivanovii_subsp_ivanovii_PAM_55_881621	0.9034
Listeria_monocytogenes_1639_v172	Listeria_ivanovii_subsp_ivanovii_PAM_55_881621	0.8916
Listeria_monocytogenes_1639_v171	Listeria_ivanovii_subsp_ivanovii_PAM_55_881621	0.8819
Listeria_monocytogenes_1639_v170	Listeria_ivanovii_subsp_ivanovii_PAM_55_881621	0.8894
Listeria_monocytogenes_1639_v16	Listeria_ivanovii_subsp_ivanovii_PAM_55_881621	0.8575
Listeria_monocytogenes_1639_v169	Listeria_ivanovii_subsp_ivanovii_PAM_55_881621	0.8895
Listeria_monocytogenes_1639_v168	Listeria_ivanovii_subsp_ivanovii_PAM_55_881621	0.8981
Listeria_monocytogenes_1639_v167	Listeria_ivanovii_subsp_ivanovii_PAM_55_881621	0.8893
Listeria_monocytogenes_1639_v166	Listeria_ivanovii_subsp_ivanovii_PAM_55_881621	0.8985
Listeria_monocytogenes_1639_v165	Listeria_ivanovii_subsp_ivanovii_PAM_55_881621	0.8891
Listeria_monocytogenes_1639_v164	Listeria_ivanovii_subsp_ivanovii_PAM_55_881621	0.9049
Listeria_monocytogenes_1639_v163	Listeria_ivanovii_subsp_ivanovii_PAM_55_881621	0.8996
Listeria_monocytogenes_1639_v162	Listeria_ivanovii_subsp_ivanovii_PAM_55_881621	0.9049
Listeria_monocytogenes_1639_v161	Listeria_ivanovii_subsp_ivanovii_PAM_55_881621	0.9042
Listeria_monocytogenes_1639_v160	Listeria_ivanovii_subsp_ivanovii_PAM_55_881621	0.9046
Listeria_monocytogenes_1639_v15	Listeria_ivanovii_subsp_ivanovii_PAM_55_881621	0.8647
Listeria_monocytogenes_1639_v159	Listeria_ivanovii_subsp_ivanovii_PAM_55_881621	0.8967
Listeria_monocytogenes_1639_v158	Listeria_ivanovii_subsp_ivanovii_PAM_55_881621	0.9038
Listeria_monocytogenes_1639_v157	Listeria_ivanovii_subsp_ivanovii_PAM_55_881621	0.9038
Listeria_monocytogenes_1639_v156	Listeria_ivanovii_subsp_ivanovii_PAM_55_881621	0.875
Listeria_monocytogenes_1639_v155	Listeria_ivanovii_subsp_ivanovii_PAM_55_881621	0.8874
Listeria_monocytogenes_1639_v154	Listeria_ivanovii_subsp_ivanovii_PAM_55_881621	0.8879
Listeria_monocytogenes_1639_v153	Listeria_ivanovii_subsp_ivanovii_PAM_55_881621	0.8878
Listeria_monocytogenes_1639_v152	Listeria_ivanovii_subsp_ivanovii_PAM_55_881621	0.8971
Listeria_monocytogenes_1639_v151	Listeria_ivanovii_subsp_ivanovii_PAM_55_881621	0.8971
Listeria_monocytogenes_1639_v150	Listeria_ivanovii_subsp_ivanovii_PAM_55_881621	0.8867
Listeria_monocytogenes_1639_v14	Listeria_ivanovii_subsp_ivanovii_PAM_55_881621	0.87
Listeria_monocytogenes_1639_v149	Listeria_ivanovii_subsp_ivanovii_PAM_55_881621	0.8854
Listeria_monocytogenes_1639_v148	Listeria_ivanovii_subsp_ivanovii_PAM_55_881621	0.8957
Listeria_monocytogenes_1639_v147	Listeria_ivanovii_subsp_ivanovii_PAM_55_881621	0.8836
Listeria_monocytogenes_1639_v146	Listeria_ivanovii_subsp_ivanovii_PAM_55_881621	0.9001
Listeria_monocytogenes_1639_v145	Listeria_ivanovii_subsp_ivanovii_PAM_55_881621	0.8915
Listeria_monocytogenes_1639_v144	Listeria_ivanovii_subsp_ivanovii_PAM_55_881621	0.9026
Listeria_monocytogenes_1639_v143	Listeria_ivanovii_subsp_ivanovii_PAM_55_881621	0.8994
Listeria_monocytogenes_1639_v142	Listeria_ivanovii_subsp_ivanovii_PAM_55_881621	0.9026
Listeria_monocytogenes_1639_v141	Listeria_ivanovii_subsp_ivanovii_PAM_55_881621	0.8978
Listeria_monocytogenes_1639_v140	Listeria_ivanovii_subsp_ivanovii_PAM_55_881621	0.9026
Listeria_monocytogenes_1639_v13	Listeria_ivanovii_subsp_ivanovii_PAM_55_881621	0.8651
Listeria_monocytogenes_1639_v139	Listeria_ivanovii_subsp_ivanovii_PAM_55_881621	0.8954
Listeria_monocytogenes_1639_v138	Listeria_ivanovii_subsp_ivanovii_PAM_55_881621	0.9023
Listeria_monocytogenes_1639_v137	Listeria_ivanovii_subsp_ivanovii_PAM_55_881621	0.9015
Listeria_monocytogenes_1639_v136	Listeria_ivanovii_subsp_ivanovii_PAM_55_881621	0.8838
Listeria_monocytogenes_1639_v135	Listeria_ivanovii_subsp_ivanovii_PAM_55_881621	0.874
Listeria_monocytogenes_1639_v134	Listeria_ivanovii_subsp_ivanovii_PAM_55_881621	0.8977
Listeria_monocytogenes_1639_v133	Listeria_ivanovii_subsp_ivanovii_PAM_55_881621	0.8933
Listeria_monocytogenes_1639_v132	Listeria_ivanovii_subsp_ivanovii_PAM_55_881621	0.8747
Listeria_monocytogenes_1639_v131	Listeria_ivanovii_subsp_ivanovii_PAM_55_881621	0.8859
Listeria_monocytogenes_1639_v130	Listeria_ivanovii_subsp_ivanovii_PAM_55_881621	0.8785
Listeria_monocytogenes_1639_v12	Listeria_ivanovii_subsp_ivanovii_PAM_55_881621	0.8591
Listeria_monocytogenes_1639_v129	Listeria_ivanovii_subsp_ivanovii_PAM_55_881621	0.8984
Listeria_monocytogenes_1639_v128	Listeria_ivanovii_subsp_ivanovii_PAM_55_881621	0.8941
Listeria_monocytogenes_1639_v127	Listeria_ivanovii_subsp_ivanovii_PAM_55_881621	0.8885
Listeria_monocytogenes_1639_v126	Listeria_ivanovii_subsp_ivanovii_PAM_55_881621	0.8929
Listeria_monocytogenes_1639_v125	Listeria_ivanovii_subsp_ivanovii_PAM_55_881621	0.8899
Listeria_monocytogenes_1639_v124	Listeria_ivanovii_subsp_ivanovii_PAM_55_881621	0.8865
Listeria_monocytogenes_1639_v123	Listeria_ivanovii_subsp_ivanovii_PAM_55_881621	0.8983
Listeria_monocytogenes_1639_v122	Listeria_ivanovii_subsp_ivanovii_PAM_55_881621	0.8987
Listeria_monocytogenes_1639_v121	Listeria_ivanovii_subsp_ivanovii_PAM_55_881621	0.8987
Listeria_monocytogenes_1639_v120	Listeria_ivanovii_subsp_ivanovii_PAM_55_881621	0.8987
Listeria_monocytogenes_1639_v11	Listeria_ivanovii_subsp_ivanovii_PAM_55_881621	0.8606
Listeria_monocytogenes_1639_v119	Listeria_ivanovii_subsp_ivanovii_PAM_55_881621	0.8987
Listeria_monocytogenes_1639_v118	Listeria_ivanovii_subsp_ivanovii_PAM_55_881621	0.8987
Listeria_monocytogenes_1639_v117	Listeria_ivanovii_subsp_ivanovii_PAM_55_881621	0.8876
Listeria_monocytogenes_1639_v116	Listeria_ivanovii_subsp_ivanovii_PAM_55_881621	0.8973
Listeria_monocytogenes_1639_v115	Listeria_ivanovii_subsp_ivanovii_PAM_55_881621	0.8976
Listeria_monocytogenes_1639_v114	Listeria_ivanovii_subsp_ivanovii_PAM_55_881621	0.8786
Listeria_monocytogenes_1639_v113	Listeria_ivanovii_subsp_ivanovii_PAM_55_881621	0.8929
Listeria_monocytogenes_1639_v112	Listeria_ivanovii_subsp_ivanovii_PAM_55_881621	0.8935
Listeria_monocytogenes_1639_v111	Listeria_ivanovii_subsp_ivanovii_PAM_55_881621	0.8924
Listeria_monocytogenes_1639_v110	Listeria_ivanovii_subsp_ivanovii_PAM_55_881621	0.8783
Listeria_monocytogenes_1639_v10	Listeria_ivanovii_subsp_ivanovii_PAM_55_881621	0.8625
Listeria_monocytogenes_1639_v109	Listeria_ivanovii_subsp_ivanovii_PAM_55_881621	0.8779
Listeria_monocytogenes_1639_v108	Listeria_ivanovii_subsp_ivanovii_PAM_55_881621	0.8869
Listeria_monocytogenes_1639_v107	Listeria_ivanovii_subsp_ivanovii_PAM_55_881621	0.8951
Listeria_monocytogenes_1639_v106	Listeria_ivanovii_subsp_ivanovii_PAM_55_881621	0.8951
Listeria_monocytogenes_1639_v105	Listeria_ivanovii_subsp_ivanovii_PAM_55_881621	0.8955
Listeria_monocytogenes_1639_v104	Listeria_ivanovii_subsp_ivanovii_PAM_55_881621	0.8955
Listeria_monocytogenes_1639_v103	Listeria_ivanovii_subsp_ivanovii_PAM_55_881621	0.8955
Listeria_monocytogenes_1639_v102	Listeria_ivanovii_subsp_ivanovii_PAM_55_881621	0.8805
Listeria_monocytogenes_1639_v101	Listeria_ivanovii_subsp_ivanovii_PAM_55_881621	0.8697
Listeria_monocytogenes_1639_v100	Listeria_ivanovii_subsp_ivanovii_PAM_55_881621	0.8691
Listeria_monocytogenes_1639	Listeria_ivanovii_subsp_ivanovii_PAM_55_881621	0.8956
Listeria_monocytogenes_10403S_393133	Listeria_ivanovii_subsp_ivanovii_PAM_55_881621	0.888
Listeria_monocytogenes_08-5923_637381	Listeria_ivanovii_subsp_ivanovii_PAM_55_881621	0.8703
Listeria_monocytogenes_08-5578_653938	Listeria_ivanovii_subsp_ivanovii_PAM_55_881621	0.8571
Listeria_monocytogenes_07PF0776_1126011	Listeria_ivanovii_subsp_ivanovii_PAM_55_881621	0.902
Listeria_marthii_FSL_S4-120_702457	Listeria_ivanovii_subsp_ivanovii_PAM_55_881621	0.8295

Listeria_marthii_529731_v1	Listeria_ivanovii_subsp_ivanovii_PAM_55_881621	0.8629
Listeria_marthii_529731	Listeria_ivanovii_subsp_ivanovii_PAM_55_881621	0.8725
Listeria_ivanovii_WSLC3009_1457190	Listeria_ivanovii_subsp_ivanovii_PAM_55_881621	0.9946
Listeria_ivanovii_subsp_londoniensis_202752_v2	Listeria_ivanovii_subsp_ivanovii_PAM_55_881621	0.9011
Listeria_ivanovii_subsp_londoniensis_202752_v1	Listeria_ivanovii_subsp_ivanovii_PAM_55_881621	0.8924
Listeria_ivanovii_subsp_londoniensis_202752	Listeria_ivanovii_subsp_ivanovii_PAM_55_881621	0.8965
Listeria_ivanovii_subsp_ivanovii_PAM_55_881621	Listeria_ivanovii_subsp_ivanovii_PAM_55_881621	0.0644
Listeria_welshimeri_1643	Listeria_ivanovii_subsp_londoniensis_202752	0.8654
Listeria_welshimeri_serovar_6b_str_SLCC5334_386043	Listeria_ivanovii_subsp_londoniensis_202752	0.8651
Listeria_seeligeri_serovar_1_2b_str_SLCC3954_683837	Listeria_ivanovii_subsp_londoniensis_202752	0.8782
Listeria_seeligeri_FSL_S4-171_702452	Listeria_ivanovii_subsp_londoniensis_202752	0.8271
Listeria_seeligeri_FSL_N1-067_702453	Listeria_ivanovii_subsp_londoniensis_202752	0.8348
Listeria_seeligeri_1640_v1	Listeria_ivanovii_subsp_londoniensis_202752	0.8928
Listeria_seeligeri_1640	Listeria_ivanovii_subsp_londoniensis_202752	0.8787
Listeria_monocytogenes_WSLC1042_1457188	Listeria_ivanovii_subsp_londoniensis_202752	0.8829
Listeria_monocytogenes_WSLC1001_1457187	Listeria_ivanovii_subsp_londoniensis_202752	0.8739
Listeria_monocytogenes_SLCC7179_879090	Listeria_ivanovii_subsp_londoniensis_202752	0.8808
Listeria_monocytogenes_SLCC5850_882096	Listeria_ivanovii_subsp_londoniensis_202752	0.8786
Listeria_monocytogenes_SLCC2755_932919	Listeria_ivanovii_subsp_londoniensis_202752	0.8763
Listeria_monocytogenes_SLCC2540_879089	Listeria_ivanovii_subsp_londoniensis_202752	0.8765
Listeria_monocytogenes_SLCC2479_882020	Listeria_ivanovii_subsp_londoniensis_202752	0.8759
Listeria_monocytogenes_SLCC2378_879088	Listeria_ivanovii_subsp_londoniensis_202752	0.8839
Listeria_monocytogenes_SLCC2376_882097	Listeria_ivanovii_subsp_londoniensis_202752	0.8837
Listeria_monocytogenes_SLCC2372_932920	Listeria_ivanovii_subsp_londoniensis_202752	0.8698
Listeria_monocytogenes_serotype_4b_str_LL195_1230340	Listeria_ivanovii_subsp_londoniensis_202752	0.8892
Listeria_monocytogenes_serotype_4b_str_LIS0087_1134669	Listeria_ivanovii_subsp_londoniensis_202752	0.8553
Listeria_monocytogenes_serotype_4b_str_F2365_265669	Listeria_ivanovii_subsp_londoniensis_202752	0.8892
Listeria_monocytogenes_serotype_4b_str_CLIP_80459_568819	Listeria_ivanovii_subsp_londoniensis_202752	0.8899
Listeria_monocytogenes_serotype_4b_str_81-0861_1196161	Listeria_ivanovii_subsp_londoniensis_202752	0.8761
Listeria_monocytogenes_serotype_4b_str_81-0592_1196160	Listeria_ivanovii_subsp_londoniensis_202752	0.8772
Listeria_monocytogenes_serotype_4b_str_81-0558_1196159	Listeria_ivanovii_subsp_londoniensis_202752	0.8779
Listeria_monocytogenes_serotype_4b_str_10-0809_1196162	Listeria_ivanovii_subsp_londoniensis_202752	0.8775
Listeria_monocytogenes_serotype_4b_str_02-6680_1196175	Listeria_ivanovii_subsp_londoniensis_202752	0.8905
Listeria_monocytogenes_serotype_4b_str_02-6679_1196174	Listeria_ivanovii_subsp_londoniensis_202752	0.8905
Listeria_monocytogenes_serotype_4b_str_02-1792_1196173	Listeria_ivanovii_subsp_londoniensis_202752	0.8779
Listeria_monocytogenes_serotype_4b_str_02-1289_1196172	Listeria_ivanovii_subsp_londoniensis_202752	0.8779
Listeria_monocytogenes_serotype_4b_str_02-1103_1196171	Listeria_ivanovii_subsp_londoniensis_202752	0.8779
Listeria_monocytogenes_serotype_3c_str_10-5027_1196195	Listeria_ivanovii_subsp_londoniensis_202752	0.8755
Listeria_monocytogenes_serotype_1_2c_str_10-5026_1196194	Listeria_ivanovii_subsp_londoniensis_202752	0.8763
Listeria_monocytogenes_serotype_1_2c_str_10-5025_1196193	Listeria_ivanovii_subsp_londoniensis_202752	0.8763
Listeria_monocytogenes_serotype_1_2b_str_10-0811_1196164	Listeria_ivanovii_subsp_londoniensis_202752	0.8686
Listeria_monocytogenes_serotype_1_2b_str_10-0810_1196163	Listeria_ivanovii_subsp_londoniensis_202752	0.8686
Listeria_monocytogenes_serotype_1_2a_str_99-6370_1196179	Listeria_ivanovii_subsp_londoniensis_202752	0.8638
Listeria_monocytogenes_serotype_1_2a_str_98-2035_1196178	Listeria_ivanovii_subsp_londoniensis_202752	0.8638
Listeria_monocytogenes_serotype_1_2a_str_95-0093_1196177	Listeria_ivanovii_subsp_londoniensis_202752	0.8693
Listeria_monocytogenes_serotype_1_2a_str_88-0478_1196176	Listeria_ivanovii_subsp_londoniensis_202752	0.8677
Listeria_monocytogenes_serotype_1_2a_str_10-5024_1196192	Listeria_ivanovii_subsp_londoniensis_202752	0.8693
Listeria_monocytogenes_serotype_1_2a_str_10-4758_1196168	Listeria_ivanovii_subsp_londoniensis_202752	0.875
Listeria_monocytogenes_serotype_1_2a_str_10-4754_1196167	Listeria_ivanovii_subsp_londoniensis_202752	0.8754
Listeria_monocytogenes_serotype_1_2a_str_10-1321_1196191	Listeria_ivanovii_subsp_londoniensis_202752	0.8693
Listeria_monocytogenes_serotype_1_2a_str_10-1047_1196190	Listeria_ivanovii_subsp_londoniensis_202752	0.8695
Listeria_monocytogenes_serotype_1_2a_str_10-1046_1196189	Listeria_ivanovii_subsp_londoniensis_202752	0.8695
Listeria_monocytogenes_serotype_1_2a_str_10-0934_1196170	Listeria_ivanovii_subsp_londoniensis_202752	0.8868
Listeria_monocytogenes_serotype_1_2a_str_10-0933_1196169	Listeria_ivanovii_subsp_londoniensis_202752	0.8868
Listeria_monocytogenes_serotype_1_2a_str_10-0815_1196188	Listeria_ivanovii_subsp_londoniensis_202752	0.8651
Listeria_monocytogenes_serotype_1_2a_str_10-0814_1196187	Listeria_ivanovii_subsp_londoniensis_202752	0.8693
Listeria_monocytogenes_serotype_1_2a_str_10-0813_1196166	Listeria_ivanovii_subsp_londoniensis_202752	0.8728
Listeria_monocytogenes_serotype_1_2a_str_10-0812_1196165	Listeria_ivanovii_subsp_londoniensis_202752	0.8728
Listeria_monocytogenes_serotype_1_2a_str_08-7669_1196186	Listeria_ivanovii_subsp_londoniensis_202752	0.8769
Listeria_monocytogenes_serotype_1_2a_str_08-7374_1196185	Listeria_ivanovii_subsp_londoniensis_202752	0.8693
Listeria_monocytogenes_serotype_1_2a_str_08-6997_1196184	Listeria_ivanovii_subsp_londoniensis_202752	0.8651
Listeria_monocytogenes_serotype_1_2a_str_08-6569_1196183	Listeria_ivanovii_subsp_londoniensis_202752	0.8651
Listeria_monocytogenes_serotype_1_2a_str_08-6056_1196182	Listeria_ivanovii_subsp_londoniensis_202752	0.8651
Listeria_monocytogenes_serotype_1_2a_str_04-5457_1196181	Listeria_ivanovii_subsp_londoniensis_202752	0.8693
Listeria_monocytogenes_serotype_1_2a_str_02-5993_1196180	Listeria_ivanovii_subsp_londoniensis_202752	0.8693
Listeria_monocytogenes_R479a_1437838	Listeria_ivanovii_subsp_londoniensis_202752	0.869
Listeria_monocytogenes_N53-1_1234142	Listeria_ivanovii_subsp_londoniensis_202752	0.8406
Listeria_monocytogenes_M7_1030009	Listeria_ivanovii_subsp_londoniensis_202752	0.8557
Listeria_monocytogenes_LO28_393132	Listeria_ivanovii_subsp_londoniensis_202752	0.8716
Listeria_monocytogenes_L99_563174	Listeria_ivanovii_subsp_londoniensis_202752	0.8557
Listeria_monocytogenes_L312_882094	Listeria_ivanovii_subsp_londoniensis_202752	0.8899
Listeria_monocytogenes_J1816_930781	Listeria_ivanovii_subsp_londoniensis_202752	0.8799
Listeria_monocytogenes_J1-220_930782	Listeria_ivanovii_subsp_londoniensis_202752	0.8699
Listeria_monocytogenes_J0161_393130	Listeria_ivanovii_subsp_londoniensis_202752	0.8791
Listeria_monocytogenes_HCC23_552536	Listeria_ivanovii_subsp_londoniensis_202752	0.8553
Listeria_monocytogenes_FSL_R2-561_393126	Listeria_ivanovii_subsp_londoniensis_202752	0.8742
Listeria_monocytogenes_FSL_J1-208_393119	Listeria_ivanovii_subsp_londoniensis_202752	0.8691
Listeria_monocytogenes_FSL_J1-175_393118	Listeria_ivanovii_subsp_londoniensis_202752	0.8798
Listeria_monocytogenes_Finland_1998_393127	Listeria_ivanovii_subsp_londoniensis_202752	0.8887
Listeria_monocytogenes_EGD-e_169963_v1	Listeria_ivanovii_subsp_londoniensis_202752	0.8767
Listeria_monocytogenes_EGD-e_169963	Listeria_ivanovii_subsp_londoniensis_202752	0.8767
Listeria_monocytogenes_EGD_1334565	Listeria_ivanovii_subsp_londoniensis_202752	0.879
Listeria_monocytogenes_CFSAN002349_1299895	Listeria_ivanovii_subsp_londoniensis_202752	0.8902
Listeria_monocytogenes_ATCC_19117_882095_v1	Listeria_ivanovii_subsp_londoniensis_202752	0.8854
Listeria_monocytogenes_ATCC_19117_882095	Listeria_ivanovii_subsp_londoniensis_202752	0.885
Listeria_monocytogenes_6179_1288295	Listeria_ivanovii_subsp_londoniensis_202752	0.866
Listeria_monocytogenes_1639_v9	Listeria_ivanovii_subsp_londoniensis_202752	0.8619
Listeria_monocytogenes_1639_v99	Listeria_ivanovii_subsp_londoniensis_202752	0.8811
Listeria_monocytogenes_1639_v98	Listeria_ivanovii_subsp_londoniensis_202752	0.8744
Listeria_monocytogenes_1639_v97	Listeria_ivanovii_subsp_londoniensis_202752	0.888
Listeria_monocytogenes_1639_v96	Listeria_ivanovii_subsp_londoniensis_202752	0.88

Listeria_monocytogenes_1639_v95	Listeria_ivanovii_subsp_londoniensis_202752	0.8801
Listeria_monocytogenes_1639_v94	Listeria_ivanovii_subsp_londoniensis_202752	0.8807
Listeria_monocytogenes_1639_v93	Listeria_ivanovii_subsp_londoniensis_202752	0.8651
Listeria_monocytogenes_1639_v92	Listeria_ivanovii_subsp_londoniensis_202752	0.8815
Listeria_monocytogenes_1639_v91	Listeria_ivanovii_subsp_londoniensis_202752	0.8753
Listeria_monocytogenes_1639_v90	Listeria_ivanovii_subsp_londoniensis_202752	0.8744
Listeria_monocytogenes_1639_v8	Listeria_ivanovii_subsp_londoniensis_202752	0.8591
Listeria_monocytogenes_1639_v89	Listeria_ivanovii_subsp_londoniensis_202752	0.8864
Listeria_monocytogenes_1639_v88	Listeria_ivanovii_subsp_londoniensis_202752	0.8775
Listeria_monocytogenes_1639_v87	Listeria_ivanovii_subsp_londoniensis_202752	0.8686
Listeria_monocytogenes_1639_v86	Listeria_ivanovii_subsp_londoniensis_202752	0.8686
Listeria_monocytogenes_1639_v85	Listeria_ivanovii_subsp_londoniensis_202752	0.8755
Listeria_monocytogenes_1639_v84	Listeria_ivanovii_subsp_londoniensis_202752	0.8813
Listeria_monocytogenes_1639_v83	Listeria_ivanovii_subsp_londoniensis_202752	0.8774
Listeria_monocytogenes_1639_v82	Listeria_ivanovii_subsp_londoniensis_202752	0.8771
Listeria_monocytogenes_1639_v81	Listeria_ivanovii_subsp_londoniensis_202752	0.8797
Listeria_monocytogenes_1639_v80	Listeria_ivanovii_subsp_londoniensis_202752	0.873
Listeria_monocytogenes_1639_v7	Listeria_ivanovii_subsp_londoniensis_202752	0.8591
Listeria_monocytogenes_1639_v79	Listeria_ivanovii_subsp_londoniensis_202752	0.8777
Listeria_monocytogenes_1639_v78	Listeria_ivanovii_subsp_londoniensis_202752	0.8787
Listeria_monocytogenes_1639_v77	Listeria_ivanovii_subsp_londoniensis_202752	0.8819
Listeria_monocytogenes_1639_v76	Listeria_ivanovii_subsp_londoniensis_202752	0.8691
Listeria_monocytogenes_1639_v75	Listeria_ivanovii_subsp_londoniensis_202752	0.8557
Listeria_monocytogenes_1639_v74	Listeria_ivanovii_subsp_londoniensis_202752	0.8675
Listeria_monocytogenes_1639_v73	Listeria_ivanovii_subsp_londoniensis_202752	0.8667
Listeria_monocytogenes_1639_v72	Listeria_ivanovii_subsp_londoniensis_202752	0.8761
Listeria_monocytogenes_1639_v71	Listeria_ivanovii_subsp_londoniensis_202752	0.8677
Listeria_monocytogenes_1639_v70	Listeria_ivanovii_subsp_londoniensis_202752	0.8749
Listeria_monocytogenes_1639_v6	Listeria_ivanovii_subsp_londoniensis_202752	0.8591
Listeria_monocytogenes_1639_v69	Listeria_ivanovii_subsp_londoniensis_202752	0.8772
Listeria_monocytogenes_1639_v68	Listeria_ivanovii_subsp_londoniensis_202752	0.8763
Listeria_monocytogenes_1639_v67	Listeria_ivanovii_subsp_londoniensis_202752	0.8805
Listeria_monocytogenes_1639_v66	Listeria_ivanovii_subsp_londoniensis_202752	0.8744
Listeria_monocytogenes_1639_v65	Listeria_ivanovii_subsp_londoniensis_202752	0.8666
Listeria_monocytogenes_1639_v64	Listeria_ivanovii_subsp_londoniensis_202752	0.8697
Listeria_monocytogenes_1639_v63	Listeria_ivanovii_subsp_londoniensis_202752	0.8759
Listeria_monocytogenes_1639_v62	Listeria_ivanovii_subsp_londoniensis_202752	0.8765
Listeria_monocytogenes_1639_v61	Listeria_ivanovii_subsp_londoniensis_202752	0.8714
Listeria_monocytogenes_1639_v60	Listeria_ivanovii_subsp_londoniensis_202752	0.8804
Listeria_monocytogenes_1639_v5	Listeria_ivanovii_subsp_londoniensis_202752	0.8382
Listeria_monocytogenes_1639_v59	Listeria_ivanovii_subsp_londoniensis_202752	0.8769
Listeria_monocytogenes_1639_v58	Listeria_ivanovii_subsp_londoniensis_202752	0.8781
Listeria_monocytogenes_1639_v57	Listeria_ivanovii_subsp_londoniensis_202752	0.8761
Listeria_monocytogenes_1639_v56	Listeria_ivanovii_subsp_londoniensis_202752	0.8852
Listeria_monocytogenes_1639_v55	Listeria_ivanovii_subsp_londoniensis_202752	0.875
Listeria_monocytogenes_1639_v54	Listeria_ivanovii_subsp_londoniensis_202752	0.8935
Listeria_monocytogenes_1639_v53	Listeria_ivanovii_subsp_londoniensis_202752	0.8706
Listeria_monocytogenes_1639_v52	Listeria_ivanovii_subsp_londoniensis_202752	0.8755
Listeria_monocytogenes_1639_v51	Listeria_ivanovii_subsp_londoniensis_202752	0.8736
Listeria_monocytogenes_1639_v50	Listeria_ivanovii_subsp_londoniensis_202752	0.8678
Listeria_monocytogenes_1639_v4	Listeria_ivanovii_subsp_londoniensis_202752	0.8504
Listeria_monocytogenes_1639_v49	Listeria_ivanovii_subsp_londoniensis_202752	0.8761
Listeria_monocytogenes_1639_v48	Listeria_ivanovii_subsp_londoniensis_202752	0.8714
Listeria_monocytogenes_1639_v47	Listeria_ivanovii_subsp_londoniensis_202752	0.8686
Listeria_monocytogenes_1639_v46	Listeria_ivanovii_subsp_londoniensis_202752	0.8828
Listeria_monocytogenes_1639_v45	Listeria_ivanovii_subsp_londoniensis_202752	0.861
Listeria_monocytogenes_1639_v44	Listeria_ivanovii_subsp_londoniensis_202752	0.8693
Listeria_monocytogenes_1639_v43	Listeria_ivanovii_subsp_londoniensis_202752	0.8693
Listeria_monocytogenes_1639_v42	Listeria_ivanovii_subsp_londoniensis_202752	0.8663
Listeria_monocytogenes_1639_v41	Listeria_ivanovii_subsp_londoniensis_202752	0.851
Listeria_monocytogenes_1639_v40	Listeria_ivanovii_subsp_londoniensis_202752	0.87
Listeria_monocytogenes_1639_v3	Listeria_ivanovii_subsp_londoniensis_202752	0.8508
Listeria_monocytogenes_1639_v39	Listeria_ivanovii_subsp_londoniensis_202752	0.8765
Listeria_monocytogenes_1639_v38	Listeria_ivanovii_subsp_londoniensis_202752	0.8659
Listeria_monocytogenes_1639_v37	Listeria_ivanovii_subsp_londoniensis_202752	0.8611
Listeria_monocytogenes_1639_v36	Listeria_ivanovii_subsp_londoniensis_202752	0.8632
Listeria_monocytogenes_1639_v35	Listeria_ivanovii_subsp_londoniensis_202752	0.8706
Listeria_monocytogenes_1639_v34	Listeria_ivanovii_subsp_londoniensis_202752	0.8762
Listeria_monocytogenes_1639_v33	Listeria_ivanovii_subsp_londoniensis_202752	0.8686
Listeria_monocytogenes_1639_v32	Listeria_ivanovii_subsp_londoniensis_202752	0.8613
Listeria_monocytogenes_1639_v31	Listeria_ivanovii_subsp_londoniensis_202752	0.8762
Listeria_monocytogenes_1639_v30	Listeria_ivanovii_subsp_londoniensis_202752	0.8687
Listeria_monocytogenes_1639_v2	Listeria_ivanovii_subsp_londoniensis_202752	0.8483
Listeria_monocytogenes_1639_v29	Listeria_ivanovii_subsp_londoniensis_202752	0.8642
Listeria_monocytogenes_1639_v28	Listeria_ivanovii_subsp_londoniensis_202752	0.869
Listeria_monocytogenes_1639_v27	Listeria_ivanovii_subsp_londoniensis_202752	0.8546
Listeria_monocytogenes_1639_v26	Listeria_ivanovii_subsp_londoniensis_202752	0.8539
Listeria_monocytogenes_1639_v25	Listeria_ivanovii_subsp_londoniensis_202752	0.8543
Listeria_monocytogenes_1639_v24	Listeria_ivanovii_subsp_londoniensis_202752	0.871
Listeria_monocytogenes_1639_v23	Listeria_ivanovii_subsp_londoniensis_202752	0.8707
Listeria_monocytogenes_1639_v22	Listeria_ivanovii_subsp_londoniensis_202752	0.8759
Listeria_monocytogenes_1639_v21	Listeria_ivanovii_subsp_londoniensis_202752	0.8711
Listeria_monocytogenes_1639_v20	Listeria_ivanovii_subsp_londoniensis_202752	0.8771
Listeria_monocytogenes_1639_v1	Listeria_ivanovii_subsp_londoniensis_202752	0.8609
Listeria_monocytogenes_1639_v19	Listeria_ivanovii_subsp_londoniensis_202752	0.8768
Listeria_monocytogenes_1639_v198	Listeria_ivanovii_subsp_londoniensis_202752	0.8433
Listeria_monocytogenes_1639_v197	Listeria_ivanovii_subsp_londoniensis_202752	0.864
Listeria_monocytogenes_1639_v196	Listeria_ivanovii_subsp_londoniensis_202752	0.8612
Listeria_monocytogenes_1639_v195	Listeria_ivanovii_subsp_londoniensis_202752	0.8851
Listeria_monocytogenes_1639_v194	Listeria_ivanovii_subsp_londoniensis_202752	0.8841
Listeria_monocytogenes_1639_v193	Listeria_ivanovii_subsp_londoniensis_202752	0.8837

Listeria_monocytogenes_1639_v192	Listeria_ivanovii_subsp_londoniensis_202752	0.8622
Listeria_monocytogenes_1639_v191	Listeria_ivanovii_subsp_londoniensis_202752	0.8876
Listeria_monocytogenes_1639_v190	Listeria_ivanovii_subsp_londoniensis_202752	0.8878
Listeria_monocytogenes_1639_v18	Listeria_ivanovii_subsp_londoniensis_202752	0.8717
Listeria_monocytogenes_1639_v189	Listeria_ivanovii_subsp_londoniensis_202752	0.8871
Listeria_monocytogenes_1639_v188	Listeria_ivanovii_subsp_londoniensis_202752	0.8864
Listeria_monocytogenes_1639_v187	Listeria_ivanovii_subsp_londoniensis_202752	0.8877
Listeria_monocytogenes_1639_v186	Listeria_ivanovii_subsp_londoniensis_202752	0.8881
Listeria_monocytogenes_1639_v185	Listeria_ivanovii_subsp_londoniensis_202752	0.8881
Listeria_monocytogenes_1639_v184	Listeria_ivanovii_subsp_londoniensis_202752	0.887
Listeria_monocytogenes_1639_v183	Listeria_ivanovii_subsp_londoniensis_202752	0.8854
Listeria_monocytogenes_1639_v182	Listeria_ivanovii_subsp_londoniensis_202752	0.8867
Listeria_monocytogenes_1639_v181	Listeria_ivanovii_subsp_londoniensis_202752	0.873
Listeria_monocytogenes_1639_v180	Listeria_ivanovii_subsp_londoniensis_202752	0.873
Listeria_monocytogenes_1639_v17	Listeria_ivanovii_subsp_londoniensis_202752	0.872
Listeria_monocytogenes_1639_v179	Listeria_ivanovii_subsp_londoniensis_202752	0.8813
Listeria_monocytogenes_1639_v178	Listeria_ivanovii_subsp_londoniensis_202752	0.8866
Listeria_monocytogenes_1639_v177	Listeria_ivanovii_subsp_londoniensis_202752	0.8879
Listeria_monocytogenes_1639_v176	Listeria_ivanovii_subsp_londoniensis_202752	0.8831
Listeria_monocytogenes_1639_v175	Listeria_ivanovii_subsp_londoniensis_202752	0.8789
Listeria_monocytogenes_1639_v174	Listeria_ivanovii_subsp_londoniensis_202752	0.8887
Listeria_monocytogenes_1639_v173	Listeria_ivanovii_subsp_londoniensis_202752	0.8903
Listeria_monocytogenes_1639_v172	Listeria_ivanovii_subsp_londoniensis_202752	0.8809
Listeria_monocytogenes_1639_v171	Listeria_ivanovii_subsp_londoniensis_202752	0.8767
Listeria_monocytogenes_1639_v170	Listeria_ivanovii_subsp_londoniensis_202752	0.8828
Listeria_monocytogenes_1639_v16	Listeria_ivanovii_subsp_londoniensis_202752	0.8569
Listeria_monocytogenes_1639_v169	Listeria_ivanovii_subsp_londoniensis_202752	0.8841
Listeria_monocytogenes_1639_v168	Listeria_ivanovii_subsp_londoniensis_202752	0.8831
Listeria_monocytogenes_1639_v167	Listeria_ivanovii_subsp_londoniensis_202752	0.8866
Listeria_monocytogenes_1639_v166	Listeria_ivanovii_subsp_londoniensis_202752	0.8834
Listeria_monocytogenes_1639_v165	Listeria_ivanovii_subsp_londoniensis_202752	0.8838
Listeria_monocytogenes_1639_v164	Listeria_ivanovii_subsp_londoniensis_202752	0.8887
Listeria_monocytogenes_1639_v163	Listeria_ivanovii_subsp_londoniensis_202752	0.8858
Listeria_monocytogenes_1639_v162	Listeria_ivanovii_subsp_londoniensis_202752	0.8887
Listeria_monocytogenes_1639_v161	Listeria_ivanovii_subsp_londoniensis_202752	0.8899
Listeria_monocytogenes_1639_v160	Listeria_ivanovii_subsp_londoniensis_202752	0.89
Listeria_monocytogenes_1639_v15	Listeria_ivanovii_subsp_londoniensis_202752	0.856
Listeria_monocytogenes_1639_v159	Listeria_ivanovii_subsp_londoniensis_202752	0.8879
Listeria_monocytogenes_1639_v158	Listeria_ivanovii_subsp_londoniensis_202752	0.8892
Listeria_monocytogenes_1639_v157	Listeria_ivanovii_subsp_londoniensis_202752	0.8892
Listeria_monocytogenes_1639_v156	Listeria_ivanovii_subsp_londoniensis_202752	0.8683
Listeria_monocytogenes_1639_v155	Listeria_ivanovii_subsp_londoniensis_202752	0.884
Listeria_monocytogenes_1639_v154	Listeria_ivanovii_subsp_londoniensis_202752	0.8788
Listeria_monocytogenes_1639_v153	Listeria_ivanovii_subsp_londoniensis_202752	0.8787
Listeria_monocytogenes_1639_v152	Listeria_ivanovii_subsp_londoniensis_202752	0.8879
Listeria_monocytogenes_1639_v151	Listeria_ivanovii_subsp_londoniensis_202752	0.8879
Listeria_monocytogenes_1639_v150	Listeria_ivanovii_subsp_londoniensis_202752	0.8798
Listeria_monocytogenes_1639_v14	Listeria_ivanovii_subsp_londoniensis_202752	0.8598
Listeria_monocytogenes_1639_v149	Listeria_ivanovii_subsp_londoniensis_202752	0.8793
Listeria_monocytogenes_1639_v148	Listeria_ivanovii_subsp_londoniensis_202752	0.8868
Listeria_monocytogenes_1639_v147	Listeria_ivanovii_subsp_londoniensis_202752	0.875
Listeria_monocytogenes_1639_v146	Listeria_ivanovii_subsp_londoniensis_202752	0.8893
Listeria_monocytogenes_1639_v145	Listeria_ivanovii_subsp_londoniensis_202752	0.883
Listeria_monocytogenes_1639_v144	Listeria_ivanovii_subsp_londoniensis_202752	0.8895
Listeria_monocytogenes_1639_v143	Listeria_ivanovii_subsp_londoniensis_202752	0.8852
Listeria_monocytogenes_1639_v142	Listeria_ivanovii_subsp_londoniensis_202752	0.8903
Listeria_monocytogenes_1639_v141	Listeria_ivanovii_subsp_londoniensis_202752	0.8878
Listeria_monocytogenes_1639_v140	Listeria_ivanovii_subsp_londoniensis_202752	0.8902
Listeria_monocytogenes_1639_v13	Listeria_ivanovii_subsp_londoniensis_202752	0.856
Listeria_monocytogenes_1639_v139	Listeria_ivanovii_subsp_londoniensis_202752	0.8854
Listeria_monocytogenes_1639_v138	Listeria_ivanovii_subsp_londoniensis_202752	0.8907
Listeria_monocytogenes_1639_v137	Listeria_ivanovii_subsp_londoniensis_202752	0.8899
Listeria_monocytogenes_1639_v136	Listeria_ivanovii_subsp_londoniensis_202752	0.8747
Listeria_monocytogenes_1639_v135	Listeria_ivanovii_subsp_londoniensis_202752	0.865
Listeria_monocytogenes_1639_v134	Listeria_ivanovii_subsp_londoniensis_202752	0.8839
Listeria_monocytogenes_1639_v133	Listeria_ivanovii_subsp_londoniensis_202752	0.8898
Listeria_monocytogenes_1639_v132	Listeria_ivanovii_subsp_londoniensis_202752	0.8635
Listeria_monocytogenes_1639_v131	Listeria_ivanovii_subsp_londoniensis_202752	0.8784
Listeria_monocytogenes_1639_v130	Listeria_ivanovii_subsp_londoniensis_202752	0.8703
Listeria_monocytogenes_1639_v12	Listeria_ivanovii_subsp_londoniensis_202752	0.8574
Listeria_monocytogenes_1639_v129	Listeria_ivanovii_subsp_londoniensis_202752	0.8853
Listeria_monocytogenes_1639_v128	Listeria_ivanovii_subsp_londoniensis_202752	0.8849
Listeria_monocytogenes_1639_v127	Listeria_ivanovii_subsp_londoniensis_202752	0.8767
Listeria_monocytogenes_1639_v126	Listeria_ivanovii_subsp_londoniensis_202752	0.8838
Listeria_monocytogenes_1639_v125	Listeria_ivanovii_subsp_londoniensis_202752	0.8834
Listeria_monocytogenes_1639_v124	Listeria_ivanovii_subsp_londoniensis_202752	0.8778
Listeria_monocytogenes_1639_v123	Listeria_ivanovii_subsp_londoniensis_202752	0.8902
Listeria_monocytogenes_1639_v122	Listeria_ivanovii_subsp_londoniensis_202752	0.8898
Listeria_monocytogenes_1639_v121	Listeria_ivanovii_subsp_londoniensis_202752	0.8898
Listeria_monocytogenes_1639_v120	Listeria_ivanovii_subsp_londoniensis_202752	0.8902
Listeria_monocytogenes_1639_v11	Listeria_ivanovii_subsp_londoniensis_202752	0.863
Listeria_monocytogenes_1639_v119	Listeria_ivanovii_subsp_londoniensis_202752	0.8898
Listeria_monocytogenes_1639_v118	Listeria_ivanovii_subsp_londoniensis_202752	0.8898
Listeria_monocytogenes_1639_v117	Listeria_ivanovii_subsp_londoniensis_202752	0.8759
Listeria_monocytogenes_1639_v116	Listeria_ivanovii_subsp_londoniensis_202752	0.8835
Listeria_monocytogenes_1639_v115	Listeria_ivanovii_subsp_londoniensis_202752	0.8839
Listeria_monocytogenes_1639_v114	Listeria_ivanovii_subsp_londoniensis_202752	0.8787
Listeria_monocytogenes_1639_v113	Listeria_ivanovii_subsp_londoniensis_202752	0.8807
Listeria_monocytogenes_1639_v112	Listeria_ivanovii_subsp_londoniensis_202752	0.8896
Listeria_monocytogenes_1639_v111	Listeria_ivanovii_subsp_londoniensis_202752	0.8803
Listeria_monocytogenes_1639_v110	Listeria_ivanovii_subsp_londoniensis_202752	0.8727

Listeria_monocytogenes_1639_v10	Listeria_ivanovii_subsp_londoniensis_202752	0.859
Listeria_monocytogenes_1639_v109	Listeria_ivanovii_subsp_londoniensis_202752	0.8798
Listeria_monocytogenes_1639_v108	Listeria_ivanovii_subsp_londoniensis_202752	0.8798
Listeria_monocytogenes_1639_v107	Listeria_ivanovii_subsp_londoniensis_202752	0.8844
Listeria_monocytogenes_1639_v106	Listeria_ivanovii_subsp_londoniensis_202752	0.8844
Listeria_monocytogenes_1639_v105	Listeria_ivanovii_subsp_londoniensis_202752	0.8848
Listeria_monocytogenes_1639_v104	Listeria_ivanovii_subsp_londoniensis_202752	0.8844
Listeria_monocytogenes_1639_v103	Listeria_ivanovii_subsp_londoniensis_202752	0.8847
Listeria_monocytogenes_1639_v102	Listeria_ivanovii_subsp_londoniensis_202752	0.8791
Listeria_monocytogenes_1639_v101	Listeria_ivanovii_subsp_londoniensis_202752	0.8687
Listeria_monocytogenes_1639_v100	Listeria_ivanovii_subsp_londoniensis_202752	0.868
Listeria_monocytogenes_1639	Listeria_ivanovii_subsp_londoniensis_202752	0.8878
Listeria_monocytogenes_10403S_393133	Listeria_ivanovii_subsp_londoniensis_202752	0.8811
Listeria_monocytogenes_08-5923_637381	Listeria_ivanovii_subsp_londoniensis_202752	0.8693
Listeria_monocytogenes_08-5578_653938	Listeria_ivanovii_subsp_londoniensis_202752	0.8569
Listeria_monocytogenes_07PF0776_1126011	Listeria_ivanovii_subsp_londoniensis_202752	0.8888
Listeria_marthii_FSL_S4-120_702457	Listeria_ivanovii_subsp_londoniensis_202752	0.8111
Listeria_marthii_529731_v1	Listeria_ivanovii_subsp_londoniensis_202752	0.8533
Listeria_marthii_529731	Listeria_ivanovii_subsp_londoniensis_202752	0.8585
Listeria_ivanovii_WSLC3009_1457190	Listeria_ivanovii_subsp_londoniensis_202752	0.897
Listeria_ivanovii_subsp_londoniensis_202752_v2	Listeria_ivanovii_subsp_londoniensis_202752	0.9821
Listeria_ivanovii_subsp_londoniensis_202752_v1	Listeria_ivanovii_subsp_londoniensis_202752	0.9437
Listeria_ivanovii_subsp_londoniensis_202752	Listeria_ivanovii_subsp_londoniensis_202752	0.0624
Listeria_welshimeri_1643	Listeria_ivanovii_subsp_londoniensis_202752_v1	0.865
Listeria_welshimeri_serovar_6b_str_SLCC5334_386043	Listeria_ivanovii_subsp_londoniensis_202752_v1	0.8647
Listeria_seeligeri_serovar_1_2b_str_SLCC3954_683837	Listeria_ivanovii_subsp_londoniensis_202752_v1	0.8746
Listeria_seeligeri_FSL_S4-171_702452	Listeria_ivanovii_subsp_londoniensis_202752_v1	0.8142
Listeria_seeligeri_FSL_N1-067_702453	Listeria_ivanovii_subsp_londoniensis_202752_v1	0.8298
Listeria_seeligeri_1640_v1	Listeria_ivanovii_subsp_londoniensis_202752_v1	0.8895
Listeria_seeligeri_1640	Listeria_ivanovii_subsp_londoniensis_202752_v1	0.8749
Listeria_monocytogenes_WSLC1042_1457188	Listeria_ivanovii_subsp_londoniensis_202752_v1	0.8805
Listeria_monocytogenes_WSLC1001_1457187	Listeria_ivanovii_subsp_londoniensis_202752_v1	0.8761
Listeria_monocytogenes_SLCC7179_879090	Listeria_ivanovii_subsp_londoniensis_202752_v1	0.8738
Listeria_monocytogenes_SLCC5850_882096	Listeria_ivanovii_subsp_londoniensis_202752_v1	0.8778
Listeria_monocytogenes_SLCC2755_932919	Listeria_ivanovii_subsp_londoniensis_202752_v1	0.8736
Listeria_monocytogenes_SLCC2540_879089	Listeria_ivanovii_subsp_londoniensis_202752_v1	0.8768
Listeria_monocytogenes_SLCC2479_882020	Listeria_ivanovii_subsp_londoniensis_202752_v1	0.8784
Listeria_monocytogenes_SLCC2378_879088	Listeria_ivanovii_subsp_londoniensis_202752_v1	0.8814
Listeria_monocytogenes_SLCC2376_882097	Listeria_ivanovii_subsp_londoniensis_202752_v1	0.8774
Listeria_monocytogenes_SLCC2372_932920	Listeria_ivanovii_subsp_londoniensis_202752_v1	0.8731
Listeria_monocytogenes_serotype_4b_str_LL195_1230340	Listeria_ivanovii_subsp_londoniensis_202752_v1	0.8848
Listeria_monocytogenes_serotype_4b_str_LIS0087_1134669	Listeria_ivanovii_subsp_londoniensis_202752_v1	0.8554
Listeria_monocytogenes_serotype_4b_str_F2365_265669	Listeria_ivanovii_subsp_londoniensis_202752_v1	0.8848
Listeria_monocytogenes_serotype_4b_str_CLIP_80459_568819	Listeria_ivanovii_subsp_londoniensis_202752_v1	0.8847
Listeria_monocytogenes_serotype_4b_str_81-0861_1196161	Listeria_ivanovii_subsp_londoniensis_202752_v1	0.8734
Listeria_monocytogenes_serotype_4b_str_81-0592_1196160	Listeria_ivanovii_subsp_londoniensis_202752_v1	0.8744
Listeria_monocytogenes_serotype_4b_str_81-0558_1196159	Listeria_ivanovii_subsp_londoniensis_202752_v1	0.8751
Listeria_monocytogenes_serotype_4b_str_10-0809_1196162	Listeria_ivanovii_subsp_londoniensis_202752_v1	0.8748
Listeria_monocytogenes_serotype_4b_str_02-6680_1196175	Listeria_ivanovii_subsp_londoniensis_202752_v1	0.8853
Listeria_monocytogenes_serotype_4b_str_02-6679_1196174	Listeria_ivanovii_subsp_londoniensis_202752_v1	0.8853
Listeria_monocytogenes_serotype_4b_str_02-1792_1196173	Listeria_ivanovii_subsp_londoniensis_202752_v1	0.8748
Listeria_monocytogenes_serotype_4b_str_02-1289_1196172	Listeria_ivanovii_subsp_londoniensis_202752_v1	0.8748
Listeria_monocytogenes_serotype_4b_str_02-1103_1196171	Listeria_ivanovii_subsp_londoniensis_202752_v1	0.8748
Listeria_monocytogenes_serotype_3c_str_10-5027_1196195	Listeria_ivanovii_subsp_londoniensis_202752_v1	0.8761
Listeria_monocytogenes_serotype_1_2c_str_10-5026_1196194	Listeria_ivanovii_subsp_londoniensis_202752_v1	0.8765
Listeria_monocytogenes_serotype_1_2c_str_10-5025_1196193	Listeria_ivanovii_subsp_londoniensis_202752_v1	0.8765
Listeria_monocytogenes_serotype_1_2b_str_10-0811_1196164	Listeria_ivanovii_subsp_londoniensis_202752_v1	0.8719
Listeria_monocytogenes_serotype_1_2b_str_10-0810_1196163	Listeria_ivanovii_subsp_londoniensis_202752_v1	0.8719
Listeria_monocytogenes_serotype_1_2a_str_99-6370_1196179	Listeria_ivanovii_subsp_londoniensis_202752_v1	0.859
Listeria_monocytogenes_serotype_1_2a_str_98-2035_1196178	Listeria_ivanovii_subsp_londoniensis_202752_v1	0.859
Listeria_monocytogenes_serotype_1_2a_str_95-0093_1196177	Listeria_ivanovii_subsp_londoniensis_202752_v1	0.8626
Listeria_monocytogenes_serotype_1_2a_str_88-0478_1196176	Listeria_ivanovii_subsp_londoniensis_202752_v1	0.861
Listeria_monocytogenes_serotype_1_2a_str_10-5024_1196192	Listeria_ivanovii_subsp_londoniensis_202752_v1	0.8626
Listeria_monocytogenes_serotype_1_2a_str_10-4758_1196168	Listeria_ivanovii_subsp_londoniensis_202752_v1	0.8787
Listeria_monocytogenes_serotype_1_2a_str_10-4754_1196167	Listeria_ivanovii_subsp_londoniensis_202752_v1	0.879
Listeria_monocytogenes_serotype_1_2a_str_10-1321_1196191	Listeria_ivanovii_subsp_londoniensis_202752_v1	0.8626
Listeria_monocytogenes_serotype_1_2a_str_10-1047_1196190	Listeria_ivanovii_subsp_londoniensis_202752_v1	0.8625
Listeria_monocytogenes_serotype_1_2a_str_10-1046_1196189	Listeria_ivanovii_subsp_londoniensis_202752_v1	0.8625
Listeria_monocytogenes_serotype_1_2a_str_10-0934_1196170	Listeria_ivanovii_subsp_londoniensis_202752_v1	0.8847
Listeria_monocytogenes_serotype_1_2a_str_10-0933_1196169	Listeria_ivanovii_subsp_londoniensis_202752_v1	0.8847
Listeria_monocytogenes_serotype_1_2a_str_10-0815_1196188	Listeria_ivanovii_subsp_londoniensis_202752_v1	0.8599
Listeria_monocytogenes_serotype_1_2a_str_10-0814_1196187	Listeria_ivanovii_subsp_londoniensis_202752_v1	0.8626
Listeria_monocytogenes_serotype_1_2a_str_10-0813_1196166	Listeria_ivanovii_subsp_londoniensis_202752_v1	0.8708
Listeria_monocytogenes_serotype_1_2a_str_10-0812_1196165	Listeria_ivanovii_subsp_londoniensis_202752_v1	0.8708
Listeria_monocytogenes_serotype_1_2a_str_08-7669_1196186	Listeria_ivanovii_subsp_londoniensis_202752_v1	0.8656
Listeria_monocytogenes_serotype_1_2a_str_08-7374_1196185	Listeria_ivanovii_subsp_londoniensis_202752_v1	0.8626
Listeria_monocytogenes_serotype_1_2a_str_08-6997_1196184	Listeria_ivanovii_subsp_londoniensis_202752_v1	0.8599
Listeria_monocytogenes_serotype_1_2a_str_08-6569_1196183	Listeria_ivanovii_subsp_londoniensis_202752_v1	0.8599
Listeria_monocytogenes_serotype_1_2a_str_08-6056_1196182	Listeria_ivanovii_subsp_londoniensis_202752_v1	0.8599
Listeria_monocytogenes_serotype_1_2a_str_04-5457_1196181	Listeria_ivanovii_subsp_londoniensis_202752_v1	0.8626
Listeria_monocytogenes_serotype_1_2a_str_02-5993_1196180	Listeria_ivanovii_subsp_londoniensis_202752_v1	0.8626
Listeria_monocytogenes_R479a_1437838	Listeria_ivanovii_subsp_londoniensis_202752_v1	0.8627
Listeria_monocytogenes_N53-1_1234142	Listeria_ivanovii_subsp_londoniensis_202752_v1	0.8382
Listeria_monocytogenes_M7_1030009	Listeria_ivanovii_subsp_londoniensis_202752_v1	0.8665
Listeria_monocytogenes_LO28_393132	Listeria_ivanovii_subsp_londoniensis_202752_v1	0.8778
Listeria_monocytogenes_L99_563174	Listeria_ivanovii_subsp_londoniensis_202752_v1	0.8664
Listeria_monocytogenes_L312_882094	Listeria_ivanovii_subsp_londoniensis_202752_v1	0.8847
Listeria_monocytogenes_J1816_930781	Listeria_ivanovii_subsp_londoniensis_202752_v1	0.8679
Listeria_monocytogenes_J1-220_930782	Listeria_ivanovii_subsp_londoniensis_202752_v1	0.8754
Listeria_monocytogenes_J0161_393130	Listeria_ivanovii_subsp_londoniensis_202752_v1	0.8659
Listeria_monocytogenes_HCC23_552536	Listeria_ivanovii_subsp_londoniensis_202752_v1	0.8661

Listeria_monocytogenes_FSL_R2-561_393126	Listeria_ivanovii_subsp_londoniensis_202752_v1	0.8759
Listeria_monocytogenes_FSL_J1-208_393119	Listeria_ivanovii_subsp_londoniensis_202752_v1	0.8634
Listeria_monocytogenes_FSL_J1-175_393118	Listeria_ivanovii_subsp_londoniensis_202752_v1	0.8819
Listeria_monocytogenes_Finland_1998_393127	Listeria_ivanovii_subsp_londoniensis_202752_v1	0.8858
Listeria_monocytogenes_EGD-e_169963_v1	Listeria_ivanovii_subsp_londoniensis_202752_v1	0.874
Listeria_monocytogenes_EGD-e_169963	Listeria_ivanovii_subsp_londoniensis_202752_v1	0.874
Listeria_monocytogenes_EGD_1334565	Listeria_ivanovii_subsp_londoniensis_202752_v1	0.8781
Listeria_monocytogenes_CFSAN002349_1299895	Listeria_ivanovii_subsp_londoniensis_202752_v1	0.8854
Listeria_monocytogenes_ATCC_19117_882095_v1	Listeria_ivanovii_subsp_londoniensis_202752_v1	0.8834
Listeria_monocytogenes_ATCC_19117_882095	Listeria_ivanovii_subsp_londoniensis_202752_v1	0.883
Listeria_monocytogenes_6179_1288295	Listeria_ivanovii_subsp_londoniensis_202752_v1	0.8631
Listeria_monocytogenes_1639_v9	Listeria_ivanovii_subsp_londoniensis_202752_v1	0.8543
Listeria_monocytogenes_1639_v99	Listeria_ivanovii_subsp_londoniensis_202752_v1	0.8687
Listeria_monocytogenes_1639_v98	Listeria_ivanovii_subsp_londoniensis_202752_v1	0.8632
Listeria_monocytogenes_1639_v97	Listeria_ivanovii_subsp_londoniensis_202752_v1	0.8755
Listeria_monocytogenes_1639_v96	Listeria_ivanovii_subsp_londoniensis_202752_v1	0.8684
Listeria_monocytogenes_1639_v95	Listeria_ivanovii_subsp_londoniensis_202752_v1	0.8685
Listeria_monocytogenes_1639_v94	Listeria_ivanovii_subsp_londoniensis_202752_v1	0.8687
Listeria_monocytogenes_1639_v93	Listeria_ivanovii_subsp_londoniensis_202752_v1	0.8684
Listeria_monocytogenes_1639_v92	Listeria_ivanovii_subsp_londoniensis_202752_v1	0.8687
Listeria_monocytogenes_1639_v91	Listeria_ivanovii_subsp_londoniensis_202752_v1	0.8785
Listeria_monocytogenes_1639_v90	Listeria_ivanovii_subsp_londoniensis_202752_v1	0.8632
Listeria_monocytogenes_1639_v8	Listeria_ivanovii_subsp_londoniensis_202752_v1	0.8595
Listeria_monocytogenes_1639_v89	Listeria_ivanovii_subsp_londoniensis_202752_v1	0.8802
Listeria_monocytogenes_1639_v88	Listeria_ivanovii_subsp_londoniensis_202752_v1	0.8748
Listeria_monocytogenes_1639_v87	Listeria_ivanovii_subsp_londoniensis_202752_v1	0.8697
Listeria_monocytogenes_1639_v86	Listeria_ivanovii_subsp_londoniensis_202752_v1	0.8697
Listeria_monocytogenes_1639_v85	Listeria_ivanovii_subsp_londoniensis_202752_v1	0.8732
Listeria_monocytogenes_1639_v84	Listeria_ivanovii_subsp_londoniensis_202752_v1	0.8763
Listeria_monocytogenes_1639_v83	Listeria_ivanovii_subsp_londoniensis_202752_v1	0.875
Listeria_monocytogenes_1639_v82	Listeria_ivanovii_subsp_londoniensis_202752_v1	0.8747
Listeria_monocytogenes_1639_v81	Listeria_ivanovii_subsp_londoniensis_202752_v1	0.874
Listeria_monocytogenes_1639_v80	Listeria_ivanovii_subsp_londoniensis_202752_v1	0.8733
Listeria_monocytogenes_1639_v7	Listeria_ivanovii_subsp_londoniensis_202752_v1	0.8595
Listeria_monocytogenes_1639_v79	Listeria_ivanovii_subsp_londoniensis_202752_v1	0.8746
Listeria_monocytogenes_1639_v78	Listeria_ivanovii_subsp_londoniensis_202752_v1	0.8744
Listeria_monocytogenes_1639_v77	Listeria_ivanovii_subsp_londoniensis_202752_v1	0.8683
Listeria_monocytogenes_1639_v76	Listeria_ivanovii_subsp_londoniensis_202752_v1	0.8661
Listeria_monocytogenes_1639_v75	Listeria_ivanovii_subsp_londoniensis_202752_v1	0.8664
Listeria_monocytogenes_1639_v74	Listeria_ivanovii_subsp_londoniensis_202752_v1	0.8619
Listeria_monocytogenes_1639_v73	Listeria_ivanovii_subsp_londoniensis_202752_v1	0.8718
Listeria_monocytogenes_1639_v72	Listeria_ivanovii_subsp_londoniensis_202752_v1	0.8738
Listeria_monocytogenes_1639_v71	Listeria_ivanovii_subsp_londoniensis_202752_v1	0.861
Listeria_monocytogenes_1639_v70	Listeria_ivanovii_subsp_londoniensis_202752_v1	0.8726
Listeria_monocytogenes_1639_v6	Listeria_ivanovii_subsp_londoniensis_202752_v1	0.8595
Listeria_monocytogenes_1639_v69	Listeria_ivanovii_subsp_londoniensis_202752_v1	0.8744
Listeria_monocytogenes_1639_v68	Listeria_ivanovii_subsp_londoniensis_202752_v1	0.8724
Listeria_monocytogenes_1639_v67	Listeria_ivanovii_subsp_londoniensis_202752_v1	0.8743
Listeria_monocytogenes_1639_v66	Listeria_ivanovii_subsp_londoniensis_202752_v1	0.8777
Listeria_monocytogenes_1639_v65	Listeria_ivanovii_subsp_londoniensis_202752_v1	0.8662
Listeria_monocytogenes_1639_v64	Listeria_ivanovii_subsp_londoniensis_202752_v1	0.8682
Listeria_monocytogenes_1639_v63	Listeria_ivanovii_subsp_londoniensis_202752_v1	0.8736
Listeria_monocytogenes_1639_v62	Listeria_ivanovii_subsp_londoniensis_202752_v1	0.8712
Listeria_monocytogenes_1639_v61	Listeria_ivanovii_subsp_londoniensis_202752_v1	0.8681
Listeria_monocytogenes_1639_v60	Listeria_ivanovii_subsp_londoniensis_202752_v1	0.8676
Listeria_monocytogenes_1639_v5	Listeria_ivanovii_subsp_londoniensis_202752_v1	0.8399
Listeria_monocytogenes_1639_v59	Listeria_ivanovii_subsp_londoniensis_202752_v1	0.8824
Listeria_monocytogenes_1639_v58	Listeria_ivanovii_subsp_londoniensis_202752_v1	0.8787
Listeria_monocytogenes_1639_v57	Listeria_ivanovii_subsp_londoniensis_202752_v1	0.8638
Listeria_monocytogenes_1639_v56	Listeria_ivanovii_subsp_londoniensis_202752_v1	0.8753
Listeria_monocytogenes_1639_v55	Listeria_ivanovii_subsp_londoniensis_202752_v1	0.8628
Listeria_monocytogenes_1639_v54	Listeria_ivanovii_subsp_londoniensis_202752_v1	0.8771
Listeria_monocytogenes_1639_v53	Listeria_ivanovii_subsp_londoniensis_202752_v1	0.8649
Listeria_monocytogenes_1639_v52	Listeria_ivanovii_subsp_londoniensis_202752_v1	0.8791
Listeria_monocytogenes_1639_v51	Listeria_ivanovii_subsp_londoniensis_202752_v1	0.8712
Listeria_monocytogenes_1639_v50	Listeria_ivanovii_subsp_londoniensis_202752_v1	0.8611
Listeria_monocytogenes_1639_v4	Listeria_ivanovii_subsp_londoniensis_202752_v1	0.852
Listeria_monocytogenes_1639_v49	Listeria_ivanovii_subsp_londoniensis_202752_v1	0.8864
Listeria_monocytogenes_1639_v48	Listeria_ivanovii_subsp_londoniensis_202752_v1	0.8732
Listeria_monocytogenes_1639_v47	Listeria_ivanovii_subsp_londoniensis_202752_v1	0.8619
Listeria_monocytogenes_1639_v46	Listeria_ivanovii_subsp_londoniensis_202752_v1	0.8718
Listeria_monocytogenes_1639_v45	Listeria_ivanovii_subsp_londoniensis_202752_v1	0.8548
Listeria_monocytogenes_1639_v44	Listeria_ivanovii_subsp_londoniensis_202752_v1	0.8626
Listeria_monocytogenes_1639_v43	Listeria_ivanovii_subsp_londoniensis_202752_v1	0.8626
Listeria_monocytogenes_1639_v42	Listeria_ivanovii_subsp_londoniensis_202752_v1	0.8553
Listeria_monocytogenes_1639_v41	Listeria_ivanovii_subsp_londoniensis_202752_v1	0.8522
Listeria_monocytogenes_1639_v40	Listeria_ivanovii_subsp_londoniensis_202752_v1	0.8774
Listeria_monocytogenes_1639_v3	Listeria_ivanovii_subsp_londoniensis_202752_v1	0.8523
Listeria_monocytogenes_1639_v39	Listeria_ivanovii_subsp_londoniensis_202752_v1	0.8742
Listeria_monocytogenes_1639_v38	Listeria_ivanovii_subsp_londoniensis_202752_v1	0.8737
Listeria_monocytogenes_1639_v37	Listeria_ivanovii_subsp_londoniensis_202752_v1	0.8645
Listeria_monocytogenes_1639_v36	Listeria_ivanovii_subsp_londoniensis_202752_v1	0.872
Listeria_monocytogenes_1639_v35	Listeria_ivanovii_subsp_londoniensis_202752_v1	0.8803
Listeria_monocytogenes_1639_v34	Listeria_ivanovii_subsp_londoniensis_202752_v1	0.8698
Listeria_monocytogenes_1639_v33	Listeria_ivanovii_subsp_londoniensis_202752_v1	0.8719
Listeria_monocytogenes_1639_v32	Listeria_ivanovii_subsp_londoniensis_202752_v1	0.8646
Listeria_monocytogenes_1639_v31	Listeria_ivanovii_subsp_londoniensis_202752_v1	0.8698
Listeria_monocytogenes_1639_v30	Listeria_ivanovii_subsp_londoniensis_202752_v1	0.8713
Listeria_monocytogenes_1639_v2	Listeria_ivanovii_subsp_londoniensis_202752_v1	0.8392
Listeria_monocytogenes_1639_v29	Listeria_ivanovii_subsp_londoniensis_202752_v1	0.8671
Listeria_monocytogenes_1639_v28	Listeria_ivanovii_subsp_londoniensis_202752_v1	0.8686

Listeria_monocytogenes_1639_v27	Listeria_ivanovii_subsp_londoniensis_202752_v1	0.863
Listeria_monocytogenes_1639_v26	Listeria_ivanovii_subsp_londoniensis_202752_v1	0.8624
Listeria_monocytogenes_1639_v25	Listeria_ivanovii_subsp_londoniensis_202752_v1	0.8624
Listeria_monocytogenes_1639_v24	Listeria_ivanovii_subsp_londoniensis_202752_v1	0.8758
Listeria_monocytogenes_1639_v23	Listeria_ivanovii_subsp_londoniensis_202752_v1	0.8755
Listeria_monocytogenes_1639_v22	Listeria_ivanovii_subsp_londoniensis_202752_v1	0.8702
Listeria_monocytogenes_1639_v21	Listeria_ivanovii_subsp_londoniensis_202752_v1	0.8758
Listeria_monocytogenes_1639_v20	Listeria_ivanovii_subsp_londoniensis_202752_v1	0.8671
Listeria_monocytogenes_1639_v1	Listeria_ivanovii_subsp_londoniensis_202752_v1	0.8583
Listeria_monocytogenes_1639_v19	Listeria_ivanovii_subsp_londoniensis_202752_v1	0.8668
Listeria_monocytogenes_1639_v198	Listeria_ivanovii_subsp_londoniensis_202752_v1	0.8406
Listeria_monocytogenes_1639_v197	Listeria_ivanovii_subsp_londoniensis_202752_v1	0.8614
Listeria_monocytogenes_1639_v196	Listeria_ivanovii_subsp_londoniensis_202752_v1	0.8604
Listeria_monocytogenes_1639_v195	Listeria_ivanovii_subsp_londoniensis_202752_v1	0.8796
Listeria_monocytogenes_1639_v194	Listeria_ivanovii_subsp_londoniensis_202752_v1	0.8778
Listeria_monocytogenes_1639_v193	Listeria_ivanovii_subsp_londoniensis_202752_v1	0.8778
Listeria_monocytogenes_1639_v192	Listeria_ivanovii_subsp_londoniensis_202752_v1	0.8566
Listeria_monocytogenes_1639_v191	Listeria_ivanovii_subsp_londoniensis_202752_v1	0.8855
Listeria_monocytogenes_1639_v190	Listeria_ivanovii_subsp_londoniensis_202752_v1	0.883
Listeria_monocytogenes_1639_v18	Listeria_ivanovii_subsp_londoniensis_202752_v1	0.8591
Listeria_monocytogenes_1639_v189	Listeria_ivanovii_subsp_londoniensis_202752_v1	0.8823
Listeria_monocytogenes_1639_v188	Listeria_ivanovii_subsp_londoniensis_202752_v1	0.8813
Listeria_monocytogenes_1639_v187	Listeria_ivanovii_subsp_londoniensis_202752_v1	0.883
Listeria_monocytogenes_1639_v186	Listeria_ivanovii_subsp_londoniensis_202752_v1	0.8833
Listeria_monocytogenes_1639_v185	Listeria_ivanovii_subsp_londoniensis_202752_v1	0.8833
Listeria_monocytogenes_1639_v184	Listeria_ivanovii_subsp_londoniensis_202752_v1	0.8822
Listeria_monocytogenes_1639_v183	Listeria_ivanovii_subsp_londoniensis_202752_v1	0.8784
Listeria_monocytogenes_1639_v182	Listeria_ivanovii_subsp_londoniensis_202752_v1	0.8801
Listeria_monocytogenes_1639_v181	Listeria_ivanovii_subsp_londoniensis_202752_v1	0.8668
Listeria_monocytogenes_1639_v180	Listeria_ivanovii_subsp_londoniensis_202752_v1	0.8668
Listeria_monocytogenes_1639_v17	Listeria_ivanovii_subsp_londoniensis_202752_v1	0.8594
Listeria_monocytogenes_1639_v179	Listeria_ivanovii_subsp_londoniensis_202752_v1	0.8706
Listeria_monocytogenes_1639_v178	Listeria_ivanovii_subsp_londoniensis_202752_v1	0.8803
Listeria_monocytogenes_1639_v177	Listeria_ivanovii_subsp_londoniensis_202752_v1	0.8824
Listeria_monocytogenes_1639_v176	Listeria_ivanovii_subsp_londoniensis_202752_v1	0.8784
Listeria_monocytogenes_1639_v175	Listeria_ivanovii_subsp_londoniensis_202752_v1	0.8773
Listeria_monocytogenes_1639_v174	Listeria_ivanovii_subsp_londoniensis_202752_v1	0.8866
Listeria_monocytogenes_1639_v173	Listeria_ivanovii_subsp_londoniensis_202752_v1	0.8858
Listeria_monocytogenes_1639_v172	Listeria_ivanovii_subsp_londoniensis_202752_v1	0.88
Listeria_monocytogenes_1639_v171	Listeria_ivanovii_subsp_londoniensis_202752_v1	0.8735
Listeria_monocytogenes_1639_v170	Listeria_ivanovii_subsp_londoniensis_202752_v1	0.8789
Listeria_monocytogenes_1639_v16	Listeria_ivanovii_subsp_londoniensis_202752_v1	0.8523
Listeria_monocytogenes_1639_v169	Listeria_ivanovii_subsp_londoniensis_202752_v1	0.8824
Listeria_monocytogenes_1639_v168	Listeria_ivanovii_subsp_londoniensis_202752_v1	0.8791
Listeria_monocytogenes_1639_v167	Listeria_ivanovii_subsp_londoniensis_202752_v1	0.8713
Listeria_monocytogenes_1639_v166	Listeria_ivanovii_subsp_londoniensis_202752_v1	0.8795
Listeria_monocytogenes_1639_v165	Listeria_ivanovii_subsp_londoniensis_202752_v1	0.8821
Listeria_monocytogenes_1639_v164	Listeria_ivanovii_subsp_londoniensis_202752_v1	0.8843
Listeria_monocytogenes_1639_v163	Listeria_ivanovii_subsp_londoniensis_202752_v1	0.8848
Listeria_monocytogenes_1639_v162	Listeria_ivanovii_subsp_londoniensis_202752_v1	0.8843
Listeria_monocytogenes_1639_v161	Listeria_ivanovii_subsp_londoniensis_202752_v1	0.8855
Listeria_monocytogenes_1639_v160	Listeria_ivanovii_subsp_londoniensis_202752_v1	0.8856
Listeria_monocytogenes_1639_v15	Listeria_ivanovii_subsp_londoniensis_202752_v1	0.859
Listeria_monocytogenes_1639_v159	Listeria_ivanovii_subsp_londoniensis_202752_v1	0.8824
Listeria_monocytogenes_1639_v158	Listeria_ivanovii_subsp_londoniensis_202752_v1	0.8848
Listeria_monocytogenes_1639_v157	Listeria_ivanovii_subsp_londoniensis_202752_v1	0.8848
Listeria_monocytogenes_1639_v156	Listeria_ivanovii_subsp_londoniensis_202752_v1	0.8627
Listeria_monocytogenes_1639_v155	Listeria_ivanovii_subsp_londoniensis_202752_v1	0.871
Listeria_monocytogenes_1639_v154	Listeria_ivanovii_subsp_londoniensis_202752_v1	0.8798
Listeria_monocytogenes_1639_v153	Listeria_ivanovii_subsp_londoniensis_202752_v1	0.8714
Listeria_monocytogenes_1639_v152	Listeria_ivanovii_subsp_londoniensis_202752_v1	0.8824
Listeria_monocytogenes_1639_v151	Listeria_ivanovii_subsp_londoniensis_202752_v1	0.8824
Listeria_monocytogenes_1639_v150	Listeria_ivanovii_subsp_londoniensis_202752_v1	0.8808
Listeria_monocytogenes_1639_v14	Listeria_ivanovii_subsp_londoniensis_202752_v1	0.8624
Listeria_monocytogenes_1639_v149	Listeria_ivanovii_subsp_londoniensis_202752_v1	0.8784
Listeria_monocytogenes_1639_v148	Listeria_ivanovii_subsp_londoniensis_202752_v1	0.8813
Listeria_monocytogenes_1639_v147	Listeria_ivanovii_subsp_londoniensis_202752_v1	0.87
Listeria_monocytogenes_1639_v146	Listeria_ivanovii_subsp_londoniensis_202752_v1	0.8845
Listeria_monocytogenes_1639_v145	Listeria_ivanovii_subsp_londoniensis_202752_v1	0.876
Listeria_monocytogenes_1639_v144	Listeria_ivanovii_subsp_londoniensis_202752_v1	0.8844
Listeria_monocytogenes_1639_v143	Listeria_ivanovii_subsp_londoniensis_202752_v1	0.8835
Listeria_monocytogenes_1639_v142	Listeria_ivanovii_subsp_londoniensis_202752_v1	0.8851
Listeria_monocytogenes_1639_v141	Listeria_ivanovii_subsp_londoniensis_202752_v1	0.8838
Listeria_monocytogenes_1639_v140	Listeria_ivanovii_subsp_londoniensis_202752_v1	0.8851
Listeria_monocytogenes_1639_v13	Listeria_ivanovii_subsp_londoniensis_202752_v1	0.859
Listeria_monocytogenes_1639_v139	Listeria_ivanovii_subsp_londoniensis_202752_v1	0.8818
Listeria_monocytogenes_1639_v138	Listeria_ivanovii_subsp_londoniensis_202752_v1	0.8855
Listeria_monocytogenes_1639_v137	Listeria_ivanovii_subsp_londoniensis_202752_v1	0.8848
Listeria_monocytogenes_1639_v136	Listeria_ivanovii_subsp_londoniensis_202752_v1	0.878
Listeria_monocytogenes_1639_v135	Listeria_ivanovii_subsp_londoniensis_202752_v1	0.8613
Listeria_monocytogenes_1639_v134	Listeria_ivanovii_subsp_londoniensis_202752_v1	0.8815
Listeria_monocytogenes_1639_v133	Listeria_ivanovii_subsp_londoniensis_202752_v1	0.8787
Listeria_monocytogenes_1639_v132	Listeria_ivanovii_subsp_londoniensis_202752_v1	0.8602
Listeria_monocytogenes_1639_v131	Listeria_ivanovii_subsp_londoniensis_202752_v1	0.873
Listeria_monocytogenes_1639_v130	Listeria_ivanovii_subsp_londoniensis_202752_v1	0.8796
Listeria_monocytogenes_1639_v12	Listeria_ivanovii_subsp_londoniensis_202752_v1	0.8625
Listeria_monocytogenes_1639_v129	Listeria_ivanovii_subsp_londoniensis_202752_v1	0.884
Listeria_monocytogenes_1639_v128	Listeria_ivanovii_subsp_londoniensis_202752_v1	0.8805
Listeria_monocytogenes_1639_v127	Listeria_ivanovii_subsp_londoniensis_202752_v1	0.8773
Listeria_monocytogenes_1639_v126	Listeria_ivanovii_subsp_londoniensis_202752_v1	0.8798
Listeria_monocytogenes_1639_v125	Listeria_ivanovii_subsp_londoniensis_202752_v1	0.8716

Listeria_monocytogenes_1639_v124	Listeria_ivanovii_subsp_londoniensis_202752_v1	0.8705
Listeria_monocytogenes_1639_v123	Listeria_ivanovii_subsp_londoniensis_202752_v1	0.8854
Listeria_monocytogenes_1639_v122	Listeria_ivanovii_subsp_londoniensis_202752_v1	0.8851
Listeria_monocytogenes_1639_v121	Listeria_ivanovii_subsp_londoniensis_202752_v1	0.8851
Listeria_monocytogenes_1639_v120	Listeria_ivanovii_subsp_londoniensis_202752_v1	0.8854
Listeria_monocytogenes_1639_v11	Listeria_ivanovii_subsp_londoniensis_202752_v1	0.8601
Listeria_monocytogenes_1639_v119	Listeria_ivanovii_subsp_londoniensis_202752_v1	0.8851
Listeria_monocytogenes_1639_v118	Listeria_ivanovii_subsp_londoniensis_202752_v1	0.8851
Listeria_monocytogenes_1639_v117	Listeria_ivanovii_subsp_londoniensis_202752_v1	0.8791
Listeria_monocytogenes_1639_v116	Listeria_ivanovii_subsp_londoniensis_202752_v1	0.8811
Listeria_monocytogenes_1639_v115	Listeria_ivanovii_subsp_londoniensis_202752_v1	0.8814
Listeria_monocytogenes_1639_v114	Listeria_ivanovii_subsp_londoniensis_202752_v1	0.8733
Listeria_monocytogenes_1639_v113	Listeria_ivanovii_subsp_londoniensis_202752_v1	0.8832
Listeria_monocytogenes_1639_v112	Listeria_ivanovii_subsp_londoniensis_202752_v1	0.8782
Listeria_monocytogenes_1639_v111	Listeria_ivanovii_subsp_londoniensis_202752_v1	0.8753
Listeria_monocytogenes_1639_v110	Listeria_ivanovii_subsp_londoniensis_202752_v1	0.8727
Listeria_monocytogenes_1639_v10	Listeria_ivanovii_subsp_londoniensis_202752_v1	0.8576
Listeria_monocytogenes_1639_v109	Listeria_ivanovii_subsp_londoniensis_202752_v1	0.8719
Listeria_monocytogenes_1639_v108	Listeria_ivanovii_subsp_londoniensis_202752_v1	0.8748
Listeria_monocytogenes_1639_v107	Listeria_ivanovii_subsp_londoniensis_202752_v1	0.8824
Listeria_monocytogenes_1639_v106	Listeria_ivanovii_subsp_londoniensis_202752_v1	0.8824
Listeria_monocytogenes_1639_v105	Listeria_ivanovii_subsp_londoniensis_202752_v1	0.8827
Listeria_monocytogenes_1639_v104	Listeria_ivanovii_subsp_londoniensis_202752_v1	0.8824
Listeria_monocytogenes_1639_v103	Listeria_ivanovii_subsp_londoniensis_202752_v1	0.8827
Listeria_monocytogenes_1639_v102	Listeria_ivanovii_subsp_londoniensis_202752_v1	0.8726
Listeria_monocytogenes_1639_v101	Listeria_ivanovii_subsp_londoniensis_202752_v1	0.8631
Listeria_monocytogenes_1639_v100	Listeria_ivanovii_subsp_londoniensis_202752_v1	0.8621
Listeria_monocytogenes_1639	Listeria_ivanovii_subsp_londoniensis_202752_v1	0.883
Listeria_monocytogenes_10403S_393133	Listeria_ivanovii_subsp_londoniensis_202752_v1	0.8756
Listeria_monocytogenes_08-5923_637381	Listeria_ivanovii_subsp_londoniensis_202752_v1	0.8626
Listeria_monocytogenes_08-5578_653938	Listeria_ivanovii_subsp_londoniensis_202752_v1	0.8523
Listeria_monocytogenes_07PF0776_1126011	Listeria_ivanovii_subsp_londoniensis_202752_v1	0.8833
Listeria_marthii_FSL_S4-120_702457	Listeria_ivanovii_subsp_londoniensis_202752_v1	0.8104
Listeria_marthii_529731_v1	Listeria_ivanovii_subsp_londoniensis_202752_v1	0.8485
Listeria_marthii_529731	Listeria_ivanovii_subsp_londoniensis_202752_v1	0.8532
Listeria_ivanovii_WSLC3009_1457190	Listeria_ivanovii_subsp_londoniensis_202752_v1	0.8903
Listeria_ivanovii_subsp_londoniensis_202752_v2	Listeria_ivanovii_subsp_londoniensis_202752_v1	0.9535
Listeria_ivanovii_subsp_londoniensis_202752_v1	Listeria_ivanovii_subsp_londoniensis_202752_v1	0.0666
Listeria_welshimeri_1643	Listeria_ivanovii_subsp_londoniensis_202752_v2	0.8683
Listeria_welshimeri_serovar_6b_str_SLCC5334_386043	Listeria_ivanovii_subsp_londoniensis_202752_v2	0.8676
Listeria_seeligeri_serovar_1_2b_str_SLCC3954_683837	Listeria_ivanovii_subsp_londoniensis_202752_v2	0.8828
Listeria_seeligeri_FSL_S4-171_702452	Listeria_ivanovii_subsp_londoniensis_202752_v2	0.8218
Listeria_seeligeri_FSL_N1-067_702453	Listeria_ivanovii_subsp_londoniensis_202752_v2	0.834
Listeria_seeligeri_1640_v1	Listeria_ivanovii_subsp_londoniensis_202752_v2	0.9028
Listeria_seeligeri_1640	Listeria_ivanovii_subsp_londoniensis_202752_v2	0.8809
Listeria_monocytogenes_WSLC1042_1457188	Listeria_ivanovii_subsp_londoniensis_202752_v2	0.8897
Listeria_monocytogenes_WSLC1001_1457187	Listeria_ivanovii_subsp_londoniensis_202752_v2	0.8779
Listeria_monocytogenes_SLCC7179_879090	Listeria_ivanovii_subsp_londoniensis_202752_v2	0.8845
Listeria_monocytogenes_SLCC5850_882096	Listeria_ivanovii_subsp_londoniensis_202752_v2	0.8827
Listeria_monocytogenes_SLCC2755_932919	Listeria_ivanovii_subsp_londoniensis_202752_v2	0.883
Listeria_monocytogenes_SLCC2540_879089	Listeria_ivanovii_subsp_londoniensis_202752_v2	0.8809
Listeria_monocytogenes_SLCC2479_882020	Listeria_ivanovii_subsp_londoniensis_202752_v2	0.8781
Listeria_monocytogenes_SLCC2378_879088	Listeria_ivanovii_subsp_londoniensis_202752_v2	0.8911
Listeria_monocytogenes_SLCC2376_882097	Listeria_ivanovii_subsp_londoniensis_202752_v2	0.8895
Listeria_monocytogenes_SLCC2372_932920	Listeria_ivanovii_subsp_londoniensis_202752_v2	0.8723
Listeria_monocytogenes_serotype_4b_str_LL195_1230340	Listeria_ivanovii_subsp_londoniensis_202752_v2	0.895
Listeria_monocytogenes_serotype_4b_str_LIS0087_1134669	Listeria_ivanovii_subsp_londoniensis_202752_v2	0.8578
Listeria_monocytogenes_serotype_4b_str_F2365_265669	Listeria_ivanovii_subsp_londoniensis_202752_v2	0.895
Listeria_monocytogenes_serotype_4b_str_CLIP_80459_568819	Listeria_ivanovii_subsp_londoniensis_202752_v2	0.8956
Listeria_monocytogenes_serotype_4b_str_81-0861_1196161	Listeria_ivanovii_subsp_londoniensis_202752_v2	0.8802
Listeria_monocytogenes_serotype_4b_str_81-0592_1196160	Listeria_ivanovii_subsp_londoniensis_202752_v2	0.8812
Listeria_monocytogenes_serotype_4b_str_81-0558_1196159	Listeria_ivanovii_subsp_londoniensis_202752_v2	0.8823
Listeria_monocytogenes_serotype_4b_str_10-0809_1196162	Listeria_ivanovii_subsp_londoniensis_202752_v2	0.8816
Listeria_monocytogenes_serotype_4b_str_02-6680_1196175	Listeria_ivanovii_subsp_londoniensis_202752_v2	0.8962
Listeria_monocytogenes_serotype_4b_str_02-6679_1196174	Listeria_ivanovii_subsp_londoniensis_202752_v2	0.8962
Listeria_monocytogenes_serotype_4b_str_02-1792_1196173	Listeria_ivanovii_subsp_londoniensis_202752_v2	0.882
Listeria_monocytogenes_serotype_4b_str_02-1289_1196172	Listeria_ivanovii_subsp_londoniensis_202752_v2	0.882
Listeria_monocytogenes_serotype_4b_str_02-1103_1196171	Listeria_ivanovii_subsp_londoniensis_202752_v2	0.882
Listeria_monocytogenes_serotype_3c_str_10-5027_1196195	Listeria_ivanovii_subsp_londoniensis_202752_v2	0.8769
Listeria_monocytogenes_serotype_1_2c_str_10-5026_1196194	Listeria_ivanovii_subsp_londoniensis_202752_v2	0.878
Listeria_monocytogenes_serotype_1_2c_str_10-5025_1196193	Listeria_ivanovii_subsp_londoniensis_202752_v2	0.878
Listeria_monocytogenes_serotype_1_2b_str_10-0811_1196164	Listeria_ivanovii_subsp_londoniensis_202752_v2	0.8707
Listeria_monocytogenes_serotype_1_2b_str_10-0810_1196163	Listeria_ivanovii_subsp_londoniensis_202752_v2	0.8707
Listeria_monocytogenes_serotype_1_2a_str_99-6370_1196179	Listeria_ivanovii_subsp_londoniensis_202752_v2	0.8603
Listeria_monocytogenes_serotype_1_2a_str_98-2035_1196178	Listeria_ivanovii_subsp_londoniensis_202752_v2	0.8603
Listeria_monocytogenes_serotype_1_2a_str_95-0093_1196177	Listeria_ivanovii_subsp_londoniensis_202752_v2	0.8654
Listeria_monocytogenes_serotype_1_2a_str_88-0478_1196176	Listeria_ivanovii_subsp_londoniensis_202752_v2	0.8645
Listeria_monocytogenes_serotype_1_2a_str_10-5024_1196192	Listeria_ivanovii_subsp_londoniensis_202752_v2	0.8657
Listeria_monocytogenes_serotype_1_2a_str_10-4758_1196168	Listeria_ivanovii_subsp_londoniensis_202752_v2	0.8799
Listeria_monocytogenes_serotype_1_2a_str_10-4754_1196167	Listeria_ivanovii_subsp_londoniensis_202752_v2	0.8799
Listeria_monocytogenes_serotype_1_2a_str_10-1321_1196191	Listeria_ivanovii_subsp_londoniensis_202752_v2	0.8657
Listeria_monocytogenes_serotype_1_2a_str_10-1047_1196190	Listeria_ivanovii_subsp_londoniensis_202752_v2	0.866
Listeria_monocytogenes_serotype_1_2a_str_10-1046_1196189	Listeria_ivanovii_subsp_londoniensis_202752_v2	0.866
Listeria_monocytogenes_serotype_1_2a_str_10-0934_1196170	Listeria_ivanovii_subsp_londoniensis_202752_v2	0.8926
Listeria_monocytogenes_serotype_1_2a_str_10-0933_1196169	Listeria_ivanovii_subsp_londoniensis_202752_v2	0.8926
Listeria_monocytogenes_serotype_1_2a_str_10-0815_1196188	Listeria_ivanovii_subsp_londoniensis_202752_v2	0.8616
Listeria_monocytogenes_serotype_1_2a_str_10-0814_1196187	Listeria_ivanovii_subsp_londoniensis_202752_v2	0.8654
Listeria_monocytogenes_serotype_1_2a_str_10-0813_1196166	Listeria_ivanovii_subsp_londoniensis_202752_v2	0.875
Listeria_monocytogenes_serotype_1_2a_str_10-0812_1196165	Listeria_ivanovii_subsp_londoniensis_202752_v2	0.875
Listeria_monocytogenes_serotype_1_2a_str_08-7669_1196186	Listeria_ivanovii_subsp_londoniensis_202752_v2	0.8746
Listeria_monocytogenes_serotype_1_2a_str_08-7374_1196185	Listeria_ivanovii_subsp_londoniensis_202752_v2	0.8657

Listeria_monocytogenes_serotype_1_2a_str_08-6997_1196184	Listeria_ivanovii_subsp_londoniensis_202752_v2	0.8616
Listeria_monocytogenes_serotype_1_2a_str_08-6569_1196183	Listeria_ivanovii_subsp_londoniensis_202752_v2	0.8616
Listeria_monocytogenes_serotype_1_2a_str_08-6056_1196182	Listeria_ivanovii_subsp_londoniensis_202752_v2	0.8616
Listeria_monocytogenes_serotype_1_2a_str_04-5457_1196181	Listeria_ivanovii_subsp_londoniensis_202752_v2	0.8657
Listeria_monocytogenes_serotype_1_2a_str_02-5993_1196180	Listeria_ivanovii_subsp_londoniensis_202752_v2	0.8654
Listeria_monocytogenes_R479a_1437838	Listeria_ivanovii_subsp_londoniensis_202752_v2	0.8659
Listeria_monocytogenes_N53-1_1234142	Listeria_ivanovii_subsp_londoniensis_202752_v2	0.845
Listeria_monocytogenes_M7_1030009	Listeria_ivanovii_subsp_londoniensis_202752_v2	0.8566
Listeria_monocytogenes_LO28_393132	Listeria_ivanovii_subsp_londoniensis_202752_v2	0.8767
Listeria_monocytogenes_L99_563174	Listeria_ivanovii_subsp_londoniensis_202752_v2	0.8569
Listeria_monocytogenes_L312_882094	Listeria_ivanovii_subsp_londoniensis_202752_v2	0.8956
Listeria_monocytogenes_J1816_930781	Listeria_ivanovii_subsp_londoniensis_202752_v2	0.8768
Listeria_monocytogenes_J1-220_930782	Listeria_ivanovii_subsp_londoniensis_202752_v2	0.8739
Listeria_monocytogenes_J0161_393130	Listeria_ivanovii_subsp_londoniensis_202752_v2	0.8748
Listeria_monocytogenes_HCC23_552536	Listeria_ivanovii_subsp_londoniensis_202752_v2	0.8566
Listeria_monocytogenes_FSL_R2-561_393126	Listeria_ivanovii_subsp_londoniensis_202752_v2	0.8763
Listeria_monocytogenes_FSL_J1-208_393119	Listeria_ivanovii_subsp_londoniensis_202752_v2	0.8754
Listeria_monocytogenes_FSL_J1-175_393118	Listeria_ivanovii_subsp_londoniensis_202752_v2	0.8847
Listeria_monocytogenes_Finland_1998_393127	Listeria_ivanovii_subsp_londoniensis_202752_v2	0.8949
Listeria_monocytogenes_EGD-e_169963_v1	Listeria_ivanovii_subsp_londoniensis_202752_v2	0.8808
Listeria_monocytogenes_EGD-e_169963	Listeria_ivanovii_subsp_londoniensis_202752_v2	0.8808
Listeria_monocytogenes_EGD_1334565	Listeria_ivanovii_subsp_londoniensis_202752_v2	0.8831
Listeria_monocytogenes_CFSAN002349_1299895	Listeria_ivanovii_subsp_londoniensis_202752_v2	0.896
Listeria_monocytogenes_ATCC_19117_882095_v1	Listeria_ivanovii_subsp_londoniensis_202752_v2	0.8927
Listeria_monocytogenes_ATCC_19117_882095	Listeria_ivanovii_subsp_londoniensis_202752_v2	0.8926
Listeria_monocytogenes_6179_1288295	Listeria_ivanovii_subsp_londoniensis_202752_v2	0.8621
Listeria_monocytogenes_1639_v9	Listeria_ivanovii_subsp_londoniensis_202752_v2	0.8573
Listeria_monocytogenes_1639_v99	Listeria_ivanovii_subsp_londoniensis_202752_v2	0.8783
Listeria_monocytogenes_1639_v98	Listeria_ivanovii_subsp_londoniensis_202752_v2	0.8705
Listeria_monocytogenes_1639_v97	Listeria_ivanovii_subsp_londoniensis_202752_v2	0.8838
Listeria_monocytogenes_1639_v96	Listeria_ivanovii_subsp_londoniensis_202752_v2	0.8773
Listeria_monocytogenes_1639_v95	Listeria_ivanovii_subsp_londoniensis_202752_v2	0.8774
Listeria_monocytogenes_1639_v94	Listeria_ivanovii_subsp_londoniensis_202752_v2	0.8777
Listeria_monocytogenes_1639_v93	Listeria_ivanovii_subsp_londoniensis_202752_v2	0.8702
Listeria_monocytogenes_1639_v92	Listeria_ivanovii_subsp_londoniensis_202752_v2	0.878
Listeria_monocytogenes_1639_v91	Listeria_ivanovii_subsp_londoniensis_202752_v2	0.8774
Listeria_monocytogenes_1639_v90	Listeria_ivanovii_subsp_londoniensis_202752_v2	0.8705
Listeria_monocytogenes_1639_v8	Listeria_ivanovii_subsp_londoniensis_202752_v2	0.8607
Listeria_monocytogenes_1639_v89	Listeria_ivanovii_subsp_londoniensis_202752_v2	0.8894
Listeria_monocytogenes_1639_v88	Listeria_ivanovii_subsp_londoniensis_202752_v2	0.8743
Listeria_monocytogenes_1639_v87	Listeria_ivanovii_subsp_londoniensis_202752_v2	0.8703
Listeria_monocytogenes_1639_v86	Listeria_ivanovii_subsp_londoniensis_202752_v2	0.87
Listeria_monocytogenes_1639_v85	Listeria_ivanovii_subsp_londoniensis_202752_v2	0.88
Listeria_monocytogenes_1639_v84	Listeria_ivanovii_subsp_londoniensis_202752_v2	0.8774
Listeria_monocytogenes_1639_v83	Listeria_ivanovii_subsp_londoniensis_202752_v2	0.8822
Listeria_monocytogenes_1639_v82	Listeria_ivanovii_subsp_londoniensis_202752_v2	0.8819
Listeria_monocytogenes_1639_v81	Listeria_ivanovii_subsp_londoniensis_202752_v2	0.8762
Listeria_monocytogenes_1639_v80	Listeria_ivanovii_subsp_londoniensis_202752_v2	0.8748
Listeria_monocytogenes_1639_v7	Listeria_ivanovii_subsp_londoniensis_202752_v2	0.8607
Listeria_monocytogenes_1639_v79	Listeria_ivanovii_subsp_londoniensis_202752_v2	0.8742
Listeria_monocytogenes_1639_v78	Listeria_ivanovii_subsp_londoniensis_202752_v2	0.8755
Listeria_monocytogenes_1639_v77	Listeria_ivanovii_subsp_londoniensis_202752_v2	0.8788
Listeria_monocytogenes_1639_v76	Listeria_ivanovii_subsp_londoniensis_202752_v2	0.8648
Listeria_monocytogenes_1639_v75	Listeria_ivanovii_subsp_londoniensis_202752_v2	0.8569
Listeria_monocytogenes_1639_v74	Listeria_ivanovii_subsp_londoniensis_202752_v2	0.8711
Listeria_monocytogenes_1639_v73	Listeria_ivanovii_subsp_londoniensis_202752_v2	0.8736
Listeria_monocytogenes_1639_v72	Listeria_ivanovii_subsp_londoniensis_202752_v2	0.8809
Listeria_monocytogenes_1639_v71	Listeria_ivanovii_subsp_londoniensis_202752_v2	0.8631
Listeria_monocytogenes_1639_v70	Listeria_ivanovii_subsp_londoniensis_202752_v2	0.8751
Listeria_monocytogenes_1639_v6	Listeria_ivanovii_subsp_londoniensis_202752_v2	0.8604
Listeria_monocytogenes_1639_v69	Listeria_ivanovii_subsp_londoniensis_202752_v2	0.8816
Listeria_monocytogenes_1639_v68	Listeria_ivanovii_subsp_londoniensis_202752_v2	0.8818
Listeria_monocytogenes_1639_v67	Listeria_ivanovii_subsp_londoniensis_202752_v2	0.8773
Listeria_monocytogenes_1639_v66	Listeria_ivanovii_subsp_londoniensis_202752_v2	0.8796
Listeria_monocytogenes_1639_v65	Listeria_ivanovii_subsp_londoniensis_202752_v2	0.8687
Listeria_monocytogenes_1639_v64	Listeria_ivanovii_subsp_londoniensis_202752_v2	0.8737
Listeria_monocytogenes_1639_v63	Listeria_ivanovii_subsp_londoniensis_202752_v2	0.8728
Listeria_monocytogenes_1639_v62	Listeria_ivanovii_subsp_londoniensis_202752_v2	0.8722
Listeria_monocytogenes_1639_v61	Listeria_ivanovii_subsp_londoniensis_202752_v2	0.8683
Listeria_monocytogenes_1639_v60	Listeria_ivanovii_subsp_londoniensis_202752_v2	0.8765
Listeria_monocytogenes_1639_v5	Listeria_ivanovii_subsp_londoniensis_202752_v2	0.8404
Listeria_monocytogenes_1639_v59	Listeria_ivanovii_subsp_londoniensis_202752_v2	0.8829
Listeria_monocytogenes_1639_v58	Listeria_ivanovii_subsp_londoniensis_202752_v2	0.8833
Listeria_monocytogenes_1639_v57	Listeria_ivanovii_subsp_londoniensis_202752_v2	0.8722
Listeria_monocytogenes_1639_v56	Listeria_ivanovii_subsp_londoniensis_202752_v2	0.8829
Listeria_monocytogenes_1639_v55	Listeria_ivanovii_subsp_londoniensis_202752_v2	0.8708
Listeria_monocytogenes_1639_v54	Listeria_ivanovii_subsp_londoniensis_202752_v2	0.889
Listeria_monocytogenes_1639_v53	Listeria_ivanovii_subsp_londoniensis_202752_v2	0.8754
Listeria_monocytogenes_1639_v52	Listeria_ivanovii_subsp_londoniensis_202752_v2	0.878
Listeria_monocytogenes_1639_v51	Listeria_ivanovii_subsp_londoniensis_202752_v2	0.8787
Listeria_monocytogenes_1639_v50	Listeria_ivanovii_subsp_londoniensis_202752_v2	0.8642
Listeria_monocytogenes_1639_v4	Listeria_ivanovii_subsp_londoniensis_202752_v2	0.8524
Listeria_monocytogenes_1639_v49	Listeria_ivanovii_subsp_londoniensis_202752_v2	0.8813
Listeria_monocytogenes_1639_v48	Listeria_ivanovii_subsp_londoniensis_202752_v2	0.8728
Listeria_monocytogenes_1639_v47	Listeria_ivanovii_subsp_londoniensis_202752_v2	0.8654
Listeria_monocytogenes_1639_v46	Listeria_ivanovii_subsp_londoniensis_202752_v2	0.8812
Listeria_monocytogenes_1639_v45	Listeria_ivanovii_subsp_londoniensis_202752_v2	0.8575
Listeria_monocytogenes_1639_v44	Listeria_ivanovii_subsp_londoniensis_202752_v2	0.8657
Listeria_monocytogenes_1639_v43	Listeria_ivanovii_subsp_londoniensis_202752_v2	0.8657
Listeria_monocytogenes_1639_v42	Listeria_ivanovii_subsp_londoniensis_202752_v2	0.862
Listeria_monocytogenes_1639_v41	Listeria_ivanovii_subsp_londoniensis_202752_v2	0.8537

Listeria_monocytogenes_1639_v40	Listeria_ivanovii_subsp_londoniensis_202752_v2	0.8786
Listeria_monocytogenes_1639_v3	Listeria_ivanovii_subsp_londoniensis_202752_v2	0.8527
Listeria_monocytogenes_1639_v39	Listeria_ivanovii_subsp_londoniensis_202752_v2	0.8746
Listeria_monocytogenes_1639_v38	Listeria_ivanovii_subsp_londoniensis_202752_v2	0.8711
Listeria_monocytogenes_1639_v37	Listeria_ivanovii_subsp_londoniensis_202752_v2	0.8646
Listeria_monocytogenes_1639_v36	Listeria_ivanovii_subsp_londoniensis_202752_v2	0.8679
Listeria_monocytogenes_1639_v35	Listeria_ivanovii_subsp_londoniensis_202752_v2	0.8792
Listeria_monocytogenes_1639_v34	Listeria_ivanovii_subsp_londoniensis_202752_v2	0.8742
Listeria_monocytogenes_1639_v33	Listeria_ivanovii_subsp_londoniensis_202752_v2	0.8707
Listeria_monocytogenes_1639_v32	Listeria_ivanovii_subsp_londoniensis_202752_v2	0.8633
Listeria_monocytogenes_1639_v31	Listeria_ivanovii_subsp_londoniensis_202752_v2	0.8742
Listeria_monocytogenes_1639_v30	Listeria_ivanovii_subsp_londoniensis_202752_v2	0.8731
Listeria_monocytogenes_1639_v2	Listeria_ivanovii_subsp_londoniensis_202752_v2	0.8444
Listeria_monocytogenes_1639_v29	Listeria_ivanovii_subsp_londoniensis_202752_v2	0.8658
Listeria_monocytogenes_1639_v28	Listeria_ivanovii_subsp_londoniensis_202752_v2	0.8685
Listeria_monocytogenes_1639_v27	Listeria_ivanovii_subsp_londoniensis_202752_v2	0.8617
Listeria_monocytogenes_1639_v26	Listeria_ivanovii_subsp_londoniensis_202752_v2	0.8614
Listeria_monocytogenes_1639_v25	Listeria_ivanovii_subsp_londoniensis_202752_v2	0.8611
Listeria_monocytogenes_1639_v24	Listeria_ivanovii_subsp_londoniensis_202752_v2	0.8739
Listeria_monocytogenes_1639_v23	Listeria_ivanovii_subsp_londoniensis_202752_v2	0.8739
Listeria_monocytogenes_1639_v22	Listeria_ivanovii_subsp_londoniensis_202752_v2	0.8712
Listeria_monocytogenes_1639_v21	Listeria_ivanovii_subsp_londoniensis_202752_v2	0.874
Listeria_monocytogenes_1639_v20	Listeria_ivanovii_subsp_londoniensis_202752_v2	0.8711
Listeria_monocytogenes_1639_v1	Listeria_ivanovii_subsp_londoniensis_202752_v2	0.8562
Listeria_monocytogenes_1639_v19	Listeria_ivanovii_subsp_londoniensis_202752_v2	0.8708
Listeria_monocytogenes_1639_v198	Listeria_ivanovii_subsp_londoniensis_202752_v2	0.8481
Listeria_monocytogenes_1639_v197	Listeria_ivanovii_subsp_londoniensis_202752_v2	0.8683
Listeria_monocytogenes_1639_v196	Listeria_ivanovii_subsp_londoniensis_202752_v2	0.8652
Listeria_monocytogenes_1639_v195	Listeria_ivanovii_subsp_londoniensis_202752_v2	0.8909
Listeria_monocytogenes_1639_v194	Listeria_ivanovii_subsp_londoniensis_202752_v2	0.8903
Listeria_monocytogenes_1639_v193	Listeria_ivanovii_subsp_londoniensis_202752_v2	0.8899
Listeria_monocytogenes_1639_v192	Listeria_ivanovii_subsp_londoniensis_202752_v2	0.8681
Listeria_monocytogenes_1639_v191	Listeria_ivanovii_subsp_londoniensis_202752_v2	0.8942
Listeria_monocytogenes_1639_v190	Listeria_ivanovii_subsp_londoniensis_202752_v2	0.8943
Listeria_monocytogenes_1639_v18	Listeria_ivanovii_subsp_londoniensis_202752_v2	0.8667
Listeria_monocytogenes_1639_v189	Listeria_ivanovii_subsp_londoniensis_202752_v2	0.8936
Listeria_monocytogenes_1639_v188	Listeria_ivanovii_subsp_londoniensis_202752_v2	0.8929
Listeria_monocytogenes_1639_v187	Listeria_ivanovii_subsp_londoniensis_202752_v2	0.8939
Listeria_monocytogenes_1639_v186	Listeria_ivanovii_subsp_londoniensis_202752_v2	0.8947
Listeria_monocytogenes_1639_v185	Listeria_ivanovii_subsp_londoniensis_202752_v2	0.8943
Listeria_monocytogenes_1639_v184	Listeria_ivanovii_subsp_londoniensis_202752_v2	0.8935
Listeria_monocytogenes_1639_v183	Listeria_ivanovii_subsp_londoniensis_202752_v2	0.8912
Listeria_monocytogenes_1639_v182	Listeria_ivanovii_subsp_londoniensis_202752_v2	0.8929
Listeria_monocytogenes_1639_v181	Listeria_ivanovii_subsp_londoniensis_202752_v2	0.879
Listeria_monocytogenes_1639_v180	Listeria_ivanovii_subsp_londoniensis_202752_v2	0.879
Listeria_monocytogenes_1639_v17	Listeria_ivanovii_subsp_londoniensis_202752_v2	0.8667
Listeria_monocytogenes_1639_v179	Listeria_ivanovii_subsp_londoniensis_202752_v2	0.8796
Listeria_monocytogenes_1639_v178	Listeria_ivanovii_subsp_londoniensis_202752_v2	0.8931
Listeria_monocytogenes_1639_v177	Listeria_ivanovii_subsp_londoniensis_202752_v2	0.8936
Listeria_monocytogenes_1639_v176	Listeria_ivanovii_subsp_londoniensis_202752_v2	0.8897
Listeria_monocytogenes_1639_v175	Listeria_ivanovii_subsp_londoniensis_202752_v2	0.8838
Listeria_monocytogenes_1639_v174	Listeria_ivanovii_subsp_londoniensis_202752_v2	0.8949
Listeria_monocytogenes_1639_v173	Listeria_ivanovii_subsp_londoniensis_202752_v2	0.8964
Listeria_monocytogenes_1639_v172	Listeria_ivanovii_subsp_londoniensis_202752_v2	0.8854
Listeria_monocytogenes_1639_v171	Listeria_ivanovii_subsp_londoniensis_202752_v2	0.8819
Listeria_monocytogenes_1639_v170	Listeria_ivanovii_subsp_londoniensis_202752_v2	0.8878
Listeria_monocytogenes_1639_v16	Listeria_ivanovii_subsp_londoniensis_202752_v2	0.8541
Listeria_monocytogenes_1639_v169	Listeria_ivanovii_subsp_londoniensis_202752_v2	0.8887
Listeria_monocytogenes_1639_v168	Listeria_ivanovii_subsp_londoniensis_202752_v2	0.8896
Listeria_monocytogenes_1639_v167	Listeria_ivanovii_subsp_londoniensis_202752_v2	0.8831
Listeria_monocytogenes_1639_v166	Listeria_ivanovii_subsp_londoniensis_202752_v2	0.8899
Listeria_monocytogenes_1639_v165	Listeria_ivanovii_subsp_londoniensis_202752_v2	0.8883
Listeria_monocytogenes_1639_v164	Listeria_ivanovii_subsp_londoniensis_202752_v2	0.8941
Listeria_monocytogenes_1639_v163	Listeria_ivanovii_subsp_londoniensis_202752_v2	0.8938
Listeria_monocytogenes_1639_v162	Listeria_ivanovii_subsp_londoniensis_202752_v2	0.8941
Listeria_monocytogenes_1639_v161	Listeria_ivanovii_subsp_londoniensis_202752_v2	0.8953
Listeria_monocytogenes_1639_v160	Listeria_ivanovii_subsp_londoniensis_202752_v2	0.8957
Listeria_monocytogenes_1639_v15	Listeria_ivanovii_subsp_londoniensis_202752_v2	0.8577
Listeria_monocytogenes_1639_v159	Listeria_ivanovii_subsp_londoniensis_202752_v2	0.8936
Listeria_monocytogenes_1639_v158	Listeria_ivanovii_subsp_londoniensis_202752_v2	0.895
Listeria_monocytogenes_1639_v157	Listeria_ivanovii_subsp_londoniensis_202752_v2	0.895
Listeria_monocytogenes_1639_v156	Listeria_ivanovii_subsp_londoniensis_202752_v2	0.8727
Listeria_monocytogenes_1639_v155	Listeria_ivanovii_subsp_londoniensis_202752_v2	0.8817
Listeria_monocytogenes_1639_v154	Listeria_ivanovii_subsp_londoniensis_202752_v2	0.8833
Listeria_monocytogenes_1639_v153	Listeria_ivanovii_subsp_londoniensis_202752_v2	0.8824
Listeria_monocytogenes_1639_v152	Listeria_ivanovii_subsp_londoniensis_202752_v2	0.894
Listeria_monocytogenes_1639_v151	Listeria_ivanovii_subsp_londoniensis_202752_v2	0.894
Listeria_monocytogenes_1639_v150	Listeria_ivanovii_subsp_londoniensis_202752_v2	0.884
Listeria_monocytogenes_1639_v14	Listeria_ivanovii_subsp_londoniensis_202752_v2	0.8633
Listeria_monocytogenes_1639_v149	Listeria_ivanovii_subsp_londoniensis_202752_v2	0.8831
Listeria_monocytogenes_1639_v148	Listeria_ivanovii_subsp_londoniensis_202752_v2	0.8926
Listeria_monocytogenes_1639_v147	Listeria_ivanovii_subsp_londoniensis_202752_v2	0.8805
Listeria_monocytogenes_1639_v146	Listeria_ivanovii_subsp_londoniensis_202752_v2	0.8958
Listeria_monocytogenes_1639_v145	Listeria_ivanovii_subsp_londoniensis_202752_v2	0.8891
Listeria_monocytogenes_1639_v144	Listeria_ivanovii_subsp_londoniensis_202752_v2	0.8953
Listeria_monocytogenes_1639_v143	Listeria_ivanovii_subsp_londoniensis_202752_v2	0.8917
Listeria_monocytogenes_1639_v142	Listeria_ivanovii_subsp_londoniensis_202752_v2	0.896
Listeria_monocytogenes_1639_v141	Listeria_ivanovii_subsp_londoniensis_202752_v2	0.8939
Listeria_monocytogenes_1639_v140	Listeria_ivanovii_subsp_londoniensis_202752_v2	0.8956
Listeria_monocytogenes_1639_v13	Listeria_ivanovii_subsp_londoniensis_202752_v2	0.858
Listeria_monocytogenes_1639_v139	Listeria_ivanovii_subsp_londoniensis_202752_v2	0.8915

Listeria_monocytogenes_1639_v138	Listeria_ivanovii_subsp_londoniensis_202752_v2	0.8961
Listeria_monocytogenes_1639_v137	Listeria_ivanovii_subsp_londoniensis_202752_v2	0.8953
Listeria_monocytogenes_1639_v136	Listeria_ivanovii_subsp_londoniensis_202752_v2	0.8792
Listeria_monocytogenes_1639_v135	Listeria_ivanovii_subsp_londoniensis_202752_v2	0.8679
Listeria_monocytogenes_1639_v134	Listeria_ivanovii_subsp_londoniensis_202752_v2	0.8911
Listeria_monocytogenes_1639_v133	Listeria_ivanovii_subsp_londoniensis_202752_v2	0.8852
Listeria_monocytogenes_1639_v132	Listeria_ivanovii_subsp_londoniensis_202752_v2	0.8659
Listeria_monocytogenes_1639_v131	Listeria_ivanovii_subsp_londoniensis_202752_v2	0.8825
Listeria_monocytogenes_1639_v130	Listeria_ivanovii_subsp_londoniensis_202752_v2	0.8796
Listeria_monocytogenes_1639_v12	Listeria_ivanovii_subsp_londoniensis_202752_v2	0.8601
Listeria_monocytogenes_1639_v129	Listeria_ivanovii_subsp_londoniensis_202752_v2	0.891
Listeria_monocytogenes_1639_v128	Listeria_ivanovii_subsp_londoniensis_202752_v2	0.8902
Listeria_monocytogenes_1639_v127	Listeria_ivanovii_subsp_londoniensis_202752_v2	0.8812
Listeria_monocytogenes_1639_v126	Listeria_ivanovii_subsp_londoniensis_202752_v2	0.8894
Listeria_monocytogenes_1639_v125	Listeria_ivanovii_subsp_londoniensis_202752_v2	0.8814
Listeria_monocytogenes_1639_v124	Listeria_ivanovii_subsp_londoniensis_202752_v2	0.8834
Listeria_monocytogenes_1639_v123	Listeria_ivanovii_subsp_londoniensis_202752_v2	0.896
Listeria_monocytogenes_1639_v122	Listeria_ivanovii_subsp_londoniensis_202752_v2	0.8959
Listeria_monocytogenes_1639_v121	Listeria_ivanovii_subsp_londoniensis_202752_v2	0.8959
Listeria_monocytogenes_1639_v120	Listeria_ivanovii_subsp_londoniensis_202752_v2	0.896
Listeria_monocytogenes_1639_v11	Listeria_ivanovii_subsp_londoniensis_202752_v2	0.8591
Listeria_monocytogenes_1639_v119	Listeria_ivanovii_subsp_londoniensis_202752_v2	0.8959
Listeria_monocytogenes_1639_v118	Listeria_ivanovii_subsp_londoniensis_202752_v2	0.8959
Listeria_monocytogenes_1639_v117	Listeria_ivanovii_subsp_londoniensis_202752_v2	0.8811
Listeria_monocytogenes_1639_v116	Listeria_ivanovii_subsp_londoniensis_202752_v2	0.8904
Listeria_monocytogenes_1639_v115	Listeria_ivanovii_subsp_londoniensis_202752_v2	0.8907
Listeria_monocytogenes_1639_v114	Listeria_ivanovii_subsp_londoniensis_202752_v2	0.8759
Listeria_monocytogenes_1639_v113	Listeria_ivanovii_subsp_londoniensis_202752_v2	0.8852
Listeria_monocytogenes_1639_v112	Listeria_ivanovii_subsp_londoniensis_202752_v2	0.8858
Listeria_monocytogenes_1639_v111	Listeria_ivanovii_subsp_londoniensis_202752_v2	0.8859
Listeria_monocytogenes_1639_v110	Listeria_ivanovii_subsp_londoniensis_202752_v2	0.8756
Listeria_monocytogenes_1639_v10	Listeria_ivanovii_subsp_londoniensis_202752_v2	0.8551
Listeria_monocytogenes_1639_v109	Listeria_ivanovii_subsp_londoniensis_202752_v2	0.8756
Listeria_monocytogenes_1639_v108	Listeria_ivanovii_subsp_londoniensis_202752_v2	0.8831
Listeria_monocytogenes_1639_v107	Listeria_ivanovii_subsp_londoniensis_202752_v2	0.8917
Listeria_monocytogenes_1639_v106	Listeria_ivanovii_subsp_londoniensis_202752_v2	0.8917
Listeria_monocytogenes_1639_v105	Listeria_ivanovii_subsp_londoniensis_202752_v2	0.892
Listeria_monocytogenes_1639_v104	Listeria_ivanovii_subsp_londoniensis_202752_v2	0.892
Listeria_monocytogenes_1639_v103	Listeria_ivanovii_subsp_londoniensis_202752_v2	0.892
Listeria_monocytogenes_1639_v102	Listeria_ivanovii_subsp_londoniensis_202752_v2	0.8759
Listeria_monocytogenes_1639_v101	Listeria_ivanovii_subsp_londoniensis_202752_v2	0.8659
Listeria_monocytogenes_1639_v100	Listeria_ivanovii_subsp_londoniensis_202752_v2	0.8649
Listeria_monocytogenes_1639	Listeria_ivanovii_subsp_londoniensis_202752_v2	0.894
Listeria_monocytogenes_10403S_393133	Listeria_ivanovii_subsp_londoniensis_202752_v2	0.886
Listeria_monocytogenes_08-5923_637381	Listeria_ivanovii_subsp_londoniensis_202752_v2	0.8657
Listeria_monocytogenes_08-5578_653938	Listeria_ivanovii_subsp_londoniensis_202752_v2	0.8538
Listeria_monocytogenes_07PF0776_1126011	Listeria_ivanovii_subsp_londoniensis_202752_v2	0.8942
Listeria_marthii_FSL_S4-120_702457	Listeria_ivanovii_subsp_londoniensis_202752_v2	0.8169
Listeria_marthii_529731_v1	Listeria_ivanovii_subsp_londoniensis_202752_v2	0.8576
Listeria_marthii_529731	Listeria_ivanovii_subsp_londoniensis_202752_v2	0.8647
Listeria_ivanovii_WSLC3009_1457190	Listeria_ivanovii_subsp_londoniensis_202752_v2	0.9013
Listeria_ivanovii_subsp_londoniensis_202752_v2	Listeria_ivanovii_subsp_londoniensis_202752_v2	0.0644
Listeria_welshimeri_1643	Listeria_ivanovii_WSLC3009_1457190	0.8612
Listeria_welshimeri_serovar_6b_str_SLCC5334_386043	Listeria_ivanovii_WSLC3009_1457190	0.8608
Listeria_seeligeri_serovar_1_2b_str_SLCC3954_683837	Listeria_ivanovii_WSLC3009_1457190	0.8822
Listeria_seeligeri_FSL_S4-171_702452	Listeria_ivanovii_WSLC3009_1457190	0.8184
Listeria_seeligeri_FSL_N1-067_702453	Listeria_ivanovii_WSLC3009_1457190	0.825
Listeria_seeligeri_1640_v1	Listeria_ivanovii_WSLC3009_1457190	0.8907
Listeria_seeligeri_1640	Listeria_ivanovii_WSLC3009_1457190	0.8758
Listeria_monocytogenes_WSLC1042_1457188	Listeria_ivanovii_WSLC3009_1457190	0.8979
Listeria_monocytogenes_WSLC1001_1457187	Listeria_ivanovii_WSLC3009_1457190	0.8809
Listeria_monocytogenes_SLCC7179_879090	Listeria_ivanovii_WSLC3009_1457190	0.8882
Listeria_monocytogenes_SLCC5850_882096	Listeria_ivanovii_WSLC3009_1457190	0.8857
Listeria_monocytogenes_SLCC2755_932919	Listeria_ivanovii_WSLC3009_1457190	0.8874
Listeria_monocytogenes_SLCC2540_879089	Listeria_ivanovii_WSLC3009_1457190	0.8899
Listeria_monocytogenes_SLCC2479_882020	Listeria_ivanovii_WSLC3009_1457190	0.8794
Listeria_monocytogenes_SLCC2378_879088	Listeria_ivanovii_WSLC3009_1457190	0.899
Listeria_monocytogenes_SLCC2376_882097	Listeria_ivanovii_WSLC3009_1457190	0.8971
Listeria_monocytogenes_SLCC2372_932920	Listeria_ivanovii_WSLC3009_1457190	0.8721
Listeria_monocytogenes_serotype_4b_str_LL195_1230340	Listeria_ivanovii_WSLC3009_1457190	0.9049
Listeria_monocytogenes_serotype_4b_str_LIS0087_1134669	Listeria_ivanovii_WSLC3009_1457190	0.8618
Listeria_monocytogenes_serotype_4b_str_F2365_265669	Listeria_ivanovii_WSLC3009_1457190	0.9049
Listeria_monocytogenes_serotype_4b_str_CLIP_80459_568819	Listeria_ivanovii_WSLC3009_1457190	0.9036
Listeria_monocytogenes_serotype_4b_str_81-0861_1196161	Listeria_ivanovii_WSLC3009_1457190	0.8883
Listeria_monocytogenes_serotype_4b_str_81-0592_1196160	Listeria_ivanovii_WSLC3009_1457190	0.8894
Listeria_monocytogenes_serotype_4b_str_81-0558_1196159	Listeria_ivanovii_WSLC3009_1457190	0.8901
Listeria_monocytogenes_serotype_4b_str_10-0809_1196162	Listeria_ivanovii_WSLC3009_1457190	0.8898
Listeria_monocytogenes_serotype_4b_str_02-6680_1196175	Listeria_ivanovii_WSLC3009_1457190	0.9022
Listeria_monocytogenes_serotype_4b_str_02-6679_1196174	Listeria_ivanovii_WSLC3009_1457190	0.9022
Listeria_monocytogenes_serotype_4b_str_02-1792_1196173	Listeria_ivanovii_WSLC3009_1457190	0.8898
Listeria_monocytogenes_serotype_4b_str_02-1289_1196172	Listeria_ivanovii_WSLC3009_1457190	0.8898
Listeria_monocytogenes_serotype_4b_str_02-1103_1196171	Listeria_ivanovii_WSLC3009_1457190	0.8898
Listeria_monocytogenes_serotype_3c_str_10-5027_1196195	Listeria_ivanovii_WSLC3009_1457190	0.8782
Listeria_monocytogenes_serotype_1_2c_str_10-5026_1196194	Listeria_ivanovii_WSLC3009_1457190	0.879
Listeria_monocytogenes_serotype_1_2c_str_10-5025_1196193	Listeria_ivanovii_WSLC3009_1457190	0.879
Listeria_monocytogenes_serotype_1_2b_str_10-0811_1196164	Listeria_ivanovii_WSLC3009_1457190	0.8791
Listeria_monocytogenes_serotype_1_2b_str_10-0810_1196163	Listeria_ivanovii_WSLC3009_1457190	0.8791
Listeria_monocytogenes_serotype_1_2a_str_99-6370_1196179	Listeria_ivanovii_WSLC3009_1457190	0.8657
Listeria_monocytogenes_serotype_1_2a_str_98-2035_1196178	Listeria_ivanovii_WSLC3009_1457190	0.8657
Listeria_monocytogenes_serotype_1_2a_str_95-0093_1196177	Listeria_ivanovii_WSLC3009_1457190	0.8708
Listeria_monocytogenes_serotype_1_2a_str_88-0478_1196176	Listeria_ivanovii_WSLC3009_1457190	0.8696

Listeria_monocytogenes_serotype_1_2a_str_10-5024_1196192	Listeria_ivanovii_WSLC3009_1457190	0.8708
Listeria_monocytogenes_serotype_1_2a_str_10-4758_1196168	Listeria_ivanovii_WSLC3009_1457190	0.8851
Listeria_monocytogenes_serotype_1_2a_str_10-4754_1196167	Listeria_ivanovii_WSLC3009_1457190	0.8854
Listeria_monocytogenes_serotype_1_2a_str_10-1321_1196191	Listeria_ivanovii_WSLC3009_1457190	0.8708
Listeria_monocytogenes_serotype_1_2a_str_10-1047_1196190	Listeria_ivanovii_WSLC3009_1457190	0.8715
Listeria_monocytogenes_serotype_1_2a_str_10-1046_1196189	Listeria_ivanovii_WSLC3009_1457190	0.8715
Listeria_monocytogenes_serotype_1_2a_str_10-0934_1196170	Listeria_ivanovii_WSLC3009_1457190	0.8955
Listeria_monocytogenes_serotype_1_2a_str_10-0933_1196169	Listeria_ivanovii_WSLC3009_1457190	0.8955
Listeria_monocytogenes_serotype_1_2a_str_10-0815_1196188	Listeria_ivanovii_WSLC3009_1457190	0.867
Listeria_monocytogenes_serotype_1_2a_str_10-0814_1196187	Listeria_ivanovii_WSLC3009_1457190	0.8712
Listeria_monocytogenes_serotype_1_2a_str_10-0813_1196166	Listeria_ivanovii_WSLC3009_1457190	0.8786
Listeria_monocytogenes_serotype_1_2a_str_10-0812_1196165	Listeria_ivanovii_WSLC3009_1457190	0.8786
Listeria_monocytogenes_serotype_1_2a_str_08-7669_1196186	Listeria_ivanovii_WSLC3009_1457190	0.8793
Listeria_monocytogenes_serotype_1_2a_str_08-7374_1196185	Listeria_ivanovii_WSLC3009_1457190	0.8708
Listeria_monocytogenes_serotype_1_2a_str_08-6997_1196184	Listeria_ivanovii_WSLC3009_1457190	0.867
Listeria_monocytogenes_serotype_1_2a_str_08-6569_1196183	Listeria_ivanovii_WSLC3009_1457190	0.867
Listeria_monocytogenes_serotype_1_2a_str_08-6056_1196182	Listeria_ivanovii_WSLC3009_1457190	0.867
Listeria_monocytogenes_serotype_1_2a_str_04-5457_1196181	Listeria_ivanovii_WSLC3009_1457190	0.8708
Listeria_monocytogenes_serotype_1_2a_str_02-5993_1196180	Listeria_ivanovii_WSLC3009_1457190	0.8708
Listeria_monocytogenes_R479a_1437838	Listeria_ivanovii_WSLC3009_1457190	0.8705
Listeria_monocytogenes_N53-1_1234142	Listeria_ivanovii_WSLC3009_1457190	0.8496
Listeria_monocytogenes_M7_1030009	Listeria_ivanovii_WSLC3009_1457190	0.8605
Listeria_monocytogenes_LO28_393132	Listeria_ivanovii_WSLC3009_1457190	0.8781
Listeria_monocytogenes_L99_563174	Listeria_ivanovii_WSLC3009_1457190	0.8608
Listeria_monocytogenes_L312_882094	Listeria_ivanovii_WSLC3009_1457190	0.9036
Listeria_monocytogenes_J1816_930781	Listeria_ivanovii_WSLC3009_1457190	0.8808
Listeria_monocytogenes_J1-220_930782	Listeria_ivanovii_WSLC3009_1457190	0.8786
Listeria_monocytogenes_J0161_393130	Listeria_ivanovii_WSLC3009_1457190	0.8777
Listeria_monocytogenes_HCC23_552536	Listeria_ivanovii_WSLC3009_1457190	0.8605
Listeria_monocytogenes_FSL_R2-561_393126	Listeria_ivanovii_WSLC3009_1457190	0.8776
Listeria_monocytogenes_FSL_J1-208_393119	Listeria_ivanovii_WSLC3009_1457190	0.8741
Listeria_monocytogenes_FSL_J1-175_393118	Listeria_ivanovii_WSLC3009_1457190	0.8945
Listeria_monocytogenes_Finland_1998_393127	Listeria_ivanovii_WSLC3009_1457190	0.8966
Listeria_monocytogenes_EGD-e_169963_v1	Listeria_ivanovii_WSLC3009_1457190	0.886
Listeria_monocytogenes_EGD-e_169963	Listeria_ivanovii_WSLC3009_1457190	0.8863
Listeria_monocytogenes_EGD_1334565	Listeria_ivanovii_WSLC3009_1457190	0.886
Listeria_monocytogenes_CFSAN002349_1299895	Listeria_ivanovii_WSLC3009_1457190	0.8997
Listeria_monocytogenes_ATCC_19117_882095_v1	Listeria_ivanovii_WSLC3009_1457190	0.8975
Listeria_monocytogenes_ATCC_19117_882095	Listeria_ivanovii_WSLC3009_1457190	0.8971
Listeria_monocytogenes_6179_1288295	Listeria_ivanovii_WSLC3009_1457190	0.8623
Listeria_monocytogenes_1639_v9	Listeria_ivanovii_WSLC3009_1457190	0.8671
Listeria_monocytogenes_1639_v99	Listeria_ivanovii_WSLC3009_1457190	0.8808
Listeria_monocytogenes_1639_v98	Listeria_ivanovii_WSLC3009_1457190	0.8764
Listeria_monocytogenes_1639_v97	Listeria_ivanovii_WSLC3009_1457190	0.8928
Listeria_monocytogenes_1639_v96	Listeria_ivanovii_WSLC3009_1457190	0.8802
Listeria_monocytogenes_1639_v95	Listeria_ivanovii_WSLC3009_1457190	0.8802
Listeria_monocytogenes_1639_v94	Listeria_ivanovii_WSLC3009_1457190	0.8805
Listeria_monocytogenes_1639_v93	Listeria_ivanovii_WSLC3009_1457190	0.8734
Listeria_monocytogenes_1639_v92	Listeria_ivanovii_WSLC3009_1457190	0.8805
Listeria_monocytogenes_1639_v91	Listeria_ivanovii_WSLC3009_1457190	0.8871
Listeria_monocytogenes_1639_v90	Listeria_ivanovii_WSLC3009_1457190	0.8764
Listeria_monocytogenes_1639_v8	Listeria_ivanovii_WSLC3009_1457190	0.8602
Listeria_monocytogenes_1639_v89	Listeria_ivanovii_WSLC3009_1457190	0.8935
Listeria_monocytogenes_1639_v88	Listeria_ivanovii_WSLC3009_1457190	0.8776
Listeria_monocytogenes_1639_v87	Listeria_ivanovii_WSLC3009_1457190	0.8732
Listeria_monocytogenes_1639_v86	Listeria_ivanovii_WSLC3009_1457190	0.8735
Listeria_monocytogenes_1639_v85	Listeria_ivanovii_WSLC3009_1457190	0.8877
Listeria_monocytogenes_1639_v84	Listeria_ivanovii_WSLC3009_1457190	0.8796
Listeria_monocytogenes_1639_v83	Listeria_ivanovii_WSLC3009_1457190	0.8869
Listeria_monocytogenes_1639_v82	Listeria_ivanovii_WSLC3009_1457190	0.8863
Listeria_monocytogenes_1639_v81	Listeria_ivanovii_WSLC3009_1457190	0.8779
Listeria_monocytogenes_1639_v80	Listeria_ivanovii_WSLC3009_1457190	0.8834
Listeria_monocytogenes_1639_v7	Listeria_ivanovii_WSLC3009_1457190	0.8602
Listeria_monocytogenes_1639_v79	Listeria_ivanovii_WSLC3009_1457190	0.8763
Listeria_monocytogenes_1639_v78	Listeria_ivanovii_WSLC3009_1457190	0.8773
Listeria_monocytogenes_1639_v77	Listeria_ivanovii_WSLC3009_1457190	0.8794
Listeria_monocytogenes_1639_v76	Listeria_ivanovii_WSLC3009_1457190	0.8699
Listeria_monocytogenes_1639_v75	Listeria_ivanovii_WSLC3009_1457190	0.8608
Listeria_monocytogenes_1639_v74	Listeria_ivanovii_WSLC3009_1457190	0.8743
Listeria_monocytogenes_1639_v73	Listeria_ivanovii_WSLC3009_1457190	0.8735
Listeria_monocytogenes_1639_v72	Listeria_ivanovii_WSLC3009_1457190	0.8857
Listeria_monocytogenes_1639_v71	Listeria_ivanovii_WSLC3009_1457190	0.8644
Listeria_monocytogenes_1639_v70	Listeria_ivanovii_WSLC3009_1457190	0.8758
Listeria_monocytogenes_1639_v6	Listeria_ivanovii_WSLC3009_1457190	0.8598
Listeria_monocytogenes_1639_v69	Listeria_ivanovii_WSLC3009_1457190	0.8894
Listeria_monocytogenes_1639_v68	Listeria_ivanovii_WSLC3009_1457190	0.8817
Listeria_monocytogenes_1639_v67	Listeria_ivanovii_WSLC3009_1457190	0.8863
Listeria_monocytogenes_1639_v66	Listeria_ivanovii_WSLC3009_1457190	0.8848
Listeria_monocytogenes_1639_v65	Listeria_ivanovii_WSLC3009_1457190	0.8738
Listeria_monocytogenes_1639_v64	Listeria_ivanovii_WSLC3009_1457190	0.8766
Listeria_monocytogenes_1639_v63	Listeria_ivanovii_WSLC3009_1457190	0.8801
Listeria_monocytogenes_1639_v62	Listeria_ivanovii_WSLC3009_1457190	0.874
Listeria_monocytogenes_1639_v61	Listeria_ivanovii_WSLC3009_1457190	0.8734
Listeria_monocytogenes_1639_v60	Listeria_ivanovii_WSLC3009_1457190	0.879
Listeria_monocytogenes_1639_v5	Listeria_ivanovii_WSLC3009_1457190	0.8475
Listeria_monocytogenes_1639_v59	Listeria_ivanovii_WSLC3009_1457190	0.8873
Listeria_monocytogenes_1639_v58	Listeria_ivanovii_WSLC3009_1457190	0.8903
Listeria_monocytogenes_1639_v57	Listeria_ivanovii_WSLC3009_1457190	0.8773
Listeria_monocytogenes_1639_v56	Listeria_ivanovii_WSLC3009_1457190	0.8873
Listeria_monocytogenes_1639_v55	Listeria_ivanovii_WSLC3009_1457190	0.8733
Listeria_monocytogenes_1639_v54	Listeria_ivanovii_WSLC3009_1457190	0.8922

Listeria_monocytogenes_1639_v53	Listeria_ivanovii_WSLC3009_1457190	0.8801
Listeria_monocytogenes_1639_v52	Listeria_ivanovii_WSLC3009_1457190	0.8843
Listeria_monocytogenes_1639_v51	Listeria_ivanovii_WSLC3009_1457190	0.8823
Listeria_monocytogenes_1639_v50	Listeria_ivanovii_WSLC3009_1457190	0.8697
Listeria_monocytogenes_1639_v4	Listeria_ivanovii_WSLC3009_1457190	0.8591
Listeria_monocytogenes_1639_v49	Listeria_ivanovii_WSLC3009_1457190	0.8895
Listeria_monocytogenes_1639_v48	Listeria_ivanovii_WSLC3009_1457190	0.8737
Listeria_monocytogenes_1639_v47	Listeria_ivanovii_WSLC3009_1457190	0.8705
Listeria_monocytogenes_1639_v46	Listeria_ivanovii_WSLC3009_1457190	0.881
Listeria_monocytogenes_1639_v45	Listeria_ivanovii_WSLC3009_1457190	0.8621
Listeria_monocytogenes_1639_v44	Listeria_ivanovii_WSLC3009_1457190	0.8708
Listeria_monocytogenes_1639_v43	Listeria_ivanovii_WSLC3009_1457190	0.8708
Listeria_monocytogenes_1639_v42	Listeria_ivanovii_WSLC3009_1457190	0.8648
Listeria_monocytogenes_1639_v41	Listeria_ivanovii_WSLC3009_1457190	0.8539
Listeria_monocytogenes_1639_v40	Listeria_ivanovii_WSLC3009_1457190	0.8788
Listeria_monocytogenes_1639_v3	Listeria_ivanovii_WSLC3009_1457190	0.8595
Listeria_monocytogenes_1639_v39	Listeria_ivanovii_WSLC3009_1457190	0.8823
Listeria_monocytogenes_1639_v38	Listeria_ivanovii_WSLC3009_1457190	0.875
Listeria_monocytogenes_1639_v37	Listeria_ivanovii_WSLC3009_1457190	0.8708
Listeria_monocytogenes_1639_v36	Listeria_ivanovii_WSLC3009_1457190	0.8677
Listeria_monocytogenes_1639_v35	Listeria_ivanovii_WSLC3009_1457190	0.8794
Listeria_monocytogenes_1639_v34	Listeria_ivanovii_WSLC3009_1457190	0.8737
Listeria_monocytogenes_1639_v33	Listeria_ivanovii_WSLC3009_1457190	0.8791
Listeria_monocytogenes_1639_v32	Listeria_ivanovii_WSLC3009_1457190	0.8695
Listeria_monocytogenes_1639_v31	Listeria_ivanovii_WSLC3009_1457190	0.8741
Listeria_monocytogenes_1639_v30	Listeria_ivanovii_WSLC3009_1457190	0.8801
Listeria_monocytogenes_1639_v2	Listeria_ivanovii_WSLC3009_1457190	0.8493
Listeria_monocytogenes_1639_v29	Listeria_ivanovii_WSLC3009_1457190	0.8656
Listeria_monocytogenes_1639_v28	Listeria_ivanovii_WSLC3009_1457190	0.8698
Listeria_monocytogenes_1639_v27	Listeria_ivanovii_WSLC3009_1457190	0.8615
Listeria_monocytogenes_1639_v26	Listeria_ivanovii_WSLC3009_1457190	0.8613
Listeria_monocytogenes_1639_v25	Listeria_ivanovii_WSLC3009_1457190	0.8613
Listeria_monocytogenes_1639_v24	Listeria_ivanovii_WSLC3009_1457190	0.879
Listeria_monocytogenes_1639_v23	Listeria_ivanovii_WSLC3009_1457190	0.8787
Listeria_monocytogenes_1639_v22	Listeria_ivanovii_WSLC3009_1457190	0.8798
Listeria_monocytogenes_1639_v21	Listeria_ivanovii_WSLC3009_1457190	0.8787
Listeria_monocytogenes_1639_v20	Listeria_ivanovii_WSLC3009_1457190	0.8761
Listeria_monocytogenes_1639_v1	Listeria_ivanovii_WSLC3009_1457190	0.8564
Listeria_monocytogenes_1639_v19	Listeria_ivanovii_WSLC3009_1457190	0.8758
Listeria_monocytogenes_1639_v198	Listeria_ivanovii_WSLC3009_1457190	0.8524
Listeria_monocytogenes_1639_v197	Listeria_ivanovii_WSLC3009_1457190	0.8738
Listeria_monocytogenes_1639_v196	Listeria_ivanovii_WSLC3009_1457190	0.8681
Listeria_monocytogenes_1639_v195	Listeria_ivanovii_WSLC3009_1457190	0.8962
Listeria_monocytogenes_1639_v194	Listeria_ivanovii_WSLC3009_1457190	0.8972
Listeria_monocytogenes_1639_v193	Listeria_ivanovii_WSLC3009_1457190	0.8975
Listeria_monocytogenes_1639_v192	Listeria_ivanovii_WSLC3009_1457190	0.8663
Listeria_monocytogenes_1639_v191	Listeria_ivanovii_WSLC3009_1457190	0.8968
Listeria_monocytogenes_1639_v190	Listeria_ivanovii_WSLC3009_1457190	0.8965
Listeria_monocytogenes_1639_v18	Listeria_ivanovii_WSLC3009_1457190	0.8717
Listeria_monocytogenes_1639_v189	Listeria_ivanovii_WSLC3009_1457190	0.8962
Listeria_monocytogenes_1639_v188	Listeria_ivanovii_WSLC3009_1457190	0.899
Listeria_monocytogenes_1639_v187	Listeria_ivanovii_WSLC3009_1457190	0.8969
Listeria_monocytogenes_1639_v186	Listeria_ivanovii_WSLC3009_1457190	0.8969
Listeria_monocytogenes_1639_v185	Listeria_ivanovii_WSLC3009_1457190	0.8969
Listeria_monocytogenes_1639_v184	Listeria_ivanovii_WSLC3009_1457190	0.8961
Listeria_monocytogenes_1639_v183	Listeria_ivanovii_WSLC3009_1457190	0.8976
Listeria_monocytogenes_1639_v182	Listeria_ivanovii_WSLC3009_1457190	0.8998
Listeria_monocytogenes_1639_v181	Listeria_ivanovii_WSLC3009_1457190	0.8842
Listeria_monocytogenes_1639_v180	Listeria_ivanovii_WSLC3009_1457190	0.8842
Listeria_monocytogenes_1639_v17	Listeria_ivanovii_WSLC3009_1457190	0.8721
Listeria_monocytogenes_1639_v179	Listeria_ivanovii_WSLC3009_1457190	0.8864
Listeria_monocytogenes_1639_v178	Listeria_ivanovii_WSLC3009_1457190	0.9008
Listeria_monocytogenes_1639_v177	Listeria_ivanovii_WSLC3009_1457190	0.8977
Listeria_monocytogenes_1639_v176	Listeria_ivanovii_WSLC3009_1457190	0.8958
Listeria_monocytogenes_1639_v175	Listeria_ivanovii_WSLC3009_1457190	0.8886
Listeria_monocytogenes_1639_v174	Listeria_ivanovii_WSLC3009_1457190	0.8974
Listeria_monocytogenes_1639_v173	Listeria_ivanovii_WSLC3009_1457190	0.9044
Listeria_monocytogenes_1639_v172	Listeria_ivanovii_WSLC3009_1457190	0.8899
Listeria_monocytogenes_1639_v171	Listeria_ivanovii_WSLC3009_1457190	0.8825
Listeria_monocytogenes_1639_v170	Listeria_ivanovii_WSLC3009_1457190	0.8903
Listeria_monocytogenes_1639_v16	Listeria_ivanovii_WSLC3009_1457190	0.858
Listeria_monocytogenes_1639_v169	Listeria_ivanovii_WSLC3009_1457190	0.8904
Listeria_monocytogenes_1639_v168	Listeria_ivanovii_WSLC3009_1457190	0.896
Listeria_monocytogenes_1639_v167	Listeria_ivanovii_WSLC3009_1457190	0.8899
Listeria_monocytogenes_1639_v166	Listeria_ivanovii_WSLC3009_1457190	0.8964
Listeria_monocytogenes_1639_v165	Listeria_ivanovii_WSLC3009_1457190	0.8901
Listeria_monocytogenes_1639_v164	Listeria_ivanovii_WSLC3009_1457190	0.9062
Listeria_monocytogenes_1639_v163	Listeria_ivanovii_WSLC3009_1457190	0.8971
Listeria_monocytogenes_1639_v162	Listeria_ivanovii_WSLC3009_1457190	0.9062
Listeria_monocytogenes_1639_v161	Listeria_ivanovii_WSLC3009_1457190	0.9055
Listeria_monocytogenes_1639_v160	Listeria_ivanovii_WSLC3009_1457190	0.9052
Listeria_monocytogenes_1639_v15	Listeria_ivanovii_WSLC3009_1457190	0.8656
Listeria_monocytogenes_1639_v159	Listeria_ivanovii_WSLC3009_1457190	0.8981
Listeria_monocytogenes_1639_v158	Listeria_ivanovii_WSLC3009_1457190	0.9049
Listeria_monocytogenes_1639_v157	Listeria_ivanovii_WSLC3009_1457190	0.9049
Listeria_monocytogenes_1639_v156	Listeria_ivanovii_WSLC3009_1457190	0.8759
Listeria_monocytogenes_1639_v155	Listeria_ivanovii_WSLC3009_1457190	0.888
Listeria_monocytogenes_1639_v154	Listeria_ivanovii_WSLC3009_1457190	0.8885
Listeria_monocytogenes_1639_v153	Listeria_ivanovii_WSLC3009_1457190	0.8884
Listeria_monocytogenes_1639_v152	Listeria_ivanovii_WSLC3009_1457190	0.8981
Listeria_monocytogenes_1639_v151	Listeria_ivanovii_WSLC3009_1457190	0.8981

Listeria_monocytogenes_1639_v150	Listeria_ivanovii_WSLC3009_1457190	0.8876
Listeria_monocytogenes_1639_v14	Listeria_ivanovii_WSLC3009_1457190	0.8709
Listeria_monocytogenes_1639_v149	Listeria_ivanovii_WSLC3009_1457190	0.8863
Listeria_monocytogenes_1639_v148	Listeria_ivanovii_WSLC3009_1457190	0.897
Listeria_monocytogenes_1639_v147	Listeria_ivanovii_WSLC3009_1457190	0.8849
Listeria_monocytogenes_1639_v146	Listeria_ivanovii_WSLC3009_1457190	0.9007
Listeria_monocytogenes_1639_v145	Listeria_ivanovii_WSLC3009_1457190	0.8921
Listeria_monocytogenes_1639_v144	Listeria_ivanovii_WSLC3009_1457190	0.9032
Listeria_monocytogenes_1639_v143	Listeria_ivanovii_WSLC3009_1457190	0.8973
Listeria_monocytogenes_1639_v142	Listeria_ivanovii_WSLC3009_1457190	0.9032
Listeria_monocytogenes_1639_v141	Listeria_ivanovii_WSLC3009_1457190	0.8988
Listeria_monocytogenes_1639_v140	Listeria_ivanovii_WSLC3009_1457190	0.9039
Listeria_monocytogenes_1639_v13	Listeria_ivanovii_WSLC3009_1457190	0.8656
Listeria_monocytogenes_1639_v139	Listeria_ivanovii_WSLC3009_1457190	0.8968
Listeria_monocytogenes_1639_v138	Listeria_ivanovii_WSLC3009_1457190	0.9032
Listeria_monocytogenes_1639_v137	Listeria_ivanovii_WSLC3009_1457190	0.9029
Listeria_monocytogenes_1639_v136	Listeria_ivanovii_WSLC3009_1457190	0.8847
Listeria_monocytogenes_1639_v135	Listeria_ivanovii_WSLC3009_1457190	0.8726
Listeria_monocytogenes_1639_v134	Listeria_ivanovii_WSLC3009_1457190	0.8987
Listeria_monocytogenes_1639_v133	Listeria_ivanovii_WSLC3009_1457190	0.8912
Listeria_monocytogenes_1639_v132	Listeria_ivanovii_WSLC3009_1457190	0.8752
Listeria_monocytogenes_1639_v131	Listeria_ivanovii_WSLC3009_1457190	0.8869
Listeria_monocytogenes_1639_v130	Listeria_ivanovii_WSLC3009_1457190	0.8787
Listeria_monocytogenes_1639_v12	Listeria_ivanovii_WSLC3009_1457190	0.8599
Listeria_monocytogenes_1639_v129	Listeria_ivanovii_WSLC3009_1457190	0.8997
Listeria_monocytogenes_1639_v128	Listeria_ivanovii_WSLC3009_1457190	0.8954
Listeria_monocytogenes_1639_v127	Listeria_ivanovii_WSLC3009_1457190	0.8863
Listeria_monocytogenes_1639_v126	Listeria_ivanovii_WSLC3009_1457190	0.8939
Listeria_monocytogenes_1639_v125	Listeria_ivanovii_WSLC3009_1457190	0.8878
Listeria_monocytogenes_1639_v124	Listeria_ivanovii_WSLC3009_1457190	0.8875
Listeria_monocytogenes_1639_v123	Listeria_ivanovii_WSLC3009_1457190	0.8997
Listeria_monocytogenes_1639_v122	Listeria_ivanovii_WSLC3009_1457190	0.8997
Listeria_monocytogenes_1639_v121	Listeria_ivanovii_WSLC3009_1457190	0.8997
Listeria_monocytogenes_1639_v120	Listeria_ivanovii_WSLC3009_1457190	0.8997
Listeria_monocytogenes_1639_v11	Listeria_ivanovii_WSLC3009_1457190	0.8586
Listeria_monocytogenes_1639_v119	Listeria_ivanovii_WSLC3009_1457190	0.8997
Listeria_monocytogenes_1639_v118	Listeria_ivanovii_WSLC3009_1457190	0.8997
Listeria_monocytogenes_1639_v117	Listeria_ivanovii_WSLC3009_1457190	0.8855
Listeria_monocytogenes_1639_v116	Listeria_ivanovii_WSLC3009_1457190	0.8986
Listeria_monocytogenes_1639_v115	Listeria_ivanovii_WSLC3009_1457190	0.899
Listeria_monocytogenes_1639_v114	Listeria_ivanovii_WSLC3009_1457190	0.8792
Listeria_monocytogenes_1639_v113	Listeria_ivanovii_WSLC3009_1457190	0.8939
Listeria_monocytogenes_1639_v112	Listeria_ivanovii_WSLC3009_1457190	0.8944
Listeria_monocytogenes_1639_v111	Listeria_ivanovii_WSLC3009_1457190	0.8907
Listeria_monocytogenes_1639_v110	Listeria_ivanovii_WSLC3009_1457190	0.8789
Listeria_monocytogenes_1639_v10	Listeria_ivanovii_WSLC3009_1457190	0.8638
Listeria_monocytogenes_1639_v109	Listeria_ivanovii_WSLC3009_1457190	0.8788
Listeria_monocytogenes_1639_v108	Listeria_ivanovii_WSLC3009_1457190	0.8883
Listeria_monocytogenes_1639_v107	Listeria_ivanovii_WSLC3009_1457190	0.8961
Listeria_monocytogenes_1639_v106	Listeria_ivanovii_WSLC3009_1457190	0.8965
Listeria_monocytogenes_1639_v105	Listeria_ivanovii_WSLC3009_1457190	0.8965
Listeria_monocytogenes_1639_v104	Listeria_ivanovii_WSLC3009_1457190	0.8965
Listeria_monocytogenes_1639_v103	Listeria_ivanovii_WSLC3009_1457190	0.8968
Listeria_monocytogenes_1639_v102	Listeria_ivanovii_WSLC3009_1457190	0.8811
Listeria_monocytogenes_1639_v101	Listeria_ivanovii_WSLC3009_1457190	0.8702
Listeria_monocytogenes_1639_v100	Listeria_ivanovii_WSLC3009_1457190	0.8699
Listeria_monocytogenes_1639	Listeria_ivanovii_WSLC3009_1457190	0.8965
Listeria_monocytogenes_10403S_393133	Listeria_ivanovii_WSLC3009_1457190	0.8889
Listeria_monocytogenes_08-5923_637381	Listeria_ivanovii_WSLC3009_1457190	0.8708
Listeria_monocytogenes_08-5578_653938	Listeria_ivanovii_WSLC3009_1457190	0.858
Listeria_monocytogenes_07PF0776_1126011	Listeria_ivanovii_WSLC3009_1457190	0.9029
Listeria_marthii_FSL_S4-120_702457	Listeria_ivanovii_WSLC3009_1457190	0.8272
Listeria_marthii_529731_v1	Listeria_ivanovii_WSLC3009_1457190	0.8631
Listeria_marthii_529731	Listeria_ivanovii_WSLC3009_1457190	0.8727
Listeria_ivanovii_WSLC3009_1457190	Listeria_ivanovii_WSLC3009_1457190	0.0631
Listeria_welshimeri_1643	Listeria_marthii_529731	0.8676
Listeria_welshimeri_serovar_6b_str_SLCC5334_386043	Listeria_marthii_529731	0.867
Listeria_seeligeri_serovar_1_2b_str_SLCC3954_683837	Listeria_marthii_529731	0.8697
Listeria_seeligeri_FSL_S4-171_702452	Listeria_marthii_529731	0.811
Listeria_seeligeri_FSL_N1-067_702453	Listeria_marthii_529731	0.8048
Listeria_seeligeri_1640_v1	Listeria_marthii_529731	0.871
Listeria_seeligeri_1640	Listeria_marthii_529731	0.8567
Listeria_monocytogenes_WSLC1042_1457188	Listeria_marthii_529731	0.9084
Listeria_monocytogenes_WSLC1001_1457187	Listeria_marthii_529731	0.8927
Listeria_monocytogenes_SLCC7179_879090	Listeria_marthii_529731	0.8994
Listeria_monocytogenes_SLCC5850_882096	Listeria_marthii_529731	0.8968
Listeria_monocytogenes_SLCC2755_932919	Listeria_marthii_529731	0.8908
Listeria_monocytogenes_SLCC2540_879089	Listeria_marthii_529731	0.8957
Listeria_monocytogenes_SLCC2479_882020	Listeria_marthii_529731	0.8923
Listeria_monocytogenes_SLCC2378_879088	Listeria_marthii_529731	0.9098
Listeria_monocytogenes_SLCC2376_882097	Listeria_marthii_529731	0.921
Listeria_monocytogenes_SLCC2372_932920	Listeria_marthii_529731	0.8856
Listeria_monocytogenes_serotype_4b_str_LL195_1230340	Listeria_marthii_529731	0.9142
Listeria_monocytogenes_serotype_4b_str_LIS0087_1134669	Listeria_marthii_529731	0.8734
Listeria_monocytogenes_serotype_4b_str_F2365_265669	Listeria_marthii_529731	0.9142
Listeria_monocytogenes_serotype_4b_str_CLIP_80459_568819	Listeria_marthii_529731	0.9137
Listeria_monocytogenes_serotype_4b_str_81-0861_1196161	Listeria_marthii_529731	0.8998
Listeria_monocytogenes_serotype_4b_str_81-0592_1196160	Listeria_marthii_529731	0.9008
Listeria_monocytogenes_serotype_4b_str_81-0558_1196159	Listeria_marthii_529731	0.9008
Listeria_monocytogenes_serotype_4b_str_10-0809_1196162	Listeria_marthii_529731	0.9008
Listeria_monocytogenes_serotype_4b_str_02-6680_1196175	Listeria_marthii_529731	0.9108

Listeria_monocytogenes_serotype_4b_str_02-6679_1196174	Listeria_marthii_529731	0.9108
Listeria_monocytogenes_serotype_4b_str_02-1792_1196173	Listeria_marthii_529731	0.9008
Listeria_monocytogenes_serotype_4b_str_02-1289_1196172	Listeria_marthii_529731	0.9008
Listeria_monocytogenes_serotype_4b_str_02-1103_1196171	Listeria_marthii_529731	0.9008
Listeria_monocytogenes_serotype_3c_str_10-5027_1196195	Listeria_marthii_529731	0.8911
Listeria_monocytogenes_serotype_1_2c_str_10-5026_1196194	Listeria_marthii_529731	0.8923
Listeria_monocytogenes_serotype_1_2c_str_10-5025_1196193	Listeria_marthii_529731	0.8923
Listeria_monocytogenes_serotype_1_2b_str_10-0811_1196164	Listeria_marthii_529731	0.8875
Listeria_monocytogenes_serotype_1_2b_str_10-0810_1196163	Listeria_marthii_529731	0.8875
Listeria_monocytogenes_serotype_1_2a_str_99-6370_1196179	Listeria_marthii_529731	0.8757
Listeria_monocytogenes_serotype_1_2a_str_98-2035_1196178	Listeria_marthii_529731	0.8757
Listeria_monocytogenes_serotype_1_2a_str_95-0093_1196177	Listeria_marthii_529731	0.881
Listeria_monocytogenes_serotype_1_2a_str_88-0478_1196176	Listeria_marthii_529731	0.8801
Listeria_monocytogenes_serotype_1_2a_str_10-5024_1196192	Listeria_marthii_529731	0.8813
Listeria_monocytogenes_serotype_1_2a_str_10-4758_1196168	Listeria_marthii_529731	0.8981
Listeria_monocytogenes_serotype_1_2a_str_10-4754_1196167	Listeria_marthii_529731	0.8981
Listeria_monocytogenes_serotype_1_2a_str_10-1321_1196191	Listeria_marthii_529731	0.8813
Listeria_monocytogenes_serotype_1_2a_str_10-1047_1196190	Listeria_marthii_529731	0.8816
Listeria_monocytogenes_serotype_1_2a_str_10-1046_1196189	Listeria_marthii_529731	0.8816
Listeria_monocytogenes_serotype_1_2a_str_10-0934_1196170	Listeria_marthii_529731	0.9095
Listeria_monocytogenes_serotype_1_2a_str_10-0933_1196169	Listeria_marthii_529731	0.9095
Listeria_monocytogenes_serotype_1_2a_str_10-0815_1196188	Listeria_marthii_529731	0.8771
Listeria_monocytogenes_serotype_1_2a_str_10-0814_1196187	Listeria_marthii_529731	0.881
Listeria_monocytogenes_serotype_1_2a_str_10-0813_1196166	Listeria_marthii_529731	0.8907
Listeria_monocytogenes_serotype_1_2a_str_10-0812_1196165	Listeria_marthii_529731	0.8907
Listeria_monocytogenes_serotype_1_2a_str_08-7669_1196186	Listeria_marthii_529731	0.8904
Listeria_monocytogenes_serotype_1_2a_str_08-7374_1196185	Listeria_marthii_529731	0.8813
Listeria_monocytogenes_serotype_1_2a_str_08-6997_1196184	Listeria_marthii_529731	0.8771
Listeria_monocytogenes_serotype_1_2a_str_08-6569_1196183	Listeria_marthii_529731	0.8771
Listeria_monocytogenes_serotype_1_2a_str_08-6056_1196182	Listeria_marthii_529731	0.8771
Listeria_monocytogenes_serotype_1_2a_str_04-5457_1196181	Listeria_marthii_529731	0.8813
Listeria_monocytogenes_serotype_1_2a_str_02-5993_1196180	Listeria_marthii_529731	0.881
Listeria_monocytogenes_R479a_1437838	Listeria_marthii_529731	0.8814
Listeria_monocytogenes_N53-1_1234142	Listeria_marthii_529731	0.8595
Listeria_monocytogenes_M7_1030009	Listeria_marthii_529731	0.8924
Listeria_monocytogenes_LO28_393132	Listeria_marthii_529731	0.891
Listeria_monocytogenes_L99_563174	Listeria_marthii_529731	0.8931
Listeria_monocytogenes_L312_882094	Listeria_marthii_529731	0.9137
Listeria_monocytogenes_J1816_930781	Listeria_marthii_529731	0.8921
Listeria_monocytogenes_J1-220_930782	Listeria_marthii_529731	0.8896
Listeria_monocytogenes_J0161_393130	Listeria_marthii_529731	0.8909
Listeria_monocytogenes_HCC23_552536	Listeria_marthii_529731	0.8928
Listeria_monocytogenes_FSL_R2-561_393126	Listeria_marthii_529731	0.8909
Listeria_monocytogenes_FSL_J1-208_393119	Listeria_marthii_529731	0.9205
Listeria_monocytogenes_FSL_J1-175_393118	Listeria_marthii_529731	0.901
Listeria_monocytogenes_Finland_1998_393127	Listeria_marthii_529731	0.908
Listeria_monocytogenes_EGD-e_169963_v1	Listeria_marthii_529731	0.9005
Listeria_monocytogenes_EGD-e_169963	Listeria_marthii_529731	0.9005
Listeria_monocytogenes_EGD_1334565	Listeria_marthii_529731	0.8975
Listeria_monocytogenes_CFSAN002349_1299895	Listeria_marthii_529731	0.9086
Listeria_monocytogenes_ATCC_19117_882095_v1	Listeria_marthii_529731	0.9076
Listeria_monocytogenes_ATCC_19117_882095	Listeria_marthii_529731	0.9076
Listeria_monocytogenes_6179_1288295	Listeria_marthii_529731	0.8786
Listeria_monocytogenes_1639_v9	Listeria_marthii_529731	0.8738
Listeria_monocytogenes_1639_v99	Listeria_marthii_529731	0.8937
Listeria_monocytogenes_1639_v98	Listeria_marthii_529731	0.8881
Listeria_monocytogenes_1639_v97	Listeria_marthii_529731	0.9001
Listeria_monocytogenes_1639_v96	Listeria_marthii_529731	0.8934
Listeria_monocytogenes_1639_v95	Listeria_marthii_529731	0.8934
Listeria_monocytogenes_1639_v94	Listeria_marthii_529731	0.8934
Listeria_monocytogenes_1639_v93	Listeria_marthii_529731	0.8862
Listeria_monocytogenes_1639_v92	Listeria_marthii_529731	0.8933
Listeria_monocytogenes_1639_v91	Listeria_marthii_529731	0.8966
Listeria_monocytogenes_1639_v90	Listeria_marthii_529731	0.8881
Listeria_monocytogenes_1639_v8	Listeria_marthii_529731	0.8709
Listeria_monocytogenes_1639_v89	Listeria_marthii_529731	0.902
Listeria_monocytogenes_1639_v88	Listeria_marthii_529731	0.8924
Listeria_monocytogenes_1639_v87	Listeria_marthii_529731	0.886
Listeria_monocytogenes_1639_v86	Listeria_marthii_529731	0.8853
Listeria_monocytogenes_1639_v85	Listeria_marthii_529731	0.8999
Listeria_monocytogenes_1639_v84	Listeria_marthii_529731	0.8879
Listeria_monocytogenes_1639_v83	Listeria_marthii_529731	0.898
Listeria_monocytogenes_1639_v82	Listeria_marthii_529731	0.8977
Listeria_monocytogenes_1639_v81	Listeria_marthii_529731	0.8905
Listeria_monocytogenes_1639_v80	Listeria_marthii_529731	0.8929
Listeria_monocytogenes_1639_v7	Listeria_marthii_529731	0.8709
Listeria_monocytogenes_1639_v79	Listeria_marthii_529731	0.8896
Listeria_monocytogenes_1639_v78	Listeria_marthii_529731	0.8902
Listeria_monocytogenes_1639_v77	Listeria_marthii_529731	0.8909
Listeria_monocytogenes_1639_v76	Listeria_marthii_529731	0.8838
Listeria_monocytogenes_1639_v75	Listeria_marthii_529731	0.8931
Listeria_monocytogenes_1639_v74	Listeria_marthii_529731	0.8887
Listeria_monocytogenes_1639_v73	Listeria_marthii_529731	0.8863
Listeria_monocytogenes_1639_v72	Listeria_marthii_529731	0.8975
Listeria_monocytogenes_1639_v71	Listeria_marthii_529731	0.8764
Listeria_monocytogenes_1639_v70	Listeria_marthii_529731	0.8893
Listeria_monocytogenes_1639_v6	Listeria_marthii_529731	0.8702
Listeria_monocytogenes_1639_v69	Listeria_marthii_529731	0.9012
Listeria_monocytogenes_1639_v68	Listeria_marthii_529731	0.8935
Listeria_monocytogenes_1639_v67	Listeria_marthii_529731	0.8931
Listeria_monocytogenes_1639_v66	Listeria_marthii_529731	0.8955

Listeria_monocytogenes_1639_v65	Listeria_marthii_529731	0.8821
Listeria_monocytogenes_1639_v64	Listeria_marthii_529731	0.8891
Listeria_monocytogenes_1639_v63	Listeria_marthii_529731	0.8892
Listeria_monocytogenes_1639_v62	Listeria_marthii_529731	0.8868
Listeria_monocytogenes_1639_v61	Listeria_marthii_529731	0.8824
Listeria_monocytogenes_1639_v60	Listeria_marthii_529731	0.8916
Listeria_monocytogenes_1639_v5	Listeria_marthii_529731	0.859
Listeria_monocytogenes_1639_v59	Listeria_marthii_529731	0.8979
Listeria_monocytogenes_1639_v58	Listeria_marthii_529731	0.9006
Listeria_monocytogenes_1639_v57	Listeria_marthii_529731	0.8889
Listeria_monocytogenes_1639_v56	Listeria_marthii_529731	0.8976
Listeria_monocytogenes_1639_v55	Listeria_marthii_529731	0.886
Listeria_monocytogenes_1639_v54	Listeria_marthii_529731	0.8908
Listeria_monocytogenes_1639_v53	Listeria_marthii_529731	0.8916
Listeria_monocytogenes_1639_v52	Listeria_marthii_529731	0.8945
Listeria_monocytogenes_1639_v51	Listeria_marthii_529731	0.8936
Listeria_monocytogenes_1639_v50	Listeria_marthii_529731	0.8802
Listeria_monocytogenes_1639_v4	Listeria_marthii_529731	0.8687
Listeria_monocytogenes_1639_v49	Listeria_marthii_529731	0.8997
Listeria_monocytogenes_1639_v48	Listeria_marthii_529731	0.8869
Listeria_monocytogenes_1639_v47	Listeria_marthii_529731	0.881
Listeria_monocytogenes_1639_v46	Listeria_marthii_529731	0.892
Listeria_monocytogenes_1639_v45	Listeria_marthii_529731	0.8729
Listeria_monocytogenes_1639_v44	Listeria_marthii_529731	0.8813
Listeria_monocytogenes_1639_v43	Listeria_marthii_529731	0.8813
Listeria_monocytogenes_1639_v42	Listeria_marthii_529731	0.8772
Listeria_monocytogenes_1639_v41	Listeria_marthii_529731	0.8693
Listeria_monocytogenes_1639_v40	Listeria_marthii_529731	0.8913
Listeria_monocytogenes_1639_v3	Listeria_marthii_529731	0.8691
Listeria_monocytogenes_1639_v39	Listeria_marthii_529731	0.891
Listeria_monocytogenes_1639_v38	Listeria_marthii_529731	0.8901
Listeria_monocytogenes_1639_v37	Listeria_marthii_529731	0.8847
Listeria_monocytogenes_1639_v36	Listeria_marthii_529731	0.88
Listeria_monocytogenes_1639_v35	Listeria_marthii_529731	0.8915
Listeria_monocytogenes_1639_v34	Listeria_marthii_529731	0.8853
Listeria_monocytogenes_1639_v33	Listeria_marthii_529731	0.8875
Listeria_monocytogenes_1639_v32	Listeria_marthii_529731	0.8826
Listeria_monocytogenes_1639_v31	Listeria_marthii_529731	0.8857
Listeria_monocytogenes_1639_v30	Listeria_marthii_529731	0.8907
Listeria_monocytogenes_1639_v2	Listeria_marthii_529731	0.8557
Listeria_monocytogenes_1639_v29	Listeria_marthii_529731	0.8794
Listeria_monocytogenes_1639_v28	Listeria_marthii_529731	0.8826
Listeria_monocytogenes_1639_v27	Listeria_marthii_529731	0.8741
Listeria_monocytogenes_1639_v26	Listeria_marthii_529731	0.8738
Listeria_monocytogenes_1639_v25	Listeria_marthii_529731	0.8735
Listeria_monocytogenes_1639_v24	Listeria_marthii_529731	0.8892
Listeria_monocytogenes_1639_v23	Listeria_marthii_529731	0.8892
Listeria_monocytogenes_1639_v22	Listeria_marthii_529731	0.8899
Listeria_monocytogenes_1639_v21	Listeria_marthii_529731	0.8888
Listeria_monocytogenes_1639_v20	Listeria_marthii_529731	0.8799
Listeria_monocytogenes_1639_v1	Listeria_marthii_529731	0.8734
Listeria_monocytogenes_1639_v19	Listeria_marthii_529731	0.8795
Listeria_monocytogenes_1639_v198	Listeria_marthii_529731	0.862
Listeria_monocytogenes_1639_v197	Listeria_marthii_529731	0.8877
Listeria_monocytogenes_1639_v196	Listeria_marthii_529731	0.8802
Listeria_monocytogenes_1639_v195	Listeria_marthii_529731	0.9229
Listeria_monocytogenes_1639_v194	Listeria_marthii_529731	0.9218
Listeria_monocytogenes_1639_v193	Listeria_marthii_529731	0.9214
Listeria_monocytogenes_1639_v192	Listeria_marthii_529731	0.9119
Listeria_monocytogenes_1639_v191	Listeria_marthii_529731	0.9085
Listeria_monocytogenes_1639_v190	Listeria_marthii_529731	0.9086
Listeria_monocytogenes_1639_v18	Listeria_marthii_529731	0.8833
Listeria_monocytogenes_1639_v189	Listeria_marthii_529731	0.9079
Listeria_monocytogenes_1639_v188	Listeria_marthii_529731	0.9104
Listeria_monocytogenes_1639_v187	Listeria_marthii_529731	0.9082
Listeria_monocytogenes_1639_v186	Listeria_marthii_529731	0.9089
Listeria_monocytogenes_1639_v185	Listeria_marthii_529731	0.9086
Listeria_monocytogenes_1639_v184	Listeria_marthii_529731	0.9082
Listeria_monocytogenes_1639_v183	Listeria_marthii_529731	0.909
Listeria_monocytogenes_1639_v182	Listeria_marthii_529731	0.9108
Listeria_monocytogenes_1639_v181	Listeria_marthii_529731	0.9184
Listeria_monocytogenes_1639_v180	Listeria_marthii_529731	0.9184
Listeria_monocytogenes_1639_v17	Listeria_marthii_529731	0.8833
Listeria_monocytogenes_1639_v179	Listeria_marthii_529731	0.9022
Listeria_monocytogenes_1639_v178	Listeria_marthii_529731	0.9102
Listeria_monocytogenes_1639_v177	Listeria_marthii_529731	0.9101
Listeria_monocytogenes_1639_v176	Listeria_marthii_529731	0.9149
Listeria_monocytogenes_1639_v175	Listeria_marthii_529731	0.9002
Listeria_monocytogenes_1639_v174	Listeria_marthii_529731	0.9068
Listeria_monocytogenes_1639_v173	Listeria_marthii_529731	0.9083
Listeria_monocytogenes_1639_v172	Listeria_marthii_529731	0.917
Listeria_monocytogenes_1639_v171	Listeria_marthii_529731	0.8955
Listeria_monocytogenes_1639_v170	Listeria_marthii_529731	0.9023
Listeria_monocytogenes_1639_v16	Listeria_marthii_529731	0.8694
Listeria_monocytogenes_1639_v169	Listeria_marthii_529731	0.8986
Listeria_monocytogenes_1639_v168	Listeria_marthii_529731	0.905
Listeria_monocytogenes_1639_v167	Listeria_marthii_529731	0.9046
Listeria_monocytogenes_1639_v166	Listeria_marthii_529731	0.9053
Listeria_monocytogenes_1639_v165	Listeria_marthii_529731	0.8982
Listeria_monocytogenes_1639_v164	Listeria_marthii_529731	0.9152
Listeria_monocytogenes_1639_v163	Listeria_marthii_529731	0.9053

Listeria_monocytogenes_1639_v162	Listeria_marthii_529731	0.9152
Listeria_monocytogenes_1639_v161	Listeria_marthii_529731	0.9141
Listeria_monocytogenes_1639_v160	Listeria_marthii_529731	0.9145
Listeria_monocytogenes_1639_v15	Listeria_marthii_529731	0.8745
Listeria_monocytogenes_1639_v159	Listeria_marthii_529731	0.9097
Listeria_monocytogenes_1639_v158	Listeria_marthii_529731	0.9142
Listeria_monocytogenes_1639_v157	Listeria_marthii_529731	0.9142
Listeria_monocytogenes_1639_v156	Listeria_marthii_529731	0.8899
Listeria_monocytogenes_1639_v155	Listeria_marthii_529731	0.8996
Listeria_monocytogenes_1639_v154	Listeria_marthii_529731	0.8973
Listeria_monocytogenes_1639_v153	Listeria_marthii_529731	0.9011
Listeria_monocytogenes_1639_v152	Listeria_marthii_529731	0.9101
Listeria_monocytogenes_1639_v151	Listeria_marthii_529731	0.9101
Listeria_monocytogenes_1639_v150	Listeria_marthii_529731	0.8988
Listeria_monocytogenes_1639_v14	Listeria_marthii_529731	0.881
Listeria_monocytogenes_1639_v149	Listeria_marthii_529731	0.8975
Listeria_monocytogenes_1639_v148	Listeria_marthii_529731	0.9086
Listeria_monocytogenes_1639_v147	Listeria_marthii_529731	0.8994
Listeria_monocytogenes_1639_v146	Listeria_marthii_529731	0.9089
Listeria_monocytogenes_1639_v145	Listeria_marthii_529731	0.9041
Listeria_monocytogenes_1639_v144	Listeria_marthii_529731	0.9137
Listeria_monocytogenes_1639_v143	Listeria_marthii_529731	0.909
Listeria_monocytogenes_1639_v142	Listeria_marthii_529731	0.9137
Listeria_monocytogenes_1639_v141	Listeria_marthii_529731	0.9035
Listeria_monocytogenes_1639_v140	Listeria_marthii_529731	0.9137
Listeria_monocytogenes_1639_v13	Listeria_marthii_529731	0.8748
Listeria_monocytogenes_1639_v139	Listeria_marthii_529731	0.9077
Listeria_monocytogenes_1639_v138	Listeria_marthii_529731	0.9138
Listeria_monocytogenes_1639_v137	Listeria_marthii_529731	0.913
Listeria_monocytogenes_1639_v136	Listeria_marthii_529731	0.8978
Listeria_monocytogenes_1639_v135	Listeria_marthii_529731	0.9047
Listeria_monocytogenes_1639_v134	Listeria_marthii_529731	0.9091
Listeria_monocytogenes_1639_v133	Listeria_marthii_529731	0.9024
Listeria_monocytogenes_1639_v132	Listeria_marthii_529731	0.9097
Listeria_monocytogenes_1639_v131	Listeria_marthii_529731	0.8987
Listeria_monocytogenes_1639_v130	Listeria_marthii_529731	0.8919
Listeria_monocytogenes_1639_v12	Listeria_marthii_529731	0.8732
Listeria_monocytogenes_1639_v129	Listeria_marthii_529731	0.9095
Listeria_monocytogenes_1639_v128	Listeria_marthii_529731	0.9009
Listeria_monocytogenes_1639_v127	Listeria_marthii_529731	0.9009
Listeria_monocytogenes_1639_v126	Listeria_marthii_529731	0.9001
Listeria_monocytogenes_1639_v125	Listeria_marthii_529731	0.8978
Listeria_monocytogenes_1639_v124	Listeria_marthii_529731	0.9151
Listeria_monocytogenes_1639_v123	Listeria_marthii_529731	0.9086
Listeria_monocytogenes_1639_v122	Listeria_marthii_529731	0.909
Listeria_monocytogenes_1639_v121	Listeria_marthii_529731	0.909
Listeria_monocytogenes_1639_v120	Listeria_marthii_529731	0.909
Listeria_monocytogenes_1639_v11	Listeria_marthii_529731	0.8759
Listeria_monocytogenes_1639_v119	Listeria_marthii_529731	0.909
Listeria_monocytogenes_1639_v118	Listeria_marthii_529731	0.909
Listeria_monocytogenes_1639_v117	Listeria_marthii_529731	0.8966
Listeria_monocytogenes_1639_v116	Listeria_marthii_529731	0.9091
Listeria_monocytogenes_1639_v115	Listeria_marthii_529731	0.9094
Listeria_monocytogenes_1639_v114	Listeria_marthii_529731	0.891
Listeria_monocytogenes_1639_v113	Listeria_marthii_529731	0.9024
Listeria_monocytogenes_1639_v112	Listeria_marthii_529731	0.9041
Listeria_monocytogenes_1639_v111	Listeria_marthii_529731	0.8938
Listeria_monocytogenes_1639_v110	Listeria_marthii_529731	0.8903
Listeria_monocytogenes_1639_v10	Listeria_marthii_529731	0.8723
Listeria_monocytogenes_1639_v109	Listeria_marthii_529731	0.8907
Listeria_monocytogenes_1639_v108	Listeria_marthii_529731	0.8997
Listeria_monocytogenes_1639_v107	Listeria_marthii_529731	0.9065
Listeria_monocytogenes_1639_v106	Listeria_marthii_529731	0.9065
Listeria_monocytogenes_1639_v105	Listeria_marthii_529731	0.9069
Listeria_monocytogenes_1639_v104	Listeria_marthii_529731	0.9069
Listeria_monocytogenes_1639_v103	Listeria_marthii_529731	0.9069
Listeria_monocytogenes_1639_v102	Listeria_marthii_529731	0.891
Listeria_monocytogenes_1639_v101	Listeria_marthii_529731	0.8814
Listeria_monocytogenes_1639_v100	Listeria_marthii_529731	0.8801
Listeria_monocytogenes_1639	Listeria_marthii_529731	0.9082
Listeria_monocytogenes_10403S_393133	Listeria_marthii_529731	0.9009
Listeria_monocytogenes_08-5923_637381	Listeria_marthii_529731	0.8813
Listeria_monocytogenes_08-5578_653938	Listeria_marthii_529731	0.8691
Listeria_monocytogenes_07PF0776_1126011	Listeria_marthii_529731	0.9119
Listeria_marthii_FSL_S4-120_702457	Listeria_marthii_529731	0.8813
Listeria_marthii_529731_v1	Listeria_marthii_529731	0.9332
Listeria_marthii_529731	Listeria_marthii_529731	0.0561
Listeria_welshimeri_1643	Listeria_marthii_529731_v1	0.8635
Listeria_welshimeri_serovar_6b_str_SLCC5334_386043	Listeria_marthii_529731_v1	0.8628
Listeria_seeligeri_serovar_1_2b_str_SLCC3954_683837	Listeria_marthii_529731_v1	0.8658
Listeria_seeligeri_FSL_S4-171_702452	Listeria_marthii_529731_v1	0.8162
Listeria_seeligeri_FSL_N1-067_702453	Listeria_marthii_529731_v1	0.8095
Listeria_seeligeri_1640_v1	Listeria_marthii_529731_v1	0.8709
Listeria_seeligeri_1640	Listeria_marthii_529731_v1	0.8708
Listeria_monocytogenes_WSLC1042_1457188	Listeria_marthii_529731_v1	0.907
Listeria_monocytogenes_WSLC1001_1457187	Listeria_marthii_529731_v1	0.8946
Listeria_monocytogenes_SLCC7179_879090	Listeria_marthii_529731_v1	0.9096
Listeria_monocytogenes_SLCC5850_882096	Listeria_marthii_529731_v1	0.8979
Listeria_monocytogenes_SLCC2755_932919	Listeria_marthii_529731_v1	0.8897
Listeria_monocytogenes_SLCC2540_879089	Listeria_marthii_529731_v1	0.893
Listeria_monocytogenes_SLCC2479_882020	Listeria_marthii_529731_v1	0.8927

Listeria_monocytogenes_SLCC2378_879088	Listeria_marthii_529731_v1	0.9084
Listeria_monocytogenes_SLCC2376_882097	Listeria_marthii_529731_v1	0.916
Listeria_monocytogenes_SLCC2372_932920	Listeria_marthii_529731_v1	0.8854
Listeria_monocytogenes_serotype_4b_str_LL195_1230340	Listeria_marthii_529731_v1	0.9131
Listeria_monocytogenes_serotype_4b_str_LIS0087_1134669	Listeria_marthii_529731_v1	0.8825
Listeria_monocytogenes_serotype_4b_str_F2365_265669	Listeria_marthii_529731_v1	0.9131
Listeria_monocytogenes_serotype_4b_str_CLIP_80459_568819	Listeria_marthii_529731_v1	0.9138
Listeria_monocytogenes_serotype_4b_str_81-0861_1196161	Listeria_marthii_529731_v1	0.9091
Listeria_monocytogenes_serotype_4b_str_81-0592_1196160	Listeria_marthii_529731_v1	0.9101
Listeria_monocytogenes_serotype_4b_str_81-0558_1196159	Listeria_marthii_529731_v1	0.9108
Listeria_monocytogenes_serotype_4b_str_10-0809_1196162	Listeria_marthii_529731_v1	0.9101
Listeria_monocytogenes_serotype_4b_str_02-6680_1196175	Listeria_marthii_529731_v1	0.911
Listeria_monocytogenes_serotype_4b_str_02-6679_1196174	Listeria_marthii_529731_v1	0.911
Listeria_monocytogenes_serotype_4b_str_02-1792_1196173	Listeria_marthii_529731_v1	0.9116
Listeria_monocytogenes_serotype_4b_str_02-1289_1196172	Listeria_marthii_529731_v1	0.9116
Listeria_monocytogenes_serotype_4b_str_02-1103_1196171	Listeria_marthii_529731_v1	0.9116
Listeria_monocytogenes_serotype_3c_str_10-5027_1196195	Listeria_marthii_529731_v1	0.8908
Listeria_monocytogenes_serotype_1_2c_str_10-5026_1196194	Listeria_marthii_529731_v1	0.8915
Listeria_monocytogenes_serotype_1_2c_str_10-5025_1196193	Listeria_marthii_529731_v1	0.8915
Listeria_monocytogenes_serotype_1_2b_str_10-0811_1196164	Listeria_marthii_529731_v1	0.8958
Listeria_monocytogenes_serotype_1_2b_str_10-0810_1196163	Listeria_marthii_529731_v1	0.8958
Listeria_monocytogenes_serotype_1_2a_str_99-6370_1196179	Listeria_marthii_529731_v1	0.8786
Listeria_monocytogenes_serotype_1_2a_str_98-2035_1196178	Listeria_marthii_529731_v1	0.8786
Listeria_monocytogenes_serotype_1_2a_str_95-0093_1196177	Listeria_marthii_529731_v1	0.8838
Listeria_monocytogenes_serotype_1_2a_str_88-0478_1196176	Listeria_marthii_529731_v1	0.8837
Listeria_monocytogenes_serotype_1_2a_str_10-5024_1196192	Listeria_marthii_529731_v1	0.8841
Listeria_monocytogenes_serotype_1_2a_str_10-4758_1196168	Listeria_marthii_529731_v1	0.8958
Listeria_monocytogenes_serotype_1_2a_str_10-4754_1196167	Listeria_marthii_529731_v1	0.8958
Listeria_monocytogenes_serotype_1_2a_str_10-1321_1196191	Listeria_marthii_529731_v1	0.8841
Listeria_monocytogenes_serotype_1_2a_str_10-1047_1196190	Listeria_marthii_529731_v1	0.8844
Listeria_monocytogenes_serotype_1_2a_str_10-1046_1196189	Listeria_marthii_529731_v1	0.8844
Listeria_monocytogenes_serotype_1_2a_str_10-0934_1196170	Listeria_marthii_529731_v1	0.9074
Listeria_monocytogenes_serotype_1_2a_str_10-0933_1196169	Listeria_marthii_529731_v1	0.9074
Listeria_monocytogenes_serotype_1_2a_str_10-0815_1196188	Listeria_marthii_529731_v1	0.8799
Listeria_monocytogenes_serotype_1_2a_str_10-0814_1196187	Listeria_marthii_529731_v1	0.8841
Listeria_monocytogenes_serotype_1_2a_str_10-0813_1196166	Listeria_marthii_529731_v1	0.8994
Listeria_monocytogenes_serotype_1_2a_str_10-0812_1196165	Listeria_marthii_529731_v1	0.8994
Listeria_monocytogenes_serotype_1_2a_str_08-7669_1196186	Listeria_marthii_529731_v1	0.8927
Listeria_monocytogenes_serotype_1_2a_str_08-7374_1196185	Listeria_marthii_529731_v1	0.8841
Listeria_monocytogenes_serotype_1_2a_str_08-6997_1196184	Listeria_marthii_529731_v1	0.8799
Listeria_monocytogenes_serotype_1_2a_str_08-6569_1196183	Listeria_marthii_529731_v1	0.8799
Listeria_monocytogenes_serotype_1_2a_str_08-6056_1196182	Listeria_marthii_529731_v1	0.8799
Listeria_monocytogenes_serotype_1_2a_str_04-5457_1196181	Listeria_marthii_529731_v1	0.8841
Listeria_monocytogenes_serotype_1_2a_str_02-5993_1196180	Listeria_marthii_529731_v1	0.8838
Listeria_monocytogenes_R479a_1437838	Listeria_marthii_529731_v1	0.8813
Listeria_monocytogenes_N53-1_1234142	Listeria_marthii_529731_v1	0.86
Listeria_monocytogenes_M7_1030009	Listeria_marthii_529731_v1	0.8999
Listeria_monocytogenes_LO28_393132	Listeria_marthii_529731_v1	0.8966
Listeria_monocytogenes_L99_563174	Listeria_marthii_529731_v1	0.9006
Listeria_monocytogenes_L312_882094	Listeria_marthii_529731_v1	0.9138
Listeria_monocytogenes_J1816_930781	Listeria_marthii_529731_v1	0.8955
Listeria_monocytogenes_J1-220_930782	Listeria_marthii_529731_v1	0.9019
Listeria_monocytogenes_J0161_393130	Listeria_marthii_529731_v1	0.8996
Listeria_monocytogenes_HCC23_552536	Listeria_marthii_529731_v1	0.9002
Listeria_monocytogenes_FSL_R2-561_393126	Listeria_marthii_529731_v1	0.8906
Listeria_monocytogenes_FSL_J1-208_393119	Listeria_marthii_529731_v1	0.91
Listeria_monocytogenes_FSL_J1-175_393118	Listeria_marthii_529731_v1	0.896
Listeria_monocytogenes_Finland_1998_393127	Listeria_marthii_529731_v1	0.9032
Listeria_monocytogenes_EGD-e_169963_v1	Listeria_marthii_529731_v1	0.9069
Listeria_monocytogenes_EGD-e_169963	Listeria_marthii_529731_v1	0.9072
Listeria_monocytogenes_EGD_1334565	Listeria_marthii_529731_v1	0.8986
Listeria_monocytogenes_CFSAN002349_1299895	Listeria_marthii_529731_v1	0.9084
Listeria_monocytogenes_ATCC_19117_882095_v1	Listeria_marthii_529731_v1	0.9066
Listeria_monocytogenes_ATCC_19117_882095	Listeria_marthii_529731_v1	0.9066
Listeria_monocytogenes_6179_1288295	Listeria_marthii_529731_v1	0.8837
Listeria_monocytogenes_1639_v9	Listeria_marthii_529731_v1	0.8811
Listeria_monocytogenes_1639_v99	Listeria_marthii_529731_v1	0.8967
Listeria_monocytogenes_1639_v98	Listeria_marthii_529731_v1	0.9021
Listeria_monocytogenes_1639_v97	Listeria_marthii_529731_v1	0.9038
Listeria_monocytogenes_1639_v96	Listeria_marthii_529731_v1	0.896
Listeria_monocytogenes_1639_v95	Listeria_marthii_529731_v1	0.8961
Listeria_monocytogenes_1639_v94	Listeria_marthii_529731_v1	0.8964
Listeria_monocytogenes_1639_v93	Listeria_marthii_529731_v1	0.8811
Listeria_monocytogenes_1639_v92	Listeria_marthii_529731_v1	0.8967
Listeria_monocytogenes_1639_v91	Listeria_marthii_529731_v1	0.9107
Listeria_monocytogenes_1639_v90	Listeria_marthii_529731_v1	0.9021
Listeria_monocytogenes_1639_v8	Listeria_marthii_529731_v1	0.8705
Listeria_monocytogenes_1639_v89	Listeria_marthii_529731_v1	0.9117
Listeria_monocytogenes_1639_v88	Listeria_marthii_529731_v1	0.8951
Listeria_monocytogenes_1639_v87	Listeria_marthii_529731_v1	0.8969
Listeria_monocytogenes_1639_v86	Listeria_marthii_529731_v1	0.8962
Listeria_monocytogenes_1639_v85	Listeria_marthii_529731_v1	0.9107
Listeria_monocytogenes_1639_v84	Listeria_marthii_529731_v1	0.8929
Listeria_monocytogenes_1639_v83	Listeria_marthii_529731_v1	0.9074
Listeria_monocytogenes_1639_v82	Listeria_marthii_529731_v1	0.907
Listeria_monocytogenes_1639_v81	Listeria_marthii_529731_v1	0.8913
Listeria_monocytogenes_1639_v80	Listeria_marthii_529731_v1	0.8997
Listeria_monocytogenes_1639_v7	Listeria_marthii_529731_v1	0.8705
Listeria_monocytogenes_1639_v79	Listeria_marthii_529731_v1	0.8893
Listeria_monocytogenes_1639_v78	Listeria_marthii_529731_v1	0.891
Listeria_monocytogenes_1639_v77	Listeria_marthii_529731_v1	0.8928

Listeria_monocytogenes_1639_v76	Listeria_marthii_529731_v1	0.8847
Listeria_monocytogenes_1639_v75	Listeria_marthii_529731_v1	0.9006
Listeria_monocytogenes_1639_v74	Listeria_marthii_529731_v1	0.8936
Listeria_monocytogenes_1639_v73	Listeria_marthii_529731_v1	0.8916
Listeria_monocytogenes_1639_v72	Listeria_marthii_529731_v1	0.9076
Listeria_monocytogenes_1639_v71	Listeria_marthii_529731_v1	0.8755
Listeria_monocytogenes_1639_v70	Listeria_marthii_529731_v1	0.8909
Listeria_monocytogenes_1639_v6	Listeria_marthii_529731_v1	0.8698
Listeria_monocytogenes_1639_v69	Listeria_marthii_529731_v1	0.9105
Listeria_monocytogenes_1639_v68	Listeria_marthii_529731_v1	0.901
Listeria_monocytogenes_1639_v67	Listeria_marthii_529731_v1	0.8935
Listeria_monocytogenes_1639_v66	Listeria_marthii_529731_v1	0.8925
Listeria_monocytogenes_1639_v65	Listeria_marthii_529731_v1	0.8916
Listeria_monocytogenes_1639_v64	Listeria_marthii_529731_v1	0.8869
Listeria_monocytogenes_1639_v63	Listeria_marthii_529731_v1	0.89
Listeria_monocytogenes_1639_v62	Listeria_marthii_529731_v1	0.8876
Listeria_monocytogenes_1639_v61	Listeria_marthii_529731_v1	0.8886
Listeria_monocytogenes_1639_v60	Listeria_marthii_529731_v1	0.8942
Listeria_monocytogenes_1639_v5	Listeria_marthii_529731_v1	0.8627
Listeria_monocytogenes_1639_v59	Listeria_marthii_529731_v1	0.9013
Listeria_monocytogenes_1639_v58	Listeria_marthii_529731_v1	0.9129
Listeria_monocytogenes_1639_v57	Listeria_marthii_529731_v1	0.8942
Listeria_monocytogenes_1639_v56	Listeria_marthii_529731_v1	0.9039
Listeria_monocytogenes_1639_v55	Listeria_marthii_529731_v1	0.8964
Listeria_monocytogenes_1639_v54	Listeria_marthii_529731_v1	0.895
Listeria_monocytogenes_1639_v53	Listeria_marthii_529731_v1	0.8886
Listeria_monocytogenes_1639_v52	Listeria_marthii_529731_v1	0.9061
Listeria_monocytogenes_1639_v51	Listeria_marthii_529731_v1	0.9041
Listeria_monocytogenes_1639_v50	Listeria_marthii_529731_v1	0.8834
Listeria_monocytogenes_1639_v4	Listeria_marthii_529731_v1	0.8745
Listeria_monocytogenes_1639_v49	Listeria_marthii_529731_v1	0.9012
Listeria_monocytogenes_1639_v48	Listeria_marthii_529731_v1	0.8874
Listeria_monocytogenes_1639_v47	Listeria_marthii_529731_v1	0.8841
Listeria_monocytogenes_1639_v46	Listeria_marthii_529731_v1	0.8932
Listeria_monocytogenes_1639_v45	Listeria_marthii_529731_v1	0.875
Listeria_monocytogenes_1639_v44	Listeria_marthii_529731_v1	0.8841
Listeria_monocytogenes_1639_v43	Listeria_marthii_529731_v1	0.8841
Listeria_monocytogenes_1639_v42	Listeria_marthii_529731_v1	0.8804
Listeria_monocytogenes_1639_v41	Listeria_marthii_529731_v1	0.8766
Listeria_monocytogenes_1639_v40	Listeria_marthii_529731_v1	0.8891
Listeria_monocytogenes_1639_v3	Listeria_marthii_529731_v1	0.8749
Listeria_monocytogenes_1639_v39	Listeria_marthii_529731_v1	0.8888
Listeria_monocytogenes_1639_v38	Listeria_marthii_529731_v1	0.8864
Listeria_monocytogenes_1639_v37	Listeria_marthii_529731_v1	0.8826
Listeria_monocytogenes_1639_v36	Listeria_marthii_529731_v1	0.8795
Listeria_monocytogenes_1639_v35	Listeria_marthii_529731_v1	0.8897
Listeria_monocytogenes_1639_v34	Listeria_marthii_529731_v1	0.8896
Listeria_monocytogenes_1639_v33	Listeria_marthii_529731_v1	0.8958
Listeria_monocytogenes_1639_v32	Listeria_marthii_529731_v1	0.8894
Listeria_monocytogenes_1639_v31	Listeria_marthii_529731_v1	0.8899
Listeria_monocytogenes_1639_v30	Listeria_marthii_529731_v1	0.887
Listeria_monocytogenes_1639_v2	Listeria_marthii_529731_v1	0.8652
Listeria_monocytogenes_1639_v29	Listeria_marthii_529731_v1	0.8782
Listeria_monocytogenes_1639_v28	Listeria_marthii_529731_v1	0.8842
Listeria_monocytogenes_1639_v27	Listeria_marthii_529731_v1	0.8795
Listeria_monocytogenes_1639_v26	Listeria_marthii_529731_v1	0.8796
Listeria_monocytogenes_1639_v25	Listeria_marthii_529731_v1	0.8793
Listeria_monocytogenes_1639_v24	Listeria_marthii_529731_v1	0.9015
Listeria_monocytogenes_1639_v23	Listeria_marthii_529731_v1	0.9012
Listeria_monocytogenes_1639_v22	Listeria_marthii_529731_v1	0.8978
Listeria_monocytogenes_1639_v21	Listeria_marthii_529731_v1	0.8997
Listeria_monocytogenes_1639_v20	Listeria_marthii_529731_v1	0.883
Listeria_monocytogenes_1639_v1	Listeria_marthii_529731_v1	0.8773
Listeria_monocytogenes_1639_v19	Listeria_marthii_529731_v1	0.8827
Listeria_monocytogenes_1639_v198	Listeria_marthii_529731_v1	0.8617
Listeria_monocytogenes_1639_v197	Listeria_marthii_529731_v1	0.8949
Listeria_monocytogenes_1639_v196	Listeria_marthii_529731_v1	0.8815
Listeria_monocytogenes_1639_v195	Listeria_marthii_529731_v1	0.9175
Listeria_monocytogenes_1639_v194	Listeria_marthii_529731_v1	0.9168
Listeria_monocytogenes_1639_v193	Listeria_marthii_529731_v1	0.9168
Listeria_monocytogenes_1639_v192	Listeria_marthii_529731_v1	0.9004
Listeria_monocytogenes_1639_v191	Listeria_marthii_529731_v1	0.9071
Listeria_monocytogenes_1639_v190	Listeria_marthii_529731_v1	0.9072
Listeria_monocytogenes_1639_v18	Listeria_marthii_529731_v1	0.8963
Listeria_monocytogenes_1639_v189	Listeria_marthii_529731_v1	0.9065
Listeria_monocytogenes_1639_v188	Listeria_marthii_529731_v1	0.9109
Listeria_monocytogenes_1639_v187	Listeria_marthii_529731_v1	0.9069
Listeria_monocytogenes_1639_v186	Listeria_marthii_529731_v1	0.9076
Listeria_monocytogenes_1639_v185	Listeria_marthii_529731_v1	0.9072
Listeria_monocytogenes_1639_v184	Listeria_marthii_529731_v1	0.9068
Listeria_monocytogenes_1639_v183	Listeria_marthii_529731_v1	0.9011
Listeria_monocytogenes_1639_v182	Listeria_marthii_529731_v1	0.9028
Listeria_monocytogenes_1639_v181	Listeria_marthii_529731_v1	0.9196
Listeria_monocytogenes_1639_v180	Listeria_marthii_529731_v1	0.9196
Listeria_monocytogenes_1639_v17	Listeria_marthii_529731_v1	0.8963
Listeria_monocytogenes_1639_v179	Listeria_marthii_529731_v1	0.8983
Listeria_monocytogenes_1639_v178	Listeria_marthii_529731_v1	0.9023
Listeria_monocytogenes_1639_v177	Listeria_marthii_529731_v1	0.9084
Listeria_monocytogenes_1639_v176	Listeria_marthii_529731_v1	0.9146
Listeria_monocytogenes_1639_v175	Listeria_marthii_529731_v1	0.9013
Listeria_monocytogenes_1639_v174	Listeria_marthii_529731_v1	0.9066

Listeria_monocytogenes_1639_v173	Listeria_marthii_529731_v1	0.9074
Listeria_monocytogenes_1639_v172	Listeria_marthii_529731_v1	0.9185
Listeria_monocytogenes_1639_v171	Listeria_marthii_529731_v1	0.8944
Listeria_monocytogenes_1639_v170	Listeria_marthii_529731_v1	0.9018
Listeria_monocytogenes_1639_v16	Listeria_marthii_529731_v1	0.8712
Listeria_monocytogenes_1639_v169	Listeria_marthii_529731_v1	0.8978
Listeria_monocytogenes_1639_v168	Listeria_marthii_529731_v1	0.8987
Listeria_monocytogenes_1639_v167	Listeria_marthii_529731_v1	0.9003
Listeria_monocytogenes_1639_v166	Listeria_marthii_529731_v1	0.899
Listeria_monocytogenes_1639_v165	Listeria_marthii_529731_v1	0.8974
Listeria_monocytogenes_1639_v164	Listeria_marthii_529731_v1	0.9123
Listeria_monocytogenes_1639_v163	Listeria_marthii_529731_v1	0.9048
Listeria_monocytogenes_1639_v162	Listeria_marthii_529731_v1	0.9123
Listeria_monocytogenes_1639_v161	Listeria_marthii_529731_v1	0.9135
Listeria_monocytogenes_1639_v160	Listeria_marthii_529731_v1	0.9139
Listeria_monocytogenes_1639_v15	Listeria_marthii_529731_v1	0.881
Listeria_monocytogenes_1639_v159	Listeria_marthii_529731_v1	0.908
Listeria_monocytogenes_1639_v158	Listeria_marthii_529731_v1	0.9131
Listeria_monocytogenes_1639_v157	Listeria_marthii_529731_v1	0.9131
Listeria_monocytogenes_1639_v156	Listeria_marthii_529731_v1	0.8952
Listeria_monocytogenes_1639_v155	Listeria_marthii_529731_v1	0.906
Listeria_monocytogenes_1639_v154	Listeria_marthii_529731_v1	0.8958
Listeria_monocytogenes_1639_v153	Listeria_marthii_529731_v1	0.9135
Listeria_monocytogenes_1639_v152	Listeria_marthii_529731_v1	0.9083
Listeria_monocytogenes_1639_v151	Listeria_marthii_529731_v1	0.9083
Listeria_monocytogenes_1639_v150	Listeria_marthii_529731_v1	0.8987
Listeria_monocytogenes_1639_v14	Listeria_marthii_529731_v1	0.8871
Listeria_monocytogenes_1639_v149	Listeria_marthii_529731_v1	0.8986
Listeria_monocytogenes_1639_v148	Listeria_marthii_529731_v1	0.9069
Listeria_monocytogenes_1639_v147	Listeria_marthii_529731_v1	0.894
Listeria_monocytogenes_1639_v146	Listeria_marthii_529731_v1	0.9037
Listeria_monocytogenes_1639_v145	Listeria_marthii_529731_v1	0.9005
Listeria_monocytogenes_1639_v144	Listeria_marthii_529731_v1	0.9138
Listeria_monocytogenes_1639_v143	Listeria_marthii_529731_v1	0.9031
Listeria_monocytogenes_1639_v142	Listeria_marthii_529731_v1	0.9138
Listeria_monocytogenes_1639_v141	Listeria_marthii_529731_v1	0.908
Listeria_monocytogenes_1639_v140	Listeria_marthii_529731_v1	0.9138
Listeria_monocytogenes_1639_v13	Listeria_marthii_529731_v1	0.8813
Listeria_monocytogenes_1639_v139	Listeria_marthii_529731_v1	0.9094
Listeria_monocytogenes_1639_v138	Listeria_marthii_529731_v1	0.915
Listeria_monocytogenes_1639_v137	Listeria_marthii_529731_v1	0.9143
Listeria_monocytogenes_1639_v136	Listeria_marthii_529731_v1	0.8962
Listeria_monocytogenes_1639_v135	Listeria_marthii_529731_v1	0.9105
Listeria_monocytogenes_1639_v134	Listeria_marthii_529731_v1	0.9078
Listeria_monocytogenes_1639_v133	Listeria_marthii_529731_v1	0.8981
Listeria_monocytogenes_1639_v132	Listeria_marthii_529731_v1	0.9132
Listeria_monocytogenes_1639_v131	Listeria_marthii_529731_v1	0.9085
Listeria_monocytogenes_1639_v130	Listeria_marthii_529731_v1	0.889
Listeria_monocytogenes_1639_v12	Listeria_marthii_529731_v1	0.875
Listeria_monocytogenes_1639_v129	Listeria_marthii_529731_v1	0.9055
Listeria_monocytogenes_1639_v128	Listeria_marthii_529731_v1	0.9046
Listeria_monocytogenes_1639_v127	Listeria_marthii_529731_v1	0.9065
Listeria_monocytogenes_1639_v126	Listeria_marthii_529731_v1	0.9031
Listeria_monocytogenes_1639_v125	Listeria_marthii_529731_v1	0.8973
Listeria_monocytogenes_1639_v124	Listeria_marthii_529731_v1	0.9137
Listeria_monocytogenes_1639_v123	Listeria_marthii_529731_v1	0.9084
Listeria_monocytogenes_1639_v122	Listeria_marthii_529731_v1	0.9087
Listeria_monocytogenes_1639_v121	Listeria_marthii_529731_v1	0.9087
Listeria_monocytogenes_1639_v120	Listeria_marthii_529731_v1	0.9088
Listeria_monocytogenes_1639_v11	Listeria_marthii_529731_v1	0.8795
Listeria_monocytogenes_1639_v119	Listeria_marthii_529731_v1	0.9087
Listeria_monocytogenes_1639_v118	Listeria_marthii_529731_v1	0.9087
Listeria_monocytogenes_1639_v117	Listeria_marthii_529731_v1	0.8932
Listeria_monocytogenes_1639_v116	Listeria_marthii_529731_v1	0.9077
Listeria_monocytogenes_1639_v115	Listeria_marthii_529731_v1	0.9081
Listeria_monocytogenes_1639_v114	Listeria_marthii_529731_v1	0.8948
Listeria_monocytogenes_1639_v113	Listeria_marthii_529731_v1	0.9
Listeria_monocytogenes_1639_v112	Listeria_marthii_529731_v1	0.9081
Listeria_monocytogenes_1639_v111	Listeria_marthii_529731_v1	0.8938
Listeria_monocytogenes_1639_v110	Listeria_marthii_529731_v1	0.899
Listeria_monocytogenes_1639_v10	Listeria_marthii_529731_v1	0.8737
Listeria_monocytogenes_1639_v109	Listeria_marthii_529731_v1	0.8934
Listeria_monocytogenes_1639_v108	Listeria_marthii_529731_v1	0.9098
Listeria_monocytogenes_1639_v107	Listeria_marthii_529731_v1	0.9056
Listeria_monocytogenes_1639_v106	Listeria_marthii_529731_v1	0.9056
Listeria_monocytogenes_1639_v105	Listeria_marthii_529731_v1	0.906
Listeria_monocytogenes_1639_v104	Listeria_marthii_529731_v1	0.906
Listeria_monocytogenes_1639_v103	Listeria_marthii_529731_v1	0.906
Listeria_monocytogenes_1639_v102	Listeria_marthii_529731_v1	0.8936
Listeria_monocytogenes_1639_v101	Listeria_marthii_529731_v1	0.8824
Listeria_monocytogenes_1639_v100	Listeria_marthii_529731_v1	0.8799
Listeria_monocytogenes_1639	Listeria_marthii_529731_v1	0.9069
Listeria_monocytogenes_10403S_393133	Listeria_marthii_529731_v1	0.908
Listeria_monocytogenes_08-5923_637381	Listeria_marthii_529731_v1	0.8841
Listeria_monocytogenes_08-5578_653938	Listeria_marthii_529731_v1	0.8709
Listeria_monocytogenes_07PF0776_1126011	Listeria_marthii_529731_v1	0.9097
Listeria_marthii_FSL_S4-120_702457	Listeria_marthii_529731_v1	0.8756
Listeria_marthii_529731_v1	Listeria_marthii_529731_v1	0.0624
Listeria_welshimeri_1643	Listeria_marthii_FSL_S4-120_702457	0.8287
Listeria_welshimeri_serovar_6b_str_SLCC5334_386043	Listeria_marthii_FSL_S4-120_702457	0.8284
Listeria_seeligeri_serovar_1_2b_str_SLCC3954_683837	Listeria_marthii_FSL_S4-120_702457	0.8323

Listeria_seeligeri_FSL_S4-171_702452	Listeria_marthii_FSL_S4-120_702457	0.7824
Listeria_seeligeri_FSL_N1-067_702453	Listeria_marthii_FSL_S4-120_702457	0.773
Listeria_seeligeri_1640_v1	Listeria_marthii_FSL_S4-120_702457	0.8312
Listeria_seeligeri_1640	Listeria_marthii_FSL_S4-120_702457	0.8187
Listeria_monocytogenes_WSLC1042_1457188	Listeria_marthii_FSL_S4-120_702457	0.8628
Listeria_monocytogenes_WSLC1001_1457187	Listeria_marthii_FSL_S4-120_702457	0.8505
Listeria_monocytogenes_SLCC7179_879090	Listeria_marthii_FSL_S4-120_702457	0.8593
Listeria_monocytogenes_SLCC5850_882096	Listeria_marthii_FSL_S4-120_702457	0.8545
Listeria_monocytogenes_SLCC2755_932919	Listeria_marthii_FSL_S4-120_702457	0.8476
Listeria_monocytogenes_SLCC2540_879089	Listeria_marthii_FSL_S4-120_702457	0.8501
Listeria_monocytogenes_SLCC2479_882020	Listeria_marthii_FSL_S4-120_702457	0.8484
Listeria_monocytogenes_SLCC2378_879088	Listeria_marthii_FSL_S4-120_702457	0.8638
Listeria_monocytogenes_SLCC2376_882097	Listeria_marthii_FSL_S4-120_702457	0.8784
Listeria_monocytogenes_SLCC2372_932920	Listeria_marthii_FSL_S4-120_702457	0.8416
Listeria_monocytogenes_serotype_4b_str_LL195_1230340	Listeria_marthii_FSL_S4-120_702457	0.8692
Listeria_monocytogenes_serotype_4b_str_LIS0087_1134669	Listeria_marthii_FSL_S4-120_702457	0.8314
Listeria_monocytogenes_serotype_4b_str_F2365_265669	Listeria_marthii_FSL_S4-120_702457	0.8688
Listeria_monocytogenes_serotype_4b_str_CLIP_80459_568819	Listeria_marthii_FSL_S4-120_702457	0.8699
Listeria_monocytogenes_serotype_4b_str_81-0861_1196161	Listeria_marthii_FSL_S4-120_702457	0.8541
Listeria_monocytogenes_serotype_4b_str_81-0592_1196160	Listeria_marthii_FSL_S4-120_702457	0.8551
Listeria_monocytogenes_serotype_4b_str_81-0558_1196159	Listeria_marthii_FSL_S4-120_702457	0.8543
Listeria_monocytogenes_serotype_4b_str_10-0809_1196162	Listeria_marthii_FSL_S4-120_702457	0.8551
Listeria_monocytogenes_serotype_4b_str_02-6680_1196175	Listeria_marthii_FSL_S4-120_702457	0.8689
Listeria_monocytogenes_serotype_4b_str_02-6679_1196174	Listeria_marthii_FSL_S4-120_702457	0.8689
Listeria_monocytogenes_serotype_4b_str_02-1792_1196173	Listeria_marthii_FSL_S4-120_702457	0.8551
Listeria_monocytogenes_serotype_4b_str_02-1289_1196172	Listeria_marthii_FSL_S4-120_702457	0.8551
Listeria_monocytogenes_serotype_4b_str_02-1103_1196171	Listeria_marthii_FSL_S4-120_702457	0.8551
Listeria_monocytogenes_serotype_3c_str_10-5027_1196195	Listeria_marthii_FSL_S4-120_702457	0.8472
Listeria_monocytogenes_serotype_1_2c_str_10-5026_1196194	Listeria_marthii_FSL_S4-120_702457	0.8481
Listeria_monocytogenes_serotype_1_2c_str_10-5025_1196193	Listeria_marthii_FSL_S4-120_702457	0.8481
Listeria_monocytogenes_serotype_1_2b_str_10-0811_1196164	Listeria_marthii_FSL_S4-120_702457	0.8445
Listeria_monocytogenes_serotype_1_2b_str_10-0810_1196163	Listeria_marthii_FSL_S4-120_702457	0.8445
Listeria_monocytogenes_serotype_1_2a_str_99-6370_1196179	Listeria_marthii_FSL_S4-120_702457	0.834
Listeria_monocytogenes_serotype_1_2a_str_98-2035_1196178	Listeria_marthii_FSL_S4-120_702457	0.834
Listeria_monocytogenes_serotype_1_2a_str_95-0093_1196177	Listeria_marthii_FSL_S4-120_702457	0.8391
Listeria_monocytogenes_serotype_1_2a_str_88-0478_1196176	Listeria_marthii_FSL_S4-120_702457	0.8379
Listeria_monocytogenes_serotype_1_2a_str_10-5024_1196192	Listeria_marthii_FSL_S4-120_702457	0.8391
Listeria_monocytogenes_serotype_1_2a_str_10-4758_1196168	Listeria_marthii_FSL_S4-120_702457	0.8551
Listeria_monocytogenes_serotype_1_2a_str_10-4754_1196167	Listeria_marthii_FSL_S4-120_702457	0.8555
Listeria_monocytogenes_serotype_1_2a_str_10-1321_1196191	Listeria_marthii_FSL_S4-120_702457	0.8391
Listeria_monocytogenes_serotype_1_2a_str_10-1047_1196190	Listeria_marthii_FSL_S4-120_702457	0.8398
Listeria_monocytogenes_serotype_1_2a_str_10-1046_1196189	Listeria_marthii_FSL_S4-120_702457	0.8398
Listeria_monocytogenes_serotype_1_2a_str_10-0934_1196170	Listeria_marthii_FSL_S4-120_702457	0.8658
Listeria_monocytogenes_serotype_1_2a_str_10-0933_1196169	Listeria_marthii_FSL_S4-120_702457	0.8658
Listeria_monocytogenes_serotype_1_2a_str_10-0815_1196188	Listeria_marthii_FSL_S4-120_702457	0.8353
Listeria_monocytogenes_serotype_1_2a_str_10-0814_1196187	Listeria_marthii_FSL_S4-120_702457	0.8395
Listeria_monocytogenes_serotype_1_2a_str_10-0813_1196166	Listeria_marthii_FSL_S4-120_702457	0.8486
Listeria_monocytogenes_serotype_1_2a_str_10-0812_1196165	Listeria_marthii_FSL_S4-120_702457	0.8486
Listeria_monocytogenes_serotype_1_2a_str_08-7669_1196186	Listeria_marthii_FSL_S4-120_702457	0.8482
Listeria_monocytogenes_serotype_1_2a_str_08-7374_1196185	Listeria_marthii_FSL_S4-120_702457	0.8391
Listeria_monocytogenes_serotype_1_2a_str_08-6997_1196184	Listeria_marthii_FSL_S4-120_702457	0.8353
Listeria_monocytogenes_serotype_1_2a_str_08-6569_1196183	Listeria_marthii_FSL_S4-120_702457	0.8353
Listeria_monocytogenes_serotype_1_2a_str_08-6056_1196182	Listeria_marthii_FSL_S4-120_702457	0.8353
Listeria_monocytogenes_serotype_1_2a_str_04-5457_1196181	Listeria_marthii_FSL_S4-120_702457	0.8391
Listeria_monocytogenes_serotype_1_2a_str_02-5993_1196180	Listeria_marthii_FSL_S4-120_702457	0.8391
Listeria_monocytogenes_R479a_1437838	Listeria_marthii_FSL_S4-120_702457	0.8388
Listeria_monocytogenes_N53-1_1234142	Listeria_marthii_FSL_S4-120_702457	0.8157
Listeria_monocytogenes_M7_1030009	Listeria_marthii_FSL_S4-120_702457	0.8513
Listeria_monocytogenes_LO28_393132	Listeria_marthii_FSL_S4-120_702457	0.8471
Listeria_monocytogenes_L99_563174	Listeria_marthii_FSL_S4-120_702457	0.8516
Listeria_monocytogenes_L312_882094	Listeria_marthii_FSL_S4-120_702457	0.8699
Listeria_monocytogenes_J1816_930781	Listeria_marthii_FSL_S4-120_702457	0.8465
Listeria_monocytogenes_J1-220_930782	Listeria_marthii_FSL_S4-120_702457	0.8471
Listeria_monocytogenes_J0161_393130	Listeria_marthii_FSL_S4-120_702457	0.8502
Listeria_monocytogenes_HCC23_552536	Listeria_marthii_FSL_S4-120_702457	0.8513
Listeria_monocytogenes_FSL_R2-561_393126	Listeria_marthii_FSL_S4-120_702457	0.8471
Listeria_monocytogenes_FSL_J1-208_393119	Listeria_marthii_FSL_S4-120_702457	0.867
Listeria_monocytogenes_FSL_J1-175_393118	Listeria_marthii_FSL_S4-120_702457	0.8553
Listeria_monocytogenes_Finland_1998_393127	Listeria_marthii_FSL_S4-120_702457	0.8674
Listeria_monocytogenes_EGD-e_169963_v1	Listeria_marthii_FSL_S4-120_702457	0.8569
Listeria_monocytogenes_EGD-e_169963	Listeria_marthii_FSL_S4-120_702457	0.8573
Listeria_monocytogenes_EGD_1334565	Listeria_marthii_FSL_S4-120_702457	0.8552
Listeria_monocytogenes_CFSAN002349_1299895	Listeria_marthii_FSL_S4-120_702457	0.8667
Listeria_monocytogenes_ATCC_19117_882095_v1	Listeria_marthii_FSL_S4-120_702457	0.8654
Listeria_monocytogenes_ATCC_19117_882095	Listeria_marthii_FSL_S4-120_702457	0.8654
Listeria_monocytogenes_6179_1288295	Listeria_marthii_FSL_S4-120_702457	0.8371
Listeria_monocytogenes_1639_v9	Listeria_marthii_FSL_S4-120_702457	0.8285
Listeria_monocytogenes_1639_v99	Listeria_marthii_FSL_S4-120_702457	0.8473
Listeria_monocytogenes_1639_v98	Listeria_marthii_FSL_S4-120_702457	0.8458
Listeria_monocytogenes_1639_v97	Listeria_marthii_FSL_S4-120_702457	0.8538
Listeria_monocytogenes_1639_v96	Listeria_marthii_FSL_S4-120_702457	0.8467
Listeria_monocytogenes_1639_v95	Listeria_marthii_FSL_S4-120_702457	0.8467
Listeria_monocytogenes_1639_v94	Listeria_marthii_FSL_S4-120_702457	0.847
Listeria_monocytogenes_1639_v93	Listeria_marthii_FSL_S4-120_702457	0.8455
Listeria_monocytogenes_1639_v92	Listeria_marthii_FSL_S4-120_702457	0.847
Listeria_monocytogenes_1639_v91	Listeria_marthii_FSL_S4-120_702457	0.8509
Listeria_monocytogenes_1639_v90	Listeria_marthii_FSL_S4-120_702457	0.8458
Listeria_monocytogenes_1639_v8	Listeria_marthii_FSL_S4-120_702457	0.829
Listeria_monocytogenes_1639_v89	Listeria_marthii_FSL_S4-120_702457	0.8587
Listeria_monocytogenes_1639_v88	Listeria_marthii_FSL_S4-120_702457	0.8481
Listeria_monocytogenes_1639_v87	Listeria_marthii_FSL_S4-120_702457	0.844

Listeria_monocytogenes_1639_v86	Listeria_marthii_FSL_S4-120_702457	0.8437
Listeria_monocytogenes_1639_v85	Listeria_marthii_FSL_S4-120_702457	0.8539
Listeria_monocytogenes_1639_v84	Listeria_marthii_FSL_S4-120_702457	0.8442
Listeria_monocytogenes_1639_v83	Listeria_marthii_FSL_S4-120_702457	0.8553
Listeria_monocytogenes_1639_v82	Listeria_marthii_FSL_S4-120_702457	0.855
Listeria_monocytogenes_1639_v81	Listeria_marthii_FSL_S4-120_702457	0.8492
Listeria_monocytogenes_1639_v80	Listeria_marthii_FSL_S4-120_702457	0.8504
Listeria_monocytogenes_1639_v7	Listeria_marthii_FSL_S4-120_702457	0.829
Listeria_monocytogenes_1639_v79	Listeria_marthii_FSL_S4-120_702457	0.8476
Listeria_monocytogenes_1639_v78	Listeria_marthii_FSL_S4-120_702457	0.8485
Listeria_monocytogenes_1639_v77	Listeria_marthii_FSL_S4-120_702457	0.8495
Listeria_monocytogenes_1639_v76	Listeria_marthii_FSL_S4-120_702457	0.8396
Listeria_monocytogenes_1639_v75	Listeria_marthii_FSL_S4-120_702457	0.8516
Listeria_monocytogenes_1639_v74	Listeria_marthii_FSL_S4-120_702457	0.8433
Listeria_monocytogenes_1639_v73	Listeria_marthii_FSL_S4-120_702457	0.8426
Listeria_monocytogenes_1639_v72	Listeria_marthii_FSL_S4-120_702457	0.854
Listeria_monocytogenes_1639_v71	Listeria_marthii_FSL_S4-120_702457	0.831
Listeria_monocytogenes_1639_v70	Listeria_marthii_FSL_S4-120_702457	0.8448
Listeria_monocytogenes_1639_v6	Listeria_marthii_FSL_S4-120_702457	0.8286
Listeria_monocytogenes_1639_v69	Listeria_marthii_FSL_S4-120_702457	0.8551
Listeria_monocytogenes_1639_v68	Listeria_marthii_FSL_S4-120_702457	0.853
Listeria_monocytogenes_1639_v67	Listeria_marthii_FSL_S4-120_702457	0.8469
Listeria_monocytogenes_1639_v66	Listeria_marthii_FSL_S4-120_702457	0.8537
Listeria_monocytogenes_1639_v65	Listeria_marthii_FSL_S4-120_702457	0.8421
Listeria_monocytogenes_1639_v64	Listeria_marthii_FSL_S4-120_702457	0.8463
Listeria_monocytogenes_1639_v63	Listeria_marthii_FSL_S4-120_702457	0.8447
Listeria_monocytogenes_1639_v62	Listeria_marthii_FSL_S4-120_702457	0.8453
Listeria_monocytogenes_1639_v61	Listeria_marthii_FSL_S4-120_702457	0.8416
Listeria_monocytogenes_1639_v60	Listeria_marthii_FSL_S4-120_702457	0.8523
Listeria_monocytogenes_1639_v5	Listeria_marthii_FSL_S4-120_702457	0.8134
Listeria_monocytogenes_1639_v59	Listeria_marthii_FSL_S4-120_702457	0.8556
Listeria_monocytogenes_1639_v58	Listeria_marthii_FSL_S4-120_702457	0.8553
Listeria_monocytogenes_1639_v57	Listeria_marthii_FSL_S4-120_702457	0.8435
Listeria_monocytogenes_1639_v56	Listeria_marthii_FSL_S4-120_702457	0.8556
Listeria_monocytogenes_1639_v55	Listeria_marthii_FSL_S4-120_702457	0.8398
Listeria_monocytogenes_1639_v54	Listeria_marthii_FSL_S4-120_702457	0.8487
Listeria_monocytogenes_1639_v53	Listeria_marthii_FSL_S4-120_702457	0.8487
Listeria_monocytogenes_1639_v52	Listeria_marthii_FSL_S4-120_702457	0.8523
Listeria_monocytogenes_1639_v51	Listeria_marthii_FSL_S4-120_702457	0.85
Listeria_monocytogenes_1639_v50	Listeria_marthii_FSL_S4-120_702457	0.8383
Listeria_monocytogenes_1639_v4	Listeria_marthii_FSL_S4-120_702457	0.8252
Listeria_monocytogenes_1639_v49	Listeria_marthii_FSL_S4-120_702457	0.8545
Listeria_monocytogenes_1639_v48	Listeria_marthii_FSL_S4-120_702457	0.8428
Listeria_monocytogenes_1639_v47	Listeria_marthii_FSL_S4-120_702457	0.8388
Listeria_monocytogenes_1639_v46	Listeria_marthii_FSL_S4-120_702457	0.8524
Listeria_monocytogenes_1639_v45	Listeria_marthii_FSL_S4-120_702457	0.8309
Listeria_monocytogenes_1639_v44	Listeria_marthii_FSL_S4-120_702457	0.8391
Listeria_monocytogenes_1639_v43	Listeria_marthii_FSL_S4-120_702457	0.8391
Listeria_monocytogenes_1639_v42	Listeria_marthii_FSL_S4-120_702457	0.8351
Listeria_monocytogenes_1639_v41	Listeria_marthii_FSL_S4-120_702457	0.8276
Listeria_monocytogenes_1639_v40	Listeria_marthii_FSL_S4-120_702457	0.8475
Listeria_monocytogenes_1639_v3	Listeria_marthii_FSL_S4-120_702457	0.8255
Listeria_monocytogenes_1639_v39	Listeria_marthii_FSL_S4-120_702457	0.8434
Listeria_monocytogenes_1639_v38	Listeria_marthii_FSL_S4-120_702457	0.8445
Listeria_monocytogenes_1639_v37	Listeria_marthii_FSL_S4-120_702457	0.84
Listeria_monocytogenes_1639_v36	Listeria_marthii_FSL_S4-120_702457	0.836
Listeria_monocytogenes_1639_v35	Listeria_marthii_FSL_S4-120_702457	0.8477
Listeria_monocytogenes_1639_v34	Listeria_marthii_FSL_S4-120_702457	0.8459
Listeria_monocytogenes_1639_v33	Listeria_marthii_FSL_S4-120_702457	0.8445
Listeria_monocytogenes_1639_v32	Listeria_marthii_FSL_S4-120_702457	0.8386
Listeria_monocytogenes_1639_v31	Listeria_marthii_FSL_S4-120_702457	0.8459
Listeria_monocytogenes_1639_v30	Listeria_marthii_FSL_S4-120_702457	0.8473
Listeria_monocytogenes_1639_v2	Listeria_marthii_FSL_S4-120_702457	0.8096
Listeria_monocytogenes_1639_v29	Listeria_marthii_FSL_S4-120_702457	0.8349
Listeria_monocytogenes_1639_v28	Listeria_marthii_FSL_S4-120_702457	0.839
Listeria_monocytogenes_1639_v27	Listeria_marthii_FSL_S4-120_702457	0.8308
Listeria_monocytogenes_1639_v26	Listeria_marthii_FSL_S4-120_702457	0.8306
Listeria_monocytogenes_1639_v25	Listeria_marthii_FSL_S4-120_702457	0.8306
Listeria_monocytogenes_1639_v24	Listeria_marthii_FSL_S4-120_702457	0.8471
Listeria_monocytogenes_1639_v23	Listeria_marthii_FSL_S4-120_702457	0.8467
Listeria_monocytogenes_1639_v22	Listeria_marthii_FSL_S4-120_702457	0.8462
Listeria_monocytogenes_1639_v21	Listeria_marthii_FSL_S4-120_702457	0.8467
Listeria_monocytogenes_1639_v20	Listeria_marthii_FSL_S4-120_702457	0.8366
Listeria_monocytogenes_1639_v1	Listeria_marthii_FSL_S4-120_702457	0.8317
Listeria_monocytogenes_1639_v19	Listeria_marthii_FSL_S4-120_702457	0.8363
Listeria_monocytogenes_1639_v198	Listeria_marthii_FSL_S4-120_702457	0.8188
Listeria_monocytogenes_1639_v197	Listeria_marthii_FSL_S4-120_702457	0.8443
Listeria_monocytogenes_1639_v196	Listeria_marthii_FSL_S4-120_702457	0.8392
Listeria_monocytogenes_1639_v195	Listeria_marthii_FSL_S4-120_702457	0.8803
Listeria_monocytogenes_1639_v194	Listeria_marthii_FSL_S4-120_702457	0.8793
Listeria_monocytogenes_1639_v193	Listeria_marthii_FSL_S4-120_702457	0.8789
Listeria_monocytogenes_1639_v192	Listeria_marthii_FSL_S4-120_702457	0.8599
Listeria_monocytogenes_1639_v191	Listeria_marthii_FSL_S4-120_702457	0.8686
Listeria_monocytogenes_1639_v190	Listeria_marthii_FSL_S4-120_702457	0.8664
Listeria_monocytogenes_1639_v18	Listeria_marthii_FSL_S4-120_702457	0.8384
Listeria_monocytogenes_1639_v189	Listeria_marthii_FSL_S4-120_702457	0.8657
Listeria_monocytogenes_1639_v188	Listeria_marthii_FSL_S4-120_702457	0.8689
Listeria_monocytogenes_1639_v187	Listeria_marthii_FSL_S4-120_702457	0.8663
Listeria_monocytogenes_1639_v186	Listeria_marthii_FSL_S4-120_702457	0.8667
Listeria_monocytogenes_1639_v185	Listeria_marthii_FSL_S4-120_702457	0.8667
Listeria_monocytogenes_1639_v184	Listeria_marthii_FSL_S4-120_702457	0.866

Listeria_monocytogenes_1639_v183	Listeria_marthii_FSL_S4-120_702457	0.8679
Listeria_monocytogenes_1639_v182	Listeria_marthii_FSL_S4-120_702457	0.8693
Listeria_monocytogenes_1639_v181	Listeria_marthii_FSL_S4-120_702457	0.8792
Listeria_monocytogenes_1639_v180	Listeria_marthii_FSL_S4-120_702457	0.8792
Listeria_monocytogenes_1639_v17	Listeria_marthii_FSL_S4-120_702457	0.8387
Listeria_monocytogenes_1639_v179	Listeria_marthii_FSL_S4-120_702457	0.8615
Listeria_monocytogenes_1639_v178	Listeria_marthii_FSL_S4-120_702457	0.8688
Listeria_monocytogenes_1639_v177	Listeria_marthii_FSL_S4-120_702457	0.8643
Listeria_monocytogenes_1639_v176	Listeria_marthii_FSL_S4-120_702457	0.8767
Listeria_monocytogenes_1639_v175	Listeria_marthii_FSL_S4-120_702457	0.859
Listeria_monocytogenes_1639_v174	Listeria_marthii_FSL_S4-120_702457	0.865
Listeria_monocytogenes_1639_v173	Listeria_marthii_FSL_S4-120_702457	0.8642
Listeria_monocytogenes_1639_v172	Listeria_marthii_FSL_S4-120_702457	0.8772
Listeria_monocytogenes_1639_v171	Listeria_marthii_FSL_S4-120_702457	0.8529
Listeria_monocytogenes_1639_v170	Listeria_marthii_FSL_S4-120_702457	0.8598
Listeria_monocytogenes_1639_v16	Listeria_marthii_FSL_S4-120_702457	0.8268
Listeria_monocytogenes_1639_v169	Listeria_marthii_FSL_S4-120_702457	0.8541
Listeria_monocytogenes_1639_v168	Listeria_marthii_FSL_S4-120_702457	0.8644
Listeria_monocytogenes_1639_v167	Listeria_marthii_FSL_S4-120_702457	0.8602
Listeria_monocytogenes_1639_v166	Listeria_marthii_FSL_S4-120_702457	0.8647
Listeria_monocytogenes_1639_v165	Listeria_marthii_FSL_S4-120_702457	0.8538
Listeria_monocytogenes_1639_v164	Listeria_marthii_FSL_S4-120_702457	0.8702
Listeria_monocytogenes_1639_v163	Listeria_marthii_FSL_S4-120_702457	0.8625
Listeria_monocytogenes_1639_v162	Listeria_marthii_FSL_S4-120_702457	0.8702
Listeria_monocytogenes_1639_v161	Listeria_marthii_FSL_S4-120_702457	0.8687
Listeria_monocytogenes_1639_v160	Listeria_marthii_FSL_S4-120_702457	0.8688
Listeria_monocytogenes_1639_v15	Listeria_marthii_FSL_S4-120_702457	0.8312
Listeria_monocytogenes_1639_v159	Listeria_marthii_FSL_S4-120_702457	0.8643
Listeria_monocytogenes_1639_v158	Listeria_marthii_FSL_S4-120_702457	0.8688
Listeria_monocytogenes_1639_v157	Listeria_marthii_FSL_S4-120_702457	0.8692
Listeria_monocytogenes_1639_v156	Listeria_marthii_FSL_S4-120_702457	0.846
Listeria_monocytogenes_1639_v155	Listeria_marthii_FSL_S4-120_702457	0.858
Listeria_monocytogenes_1639_v154	Listeria_marthii_FSL_S4-120_702457	0.8566
Listeria_monocytogenes_1639_v153	Listeria_marthii_FSL_S4-120_702457	0.8584
Listeria_monocytogenes_1639_v152	Listeria_marthii_FSL_S4-120_702457	0.8643
Listeria_monocytogenes_1639_v151	Listeria_marthii_FSL_S4-120_702457	0.8643
Listeria_monocytogenes_1639_v150	Listeria_marthii_FSL_S4-120_702457	0.8568
Listeria_monocytogenes_1639_v14	Listeria_marthii_FSL_S4-120_702457	0.8372
Listeria_monocytogenes_1639_v149	Listeria_marthii_FSL_S4-120_702457	0.8556
Listeria_monocytogenes_1639_v148	Listeria_marthii_FSL_S4-120_702457	0.8632
Listeria_monocytogenes_1639_v147	Listeria_marthii_FSL_S4-120_702457	0.8509
Listeria_monocytogenes_1639_v146	Listeria_marthii_FSL_S4-120_702457	0.866
Listeria_monocytogenes_1639_v145	Listeria_marthii_FSL_S4-120_702457	0.862
Listeria_monocytogenes_1639_v144	Listeria_marthii_FSL_S4-120_702457	0.8695
Listeria_monocytogenes_1639_v143	Listeria_marthii_FSL_S4-120_702457	0.8666
Listeria_monocytogenes_1639_v142	Listeria_marthii_FSL_S4-120_702457	0.8699
Listeria_monocytogenes_1639_v141	Listeria_marthii_FSL_S4-120_702457	0.8626
Listeria_monocytogenes_1639_v140	Listeria_marthii_FSL_S4-120_702457	0.8702
Listeria_monocytogenes_1639_v13	Listeria_marthii_FSL_S4-120_702457	0.8316
Listeria_monocytogenes_1639_v139	Listeria_marthii_FSL_S4-120_702457	0.8661
Listeria_monocytogenes_1639_v138	Listeria_marthii_FSL_S4-120_702457	0.8695
Listeria_monocytogenes_1639_v137	Listeria_marthii_FSL_S4-120_702457	0.8687
Listeria_monocytogenes_1639_v136	Listeria_marthii_FSL_S4-120_702457	0.8548
Listeria_monocytogenes_1639_v135	Listeria_marthii_FSL_S4-120_702457	0.8632
Listeria_monocytogenes_1639_v134	Listeria_marthii_FSL_S4-120_702457	0.8638
Listeria_monocytogenes_1639_v133	Listeria_marthii_FSL_S4-120_702457	0.8601
Listeria_monocytogenes_1639_v132	Listeria_marthii_FSL_S4-120_702457	0.8597
Listeria_monocytogenes_1639_v131	Listeria_marthii_FSL_S4-120_702457	0.8544
Listeria_monocytogenes_1639_v130	Listeria_marthii_FSL_S4-120_702457	0.8481
Listeria_monocytogenes_1639_v12	Listeria_marthii_FSL_S4-120_702457	0.8292
Listeria_monocytogenes_1639_v129	Listeria_marthii_FSL_S4-120_702457	0.8667
Listeria_monocytogenes_1639_v128	Listeria_marthii_FSL_S4-120_702457	0.8596
Listeria_monocytogenes_1639_v127	Listeria_marthii_FSL_S4-120_702457	0.8608
Listeria_monocytogenes_1639_v126	Listeria_marthii_FSL_S4-120_702457	0.8577
Listeria_monocytogenes_1639_v125	Listeria_marthii_FSL_S4-120_702457	0.8559
Listeria_monocytogenes_1639_v124	Listeria_marthii_FSL_S4-120_702457	0.8685
Listeria_monocytogenes_1639_v123	Listeria_marthii_FSL_S4-120_702457	0.8667
Listeria_monocytogenes_1639_v122	Listeria_marthii_FSL_S4-120_702457	0.8667
Listeria_monocytogenes_1639_v121	Listeria_marthii_FSL_S4-120_702457	0.8667
Listeria_monocytogenes_1639_v120	Listeria_marthii_FSL_S4-120_702457	0.8671
Listeria_monocytogenes_1639_v11	Listeria_marthii_FSL_S4-120_702457	0.8342
Listeria_monocytogenes_1639_v119	Listeria_marthii_FSL_S4-120_702457	0.8667
Listeria_monocytogenes_1639_v118	Listeria_marthii_FSL_S4-120_702457	0.8667
Listeria_monocytogenes_1639_v117	Listeria_marthii_FSL_S4-120_702457	0.8568
Listeria_monocytogenes_1639_v116	Listeria_marthii_FSL_S4-120_702457	0.8639
Listeria_monocytogenes_1639_v115	Listeria_marthii_FSL_S4-120_702457	0.8638
Listeria_monocytogenes_1639_v114	Listeria_marthii_FSL_S4-120_702457	0.8489
Listeria_monocytogenes_1639_v113	Listeria_marthii_FSL_S4-120_702457	0.8586
Listeria_monocytogenes_1639_v112	Listeria_marthii_FSL_S4-120_702457	0.8585
Listeria_monocytogenes_1639_v111	Listeria_marthii_FSL_S4-120_702457	0.8509
Listeria_monocytogenes_1639_v110	Listeria_marthii_FSL_S4-120_702457	0.849
Listeria_monocytogenes_1639_v10	Listeria_marthii_FSL_S4-120_702457	0.8292
Listeria_monocytogenes_1639_v109	Listeria_marthii_FSL_S4-120_702457	0.8485
Listeria_monocytogenes_1639_v108	Listeria_marthii_FSL_S4-120_702457	0.8546
Listeria_monocytogenes_1639_v107	Listeria_marthii_FSL_S4-120_702457	0.8643
Listeria_monocytogenes_1639_v106	Listeria_marthii_FSL_S4-120_702457	0.8643
Listeria_monocytogenes_1639_v105	Listeria_marthii_FSL_S4-120_702457	0.8647
Listeria_monocytogenes_1639_v104	Listeria_marthii_FSL_S4-120_702457	0.8643
Listeria_monocytogenes_1639_v103	Listeria_marthii_FSL_S4-120_702457	0.8647
Listeria_monocytogenes_1639_v102	Listeria_marthii_FSL_S4-120_702457	0.8492
Listeria_monocytogenes_1639_v101	Listeria_marthii_FSL_S4-120_702457	0.8385

Listeria_monocytogenes_1639_v100	Listeria_marthii_FSL_S4-120_702457	0.8379
Listeria_monocytogenes_1639	Listeria_marthii_FSL_S4-120_702457	0.8664
Listeria_monocytogenes_10403S_393133	Listeria_marthii_FSL_S4-120_702457	0.8592
Listeria_monocytogenes_08-5923_637381	Listeria_marthii_FSL_S4-120_702457	0.8391
Listeria_monocytogenes_08-5578_653938	Listeria_marthii_FSL_S4-120_702457	0.8268
Listeria_monocytogenes_07PF0776_1126011	Listeria_marthii_FSL_S4-120_702457	0.8661
Listeria_marthii_FSL_S4-120_702457	Listeria_marthii_FSL_S4-120_702457	0.0457
Listeria_welshimeri_1643	Listeria_monocytogenes_07PF0776_1126011	0.8841
Listeria_welshimeri_serovar_6b_str_SLCC5334_386043	Listeria_monocytogenes_07PF0776_1126011	0.8838
Listeria_seeligeri_serovar_1_2b_str_SLCC3954_683837	Listeria_monocytogenes_07PF0776_1126011	0.8875
Listeria_seeligeri_FSL_S4-171_702452	Listeria_monocytogenes_07PF0776_1126011	0.83
Listeria_seeligeri_FSL_N1-067_702453	Listeria_monocytogenes_07PF0776_1126011	0.8305
Listeria_seeligeri_1640_v1	Listeria_monocytogenes_07PF0776_1126011	0.8956
Listeria_seeligeri_1640	Listeria_monocytogenes_07PF0776_1126011	0.8855
Listeria_monocytogenes_WSLC1042_1457188	Listeria_monocytogenes_07PF0776_1126011	0.9832
Listeria_monocytogenes_WSLC1001_1457187	Listeria_monocytogenes_07PF0776_1126011	0.9299
Listeria_monocytogenes_SLCC7179_879090	Listeria_monocytogenes_07PF0776_1126011	0.94
Listeria_monocytogenes_SLCC5850_882096	Listeria_monocytogenes_07PF0776_1126011	0.9345
Listeria_monocytogenes_SLCC2755_932919	Listeria_monocytogenes_07PF0776_1126011	0.9495
Listeria_monocytogenes_SLCC2540_879089	Listeria_monocytogenes_07PF0776_1126011	0.9519
Listeria_monocytogenes_SLCC2479_882020	Listeria_monocytogenes_07PF0776_1126011	0.9288
Listeria_monocytogenes_SLCC2378_879088	Listeria_monocytogenes_07PF0776_1126011	0.9847
Listeria_monocytogenes_SLCC2376_882097	Listeria_monocytogenes_07PF0776_1126011	0.9446
Listeria_monocytogenes_SLCC2372_932920	Listeria_monocytogenes_07PF0776_1126011	0.9215
Listeria_monocytogenes_serotype_4b_str_LL195_1230340	Listeria_monocytogenes_07PF0776_1126011	0.9907
Listeria_monocytogenes_serotype_4b_str_LIS0087_1134669	Listeria_monocytogenes_07PF0776_1126011	0.9103
Listeria_monocytogenes_serotype_4b_str_F2365_265669	Listeria_monocytogenes_07PF0776_1126011	0.9907
Listeria_monocytogenes_serotype_4b_str_CLIP_80459_568819	Listeria_monocytogenes_07PF0776_1126011	0.9791
Listeria_monocytogenes_serotype_4b_str_81-0861_1196161	Listeria_monocytogenes_07PF0776_1126011	0.9727
Listeria_monocytogenes_serotype_4b_str_81-0592_1196160	Listeria_monocytogenes_07PF0776_1126011	0.9738
Listeria_monocytogenes_serotype_4b_str_81-0558_1196159	Listeria_monocytogenes_07PF0776_1126011	0.9738
Listeria_monocytogenes_serotype_4b_str_10-0809_1196162	Listeria_monocytogenes_07PF0776_1126011	0.9742
Listeria_monocytogenes_serotype_4b_str_02-6680_1196175	Listeria_monocytogenes_07PF0776_1126011	0.9773
Listeria_monocytogenes_serotype_4b_str_02-6679_1196174	Listeria_monocytogenes_07PF0776_1126011	0.9773
Listeria_monocytogenes_serotype_4b_str_02-1792_1196173	Listeria_monocytogenes_07PF0776_1126011	0.9742
Listeria_monocytogenes_serotype_4b_str_02-1289_1196172	Listeria_monocytogenes_07PF0776_1126011	0.9742
Listeria_monocytogenes_serotype_4b_str_02-1103_1196171	Listeria_monocytogenes_07PF0776_1126011	0.9742
Listeria_monocytogenes_serotype_3c_str_10-5027_1196195	Listeria_monocytogenes_07PF0776_1126011	0.928
Listeria_monocytogenes_serotype_1_2c_str_10-5026_1196194	Listeria_monocytogenes_07PF0776_1126011	0.9288
Listeria_monocytogenes_serotype_1_2c_str_10-5025_1196193	Listeria_monocytogenes_07PF0776_1126011	0.9288
Listeria_monocytogenes_serotype_1_2b_str_10-0811_1196164	Listeria_monocytogenes_07PF0776_1126011	0.9416
Listeria_monocytogenes_serotype_1_2b_str_10-0810_1196163	Listeria_monocytogenes_07PF0776_1126011	0.9416
Listeria_monocytogenes_serotype_1_2a_str_99-6370_1196179	Listeria_monocytogenes_07PF0776_1126011	0.9155
Listeria_monocytogenes_serotype_1_2a_str_98-2035_1196178	Listeria_monocytogenes_07PF0776_1126011	0.9155
Listeria_monocytogenes_serotype_1_2a_str_95-0093_1196177	Listeria_monocytogenes_07PF0776_1126011	0.921
Listeria_monocytogenes_serotype_1_2a_str_88-0478_1196176	Listeria_monocytogenes_07PF0776_1126011	0.9199
Listeria_monocytogenes_serotype_1_2a_str_10-5024_1196192	Listeria_monocytogenes_07PF0776_1126011	0.921
Listeria_monocytogenes_serotype_1_2a_str_10-4758_1196168	Listeria_monocytogenes_07PF0776_1126011	0.938
Listeria_monocytogenes_serotype_1_2a_str_10-4754_1196167	Listeria_monocytogenes_07PF0776_1126011	0.938
Listeria_monocytogenes_serotype_1_2a_str_10-1321_1196191	Listeria_monocytogenes_07PF0776_1126011	0.921
Listeria_monocytogenes_serotype_1_2a_str_10-1047_1196190	Listeria_monocytogenes_07PF0776_1126011	0.9217
Listeria_monocytogenes_serotype_1_2a_str_10-1046_1196189	Listeria_monocytogenes_07PF0776_1126011	0.9217
Listeria_monocytogenes_serotype_1_2a_str_10-0934_1196170	Listeria_monocytogenes_07PF0776_1126011	0.9461
Listeria_monocytogenes_serotype_1_2a_str_10-0933_1196169	Listeria_monocytogenes_07PF0776_1126011	0.9461
Listeria_monocytogenes_serotype_1_2a_str_10-0815_1196188	Listeria_monocytogenes_07PF0776_1126011	0.9169
Listeria_monocytogenes_serotype_1_2a_str_10-0814_1196187	Listeria_monocytogenes_07PF0776_1126011	0.9214
Listeria_monocytogenes_serotype_1_2a_str_10-0813_1196166	Listeria_monocytogenes_07PF0776_1126011	0.9274
Listeria_monocytogenes_serotype_1_2a_str_10-0812_1196165	Listeria_monocytogenes_07PF0776_1126011	0.9274
Listeria_monocytogenes_serotype_1_2a_str_08-7669_1196186	Listeria_monocytogenes_07PF0776_1126011	0.9304
Listeria_monocytogenes_serotype_1_2a_str_08-7374_1196185	Listeria_monocytogenes_07PF0776_1126011	0.921
Listeria_monocytogenes_serotype_1_2a_str_08-6997_1196184	Listeria_monocytogenes_07PF0776_1126011	0.9169
Listeria_monocytogenes_serotype_1_2a_str_08-6569_1196183	Listeria_monocytogenes_07PF0776_1126011	0.9169
Listeria_monocytogenes_serotype_1_2a_str_08-6056_1196182	Listeria_monocytogenes_07PF0776_1126011	0.9169
Listeria_monocytogenes_serotype_1_2a_str_04-5457_1196181	Listeria_monocytogenes_07PF0776_1126011	0.921
Listeria_monocytogenes_serotype_1_2a_str_02-5993_1196180	Listeria_monocytogenes_07PF0776_1126011	0.921
Listeria_monocytogenes_R479a_1437838	Listeria_monocytogenes_07PF0776_1126011	0.9203
Listeria_monocytogenes_N53-1_1234142	Listeria_monocytogenes_07PF0776_1126011	0.8955
Listeria_monocytogenes_M7_1030009	Listeria_monocytogenes_07PF0776_1126011	0.9099
Listeria_monocytogenes_LO28_393132	Listeria_monocytogenes_07PF0776_1126011	0.9274
Listeria_monocytogenes_L99_563174	Listeria_monocytogenes_07PF0776_1126011	0.9102
Listeria_monocytogenes_L312_882094	Listeria_monocytogenes_07PF0776_1126011	0.9795
Listeria_monocytogenes_J1816_930781	Listeria_monocytogenes_07PF0776_1126011	0.9516
Listeria_monocytogenes_J1-220_930782	Listeria_monocytogenes_07PF0776_1126011	0.9522
Listeria_monocytogenes_J0161_393130	Listeria_monocytogenes_07PF0776_1126011	0.9278
Listeria_monocytogenes_HCC23_552536	Listeria_monocytogenes_07PF0776_1126011	0.9095
Listeria_monocytogenes_FSL_R2-561_393126	Listeria_monocytogenes_07PF0776_1126011	0.9274
Listeria_monocytogenes_FSL_J1-208_393119	Listeria_monocytogenes_07PF0776_1126011	0.921
Listeria_monocytogenes_FSL_J1-175_393118	Listeria_monocytogenes_07PF0776_1126011	0.9556
Listeria_monocytogenes_Finland_1998_393127	Listeria_monocytogenes_07PF0776_1126011	0.9473
Listeria_monocytogenes_EGD-e_169963_v1	Listeria_monocytogenes_07PF0776_1126011	0.9366
Listeria_monocytogenes_EGD-e_169963	Listeria_monocytogenes_07PF0776_1126011	0.937
Listeria_monocytogenes_EGD_1334565	Listeria_monocytogenes_07PF0776_1126011	0.9352
Listeria_monocytogenes_CFSAN002349_1299895	Listeria_monocytogenes_07PF0776_1126011	0.9754
Listeria_monocytogenes_ATCC_19117_882095_v1	Listeria_monocytogenes_07PF0776_1126011	0.9727
Listeria_monocytogenes_ATCC_19117_882095	Listeria_monocytogenes_07PF0776_1126011	0.9723
Listeria_monocytogenes_6179_1288295	Listeria_monocytogenes_07PF0776_1126011	0.9117
Listeria_monocytogenes_1639_v9	Listeria_monocytogenes_07PF0776_1126011	0.926
Listeria_monocytogenes_1639_v99	Listeria_monocytogenes_07PF0776_1126011	0.9524
Listeria_monocytogenes_1639_v98	Listeria_monocytogenes_07PF0776_1126011	0.9247
Listeria_monocytogenes_1639_v97	Listeria_monocytogenes_07PF0776_1126011	0.9657
Listeria_monocytogenes_1639_v96	Listeria_monocytogenes_07PF0776_1126011	0.9517

Listeria_monocytogenes_1639_v95	Listeria_monocytogenes_07PF0776_1126011	0.9517
Listeria_monocytogenes_1639_v94	Listeria_monocytogenes_07PF0776_1126011	0.9521
Listeria_monocytogenes_1639_v93	Listeria_monocytogenes_07PF0776_1126011	0.9262
Listeria_monocytogenes_1639_v92	Listeria_monocytogenes_07PF0776_1126011	0.9521
Listeria_monocytogenes_1639_v91	Listeria_monocytogenes_07PF0776_1126011	0.9705
Listeria_monocytogenes_1639_v90	Listeria_monocytogenes_07PF0776_1126011	0.9247
Listeria_monocytogenes_1639_v8	Listeria_monocytogenes_07PF0776_1126011	0.9097
Listeria_monocytogenes_1639_v89	Listeria_monocytogenes_07PF0776_1126011	0.9641
Listeria_monocytogenes_1639_v88	Listeria_monocytogenes_07PF0776_1126011	0.9268
Listeria_monocytogenes_1639_v87	Listeria_monocytogenes_07PF0776_1126011	0.9229
Listeria_monocytogenes_1639_v86	Listeria_monocytogenes_07PF0776_1126011	0.9225
Listeria_monocytogenes_1639_v85	Listeria_monocytogenes_07PF0776_1126011	0.9724
Listeria_monocytogenes_1639_v84	Listeria_monocytogenes_07PF0776_1126011	0.9319
Listeria_monocytogenes_1639_v83	Listeria_monocytogenes_07PF0776_1126011	0.9612
Listeria_monocytogenes_1639_v82	Listeria_monocytogenes_07PF0776_1126011	0.9609
Listeria_monocytogenes_1639_v81	Listeria_monocytogenes_07PF0776_1126011	0.9271
Listeria_monocytogenes_1639_v80	Listeria_monocytogenes_07PF0776_1126011	0.9467
Listeria_monocytogenes_1639_v7	Listeria_monocytogenes_07PF0776_1126011	0.9097
Listeria_monocytogenes_1639_v79	Listeria_monocytogenes_07PF0776_1126011	0.9254
Listeria_monocytogenes_1639_v78	Listeria_monocytogenes_07PF0776_1126011	0.9264
Listeria_monocytogenes_1639_v77	Listeria_monocytogenes_07PF0776_1126011	0.9307
Listeria_monocytogenes_1639_v76	Listeria_monocytogenes_07PF0776_1126011	0.922
Listeria_monocytogenes_1639_v75	Listeria_monocytogenes_07PF0776_1126011	0.9102
Listeria_monocytogenes_1639_v74	Listeria_monocytogenes_07PF0776_1126011	0.9217
Listeria_monocytogenes_1639_v73	Listeria_monocytogenes_07PF0776_1126011	0.9225
Listeria_monocytogenes_1639_v72	Listeria_monocytogenes_07PF0776_1126011	0.9601
Listeria_monocytogenes_1639_v71	Listeria_monocytogenes_07PF0776_1126011	0.9114
Listeria_monocytogenes_1639_v70	Listeria_monocytogenes_07PF0776_1126011	0.925
Listeria_monocytogenes_1639_v6	Listeria_monocytogenes_07PF0776_1126011	0.9094
Listeria_monocytogenes_1639_v69	Listeria_monocytogenes_07PF0776_1126011	0.9738
Listeria_monocytogenes_1639_v68	Listeria_monocytogenes_07PF0776_1126011	0.932
Listeria_monocytogenes_1639_v67	Listeria_monocytogenes_07PF0776_1126011	0.9465
Listeria_monocytogenes_1639_v66	Listeria_monocytogenes_07PF0776_1126011	0.9347
Listeria_monocytogenes_1639_v65	Listeria_monocytogenes_07PF0776_1126011	0.9246
Listeria_monocytogenes_1639_v64	Listeria_monocytogenes_07PF0776_1126011	0.9284
Listeria_monocytogenes_1639_v63	Listeria_monocytogenes_07PF0776_1126011	0.9422
Listeria_monocytogenes_1639_v62	Listeria_monocytogenes_07PF0776_1126011	0.9229
Listeria_monocytogenes_1639_v61	Listeria_monocytogenes_07PF0776_1126011	0.9238
Listeria_monocytogenes_1639_v60	Listeria_monocytogenes_07PF0776_1126011	0.9297
Listeria_monocytogenes_1639_v5	Listeria_monocytogenes_07PF0776_1126011	0.9071
Listeria_monocytogenes_1639_v59	Listeria_monocytogenes_07PF0776_1126011	0.9616
Listeria_monocytogenes_1639_v58	Listeria_monocytogenes_07PF0776_1126011	0.9677
Listeria_monocytogenes_1639_v57	Listeria_monocytogenes_07PF0776_1126011	0.9485
Listeria_monocytogenes_1639_v56	Listeria_monocytogenes_07PF0776_1126011	0.9616
Listeria_monocytogenes_1639_v55	Listeria_monocytogenes_07PF0776_1126011	0.9442
Listeria_monocytogenes_1639_v54	Listeria_monocytogenes_07PF0776_1126011	0.9265
Listeria_monocytogenes_1639_v53	Listeria_monocytogenes_07PF0776_1126011	0.9312
Listeria_monocytogenes_1639_v52	Listeria_monocytogenes_07PF0776_1126011	0.9583
Listeria_monocytogenes_1639_v51	Listeria_monocytogenes_07PF0776_1126011	0.9554
Listeria_monocytogenes_1639_v50	Listeria_monocytogenes_07PF0776_1126011	0.9203
Listeria_monocytogenes_1639_v4	Listeria_monocytogenes_07PF0776_1126011	0.9204
Listeria_monocytogenes_1639_v49	Listeria_monocytogenes_07PF0776_1126011	0.9727
Listeria_monocytogenes_1639_v48	Listeria_monocytogenes_07PF0776_1126011	0.9232
Listeria_monocytogenes_1639_v47	Listeria_monocytogenes_07PF0776_1126011	0.9207
Listeria_monocytogenes_1639_v46	Listeria_monocytogenes_07PF0776_1126011	0.9313
Listeria_monocytogenes_1639_v45	Listeria_monocytogenes_07PF0776_1126011	0.9118
Listeria_monocytogenes_1639_v44	Listeria_monocytogenes_07PF0776_1126011	0.921
Listeria_monocytogenes_1639_v43	Listeria_monocytogenes_07PF0776_1126011	0.921
Listeria_monocytogenes_1639_v42	Listeria_monocytogenes_07PF0776_1126011	0.9166
Listeria_monocytogenes_1639_v41	Listeria_monocytogenes_07PF0776_1126011	0.9028
Listeria_monocytogenes_1639_v40	Listeria_monocytogenes_07PF0776_1126011	0.9278
Listeria_monocytogenes_1639_v3	Listeria_monocytogenes_07PF0776_1126011	0.9208
Listeria_monocytogenes_1639_v39	Listeria_monocytogenes_07PF0776_1126011	0.9426
Listeria_monocytogenes_1639_v38	Listeria_monocytogenes_07PF0776_1126011	0.9246
Listeria_monocytogenes_1639_v37	Listeria_monocytogenes_07PF0776_1126011	0.9201
Listeria_monocytogenes_1639_v36	Listeria_monocytogenes_07PF0776_1126011	0.9184
Listeria_monocytogenes_1639_v35	Listeria_monocytogenes_07PF0776_1126011	0.9284
Listeria_monocytogenes_1639_v34	Listeria_monocytogenes_07PF0776_1126011	0.9243
Listeria_monocytogenes_1639_v33	Listeria_monocytogenes_07PF0776_1126011	0.9416
Listeria_monocytogenes_1639_v32	Listeria_monocytogenes_07PF0776_1126011	0.9191
Listeria_monocytogenes_1639_v31	Listeria_monocytogenes_07PF0776_1126011	0.9243
Listeria_monocytogenes_1639_v30	Listeria_monocytogenes_07PF0776_1126011	0.9411
Listeria_monocytogenes_1639_v2	Listeria_monocytogenes_07PF0776_1126011	0.9064
Listeria_monocytogenes_1639_v29	Listeria_monocytogenes_07PF0776_1126011	0.9143
Listeria_monocytogenes_1639_v28	Listeria_monocytogenes_07PF0776_1126011	0.9187
Listeria_monocytogenes_1639_v27	Listeria_monocytogenes_07PF0776_1126011	0.9095
Listeria_monocytogenes_1639_v26	Listeria_monocytogenes_07PF0776_1126011	0.9092
Listeria_monocytogenes_1639_v25	Listeria_monocytogenes_07PF0776_1126011	0.9092
Listeria_monocytogenes_1639_v24	Listeria_monocytogenes_07PF0776_1126011	0.9522
Listeria_monocytogenes_1639_v23	Listeria_monocytogenes_07PF0776_1126011	0.9518
Listeria_monocytogenes_1639_v22	Listeria_monocytogenes_07PF0776_1126011	0.9434
Listeria_monocytogenes_1639_v21	Listeria_monocytogenes_07PF0776_1126011	0.9518
Listeria_monocytogenes_1639_v20	Listeria_monocytogenes_07PF0776_1126011	0.9367
Listeria_monocytogenes_1639_v1	Listeria_monocytogenes_07PF0776_1126011	0.9057
Listeria_monocytogenes_1639_v19	Listeria_monocytogenes_07PF0776_1126011	0.9363
Listeria_monocytogenes_1639_v198	Listeria_monocytogenes_07PF0776_1126011	0.8986
Listeria_monocytogenes_1639_v197	Listeria_monocytogenes_07PF0776_1126011	0.9242
Listeria_monocytogenes_1639_v196	Listeria_monocytogenes_07PF0776_1126011	0.9178
Listeria_monocytogenes_1639_v195	Listeria_monocytogenes_07PF0776_1126011	0.9469
Listeria_monocytogenes_1639_v194	Listeria_monocytogenes_07PF0776_1126011	0.945
Listeria_monocytogenes_1639_v193	Listeria_monocytogenes_07PF0776_1126011	0.945

Listeria_monocytogenes_1639_v192	Listeria_monocytogenes_07PF0776_1126011	0.9128
Listeria_monocytogenes_1639_v191	Listeria_monocytogenes_07PF0776_1126011	0.9499
Listeria_monocytogenes_1639_v190	Listeria_monocytogenes_07PF0776_1126011	0.9468
Listeria_monocytogenes_1639_v18	Listeria_monocytogenes_07PF0776_1126011	0.9425
Listeria_monocytogenes_1639_v189	Listeria_monocytogenes_07PF0776_1126011	0.9461
Listeria_monocytogenes_1639_v188	Listeria_monocytogenes_07PF0776_1126011	0.949
Listeria_monocytogenes_1639_v187	Listeria_monocytogenes_07PF0776_1126011	0.9468
Listeria_monocytogenes_1639_v186	Listeria_monocytogenes_07PF0776_1126011	0.9472
Listeria_monocytogenes_1639_v185	Listeria_monocytogenes_07PF0776_1126011	0.9472
Listeria_monocytogenes_1639_v184	Listeria_monocytogenes_07PF0776_1126011	0.946
Listeria_monocytogenes_1639_v183	Listeria_monocytogenes_07PF0776_1126011	0.9472
Listeria_monocytogenes_1639_v182	Listeria_monocytogenes_07PF0776_1126011	0.949
Listeria_monocytogenes_1639_v181	Listeria_monocytogenes_07PF0776_1126011	0.9366
Listeria_monocytogenes_1639_v180	Listeria_monocytogenes_07PF0776_1126011	0.9366
Listeria_monocytogenes_1639_v17	Listeria_monocytogenes_07PF0776_1126011	0.9428
Listeria_monocytogenes_1639_v179	Listeria_monocytogenes_07PF0776_1126011	0.9331
Listeria_monocytogenes_1639_v178	Listeria_monocytogenes_07PF0776_1126011	0.9491
Listeria_monocytogenes_1639_v177	Listeria_monocytogenes_07PF0776_1126011	0.9719
Listeria_monocytogenes_1639_v176	Listeria_monocytogenes_07PF0776_1126011	0.9439
Listeria_monocytogenes_1639_v175	Listeria_monocytogenes_07PF0776_1126011	0.94
Listeria_monocytogenes_1639_v174	Listeria_monocytogenes_07PF0776_1126011	0.9496
Listeria_monocytogenes_1639_v173	Listeria_monocytogenes_07PF0776_1126011	0.9713
Listeria_monocytogenes_1639_v172	Listeria_monocytogenes_07PF0776_1126011	0.9429
Listeria_monocytogenes_1639_v171	Listeria_monocytogenes_07PF0776_1126011	0.932
Listeria_monocytogenes_1639_v170	Listeria_monocytogenes_07PF0776_1126011	0.9402
Listeria_monocytogenes_1639_v16	Listeria_monocytogenes_07PF0776_1126011	0.9074
Listeria_monocytogenes_1639_v169	Listeria_monocytogenes_07PF0776_1126011	0.9426
Listeria_monocytogenes_1639_v168	Listeria_monocytogenes_07PF0776_1126011	0.9431
Listeria_monocytogenes_1639_v167	Listeria_monocytogenes_07PF0776_1126011	0.9348
Listeria_monocytogenes_1639_v166	Listeria_monocytogenes_07PF0776_1126011	0.9435
Listeria_monocytogenes_1639_v165	Listeria_monocytogenes_07PF0776_1126011	0.9423
Listeria_monocytogenes_1639_v164	Listeria_monocytogenes_07PF0776_1126011	0.9903
Listeria_monocytogenes_1639_v163	Listeria_monocytogenes_07PF0776_1126011	0.963
Listeria_monocytogenes_1639_v162	Listeria_monocytogenes_07PF0776_1126011	0.9903
Listeria_monocytogenes_1639_v161	Listeria_monocytogenes_07PF0776_1126011	0.9907
Listeria_monocytogenes_1639_v160	Listeria_monocytogenes_07PF0776_1126011	0.9907
Listeria_monocytogenes_1639_v15	Listeria_monocytogenes_07PF0776_1126011	0.9273
Listeria_monocytogenes_1639_v159	Listeria_monocytogenes_07PF0776_1126011	0.9719
Listeria_monocytogenes_1639_v158	Listeria_monocytogenes_07PF0776_1126011	0.9907
Listeria_monocytogenes_1639_v157	Listeria_monocytogenes_07PF0776_1126011	0.9907
Listeria_monocytogenes_1639_v156	Listeria_monocytogenes_07PF0776_1126011	0.9238
Listeria_monocytogenes_1639_v155	Listeria_monocytogenes_07PF0776_1126011	0.937
Listeria_monocytogenes_1639_v154	Listeria_monocytogenes_07PF0776_1126011	0.9398
Listeria_monocytogenes_1639_v153	Listeria_monocytogenes_07PF0776_1126011	0.9378
Listeria_monocytogenes_1639_v152	Listeria_monocytogenes_07PF0776_1126011	0.9719
Listeria_monocytogenes_1639_v151	Listeria_monocytogenes_07PF0776_1126011	0.9719
Listeria_monocytogenes_1639_v150	Listeria_monocytogenes_07PF0776_1126011	0.9366
Listeria_monocytogenes_1639_v14	Listeria_monocytogenes_07PF0776_1126011	0.9335
Listeria_monocytogenes_1639_v149	Listeria_monocytogenes_07PF0776_1126011	0.9356
Listeria_monocytogenes_1639_v148	Listeria_monocytogenes_07PF0776_1126011	0.9707
Listeria_monocytogenes_1639_v147	Listeria_monocytogenes_07PF0776_1126011	0.9568
Listeria_monocytogenes_1639_v146	Listeria_monocytogenes_07PF0776_1126011	0.9512
Listeria_monocytogenes_1639_v145	Listeria_monocytogenes_07PF0776_1126011	0.9456
Listeria_monocytogenes_1639_v144	Listeria_monocytogenes_07PF0776_1126011	0.9792
Listeria_monocytogenes_1639_v143	Listeria_monocytogenes_07PF0776_1126011	0.9505
Listeria_monocytogenes_1639_v142	Listeria_monocytogenes_07PF0776_1126011	0.9795
Listeria_monocytogenes_1639_v141	Listeria_monocytogenes_07PF0776_1126011	0.9695
Listeria_monocytogenes_1639_v140	Listeria_monocytogenes_07PF0776_1126011	0.9799
Listeria_monocytogenes_1639_v13	Listeria_monocytogenes_07PF0776_1126011	0.927
Listeria_monocytogenes_1639_v139	Listeria_monocytogenes_07PF0776_1126011	0.9707
Listeria_monocytogenes_1639_v138	Listeria_monocytogenes_07PF0776_1126011	0.9811
Listeria_monocytogenes_1639_v137	Listeria_monocytogenes_07PF0776_1126011	0.9807
Listeria_monocytogenes_1639_v136	Listeria_monocytogenes_07PF0776_1126011	0.9377
Listeria_monocytogenes_1639_v135	Listeria_monocytogenes_07PF0776_1126011	0.9219
Listeria_monocytogenes_1639_v134	Listeria_monocytogenes_07PF0776_1126011	0.9842
Listeria_monocytogenes_1639_v133	Listeria_monocytogenes_07PF0776_1126011	0.9378
Listeria_monocytogenes_1639_v132	Listeria_monocytogenes_07PF0776_1126011	0.922
Listeria_monocytogenes_1639_v131	Listeria_monocytogenes_07PF0776_1126011	0.9596
Listeria_monocytogenes_1639_v130	Listeria_monocytogenes_07PF0776_1126011	0.9285
Listeria_monocytogenes_1639_v12	Listeria_monocytogenes_07PF0776_1126011	0.9082
Listeria_monocytogenes_1639_v129	Listeria_monocytogenes_07PF0776_1126011	0.9649
Listeria_monocytogenes_1639_v128	Listeria_monocytogenes_07PF0776_1126011	0.9653
Listeria_monocytogenes_1639_v127	Listeria_monocytogenes_07PF0776_1126011	0.9358
Listeria_monocytogenes_1639_v126	Listeria_monocytogenes_07PF0776_1126011	0.9634
Listeria_monocytogenes_1639_v125	Listeria_monocytogenes_07PF0776_1126011	0.9344
Listeria_monocytogenes_1639_v124	Listeria_monocytogenes_07PF0776_1126011	0.9425
Listeria_monocytogenes_1639_v123	Listeria_monocytogenes_07PF0776_1126011	0.9754
Listeria_monocytogenes_1639_v122	Listeria_monocytogenes_07PF0776_1126011	0.9754
Listeria_monocytogenes_1639_v121	Listeria_monocytogenes_07PF0776_1126011	0.9754
Listeria_monocytogenes_1639_v120	Listeria_monocytogenes_07PF0776_1126011	0.9757
Listeria_monocytogenes_1639_v11	Listeria_monocytogenes_07PF0776_1126011	0.9084
Listeria_monocytogenes_1639_v119	Listeria_monocytogenes_07PF0776_1126011	0.9754
Listeria_monocytogenes_1639_v118	Listeria_monocytogenes_07PF0776_1126011	0.9754
Listeria_monocytogenes_1639_v117	Listeria_monocytogenes_07PF0776_1126011	0.9388
Listeria_monocytogenes_1639_v116	Listeria_monocytogenes_07PF0776_1126011	0.9843
Listeria_monocytogenes_1639_v115	Listeria_monocytogenes_07PF0776_1126011	0.9843
Listeria_monocytogenes_1639_v114	Listeria_monocytogenes_07PF0776_1126011	0.9277
Listeria_monocytogenes_1639_v113	Listeria_monocytogenes_07PF0776_1126011	0.9578
Listeria_monocytogenes_1639_v112	Listeria_monocytogenes_07PF0776_1126011	0.9786
Listeria_monocytogenes_1639_v111	Listeria_monocytogenes_07PF0776_1126011	0.9527
Listeria_monocytogenes_1639_v110	Listeria_monocytogenes_07PF0776_1126011	0.9278

Listeria_monocytogenes_1639_v10	Listeria_monocytogenes_07PF0776_1126011	0.9249
Listeria_monocytogenes_1639_v109	Listeria_monocytogenes_07PF0776_1126011	0.9277
Listeria_monocytogenes_1639_v108	Listeria_monocytogenes_07PF0776_1126011	0.9608
Listeria_monocytogenes_1639_v107	Listeria_monocytogenes_07PF0776_1126011	0.972
Listeria_monocytogenes_1639_v106	Listeria_monocytogenes_07PF0776_1126011	0.972
Listeria_monocytogenes_1639_v105	Listeria_monocytogenes_07PF0776_1126011	0.9724
Listeria_monocytogenes_1639_v104	Listeria_monocytogenes_07PF0776_1126011	0.972
Listeria_monocytogenes_1639_v103	Listeria_monocytogenes_07PF0776_1126011	0.9723
Listeria_monocytogenes_1639_v102	Listeria_monocytogenes_07PF0776_1126011	0.9319
Listeria_monocytogenes_1639_v101	Listeria_monocytogenes_07PF0776_1126011	0.92
Listeria_monocytogenes_1639_v100	Listeria_monocytogenes_07PF0776_1126011	0.9193
Listeria_monocytogenes_1639	Listeria_monocytogenes_07PF0776_1126011	0.9468
Listeria_monocytogenes_10403S_393133	Listeria_monocytogenes_07PF0776_1126011	0.9384
Listeria_monocytogenes_08-5923_637381	Listeria_monocytogenes_07PF0776_1126011	0.921
Listeria_monocytogenes_08-5578_653938	Listeria_monocytogenes_07PF0776_1126011	0.9074
Listeria_monocytogenes_07PF0776_1126011	Listeria_monocytogenes_07PF0776_1126011	0.066
Listeria_welshimeri_1643	Listeria_monocytogenes_08-5578_653938	0.8528
Listeria_welshimeri_serovar_6b_str_SLCC5334_386043	Listeria_monocytogenes_08-5578_653938	0.8525
Listeria_seeligeri_serovar_1_2b_str_SLCC3954_683837	Listeria_monocytogenes_08-5578_653938	0.8583
Listeria_seeligeri_FSL_S4-171_702452	Listeria_monocytogenes_08-5578_653938	0.7994
Listeria_seeligeri_FSL_N1-067_702453	Listeria_monocytogenes_08-5578_653938	0.8273
Listeria_seeligeri_1640_v1	Listeria_monocytogenes_08-5578_653938	0.8559
Listeria_seeligeri_1640	Listeria_monocytogenes_08-5578_653938	0.8531
Listeria_monocytogenes_WSLC1042_1457188	Listeria_monocytogenes_08-5578_653938	0.9033
Listeria_monocytogenes_WSLC1001_1457187	Listeria_monocytogenes_08-5578_653938	0.9381
Listeria_monocytogenes_SLCC7179_879090	Listeria_monocytogenes_08-5578_653938	0.9339
Listeria_monocytogenes_SLCC5850_882096	Listeria_monocytogenes_08-5578_653938	0.9382
Listeria_monocytogenes_SLCC2755_932919	Listeria_monocytogenes_08-5578_653938	0.9128
Listeria_monocytogenes_SLCC2540_879089	Listeria_monocytogenes_08-5578_653938	0.9212
Listeria_monocytogenes_SLCC2479_882020	Listeria_monocytogenes_08-5578_653938	0.9367
Listeria_monocytogenes_SLCC2378_879088	Listeria_monocytogenes_08-5578_653938	0.9043
Listeria_monocytogenes_SLCC2376_882097	Listeria_monocytogenes_08-5578_653938	0.9075
Listeria_monocytogenes_SLCC2372_932920	Listeria_monocytogenes_08-5578_653938	0.9375
Listeria_monocytogenes_serotype_4b_str_LL195_1230340	Listeria_monocytogenes_08-5578_653938	0.9089
Listeria_monocytogenes_serotype_4b_str_LIS0087_1134669	Listeria_monocytogenes_08-5578_653938	0.9202
Listeria_monocytogenes_serotype_4b_str_F2365_265669	Listeria_monocytogenes_08-5578_653938	0.9089
Listeria_monocytogenes_serotype_4b_str_CLIP_80459_568819	Listeria_monocytogenes_08-5578_653938	0.9155
Listeria_monocytogenes_serotype_4b_str_81-0861_1196161	Listeria_monocytogenes_08-5578_653938	0.9009
Listeria_monocytogenes_serotype_4b_str_81-0592_1196160	Listeria_monocytogenes_08-5578_653938	0.902
Listeria_monocytogenes_serotype_4b_str_81-0558_1196159	Listeria_monocytogenes_08-5578_653938	0.9023
Listeria_monocytogenes_serotype_4b_str_10-0809_1196162	Listeria_monocytogenes_08-5578_653938	0.902
Listeria_monocytogenes_serotype_4b_str_02-6680_1196175	Listeria_monocytogenes_08-5578_653938	0.9127
Listeria_monocytogenes_serotype_4b_str_02-6679_1196174	Listeria_monocytogenes_08-5578_653938	0.9127
Listeria_monocytogenes_serotype_4b_str_02-1792_1196173	Listeria_monocytogenes_08-5578_653938	0.9034
Listeria_monocytogenes_serotype_4b_str_02-1289_1196172	Listeria_monocytogenes_08-5578_653938	0.9034
Listeria_monocytogenes_serotype_4b_str_02-1103_1196171	Listeria_monocytogenes_08-5578_653938	0.9034
Listeria_monocytogenes_serotype_3c_str_10-5027_1196195	Listeria_monocytogenes_08-5578_653938	0.9396
Listeria_monocytogenes_serotype_1_2c_str_10-5026_1196194	Listeria_monocytogenes_08-5578_653938	0.94
Listeria_monocytogenes_serotype_1_2c_str_10-5025_1196193	Listeria_monocytogenes_08-5578_653938	0.94
Listeria_monocytogenes_serotype_1_2b_str_10-0811_1196164	Listeria_monocytogenes_08-5578_653938	0.9301
Listeria_monocytogenes_serotype_1_2b_str_10-0810_1196163	Listeria_monocytogenes_08-5578_653938	0.9301
Listeria_monocytogenes_serotype_1_2a_str_99-6370_1196179	Listeria_monocytogenes_08-5578_653938	0.9881
Listeria_monocytogenes_serotype_1_2a_str_98-2035_1196178	Listeria_monocytogenes_08-5578_653938	0.9881
Listeria_monocytogenes_serotype_1_2a_str_95-0093_1196177	Listeria_monocytogenes_08-5578_653938	0.9834
Listeria_monocytogenes_serotype_1_2a_str_88-0478_1196176	Listeria_monocytogenes_08-5578_653938	0.979
Listeria_monocytogenes_serotype_1_2a_str_10-5024_1196192	Listeria_monocytogenes_08-5578_653938	0.9848
Listeria_monocytogenes_serotype_1_2a_str_10-4758_1196168	Listeria_monocytogenes_08-5578_653938	0.9361
Listeria_monocytogenes_serotype_1_2a_str_10-4754_1196167	Listeria_monocytogenes_08-5578_653938	0.9364
Listeria_monocytogenes_serotype_1_2a_str_10-1321_1196191	Listeria_monocytogenes_08-5578_653938	0.9848
Listeria_monocytogenes_serotype_1_2a_str_10-1047_1196190	Listeria_monocytogenes_08-5578_653938	0.9819
Listeria_monocytogenes_serotype_1_2a_str_10-1046_1196189	Listeria_monocytogenes_08-5578_653938	0.9819
Listeria_monocytogenes_serotype_1_2a_str_10-0934_1196170	Listeria_monocytogenes_08-5578_653938	0.9349
Listeria_monocytogenes_serotype_1_2a_str_10-0933_1196169	Listeria_monocytogenes_08-5578_653938	0.9349
Listeria_monocytogenes_serotype_1_2a_str_10-0815_1196188	Listeria_monocytogenes_08-5578_653938	0.9899
Listeria_monocytogenes_serotype_1_2a_str_10-0814_1196187	Listeria_monocytogenes_08-5578_653938	0.9852
Listeria_monocytogenes_serotype_1_2a_str_10-0813_1196166	Listeria_monocytogenes_08-5578_653938	0.9401
Listeria_monocytogenes_serotype_1_2a_str_10-0812_1196165	Listeria_monocytogenes_08-5578_653938	0.9401
Listeria_monocytogenes_serotype_1_2a_str_08-7669_1196186	Listeria_monocytogenes_08-5578_653938	0.9748
Listeria_monocytogenes_serotype_1_2a_str_08-7374_1196185	Listeria_monocytogenes_08-5578_653938	0.9848
Listeria_monocytogenes_serotype_1_2a_str_08-6997_1196184	Listeria_monocytogenes_08-5578_653938	0.9899
Listeria_monocytogenes_serotype_1_2a_str_08-6569_1196183	Listeria_monocytogenes_08-5578_653938	0.9899
Listeria_monocytogenes_serotype_1_2a_str_08-6056_1196182	Listeria_monocytogenes_08-5578_653938	0.9899
Listeria_monocytogenes_serotype_1_2a_str_04-5457_1196181	Listeria_monocytogenes_08-5578_653938	0.9848
Listeria_monocytogenes_serotype_1_2a_str_02-5993_1196180	Listeria_monocytogenes_08-5578_653938	0.9848
Listeria_monocytogenes_R479a_1437838	Listeria_monocytogenes_08-5578_653938	0.9711
Listeria_monocytogenes_N53-1_1234142	Listeria_monocytogenes_08-5578_653938	0.8807
Listeria_monocytogenes_M7_1030009	Listeria_monocytogenes_08-5578_653938	0.8878
Listeria_monocytogenes_LO28_393132	Listeria_monocytogenes_08-5578_653938	0.9299
Listeria_monocytogenes_L99_563174	Listeria_monocytogenes_08-5578_653938	0.8881
Listeria_monocytogenes_L312_882094	Listeria_monocytogenes_08-5578_653938	0.9155
Listeria_monocytogenes_J1816_930781	Listeria_monocytogenes_08-5578_653938	0.9064
Listeria_monocytogenes_J1-220_930782	Listeria_monocytogenes_08-5578_653938	0.9071
Listeria_monocytogenes_J0161_393130	Listeria_monocytogenes_08-5578_653938	0.9288
Listeria_monocytogenes_HCC23_552536	Listeria_monocytogenes_08-5578_653938	0.8874
Listeria_monocytogenes_FSL_R2-561_393126	Listeria_monocytogenes_08-5578_653938	0.9375
Listeria_monocytogenes_FSL_J1-208_393119	Listeria_monocytogenes_08-5578_653938	0.8767
Listeria_monocytogenes_FSL_J1-175_393118	Listeria_monocytogenes_08-5578_653938	0.9191
Listeria_monocytogenes_Finland_1998_393127	Listeria_monocytogenes_08-5578_653938	0.9352
Listeria_monocytogenes_EGD-e_169963_v1	Listeria_monocytogenes_08-5578_653938	0.9292
Listeria_monocytogenes_EGD-e_169963	Listeria_monocytogenes_08-5578_653938	0.9296
Listeria_monocytogenes_EGD_1334565	Listeria_monocytogenes_08-5578_653938	0.9389

Listeria_monocytogenes_CFSAN002349_1299895	Listeria_monocytogenes_08-5578_653938	0.9128
Listeria_monocytogenes_ATCC_19117_882095_v1	Listeria_monocytogenes_08-5578_653938	0.9081
Listeria_monocytogenes_ATCC_19117_882095	Listeria_monocytogenes_08-5578_653938	0.9077
Listeria_monocytogenes_6179_1288295	Listeria_monocytogenes_08-5578_653938	0.9416
Listeria_monocytogenes_1639_v9	Listeria_monocytogenes_08-5578_653938	0.9099
Listeria_monocytogenes_1639_v99	Listeria_monocytogenes_08-5578_653938	0.9144
Listeria_monocytogenes_1639_v98	Listeria_monocytogenes_08-5578_653938	0.9356
Listeria_monocytogenes_1639_v97	Listeria_monocytogenes_08-5578_653938	0.9111
Listeria_monocytogenes_1639_v96	Listeria_monocytogenes_08-5578_653938	0.913
Listeria_monocytogenes_1639_v95	Listeria_monocytogenes_08-5578_653938	0.913
Listeria_monocytogenes_1639_v94	Listeria_monocytogenes_08-5578_653938	0.9137
Listeria_monocytogenes_1639_v93	Listeria_monocytogenes_08-5578_653938	0.936
Listeria_monocytogenes_1639_v92	Listeria_monocytogenes_08-5578_653938	0.9151
Listeria_monocytogenes_1639_v91	Listeria_monocytogenes_08-5578_653938	0.9081
Listeria_monocytogenes_1639_v90	Listeria_monocytogenes_08-5578_653938	0.9356
Listeria_monocytogenes_1639_v8	Listeria_monocytogenes_08-5578_653938	0.9621
Listeria_monocytogenes_1639_v89	Listeria_monocytogenes_08-5578_653938	0.9144
Listeria_monocytogenes_1639_v88	Listeria_monocytogenes_08-5578_653938	0.942
Listeria_monocytogenes_1639_v87	Listeria_monocytogenes_08-5578_653938	0.9346
Listeria_monocytogenes_1639_v86	Listeria_monocytogenes_08-5578_653938	0.9343
Listeria_monocytogenes_1639_v85	Listeria_monocytogenes_08-5578_653938	0.9011
Listeria_monocytogenes_1639_v84	Listeria_monocytogenes_08-5578_653938	0.9426
Listeria_monocytogenes_1639_v83	Listeria_monocytogenes_08-5578_653938	0.9033
Listeria_monocytogenes_1639_v82	Listeria_monocytogenes_08-5578_653938	0.903
Listeria_monocytogenes_1639_v81	Listeria_monocytogenes_08-5578_653938	0.946
Listeria_monocytogenes_1639_v80	Listeria_monocytogenes_08-5578_653938	0.9214
Listeria_monocytogenes_1639_v7	Listeria_monocytogenes_08-5578_653938	0.9621
Listeria_monocytogenes_1639_v79	Listeria_monocytogenes_08-5578_653938	0.9432
Listeria_monocytogenes_1639_v78	Listeria_monocytogenes_08-5578_653938	0.9468
Listeria_monocytogenes_1639_v77	Listeria_monocytogenes_08-5578_653938	0.9338
Listeria_monocytogenes_1639_v76	Listeria_monocytogenes_08-5578_653938	0.9396
Listeria_monocytogenes_1639_v75	Listeria_monocytogenes_08-5578_653938	0.8881
Listeria_monocytogenes_1639_v74	Listeria_monocytogenes_08-5578_653938	0.9295
Listeria_monocytogenes_1639_v73	Listeria_monocytogenes_08-5578_653938	0.9368
Listeria_monocytogenes_1639_v72	Listeria_monocytogenes_08-5578_653938	0.9036
Listeria_monocytogenes_1639_v71	Listeria_monocytogenes_08-5578_653938	0.9304
Listeria_monocytogenes_1639_v70	Listeria_monocytogenes_08-5578_653938	0.9388
Listeria_monocytogenes_1639_v6	Listeria_monocytogenes_08-5578_653938	0.9618
Listeria_monocytogenes_1639_v69	Listeria_monocytogenes_08-5578_653938	0.902
Listeria_monocytogenes_1639_v68	Listeria_monocytogenes_08-5578_653938	0.9223
Listeria_monocytogenes_1639_v67	Listeria_monocytogenes_08-5578_653938	0.9216
Listeria_monocytogenes_1639_v66	Listeria_monocytogenes_08-5578_653938	0.9354
Listeria_monocytogenes_1639_v65	Listeria_monocytogenes_08-5578_653938	0.9616
Listeria_monocytogenes_1639_v64	Listeria_monocytogenes_08-5578_653938	0.9403
Listeria_monocytogenes_1639_v63	Listeria_monocytogenes_08-5578_653938	0.93
Listeria_monocytogenes_1639_v62	Listeria_monocytogenes_08-5578_653938	0.9499
Listeria_monocytogenes_1639_v61	Listeria_monocytogenes_08-5578_653938	0.9623
Listeria_monocytogenes_1639_v60	Listeria_monocytogenes_08-5578_653938	0.9296
Listeria_monocytogenes_1639_v5	Listeria_monocytogenes_08-5578_653938	0.8994
Listeria_monocytogenes_1639_v59	Listeria_monocytogenes_08-5578_653938	0.9084
Listeria_monocytogenes_1639_v58	Listeria_monocytogenes_08-5578_653938	0.904
Listeria_monocytogenes_1639_v57	Listeria_monocytogenes_08-5578_653938	0.9043
Listeria_monocytogenes_1639_v56	Listeria_monocytogenes_08-5578_653938	0.9098
Listeria_monocytogenes_1639_v55	Listeria_monocytogenes_08-5578_653938	0.9086
Listeria_monocytogenes_1639_v54	Listeria_monocytogenes_08-5578_653938	0.9037
Listeria_monocytogenes_1639_v53	Listeria_monocytogenes_08-5578_653938	0.9197
Listeria_monocytogenes_1639_v52	Listeria_monocytogenes_08-5578_653938	0.9101
Listeria_monocytogenes_1639_v51	Listeria_monocytogenes_08-5578_653938	0.906
Listeria_monocytogenes_1639_v50	Listeria_monocytogenes_08-5578_653938	0.9837
Listeria_monocytogenes_1639_v4	Listeria_monocytogenes_08-5578_653938	0.9051
Listeria_monocytogenes_1639_v49	Listeria_monocytogenes_08-5578_653938	0.8995
Listeria_monocytogenes_1639_v48	Listeria_monocytogenes_08-5578_653938	0.9447
Listeria_monocytogenes_1639_v47	Listeria_monocytogenes_08-5578_653938	0.9801
Listeria_monocytogenes_1639_v46	Listeria_monocytogenes_08-5578_653938	0.926
Listeria_monocytogenes_1639_v45	Listeria_monocytogenes_08-5578_653938	0.9953
Listeria_monocytogenes_1639_v44	Listeria_monocytogenes_08-5578_653938	0.9848
Listeria_monocytogenes_1639_v43	Listeria_monocytogenes_08-5578_653938	0.9848
Listeria_monocytogenes_1639_v42	Listeria_monocytogenes_08-5578_653938	0.9263
Listeria_monocytogenes_1639_v41	Listeria_monocytogenes_08-5578_653938	0.9296
Listeria_monocytogenes_1639_v40	Listeria_monocytogenes_08-5578_653938	0.94
Listeria_monocytogenes_1639_v3	Listeria_monocytogenes_08-5578_653938	0.9058
Listeria_monocytogenes_1639_v39	Listeria_monocytogenes_08-5578_653938	0.9209
Listeria_monocytogenes_1639_v38	Listeria_monocytogenes_08-5578_653938	0.9246
Listeria_monocytogenes_1639_v37	Listeria_monocytogenes_08-5578_653938	0.9193
Listeria_monocytogenes_1639_v36	Listeria_monocytogenes_08-5578_653938	0.937
Listeria_monocytogenes_1639_v35	Listeria_monocytogenes_08-5578_653938	0.9363
Listeria_monocytogenes_1639_v34	Listeria_monocytogenes_08-5578_653938	0.9338
Listeria_monocytogenes_1639_v33	Listeria_monocytogenes_08-5578_653938	0.9301
Listeria_monocytogenes_1639_v32	Listeria_monocytogenes_08-5578_653938	0.9273
Listeria_monocytogenes_1639_v31	Listeria_monocytogenes_08-5578_653938	0.9338
Listeria_monocytogenes_1639_v30	Listeria_monocytogenes_08-5578_653938	0.9115
Listeria_monocytogenes_1639_v2	Listeria_monocytogenes_08-5578_653938	0.8977
Listeria_monocytogenes_1639_v29	Listeria_monocytogenes_08-5578_653938	0.9355
Listeria_monocytogenes_1639_v28	Listeria_monocytogenes_08-5578_653938	0.9327
Listeria_monocytogenes_1639_v27	Listeria_monocytogenes_08-5578_653938	0.9251
Listeria_monocytogenes_1639_v26	Listeria_monocytogenes_08-5578_653938	0.9252
Listeria_monocytogenes_1639_v25	Listeria_monocytogenes_08-5578_653938	0.9255
Listeria_monocytogenes_1639_v24	Listeria_monocytogenes_08-5578_653938	0.9056
Listeria_monocytogenes_1639_v23	Listeria_monocytogenes_08-5578_653938	0.9053
Listeria_monocytogenes_1639_v22	Listeria_monocytogenes_08-5578_653938	0.9271
Listeria_monocytogenes_1639_v21	Listeria_monocytogenes_08-5578_653938	0.9049

Listeria_monocytogenes_1639_v20	Listeria_monocytogenes_08-5578_653938	0.9312
Listeria_monocytogenes_1639_v1	Listeria_monocytogenes_08-5578_653938	0.9445
Listeria_monocytogenes_1639_v19	Listeria_monocytogenes_08-5578_653938	0.9305
Listeria_monocytogenes_1639_v198	Listeria_monocytogenes_08-5578_653938	0.8818
Listeria_monocytogenes_1639_v197	Listeria_monocytogenes_08-5578_653938	0.9166
Listeria_monocytogenes_1639_v196	Listeria_monocytogenes_08-5578_653938	0.9222
Listeria_monocytogenes_1639_v195	Listeria_monocytogenes_08-5578_653938	0.9096
Listeria_monocytogenes_1639_v194	Listeria_monocytogenes_08-5578_653938	0.9075
Listeria_monocytogenes_1639_v193	Listeria_monocytogenes_08-5578_653938	0.9079
Listeria_monocytogenes_1639_v192	Listeria_monocytogenes_08-5578_653938	0.8736
Listeria_monocytogenes_1639_v191	Listeria_monocytogenes_08-5578_653938	0.9321
Listeria_monocytogenes_1639_v190	Listeria_monocytogenes_08-5578_653938	0.9352
Listeria_monocytogenes_1639_v18	Listeria_monocytogenes_08-5578_653938	0.9002
Listeria_monocytogenes_1639_v189	Listeria_monocytogenes_08-5578_653938	0.9345
Listeria_monocytogenes_1639_v188	Listeria_monocytogenes_08-5578_653938	0.9302
Listeria_monocytogenes_1639_v187	Listeria_monocytogenes_08-5578_653938	0.9351
Listeria_monocytogenes_1639_v186	Listeria_monocytogenes_08-5578_653938	0.9355
Listeria_monocytogenes_1639_v185	Listeria_monocytogenes_08-5578_653938	0.9355
Listeria_monocytogenes_1639_v184	Listeria_monocytogenes_08-5578_653938	0.9348
Listeria_monocytogenes_1639_v183	Listeria_monocytogenes_08-5578_653938	0.9277
Listeria_monocytogenes_1639_v182	Listeria_monocytogenes_08-5578_653938	0.9298
Listeria_monocytogenes_1639_v181	Listeria_monocytogenes_08-5578_653938	0.8882
Listeria_monocytogenes_1639_v180	Listeria_monocytogenes_08-5578_653938	0.8882
Listeria_monocytogenes_1639_v17	Listeria_monocytogenes_08-5578_653938	0.9005
Listeria_monocytogenes_1639_v179	Listeria_monocytogenes_08-5578_653938	0.9317
Listeria_monocytogenes_1639_v178	Listeria_monocytogenes_08-5578_653938	0.9333
Listeria_monocytogenes_1639_v177	Listeria_monocytogenes_08-5578_653938	0.9124
Listeria_monocytogenes_1639_v176	Listeria_monocytogenes_08-5578_653938	0.9043
Listeria_monocytogenes_1639_v175	Listeria_monocytogenes_08-5578_653938	0.9431
Listeria_monocytogenes_1639_v174	Listeria_monocytogenes_08-5578_653938	0.9345
Listeria_monocytogenes_1639_v173	Listeria_monocytogenes_08-5578_653938	0.9182
Listeria_monocytogenes_1639_v172	Listeria_monocytogenes_08-5578_653938	0.903
Listeria_monocytogenes_1639_v171	Listeria_monocytogenes_08-5578_653938	0.9417
Listeria_monocytogenes_1639_v170	Listeria_monocytogenes_08-5578_653938	0.9404
Listeria_monocytogenes_1639_v16	Listeria_monocytogenes_08-5578_653938	0.9996
Listeria_monocytogenes_1639_v169	Listeria_monocytogenes_08-5578_653938	0.9427
Listeria_monocytogenes_1639_v168	Listeria_monocytogenes_08-5578_653938	0.9275
Listeria_monocytogenes_1639_v167	Listeria_monocytogenes_08-5578_653938	0.9323
Listeria_monocytogenes_1639_v166	Listeria_monocytogenes_08-5578_653938	0.9279
Listeria_monocytogenes_1639_v165	Listeria_monocytogenes_08-5578_653938	0.9424
Listeria_monocytogenes_1639_v164	Listeria_monocytogenes_08-5578_653938	0.9084
Listeria_monocytogenes_1639_v163	Listeria_monocytogenes_08-5578_653938	0.9156
Listeria_monocytogenes_1639_v162	Listeria_monocytogenes_08-5578_653938	0.9084
Listeria_monocytogenes_1639_v161	Listeria_monocytogenes_08-5578_653938	0.9095
Listeria_monocytogenes_1639_v160	Listeria_monocytogenes_08-5578_653938	0.9092
Listeria_monocytogenes_1639_v15	Listeria_monocytogenes_08-5578_653938	0.9137
Listeria_monocytogenes_1639_v159	Listeria_monocytogenes_08-5578_653938	0.9124
Listeria_monocytogenes_1639_v158	Listeria_monocytogenes_08-5578_653938	0.9089
Listeria_monocytogenes_1639_v157	Listeria_monocytogenes_08-5578_653938	0.9089
Listeria_monocytogenes_1639_v156	Listeria_monocytogenes_08-5578_653938	0.9301
Listeria_monocytogenes_1639_v155	Listeria_monocytogenes_08-5578_653938	0.9362
Listeria_monocytogenes_1639_v154	Listeria_monocytogenes_08-5578_653938	0.9598
Listeria_monocytogenes_1639_v153	Listeria_monocytogenes_08-5578_653938	0.9288
Listeria_monocytogenes_1639_v152	Listeria_monocytogenes_08-5578_653938	0.9124
Listeria_monocytogenes_1639_v151	Listeria_monocytogenes_08-5578_653938	0.9124
Listeria_monocytogenes_1639_v150	Listeria_monocytogenes_08-5578_653938	0.9384
Listeria_monocytogenes_1639_v14	Listeria_monocytogenes_08-5578_653938	0.9124
Listeria_monocytogenes_1639_v149	Listeria_monocytogenes_08-5578_653938	0.9393
Listeria_monocytogenes_1639_v148	Listeria_monocytogenes_08-5578_653938	0.9117
Listeria_monocytogenes_1639_v147	Listeria_monocytogenes_08-5578_653938	0.9078
Listeria_monocytogenes_1639_v146	Listeria_monocytogenes_08-5578_653938	0.9316
Listeria_monocytogenes_1639_v145	Listeria_monocytogenes_08-5578_653938	0.927
Listeria_monocytogenes_1639_v144	Listeria_monocytogenes_08-5578_653938	0.9151
Listeria_monocytogenes_1639_v143	Listeria_monocytogenes_08-5578_653938	0.9291
Listeria_monocytogenes_1639_v142	Listeria_monocytogenes_08-5578_653938	0.9151
Listeria_monocytogenes_1639_v141	Listeria_monocytogenes_08-5578_653938	0.9224
Listeria_monocytogenes_1639_v140	Listeria_monocytogenes_08-5578_653938	0.9158
Listeria_monocytogenes_1639_v13	Listeria_monocytogenes_08-5578_653938	0.9137
Listeria_monocytogenes_1639_v139	Listeria_monocytogenes_08-5578_653938	0.91
Listeria_monocytogenes_1639_v138	Listeria_monocytogenes_08-5578_653938	0.9181
Listeria_monocytogenes_1639_v137	Listeria_monocytogenes_08-5578_653938	0.9177
Listeria_monocytogenes_1639_v136	Listeria_monocytogenes_08-5578_653938	0.9372
Listeria_monocytogenes_1639_v135	Listeria_monocytogenes_08-5578_653938	0.8855
Listeria_monocytogenes_1639_v134	Listeria_monocytogenes_08-5578_653938	0.9041
Listeria_monocytogenes_1639_v133	Listeria_monocytogenes_08-5578_653938	0.9352
Listeria_monocytogenes_1639_v132	Listeria_monocytogenes_08-5578_653938	0.888
Listeria_monocytogenes_1639_v131	Listeria_monocytogenes_08-5578_653938	0.9099
Listeria_monocytogenes_1639_v130	Listeria_monocytogenes_08-5578_653938	0.9345
Listeria_monocytogenes_1639_v12	Listeria_monocytogenes_08-5578_653938	0.9377
Listeria_monocytogenes_1639_v129	Listeria_monocytogenes_08-5578_653938	0.9184
Listeria_monocytogenes_1639_v128	Listeria_monocytogenes_08-5578_653938	0.9198
Listeria_monocytogenes_1639_v127	Listeria_monocytogenes_08-5578_653938	0.9277
Listeria_monocytogenes_1639_v126	Listeria_monocytogenes_08-5578_653938	0.9205
Listeria_monocytogenes_1639_v125	Listeria_monocytogenes_08-5578_653938	0.9319
Listeria_monocytogenes_1639_v124	Listeria_monocytogenes_08-5578_653938	0.8918
Listeria_monocytogenes_1639_v123	Listeria_monocytogenes_08-5578_653938	0.9128
Listeria_monocytogenes_1639_v122	Listeria_monocytogenes_08-5578_653938	0.9124
Listeria_monocytogenes_1639_v121	Listeria_monocytogenes_08-5578_653938	0.9124
Listeria_monocytogenes_1639_v120	Listeria_monocytogenes_08-5578_653938	0.9124
Listeria_monocytogenes_1639_v11	Listeria_monocytogenes_08-5578_653938	0.9462
Listeria_monocytogenes_1639_v119	Listeria_monocytogenes_08-5578_653938	0.9124

Listeria_monocytogenes_1639_v118	Listeria_monocytogenes_08-5578_653938	0.9124
Listeria_monocytogenes_1639_v117	Listeria_monocytogenes_08-5578_653938	0.9336
Listeria_monocytogenes_1639_v116	Listeria_monocytogenes_08-5578_653938	0.904
Listeria_monocytogenes_1639_v115	Listeria_monocytogenes_08-5578_653938	0.9043
Listeria_monocytogenes_1639_v114	Listeria_monocytogenes_08-5578_653938	0.9408
Listeria_monocytogenes_1639_v113	Listeria_monocytogenes_08-5578_653938	0.9271
Listeria_monocytogenes_1639_v112	Listeria_monocytogenes_08-5578_653938	0.9117
Listeria_monocytogenes_1639_v111	Listeria_monocytogenes_08-5578_653938	0.9253
Listeria_monocytogenes_1639_v110	Listeria_monocytogenes_08-5578_653938	0.9364
Listeria_monocytogenes_1639_v10	Listeria_monocytogenes_08-5578_653938	0.9096
Listeria_monocytogenes_1639_v109	Listeria_monocytogenes_08-5578_653938	0.9459
Listeria_monocytogenes_1639_v108	Listeria_monocytogenes_08-5578_653938	0.9101
Listeria_monocytogenes_1639_v107	Listeria_monocytogenes_08-5578_653938	0.9067
Listeria_monocytogenes_1639_v106	Listeria_monocytogenes_08-5578_653938	0.9071
Listeria_monocytogenes_1639_v105	Listeria_monocytogenes_08-5578_653938	0.9071
Listeria_monocytogenes_1639_v104	Listeria_monocytogenes_08-5578_653938	0.9071
Listeria_monocytogenes_1639_v103	Listeria_monocytogenes_08-5578_653938	0.9074
Listeria_monocytogenes_1639_v102	Listeria_monocytogenes_08-5578_653938	0.9627
Listeria_monocytogenes_1639_v101	Listeria_monocytogenes_08-5578_653938	0.9704
Listeria_monocytogenes_1639_v100	Listeria_monocytogenes_08-5578_653938	0.9704
Listeria_monocytogenes_1639	Listeria_monocytogenes_08-5578_653938	0.9352
Listeria_monocytogenes_10403S_393133	Listeria_monocytogenes_08-5578_653938	0.9342
Listeria_monocytogenes_08-5923_637381	Listeria_monocytogenes_08-5578_653938	0.9848
Listeria_monocytogenes_08-5578_653938	Listeria_monocytogenes_08-5578_653938	0.0734
Listeria_welshimeri_1643	Listeria_monocytogenes_08-5923_637381	0.8644
Listeria_welshimeri_serovar_6b_str_SLCC5334_386043	Listeria_monocytogenes_08-5923_637381	0.8638
Listeria_seeligeri_serovar_1_2b_str_SLCC3954_683837	Listeria_monocytogenes_08-5923_637381	0.8702
Listeria_seeligeri_FSL_S4-171_702452	Listeria_monocytogenes_08-5923_637381	0.8108
Listeria_seeligeri_FSL_N1-067_702453	Listeria_monocytogenes_08-5923_637381	0.8321
Listeria_seeligeri_1640_v1	Listeria_monocytogenes_08-5923_637381	0.8676
Listeria_seeligeri_1640	Listeria_monocytogenes_08-5923_637381	0.8653
Listeria_monocytogenes_WSLC1042_1457188	Listeria_monocytogenes_08-5923_637381	0.9164
Listeria_monocytogenes_WSLC1001_1457187	Listeria_monocytogenes_08-5923_637381	0.952
Listeria_monocytogenes_SLCC7179_879090	Listeria_monocytogenes_08-5923_637381	0.9486
Listeria_monocytogenes_SLCC5850_882096	Listeria_monocytogenes_08-5923_637381	0.9522
Listeria_monocytogenes_SLCC2755_932919	Listeria_monocytogenes_08-5923_637381	0.9177
Listeria_monocytogenes_SLCC2540_879089	Listeria_monocytogenes_08-5923_637381	0.9349
Listeria_monocytogenes_SLCC2479_882020	Listeria_monocytogenes_08-5923_637381	0.9509
Listeria_monocytogenes_SLCC2378_879088	Listeria_monocytogenes_08-5923_637381	0.9178
Listeria_monocytogenes_SLCC2376_882097	Listeria_monocytogenes_08-5923_637381	0.9216
Listeria_monocytogenes_SLCC2372_932920	Listeria_monocytogenes_08-5923_637381	0.9439
Listeria_monocytogenes_serotype_4b_str_LL195_1230340	Listeria_monocytogenes_08-5923_637381	0.9229
Listeria_monocytogenes_serotype_4b_str_LIS0087_1134669	Listeria_monocytogenes_08-5923_637381	0.9337
Listeria_monocytogenes_serotype_4b_str_F2365_265669	Listeria_monocytogenes_08-5923_637381	0.9229
Listeria_monocytogenes_serotype_4b_str_CLIP_80459_568819	Listeria_monocytogenes_08-5923_637381	0.9296
Listeria_monocytogenes_serotype_4b_str_81-0861_1196161	Listeria_monocytogenes_08-5923_637381	0.9135
Listeria_monocytogenes_serotype_4b_str_81-0592_1196160	Listeria_monocytogenes_08-5923_637381	0.9145
Listeria_monocytogenes_serotype_4b_str_81-0558_1196159	Listeria_monocytogenes_08-5923_637381	0.9152
Listeria_monocytogenes_serotype_4b_str_10-0809_1196162	Listeria_monocytogenes_08-5923_637381	0.9145
Listeria_monocytogenes_serotype_4b_str_02-6680_1196175	Listeria_monocytogenes_08-5923_637381	0.9263
Listeria_monocytogenes_serotype_4b_str_02-6679_1196174	Listeria_monocytogenes_08-5923_637381	0.9263
Listeria_monocytogenes_serotype_4b_str_02-1792_1196173	Listeria_monocytogenes_08-5923_637381	0.9164
Listeria_monocytogenes_serotype_4b_str_02-1289_1196172	Listeria_monocytogenes_08-5923_637381	0.9164
Listeria_monocytogenes_serotype_4b_str_02-1103_1196171	Listeria_monocytogenes_08-5923_637381	0.9164
Listeria_monocytogenes_serotype_3c_str_10-5027_1196195	Listeria_monocytogenes_08-5923_637381	0.9538
Listeria_monocytogenes_serotype_1_2c_str_10-5026_1196194	Listeria_monocytogenes_08-5923_637381	0.9546
Listeria_monocytogenes_serotype_1_2c_str_10-5025_1196193	Listeria_monocytogenes_08-5923_637381	0.9546
Listeria_monocytogenes_serotype_1_2b_str_10-0811_1196164	Listeria_monocytogenes_08-5923_637381	0.9345
Listeria_monocytogenes_serotype_1_2b_str_10-0810_1196163	Listeria_monocytogenes_08-5923_637381	0.9345
Listeria_monocytogenes_serotype_1_2a_str_99-6370_1196179	Listeria_monocytogenes_08-5923_637381	0.9934
Listeria_monocytogenes_serotype_1_2a_str_98-2035_1196178	Listeria_monocytogenes_08-5923_637381	0.9934
Listeria_monocytogenes_serotype_1_2a_str_95-0093_1196177	Listeria_monocytogenes_08-5923_637381	0.9982
Listeria_monocytogenes_serotype_1_2a_str_88-0478_1196176	Listeria_monocytogenes_08-5923_637381	0.9941
Listeria_monocytogenes_serotype_1_2a_str_10-5024_1196192	Listeria_monocytogenes_08-5923_637381	1
Listeria_monocytogenes_serotype_1_2a_str_10-4758_1196168	Listeria_monocytogenes_08-5923_637381	0.9504
Listeria_monocytogenes_serotype_1_2a_str_10-4754_1196167	Listeria_monocytogenes_08-5923_637381	0.9504
Listeria_monocytogenes_serotype_1_2a_str_10-1321_1196191	Listeria_monocytogenes_08-5923_637381	1
Listeria_monocytogenes_serotype_1_2a_str_10-1047_1196190	Listeria_monocytogenes_08-5923_637381	0.9967
Listeria_monocytogenes_serotype_1_2a_str_10-1046_1196189	Listeria_monocytogenes_08-5923_637381	0.9967
Listeria_monocytogenes_serotype_1_2a_str_10-0934_1196170	Listeria_monocytogenes_08-5923_637381	0.9494
Listeria_monocytogenes_serotype_1_2a_str_10-0933_1196169	Listeria_monocytogenes_08-5923_637381	0.9494
Listeria_monocytogenes_serotype_1_2a_str_10-0815_1196188	Listeria_monocytogenes_08-5923_637381	0.9949
Listeria_monocytogenes_serotype_1_2a_str_10-0814_1196187	Listeria_monocytogenes_08-5923_637381	0.9996
Listeria_monocytogenes_serotype_1_2a_str_10-0813_1196166	Listeria_monocytogenes_08-5923_637381	0.954
Listeria_monocytogenes_serotype_1_2a_str_10-0812_1196165	Listeria_monocytogenes_08-5923_637381	0.954
Listeria_monocytogenes_serotype_1_2a_str_08-7669_1196186	Listeria_monocytogenes_08-5923_637381	0.9896
Listeria_monocytogenes_serotype_1_2a_str_08-7374_1196185	Listeria_monocytogenes_08-5923_637381	1
Listeria_monocytogenes_serotype_1_2a_str_08-6997_1196184	Listeria_monocytogenes_08-5923_637381	0.9949
Listeria_monocytogenes_serotype_1_2a_str_08-6569_1196183	Listeria_monocytogenes_08-5923_637381	0.9949
Listeria_monocytogenes_serotype_1_2a_str_08-6056_1196182	Listeria_monocytogenes_08-5923_637381	0.9949
Listeria_monocytogenes_serotype_1_2a_str_04-5457_1196181	Listeria_monocytogenes_08-5923_637381	1
Listeria_monocytogenes_serotype_1_2a_str_02-5993_1196180	Listeria_monocytogenes_08-5923_637381	0.9996
Listeria_monocytogenes_R479a_1437838	Listeria_monocytogenes_08-5923_637381	0.9677
Listeria_monocytogenes_N53-1_1234142	Listeria_monocytogenes_08-5923_637381	0.8937
Listeria_monocytogenes_M7_1030009	Listeria_monocytogenes_08-5923_637381	0.8993
Listeria_monocytogenes_LO28_393132	Listeria_monocytogenes_08-5923_637381	0.944
Listeria_monocytogenes_L99_563174	Listeria_monocytogenes_08-5923_637381	0.8999
Listeria_monocytogenes_L312_882094	Listeria_monocytogenes_08-5923_637381	0.9296
Listeria_monocytogenes_J1816_930781	Listeria_monocytogenes_08-5923_637381	0.92
Listeria_monocytogenes_J1-220_930782	Listeria_monocytogenes_08-5923_637381	0.9196
Listeria_monocytogenes_J0161_393130	Listeria_monocytogenes_08-5923_637381	0.9428

Listeria_monocytogenes_HCC23_552536	Listeria_monocytogenes_08-5923_637381	0.8992
Listeria_monocytogenes_FSL_R2-561_393126	Listeria_monocytogenes_08-5923_637381	0.9513
Listeria_monocytogenes_FSL_J1-208_393119	Listeria_monocytogenes_08-5923_637381	0.8886
Listeria_monocytogenes_FSL_J1-175_393118	Listeria_monocytogenes_08-5923_637381	0.9328
Listeria_monocytogenes_Finland_1998_393127	Listeria_monocytogenes_08-5923_637381	0.9497
Listeria_monocytogenes_EGD-e_169963_v1	Listeria_monocytogenes_08-5923_637381	0.9434
Listeria_monocytogenes_EGD-e_169963	Listeria_monocytogenes_08-5923_637381	0.9434
Listeria_monocytogenes_EGD_1334565	Listeria_monocytogenes_08-5923_637381	0.9529
Listeria_monocytogenes_CFSAN002349_1299895	Listeria_monocytogenes_08-5923_637381	0.9264
Listeria_monocytogenes_ATCC_19117_882095_v1	Listeria_monocytogenes_08-5923_637381	0.9213
Listeria_monocytogenes_ATCC_19117_882095	Listeria_monocytogenes_08-5923_637381	0.9213
Listeria_monocytogenes_6179_1288295	Listeria_monocytogenes_08-5923_637381	0.9425
Listeria_monocytogenes_1639_v9	Listeria_monocytogenes_08-5923_637381	0.9227
Listeria_monocytogenes_1639_v99	Listeria_monocytogenes_08-5923_637381	0.9211
Listeria_monocytogenes_1639_v98	Listeria_monocytogenes_08-5923_637381	0.9409
Listeria_monocytogenes_1639_v97	Listeria_monocytogenes_08-5923_637381	0.9246
Listeria_monocytogenes_1639_v96	Listeria_monocytogenes_08-5923_637381	0.9197
Listeria_monocytogenes_1639_v95	Listeria_monocytogenes_08-5923_637381	0.9198
Listeria_monocytogenes_1639_v94	Listeria_monocytogenes_08-5923_637381	0.9201
Listeria_monocytogenes_1639_v93	Listeria_monocytogenes_08-5923_637381	0.9361
Listeria_monocytogenes_1639_v92	Listeria_monocytogenes_08-5923_637381	0.9215
Listeria_monocytogenes_1639_v91	Listeria_monocytogenes_08-5923_637381	0.9218
Listeria_monocytogenes_1639_v90	Listeria_monocytogenes_08-5923_637381	0.9409
Listeria_monocytogenes_1639_v8	Listeria_monocytogenes_08-5923_637381	0.9572
Listeria_monocytogenes_1639_v89	Listeria_monocytogenes_08-5923_637381	0.9277
Listeria_monocytogenes_1639_v88	Listeria_monocytogenes_08-5923_637381	0.9563
Listeria_monocytogenes_1639_v87	Listeria_monocytogenes_08-5923_637381	0.9491
Listeria_monocytogenes_1639_v86	Listeria_monocytogenes_08-5923_637381	0.9484
Listeria_monocytogenes_1639_v85	Listeria_monocytogenes_08-5923_637381	0.914
Listeria_monocytogenes_1639_v84	Listeria_monocytogenes_08-5923_637381	0.9569
Listeria_monocytogenes_1639_v83	Listeria_monocytogenes_08-5923_637381	0.9159
Listeria_monocytogenes_1639_v82	Listeria_monocytogenes_08-5923_637381	0.9156
Listeria_monocytogenes_1639_v81	Listeria_monocytogenes_08-5923_637381	0.9607
Listeria_monocytogenes_1639_v80	Listeria_monocytogenes_08-5923_637381	0.9354
Listeria_monocytogenes_1639_v7	Listeria_monocytogenes_08-5923_637381	0.9572
Listeria_monocytogenes_1639_v79	Listeria_monocytogenes_08-5923_637381	0.9571
Listeria_monocytogenes_1639_v78	Listeria_monocytogenes_08-5923_637381	0.9615
Listeria_monocytogenes_1639_v77	Listeria_monocytogenes_08-5923_637381	0.9473
Listeria_monocytogenes_1639_v76	Listeria_monocytogenes_08-5923_637381	0.9453
Listeria_monocytogenes_1639_v75	Listeria_monocytogenes_08-5923_637381	0.8999
Listeria_monocytogenes_1639_v74	Listeria_monocytogenes_08-5923_637381	0.9355
Listeria_monocytogenes_1639_v73	Listeria_monocytogenes_08-5923_637381	0.942
Listeria_monocytogenes_1639_v72	Listeria_monocytogenes_08-5923_637381	0.9169
Listeria_monocytogenes_1639_v71	Listeria_monocytogenes_08-5923_637381	0.937
Listeria_monocytogenes_1639_v70	Listeria_monocytogenes_08-5923_637381	0.9518
Listeria_monocytogenes_1639_v6	Listeria_monocytogenes_08-5923_637381	0.9565
Listeria_monocytogenes_1639_v69	Listeria_monocytogenes_08-5923_637381	0.9149
Listeria_monocytogenes_1639_v68	Listeria_monocytogenes_08-5923_637381	0.9355
Listeria_monocytogenes_1639_v67	Listeria_monocytogenes_08-5923_637381	0.9356
Listeria_monocytogenes_1639_v66	Listeria_monocytogenes_08-5923_637381	0.9493
Listeria_monocytogenes_1639_v65	Listeria_monocytogenes_08-5923_637381	0.9672
Listeria_monocytogenes_1639_v64	Listeria_monocytogenes_08-5923_637381	0.9449
Listeria_monocytogenes_1639_v63	Listeria_monocytogenes_08-5923_637381	0.937
Listeria_monocytogenes_1639_v62	Listeria_monocytogenes_08-5923_637381	0.9561
Listeria_monocytogenes_1639_v61	Listeria_monocytogenes_08-5923_637381	0.9772
Listeria_monocytogenes_1639_v60	Listeria_monocytogenes_08-5923_637381	0.9437
Listeria_monocytogenes_1639_v5	Listeria_monocytogenes_08-5923_637381	0.8916
Listeria_monocytogenes_1639_v59	Listeria_monocytogenes_08-5923_637381	0.9218
Listeria_monocytogenes_1639_v58	Listeria_monocytogenes_08-5923_637381	0.917
Listeria_monocytogenes_1639_v57	Listeria_monocytogenes_08-5923_637381	0.9175
Listeria_monocytogenes_1639_v56	Listeria_monocytogenes_08-5923_637381	0.9229
Listeria_monocytogenes_1639_v55	Listeria_monocytogenes_08-5923_637381	0.9149
Listeria_monocytogenes_1639_v54	Listeria_monocytogenes_08-5923_637381	0.9168
Listeria_monocytogenes_1639_v53	Listeria_monocytogenes_08-5923_637381	0.9326
Listeria_monocytogenes_1639_v52	Listeria_monocytogenes_08-5923_637381	0.9235
Listeria_monocytogenes_1639_v51	Listeria_monocytogenes_08-5923_637381	0.9145
Listeria_monocytogenes_1639_v50	Listeria_monocytogenes_08-5923_637381	0.9985
Listeria_monocytogenes_1639_v4	Listeria_monocytogenes_08-5923_637381	0.9022
Listeria_monocytogenes_1639_v49	Listeria_monocytogenes_08-5923_637381	0.9124
Listeria_monocytogenes_1639_v48	Listeria_monocytogenes_08-5923_637381	0.9504
Listeria_monocytogenes_1639_v47	Listeria_monocytogenes_08-5923_637381	0.9952
Listeria_monocytogenes_1639_v46	Listeria_monocytogenes_08-5923_637381	0.9393
Listeria_monocytogenes_1639_v45	Listeria_monocytogenes_08-5923_637381	0.9895
Listeria_monocytogenes_1639_v44	Listeria_monocytogenes_08-5923_637381	1
Listeria_monocytogenes_1639_v43	Listeria_monocytogenes_08-5923_637381	1
Listeria_monocytogenes_1639_v42	Listeria_monocytogenes_08-5923_637381	0.9307
Listeria_monocytogenes_1639_v41	Listeria_monocytogenes_08-5923_637381	0.9282
Listeria_monocytogenes_1639_v40	Listeria_monocytogenes_08-5923_637381	0.9428
Listeria_monocytogenes_1639_v3	Listeria_monocytogenes_08-5923_637381	0.9026
Listeria_monocytogenes_1639_v39	Listeria_monocytogenes_08-5923_637381	0.9344
Listeria_monocytogenes_1639_v38	Listeria_monocytogenes_08-5923_637381	0.9378
Listeria_monocytogenes_1639_v37	Listeria_monocytogenes_08-5923_637381	0.9327
Listeria_monocytogenes_1639_v36	Listeria_monocytogenes_08-5923_637381	0.9343
Listeria_monocytogenes_1639_v35	Listeria_monocytogenes_08-5923_637381	0.942
Listeria_monocytogenes_1639_v34	Listeria_monocytogenes_08-5923_637381	0.9467
Listeria_monocytogenes_1639_v33	Listeria_monocytogenes_08-5923_637381	0.9345
Listeria_monocytogenes_1639_v32	Listeria_monocytogenes_08-5923_637381	0.9408
Listeria_monocytogenes_1639_v31	Listeria_monocytogenes_08-5923_637381	0.9467
Listeria_monocytogenes_1639_v30	Listeria_monocytogenes_08-5923_637381	0.916
Listeria_monocytogenes_1639_v2	Listeria_monocytogenes_08-5923_637381	0.8962
Listeria_monocytogenes_1639_v29	Listeria_monocytogenes_08-5923_637381	0.9404

Listeria_monocytogenes_1639_v28	Listeria_monocytogenes_08-5923_637381	0.9463
Listeria_monocytogenes_1639_v27	Listeria_monocytogenes_08-5923_637381	0.9305
Listeria_monocytogenes_1639_v26	Listeria_monocytogenes_08-5923_637381	0.9306
Listeria_monocytogenes_1639_v25	Listeria_monocytogenes_08-5923_637381	0.9306
Listeria_monocytogenes_1639_v24	Listeria_monocytogenes_08-5923_637381	0.9185
Listeria_monocytogenes_1639_v23	Listeria_monocytogenes_08-5923_637381	0.9185
Listeria_monocytogenes_1639_v22	Listeria_monocytogenes_08-5923_637381	0.9407
Listeria_monocytogenes_1639_v21	Listeria_monocytogenes_08-5923_637381	0.9178
Listeria_monocytogenes_1639_v20	Listeria_monocytogenes_08-5923_637381	0.9364
Listeria_monocytogenes_1639_v1	Listeria_monocytogenes_08-5923_637381	0.9372
Listeria_monocytogenes_1639_v19	Listeria_monocytogenes_08-5923_637381	0.936
Listeria_monocytogenes_1639_v198	Listeria_monocytogenes_08-5923_637381	0.8953
Listeria_monocytogenes_1639_v197	Listeria_monocytogenes_08-5923_637381	0.9304
Listeria_monocytogenes_1639_v196	Listeria_monocytogenes_08-5923_637381	0.9362
Listeria_monocytogenes_1639_v195	Listeria_monocytogenes_08-5923_637381	0.9238
Listeria_monocytogenes_1639_v194	Listeria_monocytogenes_08-5923_637381	0.922
Listeria_monocytogenes_1639_v193	Listeria_monocytogenes_08-5923_637381	0.9224
Listeria_monocytogenes_1639_v192	Listeria_monocytogenes_08-5923_637381	0.8839
Listeria_monocytogenes_1639_v191	Listeria_monocytogenes_08-5923_637381	0.9469
Listeria_monocytogenes_1639_v190	Listeria_monocytogenes_08-5923_637381	0.95
Listeria_monocytogenes_1639_v18	Listeria_monocytogenes_08-5923_637381	0.9133
Listeria_monocytogenes_1639_v189	Listeria_monocytogenes_08-5923_637381	0.9493
Listeria_monocytogenes_1639_v188	Listeria_monocytogenes_08-5923_637381	0.945
Listeria_monocytogenes_1639_v187	Listeria_monocytogenes_08-5923_637381	0.9497
Listeria_monocytogenes_1639_v186	Listeria_monocytogenes_08-5923_637381	0.9504
Listeria_monocytogenes_1639_v185	Listeria_monocytogenes_08-5923_637381	0.95
Listeria_monocytogenes_1639_v184	Listeria_monocytogenes_08-5923_637381	0.9496
Listeria_monocytogenes_1639_v183	Listeria_monocytogenes_08-5923_637381	0.9421
Listeria_monocytogenes_1639_v182	Listeria_monocytogenes_08-5923_637381	0.9443
Listeria_monocytogenes_1639_v181	Listeria_monocytogenes_08-5923_637381	0.9015
Listeria_monocytogenes_1639_v180	Listeria_monocytogenes_08-5923_637381	0.9015
Listeria_monocytogenes_1639_v17	Listeria_monocytogenes_08-5923_637381	0.9133
Listeria_monocytogenes_1639_v179	Listeria_monocytogenes_08-5923_637381	0.9464
Listeria_monocytogenes_1639_v178	Listeria_monocytogenes_08-5923_637381	0.9481
Listeria_monocytogenes_1639_v177	Listeria_monocytogenes_08-5923_637381	0.926
Listeria_monocytogenes_1639_v176	Listeria_monocytogenes_08-5923_637381	0.9183
Listeria_monocytogenes_1639_v175	Listeria_monocytogenes_08-5923_637381	0.958
Listeria_monocytogenes_1639_v174	Listeria_monocytogenes_08-5923_637381	0.9494
Listeria_monocytogenes_1639_v173	Listeria_monocytogenes_08-5923_637381	0.9324
Listeria_monocytogenes_1639_v172	Listeria_monocytogenes_08-5923_637381	0.9169
Listeria_monocytogenes_1639_v171	Listeria_monocytogenes_08-5923_637381	0.9396
Listeria_monocytogenes_1639_v170	Listeria_monocytogenes_08-5923_637381	0.947
Listeria_monocytogenes_1639_v16	Listeria_monocytogenes_08-5923_637381	0.9852
Listeria_monocytogenes_1639_v169	Listeria_monocytogenes_08-5923_637381	0.9572
Listeria_monocytogenes_1639_v168	Listeria_monocytogenes_08-5923_637381	0.9419
Listeria_monocytogenes_1639_v167	Listeria_monocytogenes_08-5923_637381	0.9466
Listeria_monocytogenes_1639_v166	Listeria_monocytogenes_08-5923_637381	0.9422
Listeria_monocytogenes_1639_v165	Listeria_monocytogenes_08-5923_637381	0.9568
Listeria_monocytogenes_1639_v164	Listeria_monocytogenes_08-5923_637381	0.922
Listeria_monocytogenes_1639_v163	Listeria_monocytogenes_08-5923_637381	0.9259
Listeria_monocytogenes_1639_v162	Listeria_monocytogenes_08-5923_637381	0.922
Listeria_monocytogenes_1639_v161	Listeria_monocytogenes_08-5923_637381	0.9232
Listeria_monocytogenes_1639_v160	Listeria_monocytogenes_08-5923_637381	0.9232
Listeria_monocytogenes_1639_v15	Listeria_monocytogenes_08-5923_637381	0.9193
Listeria_monocytogenes_1639_v159	Listeria_monocytogenes_08-5923_637381	0.926
Listeria_monocytogenes_1639_v158	Listeria_monocytogenes_08-5923_637381	0.9229
Listeria_monocytogenes_1639_v157	Listeria_monocytogenes_08-5923_637381	0.9229
Listeria_monocytogenes_1639_v156	Listeria_monocytogenes_08-5923_637381	0.9368
Listeria_monocytogenes_1639_v155	Listeria_monocytogenes_08-5923_637381	0.9509
Listeria_monocytogenes_1639_v154	Listeria_monocytogenes_08-5923_637381	0.9749
Listeria_monocytogenes_1639_v153	Listeria_monocytogenes_08-5923_637381	0.943
Listeria_monocytogenes_1639_v152	Listeria_monocytogenes_08-5923_637381	0.9264
Listeria_monocytogenes_1639_v151	Listeria_monocytogenes_08-5923_637381	0.9264
Listeria_monocytogenes_1639_v150	Listeria_monocytogenes_08-5923_637381	0.9524
Listeria_monocytogenes_1639_v14	Listeria_monocytogenes_08-5923_637381	0.9158
Listeria_monocytogenes_1639_v149	Listeria_monocytogenes_08-5923_637381	0.9529
Listeria_monocytogenes_1639_v148	Listeria_monocytogenes_08-5923_637381	0.9253
Listeria_monocytogenes_1639_v147	Listeria_monocytogenes_08-5923_637381	0.9135
Listeria_monocytogenes_1639_v146	Listeria_monocytogenes_08-5923_637381	0.9464
Listeria_monocytogenes_1639_v145	Listeria_monocytogenes_08-5923_637381	0.9402
Listeria_monocytogenes_1639_v144	Listeria_monocytogenes_08-5923_637381	0.9292
Listeria_monocytogenes_1639_v143	Listeria_monocytogenes_08-5923_637381	0.9435
Listeria_monocytogenes_1639_v142	Listeria_monocytogenes_08-5923_637381	0.9292
Listeria_monocytogenes_1639_v141	Listeria_monocytogenes_08-5923_637381	0.9367
Listeria_monocytogenes_1639_v140	Listeria_monocytogenes_08-5923_637381	0.9296
Listeria_monocytogenes_1639_v13	Listeria_monocytogenes_08-5923_637381	0.9197
Listeria_monocytogenes_1639_v139	Listeria_monocytogenes_08-5923_637381	0.9233
Listeria_monocytogenes_1639_v138	Listeria_monocytogenes_08-5923_637381	0.9319
Listeria_monocytogenes_1639_v137	Listeria_monocytogenes_08-5923_637381	0.9315
Listeria_monocytogenes_1639_v136	Listeria_monocytogenes_08-5923_637381	0.9515
Listeria_monocytogenes_1639_v135	Listeria_monocytogenes_08-5923_637381	0.8975
Listeria_monocytogenes_1639_v134	Listeria_monocytogenes_08-5923_637381	0.9175
Listeria_monocytogenes_1639_v133	Listeria_monocytogenes_08-5923_637381	0.9487
Listeria_monocytogenes_1639_v132	Listeria_monocytogenes_08-5923_637381	0.8988
Listeria_monocytogenes_1639_v131	Listeria_monocytogenes_08-5923_637381	0.9234
Listeria_monocytogenes_1639_v130	Listeria_monocytogenes_08-5923_637381	0.9405
Listeria_monocytogenes_1639_v12	Listeria_monocytogenes_08-5923_637381	0.935
Listeria_monocytogenes_1639_v129	Listeria_monocytogenes_08-5923_637381	0.9322
Listeria_monocytogenes_1639_v128	Listeria_monocytogenes_08-5923_637381	0.9336
Listeria_monocytogenes_1639_v127	Listeria_monocytogenes_08-5923_637381	0.9415
Listeria_monocytogenes_1639_v126	Listeria_monocytogenes_08-5923_637381	0.9351

Listeria_monocytogenes_1639_v125	Listeria_monocytogenes_08-5923_637381	0.9461
Listeria_monocytogenes_1639_v124	Listeria_monocytogenes_08-5923_637381	0.9058
Listeria_monocytogenes_1639_v123	Listeria_monocytogenes_08-5923_637381	0.9264
Listeria_monocytogenes_1639_v122	Listeria_monocytogenes_08-5923_637381	0.9264
Listeria_monocytogenes_1639_v121	Listeria_monocytogenes_08-5923_637381	0.9264
Listeria_monocytogenes_1639_v120	Listeria_monocytogenes_08-5923_637381	0.9261
Listeria_monocytogenes_1639_v11	Listeria_monocytogenes_08-5923_637381	0.9396
Listeria_monocytogenes_1639_v119	Listeria_monocytogenes_08-5923_637381	0.9264
Listeria_monocytogenes_1639_v118	Listeria_monocytogenes_08-5923_637381	0.9264
Listeria_monocytogenes_1639_v117	Listeria_monocytogenes_08-5923_637381	0.9478
Listeria_monocytogenes_1639_v116	Listeria_monocytogenes_08-5923_637381	0.9171
Listeria_monocytogenes_1639_v115	Listeria_monocytogenes_08-5923_637381	0.9174
Listeria_monocytogenes_1639_v114	Listeria_monocytogenes_08-5923_637381	0.9555
Listeria_monocytogenes_1639_v113	Listeria_monocytogenes_08-5923_637381	0.9413
Listeria_monocytogenes_1639_v112	Listeria_monocytogenes_08-5923_637381	0.9245
Listeria_monocytogenes_1639_v111	Listeria_monocytogenes_08-5923_637381	0.9234
Listeria_monocytogenes_1639_v110	Listeria_monocytogenes_08-5923_637381	0.9503
Listeria_monocytogenes_1639_v10	Listeria_monocytogenes_08-5923_637381	0.922
Listeria_monocytogenes_1639_v109	Listeria_monocytogenes_08-5923_637381	0.9603
Listeria_monocytogenes_1639_v108	Listeria_monocytogenes_08-5923_637381	0.9232
Listeria_monocytogenes_1639_v107	Listeria_monocytogenes_08-5923_637381	0.9199
Listeria_monocytogenes_1639_v106	Listeria_monocytogenes_08-5923_637381	0.9203
Listeria_monocytogenes_1639_v105	Listeria_monocytogenes_08-5923_637381	0.9203
Listeria_monocytogenes_1639_v104	Listeria_monocytogenes_08-5923_637381	0.9206
Listeria_monocytogenes_1639_v103	Listeria_monocytogenes_08-5923_637381	0.9206
Listeria_monocytogenes_1639_v102	Listeria_monocytogenes_08-5923_637381	0.9777
Listeria_monocytogenes_1639_v101	Listeria_monocytogenes_08-5923_637381	0.9674
Listeria_monocytogenes_1639_v100	Listeria_monocytogenes_08-5923_637381	0.967
Listeria_monocytogenes_1639	Listeria_monocytogenes_08-5923_637381	0.9497
Listeria_monocytogenes_10403S_393133	Listeria_monocytogenes_08-5923_637381	0.9486
Listeria_monocytogenes_08-5923_637381	Listeria_monocytogenes_08-5923_637381	0.0664
Listeria_welshimeri_1643	Listeria_monocytogenes_10403S_393133	0.8766
Listeria_welshimeri_serovar_6b_str_SLCC5334_386043	Listeria_monocytogenes_10403S_393133	0.8763
Listeria_seeligeri_serovar_1_2b_str_SLCC3954_683837	Listeria_monocytogenes_10403S_393133	0.8881
Listeria_seeligeri_FSL_S4-171_702452	Listeria_monocytogenes_10403S_393133	0.8229
Listeria_seeligeri_FSL_N1-067_702453	Listeria_monocytogenes_10403S_393133	0.8315
Listeria_seeligeri_1640_v1	Listeria_monocytogenes_10403S_393133	0.8861
Listeria_seeligeri_1640	Listeria_monocytogenes_10403S_393133	0.8846
Listeria_monocytogenes_WSLC1042_1457188	Listeria_monocytogenes_10403S_393133	0.9327
Listeria_monocytogenes_WSLC1001_1457187	Listeria_monocytogenes_10403S_393133	0.9737
Listeria_monocytogenes_SLCC7179_879090	Listeria_monocytogenes_10403S_393133	0.9731
Listeria_monocytogenes_SLCC5850_882096	Listeria_monocytogenes_10403S_393133	0.9778
Listeria_monocytogenes_SLCC2755_932919	Listeria_monocytogenes_10403S_393133	0.9371
Listeria_monocytogenes_SLCC2540_879089	Listeria_monocytogenes_10403S_393133	0.938
Listeria_monocytogenes_SLCC2479_882020	Listeria_monocytogenes_10403S_393133	0.9516
Listeria_monocytogenes_SLCC2378_879088	Listeria_monocytogenes_10403S_393133	0.9338
Listeria_monocytogenes_SLCC2376_882097	Listeria_monocytogenes_10403S_393133	0.9474
Listeria_monocytogenes_SLCC2372_932920	Listeria_monocytogenes_10403S_393133	0.9441
Listeria_monocytogenes_serotype_4b_str_LL195_1230340	Listeria_monocytogenes_10403S_393133	0.9399
Listeria_monocytogenes_serotype_4b_str_LIS0087_1134669	Listeria_monocytogenes_10403S_393133	0.9711
Listeria_monocytogenes_serotype_4b_str_F2365_265669	Listeria_monocytogenes_10403S_393133	0.9399
Listeria_monocytogenes_serotype_4b_str_CLIP_80459_568819	Listeria_monocytogenes_10403S_393133	0.9464
Listeria_monocytogenes_serotype_4b_str_81-0861_1196161	Listeria_monocytogenes_10403S_393133	0.9357
Listeria_monocytogenes_serotype_4b_str_81-0592_1196160	Listeria_monocytogenes_10403S_393133	0.9368
Listeria_monocytogenes_serotype_4b_str_81-0558_1196159	Listeria_monocytogenes_10403S_393133	0.9371
Listeria_monocytogenes_serotype_4b_str_10-0809_1196162	Listeria_monocytogenes_10403S_393133	0.9368
Listeria_monocytogenes_serotype_4b_str_02-6680_1196175	Listeria_monocytogenes_10403S_393133	0.9454
Listeria_monocytogenes_serotype_4b_str_02-6679_1196174	Listeria_monocytogenes_10403S_393133	0.9454
Listeria_monocytogenes_serotype_4b_str_02-1792_1196173	Listeria_monocytogenes_10403S_393133	0.936
Listeria_monocytogenes_serotype_4b_str_02-1289_1196172	Listeria_monocytogenes_10403S_393133	0.936
Listeria_monocytogenes_serotype_4b_str_02-1103_1196171	Listeria_monocytogenes_10403S_393133	0.936
Listeria_monocytogenes_serotype_3c_str_10-5027_1196195	Listeria_monocytogenes_10403S_393133	0.9501
Listeria_monocytogenes_serotype_1_2c_str_10-5026_1196194	Listeria_monocytogenes_10403S_393133	0.9512
Listeria_monocytogenes_serotype_1_2c_str_10-5025_1196193	Listeria_monocytogenes_10403S_393133	0.9512
Listeria_monocytogenes_serotype_1_2b_str_10-0811_1196164	Listeria_monocytogenes_10403S_393133	0.9417
Listeria_monocytogenes_serotype_1_2b_str_10-0810_1196163	Listeria_monocytogenes_10403S_393133	0.9417
Listeria_monocytogenes_serotype_1_2a_str_99-6370_1196179	Listeria_monocytogenes_10403S_393133	0.9426
Listeria_monocytogenes_serotype_1_2a_str_98-2035_1196178	Listeria_monocytogenes_10403S_393133	0.9426
Listeria_monocytogenes_serotype_1_2a_str_95-0093_1196177	Listeria_monocytogenes_10403S_393133	0.9486
Listeria_monocytogenes_serotype_1_2a_str_88-0478_1196176	Listeria_monocytogenes_10403S_393133	0.9474
Listeria_monocytogenes_serotype_1_2a_str_10-5024_1196192	Listeria_monocytogenes_10403S_393133	0.9486
Listeria_monocytogenes_serotype_1_2a_str_10-4758_1196168	Listeria_monocytogenes_10403S_393133	0.958
Listeria_monocytogenes_serotype_1_2a_str_10-4754_1196167	Listeria_monocytogenes_10403S_393133	0.9584
Listeria_monocytogenes_serotype_1_2a_str_10-1321_1196191	Listeria_monocytogenes_10403S_393133	0.9486
Listeria_monocytogenes_serotype_1_2a_str_10-1047_1196190	Listeria_monocytogenes_10403S_393133	0.9493
Listeria_monocytogenes_serotype_1_2a_str_10-1046_1196189	Listeria_monocytogenes_10403S_393133	0.9493
Listeria_monocytogenes_serotype_1_2a_str_10-0934_1196170	Listeria_monocytogenes_10403S_393133	0.9776
Listeria_monocytogenes_serotype_1_2a_str_10-0933_1196169	Listeria_monocytogenes_10403S_393133	0.9776
Listeria_monocytogenes_serotype_1_2a_str_10-0815_1196188	Listeria_monocytogenes_10403S_393133	0.944
Listeria_monocytogenes_serotype_1_2a_str_10-0814_1196187	Listeria_monocytogenes_10403S_393133	0.9486
Listeria_monocytogenes_serotype_1_2a_str_10-0813_1196166	Listeria_monocytogenes_10403S_393133	0.9825
Listeria_monocytogenes_serotype_1_2a_str_10-0812_1196165	Listeria_monocytogenes_10403S_393133	0.9825
Listeria_monocytogenes_serotype_1_2a_str_08-7669_1196186	Listeria_monocytogenes_10403S_393133	0.9586
Listeria_monocytogenes_serotype_1_2a_str_08-7374_1196185	Listeria_monocytogenes_10403S_393133	0.9486
Listeria_monocytogenes_serotype_1_2a_str_08-6997_1196184	Listeria_monocytogenes_10403S_393133	0.944
Listeria_monocytogenes_serotype_1_2a_str_08-6569_1196183	Listeria_monocytogenes_10403S_393133	0.944
Listeria_monocytogenes_serotype_1_2a_str_08-6056_1196182	Listeria_monocytogenes_10403S_393133	0.944
Listeria_monocytogenes_serotype_1_2a_str_04-5457_1196181	Listeria_monocytogenes_10403S_393133	0.9486
Listeria_monocytogenes_serotype_1_2a_str_02-5993_1196180	Listeria_monocytogenes_10403S_393133	0.9486
Listeria_monocytogenes_R479a_1437838	Listeria_monocytogenes_10403S_393133	0.9449
Listeria_monocytogenes_N53-1_1234142	Listeria_monocytogenes_10403S_393133	0.9182

Listeria_monocytogenes_M7_1030009	Listeria_monocytogenes_10403S_393133	0.9123
Listeria_monocytogenes_LO28_393132	Listeria_monocytogenes_10403S_393133	0.9562
Listeria_monocytogenes_L99_563174	Listeria_monocytogenes_10403S_393133	0.9126
Listeria_monocytogenes_L312_882094	Listeria_monocytogenes_10403S_393133	0.9464
Listeria_monocytogenes_J1816_930781	Listeria_monocytogenes_10403S_393133	0.9314
Listeria_monocytogenes_J1-220_930782	Listeria_monocytogenes_10403S_393133	0.932
Listeria_monocytogenes_J0161_393130	Listeria_monocytogenes_10403S_393133	0.9596
Listeria_monocytogenes_HCC23_552536	Listeria_monocytogenes_10403S_393133	0.9123
Listeria_monocytogenes_FSL_R2-561_393126	Listeria_monocytogenes_10403S_393133	0.949
Listeria_monocytogenes_FSL_J1-208_393119	Listeria_monocytogenes_10403S_393133	0.912
Listeria_monocytogenes_FSL_J1-175_393118	Listeria_monocytogenes_10403S_393133	0.943
Listeria_monocytogenes_Finland_1998_393127	Listeria_monocytogenes_10403S_393133	0.9656
Listeria_monocytogenes_EGD-e_169963_v1	Listeria_monocytogenes_10403S_393133	0.968
Listeria_monocytogenes_EGD-e_169963	Listeria_monocytogenes_10403S_393133	0.968
Listeria_monocytogenes_EGD_1334565	Listeria_monocytogenes_10403S_393133	0.9786
Listeria_monocytogenes_CFSAN002349_1299895	Listeria_monocytogenes_10403S_393133	0.9454
Listeria_monocytogenes_ATCC_19117_882095_v1	Listeria_monocytogenes_10403S_393133	0.9409
Listeria_monocytogenes_ATCC_19117_882095	Listeria_monocytogenes_10403S_393133	0.9405
Listeria_monocytogenes_6179_1288295	Listeria_monocytogenes_10403S_393133	0.9438
Listeria_monocytogenes_1639_v9	Listeria_monocytogenes_10403S_393133	0.9265
Listeria_monocytogenes_1639_v99	Listeria_monocytogenes_10403S_393133	0.9333
Listeria_monocytogenes_1639_v98	Listeria_monocytogenes_10403S_393133	0.9558
Listeria_monocytogenes_1639_v97	Listeria_monocytogenes_10403S_393133	0.9369
Listeria_monocytogenes_1639_v96	Listeria_monocytogenes_10403S_393133	0.9311
Listeria_monocytogenes_1639_v95	Listeria_monocytogenes_10403S_393133	0.9311
Listeria_monocytogenes_1639_v94	Listeria_monocytogenes_10403S_393133	0.9318
Listeria_monocytogenes_1639_v93	Listeria_monocytogenes_10403S_393133	0.9437
Listeria_monocytogenes_1639_v92	Listeria_monocytogenes_10403S_393133	0.9333
Listeria_monocytogenes_1639_v91	Listeria_monocytogenes_10403S_393133	0.9324
Listeria_monocytogenes_1639_v90	Listeria_monocytogenes_10403S_393133	0.9558
Listeria_monocytogenes_1639_v8	Listeria_monocytogenes_10403S_393133	0.9355
Listeria_monocytogenes_1639_v89	Listeria_monocytogenes_10403S_393133	0.9469
Listeria_monocytogenes_1639_v88	Listeria_monocytogenes_10403S_393133	0.9617
Listeria_monocytogenes_1639_v87	Listeria_monocytogenes_10403S_393133	0.9766
Listeria_monocytogenes_1639_v86	Listeria_monocytogenes_10403S_393133	0.977
Listeria_monocytogenes_1639_v85	Listeria_monocytogenes_10403S_393133	0.935
Listeria_monocytogenes_1639_v84	Listeria_monocytogenes_10403S_393133	0.9548
Listeria_monocytogenes_1639_v83	Listeria_monocytogenes_10403S_393133	0.9416
Listeria_monocytogenes_1639_v82	Listeria_monocytogenes_10403S_393133	0.9413
Listeria_monocytogenes_1639_v81	Listeria_monocytogenes_10403S_393133	0.95
Listeria_monocytogenes_1639_v80	Listeria_monocytogenes_10403S_393133	0.9438
Listeria_monocytogenes_1639_v7	Listeria_monocytogenes_10403S_393133	0.9355
Listeria_monocytogenes_1639_v79	Listeria_monocytogenes_10403S_393133	0.9482
Listeria_monocytogenes_1639_v78	Listeria_monocytogenes_10403S_393133	0.9496
Listeria_monocytogenes_1639_v77	Listeria_monocytogenes_10403S_393133	0.954
Listeria_monocytogenes_1639_v76	Listeria_monocytogenes_10403S_393133	0.9444
Listeria_monocytogenes_1639_v75	Listeria_monocytogenes_10403S_393133	0.9126
Listeria_monocytogenes_1639_v74	Listeria_monocytogenes_10403S_393133	0.9743
Listeria_monocytogenes_1639_v73	Listeria_monocytogenes_10403S_393133	0.952
Listeria_monocytogenes_1639_v72	Listeria_monocytogenes_10403S_393133	0.9389
Listeria_monocytogenes_1639_v71	Listeria_monocytogenes_10403S_393133	0.9363
Listeria_monocytogenes_1639_v70	Listeria_monocytogenes_10403S_393133	0.9473
Listeria_monocytogenes_1639_v6	Listeria_monocytogenes_10403S_393133	0.9351
Listeria_monocytogenes_1639_v69	Listeria_monocytogenes_10403S_393133	0.9368
Listeria_monocytogenes_1639_v68	Listeria_monocytogenes_10403S_393133	0.9591
Listeria_monocytogenes_1639_v67	Listeria_monocytogenes_10403S_393133	0.9397
Listeria_monocytogenes_1639_v66	Listeria_monocytogenes_10403S_393133	0.9554
Listeria_monocytogenes_1639_v65	Listeria_monocytogenes_10403S_393133	0.9567
Listeria_monocytogenes_1639_v64	Listeria_monocytogenes_10403S_393133	0.9474
Listeria_monocytogenes_1639_v63	Listeria_monocytogenes_10403S_393133	0.9314
Listeria_monocytogenes_1639_v62	Listeria_monocytogenes_10403S_393133	0.9472
Listeria_monocytogenes_1639_v61	Listeria_monocytogenes_10403S_393133	0.9514
Listeria_monocytogenes_1639_v60	Listeria_monocytogenes_10403S_393133	0.9553
Listeria_monocytogenes_1639_v5	Listeria_monocytogenes_10403S_393133	0.9062
Listeria_monocytogenes_1639_v59	Listeria_monocytogenes_10403S_393133	0.9325
Listeria_monocytogenes_1639_v58	Listeria_monocytogenes_10403S_393133	0.9404
Listeria_monocytogenes_1639_v57	Listeria_monocytogenes_10403S_393133	0.9265
Listeria_monocytogenes_1639_v56	Listeria_monocytogenes_10403S_393133	0.9359
Listeria_monocytogenes_1639_v55	Listeria_monocytogenes_10403S_393133	0.9305
Listeria_monocytogenes_1639_v54	Listeria_monocytogenes_10403S_393133	0.9293
Listeria_monocytogenes_1639_v53	Listeria_monocytogenes_10403S_393133	0.9488
Listeria_monocytogenes_1639_v52	Listeria_monocytogenes_10403S_393133	0.9395
Listeria_monocytogenes_1639_v51	Listeria_monocytogenes_10403S_393133	0.9378
Listeria_monocytogenes_1639_v50	Listeria_monocytogenes_10403S_393133	0.9471
Listeria_monocytogenes_1639_v4	Listeria_monocytogenes_10403S_393133	0.9195
Listeria_monocytogenes_1639_v49	Listeria_monocytogenes_10403S_393133	0.9247
Listeria_monocytogenes_1639_v48	Listeria_monocytogenes_10403S_393133	0.9459
Listeria_monocytogenes_1639_v47	Listeria_monocytogenes_10403S_393133	0.9482
Listeria_monocytogenes_1639_v46	Listeria_monocytogenes_10403S_393133	0.9508
Listeria_monocytogenes_1639_v45	Listeria_monocytogenes_10403S_393133	0.9387
Listeria_monocytogenes_1639_v44	Listeria_monocytogenes_10403S_393133	0.9486
Listeria_monocytogenes_1639_v43	Listeria_monocytogenes_10403S_393133	0.9486
Listeria_monocytogenes_1639_v42	Listeria_monocytogenes_10403S_393133	0.9403
Listeria_monocytogenes_1639_v41	Listeria_monocytogenes_10403S_393133	0.9351
Listeria_monocytogenes_1639_v40	Listeria_monocytogenes_10403S_393133	0.9483
Listeria_monocytogenes_1639_v3	Listeria_monocytogenes_10403S_393133	0.9199
Listeria_monocytogenes_1639_v39	Listeria_monocytogenes_10403S_393133	0.9348
Listeria_monocytogenes_1639_v38	Listeria_monocytogenes_10403S_393133	0.9447
Listeria_monocytogenes_1639_v37	Listeria_monocytogenes_10403S_393133	0.9405
Listeria_monocytogenes_1639_v36	Listeria_monocytogenes_10403S_393133	0.9432
Listeria_monocytogenes_1639_v35	Listeria_monocytogenes_10403S_393133	0.949

Listeria_monocytogenes_1639_v34	Listeria_monocytogenes_10403S_393133	0.9463
Listeria_monocytogenes_1639_v33	Listeria_monocytogenes_10403S_393133	0.9417
Listeria_monocytogenes_1639_v32	Listeria_monocytogenes_10403S_393133	0.9492
Listeria_monocytogenes_1639_v31	Listeria_monocytogenes_10403S_393133	0.9466
Listeria_monocytogenes_1639_v30	Listeria_monocytogenes_10403S_393133	0.9306
Listeria_monocytogenes_1639_v2	Listeria_monocytogenes_10403S_393133	0.9073
Listeria_monocytogenes_1639_v29	Listeria_monocytogenes_10403S_393133	0.9364
Listeria_monocytogenes_1639_v28	Listeria_monocytogenes_10403S_393133	0.942
Listeria_monocytogenes_1639_v27	Listeria_monocytogenes_10403S_393133	0.9389
Listeria_monocytogenes_1639_v26	Listeria_monocytogenes_10403S_393133	0.9386
Listeria_monocytogenes_1639_v25	Listeria_monocytogenes_10403S_393133	0.9382
Listeria_monocytogenes_1639_v24	Listeria_monocytogenes_10403S_393133	0.932
Listeria_monocytogenes_1639_v23	Listeria_monocytogenes_10403S_393133	0.9313
Listeria_monocytogenes_1639_v22	Listeria_monocytogenes_10403S_393133	0.9424
Listeria_monocytogenes_1639_v21	Listeria_monocytogenes_10403S_393133	0.9335
Listeria_monocytogenes_1639_v20	Listeria_monocytogenes_10403S_393133	0.9312
Listeria_monocytogenes_1639_v1	Listeria_monocytogenes_10403S_393133	0.9387
Listeria_monocytogenes_1639_v19	Listeria_monocytogenes_10403S_393133	0.9308
Listeria_monocytogenes_1639_v198	Listeria_monocytogenes_10403S_393133	0.9192
Listeria_monocytogenes_1639_v197	Listeria_monocytogenes_10403S_393133	0.954
Listeria_monocytogenes_1639_v196	Listeria_monocytogenes_10403S_393133	0.9623
Listeria_monocytogenes_1639_v195	Listeria_monocytogenes_10403S_393133	0.9497
Listeria_monocytogenes_1639_v194	Listeria_monocytogenes_10403S_393133	0.9474
Listeria_monocytogenes_1639_v193	Listeria_monocytogenes_10403S_393133	0.9478
Listeria_monocytogenes_1639_v192	Listeria_monocytogenes_10403S_393133	0.905
Listeria_monocytogenes_1639_v191	Listeria_monocytogenes_10403S_393133	0.9736
Listeria_monocytogenes_1639_v190	Listeria_monocytogenes_10403S_393133	0.9903
Listeria_monocytogenes_1639_v18	Listeria_monocytogenes_10403S_393133	0.9277
Listeria_monocytogenes_1639_v189	Listeria_monocytogenes_10403S_393133	0.9895
Listeria_monocytogenes_1639_v188	Listeria_monocytogenes_10403S_393133	0.9689
Listeria_monocytogenes_1639_v187	Listeria_monocytogenes_10403S_393133	0.9907
Listeria_monocytogenes_1639_v186	Listeria_monocytogenes_10403S_393133	0.9903
Listeria_monocytogenes_1639_v185	Listeria_monocytogenes_10403S_393133	0.9907
Listeria_monocytogenes_1639_v184	Listeria_monocytogenes_10403S_393133	0.9903
Listeria_monocytogenes_1639_v183	Listeria_monocytogenes_10403S_393133	0.9655
Listeria_monocytogenes_1639_v182	Listeria_monocytogenes_10403S_393133	0.9678
Listeria_monocytogenes_1639_v181	Listeria_monocytogenes_10403S_393133	0.9229
Listeria_monocytogenes_1639_v180	Listeria_monocytogenes_10403S_393133	0.9229
Listeria_monocytogenes_1639_v17	Listeria_monocytogenes_10403S_393133	0.928
Listeria_monocytogenes_1639_v179	Listeria_monocytogenes_10403S_393133	0.9609
Listeria_monocytogenes_1639_v178	Listeria_monocytogenes_10403S_393133	0.9709
Listeria_monocytogenes_1639_v177	Listeria_monocytogenes_10403S_393133	0.945
Listeria_monocytogenes_1639_v176	Listeria_monocytogenes_10403S_393133	0.9386
Listeria_monocytogenes_1639_v175	Listeria_monocytogenes_10403S_393133	0.9631
Listeria_monocytogenes_1639_v174	Listeria_monocytogenes_10403S_393133	0.9741
Listeria_monocytogenes_1639_v173	Listeria_monocytogenes_10403S_393133	0.9544
Listeria_monocytogenes_1639_v172	Listeria_monocytogenes_10403S_393133	0.9396
Listeria_monocytogenes_1639_v171	Listeria_monocytogenes_10403S_393133	0.976
Listeria_monocytogenes_1639_v170	Listeria_monocytogenes_10403S_393133	0.9842
Listeria_monocytogenes_1639_v16	Listeria_monocytogenes_10403S_393133	0.9342
Listeria_monocytogenes_1639_v169	Listeria_monocytogenes_10403S_393133	0.9639
Listeria_monocytogenes_1639_v168	Listeria_monocytogenes_10403S_393133	0.9652
Listeria_monocytogenes_1639_v167	Listeria_monocytogenes_10403S_393133	0.9634
Listeria_monocytogenes_1639_v166	Listeria_monocytogenes_10403S_393133	0.9656
Listeria_monocytogenes_1639_v165	Listeria_monocytogenes_10403S_393133	0.9635
Listeria_monocytogenes_1639_v164	Listeria_monocytogenes_10403S_393133	0.9395
Listeria_monocytogenes_1639_v163	Listeria_monocytogenes_10403S_393133	0.9496
Listeria_monocytogenes_1639_v162	Listeria_monocytogenes_10403S_393133	0.9395
Listeria_monocytogenes_1639_v161	Listeria_monocytogenes_10403S_393133	0.9406
Listeria_monocytogenes_1639_v160	Listeria_monocytogenes_10403S_393133	0.9403
Listeria_monocytogenes_1639_v15	Listeria_monocytogenes_10403S_393133	0.9264
Listeria_monocytogenes_1639_v159	Listeria_monocytogenes_10403S_393133	0.9454
Listeria_monocytogenes_1639_v158	Listeria_monocytogenes_10403S_393133	0.9399
Listeria_monocytogenes_1639_v157	Listeria_monocytogenes_10403S_393133	0.9399
Listeria_monocytogenes_1639_v156	Listeria_monocytogenes_10403S_393133	0.9834
Listeria_monocytogenes_1639_v155	Listeria_monocytogenes_10403S_393133	0.9624
Listeria_monocytogenes_1639_v154	Listeria_monocytogenes_10403S_393133	0.9613
Listeria_monocytogenes_1639_v153	Listeria_monocytogenes_10403S_393133	0.9689
Listeria_monocytogenes_1639_v152	Listeria_monocytogenes_10403S_393133	0.9454
Listeria_monocytogenes_1639_v151	Listeria_monocytogenes_10403S_393133	0.9454
Listeria_monocytogenes_1639_v150	Listeria_monocytogenes_10403S_393133	0.9812
Listeria_monocytogenes_1639_v14	Listeria_monocytogenes_10403S_393133	0.9321
Listeria_monocytogenes_1639_v149	Listeria_monocytogenes_10403S_393133	0.979
Listeria_monocytogenes_1639_v148	Listeria_monocytogenes_10403S_393133	0.9447
Listeria_monocytogenes_1639_v147	Listeria_monocytogenes_10403S_393133	0.9304
Listeria_monocytogenes_1639_v146	Listeria_monocytogenes_10403S_393133	0.9668
Listeria_monocytogenes_1639_v145	Listeria_monocytogenes_10403S_393133	0.9638
Listeria_monocytogenes_1639_v144	Listeria_monocytogenes_10403S_393133	0.9457
Listeria_monocytogenes_1639_v143	Listeria_monocytogenes_10403S_393133	0.9664
Listeria_monocytogenes_1639_v142	Listeria_monocytogenes_10403S_393133	0.9461
Listeria_monocytogenes_1639_v141	Listeria_monocytogenes_10403S_393133	0.9533
Listeria_monocytogenes_1639_v140	Listeria_monocytogenes_10403S_393133	0.9468
Listeria_monocytogenes_1639_v13	Listeria_monocytogenes_10403S_393133	0.9264
Listeria_monocytogenes_1639_v139	Listeria_monocytogenes_10403S_393133	0.9449
Listeria_monocytogenes_1639_v138	Listeria_monocytogenes_10403S_393133	0.9491
Listeria_monocytogenes_1639_v137	Listeria_monocytogenes_10403S_393133	0.9487
Listeria_monocytogenes_1639_v136	Listeria_monocytogenes_10403S_393133	0.9569
Listeria_monocytogenes_1639_v135	Listeria_monocytogenes_10403S_393133	0.924
Listeria_monocytogenes_1639_v134	Listeria_monocytogenes_10403S_393133	0.9336
Listeria_monocytogenes_1639_v133	Listeria_monocytogenes_10403S_393133	0.9621
Listeria_monocytogenes_1639_v132	Listeria_monocytogenes_10403S_393133	0.9229

Listeria_monocytogenes_1639_v131	Listeria_monocytogenes_10403S_393133	0.9437
Listeria_monocytogenes_1639_v130	Listeria_monocytogenes_10403S_393133	0.9479
Listeria_monocytogenes_1639_v12	Listeria_monocytogenes_10403S_393133	0.9309
Listeria_monocytogenes_1639_v129	Listeria_monocytogenes_10403S_393133	0.9503
Listeria_monocytogenes_1639_v128	Listeria_monocytogenes_10403S_393133	0.9493
Listeria_monocytogenes_1639_v127	Listeria_monocytogenes_10403S_393133	0.9684
Listeria_monocytogenes_1639_v126	Listeria_monocytogenes_10403S_393133	0.9501
Listeria_monocytogenes_1639_v125	Listeria_monocytogenes_10403S_393133	0.9598
Listeria_monocytogenes_1639_v124	Listeria_monocytogenes_10403S_393133	0.9311
Listeria_monocytogenes_1639_v123	Listeria_monocytogenes_10403S_393133	0.9454
Listeria_monocytogenes_1639_v122	Listeria_monocytogenes_10403S_393133	0.945
Listeria_monocytogenes_1639_v121	Listeria_monocytogenes_10403S_393133	0.945
Listeria_monocytogenes_1639_v120	Listeria_monocytogenes_10403S_393133	0.945
Listeria_monocytogenes_1639_v11	Listeria_monocytogenes_10403S_393133	0.9415
Listeria_monocytogenes_1639_v119	Listeria_monocytogenes_10403S_393133	0.945
Listeria_monocytogenes_1639_v118	Listeria_monocytogenes_10403S_393133	0.945
Listeria_monocytogenes_1639_v117	Listeria_monocytogenes_10403S_393133	0.9569
Listeria_monocytogenes_1639_v116	Listeria_monocytogenes_10403S_393133	0.9342
Listeria_monocytogenes_1639_v115	Listeria_monocytogenes_10403S_393133	0.9338
Listeria_monocytogenes_1639_v114	Listeria_monocytogenes_10403S_393133	0.9768
Listeria_monocytogenes_1639_v113	Listeria_monocytogenes_10403S_393133	0.9445
Listeria_monocytogenes_1639_v112	Listeria_monocytogenes_10403S_393133	0.9356
Listeria_monocytogenes_1639_v111	Listeria_monocytogenes_10403S_393133	0.9418
Listeria_monocytogenes_1639_v110	Listeria_monocytogenes_10403S_393133	0.9871
Listeria_monocytogenes_1639_v10	Listeria_monocytogenes_10403S_393133	0.9158
Listeria_monocytogenes_1639_v109	Listeria_monocytogenes_10403S_393133	0.9742
Listeria_monocytogenes_1639_v108	Listeria_monocytogenes_10403S_393133	0.9438
Listeria_monocytogenes_1639_v107	Listeria_monocytogenes_10403S_393133	0.9394
Listeria_monocytogenes_1639_v106	Listeria_monocytogenes_10403S_393133	0.9398
Listeria_monocytogenes_1639_v105	Listeria_monocytogenes_10403S_393133	0.9398
Listeria_monocytogenes_1639_v104	Listeria_monocytogenes_10403S_393133	0.9398
Listeria_monocytogenes_1639_v103	Listeria_monocytogenes_10403S_393133	0.9402
Listeria_monocytogenes_1639_v102	Listeria_monocytogenes_10403S_393133	0.9567
Listeria_monocytogenes_1639_v101	Listeria_monocytogenes_10403S_393133	0.9445
Listeria_monocytogenes_1639_v100	Listeria_monocytogenes_10403S_393133	0.9438
Listeria_monocytogenes_1639	Listeria_monocytogenes_10403S_393133	0.9899
Listeria_monocytogenes_10403S_393133	Listeria_monocytogenes_10403S_393133	0.0676
Listeria_welshimeri_1643	Listeria_monocytogenes_1639	0.883
Listeria_welshimeri_serovar_6b_str_SLCC5334_386043	Listeria_monocytogenes_1639	0.8827
Listeria_seeligeri_serovar_1_2b_str_SLCC3954_683837	Listeria_monocytogenes_1639	0.895
Listeria_seeligeri_FSL_S4-171_702452	Listeria_monocytogenes_1639	0.8266
Listeria_seeligeri_FSL_N1-067_702453	Listeria_monocytogenes_1639	0.836
Listeria_seeligeri_1640_v1	Listeria_monocytogenes_1639	0.8933
Listeria_seeligeri_1640	Listeria_monocytogenes_1639	0.8837
Listeria_monocytogenes_WSLC1042_1457188	Listeria_monocytogenes_1639	0.9411
Listeria_monocytogenes_WSLC1001_1457187	Listeria_monocytogenes_1639	0.9804
Listeria_monocytogenes_SLCC7179_879090	Listeria_monocytogenes_1639	0.9698
Listeria_monocytogenes_SLCC5850_882096	Listeria_monocytogenes_1639	0.9854
Listeria_monocytogenes_SLCC2755_932919	Listeria_monocytogenes_1639	0.9454
Listeria_monocytogenes_SLCC2540_879089	Listeria_monocytogenes_1639	0.9447
Listeria_monocytogenes_SLCC2479_882020	Listeria_monocytogenes_1639	0.9547
Listeria_monocytogenes_SLCC2378_879088	Listeria_monocytogenes_1639	0.9422
Listeria_monocytogenes_SLCC2376_882097	Listeria_monocytogenes_1639	0.9556
Listeria_monocytogenes_SLCC2372_932920	Listeria_monocytogenes_1639	0.9471
Listeria_monocytogenes_serotype_4b_str_LL195_1230340	Listeria_monocytogenes_1639	0.9484
Listeria_monocytogenes_serotype_4b_str_LIS0087_1134669	Listeria_monocytogenes_1639	0.9611
Listeria_monocytogenes_serotype_4b_str_F2365_265669	Listeria_monocytogenes_1639	0.9484
Listeria_monocytogenes_serotype_4b_str_CLIP_80459_568819	Listeria_monocytogenes_1639	0.9549
Listeria_monocytogenes_serotype_4b_str_81-0861_1196161	Listeria_monocytogenes_1639	0.931
Listeria_monocytogenes_serotype_4b_str_81-0592_1196160	Listeria_monocytogenes_1639	0.9321
Listeria_monocytogenes_serotype_4b_str_81-0558_1196159	Listeria_monocytogenes_1639	0.9328
Listeria_monocytogenes_serotype_4b_str_10-0809_1196162	Listeria_monocytogenes_1639	0.9324
Listeria_monocytogenes_serotype_4b_str_02-6680_1196175	Listeria_monocytogenes_1639	0.9538
Listeria_monocytogenes_serotype_4b_str_02-6679_1196174	Listeria_monocytogenes_1639	0.9538
Listeria_monocytogenes_serotype_4b_str_02-1792_1196173	Listeria_monocytogenes_1639	0.9324
Listeria_monocytogenes_serotype_4b_str_02-1289_1196172	Listeria_monocytogenes_1639	0.9324
Listeria_monocytogenes_serotype_4b_str_02-1103_1196171	Listeria_monocytogenes_1639	0.9324
Listeria_monocytogenes_serotype_3c_str_10-5027_1196195	Listeria_monocytogenes_1639	0.9539
Listeria_monocytogenes_serotype_1_2c_str_10-5026_1196194	Listeria_monocytogenes_1639	0.9547
Listeria_monocytogenes_serotype_1_2c_str_10-5025_1196193	Listeria_monocytogenes_1639	0.9547
Listeria_monocytogenes_serotype_1_2b_str_10-0811_1196164	Listeria_monocytogenes_1639	0.9355
Listeria_monocytogenes_serotype_1_2b_str_10-0810_1196163	Listeria_monocytogenes_1639	0.9355
Listeria_monocytogenes_serotype_1_2a_str_99-6370_1196179	Listeria_monocytogenes_1639	0.9436
Listeria_monocytogenes_serotype_1_2a_str_98-2035_1196178	Listeria_monocytogenes_1639	0.9436
Listeria_monocytogenes_serotype_1_2a_str_95-0093_1196177	Listeria_monocytogenes_1639	0.9497
Listeria_monocytogenes_serotype_1_2a_str_88-0478_1196176	Listeria_monocytogenes_1639	0.9485
Listeria_monocytogenes_serotype_1_2a_str_10-5024_1196192	Listeria_monocytogenes_1639	0.9497
Listeria_monocytogenes_serotype_1_2a_str_10-4758_1196168	Listeria_monocytogenes_1639	0.9634
Listeria_monocytogenes_serotype_1_2a_str_10-4754_1196167	Listeria_monocytogenes_1639	0.9638
Listeria_monocytogenes_serotype_1_2a_str_10-1321_1196191	Listeria_monocytogenes_1639	0.9497
Listeria_monocytogenes_serotype_1_2a_str_10-1047_1196190	Listeria_monocytogenes_1639	0.9504
Listeria_monocytogenes_serotype_1_2a_str_10-1046_1196189	Listeria_monocytogenes_1639	0.9504
Listeria_monocytogenes_serotype_1_2a_str_10-0934_1196170	Listeria_monocytogenes_1639	0.986
Listeria_monocytogenes_serotype_1_2a_str_10-0933_1196169	Listeria_monocytogenes_1639	0.986
Listeria_monocytogenes_serotype_1_2a_str_10-0815_1196188	Listeria_monocytogenes_1639	0.945
Listeria_monocytogenes_serotype_1_2a_str_10-0814_1196187	Listeria_monocytogenes_1639	0.9497
Listeria_monocytogenes_serotype_1_2a_str_10-0813_1196166	Listeria_monocytogenes_1639	0.9774
Listeria_monocytogenes_serotype_1_2a_str_10-0812_1196165	Listeria_monocytogenes_1639	0.9774
Listeria_monocytogenes_serotype_1_2a_str_08-7669_1196186	Listeria_monocytogenes_1639	0.9598
Listeria_monocytogenes_serotype_1_2a_str_08-7374_1196185	Listeria_monocytogenes_1639	0.9497
Listeria_monocytogenes_serotype_1_2a_str_08-6997_1196184	Listeria_monocytogenes_1639	0.945

Listeria_monocytogenes_serotype_1_2a_str_08-6569_1196183	Listeria_monocytogenes_1639	0.945
Listeria_monocytogenes_serotype_1_2a_str_08-6056_1196182	Listeria_monocytogenes_1639	0.945
Listeria_monocytogenes_serotype_1_2a_str_04-5457_1196181	Listeria_monocytogenes_1639	0.9497
Listeria_monocytogenes_serotype_1_2a_str_02-5993_1196180	Listeria_monocytogenes_1639	0.9497
Listeria_monocytogenes_R479a_1437838	Listeria_monocytogenes_1639	0.9486
Listeria_monocytogenes_N53-1_1234142	Listeria_monocytogenes_1639	0.9183
Listeria_monocytogenes_M7_1030009	Listeria_monocytogenes_1639	0.9086
Listeria_monocytogenes_LO28_393132	Listeria_monocytogenes_1639	0.9532
Listeria_monocytogenes_L99_563174	Listeria_monocytogenes_1639	0.9089
Listeria_monocytogenes_L312_882094	Listeria_monocytogenes_1639	0.9549
Listeria_monocytogenes_J1816_930781	Listeria_monocytogenes_1639	0.9342
Listeria_monocytogenes_J1-220_930782	Listeria_monocytogenes_1639	0.9284
Listeria_monocytogenes_J0161_393130	Listeria_monocytogenes_1639	0.9536
Listeria_monocytogenes_HCC23_552536	Listeria_monocytogenes_1639	0.9086
Listeria_monocytogenes_FSL_R2-561_393126	Listeria_monocytogenes_1639	0.9532
Listeria_monocytogenes_FSL_J1-208_393119	Listeria_monocytogenes_1639	0.919
Listeria_monocytogenes_FSL_J1-175_393118	Listeria_monocytogenes_1639	0.9495
Listeria_monocytogenes_Finland_1998_393127	Listeria_monocytogenes_1639	0.9739
Listeria_monocytogenes_EGD-e_169963_v1	Listeria_monocytogenes_1639	0.9631
Listeria_monocytogenes_EGD-e_169963	Listeria_monocytogenes_1639	0.9631
Listeria_monocytogenes_EGD_1334565	Listeria_monocytogenes_1639	0.9861
Listeria_monocytogenes_CFSAN002349_1299895	Listeria_monocytogenes_1639	0.9535
Listeria_monocytogenes_ATCC_19117_882095_v1	Listeria_monocytogenes_1639	0.9485
Listeria_monocytogenes_ATCC_19117_882095	Listeria_monocytogenes_1639	0.9482
Listeria_monocytogenes_6179_1288295	Listeria_monocytogenes_1639	0.9393
Listeria_monocytogenes_1639_v9	Listeria_monocytogenes_1639	0.9203
Listeria_monocytogenes_1639_v99	Listeria_monocytogenes_1639	0.9347
Listeria_monocytogenes_1639_v98	Listeria_monocytogenes_1639	0.9509
Listeria_monocytogenes_1639_v97	Listeria_monocytogenes_1639	0.9418
Listeria_monocytogenes_1639_v96	Listeria_monocytogenes_1639	0.9336
Listeria_monocytogenes_1639_v95	Listeria_monocytogenes_1639	0.9336
Listeria_monocytogenes_1639_v94	Listeria_monocytogenes_1639	0.9343
Listeria_monocytogenes_1639_v93	Listeria_monocytogenes_1639	0.9493
Listeria_monocytogenes_1639_v92	Listeria_monocytogenes_1639	0.9343
Listeria_monocytogenes_1639_v91	Listeria_monocytogenes_1639	0.9296
Listeria_monocytogenes_1639_v90	Listeria_monocytogenes_1639	0.9509
Listeria_monocytogenes_1639_v8	Listeria_monocytogenes_1639	0.938
Listeria_monocytogenes_1639_v89	Listeria_monocytogenes_1639	0.945
Listeria_monocytogenes_1639_v88	Listeria_monocytogenes_1639	0.9653
Listeria_monocytogenes_1639_v87	Listeria_monocytogenes_1639	0.9719
Listeria_monocytogenes_1639_v86	Listeria_monocytogenes_1639	0.9719
Listeria_monocytogenes_1639_v85	Listeria_monocytogenes_1639	0.9303
Listeria_monocytogenes_1639_v84	Listeria_monocytogenes_1639	0.9575
Listeria_monocytogenes_1639_v83	Listeria_monocytogenes_1639	0.9373
Listeria_monocytogenes_1639_v82	Listeria_monocytogenes_1639	0.937
Listeria_monocytogenes_1639_v81	Listeria_monocytogenes_1639	0.953
Listeria_monocytogenes_1639_v80	Listeria_monocytogenes_1639	0.9399
Listeria_monocytogenes_1639_v7	Listeria_monocytogenes_1639	0.938
Listeria_monocytogenes_1639_v79	Listeria_monocytogenes_1639	0.9512
Listeria_monocytogenes_1639_v78	Listeria_monocytogenes_1639	0.9523
Listeria_monocytogenes_1639_v77	Listeria_monocytogenes_1639	0.9578
Listeria_monocytogenes_1639_v76	Listeria_monocytogenes_1639	0.9469
Listeria_monocytogenes_1639_v75	Listeria_monocytogenes_1639	0.9089
Listeria_monocytogenes_1639_v74	Listeria_monocytogenes_1639	0.9715
Listeria_monocytogenes_1639_v73	Listeria_monocytogenes_1639	0.9482
Listeria_monocytogenes_1639_v72	Listeria_monocytogenes_1639	0.9357
Listeria_monocytogenes_1639_v71	Listeria_monocytogenes_1639	0.9392
Listeria_monocytogenes_1639_v70	Listeria_monocytogenes_1639	0.9507
Listeria_monocytogenes_1639_v6	Listeria_monocytogenes_1639	0.9376
Listeria_monocytogenes_1639_v69	Listeria_monocytogenes_1639	0.9321
Listeria_monocytogenes_1639_v68	Listeria_monocytogenes_1639	0.9568
Listeria_monocytogenes_1639_v67	Listeria_monocytogenes_1639	0.9412
Listeria_monocytogenes_1639_v66	Listeria_monocytogenes_1639	0.962
Listeria_monocytogenes_1639_v65	Listeria_monocytogenes_1639	0.9522
Listeria_monocytogenes_1639_v64	Listeria_monocytogenes_1639	0.9542
Listeria_monocytogenes_1639_v63	Listeria_monocytogenes_1639	0.9366
Listeria_monocytogenes_1639_v62	Listeria_monocytogenes_1639	0.9487
Listeria_monocytogenes_1639_v61	Listeria_monocytogenes_1639	0.9522
Listeria_monocytogenes_1639_v60	Listeria_monocytogenes_1639	0.9561
Listeria_monocytogenes_1639_v5	Listeria_monocytogenes_1639	0.9007
Listeria_monocytogenes_1639_v59	Listeria_monocytogenes_1639	0.9377
Listeria_monocytogenes_1639_v58	Listeria_monocytogenes_1639	0.9377
Listeria_monocytogenes_1639_v57	Listeria_monocytogenes_1639	0.9301
Listeria_monocytogenes_1639_v56	Listeria_monocytogenes_1639	0.938
Listeria_monocytogenes_1639_v55	Listeria_monocytogenes_1639	0.9266
Listeria_monocytogenes_1639_v54	Listeria_monocytogenes_1639	0.9334
Listeria_monocytogenes_1639_v53	Listeria_monocytogenes_1639	0.9553
Listeria_monocytogenes_1639_v52	Listeria_monocytogenes_1639	0.9348
Listeria_monocytogenes_1639_v51	Listeria_monocytogenes_1639	0.9323
Listeria_monocytogenes_1639_v50	Listeria_monocytogenes_1639	0.9482
Listeria_monocytogenes_1639_v4	Listeria_monocytogenes_1639	0.914
Listeria_monocytogenes_1639_v49	Listeria_monocytogenes_1639	0.9317
Listeria_monocytogenes_1639_v48	Listeria_monocytogenes_1639	0.9489
Listeria_monocytogenes_1639_v47	Listeria_monocytogenes_1639	0.9493
Listeria_monocytogenes_1639_v46	Listeria_monocytogenes_1639	0.9561
Listeria_monocytogenes_1639_v45	Listeria_monocytogenes_1639	0.9397
Listeria_monocytogenes_1639_v44	Listeria_monocytogenes_1639	0.9497
Listeria_monocytogenes_1639_v43	Listeria_monocytogenes_1639	0.9497
Listeria_monocytogenes_1639_v42	Listeria_monocytogenes_1639	0.9413
Listeria_monocytogenes_1639_v41	Listeria_monocytogenes_1639	0.9316
Listeria_monocytogenes_1639_v40	Listeria_monocytogenes_1639	0.9536

Listeria_monocytogenes_1639_v3	Listeria_monocytogenes_1639	0.9144
Listeria_monocytogenes_1639_v39	Listeria_monocytogenes_1639	0.9365
Listeria_monocytogenes_1639_v38	Listeria_monocytogenes_1639	0.9507
Listeria_monocytogenes_1639_v37	Listeria_monocytogenes_1639	0.9468
Listeria_monocytogenes_1639_v36	Listeria_monocytogenes_1639	0.9492
Listeria_monocytogenes_1639_v35	Listeria_monocytogenes_1639	0.9543
Listeria_monocytogenes_1639_v34	Listeria_monocytogenes_1639	0.9481
Listeria_monocytogenes_1639_v33	Listeria_monocytogenes_1639	0.9355
Listeria_monocytogenes_1639_v32	Listeria_monocytogenes_1639	0.9454
Listeria_monocytogenes_1639_v31	Listeria_monocytogenes_1639	0.9485
Listeria_monocytogenes_1639_v30	Listeria_monocytogenes_1639	0.9369
Listeria_monocytogenes_1639_v2	Listeria_monocytogenes_1639	0.9033
Listeria_monocytogenes_1639_v29	Listeria_monocytogenes_1639	0.94
Listeria_monocytogenes_1639_v28	Listeria_monocytogenes_1639	0.9446
Listeria_monocytogenes_1639_v27	Listeria_monocytogenes_1639	0.9351
Listeria_monocytogenes_1639_v26	Listeria_monocytogenes_1639	0.9344
Listeria_monocytogenes_1639_v25	Listeria_monocytogenes_1639	0.9344
Listeria_monocytogenes_1639_v24	Listeria_monocytogenes_1639	0.9284
Listeria_monocytogenes_1639_v23	Listeria_monocytogenes_1639	0.9281
Listeria_monocytogenes_1639_v22	Listeria_monocytogenes_1639	0.9366
Listeria_monocytogenes_1639_v21	Listeria_monocytogenes_1639	0.9285
Listeria_monocytogenes_1639_v20	Listeria_monocytogenes_1639	0.9329
Listeria_monocytogenes_1639_v1	Listeria_monocytogenes_1639	0.9327
Listeria_monocytogenes_1639_v19	Listeria_monocytogenes_1639	0.9325
Listeria_monocytogenes_1639_v198	Listeria_monocytogenes_1639	0.9201
Listeria_monocytogenes_1639_v197	Listeria_monocytogenes_1639	0.9491
Listeria_monocytogenes_1639_v196	Listeria_monocytogenes_1639	0.9694
Listeria_monocytogenes_1639_v195	Listeria_monocytogenes_1639	0.9579
Listeria_monocytogenes_1639_v194	Listeria_monocytogenes_1639	0.9556
Listeria_monocytogenes_1639_v193	Listeria_monocytogenes_1639	0.956
Listeria_monocytogenes_1639_v192	Listeria_monocytogenes_1639	0.9119
Listeria_monocytogenes_1639_v191	Listeria_monocytogenes_1639	0.982
Listeria_monocytogenes_1639_v190	Listeria_monocytogenes_1639	0.9988
Listeria_monocytogenes_1639_v18	Listeria_monocytogenes_1639	0.9241
Listeria_monocytogenes_1639_v189	Listeria_monocytogenes_1639	0.9984
Listeria_monocytogenes_1639_v188	Listeria_monocytogenes_1639	0.9769
Listeria_monocytogenes_1639_v187	Listeria_monocytogenes_1639	0.9988
Listeria_monocytogenes_1639_v186	Listeria_monocytogenes_1639	0.9992
Listeria_monocytogenes_1639_v185	Listeria_monocytogenes_1639	0.9992
Listeria_monocytogenes_1639_v184	Listeria_monocytogenes_1639	0.998
Listeria_monocytogenes_1639_v183	Listeria_monocytogenes_1639	0.9738
Listeria_monocytogenes_1639_v182	Listeria_monocytogenes_1639	0.9761
Listeria_monocytogenes_1639_v181	Listeria_monocytogenes_1639	0.9308
Listeria_monocytogenes_1639_v180	Listeria_monocytogenes_1639	0.9308
Listeria_monocytogenes_1639_v17	Listeria_monocytogenes_1639	0.9245
Listeria_monocytogenes_1639_v179	Listeria_monocytogenes_1639	0.9656
Listeria_monocytogenes_1639_v178	Listeria_monocytogenes_1639	0.9789
Listeria_monocytogenes_1639_v177	Listeria_monocytogenes_1639	0.9531
Listeria_monocytogenes_1639_v176	Listeria_monocytogenes_1639	0.9463
Listeria_monocytogenes_1639_v175	Listeria_monocytogenes_1639	0.969
Listeria_monocytogenes_1639_v174	Listeria_monocytogenes_1639	0.9824
Listeria_monocytogenes_1639_v173	Listeria_monocytogenes_1639	0.9626
Listeria_monocytogenes_1639_v172	Listeria_monocytogenes_1639	0.9468
Listeria_monocytogenes_1639_v171	Listeria_monocytogenes_1639	0.9827
Listeria_monocytogenes_1639_v170	Listeria_monocytogenes_1639	0.9911
Listeria_monocytogenes_1639_v16	Listeria_monocytogenes_1639	0.9352
Listeria_monocytogenes_1639_v169	Listeria_monocytogenes_1639	0.9709
Listeria_monocytogenes_1639_v168	Listeria_monocytogenes_1639	0.9735
Listeria_monocytogenes_1639_v167	Listeria_monocytogenes_1639	0.967
Listeria_monocytogenes_1639_v166	Listeria_monocytogenes_1639	0.9739
Listeria_monocytogenes_1639_v165	Listeria_monocytogenes_1639	0.9706
Listeria_monocytogenes_1639_v164	Listeria_monocytogenes_1639	0.9479
Listeria_monocytogenes_1639_v163	Listeria_monocytogenes_1639	0.9581
Listeria_monocytogenes_1639_v162	Listeria_monocytogenes_1639	0.9479
Listeria_monocytogenes_1639_v161	Listeria_monocytogenes_1639	0.9491
Listeria_monocytogenes_1639_v160	Listeria_monocytogenes_1639	0.9487
Listeria_monocytogenes_1639_v15	Listeria_monocytogenes_1639	0.9202
Listeria_monocytogenes_1639_v159	Listeria_monocytogenes_1639	0.9535
Listeria_monocytogenes_1639_v158	Listeria_monocytogenes_1639	0.9484
Listeria_monocytogenes_1639_v157	Listeria_monocytogenes_1639	0.9484
Listeria_monocytogenes_1639_v156	Listeria_monocytogenes_1639	0.9741
Listeria_monocytogenes_1639_v155	Listeria_monocytogenes_1639	0.9645
Listeria_monocytogenes_1639_v154	Listeria_monocytogenes_1639	0.9687
Listeria_monocytogenes_1639_v153	Listeria_monocytogenes_1639	0.9652
Listeria_monocytogenes_1639_v152	Listeria_monocytogenes_1639	0.9535
Listeria_monocytogenes_1639_v151	Listeria_monocytogenes_1639	0.9535
Listeria_monocytogenes_1639_v150	Listeria_monocytogenes_1639	0.9872
Listeria_monocytogenes_1639_v14	Listeria_monocytogenes_1639	0.9267
Listeria_monocytogenes_1639_v149	Listeria_monocytogenes_1639	0.9865
Listeria_monocytogenes_1639_v148	Listeria_monocytogenes_1639	0.9527
Listeria_monocytogenes_1639_v147	Listeria_monocytogenes_1639	0.9382
Listeria_monocytogenes_1639_v146	Listeria_monocytogenes_1639	0.9751
Listeria_monocytogenes_1639_v145	Listeria_monocytogenes_1639	0.9716
Listeria_monocytogenes_1639_v144	Listeria_monocytogenes_1639	0.9542
Listeria_monocytogenes_1639_v143	Listeria_monocytogenes_1639	0.9739
Listeria_monocytogenes_1639_v142	Listeria_monocytogenes_1639	0.9545
Listeria_monocytogenes_1639_v141	Listeria_monocytogenes_1639	0.9603
Listeria_monocytogenes_1639_v140	Listeria_monocytogenes_1639	0.9553
Listeria_monocytogenes_1639_v13	Listeria_monocytogenes_1639	0.9202
Listeria_monocytogenes_1639_v139	Listeria_monocytogenes_1639	0.953
Listeria_monocytogenes_1639_v138	Listeria_monocytogenes_1639	0.9577

Listeria_monocytogenes_1639_v137	Listeria_monocytogenes_1639	0.9573
Listeria_monocytogenes_1639_v136	Listeria_monocytogenes_1639	0.9631
Listeria_monocytogenes_1639_v135	Listeria_monocytogenes_1639	0.9211
Listeria_monocytogenes_1639_v134	Listeria_monocytogenes_1639	0.942
Listeria_monocytogenes_1639_v133	Listeria_monocytogenes_1639	0.9664
Listeria_monocytogenes_1639_v132	Listeria_monocytogenes_1639	0.9211
Listeria_monocytogenes_1639_v131	Listeria_monocytogenes_1639	0.9421
Listeria_monocytogenes_1639_v130	Listeria_monocytogenes_1639	0.9543
Listeria_monocytogenes_1639_v12	Listeria_monocytogenes_1639	0.9334
Listeria_monocytogenes_1639_v129	Listeria_monocytogenes_1639	0.9584
Listeria_monocytogenes_1639_v128	Listeria_monocytogenes_1639	0.9562
Listeria_monocytogenes_1639_v127	Listeria_monocytogenes_1639	0.9643
Listeria_monocytogenes_1639_v126	Listeria_monocytogenes_1639	0.957
Listeria_monocytogenes_1639_v125	Listeria_monocytogenes_1639	0.9641
Listeria_monocytogenes_1639_v124	Listeria_monocytogenes_1639	0.9391
Listeria_monocytogenes_1639_v123	Listeria_monocytogenes_1639	0.9535
Listeria_monocytogenes_1639_v122	Listeria_monocytogenes_1639	0.9531
Listeria_monocytogenes_1639_v121	Listeria_monocytogenes_1639	0.9531
Listeria_monocytogenes_1639_v120	Listeria_monocytogenes_1639	0.9531
Listeria_monocytogenes_1639_v11	Listeria_monocytogenes_1639	0.9355
Listeria_monocytogenes_1639_v119	Listeria_monocytogenes_1639	0.9531
Listeria_monocytogenes_1639_v118	Listeria_monocytogenes_1639	0.9531
Listeria_monocytogenes_1639_v117	Listeria_monocytogenes_1639	0.9623
Listeria_monocytogenes_1639_v116	Listeria_monocytogenes_1639	0.9426
Listeria_monocytogenes_1639_v115	Listeria_monocytogenes_1639	0.9422
Listeria_monocytogenes_1639_v114	Listeria_monocytogenes_1639	0.9782
Listeria_monocytogenes_1639_v113	Listeria_monocytogenes_1639	0.9518
Listeria_monocytogenes_1639_v112	Listeria_monocytogenes_1639	0.9382
Listeria_monocytogenes_1639_v111	Listeria_monocytogenes_1639	0.9486
Listeria_monocytogenes_1639_v110	Listeria_monocytogenes_1639	0.9778
Listeria_monocytogenes_1639_v10	Listeria_monocytogenes_1639	0.9192
Listeria_monocytogenes_1639_v109	Listeria_monocytogenes_1639	0.9778
Listeria_monocytogenes_1639_v108	Listeria_monocytogenes_1639	0.9422
Listeria_monocytogenes_1639_v107	Listeria_monocytogenes_1639	0.9471
Listeria_monocytogenes_1639_v106	Listeria_monocytogenes_1639	0.9474
Listeria_monocytogenes_1639_v105	Listeria_monocytogenes_1639	0.9474
Listeria_monocytogenes_1639_v104	Listeria_monocytogenes_1639	0.9474
Listeria_monocytogenes_1639_v103	Listeria_monocytogenes_1639	0.9478
Listeria_monocytogenes_1639_v102	Listeria_monocytogenes_1639	0.9606
Listeria_monocytogenes_1639_v101	Listeria_monocytogenes_1639	0.9482
Listeria_monocytogenes_1639_v100	Listeria_monocytogenes_1639	0.9475
Listeria_monocytogenes_1639	Listeria_monocytogenes_1639	0.0676
Listeria_welshimeri_1643	Listeria_monocytogenes_1639_v100	0.8632
Listeria_welshimeri_serovar_6b_str_SLCC5334_386043	Listeria_monocytogenes_1639_v100	0.8629
Listeria_seeligeri_serovar_1_2b_str_SLCC3954_683837	Listeria_monocytogenes_1639_v100	0.8682
Listeria_seeligeri_FSL_S4-171_702452	Listeria_monocytogenes_1639_v100	0.8093
Listeria_seeligeri_FSL_N1-067_702453	Listeria_monocytogenes_1639_v100	0.8369
Listeria_seeligeri_1640_v1	Listeria_monocytogenes_1639_v100	0.8668
Listeria_seeligeri_1640	Listeria_monocytogenes_1639_v100	0.8633
Listeria_monocytogenes_WSLC1042_1457188	Listeria_monocytogenes_1639_v100	0.9147
Listeria_monocytogenes_WSLC1001_1457187	Listeria_monocytogenes_1639_v100	0.9488
Listeria_monocytogenes_SLCC7179_879090	Listeria_monocytogenes_1639_v100	0.9435
Listeria_monocytogenes_SLCC5850_882096	Listeria_monocytogenes_1639_v100	0.9486
Listeria_monocytogenes_SLCC2755_932919	Listeria_monocytogenes_1639_v100	0.922
Listeria_monocytogenes_SLCC2540_879089	Listeria_monocytogenes_1639_v100	0.9321
Listeria_monocytogenes_SLCC2479_882020	Listeria_monocytogenes_1639_v100	0.9513
Listeria_monocytogenes_SLCC2378_879088	Listeria_monocytogenes_1639_v100	0.9157
Listeria_monocytogenes_SLCC2376_882097	Listeria_monocytogenes_1639_v100	0.9203
Listeria_monocytogenes_SLCC2372_932920	Listeria_monocytogenes_1639_v100	0.951
Listeria_monocytogenes_serotype_4b_str_LL195_1230340	Listeria_monocytogenes_1639_v100	0.9208
Listeria_monocytogenes_serotype_4b_str_LIS0087_1134669	Listeria_monocytogenes_1639_v100	0.9285
Listeria_monocytogenes_serotype_4b_str_F2365_265669	Listeria_monocytogenes_1639_v100	0.9208
Listeria_monocytogenes_serotype_4b_str_CLIP_80459_568819	Listeria_monocytogenes_1639_v100	0.9275
Listeria_monocytogenes_serotype_4b_str_81-0861_1196161	Listeria_monocytogenes_1639_v100	0.9107
Listeria_monocytogenes_serotype_4b_str_81-0592_1196160	Listeria_monocytogenes_1639_v100	0.9117
Listeria_monocytogenes_serotype_4b_str_81-0558_1196159	Listeria_monocytogenes_1639_v100	0.9121
Listeria_monocytogenes_serotype_4b_str_10-0809_1196162	Listeria_monocytogenes_1639_v100	0.9117
Listeria_monocytogenes_serotype_4b_str_02-6680_1196175	Listeria_monocytogenes_1639_v100	0.9242
Listeria_monocytogenes_serotype_4b_str_02-6679_1196174	Listeria_monocytogenes_1639_v100	0.9242
Listeria_monocytogenes_serotype_4b_str_02-1792_1196173	Listeria_monocytogenes_1639_v100	0.9125
Listeria_monocytogenes_serotype_4b_str_02-1289_1196172	Listeria_monocytogenes_1639_v100	0.9125
Listeria_monocytogenes_serotype_4b_str_02-1103_1196171	Listeria_monocytogenes_1639_v100	0.9125
Listeria_monocytogenes_serotype_3c_str_10-5027_1196195	Listeria_monocytogenes_1639_v100	0.9558
Listeria_monocytogenes_serotype_1_2c_str_10-5026_1196194	Listeria_monocytogenes_1639_v100	0.9569
Listeria_monocytogenes_serotype_1_2c_str_10-5025_1196193	Listeria_monocytogenes_1639_v100	0.9569
Listeria_monocytogenes_serotype_1_2b_str_10-0811_1196164	Listeria_monocytogenes_1639_v100	0.9325
Listeria_monocytogenes_serotype_1_2b_str_10-0810_1196163	Listeria_monocytogenes_1639_v100	0.9325
Listeria_monocytogenes_serotype_1_2a_str_99-6370_1196179	Listeria_monocytogenes_1639_v100	0.9614
Listeria_monocytogenes_serotype_1_2a_str_98-2035_1196178	Listeria_monocytogenes_1639_v100	0.9614
Listeria_monocytogenes_serotype_1_2a_str_95-0093_1196177	Listeria_monocytogenes_1639_v100	0.9674
Listeria_monocytogenes_serotype_1_2a_str_88-0478_1196176	Listeria_monocytogenes_1639_v100	0.967
Listeria_monocytogenes_serotype_1_2a_str_10-5024_1196192	Listeria_monocytogenes_1639_v100	0.967
Listeria_monocytogenes_serotype_1_2a_str_10-4758_1196168	Listeria_monocytogenes_1639_v100	0.9472
Listeria_monocytogenes_serotype_1_2a_str_10-4754_1196167	Listeria_monocytogenes_1639_v100	0.9475
Listeria_monocytogenes_serotype_1_2a_str_10-1321_1196191	Listeria_monocytogenes_1639_v100	0.967
Listeria_monocytogenes_serotype_1_2a_str_10-1047_1196190	Listeria_monocytogenes_1639_v100	0.97
Listeria_monocytogenes_serotype_1_2a_str_10-1046_1196189	Listeria_monocytogenes_1639_v100	0.97
Listeria_monocytogenes_serotype_1_2a_str_10-0934_1196170	Listeria_monocytogenes_1639_v100	0.9476
Listeria_monocytogenes_serotype_1_2a_str_10-0933_1196169	Listeria_monocytogenes_1639_v100	0.9476
Listeria_monocytogenes_serotype_1_2a_str_10-0815_1196188	Listeria_monocytogenes_1639_v100	0.9625
Listeria_monocytogenes_serotype_1_2a_str_10-0814_1196187	Listeria_monocytogenes_1639_v100	0.967

Listeria_monocytogenes_serotype_1_2a_str_10-0813_1196166	Listeria_monocytogenes_1639_v100	0.9515
Listeria_monocytogenes_serotype_1_2a_str_10-0812_1196165	Listeria_monocytogenes_1639_v100	0.9515
Listeria_monocytogenes_serotype_1_2a_str_08-7669_1196186	Listeria_monocytogenes_1639_v100	0.9612
Listeria_monocytogenes_serotype_1_2a_str_08-7374_1196185	Listeria_monocytogenes_1639_v100	0.967
Listeria_monocytogenes_serotype_1_2a_str_08-6997_1196184	Listeria_monocytogenes_1639_v100	0.9625
Listeria_monocytogenes_serotype_1_2a_str_08-6569_1196183	Listeria_monocytogenes_1639_v100	0.9625
Listeria_monocytogenes_serotype_1_2a_str_08-6056_1196182	Listeria_monocytogenes_1639_v100	0.9625
Listeria_monocytogenes_serotype_1_2a_str_04-5457_1196181	Listeria_monocytogenes_1639_v100	0.967
Listeria_monocytogenes_serotype_1_2a_str_02-5993_1196180	Listeria_monocytogenes_1639_v100	0.967
Listeria_monocytogenes_R479a_1437838	Listeria_monocytogenes_1639_v100	0.9971
Listeria_monocytogenes_N53-1_1234142	Listeria_monocytogenes_1639_v100	0.8907
Listeria_monocytogenes_M7_1030009	Listeria_monocytogenes_1639_v100	0.8954
Listeria_monocytogenes_LO28_393132	Listeria_monocytogenes_1639_v100	0.9466
Listeria_monocytogenes_L99_563174	Listeria_monocytogenes_1639_v100	0.8958
Listeria_monocytogenes_L312_882094	Listeria_monocytogenes_1639_v100	0.9275
Listeria_monocytogenes_J1816_930781	Listeria_monocytogenes_1639_v100	0.9169
Listeria_monocytogenes_J1-220_930782	Listeria_monocytogenes_1639_v100	0.9157
Listeria_monocytogenes_J0161_393130	Listeria_monocytogenes_1639_v100	0.9385
Listeria_monocytogenes_HCC23_552536	Listeria_monocytogenes_1639_v100	0.8951
Listeria_monocytogenes_FSL_R2-561_393126	Listeria_monocytogenes_1639_v100	0.9525
Listeria_monocytogenes_FSL_J1-208_393119	Listeria_monocytogenes_1639_v100	0.8874
Listeria_monocytogenes_FSL_J1-175_393118	Listeria_monocytogenes_1639_v100	0.9299
Listeria_monocytogenes_Finland_1998_393127	Listeria_monocytogenes_1639_v100	0.9479
Listeria_monocytogenes_EGD-e_169963_v1	Listeria_monocytogenes_1639_v100	0.9387
Listeria_monocytogenes_EGD-e_169963	Listeria_monocytogenes_1639_v100	0.9387
Listeria_monocytogenes_EGD_1334565	Listeria_monocytogenes_1639_v100	0.9493
Listeria_monocytogenes_CFSAN002349_1299895	Listeria_monocytogenes_1639_v100	0.9251
Listeria_monocytogenes_ATCC_19117_882095_v1	Listeria_monocytogenes_1639_v100	0.9196
Listeria_monocytogenes_ATCC_19117_882095	Listeria_monocytogenes_1639_v100	0.9192
Listeria_monocytogenes_6179_1288295	Listeria_monocytogenes_1639_v100	0.9506
Listeria_monocytogenes_1639_v9	Listeria_monocytogenes_1639_v100	0.9178
Listeria_monocytogenes_1639_v99	Listeria_monocytogenes_1639_v100	0.9239
Listeria_monocytogenes_1639_v98	Listeria_monocytogenes_1639_v100	0.9355
Listeria_monocytogenes_1639_v97	Listeria_monocytogenes_1639_v100	0.9214
Listeria_monocytogenes_1639_v96	Listeria_monocytogenes_1639_v100	0.9236
Listeria_monocytogenes_1639_v95	Listeria_monocytogenes_1639_v100	0.9236
Listeria_monocytogenes_1639_v94	Listeria_monocytogenes_1639_v100	0.9243
Listeria_monocytogenes_1639_v93	Listeria_monocytogenes_1639_v100	0.9483
Listeria_monocytogenes_1639_v92	Listeria_monocytogenes_1639_v100	0.9236
Listeria_monocytogenes_1639_v91	Listeria_monocytogenes_1639_v100	0.9168
Listeria_monocytogenes_1639_v90	Listeria_monocytogenes_1639_v100	0.9355
Listeria_monocytogenes_1639_v8	Listeria_monocytogenes_1639_v100	0.9747
Listeria_monocytogenes_1639_v89	Listeria_monocytogenes_1639_v100	0.9233
Listeria_monocytogenes_1639_v88	Listeria_monocytogenes_1639_v100	0.9523
Listeria_monocytogenes_1639_v87	Listeria_monocytogenes_1639_v100	0.9459
Listeria_monocytogenes_1639_v86	Listeria_monocytogenes_1639_v100	0.9455
Listeria_monocytogenes_1639_v85	Listeria_monocytogenes_1639_v100	0.9104
Listeria_monocytogenes_1639_v84	Listeria_monocytogenes_1639_v100	0.953
Listeria_monocytogenes_1639_v83	Listeria_monocytogenes_1639_v100	0.9135
Listeria_monocytogenes_1639_v82	Listeria_monocytogenes_1639_v100	0.9132
Listeria_monocytogenes_1639_v81	Listeria_monocytogenes_1639_v100	0.9593
Listeria_monocytogenes_1639_v80	Listeria_monocytogenes_1639_v100	0.9322
Listeria_monocytogenes_1639_v7	Listeria_monocytogenes_1639_v100	0.9747
Listeria_monocytogenes_1639_v79	Listeria_monocytogenes_1639_v100	0.9554
Listeria_monocytogenes_1639_v78	Listeria_monocytogenes_1639_v100	0.959
Listeria_monocytogenes_1639_v77	Listeria_monocytogenes_1639_v100	0.944
Listeria_monocytogenes_1639_v76	Listeria_monocytogenes_1639_v100	0.941
Listeria_monocytogenes_1639_v75	Listeria_monocytogenes_1639_v100	0.8958
Listeria_monocytogenes_1639_v74	Listeria_monocytogenes_1639_v100	0.9418
Listeria_monocytogenes_1639_v73	Listeria_monocytogenes_1639_v100	0.9509
Listeria_monocytogenes_1639_v72	Listeria_monocytogenes_1639_v100	0.9115
Listeria_monocytogenes_1639_v71	Listeria_monocytogenes_1639_v100	0.9408
Listeria_monocytogenes_1639_v70	Listeria_monocytogenes_1639_v100	0.9519
Listeria_monocytogenes_1639_v6	Listeria_monocytogenes_1639_v100	0.9743
Listeria_monocytogenes_1639_v69	Listeria_monocytogenes_1639_v100	0.9117
Listeria_monocytogenes_1639_v68	Listeria_monocytogenes_1639_v100	0.9353
Listeria_monocytogenes_1639_v67	Listeria_monocytogenes_1639_v100	0.9309
Listeria_monocytogenes_1639_v66	Listeria_monocytogenes_1639_v100	0.9472
Listeria_monocytogenes_1639_v65	Listeria_monocytogenes_1639_v100	0.9644
Listeria_monocytogenes_1639_v64	Listeria_monocytogenes_1639_v100	0.9432
Listeria_monocytogenes_1639_v63	Listeria_monocytogenes_1639_v100	0.9416
Listeria_monocytogenes_1639_v62	Listeria_monocytogenes_1639_v100	0.9522
Listeria_monocytogenes_1639_v61	Listeria_monocytogenes_1639_v100	0.9677
Listeria_monocytogenes_1639_v60	Listeria_monocytogenes_1639_v100	0.9427
Listeria_monocytogenes_1639_v5	Listeria_monocytogenes_1639_v100	0.9006
Listeria_monocytogenes_1639_v59	Listeria_monocytogenes_1639_v100	0.9175
Listeria_monocytogenes_1639_v58	Listeria_monocytogenes_1639_v100	0.9123
Listeria_monocytogenes_1639_v57	Listeria_monocytogenes_1639_v100	0.914
Listeria_monocytogenes_1639_v56	Listeria_monocytogenes_1639_v100	0.9197
Listeria_monocytogenes_1639_v55	Listeria_monocytogenes_1639_v100	0.9176
Listeria_monocytogenes_1639_v54	Listeria_monocytogenes_1639_v100	0.9125
Listeria_monocytogenes_1639_v53	Listeria_monocytogenes_1639_v100	0.932
Listeria_monocytogenes_1639_v52	Listeria_monocytogenes_1639_v100	0.917
Listeria_monocytogenes_1639_v51	Listeria_monocytogenes_1639_v100	0.9132
Listeria_monocytogenes_1639_v50	Listeria_monocytogenes_1639_v100	0.9656
Listeria_monocytogenes_1639_v4	Listeria_monocytogenes_1639_v100	0.9064
Listeria_monocytogenes_1639_v49	Listeria_monocytogenes_1639_v100	0.9096
Listeria_monocytogenes_1639_v48	Listeria_monocytogenes_1639_v100	0.9498
Listeria_monocytogenes_1639_v47	Listeria_monocytogenes_1639_v100	0.9656
Listeria_monocytogenes_1639_v46	Listeria_monocytogenes_1639_v100	0.9379
Listeria_monocytogenes_1639_v45	Listeria_monocytogenes_1639_v100	0.9746

Listeria_monocytogenes_1639_v44	Listeria_monocytogenes_1639_v100	0.967
Listeria_monocytogenes_1639_v43	Listeria_monocytogenes_1639_v100	0.967
Listeria_monocytogenes_1639_v42	Listeria_monocytogenes_1639_v100	0.9283
Listeria_monocytogenes_1639_v41	Listeria_monocytogenes_1639_v100	0.9294
Listeria_monocytogenes_1639_v40	Listeria_monocytogenes_1639_v100	0.9429
Listeria_monocytogenes_1639_v3	Listeria_monocytogenes_1639_v100	0.9071
Listeria_monocytogenes_1639_v39	Listeria_monocytogenes_1639_v100	0.9342
Listeria_monocytogenes_1639_v38	Listeria_monocytogenes_1639_v100	0.9365
Listeria_monocytogenes_1639_v37	Listeria_monocytogenes_1639_v100	0.9303
Listeria_monocytogenes_1639_v36	Listeria_monocytogenes_1639_v100	0.9384
Listeria_monocytogenes_1639_v35	Listeria_monocytogenes_1639_v100	0.9403
Listeria_monocytogenes_1639_v34	Listeria_monocytogenes_1639_v100	0.9424
Listeria_monocytogenes_1639_v33	Listeria_monocytogenes_1639_v100	0.9325
Listeria_monocytogenes_1639_v32	Listeria_monocytogenes_1639_v100	0.9395
Listeria_monocytogenes_1639_v31	Listeria_monocytogenes_1639_v100	0.9424
Listeria_monocytogenes_1639_v30	Listeria_monocytogenes_1639_v100	0.9132
Listeria_monocytogenes_1639_v2	Listeria_monocytogenes_1639_v100	0.9035
Listeria_monocytogenes_1639_v29	Listeria_monocytogenes_1639_v100	0.9514
Listeria_monocytogenes_1639_v28	Listeria_monocytogenes_1639_v100	0.942
Listeria_monocytogenes_1639_v27	Listeria_monocytogenes_1639_v100	0.9354
Listeria_monocytogenes_1639_v26	Listeria_monocytogenes_1639_v100	0.9358
Listeria_monocytogenes_1639_v25	Listeria_monocytogenes_1639_v100	0.9358
Listeria_monocytogenes_1639_v24	Listeria_monocytogenes_1639_v100	0.915
Listeria_monocytogenes_1639_v23	Listeria_monocytogenes_1639_v100	0.9147
Listeria_monocytogenes_1639_v22	Listeria_monocytogenes_1639_v100	0.9372
Listeria_monocytogenes_1639_v21	Listeria_monocytogenes_1639_v100	0.9147
Listeria_monocytogenes_1639_v20	Listeria_monocytogenes_1639_v100	0.9431
Listeria_monocytogenes_1639_v1	Listeria_monocytogenes_1639_v100	0.9463
Listeria_monocytogenes_1639_v19	Listeria_monocytogenes_1639_v100	0.9424
Listeria_monocytogenes_1639_v198	Listeria_monocytogenes_1639_v100	0.8926
Listeria_monocytogenes_1639_v197	Listeria_monocytogenes_1639_v100	0.9251
Listeria_monocytogenes_1639_v196	Listeria_monocytogenes_1639_v100	0.9326
Listeria_monocytogenes_1639_v195	Listeria_monocytogenes_1639_v100	0.9221
Listeria_monocytogenes_1639_v194	Listeria_monocytogenes_1639_v100	0.9203
Listeria_monocytogenes_1639_v193	Listeria_monocytogenes_1639_v100	0.9207
Listeria_monocytogenes_1639_v192	Listeria_monocytogenes_1639_v100	0.8826
Listeria_monocytogenes_1639_v191	Listeria_monocytogenes_1639_v100	0.9448
Listeria_monocytogenes_1639_v190	Listeria_monocytogenes_1639_v100	0.9475
Listeria_monocytogenes_1639_v18	Listeria_monocytogenes_1639_v100	0.9098
Listeria_monocytogenes_1639_v189	Listeria_monocytogenes_1639_v100	0.9468
Listeria_monocytogenes_1639_v188	Listeria_monocytogenes_1639_v100	0.9429
Listeria_monocytogenes_1639_v187	Listeria_monocytogenes_1639_v100	0.9475
Listeria_monocytogenes_1639_v186	Listeria_monocytogenes_1639_v100	0.9479
Listeria_monocytogenes_1639_v185	Listeria_monocytogenes_1639_v100	0.9479
Listeria_monocytogenes_1639_v184	Listeria_monocytogenes_1639_v100	0.9471
Listeria_monocytogenes_1639_v183	Listeria_monocytogenes_1639_v100	0.9403
Listeria_monocytogenes_1639_v182	Listeria_monocytogenes_1639_v100	0.9425
Listeria_monocytogenes_1639_v181	Listeria_monocytogenes_1639_v100	0.8998
Listeria_monocytogenes_1639_v180	Listeria_monocytogenes_1639_v100	0.8998
Listeria_monocytogenes_1639_v17	Listeria_monocytogenes_1639_v100	0.9101
Listeria_monocytogenes_1639_v179	Listeria_monocytogenes_1639_v100	0.9461
Listeria_monocytogenes_1639_v178	Listeria_monocytogenes_1639_v100	0.946
Listeria_monocytogenes_1639_v177	Listeria_monocytogenes_1639_v100	0.9243
Listeria_monocytogenes_1639_v176	Listeria_monocytogenes_1639_v100	0.9166
Listeria_monocytogenes_1639_v175	Listeria_monocytogenes_1639_v100	0.9528
Listeria_monocytogenes_1639_v174	Listeria_monocytogenes_1639_v100	0.9468
Listeria_monocytogenes_1639_v173	Listeria_monocytogenes_1639_v100	0.9302
Listeria_monocytogenes_1639_v172	Listeria_monocytogenes_1639_v100	0.9152
Listeria_monocytogenes_1639_v171	Listeria_monocytogenes_1639_v100	0.945
Listeria_monocytogenes_1639_v170	Listeria_monocytogenes_1639_v100	0.9441
Listeria_monocytogenes_1639_v16	Listeria_monocytogenes_1639_v100	0.9701
Listeria_monocytogenes_1639_v169	Listeria_monocytogenes_1639_v100	0.9539
Listeria_monocytogenes_1639_v168	Listeria_monocytogenes_1639_v100	0.9405
Listeria_monocytogenes_1639_v167	Listeria_monocytogenes_1639_v100	0.9426
Listeria_monocytogenes_1639_v166	Listeria_monocytogenes_1639_v100	0.9408
Listeria_monocytogenes_1639_v165	Listeria_monocytogenes_1639_v100	0.9536
Listeria_monocytogenes_1639_v164	Listeria_monocytogenes_1639_v100	0.9203
Listeria_monocytogenes_1639_v163	Listeria_monocytogenes_1639_v100	0.9279
Listeria_monocytogenes_1639_v162	Listeria_monocytogenes_1639_v100	0.9203
Listeria_monocytogenes_1639_v161	Listeria_monocytogenes_1639_v100	0.9215
Listeria_monocytogenes_1639_v160	Listeria_monocytogenes_1639_v100	0.9211
Listeria_monocytogenes_1639_v15	Listeria_monocytogenes_1639_v100	0.9227
Listeria_monocytogenes_1639_v159	Listeria_monocytogenes_1639_v100	0.9243
Listeria_monocytogenes_1639_v158	Listeria_monocytogenes_1639_v100	0.9208
Listeria_monocytogenes_1639_v157	Listeria_monocytogenes_1639_v100	0.9208
Listeria_monocytogenes_1639_v156	Listeria_monocytogenes_1639_v100	0.9377
Listeria_monocytogenes_1639_v155	Listeria_monocytogenes_1639_v100	0.9458
Listeria_monocytogenes_1639_v154	Listeria_monocytogenes_1639_v100	0.9739
Listeria_monocytogenes_1639_v153	Listeria_monocytogenes_1639_v100	0.9398
Listeria_monocytogenes_1639_v152	Listeria_monocytogenes_1639_v100	0.9243
Listeria_monocytogenes_1639_v151	Listeria_monocytogenes_1639_v100	0.9243
Listeria_monocytogenes_1639_v150	Listeria_monocytogenes_1639_v100	0.9499
Listeria_monocytogenes_1639_v14	Listeria_monocytogenes_1639_v100	0.9127
Listeria_monocytogenes_1639_v149	Listeria_monocytogenes_1639_v100	0.9496
Listeria_monocytogenes_1639_v148	Listeria_monocytogenes_1639_v100	0.9236
Listeria_monocytogenes_1639_v147	Listeria_monocytogenes_1639_v100	0.9192
Listeria_monocytogenes_1639_v146	Listeria_monocytogenes_1639_v100	0.9439
Listeria_monocytogenes_1639_v145	Listeria_monocytogenes_1639_v100	0.9399
Listeria_monocytogenes_1639_v144	Listeria_monocytogenes_1639_v100	0.9271
Listeria_monocytogenes_1639_v143	Listeria_monocytogenes_1639_v100	0.9417
Listeria_monocytogenes_1639_v142	Listeria_monocytogenes_1639_v100	0.9271

Listeria_monocytogenes_1639_v141	Listeria_monocytogenes_1639_v100	0.9342
Listeria_monocytogenes_1639_v140	Listeria_monocytogenes_1639_v100	0.9278
Listeria_monocytogenes_1639_v13	Listeria_monocytogenes_1639_v100	0.9227
Listeria_monocytogenes_1639_v139	Listeria_monocytogenes_1639_v100	0.9219
Listeria_monocytogenes_1639_v138	Listeria_monocytogenes_1639_v100	0.9301
Listeria_monocytogenes_1639_v137	Listeria_monocytogenes_1639_v100	0.9298
Listeria_monocytogenes_1639_v136	Listeria_monocytogenes_1639_v100	0.9479
Listeria_monocytogenes_1639_v135	Listeria_monocytogenes_1639_v100	0.8955
Listeria_monocytogenes_1639_v134	Listeria_monocytogenes_1639_v100	0.9155
Listeria_monocytogenes_1639_v133	Listeria_monocytogenes_1639_v100	0.9481
Listeria_monocytogenes_1639_v132	Listeria_monocytogenes_1639_v100	0.899
Listeria_monocytogenes_1639_v131	Listeria_monocytogenes_1639_v100	0.9187
Listeria_monocytogenes_1639_v130	Listeria_monocytogenes_1639_v100	0.9462
Listeria_monocytogenes_1639_v12	Listeria_monocytogenes_1639_v100	0.943
Listeria_monocytogenes_1639_v129	Listeria_monocytogenes_1639_v100	0.9301
Listeria_monocytogenes_1639_v128	Listeria_monocytogenes_1639_v100	0.9311
Listeria_monocytogenes_1639_v127	Listeria_monocytogenes_1639_v100	0.9383
Listeria_monocytogenes_1639_v126	Listeria_monocytogenes_1639_v100	0.9322
Listeria_monocytogenes_1639_v125	Listeria_monocytogenes_1639_v100	0.9421
Listeria_monocytogenes_1639_v124	Listeria_monocytogenes_1639_v100	0.9034
Listeria_monocytogenes_1639_v123	Listeria_monocytogenes_1639_v100	0.9251
Listeria_monocytogenes_1639_v122	Listeria_monocytogenes_1639_v100	0.9247
Listeria_monocytogenes_1639_v121	Listeria_monocytogenes_1639_v100	0.9247
Listeria_monocytogenes_1639_v120	Listeria_monocytogenes_1639_v100	0.9247
Listeria_monocytogenes_1639_v11	Listeria_monocytogenes_1639_v100	0.948
Listeria_monocytogenes_1639_v119	Listeria_monocytogenes_1639_v100	0.9247
Listeria_monocytogenes_1639_v118	Listeria_monocytogenes_1639_v100	0.9247
Listeria_monocytogenes_1639_v117	Listeria_monocytogenes_1639_v100	0.9464
Listeria_monocytogenes_1639_v116	Listeria_monocytogenes_1639_v100	0.9154
Listeria_monocytogenes_1639_v115	Listeria_monocytogenes_1639_v100	0.9157
Listeria_monocytogenes_1639_v114	Listeria_monocytogenes_1639_v100	0.9519
Listeria_monocytogenes_1639_v113	Listeria_monocytogenes_1639_v100	0.9388
Listeria_monocytogenes_1639_v112	Listeria_monocytogenes_1639_v100	0.9227
Listeria_monocytogenes_1639_v111	Listeria_monocytogenes_1639_v100	0.928
Listeria_monocytogenes_1639_v110	Listeria_monocytogenes_1639_v100	0.9445
Listeria_monocytogenes_1639_v10	Listeria_monocytogenes_1639_v100	0.9193
Listeria_monocytogenes_1639_v109	Listeria_monocytogenes_1639_v100	0.9563
Listeria_monocytogenes_1639_v108	Listeria_monocytogenes_1639_v100	0.9189
Listeria_monocytogenes_1639_v107	Listeria_monocytogenes_1639_v100	0.9182
Listeria_monocytogenes_1639_v106	Listeria_monocytogenes_1639_v100	0.9185
Listeria_monocytogenes_1639_v105	Listeria_monocytogenes_1639_v100	0.9185
Listeria_monocytogenes_1639_v104	Listeria_monocytogenes_1639_v100	0.9185
Listeria_monocytogenes_1639_v103	Listeria_monocytogenes_1639_v100	0.9189
Listeria_monocytogenes_1639_v102	Listeria_monocytogenes_1639_v100	0.9819
Listeria_monocytogenes_1639_v101	Listeria_monocytogenes_1639_v100	0.9941
Listeria_monocytogenes_1639_v100	Listeria_monocytogenes_1639_v100	0.0731
Listeria_welshimeri_1643	Listeria_monocytogenes_1639_v101	0.8646
Listeria_welshimeri_serovar_6b_str_SLCC5334_386043	Listeria_monocytogenes_1639_v101	0.8639
Listeria_seeligeri_serovar_1_2b_str_SLCC3954_683837	Listeria_monocytogenes_1639_v101	0.8688
Listeria_seeligeri_FSL_S4-171_702452	Listeria_monocytogenes_1639_v101	0.8106
Listeria_seeligeri_FSL_N1-067_702453	Listeria_monocytogenes_1639_v101	0.8375
Listeria_seeligeri_1640_v1	Listeria_monocytogenes_1639_v101	0.8674
Listeria_seeligeri_1640	Listeria_monocytogenes_1639_v101	0.8647
Listeria_monocytogenes_WSLC1042_1457188	Listeria_monocytogenes_1639_v101	0.9154
Listeria_monocytogenes_WSLC1001_1457187	Listeria_monocytogenes_1639_v101	0.9495
Listeria_monocytogenes_SLCC7179_879090	Listeria_monocytogenes_1639_v101	0.9445
Listeria_monocytogenes_SLCC5850_882096	Listeria_monocytogenes_1639_v101	0.9496
Listeria_monocytogenes_SLCC2755_932919	Listeria_monocytogenes_1639_v101	0.923
Listeria_monocytogenes_SLCC2540_879089	Listeria_monocytogenes_1639_v101	0.9339
Listeria_monocytogenes_SLCC2479_882020	Listeria_monocytogenes_1639_v101	0.952
Listeria_monocytogenes_SLCC2378_879088	Listeria_monocytogenes_1639_v101	0.9167
Listeria_monocytogenes_SLCC2376_882097	Listeria_monocytogenes_1639_v101	0.921
Listeria_monocytogenes_SLCC2372_932920	Listeria_monocytogenes_1639_v101	0.9517
Listeria_monocytogenes_serotype_4b_str_LL195_1230340	Listeria_monocytogenes_1639_v101	0.9218
Listeria_monocytogenes_serotype_4b_str_LIS0087_1134669	Listeria_monocytogenes_1639_v101	0.9289
Listeria_monocytogenes_serotype_4b_str_F2365_265669	Listeria_monocytogenes_1639_v101	0.9218
Listeria_monocytogenes_serotype_4b_str_CLIP_80459_568819	Listeria_monocytogenes_1639_v101	0.9285
Listeria_monocytogenes_serotype_4b_str_81-0861_1196161	Listeria_monocytogenes_1639_v101	0.9114
Listeria_monocytogenes_serotype_4b_str_81-0592_1196160	Listeria_monocytogenes_1639_v101	0.9124
Listeria_monocytogenes_serotype_4b_str_81-0558_1196159	Listeria_monocytogenes_1639_v101	0.9131
Listeria_monocytogenes_serotype_4b_str_10-0809_1196162	Listeria_monocytogenes_1639_v101	0.9124
Listeria_monocytogenes_serotype_4b_str_02-6680_1196175	Listeria_monocytogenes_1639_v101	0.9249
Listeria_monocytogenes_serotype_4b_str_02-6679_1196174	Listeria_monocytogenes_1639_v101	0.9249
Listeria_monocytogenes_serotype_4b_str_02-1792_1196173	Listeria_monocytogenes_1639_v101	0.9142
Listeria_monocytogenes_serotype_4b_str_02-1289_1196172	Listeria_monocytogenes_1639_v101	0.9142
Listeria_monocytogenes_serotype_4b_str_02-1103_1196171	Listeria_monocytogenes_1639_v101	0.9142
Listeria_monocytogenes_serotype_3c_str_10-5027_1196195	Listeria_monocytogenes_1639_v101	0.9565
Listeria_monocytogenes_serotype_1_2c_str_10-5026_1196194	Listeria_monocytogenes_1639_v101	0.9572
Listeria_monocytogenes_serotype_1_2c_str_10-5025_1196193	Listeria_monocytogenes_1639_v101	0.9572
Listeria_monocytogenes_serotype_1_2b_str_10-0811_1196164	Listeria_monocytogenes_1639_v101	0.9328
Listeria_monocytogenes_serotype_1_2b_str_10-0810_1196163	Listeria_monocytogenes_1639_v101	0.9328
Listeria_monocytogenes_serotype_1_2a_str_99-6370_1196179	Listeria_monocytogenes_1639_v101	0.9614
Listeria_monocytogenes_serotype_1_2a_str_98-2035_1196178	Listeria_monocytogenes_1639_v101	0.9614
Listeria_monocytogenes_serotype_1_2a_str_95-0093_1196177	Listeria_monocytogenes_1639_v101	0.9674
Listeria_monocytogenes_serotype_1_2a_str_88-0478_1196176	Listeria_monocytogenes_1639_v101	0.9674
Listeria_monocytogenes_serotype_1_2a_str_10-5024_1196192	Listeria_monocytogenes_1639_v101	0.9674
Listeria_monocytogenes_serotype_1_2a_str_10-4758_1196168	Listeria_monocytogenes_1639_v101	0.9475
Listeria_monocytogenes_serotype_1_2a_str_10-4754_1196167	Listeria_monocytogenes_1639_v101	0.9475
Listeria_monocytogenes_serotype_1_2a_str_10-1321_1196191	Listeria_monocytogenes_1639_v101	0.9674
Listeria_monocytogenes_serotype_1_2a_str_10-1047_1196190	Listeria_monocytogenes_1639_v101	0.967
Listeria_monocytogenes_serotype_1_2a_str_10-1046_1196189	Listeria_monocytogenes_1639_v101	0.967

Listeria_monocytogenes_serotype_1_2a_str_10-0934_1196170	Listeria_monocytogenes_1639_v101	0.9483
Listeria_monocytogenes_serotype_1_2a_str_10-0933_1196169	Listeria_monocytogenes_1639_v101	0.9483
Listeria_monocytogenes_serotype_1_2a_str_10-0815_1196188	Listeria_monocytogenes_1639_v101	0.9624
Listeria_monocytogenes_serotype_1_2a_str_10-0814_1196187	Listeria_monocytogenes_1639_v101	0.967
Listeria_monocytogenes_serotype_1_2a_str_10-0813_1196166	Listeria_monocytogenes_1639_v101	0.9493
Listeria_monocytogenes_serotype_1_2a_str_10-0812_1196165	Listeria_monocytogenes_1639_v101	0.9493
Listeria_monocytogenes_serotype_1_2a_str_08-7669_1196186	Listeria_monocytogenes_1639_v101	0.9619
Listeria_monocytogenes_serotype_1_2a_str_08-7374_1196185	Listeria_monocytogenes_1639_v101	0.9674
Listeria_monocytogenes_serotype_1_2a_str_08-6997_1196184	Listeria_monocytogenes_1639_v101	0.9624
Listeria_monocytogenes_serotype_1_2a_str_08-6569_1196183	Listeria_monocytogenes_1639_v101	0.9624
Listeria_monocytogenes_serotype_1_2a_str_08-6056_1196182	Listeria_monocytogenes_1639_v101	0.9624
Listeria_monocytogenes_serotype_1_2a_str_04-5457_1196181	Listeria_monocytogenes_1639_v101	0.9674
Listeria_monocytogenes_serotype_1_2a_str_02-5993_1196180	Listeria_monocytogenes_1639_v101	0.967
Listeria_monocytogenes_R479a_1437838	Listeria_monocytogenes_1639_v101	0.9956
Listeria_monocytogenes_N53-1_1234142	Listeria_monocytogenes_1639_v101	0.8906
Listeria_monocytogenes_M7_1030009	Listeria_monocytogenes_1639_v101	0.8954
Listeria_monocytogenes_LO28_393132	Listeria_monocytogenes_1639_v101	0.9477
Listeria_monocytogenes_L99_563174	Listeria_monocytogenes_1639_v101	0.896
Listeria_monocytogenes_L312_882094	Listeria_monocytogenes_1639_v101	0.9285
Listeria_monocytogenes_J1816_930781	Listeria_monocytogenes_1639_v101	0.9186
Listeria_monocytogenes_J1-220_930782	Listeria_monocytogenes_1639_v101	0.919
Listeria_monocytogenes_J0161_393130	Listeria_monocytogenes_1639_v101	0.9403
Listeria_monocytogenes_HCC23_552536	Listeria_monocytogenes_1639_v101	0.8957
Listeria_monocytogenes_FSL_R2-561_393126	Listeria_monocytogenes_1639_v101	0.9539
Listeria_monocytogenes_FSL_J1-208_393119	Listeria_monocytogenes_1639_v101	0.8884
Listeria_monocytogenes_FSL_J1-175_393118	Listeria_monocytogenes_1639_v101	0.931
Listeria_monocytogenes_Finland_1998_393127	Listeria_monocytogenes_1639_v101	0.9487
Listeria_monocytogenes_EGD-e_169963_v1	Listeria_monocytogenes_1639_v101	0.939
Listeria_monocytogenes_EGD-e_169963	Listeria_monocytogenes_1639_v101	0.939
Listeria_monocytogenes_EGD_1334565	Listeria_monocytogenes_1639_v101	0.9504
Listeria_monocytogenes_CFSAN002349_1299895	Listeria_monocytogenes_1639_v101	0.9258
Listeria_monocytogenes_ATCC_19117_882095_v1	Listeria_monocytogenes_1639_v101	0.9203
Listeria_monocytogenes_ATCC_19117_882095	Listeria_monocytogenes_1639_v101	0.9202
Listeria_monocytogenes_6179_1288295	Listeria_monocytogenes_1639_v101	0.9513
Listeria_monocytogenes_1639_v9	Listeria_monocytogenes_1639_v101	0.9192
Listeria_monocytogenes_1639_v99	Listeria_monocytogenes_1639_v101	0.9249
Listeria_monocytogenes_1639_v98	Listeria_monocytogenes_1639_v101	0.9366
Listeria_monocytogenes_1639_v97	Listeria_monocytogenes_1639_v101	0.9225
Listeria_monocytogenes_1639_v96	Listeria_monocytogenes_1639_v101	0.9253
Listeria_monocytogenes_1639_v95	Listeria_monocytogenes_1639_v101	0.9254
Listeria_monocytogenes_1639_v94	Listeria_monocytogenes_1639_v101	0.9257
Listeria_monocytogenes_1639_v93	Listeria_monocytogenes_1639_v101	0.9476
Listeria_monocytogenes_1639_v92	Listeria_monocytogenes_1639_v101	0.9253
Listeria_monocytogenes_1639_v91	Listeria_monocytogenes_1639_v101	0.9204
Listeria_monocytogenes_1639_v90	Listeria_monocytogenes_1639_v101	0.9366
Listeria_monocytogenes_1639_v8	Listeria_monocytogenes_1639_v101	0.9736
Listeria_monocytogenes_1639_v89	Listeria_monocytogenes_1639_v101	0.9251
Listeria_monocytogenes_1639_v88	Listeria_monocytogenes_1639_v101	0.9541
Listeria_monocytogenes_1639_v87	Listeria_monocytogenes_1639_v101	0.9484
Listeria_monocytogenes_1639_v86	Listeria_monocytogenes_1639_v101	0.9477
Listeria_monocytogenes_1639_v85	Listeria_monocytogenes_1639_v101	0.9118
Listeria_monocytogenes_1639_v84	Listeria_monocytogenes_1639_v101	0.9544
Listeria_monocytogenes_1639_v83	Listeria_monocytogenes_1639_v101	0.9145
Listeria_monocytogenes_1639_v82	Listeria_monocytogenes_1639_v101	0.9142
Listeria_monocytogenes_1639_v81	Listeria_monocytogenes_1639_v101	0.9612
Listeria_monocytogenes_1639_v80	Listeria_monocytogenes_1639_v101	0.9329
Listeria_monocytogenes_1639_v7	Listeria_monocytogenes_1639_v101	0.9736
Listeria_monocytogenes_1639_v79	Listeria_monocytogenes_1639_v101	0.9557
Listeria_monocytogenes_1639_v78	Listeria_monocytogenes_1639_v101	0.9604
Listeria_monocytogenes_1639_v77	Listeria_monocytogenes_1639_v101	0.9451
Listeria_monocytogenes_1639_v76	Listeria_monocytogenes_1639_v101	0.9435
Listeria_monocytogenes_1639_v75	Listeria_monocytogenes_1639_v101	0.896
Listeria_monocytogenes_1639_v74	Listeria_monocytogenes_1639_v101	0.9425
Listeria_monocytogenes_1639_v73	Listeria_monocytogenes_1639_v101	0.952
Listeria_monocytogenes_1639_v72	Listeria_monocytogenes_1639_v101	0.9144
Listeria_monocytogenes_1639_v71	Listeria_monocytogenes_1639_v101	0.9408
Listeria_monocytogenes_1639_v70	Listeria_monocytogenes_1639_v101	0.9526
Listeria_monocytogenes_1639_v6	Listeria_monocytogenes_1639_v101	0.9729
Listeria_monocytogenes_1639_v69	Listeria_monocytogenes_1639_v101	0.9128
Listeria_monocytogenes_1639_v68	Listeria_monocytogenes_1639_v101	0.9363
Listeria_monocytogenes_1639_v67	Listeria_monocytogenes_1639_v101	0.9341
Listeria_monocytogenes_1639_v66	Listeria_monocytogenes_1639_v101	0.9479
Listeria_monocytogenes_1639_v65	Listeria_monocytogenes_1639_v101	0.9651
Listeria_monocytogenes_1639_v64	Listeria_monocytogenes_1639_v101	0.9431
Listeria_monocytogenes_1639_v63	Listeria_monocytogenes_1639_v101	0.9415
Listeria_monocytogenes_1639_v62	Listeria_monocytogenes_1639_v101	0.9551
Listeria_monocytogenes_1639_v61	Listeria_monocytogenes_1639_v101	0.9684
Listeria_monocytogenes_1639_v60	Listeria_monocytogenes_1639_v101	0.9437
Listeria_monocytogenes_1639_v5	Listeria_monocytogenes_1639_v101	0.9019
Listeria_monocytogenes_1639_v59	Listeria_monocytogenes_1639_v101	0.9189
Listeria_monocytogenes_1639_v58	Listeria_monocytogenes_1639_v101	0.9137
Listeria_monocytogenes_1639_v57	Listeria_monocytogenes_1639_v101	0.9158
Listeria_monocytogenes_1639_v56	Listeria_monocytogenes_1639_v101	0.92
Listeria_monocytogenes_1639_v55	Listeria_monocytogenes_1639_v101	0.919
Listeria_monocytogenes_1639_v54	Listeria_monocytogenes_1639_v101	0.9135
Listeria_monocytogenes_1639_v53	Listeria_monocytogenes_1639_v101	0.933
Listeria_monocytogenes_1639_v52	Listeria_monocytogenes_1639_v101	0.9199
Listeria_monocytogenes_1639_v51	Listeria_monocytogenes_1639_v101	0.9146
Listeria_monocytogenes_1639_v50	Listeria_monocytogenes_1639_v101	0.9659
Listeria_monocytogenes_1639_v4	Listeria_monocytogenes_1639_v101	0.9074
Listeria_monocytogenes_1639_v49	Listeria_monocytogenes_1639_v101	0.9106

Listeria_monocytogenes_1639_v48	Listeria_monocytogenes_1639_v101	0.9512
Listeria_monocytogenes_1639_v47	Listeria_monocytogenes_1639_v101	0.9659
Listeria_monocytogenes_1639_v46	Listeria_monocytogenes_1639_v101	0.939
Listeria_monocytogenes_1639_v45	Listeria_monocytogenes_1639_v101	0.9746
Listeria_monocytogenes_1639_v44	Listeria_monocytogenes_1639_v101	0.9674
Listeria_monocytogenes_1639_v43	Listeria_monocytogenes_1639_v101	0.9674
Listeria_monocytogenes_1639_v42	Listeria_monocytogenes_1639_v101	0.9293
Listeria_monocytogenes_1639_v41	Listeria_monocytogenes_1639_v101	0.9297
Listeria_monocytogenes_1639_v40	Listeria_monocytogenes_1639_v101	0.9436
Listeria_monocytogenes_1639_v3	Listeria_monocytogenes_1639_v101	0.9081
Listeria_monocytogenes_1639_v39	Listeria_monocytogenes_1639_v101	0.9341
Listeria_monocytogenes_1639_v38	Listeria_monocytogenes_1639_v101	0.9361
Listeria_monocytogenes_1639_v37	Listeria_monocytogenes_1639_v101	0.9317
Listeria_monocytogenes_1639_v36	Listeria_monocytogenes_1639_v101	0.9383
Listeria_monocytogenes_1639_v35	Listeria_monocytogenes_1639_v101	0.9406
Listeria_monocytogenes_1639_v34	Listeria_monocytogenes_1639_v101	0.9439
Listeria_monocytogenes_1639_v33	Listeria_monocytogenes_1639_v101	0.9328
Listeria_monocytogenes_1639_v32	Listeria_monocytogenes_1639_v101	0.9401
Listeria_monocytogenes_1639_v31	Listeria_monocytogenes_1639_v101	0.9439
Listeria_monocytogenes_1639_v30	Listeria_monocytogenes_1639_v101	0.915
Listeria_monocytogenes_1639_v2	Listeria_monocytogenes_1639_v101	0.9041
Listeria_monocytogenes_1639_v29	Listeria_monocytogenes_1639_v101	0.9524
Listeria_monocytogenes_1639_v28	Listeria_monocytogenes_1639_v101	0.9427
Listeria_monocytogenes_1639_v27	Listeria_monocytogenes_1639_v101	0.9364
Listeria_monocytogenes_1639_v26	Listeria_monocytogenes_1639_v101	0.9371
Listeria_monocytogenes_1639_v25	Listeria_monocytogenes_1639_v101	0.9368
Listeria_monocytogenes_1639_v24	Listeria_monocytogenes_1639_v101	0.9168
Listeria_monocytogenes_1639_v23	Listeria_monocytogenes_1639_v101	0.9168
Listeria_monocytogenes_1639_v22	Listeria_monocytogenes_1639_v101	0.9382
Listeria_monocytogenes_1639_v21	Listeria_monocytogenes_1639_v101	0.9161
Listeria_monocytogenes_1639_v20	Listeria_monocytogenes_1639_v101	0.943
Listeria_monocytogenes_1639_v1	Listeria_monocytogenes_1639_v101	0.947
Listeria_monocytogenes_1639_v19	Listeria_monocytogenes_1639_v101	0.9423
Listeria_monocytogenes_1639_v198	Listeria_monocytogenes_1639_v101	0.8928
Listeria_monocytogenes_1639_v197	Listeria_monocytogenes_1639_v101	0.9261
Listeria_monocytogenes_1639_v196	Listeria_monocytogenes_1639_v101	0.9336
Listeria_monocytogenes_1639_v195	Listeria_monocytogenes_1639_v101	0.9228
Listeria_monocytogenes_1639_v194	Listeria_monocytogenes_1639_v101	0.9214
Listeria_monocytogenes_1639_v193	Listeria_monocytogenes_1639_v101	0.9217
Listeria_monocytogenes_1639_v192	Listeria_monocytogenes_1639_v101	0.8833
Listeria_monocytogenes_1639_v191	Listeria_monocytogenes_1639_v101	0.9459
Listeria_monocytogenes_1639_v190	Listeria_monocytogenes_1639_v101	0.9486
Listeria_monocytogenes_1639_v18	Listeria_monocytogenes_1639_v101	0.9126
Listeria_monocytogenes_1639_v189	Listeria_monocytogenes_1639_v101	0.9478
Listeria_monocytogenes_1639_v188	Listeria_monocytogenes_1639_v101	0.9439
Listeria_monocytogenes_1639_v187	Listeria_monocytogenes_1639_v101	0.9482
Listeria_monocytogenes_1639_v186	Listeria_monocytogenes_1639_v101	0.9489
Listeria_monocytogenes_1639_v185	Listeria_monocytogenes_1639_v101	0.9486
Listeria_monocytogenes_1639_v184	Listeria_monocytogenes_1639_v101	0.9482
Listeria_monocytogenes_1639_v183	Listeria_monocytogenes_1639_v101	0.941
Listeria_monocytogenes_1639_v182	Listeria_monocytogenes_1639_v101	0.9432
Listeria_monocytogenes_1639_v181	Listeria_monocytogenes_1639_v101	0.9005
Listeria_monocytogenes_1639_v180	Listeria_monocytogenes_1639_v101	0.9005
Listeria_monocytogenes_1639_v17	Listeria_monocytogenes_1639_v101	0.9126
Listeria_monocytogenes_1639_v179	Listeria_monocytogenes_1639_v101	0.9476
Listeria_monocytogenes_1639_v178	Listeria_monocytogenes_1639_v101	0.9471
Listeria_monocytogenes_1639_v177	Listeria_monocytogenes_1639_v101	0.925
Listeria_monocytogenes_1639_v176	Listeria_monocytogenes_1639_v101	0.9177
Listeria_monocytogenes_1639_v175	Listeria_monocytogenes_1639_v101	0.9562
Listeria_monocytogenes_1639_v174	Listeria_monocytogenes_1639_v101	0.9479
Listeria_monocytogenes_1639_v173	Listeria_monocytogenes_1639_v101	0.9309
Listeria_monocytogenes_1639_v172	Listeria_monocytogenes_1639_v101	0.9159
Listeria_monocytogenes_1639_v171	Listeria_monocytogenes_1639_v101	0.9457
Listeria_monocytogenes_1639_v170	Listeria_monocytogenes_1639_v101	0.9448
Listeria_monocytogenes_1639_v16	Listeria_monocytogenes_1639_v101	0.9704
Listeria_monocytogenes_1639_v169	Listeria_monocytogenes_1639_v101	0.9543
Listeria_monocytogenes_1639_v168	Listeria_monocytogenes_1639_v101	0.9416
Listeria_monocytogenes_1639_v167	Listeria_monocytogenes_1639_v101	0.9437
Listeria_monocytogenes_1639_v166	Listeria_monocytogenes_1639_v101	0.9419
Listeria_monocytogenes_1639_v165	Listeria_monocytogenes_1639_v101	0.9539
Listeria_monocytogenes_1639_v164	Listeria_monocytogenes_1639_v101	0.921
Listeria_monocytogenes_1639_v163	Listeria_monocytogenes_1639_v101	0.929
Listeria_monocytogenes_1639_v162	Listeria_monocytogenes_1639_v101	0.921
Listeria_monocytogenes_1639_v161	Listeria_monocytogenes_1639_v101	0.9222
Listeria_monocytogenes_1639_v160	Listeria_monocytogenes_1639_v101	0.9222
Listeria_monocytogenes_1639_v15	Listeria_monocytogenes_1639_v101	0.9241
Listeria_monocytogenes_1639_v159	Listeria_monocytogenes_1639_v101	0.925
Listeria_monocytogenes_1639_v158	Listeria_monocytogenes_1639_v101	0.9218
Listeria_monocytogenes_1639_v157	Listeria_monocytogenes_1639_v101	0.9218
Listeria_monocytogenes_1639_v156	Listeria_monocytogenes_1639_v101	0.9387
Listeria_monocytogenes_1639_v155	Listeria_monocytogenes_1639_v101	0.9469
Listeria_monocytogenes_1639_v154	Listeria_monocytogenes_1639_v101	0.975
Listeria_monocytogenes_1639_v153	Listeria_monocytogenes_1639_v101	0.9412
Listeria_monocytogenes_1639_v152	Listeria_monocytogenes_1639_v101	0.9253
Listeria_monocytogenes_1639_v151	Listeria_monocytogenes_1639_v101	0.9253
Listeria_monocytogenes_1639_v150	Listeria_monocytogenes_1639_v101	0.9502
Listeria_monocytogenes_1639_v14	Listeria_monocytogenes_1639_v101	0.9137
Listeria_monocytogenes_1639_v149	Listeria_monocytogenes_1639_v101	0.9504
Listeria_monocytogenes_1639_v148	Listeria_monocytogenes_1639_v101	0.9243
Listeria_monocytogenes_1639_v147	Listeria_monocytogenes_1639_v101	0.9198
Listeria_monocytogenes_1639_v146	Listeria_monocytogenes_1639_v101	0.945

Listeria_monocytogenes_1639_v145	Listeria_monocytogenes_1639_v101	0.9406
Listeria_monocytogenes_1639_v144	Listeria_monocytogenes_1639_v101	0.9282
Listeria_monocytogenes_1639_v143	Listeria_monocytogenes_1639_v101	0.9428
Listeria_monocytogenes_1639_v142	Listeria_monocytogenes_1639_v101	0.9282
Listeria_monocytogenes_1639_v141	Listeria_monocytogenes_1639_v101	0.9352
Listeria_monocytogenes_1639_v140	Listeria_monocytogenes_1639_v101	0.9285
Listeria_monocytogenes_1639_v13	Listeria_monocytogenes_1639_v101	0.9244
Listeria_monocytogenes_1639_v139	Listeria_monocytogenes_1639_v101	0.9226
Listeria_monocytogenes_1639_v138	Listeria_monocytogenes_1639_v101	0.9308
Listeria_monocytogenes_1639_v137	Listeria_monocytogenes_1639_v101	0.9305
Listeria_monocytogenes_1639_v136	Listeria_monocytogenes_1639_v101	0.9483
Listeria_monocytogenes_1639_v135	Listeria_monocytogenes_1639_v101	0.8961
Listeria_monocytogenes_1639_v134	Listeria_monocytogenes_1639_v101	0.9165
Listeria_monocytogenes_1639_v133	Listeria_monocytogenes_1639_v101	0.9492
Listeria_monocytogenes_1639_v132	Listeria_monocytogenes_1639_v101	0.9
Listeria_monocytogenes_1639_v131	Listeria_monocytogenes_1639_v101	0.9208
Listeria_monocytogenes_1639_v130	Listeria_monocytogenes_1639_v101	0.9469
Listeria_monocytogenes_1639_v12	Listeria_monocytogenes_1639_v101	0.9437
Listeria_monocytogenes_1639_v129	Listeria_monocytogenes_1639_v101	0.9308
Listeria_monocytogenes_1639_v128	Listeria_monocytogenes_1639_v101	0.9318
Listeria_monocytogenes_1639_v127	Listeria_monocytogenes_1639_v101	0.939
Listeria_monocytogenes_1639_v126	Listeria_monocytogenes_1639_v101	0.9333
Listeria_monocytogenes_1639_v125	Listeria_monocytogenes_1639_v101	0.9436
Listeria_monocytogenes_1639_v124	Listeria_monocytogenes_1639_v101	0.9044
Listeria_monocytogenes_1639_v123	Listeria_monocytogenes_1639_v101	0.9258
Listeria_monocytogenes_1639_v122	Listeria_monocytogenes_1639_v101	0.9257
Listeria_monocytogenes_1639_v121	Listeria_monocytogenes_1639_v101	0.9257
Listeria_monocytogenes_1639_v120	Listeria_monocytogenes_1639_v101	0.9254
Listeria_monocytogenes_1639_v11	Listeria_monocytogenes_1639_v101	0.9483
Listeria_monocytogenes_1639_v119	Listeria_monocytogenes_1639_v101	0.9257
Listeria_monocytogenes_1639_v118	Listeria_monocytogenes_1639_v101	0.9257
Listeria_monocytogenes_1639_v117	Listeria_monocytogenes_1639_v101	0.946
Listeria_monocytogenes_1639_v116	Listeria_monocytogenes_1639_v101	0.916
Listeria_monocytogenes_1639_v115	Listeria_monocytogenes_1639_v101	0.9164
Listeria_monocytogenes_1639_v114	Listeria_monocytogenes_1639_v101	0.9533
Listeria_monocytogenes_1639_v113	Listeria_monocytogenes_1639_v101	0.9395
Listeria_monocytogenes_1639_v112	Listeria_monocytogenes_1639_v101	0.923
Listeria_monocytogenes_1639_v111	Listeria_monocytogenes_1639_v101	0.929
Listeria_monocytogenes_1639_v110	Listeria_monocytogenes_1639_v101	0.9455
Listeria_monocytogenes_1639_v10	Listeria_monocytogenes_1639_v101	0.9192
Listeria_monocytogenes_1639_v109	Listeria_monocytogenes_1639_v101	0.957
Listeria_monocytogenes_1639_v108	Listeria_monocytogenes_1639_v101	0.9207
Listeria_monocytogenes_1639_v107	Listeria_monocytogenes_1639_v101	0.9188
Listeria_monocytogenes_1639_v106	Listeria_monocytogenes_1639_v101	0.9192
Listeria_monocytogenes_1639_v105	Listeria_monocytogenes_1639_v101	0.9192
Listeria_monocytogenes_1639_v104	Listeria_monocytogenes_1639_v101	0.9196
Listeria_monocytogenes_1639_v103	Listeria_monocytogenes_1639_v101	0.9196
Listeria_monocytogenes_1639_v102	Listeria_monocytogenes_1639_v101	0.9874
Listeria_monocytogenes_1639_v101	Listeria_monocytogenes_1639_v101	0.0729
Listeria_welshimeri_1643	Listeria_monocytogenes_1639_v102	0.875
Listeria_welshimeri_serovar_6b_str_SLCC5334_386043	Listeria_monocytogenes_1639_v102	0.8743
Listeria_seeligeri_serovar_1_2b_str_SLCC3954_683837	Listeria_monocytogenes_1639_v102	0.8802
Listeria_seeligeri_FSL_S4-171_702452	Listeria_monocytogenes_1639_v102	0.8199
Listeria_seeligeri_FSL_N1-067_702453	Listeria_monocytogenes_1639_v102	0.84
Listeria_seeligeri_1640_v1	Listeria_monocytogenes_1639_v102	0.8771
Listeria_seeligeri_1640	Listeria_monocytogenes_1639_v102	0.8754
Listeria_monocytogenes_WSLC1042_1457188	Listeria_monocytogenes_1639_v102	0.9267
Listeria_monocytogenes_WSLC1001_1457187	Listeria_monocytogenes_1639_v102	0.9609
Listeria_monocytogenes_SLCC7179_879090	Listeria_monocytogenes_1639_v102	0.9567
Listeria_monocytogenes_SLCC5850_882096	Listeria_monocytogenes_1639_v102	0.9618
Listeria_monocytogenes_SLCC2755_932919	Listeria_monocytogenes_1639_v102	0.9265
Listeria_monocytogenes_SLCC2540_879089	Listeria_monocytogenes_1639_v102	0.9447
Listeria_monocytogenes_SLCC2479_882020	Listeria_monocytogenes_1639_v102	0.9634
Listeria_monocytogenes_SLCC2378_879088	Listeria_monocytogenes_1639_v102	0.9281
Listeria_monocytogenes_SLCC2376_882097	Listeria_monocytogenes_1639_v102	0.9326
Listeria_monocytogenes_SLCC2372_932920	Listeria_monocytogenes_1639_v102	0.9555
Listeria_monocytogenes_serotype_4b_str_LL195_1230340	Listeria_monocytogenes_1639_v102	0.9338
Listeria_monocytogenes_serotype_4b_str_LIS0087_1134669	Listeria_monocytogenes_1639_v102	0.9405
Listeria_monocytogenes_serotype_4b_str_F2365_265669	Listeria_monocytogenes_1639_v102	0.9338
Listeria_monocytogenes_serotype_4b_str_CLIP_80459_568819	Listeria_monocytogenes_1639_v102	0.9405
Listeria_monocytogenes_serotype_4b_str_81-0861_1196161	Listeria_monocytogenes_1639_v102	0.9226
Listeria_monocytogenes_serotype_4b_str_81-0592_1196160	Listeria_monocytogenes_1639_v102	0.9237
Listeria_monocytogenes_serotype_4b_str_81-0558_1196159	Listeria_monocytogenes_1639_v102	0.9243
Listeria_monocytogenes_serotype_4b_str_10-0809_1196162	Listeria_monocytogenes_1639_v102	0.9237
Listeria_monocytogenes_serotype_4b_str_02-6680_1196175	Listeria_monocytogenes_1639_v102	0.9369
Listeria_monocytogenes_serotype_4b_str_02-6679_1196174	Listeria_monocytogenes_1639_v102	0.9369
Listeria_monocytogenes_serotype_4b_str_02-1792_1196173	Listeria_monocytogenes_1639_v102	0.9255
Listeria_monocytogenes_serotype_4b_str_02-1289_1196172	Listeria_monocytogenes_1639_v102	0.9255
Listeria_monocytogenes_serotype_4b_str_02-1103_1196171	Listeria_monocytogenes_1639_v102	0.9255
Listeria_monocytogenes_serotype_3c_str_10-5027_1196195	Listeria_monocytogenes_1639_v102	0.9686
Listeria_monocytogenes_serotype_1_2c_str_10-5026_1196194	Listeria_monocytogenes_1639_v102	0.9694
Listeria_monocytogenes_serotype_1_2c_str_10-5025_1196193	Listeria_monocytogenes_1639_v102	0.9694
Listeria_monocytogenes_serotype_1_2b_str_10-0811_1196164	Listeria_monocytogenes_1639_v102	0.9442
Listeria_monocytogenes_serotype_1_2b_str_10-0810_1196163	Listeria_monocytogenes_1639_v102	0.9442
Listeria_monocytogenes_serotype_1_2a_str_99-6370_1196179	Listeria_monocytogenes_1639_v102	0.9716
Listeria_monocytogenes_serotype_1_2a_str_98-2035_1196178	Listeria_monocytogenes_1639_v102	0.9716
Listeria_monocytogenes_serotype_1_2a_str_95-0093_1196177	Listeria_monocytogenes_1639_v102	0.9777
Listeria_monocytogenes_serotype_1_2a_str_88-0478_1196176	Listeria_monocytogenes_1639_v102	0.9777
Listeria_monocytogenes_serotype_1_2a_str_10-5024_1196192	Listeria_monocytogenes_1639_v102	0.9777
Listeria_monocytogenes_serotype_1_2a_str_10-4758_1196168	Listeria_monocytogenes_1639_v102	0.9592
Listeria_monocytogenes_serotype_1_2a_str_10-4754_1196167	Listeria_monocytogenes_1639_v102	0.9592

Listeria_monocytogenes_serotype_1_2a_str_10-1321_1196191	Listeria_monocytogenes_1639_v102	0.9777
Listeria_monocytogenes_serotype_1_2a_str_10-1047_1196190	Listeria_monocytogenes_1639_v102	0.9773
Listeria_monocytogenes_serotype_1_2a_str_10-1046_1196189	Listeria_monocytogenes_1639_v102	0.9773
Listeria_monocytogenes_serotype_1_2a_str_10-0934_1196170	Listeria_monocytogenes_1639_v102	0.9606
Listeria_monocytogenes_serotype_1_2a_str_10-0933_1196169	Listeria_monocytogenes_1639_v102	0.9606
Listeria_monocytogenes_serotype_1_2a_str_10-0815_1196188	Listeria_monocytogenes_1639_v102	0.9727
Listeria_monocytogenes_serotype_1_2a_str_10-0814_1196187	Listeria_monocytogenes_1639_v102	0.9774
Listeria_monocytogenes_serotype_1_2a_str_10-0813_1196166	Listeria_monocytogenes_1639_v102	0.961
Listeria_monocytogenes_serotype_1_2a_str_10-0812_1196165	Listeria_monocytogenes_1639_v102	0.961
Listeria_monocytogenes_serotype_1_2a_str_08-7669_1196186	Listeria_monocytogenes_1639_v102	0.973
Listeria_monocytogenes_serotype_1_2a_str_08-7374_1196185	Listeria_monocytogenes_1639_v102	0.9777
Listeria_monocytogenes_serotype_1_2a_str_08-6997_1196184	Listeria_monocytogenes_1639_v102	0.9727
Listeria_monocytogenes_serotype_1_2a_str_08-6569_1196183	Listeria_monocytogenes_1639_v102	0.9727
Listeria_monocytogenes_serotype_1_2a_str_08-6056_1196182	Listeria_monocytogenes_1639_v102	0.9727
Listeria_monocytogenes_serotype_1_2a_str_04-5457_1196181	Listeria_monocytogenes_1639_v102	0.9777
Listeria_monocytogenes_serotype_1_2a_str_02-5993_1196180	Listeria_monocytogenes_1639_v102	0.9774
Listeria_monocytogenes_R479a_1437838	Listeria_monocytogenes_1639_v102	0.9837
Listeria_monocytogenes_N53-1_1234142	Listeria_monocytogenes_1639_v102	0.9016
Listeria_monocytogenes_M7_1030009	Listeria_monocytogenes_1639_v102	0.9052
Listeria_monocytogenes_LO28_393132	Listeria_monocytogenes_1639_v102	0.959
Listeria_monocytogenes_L99_563174	Listeria_monocytogenes_1639_v102	0.9058
Listeria_monocytogenes_L312_882094	Listeria_monocytogenes_1639_v102	0.9405
Listeria_monocytogenes_J1816_930781	Listeria_monocytogenes_1639_v102	0.9303
Listeria_monocytogenes_J1-220_930782	Listeria_monocytogenes_1639_v102	0.9302
Listeria_monocytogenes_J0161_393130	Listeria_monocytogenes_1639_v102	0.9519
Listeria_monocytogenes_HCC23_552536	Listeria_monocytogenes_1639_v102	0.9055
Listeria_monocytogenes_FSL_R2-561_393126	Listeria_monocytogenes_1639_v102	0.9653
Listeria_monocytogenes_FSL_J1-208_393119	Listeria_monocytogenes_1639_v102	0.8979
Listeria_monocytogenes_FSL_J1-175_393118	Listeria_monocytogenes_1639_v102	0.9422
Listeria_monocytogenes_Finland_1998_393127	Listeria_monocytogenes_1639_v102	0.961
Listeria_monocytogenes_EGD-e_169963_v1	Listeria_monocytogenes_1639_v102	0.951
Listeria_monocytogenes_EGD-e_169963	Listeria_monocytogenes_1639_v102	0.951
Listeria_monocytogenes_EGD_1334565	Listeria_monocytogenes_1639_v102	0.9626
Listeria_monocytogenes_CFSAN002349_1299895	Listeria_monocytogenes_1639_v102	0.9373
Listeria_monocytogenes_ATCC_19117_882095_v1	Listeria_monocytogenes_1639_v102	0.9317
Listeria_monocytogenes_ATCC_19117_882095	Listeria_monocytogenes_1639_v102	0.9317
Listeria_monocytogenes_6179_1288295	Listeria_monocytogenes_1639_v102	0.9481
Listeria_monocytogenes_1639_v9	Listeria_monocytogenes_1639_v102	0.9303
Listeria_monocytogenes_1639_v99	Listeria_monocytogenes_1639_v102	0.9299
Listeria_monocytogenes_1639_v98	Listeria_monocytogenes_1639_v102	0.9484
Listeria_monocytogenes_1639_v97	Listeria_monocytogenes_1639_v102	0.9343
Listeria_monocytogenes_1639_v96	Listeria_monocytogenes_1639_v102	0.9304
Listeria_monocytogenes_1639_v95	Listeria_monocytogenes_1639_v102	0.9304
Listeria_monocytogenes_1639_v94	Listeria_monocytogenes_1639_v102	0.9307
Listeria_monocytogenes_1639_v93	Listeria_monocytogenes_1639_v102	0.9439
Listeria_monocytogenes_1639_v92	Listeria_monocytogenes_1639_v102	0.9303
Listeria_monocytogenes_1639_v91	Listeria_monocytogenes_1639_v102	0.9321
Listeria_monocytogenes_1639_v90	Listeria_monocytogenes_1639_v102	0.9484
Listeria_monocytogenes_1639_v8	Listeria_monocytogenes_1639_v102	0.9641
Listeria_monocytogenes_1639_v89	Listeria_monocytogenes_1639_v102	0.937
Listeria_monocytogenes_1639_v88	Listeria_monocytogenes_1639_v102	0.9659
Listeria_monocytogenes_1639_v87	Listeria_monocytogenes_1639_v102	0.9597
Listeria_monocytogenes_1639_v86	Listeria_monocytogenes_1639_v102	0.959
Listeria_monocytogenes_1639_v85	Listeria_monocytogenes_1639_v102	0.923
Listeria_monocytogenes_1639_v84	Listeria_monocytogenes_1639_v102	0.9658
Listeria_monocytogenes_1639_v83	Listeria_monocytogenes_1639_v102	0.9258
Listeria_monocytogenes_1639_v82	Listeria_monocytogenes_1639_v102	0.9255
Listeria_monocytogenes_1639_v81	Listeria_monocytogenes_1639_v102	0.973
Listeria_monocytogenes_1639_v80	Listeria_monocytogenes_1639_v102	0.9444
Listeria_monocytogenes_1639_v7	Listeria_monocytogenes_1639_v102	0.9641
Listeria_monocytogenes_1639_v79	Listeria_monocytogenes_1639_v102	0.9671
Listeria_monocytogenes_1639_v78	Listeria_monocytogenes_1639_v102	0.9719
Listeria_monocytogenes_1639_v77	Listeria_monocytogenes_1639_v102	0.9564
Listeria_monocytogenes_1639_v76	Listeria_monocytogenes_1639_v102	0.955
Listeria_monocytogenes_1639_v75	Listeria_monocytogenes_1639_v102	0.9058
Listeria_monocytogenes_1639_v74	Listeria_monocytogenes_1639_v102	0.9455
Listeria_monocytogenes_1639_v73	Listeria_monocytogenes_1639_v102	0.954
Listeria_monocytogenes_1639_v72	Listeria_monocytogenes_1639_v102	0.9257
Listeria_monocytogenes_1639_v71	Listeria_monocytogenes_1639_v102	0.9437
Listeria_monocytogenes_1639_v70	Listeria_monocytogenes_1639_v102	0.9624
Listeria_monocytogenes_1639_v6	Listeria_monocytogenes_1639_v102	0.9634
Listeria_monocytogenes_1639_v69	Listeria_monocytogenes_1639_v102	0.924
Listeria_monocytogenes_1639_v68	Listeria_monocytogenes_1639_v102	0.9475
Listeria_monocytogenes_1639_v67	Listeria_monocytogenes_1639_v102	0.9457
Listeria_monocytogenes_1639_v66	Listeria_monocytogenes_1639_v102	0.9589
Listeria_monocytogenes_1639_v65	Listeria_monocytogenes_1639_v102	0.9773
Listeria_monocytogenes_1639_v64	Listeria_monocytogenes_1639_v102	0.9529
Listeria_monocytogenes_1639_v63	Listeria_monocytogenes_1639_v102	0.9456
Listeria_monocytogenes_1639_v62	Listeria_monocytogenes_1639_v102	0.9668
Listeria_monocytogenes_1639_v61	Listeria_monocytogenes_1639_v102	0.9803
Listeria_monocytogenes_1639_v60	Listeria_monocytogenes_1639_v102	0.9554
Listeria_monocytogenes_1639_v5	Listeria_monocytogenes_1639_v102	0.899
Listeria_monocytogenes_1639_v59	Listeria_monocytogenes_1639_v102	0.9302
Listeria_monocytogenes_1639_v58	Listeria_monocytogenes_1639_v102	0.925
Listeria_monocytogenes_1639_v57	Listeria_monocytogenes_1639_v102	0.9273
Listeria_monocytogenes_1639_v56	Listeria_monocytogenes_1639_v102	0.9314
Listeria_monocytogenes_1639_v55	Listeria_monocytogenes_1639_v102	0.9239
Listeria_monocytogenes_1639_v54	Listeria_monocytogenes_1639_v102	0.9249
Listeria_monocytogenes_1639_v53	Listeria_monocytogenes_1639_v102	0.9434
Listeria_monocytogenes_1639_v52	Listeria_monocytogenes_1639_v102	0.9316
Listeria_monocytogenes_1639_v51	Listeria_monocytogenes_1639_v102	0.9221

Listeria_monocytogenes_1639_v50	Listeria_monocytogenes_1639_v102	0.9762
Listeria_monocytogenes_1639_v4	Listeria_monocytogenes_1639_v102	0.9113
Listeria_monocytogenes_1639_v49	Listeria_monocytogenes_1639_v102	0.9214
Listeria_monocytogenes_1639_v48	Listeria_monocytogenes_1639_v102	0.9632
Listeria_monocytogenes_1639_v47	Listeria_monocytogenes_1639_v102	0.9763
Listeria_monocytogenes_1639_v46	Listeria_monocytogenes_1639_v102	0.9502
Listeria_monocytogenes_1639_v45	Listeria_monocytogenes_1639_v102	0.9673
Listeria_monocytogenes_1639_v44	Listeria_monocytogenes_1639_v102	0.9777
Listeria_monocytogenes_1639_v43	Listeria_monocytogenes_1639_v102	0.9777
Listeria_monocytogenes_1639_v42	Listeria_monocytogenes_1639_v102	0.9395
Listeria_monocytogenes_1639_v41	Listeria_monocytogenes_1639_v102	0.934
Listeria_monocytogenes_1639_v40	Listeria_monocytogenes_1639_v102	0.9526
Listeria_monocytogenes_1639_v3	Listeria_monocytogenes_1639_v102	0.9117
Listeria_monocytogenes_1639_v39	Listeria_monocytogenes_1639_v102	0.9452
Listeria_monocytogenes_1639_v38	Listeria_monocytogenes_1639_v102	0.9472
Listeria_monocytogenes_1639_v37	Listeria_monocytogenes_1639_v102	0.9434
Listeria_monocytogenes_1639_v36	Listeria_monocytogenes_1639_v102	0.942
Listeria_monocytogenes_1639_v35	Listeria_monocytogenes_1639_v102	0.9511
Listeria_monocytogenes_1639_v34	Listeria_monocytogenes_1639_v102	0.955
Listeria_monocytogenes_1639_v33	Listeria_monocytogenes_1639_v102	0.9442
Listeria_monocytogenes_1639_v32	Listeria_monocytogenes_1639_v102	0.9512
Listeria_monocytogenes_1639_v31	Listeria_monocytogenes_1639_v102	0.955
Listeria_monocytogenes_1639_v30	Listeria_monocytogenes_1639_v102	0.9258
Listeria_monocytogenes_1639_v2	Listeria_monocytogenes_1639_v102	0.9008
Listeria_monocytogenes_1639_v29	Listeria_monocytogenes_1639_v102	0.9537
Listeria_monocytogenes_1639_v28	Listeria_monocytogenes_1639_v102	0.9538
Listeria_monocytogenes_1639_v27	Listeria_monocytogenes_1639_v102	0.9396
Listeria_monocytogenes_1639_v26	Listeria_monocytogenes_1639_v102	0.9404
Listeria_monocytogenes_1639_v25	Listeria_monocytogenes_1639_v102	0.94
Listeria_monocytogenes_1639_v24	Listeria_monocytogenes_1639_v102	0.928
Listeria_monocytogenes_1639_v23	Listeria_monocytogenes_1639_v102	0.928
Listeria_monocytogenes_1639_v22	Listeria_monocytogenes_1639_v102	0.9497
Listeria_monocytogenes_1639_v21	Listeria_monocytogenes_1639_v102	0.9269
Listeria_monocytogenes_1639_v20	Listeria_monocytogenes_1639_v102	0.9464
Listeria_monocytogenes_1639_v1	Listeria_monocytogenes_1639_v102	0.9438
Listeria_monocytogenes_1639_v19	Listeria_monocytogenes_1639_v102	0.946
Listeria_monocytogenes_1639_v198	Listeria_monocytogenes_1639_v102	0.904
Listeria_monocytogenes_1639_v197	Listeria_monocytogenes_1639_v102	0.9378
Listeria_monocytogenes_1639_v196	Listeria_monocytogenes_1639_v102	0.9458
Listeria_monocytogenes_1639_v195	Listeria_monocytogenes_1639_v102	0.9349
Listeria_monocytogenes_1639_v194	Listeria_monocytogenes_1639_v102	0.933
Listeria_monocytogenes_1639_v193	Listeria_monocytogenes_1639_v102	0.9334
Listeria_monocytogenes_1639_v192	Listeria_monocytogenes_1639_v102	0.8919
Listeria_monocytogenes_1639_v191	Listeria_monocytogenes_1639_v102	0.9582
Listeria_monocytogenes_1639_v190	Listeria_monocytogenes_1639_v102	0.9609
Listeria_monocytogenes_1639_v18	Listeria_monocytogenes_1639_v102	0.9241
Listeria_monocytogenes_1639_v189	Listeria_monocytogenes_1639_v102	0.9602
Listeria_monocytogenes_1639_v188	Listeria_monocytogenes_1639_v102	0.9563
Listeria_monocytogenes_1639_v187	Listeria_monocytogenes_1639_v102	0.9606
Listeria_monocytogenes_1639_v186	Listeria_monocytogenes_1639_v102	0.9613
Listeria_monocytogenes_1639_v185	Listeria_monocytogenes_1639_v102	0.9609
Listeria_monocytogenes_1639_v184	Listeria_monocytogenes_1639_v102	0.9605
Listeria_monocytogenes_1639_v183	Listeria_monocytogenes_1639_v102	0.9533
Listeria_monocytogenes_1639_v182	Listeria_monocytogenes_1639_v102	0.9555
Listeria_monocytogenes_1639_v181	Listeria_monocytogenes_1639_v102	0.9117
Listeria_monocytogenes_1639_v180	Listeria_monocytogenes_1639_v102	0.9117
Listeria_monocytogenes_1639_v17	Listeria_monocytogenes_1639_v102	0.9241
Listeria_monocytogenes_1639_v179	Listeria_monocytogenes_1639_v102	0.9598
Listeria_monocytogenes_1639_v178	Listeria_monocytogenes_1639_v102	0.9594
Listeria_monocytogenes_1639_v177	Listeria_monocytogenes_1639_v102	0.9369
Listeria_monocytogenes_1639_v176	Listeria_monocytogenes_1639_v102	0.9293
Listeria_monocytogenes_1639_v175	Listeria_monocytogenes_1639_v102	0.9681
Listeria_monocytogenes_1639_v174	Listeria_monocytogenes_1639_v102	0.9602
Listeria_monocytogenes_1639_v173	Listeria_monocytogenes_1639_v102	0.943
Listeria_monocytogenes_1639_v172	Listeria_monocytogenes_1639_v102	0.9277
Listeria_monocytogenes_1639_v171	Listeria_monocytogenes_1639_v102	0.9495
Listeria_monocytogenes_1639_v170	Listeria_monocytogenes_1639_v102	0.957
Listeria_monocytogenes_1639_v16	Listeria_monocytogenes_1639_v102	0.9631
Listeria_monocytogenes_1639_v169	Listeria_monocytogenes_1639_v102	0.9658
Listeria_monocytogenes_1639_v168	Listeria_monocytogenes_1639_v102	0.953
Listeria_monocytogenes_1639_v167	Listeria_monocytogenes_1639_v102	0.9559
Listeria_monocytogenes_1639_v166	Listeria_monocytogenes_1639_v102	0.9534
Listeria_monocytogenes_1639_v165	Listeria_monocytogenes_1639_v102	0.9655
Listeria_monocytogenes_1639_v164	Listeria_monocytogenes_1639_v102	0.9329
Listeria_monocytogenes_1639_v163	Listeria_monocytogenes_1639_v102	0.9368
Listeria_monocytogenes_1639_v162	Listeria_monocytogenes_1639_v102	0.9329
Listeria_monocytogenes_1639_v161	Listeria_monocytogenes_1639_v102	0.9341
Listeria_monocytogenes_1639_v160	Listeria_monocytogenes_1639_v102	0.9341
Listeria_monocytogenes_1639_v15	Listeria_monocytogenes_1639_v102	0.9286
Listeria_monocytogenes_1639_v159	Listeria_monocytogenes_1639_v102	0.9369
Listeria_monocytogenes_1639_v158	Listeria_monocytogenes_1639_v102	0.9338
Listeria_monocytogenes_1639_v157	Listeria_monocytogenes_1639_v102	0.9338
Listeria_monocytogenes_1639_v156	Listeria_monocytogenes_1639_v102	0.9424
Listeria_monocytogenes_1639_v155	Listeria_monocytogenes_1639_v102	0.9591
Listeria_monocytogenes_1639_v154	Listeria_monocytogenes_1639_v102	0.9875
Listeria_monocytogenes_1639_v153	Listeria_monocytogenes_1639_v102	0.9534
Listeria_monocytogenes_1639_v152	Listeria_monocytogenes_1639_v102	0.9373
Listeria_monocytogenes_1639_v151	Listeria_monocytogenes_1639_v102	0.9373
Listeria_monocytogenes_1639_v150	Listeria_monocytogenes_1639_v102	0.9621
Listeria_monocytogenes_1639_v14	Listeria_monocytogenes_1639_v102	0.9226
Listeria_monocytogenes_1639_v149	Listeria_monocytogenes_1639_v102	0.9626

Listeria_monocytogenes_1639_v148	Listeria_monocytogenes_1639_v102	0.9362
Listeria_monocytogenes_1639_v147	Listeria_monocytogenes_1639_v102	0.9226
Listeria_monocytogenes_1639_v146	Listeria_monocytogenes_1639_v102	0.9572
Listeria_monocytogenes_1639_v145	Listeria_monocytogenes_1639_v102	0.9513
Listeria_monocytogenes_1639_v144	Listeria_monocytogenes_1639_v102	0.9402
Listeria_monocytogenes_1639_v143	Listeria_monocytogenes_1639_v102	0.9546
Listeria_monocytogenes_1639_v142	Listeria_monocytogenes_1639_v102	0.9402
Listeria_monocytogenes_1639_v141	Listeria_monocytogenes_1639_v102	0.9473
Listeria_monocytogenes_1639_v140	Listeria_monocytogenes_1639_v102	0.9405
Listeria_monocytogenes_1639_v13	Listeria_monocytogenes_1639_v102	0.929
Listeria_monocytogenes_1639_v139	Listeria_monocytogenes_1639_v102	0.9342
Listeria_monocytogenes_1639_v138	Listeria_monocytogenes_1639_v102	0.9429
Listeria_monocytogenes_1639_v137	Listeria_monocytogenes_1639_v102	0.9425
Listeria_monocytogenes_1639_v136	Listeria_monocytogenes_1639_v102	0.96
Listeria_monocytogenes_1639_v135	Listeria_monocytogenes_1639_v102	0.9065
Listeria_monocytogenes_1639_v134	Listeria_monocytogenes_1639_v102	0.9279
Listeria_monocytogenes_1639_v133	Listeria_monocytogenes_1639_v102	0.961
Listeria_monocytogenes_1639_v132	Listeria_monocytogenes_1639_v102	0.9081
Listeria_monocytogenes_1639_v131	Listeria_monocytogenes_1639_v102	0.9326
Listeria_monocytogenes_1639_v130	Listeria_monocytogenes_1639_v102	0.9499
Listeria_monocytogenes_1639_v12	Listeria_monocytogenes_1639_v102	0.9449
Listeria_monocytogenes_1639_v129	Listeria_monocytogenes_1639_v102	0.9429
Listeria_monocytogenes_1639_v128	Listeria_monocytogenes_1639_v102	0.9438
Listeria_monocytogenes_1639_v127	Listeria_monocytogenes_1639_v102	0.951
Listeria_monocytogenes_1639_v126	Listeria_monocytogenes_1639_v102	0.9453
Listeria_monocytogenes_1639_v125	Listeria_monocytogenes_1639_v102	0.955
Listeria_monocytogenes_1639_v124	Listeria_monocytogenes_1639_v102	0.916
Listeria_monocytogenes_1639_v123	Listeria_monocytogenes_1639_v102	0.9373
Listeria_monocytogenes_1639_v122	Listeria_monocytogenes_1639_v102	0.9373
Listeria_monocytogenes_1639_v121	Listeria_monocytogenes_1639_v102	0.9373
Listeria_monocytogenes_1639_v120	Listeria_monocytogenes_1639_v102	0.937
Listeria_monocytogenes_1639_v11	Listeria_monocytogenes_1639_v102	0.9451
Listeria_monocytogenes_1639_v119	Listeria_monocytogenes_1639_v102	0.9373
Listeria_monocytogenes_1639_v118	Listeria_monocytogenes_1639_v102	0.9373
Listeria_monocytogenes_1639_v117	Listeria_monocytogenes_1639_v102	0.957
Listeria_monocytogenes_1639_v116	Listeria_monocytogenes_1639_v102	0.9274
Listeria_monocytogenes_1639_v115	Listeria_monocytogenes_1639_v102	0.9278
Listeria_monocytogenes_1639_v114	Listeria_monocytogenes_1639_v102	0.9651
Listeria_monocytogenes_1639_v113	Listeria_monocytogenes_1639_v102	0.9512
Listeria_monocytogenes_1639_v112	Listeria_monocytogenes_1639_v102	0.9345
Listeria_monocytogenes_1639_v111	Listeria_monocytogenes_1639_v102	0.9334
Listeria_monocytogenes_1639_v110	Listeria_monocytogenes_1639_v102	0.9572
Listeria_monocytogenes_1639_v10	Listeria_monocytogenes_1639_v102	0.9303
Listeria_monocytogenes_1639_v109	Listeria_monocytogenes_1639_v102	0.9689
Listeria_monocytogenes_1639_v108	Listeria_monocytogenes_1639_v102	0.9324
Listeria_monocytogenes_1639_v107	Listeria_monocytogenes_1639_v102	0.9303
Listeria_monocytogenes_1639_v106	Listeria_monocytogenes_1639_v102	0.9307
Listeria_monocytogenes_1639_v105	Listeria_monocytogenes_1639_v102	0.9307
Listeria_monocytogenes_1639_v104	Listeria_monocytogenes_1639_v102	0.931
Listeria_monocytogenes_1639_v103	Listeria_monocytogenes_1639_v102	0.931
Listeria_monocytogenes_1639_v102	Listeria_monocytogenes_1639_v102	0.0695
Listeria_welshimeri_1643	Listeria_monocytogenes_1639_v103	0.8904
Listeria_welshimeri_serovar_6b_str_SLCC5334_386043	Listeria_monocytogenes_1639_v103	0.89
Listeria_seeligeri_serovar_1_2b_str_SLCC3954_683837	Listeria_monocytogenes_1639_v103	0.8872
Listeria_seeligeri_FSL_S4-171_702452	Listeria_monocytogenes_1639_v103	0.8276
Listeria_seeligeri_FSL_N1-067_702453	Listeria_monocytogenes_1639_v103	0.8296
Listeria_seeligeri_1640_v1	Listeria_monocytogenes_1639_v103	0.8941
Listeria_seeligeri_1640	Listeria_monocytogenes_1639_v103	0.8807
Listeria_monocytogenes_WSLC1042_1457188	Listeria_monocytogenes_1639_v103	0.9802
Listeria_monocytogenes_WSLC1001_1457187	Listeria_monocytogenes_1639_v103	0.9313
Listeria_monocytogenes_SLCC7179_879090	Listeria_monocytogenes_1639_v103	0.9379
Listeria_monocytogenes_SLCC5850_882096	Listeria_monocytogenes_1639_v103	0.9355
Listeria_monocytogenes_SLCC2755_932919	Listeria_monocytogenes_1639_v103	0.9448
Listeria_monocytogenes_SLCC2540_879089	Listeria_monocytogenes_1639_v103	0.948
Listeria_monocytogenes_SLCC2479_882020	Listeria_monocytogenes_1639_v103	0.9261
Listeria_monocytogenes_SLCC2378_879088	Listeria_monocytogenes_1639_v103	0.9817
Listeria_monocytogenes_SLCC2376_882097	Listeria_monocytogenes_1639_v103	0.9413
Listeria_monocytogenes_SLCC2372_932920	Listeria_monocytogenes_1639_v103	0.9188
Listeria_monocytogenes_serotype_4b_str_LL195_1230340	Listeria_monocytogenes_1639_v103	0.9766
Listeria_monocytogenes_serotype_4b_str_LIS0087_1134669	Listeria_monocytogenes_1639_v103	0.9122
Listeria_monocytogenes_serotype_4b_str_F2365_265669	Listeria_monocytogenes_1639_v103	0.9766
Listeria_monocytogenes_serotype_4b_str_CLIP_80459_568819	Listeria_monocytogenes_1639_v103	0.9719
Listeria_monocytogenes_serotype_4b_str_81-0861_1196161	Listeria_monocytogenes_1639_v103	0.9702
Listeria_monocytogenes_serotype_4b_str_81-0592_1196160	Listeria_monocytogenes_1639_v103	0.9713
Listeria_monocytogenes_serotype_4b_str_81-0558_1196159	Listeria_monocytogenes_1639_v103	0.9705
Listeria_monocytogenes_serotype_4b_str_10-0809_1196162	Listeria_monocytogenes_1639_v103	0.9713
Listeria_monocytogenes_serotype_4b_str_02-6680_1196175	Listeria_monocytogenes_1639_v103	0.9785
Listeria_monocytogenes_serotype_4b_str_02-6679_1196174	Listeria_monocytogenes_1639_v103	0.9785
Listeria_monocytogenes_serotype_4b_str_02-1792_1196173	Listeria_monocytogenes_1639_v103	0.9705
Listeria_monocytogenes_serotype_4b_str_02-1289_1196172	Listeria_monocytogenes_1639_v103	0.9705
Listeria_monocytogenes_serotype_4b_str_02-1103_1196171	Listeria_monocytogenes_1639_v103	0.9705
Listeria_monocytogenes_serotype_3c_str_10-5027_1196195	Listeria_monocytogenes_1639_v103	0.9253
Listeria_monocytogenes_serotype_1_2c_str_10-5026_1196194	Listeria_monocytogenes_1639_v103	0.9261
Listeria_monocytogenes_serotype_1_2c_str_10-5025_1196193	Listeria_monocytogenes_1639_v103	0.9261
Listeria_monocytogenes_serotype_1_2b_str_10-0811_1196164	Listeria_monocytogenes_1639_v103	0.9388
Listeria_monocytogenes_serotype_1_2b_str_10-0810_1196163	Listeria_monocytogenes_1639_v103	0.9388
Listeria_monocytogenes_serotype_1_2a_str_99-6370_1196179	Listeria_monocytogenes_1639_v103	0.9151
Listeria_monocytogenes_serotype_1_2a_str_98-2035_1196178	Listeria_monocytogenes_1639_v103	0.9151
Listeria_monocytogenes_serotype_1_2a_str_95-0093_1196177	Listeria_monocytogenes_1639_v103	0.9206
Listeria_monocytogenes_serotype_1_2a_str_88-0478_1196176	Listeria_monocytogenes_1639_v103	0.9194
Listeria_monocytogenes_serotype_1_2a_str_10-5024_1196192	Listeria_monocytogenes_1639_v103	0.9206

Listeria_monocytogenes_serotype_1_2a_str_10-4758_1196168	Listeria_monocytogenes_1639_v103	0.9387
Listeria_monocytogenes_serotype_1_2a_str_10-4754_1196167	Listeria_monocytogenes_1639_v103	0.939
Listeria_monocytogenes_serotype_1_2a_str_10-1321_1196191	Listeria_monocytogenes_1639_v103	0.9206
Listeria_monocytogenes_serotype_1_2a_str_10-1047_1196190	Listeria_monocytogenes_1639_v103	0.9213
Listeria_monocytogenes_serotype_1_2a_str_10-1046_1196189	Listeria_monocytogenes_1639_v103	0.9213
Listeria_monocytogenes_serotype_1_2a_str_10-0934_1196170	Listeria_monocytogenes_1639_v103	0.9456
Listeria_monocytogenes_serotype_1_2a_str_10-0933_1196169	Listeria_monocytogenes_1639_v103	0.9456
Listeria_monocytogenes_serotype_1_2a_str_10-0815_1196188	Listeria_monocytogenes_1639_v103	0.9165
Listeria_monocytogenes_serotype_1_2a_str_10-0814_1196187	Listeria_monocytogenes_1639_v103	0.921
Listeria_monocytogenes_serotype_1_2a_str_10-0813_1196166	Listeria_monocytogenes_1639_v103	0.9288
Listeria_monocytogenes_serotype_1_2a_str_10-0812_1196165	Listeria_monocytogenes_1639_v103	0.9288
Listeria_monocytogenes_serotype_1_2a_str_08-7669_1196186	Listeria_monocytogenes_1639_v103	0.93
Listeria_monocytogenes_serotype_1_2a_str_08-7374_1196185	Listeria_monocytogenes_1639_v103	0.9206
Listeria_monocytogenes_serotype_1_2a_str_08-6997_1196184	Listeria_monocytogenes_1639_v103	0.9165
Listeria_monocytogenes_serotype_1_2a_str_08-6569_1196183	Listeria_monocytogenes_1639_v103	0.9165
Listeria_monocytogenes_serotype_1_2a_str_08-6056_1196182	Listeria_monocytogenes_1639_v103	0.9165
Listeria_monocytogenes_serotype_1_2a_str_04-5457_1196181	Listeria_monocytogenes_1639_v103	0.9206
Listeria_monocytogenes_serotype_1_2a_str_02-5993_1196180	Listeria_monocytogenes_1639_v103	0.9206
Listeria_monocytogenes_R479a_1437838	Listeria_monocytogenes_1639_v103	0.9199
Listeria_monocytogenes_N53-1_1234142	Listeria_monocytogenes_1639_v103	0.8915
Listeria_monocytogenes_M7_1030009	Listeria_monocytogenes_1639_v103	0.9061
Listeria_monocytogenes_LO28_393132	Listeria_monocytogenes_1639_v103	0.9247
Listeria_monocytogenes_L99_563174	Listeria_monocytogenes_1639_v103	0.9065
Listeria_monocytogenes_L312_882094	Listeria_monocytogenes_1639_v103	0.9723
Listeria_monocytogenes_J1816_930781	Listeria_monocytogenes_1639_v103	0.9503
Listeria_monocytogenes_J1-220_930782	Listeria_monocytogenes_1639_v103	0.9783
Listeria_monocytogenes_J0161_393130	Listeria_monocytogenes_1639_v103	0.9277
Listeria_monocytogenes_HCC23_552536	Listeria_monocytogenes_1639_v103	0.9058
Listeria_monocytogenes_FSL_R2-561_393126	Listeria_monocytogenes_1639_v103	0.925
Listeria_monocytogenes_FSL_J1-208_393119	Listeria_monocytogenes_1639_v103	0.921
Listeria_monocytogenes_FSL_J1-175_393118	Listeria_monocytogenes_1639_v103	0.9554
Listeria_monocytogenes_Finland_1998_393127	Listeria_monocytogenes_1639_v103	0.9471
Listeria_monocytogenes_EGD-e_169963_v1	Listeria_monocytogenes_1639_v103	0.9346
Listeria_monocytogenes_EGD-e_169963	Listeria_monocytogenes_1639_v103	0.935
Listeria_monocytogenes_EGD_1334565	Listeria_monocytogenes_1639_v103	0.9363
Listeria_monocytogenes_CFSAN002349_1299895	Listeria_monocytogenes_1639_v103	0.972
Listeria_monocytogenes_ATCC_19117_882095_v1	Listeria_monocytogenes_1639_v103	0.9992
Listeria_monocytogenes_ATCC_19117_882095	Listeria_monocytogenes_1639_v103	0.9989
Listeria_monocytogenes_6179_1288295	Listeria_monocytogenes_1639_v103	0.9117
Listeria_monocytogenes_1639_v9	Listeria_monocytogenes_1639_v103	0.926
Listeria_monocytogenes_1639_v99	Listeria_monocytogenes_1639_v103	0.9507
Listeria_monocytogenes_1639_v98	Listeria_monocytogenes_1639_v103	0.9254
Listeria_monocytogenes_1639_v97	Listeria_monocytogenes_1639_v103	0.9659
Listeria_monocytogenes_1639_v96	Listeria_monocytogenes_1639_v103	0.95
Listeria_monocytogenes_1639_v95	Listeria_monocytogenes_1639_v103	0.95
Listeria_monocytogenes_1639_v94	Listeria_monocytogenes_1639_v103	0.9504
Listeria_monocytogenes_1639_v93	Listeria_monocytogenes_1639_v103	0.9243
Listeria_monocytogenes_1639_v92	Listeria_monocytogenes_1639_v103	0.9504
Listeria_monocytogenes_1639_v91	Listeria_monocytogenes_1639_v103	0.9571
Listeria_monocytogenes_1639_v90	Listeria_monocytogenes_1639_v103	0.9254
Listeria_monocytogenes_1639_v8	Listeria_monocytogenes_1639_v103	0.9119
Listeria_monocytogenes_1639_v89	Listeria_monocytogenes_1639_v103	0.9673
Listeria_monocytogenes_1639_v88	Listeria_monocytogenes_1639_v103	0.9263
Listeria_monocytogenes_1639_v87	Listeria_monocytogenes_1639_v103	0.9251
Listeria_monocytogenes_1639_v86	Listeria_monocytogenes_1639_v103	0.9247
Listeria_monocytogenes_1639_v85	Listeria_monocytogenes_1639_v103	0.9695
Listeria_monocytogenes_1639_v84	Listeria_monocytogenes_1639_v103	0.9318
Listeria_monocytogenes_1639_v83	Listeria_monocytogenes_1639_v103	0.9875
Listeria_monocytogenes_1639_v82	Listeria_monocytogenes_1639_v103	0.9868
Listeria_monocytogenes_1639_v81	Listeria_monocytogenes_1639_v103	0.9278
Listeria_monocytogenes_1639_v80	Listeria_monocytogenes_1639_v103	0.9451
Listeria_monocytogenes_1639_v7	Listeria_monocytogenes_1639_v103	0.9119
Listeria_monocytogenes_1639_v79	Listeria_monocytogenes_1639_v103	0.926
Listeria_monocytogenes_1639_v78	Listeria_monocytogenes_1639_v103	0.9271
Listeria_monocytogenes_1639_v77	Listeria_monocytogenes_1639_v103	0.9313
Listeria_monocytogenes_1639_v76	Listeria_monocytogenes_1639_v103	0.9227
Listeria_monocytogenes_1639_v75	Listeria_monocytogenes_1639_v103	0.9065
Listeria_monocytogenes_1639_v74	Listeria_monocytogenes_1639_v103	0.9236
Listeria_monocytogenes_1639_v73	Listeria_monocytogenes_1639_v103	0.9202
Listeria_monocytogenes_1639_v72	Listeria_monocytogenes_1639_v103	0.986
Listeria_monocytogenes_1639_v71	Listeria_monocytogenes_1639_v103	0.9118
Listeria_monocytogenes_1639_v70	Listeria_monocytogenes_1639_v103	0.9223
Listeria_monocytogenes_1639_v6	Listeria_monocytogenes_1639_v103	0.9116
Listeria_monocytogenes_1639_v69	Listeria_monocytogenes_1639_v103	0.9713
Listeria_monocytogenes_1639_v68	Listeria_monocytogenes_1639_v103	0.9414
Listeria_monocytogenes_1639_v67	Listeria_monocytogenes_1639_v103	0.9448
Listeria_monocytogenes_1639_v66	Listeria_monocytogenes_1639_v103	0.9327
Listeria_monocytogenes_1639_v65	Listeria_monocytogenes_1639_v103	0.9234
Listeria_monocytogenes_1639_v64	Listeria_monocytogenes_1639_v103	0.9268
Listeria_monocytogenes_1639_v63	Listeria_monocytogenes_1639_v103	0.9402
Listeria_monocytogenes_1639_v62	Listeria_monocytogenes_1639_v103	0.9232
Listeria_monocytogenes_1639_v61	Listeria_monocytogenes_1639_v103	0.9233
Listeria_monocytogenes_1639_v60	Listeria_monocytogenes_1639_v103	0.9297
Listeria_monocytogenes_1639_v5	Listeria_monocytogenes_1639_v103	0.9064
Listeria_monocytogenes_1639_v59	Listeria_monocytogenes_1639_v103	0.9879
Listeria_monocytogenes_1639_v58	Listeria_monocytogenes_1639_v103	0.9769
Listeria_monocytogenes_1639_v57	Listeria_monocytogenes_1639_v103	0.9472
Listeria_monocytogenes_1639_v56	Listeria_monocytogenes_1639_v103	0.9879
Listeria_monocytogenes_1639_v55	Listeria_monocytogenes_1639_v103	0.943
Listeria_monocytogenes_1639_v54	Listeria_monocytogenes_1639_v103	0.9257
Listeria_monocytogenes_1639_v53	Listeria_monocytogenes_1639_v103	0.93

Listeria_monocytogenes_1639_v52	Listeria_monocytogenes_1639_v103	0.9743
Listeria_monocytogenes_1639_v51	Listeria_monocytogenes_1639_v103	0.9816
Listeria_monocytogenes_1639_v50	Listeria_monocytogenes_1639_v103	0.9199
Listeria_monocytogenes_1639_v4	Listeria_monocytogenes_1639_v103	0.9197
Listeria_monocytogenes_1639_v49	Listeria_monocytogenes_1639_v103	0.9687
Listeria_monocytogenes_1639_v48	Listeria_monocytogenes_1639_v103	0.9205
Listeria_monocytogenes_1639_v47	Listeria_monocytogenes_1639_v103	0.9203
Listeria_monocytogenes_1639_v46	Listeria_monocytogenes_1639_v103	0.9407
Listeria_monocytogenes_1639_v45	Listeria_monocytogenes_1639_v103	0.9118
Listeria_monocytogenes_1639_v44	Listeria_monocytogenes_1639_v103	0.9206
Listeria_monocytogenes_1639_v43	Listeria_monocytogenes_1639_v103	0.9206
Listeria_monocytogenes_1639_v42	Listeria_monocytogenes_1639_v103	0.9147
Listeria_monocytogenes_1639_v41	Listeria_monocytogenes_1639_v103	0.9017
Listeria_monocytogenes_1639_v40	Listeria_monocytogenes_1639_v103	0.925
Listeria_monocytogenes_1639_v3	Listeria_monocytogenes_1639_v103	0.92
Listeria_monocytogenes_1639_v39	Listeria_monocytogenes_1639_v103	0.9439
Listeria_monocytogenes_1639_v38	Listeria_monocytogenes_1639_v103	0.9305
Listeria_monocytogenes_1639_v37	Listeria_monocytogenes_1639_v103	0.9164
Listeria_monocytogenes_1639_v36	Listeria_monocytogenes_1639_v103	0.918
Listeria_monocytogenes_1639_v35	Listeria_monocytogenes_1639_v103	0.9257
Listeria_monocytogenes_1639_v34	Listeria_monocytogenes_1639_v103	0.9333
Listeria_monocytogenes_1639_v33	Listeria_monocytogenes_1639_v103	0.9388
Listeria_monocytogenes_1639_v32	Listeria_monocytogenes_1639_v103	0.9153
Listeria_monocytogenes_1639_v31	Listeria_monocytogenes_1639_v103	0.9336
Listeria_monocytogenes_1639_v30	Listeria_monocytogenes_1639_v103	0.9436
Listeria_monocytogenes_1639_v2	Listeria_monocytogenes_1639_v103	0.9028
Listeria_monocytogenes_1639_v29	Listeria_monocytogenes_1639_v103	0.9131
Listeria_monocytogenes_1639_v28	Listeria_monocytogenes_1639_v103	0.9172
Listeria_monocytogenes_1639_v27	Listeria_monocytogenes_1639_v103	0.9084
Listeria_monocytogenes_1639_v26	Listeria_monocytogenes_1639_v103	0.9081
Listeria_monocytogenes_1639_v25	Listeria_monocytogenes_1639_v103	0.9081
Listeria_monocytogenes_1639_v24	Listeria_monocytogenes_1639_v103	0.9783
Listeria_monocytogenes_1639_v23	Listeria_monocytogenes_1639_v103	0.9779
Listeria_monocytogenes_1639_v22	Listeria_monocytogenes_1639_v103	0.941
Listeria_monocytogenes_1639_v21	Listeria_monocytogenes_1639_v103	0.9779
Listeria_monocytogenes_1639_v20	Listeria_monocytogenes_1639_v103	0.9313
Listeria_monocytogenes_1639_v1	Listeria_monocytogenes_1639_v103	0.9057
Listeria_monocytogenes_1639_v19	Listeria_monocytogenes_1639_v103	0.931
Listeria_monocytogenes_1639_v198	Listeria_monocytogenes_1639_v103	0.8939
Listeria_monocytogenes_1639_v197	Listeria_monocytogenes_1639_v103	0.9222
Listeria_monocytogenes_1639_v196	Listeria_monocytogenes_1639_v103	0.9189
Listeria_monocytogenes_1639_v195	Listeria_monocytogenes_1639_v103	0.9428
Listeria_monocytogenes_1639_v194	Listeria_monocytogenes_1639_v103	0.9417
Listeria_monocytogenes_1639_v193	Listeria_monocytogenes_1639_v103	0.9417
Listeria_monocytogenes_1639_v192	Listeria_monocytogenes_1639_v103	0.9147
Listeria_monocytogenes_1639_v191	Listeria_monocytogenes_1639_v103	0.9485
Listeria_monocytogenes_1639_v190	Listeria_monocytogenes_1639_v103	0.9482
Listeria_monocytogenes_1639_v18	Listeria_monocytogenes_1639_v103	0.9412
Listeria_monocytogenes_1639_v189	Listeria_monocytogenes_1639_v103	0.9471
Listeria_monocytogenes_1639_v188	Listeria_monocytogenes_1639_v103	0.9493
Listeria_monocytogenes_1639_v187	Listeria_monocytogenes_1639_v103	0.9482
Listeria_monocytogenes_1639_v186	Listeria_monocytogenes_1639_v103	0.9482
Listeria_monocytogenes_1639_v185	Listeria_monocytogenes_1639_v103	0.9486
Listeria_monocytogenes_1639_v184	Listeria_monocytogenes_1639_v103	0.9478
Listeria_monocytogenes_1639_v183	Listeria_monocytogenes_1639_v103	0.9428
Listeria_monocytogenes_1639_v182	Listeria_monocytogenes_1639_v103	0.945
Listeria_monocytogenes_1639_v181	Listeria_monocytogenes_1639_v103	0.9353
Listeria_monocytogenes_1639_v180	Listeria_monocytogenes_1639_v103	0.9353
Listeria_monocytogenes_1639_v17	Listeria_monocytogenes_1639_v103	0.9416
Listeria_monocytogenes_1639_v179	Listeria_monocytogenes_1639_v103	0.9326
Listeria_monocytogenes_1639_v178	Listeria_monocytogenes_1639_v103	0.9494
Listeria_monocytogenes_1639_v177	Listeria_monocytogenes_1639_v103	0.9697
Listeria_monocytogenes_1639_v176	Listeria_monocytogenes_1639_v103	0.9426
Listeria_monocytogenes_1639_v175	Listeria_monocytogenes_1639_v103	0.9376
Listeria_monocytogenes_1639_v174	Listeria_monocytogenes_1639_v103	0.9483
Listeria_monocytogenes_1639_v173	Listeria_monocytogenes_1639_v103	0.9649
Listeria_monocytogenes_1639_v172	Listeria_monocytogenes_1639_v103	0.9424
Listeria_monocytogenes_1639_v171	Listeria_monocytogenes_1639_v103	0.9342
Listeria_monocytogenes_1639_v170	Listeria_monocytogenes_1639_v103	0.942
Listeria_monocytogenes_1639_v16	Listeria_monocytogenes_1639_v103	0.9074
Listeria_monocytogenes_1639_v169	Listeria_monocytogenes_1639_v103	0.9425
Listeria_monocytogenes_1639_v168	Listeria_monocytogenes_1639_v103	0.9426
Listeria_monocytogenes_1639_v167	Listeria_monocytogenes_1639_v103	0.9351
Listeria_monocytogenes_1639_v166	Listeria_monocytogenes_1639_v103	0.9429
Listeria_monocytogenes_1639_v165	Listeria_monocytogenes_1639_v103	0.9421
Listeria_monocytogenes_1639_v164	Listeria_monocytogenes_1639_v103	0.9761
Listeria_monocytogenes_1639_v163	Listeria_monocytogenes_1639_v103	0.9589
Listeria_monocytogenes_1639_v162	Listeria_monocytogenes_1639_v103	0.9761
Listeria_monocytogenes_1639_v161	Listeria_monocytogenes_1639_v103	0.9765
Listeria_monocytogenes_1639_v160	Listeria_monocytogenes_1639_v103	0.9762
Listeria_monocytogenes_1639_v15	Listeria_monocytogenes_1639_v103	0.9247
Listeria_monocytogenes_1639_v159	Listeria_monocytogenes_1639_v103	0.9701
Listeria_monocytogenes_1639_v158	Listeria_monocytogenes_1639_v103	0.9766
Listeria_monocytogenes_1639_v157	Listeria_monocytogenes_1639_v103	0.9766
Listeria_monocytogenes_1639_v156	Listeria_monocytogenes_1639_v103	0.9253
Listeria_monocytogenes_1639_v155	Listeria_monocytogenes_1639_v103	0.9373
Listeria_monocytogenes_1639_v154	Listeria_monocytogenes_1639_v103	0.9389
Listeria_monocytogenes_1639_v153	Listeria_monocytogenes_1639_v103	0.938
Listeria_monocytogenes_1639_v152	Listeria_monocytogenes_1639_v103	0.9701
Listeria_monocytogenes_1639_v151	Listeria_monocytogenes_1639_v103	0.9701
Listeria_monocytogenes_1639_v150	Listeria_monocytogenes_1639_v103	0.938

Listeria_monocytogenes_1639_v14	Listeria_monocytogenes_1639_v103	0.9323
Listeria_monocytogenes_1639_v149	Listeria_monocytogenes_1639_v103	0.9366
Listeria_monocytogenes_1639_v148	Listeria_monocytogenes_1639_v103	0.969
Listeria_monocytogenes_1639_v147	Listeria_monocytogenes_1639_v103	0.9554
Listeria_monocytogenes_1639_v146	Listeria_monocytogenes_1639_v103	0.9468
Listeria_monocytogenes_1639_v145	Listeria_monocytogenes_1639_v103	0.9435
Listeria_monocytogenes_1639_v144	Listeria_monocytogenes_1639_v103	0.9715
Listeria_monocytogenes_1639_v143	Listeria_monocytogenes_1639_v103	0.9473
Listeria_monocytogenes_1639_v142	Listeria_monocytogenes_1639_v103	0.9719
Listeria_monocytogenes_1639_v141	Listeria_monocytogenes_1639_v103	0.9642
Listeria_monocytogenes_1639_v140	Listeria_monocytogenes_1639_v103	0.9727
Listeria_monocytogenes_1639_v13	Listeria_monocytogenes_1639_v103	0.9247
Listeria_monocytogenes_1639_v139	Listeria_monocytogenes_1639_v103	0.9727
Listeria_monocytogenes_1639_v138	Listeria_monocytogenes_1639_v103	0.9742
Listeria_monocytogenes_1639_v137	Listeria_monocytogenes_1639_v103	0.9742
Listeria_monocytogenes_1639_v136	Listeria_monocytogenes_1639_v103	0.9383
Listeria_monocytogenes_1639_v135	Listeria_monocytogenes_1639_v103	0.9204
Listeria_monocytogenes_1639_v134	Listeria_monocytogenes_1639_v103	0.9805
Listeria_monocytogenes_1639_v133	Listeria_monocytogenes_1639_v103	0.9354
Listeria_monocytogenes_1639_v132	Listeria_monocytogenes_1639_v103	0.9208
Listeria_monocytogenes_1639_v131	Listeria_monocytogenes_1639_v103	0.9578
Listeria_monocytogenes_1639_v130	Listeria_monocytogenes_1639_v103	0.9261
Listeria_monocytogenes_1639_v12	Listeria_monocytogenes_1639_v103	0.9071
Listeria_monocytogenes_1639_v129	Listeria_monocytogenes_1639_v103	0.9642
Listeria_monocytogenes_1639_v128	Listeria_monocytogenes_1639_v103	0.962
Listeria_monocytogenes_1639_v127	Listeria_monocytogenes_1639_v103	0.9346
Listeria_monocytogenes_1639_v126	Listeria_monocytogenes_1639_v103	0.9601
Listeria_monocytogenes_1639_v125	Listeria_monocytogenes_1639_v103	0.9343
Listeria_monocytogenes_1639_v124	Listeria_monocytogenes_1639_v103	0.9435
Listeria_monocytogenes_1639_v123	Listeria_monocytogenes_1639_v103	0.972
Listeria_monocytogenes_1639_v122	Listeria_monocytogenes_1639_v103	0.972
Listeria_monocytogenes_1639_v121	Listeria_monocytogenes_1639_v103	0.972
Listeria_monocytogenes_1639_v120	Listeria_monocytogenes_1639_v103	0.972
Listeria_monocytogenes_1639_v11	Listeria_monocytogenes_1639_v103	0.9084
Listeria_monocytogenes_1639_v119	Listeria_monocytogenes_1639_v103	0.972
Listeria_monocytogenes_1639_v118	Listeria_monocytogenes_1639_v103	0.972
Listeria_monocytogenes_1639_v117	Listeria_monocytogenes_1639_v103	0.9368
Listeria_monocytogenes_1639_v116	Listeria_monocytogenes_1639_v103	0.9817
Listeria_monocytogenes_1639_v115	Listeria_monocytogenes_1639_v103	0.9813
Listeria_monocytogenes_1639_v114	Listeria_monocytogenes_1639_v103	0.9299
Listeria_monocytogenes_1639_v113	Listeria_monocytogenes_1639_v103	0.9549
Listeria_monocytogenes_1639_v112	Listeria_monocytogenes_1639_v103	0.9654
Listeria_monocytogenes_1639_v111	Listeria_monocytogenes_1639_v103	0.9465
Listeria_monocytogenes_1639_v110	Listeria_monocytogenes_1639_v103	0.9296
Listeria_monocytogenes_1639_v10	Listeria_monocytogenes_1639_v103	0.923
Listeria_monocytogenes_1639_v109	Listeria_monocytogenes_1639_v103	0.9292
Listeria_monocytogenes_1639_v108	Listeria_monocytogenes_1639_v103	0.9591
Listeria_monocytogenes_1639_v107	Listeria_monocytogenes_1639_v103	0.9985
Listeria_monocytogenes_1639_v106	Listeria_monocytogenes_1639_v103	0.9996
Listeria_monocytogenes_1639_v105	Listeria_monocytogenes_1639_v103	0.9996
Listeria_monocytogenes_1639_v104	Listeria_monocytogenes_1639_v103	0.9996
Listeria_monocytogenes_1639_v103	Listeria_monocytogenes_1639_v103	0.0685
Listeria_welshimeri_1643	Listeria_monocytogenes_1639_v104	0.8904
Listeria_welshimeri_serovar_6b_str_SLCC5334_386043	Listeria_monocytogenes_1639_v104	0.8897
Listeria_seeligeri_serovar_1_2b_str_SLCC3954_683837	Listeria_monocytogenes_1639_v104	0.8869
Listeria_seeligeri_FSL_S4-171_702452	Listeria_monocytogenes_1639_v104	0.8276
Listeria_seeligeri_FSL_N1-067_702453	Listeria_monocytogenes_1639_v104	0.8293
Listeria_seeligeri_1640_v1	Listeria_monocytogenes_1639_v104	0.8937
Listeria_seeligeri_1640	Listeria_monocytogenes_1639_v104	0.8807
Listeria_monocytogenes_WSLC1042_1457188	Listeria_monocytogenes_1639_v104	0.9798
Listeria_monocytogenes_WSLC1001_1457187	Listeria_monocytogenes_1639_v104	0.9313
Listeria_monocytogenes_SLCC7179_879090	Listeria_monocytogenes_1639_v104	0.9379
Listeria_monocytogenes_SLCC5850_882096	Listeria_monocytogenes_1639_v104	0.9355
Listeria_monocytogenes_SLCC2755_932919	Listeria_monocytogenes_1639_v104	0.9448
Listeria_monocytogenes_SLCC2540_879089	Listeria_monocytogenes_1639_v104	0.948
Listeria_monocytogenes_SLCC2479_882020	Listeria_monocytogenes_1639_v104	0.9257
Listeria_monocytogenes_SLCC2378_879088	Listeria_monocytogenes_1639_v104	0.9817
Listeria_monocytogenes_SLCC2376_882097	Listeria_monocytogenes_1639_v104	0.9409
Listeria_monocytogenes_SLCC2372_932920	Listeria_monocytogenes_1639_v104	0.9185
Listeria_monocytogenes_serotype_4b_str_LL195_1230340	Listeria_monocytogenes_1639_v104	0.9766
Listeria_monocytogenes_serotype_4b_str_LIS0087_1134669	Listeria_monocytogenes_1639_v104	0.9119
Listeria_monocytogenes_serotype_4b_str_F2365_265669	Listeria_monocytogenes_1639_v104	0.9766
Listeria_monocytogenes_serotype_4b_str_CLIP_80459_568819	Listeria_monocytogenes_1639_v104	0.9719
Listeria_monocytogenes_serotype_4b_str_81-0861_1196161	Listeria_monocytogenes_1639_v104	0.9698
Listeria_monocytogenes_serotype_4b_str_81-0592_1196160	Listeria_monocytogenes_1639_v104	0.9709
Listeria_monocytogenes_serotype_4b_str_81-0558_1196159	Listeria_monocytogenes_1639_v104	0.9705
Listeria_monocytogenes_serotype_4b_str_10-0809_1196162	Listeria_monocytogenes_1639_v104	0.9709
Listeria_monocytogenes_serotype_4b_str_02-6680_1196175	Listeria_monocytogenes_1639_v104	0.9781
Listeria_monocytogenes_serotype_4b_str_02-6679_1196174	Listeria_monocytogenes_1639_v104	0.9781
Listeria_monocytogenes_serotype_4b_str_02-1792_1196173	Listeria_monocytogenes_1639_v104	0.9705
Listeria_monocytogenes_serotype_4b_str_02-1289_1196172	Listeria_monocytogenes_1639_v104	0.9705
Listeria_monocytogenes_serotype_4b_str_02-1103_1196171	Listeria_monocytogenes_1639_v104	0.9705
Listeria_monocytogenes_serotype_3c_str_10-5027_1196195	Listeria_monocytogenes_1639_v104	0.925
Listeria_monocytogenes_serotype_1_2c_str_10-5026_1196194	Listeria_monocytogenes_1639_v104	0.9261
Listeria_monocytogenes_serotype_1_2c_str_10-5025_1196193	Listeria_monocytogenes_1639_v104	0.9261
Listeria_monocytogenes_serotype_1_2b_str_10-0811_1196164	Listeria_monocytogenes_1639_v104	0.9384
Listeria_monocytogenes_serotype_1_2b_str_10-0810_1196163	Listeria_monocytogenes_1639_v104	0.9384
Listeria_monocytogenes_serotype_1_2a_str_99-6370_1196179	Listeria_monocytogenes_1639_v104	0.9148
Listeria_monocytogenes_serotype_1_2a_str_98-2035_1196178	Listeria_monocytogenes_1639_v104	0.9148
Listeria_monocytogenes_serotype_1_2a_str_95-0093_1196177	Listeria_monocytogenes_1639_v104	0.9203
Listeria_monocytogenes_serotype_1_2a_str_88-0478_1196176	Listeria_monocytogenes_1639_v104	0.9194

Listeria_monocytogenes_serotype_1_2a_str_10-5024_1196192	Listeria_monocytogenes_1639_v104	0.9206
Listeria_monocytogenes_serotype_1_2a_str_10-4758_1196168	Listeria_monocytogenes_1639_v104	0.9387
Listeria_monocytogenes_serotype_1_2a_str_10-4754_1196167	Listeria_monocytogenes_1639_v104	0.9387
Listeria_monocytogenes_serotype_1_2a_str_10-1321_1196191	Listeria_monocytogenes_1639_v104	0.9206
Listeria_monocytogenes_serotype_1_2a_str_10-1047_1196190	Listeria_monocytogenes_1639_v104	0.9209
Listeria_monocytogenes_serotype_1_2a_str_10-1046_1196189	Listeria_monocytogenes_1639_v104	0.9209
Listeria_monocytogenes_serotype_1_2a_str_10-0934_1196170	Listeria_monocytogenes_1639_v104	0.9452
Listeria_monocytogenes_serotype_1_2a_str_10-0933_1196169	Listeria_monocytogenes_1639_v104	0.9452
Listeria_monocytogenes_serotype_1_2a_str_10-0815_1196188	Listeria_monocytogenes_1639_v104	0.9162
Listeria_monocytogenes_serotype_1_2a_str_10-0814_1196187	Listeria_monocytogenes_1639_v104	0.9206
Listeria_monocytogenes_serotype_1_2a_str_10-0813_1196166	Listeria_monocytogenes_1639_v104	0.9285
Listeria_monocytogenes_serotype_1_2a_str_10-0812_1196165	Listeria_monocytogenes_1639_v104	0.9285
Listeria_monocytogenes_serotype_1_2a_str_08-7669_1196186	Listeria_monocytogenes_1639_v104	0.9296
Listeria_monocytogenes_serotype_1_2a_str_08-7374_1196185	Listeria_monocytogenes_1639_v104	0.9206
Listeria_monocytogenes_serotype_1_2a_str_08-6997_1196184	Listeria_monocytogenes_1639_v104	0.9162
Listeria_monocytogenes_serotype_1_2a_str_08-6569_1196183	Listeria_monocytogenes_1639_v104	0.9162
Listeria_monocytogenes_serotype_1_2a_str_08-6056_1196182	Listeria_monocytogenes_1639_v104	0.9162
Listeria_monocytogenes_serotype_1_2a_str_04-5457_1196181	Listeria_monocytogenes_1639_v104	0.9206
Listeria_monocytogenes_serotype_1_2a_str_02-5993_1196180	Listeria_monocytogenes_1639_v104	0.9203
Listeria_monocytogenes_R479a_1437838	Listeria_monocytogenes_1639_v104	0.9196
Listeria_monocytogenes_N53-1_1234142	Listeria_monocytogenes_1639_v104	0.8912
Listeria_monocytogenes_M7_1030009	Listeria_monocytogenes_1639_v104	0.9058
Listeria_monocytogenes_LO28_393132	Listeria_monocytogenes_1639_v104	0.9244
Listeria_monocytogenes_L99_563174	Listeria_monocytogenes_1639_v104	0.9065
Listeria_monocytogenes_L312_882094	Listeria_monocytogenes_1639_v104	0.9723
Listeria_monocytogenes_J1816_930781	Listeria_monocytogenes_1639_v104	0.9503
Listeria_monocytogenes_J1-220_930782	Listeria_monocytogenes_1639_v104	0.9779
Listeria_monocytogenes_J0161_393130	Listeria_monocytogenes_1639_v104	0.9277
Listeria_monocytogenes_HCC23_552536	Listeria_monocytogenes_1639_v104	0.9058
Listeria_monocytogenes_FSL_R2-561_393126	Listeria_monocytogenes_1639_v104	0.9247
Listeria_monocytogenes_FSL_J1-208_393119	Listeria_monocytogenes_1639_v104	0.9206
Listeria_monocytogenes_FSL_J1-175_393118	Listeria_monocytogenes_1639_v104	0.955
Listeria_monocytogenes_Finland_1998_393127	Listeria_monocytogenes_1639_v104	0.9467
Listeria_monocytogenes_EGD-e_169963_v1	Listeria_monocytogenes_1639_v104	0.9342
Listeria_monocytogenes_EGD-e_169963	Listeria_monocytogenes_1639_v104	0.9346
Listeria_monocytogenes_EGD_1334565	Listeria_monocytogenes_1639_v104	0.9363
Listeria_monocytogenes_CFSAN002349_1299895	Listeria_monocytogenes_1639_v104	0.9716
Listeria_monocytogenes_ATCC_19117_882095_v1	Listeria_monocytogenes_1639_v104	0.9989
Listeria_monocytogenes_ATCC_19117_882095	Listeria_monocytogenes_1639_v104	0.9989
Listeria_monocytogenes_6179_1288295	Listeria_monocytogenes_1639_v104	0.9117
Listeria_monocytogenes_1639_v9	Listeria_monocytogenes_1639_v104	0.9256
Listeria_monocytogenes_1639_v99	Listeria_monocytogenes_1639_v104	0.9507
Listeria_monocytogenes_1639_v98	Listeria_monocytogenes_1639_v104	0.9254
Listeria_monocytogenes_1639_v97	Listeria_monocytogenes_1639_v104	0.9655
Listeria_monocytogenes_1639_v96	Listeria_monocytogenes_1639_v104	0.95
Listeria_monocytogenes_1639_v95	Listeria_monocytogenes_1639_v104	0.95
Listeria_monocytogenes_1639_v94	Listeria_monocytogenes_1639_v104	0.95
Listeria_monocytogenes_1639_v93	Listeria_monocytogenes_1639_v104	0.9243
Listeria_monocytogenes_1639_v92	Listeria_monocytogenes_1639_v104	0.9504
Listeria_monocytogenes_1639_v91	Listeria_monocytogenes_1639_v104	0.9571
Listeria_monocytogenes_1639_v90	Listeria_monocytogenes_1639_v104	0.9254
Listeria_monocytogenes_1639_v8	Listeria_monocytogenes_1639_v104	0.9119
Listeria_monocytogenes_1639_v89	Listeria_monocytogenes_1639_v104	0.9669
Listeria_monocytogenes_1639_v88	Listeria_monocytogenes_1639_v104	0.9263
Listeria_monocytogenes_1639_v87	Listeria_monocytogenes_1639_v104	0.9251
Listeria_monocytogenes_1639_v86	Listeria_monocytogenes_1639_v104	0.9244
Listeria_monocytogenes_1639_v85	Listeria_monocytogenes_1639_v104	0.9691
Listeria_monocytogenes_1639_v84	Listeria_monocytogenes_1639_v104	0.9318
Listeria_monocytogenes_1639_v83	Listeria_monocytogenes_1639_v104	0.9871
Listeria_monocytogenes_1639_v82	Listeria_monocytogenes_1639_v104	0.9864
Listeria_monocytogenes_1639_v81	Listeria_monocytogenes_1639_v104	0.9278
Listeria_monocytogenes_1639_v80	Listeria_monocytogenes_1639_v104	0.9451
Listeria_monocytogenes_1639_v7	Listeria_monocytogenes_1639_v104	0.9119
Listeria_monocytogenes_1639_v79	Listeria_monocytogenes_1639_v104	0.9257
Listeria_monocytogenes_1639_v78	Listeria_monocytogenes_1639_v104	0.9271
Listeria_monocytogenes_1639_v77	Listeria_monocytogenes_1639_v104	0.931
Listeria_monocytogenes_1639_v76	Listeria_monocytogenes_1639_v104	0.9224
Listeria_monocytogenes_1639_v75	Listeria_monocytogenes_1639_v104	0.9065
Listeria_monocytogenes_1639_v74	Listeria_monocytogenes_1639_v104	0.9232
Listeria_monocytogenes_1639_v73	Listeria_monocytogenes_1639_v104	0.9199
Listeria_monocytogenes_1639_v72	Listeria_monocytogenes_1639_v104	0.986
Listeria_monocytogenes_1639_v71	Listeria_monocytogenes_1639_v104	0.9115
Listeria_monocytogenes_1639_v70	Listeria_monocytogenes_1639_v104	0.9223
Listeria_monocytogenes_1639_v6	Listeria_monocytogenes_1639_v104	0.9113
Listeria_monocytogenes_1639_v69	Listeria_monocytogenes_1639_v104	0.9713
Listeria_monocytogenes_1639_v68	Listeria_monocytogenes_1639_v104	0.9414
Listeria_monocytogenes_1639_v67	Listeria_monocytogenes_1639_v104	0.9448
Listeria_monocytogenes_1639_v66	Listeria_monocytogenes_1639_v104	0.9327
Listeria_monocytogenes_1639_v65	Listeria_monocytogenes_1639_v104	0.9234
Listeria_monocytogenes_1639_v64	Listeria_monocytogenes_1639_v104	0.9268
Listeria_monocytogenes_1639_v63	Listeria_monocytogenes_1639_v104	0.9399
Listeria_monocytogenes_1639_v62	Listeria_monocytogenes_1639_v104	0.9232
Listeria_monocytogenes_1639_v61	Listeria_monocytogenes_1639_v104	0.9233
Listeria_monocytogenes_1639_v60	Listeria_monocytogenes_1639_v104	0.9297
Listeria_monocytogenes_1639_v5	Listeria_monocytogenes_1639_v104	0.9064
Listeria_monocytogenes_1639_v59	Listeria_monocytogenes_1639_v104	0.9879
Listeria_monocytogenes_1639_v58	Listeria_monocytogenes_1639_v104	0.9765
Listeria_monocytogenes_1639_v57	Listeria_monocytogenes_1639_v104	0.9472
Listeria_monocytogenes_1639_v56	Listeria_monocytogenes_1639_v104	0.9875
Listeria_monocytogenes_1639_v55	Listeria_monocytogenes_1639_v104	0.943
Listeria_monocytogenes_1639_v54	Listeria_monocytogenes_1639_v104	0.9253

Listeria_monocytogenes_1639_v53	Listeria_monocytogenes_1639_v104	0.9297
Listeria_monocytogenes_1639_v52	Listeria_monocytogenes_1639_v104	0.974
Listeria_monocytogenes_1639_v51	Listeria_monocytogenes_1639_v104	0.9816
Listeria_monocytogenes_1639_v50	Listeria_monocytogenes_1639_v104	0.9199
Listeria_monocytogenes_1639_v4	Listeria_monocytogenes_1639_v104	0.9193
Listeria_monocytogenes_1639_v49	Listeria_monocytogenes_1639_v104	0.9687
Listeria_monocytogenes_1639_v48	Listeria_monocytogenes_1639_v104	0.9202
Listeria_monocytogenes_1639_v47	Listeria_monocytogenes_1639_v104	0.9203
Listeria_monocytogenes_1639_v46	Listeria_monocytogenes_1639_v104	0.9407
Listeria_monocytogenes_1639_v45	Listeria_monocytogenes_1639_v104	0.9114
Listeria_monocytogenes_1639_v44	Listeria_monocytogenes_1639_v104	0.9206
Listeria_monocytogenes_1639_v43	Listeria_monocytogenes_1639_v104	0.9206
Listeria_monocytogenes_1639_v42	Listeria_monocytogenes_1639_v104	0.9144
Listeria_monocytogenes_1639_v41	Listeria_monocytogenes_1639_v104	0.9017
Listeria_monocytogenes_1639_v40	Listeria_monocytogenes_1639_v104	0.9247
Listeria_monocytogenes_1639_v3	Listeria_monocytogenes_1639_v104	0.9197
Listeria_monocytogenes_1639_v39	Listeria_monocytogenes_1639_v104	0.9436
Listeria_monocytogenes_1639_v38	Listeria_monocytogenes_1639_v104	0.9302
Listeria_monocytogenes_1639_v37	Listeria_monocytogenes_1639_v104	0.9164
Listeria_monocytogenes_1639_v36	Listeria_monocytogenes_1639_v104	0.9176
Listeria_monocytogenes_1639_v35	Listeria_monocytogenes_1639_v104	0.9254
Listeria_monocytogenes_1639_v34	Listeria_monocytogenes_1639_v104	0.9333
Listeria_monocytogenes_1639_v33	Listeria_monocytogenes_1639_v104	0.9384
Listeria_monocytogenes_1639_v32	Listeria_monocytogenes_1639_v104	0.9153
Listeria_monocytogenes_1639_v31	Listeria_monocytogenes_1639_v104	0.9336
Listeria_monocytogenes_1639_v30	Listeria_monocytogenes_1639_v104	0.9436
Listeria_monocytogenes_1639_v2	Listeria_monocytogenes_1639_v104	0.9028
Listeria_monocytogenes_1639_v29	Listeria_monocytogenes_1639_v104	0.9128
Listeria_monocytogenes_1639_v28	Listeria_monocytogenes_1639_v104	0.9168
Listeria_monocytogenes_1639_v27	Listeria_monocytogenes_1639_v104	0.9081
Listeria_monocytogenes_1639_v26	Listeria_monocytogenes_1639_v104	0.9081
Listeria_monocytogenes_1639_v25	Listeria_monocytogenes_1639_v104	0.9078
Listeria_monocytogenes_1639_v24	Listeria_monocytogenes_1639_v104	0.9779
Listeria_monocytogenes_1639_v23	Listeria_monocytogenes_1639_v104	0.9779
Listeria_monocytogenes_1639_v22	Listeria_monocytogenes_1639_v104	0.941
Listeria_monocytogenes_1639_v21	Listeria_monocytogenes_1639_v104	0.9775
Listeria_monocytogenes_1639_v20	Listeria_monocytogenes_1639_v104	0.931
Listeria_monocytogenes_1639_v1	Listeria_monocytogenes_1639_v104	0.9054
Listeria_monocytogenes_1639_v19	Listeria_monocytogenes_1639_v104	0.9306
Listeria_monocytogenes_1639_v198	Listeria_monocytogenes_1639_v104	0.8939
Listeria_monocytogenes_1639_v197	Listeria_monocytogenes_1639_v104	0.9222
Listeria_monocytogenes_1639_v196	Listeria_monocytogenes_1639_v104	0.9189
Listeria_monocytogenes_1639_v195	Listeria_monocytogenes_1639_v104	0.9424
Listeria_monocytogenes_1639_v194	Listeria_monocytogenes_1639_v104	0.9417
Listeria_monocytogenes_1639_v193	Listeria_monocytogenes_1639_v104	0.9417
Listeria_monocytogenes_1639_v192	Listeria_monocytogenes_1639_v104	0.9143
Listeria_monocytogenes_1639_v191	Listeria_monocytogenes_1639_v104	0.9485
Listeria_monocytogenes_1639_v190	Listeria_monocytogenes_1639_v104	0.9482
Listeria_monocytogenes_1639_v18	Listeria_monocytogenes_1639_v104	0.9412
Listeria_monocytogenes_1639_v189	Listeria_monocytogenes_1639_v104	0.9471
Listeria_monocytogenes_1639_v188	Listeria_monocytogenes_1639_v104	0.9493
Listeria_monocytogenes_1639_v187	Listeria_monocytogenes_1639_v104	0.9478
Listeria_monocytogenes_1639_v186	Listeria_monocytogenes_1639_v104	0.9482
Listeria_monocytogenes_1639_v185	Listeria_monocytogenes_1639_v104	0.9482
Listeria_monocytogenes_1639_v184	Listeria_monocytogenes_1639_v104	0.9478
Listeria_monocytogenes_1639_v183	Listeria_monocytogenes_1639_v104	0.9424
Listeria_monocytogenes_1639_v182	Listeria_monocytogenes_1639_v104	0.9446
Listeria_monocytogenes_1639_v181	Listeria_monocytogenes_1639_v104	0.935
Listeria_monocytogenes_1639_v180	Listeria_monocytogenes_1639_v104	0.935
Listeria_monocytogenes_1639_v17	Listeria_monocytogenes_1639_v104	0.9412
Listeria_monocytogenes_1639_v179	Listeria_monocytogenes_1639_v104	0.9326
Listeria_monocytogenes_1639_v178	Listeria_monocytogenes_1639_v104	0.9494
Listeria_monocytogenes_1639_v177	Listeria_monocytogenes_1639_v104	0.9693
Listeria_monocytogenes_1639_v176	Listeria_monocytogenes_1639_v104	0.9426
Listeria_monocytogenes_1639_v175	Listeria_monocytogenes_1639_v104	0.9376
Listeria_monocytogenes_1639_v174	Listeria_monocytogenes_1639_v104	0.9483
Listeria_monocytogenes_1639_v173	Listeria_monocytogenes_1639_v104	0.9649
Listeria_monocytogenes_1639_v172	Listeria_monocytogenes_1639_v104	0.942
Listeria_monocytogenes_1639_v171	Listeria_monocytogenes_1639_v104	0.9338
Listeria_monocytogenes_1639_v170	Listeria_monocytogenes_1639_v104	0.9417
Listeria_monocytogenes_1639_v16	Listeria_monocytogenes_1639_v104	0.9074
Listeria_monocytogenes_1639_v169	Listeria_monocytogenes_1639_v104	0.9421
Listeria_monocytogenes_1639_v168	Listeria_monocytogenes_1639_v104	0.9426
Listeria_monocytogenes_1639_v167	Listeria_monocytogenes_1639_v104	0.9351
Listeria_monocytogenes_1639_v166	Listeria_monocytogenes_1639_v104	0.9429
Listeria_monocytogenes_1639_v165	Listeria_monocytogenes_1639_v104	0.9418
Listeria_monocytogenes_1639_v164	Listeria_monocytogenes_1639_v104	0.9758
Listeria_monocytogenes_1639_v163	Listeria_monocytogenes_1639_v104	0.9589
Listeria_monocytogenes_1639_v162	Listeria_monocytogenes_1639_v104	0.9758
Listeria_monocytogenes_1639_v161	Listeria_monocytogenes_1639_v104	0.9762
Listeria_monocytogenes_1639_v160	Listeria_monocytogenes_1639_v104	0.9762
Listeria_monocytogenes_1639_v15	Listeria_monocytogenes_1639_v104	0.9243
Listeria_monocytogenes_1639_v159	Listeria_monocytogenes_1639_v104	0.9697
Listeria_monocytogenes_1639_v158	Listeria_monocytogenes_1639_v104	0.9766
Listeria_monocytogenes_1639_v157	Listeria_monocytogenes_1639_v104	0.9766
Listeria_monocytogenes_1639_v156	Listeria_monocytogenes_1639_v104	0.9249
Listeria_monocytogenes_1639_v155	Listeria_monocytogenes_1639_v104	0.9373
Listeria_monocytogenes_1639_v154	Listeria_monocytogenes_1639_v104	0.9389
Listeria_monocytogenes_1639_v153	Listeria_monocytogenes_1639_v104	0.938
Listeria_monocytogenes_1639_v152	Listeria_monocytogenes_1639_v104	0.9701
Listeria_monocytogenes_1639_v151	Listeria_monocytogenes_1639_v104	0.9701

Listeria_monocytogenes_1639_v150	Listeria_monocytogenes_1639_v104	0.9377
Listeria_monocytogenes_1639_v14	Listeria_monocytogenes_1639_v104	0.9323
Listeria_monocytogenes_1639_v149	Listeria_monocytogenes_1639_v104	0.9363
Listeria_monocytogenes_1639_v148	Listeria_monocytogenes_1639_v104	0.9686
Listeria_monocytogenes_1639_v147	Listeria_monocytogenes_1639_v104	0.9551
Listeria_monocytogenes_1639_v146	Listeria_monocytogenes_1639_v104	0.9468
Listeria_monocytogenes_1639_v145	Listeria_monocytogenes_1639_v104	0.9431
Listeria_monocytogenes_1639_v144	Listeria_monocytogenes_1639_v104	0.9715
Listeria_monocytogenes_1639_v143	Listeria_monocytogenes_1639_v104	0.9473
Listeria_monocytogenes_1639_v142	Listeria_monocytogenes_1639_v104	0.9719
Listeria_monocytogenes_1639_v141	Listeria_monocytogenes_1639_v104	0.9642
Listeria_monocytogenes_1639_v140	Listeria_monocytogenes_1639_v104	0.9723
Listeria_monocytogenes_1639_v13	Listeria_monocytogenes_1639_v104	0.9247
Listeria_monocytogenes_1639_v139	Listeria_monocytogenes_1639_v104	0.9724
Listeria_monocytogenes_1639_v138	Listeria_monocytogenes_1639_v104	0.9739
Listeria_monocytogenes_1639_v137	Listeria_monocytogenes_1639_v104	0.9739
Listeria_monocytogenes_1639_v136	Listeria_monocytogenes_1639_v104	0.938
Listeria_monocytogenes_1639_v135	Listeria_monocytogenes_1639_v104	0.92
Listeria_monocytogenes_1639_v134	Listeria_monocytogenes_1639_v104	0.9805
Listeria_monocytogenes_1639_v133	Listeria_monocytogenes_1639_v104	0.9351
Listeria_monocytogenes_1639_v132	Listeria_monocytogenes_1639_v104	0.9208
Listeria_monocytogenes_1639_v131	Listeria_monocytogenes_1639_v104	0.9578
Listeria_monocytogenes_1639_v130	Listeria_monocytogenes_1639_v104	0.9261
Listeria_monocytogenes_1639_v12	Listeria_monocytogenes_1639_v104	0.9068
Listeria_monocytogenes_1639_v129	Listeria_monocytogenes_1639_v104	0.9639
Listeria_monocytogenes_1639_v128	Listeria_monocytogenes_1639_v104	0.9617
Listeria_monocytogenes_1639_v127	Listeria_monocytogenes_1639_v104	0.9342
Listeria_monocytogenes_1639_v126	Listeria_monocytogenes_1639_v104	0.9601
Listeria_monocytogenes_1639_v125	Listeria_monocytogenes_1639_v104	0.9343
Listeria_monocytogenes_1639_v124	Listeria_monocytogenes_1639_v104	0.9435
Listeria_monocytogenes_1639_v123	Listeria_monocytogenes_1639_v104	0.9716
Listeria_monocytogenes_1639_v122	Listeria_monocytogenes_1639_v104	0.972
Listeria_monocytogenes_1639_v121	Listeria_monocytogenes_1639_v104	0.972
Listeria_monocytogenes_1639_v120	Listeria_monocytogenes_1639_v104	0.9716
Listeria_monocytogenes_1639_v11	Listeria_monocytogenes_1639_v104	0.9084
Listeria_monocytogenes_1639_v119	Listeria_monocytogenes_1639_v104	0.972
Listeria_monocytogenes_1639_v118	Listeria_monocytogenes_1639_v104	0.972
Listeria_monocytogenes_1639_v117	Listeria_monocytogenes_1639_v104	0.9368
Listeria_monocytogenes_1639_v116	Listeria_monocytogenes_1639_v104	0.9813
Listeria_monocytogenes_1639_v115	Listeria_monocytogenes_1639_v104	0.9809
Listeria_monocytogenes_1639_v114	Listeria_monocytogenes_1639_v104	0.9299
Listeria_monocytogenes_1639_v113	Listeria_monocytogenes_1639_v104	0.9549
Listeria_monocytogenes_1639_v112	Listeria_monocytogenes_1639_v104	0.965
Listeria_monocytogenes_1639_v111	Listeria_monocytogenes_1639_v104	0.9465
Listeria_monocytogenes_1639_v110	Listeria_monocytogenes_1639_v104	0.9296
Listeria_monocytogenes_1639_v10	Listeria_monocytogenes_1639_v104	0.9226
Listeria_monocytogenes_1639_v109	Listeria_monocytogenes_1639_v104	0.9288
Listeria_monocytogenes_1639_v108	Listeria_monocytogenes_1639_v104	0.9587
Listeria_monocytogenes_1639_v107	Listeria_monocytogenes_1639_v104	0.9981
Listeria_monocytogenes_1639_v106	Listeria_monocytogenes_1639_v104	0.9992
Listeria_monocytogenes_1639_v105	Listeria_monocytogenes_1639_v104	0.9992
Listeria_monocytogenes_1639_v104	Listeria_monocytogenes_1639_v104	0.0685
Listeria_welshimeri_1643	Listeria_monocytogenes_1639_v105	0.8904
Listeria_welshimeri_serovar_6b_str_SLCC5334_386043	Listeria_monocytogenes_1639_v105	0.8901
Listeria_seeligeri_serovar_1_2b_str_SLCC3954_683837	Listeria_monocytogenes_1639_v105	0.8872
Listeria_seeligeri_FSL_S4-171_702452	Listeria_monocytogenes_1639_v105	0.8277
Listeria_seeligeri_FSL_N1-067_702453	Listeria_monocytogenes_1639_v105	0.8297
Listeria_seeligeri_1640_v1	Listeria_monocytogenes_1639_v105	0.8941
Listeria_seeligeri_1640	Listeria_monocytogenes_1639_v105	0.8807
Listeria_monocytogenes_WSLC1042_1457188	Listeria_monocytogenes_1639_v105	0.9798
Listeria_monocytogenes_WSLC1001_1457187	Listeria_monocytogenes_1639_v105	0.9309
Listeria_monocytogenes_SLCC7179_879090	Listeria_monocytogenes_1639_v105	0.9376
Listeria_monocytogenes_SLCC5850_882096	Listeria_monocytogenes_1639_v105	0.9352
Listeria_monocytogenes_SLCC2755_932919	Listeria_monocytogenes_1639_v105	0.9444
Listeria_monocytogenes_SLCC2540_879089	Listeria_monocytogenes_1639_v105	0.9476
Listeria_monocytogenes_SLCC2479_882020	Listeria_monocytogenes_1639_v105	0.9257
Listeria_monocytogenes_SLCC2378_879088	Listeria_monocytogenes_1639_v105	0.9813
Listeria_monocytogenes_SLCC2376_882097	Listeria_monocytogenes_1639_v105	0.9409
Listeria_monocytogenes_SLCC2372_932920	Listeria_monocytogenes_1639_v105	0.9185
Listeria_monocytogenes_serotype_4b_str_LL195_1230340	Listeria_monocytogenes_1639_v105	0.9762
Listeria_monocytogenes_serotype_4b_str_LIS0087_1134669	Listeria_monocytogenes_1639_v105	0.9119
Listeria_monocytogenes_serotype_4b_str_F2365_265669	Listeria_monocytogenes_1639_v105	0.9762
Listeria_monocytogenes_serotype_4b_str_CLIP_80459_568819	Listeria_monocytogenes_1639_v105	0.9715
Listeria_monocytogenes_serotype_4b_str_81-0861_1196161	Listeria_monocytogenes_1639_v105	0.9698
Listeria_monocytogenes_serotype_4b_str_81-0592_1196160	Listeria_monocytogenes_1639_v105	0.9709
Listeria_monocytogenes_serotype_4b_str_81-0558_1196159	Listeria_monocytogenes_1639_v105	0.9702
Listeria_monocytogenes_serotype_4b_str_10-0809_1196162	Listeria_monocytogenes_1639_v105	0.9709
Listeria_monocytogenes_serotype_4b_str_02-6680_1196175	Listeria_monocytogenes_1639_v105	0.9785
Listeria_monocytogenes_serotype_4b_str_02-6679_1196174	Listeria_monocytogenes_1639_v105	0.9785
Listeria_monocytogenes_serotype_4b_str_02-1792_1196173	Listeria_monocytogenes_1639_v105	0.9702
Listeria_monocytogenes_serotype_4b_str_02-1289_1196172	Listeria_monocytogenes_1639_v105	0.9702
Listeria_monocytogenes_serotype_4b_str_02-1103_1196171	Listeria_monocytogenes_1639_v105	0.9702
Listeria_monocytogenes_serotype_3c_str_10-5027_1196195	Listeria_monocytogenes_1639_v105	0.925
Listeria_monocytogenes_serotype_1_2c_str_10-5026_1196194	Listeria_monocytogenes_1639_v105	0.9257
Listeria_monocytogenes_serotype_1_2c_str_10-5025_1196193	Listeria_monocytogenes_1639_v105	0.9257
Listeria_monocytogenes_serotype_1_2b_str_10-0811_1196164	Listeria_monocytogenes_1639_v105	0.9384
Listeria_monocytogenes_serotype_1_2b_str_10-0810_1196163	Listeria_monocytogenes_1639_v105	0.9384
Listeria_monocytogenes_serotype_1_2a_str_99-6370_1196179	Listeria_monocytogenes_1639_v105	0.9148
Listeria_monocytogenes_serotype_1_2a_str_98-2035_1196178	Listeria_monocytogenes_1639_v105	0.9148
Listeria_monocytogenes_serotype_1_2a_str_95-0093_1196177	Listeria_monocytogenes_1639_v105	0.9203
Listeria_monocytogenes_serotype_1_2a_str_88-0478_1196176	Listeria_monocytogenes_1639_v105	0.9191

Listeria_monocytogenes_serotype_1_2a_str_10-5024_1196192	Listeria_monocytogenes_1639_v105	0.9203
Listeria_monocytogenes_serotype_1_2a_str_10-4758_1196168	Listeria_monocytogenes_1639_v105	0.9383
Listeria_monocytogenes_serotype_1_2a_str_10-4754_1196167	Listeria_monocytogenes_1639_v105	0.9387
Listeria_monocytogenes_serotype_1_2a_str_10-1321_1196191	Listeria_monocytogenes_1639_v105	0.9203
Listeria_monocytogenes_serotype_1_2a_str_10-1047_1196190	Listeria_monocytogenes_1639_v105	0.9209
Listeria_monocytogenes_serotype_1_2a_str_10-1046_1196189	Listeria_monocytogenes_1639_v105	0.9209
Listeria_monocytogenes_serotype_1_2a_str_10-0934_1196170	Listeria_monocytogenes_1639_v105	0.9452
Listeria_monocytogenes_serotype_1_2a_str_10-0933_1196169	Listeria_monocytogenes_1639_v105	0.9452
Listeria_monocytogenes_serotype_1_2a_str_10-0815_1196188	Listeria_monocytogenes_1639_v105	0.9162
Listeria_monocytogenes_serotype_1_2a_str_10-0814_1196187	Listeria_monocytogenes_1639_v105	0.9206
Listeria_monocytogenes_serotype_1_2a_str_10-0813_1196166	Listeria_monocytogenes_1639_v105	0.9285
Listeria_monocytogenes_serotype_1_2a_str_10-0812_1196165	Listeria_monocytogenes_1639_v105	0.9285
Listeria_monocytogenes_serotype_1_2a_str_08-7669_1196186	Listeria_monocytogenes_1639_v105	0.9296
Listeria_monocytogenes_serotype_1_2a_str_08-7374_1196185	Listeria_monocytogenes_1639_v105	0.9203
Listeria_monocytogenes_serotype_1_2a_str_08-6997_1196184	Listeria_monocytogenes_1639_v105	0.9162
Listeria_monocytogenes_serotype_1_2a_str_08-6569_1196183	Listeria_monocytogenes_1639_v105	0.9162
Listeria_monocytogenes_serotype_1_2a_str_08-6056_1196182	Listeria_monocytogenes_1639_v105	0.9162
Listeria_monocytogenes_serotype_1_2a_str_04-5457_1196181	Listeria_monocytogenes_1639_v105	0.9203
Listeria_monocytogenes_serotype_1_2a_str_02-5993_1196180	Listeria_monocytogenes_1639_v105	0.9203
Listeria_monocytogenes_R479a_1437838	Listeria_monocytogenes_1639_v105	0.9196
Listeria_monocytogenes_N53-1_1234142	Listeria_monocytogenes_1639_v105	0.8912
Listeria_monocytogenes_M7_1030009	Listeria_monocytogenes_1639_v105	0.9058
Listeria_monocytogenes_LO28_393132	Listeria_monocytogenes_1639_v105	0.9244
Listeria_monocytogenes_L99_563174	Listeria_monocytogenes_1639_v105	0.9061
Listeria_monocytogenes_L312_882094	Listeria_monocytogenes_1639_v105	0.9719
Listeria_monocytogenes_J1816_930781	Listeria_monocytogenes_1639_v105	0.9504
Listeria_monocytogenes_J1-220_930782	Listeria_monocytogenes_1639_v105	0.9779
Listeria_monocytogenes_J0161_393130	Listeria_monocytogenes_1639_v105	0.9274
Listeria_monocytogenes_HCC23_552536	Listeria_monocytogenes_1639_v105	0.9054
Listeria_monocytogenes_FSL_R2-561_393126	Listeria_monocytogenes_1639_v105	0.9247
Listeria_monocytogenes_FSL_J1-208_393119	Listeria_monocytogenes_1639_v105	0.9206
Listeria_monocytogenes_FSL_J1-175_393118	Listeria_monocytogenes_1639_v105	0.955
Listeria_monocytogenes_Finland_1998_393127	Listeria_monocytogenes_1639_v105	0.9471
Listeria_monocytogenes_EGD-e_169963_v1	Listeria_monocytogenes_1639_v105	0.9342
Listeria_monocytogenes_EGD-e_169963	Listeria_monocytogenes_1639_v105	0.9346
Listeria_monocytogenes_EGD_1334565	Listeria_monocytogenes_1639_v105	0.9359
Listeria_monocytogenes_CFSAN002349_1299895	Listeria_monocytogenes_1639_v105	0.9716
Listeria_monocytogenes_ATCC_19117_882095_v1	Listeria_monocytogenes_1639_v105	0.9989
Listeria_monocytogenes_ATCC_19117_882095	Listeria_monocytogenes_1639_v105	0.9985
Listeria_monocytogenes_6179_1288295	Listeria_monocytogenes_1639_v105	0.9113
Listeria_monocytogenes_1639_v9	Listeria_monocytogenes_1639_v105	0.926
Listeria_monocytogenes_1639_v99	Listeria_monocytogenes_1639_v105	0.9504
Listeria_monocytogenes_1639_v98	Listeria_monocytogenes_1639_v105	0.9254
Listeria_monocytogenes_1639_v97	Listeria_monocytogenes_1639_v105	0.9655
Listeria_monocytogenes_1639_v96	Listeria_monocytogenes_1639_v105	0.9497
Listeria_monocytogenes_1639_v95	Listeria_monocytogenes_1639_v105	0.9497
Listeria_monocytogenes_1639_v94	Listeria_monocytogenes_1639_v105	0.95
Listeria_monocytogenes_1639_v93	Listeria_monocytogenes_1639_v105	0.9239
Listeria_monocytogenes_1639_v92	Listeria_monocytogenes_1639_v105	0.95
Listeria_monocytogenes_1639_v91	Listeria_monocytogenes_1639_v105	0.9567
Listeria_monocytogenes_1639_v90	Listeria_monocytogenes_1639_v105	0.9254
Listeria_monocytogenes_1639_v8	Listeria_monocytogenes_1639_v105	0.9116
Listeria_monocytogenes_1639_v89	Listeria_monocytogenes_1639_v105	0.9669
Listeria_monocytogenes_1639_v88	Listeria_monocytogenes_1639_v105	0.926
Listeria_monocytogenes_1639_v87	Listeria_monocytogenes_1639_v105	0.9247
Listeria_monocytogenes_1639_v86	Listeria_monocytogenes_1639_v105	0.9244
Listeria_monocytogenes_1639_v85	Listeria_monocytogenes_1639_v105	0.9695
Listeria_monocytogenes_1639_v84	Listeria_monocytogenes_1639_v105	0.9314
Listeria_monocytogenes_1639_v83	Listeria_monocytogenes_1639_v105	0.9871
Listeria_monocytogenes_1639_v82	Listeria_monocytogenes_1639_v105	0.9868
Listeria_monocytogenes_1639_v81	Listeria_monocytogenes_1639_v105	0.9278
Listeria_monocytogenes_1639_v80	Listeria_monocytogenes_1639_v105	0.9447
Listeria_monocytogenes_1639_v7	Listeria_monocytogenes_1639_v105	0.9116
Listeria_monocytogenes_1639_v79	Listeria_monocytogenes_1639_v105	0.9261
Listeria_monocytogenes_1639_v78	Listeria_monocytogenes_1639_v105	0.9271
Listeria_monocytogenes_1639_v77	Listeria_monocytogenes_1639_v105	0.931
Listeria_monocytogenes_1639_v76	Listeria_monocytogenes_1639_v105	0.9224
Listeria_monocytogenes_1639_v75	Listeria_monocytogenes_1639_v105	0.9061
Listeria_monocytogenes_1639_v74	Listeria_monocytogenes_1639_v105	0.9232
Listeria_monocytogenes_1639_v73	Listeria_monocytogenes_1639_v105	0.9199
Listeria_monocytogenes_1639_v72	Listeria_monocytogenes_1639_v105	0.9856
Listeria_monocytogenes_1639_v71	Listeria_monocytogenes_1639_v105	0.9115
Listeria_monocytogenes_1639_v70	Listeria_monocytogenes_1639_v105	0.9219
Listeria_monocytogenes_1639_v6	Listeria_monocytogenes_1639_v105	0.9113
Listeria_monocytogenes_1639_v69	Listeria_monocytogenes_1639_v105	0.9709
Listeria_monocytogenes_1639_v68	Listeria_monocytogenes_1639_v105	0.941
Listeria_monocytogenes_1639_v67	Listeria_monocytogenes_1639_v105	0.9444
Listeria_monocytogenes_1639_v66	Listeria_monocytogenes_1639_v105	0.9323
Listeria_monocytogenes_1639_v65	Listeria_monocytogenes_1639_v105	0.923
Listeria_monocytogenes_1639_v64	Listeria_monocytogenes_1639_v105	0.9264
Listeria_monocytogenes_1639_v63	Listeria_monocytogenes_1639_v105	0.9399
Listeria_monocytogenes_1639_v62	Listeria_monocytogenes_1639_v105	0.9233
Listeria_monocytogenes_1639_v61	Listeria_monocytogenes_1639_v105	0.923
Listeria_monocytogenes_1639_v60	Listeria_monocytogenes_1639_v105	0.9293
Listeria_monocytogenes_1639_v5	Listeria_monocytogenes_1639_v105	0.9061
Listeria_monocytogenes_1639_v59	Listeria_monocytogenes_1639_v105	0.9875
Listeria_monocytogenes_1639_v58	Listeria_monocytogenes_1639_v105	0.9765
Listeria_monocytogenes_1639_v57	Listeria_monocytogenes_1639_v105	0.9469
Listeria_monocytogenes_1639_v56	Listeria_monocytogenes_1639_v105	0.9875
Listeria_monocytogenes_1639_v55	Listeria_monocytogenes_1639_v105	0.9426
Listeria_monocytogenes_1639_v54	Listeria_monocytogenes_1639_v105	0.9253

Listeria_monocytogenes_1639_v53	Listeria_monocytogenes_1639_v105	0.9297
Listeria_monocytogenes_1639_v52	Listeria_monocytogenes_1639_v105	0.974
Listeria_monocytogenes_1639_v51	Listeria_monocytogenes_1639_v105	0.9812
Listeria_monocytogenes_1639_v50	Listeria_monocytogenes_1639_v105	0.9195
Listeria_monocytogenes_1639_v4	Listeria_monocytogenes_1639_v105	0.9193
Listeria_monocytogenes_1639_v49	Listeria_monocytogenes_1639_v105	0.9683
Listeria_monocytogenes_1639_v48	Listeria_monocytogenes_1639_v105	0.9202
Listeria_monocytogenes_1639_v47	Listeria_monocytogenes_1639_v105	0.9199
Listeria_monocytogenes_1639_v46	Listeria_monocytogenes_1639_v105	0.9403
Listeria_monocytogenes_1639_v45	Listeria_monocytogenes_1639_v105	0.9114
Listeria_monocytogenes_1639_v44	Listeria_monocytogenes_1639_v105	0.9203
Listeria_monocytogenes_1639_v43	Listeria_monocytogenes_1639_v105	0.9203
Listeria_monocytogenes_1639_v42	Listeria_monocytogenes_1639_v105	0.9147
Listeria_monocytogenes_1639_v41	Listeria_monocytogenes_1639_v105	0.9021
Listeria_monocytogenes_1639_v40	Listeria_monocytogenes_1639_v105	0.9247
Listeria_monocytogenes_1639_v3	Listeria_monocytogenes_1639_v105	0.9197
Listeria_monocytogenes_1639_v39	Listeria_monocytogenes_1639_v105	0.9436
Listeria_monocytogenes_1639_v38	Listeria_monocytogenes_1639_v105	0.9302
Listeria_monocytogenes_1639_v37	Listeria_monocytogenes_1639_v105	0.916
Listeria_monocytogenes_1639_v36	Listeria_monocytogenes_1639_v105	0.9176
Listeria_monocytogenes_1639_v35	Listeria_monocytogenes_1639_v105	0.9254
Listeria_monocytogenes_1639_v34	Listeria_monocytogenes_1639_v105	0.9329
Listeria_monocytogenes_1639_v33	Listeria_monocytogenes_1639_v105	0.9384
Listeria_monocytogenes_1639_v32	Listeria_monocytogenes_1639_v105	0.915
Listeria_monocytogenes_1639_v31	Listeria_monocytogenes_1639_v105	0.9333
Listeria_monocytogenes_1639_v30	Listeria_monocytogenes_1639_v105	0.9432
Listeria_monocytogenes_1639_v2	Listeria_monocytogenes_1639_v105	0.9025
Listeria_monocytogenes_1639_v29	Listeria_monocytogenes_1639_v105	0.9128
Listeria_monocytogenes_1639_v28	Listeria_monocytogenes_1639_v105	0.9168
Listeria_monocytogenes_1639_v27	Listeria_monocytogenes_1639_v105	0.9081
Listeria_monocytogenes_1639_v26	Listeria_monocytogenes_1639_v105	0.9078
Listeria_monocytogenes_1639_v25	Listeria_monocytogenes_1639_v105	0.9078
Listeria_monocytogenes_1639_v24	Listeria_monocytogenes_1639_v105	0.9779
Listeria_monocytogenes_1639_v23	Listeria_monocytogenes_1639_v105	0.9775
Listeria_monocytogenes_1639_v22	Listeria_monocytogenes_1639_v105	0.9406
Listeria_monocytogenes_1639_v21	Listeria_monocytogenes_1639_v105	0.9775
Listeria_monocytogenes_1639_v20	Listeria_monocytogenes_1639_v105	0.931
Listeria_monocytogenes_1639_v1	Listeria_monocytogenes_1639_v105	0.9054
Listeria_monocytogenes_1639_v19	Listeria_monocytogenes_1639_v105	0.9306
Listeria_monocytogenes_1639_v198	Listeria_monocytogenes_1639_v105	0.8939
Listeria_monocytogenes_1639_v197	Listeria_monocytogenes_1639_v105	0.9219
Listeria_monocytogenes_1639_v196	Listeria_monocytogenes_1639_v105	0.9186
Listeria_monocytogenes_1639_v195	Listeria_monocytogenes_1639_v105	0.9424
Listeria_monocytogenes_1639_v194	Listeria_monocytogenes_1639_v105	0.9414
Listeria_monocytogenes_1639_v193	Listeria_monocytogenes_1639_v105	0.9414
Listeria_monocytogenes_1639_v192	Listeria_monocytogenes_1639_v105	0.9143
Listeria_monocytogenes_1639_v191	Listeria_monocytogenes_1639_v105	0.9485
Listeria_monocytogenes_1639_v190	Listeria_monocytogenes_1639_v105	0.9478
Listeria_monocytogenes_1639_v18	Listeria_monocytogenes_1639_v105	0.9409
Listeria_monocytogenes_1639_v189	Listeria_monocytogenes_1639_v105	0.9467
Listeria_monocytogenes_1639_v188	Listeria_monocytogenes_1639_v105	0.9493
Listeria_monocytogenes_1639_v187	Listeria_monocytogenes_1639_v105	0.9478
Listeria_monocytogenes_1639_v186	Listeria_monocytogenes_1639_v105	0.9478
Listeria_monocytogenes_1639_v185	Listeria_monocytogenes_1639_v105	0.9482
Listeria_monocytogenes_1639_v184	Listeria_monocytogenes_1639_v105	0.9474
Listeria_monocytogenes_1639_v183	Listeria_monocytogenes_1639_v105	0.9428
Listeria_monocytogenes_1639_v182	Listeria_monocytogenes_1639_v105	0.9446
Listeria_monocytogenes_1639_v181	Listeria_monocytogenes_1639_v105	0.935
Listeria_monocytogenes_1639_v180	Listeria_monocytogenes_1639_v105	0.935
Listeria_monocytogenes_1639_v17	Listeria_monocytogenes_1639_v105	0.9412
Listeria_monocytogenes_1639_v179	Listeria_monocytogenes_1639_v105	0.9323
Listeria_monocytogenes_1639_v178	Listeria_monocytogenes_1639_v105	0.949
Listeria_monocytogenes_1639_v177	Listeria_monocytogenes_1639_v105	0.9697
Listeria_monocytogenes_1639_v176	Listeria_monocytogenes_1639_v105	0.9422
Listeria_monocytogenes_1639_v175	Listeria_monocytogenes_1639_v105	0.9372
Listeria_monocytogenes_1639_v174	Listeria_monocytogenes_1639_v105	0.9479
Listeria_monocytogenes_1639_v173	Listeria_monocytogenes_1639_v105	0.9645
Listeria_monocytogenes_1639_v172	Listeria_monocytogenes_1639_v105	0.942
Listeria_monocytogenes_1639_v171	Listeria_monocytogenes_1639_v105	0.9338
Listeria_monocytogenes_1639_v170	Listeria_monocytogenes_1639_v105	0.9417
Listeria_monocytogenes_1639_v16	Listeria_monocytogenes_1639_v105	0.9071
Listeria_monocytogenes_1639_v169	Listeria_monocytogenes_1639_v105	0.9421
Listeria_monocytogenes_1639_v168	Listeria_monocytogenes_1639_v105	0.9426
Listeria_monocytogenes_1639_v167	Listeria_monocytogenes_1639_v105	0.9348
Listeria_monocytogenes_1639_v166	Listeria_monocytogenes_1639_v105	0.9429
Listeria_monocytogenes_1639_v165	Listeria_monocytogenes_1639_v105	0.9418
Listeria_monocytogenes_1639_v164	Listeria_monocytogenes_1639_v105	0.9758
Listeria_monocytogenes_1639_v163	Listeria_monocytogenes_1639_v105	0.9589
Listeria_monocytogenes_1639_v162	Listeria_monocytogenes_1639_v105	0.9758
Listeria_monocytogenes_1639_v161	Listeria_monocytogenes_1639_v105	0.9762
Listeria_monocytogenes_1639_v160	Listeria_monocytogenes_1639_v105	0.9762
Listeria_monocytogenes_1639_v15	Listeria_monocytogenes_1639_v105	0.9243
Listeria_monocytogenes_1639_v159	Listeria_monocytogenes_1639_v105	0.9697
Listeria_monocytogenes_1639_v158	Listeria_monocytogenes_1639_v105	0.9762
Listeria_monocytogenes_1639_v157	Listeria_monocytogenes_1639_v105	0.9762
Listeria_monocytogenes_1639_v156	Listeria_monocytogenes_1639_v105	0.9249
Listeria_monocytogenes_1639_v155	Listeria_monocytogenes_1639_v105	0.9373
Listeria_monocytogenes_1639_v154	Listeria_monocytogenes_1639_v105	0.9385
Listeria_monocytogenes_1639_v153	Listeria_monocytogenes_1639_v105	0.938
Listeria_monocytogenes_1639_v152	Listeria_monocytogenes_1639_v105	0.9697
Listeria_monocytogenes_1639_v151	Listeria_monocytogenes_1639_v105	0.9697

Listeria_monocytogenes_1639_v150	Listeria_monocytogenes_1639_v105	0.9377
Listeria_monocytogenes_1639_v14	Listeria_monocytogenes_1639_v105	0.9319
Listeria_monocytogenes_1639_v149	Listeria_monocytogenes_1639_v105	0.9363
Listeria_monocytogenes_1639_v148	Listeria_monocytogenes_1639_v105	0.9686
Listeria_monocytogenes_1639_v147	Listeria_monocytogenes_1639_v105	0.9551
Listeria_monocytogenes_1639_v146	Listeria_monocytogenes_1639_v105	0.9465
Listeria_monocytogenes_1639_v145	Listeria_monocytogenes_1639_v105	0.9435
Listeria_monocytogenes_1639_v144	Listeria_monocytogenes_1639_v105	0.9716
Listeria_monocytogenes_1639_v143	Listeria_monocytogenes_1639_v105	0.9473
Listeria_monocytogenes_1639_v142	Listeria_monocytogenes_1639_v105	0.9719
Listeria_monocytogenes_1639_v141	Listeria_monocytogenes_1639_v105	0.9638
Listeria_monocytogenes_1639_v140	Listeria_monocytogenes_1639_v105	0.9723
Listeria_monocytogenes_1639_v13	Listeria_monocytogenes_1639_v105	0.9243
Listeria_monocytogenes_1639_v139	Listeria_monocytogenes_1639_v105	0.9724
Listeria_monocytogenes_1639_v138	Listeria_monocytogenes_1639_v105	0.9743
Listeria_monocytogenes_1639_v137	Listeria_monocytogenes_1639_v105	0.9739
Listeria_monocytogenes_1639_v136	Listeria_monocytogenes_1639_v105	0.938
Listeria_monocytogenes_1639_v135	Listeria_monocytogenes_1639_v105	0.92
Listeria_monocytogenes_1639_v134	Listeria_monocytogenes_1639_v105	0.9801
Listeria_monocytogenes_1639_v133	Listeria_monocytogenes_1639_v105	0.9351
Listeria_monocytogenes_1639_v132	Listeria_monocytogenes_1639_v105	0.9205
Listeria_monocytogenes_1639_v131	Listeria_monocytogenes_1639_v105	0.9575
Listeria_monocytogenes_1639_v130	Listeria_monocytogenes_1639_v105	0.9258
Listeria_monocytogenes_1639_v12	Listeria_monocytogenes_1639_v105	0.9068
Listeria_monocytogenes_1639_v129	Listeria_monocytogenes_1639_v105	0.9639
Listeria_monocytogenes_1639_v128	Listeria_monocytogenes_1639_v105	0.9617
Listeria_monocytogenes_1639_v127	Listeria_monocytogenes_1639_v105	0.9342
Listeria_monocytogenes_1639_v126	Listeria_monocytogenes_1639_v105	0.9598
Listeria_monocytogenes_1639_v125	Listeria_monocytogenes_1639_v105	0.9343
Listeria_monocytogenes_1639_v124	Listeria_monocytogenes_1639_v105	0.9432
Listeria_monocytogenes_1639_v123	Listeria_monocytogenes_1639_v105	0.9716
Listeria_monocytogenes_1639_v122	Listeria_monocytogenes_1639_v105	0.9716
Listeria_monocytogenes_1639_v121	Listeria_monocytogenes_1639_v105	0.9716
Listeria_monocytogenes_1639_v120	Listeria_monocytogenes_1639_v105	0.972
Listeria_monocytogenes_1639_v11	Listeria_monocytogenes_1639_v105	0.9081
Listeria_monocytogenes_1639_v119	Listeria_monocytogenes_1639_v105	0.9716
Listeria_monocytogenes_1639_v118	Listeria_monocytogenes_1639_v105	0.9716
Listeria_monocytogenes_1639_v117	Listeria_monocytogenes_1639_v105	0.9365
Listeria_monocytogenes_1639_v116	Listeria_monocytogenes_1639_v105	0.9813
Listeria_monocytogenes_1639_v115	Listeria_monocytogenes_1639_v105	0.9809
Listeria_monocytogenes_1639_v114	Listeria_monocytogenes_1639_v105	0.9295
Listeria_monocytogenes_1639_v113	Listeria_monocytogenes_1639_v105	0.9546
Listeria_monocytogenes_1639_v112	Listeria_monocytogenes_1639_v105	0.9654
Listeria_monocytogenes_1639_v111	Listeria_monocytogenes_1639_v105	0.9461
Listeria_monocytogenes_1639_v110	Listeria_monocytogenes_1639_v105	0.9292
Listeria_monocytogenes_1639_v10	Listeria_monocytogenes_1639_v105	0.923
Listeria_monocytogenes_1639_v109	Listeria_monocytogenes_1639_v105	0.9288
Listeria_monocytogenes_1639_v108	Listeria_monocytogenes_1639_v105	0.9587
Listeria_monocytogenes_1639_v107	Listeria_monocytogenes_1639_v105	0.9985
Listeria_monocytogenes_1639_v106	Listeria_monocytogenes_1639_v105	0.9996
Listeria_monocytogenes_1639_v105	Listeria_monocytogenes_1639_v105	0.0684
Listeria_welshimeri_1643	Listeria_monocytogenes_1639_v106	0.89
Listeria_welshimeri_serovar_6b_str_SLCC5334_386043	Listeria_monocytogenes_1639_v106	0.8897
Listeria_seeligeri_serovar_1_2b_str_SLCC3954_683837	Listeria_monocytogenes_1639_v106	0.8869
Listeria_seeligeri_FSL_S4-171_702452	Listeria_monocytogenes_1639_v106	0.8273
Listeria_seeligeri_FSL_N1-067_702453	Listeria_monocytogenes_1639_v106	0.8293
Listeria_seeligeri_1640_v1	Listeria_monocytogenes_1639_v106	0.8937
Listeria_seeligeri_1640	Listeria_monocytogenes_1639_v106	0.8804
Listeria_monocytogenes_WSLC1042_1457188	Listeria_monocytogenes_1639_v106	0.9798
Listeria_monocytogenes_WSLC1001_1457187	Listeria_monocytogenes_1639_v106	0.9309
Listeria_monocytogenes_SLCC7179_879090	Listeria_monocytogenes_1639_v106	0.9376
Listeria_monocytogenes_SLCC5850_882096	Listeria_monocytogenes_1639_v106	0.9352
Listeria_monocytogenes_SLCC2755_932919	Listeria_monocytogenes_1639_v106	0.9444
Listeria_monocytogenes_SLCC2540_879089	Listeria_monocytogenes_1639_v106	0.9476
Listeria_monocytogenes_SLCC2479_882020	Listeria_monocytogenes_1639_v106	0.9257
Listeria_monocytogenes_SLCC2378_879088	Listeria_monocytogenes_1639_v106	0.9813
Listeria_monocytogenes_SLCC2376_882097	Listeria_monocytogenes_1639_v106	0.9409
Listeria_monocytogenes_SLCC2372_932920	Listeria_monocytogenes_1639_v106	0.9185
Listeria_monocytogenes_serotype_4b_str_LL195_1230340	Listeria_monocytogenes_1639_v106	0.9762
Listeria_monocytogenes_serotype_4b_str_LIS0087_1134669	Listeria_monocytogenes_1639_v106	0.9119
Listeria_monocytogenes_serotype_4b_str_F2365_265669	Listeria_monocytogenes_1639_v106	0.9762
Listeria_monocytogenes_serotype_4b_str_CLIP_80459_568819	Listeria_monocytogenes_1639_v106	0.9715
Listeria_monocytogenes_serotype_4b_str_81-0861_1196161	Listeria_monocytogenes_1639_v106	0.9698
Listeria_monocytogenes_serotype_4b_str_81-0592_1196160	Listeria_monocytogenes_1639_v106	0.9709
Listeria_monocytogenes_serotype_4b_str_81-0558_1196159	Listeria_monocytogenes_1639_v106	0.9702
Listeria_monocytogenes_serotype_4b_str_10-0809_1196162	Listeria_monocytogenes_1639_v106	0.9709
Listeria_monocytogenes_serotype_4b_str_02-6680_1196175	Listeria_monocytogenes_1639_v106	0.9781
Listeria_monocytogenes_serotype_4b_str_02-6679_1196174	Listeria_monocytogenes_1639_v106	0.9781
Listeria_monocytogenes_serotype_4b_str_02-1792_1196173	Listeria_monocytogenes_1639_v106	0.9702
Listeria_monocytogenes_serotype_4b_str_02-1289_1196172	Listeria_monocytogenes_1639_v106	0.9702
Listeria_monocytogenes_serotype_4b_str_02-1103_1196171	Listeria_monocytogenes_1639_v106	0.9702
Listeria_monocytogenes_serotype_3c_str_10-5027_1196195	Listeria_monocytogenes_1639_v106	0.925
Listeria_monocytogenes_serotype_1_2c_str_10-5026_1196194	Listeria_monocytogenes_1639_v106	0.9257
Listeria_monocytogenes_serotype_1_2c_str_10-5025_1196193	Listeria_monocytogenes_1639_v106	0.9257
Listeria_monocytogenes_serotype_1_2b_str_10-0811_1196164	Listeria_monocytogenes_1639_v106	0.9384
Listeria_monocytogenes_serotype_1_2b_str_10-0810_1196163	Listeria_monocytogenes_1639_v106	0.9384
Listeria_monocytogenes_serotype_1_2a_str_99-6370_1196179	Listeria_monocytogenes_1639_v106	0.9148
Listeria_monocytogenes_serotype_1_2a_str_98-2035_1196178	Listeria_monocytogenes_1639_v106	0.9148
Listeria_monocytogenes_serotype_1_2a_str_95-0093_1196177	Listeria_monocytogenes_1639_v106	0.9203
Listeria_monocytogenes_serotype_1_2a_str_88-0478_1196176	Listeria_monocytogenes_1639_v106	0.9191
Listeria_monocytogenes_serotype_1_2a_str_10-5024_1196192	Listeria_monocytogenes_1639_v106	0.9203

Listeria_monocytogenes_serotype_1_2a_str_10-4758_1196168	Listeria_monocytogenes_1639_v106	0.9383
Listeria_monocytogenes_serotype_1_2a_str_10-4754_1196167	Listeria_monocytogenes_1639_v106	0.9387
Listeria_monocytogenes_serotype_1_2a_str_10-1321_1196191	Listeria_monocytogenes_1639_v106	0.9203
Listeria_monocytogenes_serotype_1_2a_str_10-1047_1196190	Listeria_monocytogenes_1639_v106	0.9209
Listeria_monocytogenes_serotype_1_2a_str_10-1046_1196189	Listeria_monocytogenes_1639_v106	0.9209
Listeria_monocytogenes_serotype_1_2a_str_10-0934_1196170	Listeria_monocytogenes_1639_v106	0.9452
Listeria_monocytogenes_serotype_1_2a_str_10-0933_1196169	Listeria_monocytogenes_1639_v106	0.9452
Listeria_monocytogenes_serotype_1_2a_str_10-0815_1196188	Listeria_monocytogenes_1639_v106	0.9162
Listeria_monocytogenes_serotype_1_2a_str_10-0814_1196187	Listeria_monocytogenes_1639_v106	0.9206
Listeria_monocytogenes_serotype_1_2a_str_10-0813_1196166	Listeria_monocytogenes_1639_v106	0.9285
Listeria_monocytogenes_serotype_1_2a_str_10-0812_1196165	Listeria_monocytogenes_1639_v106	0.9285
Listeria_monocytogenes_serotype_1_2a_str_08-7669_1196186	Listeria_monocytogenes_1639_v106	0.9296
Listeria_monocytogenes_serotype_1_2a_str_08-7374_1196185	Listeria_monocytogenes_1639_v106	0.9203
Listeria_monocytogenes_serotype_1_2a_str_08-6997_1196184	Listeria_monocytogenes_1639_v106	0.9162
Listeria_monocytogenes_serotype_1_2a_str_08-6569_1196183	Listeria_monocytogenes_1639_v106	0.9162
Listeria_monocytogenes_serotype_1_2a_str_08-6056_1196182	Listeria_monocytogenes_1639_v106	0.9162
Listeria_monocytogenes_serotype_1_2a_str_04-5457_1196181	Listeria_monocytogenes_1639_v106	0.9203
Listeria_monocytogenes_serotype_1_2a_str_02-5993_1196180	Listeria_monocytogenes_1639_v106	0.9203
Listeria_monocytogenes_R479a_1437838	Listeria_monocytogenes_1639_v106	0.9196
Listeria_monocytogenes_N53-1_1234142	Listeria_monocytogenes_1639_v106	0.8912
Listeria_monocytogenes_M7_1030009	Listeria_monocytogenes_1639_v106	0.9058
Listeria_monocytogenes_LO28_393132	Listeria_monocytogenes_1639_v106	0.9244
Listeria_monocytogenes_L99_563174	Listeria_monocytogenes_1639_v106	0.9061
Listeria_monocytogenes_L312_882094	Listeria_monocytogenes_1639_v106	0.9719
Listeria_monocytogenes_J1816_930781	Listeria_monocytogenes_1639_v106	0.95
Listeria_monocytogenes_J1-220_930782	Listeria_monocytogenes_1639_v106	0.9779
Listeria_monocytogenes_J0161_393130	Listeria_monocytogenes_1639_v106	0.9274
Listeria_monocytogenes_HCC23_552536	Listeria_monocytogenes_1639_v106	0.9054
Listeria_monocytogenes_FSL_R2-561_393126	Listeria_monocytogenes_1639_v106	0.9247
Listeria_monocytogenes_FSL_J1-208_393119	Listeria_monocytogenes_1639_v106	0.9206
Listeria_monocytogenes_FSL_J1-175_393118	Listeria_monocytogenes_1639_v106	0.955
Listeria_monocytogenes_Finland_1998_393127	Listeria_monocytogenes_1639_v106	0.9471
Listeria_monocytogenes_EGD-e_169963_v1	Listeria_monocytogenes_1639_v106	0.9342
Listeria_monocytogenes_EGD-e_169963	Listeria_monocytogenes_1639_v106	0.9346
Listeria_monocytogenes_EGD_1334565	Listeria_monocytogenes_1639_v106	0.9359
Listeria_monocytogenes_CFSAN002349_1299895	Listeria_monocytogenes_1639_v106	0.9716
Listeria_monocytogenes_ATCC_19117_882095_v1	Listeria_monocytogenes_1639_v106	0.9989
Listeria_monocytogenes_ATCC_19117_882095	Listeria_monocytogenes_1639_v106	0.9985
Listeria_monocytogenes_6179_1288295	Listeria_monocytogenes_1639_v106	0.9113
Listeria_monocytogenes_1639_v9	Listeria_monocytogenes_1639_v106	0.926
Listeria_monocytogenes_1639_v99	Listeria_monocytogenes_1639_v106	0.9504
Listeria_monocytogenes_1639_v98	Listeria_monocytogenes_1639_v106	0.9254
Listeria_monocytogenes_1639_v97	Listeria_monocytogenes_1639_v106	0.9655
Listeria_monocytogenes_1639_v96	Listeria_monocytogenes_1639_v106	0.9497
Listeria_monocytogenes_1639_v95	Listeria_monocytogenes_1639_v106	0.9497
Listeria_monocytogenes_1639_v94	Listeria_monocytogenes_1639_v106	0.95
Listeria_monocytogenes_1639_v93	Listeria_monocytogenes_1639_v106	0.9239
Listeria_monocytogenes_1639_v92	Listeria_monocytogenes_1639_v106	0.95
Listeria_monocytogenes_1639_v91	Listeria_monocytogenes_1639_v106	0.9567
Listeria_monocytogenes_1639_v90	Listeria_monocytogenes_1639_v106	0.9254
Listeria_monocytogenes_1639_v8	Listeria_monocytogenes_1639_v106	0.9116
Listeria_monocytogenes_1639_v89	Listeria_monocytogenes_1639_v106	0.9669
Listeria_monocytogenes_1639_v88	Listeria_monocytogenes_1639_v106	0.926
Listeria_monocytogenes_1639_v87	Listeria_monocytogenes_1639_v106	0.9247
Listeria_monocytogenes_1639_v86	Listeria_monocytogenes_1639_v106	0.9244
Listeria_monocytogenes_1639_v85	Listeria_monocytogenes_1639_v106	0.9691
Listeria_monocytogenes_1639_v84	Listeria_monocytogenes_1639_v106	0.9314
Listeria_monocytogenes_1639_v83	Listeria_monocytogenes_1639_v106	0.9871
Listeria_monocytogenes_1639_v82	Listeria_monocytogenes_1639_v106	0.9864
Listeria_monocytogenes_1639_v81	Listeria_monocytogenes_1639_v106	0.9278
Listeria_monocytogenes_1639_v80	Listeria_monocytogenes_1639_v106	0.9447
Listeria_monocytogenes_1639_v7	Listeria_monocytogenes_1639_v106	0.9116
Listeria_monocytogenes_1639_v79	Listeria_monocytogenes_1639_v106	0.9261
Listeria_monocytogenes_1639_v78	Listeria_monocytogenes_1639_v106	0.9271
Listeria_monocytogenes_1639_v77	Listeria_monocytogenes_1639_v106	0.931
Listeria_monocytogenes_1639_v76	Listeria_monocytogenes_1639_v106	0.9224
Listeria_monocytogenes_1639_v75	Listeria_monocytogenes_1639_v106	0.9061
Listeria_monocytogenes_1639_v74	Listeria_monocytogenes_1639_v106	0.9232
Listeria_monocytogenes_1639_v73	Listeria_monocytogenes_1639_v106	0.9199
Listeria_monocytogenes_1639_v72	Listeria_monocytogenes_1639_v106	0.9856
Listeria_monocytogenes_1639_v71	Listeria_monocytogenes_1639_v106	0.9115
Listeria_monocytogenes_1639_v70	Listeria_monocytogenes_1639_v106	0.9219
Listeria_monocytogenes_1639_v6	Listeria_monocytogenes_1639_v106	0.9113
Listeria_monocytogenes_1639_v69	Listeria_monocytogenes_1639_v106	0.9709
Listeria_monocytogenes_1639_v68	Listeria_monocytogenes_1639_v106	0.941
Listeria_monocytogenes_1639_v67	Listeria_monocytogenes_1639_v106	0.9444
Listeria_monocytogenes_1639_v66	Listeria_monocytogenes_1639_v106	0.9323
Listeria_monocytogenes_1639_v65	Listeria_monocytogenes_1639_v106	0.923
Listeria_monocytogenes_1639_v64	Listeria_monocytogenes_1639_v106	0.9264
Listeria_monocytogenes_1639_v63	Listeria_monocytogenes_1639_v106	0.9399
Listeria_monocytogenes_1639_v62	Listeria_monocytogenes_1639_v106	0.9233
Listeria_monocytogenes_1639_v61	Listeria_monocytogenes_1639_v106	0.923
Listeria_monocytogenes_1639_v60	Listeria_monocytogenes_1639_v106	0.9293
Listeria_monocytogenes_1639_v5	Listeria_monocytogenes_1639_v106	0.9061
Listeria_monocytogenes_1639_v59	Listeria_monocytogenes_1639_v106	0.9875
Listeria_monocytogenes_1639_v58	Listeria_monocytogenes_1639_v106	0.9765
Listeria_monocytogenes_1639_v57	Listeria_monocytogenes_1639_v106	0.9469
Listeria_monocytogenes_1639_v56	Listeria_monocytogenes_1639_v106	0.9875
Listeria_monocytogenes_1639_v55	Listeria_monocytogenes_1639_v106	0.9426
Listeria_monocytogenes_1639_v54	Listeria_monocytogenes_1639_v106	0.9253
Listeria_monocytogenes_1639_v53	Listeria_monocytogenes_1639_v106	0.9297

Listeria_monocytogenes_1639_v52	Listeria_monocytogenes_1639_v106	0.974
Listeria_monocytogenes_1639_v51	Listeria_monocytogenes_1639_v106	0.9816
Listeria_monocytogenes_1639_v50	Listeria_monocytogenes_1639_v106	0.9195
Listeria_monocytogenes_1639_v4	Listeria_monocytogenes_1639_v106	0.9193
Listeria_monocytogenes_1639_v49	Listeria_monocytogenes_1639_v106	0.9683
Listeria_monocytogenes_1639_v48	Listeria_monocytogenes_1639_v106	0.9202
Listeria_monocytogenes_1639_v47	Listeria_monocytogenes_1639_v106	0.9199
Listeria_monocytogenes_1639_v46	Listeria_monocytogenes_1639_v106	0.9403
Listeria_monocytogenes_1639_v45	Listeria_monocytogenes_1639_v106	0.9114
Listeria_monocytogenes_1639_v44	Listeria_monocytogenes_1639_v106	0.9203
Listeria_monocytogenes_1639_v43	Listeria_monocytogenes_1639_v106	0.9203
Listeria_monocytogenes_1639_v42	Listeria_monocytogenes_1639_v106	0.9144
Listeria_monocytogenes_1639_v41	Listeria_monocytogenes_1639_v106	0.9018
Listeria_monocytogenes_1639_v40	Listeria_monocytogenes_1639_v106	0.9247
Listeria_monocytogenes_1639_v3	Listeria_monocytogenes_1639_v106	0.9197
Listeria_monocytogenes_1639_v39	Listeria_monocytogenes_1639_v106	0.9436
Listeria_monocytogenes_1639_v38	Listeria_monocytogenes_1639_v106	0.9302
Listeria_monocytogenes_1639_v37	Listeria_monocytogenes_1639_v106	0.916
Listeria_monocytogenes_1639_v36	Listeria_monocytogenes_1639_v106	0.9176
Listeria_monocytogenes_1639_v35	Listeria_monocytogenes_1639_v106	0.9254
Listeria_monocytogenes_1639_v34	Listeria_monocytogenes_1639_v106	0.9329
Listeria_monocytogenes_1639_v33	Listeria_monocytogenes_1639_v106	0.9384
Listeria_monocytogenes_1639_v32	Listeria_monocytogenes_1639_v106	0.915
Listeria_monocytogenes_1639_v31	Listeria_monocytogenes_1639_v106	0.9333
Listeria_monocytogenes_1639_v30	Listeria_monocytogenes_1639_v106	0.9432
Listeria_monocytogenes_1639_v2	Listeria_monocytogenes_1639_v106	0.9025
Listeria_monocytogenes_1639_v29	Listeria_monocytogenes_1639_v106	0.9128
Listeria_monocytogenes_1639_v28	Listeria_monocytogenes_1639_v106	0.9168
Listeria_monocytogenes_1639_v27	Listeria_monocytogenes_1639_v106	0.9081
Listeria_monocytogenes_1639_v26	Listeria_monocytogenes_1639_v106	0.9078
Listeria_monocytogenes_1639_v25	Listeria_monocytogenes_1639_v106	0.9078
Listeria_monocytogenes_1639_v24	Listeria_monocytogenes_1639_v106	0.9779
Listeria_monocytogenes_1639_v23	Listeria_monocytogenes_1639_v106	0.9775
Listeria_monocytogenes_1639_v22	Listeria_monocytogenes_1639_v106	0.9406
Listeria_monocytogenes_1639_v21	Listeria_monocytogenes_1639_v106	0.9775
Listeria_monocytogenes_1639_v20	Listeria_monocytogenes_1639_v106	0.931
Listeria_monocytogenes_1639_v1	Listeria_monocytogenes_1639_v106	0.9054
Listeria_monocytogenes_1639_v19	Listeria_monocytogenes_1639_v106	0.9306
Listeria_monocytogenes_1639_v198	Listeria_monocytogenes_1639_v106	0.8935
Listeria_monocytogenes_1639_v197	Listeria_monocytogenes_1639_v106	0.9219
Listeria_monocytogenes_1639_v196	Listeria_monocytogenes_1639_v106	0.9186
Listeria_monocytogenes_1639_v195	Listeria_monocytogenes_1639_v106	0.9424
Listeria_monocytogenes_1639_v194	Listeria_monocytogenes_1639_v106	0.9414
Listeria_monocytogenes_1639_v193	Listeria_monocytogenes_1639_v106	0.9414
Listeria_monocytogenes_1639_v192	Listeria_monocytogenes_1639_v106	0.9143
Listeria_monocytogenes_1639_v191	Listeria_monocytogenes_1639_v106	0.9482
Listeria_monocytogenes_1639_v190	Listeria_monocytogenes_1639_v106	0.9478
Listeria_monocytogenes_1639_v18	Listeria_monocytogenes_1639_v106	0.9409
Listeria_monocytogenes_1639_v189	Listeria_monocytogenes_1639_v106	0.9467
Listeria_monocytogenes_1639_v188	Listeria_monocytogenes_1639_v106	0.9493
Listeria_monocytogenes_1639_v187	Listeria_monocytogenes_1639_v106	0.9478
Listeria_monocytogenes_1639_v186	Listeria_monocytogenes_1639_v106	0.9478
Listeria_monocytogenes_1639_v185	Listeria_monocytogenes_1639_v106	0.9482
Listeria_monocytogenes_1639_v184	Listeria_monocytogenes_1639_v106	0.9474
Listeria_monocytogenes_1639_v183	Listeria_monocytogenes_1639_v106	0.9424
Listeria_monocytogenes_1639_v182	Listeria_monocytogenes_1639_v106	0.9446
Listeria_monocytogenes_1639_v181	Listeria_monocytogenes_1639_v106	0.935
Listeria_monocytogenes_1639_v180	Listeria_monocytogenes_1639_v106	0.935
Listeria_monocytogenes_1639_v17	Listeria_monocytogenes_1639_v106	0.9412
Listeria_monocytogenes_1639_v179	Listeria_monocytogenes_1639_v106	0.9323
Listeria_monocytogenes_1639_v178	Listeria_monocytogenes_1639_v106	0.949
Listeria_monocytogenes_1639_v177	Listeria_monocytogenes_1639_v106	0.9693
Listeria_monocytogenes_1639_v176	Listeria_monocytogenes_1639_v106	0.9422
Listeria_monocytogenes_1639_v175	Listeria_monocytogenes_1639_v106	0.9372
Listeria_monocytogenes_1639_v174	Listeria_monocytogenes_1639_v106	0.9479
Listeria_monocytogenes_1639_v173	Listeria_monocytogenes_1639_v106	0.9645
Listeria_monocytogenes_1639_v172	Listeria_monocytogenes_1639_v106	0.942
Listeria_monocytogenes_1639_v171	Listeria_monocytogenes_1639_v106	0.9338
Listeria_monocytogenes_1639_v170	Listeria_monocytogenes_1639_v106	0.9417
Listeria_monocytogenes_1639_v16	Listeria_monocytogenes_1639_v106	0.9071
Listeria_monocytogenes_1639_v169	Listeria_monocytogenes_1639_v106	0.9421
Listeria_monocytogenes_1639_v168	Listeria_monocytogenes_1639_v106	0.9426
Listeria_monocytogenes_1639_v167	Listeria_monocytogenes_1639_v106	0.9348
Listeria_monocytogenes_1639_v166	Listeria_monocytogenes_1639_v106	0.9429
Listeria_monocytogenes_1639_v165	Listeria_monocytogenes_1639_v106	0.9418
Listeria_monocytogenes_1639_v164	Listeria_monocytogenes_1639_v106	0.9758
Listeria_monocytogenes_1639_v163	Listeria_monocytogenes_1639_v106	0.9585
Listeria_monocytogenes_1639_v162	Listeria_monocytogenes_1639_v106	0.9758
Listeria_monocytogenes_1639_v161	Listeria_monocytogenes_1639_v106	0.9762
Listeria_monocytogenes_1639_v160	Listeria_monocytogenes_1639_v106	0.9758
Listeria_monocytogenes_1639_v15	Listeria_monocytogenes_1639_v106	0.9243
Listeria_monocytogenes_1639_v159	Listeria_monocytogenes_1639_v106	0.9697
Listeria_monocytogenes_1639_v158	Listeria_monocytogenes_1639_v106	0.9762
Listeria_monocytogenes_1639_v157	Listeria_monocytogenes_1639_v106	0.9762
Listeria_monocytogenes_1639_v156	Listeria_monocytogenes_1639_v106	0.9249
Listeria_monocytogenes_1639_v155	Listeria_monocytogenes_1639_v106	0.9373
Listeria_monocytogenes_1639_v154	Listeria_monocytogenes_1639_v106	0.9385
Listeria_monocytogenes_1639_v153	Listeria_monocytogenes_1639_v106	0.938
Listeria_monocytogenes_1639_v152	Listeria_monocytogenes_1639_v106	0.9697
Listeria_monocytogenes_1639_v151	Listeria_monocytogenes_1639_v106	0.9697
Listeria_monocytogenes_1639_v150	Listeria_monocytogenes_1639_v106	0.9377

Listeria_monocytogenes_1639_v14	Listeria_monocytogenes_1639_v106	0.9319
Listeria_monocytogenes_1639_v149	Listeria_monocytogenes_1639_v106	0.9363
Listeria_monocytogenes_1639_v148	Listeria_monocytogenes_1639_v106	0.9686
Listeria_monocytogenes_1639_v147	Listeria_monocytogenes_1639_v106	0.9551
Listeria_monocytogenes_1639_v146	Listeria_monocytogenes_1639_v106	0.9465
Listeria_monocytogenes_1639_v145	Listeria_monocytogenes_1639_v106	0.9431
Listeria_monocytogenes_1639_v144	Listeria_monocytogenes_1639_v106	0.9712
Listeria_monocytogenes_1639_v143	Listeria_monocytogenes_1639_v106	0.9473
Listeria_monocytogenes_1639_v142	Listeria_monocytogenes_1639_v106	0.9716
Listeria_monocytogenes_1639_v141	Listeria_monocytogenes_1639_v106	0.9638
Listeria_monocytogenes_1639_v140	Listeria_monocytogenes_1639_v106	0.9723
Listeria_monocytogenes_1639_v13	Listeria_monocytogenes_1639_v106	0.9243
Listeria_monocytogenes_1639_v139	Listeria_monocytogenes_1639_v106	0.9724
Listeria_monocytogenes_1639_v138	Listeria_monocytogenes_1639_v106	0.9739
Listeria_monocytogenes_1639_v137	Listeria_monocytogenes_1639_v106	0.9739
Listeria_monocytogenes_1639_v136	Listeria_monocytogenes_1639_v106	0.938
Listeria_monocytogenes_1639_v135	Listeria_monocytogenes_1639_v106	0.92
Listeria_monocytogenes_1639_v134	Listeria_monocytogenes_1639_v106	0.9801
Listeria_monocytogenes_1639_v133	Listeria_monocytogenes_1639_v106	0.9351
Listeria_monocytogenes_1639_v132	Listeria_monocytogenes_1639_v106	0.9205
Listeria_monocytogenes_1639_v131	Listeria_monocytogenes_1639_v106	0.9575
Listeria_monocytogenes_1639_v130	Listeria_monocytogenes_1639_v106	0.9258
Listeria_monocytogenes_1639_v12	Listeria_monocytogenes_1639_v106	0.9068
Listeria_monocytogenes_1639_v129	Listeria_monocytogenes_1639_v106	0.9639
Listeria_monocytogenes_1639_v128	Listeria_monocytogenes_1639_v106	0.9617
Listeria_monocytogenes_1639_v127	Listeria_monocytogenes_1639_v106	0.9342
Listeria_monocytogenes_1639_v126	Listeria_monocytogenes_1639_v106	0.9598
Listeria_monocytogenes_1639_v125	Listeria_monocytogenes_1639_v106	0.9343
Listeria_monocytogenes_1639_v124	Listeria_monocytogenes_1639_v106	0.9432
Listeria_monocytogenes_1639_v123	Listeria_monocytogenes_1639_v106	0.9716
Listeria_monocytogenes_1639_v122	Listeria_monocytogenes_1639_v106	0.9716
Listeria_monocytogenes_1639_v121	Listeria_monocytogenes_1639_v106	0.9716
Listeria_monocytogenes_1639_v120	Listeria_monocytogenes_1639_v106	0.9716
Listeria_monocytogenes_1639_v11	Listeria_monocytogenes_1639_v106	0.9081
Listeria_monocytogenes_1639_v119	Listeria_monocytogenes_1639_v106	0.9716
Listeria_monocytogenes_1639_v118	Listeria_monocytogenes_1639_v106	0.9716
Listeria_monocytogenes_1639_v117	Listeria_monocytogenes_1639_v106	0.9365
Listeria_monocytogenes_1639_v116	Listeria_monocytogenes_1639_v106	0.9813
Listeria_monocytogenes_1639_v115	Listeria_monocytogenes_1639_v106	0.9809
Listeria_monocytogenes_1639_v114	Listeria_monocytogenes_1639_v106	0.9295
Listeria_monocytogenes_1639_v113	Listeria_monocytogenes_1639_v106	0.9546
Listeria_monocytogenes_1639_v112	Listeria_monocytogenes_1639_v106	0.965
Listeria_monocytogenes_1639_v111	Listeria_monocytogenes_1639_v106	0.9461
Listeria_monocytogenes_1639_v110	Listeria_monocytogenes_1639_v106	0.9292
Listeria_monocytogenes_1639_v10	Listeria_monocytogenes_1639_v106	0.923
Listeria_monocytogenes_1639_v109	Listeria_monocytogenes_1639_v106	0.9288
Listeria_monocytogenes_1639_v108	Listeria_monocytogenes_1639_v106	0.9587
Listeria_monocytogenes_1639_v107	Listeria_monocytogenes_1639_v106	0.9981
Listeria_monocytogenes_1639_v106	Listeria_monocytogenes_1639_v106	0.0684
Listeria_welshimeri_1643	Listeria_monocytogenes_1639_v107	0.89
Listeria_welshimeri_serovar_6b_str_SLCC5334_386043	Listeria_monocytogenes_1639_v107	0.8897
Listeria_seeligeri_serovar_1_2b_str_SLCC3954_683837	Listeria_monocytogenes_1639_v107	0.8869
Listeria_seeligeri_FSL_S4-171_702452	Listeria_monocytogenes_1639_v107	0.8277
Listeria_seeligeri_FSL_N1-067_702453	Listeria_monocytogenes_1639_v107	0.8293
Listeria_seeligeri_1640_v1	Listeria_monocytogenes_1639_v107	0.8937
Listeria_seeligeri_1640	Listeria_monocytogenes_1639_v107	0.8804
Listeria_monocytogenes_WSLC1042_1457188	Listeria_monocytogenes_1639_v107	0.9794
Listeria_monocytogenes_WSLC1001_1457187	Listeria_monocytogenes_1639_v107	0.9306
Listeria_monocytogenes_SLCC7179_879090	Listeria_monocytogenes_1639_v107	0.9372
Listeria_monocytogenes_SLCC5850_882096	Listeria_monocytogenes_1639_v107	0.9348
Listeria_monocytogenes_SLCC2755_932919	Listeria_monocytogenes_1639_v107	0.944
Listeria_monocytogenes_SLCC2540_879089	Listeria_monocytogenes_1639_v107	0.9472
Listeria_monocytogenes_SLCC2479_882020	Listeria_monocytogenes_1639_v107	0.9254
Listeria_monocytogenes_SLCC2378_879088	Listeria_monocytogenes_1639_v107	0.9809
Listeria_monocytogenes_SLCC2376_882097	Listeria_monocytogenes_1639_v107	0.9406
Listeria_monocytogenes_SLCC2372_932920	Listeria_monocytogenes_1639_v107	0.9181
Listeria_monocytogenes_serotype_4b_str_LL195_1230340	Listeria_monocytogenes_1639_v107	0.9758
Listeria_monocytogenes_serotype_4b_str_LIS0087_1134669	Listeria_monocytogenes_1639_v107	0.9115
Listeria_monocytogenes_serotype_4b_str_F2365_265669	Listeria_monocytogenes_1639_v107	0.9758
Listeria_monocytogenes_serotype_4b_str_CLIP_80459_568819	Listeria_monocytogenes_1639_v107	0.9712
Listeria_monocytogenes_serotype_4b_str_81-0861_1196161	Listeria_monocytogenes_1639_v107	0.9694
Listeria_monocytogenes_serotype_4b_str_81-0592_1196160	Listeria_monocytogenes_1639_v107	0.9705
Listeria_monocytogenes_serotype_4b_str_81-0558_1196159	Listeria_monocytogenes_1639_v107	0.9698
Listeria_monocytogenes_serotype_4b_str_10-0809_1196162	Listeria_monocytogenes_1639_v107	0.9705
Listeria_monocytogenes_serotype_4b_str_02-6680_1196175	Listeria_monocytogenes_1639_v107	0.9781
Listeria_monocytogenes_serotype_4b_str_02-6679_1196174	Listeria_monocytogenes_1639_v107	0.9781
Listeria_monocytogenes_serotype_4b_str_02-1792_1196173	Listeria_monocytogenes_1639_v107	0.9698
Listeria_monocytogenes_serotype_4b_str_02-1289_1196172	Listeria_monocytogenes_1639_v107	0.9698
Listeria_monocytogenes_serotype_4b_str_02-1103_1196171	Listeria_monocytogenes_1639_v107	0.9698
Listeria_monocytogenes_serotype_3c_str_10-5027_1196195	Listeria_monocytogenes_1639_v107	0.9246
Listeria_monocytogenes_serotype_1_2c_str_10-5026_1196194	Listeria_monocytogenes_1639_v107	0.9254
Listeria_monocytogenes_serotype_1_2c_str_10-5025_1196193	Listeria_monocytogenes_1639_v107	0.9254
Listeria_monocytogenes_serotype_1_2b_str_10-0811_1196164	Listeria_monocytogenes_1639_v107	0.9381
Listeria_monocytogenes_serotype_1_2b_str_10-0810_1196163	Listeria_monocytogenes_1639_v107	0.9381
Listeria_monocytogenes_serotype_1_2a_str_99-6370_1196179	Listeria_monocytogenes_1639_v107	0.9144
Listeria_monocytogenes_serotype_1_2a_str_98-2035_1196178	Listeria_monocytogenes_1639_v107	0.9144
Listeria_monocytogenes_serotype_1_2a_str_95-0093_1196177	Listeria_monocytogenes_1639_v107	0.9199
Listeria_monocytogenes_serotype_1_2a_str_88-0478_1196176	Listeria_monocytogenes_1639_v107	0.9187
Listeria_monocytogenes_serotype_1_2a_str_10-5024_1196192	Listeria_monocytogenes_1639_v107	0.9199
Listeria_monocytogenes_serotype_1_2a_str_10-4758_1196168	Listeria_monocytogenes_1639_v107	0.938
Listeria_monocytogenes_serotype_1_2a_str_10-4754_1196167	Listeria_monocytogenes_1639_v107	0.9383

Listeria_monocytogenes_serotype_1_2a_str_10-1321_1196191	Listeria_monocytogenes_1639_v107	0.9199
Listeria_monocytogenes_serotype_1_2a_str_10-1047_1196190	Listeria_monocytogenes_1639_v107	0.9206
Listeria_monocytogenes_serotype_1_2a_str_10-1046_1196189	Listeria_monocytogenes_1639_v107	0.9206
Listeria_monocytogenes_serotype_1_2a_str_10-0934_1196170	Listeria_monocytogenes_1639_v107	0.9448
Listeria_monocytogenes_serotype_1_2a_str_10-0933_1196169	Listeria_monocytogenes_1639_v107	0.9448
Listeria_monocytogenes_serotype_1_2a_str_10-0815_1196188	Listeria_monocytogenes_1639_v107	0.9158
Listeria_monocytogenes_serotype_1_2a_str_10-0814_1196187	Listeria_monocytogenes_1639_v107	0.9203
Listeria_monocytogenes_serotype_1_2a_str_10-0813_1196166	Listeria_monocytogenes_1639_v107	0.9281
Listeria_monocytogenes_serotype_1_2a_str_10-0812_1196165	Listeria_monocytogenes_1639_v107	0.9281
Listeria_monocytogenes_serotype_1_2a_str_08-7669_1196186	Listeria_monocytogenes_1639_v107	0.9292
Listeria_monocytogenes_serotype_1_2a_str_08-7374_1196185	Listeria_monocytogenes_1639_v107	0.9199
Listeria_monocytogenes_serotype_1_2a_str_08-6997_1196184	Listeria_monocytogenes_1639_v107	0.9158
Listeria_monocytogenes_serotype_1_2a_str_08-6569_1196183	Listeria_monocytogenes_1639_v107	0.9158
Listeria_monocytogenes_serotype_1_2a_str_08-6056_1196182	Listeria_monocytogenes_1639_v107	0.9158
Listeria_monocytogenes_serotype_1_2a_str_04-5457_1196181	Listeria_monocytogenes_1639_v107	0.9199
Listeria_monocytogenes_serotype_1_2a_str_02-5993_1196180	Listeria_monocytogenes_1639_v107	0.9199
Listeria_monocytogenes_R479a_1437838	Listeria_monocytogenes_1639_v107	0.9192
Listeria_monocytogenes_N53-1_1234142	Listeria_monocytogenes_1639_v107	0.8908
Listeria_monocytogenes_M7_1030009	Listeria_monocytogenes_1639_v107	0.9054
Listeria_monocytogenes_LO28_393132	Listeria_monocytogenes_1639_v107	0.924
Listeria_monocytogenes_L99_563174	Listeria_monocytogenes_1639_v107	0.9058
Listeria_monocytogenes_L312_882094	Listeria_monocytogenes_1639_v107	0.9715
Listeria_monocytogenes_J1816_930781	Listeria_monocytogenes_1639_v107	0.95
Listeria_monocytogenes_J1-220_930782	Listeria_monocytogenes_1639_v107	0.9775
Listeria_monocytogenes_J0161_393130	Listeria_monocytogenes_1639_v107	0.927
Listeria_monocytogenes_HCC23_552536	Listeria_monocytogenes_1639_v107	0.905
Listeria_monocytogenes_FSL_R2-561_393126	Listeria_monocytogenes_1639_v107	0.9239
Listeria_monocytogenes_FSL_J1-208_393119	Listeria_monocytogenes_1639_v107	0.9203
Listeria_monocytogenes_FSL_J1-175_393118	Listeria_monocytogenes_1639_v107	0.9546
Listeria_monocytogenes_Finland_1998_393127	Listeria_monocytogenes_1639_v107	0.9464
Listeria_monocytogenes_EGD-e_169963_v1	Listeria_monocytogenes_1639_v107	0.9338
Listeria_monocytogenes_EGD-e_169963	Listeria_monocytogenes_1639_v107	0.9342
Listeria_monocytogenes_EGD_1334565	Listeria_monocytogenes_1639_v107	0.9355
Listeria_monocytogenes_CFSAN002349_1299895	Listeria_monocytogenes_1639_v107	0.9713
Listeria_monocytogenes_ATCC_19117_882095_v1	Listeria_monocytogenes_1639_v107	0.9985
Listeria_monocytogenes_ATCC_19117_882095	Listeria_monocytogenes_1639_v107	0.9981
Listeria_monocytogenes_6179_1288295	Listeria_monocytogenes_1639_v107	0.9109
Listeria_monocytogenes_1639_v9	Listeria_monocytogenes_1639_v107	0.9252
Listeria_monocytogenes_1639_v99	Listeria_monocytogenes_1639_v107	0.95
Listeria_monocytogenes_1639_v98	Listeria_monocytogenes_1639_v107	0.9246
Listeria_monocytogenes_1639_v97	Listeria_monocytogenes_1639_v107	0.9651
Listeria_monocytogenes_1639_v96	Listeria_monocytogenes_1639_v107	0.9493
Listeria_monocytogenes_1639_v95	Listeria_monocytogenes_1639_v107	0.9493
Listeria_monocytogenes_1639_v94	Listeria_monocytogenes_1639_v107	0.9496
Listeria_monocytogenes_1639_v93	Listeria_monocytogenes_1639_v107	0.9236
Listeria_monocytogenes_1639_v92	Listeria_monocytogenes_1639_v107	0.9496
Listeria_monocytogenes_1639_v91	Listeria_monocytogenes_1639_v107	0.9563
Listeria_monocytogenes_1639_v90	Listeria_monocytogenes_1639_v107	0.9246
Listeria_monocytogenes_1639_v8	Listeria_monocytogenes_1639_v107	0.9112
Listeria_monocytogenes_1639_v89	Listeria_monocytogenes_1639_v107	0.9665
Listeria_monocytogenes_1639_v88	Listeria_monocytogenes_1639_v107	0.9256
Listeria_monocytogenes_1639_v87	Listeria_monocytogenes_1639_v107	0.9244
Listeria_monocytogenes_1639_v86	Listeria_monocytogenes_1639_v107	0.924
Listeria_monocytogenes_1639_v85	Listeria_monocytogenes_1639_v107	0.9691
Listeria_monocytogenes_1639_v84	Listeria_monocytogenes_1639_v107	0.9311
Listeria_monocytogenes_1639_v83	Listeria_monocytogenes_1639_v107	0.9868
Listeria_monocytogenes_1639_v82	Listeria_monocytogenes_1639_v107	0.9864
Listeria_monocytogenes_1639_v81	Listeria_monocytogenes_1639_v107	0.9271
Listeria_monocytogenes_1639_v80	Listeria_monocytogenes_1639_v107	0.9443
Listeria_monocytogenes_1639_v7	Listeria_monocytogenes_1639_v107	0.9112
Listeria_monocytogenes_1639_v79	Listeria_monocytogenes_1639_v107	0.9253
Listeria_monocytogenes_1639_v78	Listeria_monocytogenes_1639_v107	0.9264
Listeria_monocytogenes_1639_v77	Listeria_monocytogenes_1639_v107	0.9306
Listeria_monocytogenes_1639_v76	Listeria_monocytogenes_1639_v107	0.922
Listeria_monocytogenes_1639_v75	Listeria_monocytogenes_1639_v107	0.9058
Listeria_monocytogenes_1639_v74	Listeria_monocytogenes_1639_v107	0.9228
Listeria_monocytogenes_1639_v73	Listeria_monocytogenes_1639_v107	0.9195
Listeria_monocytogenes_1639_v72	Listeria_monocytogenes_1639_v107	0.9852
Listeria_monocytogenes_1639_v71	Listeria_monocytogenes_1639_v107	0.9111
Listeria_monocytogenes_1639_v70	Listeria_monocytogenes_1639_v107	0.9215
Listeria_monocytogenes_1639_v6	Listeria_monocytogenes_1639_v107	0.9109
Listeria_monocytogenes_1639_v69	Listeria_monocytogenes_1639_v107	0.9705
Listeria_monocytogenes_1639_v68	Listeria_monocytogenes_1639_v107	0.9406
Listeria_monocytogenes_1639_v67	Listeria_monocytogenes_1639_v107	0.9441
Listeria_monocytogenes_1639_v66	Listeria_monocytogenes_1639_v107	0.9319
Listeria_monocytogenes_1639_v65	Listeria_monocytogenes_1639_v107	0.9226
Listeria_monocytogenes_1639_v64	Listeria_monocytogenes_1639_v107	0.926
Listeria_monocytogenes_1639_v63	Listeria_monocytogenes_1639_v107	0.9395
Listeria_monocytogenes_1639_v62	Listeria_monocytogenes_1639_v107	0.9225
Listeria_monocytogenes_1639_v61	Listeria_monocytogenes_1639_v107	0.9226
Listeria_monocytogenes_1639_v60	Listeria_monocytogenes_1639_v107	0.9289
Listeria_monocytogenes_1639_v5	Listeria_monocytogenes_1639_v107	0.9057
Listeria_monocytogenes_1639_v59	Listeria_monocytogenes_1639_v107	0.9871
Listeria_monocytogenes_1639_v58	Listeria_monocytogenes_1639_v107	0.9762
Listeria_monocytogenes_1639_v57	Listeria_monocytogenes_1639_v107	0.9465
Listeria_monocytogenes_1639_v56	Listeria_monocytogenes_1639_v107	0.9871
Listeria_monocytogenes_1639_v55	Listeria_monocytogenes_1639_v107	0.9422
Listeria_monocytogenes_1639_v54	Listeria_monocytogenes_1639_v107	0.925
Listeria_monocytogenes_1639_v53	Listeria_monocytogenes_1639_v107	0.9293
Listeria_monocytogenes_1639_v52	Listeria_monocytogenes_1639_v107	0.9736
Listeria_monocytogenes_1639_v51	Listeria_monocytogenes_1639_v107	0.9808

Listeria_monocytogenes_1639_v50	Listeria_monocytogenes_1639_v107	0.9191
Listeria_monocytogenes_1639_v4	Listeria_monocytogenes_1639_v107	0.919
Listeria_monocytogenes_1639_v49	Listeria_monocytogenes_1639_v107	0.9679
Listeria_monocytogenes_1639_v48	Listeria_monocytogenes_1639_v107	0.9198
Listeria_monocytogenes_1639_v47	Listeria_monocytogenes_1639_v107	0.9195
Listeria_monocytogenes_1639_v46	Listeria_monocytogenes_1639_v107	0.9399
Listeria_monocytogenes_1639_v45	Listeria_monocytogenes_1639_v107	0.9111
Listeria_monocytogenes_1639_v44	Listeria_monocytogenes_1639_v107	0.9199
Listeria_monocytogenes_1639_v43	Listeria_monocytogenes_1639_v107	0.9199
Listeria_monocytogenes_1639_v42	Listeria_monocytogenes_1639_v107	0.9144
Listeria_monocytogenes_1639_v41	Listeria_monocytogenes_1639_v107	0.9014
Listeria_monocytogenes_1639_v40	Listeria_monocytogenes_1639_v107	0.9243
Listeria_monocytogenes_1639_v3	Listeria_monocytogenes_1639_v107	0.9193
Listeria_monocytogenes_1639_v39	Listeria_monocytogenes_1639_v107	0.9432
Listeria_monocytogenes_1639_v38	Listeria_monocytogenes_1639_v107	0.9298
Listeria_monocytogenes_1639_v37	Listeria_monocytogenes_1639_v107	0.9156
Listeria_monocytogenes_1639_v36	Listeria_monocytogenes_1639_v107	0.9173
Listeria_monocytogenes_1639_v35	Listeria_monocytogenes_1639_v107	0.925
Listeria_monocytogenes_1639_v34	Listeria_monocytogenes_1639_v107	0.9325
Listeria_monocytogenes_1639_v33	Listeria_monocytogenes_1639_v107	0.9381
Listeria_monocytogenes_1639_v32	Listeria_monocytogenes_1639_v107	0.9146
Listeria_monocytogenes_1639_v31	Listeria_monocytogenes_1639_v107	0.9329
Listeria_monocytogenes_1639_v30	Listeria_monocytogenes_1639_v107	0.9429
Listeria_monocytogenes_1639_v2	Listeria_monocytogenes_1639_v107	0.9021
Listeria_monocytogenes_1639_v29	Listeria_monocytogenes_1639_v107	0.9124
Listeria_monocytogenes_1639_v28	Listeria_monocytogenes_1639_v107	0.9164
Listeria_monocytogenes_1639_v27	Listeria_monocytogenes_1639_v107	0.9077
Listeria_monocytogenes_1639_v26	Listeria_monocytogenes_1639_v107	0.9074
Listeria_monocytogenes_1639_v25	Listeria_monocytogenes_1639_v107	0.9074
Listeria_monocytogenes_1639_v24	Listeria_monocytogenes_1639_v107	0.9775
Listeria_monocytogenes_1639_v23	Listeria_monocytogenes_1639_v107	0.9772
Listeria_monocytogenes_1639_v22	Listeria_monocytogenes_1639_v107	0.9402
Listeria_monocytogenes_1639_v21	Listeria_monocytogenes_1639_v107	0.9772
Listeria_monocytogenes_1639_v20	Listeria_monocytogenes_1639_v107	0.9306
Listeria_monocytogenes_1639_v1	Listeria_monocytogenes_1639_v107	0.905
Listeria_monocytogenes_1639_v19	Listeria_monocytogenes_1639_v107	0.9302
Listeria_monocytogenes_1639_v198	Listeria_monocytogenes_1639_v107	0.8935
Listeria_monocytogenes_1639_v197	Listeria_monocytogenes_1639_v107	0.9215
Listeria_monocytogenes_1639_v196	Listeria_monocytogenes_1639_v107	0.9182
Listeria_monocytogenes_1639_v195	Listeria_monocytogenes_1639_v107	0.942
Listeria_monocytogenes_1639_v194	Listeria_monocytogenes_1639_v107	0.941
Listeria_monocytogenes_1639_v193	Listeria_monocytogenes_1639_v107	0.941
Listeria_monocytogenes_1639_v192	Listeria_monocytogenes_1639_v107	0.9139
Listeria_monocytogenes_1639_v191	Listeria_monocytogenes_1639_v107	0.9482
Listeria_monocytogenes_1639_v190	Listeria_monocytogenes_1639_v107	0.9474
Listeria_monocytogenes_1639_v18	Listeria_monocytogenes_1639_v107	0.9405
Listeria_monocytogenes_1639_v189	Listeria_monocytogenes_1639_v107	0.9463
Listeria_monocytogenes_1639_v188	Listeria_monocytogenes_1639_v107	0.9485
Listeria_monocytogenes_1639_v187	Listeria_monocytogenes_1639_v107	0.9474
Listeria_monocytogenes_1639_v186	Listeria_monocytogenes_1639_v107	0.9474
Listeria_monocytogenes_1639_v185	Listeria_monocytogenes_1639_v107	0.9478
Listeria_monocytogenes_1639_v184	Listeria_monocytogenes_1639_v107	0.947
Listeria_monocytogenes_1639_v183	Listeria_monocytogenes_1639_v107	0.9424
Listeria_monocytogenes_1639_v182	Listeria_monocytogenes_1639_v107	0.9442
Listeria_monocytogenes_1639_v181	Listeria_monocytogenes_1639_v107	0.9346
Listeria_monocytogenes_1639_v180	Listeria_monocytogenes_1639_v107	0.9346
Listeria_monocytogenes_1639_v17	Listeria_monocytogenes_1639_v107	0.9409
Listeria_monocytogenes_1639_v179	Listeria_monocytogenes_1639_v107	0.9319
Listeria_monocytogenes_1639_v178	Listeria_monocytogenes_1639_v107	0.9486
Listeria_monocytogenes_1639_v177	Listeria_monocytogenes_1639_v107	0.9693
Listeria_monocytogenes_1639_v176	Listeria_monocytogenes_1639_v107	0.9418
Listeria_monocytogenes_1639_v175	Listeria_monocytogenes_1639_v107	0.9368
Listeria_monocytogenes_1639_v174	Listeria_monocytogenes_1639_v107	0.9475
Listeria_monocytogenes_1639_v173	Listeria_monocytogenes_1639_v107	0.9641
Listeria_monocytogenes_1639_v172	Listeria_monocytogenes_1639_v107	0.9417
Listeria_monocytogenes_1639_v171	Listeria_monocytogenes_1639_v107	0.9334
Listeria_monocytogenes_1639_v170	Listeria_monocytogenes_1639_v107	0.9413
Listeria_monocytogenes_1639_v16	Listeria_monocytogenes_1639_v107	0.9067
Listeria_monocytogenes_1639_v169	Listeria_monocytogenes_1639_v107	0.9417
Listeria_monocytogenes_1639_v168	Listeria_monocytogenes_1639_v107	0.9418
Listeria_monocytogenes_1639_v167	Listeria_monocytogenes_1639_v107	0.9344
Listeria_monocytogenes_1639_v166	Listeria_monocytogenes_1639_v107	0.9422
Listeria_monocytogenes_1639_v165	Listeria_monocytogenes_1639_v107	0.9414
Listeria_monocytogenes_1639_v164	Listeria_monocytogenes_1639_v107	0.9754
Listeria_monocytogenes_1639_v163	Listeria_monocytogenes_1639_v107	0.9585
Listeria_monocytogenes_1639_v162	Listeria_monocytogenes_1639_v107	0.9754
Listeria_monocytogenes_1639_v161	Listeria_monocytogenes_1639_v107	0.9758
Listeria_monocytogenes_1639_v160	Listeria_monocytogenes_1639_v107	0.9758
Listeria_monocytogenes_1639_v15	Listeria_monocytogenes_1639_v107	0.924
Listeria_monocytogenes_1639_v159	Listeria_monocytogenes_1639_v107	0.9693
Listeria_monocytogenes_1639_v158	Listeria_monocytogenes_1639_v107	0.9758
Listeria_monocytogenes_1639_v157	Listeria_monocytogenes_1639_v107	0.9758
Listeria_monocytogenes_1639_v156	Listeria_monocytogenes_1639_v107	0.9246
Listeria_monocytogenes_1639_v155	Listeria_monocytogenes_1639_v107	0.9365
Listeria_monocytogenes_1639_v154	Listeria_monocytogenes_1639_v107	0.9381
Listeria_monocytogenes_1639_v153	Listeria_monocytogenes_1639_v107	0.9373
Listeria_monocytogenes_1639_v152	Listeria_monocytogenes_1639_v107	0.9693
Listeria_monocytogenes_1639_v151	Listeria_monocytogenes_1639_v107	0.9693
Listeria_monocytogenes_1639_v150	Listeria_monocytogenes_1639_v107	0.9373
Listeria_monocytogenes_1639_v14	Listeria_monocytogenes_1639_v107	0.9316
Listeria_monocytogenes_1639_v149	Listeria_monocytogenes_1639_v107	0.9359

Listeria_monocytogenes_1639_v148	Listeria_monocytogenes_1639_v107	0.9682
Listeria_monocytogenes_1639_v147	Listeria_monocytogenes_1639_v107	0.9547
Listeria_monocytogenes_1639_v146	Listeria_monocytogenes_1639_v107	0.9461
Listeria_monocytogenes_1639_v145	Listeria_monocytogenes_1639_v107	0.9431
Listeria_monocytogenes_1639_v144	Listeria_monocytogenes_1639_v107	0.9712
Listeria_monocytogenes_1639_v143	Listeria_monocytogenes_1639_v107	0.9465
Listeria_monocytogenes_1639_v142	Listeria_monocytogenes_1639_v107	0.9716
Listeria_monocytogenes_1639_v141	Listeria_monocytogenes_1639_v107	0.9634
Listeria_monocytogenes_1639_v140	Listeria_monocytogenes_1639_v107	0.9719
Listeria_monocytogenes_1639_v13	Listeria_monocytogenes_1639_v107	0.924
Listeria_monocytogenes_1639_v139	Listeria_monocytogenes_1639_v107	0.972
Listeria_monocytogenes_1639_v138	Listeria_monocytogenes_1639_v107	0.9739
Listeria_monocytogenes_1639_v137	Listeria_monocytogenes_1639_v107	0.9735
Listeria_monocytogenes_1639_v136	Listeria_monocytogenes_1639_v107	0.9376
Listeria_monocytogenes_1639_v135	Listeria_monocytogenes_1639_v107	0.9196
Listeria_monocytogenes_1639_v134	Listeria_monocytogenes_1639_v107	0.9797
Listeria_monocytogenes_1639_v133	Listeria_monocytogenes_1639_v107	0.9347
Listeria_monocytogenes_1639_v132	Listeria_monocytogenes_1639_v107	0.9201
Listeria_monocytogenes_1639_v131	Listeria_monocytogenes_1639_v107	0.9571
Listeria_monocytogenes_1639_v130	Listeria_monocytogenes_1639_v107	0.9254
Listeria_monocytogenes_1639_v12	Listeria_monocytogenes_1639_v107	0.9064
Listeria_monocytogenes_1639_v129	Listeria_monocytogenes_1639_v107	0.9635
Listeria_monocytogenes_1639_v128	Listeria_monocytogenes_1639_v107	0.9613
Listeria_monocytogenes_1639_v127	Listeria_monocytogenes_1639_v107	0.9338
Listeria_monocytogenes_1639_v126	Listeria_monocytogenes_1639_v107	0.9594
Listeria_monocytogenes_1639_v125	Listeria_monocytogenes_1639_v107	0.9336
Listeria_monocytogenes_1639_v124	Listeria_monocytogenes_1639_v107	0.9428
Listeria_monocytogenes_1639_v123	Listeria_monocytogenes_1639_v107	0.9713
Listeria_monocytogenes_1639_v122	Listeria_monocytogenes_1639_v107	0.9713
Listeria_monocytogenes_1639_v121	Listeria_monocytogenes_1639_v107	0.9713
Listeria_monocytogenes_1639_v120	Listeria_monocytogenes_1639_v107	0.9716
Listeria_monocytogenes_1639_v11	Listeria_monocytogenes_1639_v107	0.9077
Listeria_monocytogenes_1639_v119	Listeria_monocytogenes_1639_v107	0.9713
Listeria_monocytogenes_1639_v118	Listeria_monocytogenes_1639_v107	0.9713
Listeria_monocytogenes_1639_v117	Listeria_monocytogenes_1639_v107	0.9361
Listeria_monocytogenes_1639_v116	Listeria_monocytogenes_1639_v107	0.9809
Listeria_monocytogenes_1639_v115	Listeria_monocytogenes_1639_v107	0.9805
Listeria_monocytogenes_1639_v114	Listeria_monocytogenes_1639_v107	0.9292
Listeria_monocytogenes_1639_v113	Listeria_monocytogenes_1639_v107	0.9542
Listeria_monocytogenes_1639_v112	Listeria_monocytogenes_1639_v107	0.965
Listeria_monocytogenes_1639_v111	Listeria_monocytogenes_1639_v107	0.9457
Listeria_monocytogenes_1639_v110	Listeria_monocytogenes_1639_v107	0.9288
Listeria_monocytogenes_1639_v10	Listeria_monocytogenes_1639_v107	0.9223
Listeria_monocytogenes_1639_v109	Listeria_monocytogenes_1639_v107	0.9284
Listeria_monocytogenes_1639_v108	Listeria_monocytogenes_1639_v107	0.9583
Listeria_monocytogenes_1639_v107	Listeria_monocytogenes_1639_v107	0.0711
Listeria_welshimeri_1643	Listeria_monocytogenes_1639_v108	0.875
Listeria_welshimeri_serovar_6b_str_SLCC5334_386043	Listeria_monocytogenes_1639_v108	0.8747
Listeria_seeligeri_serovar_1_2b_str_SLCC3954_683837	Listeria_monocytogenes_1639_v108	0.8798
Listeria_seeligeri_FSL_S4-171_702452	Listeria_monocytogenes_1639_v108	0.8257
Listeria_seeligeri_FSL_N1-067_702453	Listeria_monocytogenes_1639_v108	0.8319
Listeria_seeligeri_1640_v1	Listeria_monocytogenes_1639_v108	0.8874
Listeria_seeligeri_1640	Listeria_monocytogenes_1639_v108	0.8908
Listeria_monocytogenes_WSLC1042_1457188	Listeria_monocytogenes_1639_v108	0.9573
Listeria_monocytogenes_WSLC1001_1457187	Listeria_monocytogenes_1639_v108	0.93
Listeria_monocytogenes_SLCC7179_879090	Listeria_monocytogenes_1639_v108	0.9396
Listeria_monocytogenes_SLCC5850_882096	Listeria_monocytogenes_1639_v108	0.9342
Listeria_monocytogenes_SLCC2755_932919	Listeria_monocytogenes_1639_v108	0.9397
Listeria_monocytogenes_SLCC2540_879089	Listeria_monocytogenes_1639_v108	0.942
Listeria_monocytogenes_SLCC2479_882020	Listeria_monocytogenes_1639_v108	0.929
Listeria_monocytogenes_SLCC2378_879088	Listeria_monocytogenes_1639_v108	0.9584
Listeria_monocytogenes_SLCC2376_882097	Listeria_monocytogenes_1639_v108	0.9365
Listeria_monocytogenes_SLCC2372_932920	Listeria_monocytogenes_1639_v108	0.9218
Listeria_monocytogenes_serotype_4b_str_LL195_1230340	Listeria_monocytogenes_1639_v108	0.9642
Listeria_monocytogenes_serotype_4b_str_LIS0087_1134669	Listeria_monocytogenes_1639_v108	0.9195
Listeria_monocytogenes_serotype_4b_str_F2365_265669	Listeria_monocytogenes_1639_v108	0.9642
Listeria_monocytogenes_serotype_4b_str_CLIP_80459_568819	Listeria_monocytogenes_1639_v108	0.9657
Listeria_monocytogenes_serotype_4b_str_81-0861_1196161	Listeria_monocytogenes_1639_v108	0.9596
Listeria_monocytogenes_serotype_4b_str_81-0592_1196160	Listeria_monocytogenes_1639_v108	0.9607
Listeria_monocytogenes_serotype_4b_str_81-0558_1196159	Listeria_monocytogenes_1639_v108	0.9603
Listeria_monocytogenes_serotype_4b_str_10-0809_1196162	Listeria_monocytogenes_1639_v108	0.9611
Listeria_monocytogenes_serotype_4b_str_02-6680_1196175	Listeria_monocytogenes_1639_v108	0.9631
Listeria_monocytogenes_serotype_4b_str_02-6679_1196174	Listeria_monocytogenes_1639_v108	0.9631
Listeria_monocytogenes_serotype_4b_str_02-1792_1196173	Listeria_monocytogenes_1639_v108	0.9622
Listeria_monocytogenes_serotype_4b_str_02-1289_1196172	Listeria_monocytogenes_1639_v108	0.9622
Listeria_monocytogenes_serotype_4b_str_02-1103_1196171	Listeria_monocytogenes_1639_v108	0.9622
Listeria_monocytogenes_serotype_3c_str_10-5027_1196195	Listeria_monocytogenes_1639_v108	0.9279
Listeria_monocytogenes_serotype_1_2c_str_10-5026_1196194	Listeria_monocytogenes_1639_v108	0.9279
Listeria_monocytogenes_serotype_1_2c_str_10-5025_1196193	Listeria_monocytogenes_1639_v108	0.9279
Listeria_monocytogenes_serotype_1_2b_str_10-0811_1196164	Listeria_monocytogenes_1639_v108	0.9468
Listeria_monocytogenes_serotype_1_2b_str_10-0810_1196163	Listeria_monocytogenes_1639_v108	0.9468
Listeria_monocytogenes_serotype_1_2a_str_99-6370_1196179	Listeria_monocytogenes_1639_v108	0.9188
Listeria_monocytogenes_serotype_1_2a_str_98-2035_1196178	Listeria_monocytogenes_1639_v108	0.9188
Listeria_monocytogenes_serotype_1_2a_str_95-0093_1196177	Listeria_monocytogenes_1639_v108	0.9232
Listeria_monocytogenes_serotype_1_2a_str_88-0478_1196176	Listeria_monocytogenes_1639_v108	0.9224
Listeria_monocytogenes_serotype_1_2a_str_10-5024_1196192	Listeria_monocytogenes_1639_v108	0.9232
Listeria_monocytogenes_serotype_1_2a_str_10-4758_1196168	Listeria_monocytogenes_1639_v108	0.9343
Listeria_monocytogenes_serotype_1_2a_str_10-4754_1196167	Listeria_monocytogenes_1639_v108	0.9343
Listeria_monocytogenes_serotype_1_2a_str_10-1321_1196191	Listeria_monocytogenes_1639_v108	0.9232
Listeria_monocytogenes_serotype_1_2a_str_10-1047_1196190	Listeria_monocytogenes_1639_v108	0.9238
Listeria_monocytogenes_serotype_1_2a_str_10-1046_1196189	Listeria_monocytogenes_1639_v108	0.9238

Listeria_monocytogenes_serotype_1_2a_str_10-0934_1196170	Listeria_monocytogenes_1639_v108	0.9407
Listeria_monocytogenes_serotype_1_2a_str_10-0933_1196169	Listeria_monocytogenes_1639_v108	0.9407
Listeria_monocytogenes_serotype_1_2a_str_10-0815_1196188	Listeria_monocytogenes_1639_v108	0.9195
Listeria_monocytogenes_serotype_1_2a_str_10-0814_1196187	Listeria_monocytogenes_1639_v108	0.9235
Listeria_monocytogenes_serotype_1_2a_str_10-0813_1196166	Listeria_monocytogenes_1639_v108	0.9359
Listeria_monocytogenes_serotype_1_2a_str_10-0812_1196165	Listeria_monocytogenes_1639_v108	0.9359
Listeria_monocytogenes_serotype_1_2a_str_08-7669_1196186	Listeria_monocytogenes_1639_v108	0.9317
Listeria_monocytogenes_serotype_1_2a_str_08-7374_1196185	Listeria_monocytogenes_1639_v108	0.9232
Listeria_monocytogenes_serotype_1_2a_str_08-6997_1196184	Listeria_monocytogenes_1639_v108	0.9195
Listeria_monocytogenes_serotype_1_2a_str_08-6569_1196183	Listeria_monocytogenes_1639_v108	0.9195
Listeria_monocytogenes_serotype_1_2a_str_08-6056_1196182	Listeria_monocytogenes_1639_v108	0.9195
Listeria_monocytogenes_serotype_1_2a_str_04-5457_1196181	Listeria_monocytogenes_1639_v108	0.9232
Listeria_monocytogenes_serotype_1_2a_str_02-5993_1196180	Listeria_monocytogenes_1639_v108	0.9232
Listeria_monocytogenes_R479a_1437838	Listeria_monocytogenes_1639_v108	0.9203
Listeria_monocytogenes_N53-1_1234142	Listeria_monocytogenes_1639_v108	0.8925
Listeria_monocytogenes_M7_1030009	Listeria_monocytogenes_1639_v108	0.914
Listeria_monocytogenes_LO28_393132	Listeria_monocytogenes_1639_v108	0.9325
Listeria_monocytogenes_L99_563174	Listeria_monocytogenes_1639_v108	0.9143
Listeria_monocytogenes_L312_882094	Listeria_monocytogenes_1639_v108	0.9661
Listeria_monocytogenes_J1816_930781	Listeria_monocytogenes_1639_v108	0.9739
Listeria_monocytogenes_J1-220_930782	Listeria_monocytogenes_1639_v108	0.9547
Listeria_monocytogenes_J0161_393130	Listeria_monocytogenes_1639_v108	0.9321
Listeria_monocytogenes_HCC23_552536	Listeria_monocytogenes_1639_v108	0.9136
Listeria_monocytogenes_FSL_R2-561_393126	Listeria_monocytogenes_1639_v108	0.9272
Listeria_monocytogenes_FSL_J1-208_393119	Listeria_monocytogenes_1639_v108	0.9092
Listeria_monocytogenes_FSL_J1-175_393118	Listeria_monocytogenes_1639_v108	0.9482
Listeria_monocytogenes_Finland_1998_393127	Listeria_monocytogenes_1639_v108	0.9415
Listeria_monocytogenes_EGD-e_169963_v1	Listeria_monocytogenes_1639_v108	0.9431
Listeria_monocytogenes_EGD-e_169963	Listeria_monocytogenes_1639_v108	0.9435
Listeria_monocytogenes_EGD_1334565	Listeria_monocytogenes_1639_v108	0.935
Listeria_monocytogenes_CFSAN002349_1299895	Listeria_monocytogenes_1639_v108	0.9715
Listeria_monocytogenes_ATCC_19117_882095_v1	Listeria_monocytogenes_1639_v108	0.9598
Listeria_monocytogenes_ATCC_19117_882095	Listeria_monocytogenes_1639_v108	0.9594
Listeria_monocytogenes_6179_1288295	Listeria_monocytogenes_1639_v108	0.9183
Listeria_monocytogenes_1639_v9	Listeria_monocytogenes_1639_v108	0.9314
Listeria_monocytogenes_1639_v99	Listeria_monocytogenes_1639_v108	0.9788
Listeria_monocytogenes_1639_v98	Listeria_monocytogenes_1639_v108	0.9343
Listeria_monocytogenes_1639_v97	Listeria_monocytogenes_1639_v108	0.9635
Listeria_monocytogenes_1639_v96	Listeria_monocytogenes_1639_v108	0.975
Listeria_monocytogenes_1639_v95	Listeria_monocytogenes_1639_v108	0.9751
Listeria_monocytogenes_1639_v94	Listeria_monocytogenes_1639_v108	0.9754
Listeria_monocytogenes_1639_v93	Listeria_monocytogenes_1639_v108	0.919
Listeria_monocytogenes_1639_v92	Listeria_monocytogenes_1639_v108	0.9814
Listeria_monocytogenes_1639_v91	Listeria_monocytogenes_1639_v108	0.9597
Listeria_monocytogenes_1639_v90	Listeria_monocytogenes_1639_v108	0.9343
Listeria_monocytogenes_1639_v8	Listeria_monocytogenes_1639_v108	0.9098
Listeria_monocytogenes_1639_v89	Listeria_monocytogenes_1639_v108	0.9796
Listeria_monocytogenes_1639_v88	Listeria_monocytogenes_1639_v108	0.9285
Listeria_monocytogenes_1639_v87	Listeria_monocytogenes_1639_v108	0.931
Listeria_monocytogenes_1639_v86	Listeria_monocytogenes_1639_v108	0.9306
Listeria_monocytogenes_1639_v85	Listeria_monocytogenes_1639_v108	0.9593
Listeria_monocytogenes_1639_v84	Listeria_monocytogenes_1639_v108	0.9339
Listeria_monocytogenes_1639_v83	Listeria_monocytogenes_1639_v108	0.9595
Listeria_monocytogenes_1639_v82	Listeria_monocytogenes_1639_v108	0.9588
Listeria_monocytogenes_1639_v81	Listeria_monocytogenes_1639_v108	0.9299
Listeria_monocytogenes_1639_v80	Listeria_monocytogenes_1639_v108	0.9501
Listeria_monocytogenes_1639_v7	Listeria_monocytogenes_1639_v108	0.9098
Listeria_monocytogenes_1639_v79	Listeria_monocytogenes_1639_v108	0.9282
Listeria_monocytogenes_1639_v78	Listeria_monocytogenes_1639_v108	0.9289
Listeria_monocytogenes_1639_v77	Listeria_monocytogenes_1639_v108	0.9286
Listeria_monocytogenes_1639_v76	Listeria_monocytogenes_1639_v108	0.9223
Listeria_monocytogenes_1639_v75	Listeria_monocytogenes_1639_v108	0.9143
Listeria_monocytogenes_1639_v74	Listeria_monocytogenes_1639_v108	0.9276
Listeria_monocytogenes_1639_v73	Listeria_monocytogenes_1639_v108	0.9276
Listeria_monocytogenes_1639_v72	Listeria_monocytogenes_1639_v108	0.9614
Listeria_monocytogenes_1639_v71	Listeria_monocytogenes_1639_v108	0.9115
Listeria_monocytogenes_1639_v70	Listeria_monocytogenes_1639_v108	0.9263
Listeria_monocytogenes_1639_v6	Listeria_monocytogenes_1639_v108	0.9095
Listeria_monocytogenes_1639_v69	Listeria_monocytogenes_1639_v108	0.9607
Listeria_monocytogenes_1639_v68	Listeria_monocytogenes_1639_v108	0.934
Listeria_monocytogenes_1639_v67	Listeria_monocytogenes_1639_v108	0.9408
Listeria_monocytogenes_1639_v66	Listeria_monocytogenes_1639_v108	0.9295
Listeria_monocytogenes_1639_v65	Listeria_monocytogenes_1639_v108	0.9319
Listeria_monocytogenes_1639_v64	Listeria_monocytogenes_1639_v108	0.9203
Listeria_monocytogenes_1639_v63	Listeria_monocytogenes_1639_v108	0.9419
Listeria_monocytogenes_1639_v62	Listeria_monocytogenes_1639_v108	0.9243
Listeria_monocytogenes_1639_v61	Listeria_monocytogenes_1639_v108	0.9263
Listeria_monocytogenes_1639_v60	Listeria_monocytogenes_1639_v108	0.9284
Listeria_monocytogenes_1639_v5	Listeria_monocytogenes_1639_v108	0.9129
Listeria_monocytogenes_1639_v59	Listeria_monocytogenes_1639_v108	0.9527
Listeria_monocytogenes_1639_v58	Listeria_monocytogenes_1639_v108	0.9632
Listeria_monocytogenes_1639_v57	Listeria_monocytogenes_1639_v108	0.9737
Listeria_monocytogenes_1639_v56	Listeria_monocytogenes_1639_v108	0.9557
Listeria_monocytogenes_1639_v55	Listeria_monocytogenes_1639_v108	0.9797
Listeria_monocytogenes_1639_v54	Listeria_monocytogenes_1639_v108	0.9211
Listeria_monocytogenes_1639_v53	Listeria_monocytogenes_1639_v108	0.9235
Listeria_monocytogenes_1639_v52	Listeria_monocytogenes_1639_v108	0.96
Listeria_monocytogenes_1639_v51	Listeria_monocytogenes_1639_v108	0.9556
Listeria_monocytogenes_1639_v50	Listeria_monocytogenes_1639_v108	0.9224
Listeria_monocytogenes_1639_v4	Listeria_monocytogenes_1639_v108	0.9262
Listeria_monocytogenes_1639_v49	Listeria_monocytogenes_1639_v108	0.9499

Listeria_monocytogenes_1639_v48	Listeria_monocytogenes_1639_v108	0.9235
Listeria_monocytogenes_1639_v47	Listeria_monocytogenes_1639_v108	0.9232
Listeria_monocytogenes_1639_v46	Listeria_monocytogenes_1639_v108	0.9281
Listeria_monocytogenes_1639_v45	Listeria_monocytogenes_1639_v108	0.9144
Listeria_monocytogenes_1639_v44	Listeria_monocytogenes_1639_v108	0.9232
Listeria_monocytogenes_1639_v43	Listeria_monocytogenes_1639_v108	0.9232
Listeria_monocytogenes_1639_v42	Listeria_monocytogenes_1639_v108	0.911
Listeria_monocytogenes_1639_v41	Listeria_monocytogenes_1639_v108	0.9059
Listeria_monocytogenes_1639_v40	Listeria_monocytogenes_1639_v108	0.9231
Listeria_monocytogenes_1639_v3	Listeria_monocytogenes_1639_v108	0.9265
Listeria_monocytogenes_1639_v39	Listeria_monocytogenes_1639_v108	0.9407
Listeria_monocytogenes_1639_v38	Listeria_monocytogenes_1639_v108	0.9207
Listeria_monocytogenes_1639_v37	Listeria_monocytogenes_1639_v108	0.9182
Listeria_monocytogenes_1639_v36	Listeria_monocytogenes_1639_v108	0.915
Listeria_monocytogenes_1639_v35	Listeria_monocytogenes_1639_v108	0.9241
Listeria_monocytogenes_1639_v34	Listeria_monocytogenes_1639_v108	0.9212
Listeria_monocytogenes_1639_v33	Listeria_monocytogenes_1639_v108	0.9468
Listeria_monocytogenes_1639_v32	Listeria_monocytogenes_1639_v108	0.9257
Listeria_monocytogenes_1639_v31	Listeria_monocytogenes_1639_v108	0.9216
Listeria_monocytogenes_1639_v30	Listeria_monocytogenes_1639_v108	0.9381
Listeria_monocytogenes_1639_v2	Listeria_monocytogenes_1639_v108	0.9108
Listeria_monocytogenes_1639_v29	Listeria_monocytogenes_1639_v108	0.9139
Listeria_monocytogenes_1639_v28	Listeria_monocytogenes_1639_v108	0.9197
Listeria_monocytogenes_1639_v27	Listeria_monocytogenes_1639_v108	0.9154
Listeria_monocytogenes_1639_v26	Listeria_monocytogenes_1639_v108	0.9151
Listeria_monocytogenes_1639_v25	Listeria_monocytogenes_1639_v108	0.9151
Listeria_monocytogenes_1639_v24	Listeria_monocytogenes_1639_v108	0.9547
Listeria_monocytogenes_1639_v23	Listeria_monocytogenes_1639_v108	0.954
Listeria_monocytogenes_1639_v22	Listeria_monocytogenes_1639_v108	0.9501
Listeria_monocytogenes_1639_v21	Listeria_monocytogenes_1639_v108	0.9528
Listeria_monocytogenes_1639_v20	Listeria_monocytogenes_1639_v108	0.933
Listeria_monocytogenes_1639_v1	Listeria_monocytogenes_1639_v108	0.9135
Listeria_monocytogenes_1639_v19	Listeria_monocytogenes_1639_v108	0.9327
Listeria_monocytogenes_1639_v198	Listeria_monocytogenes_1639_v108	0.8937
Listeria_monocytogenes_1639_v197	Listeria_monocytogenes_1639_v108	0.9311
Listeria_monocytogenes_1639_v196	Listeria_monocytogenes_1639_v108	0.9178
Listeria_monocytogenes_1639_v195	Listeria_monocytogenes_1639_v108	0.9375
Listeria_monocytogenes_1639_v194	Listeria_monocytogenes_1639_v108	0.9369
Listeria_monocytogenes_1639_v193	Listeria_monocytogenes_1639_v108	0.9369
Listeria_monocytogenes_1639_v192	Listeria_monocytogenes_1639_v108	0.9023
Listeria_monocytogenes_1639_v191	Listeria_monocytogenes_1639_v108	0.9417
Listeria_monocytogenes_1639_v190	Listeria_monocytogenes_1639_v108	0.9426
Listeria_monocytogenes_1639_v18	Listeria_monocytogenes_1639_v108	0.9735
Listeria_monocytogenes_1639_v189	Listeria_monocytogenes_1639_v108	0.9414
Listeria_monocytogenes_1639_v188	Listeria_monocytogenes_1639_v108	0.9421
Listeria_monocytogenes_1639_v187	Listeria_monocytogenes_1639_v108	0.9425
Listeria_monocytogenes_1639_v186	Listeria_monocytogenes_1639_v108	0.9425
Listeria_monocytogenes_1639_v185	Listeria_monocytogenes_1639_v108	0.9429
Listeria_monocytogenes_1639_v184	Listeria_monocytogenes_1639_v108	0.9418
Listeria_monocytogenes_1639_v183	Listeria_monocytogenes_1639_v108	0.9356
Listeria_monocytogenes_1639_v182	Listeria_monocytogenes_1639_v108	0.9378
Listeria_monocytogenes_1639_v181	Listeria_monocytogenes_1639_v108	0.9238
Listeria_monocytogenes_1639_v180	Listeria_monocytogenes_1639_v108	0.9238
Listeria_monocytogenes_1639_v17	Listeria_monocytogenes_1639_v108	0.9738
Listeria_monocytogenes_1639_v179	Listeria_monocytogenes_1639_v108	0.9321
Listeria_monocytogenes_1639_v178	Listeria_monocytogenes_1639_v108	0.9422
Listeria_monocytogenes_1639_v177	Listeria_monocytogenes_1639_v108	0.9878
Listeria_monocytogenes_1639_v176	Listeria_monocytogenes_1639_v108	0.935
Listeria_monocytogenes_1639_v175	Listeria_monocytogenes_1639_v108	0.9321
Listeria_monocytogenes_1639_v174	Listeria_monocytogenes_1639_v108	0.9426
Listeria_monocytogenes_1639_v173	Listeria_monocytogenes_1639_v108	0.9598
Listeria_monocytogenes_1639_v172	Listeria_monocytogenes_1639_v108	0.9334
Listeria_monocytogenes_1639_v171	Listeria_monocytogenes_1639_v108	0.9284
Listeria_monocytogenes_1639_v170	Listeria_monocytogenes_1639_v108	0.9365
Listeria_monocytogenes_1639_v16	Listeria_monocytogenes_1639_v108	0.9101
Listeria_monocytogenes_1639_v169	Listeria_monocytogenes_1639_v108	0.9381
Listeria_monocytogenes_1639_v168	Listeria_monocytogenes_1639_v108	0.94
Listeria_monocytogenes_1639_v167	Listeria_monocytogenes_1639_v108	0.9312
Listeria_monocytogenes_1639_v166	Listeria_monocytogenes_1639_v108	0.9404
Listeria_monocytogenes_1639_v165	Listeria_monocytogenes_1639_v108	0.9377
Listeria_monocytogenes_1639_v164	Listeria_monocytogenes_1639_v108	0.9638
Listeria_monocytogenes_1639_v163	Listeria_monocytogenes_1639_v108	0.9532
Listeria_monocytogenes_1639_v162	Listeria_monocytogenes_1639_v108	0.9638
Listeria_monocytogenes_1639_v161	Listeria_monocytogenes_1639_v108	0.9642
Listeria_monocytogenes_1639_v160	Listeria_monocytogenes_1639_v108	0.9638
Listeria_monocytogenes_1639_v15	Listeria_monocytogenes_1639_v108	0.9334
Listeria_monocytogenes_1639_v159	Listeria_monocytogenes_1639_v108	0.9878
Listeria_monocytogenes_1639_v158	Listeria_monocytogenes_1639_v108	0.9642
Listeria_monocytogenes_1639_v157	Listeria_monocytogenes_1639_v108	0.9642
Listeria_monocytogenes_1639_v156	Listeria_monocytogenes_1639_v108	0.9297
Listeria_monocytogenes_1639_v155	Listeria_monocytogenes_1639_v108	0.9382
Listeria_monocytogenes_1639_v154	Listeria_monocytogenes_1639_v108	0.936
Listeria_monocytogenes_1639_v153	Listeria_monocytogenes_1639_v108	0.9424
Listeria_monocytogenes_1639_v152	Listeria_monocytogenes_1639_v108	0.9878
Listeria_monocytogenes_1639_v151	Listeria_monocytogenes_1639_v108	0.9878
Listeria_monocytogenes_1639_v150	Listeria_monocytogenes_1639_v108	0.9352
Listeria_monocytogenes_1639_v14	Listeria_monocytogenes_1639_v108	0.9384
Listeria_monocytogenes_1639_v149	Listeria_monocytogenes_1639_v108	0.9353
Listeria_monocytogenes_1639_v148	Listeria_monocytogenes_1639_v108	0.9867
Listeria_monocytogenes_1639_v147	Listeria_monocytogenes_1639_v108	0.9727
Listeria_monocytogenes_1639_v146	Listeria_monocytogenes_1639_v108	0.9385

Listeria_monocytogenes_1639_v145	Listeria_monocytogenes_1639_v108	0.9337
Listeria_monocytogenes_1639_v144	Listeria_monocytogenes_1639_v108	0.9653
Listeria_monocytogenes_1639_v143	Listeria_monocytogenes_1639_v108	0.9405
Listeria_monocytogenes_1639_v142	Listeria_monocytogenes_1639_v108	0.9657
Listeria_monocytogenes_1639_v141	Listeria_monocytogenes_1639_v108	0.9599
Listeria_monocytogenes_1639_v140	Listeria_monocytogenes_1639_v108	0.9665
Listeria_monocytogenes_1639_v13	Listeria_monocytogenes_1639_v108	0.9334
Listeria_monocytogenes_1639_v139	Listeria_monocytogenes_1639_v108	0.965
Listeria_monocytogenes_1639_v138	Listeria_monocytogenes_1639_v108	0.9688
Listeria_monocytogenes_1639_v137	Listeria_monocytogenes_1639_v108	0.9684
Listeria_monocytogenes_1639_v136	Listeria_monocytogenes_1639_v108	0.934
Listeria_monocytogenes_1639_v135	Listeria_monocytogenes_1639_v108	0.9245
Listeria_monocytogenes_1639_v134	Listeria_monocytogenes_1639_v108	0.9583
Listeria_monocytogenes_1639_v133	Listeria_monocytogenes_1639_v108	0.933
Listeria_monocytogenes_1639_v132	Listeria_monocytogenes_1639_v108	0.9249
Listeria_monocytogenes_1639_v131	Listeria_monocytogenes_1639_v108	0.9977
Listeria_monocytogenes_1639_v130	Listeria_monocytogenes_1639_v108	0.9242
Listeria_monocytogenes_1639_v12	Listeria_monocytogenes_1639_v108	0.9094
Listeria_monocytogenes_1639_v129	Listeria_monocytogenes_1639_v108	0.9573
Listeria_monocytogenes_1639_v128	Listeria_monocytogenes_1639_v108	0.9559
Listeria_monocytogenes_1639_v127	Listeria_monocytogenes_1639_v108	0.9408
Listeria_monocytogenes_1639_v126	Listeria_monocytogenes_1639_v108	0.954
Listeria_monocytogenes_1639_v125	Listeria_monocytogenes_1639_v108	0.936
Listeria_monocytogenes_1639_v124	Listeria_monocytogenes_1639_v108	0.93
Listeria_monocytogenes_1639_v123	Listeria_monocytogenes_1639_v108	0.9715
Listeria_monocytogenes_1639_v122	Listeria_monocytogenes_1639_v108	0.9715
Listeria_monocytogenes_1639_v121	Listeria_monocytogenes_1639_v108	0.9715
Listeria_monocytogenes_1639_v120	Listeria_monocytogenes_1639_v108	0.9715
Listeria_monocytogenes_1639_v11	Listeria_monocytogenes_1639_v108	0.9143
Listeria_monocytogenes_1639_v119	Listeria_monocytogenes_1639_v108	0.9715
Listeria_monocytogenes_1639_v118	Listeria_monocytogenes_1639_v108	0.9715
Listeria_monocytogenes_1639_v117	Listeria_monocytogenes_1639_v108	0.9313
Listeria_monocytogenes_1639_v116	Listeria_monocytogenes_1639_v108	0.958
Listeria_monocytogenes_1639_v115	Listeria_monocytogenes_1639_v108	0.9584
Listeria_monocytogenes_1639_v114	Listeria_monocytogenes_1639_v108	0.9287
Listeria_monocytogenes_1639_v113	Listeria_monocytogenes_1639_v108	0.9527
Listeria_monocytogenes_1639_v112	Listeria_monocytogenes_1639_v108	0.9616
Listeria_monocytogenes_1639_v111	Listeria_monocytogenes_1639_v108	0.9439
Listeria_monocytogenes_1639_v110	Listeria_monocytogenes_1639_v108	0.9358
Listeria_monocytogenes_1639_v10	Listeria_monocytogenes_1639_v108	0.9248
Listeria_monocytogenes_1639_v109	Listeria_monocytogenes_1639_v108	0.9291
Listeria_monocytogenes_1639_v108	Listeria_monocytogenes_1639_v108	0.065
Listeria_welshimeri_1643	Listeria_monocytogenes_1639_v109	0.8732
Listeria_welshimeri_serovar_6b_str_SLCC5334_386043	Listeria_monocytogenes_1639_v109	0.8729
Listeria_seeligeri_serovar_1_2b_str_SLCC3954_683837	Listeria_monocytogenes_1639_v109	0.8783
Listeria_seeligeri_FSL_S4-171_702452	Listeria_monocytogenes_1639_v109	0.8223
Listeria_seeligeri_FSL_N1-067_702453	Listeria_monocytogenes_1639_v109	0.8381
Listeria_seeligeri_1640_v1	Listeria_monocytogenes_1639_v109	0.8749
Listeria_seeligeri_1640	Listeria_monocytogenes_1639_v109	0.8736
Listeria_monocytogenes_WSLC1042_1457188	Listeria_monocytogenes_1639_v109	0.9222
Listeria_monocytogenes_WSLC1001_1457187	Listeria_monocytogenes_1639_v109	0.9786
Listeria_monocytogenes_SLCC7179_879090	Listeria_monocytogenes_1639_v109	0.9537
Listeria_monocytogenes_SLCC5850_882096	Listeria_monocytogenes_1639_v109	0.9804
Listeria_monocytogenes_SLCC2755_932919	Listeria_monocytogenes_1639_v109	0.9273
Listeria_monocytogenes_SLCC2540_879089	Listeria_monocytogenes_1639_v109	0.9413
Listeria_monocytogenes_SLCC2479_882020	Listeria_monocytogenes_1639_v109	0.9589
Listeria_monocytogenes_SLCC2378_879088	Listeria_monocytogenes_1639_v109	0.9233
Listeria_monocytogenes_SLCC2376_882097	Listeria_monocytogenes_1639_v109	0.9361
Listeria_monocytogenes_SLCC2372_932920	Listeria_monocytogenes_1639_v109	0.9511
Listeria_monocytogenes_serotype_4b_str_LL195_1230340	Listeria_monocytogenes_1639_v109	0.9293
Listeria_monocytogenes_serotype_4b_str_LIS0087_1134669	Listeria_monocytogenes_1639_v109	0.9604
Listeria_monocytogenes_serotype_4b_str_F2365_265669	Listeria_monocytogenes_1639_v109	0.9293
Listeria_monocytogenes_serotype_4b_str_CLIP_80459_568819	Listeria_monocytogenes_1639_v109	0.936
Listeria_monocytogenes_serotype_4b_str_81-0861_1196161	Listeria_monocytogenes_1639_v109	0.9185
Listeria_monocytogenes_serotype_4b_str_81-0592_1196160	Listeria_monocytogenes_1639_v109	0.9196
Listeria_monocytogenes_serotype_4b_str_81-0558_1196159	Listeria_monocytogenes_1639_v109	0.9199
Listeria_monocytogenes_serotype_4b_str_10-0809_1196162	Listeria_monocytogenes_1639_v109	0.9196
Listeria_monocytogenes_serotype_4b_str_02-6680_1196175	Listeria_monocytogenes_1639_v109	0.9346
Listeria_monocytogenes_serotype_4b_str_02-6679_1196174	Listeria_monocytogenes_1639_v109	0.9346
Listeria_monocytogenes_serotype_4b_str_02-1792_1196173	Listeria_monocytogenes_1639_v109	0.9196
Listeria_monocytogenes_serotype_4b_str_02-1289_1196172	Listeria_monocytogenes_1639_v109	0.9196
Listeria_monocytogenes_serotype_4b_str_02-1103_1196171	Listeria_monocytogenes_1639_v109	0.9196
Listeria_monocytogenes_serotype_3c_str_10-5027_1196195	Listeria_monocytogenes_1639_v109	0.9645
Listeria_monocytogenes_serotype_1_2c_str_10-5026_1196194	Listeria_monocytogenes_1639_v109	0.9653
Listeria_monocytogenes_serotype_1_2c_str_10-5025_1196193	Listeria_monocytogenes_1639_v109	0.9653
Listeria_monocytogenes_serotype_1_2b_str_10-0811_1196164	Listeria_monocytogenes_1639_v109	0.9398
Listeria_monocytogenes_serotype_1_2b_str_10-0810_1196163	Listeria_monocytogenes_1639_v109	0.9398
Listeria_monocytogenes_serotype_1_2a_str_99-6370_1196179	Listeria_monocytogenes_1639_v109	0.955
Listeria_monocytogenes_serotype_1_2a_str_98-2035_1196178	Listeria_monocytogenes_1639_v109	0.955
Listeria_monocytogenes_serotype_1_2a_str_95-0093_1196177	Listeria_monocytogenes_1639_v109	0.9603
Listeria_monocytogenes_serotype_1_2a_str_88-0478_1196176	Listeria_monocytogenes_1639_v109	0.9573
Listeria_monocytogenes_serotype_1_2a_str_10-5024_1196192	Listeria_monocytogenes_1639_v109	0.9603
Listeria_monocytogenes_serotype_1_2a_str_10-4758_1196168	Listeria_monocytogenes_1639_v109	0.9597
Listeria_monocytogenes_serotype_1_2a_str_10-4754_1196167	Listeria_monocytogenes_1639_v109	0.96
Listeria_monocytogenes_serotype_1_2a_str_10-1321_1196191	Listeria_monocytogenes_1639_v109	0.9603
Listeria_monocytogenes_serotype_1_2a_str_10-1047_1196190	Listeria_monocytogenes_1639_v109	0.9581
Listeria_monocytogenes_serotype_1_2a_str_10-1046_1196189	Listeria_monocytogenes_1639_v109	0.9581
Listeria_monocytogenes_serotype_1_2a_str_10-0934_1196170	Listeria_monocytogenes_1639_v109	0.9664
Listeria_monocytogenes_serotype_1_2a_str_10-0933_1196169	Listeria_monocytogenes_1639_v109	0.9664
Listeria_monocytogenes_serotype_1_2a_str_10-0815_1196188	Listeria_monocytogenes_1639_v109	0.9557
Listeria_monocytogenes_serotype_1_2a_str_10-0814_1196187	Listeria_monocytogenes_1639_v109	0.9603

Listeria_monocytogenes_serotype_1_2a_str_10-0813_1196166	Listeria_monocytogenes_1639_v109	0.9794
Listeria_monocytogenes_serotype_1_2a_str_10-0812_1196165	Listeria_monocytogenes_1639_v109	0.9794
Listeria_monocytogenes_serotype_1_2a_str_08-7669_1196186	Listeria_monocytogenes_1639_v109	0.9535
Listeria_monocytogenes_serotype_1_2a_str_08-7374_1196185	Listeria_monocytogenes_1639_v109	0.9603
Listeria_monocytogenes_serotype_1_2a_str_08-6997_1196184	Listeria_monocytogenes_1639_v109	0.9557
Listeria_monocytogenes_serotype_1_2a_str_08-6569_1196183	Listeria_monocytogenes_1639_v109	0.9557
Listeria_monocytogenes_serotype_1_2a_str_08-6056_1196182	Listeria_monocytogenes_1639_v109	0.9557
Listeria_monocytogenes_serotype_1_2a_str_04-5457_1196181	Listeria_monocytogenes_1639_v109	0.9603
Listeria_monocytogenes_serotype_1_2a_str_02-5993_1196180	Listeria_monocytogenes_1639_v109	0.9603
Listeria_monocytogenes_R479a_1437838	Listeria_monocytogenes_1639_v109	0.9574
Listeria_monocytogenes_N53-1_1234142	Listeria_monocytogenes_1639_v109	0.9035
Listeria_monocytogenes_M7_1030009	Listeria_monocytogenes_1639_v109	0.9111
Listeria_monocytogenes_LO28_393132	Listeria_monocytogenes_1639_v109	0.9541
Listeria_monocytogenes_L99_563174	Listeria_monocytogenes_1639_v109	0.9114
Listeria_monocytogenes_L312_882094	Listeria_monocytogenes_1639_v109	0.936
Listeria_monocytogenes_J1816_930781	Listeria_monocytogenes_1639_v109	0.9285
Listeria_monocytogenes_J1-220_930782	Listeria_monocytogenes_1639_v109	0.9254
Listeria_monocytogenes_J0161_393130	Listeria_monocytogenes_1639_v109	0.95
Listeria_monocytogenes_HCC23_552536	Listeria_monocytogenes_1639_v109	0.9107
Listeria_monocytogenes_FSL_R2-561_393126	Listeria_monocytogenes_1639_v109	0.9612
Listeria_monocytogenes_FSL_J1-208_393119	Listeria_monocytogenes_1639_v109	0.9014
Listeria_monocytogenes_FSL_J1-175_393118	Listeria_monocytogenes_1639_v109	0.9456
Listeria_monocytogenes_Finland_1998_393127	Listeria_monocytogenes_1639_v109	0.9537
Listeria_monocytogenes_EGD-e_169963_v1	Listeria_monocytogenes_1639_v109	0.9522
Listeria_monocytogenes_EGD-e_169963	Listeria_monocytogenes_1639_v109	0.9522
Listeria_monocytogenes_EGD_1334565	Listeria_monocytogenes_1639_v109	0.9811
Listeria_monocytogenes_CFSAN002349_1299895	Listeria_monocytogenes_1639_v109	0.9347
Listeria_monocytogenes_ATCC_19117_882095_v1	Listeria_monocytogenes_1639_v109	0.9299
Listeria_monocytogenes_ATCC_19117_882095	Listeria_monocytogenes_1639_v109	0.9295
Listeria_monocytogenes_6179_1288295	Listeria_monocytogenes_1639_v109	0.9511
Listeria_monocytogenes_1639_v9	Listeria_monocytogenes_1639_v109	0.9322
Listeria_monocytogenes_1639_v99	Listeria_monocytogenes_1639_v109	0.9285
Listeria_monocytogenes_1639_v98	Listeria_monocytogenes_1639_v109	0.9492
Listeria_monocytogenes_1639_v97	Listeria_monocytogenes_1639_v109	0.935
Listeria_monocytogenes_1639_v96	Listeria_monocytogenes_1639_v109	0.9282
Listeria_monocytogenes_1639_v95	Listeria_monocytogenes_1639_v109	0.9282
Listeria_monocytogenes_1639_v94	Listeria_monocytogenes_1639_v109	0.9289
Listeria_monocytogenes_1639_v93	Listeria_monocytogenes_1639_v109	0.9473
Listeria_monocytogenes_1639_v92	Listeria_monocytogenes_1639_v109	0.9293
Listeria_monocytogenes_1639_v91	Listeria_monocytogenes_1639_v109	0.925
Listeria_monocytogenes_1639_v90	Listeria_monocytogenes_1639_v109	0.9492
Listeria_monocytogenes_1639_v8	Listeria_monocytogenes_1639_v109	0.9472
Listeria_monocytogenes_1639_v89	Listeria_monocytogenes_1639_v109	0.9321
Listeria_monocytogenes_1639_v88	Listeria_monocytogenes_1639_v109	0.9708
Listeria_monocytogenes_1639_v87	Listeria_monocytogenes_1639_v109	0.9754
Listeria_monocytogenes_1639_v86	Listeria_monocytogenes_1639_v109	0.975
Listeria_monocytogenes_1639_v85	Listeria_monocytogenes_1639_v109	0.9186
Listeria_monocytogenes_1639_v84	Listeria_monocytogenes_1639_v109	0.9681
Listeria_monocytogenes_1639_v83	Listeria_monocytogenes_1639_v109	0.9243
Listeria_monocytogenes_1639_v82	Listeria_monocytogenes_1639_v109	0.924
Listeria_monocytogenes_1639_v81	Listeria_monocytogenes_1639_v109	0.9629
Listeria_monocytogenes_1639_v80	Listeria_monocytogenes_1639_v109	0.9392
Listeria_monocytogenes_1639_v7	Listeria_monocytogenes_1639_v109	0.9472
Listeria_monocytogenes_1639_v79	Listeria_monocytogenes_1639_v109	0.9581
Listeria_monocytogenes_1639_v78	Listeria_monocytogenes_1639_v109	0.9626
Listeria_monocytogenes_1639_v77	Listeria_monocytogenes_1639_v109	0.9534
Listeria_monocytogenes_1639_v76	Listeria_monocytogenes_1639_v109	0.9536
Listeria_monocytogenes_1639_v75	Listeria_monocytogenes_1639_v109	0.9114
Listeria_monocytogenes_1639_v74	Listeria_monocytogenes_1639_v109	0.9594
Listeria_monocytogenes_1639_v73	Listeria_monocytogenes_1639_v109	0.9514
Listeria_monocytogenes_1639_v72	Listeria_monocytogenes_1639_v109	0.9224
Listeria_monocytogenes_1639_v71	Listeria_monocytogenes_1639_v109	0.9482
Listeria_monocytogenes_1639_v70	Listeria_monocytogenes_1639_v109	0.9602
Listeria_monocytogenes_1639_v6	Listeria_monocytogenes_1639_v109	0.9469
Listeria_monocytogenes_1639_v69	Listeria_monocytogenes_1639_v109	0.9196
Listeria_monocytogenes_1639_v68	Listeria_monocytogenes_1639_v109	0.9434
Listeria_monocytogenes_1639_v67	Listeria_monocytogenes_1639_v109	0.9472
Listeria_monocytogenes_1639_v66	Listeria_monocytogenes_1639_v109	0.9578
Listeria_monocytogenes_1639_v65	Listeria_monocytogenes_1639_v109	0.9561
Listeria_monocytogenes_1639_v64	Listeria_monocytogenes_1639_v109	0.9544
Listeria_monocytogenes_1639_v63	Listeria_monocytogenes_1639_v109	0.9442
Listeria_monocytogenes_1639_v62	Listeria_monocytogenes_1639_v109	0.9605
Listeria_monocytogenes_1639_v61	Listeria_monocytogenes_1639_v109	0.9606
Listeria_monocytogenes_1639_v60	Listeria_monocytogenes_1639_v109	0.9521
Listeria_monocytogenes_1639_v5	Listeria_monocytogenes_1639_v109	0.8937
Listeria_monocytogenes_1639_v59	Listeria_monocytogenes_1639_v109	0.9288
Listeria_monocytogenes_1639_v58	Listeria_monocytogenes_1639_v109	0.9254
Listeria_monocytogenes_1639_v57	Listeria_monocytogenes_1639_v109	0.9259
Listeria_monocytogenes_1639_v56	Listeria_monocytogenes_1639_v109	0.9318
Listeria_monocytogenes_1639_v55	Listeria_monocytogenes_1639_v109	0.9221
Listeria_monocytogenes_1639_v54	Listeria_monocytogenes_1639_v109	0.9257
Listeria_monocytogenes_1639_v53	Listeria_monocytogenes_1639_v109	0.9404
Listeria_monocytogenes_1639_v52	Listeria_monocytogenes_1639_v109	0.9309
Listeria_monocytogenes_1639_v51	Listeria_monocytogenes_1639_v109	0.9218
Listeria_monocytogenes_1639_v50	Listeria_monocytogenes_1639_v109	0.9588
Listeria_monocytogenes_1639_v4	Listeria_monocytogenes_1639_v109	0.9066
Listeria_monocytogenes_1639_v49	Listeria_monocytogenes_1639_v109	0.9174
Listeria_monocytogenes_1639_v48	Listeria_monocytogenes_1639_v109	0.9592
Listeria_monocytogenes_1639_v47	Listeria_monocytogenes_1639_v109	0.957
Listeria_monocytogenes_1639_v46	Listeria_monocytogenes_1639_v109	0.9499
Listeria_monocytogenes_1639_v45	Listeria_monocytogenes_1639_v109	0.9504

Listeria_monocytogenes_1639_v44	Listeria_monocytogenes_1639_v109	0.9603
Listeria_monocytogenes_1639_v43	Listeria_monocytogenes_1639_v109	0.9603
Listeria_monocytogenes_1639_v42	Listeria_monocytogenes_1639_v109	0.9425
Listeria_monocytogenes_1639_v41	Listeria_monocytogenes_1639_v109	0.9366
Listeria_monocytogenes_1639_v40	Listeria_monocytogenes_1639_v109	0.9526
Listeria_monocytogenes_1639_v3	Listeria_monocytogenes_1639_v109	0.907
Listeria_monocytogenes_1639_v39	Listeria_monocytogenes_1639_v109	0.9441
Listeria_monocytogenes_1639_v38	Listeria_monocytogenes_1639_v109	0.9457
Listeria_monocytogenes_1639_v37	Listeria_monocytogenes_1639_v109	0.9434
Listeria_monocytogenes_1639_v36	Listeria_monocytogenes_1639_v109	0.9446
Listeria_monocytogenes_1639_v35	Listeria_monocytogenes_1639_v109	0.9507
Listeria_monocytogenes_1639_v34	Listeria_monocytogenes_1639_v109	0.954
Listeria_monocytogenes_1639_v33	Listeria_monocytogenes_1639_v109	0.9398
Listeria_monocytogenes_1639_v32	Listeria_monocytogenes_1639_v109	0.9483
Listeria_monocytogenes_1639_v31	Listeria_monocytogenes_1639_v109	0.9543
Listeria_monocytogenes_1639_v30	Listeria_monocytogenes_1639_v109	0.924
Listeria_monocytogenes_1639_v2	Listeria_monocytogenes_1639_v109	0.9037
Listeria_monocytogenes_1639_v29	Listeria_monocytogenes_1639_v109	0.9489
Listeria_monocytogenes_1639_v28	Listeria_monocytogenes_1639_v109	0.9549
Listeria_monocytogenes_1639_v27	Listeria_monocytogenes_1639_v109	0.9389
Listeria_monocytogenes_1639_v26	Listeria_monocytogenes_1639_v109	0.9382
Listeria_monocytogenes_1639_v25	Listeria_monocytogenes_1639_v109	0.9382
Listeria_monocytogenes_1639_v24	Listeria_monocytogenes_1639_v109	0.9246
Listeria_monocytogenes_1639_v23	Listeria_monocytogenes_1639_v109	0.9243
Listeria_monocytogenes_1639_v22	Listeria_monocytogenes_1639_v109	0.9456
Listeria_monocytogenes_1639_v21	Listeria_monocytogenes_1639_v109	0.925
Listeria_monocytogenes_1639_v20	Listeria_monocytogenes_1639_v109	0.9464
Listeria_monocytogenes_1639_v1	Listeria_monocytogenes_1639_v109	0.9468
Listeria_monocytogenes_1639_v19	Listeria_monocytogenes_1639_v109	0.946
Listeria_monocytogenes_1639_v198	Listeria_monocytogenes_1639_v109	0.9048
Listeria_monocytogenes_1639_v197	Listeria_monocytogenes_1639_v109	0.9386
Listeria_monocytogenes_1639_v196	Listeria_monocytogenes_1639_v109	0.9647
Listeria_monocytogenes_1639_v195	Listeria_monocytogenes_1639_v109	0.9384
Listeria_monocytogenes_1639_v194	Listeria_monocytogenes_1639_v109	0.9362
Listeria_monocytogenes_1639_v193	Listeria_monocytogenes_1639_v109	0.9365
Listeria_monocytogenes_1639_v192	Listeria_monocytogenes_1639_v109	0.896
Listeria_monocytogenes_1639_v191	Listeria_monocytogenes_1639_v109	0.9613
Listeria_monocytogenes_1639_v190	Listeria_monocytogenes_1639_v109	0.9778
Listeria_monocytogenes_1639_v18	Listeria_monocytogenes_1639_v109	0.9215
Listeria_monocytogenes_1639_v189	Listeria_monocytogenes_1639_v109	0.977
Listeria_monocytogenes_1639_v188	Listeria_monocytogenes_1639_v109	0.9571
Listeria_monocytogenes_1639_v187	Listeria_monocytogenes_1639_v109	0.9778
Listeria_monocytogenes_1639_v186	Listeria_monocytogenes_1639_v109	0.9782
Listeria_monocytogenes_1639_v185	Listeria_monocytogenes_1639_v109	0.9782
Listeria_monocytogenes_1639_v184	Listeria_monocytogenes_1639_v109	0.9774
Listeria_monocytogenes_1639_v183	Listeria_monocytogenes_1639_v109	0.9541
Listeria_monocytogenes_1639_v182	Listeria_monocytogenes_1639_v109	0.9563
Listeria_monocytogenes_1639_v181	Listeria_monocytogenes_1639_v109	0.9121
Listeria_monocytogenes_1639_v180	Listeria_monocytogenes_1639_v109	0.9121
Listeria_monocytogenes_1639_v17	Listeria_monocytogenes_1639_v109	0.9219
Listeria_monocytogenes_1639_v179	Listeria_monocytogenes_1639_v109	0.9587
Listeria_monocytogenes_1639_v178	Listeria_monocytogenes_1639_v109	0.9591
Listeria_monocytogenes_1639_v177	Listeria_monocytogenes_1639_v109	0.9343
Listeria_monocytogenes_1639_v176	Listeria_monocytogenes_1639_v109	0.927
Listeria_monocytogenes_1639_v175	Listeria_monocytogenes_1639_v109	0.9651
Listeria_monocytogenes_1639_v174	Listeria_monocytogenes_1639_v109	0.9629
Listeria_monocytogenes_1639_v173	Listeria_monocytogenes_1639_v109	0.9434
Listeria_monocytogenes_1639_v172	Listeria_monocytogenes_1639_v109	0.9281
Listeria_monocytogenes_1639_v171	Listeria_monocytogenes_1639_v109	0.9661
Listeria_monocytogenes_1639_v170	Listeria_monocytogenes_1639_v109	0.9745
Listeria_monocytogenes_1639_v16	Listeria_monocytogenes_1639_v109	0.9459
Listeria_monocytogenes_1639_v169	Listeria_monocytogenes_1639_v109	0.9666
Listeria_monocytogenes_1639_v168	Listeria_monocytogenes_1639_v109	0.9534
Listeria_monocytogenes_1639_v167	Listeria_monocytogenes_1639_v109	0.9593
Listeria_monocytogenes_1639_v166	Listeria_monocytogenes_1639_v109	0.9538
Listeria_monocytogenes_1639_v165	Listeria_monocytogenes_1639_v109	0.9662
Listeria_monocytogenes_1639_v164	Listeria_monocytogenes_1639_v109	0.9288
Listeria_monocytogenes_1639_v163	Listeria_monocytogenes_1639_v109	0.9384
Listeria_monocytogenes_1639_v162	Listeria_monocytogenes_1639_v109	0.9288
Listeria_monocytogenes_1639_v161	Listeria_monocytogenes_1639_v109	0.9299
Listeria_monocytogenes_1639_v160	Listeria_monocytogenes_1639_v109	0.9296
Listeria_monocytogenes_1639_v15	Listeria_monocytogenes_1639_v109	0.9243
Listeria_monocytogenes_1639_v159	Listeria_monocytogenes_1639_v109	0.9343
Listeria_monocytogenes_1639_v158	Listeria_monocytogenes_1639_v109	0.9293
Listeria_monocytogenes_1639_v157	Listeria_monocytogenes_1639_v109	0.9293
Listeria_monocytogenes_1639_v156	Listeria_monocytogenes_1639_v109	0.96
Listeria_monocytogenes_1639_v155	Listeria_monocytogenes_1639_v109	0.9614
Listeria_monocytogenes_1639_v154	Listeria_monocytogenes_1639_v109	0.9667
Listeria_monocytogenes_1639_v153	Listeria_monocytogenes_1639_v109	0.9523
Listeria_monocytogenes_1639_v152	Listeria_monocytogenes_1639_v109	0.9343
Listeria_monocytogenes_1639_v151	Listeria_monocytogenes_1639_v109	0.9343
Listeria_monocytogenes_1639_v150	Listeria_monocytogenes_1639_v109	0.9811
Listeria_monocytogenes_1639_v14	Listeria_monocytogenes_1639_v109	0.9193
Listeria_monocytogenes_1639_v149	Listeria_monocytogenes_1639_v109	0.9815
Listeria_monocytogenes_1639_v148	Listeria_monocytogenes_1639_v109	0.9336
Listeria_monocytogenes_1639_v147	Listeria_monocytogenes_1639_v109	0.9196
Listeria_monocytogenes_1639_v146	Listeria_monocytogenes_1639_v109	0.9554
Listeria_monocytogenes_1639_v145	Listeria_monocytogenes_1639_v109	0.952
Listeria_monocytogenes_1639_v144	Listeria_monocytogenes_1639_v109	0.9353
Listeria_monocytogenes_1639_v143	Listeria_monocytogenes_1639_v109	0.9543
Listeria_monocytogenes_1639_v142	Listeria_monocytogenes_1639_v109	0.9357

Listeria_monocytogenes_1639_v141	Listeria_monocytogenes_1639_v109	0.9432
Listeria_monocytogenes_1639_v140	Listeria_monocytogenes_1639_v109	0.9364
Listeria_monocytogenes_1639_v13	Listeria_monocytogenes_1639_v109	0.9243
Listeria_monocytogenes_1639_v139	Listeria_monocytogenes_1639_v109	0.9342
Listeria_monocytogenes_1639_v138	Listeria_monocytogenes_1639_v109	0.9387
Listeria_monocytogenes_1639_v137	Listeria_monocytogenes_1639_v109	0.9383
Listeria_monocytogenes_1639_v136	Listeria_monocytogenes_1639_v109	0.9589
Listeria_monocytogenes_1639_v135	Listeria_monocytogenes_1639_v109	0.9084
Listeria_monocytogenes_1639_v134	Listeria_monocytogenes_1639_v109	0.9231
Listeria_monocytogenes_1639_v133	Listeria_monocytogenes_1639_v109	0.9603
Listeria_monocytogenes_1639_v132	Listeria_monocytogenes_1639_v109	0.9085
Listeria_monocytogenes_1639_v131	Listeria_monocytogenes_1639_v109	0.9289
Listeria_monocytogenes_1639_v130	Listeria_monocytogenes_1639_v109	0.9492
Listeria_monocytogenes_1639_v12	Listeria_monocytogenes_1639_v109	0.9416
Listeria_monocytogenes_1639_v129	Listeria_monocytogenes_1639_v109	0.9391
Listeria_monocytogenes_1639_v128	Listeria_monocytogenes_1639_v109	0.9396
Listeria_monocytogenes_1639_v127	Listeria_monocytogenes_1639_v109	0.9529
Listeria_monocytogenes_1639_v126	Listeria_monocytogenes_1639_v109	0.9404
Listeria_monocytogenes_1639_v125	Listeria_monocytogenes_1639_v109	0.9573
Listeria_monocytogenes_1639_v124	Listeria_monocytogenes_1639_v109	0.9206
Listeria_monocytogenes_1639_v123	Listeria_monocytogenes_1639_v109	0.9347
Listeria_monocytogenes_1639_v122	Listeria_monocytogenes_1639_v109	0.9343
Listeria_monocytogenes_1639_v121	Listeria_monocytogenes_1639_v109	0.9343
Listeria_monocytogenes_1639_v120	Listeria_monocytogenes_1639_v109	0.9343
Listeria_monocytogenes_1639_v11	Listeria_monocytogenes_1639_v109	0.9481
Listeria_monocytogenes_1639_v119	Listeria_monocytogenes_1639_v109	0.9343
Listeria_monocytogenes_1639_v118	Listeria_monocytogenes_1639_v109	0.9343
Listeria_monocytogenes_1639_v117	Listeria_monocytogenes_1639_v109	0.96
Listeria_monocytogenes_1639_v116	Listeria_monocytogenes_1639_v109	0.9237
Listeria_monocytogenes_1639_v115	Listeria_monocytogenes_1639_v109	0.9233
Listeria_monocytogenes_1639_v114	Listeria_monocytogenes_1639_v109	0.9835
Listeria_monocytogenes_1639_v113	Listeria_monocytogenes_1639_v109	0.9474
Listeria_monocytogenes_1639_v112	Listeria_monocytogenes_1639_v109	0.9319
Listeria_monocytogenes_1639_v111	Listeria_monocytogenes_1639_v109	0.935
Listeria_monocytogenes_1639_v110	Listeria_monocytogenes_1639_v109	0.9764
Listeria_monocytogenes_1639_v10	Listeria_monocytogenes_1639_v109	0.9274
Listeria_monocytogenes_1639_v109	Listeria_monocytogenes_1639_v109	0.0694
Listeria_welshimeri_1643	Listeria_monocytogenes_1639_v10	0.8534
Listeria_welshimeri_serovar_6b_str_SLCC5334_386043	Listeria_monocytogenes_1639_v10	0.8531
Listeria_seeligeri_serovar_1_2b_str_SLCC3954_683837	Listeria_monocytogenes_1639_v10	0.8589
Listeria_seeligeri_FSL_S4-171_702452	Listeria_monocytogenes_1639_v10	0.8024
Listeria_seeligeri_FSL_N1-067_702453	Listeria_monocytogenes_1639_v10	0.8185
Listeria_seeligeri_1640_v1	Listeria_monocytogenes_1639_v10	0.8599
Listeria_seeligeri_1640	Listeria_monocytogenes_1639_v10	0.8705
Listeria_monocytogenes_WSLC1042_1457188	Listeria_monocytogenes_1639_v10	0.9214
Listeria_monocytogenes_WSLC1001_1457187	Listeria_monocytogenes_1639_v10	0.9236
Listeria_monocytogenes_SLCC7179_879090	Listeria_monocytogenes_1639_v10	0.9158
Listeria_monocytogenes_SLCC5850_882096	Listeria_monocytogenes_1639_v10	0.9232
Listeria_monocytogenes_SLCC2755_932919	Listeria_monocytogenes_1639_v10	0.9301
Listeria_monocytogenes_SLCC2540_879089	Listeria_monocytogenes_1639_v10	0.9397
Listeria_monocytogenes_SLCC2479_882020	Listeria_monocytogenes_1639_v10	0.9263
Listeria_monocytogenes_SLCC2378_879088	Listeria_monocytogenes_1639_v10	0.9221
Listeria_monocytogenes_SLCC2376_882097	Listeria_monocytogenes_1639_v10	0.9105
Listeria_monocytogenes_SLCC2372_932920	Listeria_monocytogenes_1639_v10	0.9199
Listeria_monocytogenes_serotype_4b_str_LL195_1230340	Listeria_monocytogenes_1639_v10	0.9275
Listeria_monocytogenes_serotype_4b_str_LIS0087_1134669	Listeria_monocytogenes_1639_v10	0.9047
Listeria_monocytogenes_serotype_4b_str_F2365_265669	Listeria_monocytogenes_1639_v10	0.9275
Listeria_monocytogenes_serotype_4b_str_CLIP_80459_568819	Listeria_monocytogenes_1639_v10	0.9293
Listeria_monocytogenes_serotype_4b_str_81-0861_1196161	Listeria_monocytogenes_1639_v10	0.9163
Listeria_monocytogenes_serotype_4b_str_81-0592_1196160	Listeria_monocytogenes_1639_v10	0.9173
Listeria_monocytogenes_serotype_4b_str_81-0558_1196159	Listeria_monocytogenes_1639_v10	0.9177
Listeria_monocytogenes_serotype_4b_str_10-0809_1196162	Listeria_monocytogenes_1639_v10	0.9173
Listeria_monocytogenes_serotype_4b_str_02-6680_1196175	Listeria_monocytogenes_1639_v10	0.9253
Listeria_monocytogenes_serotype_4b_str_02-6679_1196174	Listeria_monocytogenes_1639_v10	0.9253
Listeria_monocytogenes_serotype_4b_str_02-1792_1196173	Listeria_monocytogenes_1639_v10	0.9188
Listeria_monocytogenes_serotype_4b_str_02-1289_1196172	Listeria_monocytogenes_1639_v10	0.9188
Listeria_monocytogenes_serotype_4b_str_02-1103_1196171	Listeria_monocytogenes_1639_v10	0.9188
Listeria_monocytogenes_serotype_3c_str_10-5027_1196195	Listeria_monocytogenes_1639_v10	0.9295
Listeria_monocytogenes_serotype_1_2c_str_10-5026_1196194	Listeria_monocytogenes_1639_v10	0.9299
Listeria_monocytogenes_serotype_1_2c_str_10-5025_1196193	Listeria_monocytogenes_1639_v10	0.9299
Listeria_monocytogenes_serotype_1_2b_str_10-0811_1196164	Listeria_monocytogenes_1639_v10	0.9509
Listeria_monocytogenes_serotype_1_2b_str_10-0810_1196163	Listeria_monocytogenes_1639_v10	0.9509
Listeria_monocytogenes_serotype_1_2a_str_99-6370_1196179	Listeria_monocytogenes_1639_v10	0.9182
Listeria_monocytogenes_serotype_1_2a_str_98-2035_1196178	Listeria_monocytogenes_1639_v10	0.9182
Listeria_monocytogenes_serotype_1_2a_str_95-0093_1196177	Listeria_monocytogenes_1639_v10	0.9217
Listeria_monocytogenes_serotype_1_2a_str_88-0478_1196176	Listeria_monocytogenes_1639_v10	0.9205
Listeria_monocytogenes_serotype_1_2a_str_10-5024_1196192	Listeria_monocytogenes_1639_v10	0.922
Listeria_monocytogenes_serotype_1_2a_str_10-4758_1196168	Listeria_monocytogenes_1639_v10	0.9255
Listeria_monocytogenes_serotype_1_2a_str_10-4754_1196167	Listeria_monocytogenes_1639_v10	0.9259
Listeria_monocytogenes_serotype_1_2a_str_10-1321_1196191	Listeria_monocytogenes_1639_v10	0.922
Listeria_monocytogenes_serotype_1_2a_str_10-1047_1196190	Listeria_monocytogenes_1639_v10	0.9227
Listeria_monocytogenes_serotype_1_2a_str_10-1046_1196189	Listeria_monocytogenes_1639_v10	0.9227
Listeria_monocytogenes_serotype_1_2a_str_10-0934_1196170	Listeria_monocytogenes_1639_v10	0.9197
Listeria_monocytogenes_serotype_1_2a_str_10-0933_1196169	Listeria_monocytogenes_1639_v10	0.9197
Listeria_monocytogenes_serotype_1_2a_str_10-0815_1196188	Listeria_monocytogenes_1639_v10	0.9188
Listeria_monocytogenes_serotype_1_2a_str_10-0814_1196187	Listeria_monocytogenes_1639_v10	0.9224
Listeria_monocytogenes_serotype_1_2a_str_10-0813_1196166	Listeria_monocytogenes_1639_v10	0.9219
Listeria_monocytogenes_serotype_1_2a_str_10-0812_1196165	Listeria_monocytogenes_1639_v10	0.9219
Listeria_monocytogenes_serotype_1_2a_str_08-7669_1196186	Listeria_monocytogenes_1639_v10	0.9157
Listeria_monocytogenes_serotype_1_2a_str_08-7374_1196185	Listeria_monocytogenes_1639_v10	0.922
Listeria_monocytogenes_serotype_1_2a_str_08-6997_1196184	Listeria_monocytogenes_1639_v10	0.9188

Listeria_monocytogenes_serotype_1_2a_str_08-6569_1196183	Listeria_monocytogenes_1639_v10	0.9188
Listeria_monocytogenes_serotype_1_2a_str_08-6056_1196182	Listeria_monocytogenes_1639_v10	0.9188
Listeria_monocytogenes_serotype_1_2a_str_04-5457_1196181	Listeria_monocytogenes_1639_v10	0.922
Listeria_monocytogenes_serotype_1_2a_str_02-5993_1196180	Listeria_monocytogenes_1639_v10	0.922
Listeria_monocytogenes_R479a_1437838	Listeria_monocytogenes_1639_v10	0.9203
Listeria_monocytogenes_N53-1_1234142	Listeria_monocytogenes_1639_v10	0.8703
Listeria_monocytogenes_M7_1030009	Listeria_monocytogenes_1639_v10	0.895
Listeria_monocytogenes_LO28_393132	Listeria_monocytogenes_1639_v10	0.9209
Listeria_monocytogenes_L99_563174	Listeria_monocytogenes_1639_v10	0.8953
Listeria_monocytogenes_L312_882094	Listeria_monocytogenes_1639_v10	0.9297
Listeria_monocytogenes_J1816_930781	Listeria_monocytogenes_1639_v10	0.9213
Listeria_monocytogenes_J1-220_930782	Listeria_monocytogenes_1639_v10	0.9194
Listeria_monocytogenes_J0161_393130	Listeria_monocytogenes_1639_v10	0.9158
Listeria_monocytogenes_HCC23_552536	Listeria_monocytogenes_1639_v10	0.895
Listeria_monocytogenes_FSL_R2-561_393126	Listeria_monocytogenes_1639_v10	0.9263
Listeria_monocytogenes_FSL_J1-208_393119	Listeria_monocytogenes_1639_v10	0.8799
Listeria_monocytogenes_FSL_J1-175_393118	Listeria_monocytogenes_1639_v10	0.9409
Listeria_monocytogenes_Finland_1998_393127	Listeria_monocytogenes_1639_v10	0.9204
Listeria_monocytogenes_EGD-e_169963_v1	Listeria_monocytogenes_1639_v10	0.9168
Listeria_monocytogenes_EGD-e_169963	Listeria_monocytogenes_1639_v10	0.9168
Listeria_monocytogenes_EGD_1334565	Listeria_monocytogenes_1639_v10	0.9239
Listeria_monocytogenes_CFSAN002349_1299895	Listeria_monocytogenes_1639_v10	0.9299
Listeria_monocytogenes_ATCC_19117_882095_v1	Listeria_monocytogenes_1639_v10	0.9237
Listeria_monocytogenes_ATCC_19117_882095	Listeria_monocytogenes_1639_v10	0.9233
Listeria_monocytogenes_6179_1288295	Listeria_monocytogenes_1639_v10	0.9108
Listeria_monocytogenes_1639_v9	Listeria_monocytogenes_1639_v10	0.9491
Listeria_monocytogenes_1639_v99	Listeria_monocytogenes_1639_v10	0.9224
Listeria_monocytogenes_1639_v98	Listeria_monocytogenes_1639_v10	0.9172
Listeria_monocytogenes_1639_v97	Listeria_monocytogenes_1639_v10	0.9255
Listeria_monocytogenes_1639_v96	Listeria_monocytogenes_1639_v10	0.9214
Listeria_monocytogenes_1639_v95	Listeria_monocytogenes_1639_v10	0.9214
Listeria_monocytogenes_1639_v94	Listeria_monocytogenes_1639_v10	0.9217
Listeria_monocytogenes_1639_v93	Listeria_monocytogenes_1639_v10	0.9136
Listeria_monocytogenes_1639_v92	Listeria_monocytogenes_1639_v10	0.9225
Listeria_monocytogenes_1639_v91	Listeria_monocytogenes_1639_v10	0.9241
Listeria_monocytogenes_1639_v90	Listeria_monocytogenes_1639_v10	0.9172
Listeria_monocytogenes_1639_v8	Listeria_monocytogenes_1639_v10	0.9237
Listeria_monocytogenes_1639_v89	Listeria_monocytogenes_1639_v10	0.927
Listeria_monocytogenes_1639_v88	Listeria_monocytogenes_1639_v10	0.925
Listeria_monocytogenes_1639_v87	Listeria_monocytogenes_1639_v10	0.9183
Listeria_monocytogenes_1639_v86	Listeria_monocytogenes_1639_v10	0.9176
Listeria_monocytogenes_1639_v85	Listeria_monocytogenes_1639_v10	0.9171
Listeria_monocytogenes_1639_v84	Listeria_monocytogenes_1639_v10	0.9255
Listeria_monocytogenes_1639_v83	Listeria_monocytogenes_1639_v10	0.9187
Listeria_monocytogenes_1639_v82	Listeria_monocytogenes_1639_v10	0.9184
Listeria_monocytogenes_1639_v81	Listeria_monocytogenes_1639_v10	0.9257
Listeria_monocytogenes_1639_v80	Listeria_monocytogenes_1639_v10	0.9571
Listeria_monocytogenes_1639_v7	Listeria_monocytogenes_1639_v10	0.9237
Listeria_monocytogenes_1639_v79	Listeria_monocytogenes_1639_v10	0.9244
Listeria_monocytogenes_1639_v78	Listeria_monocytogenes_1639_v10	0.9254
Listeria_monocytogenes_1639_v77	Listeria_monocytogenes_1639_v10	0.9122
Listeria_monocytogenes_1639_v76	Listeria_monocytogenes_1639_v10	0.9219
Listeria_monocytogenes_1639_v75	Listeria_monocytogenes_1639_v10	0.8953
Listeria_monocytogenes_1639_v74	Listeria_monocytogenes_1639_v10	0.9019
Listeria_monocytogenes_1639_v73	Listeria_monocytogenes_1639_v10	0.9169
Listeria_monocytogenes_1639_v72	Listeria_monocytogenes_1639_v10	0.9168
Listeria_monocytogenes_1639_v71	Listeria_monocytogenes_1639_v10	0.9139
Listeria_monocytogenes_1639_v70	Listeria_monocytogenes_1639_v10	0.9265
Listeria_monocytogenes_1639_v6	Listeria_monocytogenes_1639_v10	0.9233
Listeria_monocytogenes_1639_v69	Listeria_monocytogenes_1639_v10	0.9173
Listeria_monocytogenes_1639_v68	Listeria_monocytogenes_1639_v10	0.9088
Listeria_monocytogenes_1639_v67	Listeria_monocytogenes_1639_v10	0.94
Listeria_monocytogenes_1639_v66	Listeria_monocytogenes_1639_v10	0.9227
Listeria_monocytogenes_1639_v65	Listeria_monocytogenes_1639_v10	0.9192
Listeria_monocytogenes_1639_v64	Listeria_monocytogenes_1639_v10	0.9174
Listeria_monocytogenes_1639_v63	Listeria_monocytogenes_1639_v10	0.9563
Listeria_monocytogenes_1639_v62	Listeria_monocytogenes_1639_v10	0.9227
Listeria_monocytogenes_1639_v61	Listeria_monocytogenes_1639_v10	0.9236
Listeria_monocytogenes_1639_v60	Listeria_monocytogenes_1639_v10	0.918
Listeria_monocytogenes_1639_v5	Listeria_monocytogenes_1639_v10	0.9415
Listeria_monocytogenes_1639_v59	Listeria_monocytogenes_1639_v10	0.9245
Listeria_monocytogenes_1639_v58	Listeria_monocytogenes_1639_v10	0.923
Listeria_monocytogenes_1639_v57	Listeria_monocytogenes_1639_v10	0.9398
Listeria_monocytogenes_1639_v56	Listeria_monocytogenes_1639_v10	0.923
Listeria_monocytogenes_1639_v55	Listeria_monocytogenes_1639_v10	0.9173
Listeria_monocytogenes_1639_v54	Listeria_monocytogenes_1639_v10	0.8986
Listeria_monocytogenes_1639_v53	Listeria_monocytogenes_1639_v10	0.9062
Listeria_monocytogenes_1639_v52	Listeria_monocytogenes_1639_v10	0.9248
Listeria_monocytogenes_1639_v51	Listeria_monocytogenes_1639_v10	0.9129
Listeria_monocytogenes_1639_v50	Listeria_monocytogenes_1639_v10	0.9217
Listeria_monocytogenes_1639_v4	Listeria_monocytogenes_1639_v10	0.9549
Listeria_monocytogenes_1639_v49	Listeria_monocytogenes_1639_v10	0.9196
Listeria_monocytogenes_1639_v48	Listeria_monocytogenes_1639_v10	0.926
Listeria_monocytogenes_1639_v47	Listeria_monocytogenes_1639_v10	0.9206
Listeria_monocytogenes_1639_v46	Listeria_monocytogenes_1639_v10	0.9155
Listeria_monocytogenes_1639_v45	Listeria_monocytogenes_1639_v10	0.9131
Listeria_monocytogenes_1639_v44	Listeria_monocytogenes_1639_v10	0.922
Listeria_monocytogenes_1639_v43	Listeria_monocytogenes_1639_v10	0.922
Listeria_monocytogenes_1639_v42	Listeria_monocytogenes_1639_v10	0.9037
Listeria_monocytogenes_1639_v41	Listeria_monocytogenes_1639_v10	0.8976
Listeria_monocytogenes_1639_v40	Listeria_monocytogenes_1639_v10	0.9209

Listeria_monocytogenes_1639_v3	Listeria_monocytogenes_1639_v10	0.9552
Listeria_monocytogenes_1639_v39	Listeria_monocytogenes_1639_v10	0.9373
Listeria_monocytogenes_1639_v38	Listeria_monocytogenes_1639_v10	0.9258
Listeria_monocytogenes_1639_v37	Listeria_monocytogenes_1639_v10	0.9523
Listeria_monocytogenes_1639_v36	Listeria_monocytogenes_1639_v10	0.9162
Listeria_monocytogenes_1639_v35	Listeria_monocytogenes_1639_v10	0.92
Listeria_monocytogenes_1639_v34	Listeria_monocytogenes_1639_v10	0.9201
Listeria_monocytogenes_1639_v33	Listeria_monocytogenes_1639_v10	0.9509
Listeria_monocytogenes_1639_v32	Listeria_monocytogenes_1639_v10	0.9368
Listeria_monocytogenes_1639_v31	Listeria_monocytogenes_1639_v10	0.9205
Listeria_monocytogenes_1639_v30	Listeria_monocytogenes_1639_v10	0.9432
Listeria_monocytogenes_1639_v2	Listeria_monocytogenes_1639_v10	0.9282
Listeria_monocytogenes_1639_v29	Listeria_monocytogenes_1639_v10	0.9302
Listeria_monocytogenes_1639_v28	Listeria_monocytogenes_1639_v10	0.9331
Listeria_monocytogenes_1639_v27	Listeria_monocytogenes_1639_v10	0.9199
Listeria_monocytogenes_1639_v26	Listeria_monocytogenes_1639_v10	0.9196
Listeria_monocytogenes_1639_v25	Listeria_monocytogenes_1639_v10	0.9199
Listeria_monocytogenes_1639_v24	Listeria_monocytogenes_1639_v10	0.9205
Listeria_monocytogenes_1639_v23	Listeria_monocytogenes_1639_v10	0.9202
Listeria_monocytogenes_1639_v22	Listeria_monocytogenes_1639_v10	0.9549
Listeria_monocytogenes_1639_v21	Listeria_monocytogenes_1639_v10	0.9216
Listeria_monocytogenes_1639_v20	Listeria_monocytogenes_1639_v10	0.9317
Listeria_monocytogenes_1639_v1	Listeria_monocytogenes_1639_v10	0.9072
Listeria_monocytogenes_1639_v19	Listeria_monocytogenes_1639_v10	0.9313
Listeria_monocytogenes_1639_v198	Listeria_monocytogenes_1639_v10	0.8724
Listeria_monocytogenes_1639_v197	Listeria_monocytogenes_1639_v10	0.905
Listeria_monocytogenes_1639_v196	Listeria_monocytogenes_1639_v10	0.9069
Listeria_monocytogenes_1639_v195	Listeria_monocytogenes_1639_v10	0.9116
Listeria_monocytogenes_1639_v194	Listeria_monocytogenes_1639_v10	0.9109
Listeria_monocytogenes_1639_v193	Listeria_monocytogenes_1639_v10	0.9109
Listeria_monocytogenes_1639_v192	Listeria_monocytogenes_1639_v10	0.8757
Listeria_monocytogenes_1639_v191	Listeria_monocytogenes_1639_v10	0.918
Listeria_monocytogenes_1639_v190	Listeria_monocytogenes_1639_v10	0.9196
Listeria_monocytogenes_1639_v18	Listeria_monocytogenes_1639_v10	0.9359
Listeria_monocytogenes_1639_v189	Listeria_monocytogenes_1639_v10	0.9185
Listeria_monocytogenes_1639_v188	Listeria_monocytogenes_1639_v10	0.9209
Listeria_monocytogenes_1639_v187	Listeria_monocytogenes_1639_v10	0.9196
Listeria_monocytogenes_1639_v186	Listeria_monocytogenes_1639_v10	0.9196
Listeria_monocytogenes_1639_v185	Listeria_monocytogenes_1639_v10	0.9199
Listeria_monocytogenes_1639_v184	Listeria_monocytogenes_1639_v10	0.9192
Listeria_monocytogenes_1639_v183	Listeria_monocytogenes_1639_v10	0.9143
Listeria_monocytogenes_1639_v182	Listeria_monocytogenes_1639_v10	0.9164
Listeria_monocytogenes_1639_v181	Listeria_monocytogenes_1639_v10	0.8926
Listeria_monocytogenes_1639_v180	Listeria_monocytogenes_1639_v10	0.8926
Listeria_monocytogenes_1639_v17	Listeria_monocytogenes_1639_v10	0.9362
Listeria_monocytogenes_1639_v179	Listeria_monocytogenes_1639_v10	0.9122
Listeria_monocytogenes_1639_v178	Listeria_monocytogenes_1639_v10	0.9192
Listeria_monocytogenes_1639_v177	Listeria_monocytogenes_1639_v10	0.9284
Listeria_monocytogenes_1639_v176	Listeria_monocytogenes_1639_v10	0.9062
Listeria_monocytogenes_1639_v175	Listeria_monocytogenes_1639_v10	0.9248
Listeria_monocytogenes_1639_v174	Listeria_monocytogenes_1639_v10	0.9197
Listeria_monocytogenes_1639_v173	Listeria_monocytogenes_1639_v10	0.935
Listeria_monocytogenes_1639_v172	Listeria_monocytogenes_1639_v10	0.9064
Listeria_monocytogenes_1639_v171	Listeria_monocytogenes_1639_v10	0.9098
Listeria_monocytogenes_1639_v170	Listeria_monocytogenes_1639_v10	0.9175
Listeria_monocytogenes_1639_v16	Listeria_monocytogenes_1639_v10	0.9096
Listeria_monocytogenes_1639_v169	Listeria_monocytogenes_1639_v10	0.9295
Listeria_monocytogenes_1639_v168	Listeria_monocytogenes_1639_v10	0.9116
Listeria_monocytogenes_1639_v167	Listeria_monocytogenes_1639_v10	0.9168
Listeria_monocytogenes_1639_v166	Listeria_monocytogenes_1639_v10	0.9123
Listeria_monocytogenes_1639_v165	Listeria_monocytogenes_1639_v10	0.9292
Listeria_monocytogenes_1639_v164	Listeria_monocytogenes_1639_v10	0.927
Listeria_monocytogenes_1639_v163	Listeria_monocytogenes_1639_v10	0.929
Listeria_monocytogenes_1639_v162	Listeria_monocytogenes_1639_v10	0.927
Listeria_monocytogenes_1639_v161	Listeria_monocytogenes_1639_v10	0.9282
Listeria_monocytogenes_1639_v160	Listeria_monocytogenes_1639_v10	0.9282
Listeria_monocytogenes_1639_v15	Listeria_monocytogenes_1639_v10	0.9585
Listeria_monocytogenes_1639_v159	Listeria_monocytogenes_1639_v10	0.9284
Listeria_monocytogenes_1639_v158	Listeria_monocytogenes_1639_v10	0.9275
Listeria_monocytogenes_1639_v157	Listeria_monocytogenes_1639_v10	0.9275
Listeria_monocytogenes_1639_v156	Listeria_monocytogenes_1639_v10	0.9043
Listeria_monocytogenes_1639_v155	Listeria_monocytogenes_1639_v10	0.9226
Listeria_monocytogenes_1639_v154	Listeria_monocytogenes_1639_v10	0.929
Listeria_monocytogenes_1639_v153	Listeria_monocytogenes_1639_v10	0.9141
Listeria_monocytogenes_1639_v152	Listeria_monocytogenes_1639_v10	0.9284
Listeria_monocytogenes_1639_v151	Listeria_monocytogenes_1639_v10	0.9284
Listeria_monocytogenes_1639_v150	Listeria_monocytogenes_1639_v10	0.9245
Listeria_monocytogenes_1639_v14	Listeria_monocytogenes_1639_v10	0.9438
Listeria_monocytogenes_1639_v149	Listeria_monocytogenes_1639_v10	0.9243
Listeria_monocytogenes_1639_v148	Listeria_monocytogenes_1639_v10	0.9277
Listeria_monocytogenes_1639_v147	Listeria_monocytogenes_1639_v10	0.9155
Listeria_monocytogenes_1639_v146	Listeria_monocytogenes_1639_v10	0.9224
Listeria_monocytogenes_1639_v145	Listeria_monocytogenes_1639_v10	0.9137
Listeria_monocytogenes_1639_v144	Listeria_monocytogenes_1639_v10	0.9289
Listeria_monocytogenes_1639_v143	Listeria_monocytogenes_1639_v10	0.9214
Listeria_monocytogenes_1639_v142	Listeria_monocytogenes_1639_v10	0.9293
Listeria_monocytogenes_1639_v141	Listeria_monocytogenes_1639_v10	0.9367
Listeria_monocytogenes_1639_v140	Listeria_monocytogenes_1639_v10	0.93
Listeria_monocytogenes_1639_v13	Listeria_monocytogenes_1639_v10	0.9585
Listeria_monocytogenes_1639_v139	Listeria_monocytogenes_1639_v10	0.9283
Listeria_monocytogenes_1639_v138	Listeria_monocytogenes_1639_v10	0.9312

Listeria_monocytogenes_1639_v137	Listeria_monocytogenes_1639_v10	0.9312
Listeria_monocytogenes_1639_v136	Listeria_monocytogenes_1639_v10	0.9274
Listeria_monocytogenes_1639_v135	Listeria_monocytogenes_1639_v10	0.8899
Listeria_monocytogenes_1639_v134	Listeria_monocytogenes_1639_v10	0.9226
Listeria_monocytogenes_1639_v133	Listeria_monocytogenes_1639_v10	0.9234
Listeria_monocytogenes_1639_v132	Listeria_monocytogenes_1639_v10	0.889
Listeria_monocytogenes_1639_v131	Listeria_monocytogenes_1639_v10	0.9239
Listeria_monocytogenes_1639_v130	Listeria_monocytogenes_1639_v10	0.9193
Listeria_monocytogenes_1639_v12	Listeria_monocytogenes_1639_v10	0.9243
Listeria_monocytogenes_1639_v129	Listeria_monocytogenes_1639_v10	0.9494
Listeria_monocytogenes_1639_v128	Listeria_monocytogenes_1639_v10	0.9332
Listeria_monocytogenes_1639_v127	Listeria_monocytogenes_1639_v10	0.9161
Listeria_monocytogenes_1639_v126	Listeria_monocytogenes_1639_v10	0.9314
Listeria_monocytogenes_1639_v125	Listeria_monocytogenes_1639_v10	0.915
Listeria_monocytogenes_1639_v124	Listeria_monocytogenes_1639_v10	0.9014
Listeria_monocytogenes_1639_v123	Listeria_monocytogenes_1639_v10	0.9299
Listeria_monocytogenes_1639_v122	Listeria_monocytogenes_1639_v10	0.9299
Listeria_monocytogenes_1639_v121	Listeria_monocytogenes_1639_v10	0.9299
Listeria_monocytogenes_1639_v120	Listeria_monocytogenes_1639_v10	0.9299
Listeria_monocytogenes_1639_v11	Listeria_monocytogenes_1639_v10	0.9077
Listeria_monocytogenes_1639_v119	Listeria_monocytogenes_1639_v10	0.9299
Listeria_monocytogenes_1639_v118	Listeria_monocytogenes_1639_v10	0.9299
Listeria_monocytogenes_1639_v117	Listeria_monocytogenes_1639_v10	0.9256
Listeria_monocytogenes_1639_v116	Listeria_monocytogenes_1639_v10	0.9221
Listeria_monocytogenes_1639_v115	Listeria_monocytogenes_1639_v10	0.9221
Listeria_monocytogenes_1639_v114	Listeria_monocytogenes_1639_v10	0.9252
Listeria_monocytogenes_1639_v113	Listeria_monocytogenes_1639_v10	0.9597
Listeria_monocytogenes_1639_v112	Listeria_monocytogenes_1639_v10	0.9297
Listeria_monocytogenes_1639_v111	Listeria_monocytogenes_1639_v10	0.9221
Listeria_monocytogenes_1639_v110	Listeria_monocytogenes_1639_v10	0.9205
Listeria_monocytogenes_1639_v10	Listeria_monocytogenes_1639_v10	0.0714
Listeria_welshimeri_1643	Listeria_monocytogenes_1639_v110	0.8705
Listeria_welshimeri_serovar_6b_str_SLCC5334_386043	Listeria_monocytogenes_1639_v110	0.8699
Listeria_seeligeri_serovar_1_2b_str_SLCC3954_683837	Listeria_monocytogenes_1639_v110	0.878
Listeria_seeligeri_FSL_S4-171_702452	Listeria_monocytogenes_1639_v110	0.8153
Listeria_seeligeri_FSL_N1-067_702453	Listeria_monocytogenes_1639_v110	0.828
Listeria_seeligeri_1640_v1	Listeria_monocytogenes_1639_v110	0.8757
Listeria_seeligeri_1640	Listeria_monocytogenes_1639_v110	0.8774
Listeria_monocytogenes_WSLC1042_1457188	Listeria_monocytogenes_1639_v110	0.9223
Listeria_monocytogenes_WSLC1001_1457187	Listeria_monocytogenes_1639_v110	0.9801
Listeria_monocytogenes_SLCC7179_879090	Listeria_monocytogenes_1639_v110	0.9617
Listeria_monocytogenes_SLCC5850_882096	Listeria_monocytogenes_1639_v110	0.98
Listeria_monocytogenes_SLCC2755_932919	Listeria_monocytogenes_1639_v110	0.9277
Listeria_monocytogenes_SLCC2540_879089	Listeria_monocytogenes_1639_v110	0.9398
Listeria_monocytogenes_SLCC2479_882020	Listeria_monocytogenes_1639_v110	0.9541
Listeria_monocytogenes_SLCC2378_879088	Listeria_monocytogenes_1639_v110	0.9237
Listeria_monocytogenes_SLCC2376_882097	Listeria_monocytogenes_1639_v110	0.9358
Listeria_monocytogenes_SLCC2372_932920	Listeria_monocytogenes_1639_v110	0.947
Listeria_monocytogenes_serotype_4b_str_LL195_1230340	Listeria_monocytogenes_1639_v110	0.9296
Listeria_monocytogenes_serotype_4b_str_LIS0087_1134669	Listeria_monocytogenes_1639_v110	0.9825
Listeria_monocytogenes_serotype_4b_str_F2365_265669	Listeria_monocytogenes_1639_v110	0.9296
Listeria_monocytogenes_serotype_4b_str_CLIP_80459_568819	Listeria_monocytogenes_1639_v110	0.9364
Listeria_monocytogenes_serotype_4b_str_81-0861_1196161	Listeria_monocytogenes_1639_v110	0.9256
Listeria_monocytogenes_serotype_4b_str_81-0592_1196160	Listeria_monocytogenes_1639_v110	0.9267
Listeria_monocytogenes_serotype_4b_str_81-0558_1196159	Listeria_monocytogenes_1639_v110	0.9274
Listeria_monocytogenes_serotype_4b_str_10-0809_1196162	Listeria_monocytogenes_1639_v110	0.9267
Listeria_monocytogenes_serotype_4b_str_02-6680_1196175	Listeria_monocytogenes_1639_v110	0.9347
Listeria_monocytogenes_serotype_4b_str_02-6679_1196174	Listeria_monocytogenes_1639_v110	0.9347
Listeria_monocytogenes_serotype_4b_str_02-1792_1196173	Listeria_monocytogenes_1639_v110	0.927
Listeria_monocytogenes_serotype_4b_str_02-1289_1196172	Listeria_monocytogenes_1639_v110	0.927
Listeria_monocytogenes_serotype_4b_str_02-1103_1196171	Listeria_monocytogenes_1639_v110	0.927
Listeria_monocytogenes_serotype_3c_str_10-5027_1196195	Listeria_monocytogenes_1639_v110	0.9507
Listeria_monocytogenes_serotype_1_2c_str_10-5026_1196194	Listeria_monocytogenes_1639_v110	0.9518
Listeria_monocytogenes_serotype_1_2c_str_10-5025_1196193	Listeria_monocytogenes_1639_v110	0.9518
Listeria_monocytogenes_serotype_1_2b_str_10-0811_1196164	Listeria_monocytogenes_1639_v110	0.9435
Listeria_monocytogenes_serotype_1_2b_str_10-0810_1196163	Listeria_monocytogenes_1639_v110	0.9435
Listeria_monocytogenes_serotype_1_2a_str_99-6370_1196179	Listeria_monocytogenes_1639_v110	0.9447
Listeria_monocytogenes_serotype_1_2a_str_98-2035_1196178	Listeria_monocytogenes_1639_v110	0.9447
Listeria_monocytogenes_serotype_1_2a_str_95-0093_1196177	Listeria_monocytogenes_1639_v110	0.9496
Listeria_monocytogenes_serotype_1_2a_str_88-0478_1196176	Listeria_monocytogenes_1639_v110	0.9492
Listeria_monocytogenes_serotype_1_2a_str_10-5024_1196192	Listeria_monocytogenes_1639_v110	0.9503
Listeria_monocytogenes_serotype_1_2a_str_10-4758_1196168	Listeria_monocytogenes_1639_v110	0.9604
Listeria_monocytogenes_serotype_1_2a_str_10-4754_1196167	Listeria_monocytogenes_1639_v110	0.9604
Listeria_monocytogenes_serotype_1_2a_str_10-1321_1196191	Listeria_monocytogenes_1639_v110	0.9503
Listeria_monocytogenes_serotype_1_2a_str_10-1047_1196190	Listeria_monocytogenes_1639_v110	0.9503
Listeria_monocytogenes_serotype_1_2a_str_10-1046_1196189	Listeria_monocytogenes_1639_v110	0.9503
Listeria_monocytogenes_serotype_1_2a_str_10-0934_1196170	Listeria_monocytogenes_1639_v110	0.966
Listeria_monocytogenes_serotype_1_2a_str_10-0933_1196169	Listeria_monocytogenes_1639_v110	0.966
Listeria_monocytogenes_serotype_1_2a_str_10-0815_1196188	Listeria_monocytogenes_1639_v110	0.9461
Listeria_monocytogenes_serotype_1_2a_str_10-0814_1196187	Listeria_monocytogenes_1639_v110	0.9503
Listeria_monocytogenes_serotype_1_2a_str_10-0813_1196166	Listeria_monocytogenes_1639_v110	0.9831
Listeria_monocytogenes_serotype_1_2a_str_10-0812_1196165	Listeria_monocytogenes_1639_v110	0.9831
Listeria_monocytogenes_serotype_1_2a_str_08-7669_1196186	Listeria_monocytogenes_1639_v110	0.9494
Listeria_monocytogenes_serotype_1_2a_str_08-7374_1196185	Listeria_monocytogenes_1639_v110	0.9503
Listeria_monocytogenes_serotype_1_2a_str_08-6997_1196184	Listeria_monocytogenes_1639_v110	0.9461
Listeria_monocytogenes_serotype_1_2a_str_08-6569_1196183	Listeria_monocytogenes_1639_v110	0.9461
Listeria_monocytogenes_serotype_1_2a_str_08-6056_1196182	Listeria_monocytogenes_1639_v110	0.9461
Listeria_monocytogenes_serotype_1_2a_str_04-5457_1196181	Listeria_monocytogenes_1639_v110	0.9503
Listeria_monocytogenes_serotype_1_2a_str_02-5993_1196180	Listeria_monocytogenes_1639_v110	0.9499
Listeria_monocytogenes_R479a_1437838	Listeria_monocytogenes_1639_v110	0.9455
Listeria_monocytogenes_N53-1_1234142	Listeria_monocytogenes_1639_v110	0.9083

Listeria_monocytogenes_M7_1030009	Listeria_monocytogenes_1639_v110	0.9141
Listeria_monocytogenes_LO28_393132	Listeria_monocytogenes_1639_v110	0.9594
Listeria_monocytogenes_L99_563174	Listeria_monocytogenes_1639_v110	0.9148
Listeria_monocytogenes_L312_882094	Listeria_monocytogenes_1639_v110	0.9364
Listeria_monocytogenes_J1816_930781	Listeria_monocytogenes_1639_v110	0.9221
Listeria_monocytogenes_J1-220_930782	Listeria_monocytogenes_1639_v110	0.928
Listeria_monocytogenes_J0161_393130	Listeria_monocytogenes_1639_v110	0.9512
Listeria_monocytogenes_HCC23_552536	Listeria_monocytogenes_1639_v110	0.9144
Listeria_monocytogenes_FSL_R2-561_393126	Listeria_monocytogenes_1639_v110	0.9493
Listeria_monocytogenes_FSL_J1-208_393119	Listeria_monocytogenes_1639_v110	0.901
Listeria_monocytogenes_FSL_J1-175_393118	Listeria_monocytogenes_1639_v110	0.943
Listeria_monocytogenes_Finland_1998_393127	Listeria_monocytogenes_1639_v110	0.9534
Listeria_monocytogenes_EGD-e_169963_v1	Listeria_monocytogenes_1639_v110	0.9582
Listeria_monocytogenes_EGD-e_169963	Listeria_monocytogenes_1639_v110	0.9586
Listeria_monocytogenes_EGD_1334565	Listeria_monocytogenes_1639_v110	0.9807
Listeria_monocytogenes_CFSAN002349_1299895	Listeria_monocytogenes_1639_v110	0.9347
Listeria_monocytogenes_ATCC_19117_882095_v1	Listeria_monocytogenes_1639_v110	0.9303
Listeria_monocytogenes_ATCC_19117_882095	Listeria_monocytogenes_1639_v110	0.9303
Listeria_monocytogenes_6179_1288295	Listeria_monocytogenes_1639_v110	0.9463
Listeria_monocytogenes_1639_v9	Listeria_monocytogenes_1639_v110	0.9296
Listeria_monocytogenes_1639_v99	Listeria_monocytogenes_1639_v110	0.9248
Listeria_monocytogenes_1639_v98	Listeria_monocytogenes_1639_v110	0.9504
Listeria_monocytogenes_1639_v97	Listeria_monocytogenes_1639_v110	0.9279
Listeria_monocytogenes_1639_v96	Listeria_monocytogenes_1639_v110	0.9215
Listeria_monocytogenes_1639_v95	Listeria_monocytogenes_1639_v110	0.9215
Listeria_monocytogenes_1639_v94	Listeria_monocytogenes_1639_v110	0.9218
Listeria_monocytogenes_1639_v93	Listeria_monocytogenes_1639_v110	0.9436
Listeria_monocytogenes_1639_v92	Listeria_monocytogenes_1639_v110	0.9252
Listeria_monocytogenes_1639_v91	Listeria_monocytogenes_1639_v110	0.9291
Listeria_monocytogenes_1639_v90	Listeria_monocytogenes_1639_v110	0.9504
Listeria_monocytogenes_1639_v8	Listeria_monocytogenes_1639_v110	0.9362
Listeria_monocytogenes_1639_v89	Listeria_monocytogenes_1639_v110	0.9389
Listeria_monocytogenes_1639_v88	Listeria_monocytogenes_1639_v110	0.9671
Listeria_monocytogenes_1639_v87	Listeria_monocytogenes_1639_v110	0.9806
Listeria_monocytogenes_1639_v86	Listeria_monocytogenes_1639_v110	0.9799
Listeria_monocytogenes_1639_v85	Listeria_monocytogenes_1639_v110	0.9253
Listeria_monocytogenes_1639_v84	Listeria_monocytogenes_1639_v110	0.961
Listeria_monocytogenes_1639_v83	Listeria_monocytogenes_1639_v110	0.9315
Listeria_monocytogenes_1639_v82	Listeria_monocytogenes_1639_v110	0.9311
Listeria_monocytogenes_1639_v81	Listeria_monocytogenes_1639_v110	0.9502
Listeria_monocytogenes_1639_v80	Listeria_monocytogenes_1639_v110	0.9467
Listeria_monocytogenes_1639_v7	Listeria_monocytogenes_1639_v110	0.9362
Listeria_monocytogenes_1639_v79	Listeria_monocytogenes_1639_v110	0.9511
Listeria_monocytogenes_1639_v78	Listeria_monocytogenes_1639_v110	0.9499
Listeria_monocytogenes_1639_v77	Listeria_monocytogenes_1639_v110	0.944
Listeria_monocytogenes_1639_v76	Listeria_monocytogenes_1639_v110	0.9484
Listeria_monocytogenes_1639_v75	Listeria_monocytogenes_1639_v110	0.9148
Listeria_monocytogenes_1639_v74	Listeria_monocytogenes_1639_v110	0.9638
Listeria_monocytogenes_1639_v73	Listeria_monocytogenes_1639_v110	0.9522
Listeria_monocytogenes_1639_v72	Listeria_monocytogenes_1639_v110	0.9295
Listeria_monocytogenes_1639_v71	Listeria_monocytogenes_1639_v110	0.9404
Listeria_monocytogenes_1639_v70	Listeria_monocytogenes_1639_v110	0.9479
Listeria_monocytogenes_1639_v6	Listeria_monocytogenes_1639_v110	0.9356
Listeria_monocytogenes_1639_v69	Listeria_monocytogenes_1639_v110	0.927
Listeria_monocytogenes_1639_v68	Listeria_monocytogenes_1639_v110	0.9487
Listeria_monocytogenes_1639_v67	Listeria_monocytogenes_1639_v110	0.9431
Listeria_monocytogenes_1639_v66	Listeria_monocytogenes_1639_v110	0.9567
Listeria_monocytogenes_1639_v65	Listeria_monocytogenes_1639_v110	0.9568
Listeria_monocytogenes_1639_v64	Listeria_monocytogenes_1639_v110	0.9495
Listeria_monocytogenes_1639_v63	Listeria_monocytogenes_1639_v110	0.9345
Listeria_monocytogenes_1639_v62	Listeria_monocytogenes_1639_v110	0.9471
Listeria_monocytogenes_1639_v61	Listeria_monocytogenes_1639_v110	0.952
Listeria_monocytogenes_1639_v60	Listeria_monocytogenes_1639_v110	0.9461
Listeria_monocytogenes_1639_v5	Listeria_monocytogenes_1639_v110	0.9015
Listeria_monocytogenes_1639_v59	Listeria_monocytogenes_1639_v110	0.9284
Listeria_monocytogenes_1639_v58	Listeria_monocytogenes_1639_v110	0.9307
Listeria_monocytogenes_1639_v57	Listeria_monocytogenes_1639_v110	0.9199
Listeria_monocytogenes_1639_v56	Listeria_monocytogenes_1639_v110	0.9258
Listeria_monocytogenes_1639_v55	Listeria_monocytogenes_1639_v110	0.9236
Listeria_monocytogenes_1639_v54	Listeria_monocytogenes_1639_v110	0.9197
Listeria_monocytogenes_1639_v53	Listeria_monocytogenes_1639_v110	0.9386
Listeria_monocytogenes_1639_v52	Listeria_monocytogenes_1639_v110	0.9354
Listeria_monocytogenes_1639_v51	Listeria_monocytogenes_1639_v110	0.9292
Listeria_monocytogenes_1639_v50	Listeria_monocytogenes_1639_v110	0.9492
Listeria_monocytogenes_1639_v4	Listeria_monocytogenes_1639_v110	0.9143
Listeria_monocytogenes_1639_v49	Listeria_monocytogenes_1639_v110	0.9178
Listeria_monocytogenes_1639_v48	Listeria_monocytogenes_1639_v110	0.9473
Listeria_monocytogenes_1639_v47	Listeria_monocytogenes_1639_v110	0.9511
Listeria_monocytogenes_1639_v46	Listeria_monocytogenes_1639_v110	0.9427
Listeria_monocytogenes_1639_v45	Listeria_monocytogenes_1639_v110	0.9409
Listeria_monocytogenes_1639_v44	Listeria_monocytogenes_1639_v110	0.9503
Listeria_monocytogenes_1639_v43	Listeria_monocytogenes_1639_v110	0.9503
Listeria_monocytogenes_1639_v42	Listeria_monocytogenes_1639_v110	0.9347
Listeria_monocytogenes_1639_v41	Listeria_monocytogenes_1639_v110	0.9369
Listeria_monocytogenes_1639_v40	Listeria_monocytogenes_1639_v110	0.9482
Listeria_monocytogenes_1639_v3	Listeria_monocytogenes_1639_v110	0.9146
Listeria_monocytogenes_1639_v39	Listeria_monocytogenes_1639_v110	0.9382
Listeria_monocytogenes_1639_v38	Listeria_monocytogenes_1639_v110	0.9483
Listeria_monocytogenes_1639_v37	Listeria_monocytogenes_1639_v110	0.9412
Listeria_monocytogenes_1639_v36	Listeria_monocytogenes_1639_v110	0.9468
Listeria_monocytogenes_1639_v35	Listeria_monocytogenes_1639_v110	0.9518

Listeria_monocytogenes_1639_v34	Listeria_monocytogenes_1639_v110	0.9473
Listeria_monocytogenes_1639_v33	Listeria_monocytogenes_1639_v110	0.9435
Listeria_monocytogenes_1639_v32	Listeria_monocytogenes_1639_v110	0.9509
Listeria_monocytogenes_1639_v31	Listeria_monocytogenes_1639_v110	0.9476
Listeria_monocytogenes_1639_v30	Listeria_monocytogenes_1639_v110	0.9248
Listeria_monocytogenes_1639_v2	Listeria_monocytogenes_1639_v110	0.904
Listeria_monocytogenes_1639_v29	Listeria_monocytogenes_1639_v110	0.9379
Listeria_monocytogenes_1639_v28	Listeria_monocytogenes_1639_v110	0.9427
Listeria_monocytogenes_1639_v27	Listeria_monocytogenes_1639_v110	0.94
Listeria_monocytogenes_1639_v26	Listeria_monocytogenes_1639_v110	0.9404
Listeria_monocytogenes_1639_v25	Listeria_monocytogenes_1639_v110	0.9397
Listeria_monocytogenes_1639_v24	Listeria_monocytogenes_1639_v110	0.9295
Listeria_monocytogenes_1639_v23	Listeria_monocytogenes_1639_v110	0.9291
Listeria_monocytogenes_1639_v22	Listeria_monocytogenes_1639_v110	0.9445
Listeria_monocytogenes_1639_v21	Listeria_monocytogenes_1639_v110	0.9303
Listeria_monocytogenes_1639_v20	Listeria_monocytogenes_1639_v110	0.9339
Listeria_monocytogenes_1639_v1	Listeria_monocytogenes_1639_v110	0.942
Listeria_monocytogenes_1639_v19	Listeria_monocytogenes_1639_v110	0.9335
Listeria_monocytogenes_1639_v198	Listeria_monocytogenes_1639_v110	0.9095
Listeria_monocytogenes_1639_v197	Listeria_monocytogenes_1639_v110	0.9453
Listeria_monocytogenes_1639_v196	Listeria_monocytogenes_1639_v110	0.9643
Listeria_monocytogenes_1639_v195	Listeria_monocytogenes_1639_v110	0.938
Listeria_monocytogenes_1639_v194	Listeria_monocytogenes_1639_v110	0.9362
Listeria_monocytogenes_1639_v193	Listeria_monocytogenes_1639_v110	0.9365
Listeria_monocytogenes_1639_v192	Listeria_monocytogenes_1639_v110	0.8957
Listeria_monocytogenes_1639_v191	Listeria_monocytogenes_1639_v110	0.9617
Listeria_monocytogenes_1639_v190	Listeria_monocytogenes_1639_v110	0.9786
Listeria_monocytogenes_1639_v18	Listeria_monocytogenes_1639_v110	0.9219
Listeria_monocytogenes_1639_v189	Listeria_monocytogenes_1639_v110	0.9774
Listeria_monocytogenes_1639_v188	Listeria_monocytogenes_1639_v110	0.9571
Listeria_monocytogenes_1639_v187	Listeria_monocytogenes_1639_v110	0.9782
Listeria_monocytogenes_1639_v186	Listeria_monocytogenes_1639_v110	0.9786
Listeria_monocytogenes_1639_v185	Listeria_monocytogenes_1639_v110	0.9786
Listeria_monocytogenes_1639_v184	Listeria_monocytogenes_1639_v110	0.9782
Listeria_monocytogenes_1639_v183	Listeria_monocytogenes_1639_v110	0.9537
Listeria_monocytogenes_1639_v182	Listeria_monocytogenes_1639_v110	0.9559
Listeria_monocytogenes_1639_v181	Listeria_monocytogenes_1639_v110	0.9122
Listeria_monocytogenes_1639_v180	Listeria_monocytogenes_1639_v110	0.9122
Listeria_monocytogenes_1639_v17	Listeria_monocytogenes_1639_v110	0.9219
Listeria_monocytogenes_1639_v179	Listeria_monocytogenes_1639_v110	0.9515
Listeria_monocytogenes_1639_v178	Listeria_monocytogenes_1639_v110	0.9591
Listeria_monocytogenes_1639_v177	Listeria_monocytogenes_1639_v110	0.9343
Listeria_monocytogenes_1639_v176	Listeria_monocytogenes_1639_v110	0.9274
Listeria_monocytogenes_1639_v175	Listeria_monocytogenes_1639_v110	0.9651
Listeria_monocytogenes_1639_v174	Listeria_monocytogenes_1639_v110	0.9626
Listeria_monocytogenes_1639_v173	Listeria_monocytogenes_1639_v110	0.9438
Listeria_monocytogenes_1639_v172	Listeria_monocytogenes_1639_v110	0.9282
Listeria_monocytogenes_1639_v171	Listeria_monocytogenes_1639_v110	0.9665
Listeria_monocytogenes_1639_v170	Listeria_monocytogenes_1639_v110	0.9745
Listeria_monocytogenes_1639_v16	Listeria_monocytogenes_1639_v110	0.9368
Listeria_monocytogenes_1639_v169	Listeria_monocytogenes_1639_v110	0.9655
Listeria_monocytogenes_1639_v168	Listeria_monocytogenes_1639_v110	0.9538
Listeria_monocytogenes_1639_v167	Listeria_monocytogenes_1639_v110	0.954
Listeria_monocytogenes_1639_v166	Listeria_monocytogenes_1639_v110	0.9542
Listeria_monocytogenes_1639_v165	Listeria_monocytogenes_1639_v110	0.9651
Listeria_monocytogenes_1639_v164	Listeria_monocytogenes_1639_v110	0.9288
Listeria_monocytogenes_1639_v163	Listeria_monocytogenes_1639_v110	0.9388
Listeria_monocytogenes_1639_v162	Listeria_monocytogenes_1639_v110	0.9288
Listeria_monocytogenes_1639_v161	Listeria_monocytogenes_1639_v110	0.93
Listeria_monocytogenes_1639_v160	Listeria_monocytogenes_1639_v110	0.93
Listeria_monocytogenes_1639_v15	Listeria_monocytogenes_1639_v110	0.9294
Listeria_monocytogenes_1639_v159	Listeria_monocytogenes_1639_v110	0.9347
Listeria_monocytogenes_1639_v158	Listeria_monocytogenes_1639_v110	0.9296
Listeria_monocytogenes_1639_v157	Listeria_monocytogenes_1639_v110	0.9296
Listeria_monocytogenes_1639_v156	Listeria_monocytogenes_1639_v110	0.9739
Listeria_monocytogenes_1639_v155	Listeria_monocytogenes_1639_v110	0.9546
Listeria_monocytogenes_1639_v154	Listeria_monocytogenes_1639_v110	0.961
Listeria_monocytogenes_1639_v153	Listeria_monocytogenes_1639_v110	0.958
Listeria_monocytogenes_1639_v152	Listeria_monocytogenes_1639_v110	0.9351
Listeria_monocytogenes_1639_v151	Listeria_monocytogenes_1639_v110	0.9351
Listeria_monocytogenes_1639_v150	Listeria_monocytogenes_1639_v110	0.9898
Listeria_monocytogenes_1639_v14	Listeria_monocytogenes_1639_v110	0.9248
Listeria_monocytogenes_1639_v149	Listeria_monocytogenes_1639_v110	0.9807
Listeria_monocytogenes_1639_v148	Listeria_monocytogenes_1639_v110	0.934
Listeria_monocytogenes_1639_v147	Listeria_monocytogenes_1639_v110	0.92
Listeria_monocytogenes_1639_v146	Listeria_monocytogenes_1639_v110	0.9554
Listeria_monocytogenes_1639_v145	Listeria_monocytogenes_1639_v110	0.9517
Listeria_monocytogenes_1639_v144	Listeria_monocytogenes_1639_v110	0.9357
Listeria_monocytogenes_1639_v143	Listeria_monocytogenes_1639_v110	0.9546
Listeria_monocytogenes_1639_v142	Listeria_monocytogenes_1639_v110	0.936
Listeria_monocytogenes_1639_v141	Listeria_monocytogenes_1639_v110	0.9432
Listeria_monocytogenes_1639_v140	Listeria_monocytogenes_1639_v110	0.9364
Listeria_monocytogenes_1639_v13	Listeria_monocytogenes_1639_v110	0.9298
Listeria_monocytogenes_1639_v139	Listeria_monocytogenes_1639_v110	0.9342
Listeria_monocytogenes_1639_v138	Listeria_monocytogenes_1639_v110	0.9387
Listeria_monocytogenes_1639_v137	Listeria_monocytogenes_1639_v110	0.9384
Listeria_monocytogenes_1639_v136	Listeria_monocytogenes_1639_v110	0.9582
Listeria_monocytogenes_1639_v135	Listeria_monocytogenes_1639_v110	0.9148
Listeria_monocytogenes_1639_v134	Listeria_monocytogenes_1639_v110	0.9235
Listeria_monocytogenes_1639_v133	Listeria_monocytogenes_1639_v110	0.9539
Listeria_monocytogenes_1639_v132	Listeria_monocytogenes_1639_v110	0.9137

Listeria_monocytogenes_1639_v131	Listeria_monocytogenes_1639_v110	0.9357
Listeria_monocytogenes_1639_v130	Listeria_monocytogenes_1639_v110	0.9511
Listeria_monocytogenes_1639_v12	Listeria_monocytogenes_1639_v110	0.9321
Listeria_monocytogenes_1639_v129	Listeria_monocytogenes_1639_v110	0.9395
Listeria_monocytogenes_1639_v128	Listeria_monocytogenes_1639_v110	0.9393
Listeria_monocytogenes_1639_v127	Listeria_monocytogenes_1639_v110	0.9582
Listeria_monocytogenes_1639_v126	Listeria_monocytogenes_1639_v110	0.94
Listeria_monocytogenes_1639_v125	Listeria_monocytogenes_1639_v110	0.9508
Listeria_monocytogenes_1639_v124	Listeria_monocytogenes_1639_v110	0.9206
Listeria_monocytogenes_1639_v123	Listeria_monocytogenes_1639_v110	0.9347
Listeria_monocytogenes_1639_v122	Listeria_monocytogenes_1639_v110	0.9347
Listeria_monocytogenes_1639_v121	Listeria_monocytogenes_1639_v110	0.9347
Listeria_monocytogenes_1639_v120	Listeria_monocytogenes_1639_v110	0.9343
Listeria_monocytogenes_1639_v11	Listeria_monocytogenes_1639_v110	0.9437
Listeria_monocytogenes_1639_v119	Listeria_monocytogenes_1639_v110	0.9347
Listeria_monocytogenes_1639_v118	Listeria_monocytogenes_1639_v110	0.9347
Listeria_monocytogenes_1639_v117	Listeria_monocytogenes_1639_v110	0.9567
Listeria_monocytogenes_1639_v116	Listeria_monocytogenes_1639_v110	0.9237
Listeria_monocytogenes_1639_v115	Listeria_monocytogenes_1639_v110	0.9233
Listeria_monocytogenes_1639_v114	Listeria_monocytogenes_1639_v110	0.9797
Listeria_monocytogenes_1639_v113	Listeria_monocytogenes_1639_v110	0.9474
Listeria_monocytogenes_1639_v112	Listeria_monocytogenes_1639_v110	0.927
Listeria_monocytogenes_1639_v111	Listeria_monocytogenes_1639_v110	0.9353
Listeria_monocytogenes_1639_v110	Listeria_monocytogenes_1639_v110	0.0686
Listeria_welshimeri_1643	Listeria_monocytogenes_1639_v111	0.8725
Listeria_welshimeri_serovar_6b_str_SLCC5334_386043	Listeria_monocytogenes_1639_v111	0.8718
Listeria_seeligeri_serovar_1_2b_str_SLCC3954_683837	Listeria_monocytogenes_1639_v111	0.8885
Listeria_seeligeri_FSL_S4-171_702452	Listeria_monocytogenes_1639_v111	0.8215
Listeria_seeligeri_FSL_N1-067_702453	Listeria_monocytogenes_1639_v111	0.8343
Listeria_seeligeri_1640_v1	Listeria_monocytogenes_1639_v111	0.8911
Listeria_seeligeri_1640	Listeria_monocytogenes_1639_v111	0.8819
Listeria_monocytogenes_WSLC1042_1457188	Listeria_monocytogenes_1639_v111	0.9474
Listeria_monocytogenes_WSLC1001_1457187	Listeria_monocytogenes_1639_v111	0.9363
Listeria_monocytogenes_SLCC7179_879090	Listeria_monocytogenes_1639_v111	0.9391
Listeria_monocytogenes_SLCC5850_882096	Listeria_monocytogenes_1639_v111	0.9402
Listeria_monocytogenes_SLCC2755_932919	Listeria_monocytogenes_1639_v111	0.9797
Listeria_monocytogenes_SLCC2540_879089	Listeria_monocytogenes_1639_v111	0.9483
Listeria_monocytogenes_SLCC2479_882020	Listeria_monocytogenes_1639_v111	0.9289
Listeria_monocytogenes_SLCC2378_879088	Listeria_monocytogenes_1639_v111	0.9488
Listeria_monocytogenes_SLCC2376_882097	Listeria_monocytogenes_1639_v111	0.9325
Listeria_monocytogenes_SLCC2372_932920	Listeria_monocytogenes_1639_v111	0.9369
Listeria_monocytogenes_serotype_4b_str_LL195_1230340	Listeria_monocytogenes_1639_v111	0.955
Listeria_monocytogenes_serotype_4b_str_LIS0087_1134669	Listeria_monocytogenes_1639_v111	0.9186
Listeria_monocytogenes_serotype_4b_str_F2365_265669	Listeria_monocytogenes_1639_v111	0.955
Listeria_monocytogenes_serotype_4b_str_CLIP_80459_568819	Listeria_monocytogenes_1639_v111	0.9572
Listeria_monocytogenes_serotype_4b_str_81-0861_1196161	Listeria_monocytogenes_1639_v111	0.9389
Listeria_monocytogenes_serotype_4b_str_81-0592_1196160	Listeria_monocytogenes_1639_v111	0.94
Listeria_monocytogenes_serotype_4b_str_81-0558_1196159	Listeria_monocytogenes_1639_v111	0.9411
Listeria_monocytogenes_serotype_4b_str_10-0809_1196162	Listeria_monocytogenes_1639_v111	0.9404
Listeria_monocytogenes_serotype_4b_str_02-6680_1196175	Listeria_monocytogenes_1639_v111	0.9547
Listeria_monocytogenes_serotype_4b_str_02-6679_1196174	Listeria_monocytogenes_1639_v111	0.9547
Listeria_monocytogenes_serotype_4b_str_02-1792_1196173	Listeria_monocytogenes_1639_v111	0.9404
Listeria_monocytogenes_serotype_4b_str_02-1289_1196172	Listeria_monocytogenes_1639_v111	0.9404
Listeria_monocytogenes_serotype_4b_str_02-1103_1196171	Listeria_monocytogenes_1639_v111	0.9404
Listeria_monocytogenes_serotype_3c_str_10-5027_1196195	Listeria_monocytogenes_1639_v111	0.9289
Listeria_monocytogenes_serotype_1_2c_str_10-5026_1196194	Listeria_monocytogenes_1639_v111	0.9304
Listeria_monocytogenes_serotype_1_2c_str_10-5025_1196193	Listeria_monocytogenes_1639_v111	0.9304
Listeria_monocytogenes_serotype_1_2b_str_10-0811_1196164	Listeria_monocytogenes_1639_v111	0.9463
Listeria_monocytogenes_serotype_1_2b_str_10-0810_1196163	Listeria_monocytogenes_1639_v111	0.9463
Listeria_monocytogenes_serotype_1_2a_str_99-6370_1196179	Listeria_monocytogenes_1639_v111	0.9265
Listeria_monocytogenes_serotype_1_2a_str_98-2035_1196178	Listeria_monocytogenes_1639_v111	0.9265
Listeria_monocytogenes_serotype_1_2a_str_95-0093_1196177	Listeria_monocytogenes_1639_v111	0.923
Listeria_monocytogenes_serotype_1_2a_str_88-0478_1196176	Listeria_monocytogenes_1639_v111	0.9226
Listeria_monocytogenes_serotype_1_2a_str_10-5024_1196192	Listeria_monocytogenes_1639_v111	0.9234
Listeria_monocytogenes_serotype_1_2a_str_10-4758_1196168	Listeria_monocytogenes_1639_v111	0.9361
Listeria_monocytogenes_serotype_1_2a_str_10-4754_1196167	Listeria_monocytogenes_1639_v111	0.9361
Listeria_monocytogenes_serotype_1_2a_str_10-1321_1196191	Listeria_monocytogenes_1639_v111	0.9234
Listeria_monocytogenes_serotype_1_2a_str_10-1047_1196190	Listeria_monocytogenes_1639_v111	0.9234
Listeria_monocytogenes_serotype_1_2a_str_10-1046_1196189	Listeria_monocytogenes_1639_v111	0.9234
Listeria_monocytogenes_serotype_1_2a_str_10-0934_1196170	Listeria_monocytogenes_1639_v111	0.9448
Listeria_monocytogenes_serotype_1_2a_str_10-0933_1196169	Listeria_monocytogenes_1639_v111	0.9448
Listeria_monocytogenes_serotype_1_2a_str_10-0815_1196188	Listeria_monocytogenes_1639_v111	0.9271
Listeria_monocytogenes_serotype_1_2a_str_10-0814_1196187	Listeria_monocytogenes_1639_v111	0.9234
Listeria_monocytogenes_serotype_1_2a_str_10-0813_1196166	Listeria_monocytogenes_1639_v111	0.9335
Listeria_monocytogenes_serotype_1_2a_str_10-0812_1196165	Listeria_monocytogenes_1639_v111	0.9335
Listeria_monocytogenes_serotype_1_2a_str_08-7669_1196186	Listeria_monocytogenes_1639_v111	0.9313
Listeria_monocytogenes_serotype_1_2a_str_08-7374_1196185	Listeria_monocytogenes_1639_v111	0.9234
Listeria_monocytogenes_serotype_1_2a_str_08-6997_1196184	Listeria_monocytogenes_1639_v111	0.9271
Listeria_monocytogenes_serotype_1_2a_str_08-6569_1196183	Listeria_monocytogenes_1639_v111	0.9271
Listeria_monocytogenes_serotype_1_2a_str_08-6056_1196182	Listeria_monocytogenes_1639_v111	0.9271
Listeria_monocytogenes_serotype_1_2a_str_04-5457_1196181	Listeria_monocytogenes_1639_v111	0.9234
Listeria_monocytogenes_serotype_1_2a_str_02-5993_1196180	Listeria_monocytogenes_1639_v111	0.923
Listeria_monocytogenes_R479a_1437838	Listeria_monocytogenes_1639_v111	0.929
Listeria_monocytogenes_N53-1_1234142	Listeria_monocytogenes_1639_v111	0.8923
Listeria_monocytogenes_M7_1030009	Listeria_monocytogenes_1639_v111	0.8979
Listeria_monocytogenes_LO28_393132	Listeria_monocytogenes_1639_v111	0.9275
Listeria_monocytogenes_L99_563174	Listeria_monocytogenes_1639_v111	0.8986
Listeria_monocytogenes_L312_882094	Listeria_monocytogenes_1639_v111	0.9576
Listeria_monocytogenes_J1816_930781	Listeria_monocytogenes_1639_v111	0.9354
Listeria_monocytogenes_J1-220_930782	Listeria_monocytogenes_1639_v111	0.9297
Listeria_monocytogenes_J0161_393130	Listeria_monocytogenes_1639_v111	0.935

Listeria_monocytogenes_HCC23_552536	Listeria_monocytogenes_1639_v111	0.8983
Listeria_monocytogenes_FSL_R2-561_393126	Listeria_monocytogenes_1639_v111	0.9282
Listeria_monocytogenes_FSL_J1-208_393119	Listeria_monocytogenes_1639_v111	0.9061
Listeria_monocytogenes_FSL_J1-175_393118	Listeria_monocytogenes_1639_v111	0.955
Listeria_monocytogenes_Finland_1998_393127	Listeria_monocytogenes_1639_v111	0.9483
Listeria_monocytogenes_EGD-e_169963_v1	Listeria_monocytogenes_1639_v111	0.937
Listeria_monocytogenes_EGD-e_169963	Listeria_monocytogenes_1639_v111	0.9373
Listeria_monocytogenes_EGD_1334565	Listeria_monocytogenes_1639_v111	0.9409
Listeria_monocytogenes_CFSAN002349_1299895	Listeria_monocytogenes_1639_v111	0.9559
Listeria_monocytogenes_ATCC_19117_882095_v1	Listeria_monocytogenes_1639_v111	0.9472
Listeria_monocytogenes_ATCC_19117_882095	Listeria_monocytogenes_1639_v111	0.9472
Listeria_monocytogenes_6179_1288295	Listeria_monocytogenes_1639_v111	0.9266
Listeria_monocytogenes_1639_v9	Listeria_monocytogenes_1639_v111	0.9298
Listeria_monocytogenes_1639_v99	Listeria_monocytogenes_1639_v111	0.9459
Listeria_monocytogenes_1639_v98	Listeria_monocytogenes_1639_v111	0.9375
Listeria_monocytogenes_1639_v97	Listeria_monocytogenes_1639_v111	0.9462
Listeria_monocytogenes_1639_v96	Listeria_monocytogenes_1639_v111	0.9451
Listeria_monocytogenes_1639_v95	Listeria_monocytogenes_1639_v111	0.9452
Listeria_monocytogenes_1639_v94	Listeria_monocytogenes_1639_v111	0.9455
Listeria_monocytogenes_1639_v93	Listeria_monocytogenes_1639_v111	0.9349
Listeria_monocytogenes_1639_v92	Listeria_monocytogenes_1639_v111	0.9462
Listeria_monocytogenes_1639_v91	Listeria_monocytogenes_1639_v111	0.9387
Listeria_monocytogenes_1639_v90	Listeria_monocytogenes_1639_v111	0.9375
Listeria_monocytogenes_1639_v8	Listeria_monocytogenes_1639_v111	0.9206
Listeria_monocytogenes_1639_v89	Listeria_monocytogenes_1639_v111	0.9482
Listeria_monocytogenes_1639_v88	Listeria_monocytogenes_1639_v111	0.9287
Listeria_monocytogenes_1639_v87	Listeria_monocytogenes_1639_v111	0.9282
Listeria_monocytogenes_1639_v86	Listeria_monocytogenes_1639_v111	0.9279
Listeria_monocytogenes_1639_v85	Listeria_monocytogenes_1639_v111	0.9379
Listeria_monocytogenes_1639_v84	Listeria_monocytogenes_1639_v111	0.933
Listeria_monocytogenes_1639_v83	Listeria_monocytogenes_1639_v111	0.9369
Listeria_monocytogenes_1639_v82	Listeria_monocytogenes_1639_v111	0.9366
Listeria_monocytogenes_1639_v81	Listeria_monocytogenes_1639_v111	0.9324
Listeria_monocytogenes_1639_v80	Listeria_monocytogenes_1639_v111	0.9421
Listeria_monocytogenes_1639_v7	Listeria_monocytogenes_1639_v111	0.9206
Listeria_monocytogenes_1639_v79	Listeria_monocytogenes_1639_v111	0.9307
Listeria_monocytogenes_1639_v78	Listeria_monocytogenes_1639_v111	0.9321
Listeria_monocytogenes_1639_v77	Listeria_monocytogenes_1639_v111	0.93
Listeria_monocytogenes_1639_v76	Listeria_monocytogenes_1639_v111	0.93
Listeria_monocytogenes_1639_v75	Listeria_monocytogenes_1639_v111	0.8986
Listeria_monocytogenes_1639_v74	Listeria_monocytogenes_1639_v111	0.929
Listeria_monocytogenes_1639_v73	Listeria_monocytogenes_1639_v111	0.9327
Listeria_monocytogenes_1639_v72	Listeria_monocytogenes_1639_v111	0.9357
Listeria_monocytogenes_1639_v71	Listeria_monocytogenes_1639_v111	0.9236
Listeria_monocytogenes_1639_v70	Listeria_monocytogenes_1639_v111	0.9299
Listeria_monocytogenes_1639_v6	Listeria_monocytogenes_1639_v111	0.92
Listeria_monocytogenes_1639_v69	Listeria_monocytogenes_1639_v111	0.9404
Listeria_monocytogenes_1639_v68	Listeria_monocytogenes_1639_v111	0.9377
Listeria_monocytogenes_1639_v67	Listeria_monocytogenes_1639_v111	0.9501
Listeria_monocytogenes_1639_v66	Listeria_monocytogenes_1639_v111	0.9384
Listeria_monocytogenes_1639_v65	Listeria_monocytogenes_1639_v111	0.9344
Listeria_monocytogenes_1639_v64	Listeria_monocytogenes_1639_v111	0.9367
Listeria_monocytogenes_1639_v63	Listeria_monocytogenes_1639_v111	0.9474
Listeria_monocytogenes_1639_v62	Listeria_monocytogenes_1639_v111	0.9372
Listeria_monocytogenes_1639_v61	Listeria_monocytogenes_1639_v111	0.9254
Listeria_monocytogenes_1639_v60	Listeria_monocytogenes_1639_v111	0.9369
Listeria_monocytogenes_1639_v5	Listeria_monocytogenes_1639_v111	0.9257
Listeria_monocytogenes_1639_v59	Listeria_monocytogenes_1639_v111	0.9391
Listeria_monocytogenes_1639_v58	Listeria_monocytogenes_1639_v111	0.9403
Listeria_monocytogenes_1639_v57	Listeria_monocytogenes_1639_v111	0.9335
Listeria_monocytogenes_1639_v56	Listeria_monocytogenes_1639_v111	0.9384
Listeria_monocytogenes_1639_v55	Listeria_monocytogenes_1639_v111	0.9385
Listeria_monocytogenes_1639_v54	Listeria_monocytogenes_1639_v111	0.9206
Listeria_monocytogenes_1639_v53	Listeria_monocytogenes_1639_v111	0.929
Listeria_monocytogenes_1639_v52	Listeria_monocytogenes_1639_v111	0.9356
Listeria_monocytogenes_1639_v51	Listeria_monocytogenes_1639_v111	0.9406
Listeria_monocytogenes_1639_v50	Listeria_monocytogenes_1639_v111	0.9223
Listeria_monocytogenes_1639_v4	Listeria_monocytogenes_1639_v111	0.9388
Listeria_monocytogenes_1639_v49	Listeria_monocytogenes_1639_v111	0.9397
Listeria_monocytogenes_1639_v48	Listeria_monocytogenes_1639_v111	0.9342
Listeria_monocytogenes_1639_v47	Listeria_monocytogenes_1639_v111	0.9227
Listeria_monocytogenes_1639_v46	Listeria_monocytogenes_1639_v111	0.9381
Listeria_monocytogenes_1639_v45	Listeria_monocytogenes_1639_v111	0.9216
Listeria_monocytogenes_1639_v44	Listeria_monocytogenes_1639_v111	0.9234
Listeria_monocytogenes_1639_v43	Listeria_monocytogenes_1639_v111	0.9234
Listeria_monocytogenes_1639_v42	Listeria_monocytogenes_1639_v111	0.9279
Listeria_monocytogenes_1639_v41	Listeria_monocytogenes_1639_v111	0.9207
Listeria_monocytogenes_1639_v40	Listeria_monocytogenes_1639_v111	0.9436
Listeria_monocytogenes_1639_v3	Listeria_monocytogenes_1639_v111	0.9395
Listeria_monocytogenes_1639_v39	Listeria_monocytogenes_1639_v111	0.9447
Listeria_monocytogenes_1639_v38	Listeria_monocytogenes_1639_v111	0.9251
Listeria_monocytogenes_1639_v37	Listeria_monocytogenes_1639_v111	0.9218
Listeria_monocytogenes_1639_v36	Listeria_monocytogenes_1639_v111	0.9474
Listeria_monocytogenes_1639_v35	Listeria_monocytogenes_1639_v111	0.9394
Listeria_monocytogenes_1639_v34	Listeria_monocytogenes_1639_v111	0.9326
Listeria_monocytogenes_1639_v33	Listeria_monocytogenes_1639_v111	0.9463
Listeria_monocytogenes_1639_v32	Listeria_monocytogenes_1639_v111	0.9204
Listeria_monocytogenes_1639_v31	Listeria_monocytogenes_1639_v111	0.933
Listeria_monocytogenes_1639_v30	Listeria_monocytogenes_1639_v111	0.9507
Listeria_monocytogenes_1639_v2	Listeria_monocytogenes_1639_v111	0.9415
Listeria_monocytogenes_1639_v29	Listeria_monocytogenes_1639_v111	0.9256

Listeria_monocytogenes_1639_v28	Listeria_monocytogenes_1639_v111	0.9211
Listeria_monocytogenes_1639_v27	Listeria_monocytogenes_1639_v111	0.9252
Listeria_monocytogenes_1639_v26	Listeria_monocytogenes_1639_v111	0.9252
Listeria_monocytogenes_1639_v25	Listeria_monocytogenes_1639_v111	0.9249
Listeria_monocytogenes_1639_v24	Listeria_monocytogenes_1639_v111	0.9289
Listeria_monocytogenes_1639_v23	Listeria_monocytogenes_1639_v111	0.9289
Listeria_monocytogenes_1639_v22	Listeria_monocytogenes_1639_v111	0.9392
Listeria_monocytogenes_1639_v21	Listeria_monocytogenes_1639_v111	0.9293
Listeria_monocytogenes_1639_v20	Listeria_monocytogenes_1639_v111	0.973
Listeria_monocytogenes_1639_v1	Listeria_monocytogenes_1639_v111	0.9313
Listeria_monocytogenes_1639_v19	Listeria_monocytogenes_1639_v111	0.9722
Listeria_monocytogenes_1639_v198	Listeria_monocytogenes_1639_v111	0.8953
Listeria_monocytogenes_1639_v197	Listeria_monocytogenes_1639_v111	0.9246
Listeria_monocytogenes_1639_v196	Listeria_monocytogenes_1639_v111	0.9238
Listeria_monocytogenes_1639_v195	Listeria_monocytogenes_1639_v111	0.9344
Listeria_monocytogenes_1639_v194	Listeria_monocytogenes_1639_v111	0.9333
Listeria_monocytogenes_1639_v193	Listeria_monocytogenes_1639_v111	0.9333
Listeria_monocytogenes_1639_v192	Listeria_monocytogenes_1639_v111	0.9003
Listeria_monocytogenes_1639_v191	Listeria_monocytogenes_1639_v111	0.9493
Listeria_monocytogenes_1639_v190	Listeria_monocytogenes_1639_v111	0.949
Listeria_monocytogenes_1639_v18	Listeria_monocytogenes_1639_v111	0.9276
Listeria_monocytogenes_1639_v189	Listeria_monocytogenes_1639_v111	0.9482
Listeria_monocytogenes_1639_v188	Listeria_monocytogenes_1639_v111	0.9481
Listeria_monocytogenes_1639_v187	Listeria_monocytogenes_1639_v111	0.9486
Listeria_monocytogenes_1639_v186	Listeria_monocytogenes_1639_v111	0.9493
Listeria_monocytogenes_1639_v185	Listeria_monocytogenes_1639_v111	0.949
Listeria_monocytogenes_1639_v184	Listeria_monocytogenes_1639_v111	0.9482
Listeria_monocytogenes_1639_v183	Listeria_monocytogenes_1639_v111	0.9436
Listeria_monocytogenes_1639_v182	Listeria_monocytogenes_1639_v111	0.9458
Listeria_monocytogenes_1639_v181	Listeria_monocytogenes_1639_v111	0.9184
Listeria_monocytogenes_1639_v180	Listeria_monocytogenes_1639_v111	0.9184
Listeria_monocytogenes_1639_v17	Listeria_monocytogenes_1639_v111	0.9276
Listeria_monocytogenes_1639_v179	Listeria_monocytogenes_1639_v111	0.9358
Listeria_monocytogenes_1639_v178	Listeria_monocytogenes_1639_v111	0.949
Listeria_monocytogenes_1639_v177	Listeria_monocytogenes_1639_v111	0.9539
Listeria_monocytogenes_1639_v176	Listeria_monocytogenes_1639_v111	0.9334
Listeria_monocytogenes_1639_v175	Listeria_monocytogenes_1639_v111	0.9403
Listeria_monocytogenes_1639_v174	Listeria_monocytogenes_1639_v111	0.9525
Listeria_monocytogenes_1639_v173	Listeria_monocytogenes_1639_v111	0.9697
Listeria_monocytogenes_1639_v172	Listeria_monocytogenes_1639_v111	0.9287
Listeria_monocytogenes_1639_v171	Listeria_monocytogenes_1639_v111	0.963
Listeria_monocytogenes_1639_v170	Listeria_monocytogenes_1639_v111	0.955
Listeria_monocytogenes_1639_v16	Listeria_monocytogenes_1639_v111	0.9256
Listeria_monocytogenes_1639_v169	Listeria_monocytogenes_1639_v111	0.9433
Listeria_monocytogenes_1639_v168	Listeria_monocytogenes_1639_v111	0.9441
Listeria_monocytogenes_1639_v167	Listeria_monocytogenes_1639_v111	0.9364
Listeria_monocytogenes_1639_v166	Listeria_monocytogenes_1639_v111	0.9445
Listeria_monocytogenes_1639_v165	Listeria_monocytogenes_1639_v111	0.9433
Listeria_monocytogenes_1639_v164	Listeria_monocytogenes_1639_v111	0.9542
Listeria_monocytogenes_1639_v163	Listeria_monocytogenes_1639_v111	0.9699
Listeria_monocytogenes_1639_v162	Listeria_monocytogenes_1639_v111	0.9542
Listeria_monocytogenes_1639_v161	Listeria_monocytogenes_1639_v111	0.9553
Listeria_monocytogenes_1639_v160	Listeria_monocytogenes_1639_v111	0.9554
Listeria_monocytogenes_1639_v15	Listeria_monocytogenes_1639_v111	0.9373
Listeria_monocytogenes_1639_v159	Listeria_monocytogenes_1639_v111	0.9539
Listeria_monocytogenes_1639_v158	Listeria_monocytogenes_1639_v111	0.955
Listeria_monocytogenes_1639_v157	Listeria_monocytogenes_1639_v111	0.955
Listeria_monocytogenes_1639_v156	Listeria_monocytogenes_1639_v111	0.9303
Listeria_monocytogenes_1639_v155	Listeria_monocytogenes_1639_v111	0.9396
Listeria_monocytogenes_1639_v154	Listeria_monocytogenes_1639_v111	0.9385
Listeria_monocytogenes_1639_v153	Listeria_monocytogenes_1639_v111	0.9392
Listeria_monocytogenes_1639_v152	Listeria_monocytogenes_1639_v111	0.9543
Listeria_monocytogenes_1639_v151	Listeria_monocytogenes_1639_v111	0.9543
Listeria_monocytogenes_1639_v150	Listeria_monocytogenes_1639_v111	0.9423
Listeria_monocytogenes_1639_v14	Listeria_monocytogenes_1639_v111	0.9405
Listeria_monocytogenes_1639_v149	Listeria_monocytogenes_1639_v111	0.9409
Listeria_monocytogenes_1639_v148	Listeria_monocytogenes_1639_v111	0.9532
Listeria_monocytogenes_1639_v147	Listeria_monocytogenes_1639_v111	0.9415
Listeria_monocytogenes_1639_v146	Listeria_monocytogenes_1639_v111	0.9499
Listeria_monocytogenes_1639_v145	Listeria_monocytogenes_1639_v111	0.9412
Listeria_monocytogenes_1639_v144	Listeria_monocytogenes_1639_v111	0.9569
Listeria_monocytogenes_1639_v143	Listeria_monocytogenes_1639_v111	0.9484
Listeria_monocytogenes_1639_v142	Listeria_monocytogenes_1639_v111	0.9573
Listeria_monocytogenes_1639_v141	Listeria_monocytogenes_1639_v111	0.9641
Listeria_monocytogenes_1639_v140	Listeria_monocytogenes_1639_v111	0.9576
Listeria_monocytogenes_1639_v13	Listeria_monocytogenes_1639_v111	0.9377
Listeria_monocytogenes_1639_v139	Listeria_monocytogenes_1639_v111	0.9516
Listeria_monocytogenes_1639_v138	Listeria_monocytogenes_1639_v111	0.9599
Listeria_monocytogenes_1639_v137	Listeria_monocytogenes_1639_v111	0.9599
Listeria_monocytogenes_1639_v136	Listeria_monocytogenes_1639_v111	0.9343
Listeria_monocytogenes_1639_v135	Listeria_monocytogenes_1639_v111	0.9067
Listeria_monocytogenes_1639_v134	Listeria_monocytogenes_1639_v111	0.9487
Listeria_monocytogenes_1639_v133	Listeria_monocytogenes_1639_v111	0.9427
Listeria_monocytogenes_1639_v132	Listeria_monocytogenes_1639_v111	0.9146
Listeria_monocytogenes_1639_v131	Listeria_monocytogenes_1639_v111	0.9442
Listeria_monocytogenes_1639_v130	Listeria_monocytogenes_1639_v111	0.9439
Listeria_monocytogenes_1639_v12	Listeria_monocytogenes_1639_v111	0.9283
Listeria_monocytogenes_1639_v129	Listeria_monocytogenes_1639_v111	0.9561
Listeria_monocytogenes_1639_v128	Listeria_monocytogenes_1639_v111	0.96
Listeria_monocytogenes_1639_v127	Listeria_monocytogenes_1639_v111	0.9388
Listeria_monocytogenes_1639_v126	Listeria_monocytogenes_1639_v111	0.9585

Listeria_monocytogenes_1639_v125	Listeria_monocytogenes_1639_v111	0.9389
Listeria_monocytogenes_1639_v124	Listeria_monocytogenes_1639_v111	0.9299
Listeria_monocytogenes_1639_v123	Listeria_monocytogenes_1639_v111	0.9559
Listeria_monocytogenes_1639_v122	Listeria_monocytogenes_1639_v111	0.9562
Listeria_monocytogenes_1639_v121	Listeria_monocytogenes_1639_v111	0.9562
Listeria_monocytogenes_1639_v120	Listeria_monocytogenes_1639_v111	0.9559
Listeria_monocytogenes_1639_v11	Listeria_monocytogenes_1639_v111	0.9325
Listeria_monocytogenes_1639_v119	Listeria_monocytogenes_1639_v111	0.9562
Listeria_monocytogenes_1639_v118	Listeria_monocytogenes_1639_v111	0.9562
Listeria_monocytogenes_1639_v117	Listeria_monocytogenes_1639_v111	0.9392
Listeria_monocytogenes_1639_v116	Listeria_monocytogenes_1639_v111	0.9485
Listeria_monocytogenes_1639_v115	Listeria_monocytogenes_1639_v111	0.9485
Listeria_monocytogenes_1639_v114	Listeria_monocytogenes_1639_v111	0.9342
Listeria_monocytogenes_1639_v113	Listeria_monocytogenes_1639_v111	0.953
Listeria_monocytogenes_1639_v112	Listeria_monocytogenes_1639_v111	0.9449
Listeria_monocytogenes_1639_v111	Listeria_monocytogenes_1639_v111	0.0682
Listeria_welshimeri_1643	Listeria_monocytogenes_1639_v112	0.8804
Listeria_welshimeri_serovar_6b_str_SLCC5334_386043	Listeria_monocytogenes_1639_v112	0.8801
Listeria_seeligeri_serovar_1_2b_str_SLCC3954_683837	Listeria_monocytogenes_1639_v112	0.8802
Listeria_seeligeri_FSL_S4-171_702452	Listeria_monocytogenes_1639_v112	0.8339
Listeria_seeligeri_FSL_N1-067_702453	Listeria_monocytogenes_1639_v112	0.838
Listeria_seeligeri_1640_v1	Listeria_monocytogenes_1639_v112	0.8905
Listeria_seeligeri_1640	Listeria_monocytogenes_1639_v112	0.8849
Listeria_monocytogenes_WSLC1042_1457188	Listeria_monocytogenes_1639_v112	0.9788
Listeria_monocytogenes_WSLC1001_1457187	Listeria_monocytogenes_1639_v112	0.9264
Listeria_monocytogenes_SLCC7179_879090	Listeria_monocytogenes_1639_v112	0.9357
Listeria_monocytogenes_SLCC5850_882096	Listeria_monocytogenes_1639_v112	0.9307
Listeria_monocytogenes_SLCC2755_932919	Listeria_monocytogenes_1639_v112	0.9402
Listeria_monocytogenes_SLCC2540_879089	Listeria_monocytogenes_1639_v112	0.946
Listeria_monocytogenes_SLCC2479_882020	Listeria_monocytogenes_1639_v112	0.9318
Listeria_monocytogenes_SLCC2378_879088	Listeria_monocytogenes_1639_v112	0.9795
Listeria_monocytogenes_SLCC2376_882097	Listeria_monocytogenes_1639_v112	0.9359
Listeria_monocytogenes_SLCC2372_932920	Listeria_monocytogenes_1639_v112	0.9249
Listeria_monocytogenes_serotype_4b_str_LL195_1230340	Listeria_monocytogenes_1639_v112	0.9859
Listeria_monocytogenes_serotype_4b_str_LIS0087_1134669	Listeria_monocytogenes_1639_v112	0.9098
Listeria_monocytogenes_serotype_4b_str_F2365_265669	Listeria_monocytogenes_1639_v112	0.9859
Listeria_monocytogenes_serotype_4b_str_CLIP_80459_568819	Listeria_monocytogenes_1639_v112	0.9671
Listeria_monocytogenes_serotype_4b_str_81-0861_1196161	Listeria_monocytogenes_1639_v112	0.9786
Listeria_monocytogenes_serotype_4b_str_81-0592_1196160	Listeria_monocytogenes_1639_v112	0.9797
Listeria_monocytogenes_serotype_4b_str_81-0558_1196159	Listeria_monocytogenes_1639_v112	0.9805
Listeria_monocytogenes_serotype_4b_str_10-0809_1196162	Listeria_monocytogenes_1639_v112	0.9801
Listeria_monocytogenes_serotype_4b_str_02-6680_1196175	Listeria_monocytogenes_1639_v112	0.968
Listeria_monocytogenes_serotype_4b_str_02-6679_1196174	Listeria_monocytogenes_1639_v112	0.968
Listeria_monocytogenes_serotype_4b_str_02-1792_1196173	Listeria_monocytogenes_1639_v112	0.976
Listeria_monocytogenes_serotype_4b_str_02-1289_1196172	Listeria_monocytogenes_1639_v112	0.976
Listeria_monocytogenes_serotype_4b_str_02-1103_1196171	Listeria_monocytogenes_1639_v112	0.976
Listeria_monocytogenes_serotype_3c_str_10-5027_1196195	Listeria_monocytogenes_1639_v112	0.9318
Listeria_monocytogenes_serotype_1_2c_str_10-5026_1196194	Listeria_monocytogenes_1639_v112	0.9329
Listeria_monocytogenes_serotype_1_2c_str_10-5025_1196193	Listeria_monocytogenes_1639_v112	0.9329
Listeria_monocytogenes_serotype_1_2b_str_10-0811_1196164	Listeria_monocytogenes_1639_v112	0.9433
Listeria_monocytogenes_serotype_1_2b_str_10-0810_1196163	Listeria_monocytogenes_1639_v112	0.9433
Listeria_monocytogenes_serotype_1_2a_str_99-6370_1196179	Listeria_monocytogenes_1639_v112	0.9197
Listeria_monocytogenes_serotype_1_2a_str_98-2035_1196178	Listeria_monocytogenes_1639_v112	0.9197
Listeria_monocytogenes_serotype_1_2a_str_95-0093_1196177	Listeria_monocytogenes_1639_v112	0.9245
Listeria_monocytogenes_serotype_1_2a_str_88-0478_1196176	Listeria_monocytogenes_1639_v112	0.9244
Listeria_monocytogenes_serotype_1_2a_str_10-5024_1196192	Listeria_monocytogenes_1639_v112	0.9245
Listeria_monocytogenes_serotype_1_2a_str_10-4758_1196168	Listeria_monocytogenes_1639_v112	0.9327
Listeria_monocytogenes_serotype_1_2a_str_10-4754_1196167	Listeria_monocytogenes_1639_v112	0.933
Listeria_monocytogenes_serotype_1_2a_str_10-1321_1196191	Listeria_monocytogenes_1639_v112	0.9245
Listeria_monocytogenes_serotype_1_2a_str_10-1047_1196190	Listeria_monocytogenes_1639_v112	0.9255
Listeria_monocytogenes_serotype_1_2a_str_10-1046_1196189	Listeria_monocytogenes_1639_v112	0.9255
Listeria_monocytogenes_serotype_1_2a_str_10-0934_1196170	Listeria_monocytogenes_1639_v112	0.9383
Listeria_monocytogenes_serotype_1_2a_str_10-0933_1196169	Listeria_monocytogenes_1639_v112	0.9383
Listeria_monocytogenes_serotype_1_2a_str_10-0815_1196188	Listeria_monocytogenes_1639_v112	0.9207
Listeria_monocytogenes_serotype_1_2a_str_10-0814_1196187	Listeria_monocytogenes_1639_v112	0.9248
Listeria_monocytogenes_serotype_1_2a_str_10-0813_1196166	Listeria_monocytogenes_1639_v112	0.9282
Listeria_monocytogenes_serotype_1_2a_str_10-0812_1196165	Listeria_monocytogenes_1639_v112	0.9282
Listeria_monocytogenes_serotype_1_2a_str_08-7669_1196186	Listeria_monocytogenes_1639_v112	0.9331
Listeria_monocytogenes_serotype_1_2a_str_08-7374_1196185	Listeria_monocytogenes_1639_v112	0.9245
Listeria_monocytogenes_serotype_1_2a_str_08-6997_1196184	Listeria_monocytogenes_1639_v112	0.9207
Listeria_monocytogenes_serotype_1_2a_str_08-6569_1196183	Listeria_monocytogenes_1639_v112	0.9207
Listeria_monocytogenes_serotype_1_2a_str_08-6056_1196182	Listeria_monocytogenes_1639_v112	0.9207
Listeria_monocytogenes_serotype_1_2a_str_04-5457_1196181	Listeria_monocytogenes_1639_v112	0.9245
Listeria_monocytogenes_serotype_1_2a_str_02-5993_1196180	Listeria_monocytogenes_1639_v112	0.9245
Listeria_monocytogenes_R479a_1437838	Listeria_monocytogenes_1639_v112	0.9238
Listeria_monocytogenes_N53-1_1234142	Listeria_monocytogenes_1639_v112	0.8911
Listeria_monocytogenes_M7_1030009	Listeria_monocytogenes_1639_v112	0.9101
Listeria_monocytogenes_LO28_393132	Listeria_monocytogenes_1639_v112	0.927
Listeria_monocytogenes_L99_563174	Listeria_monocytogenes_1639_v112	0.9104
Listeria_monocytogenes_L312_882094	Listeria_monocytogenes_1639_v112	0.9675
Listeria_monocytogenes_J1816_930781	Listeria_monocytogenes_1639_v112	0.9581
Listeria_monocytogenes_J1-220_930782	Listeria_monocytogenes_1639_v112	0.9527
Listeria_monocytogenes_J0161_393130	Listeria_monocytogenes_1639_v112	0.933
Listeria_monocytogenes_HCC23_552536	Listeria_monocytogenes_1639_v112	0.9097
Listeria_monocytogenes_FSL_R2-561_393126	Listeria_monocytogenes_1639_v112	0.9308
Listeria_monocytogenes_FSL_J1-208_393119	Listeria_monocytogenes_1639_v112	0.9151
Listeria_monocytogenes_FSL_J1-175_393118	Listeria_monocytogenes_1639_v112	0.9488
Listeria_monocytogenes_Finland_1998_393127	Listeria_monocytogenes_1639_v112	0.9382
Listeria_monocytogenes_EGD-e_169963_v1	Listeria_monocytogenes_1639_v112	0.9361
Listeria_monocytogenes_EGD-e_169963	Listeria_monocytogenes_1639_v112	0.9361
Listeria_monocytogenes_EGD_1334565	Listeria_monocytogenes_1639_v112	0.9314

Listeria_monocytogenes_CFSAN002349_1299895	Listeria_monocytogenes_1639_v112	0.9669
Listeria_monocytogenes_ATCC_19117_882095_v1	Listeria_monocytogenes_1639_v112	0.9661
Listeria_monocytogenes_ATCC_19117_882095	Listeria_monocytogenes_1639_v112	0.9658
Listeria_monocytogenes_6179_1288295	Listeria_monocytogenes_1639_v112	0.9192
Listeria_monocytogenes_1639_v9	Listeria_monocytogenes_1639_v112	0.9305
Listeria_monocytogenes_1639_v99	Listeria_monocytogenes_1639_v112	0.9619
Listeria_monocytogenes_1639_v98	Listeria_monocytogenes_1639_v112	0.9311
Listeria_monocytogenes_1639_v97	Listeria_monocytogenes_1639_v112	0.9732
Listeria_monocytogenes_1639_v96	Listeria_monocytogenes_1639_v112	0.9593
Listeria_monocytogenes_1639_v95	Listeria_monocytogenes_1639_v112	0.9593
Listeria_monocytogenes_1639_v94	Listeria_monocytogenes_1639_v112	0.9597
Listeria_monocytogenes_1639_v93	Listeria_monocytogenes_1639_v112	0.9199
Listeria_monocytogenes_1639_v92	Listeria_monocytogenes_1639_v112	0.9608
Listeria_monocytogenes_1639_v91	Listeria_monocytogenes_1639_v112	0.9742
Listeria_monocytogenes_1639_v90	Listeria_monocytogenes_1639_v112	0.9311
Listeria_monocytogenes_1639_v8	Listeria_monocytogenes_1639_v112	0.914
Listeria_monocytogenes_1639_v89	Listeria_monocytogenes_1639_v112	0.9633
Listeria_monocytogenes_1639_v88	Listeria_monocytogenes_1639_v112	0.932
Listeria_monocytogenes_1639_v87	Listeria_monocytogenes_1639_v112	0.9229
Listeria_monocytogenes_1639_v86	Listeria_monocytogenes_1639_v112	0.9226
Listeria_monocytogenes_1639_v85	Listeria_monocytogenes_1639_v112	0.9749
Listeria_monocytogenes_1639_v84	Listeria_monocytogenes_1639_v112	0.9375
Listeria_monocytogenes_1639_v83	Listeria_monocytogenes_1639_v112	0.9613
Listeria_monocytogenes_1639_v82	Listeria_monocytogenes_1639_v112	0.9609
Listeria_monocytogenes_1639_v81	Listeria_monocytogenes_1639_v112	0.9328
Listeria_monocytogenes_1639_v80	Listeria_monocytogenes_1639_v112	0.9443
Listeria_monocytogenes_1639_v7	Listeria_monocytogenes_1639_v112	0.914
Listeria_monocytogenes_1639_v79	Listeria_monocytogenes_1639_v112	0.9299
Listeria_monocytogenes_1639_v78	Listeria_monocytogenes_1639_v112	0.9313
Listeria_monocytogenes_1639_v77	Listeria_monocytogenes_1639_v112	0.9355
Listeria_monocytogenes_1639_v76	Listeria_monocytogenes_1639_v112	0.9258
Listeria_monocytogenes_1639_v75	Listeria_monocytogenes_1639_v112	0.9104
Listeria_monocytogenes_1639_v74	Listeria_monocytogenes_1639_v112	0.9203
Listeria_monocytogenes_1639_v73	Listeria_monocytogenes_1639_v112	0.9215
Listeria_monocytogenes_1639_v72	Listeria_monocytogenes_1639_v112	0.9594
Listeria_monocytogenes_1639_v71	Listeria_monocytogenes_1639_v112	0.9165
Listeria_monocytogenes_1639_v70	Listeria_monocytogenes_1639_v112	0.9302
Listeria_monocytogenes_1639_v6	Listeria_monocytogenes_1639_v112	0.9136
Listeria_monocytogenes_1639_v69	Listeria_monocytogenes_1639_v112	0.9797
Listeria_monocytogenes_1639_v68	Listeria_monocytogenes_1639_v112	0.9293
Listeria_monocytogenes_1639_v67	Listeria_monocytogenes_1639_v112	0.9504
Listeria_monocytogenes_1639_v66	Listeria_monocytogenes_1639_v112	0.9301
Listeria_monocytogenes_1639_v65	Listeria_monocytogenes_1639_v112	0.9235
Listeria_monocytogenes_1639_v64	Listeria_monocytogenes_1639_v112	0.9246
Listeria_monocytogenes_1639_v63	Listeria_monocytogenes_1639_v112	0.9462
Listeria_monocytogenes_1639_v62	Listeria_monocytogenes_1639_v112	0.9274
Listeria_monocytogenes_1639_v61	Listeria_monocytogenes_1639_v112	0.9272
Listeria_monocytogenes_1639_v60	Listeria_monocytogenes_1639_v112	0.9357
Listeria_monocytogenes_1639_v5	Listeria_monocytogenes_1639_v112	0.9091
Listeria_monocytogenes_1639_v59	Listeria_monocytogenes_1639_v112	0.959
Listeria_monocytogenes_1639_v58	Listeria_monocytogenes_1639_v112	0.9692
Listeria_monocytogenes_1639_v57	Listeria_monocytogenes_1639_v112	0.9561
Listeria_monocytogenes_1639_v56	Listeria_monocytogenes_1639_v112	0.9688
Listeria_monocytogenes_1639_v55	Listeria_monocytogenes_1639_v112	0.9544
Listeria_monocytogenes_1639_v54	Listeria_monocytogenes_1639_v112	0.93
Listeria_monocytogenes_1639_v53	Listeria_monocytogenes_1639_v112	0.9259
Listeria_monocytogenes_1639_v52	Listeria_monocytogenes_1639_v112	0.9595
Listeria_monocytogenes_1639_v51	Listeria_monocytogenes_1639_v112	0.9589
Listeria_monocytogenes_1639_v50	Listeria_monocytogenes_1639_v112	0.9237
Listeria_monocytogenes_1639_v4	Listeria_monocytogenes_1639_v112	0.9216
Listeria_monocytogenes_1639_v49	Listeria_monocytogenes_1639_v112	0.9719
Listeria_monocytogenes_1639_v48	Listeria_monocytogenes_1639_v112	0.9266
Listeria_monocytogenes_1639_v47	Listeria_monocytogenes_1639_v112	0.9252
Listeria_monocytogenes_1639_v46	Listeria_monocytogenes_1639_v112	0.9332
Listeria_monocytogenes_1639_v45	Listeria_monocytogenes_1639_v112	0.916
Listeria_monocytogenes_1639_v44	Listeria_monocytogenes_1639_v112	0.9245
Listeria_monocytogenes_1639_v43	Listeria_monocytogenes_1639_v112	0.9245
Listeria_monocytogenes_1639_v42	Listeria_monocytogenes_1639_v112	0.9212
Listeria_monocytogenes_1639_v41	Listeria_monocytogenes_1639_v112	0.902
Listeria_monocytogenes_1639_v40	Listeria_monocytogenes_1639_v112	0.9233
Listeria_monocytogenes_1639_v3	Listeria_monocytogenes_1639_v112	0.922
Listeria_monocytogenes_1639_v39	Listeria_monocytogenes_1639_v112	0.9477
Listeria_monocytogenes_1639_v38	Listeria_monocytogenes_1639_v112	0.9198
Listeria_monocytogenes_1639_v37	Listeria_monocytogenes_1639_v112	0.9161
Listeria_monocytogenes_1639_v36	Listeria_monocytogenes_1639_v112	0.914
Listeria_monocytogenes_1639_v35	Listeria_monocytogenes_1639_v112	0.9243
Listeria_monocytogenes_1639_v34	Listeria_monocytogenes_1639_v112	0.9262
Listeria_monocytogenes_1639_v33	Listeria_monocytogenes_1639_v112	0.9433
Listeria_monocytogenes_1639_v32	Listeria_monocytogenes_1639_v112	0.9199
Listeria_monocytogenes_1639_v31	Listeria_monocytogenes_1639_v112	0.9266
Listeria_monocytogenes_1639_v30	Listeria_monocytogenes_1639_v112	0.9361
Listeria_monocytogenes_1639_v2	Listeria_monocytogenes_1639_v112	0.9134
Listeria_monocytogenes_1639_v29	Listeria_monocytogenes_1639_v112	0.9181
Listeria_monocytogenes_1639_v28	Listeria_monocytogenes_1639_v112	0.9236
Listeria_monocytogenes_1639_v27	Listeria_monocytogenes_1639_v112	0.909
Listeria_monocytogenes_1639_v26	Listeria_monocytogenes_1639_v112	0.9087
Listeria_monocytogenes_1639_v25	Listeria_monocytogenes_1639_v112	0.9087
Listeria_monocytogenes_1639_v24	Listeria_monocytogenes_1639_v112	0.9549
Listeria_monocytogenes_1639_v23	Listeria_monocytogenes_1639_v112	0.9546
Listeria_monocytogenes_1639_v22	Listeria_monocytogenes_1639_v112	0.9499
Listeria_monocytogenes_1639_v21	Listeria_monocytogenes_1639_v112	0.9542

Listeria_monocytogenes_1639_v20	Listeria_monocytogenes_1639_v112	0.9399
Listeria_monocytogenes_1639_v1	Listeria_monocytogenes_1639_v112	0.914
Listeria_monocytogenes_1639_v19	Listeria_monocytogenes_1639_v112	0.9396
Listeria_monocytogenes_1639_v198	Listeria_monocytogenes_1639_v112	0.8938
Listeria_monocytogenes_1639_v197	Listeria_monocytogenes_1639_v112	0.9238
Listeria_monocytogenes_1639_v196	Listeria_monocytogenes_1639_v112	0.9141
Listeria_monocytogenes_1639_v195	Listeria_monocytogenes_1639_v112	0.9385
Listeria_monocytogenes_1639_v194	Listeria_monocytogenes_1639_v112	0.9363
Listeria_monocytogenes_1639_v193	Listeria_monocytogenes_1639_v112	0.9363
Listeria_monocytogenes_1639_v192	Listeria_monocytogenes_1639_v112	0.9062
Listeria_monocytogenes_1639_v191	Listeria_monocytogenes_1639_v112	0.9419
Listeria_monocytogenes_1639_v190	Listeria_monocytogenes_1639_v112	0.9382
Listeria_monocytogenes_1639_v18	Listeria_monocytogenes_1639_v112	0.9512
Listeria_monocytogenes_1639_v189	Listeria_monocytogenes_1639_v112	0.9374
Listeria_monocytogenes_1639_v188	Listeria_monocytogenes_1639_v112	0.9396
Listeria_monocytogenes_1639_v187	Listeria_monocytogenes_1639_v112	0.9381
Listeria_monocytogenes_1639_v186	Listeria_monocytogenes_1639_v112	0.9385
Listeria_monocytogenes_1639_v185	Listeria_monocytogenes_1639_v112	0.9385
Listeria_monocytogenes_1639_v184	Listeria_monocytogenes_1639_v112	0.9374
Listeria_monocytogenes_1639_v183	Listeria_monocytogenes_1639_v112	0.9381
Listeria_monocytogenes_1639_v182	Listeria_monocytogenes_1639_v112	0.94
Listeria_monocytogenes_1639_v181	Listeria_monocytogenes_1639_v112	0.927
Listeria_monocytogenes_1639_v180	Listeria_monocytogenes_1639_v112	0.927
Listeria_monocytogenes_1639_v17	Listeria_monocytogenes_1639_v112	0.9516
Listeria_monocytogenes_1639_v179	Listeria_monocytogenes_1639_v112	0.9327
Listeria_monocytogenes_1639_v178	Listeria_monocytogenes_1639_v112	0.9401
Listeria_monocytogenes_1639_v177	Listeria_monocytogenes_1639_v112	0.9646
Listeria_monocytogenes_1639_v176	Listeria_monocytogenes_1639_v112	0.9349
Listeria_monocytogenes_1639_v175	Listeria_monocytogenes_1639_v112	0.933
Listeria_monocytogenes_1639_v174	Listeria_monocytogenes_1639_v112	0.941
Listeria_monocytogenes_1639_v173	Listeria_monocytogenes_1639_v112	0.9628
Listeria_monocytogenes_1639_v172	Listeria_monocytogenes_1639_v112	0.9367
Listeria_monocytogenes_1639_v171	Listeria_monocytogenes_1639_v112	0.9251
Listeria_monocytogenes_1639_v170	Listeria_monocytogenes_1639_v112	0.9329
Listeria_monocytogenes_1639_v16	Listeria_monocytogenes_1639_v112	0.9117
Listeria_monocytogenes_1639_v169	Listeria_monocytogenes_1639_v112	0.9352
Listeria_monocytogenes_1639_v168	Listeria_monocytogenes_1639_v112	0.9349
Listeria_monocytogenes_1639_v167	Listeria_monocytogenes_1639_v112	0.9348
Listeria_monocytogenes_1639_v166	Listeria_monocytogenes_1639_v112	0.9356
Listeria_monocytogenes_1639_v165	Listeria_monocytogenes_1639_v112	0.9349
Listeria_monocytogenes_1639_v164	Listeria_monocytogenes_1639_v112	0.9855
Listeria_monocytogenes_1639_v163	Listeria_monocytogenes_1639_v112	0.9553
Listeria_monocytogenes_1639_v162	Listeria_monocytogenes_1639_v112	0.9855
Listeria_monocytogenes_1639_v161	Listeria_monocytogenes_1639_v112	0.9866
Listeria_monocytogenes_1639_v160	Listeria_monocytogenes_1639_v112	0.987
Listeria_monocytogenes_1639_v15	Listeria_monocytogenes_1639_v112	0.9277
Listeria_monocytogenes_1639_v159	Listeria_monocytogenes_1639_v112	0.9642
Listeria_monocytogenes_1639_v158	Listeria_monocytogenes_1639_v112	0.9859
Listeria_monocytogenes_1639_v157	Listeria_monocytogenes_1639_v112	0.9859
Listeria_monocytogenes_1639_v156	Listeria_monocytogenes_1639_v112	0.9224
Listeria_monocytogenes_1639_v155	Listeria_monocytogenes_1639_v112	0.9403
Listeria_monocytogenes_1639_v154	Listeria_monocytogenes_1639_v112	0.9328
Listeria_monocytogenes_1639_v153	Listeria_monocytogenes_1639_v112	0.9365
Listeria_monocytogenes_1639_v152	Listeria_monocytogenes_1639_v112	0.9642
Listeria_monocytogenes_1639_v151	Listeria_monocytogenes_1639_v112	0.9642
Listeria_monocytogenes_1639_v150	Listeria_monocytogenes_1639_v112	0.9312
Listeria_monocytogenes_1639_v14	Listeria_monocytogenes_1639_v112	0.9357
Listeria_monocytogenes_1639_v149	Listeria_monocytogenes_1639_v112	0.9317
Listeria_monocytogenes_1639_v148	Listeria_monocytogenes_1639_v112	0.9631
Listeria_monocytogenes_1639_v147	Listeria_monocytogenes_1639_v112	0.9505
Listeria_monocytogenes_1639_v146	Listeria_monocytogenes_1639_v112	0.9426
Listeria_monocytogenes_1639_v145	Listeria_monocytogenes_1639_v112	0.9377
Listeria_monocytogenes_1639_v144	Listeria_monocytogenes_1639_v112	0.9672
Listeria_monocytogenes_1639_v143	Listeria_monocytogenes_1639_v112	0.9434
Listeria_monocytogenes_1639_v142	Listeria_monocytogenes_1639_v112	0.9675
Listeria_monocytogenes_1639_v141	Listeria_monocytogenes_1639_v112	0.9614
Listeria_monocytogenes_1639_v140	Listeria_monocytogenes_1639_v112	0.9679
Listeria_monocytogenes_1639_v13	Listeria_monocytogenes_1639_v112	0.9277
Listeria_monocytogenes_1639_v139	Listeria_monocytogenes_1639_v112	0.9634
Listeria_monocytogenes_1639_v138	Listeria_monocytogenes_1639_v112	0.9706
Listeria_monocytogenes_1639_v137	Listeria_monocytogenes_1639_v112	0.9702
Listeria_monocytogenes_1639_v136	Listeria_monocytogenes_1639_v112	0.9312
Listeria_monocytogenes_1639_v135	Listeria_monocytogenes_1639_v112	0.9213
Listeria_monocytogenes_1639_v134	Listeria_monocytogenes_1639_v112	0.9795
Listeria_monocytogenes_1639_v133	Listeria_monocytogenes_1639_v112	0.9385
Listeria_monocytogenes_1639_v132	Listeria_monocytogenes_1639_v112	0.9179
Listeria_monocytogenes_1639_v131	Listeria_monocytogenes_1639_v112	0.96
Listeria_monocytogenes_1639_v130	Listeria_monocytogenes_1639_v112	0.9236
Listeria_monocytogenes_1639_v12	Listeria_monocytogenes_1639_v112	0.9121
Listeria_monocytogenes_1639_v129	Listeria_monocytogenes_1639_v112	0.9564
Listeria_monocytogenes_1639_v128	Listeria_monocytogenes_1639_v112	0.9573
Listeria_monocytogenes_1639_v127	Listeria_monocytogenes_1639_v112	0.9339
Listeria_monocytogenes_1639_v126	Listeria_monocytogenes_1639_v112	0.9554
Listeria_monocytogenes_1639_v125	Listeria_monocytogenes_1639_v112	0.9366
Listeria_monocytogenes_1639_v124	Listeria_monocytogenes_1639_v112	0.9355
Listeria_monocytogenes_1639_v123	Listeria_monocytogenes_1639_v112	0.9669
Listeria_monocytogenes_1639_v122	Listeria_monocytogenes_1639_v112	0.9669
Listeria_monocytogenes_1639_v121	Listeria_monocytogenes_1639_v112	0.9669
Listeria_monocytogenes_1639_v120	Listeria_monocytogenes_1639_v112	0.9672
Listeria_monocytogenes_1639_v11	Listeria_monocytogenes_1639_v112	0.9163
Listeria_monocytogenes_1639_v119	Listeria_monocytogenes_1639_v112	0.9669

Listeria_monocytogenes_1639_v118	Listeria_monocytogenes_1639_v112	0.9669
Listeria_monocytogenes_1639_v117	Listeria_monocytogenes_1639_v112	0.9323
Listeria_monocytogenes_1639_v116	Listeria_monocytogenes_1639_v112	0.9792
Listeria_monocytogenes_1639_v115	Listeria_monocytogenes_1639_v112	0.9795
Listeria_monocytogenes_1639_v114	Listeria_monocytogenes_1639_v112	0.9292
Listeria_monocytogenes_1639_v113	Listeria_monocytogenes_1639_v112	0.9526
Listeria_monocytogenes_1639_v112	Listeria_monocytogenes_1639_v112	0.0653
Listeria_welshimeri_1643	Listeria_monocytogenes_1639_v113	0.8813
Listeria_welshimeri_serovar_6b_str_SLCC5334_386043	Listeria_monocytogenes_1639_v113	0.8806
Listeria_seeligeri_serovar_1_2b_str_SLCC3954_683837	Listeria_monocytogenes_1639_v113	0.8882
Listeria_seeligeri_FSL_S4-171_702452	Listeria_monocytogenes_1639_v113	0.8243
Listeria_seeligeri_FSL_N1-067_702453	Listeria_monocytogenes_1639_v113	0.8356
Listeria_seeligeri_1640_v1	Listeria_monocytogenes_1639_v113	0.8912
Listeria_seeligeri_1640	Listeria_monocytogenes_1639_v113	0.8831
Listeria_monocytogenes_WSLC1042_1457188	Listeria_monocytogenes_1639_v113	0.9532
Listeria_monocytogenes_WSLC1001_1457187	Listeria_monocytogenes_1639_v113	0.9518
Listeria_monocytogenes_SLCC7179_879090	Listeria_monocytogenes_1639_v113	0.9445
Listeria_monocytogenes_SLCC5850_882096	Listeria_monocytogenes_1639_v113	0.9543
Listeria_monocytogenes_SLCC2755_932919	Listeria_monocytogenes_1639_v113	0.9471
Listeria_monocytogenes_SLCC2540_879089	Listeria_monocytogenes_1639_v113	0.9708
Listeria_monocytogenes_SLCC2479_882020	Listeria_monocytogenes_1639_v113	0.9488
Listeria_monocytogenes_SLCC2378_879088	Listeria_monocytogenes_1639_v113	0.9546
Listeria_monocytogenes_SLCC2376_882097	Listeria_monocytogenes_1639_v113	0.943
Listeria_monocytogenes_SLCC2372_932920	Listeria_monocytogenes_1639_v113	0.9414
Listeria_monocytogenes_serotype_4b_str_LL195_1230340	Listeria_monocytogenes_1639_v113	0.9609
Listeria_monocytogenes_serotype_4b_str_LIS0087_1134669	Listeria_monocytogenes_1639_v113	0.932
Listeria_monocytogenes_serotype_4b_str_F2365_265669	Listeria_monocytogenes_1639_v113	0.9609
Listeria_monocytogenes_serotype_4b_str_CLIP_80459_568819	Listeria_monocytogenes_1639_v113	0.9627
Listeria_monocytogenes_serotype_4b_str_81-0861_1196161	Listeria_monocytogenes_1639_v113	0.9442
Listeria_monocytogenes_serotype_4b_str_81-0592_1196160	Listeria_monocytogenes_1639_v113	0.9453
Listeria_monocytogenes_serotype_4b_str_81-0558_1196159	Listeria_monocytogenes_1639_v113	0.9464
Listeria_monocytogenes_serotype_4b_str_10-0809_1196162	Listeria_monocytogenes_1639_v113	0.9457
Listeria_monocytogenes_serotype_4b_str_02-6680_1196175	Listeria_monocytogenes_1639_v113	0.9578
Listeria_monocytogenes_serotype_4b_str_02-6679_1196174	Listeria_monocytogenes_1639_v113	0.9578
Listeria_monocytogenes_serotype_4b_str_02-1792_1196173	Listeria_monocytogenes_1639_v113	0.9468
Listeria_monocytogenes_serotype_4b_str_02-1289_1196172	Listeria_monocytogenes_1639_v113	0.9468
Listeria_monocytogenes_serotype_4b_str_02-1103_1196171	Listeria_monocytogenes_1639_v113	0.9468
Listeria_monocytogenes_serotype_3c_str_10-5027_1196195	Listeria_monocytogenes_1639_v113	0.9488
Listeria_monocytogenes_serotype_1_2c_str_10-5026_1196194	Listeria_monocytogenes_1639_v113	0.9499
Listeria_monocytogenes_serotype_1_2c_str_10-5025_1196193	Listeria_monocytogenes_1639_v113	0.9499
Listeria_monocytogenes_serotype_1_2b_str_10-0811_1196164	Listeria_monocytogenes_1639_v113	0.9771
Listeria_monocytogenes_serotype_1_2b_str_10-0810_1196163	Listeria_monocytogenes_1639_v113	0.9771
Listeria_monocytogenes_serotype_1_2a_str_99-6370_1196179	Listeria_monocytogenes_1639_v113	0.9357
Listeria_monocytogenes_serotype_1_2a_str_98-2035_1196178	Listeria_monocytogenes_1639_v113	0.9357
Listeria_monocytogenes_serotype_1_2a_str_95-0093_1196177	Listeria_monocytogenes_1639_v113	0.9409
Listeria_monocytogenes_serotype_1_2a_str_88-0478_1196176	Listeria_monocytogenes_1639_v113	0.939
Listeria_monocytogenes_serotype_1_2a_str_10-5024_1196192	Listeria_monocytogenes_1639_v113	0.9413
Listeria_monocytogenes_serotype_1_2a_str_10-4758_1196168	Listeria_monocytogenes_1639_v113	0.9593
Listeria_monocytogenes_serotype_1_2a_str_10-4754_1196167	Listeria_monocytogenes_1639_v113	0.9593
Listeria_monocytogenes_serotype_1_2a_str_10-1321_1196191	Listeria_monocytogenes_1639_v113	0.9413
Listeria_monocytogenes_serotype_1_2a_str_10-1047_1196190	Listeria_monocytogenes_1639_v113	0.9416
Listeria_monocytogenes_serotype_1_2a_str_10-1046_1196189	Listeria_monocytogenes_1639_v113	0.9416
Listeria_monocytogenes_serotype_1_2a_str_10-0934_1196170	Listeria_monocytogenes_1639_v113	0.9518
Listeria_monocytogenes_serotype_1_2a_str_10-0933_1196169	Listeria_monocytogenes_1639_v113	0.9518
Listeria_monocytogenes_serotype_1_2a_str_10-0815_1196188	Listeria_monocytogenes_1639_v113	0.9367
Listeria_monocytogenes_serotype_1_2a_str_10-0814_1196187	Listeria_monocytogenes_1639_v113	0.9413
Listeria_monocytogenes_serotype_1_2a_str_10-0813_1196166	Listeria_monocytogenes_1639_v113	0.9501
Listeria_monocytogenes_serotype_1_2a_str_10-0812_1196165	Listeria_monocytogenes_1639_v113	0.9501
Listeria_monocytogenes_serotype_1_2a_str_08-7669_1196186	Listeria_monocytogenes_1639_v113	0.9369
Listeria_monocytogenes_serotype_1_2a_str_08-7374_1196185	Listeria_monocytogenes_1639_v113	0.9413
Listeria_monocytogenes_serotype_1_2a_str_08-6997_1196184	Listeria_monocytogenes_1639_v113	0.9367
Listeria_monocytogenes_serotype_1_2a_str_08-6569_1196183	Listeria_monocytogenes_1639_v113	0.9367
Listeria_monocytogenes_serotype_1_2a_str_08-6056_1196182	Listeria_monocytogenes_1639_v113	0.9367
Listeria_monocytogenes_serotype_1_2a_str_04-5457_1196181	Listeria_monocytogenes_1639_v113	0.9413
Listeria_monocytogenes_serotype_1_2a_str_02-5993_1196180	Listeria_monocytogenes_1639_v113	0.9409
Listeria_monocytogenes_R479a_1437838	Listeria_monocytogenes_1639_v113	0.9395
Listeria_monocytogenes_N53-1_1234142	Listeria_monocytogenes_1639_v113	0.899
Listeria_monocytogenes_M7_1030009	Listeria_monocytogenes_1639_v113	0.9164
Listeria_monocytogenes_LO28_393132	Listeria_monocytogenes_1639_v113	0.9474
Listeria_monocytogenes_L99_563174	Listeria_monocytogenes_1639_v113	0.917
Listeria_monocytogenes_L312_882094	Listeria_monocytogenes_1639_v113	0.9631
Listeria_monocytogenes_J1816_930781	Listeria_monocytogenes_1639_v113	0.9429
Listeria_monocytogenes_J1-220_930782	Listeria_monocytogenes_1639_v113	0.945
Listeria_monocytogenes_J0161_393130	Listeria_monocytogenes_1639_v113	0.9368
Listeria_monocytogenes_HCC23_552536	Listeria_monocytogenes_1639_v113	0.9163
Listeria_monocytogenes_FSL_R2-561_393126	Listeria_monocytogenes_1639_v113	0.9481
Listeria_monocytogenes_FSL_J1-208_393119	Listeria_monocytogenes_1639_v113	0.9112
Listeria_monocytogenes_FSL_J1-175_393118	Listeria_monocytogenes_1639_v113	0.9714
Listeria_monocytogenes_Finland_1998_393127	Listeria_monocytogenes_1639_v113	0.9522
Listeria_monocytogenes_EGD-e_169963_v1	Listeria_monocytogenes_1639_v113	0.9464
Listeria_monocytogenes_EGD-e_169963	Listeria_monocytogenes_1639_v113	0.9468
Listeria_monocytogenes_EGD_1334565	Listeria_monocytogenes_1639_v113	0.955
Listeria_monocytogenes_CFSAN002349_1299895	Listeria_monocytogenes_1639_v113	0.9625
Listeria_monocytogenes_ATCC_19117_882095_v1	Listeria_monocytogenes_1639_v113	0.9557
Listeria_monocytogenes_ATCC_19117_882095	Listeria_monocytogenes_1639_v113	0.9556
Listeria_monocytogenes_6179_1288295	Listeria_monocytogenes_1639_v113	0.9318
Listeria_monocytogenes_1639_v9	Listeria_monocytogenes_1639_v113	0.9445
Listeria_monocytogenes_1639_v99	Listeria_monocytogenes_1639_v113	0.9444
Listeria_monocytogenes_1639_v98	Listeria_monocytogenes_1639_v113	0.9349
Listeria_monocytogenes_1639_v97	Listeria_monocytogenes_1639_v113	0.9489
Listeria_monocytogenes_1639_v96	Listeria_monocytogenes_1639_v113	0.9429

Listeria_monocytogenes_1639_v95	Listeria_monocytogenes_1639_v113	0.943
Listeria_monocytogenes_1639_v94	Listeria_monocytogenes_1639_v113	0.9429
Listeria_monocytogenes_1639_v93	Listeria_monocytogenes_1639_v113	0.9435
Listeria_monocytogenes_1639_v92	Listeria_monocytogenes_1639_v113	0.944
Listeria_monocytogenes_1639_v91	Listeria_monocytogenes_1639_v113	0.9511
Listeria_monocytogenes_1639_v90	Listeria_monocytogenes_1639_v113	0.9349
Listeria_monocytogenes_1639_v8	Listeria_monocytogenes_1639_v113	0.929
Listeria_monocytogenes_1639_v89	Listeria_monocytogenes_1639_v113	0.9551
Listeria_monocytogenes_1639_v88	Listeria_monocytogenes_1639_v113	0.9468
Listeria_monocytogenes_1639_v87	Listeria_monocytogenes_1639_v113	0.9436
Listeria_monocytogenes_1639_v86	Listeria_monocytogenes_1639_v113	0.9425
Listeria_monocytogenes_1639_v85	Listeria_monocytogenes_1639_v113	0.9439
Listeria_monocytogenes_1639_v84	Listeria_monocytogenes_1639_v113	0.9474
Listeria_monocytogenes_1639_v83	Listeria_monocytogenes_1639_v113	0.946
Listeria_monocytogenes_1639_v82	Listeria_monocytogenes_1639_v113	0.9456
Listeria_monocytogenes_1639_v81	Listeria_monocytogenes_1639_v113	0.9479
Listeria_monocytogenes_1639_v80	Listeria_monocytogenes_1639_v113	0.983
Listeria_monocytogenes_1639_v7	Listeria_monocytogenes_1639_v113	0.929
Listeria_monocytogenes_1639_v79	Listeria_monocytogenes_1639_v113	0.9439
Listeria_monocytogenes_1639_v78	Listeria_monocytogenes_1639_v113	0.9468
Listeria_monocytogenes_1639_v77	Listeria_monocytogenes_1639_v113	0.9356
Listeria_monocytogenes_1639_v76	Listeria_monocytogenes_1639_v113	0.9419
Listeria_monocytogenes_1639_v75	Listeria_monocytogenes_1639_v113	0.917
Listeria_monocytogenes_1639_v74	Listeria_monocytogenes_1639_v113	0.9286
Listeria_monocytogenes_1639_v73	Listeria_monocytogenes_1639_v113	0.9432
Listeria_monocytogenes_1639_v72	Listeria_monocytogenes_1639_v113	0.944
Listeria_monocytogenes_1639_v71	Listeria_monocytogenes_1639_v113	0.9325
Listeria_monocytogenes_1639_v70	Listeria_monocytogenes_1639_v113	0.946
Listeria_monocytogenes_1639_v6	Listeria_monocytogenes_1639_v113	0.9284
Listeria_monocytogenes_1639_v69	Listeria_monocytogenes_1639_v113	0.9457
Listeria_monocytogenes_1639_v68	Listeria_monocytogenes_1639_v113	0.9369
Listeria_monocytogenes_1639_v67	Listeria_monocytogenes_1639_v113	0.9634
Listeria_monocytogenes_1639_v66	Listeria_monocytogenes_1639_v113	0.9529
Listeria_monocytogenes_1639_v65	Listeria_monocytogenes_1639_v113	0.9441
Listeria_monocytogenes_1639_v64	Listeria_monocytogenes_1639_v113	0.9453
Listeria_monocytogenes_1639_v63	Listeria_monocytogenes_1639_v113	0.9771
Listeria_monocytogenes_1639_v62	Listeria_monocytogenes_1639_v113	0.9429
Listeria_monocytogenes_1639_v61	Listeria_monocytogenes_1639_v113	0.9429
Listeria_monocytogenes_1639_v60	Listeria_monocytogenes_1639_v113	0.9373
Listeria_monocytogenes_1639_v5	Listeria_monocytogenes_1639_v113	0.93
Listeria_monocytogenes_1639_v59	Listeria_monocytogenes_1639_v113	0.9543
Listeria_monocytogenes_1639_v58	Listeria_monocytogenes_1639_v113	0.9505
Listeria_monocytogenes_1639_v57	Listeria_monocytogenes_1639_v113	0.9409
Listeria_monocytogenes_1639_v56	Listeria_monocytogenes_1639_v113	0.9463
Listeria_monocytogenes_1639_v55	Listeria_monocytogenes_1639_v113	0.9367
Listeria_monocytogenes_1639_v54	Listeria_monocytogenes_1639_v113	0.9209
Listeria_monocytogenes_1639_v53	Listeria_monocytogenes_1639_v113	0.935
Listeria_monocytogenes_1639_v52	Listeria_monocytogenes_1639_v113	0.9507
Listeria_monocytogenes_1639_v51	Listeria_monocytogenes_1639_v113	0.9406
Listeria_monocytogenes_1639_v50	Listeria_monocytogenes_1639_v113	0.9405
Listeria_monocytogenes_1639_v4	Listeria_monocytogenes_1639_v113	0.9432
Listeria_monocytogenes_1639_v49	Listeria_monocytogenes_1639_v113	0.9476
Listeria_monocytogenes_1639_v48	Listeria_monocytogenes_1639_v113	0.9439
Listeria_monocytogenes_1639_v47	Listeria_monocytogenes_1639_v113	0.9402
Listeria_monocytogenes_1639_v46	Listeria_monocytogenes_1639_v113	0.9373
Listeria_monocytogenes_1639_v45	Listeria_monocytogenes_1639_v113	0.9315
Listeria_monocytogenes_1639_v44	Listeria_monocytogenes_1639_v113	0.9413
Listeria_monocytogenes_1639_v43	Listeria_monocytogenes_1639_v113	0.9413
Listeria_monocytogenes_1639_v42	Listeria_monocytogenes_1639_v113	0.9219
Listeria_monocytogenes_1639_v41	Listeria_monocytogenes_1639_v113	0.9176
Listeria_monocytogenes_1639_v40	Listeria_monocytogenes_1639_v113	0.9477
Listeria_monocytogenes_1639_v3	Listeria_monocytogenes_1639_v113	0.9436
Listeria_monocytogenes_1639_v39	Listeria_monocytogenes_1639_v113	0.9587
Listeria_monocytogenes_1639_v38	Listeria_monocytogenes_1639_v113	0.9438
Listeria_monocytogenes_1639_v37	Listeria_monocytogenes_1639_v113	0.9388
Listeria_monocytogenes_1639_v36	Listeria_monocytogenes_1639_v113	0.9345
Listeria_monocytogenes_1639_v35	Listeria_monocytogenes_1639_v113	0.9484
Listeria_monocytogenes_1639_v34	Listeria_monocytogenes_1639_v113	0.9427
Listeria_monocytogenes_1639_v33	Listeria_monocytogenes_1639_v113	0.9771
Listeria_monocytogenes_1639_v32	Listeria_monocytogenes_1639_v113	0.9404
Listeria_monocytogenes_1639_v31	Listeria_monocytogenes_1639_v113	0.9431
Listeria_monocytogenes_1639_v30	Listeria_monocytogenes_1639_v113	0.9553
Listeria_monocytogenes_1639_v2	Listeria_monocytogenes_1639_v113	0.9105
Listeria_monocytogenes_1639_v29	Listeria_monocytogenes_1639_v113	0.9351
Listeria_monocytogenes_1639_v28	Listeria_monocytogenes_1639_v113	0.94
Listeria_monocytogenes_1639_v27	Listeria_monocytogenes_1639_v113	0.9299
Listeria_monocytogenes_1639_v26	Listeria_monocytogenes_1639_v113	0.93
Listeria_monocytogenes_1639_v25	Listeria_monocytogenes_1639_v113	0.93
Listeria_monocytogenes_1639_v24	Listeria_monocytogenes_1639_v113	0.9461
Listeria_monocytogenes_1639_v23	Listeria_monocytogenes_1639_v113	0.9461
Listeria_monocytogenes_1639_v22	Listeria_monocytogenes_1639_v113	0.9786
Listeria_monocytogenes_1639_v21	Listeria_monocytogenes_1639_v113	0.9446
Listeria_monocytogenes_1639_v20	Listeria_monocytogenes_1639_v113	0.9493
Listeria_monocytogenes_1639_v1	Listeria_monocytogenes_1639_v113	0.9246
Listeria_monocytogenes_1639_v19	Listeria_monocytogenes_1639_v113	0.949
Listeria_monocytogenes_1639_v198	Listeria_monocytogenes_1639_v113	0.9017
Listeria_monocytogenes_1639_v197	Listeria_monocytogenes_1639_v113	0.9339
Listeria_monocytogenes_1639_v196	Listeria_monocytogenes_1639_v113	0.938
Listeria_monocytogenes_1639_v195	Listeria_monocytogenes_1639_v113	0.9441
Listeria_monocytogenes_1639_v194	Listeria_monocytogenes_1639_v113	0.9437
Listeria_monocytogenes_1639_v193	Listeria_monocytogenes_1639_v113	0.9437

Listeria_monocytogenes_1639_v192	Listeria_monocytogenes_1639_v113	0.9042
Listeria_monocytogenes_1639_v191	Listeria_monocytogenes_1639_v113	0.9509
Listeria_monocytogenes_1639_v190	Listeria_monocytogenes_1639_v113	0.9525
Listeria_monocytogenes_1639_v18	Listeria_monocytogenes_1639_v113	0.9354
Listeria_monocytogenes_1639_v189	Listeria_monocytogenes_1639_v113	0.9514
Listeria_monocytogenes_1639_v188	Listeria_monocytogenes_1639_v113	0.9536
Listeria_monocytogenes_1639_v187	Listeria_monocytogenes_1639_v113	0.9521
Listeria_monocytogenes_1639_v186	Listeria_monocytogenes_1639_v113	0.9525
Listeria_monocytogenes_1639_v185	Listeria_monocytogenes_1639_v113	0.9525
Listeria_monocytogenes_1639_v184	Listeria_monocytogenes_1639_v113	0.9517
Listeria_monocytogenes_1639_v183	Listeria_monocytogenes_1639_v113	0.9467
Listeria_monocytogenes_1639_v182	Listeria_monocytogenes_1639_v113	0.9489
Listeria_monocytogenes_1639_v181	Listeria_monocytogenes_1639_v113	0.9236
Listeria_monocytogenes_1639_v180	Listeria_monocytogenes_1639_v113	0.9236
Listeria_monocytogenes_1639_v17	Listeria_monocytogenes_1639_v113	0.9354
Listeria_monocytogenes_1639_v179	Listeria_monocytogenes_1639_v113	0.9362
Listeria_monocytogenes_1639_v178	Listeria_monocytogenes_1639_v113	0.9521
Listeria_monocytogenes_1639_v177	Listeria_monocytogenes_1639_v113	0.9609
Listeria_monocytogenes_1639_v176	Listeria_monocytogenes_1639_v113	0.9388
Listeria_monocytogenes_1639_v175	Listeria_monocytogenes_1639_v113	0.9583
Listeria_monocytogenes_1639_v174	Listeria_monocytogenes_1639_v113	0.9522
Listeria_monocytogenes_1639_v173	Listeria_monocytogenes_1639_v113	0.9688
Listeria_monocytogenes_1639_v172	Listeria_monocytogenes_1639_v113	0.9383
Listeria_monocytogenes_1639_v171	Listeria_monocytogenes_1639_v113	0.9392
Listeria_monocytogenes_1639_v170	Listeria_monocytogenes_1639_v113	0.9471
Listeria_monocytogenes_1639_v16	Listeria_monocytogenes_1639_v113	0.9274
Listeria_monocytogenes_1639_v169	Listeria_monocytogenes_1639_v113	0.9606
Listeria_monocytogenes_1639_v168	Listeria_monocytogenes_1639_v113	0.9442
Listeria_monocytogenes_1639_v167	Listeria_monocytogenes_1639_v113	0.9383
Listeria_monocytogenes_1639_v166	Listeria_monocytogenes_1639_v113	0.9445
Listeria_monocytogenes_1639_v165	Listeria_monocytogenes_1639_v113	0.9602
Listeria_monocytogenes_1639_v164	Listeria_monocytogenes_1639_v113	0.96
Listeria_monocytogenes_1639_v163	Listeria_monocytogenes_1639_v113	0.9628
Listeria_monocytogenes_1639_v162	Listeria_monocytogenes_1639_v113	0.96
Listeria_monocytogenes_1639_v161	Listeria_monocytogenes_1639_v113	0.9612
Listeria_monocytogenes_1639_v160	Listeria_monocytogenes_1639_v113	0.9612
Listeria_monocytogenes_1639_v15	Listeria_monocytogenes_1639_v113	0.9613
Listeria_monocytogenes_1639_v159	Listeria_monocytogenes_1639_v113	0.9609
Listeria_monocytogenes_1639_v158	Listeria_monocytogenes_1639_v113	0.9609
Listeria_monocytogenes_1639_v157	Listeria_monocytogenes_1639_v113	0.9609
Listeria_monocytogenes_1639_v156	Listeria_monocytogenes_1639_v113	0.9295
Listeria_monocytogenes_1639_v155	Listeria_monocytogenes_1639_v113	0.9446
Listeria_monocytogenes_1639_v154	Listeria_monocytogenes_1639_v113	0.9588
Listeria_monocytogenes_1639_v153	Listeria_monocytogenes_1639_v113	0.9438
Listeria_monocytogenes_1639_v152	Listeria_monocytogenes_1639_v113	0.9613
Listeria_monocytogenes_1639_v151	Listeria_monocytogenes_1639_v113	0.9613
Listeria_monocytogenes_1639_v150	Listeria_monocytogenes_1639_v113	0.9553
Listeria_monocytogenes_1639_v14	Listeria_monocytogenes_1639_v113	0.9558
Listeria_monocytogenes_1639_v149	Listeria_monocytogenes_1639_v113	0.955
Listeria_monocytogenes_1639_v148	Listeria_monocytogenes_1639_v113	0.9602
Listeria_monocytogenes_1639_v147	Listeria_monocytogenes_1639_v113	0.9461
Listeria_monocytogenes_1639_v146	Listeria_monocytogenes_1639_v113	0.9554
Listeria_monocytogenes_1639_v145	Listeria_monocytogenes_1639_v113	0.9459
Listeria_monocytogenes_1639_v144	Listeria_monocytogenes_1639_v113	0.9624
Listeria_monocytogenes_1639_v143	Listeria_monocytogenes_1639_v113	0.9539
Listeria_monocytogenes_1639_v142	Listeria_monocytogenes_1639_v113	0.9627
Listeria_monocytogenes_1639_v141	Listeria_monocytogenes_1639_v113	0.9696
Listeria_monocytogenes_1639_v140	Listeria_monocytogenes_1639_v113	0.9631
Listeria_monocytogenes_1639_v13	Listeria_monocytogenes_1639_v113	0.9613
Listeria_monocytogenes_1639_v139	Listeria_monocytogenes_1639_v113	0.959
Listeria_monocytogenes_1639_v138	Listeria_monocytogenes_1639_v113	0.9643
Listeria_monocytogenes_1639_v137	Listeria_monocytogenes_1639_v113	0.9643
Listeria_monocytogenes_1639_v136	Listeria_monocytogenes_1639_v113	0.9578
Listeria_monocytogenes_1639_v135	Listeria_monocytogenes_1639_v113	0.9156
Listeria_monocytogenes_1639_v134	Listeria_monocytogenes_1639_v113	0.9545
Listeria_monocytogenes_1639_v133	Listeria_monocytogenes_1639_v113	0.9458
Listeria_monocytogenes_1639_v132	Listeria_monocytogenes_1639_v113	0.9164
Listeria_monocytogenes_1639_v131	Listeria_monocytogenes_1639_v113	0.9522
Listeria_monocytogenes_1639_v130	Listeria_monocytogenes_1639_v113	0.9488
Listeria_monocytogenes_1639_v12	Listeria_monocytogenes_1639_v113	0.9297
Listeria_monocytogenes_1639_v129	Listeria_monocytogenes_1639_v113	0.9835
Listeria_monocytogenes_1639_v128	Listeria_monocytogenes_1639_v113	0.9655
Listeria_monocytogenes_1639_v127	Listeria_monocytogenes_1639_v113	0.9453
Listeria_monocytogenes_1639_v126	Listeria_monocytogenes_1639_v113	0.964
Listeria_monocytogenes_1639_v125	Listeria_monocytogenes_1639_v113	0.9386
Listeria_monocytogenes_1639_v124	Listeria_monocytogenes_1639_v113	0.9325
Listeria_monocytogenes_1639_v123	Listeria_monocytogenes_1639_v113	0.9625
Listeria_monocytogenes_1639_v122	Listeria_monocytogenes_1639_v113	0.9628
Listeria_monocytogenes_1639_v121	Listeria_monocytogenes_1639_v113	0.9628
Listeria_monocytogenes_1639_v120	Listeria_monocytogenes_1639_v113	0.9625
Listeria_monocytogenes_1639_v11	Listeria_monocytogenes_1639_v113	0.9273
Listeria_monocytogenes_1639_v119	Listeria_monocytogenes_1639_v113	0.9628
Listeria_monocytogenes_1639_v118	Listeria_monocytogenes_1639_v113	0.9628
Listeria_monocytogenes_1639_v117	Listeria_monocytogenes_1639_v113	0.957
Listeria_monocytogenes_1639_v116	Listeria_monocytogenes_1639_v113	0.9543
Listeria_monocytogenes_1639_v115	Listeria_monocytogenes_1639_v113	0.9543
Listeria_monocytogenes_1639_v114	Listeria_monocytogenes_1639_v113	0.9463
Listeria_monocytogenes_1639_v113	Listeria_monocytogenes_1639_v113	0.0664
Listeria_welshimeri_1643	Listeria_monocytogenes_1639_v114	0.872
Listeria_welshimeri_serovar_6b_str_SLCC5334_386043	Listeria_monocytogenes_1639_v114	0.8713
Listeria_seeligeri_serovar_1_2b_str_SLCC3954_683837	Listeria_monocytogenes_1639_v114	0.8783

Listeria_seeligeri_FSL_S4-171_702452	Listeria_monocytogenes_1639_v114	0.8195
Listeria_seeligeri_FSL_N1-067_702453	Listeria_monocytogenes_1639_v114	0.8347
Listeria_seeligeri_1640_v1	Listeria_monocytogenes_1639_v114	0.8752
Listeria_seeligeri_1640	Listeria_monocytogenes_1639_v114	0.8716
Listeria_monocytogenes_WSLC1042_1457188	Listeria_monocytogenes_1639_v114	0.9226
Listeria_monocytogenes_WSLC1001_1457187	Listeria_monocytogenes_1639_v114	0.9823
Listeria_monocytogenes_SLCC7179_879090	Listeria_monocytogenes_1639_v114	0.9575
Listeria_monocytogenes_SLCC5850_882096	Listeria_monocytogenes_1639_v114	0.9785
Listeria_monocytogenes_SLCC2755_932919	Listeria_monocytogenes_1639_v114	0.9273
Listeria_monocytogenes_SLCC2540_879089	Listeria_monocytogenes_1639_v114	0.939
Listeria_monocytogenes_SLCC2479_882020	Listeria_monocytogenes_1639_v114	0.9626
Listeria_monocytogenes_SLCC2378_879088	Listeria_monocytogenes_1639_v114	0.924
Listeria_monocytogenes_SLCC2376_882097	Listeria_monocytogenes_1639_v114	0.9361
Listeria_monocytogenes_SLCC2372_932920	Listeria_monocytogenes_1639_v114	0.9548
Listeria_monocytogenes_serotype_4b_str_LL195_1230340	Listeria_monocytogenes_1639_v114	0.9296
Listeria_monocytogenes_serotype_4b_str_LIS0087_1134669	Listeria_monocytogenes_1639_v114	0.9638
Listeria_monocytogenes_serotype_4b_str_F2365_265669	Listeria_monocytogenes_1639_v114	0.9296
Listeria_monocytogenes_serotype_4b_str_CLIP_80459_568819	Listeria_monocytogenes_1639_v114	0.9364
Listeria_monocytogenes_serotype_4b_str_81-0861_1196161	Listeria_monocytogenes_1639_v114	0.9188
Listeria_monocytogenes_serotype_4b_str_81-0592_1196160	Listeria_monocytogenes_1639_v114	0.9199
Listeria_monocytogenes_serotype_4b_str_81-0558_1196159	Listeria_monocytogenes_1639_v114	0.9206
Listeria_monocytogenes_serotype_4b_str_10-0809_1196162	Listeria_monocytogenes_1639_v114	0.9199
Listeria_monocytogenes_serotype_4b_str_02-6680_1196175	Listeria_monocytogenes_1639_v114	0.9346
Listeria_monocytogenes_serotype_4b_str_02-6679_1196174	Listeria_monocytogenes_1639_v114	0.9346
Listeria_monocytogenes_serotype_4b_str_02-1792_1196173	Listeria_monocytogenes_1639_v114	0.921
Listeria_monocytogenes_serotype_4b_str_02-1289_1196172	Listeria_monocytogenes_1639_v114	0.921
Listeria_monocytogenes_serotype_4b_str_02-1103_1196171	Listeria_monocytogenes_1639_v114	0.921
Listeria_monocytogenes_serotype_3c_str_10-5027_1196195	Listeria_monocytogenes_1639_v114	0.9593
Listeria_monocytogenes_serotype_1_2c_str_10-5026_1196194	Listeria_monocytogenes_1639_v114	0.9608
Listeria_monocytogenes_serotype_1_2c_str_10-5025_1196193	Listeria_monocytogenes_1639_v114	0.9608
Listeria_monocytogenes_serotype_1_2b_str_10-0811_1196164	Listeria_monocytogenes_1639_v114	0.9375
Listeria_monocytogenes_serotype_1_2b_str_10-0810_1196163	Listeria_monocytogenes_1639_v114	0.9375
Listeria_monocytogenes_serotype_1_2a_str_99-6370_1196179	Listeria_monocytogenes_1639_v114	0.9491
Listeria_monocytogenes_serotype_1_2a_str_98-2035_1196178	Listeria_monocytogenes_1639_v114	0.9491
Listeria_monocytogenes_serotype_1_2a_str_95-0093_1196177	Listeria_monocytogenes_1639_v114	0.9551
Listeria_monocytogenes_serotype_1_2a_str_88-0478_1196176	Listeria_monocytogenes_1639_v114	0.9573
Listeria_monocytogenes_serotype_1_2a_str_10-5024_1196192	Listeria_monocytogenes_1639_v114	0.9555
Listeria_monocytogenes_serotype_1_2a_str_10-4758_1196168	Listeria_monocytogenes_1639_v114	0.9589
Listeria_monocytogenes_serotype_1_2a_str_10-4754_1196167	Listeria_monocytogenes_1639_v114	0.9589
Listeria_monocytogenes_serotype_1_2a_str_10-1321_1196191	Listeria_monocytogenes_1639_v114	0.9555
Listeria_monocytogenes_serotype_1_2a_str_10-1047_1196190	Listeria_monocytogenes_1639_v114	0.9551
Listeria_monocytogenes_serotype_1_2a_str_10-1046_1196189	Listeria_monocytogenes_1639_v114	0.9551
Listeria_monocytogenes_serotype_1_2a_str_10-0934_1196170	Listeria_monocytogenes_1639_v114	0.9667
Listeria_monocytogenes_serotype_1_2a_str_10-0933_1196169	Listeria_monocytogenes_1639_v114	0.9667
Listeria_monocytogenes_serotype_1_2a_str_10-0815_1196188	Listeria_monocytogenes_1639_v114	0.9505
Listeria_monocytogenes_serotype_1_2a_str_10-0814_1196187	Listeria_monocytogenes_1639_v114	0.9551
Listeria_monocytogenes_serotype_1_2a_str_10-0813_1196166	Listeria_monocytogenes_1639_v114	0.9775
Listeria_monocytogenes_serotype_1_2a_str_10-0812_1196165	Listeria_monocytogenes_1639_v114	0.9775
Listeria_monocytogenes_serotype_1_2a_str_08-7669_1196186	Listeria_monocytogenes_1639_v114	0.9528
Listeria_monocytogenes_serotype_1_2a_str_08-7374_1196185	Listeria_monocytogenes_1639_v114	0.9555
Listeria_monocytogenes_serotype_1_2a_str_08-6997_1196184	Listeria_monocytogenes_1639_v114	0.9505
Listeria_monocytogenes_serotype_1_2a_str_08-6569_1196183	Listeria_monocytogenes_1639_v114	0.9505
Listeria_monocytogenes_serotype_1_2a_str_08-6056_1196182	Listeria_monocytogenes_1639_v114	0.9505
Listeria_monocytogenes_serotype_1_2a_str_04-5457_1196181	Listeria_monocytogenes_1639_v114	0.9555
Listeria_monocytogenes_serotype_1_2a_str_02-5993_1196180	Listeria_monocytogenes_1639_v114	0.9551
Listeria_monocytogenes_R479a_1437838	Listeria_monocytogenes_1639_v114	0.9533
Listeria_monocytogenes_N53-1_1234142	Listeria_monocytogenes_1639_v114	0.9038
Listeria_monocytogenes_M7_1030009	Listeria_monocytogenes_1639_v114	0.9081
Listeria_monocytogenes_LO28_393132	Listeria_monocytogenes_1639_v114	0.9593
Listeria_monocytogenes_L99_563174	Listeria_monocytogenes_1639_v114	0.9088
Listeria_monocytogenes_L312_882094	Listeria_monocytogenes_1639_v114	0.9364
Listeria_monocytogenes_J1816_930781	Listeria_monocytogenes_1639_v114	0.928
Listeria_monocytogenes_J1-220_930782	Listeria_monocytogenes_1639_v114	0.9228
Listeria_monocytogenes_J0161_393130	Listeria_monocytogenes_1639_v114	0.9515
Listeria_monocytogenes_HCC23_552536	Listeria_monocytogenes_1639_v114	0.9081
Listeria_monocytogenes_FSL_R2-561_393126	Listeria_monocytogenes_1639_v114	0.9556
Listeria_monocytogenes_FSL_J1-208_393119	Listeria_monocytogenes_1639_v114	0.9017
Listeria_monocytogenes_FSL_J1-175_393118	Listeria_monocytogenes_1639_v114	0.9441
Listeria_monocytogenes_Finland_1998_393127	Listeria_monocytogenes_1639_v114	0.9545
Listeria_monocytogenes_EGD-e_169963_v1	Listeria_monocytogenes_1639_v114	0.9514
Listeria_monocytogenes_EGD-e_169963	Listeria_monocytogenes_1639_v114	0.9514
Listeria_monocytogenes_EGD_1334565	Listeria_monocytogenes_1639_v114	0.9792
Listeria_monocytogenes_CFSAN002349_1299895	Listeria_monocytogenes_1639_v114	0.9347
Listeria_monocytogenes_ATCC_19117_882095_v1	Listeria_monocytogenes_1639_v114	0.9306
Listeria_monocytogenes_ATCC_19117_882095	Listeria_monocytogenes_1639_v114	0.9306
Listeria_monocytogenes_6179_1288295	Listeria_monocytogenes_1639_v114	0.9474
Listeria_monocytogenes_1639_v9	Listeria_monocytogenes_1639_v114	0.9288
Listeria_monocytogenes_1639_v99	Listeria_monocytogenes_1639_v114	0.9281
Listeria_monocytogenes_1639_v98	Listeria_monocytogenes_1639_v114	0.9522
Listeria_monocytogenes_1639_v97	Listeria_monocytogenes_1639_v114	0.9331
Listeria_monocytogenes_1639_v96	Listeria_monocytogenes_1639_v114	0.9277
Listeria_monocytogenes_1639_v95	Listeria_monocytogenes_1639_v114	0.9278
Listeria_monocytogenes_1639_v94	Listeria_monocytogenes_1639_v114	0.9281
Listeria_monocytogenes_1639_v93	Listeria_monocytogenes_1639_v114	0.9439
Listeria_monocytogenes_1639_v92	Listeria_monocytogenes_1639_v114	0.9285
Listeria_monocytogenes_1639_v91	Listeria_monocytogenes_1639_v114	0.925
Listeria_monocytogenes_1639_v90	Listeria_monocytogenes_1639_v114	0.9522
Listeria_monocytogenes_1639_v8	Listeria_monocytogenes_1639_v114	0.9446
Listeria_monocytogenes_1639_v89	Listeria_monocytogenes_1639_v114	0.9317
Listeria_monocytogenes_1639_v88	Listeria_monocytogenes_1639_v114	0.9708
Listeria_monocytogenes_1639_v87	Listeria_monocytogenes_1639_v114	0.975

Listeria_monocytogenes_1639_v86	Listeria_monocytogenes_1639_v114	0.9746
Listeria_monocytogenes_1639_v85	Listeria_monocytogenes_1639_v114	0.9182
Listeria_monocytogenes_1639_v84	Listeria_monocytogenes_1639_v114	0.9651
Listeria_monocytogenes_1639_v83	Listeria_monocytogenes_1639_v114	0.9277
Listeria_monocytogenes_1639_v82	Listeria_monocytogenes_1639_v114	0.9273
Listeria_monocytogenes_1639_v81	Listeria_monocytogenes_1639_v114	0.9592
Listeria_monocytogenes_1639_v80	Listeria_monocytogenes_1639_v114	0.9403
Listeria_monocytogenes_1639_v7	Listeria_monocytogenes_1639_v114	0.9446
Listeria_monocytogenes_1639_v79	Listeria_monocytogenes_1639_v114	0.9585
Listeria_monocytogenes_1639_v78	Listeria_monocytogenes_1639_v114	0.9584
Listeria_monocytogenes_1639_v77	Listeria_monocytogenes_1639_v114	0.9519
Listeria_monocytogenes_1639_v76	Listeria_monocytogenes_1639_v114	0.9513
Listeria_monocytogenes_1639_v75	Listeria_monocytogenes_1639_v114	0.9088
Listeria_monocytogenes_1639_v74	Listeria_monocytogenes_1639_v114	0.9582
Listeria_monocytogenes_1639_v73	Listeria_monocytogenes_1639_v114	0.9492
Listeria_monocytogenes_1639_v72	Listeria_monocytogenes_1639_v114	0.9238
Listeria_monocytogenes_1639_v71	Listeria_monocytogenes_1639_v114	0.9441
Listeria_monocytogenes_1639_v70	Listeria_monocytogenes_1639_v114	0.9594
Listeria_monocytogenes_1639_v6	Listeria_monocytogenes_1639_v114	0.9439
Listeria_monocytogenes_1639_v69	Listeria_monocytogenes_1639_v114	0.9203
Listeria_monocytogenes_1639_v68	Listeria_monocytogenes_1639_v114	0.9438
Listeria_monocytogenes_1639_v67	Listeria_monocytogenes_1639_v114	0.949
Listeria_monocytogenes_1639_v66	Listeria_monocytogenes_1639_v114	0.9589
Listeria_monocytogenes_1639_v65	Listeria_monocytogenes_1639_v114	0.9531
Listeria_monocytogenes_1639_v64	Listeria_monocytogenes_1639_v114	0.9536
Listeria_monocytogenes_1639_v63	Listeria_monocytogenes_1639_v114	0.9393
Listeria_monocytogenes_1639_v62	Listeria_monocytogenes_1639_v114	0.9556
Listeria_monocytogenes_1639_v61	Listeria_monocytogenes_1639_v114	0.9572
Listeria_monocytogenes_1639_v60	Listeria_monocytogenes_1639_v114	0.952
Listeria_monocytogenes_1639_v5	Listeria_monocytogenes_1639_v114	0.8947
Listeria_monocytogenes_1639_v59	Listeria_monocytogenes_1639_v114	0.928
Listeria_monocytogenes_1639_v58	Listeria_monocytogenes_1639_v114	0.9243
Listeria_monocytogenes_1639_v57	Listeria_monocytogenes_1639_v114	0.9214
Listeria_monocytogenes_1639_v56	Listeria_monocytogenes_1639_v114	0.9321
Listeria_monocytogenes_1639_v55	Listeria_monocytogenes_1639_v114	0.9228
Listeria_monocytogenes_1639_v54	Listeria_monocytogenes_1639_v114	0.9241
Listeria_monocytogenes_1639_v53	Listeria_monocytogenes_1639_v114	0.9408
Listeria_monocytogenes_1639_v52	Listeria_monocytogenes_1639_v114	0.9301
Listeria_monocytogenes_1639_v51	Listeria_monocytogenes_1639_v114	0.921
Listeria_monocytogenes_1639_v50	Listeria_monocytogenes_1639_v114	0.954
Listeria_monocytogenes_1639_v4	Listeria_monocytogenes_1639_v114	0.9073
Listeria_monocytogenes_1639_v49	Listeria_monocytogenes_1639_v114	0.9177
Listeria_monocytogenes_1639_v48	Listeria_monocytogenes_1639_v114	0.954
Listeria_monocytogenes_1639_v47	Listeria_monocytogenes_1639_v114	0.9558
Listeria_monocytogenes_1639_v46	Listeria_monocytogenes_1639_v114	0.9494
Listeria_monocytogenes_1639_v45	Listeria_monocytogenes_1639_v114	0.9453
Listeria_monocytogenes_1639_v44	Listeria_monocytogenes_1639_v114	0.9555
Listeria_monocytogenes_1639_v43	Listeria_monocytogenes_1639_v114	0.9555
Listeria_monocytogenes_1639_v42	Listeria_monocytogenes_1639_v114	0.9413
Listeria_monocytogenes_1639_v41	Listeria_monocytogenes_1639_v114	0.9336
Listeria_monocytogenes_1639_v40	Listeria_monocytogenes_1639_v114	0.9522
Listeria_monocytogenes_1639_v3	Listeria_monocytogenes_1639_v114	0.9077
Listeria_monocytogenes_1639_v39	Listeria_monocytogenes_1639_v114	0.9448
Listeria_monocytogenes_1639_v38	Listeria_monocytogenes_1639_v114	0.9501
Listeria_monocytogenes_1639_v37	Listeria_monocytogenes_1639_v114	0.9423
Listeria_monocytogenes_1639_v36	Listeria_monocytogenes_1639_v114	0.9479
Listeria_monocytogenes_1639_v35	Listeria_monocytogenes_1639_v114	0.9567
Listeria_monocytogenes_1639_v34	Listeria_monocytogenes_1639_v114	0.9528
Listeria_monocytogenes_1639_v33	Listeria_monocytogenes_1639_v114	0.9375
Listeria_monocytogenes_1639_v32	Listeria_monocytogenes_1639_v114	0.9479
Listeria_monocytogenes_1639_v31	Listeria_monocytogenes_1639_v114	0.9532
Listeria_monocytogenes_1639_v30	Listeria_monocytogenes_1639_v114	0.9243
Listeria_monocytogenes_1639_v2	Listeria_monocytogenes_1639_v114	0.9018
Listeria_monocytogenes_1639_v29	Listeria_monocytogenes_1639_v114	0.9463
Listeria_monocytogenes_1639_v28	Listeria_monocytogenes_1639_v114	0.9523
Listeria_monocytogenes_1639_v27	Listeria_monocytogenes_1639_v114	0.9382
Listeria_monocytogenes_1639_v26	Listeria_monocytogenes_1639_v114	0.9382
Listeria_monocytogenes_1639_v25	Listeria_monocytogenes_1639_v114	0.9378
Listeria_monocytogenes_1639_v24	Listeria_monocytogenes_1639_v114	0.9224
Listeria_monocytogenes_1639_v23	Listeria_monocytogenes_1639_v114	0.9224
Listeria_monocytogenes_1639_v22	Listeria_monocytogenes_1639_v114	0.9437
Listeria_monocytogenes_1639_v21	Listeria_monocytogenes_1639_v114	0.9243
Listeria_monocytogenes_1639_v20	Listeria_monocytogenes_1639_v114	0.9427
Listeria_monocytogenes_1639_v1	Listeria_monocytogenes_1639_v114	0.9405
Listeria_monocytogenes_1639_v19	Listeria_monocytogenes_1639_v114	0.9423
Listeria_monocytogenes_1639_v198	Listeria_monocytogenes_1639_v114	0.9054
Listeria_monocytogenes_1639_v197	Listeria_monocytogenes_1639_v114	0.9382
Listeria_monocytogenes_1639_v196	Listeria_monocytogenes_1639_v114	0.9631
Listeria_monocytogenes_1639_v195	Listeria_monocytogenes_1639_v114	0.9383
Listeria_monocytogenes_1639_v194	Listeria_monocytogenes_1639_v114	0.9365
Listeria_monocytogenes_1639_v193	Listeria_monocytogenes_1639_v114	0.9369
Listeria_monocytogenes_1639_v192	Listeria_monocytogenes_1639_v114	0.8952
Listeria_monocytogenes_1639_v191	Listeria_monocytogenes_1639_v114	0.9624
Listeria_monocytogenes_1639_v190	Listeria_monocytogenes_1639_v114	0.9789
Listeria_monocytogenes_1639_v18	Listeria_monocytogenes_1639_v114	0.9178
Listeria_monocytogenes_1639_v189	Listeria_monocytogenes_1639_v114	0.9782
Listeria_monocytogenes_1639_v188	Listeria_monocytogenes_1639_v114	0.9578
Listeria_monocytogenes_1639_v187	Listeria_monocytogenes_1639_v114	0.9789
Listeria_monocytogenes_1639_v186	Listeria_monocytogenes_1639_v114	0.9789
Listeria_monocytogenes_1639_v185	Listeria_monocytogenes_1639_v114	0.9789
Listeria_monocytogenes_1639_v184	Listeria_monocytogenes_1639_v114	0.9789

Listeria_monocytogenes_1639_v183	Listeria_monocytogenes_1639_v114	0.954
Listeria_monocytogenes_1639_v182	Listeria_monocytogenes_1639_v114	0.9563
Listeria_monocytogenes_1639_v181	Listeria_monocytogenes_1639_v114	0.9121
Listeria_monocytogenes_1639_v180	Listeria_monocytogenes_1639_v114	0.9121
Listeria_monocytogenes_1639_v17	Listeria_monocytogenes_1639_v114	0.9178
Listeria_monocytogenes_1639_v179	Listeria_monocytogenes_1639_v114	0.956
Listeria_monocytogenes_1639_v178	Listeria_monocytogenes_1639_v114	0.9598
Listeria_monocytogenes_1639_v177	Listeria_monocytogenes_1639_v114	0.9347
Listeria_monocytogenes_1639_v176	Listeria_monocytogenes_1639_v114	0.9274
Listeria_monocytogenes_1639_v175	Listeria_monocytogenes_1639_v114	0.9643
Listeria_monocytogenes_1639_v174	Listeria_monocytogenes_1639_v114	0.9633
Listeria_monocytogenes_1639_v173	Listeria_monocytogenes_1639_v114	0.9438
Listeria_monocytogenes_1639_v172	Listeria_monocytogenes_1639_v114	0.9285
Listeria_monocytogenes_1639_v171	Listeria_monocytogenes_1639_v114	0.9668
Listeria_monocytogenes_1639_v170	Listeria_monocytogenes_1639_v114	0.9749
Listeria_monocytogenes_1639_v16	Listeria_monocytogenes_1639_v114	0.9411
Listeria_monocytogenes_1639_v169	Listeria_monocytogenes_1639_v114	0.9654
Listeria_monocytogenes_1639_v168	Listeria_monocytogenes_1639_v114	0.9542
Listeria_monocytogenes_1639_v167	Listeria_monocytogenes_1639_v114	0.9612
Listeria_monocytogenes_1639_v166	Listeria_monocytogenes_1639_v114	0.9545
Listeria_monocytogenes_1639_v165	Listeria_monocytogenes_1639_v114	0.9651
Listeria_monocytogenes_1639_v164	Listeria_monocytogenes_1639_v114	0.9287
Listeria_monocytogenes_1639_v163	Listeria_monocytogenes_1639_v114	0.9387
Listeria_monocytogenes_1639_v162	Listeria_monocytogenes_1639_v114	0.9287
Listeria_monocytogenes_1639_v161	Listeria_monocytogenes_1639_v114	0.9299
Listeria_monocytogenes_1639_v160	Listeria_monocytogenes_1639_v114	0.9299
Listeria_monocytogenes_1639_v15	Listeria_monocytogenes_1639_v114	0.9224
Listeria_monocytogenes_1639_v159	Listeria_monocytogenes_1639_v114	0.9347
Listeria_monocytogenes_1639_v158	Listeria_monocytogenes_1639_v114	0.9296
Listeria_monocytogenes_1639_v157	Listeria_monocytogenes_1639_v114	0.9296
Listeria_monocytogenes_1639_v156	Listeria_monocytogenes_1639_v114	0.9615
Listeria_monocytogenes_1639_v155	Listeria_monocytogenes_1639_v114	0.9602
Listeria_monocytogenes_1639_v154	Listeria_monocytogenes_1639_v114	0.9644
Listeria_monocytogenes_1639_v153	Listeria_monocytogenes_1639_v114	0.9515
Listeria_monocytogenes_1639_v152	Listeria_monocytogenes_1639_v114	0.935
Listeria_monocytogenes_1639_v151	Listeria_monocytogenes_1639_v114	0.935
Listeria_monocytogenes_1639_v150	Listeria_monocytogenes_1639_v114	0.9833
Listeria_monocytogenes_1639_v14	Listeria_monocytogenes_1639_v114	0.9189
Listeria_monocytogenes_1639_v149	Listeria_monocytogenes_1639_v114	0.9792
Listeria_monocytogenes_1639_v148	Listeria_monocytogenes_1639_v114	0.9339
Listeria_monocytogenes_1639_v147	Listeria_monocytogenes_1639_v114	0.9199
Listeria_monocytogenes_1639_v146	Listeria_monocytogenes_1639_v114	0.9557
Listeria_monocytogenes_1639_v145	Listeria_monocytogenes_1639_v114	0.9524
Listeria_monocytogenes_1639_v144	Listeria_monocytogenes_1639_v114	0.9356
Listeria_monocytogenes_1639_v143	Listeria_monocytogenes_1639_v114	0.955
Listeria_monocytogenes_1639_v142	Listeria_monocytogenes_1639_v114	0.936
Listeria_monocytogenes_1639_v141	Listeria_monocytogenes_1639_v114	0.9443
Listeria_monocytogenes_1639_v140	Listeria_monocytogenes_1639_v114	0.9364
Listeria_monocytogenes_1639_v13	Listeria_monocytogenes_1639_v114	0.9228
Listeria_monocytogenes_1639_v139	Listeria_monocytogenes_1639_v114	0.9342
Listeria_monocytogenes_1639_v138	Listeria_monocytogenes_1639_v114	0.9387
Listeria_monocytogenes_1639_v137	Listeria_monocytogenes_1639_v114	0.9383
Listeria_monocytogenes_1639_v136	Listeria_monocytogenes_1639_v114	0.9589
Listeria_monocytogenes_1639_v135	Listeria_monocytogenes_1639_v114	0.9095
Listeria_monocytogenes_1639_v134	Listeria_monocytogenes_1639_v114	0.9238
Listeria_monocytogenes_1639_v133	Listeria_monocytogenes_1639_v114	0.9603
Listeria_monocytogenes_1639_v132	Listeria_monocytogenes_1639_v114	0.9081
Listeria_monocytogenes_1639_v131	Listeria_monocytogenes_1639_v114	0.9289
Listeria_monocytogenes_1639_v130	Listeria_monocytogenes_1639_v114	0.9559
Listeria_monocytogenes_1639_v12	Listeria_monocytogenes_1639_v114	0.9405
Listeria_monocytogenes_1639_v129	Listeria_monocytogenes_1639_v114	0.9394
Listeria_monocytogenes_1639_v128	Listeria_monocytogenes_1639_v114	0.9404
Listeria_monocytogenes_1639_v127	Listeria_monocytogenes_1639_v114	0.9529
Listeria_monocytogenes_1639_v126	Listeria_monocytogenes_1639_v114	0.9415
Listeria_monocytogenes_1639_v125	Listeria_monocytogenes_1639_v114	0.9565
Listeria_monocytogenes_1639_v124	Listeria_monocytogenes_1639_v114	0.921
Listeria_monocytogenes_1639_v123	Listeria_monocytogenes_1639_v114	0.9347
Listeria_monocytogenes_1639_v122	Listeria_monocytogenes_1639_v114	0.9347
Listeria_monocytogenes_1639_v121	Listeria_monocytogenes_1639_v114	0.9347
Listeria_monocytogenes_1639_v120	Listeria_monocytogenes_1639_v114	0.9343
Listeria_monocytogenes_1639_v11	Listeria_monocytogenes_1639_v114	0.9436
Listeria_monocytogenes_1639_v119	Listeria_monocytogenes_1639_v114	0.9347
Listeria_monocytogenes_1639_v118	Listeria_monocytogenes_1639_v114	0.9347
Listeria_monocytogenes_1639_v117	Listeria_monocytogenes_1639_v114	0.9574
Listeria_monocytogenes_1639_v116	Listeria_monocytogenes_1639_v114	0.924
Listeria_monocytogenes_1639_v115	Listeria_monocytogenes_1639_v114	0.9236
Listeria_monocytogenes_1639_v114	Listeria_monocytogenes_1639_v114	0.0683
Listeria_welshimeri_1643	Listeria_monocytogenes_1639_v115	0.888
Listeria_welshimeri_serovar_6b_str_SLCC5334_386043	Listeria_monocytogenes_1639_v115	0.8876
Listeria_seeligeri_serovar_1_2b_str_SLCC3954_683837	Listeria_monocytogenes_1639_v115	0.8828
Listeria_seeligeri_FSL_S4-171_702452	Listeria_monocytogenes_1639_v115	0.8245
Listeria_seeligeri_FSL_N1-067_702453	Listeria_monocytogenes_1639_v115	0.8285
Listeria_seeligeri_1640_v1	Listeria_monocytogenes_1639_v115	0.8939
Listeria_seeligeri_1640	Listeria_monocytogenes_1639_v115	0.8813
Listeria_monocytogenes_WSLC1042_1457188	Listeria_monocytogenes_1639_v115	0.9966
Listeria_monocytogenes_WSLC1001_1457187	Listeria_monocytogenes_1639_v115	0.9254
Listeria_monocytogenes_SLCC7179_879090	Listeria_monocytogenes_1639_v115	0.9358
Listeria_monocytogenes_SLCC5850_882096	Listeria_monocytogenes_1639_v115	0.93
Listeria_monocytogenes_SLCC2755_932919	Listeria_monocytogenes_1639_v115	0.9453
Listeria_monocytogenes_SLCC2540_879089	Listeria_monocytogenes_1639_v115	0.95
Listeria_monocytogenes_SLCC2479_882020	Listeria_monocytogenes_1639_v115	0.9266

Listeria_monocytogenes_SLCC2378_879088	Listeria_monocytogenes_1639_v115	0.9989
Listeria_monocytogenes_SLCC2376_882097	Listeria_monocytogenes_1639_v115	0.9403
Listeria_monocytogenes_SLCC2372_932920	Listeria_monocytogenes_1639_v115	0.9194
Listeria_monocytogenes_serotype_4b_str_LL195_1230340	Listeria_monocytogenes_1639_v115	0.9923
Listeria_monocytogenes_serotype_4b_str_LIS0087_1134669	Listeria_monocytogenes_1639_v115	0.9059
Listeria_monocytogenes_serotype_4b_str_F2365_265669	Listeria_monocytogenes_1639_v115	0.9923
Listeria_monocytogenes_serotype_4b_str_CLIP_80459_568819	Listeria_monocytogenes_1639_v115	0.9724
Listeria_monocytogenes_serotype_4b_str_81-0861_1196161	Listeria_monocytogenes_1639_v115	0.9861
Listeria_monocytogenes_serotype_4b_str_81-0592_1196160	Listeria_monocytogenes_1639_v115	0.9872
Listeria_monocytogenes_serotype_4b_str_81-0558_1196159	Listeria_monocytogenes_1639_v115	0.9864
Listeria_monocytogenes_serotype_4b_str_10-0809_1196162	Listeria_monocytogenes_1639_v115	0.9876
Listeria_monocytogenes_serotype_4b_str_02-6680_1196175	Listeria_monocytogenes_1639_v115	0.9728
Listeria_monocytogenes_serotype_4b_str_02-6679_1196174	Listeria_monocytogenes_1639_v115	0.9728
Listeria_monocytogenes_serotype_4b_str_02-1792_1196173	Listeria_monocytogenes_1639_v115	0.9861
Listeria_monocytogenes_serotype_4b_str_02-1289_1196172	Listeria_monocytogenes_1639_v115	0.9861
Listeria_monocytogenes_serotype_4b_str_02-1103_1196171	Listeria_monocytogenes_1639_v115	0.9861
Listeria_monocytogenes_serotype_3c_str_10-5027_1196195	Listeria_monocytogenes_1639_v115	0.9259
Listeria_monocytogenes_serotype_1_2c_str_10-5026_1196194	Listeria_monocytogenes_1639_v115	0.9266
Listeria_monocytogenes_serotype_1_2c_str_10-5025_1196193	Listeria_monocytogenes_1639_v115	0.9266
Listeria_monocytogenes_serotype_1_2b_str_10-0811_1196164	Listeria_monocytogenes_1639_v115	0.9382
Listeria_monocytogenes_serotype_1_2b_str_10-0810_1196163	Listeria_monocytogenes_1639_v115	0.9382
Listeria_monocytogenes_serotype_1_2a_str_99-6370_1196179	Listeria_monocytogenes_1639_v115	0.912
Listeria_monocytogenes_serotype_1_2a_str_98-2035_1196178	Listeria_monocytogenes_1639_v115	0.912
Listeria_monocytogenes_serotype_1_2a_str_95-0093_1196177	Listeria_monocytogenes_1639_v115	0.9174
Listeria_monocytogenes_serotype_1_2a_str_88-0478_1196176	Listeria_monocytogenes_1639_v115	0.9163
Listeria_monocytogenes_serotype_1_2a_str_10-5024_1196192	Listeria_monocytogenes_1639_v115	0.9174
Listeria_monocytogenes_serotype_1_2a_str_10-4758_1196168	Listeria_monocytogenes_1639_v115	0.9354
Listeria_monocytogenes_serotype_1_2a_str_10-4754_1196167	Listeria_monocytogenes_1639_v115	0.9358
Listeria_monocytogenes_serotype_1_2a_str_10-1321_1196191	Listeria_monocytogenes_1639_v115	0.9174
Listeria_monocytogenes_serotype_1_2a_str_10-1047_1196190	Listeria_monocytogenes_1639_v115	0.9181
Listeria_monocytogenes_serotype_1_2a_str_10-1046_1196189	Listeria_monocytogenes_1639_v115	0.9181
Listeria_monocytogenes_serotype_1_2a_str_10-0934_1196170	Listeria_monocytogenes_1639_v115	0.943
Listeria_monocytogenes_serotype_1_2a_str_10-0933_1196169	Listeria_monocytogenes_1639_v115	0.943
Listeria_monocytogenes_serotype_1_2a_str_10-0815_1196188	Listeria_monocytogenes_1639_v115	0.9134
Listeria_monocytogenes_serotype_1_2a_str_10-0814_1196187	Listeria_monocytogenes_1639_v115	0.9178
Listeria_monocytogenes_serotype_1_2a_str_10-0813_1196166	Listeria_monocytogenes_1639_v115	0.923
Listeria_monocytogenes_serotype_1_2a_str_10-0812_1196165	Listeria_monocytogenes_1639_v115	0.923
Listeria_monocytogenes_serotype_1_2a_str_08-7669_1196186	Listeria_monocytogenes_1639_v115	0.9267
Listeria_monocytogenes_serotype_1_2a_str_08-7374_1196185	Listeria_monocytogenes_1639_v115	0.9174
Listeria_monocytogenes_serotype_1_2a_str_08-6997_1196184	Listeria_monocytogenes_1639_v115	0.9134
Listeria_monocytogenes_serotype_1_2a_str_08-6569_1196183	Listeria_monocytogenes_1639_v115	0.9134
Listeria_monocytogenes_serotype_1_2a_str_08-6056_1196182	Listeria_monocytogenes_1639_v115	0.9134
Listeria_monocytogenes_serotype_1_2a_str_04-5457_1196181	Listeria_monocytogenes_1639_v115	0.9174
Listeria_monocytogenes_serotype_1_2a_str_02-5993_1196180	Listeria_monocytogenes_1639_v115	0.9174
Listeria_monocytogenes_R479a_1437838	Listeria_monocytogenes_1639_v115	0.9167
Listeria_monocytogenes_N53-1_1234142	Listeria_monocytogenes_1639_v115	0.8921
Listeria_monocytogenes_M7_1030009	Listeria_monocytogenes_1639_v115	0.9052
Listeria_monocytogenes_LO28_393132	Listeria_monocytogenes_1639_v115	0.9252
Listeria_monocytogenes_L99_563174	Listeria_monocytogenes_1639_v115	0.9056
Listeria_monocytogenes_L312_882094	Listeria_monocytogenes_1639_v115	0.9728
Listeria_monocytogenes_J1816_930781	Listeria_monocytogenes_1639_v115	0.9493
Listeria_monocytogenes_J1-220_930782	Listeria_monocytogenes_1639_v115	0.9615
Listeria_monocytogenes_J0161_393130	Listeria_monocytogenes_1639_v115	0.9234
Listeria_monocytogenes_HCC23_552536	Listeria_monocytogenes_1639_v115	0.9049
Listeria_monocytogenes_FSL_R2-561_393126	Listeria_monocytogenes_1639_v115	0.9252
Listeria_monocytogenes_FSL_J1-208_393119	Listeria_monocytogenes_1639_v115	0.9196
Listeria_monocytogenes_FSL_J1-175_393118	Listeria_monocytogenes_1639_v115	0.9536
Listeria_monocytogenes_Finland_1998_393127	Listeria_monocytogenes_1639_v115	0.9426
Listeria_monocytogenes_EGD-e_169963_v1	Listeria_monocytogenes_1639_v115	0.9351
Listeria_monocytogenes_EGD-e_169963	Listeria_monocytogenes_1639_v115	0.9355
Listeria_monocytogenes_EGD_1334565	Listeria_monocytogenes_1639_v115	0.9307
Listeria_monocytogenes_CFSAN002349_1299895	Listeria_monocytogenes_1639_v115	0.9728
Listeria_monocytogenes_ATCC_19117_882095_v1	Listeria_monocytogenes_1639_v115	0.982
Listeria_monocytogenes_ATCC_19117_882095	Listeria_monocytogenes_1639_v115	0.9817
Listeria_monocytogenes_6179_1288295	Listeria_monocytogenes_1639_v115	0.9085
Listeria_monocytogenes_1639_v9	Listeria_monocytogenes_1639_v115	0.9243
Listeria_monocytogenes_1639_v99	Listeria_monocytogenes_1639_v115	0.9501
Listeria_monocytogenes_1639_v98	Listeria_monocytogenes_1639_v115	0.9199
Listeria_monocytogenes_1639_v97	Listeria_monocytogenes_1639_v115	0.9644
Listeria_monocytogenes_1639_v96	Listeria_monocytogenes_1639_v115	0.9494
Listeria_monocytogenes_1639_v95	Listeria_monocytogenes_1639_v115	0.9494
Listeria_monocytogenes_1639_v94	Listeria_monocytogenes_1639_v115	0.9497
Listeria_monocytogenes_1639_v93	Listeria_monocytogenes_1639_v115	0.923
Listeria_monocytogenes_1639_v92	Listeria_monocytogenes_1639_v115	0.9497
Listeria_monocytogenes_1639_v91	Listeria_monocytogenes_1639_v115	0.9722
Listeria_monocytogenes_1639_v90	Listeria_monocytogenes_1639_v115	0.9199
Listeria_monocytogenes_1639_v8	Listeria_monocytogenes_1639_v115	0.9077
Listeria_monocytogenes_1639_v89	Listeria_monocytogenes_1639_v115	0.9601
Listeria_monocytogenes_1639_v88	Listeria_monocytogenes_1639_v115	0.9238
Listeria_monocytogenes_1639_v87	Listeria_monocytogenes_1639_v115	0.9189
Listeria_monocytogenes_1639_v86	Listeria_monocytogenes_1639_v115	0.9185
Listeria_monocytogenes_1639_v85	Listeria_monocytogenes_1639_v115	0.9853
Listeria_monocytogenes_1639_v84	Listeria_monocytogenes_1639_v115	0.927
Listeria_monocytogenes_1639_v83	Listeria_monocytogenes_1639_v115	0.9706
Listeria_monocytogenes_1639_v82	Listeria_monocytogenes_1639_v115	0.9698
Listeria_monocytogenes_1639_v81	Listeria_monocytogenes_1639_v115	0.923
Listeria_monocytogenes_1639_v80	Listeria_monocytogenes_1639_v115	0.9444
Listeria_monocytogenes_1639_v7	Listeria_monocytogenes_1639_v115	0.9077
Listeria_monocytogenes_1639_v79	Listeria_monocytogenes_1639_v115	0.9213
Listeria_monocytogenes_1639_v78	Listeria_monocytogenes_1639_v115	0.9224
Listeria_monocytogenes_1639_v77	Listeria_monocytogenes_1639_v115	0.93

Listeria_monocytogenes_1639_v76	Listeria_monocytogenes_1639_v115	0.9195
Listeria_monocytogenes_1639_v75	Listeria_monocytogenes_1639_v115	0.9056
Listeria_monocytogenes_1639_v74	Listeria_monocytogenes_1639_v115	0.9177
Listeria_monocytogenes_1639_v73	Listeria_monocytogenes_1639_v115	0.9208
Listeria_monocytogenes_1639_v72	Listeria_monocytogenes_1639_v115	0.969
Listeria_monocytogenes_1639_v71	Listeria_monocytogenes_1639_v115	0.9068
Listeria_monocytogenes_1639_v70	Listeria_monocytogenes_1639_v115	0.9228
Listeria_monocytogenes_1639_v6	Listeria_monocytogenes_1639_v115	0.9074
Listeria_monocytogenes_1639_v69	Listeria_monocytogenes_1639_v115	0.9872
Listeria_monocytogenes_1639_v68	Listeria_monocytogenes_1639_v115	0.9374
Listeria_monocytogenes_1639_v67	Listeria_monocytogenes_1639_v115	0.9434
Listeria_monocytogenes_1639_v66	Listeria_monocytogenes_1639_v115	0.9313
Listeria_monocytogenes_1639_v65	Listeria_monocytogenes_1639_v115	0.9202
Listeria_monocytogenes_1639_v64	Listeria_monocytogenes_1639_v115	0.9266
Listeria_monocytogenes_1639_v63	Listeria_monocytogenes_1639_v115	0.9396
Listeria_monocytogenes_1639_v62	Listeria_monocytogenes_1639_v115	0.9185
Listeria_monocytogenes_1639_v61	Listeria_monocytogenes_1639_v115	0.9201
Listeria_monocytogenes_1639_v60	Listeria_monocytogenes_1639_v115	0.9253
Listeria_monocytogenes_1639_v5	Listeria_monocytogenes_1639_v115	0.9052
Listeria_monocytogenes_1639_v59	Listeria_monocytogenes_1639_v115	0.9709
Listeria_monocytogenes_1639_v58	Listeria_monocytogenes_1639_v115	0.9773
Listeria_monocytogenes_1639_v57	Listeria_monocytogenes_1639_v115	0.9466
Listeria_monocytogenes_1639_v56	Listeria_monocytogenes_1639_v115	0.9709
Listeria_monocytogenes_1639_v55	Listeria_monocytogenes_1639_v115	0.9423
Listeria_monocytogenes_1639_v54	Listeria_monocytogenes_1639_v115	0.9228
Listeria_monocytogenes_1639_v53	Listeria_monocytogenes_1639_v115	0.9294
Listeria_monocytogenes_1639_v52	Listeria_monocytogenes_1639_v115	0.9575
Listeria_monocytogenes_1639_v51	Listeria_monocytogenes_1639_v115	0.9651
Listeria_monocytogenes_1639_v50	Listeria_monocytogenes_1639_v115	0.9167
Listeria_monocytogenes_1639_v4	Listeria_monocytogenes_1639_v115	0.9184
Listeria_monocytogenes_1639_v49	Listeria_monocytogenes_1639_v115	0.9838
Listeria_monocytogenes_1639_v48	Listeria_monocytogenes_1639_v115	0.9211
Listeria_monocytogenes_1639_v47	Listeria_monocytogenes_1639_v115	0.9171
Listeria_monocytogenes_1639_v46	Listeria_monocytogenes_1639_v115	0.9367
Listeria_monocytogenes_1639_v45	Listeria_monocytogenes_1639_v115	0.9087
Listeria_monocytogenes_1639_v44	Listeria_monocytogenes_1639_v115	0.9174
Listeria_monocytogenes_1639_v43	Listeria_monocytogenes_1639_v115	0.9174
Listeria_monocytogenes_1639_v42	Listeria_monocytogenes_1639_v115	0.9145
Listeria_monocytogenes_1639_v41	Listeria_monocytogenes_1639_v115	0.8986
Listeria_monocytogenes_1639_v40	Listeria_monocytogenes_1639_v115	0.9256
Listeria_monocytogenes_1639_v3	Listeria_monocytogenes_1639_v115	0.9187
Listeria_monocytogenes_1639_v39	Listeria_monocytogenes_1639_v115	0.9422
Listeria_monocytogenes_1639_v38	Listeria_monocytogenes_1639_v115	0.9311
Listeria_monocytogenes_1639_v37	Listeria_monocytogenes_1639_v115	0.9165
Listeria_monocytogenes_1639_v36	Listeria_monocytogenes_1639_v115	0.9152
Listeria_monocytogenes_1639_v35	Listeria_monocytogenes_1639_v115	0.9263
Listeria_monocytogenes_1639_v34	Listeria_monocytogenes_1639_v115	0.9293
Listeria_monocytogenes_1639_v33	Listeria_monocytogenes_1639_v115	0.9382
Listeria_monocytogenes_1639_v32	Listeria_monocytogenes_1639_v115	0.9155
Listeria_monocytogenes_1639_v31	Listeria_monocytogenes_1639_v115	0.9297
Listeria_monocytogenes_1639_v30	Listeria_monocytogenes_1639_v115	0.9407
Listeria_monocytogenes_1639_v2	Listeria_monocytogenes_1639_v115	0.9037
Listeria_monocytogenes_1639_v29	Listeria_monocytogenes_1639_v115	0.9126
Listeria_monocytogenes_1639_v28	Listeria_monocytogenes_1639_v115	0.9166
Listeria_monocytogenes_1639_v27	Listeria_monocytogenes_1639_v115	0.9079
Listeria_monocytogenes_1639_v26	Listeria_monocytogenes_1639_v115	0.9076
Listeria_monocytogenes_1639_v25	Listeria_monocytogenes_1639_v115	0.9076
Listeria_monocytogenes_1639_v24	Listeria_monocytogenes_1639_v115	0.9615
Listeria_monocytogenes_1639_v23	Listeria_monocytogenes_1639_v115	0.9611
Listeria_monocytogenes_1639_v22	Listeria_monocytogenes_1639_v115	0.9407
Listeria_monocytogenes_1639_v21	Listeria_monocytogenes_1639_v115	0.9611
Listeria_monocytogenes_1639_v20	Listeria_monocytogenes_1639_v115	0.9333
Listeria_monocytogenes_1639_v1	Listeria_monocytogenes_1639_v115	0.9026
Listeria_monocytogenes_1639_v19	Listeria_monocytogenes_1639_v115	0.933
Listeria_monocytogenes_1639_v198	Listeria_monocytogenes_1639_v115	0.8948
Listeria_monocytogenes_1639_v197	Listeria_monocytogenes_1639_v115	0.9228
Listeria_monocytogenes_1639_v196	Listeria_monocytogenes_1639_v115	0.9133
Listeria_monocytogenes_1639_v195	Listeria_monocytogenes_1639_v115	0.9429
Listeria_monocytogenes_1639_v194	Listeria_monocytogenes_1639_v115	0.9407
Listeria_monocytogenes_1639_v193	Listeria_monocytogenes_1639_v115	0.9407
Listeria_monocytogenes_1639_v192	Listeria_monocytogenes_1639_v115	0.9099
Listeria_monocytogenes_1639_v191	Listeria_monocytogenes_1639_v115	0.9459
Listeria_monocytogenes_1639_v190	Listeria_monocytogenes_1639_v115	0.9422
Listeria_monocytogenes_1639_v18	Listeria_monocytogenes_1639_v115	0.9406
Listeria_monocytogenes_1639_v189	Listeria_monocytogenes_1639_v115	0.9414
Listeria_monocytogenes_1639_v188	Listeria_monocytogenes_1639_v115	0.9436
Listeria_monocytogenes_1639_v187	Listeria_monocytogenes_1639_v115	0.9421
Listeria_monocytogenes_1639_v186	Listeria_monocytogenes_1639_v115	0.9425
Listeria_monocytogenes_1639_v185	Listeria_monocytogenes_1639_v115	0.9425
Listeria_monocytogenes_1639_v184	Listeria_monocytogenes_1639_v115	0.9414
Listeria_monocytogenes_1639_v183	Listeria_monocytogenes_1639_v115	0.9417
Listeria_monocytogenes_1639_v182	Listeria_monocytogenes_1639_v115	0.944
Listeria_monocytogenes_1639_v181	Listeria_monocytogenes_1639_v115	0.9332
Listeria_monocytogenes_1639_v180	Listeria_monocytogenes_1639_v115	0.9332
Listeria_monocytogenes_1639_v17	Listeria_monocytogenes_1639_v115	0.941
Listeria_monocytogenes_1639_v179	Listeria_monocytogenes_1639_v115	0.9282
Listeria_monocytogenes_1639_v178	Listeria_monocytogenes_1639_v115	0.9445
Listeria_monocytogenes_1639_v177	Listeria_monocytogenes_1639_v115	0.969
Listeria_monocytogenes_1639_v176	Listeria_monocytogenes_1639_v115	0.9389
Listeria_monocytogenes_1639_v175	Listeria_monocytogenes_1639_v115	0.9354
Listeria_monocytogenes_1639_v174	Listeria_monocytogenes_1639_v115	0.9453

Listeria_monocytogenes_1639_v173	Listeria_monocytogenes_1639_v115	0.9669
Listeria_monocytogenes_1639_v172	Listeria_monocytogenes_1639_v115	0.941
Listeria_monocytogenes_1639_v171	Listeria_monocytogenes_1639_v115	0.9279
Listeria_monocytogenes_1639_v170	Listeria_monocytogenes_1639_v115	0.9357
Listeria_monocytogenes_1639_v16	Listeria_monocytogenes_1639_v115	0.9043
Listeria_monocytogenes_1639_v169	Listeria_monocytogenes_1639_v115	0.9376
Listeria_monocytogenes_1639_v168	Listeria_monocytogenes_1639_v115	0.9385
Listeria_monocytogenes_1639_v167	Listeria_monocytogenes_1639_v115	0.9307
Listeria_monocytogenes_1639_v166	Listeria_monocytogenes_1639_v115	0.9388
Listeria_monocytogenes_1639_v165	Listeria_monocytogenes_1639_v115	0.9373
Listeria_monocytogenes_1639_v164	Listeria_monocytogenes_1639_v115	0.9919
Listeria_monocytogenes_1639_v163	Listeria_monocytogenes_1639_v115	0.9582
Listeria_monocytogenes_1639_v162	Listeria_monocytogenes_1639_v115	0.9919
Listeria_monocytogenes_1639_v161	Listeria_monocytogenes_1639_v115	0.9919
Listeria_monocytogenes_1639_v160	Listeria_monocytogenes_1639_v115	0.9916
Listeria_monocytogenes_1639_v15	Listeria_monocytogenes_1639_v115	0.9245
Listeria_monocytogenes_1639_v159	Listeria_monocytogenes_1639_v115	0.9694
Listeria_monocytogenes_1639_v158	Listeria_monocytogenes_1639_v115	0.9923
Listeria_monocytogenes_1639_v157	Listeria_monocytogenes_1639_v115	0.9923
Listeria_monocytogenes_1639_v156	Listeria_monocytogenes_1639_v115	0.9194
Listeria_monocytogenes_1639_v155	Listeria_monocytogenes_1639_v115	0.9317
Listeria_monocytogenes_1639_v154	Listeria_monocytogenes_1639_v115	0.9356
Listeria_monocytogenes_1639_v153	Listeria_monocytogenes_1639_v115	0.9324
Listeria_monocytogenes_1639_v152	Listeria_monocytogenes_1639_v115	0.9694
Listeria_monocytogenes_1639_v151	Listeria_monocytogenes_1639_v115	0.9694
Listeria_monocytogenes_1639_v150	Listeria_monocytogenes_1639_v115	0.9321
Listeria_monocytogenes_1639_v14	Listeria_monocytogenes_1639_v115	0.931
Listeria_monocytogenes_1639_v149	Listeria_monocytogenes_1639_v115	0.9311
Listeria_monocytogenes_1639_v148	Listeria_monocytogenes_1639_v115	0.9683
Listeria_monocytogenes_1639_v147	Listeria_monocytogenes_1639_v115	0.9548
Listeria_monocytogenes_1639_v146	Listeria_monocytogenes_1639_v115	0.9466
Listeria_monocytogenes_1639_v145	Listeria_monocytogenes_1639_v115	0.9432
Listeria_monocytogenes_1639_v144	Listeria_monocytogenes_1639_v115	0.972
Listeria_monocytogenes_1639_v143	Listeria_monocytogenes_1639_v115	0.9482
Listeria_monocytogenes_1639_v142	Listeria_monocytogenes_1639_v115	0.9724
Listeria_monocytogenes_1639_v141	Listeria_monocytogenes_1639_v115	0.9647
Listeria_monocytogenes_1639_v140	Listeria_monocytogenes_1639_v115	0.9731
Listeria_monocytogenes_1639_v13	Listeria_monocytogenes_1639_v115	0.9241
Listeria_monocytogenes_1639_v139	Listeria_monocytogenes_1639_v115	0.9674
Listeria_monocytogenes_1639_v138	Listeria_monocytogenes_1639_v115	0.9755
Listeria_monocytogenes_1639_v137	Listeria_monocytogenes_1639_v115	0.9755
Listeria_monocytogenes_1639_v136	Listeria_monocytogenes_1639_v115	0.9351
Listeria_monocytogenes_1639_v135	Listeria_monocytogenes_1639_v115	0.9194
Listeria_monocytogenes_1639_v134	Listeria_monocytogenes_1639_v115	0.9962
Listeria_monocytogenes_1639_v133	Listeria_monocytogenes_1639_v115	0.934
Listeria_monocytogenes_1639_v132	Listeria_monocytogenes_1639_v115	0.9176
Listeria_monocytogenes_1639_v131	Listeria_monocytogenes_1639_v115	0.9572
Listeria_monocytogenes_1639_v130	Listeria_monocytogenes_1639_v115	0.9267
Listeria_monocytogenes_1639_v12	Listeria_monocytogenes_1639_v115	0.9066
Listeria_monocytogenes_1639_v129	Listeria_monocytogenes_1639_v115	0.9639
Listeria_monocytogenes_1639_v128	Listeria_monocytogenes_1639_v115	0.9625
Listeria_monocytogenes_1639_v127	Listeria_monocytogenes_1639_v115	0.9317
Listeria_monocytogenes_1639_v126	Listeria_monocytogenes_1639_v115	0.9606
Listeria_monocytogenes_1639_v125	Listeria_monocytogenes_1639_v115	0.9303
Listeria_monocytogenes_1639_v124	Listeria_monocytogenes_1639_v115	0.9444
Listeria_monocytogenes_1639_v123	Listeria_monocytogenes_1639_v115	0.9728
Listeria_monocytogenes_1639_v122	Listeria_monocytogenes_1639_v115	0.9728
Listeria_monocytogenes_1639_v121	Listeria_monocytogenes_1639_v115	0.9728
Listeria_monocytogenes_1639_v120	Listeria_monocytogenes_1639_v115	0.9728
Listeria_monocytogenes_1639_v11	Listeria_monocytogenes_1639_v115	0.9053
Listeria_monocytogenes_1639_v119	Listeria_monocytogenes_1639_v115	0.9728
Listeria_monocytogenes_1639_v118	Listeria_monocytogenes_1639_v115	0.9728
Listeria_monocytogenes_1639_v117	Listeria_monocytogenes_1639_v115	0.9354
Listeria_monocytogenes_1639_v116	Listeria_monocytogenes_1639_v115	0.9977
Listeria_monocytogenes_1639_v115	Listeria_monocytogenes_1639_v115	0.0659
Listeria_welshimeri_1643	Listeria_monocytogenes_1639_v116	0.8876
Listeria_welshimeri_serovar_6b_str_SLCC5334_386043	Listeria_monocytogenes_1639_v116	0.8873
Listeria_seeligeri_serovar_1_2b_str_SLCC3954_683837	Listeria_monocytogenes_1639_v116	0.8829
Listeria_seeligeri_FSL_S4-171_702452	Listeria_monocytogenes_1639_v116	0.8242
Listeria_seeligeri_FSL_N1-067_702453	Listeria_monocytogenes_1639_v116	0.8282
Listeria_seeligeri_1640_v1	Listeria_monocytogenes_1639_v116	0.894
Listeria_seeligeri_1640	Listeria_monocytogenes_1639_v116	0.881
Listeria_monocytogenes_WSLC1042_1457188	Listeria_monocytogenes_1639_v116	0.9962
Listeria_monocytogenes_WSLC1001_1457187	Listeria_monocytogenes_1639_v116	0.9258
Listeria_monocytogenes_SLCC7179_879090	Listeria_monocytogenes_1639_v116	0.9358
Listeria_monocytogenes_SLCC5850_882096	Listeria_monocytogenes_1639_v116	0.9304
Listeria_monocytogenes_SLCC2755_932919	Listeria_monocytogenes_1639_v116	0.9457
Listeria_monocytogenes_SLCC2540_879089	Listeria_monocytogenes_1639_v116	0.9496
Listeria_monocytogenes_SLCC2479_882020	Listeria_monocytogenes_1639_v116	0.9263
Listeria_monocytogenes_SLCC2378_879088	Listeria_monocytogenes_1639_v116	0.9985
Listeria_monocytogenes_SLCC2376_882097	Listeria_monocytogenes_1639_v116	0.9399
Listeria_monocytogenes_SLCC2372_932920	Listeria_monocytogenes_1639_v116	0.919
Listeria_monocytogenes_serotype_4b_str_LL195_1230340	Listeria_monocytogenes_1639_v116	0.992
Listeria_monocytogenes_serotype_4b_str_LIS0087_1134669	Listeria_monocytogenes_1639_v116	0.9063
Listeria_monocytogenes_serotype_4b_str_F2365_265669	Listeria_monocytogenes_1639_v116	0.992
Listeria_monocytogenes_serotype_4b_str_CLIP_80459_568819	Listeria_monocytogenes_1639_v116	0.972
Listeria_monocytogenes_serotype_4b_str_81-0861_1196161	Listeria_monocytogenes_1639_v116	0.9857
Listeria_monocytogenes_serotype_4b_str_81-0592_1196160	Listeria_monocytogenes_1639_v116	0.9868
Listeria_monocytogenes_serotype_4b_str_81-0558_1196159	Listeria_monocytogenes_1639_v116	0.9861
Listeria_monocytogenes_serotype_4b_str_10-0809_1196162	Listeria_monocytogenes_1639_v116	0.9872
Listeria_monocytogenes_serotype_4b_str_02-6680_1196175	Listeria_monocytogenes_1639_v116	0.9728

Listeria_monocytogenes_serotype_4b_str_02-6679_1196174	Listeria_monocytogenes_1639_v116	0.9728
Listeria_monocytogenes_serotype_4b_str_02-1792_1196173	Listeria_monocytogenes_1639_v116	0.9857
Listeria_monocytogenes_serotype_4b_str_02-1289_1196172	Listeria_monocytogenes_1639_v116	0.9857
Listeria_monocytogenes_serotype_4b_str_02-1103_1196171	Listeria_monocytogenes_1639_v116	0.9857
Listeria_monocytogenes_serotype_3c_str_10-5027_1196195	Listeria_monocytogenes_1639_v116	0.9255
Listeria_monocytogenes_serotype_1_2c_str_10-5026_1196194	Listeria_monocytogenes_1639_v116	0.9263
Listeria_monocytogenes_serotype_1_2c_str_10-5025_1196193	Listeria_monocytogenes_1639_v116	0.9263
Listeria_monocytogenes_serotype_1_2b_str_10-0811_1196164	Listeria_monocytogenes_1639_v116	0.9382
Listeria_monocytogenes_serotype_1_2b_str_10-0810_1196163	Listeria_monocytogenes_1639_v116	0.9382
Listeria_monocytogenes_serotype_1_2a_str_99-6370_1196179	Listeria_monocytogenes_1639_v116	0.9117
Listeria_monocytogenes_serotype_1_2a_str_98-2035_1196178	Listeria_monocytogenes_1639_v116	0.9117
Listeria_monocytogenes_serotype_1_2a_str_95-0093_1196177	Listeria_monocytogenes_1639_v116	0.9171
Listeria_monocytogenes_serotype_1_2a_str_88-0478_1196176	Listeria_monocytogenes_1639_v116	0.9159
Listeria_monocytogenes_serotype_1_2a_str_10-5024_1196192	Listeria_monocytogenes_1639_v116	0.9171
Listeria_monocytogenes_serotype_1_2a_str_10-4758_1196168	Listeria_monocytogenes_1639_v116	0.9354
Listeria_monocytogenes_serotype_1_2a_str_10-4754_1196167	Listeria_monocytogenes_1639_v116	0.9358
Listeria_monocytogenes_serotype_1_2a_str_10-1321_1196191	Listeria_monocytogenes_1639_v116	0.9171
Listeria_monocytogenes_serotype_1_2a_str_10-1047_1196190	Listeria_monocytogenes_1639_v116	0.9178
Listeria_monocytogenes_serotype_1_2a_str_10-1046_1196189	Listeria_monocytogenes_1639_v116	0.9178
Listeria_monocytogenes_serotype_1_2a_str_10-0934_1196170	Listeria_monocytogenes_1639_v116	0.943
Listeria_monocytogenes_serotype_1_2a_str_10-0933_1196169	Listeria_monocytogenes_1639_v116	0.943
Listeria_monocytogenes_serotype_1_2a_str_10-0815_1196188	Listeria_monocytogenes_1639_v116	0.913
Listeria_monocytogenes_serotype_1_2a_str_10-0814_1196187	Listeria_monocytogenes_1639_v116	0.9174
Listeria_monocytogenes_serotype_1_2a_str_10-0813_1196166	Listeria_monocytogenes_1639_v116	0.9234
Listeria_monocytogenes_serotype_1_2a_str_10-0812_1196165	Listeria_monocytogenes_1639_v116	0.9234
Listeria_monocytogenes_serotype_1_2a_str_08-7669_1196186	Listeria_monocytogenes_1639_v116	0.9264
Listeria_monocytogenes_serotype_1_2a_str_08-7374_1196185	Listeria_monocytogenes_1639_v116	0.9171
Listeria_monocytogenes_serotype_1_2a_str_08-6997_1196184	Listeria_monocytogenes_1639_v116	0.913
Listeria_monocytogenes_serotype_1_2a_str_08-6569_1196183	Listeria_monocytogenes_1639_v116	0.913
Listeria_monocytogenes_serotype_1_2a_str_08-6056_1196182	Listeria_monocytogenes_1639_v116	0.913
Listeria_monocytogenes_serotype_1_2a_str_04-5457_1196181	Listeria_monocytogenes_1639_v116	0.9171
Listeria_monocytogenes_serotype_1_2a_str_02-5993_1196180	Listeria_monocytogenes_1639_v116	0.9171
Listeria_monocytogenes_R479a_1437838	Listeria_monocytogenes_1639_v116	0.9164
Listeria_monocytogenes_N53-1_1234142	Listeria_monocytogenes_1639_v116	0.8907
Listeria_monocytogenes_M7_1030009	Listeria_monocytogenes_1639_v116	0.9049
Listeria_monocytogenes_LO28_393132	Listeria_monocytogenes_1639_v116	0.9249
Listeria_monocytogenes_L99_563174	Listeria_monocytogenes_1639_v116	0.9052
Listeria_monocytogenes_L312_882094	Listeria_monocytogenes_1639_v116	0.9724
Listeria_monocytogenes_J1816_930781	Listeria_monocytogenes_1639_v116	0.949
Listeria_monocytogenes_J1-220_930782	Listeria_monocytogenes_1639_v116	0.9615
Listeria_monocytogenes_J0161_393130	Listeria_monocytogenes_1639_v116	0.9234
Listeria_monocytogenes_HCC23_552536	Listeria_monocytogenes_1639_v116	0.9045
Listeria_monocytogenes_FSL_R2-561_393126	Listeria_monocytogenes_1639_v116	0.9249
Listeria_monocytogenes_FSL_J1-208_393119	Listeria_monocytogenes_1639_v116	0.9193
Listeria_monocytogenes_FSL_J1-175_393118	Listeria_monocytogenes_1639_v116	0.9536
Listeria_monocytogenes_Finland_1998_393127	Listeria_monocytogenes_1639_v116	0.9422
Listeria_monocytogenes_EGD-e_169963_v1	Listeria_monocytogenes_1639_v116	0.9347
Listeria_monocytogenes_EGD-e_169963	Listeria_monocytogenes_1639_v116	0.9351
Listeria_monocytogenes_EGD_1334565	Listeria_monocytogenes_1639_v116	0.9311
Listeria_monocytogenes_CFSAN002349_1299895	Listeria_monocytogenes_1639_v116	0.9725
Listeria_monocytogenes_ATCC_19117_882095_v1	Listeria_monocytogenes_1639_v116	0.9821
Listeria_monocytogenes_ATCC_19117_882095	Listeria_monocytogenes_1639_v116	0.9817
Listeria_monocytogenes_6179_1288295	Listeria_monocytogenes_1639_v116	0.9086
Listeria_monocytogenes_1639_v9	Listeria_monocytogenes_1639_v116	0.9247
Listeria_monocytogenes_1639_v99	Listeria_monocytogenes_1639_v116	0.9497
Listeria_monocytogenes_1639_v98	Listeria_monocytogenes_1639_v116	0.9199
Listeria_monocytogenes_1639_v97	Listeria_monocytogenes_1639_v116	0.9644
Listeria_monocytogenes_1639_v96	Listeria_monocytogenes_1639_v116	0.949
Listeria_monocytogenes_1639_v95	Listeria_monocytogenes_1639_v116	0.949
Listeria_monocytogenes_1639_v94	Listeria_monocytogenes_1639_v116	0.9494
Listeria_monocytogenes_1639_v93	Listeria_monocytogenes_1639_v116	0.9234
Listeria_monocytogenes_1639_v92	Listeria_monocytogenes_1639_v116	0.9494
Listeria_monocytogenes_1639_v91	Listeria_monocytogenes_1639_v116	0.9718
Listeria_monocytogenes_1639_v90	Listeria_monocytogenes_1639_v116	0.9199
Listeria_monocytogenes_1639_v8	Listeria_monocytogenes_1639_v116	0.9074
Listeria_monocytogenes_1639_v89	Listeria_monocytogenes_1639_v116	0.9597
Listeria_monocytogenes_1639_v88	Listeria_monocytogenes_1639_v116	0.9239
Listeria_monocytogenes_1639_v87	Listeria_monocytogenes_1639_v116	0.9193
Listeria_monocytogenes_1639_v86	Listeria_monocytogenes_1639_v116	0.9189
Listeria_monocytogenes_1639_v85	Listeria_monocytogenes_1639_v116	0.985
Listeria_monocytogenes_1639_v84	Listeria_monocytogenes_1639_v116	0.9271
Listeria_monocytogenes_1639_v83	Listeria_monocytogenes_1639_v116	0.9706
Listeria_monocytogenes_1639_v82	Listeria_monocytogenes_1639_v116	0.9698
Listeria_monocytogenes_1639_v81	Listeria_monocytogenes_1639_v116	0.9227
Listeria_monocytogenes_1639_v80	Listeria_monocytogenes_1639_v116	0.9444
Listeria_monocytogenes_1639_v7	Listeria_monocytogenes_1639_v116	0.9074
Listeria_monocytogenes_1639_v79	Listeria_monocytogenes_1639_v116	0.921
Listeria_monocytogenes_1639_v78	Listeria_monocytogenes_1639_v116	0.922
Listeria_monocytogenes_1639_v77	Listeria_monocytogenes_1639_v116	0.9296
Listeria_monocytogenes_1639_v76	Listeria_monocytogenes_1639_v116	0.9196
Listeria_monocytogenes_1639_v75	Listeria_monocytogenes_1639_v116	0.9052
Listeria_monocytogenes_1639_v74	Listeria_monocytogenes_1639_v116	0.9181
Listeria_monocytogenes_1639_v73	Listeria_monocytogenes_1639_v116	0.9204
Listeria_monocytogenes_1639_v72	Listeria_monocytogenes_1639_v116	0.969
Listeria_monocytogenes_1639_v71	Listeria_monocytogenes_1639_v116	0.9064
Listeria_monocytogenes_1639_v70	Listeria_monocytogenes_1639_v116	0.9225
Listeria_monocytogenes_1639_v6	Listeria_monocytogenes_1639_v116	0.907
Listeria_monocytogenes_1639_v69	Listeria_monocytogenes_1639_v116	0.9868
Listeria_monocytogenes_1639_v68	Listeria_monocytogenes_1639_v116	0.9374
Listeria_monocytogenes_1639_v67	Listeria_monocytogenes_1639_v116	0.9434
Listeria_monocytogenes_1639_v66	Listeria_monocytogenes_1639_v116	0.9313

Listeria_monocytogenes_1639_v65	Listeria_monocytogenes_1639_v116	0.9198
Listeria_monocytogenes_1639_v64	Listeria_monocytogenes_1639_v116	0.9266
Listeria_monocytogenes_1639_v63	Listeria_monocytogenes_1639_v116	0.9396
Listeria_monocytogenes_1639_v62	Listeria_monocytogenes_1639_v116	0.9182
Listeria_monocytogenes_1639_v61	Listeria_monocytogenes_1639_v116	0.9198
Listeria_monocytogenes_1639_v60	Listeria_monocytogenes_1639_v116	0.9253
Listeria_monocytogenes_1639_v5	Listeria_monocytogenes_1639_v116	0.9052
Listeria_monocytogenes_1639_v59	Listeria_monocytogenes_1639_v116	0.9709
Listeria_monocytogenes_1639_v58	Listeria_monocytogenes_1639_v116	0.9777
Listeria_monocytogenes_1639_v57	Listeria_monocytogenes_1639_v116	0.9462
Listeria_monocytogenes_1639_v56	Listeria_monocytogenes_1639_v116	0.9709
Listeria_monocytogenes_1639_v55	Listeria_monocytogenes_1639_v116	0.942
Listeria_monocytogenes_1639_v54	Listeria_monocytogenes_1639_v116	0.9228
Listeria_monocytogenes_1639_v53	Listeria_monocytogenes_1639_v116	0.9294
Listeria_monocytogenes_1639_v52	Listeria_monocytogenes_1639_v116	0.9575
Listeria_monocytogenes_1639_v51	Listeria_monocytogenes_1639_v116	0.9651
Listeria_monocytogenes_1639_v50	Listeria_monocytogenes_1639_v116	0.9163
Listeria_monocytogenes_1639_v4	Listeria_monocytogenes_1639_v116	0.9184
Listeria_monocytogenes_1639_v49	Listeria_monocytogenes_1639_v116	0.9834
Listeria_monocytogenes_1639_v48	Listeria_monocytogenes_1639_v116	0.9207
Listeria_monocytogenes_1639_v47	Listeria_monocytogenes_1639_v116	0.9167
Listeria_monocytogenes_1639_v46	Listeria_monocytogenes_1639_v116	0.9367
Listeria_monocytogenes_1639_v45	Listeria_monocytogenes_1639_v116	0.9083
Listeria_monocytogenes_1639_v44	Listeria_monocytogenes_1639_v116	0.9171
Listeria_monocytogenes_1639_v43	Listeria_monocytogenes_1639_v116	0.9171
Listeria_monocytogenes_1639_v42	Listeria_monocytogenes_1639_v116	0.9146
Listeria_monocytogenes_1639_v41	Listeria_monocytogenes_1639_v116	0.8987
Listeria_monocytogenes_1639_v40	Listeria_monocytogenes_1639_v116	0.9252
Listeria_monocytogenes_1639_v3	Listeria_monocytogenes_1639_v116	0.9188
Listeria_monocytogenes_1639_v39	Listeria_monocytogenes_1639_v116	0.9422
Listeria_monocytogenes_1639_v38	Listeria_monocytogenes_1639_v116	0.9307
Listeria_monocytogenes_1639_v37	Listeria_monocytogenes_1639_v116	0.9162
Listeria_monocytogenes_1639_v36	Listeria_monocytogenes_1639_v116	0.9148
Listeria_monocytogenes_1639_v35	Listeria_monocytogenes_1639_v116	0.9259
Listeria_monocytogenes_1639_v34	Listeria_monocytogenes_1639_v116	0.9293
Listeria_monocytogenes_1639_v33	Listeria_monocytogenes_1639_v116	0.9382
Listeria_monocytogenes_1639_v32	Listeria_monocytogenes_1639_v116	0.9152
Listeria_monocytogenes_1639_v31	Listeria_monocytogenes_1639_v116	0.9297
Listeria_monocytogenes_1639_v30	Listeria_monocytogenes_1639_v116	0.9407
Listeria_monocytogenes_1639_v2	Listeria_monocytogenes_1639_v116	0.9037
Listeria_monocytogenes_1639_v29	Listeria_monocytogenes_1639_v116	0.9123
Listeria_monocytogenes_1639_v28	Listeria_monocytogenes_1639_v116	0.9163
Listeria_monocytogenes_1639_v27	Listeria_monocytogenes_1639_v116	0.9076
Listeria_monocytogenes_1639_v26	Listeria_monocytogenes_1639_v116	0.9073
Listeria_monocytogenes_1639_v25	Listeria_monocytogenes_1639_v116	0.9073
Listeria_monocytogenes_1639_v24	Listeria_monocytogenes_1639_v116	0.9615
Listeria_monocytogenes_1639_v23	Listeria_monocytogenes_1639_v116	0.9611
Listeria_monocytogenes_1639_v22	Listeria_monocytogenes_1639_v116	0.9408
Listeria_monocytogenes_1639_v21	Listeria_monocytogenes_1639_v116	0.9611
Listeria_monocytogenes_1639_v20	Listeria_monocytogenes_1639_v116	0.9334
Listeria_monocytogenes_1639_v1	Listeria_monocytogenes_1639_v116	0.9027
Listeria_monocytogenes_1639_v19	Listeria_monocytogenes_1639_v116	0.933
Listeria_monocytogenes_1639_v198	Listeria_monocytogenes_1639_v116	0.8934
Listeria_monocytogenes_1639_v197	Listeria_monocytogenes_1639_v116	0.9224
Listeria_monocytogenes_1639_v196	Listeria_monocytogenes_1639_v116	0.9137
Listeria_monocytogenes_1639_v195	Listeria_monocytogenes_1639_v116	0.9426
Listeria_monocytogenes_1639_v194	Listeria_monocytogenes_1639_v116	0.9403
Listeria_monocytogenes_1639_v193	Listeria_monocytogenes_1639_v116	0.9403
Listeria_monocytogenes_1639_v192	Listeria_monocytogenes_1639_v116	0.9096
Listeria_monocytogenes_1639_v191	Listeria_monocytogenes_1639_v116	0.946
Listeria_monocytogenes_1639_v190	Listeria_monocytogenes_1639_v116	0.9426
Listeria_monocytogenes_1639_v18	Listeria_monocytogenes_1639_v116	0.9403
Listeria_monocytogenes_1639_v189	Listeria_monocytogenes_1639_v116	0.9418
Listeria_monocytogenes_1639_v188	Listeria_monocytogenes_1639_v116	0.9436
Listeria_monocytogenes_1639_v187	Listeria_monocytogenes_1639_v116	0.9425
Listeria_monocytogenes_1639_v186	Listeria_monocytogenes_1639_v116	0.9429
Listeria_monocytogenes_1639_v185	Listeria_monocytogenes_1639_v116	0.9429
Listeria_monocytogenes_1639_v184	Listeria_monocytogenes_1639_v116	0.9418
Listeria_monocytogenes_1639_v183	Listeria_monocytogenes_1639_v116	0.9418
Listeria_monocytogenes_1639_v182	Listeria_monocytogenes_1639_v116	0.944
Listeria_monocytogenes_1639_v181	Listeria_monocytogenes_1639_v116	0.9329
Listeria_monocytogenes_1639_v180	Listeria_monocytogenes_1639_v116	0.9329
Listeria_monocytogenes_1639_v17	Listeria_monocytogenes_1639_v116	0.9406
Listeria_monocytogenes_1639_v179	Listeria_monocytogenes_1639_v116	0.9282
Listeria_monocytogenes_1639_v178	Listeria_monocytogenes_1639_v116	0.9445
Listeria_monocytogenes_1639_v177	Listeria_monocytogenes_1639_v116	0.9686
Listeria_monocytogenes_1639_v176	Listeria_monocytogenes_1639_v116	0.9389
Listeria_monocytogenes_1639_v175	Listeria_monocytogenes_1639_v116	0.9354
Listeria_monocytogenes_1639_v174	Listeria_monocytogenes_1639_v116	0.945
Listeria_monocytogenes_1639_v173	Listeria_monocytogenes_1639_v116	0.9669
Listeria_monocytogenes_1639_v172	Listeria_monocytogenes_1639_v116	0.9406
Listeria_monocytogenes_1639_v171	Listeria_monocytogenes_1639_v116	0.9283
Listeria_monocytogenes_1639_v170	Listeria_monocytogenes_1639_v116	0.9361
Listeria_monocytogenes_1639_v16	Listeria_monocytogenes_1639_v116	0.904
Listeria_monocytogenes_1639_v169	Listeria_monocytogenes_1639_v116	0.9377
Listeria_monocytogenes_1639_v168	Listeria_monocytogenes_1639_v116	0.9385
Listeria_monocytogenes_1639_v167	Listeria_monocytogenes_1639_v116	0.9307
Listeria_monocytogenes_1639_v166	Listeria_monocytogenes_1639_v116	0.9388
Listeria_monocytogenes_1639_v165	Listeria_monocytogenes_1639_v116	0.9373
Listeria_monocytogenes_1639_v164	Listeria_monocytogenes_1639_v116	0.9916
Listeria_monocytogenes_1639_v163	Listeria_monocytogenes_1639_v116	0.9582

Listeria_monocytogenes_1639_v162	Listeria_monocytogenes_1639_v116	0.9916
Listeria_monocytogenes_1639_v161	Listeria_monocytogenes_1639_v116	0.9916
Listeria_monocytogenes_1639_v160	Listeria_monocytogenes_1639_v116	0.9912
Listeria_monocytogenes_1639_v15	Listeria_monocytogenes_1639_v116	0.9245
Listeria_monocytogenes_1639_v159	Listeria_monocytogenes_1639_v116	0.969
Listeria_monocytogenes_1639_v158	Listeria_monocytogenes_1639_v116	0.992
Listeria_monocytogenes_1639_v157	Listeria_monocytogenes_1639_v116	0.992
Listeria_monocytogenes_1639_v156	Listeria_monocytogenes_1639_v116	0.9198
Listeria_monocytogenes_1639_v155	Listeria_monocytogenes_1639_v116	0.9317
Listeria_monocytogenes_1639_v154	Listeria_monocytogenes_1639_v116	0.9352
Listeria_monocytogenes_1639_v153	Listeria_monocytogenes_1639_v116	0.9325
Listeria_monocytogenes_1639_v152	Listeria_monocytogenes_1639_v116	0.969
Listeria_monocytogenes_1639_v151	Listeria_monocytogenes_1639_v116	0.969
Listeria_monocytogenes_1639_v150	Listeria_monocytogenes_1639_v116	0.9325
Listeria_monocytogenes_1639_v14	Listeria_monocytogenes_1639_v116	0.931
Listeria_monocytogenes_1639_v149	Listeria_monocytogenes_1639_v116	0.9315
Listeria_monocytogenes_1639_v148	Listeria_monocytogenes_1639_v116	0.9679
Listeria_monocytogenes_1639_v147	Listeria_monocytogenes_1639_v116	0.9544
Listeria_monocytogenes_1639_v146	Listeria_monocytogenes_1639_v116	0.9466
Listeria_monocytogenes_1639_v145	Listeria_monocytogenes_1639_v116	0.9433
Listeria_monocytogenes_1639_v144	Listeria_monocytogenes_1639_v116	0.9716
Listeria_monocytogenes_1639_v143	Listeria_monocytogenes_1639_v116	0.9482
Listeria_monocytogenes_1639_v142	Listeria_monocytogenes_1639_v116	0.972
Listeria_monocytogenes_1639_v141	Listeria_monocytogenes_1639_v116	0.9643
Listeria_monocytogenes_1639_v140	Listeria_monocytogenes_1639_v116	0.9728
Listeria_monocytogenes_1639_v13	Listeria_monocytogenes_1639_v116	0.9241
Listeria_monocytogenes_1639_v139	Listeria_monocytogenes_1639_v116	0.9674
Listeria_monocytogenes_1639_v138	Listeria_monocytogenes_1639_v116	0.9751
Listeria_monocytogenes_1639_v137	Listeria_monocytogenes_1639_v116	0.9751
Listeria_monocytogenes_1639_v136	Listeria_monocytogenes_1639_v116	0.9351
Listeria_monocytogenes_1639_v135	Listeria_monocytogenes_1639_v116	0.9191
Listeria_monocytogenes_1639_v134	Listeria_monocytogenes_1639_v116	0.9958
Listeria_monocytogenes_1639_v133	Listeria_monocytogenes_1639_v116	0.9341
Listeria_monocytogenes_1639_v132	Listeria_monocytogenes_1639_v116	0.9176
Listeria_monocytogenes_1639_v131	Listeria_monocytogenes_1639_v116	0.9568
Listeria_monocytogenes_1639_v130	Listeria_monocytogenes_1639_v116	0.9263
Listeria_monocytogenes_1639_v12	Listeria_monocytogenes_1639_v116	0.9062
Listeria_monocytogenes_1639_v129	Listeria_monocytogenes_1639_v116	0.9639
Listeria_monocytogenes_1639_v128	Listeria_monocytogenes_1639_v116	0.9625
Listeria_monocytogenes_1639_v127	Listeria_monocytogenes_1639_v116	0.9313
Listeria_monocytogenes_1639_v126	Listeria_monocytogenes_1639_v116	0.9606
Listeria_monocytogenes_1639_v125	Listeria_monocytogenes_1639_v116	0.9303
Listeria_monocytogenes_1639_v124	Listeria_monocytogenes_1639_v116	0.9444
Listeria_monocytogenes_1639_v123	Listeria_monocytogenes_1639_v116	0.9725
Listeria_monocytogenes_1639_v122	Listeria_monocytogenes_1639_v116	0.9725
Listeria_monocytogenes_1639_v121	Listeria_monocytogenes_1639_v116	0.9725
Listeria_monocytogenes_1639_v120	Listeria_monocytogenes_1639_v116	0.9725
Listeria_monocytogenes_1639_v11	Listeria_monocytogenes_1639_v116	0.9053
Listeria_monocytogenes_1639_v119	Listeria_monocytogenes_1639_v116	0.9725
Listeria_monocytogenes_1639_v118	Listeria_monocytogenes_1639_v116	0.9725
Listeria_monocytogenes_1639_v117	Listeria_monocytogenes_1639_v116	0.9358
Listeria_monocytogenes_1639_v116	Listeria_monocytogenes_1639_v116	0.0654
Listeria_welshimeri_1643	Listeria_monocytogenes_1639_v117	0.8737
Listeria_welshimeri_serovar_6b_str_SLCC5334_386043	Listeria_monocytogenes_1639_v117	0.873
Listeria_seeligeri_serovar_1_2b_str_SLCC3954_683837	Listeria_monocytogenes_1639_v117	0.8851
Listeria_seeligeri_FSL_S4-171_702452	Listeria_monocytogenes_1639_v117	0.8131
Listeria_seeligeri_FSL_N1-067_702453	Listeria_monocytogenes_1639_v117	0.832
Listeria_seeligeri_1640_v1	Listeria_monocytogenes_1639_v117	0.8854
Listeria_seeligeri_1640	Listeria_monocytogenes_1639_v117	0.8771
Listeria_monocytogenes_WSLC1042_1457188	Listeria_monocytogenes_1639_v117	0.9344
Listeria_monocytogenes_WSLC1001_1457187	Listeria_monocytogenes_1639_v117	0.9618
Listeria_monocytogenes_SLCC7179_879090	Listeria_monocytogenes_1639_v117	0.955
Listeria_monocytogenes_SLCC5850_882096	Listeria_monocytogenes_1639_v117	0.9636
Listeria_monocytogenes_SLCC2755_932919	Listeria_monocytogenes_1639_v117	0.9334
Listeria_monocytogenes_SLCC2540_879089	Listeria_monocytogenes_1639_v117	0.9482
Listeria_monocytogenes_SLCC2479_882020	Listeria_monocytogenes_1639_v117	0.9568
Listeria_monocytogenes_SLCC2378_879088	Listeria_monocytogenes_1639_v117	0.9362
Listeria_monocytogenes_SLCC2376_882097	Listeria_monocytogenes_1639_v117	0.937
Listeria_monocytogenes_SLCC2372_932920	Listeria_monocytogenes_1639_v117	0.9501
Listeria_monocytogenes_serotype_4b_str_LL195_1230340	Listeria_monocytogenes_1639_v117	0.9415
Listeria_monocytogenes_serotype_4b_str_LIS0087_1134669	Listeria_monocytogenes_1639_v117	0.9406
Listeria_monocytogenes_serotype_4b_str_F2365_265669	Listeria_monocytogenes_1639_v117	0.9415
Listeria_monocytogenes_serotype_4b_str_CLIP_80459_568819	Listeria_monocytogenes_1639_v117	0.941
Listeria_monocytogenes_serotype_4b_str_81-0861_1196161	Listeria_monocytogenes_1639_v117	0.9267
Listeria_monocytogenes_serotype_4b_str_81-0592_1196160	Listeria_monocytogenes_1639_v117	0.9278
Listeria_monocytogenes_serotype_4b_str_81-0558_1196159	Listeria_monocytogenes_1639_v117	0.9278
Listeria_monocytogenes_serotype_4b_str_10-0809_1196162	Listeria_monocytogenes_1639_v117	0.9278
Listeria_monocytogenes_serotype_4b_str_02-6680_1196175	Listeria_monocytogenes_1639_v117	0.9389
Listeria_monocytogenes_serotype_4b_str_02-6679_1196174	Listeria_monocytogenes_1639_v117	0.9389
Listeria_monocytogenes_serotype_4b_str_02-1792_1196173	Listeria_monocytogenes_1639_v117	0.9285
Listeria_monocytogenes_serotype_4b_str_02-1289_1196172	Listeria_monocytogenes_1639_v117	0.9285
Listeria_monocytogenes_serotype_4b_str_02-1103_1196171	Listeria_monocytogenes_1639_v117	0.9285
Listeria_monocytogenes_serotype_3c_str_10-5027_1196195	Listeria_monocytogenes_1639_v117	0.958
Listeria_monocytogenes_serotype_1_2c_str_10-5026_1196194	Listeria_monocytogenes_1639_v117	0.9583
Listeria_monocytogenes_serotype_1_2c_str_10-5025_1196193	Listeria_monocytogenes_1639_v117	0.9583
Listeria_monocytogenes_serotype_1_2b_str_10-0811_1196164	Listeria_monocytogenes_1639_v117	0.9432
Listeria_monocytogenes_serotype_1_2b_str_10-0810_1196163	Listeria_monocytogenes_1639_v117	0.9432
Listeria_monocytogenes_serotype_1_2a_str_99-6370_1196179	Listeria_monocytogenes_1639_v117	0.9415
Listeria_monocytogenes_serotype_1_2a_str_98-2035_1196178	Listeria_monocytogenes_1639_v117	0.9415
Listeria_monocytogenes_serotype_1_2a_str_95-0093_1196177	Listeria_monocytogenes_1639_v117	0.9474
Listeria_monocytogenes_serotype_1_2a_str_88-0478_1196176	Listeria_monocytogenes_1639_v117	0.9463

Listeria_monocytogenes_serotype_1_2a_str_10-5024_1196192	Listeria_monocytogenes_1639_v117	0.9478
Listeria_monocytogenes_serotype_1_2a_str_10-4758_1196168	Listeria_monocytogenes_1639_v117	0.9697
Listeria_monocytogenes_serotype_1_2a_str_10-4754_1196167	Listeria_monocytogenes_1639_v117	0.9697
Listeria_monocytogenes_serotype_1_2a_str_10-1321_1196191	Listeria_monocytogenes_1639_v117	0.9478
Listeria_monocytogenes_serotype_1_2a_str_10-1047_1196190	Listeria_monocytogenes_1639_v117	0.9482
Listeria_monocytogenes_serotype_1_2a_str_10-1046_1196189	Listeria_monocytogenes_1639_v117	0.9482
Listeria_monocytogenes_serotype_1_2a_str_10-0934_1196170	Listeria_monocytogenes_1639_v117	0.9627
Listeria_monocytogenes_serotype_1_2a_str_10-0933_1196169	Listeria_monocytogenes_1639_v117	0.9627
Listeria_monocytogenes_serotype_1_2a_str_10-0815_1196188	Listeria_monocytogenes_1639_v117	0.9429
Listeria_monocytogenes_serotype_1_2a_str_10-0814_1196187	Listeria_monocytogenes_1639_v117	0.9474
Listeria_monocytogenes_serotype_1_2a_str_10-0813_1196166	Listeria_monocytogenes_1639_v117	0.9589
Listeria_monocytogenes_serotype_1_2a_str_10-0812_1196165	Listeria_monocytogenes_1639_v117	0.9589
Listeria_monocytogenes_serotype_1_2a_str_08-7669_1196186	Listeria_monocytogenes_1639_v117	0.9435
Listeria_monocytogenes_serotype_1_2a_str_08-7374_1196185	Listeria_monocytogenes_1639_v117	0.9478
Listeria_monocytogenes_serotype_1_2a_str_08-6997_1196184	Listeria_monocytogenes_1639_v117	0.9429
Listeria_monocytogenes_serotype_1_2a_str_08-6569_1196183	Listeria_monocytogenes_1639_v117	0.9429
Listeria_monocytogenes_serotype_1_2a_str_08-6056_1196182	Listeria_monocytogenes_1639_v117	0.9429
Listeria_monocytogenes_serotype_1_2a_str_04-5457_1196181	Listeria_monocytogenes_1639_v117	0.9478
Listeria_monocytogenes_serotype_1_2a_str_02-5993_1196180	Listeria_monocytogenes_1639_v117	0.9474
Listeria_monocytogenes_R479a_1437838	Listeria_monocytogenes_1639_v117	0.9467
Listeria_monocytogenes_N53-1_1234142	Listeria_monocytogenes_1639_v117	0.9085
Listeria_monocytogenes_M7_1030009	Listeria_monocytogenes_1639_v117	0.9122
Listeria_monocytogenes_LO28_393132	Listeria_monocytogenes_1639_v117	0.955
Listeria_monocytogenes_L99_563174	Listeria_monocytogenes_1639_v117	0.9128
Listeria_monocytogenes_L312_882094	Listeria_monocytogenes_1639_v117	0.941
Listeria_monocytogenes_J1816_930781	Listeria_monocytogenes_1639_v117	0.9228
Listeria_monocytogenes_J1-220_930782	Listeria_monocytogenes_1639_v117	0.9284
Listeria_monocytogenes_J0161_393130	Listeria_monocytogenes_1639_v117	0.9479
Listeria_monocytogenes_HCC23_552536	Listeria_monocytogenes_1639_v117	0.9121
Listeria_monocytogenes_FSL_R2-561_393126	Listeria_monocytogenes_1639_v117	0.9569
Listeria_monocytogenes_FSL_J1-208_393119	Listeria_monocytogenes_1639_v117	0.9013
Listeria_monocytogenes_FSL_J1-175_393118	Listeria_monocytogenes_1639_v117	0.9514
Listeria_monocytogenes_Finland_1998_393127	Listeria_monocytogenes_1639_v117	0.9612
Listeria_monocytogenes_EGD-e_169963_v1	Listeria_monocytogenes_1639_v117	0.9534
Listeria_monocytogenes_EGD-e_169963	Listeria_monocytogenes_1639_v117	0.9534
Listeria_monocytogenes_EGD_1334565	Listeria_monocytogenes_1639_v117	0.9643
Listeria_monocytogenes_CFSAN002349_1299895	Listeria_monocytogenes_1639_v117	0.942
Listeria_monocytogenes_ATCC_19117_882095_v1	Listeria_monocytogenes_1639_v117	0.9371
Listeria_monocytogenes_ATCC_19117_882095	Listeria_monocytogenes_1639_v117	0.9371
Listeria_monocytogenes_6179_1288295	Listeria_monocytogenes_1639_v117	0.9486
Listeria_monocytogenes_1639_v9	Listeria_monocytogenes_1639_v117	0.9263
Listeria_monocytogenes_1639_v99	Listeria_monocytogenes_1639_v117	0.9244
Listeria_monocytogenes_1639_v98	Listeria_monocytogenes_1639_v117	0.9388
Listeria_monocytogenes_1639_v97	Listeria_monocytogenes_1639_v117	0.9309
Listeria_monocytogenes_1639_v96	Listeria_monocytogenes_1639_v117	0.9233
Listeria_monocytogenes_1639_v95	Listeria_monocytogenes_1639_v117	0.9233
Listeria_monocytogenes_1639_v94	Listeria_monocytogenes_1639_v117	0.9233
Listeria_monocytogenes_1639_v93	Listeria_monocytogenes_1639_v117	0.984
Listeria_monocytogenes_1639_v92	Listeria_monocytogenes_1639_v117	0.924
Listeria_monocytogenes_1639_v91	Listeria_monocytogenes_1639_v117	0.9325
Listeria_monocytogenes_1639_v90	Listeria_monocytogenes_1639_v117	0.9388
Listeria_monocytogenes_1639_v8	Listeria_monocytogenes_1639_v117	0.9352
Listeria_monocytogenes_1639_v89	Listeria_monocytogenes_1639_v117	0.9382
Listeria_monocytogenes_1639_v88	Listeria_monocytogenes_1639_v117	0.9549
Listeria_monocytogenes_1639_v87	Listeria_monocytogenes_1639_v117	0.9543
Listeria_monocytogenes_1639_v86	Listeria_monocytogenes_1639_v117	0.9539
Listeria_monocytogenes_1639_v85	Listeria_monocytogenes_1639_v117	0.9265
Listeria_monocytogenes_1639_v84	Listeria_monocytogenes_1639_v117	0.9547
Listeria_monocytogenes_1639_v83	Listeria_monocytogenes_1639_v117	0.9277
Listeria_monocytogenes_1639_v82	Listeria_monocytogenes_1639_v117	0.9274
Listeria_monocytogenes_1639_v81	Listeria_monocytogenes_1639_v117	0.9582
Listeria_monocytogenes_1639_v80	Listeria_monocytogenes_1639_v117	0.9483
Listeria_monocytogenes_1639_v7	Listeria_monocytogenes_1639_v117	0.9352
Listeria_monocytogenes_1639_v79	Listeria_monocytogenes_1639_v117	0.9557
Listeria_monocytogenes_1639_v78	Listeria_monocytogenes_1639_v117	0.959
Listeria_monocytogenes_1639_v77	Listeria_monocytogenes_1639_v117	0.9498
Listeria_monocytogenes_1639_v76	Listeria_monocytogenes_1639_v117	0.953
Listeria_monocytogenes_1639_v75	Listeria_monocytogenes_1639_v117	0.9128
Listeria_monocytogenes_1639_v74	Listeria_monocytogenes_1639_v117	0.9393
Listeria_monocytogenes_1639_v73	Listeria_monocytogenes_1639_v117	0.9515
Listeria_monocytogenes_1639_v72	Listeria_monocytogenes_1639_v117	0.9269
Listeria_monocytogenes_1639_v71	Listeria_monocytogenes_1639_v117	0.9479
Listeria_monocytogenes_1639_v70	Listeria_monocytogenes_1639_v117	0.9544
Listeria_monocytogenes_1639_v6	Listeria_monocytogenes_1639_v117	0.9345
Listeria_monocytogenes_1639_v69	Listeria_monocytogenes_1639_v117	0.9282
Listeria_monocytogenes_1639_v68	Listeria_monocytogenes_1639_v117	0.9503
Listeria_monocytogenes_1639_v67	Listeria_monocytogenes_1639_v117	0.9428
Listeria_monocytogenes_1639_v66	Listeria_monocytogenes_1639_v117	0.9667
Listeria_monocytogenes_1639_v65	Listeria_monocytogenes_1639_v117	0.9495
Listeria_monocytogenes_1639_v64	Listeria_monocytogenes_1639_v117	0.9575
Listeria_monocytogenes_1639_v63	Listeria_monocytogenes_1639_v117	0.9435
Listeria_monocytogenes_1639_v62	Listeria_monocytogenes_1639_v117	0.9543
Listeria_monocytogenes_1639_v61	Listeria_monocytogenes_1639_v117	0.9506
Listeria_monocytogenes_1639_v60	Listeria_monocytogenes_1639_v117	0.9503
Listeria_monocytogenes_1639_v5	Listeria_monocytogenes_1639_v117	0.8982
Listeria_monocytogenes_1639_v59	Listeria_monocytogenes_1639_v117	0.9367
Listeria_monocytogenes_1639_v58	Listeria_monocytogenes_1639_v117	0.9292
Listeria_monocytogenes_1639_v57	Listeria_monocytogenes_1639_v117	0.921
Listeria_monocytogenes_1639_v56	Listeria_monocytogenes_1639_v117	0.9288
Listeria_monocytogenes_1639_v55	Listeria_monocytogenes_1639_v117	0.9165
Listeria_monocytogenes_1639_v54	Listeria_monocytogenes_1639_v117	0.9177

Listeria_monocytogenes_1639_v53	Listeria_monocytogenes_1639_v117	0.9462
Listeria_monocytogenes_1639_v52	Listeria_monocytogenes_1639_v117	0.9339
Listeria_monocytogenes_1639_v51	Listeria_monocytogenes_1639_v117	0.9243
Listeria_monocytogenes_1639_v50	Listeria_monocytogenes_1639_v117	0.9463
Listeria_monocytogenes_1639_v4	Listeria_monocytogenes_1639_v117	0.9109
Listeria_monocytogenes_1639_v49	Listeria_monocytogenes_1639_v117	0.9278
Listeria_monocytogenes_1639_v48	Listeria_monocytogenes_1639_v117	0.9534
Listeria_monocytogenes_1639_v47	Listeria_monocytogenes_1639_v117	0.946
Listeria_monocytogenes_1639_v46	Listeria_monocytogenes_1639_v117	0.9503
Listeria_monocytogenes_1639_v45	Listeria_monocytogenes_1639_v117	0.938
Listeria_monocytogenes_1639_v44	Listeria_monocytogenes_1639_v117	0.9478
Listeria_monocytogenes_1639_v43	Listeria_monocytogenes_1639_v117	0.9478
Listeria_monocytogenes_1639_v42	Listeria_monocytogenes_1639_v117	0.934
Listeria_monocytogenes_1639_v41	Listeria_monocytogenes_1639_v117	0.9278
Listeria_monocytogenes_1639_v40	Listeria_monocytogenes_1639_v117	0.9573
Listeria_monocytogenes_1639_v3	Listeria_monocytogenes_1639_v117	0.9113
Listeria_monocytogenes_1639_v39	Listeria_monocytogenes_1639_v117	0.9423
Listeria_monocytogenes_1639_v38	Listeria_monocytogenes_1639_v117	0.9514
Listeria_monocytogenes_1639_v37	Listeria_monocytogenes_1639_v117	0.9472
Listeria_monocytogenes_1639_v36	Listeria_monocytogenes_1639_v117	0.9455
Listeria_monocytogenes_1639_v35	Listeria_monocytogenes_1639_v117	0.9561
Listeria_monocytogenes_1639_v34	Listeria_monocytogenes_1639_v117	0.956
Listeria_monocytogenes_1639_v33	Listeria_monocytogenes_1639_v117	0.9432
Listeria_monocytogenes_1639_v32	Listeria_monocytogenes_1639_v117	0.9488
Listeria_monocytogenes_1639_v31	Listeria_monocytogenes_1639_v117	0.9563
Listeria_monocytogenes_1639_v30	Listeria_monocytogenes_1639_v117	0.9311
Listeria_monocytogenes_1639_v2	Listeria_monocytogenes_1639_v117	0.8967
Listeria_monocytogenes_1639_v29	Listeria_monocytogenes_1639_v117	0.9438
Listeria_monocytogenes_1639_v28	Listeria_monocytogenes_1639_v117	0.9484
Listeria_monocytogenes_1639_v27	Listeria_monocytogenes_1639_v117	0.9394
Listeria_monocytogenes_1639_v26	Listeria_monocytogenes_1639_v117	0.939
Listeria_monocytogenes_1639_v25	Listeria_monocytogenes_1639_v117	0.9387
Listeria_monocytogenes_1639_v24	Listeria_monocytogenes_1639_v117	0.9269
Listeria_monocytogenes_1639_v23	Listeria_monocytogenes_1639_v117	0.9269
Listeria_monocytogenes_1639_v22	Listeria_monocytogenes_1639_v117	0.945
Listeria_monocytogenes_1639_v21	Listeria_monocytogenes_1639_v117	0.9284
Listeria_monocytogenes_1639_v20	Listeria_monocytogenes_1639_v117	0.9372
Listeria_monocytogenes_1639_v1	Listeria_monocytogenes_1639_v117	0.9432
Listeria_monocytogenes_1639_v19	Listeria_monocytogenes_1639_v117	0.9369
Listeria_monocytogenes_1639_v198	Listeria_monocytogenes_1639_v117	0.9105
Listeria_monocytogenes_1639_v197	Listeria_monocytogenes_1639_v117	0.9405
Listeria_monocytogenes_1639_v196	Listeria_monocytogenes_1639_v117	0.9479
Listeria_monocytogenes_1639_v195	Listeria_monocytogenes_1639_v117	0.9396
Listeria_monocytogenes_1639_v194	Listeria_monocytogenes_1639_v117	0.9374
Listeria_monocytogenes_1639_v193	Listeria_monocytogenes_1639_v117	0.9377
Listeria_monocytogenes_1639_v192	Listeria_monocytogenes_1639_v117	0.8948
Listeria_monocytogenes_1639_v191	Listeria_monocytogenes_1639_v117	0.9646
Listeria_monocytogenes_1639_v190	Listeria_monocytogenes_1639_v117	0.9631
Listeria_monocytogenes_1639_v18	Listeria_monocytogenes_1639_v117	0.9156
Listeria_monocytogenes_1639_v189	Listeria_monocytogenes_1639_v117	0.9623
Listeria_monocytogenes_1639_v188	Listeria_monocytogenes_1639_v117	0.9599
Listeria_monocytogenes_1639_v187	Listeria_monocytogenes_1639_v117	0.9631
Listeria_monocytogenes_1639_v186	Listeria_monocytogenes_1639_v117	0.963
Listeria_monocytogenes_1639_v185	Listeria_monocytogenes_1639_v117	0.9631
Listeria_monocytogenes_1639_v184	Listeria_monocytogenes_1639_v117	0.963
Listeria_monocytogenes_1639_v183	Listeria_monocytogenes_1639_v117	0.96
Listeria_monocytogenes_1639_v182	Listeria_monocytogenes_1639_v117	0.9622
Listeria_monocytogenes_1639_v181	Listeria_monocytogenes_1639_v117	0.9174
Listeria_monocytogenes_1639_v180	Listeria_monocytogenes_1639_v117	0.9174
Listeria_monocytogenes_1639_v17	Listeria_monocytogenes_1639_v117	0.9156
Listeria_monocytogenes_1639_v179	Listeria_monocytogenes_1639_v117	0.9509
Listeria_monocytogenes_1639_v178	Listeria_monocytogenes_1639_v117	0.9642
Listeria_monocytogenes_1639_v177	Listeria_monocytogenes_1639_v117	0.9405
Listeria_monocytogenes_1639_v176	Listeria_monocytogenes_1639_v117	0.9367
Listeria_monocytogenes_1639_v175	Listeria_monocytogenes_1639_v117	0.9684
Listeria_monocytogenes_1639_v174	Listeria_monocytogenes_1639_v117	0.9643
Listeria_monocytogenes_1639_v173	Listeria_monocytogenes_1639_v117	0.9501
Listeria_monocytogenes_1639_v172	Listeria_monocytogenes_1639_v117	0.9358
Listeria_monocytogenes_1639_v171	Listeria_monocytogenes_1639_v117	0.9512
Listeria_monocytogenes_1639_v170	Listeria_monocytogenes_1639_v117	0.9587
Listeria_monocytogenes_1639_v16	Listeria_monocytogenes_1639_v117	0.9339
Listeria_monocytogenes_1639_v169	Listeria_monocytogenes_1639_v117	0.9672
Listeria_monocytogenes_1639_v168	Listeria_monocytogenes_1639_v117	0.9628
Listeria_monocytogenes_1639_v167	Listeria_monocytogenes_1639_v117	0.9526
Listeria_monocytogenes_1639_v166	Listeria_monocytogenes_1639_v117	0.9631
Listeria_monocytogenes_1639_v165	Listeria_monocytogenes_1639_v117	0.9668
Listeria_monocytogenes_1639_v164	Listeria_monocytogenes_1639_v117	0.9407
Listeria_monocytogenes_1639_v163	Listeria_monocytogenes_1639_v117	0.9465
Listeria_monocytogenes_1639_v162	Listeria_monocytogenes_1639_v117	0.9407
Listeria_monocytogenes_1639_v161	Listeria_monocytogenes_1639_v117	0.9414
Listeria_monocytogenes_1639_v160	Listeria_monocytogenes_1639_v117	0.9415
Listeria_monocytogenes_1639_v15	Listeria_monocytogenes_1639_v117	0.9283
Listeria_monocytogenes_1639_v159	Listeria_monocytogenes_1639_v117	0.9405
Listeria_monocytogenes_1639_v158	Listeria_monocytogenes_1639_v117	0.9415
Listeria_monocytogenes_1639_v157	Listeria_monocytogenes_1639_v117	0.9415
Listeria_monocytogenes_1639_v156	Listeria_monocytogenes_1639_v117	0.9418
Listeria_monocytogenes_1639_v155	Listeria_monocytogenes_1639_v117	0.9505
Listeria_monocytogenes_1639_v154	Listeria_monocytogenes_1639_v117	0.9658
Listeria_monocytogenes_1639_v153	Listeria_monocytogenes_1639_v117	0.9505
Listeria_monocytogenes_1639_v152	Listeria_monocytogenes_1639_v117	0.9408
Listeria_monocytogenes_1639_v151	Listeria_monocytogenes_1639_v117	0.9408

Listeria_monocytogenes_1639_v150	Listeria_monocytogenes_1639_v117	0.9646
Listeria_monocytogenes_1639_v14	Listeria_monocytogenes_1639_v117	0.9237
Listeria_monocytogenes_1639_v149	Listeria_monocytogenes_1639_v117	0.9643
Listeria_monocytogenes_1639_v148	Listeria_monocytogenes_1639_v117	0.9397
Listeria_monocytogenes_1639_v147	Listeria_monocytogenes_1639_v117	0.9263
Listeria_monocytogenes_1639_v146	Listeria_monocytogenes_1639_v117	0.9624
Listeria_monocytogenes_1639_v145	Listeria_monocytogenes_1639_v117	0.9583
Listeria_monocytogenes_1639_v144	Listeria_monocytogenes_1639_v117	0.9403
Listeria_monocytogenes_1639_v143	Listeria_monocytogenes_1639_v117	0.9693
Listeria_monocytogenes_1639_v142	Listeria_monocytogenes_1639_v117	0.9407
Listeria_monocytogenes_1639_v141	Listeria_monocytogenes_1639_v117	0.9502
Listeria_monocytogenes_1639_v140	Listeria_monocytogenes_1639_v117	0.941
Listeria_monocytogenes_1639_v13	Listeria_monocytogenes_1639_v117	0.9287
Listeria_monocytogenes_1639_v139	Listeria_monocytogenes_1639_v117	0.9381
Listeria_monocytogenes_1639_v138	Listeria_monocytogenes_1639_v117	0.9434
Listeria_monocytogenes_1639_v137	Listeria_monocytogenes_1639_v117	0.943
Listeria_monocytogenes_1639_v136	Listeria_monocytogenes_1639_v117	0.9682
Listeria_monocytogenes_1639_v135	Listeria_monocytogenes_1639_v117	0.9109
Listeria_monocytogenes_1639_v134	Listeria_monocytogenes_1639_v117	0.936
Listeria_monocytogenes_1639_v133	Listeria_monocytogenes_1639_v117	0.9574
Listeria_monocytogenes_1639_v132	Listeria_monocytogenes_1639_v117	0.9099
Listeria_monocytogenes_1639_v131	Listeria_monocytogenes_1639_v117	0.9312
Listeria_monocytogenes_1639_v130	Listeria_monocytogenes_1639_v117	0.9565
Listeria_monocytogenes_1639_v12	Listeria_monocytogenes_1639_v117	0.938
Listeria_monocytogenes_1639_v129	Listeria_monocytogenes_1639_v117	0.9495
Listeria_monocytogenes_1639_v128	Listeria_monocytogenes_1639_v117	0.9462
Listeria_monocytogenes_1639_v127	Listeria_monocytogenes_1639_v117	0.9576
Listeria_monocytogenes_1639_v126	Listeria_monocytogenes_1639_v117	0.9473
Listeria_monocytogenes_1639_v125	Listeria_monocytogenes_1639_v117	0.9567
Listeria_monocytogenes_1639_v124	Listeria_monocytogenes_1639_v117	0.9233
Listeria_monocytogenes_1639_v123	Listeria_monocytogenes_1639_v117	0.942
Listeria_monocytogenes_1639_v122	Listeria_monocytogenes_1639_v117	0.942
Listeria_monocytogenes_1639_v121	Listeria_monocytogenes_1639_v117	0.942
Listeria_monocytogenes_1639_v120	Listeria_monocytogenes_1639_v117	0.9416
Listeria_monocytogenes_1639_v11	Listeria_monocytogenes_1639_v117	0.9452
Listeria_monocytogenes_1639_v119	Listeria_monocytogenes_1639_v117	0.942
Listeria_monocytogenes_1639_v118	Listeria_monocytogenes_1639_v117	0.942
Listeria_monocytogenes_1639_v117	Listeria_monocytogenes_1639_v117	0.0678
Listeria_welshimeri_1643	Listeria_monocytogenes_1639_v118	0.8835
Listeria_welshimeri_serovar_6b_str_SLCC5334_386043	Listeria_monocytogenes_1639_v118	0.8829
Listeria_seeligeri_serovar_1_2b_str_SLCC3954_683837	Listeria_monocytogenes_1639_v118	0.8908
Listeria_seeligeri_FSL_S4-171_702452	Listeria_monocytogenes_1639_v118	0.8311
Listeria_seeligeri_FSL_N1-067_702453	Listeria_monocytogenes_1639_v118	0.8296
Listeria_seeligeri_1640_v1	Listeria_monocytogenes_1639_v118	0.8988
Listeria_seeligeri_1640	Listeria_monocytogenes_1639_v118	0.8869
Listeria_monocytogenes_WSLC1042_1457188	Listeria_monocytogenes_1639_v118	0.9721
Listeria_monocytogenes_WSLC1001_1457187	Listeria_monocytogenes_1639_v118	0.9368
Listeria_monocytogenes_SLCC7179_879090	Listeria_monocytogenes_1639_v118	0.9424
Listeria_monocytogenes_SLCC5850_882096	Listeria_monocytogenes_1639_v118	0.9411
Listeria_monocytogenes_SLCC2755_932919	Listeria_monocytogenes_1639_v118	0.9526
Listeria_monocytogenes_SLCC2540_879089	Listeria_monocytogenes_1639_v118	0.9516
Listeria_monocytogenes_SLCC2479_882020	Listeria_monocytogenes_1639_v118	0.9339
Listeria_monocytogenes_SLCC2378_879088	Listeria_monocytogenes_1639_v118	0.9732
Listeria_monocytogenes_SLCC2376_882097	Listeria_monocytogenes_1639_v118	0.9462
Listeria_monocytogenes_SLCC2372_932920	Listeria_monocytogenes_1639_v118	0.9265
Listeria_monocytogenes_serotype_4b_str_LL195_1230340	Listeria_monocytogenes_1639_v118	0.9788
Listeria_monocytogenes_serotype_4b_str_LIS0087_1134669	Listeria_monocytogenes_1639_v118	0.9171
Listeria_monocytogenes_serotype_4b_str_F2365_265669	Listeria_monocytogenes_1639_v118	0.9788
Listeria_monocytogenes_serotype_4b_str_CLIP_80459_568819	Listeria_monocytogenes_1639_v118	0.9807
Listeria_monocytogenes_serotype_4b_str_81-0861_1196161	Listeria_monocytogenes_1639_v118	0.9618
Listeria_monocytogenes_serotype_4b_str_81-0592_1196160	Listeria_monocytogenes_1639_v118	0.9629
Listeria_monocytogenes_serotype_4b_str_81-0558_1196159	Listeria_monocytogenes_1639_v118	0.9621
Listeria_monocytogenes_serotype_4b_str_10-0809_1196162	Listeria_monocytogenes_1639_v118	0.9629
Listeria_monocytogenes_serotype_4b_str_02-6680_1196175	Listeria_monocytogenes_1639_v118	0.9769
Listeria_monocytogenes_serotype_4b_str_02-6679_1196174	Listeria_monocytogenes_1639_v118	0.9769
Listeria_monocytogenes_serotype_4b_str_02-1792_1196173	Listeria_monocytogenes_1639_v118	0.9633
Listeria_monocytogenes_serotype_4b_str_02-1289_1196172	Listeria_monocytogenes_1639_v118	0.9633
Listeria_monocytogenes_serotype_4b_str_02-1103_1196171	Listeria_monocytogenes_1639_v118	0.9633
Listeria_monocytogenes_serotype_3c_str_10-5027_1196195	Listeria_monocytogenes_1639_v118	0.9331
Listeria_monocytogenes_serotype_1_2c_str_10-5026_1196194	Listeria_monocytogenes_1639_v118	0.9342
Listeria_monocytogenes_serotype_1_2c_str_10-5025_1196193	Listeria_monocytogenes_1639_v118	0.9342
Listeria_monocytogenes_serotype_1_2b_str_10-0811_1196164	Listeria_monocytogenes_1639_v118	0.9462
Listeria_monocytogenes_serotype_1_2b_str_10-0810_1196163	Listeria_monocytogenes_1639_v118	0.9462
Listeria_monocytogenes_serotype_1_2a_str_99-6370_1196179	Listeria_monocytogenes_1639_v118	0.9206
Listeria_monocytogenes_serotype_1_2a_str_98-2035_1196178	Listeria_monocytogenes_1639_v118	0.9206
Listeria_monocytogenes_serotype_1_2a_str_95-0093_1196177	Listeria_monocytogenes_1639_v118	0.9261
Listeria_monocytogenes_serotype_1_2a_str_88-0478_1196176	Listeria_monocytogenes_1639_v118	0.9252
Listeria_monocytogenes_serotype_1_2a_str_10-5024_1196192	Listeria_monocytogenes_1639_v118	0.9264
Listeria_monocytogenes_serotype_1_2a_str_10-4758_1196168	Listeria_monocytogenes_1639_v118	0.9423
Listeria_monocytogenes_serotype_1_2a_str_10-4754_1196167	Listeria_monocytogenes_1639_v118	0.942
Listeria_monocytogenes_serotype_1_2a_str_10-1321_1196191	Listeria_monocytogenes_1639_v118	0.9264
Listeria_monocytogenes_serotype_1_2a_str_10-1047_1196190	Listeria_monocytogenes_1639_v118	0.9267
Listeria_monocytogenes_serotype_1_2a_str_10-1046_1196189	Listeria_monocytogenes_1639_v118	0.9267
Listeria_monocytogenes_serotype_1_2a_str_10-0934_1196170	Listeria_monocytogenes_1639_v118	0.9532
Listeria_monocytogenes_serotype_1_2a_str_10-0933_1196169	Listeria_monocytogenes_1639_v118	0.9532
Listeria_monocytogenes_serotype_1_2a_str_10-0815_1196188	Listeria_monocytogenes_1639_v118	0.9219
Listeria_monocytogenes_serotype_1_2a_str_10-0814_1196187	Listeria_monocytogenes_1639_v118	0.9264
Listeria_monocytogenes_serotype_1_2a_str_10-0813_1196166	Listeria_monocytogenes_1639_v118	0.934
Listeria_monocytogenes_serotype_1_2a_str_10-0812_1196165	Listeria_monocytogenes_1639_v118	0.934
Listeria_monocytogenes_serotype_1_2a_str_08-7669_1196186	Listeria_monocytogenes_1639_v118	0.9355
Listeria_monocytogenes_serotype_1_2a_str_08-7374_1196185	Listeria_monocytogenes_1639_v118	0.9264

Listeria_monocytogenes_serotype_1_2a_str_08-6997_1196184	Listeria_monocytogenes_1639_v118	0.9219
Listeria_monocytogenes_serotype_1_2a_str_08-6569_1196183	Listeria_monocytogenes_1639_v118	0.9219
Listeria_monocytogenes_serotype_1_2a_str_08-6056_1196182	Listeria_monocytogenes_1639_v118	0.9219
Listeria_monocytogenes_serotype_1_2a_str_04-5457_1196181	Listeria_monocytogenes_1639_v118	0.9264
Listeria_monocytogenes_serotype_1_2a_str_02-5993_1196180	Listeria_monocytogenes_1639_v118	0.9261
Listeria_monocytogenes_R479a_1437838	Listeria_monocytogenes_1639_v118	0.9257
Listeria_monocytogenes_N53-1_1234142	Listeria_monocytogenes_1639_v118	0.8972
Listeria_monocytogenes_M7_1030009	Listeria_monocytogenes_1639_v118	0.9093
Listeria_monocytogenes_LO28_393132	Listeria_monocytogenes_1639_v118	0.9325
Listeria_monocytogenes_L99_563174	Listeria_monocytogenes_1639_v118	0.91
Listeria_monocytogenes_L312_882094	Listeria_monocytogenes_1639_v118	0.9811
Listeria_monocytogenes_J1816_930781	Listeria_monocytogenes_1639_v118	0.9619
Listeria_monocytogenes_J1-220_930782	Listeria_monocytogenes_1639_v118	0.9523
Listeria_monocytogenes_J0161_393130	Listeria_monocytogenes_1639_v118	0.9317
Listeria_monocytogenes_HCC23_552536	Listeria_monocytogenes_1639_v118	0.9093
Listeria_monocytogenes_FSL_R2-561_393126	Listeria_monocytogenes_1639_v118	0.9324
Listeria_monocytogenes_FSL_J1-208_393119	Listeria_monocytogenes_1639_v118	0.9212
Listeria_monocytogenes_FSL_J1-175_393118	Listeria_monocytogenes_1639_v118	0.9614
Listeria_monocytogenes_Finland_1998_393127	Listeria_monocytogenes_1639_v118	0.9536
Listeria_monocytogenes_EGD-e_169963_v1	Listeria_monocytogenes_1639_v118	0.9421
Listeria_monocytogenes_EGD-e_169963	Listeria_monocytogenes_1639_v118	0.9424
Listeria_monocytogenes_EGD_1334565	Listeria_monocytogenes_1639_v118	0.9419
Listeria_monocytogenes_CFSAN002349_1299895	Listeria_monocytogenes_1639_v118	0.9996
Listeria_monocytogenes_ATCC_19117_882095_v1	Listeria_monocytogenes_1639_v118	0.9728
Listeria_monocytogenes_ATCC_19117_882095	Listeria_monocytogenes_1639_v118	0.9727
Listeria_monocytogenes_6179_1288295	Listeria_monocytogenes_1639_v118	0.9155
Listeria_monocytogenes_1639_v9	Listeria_monocytogenes_1639_v118	0.9317
Listeria_monocytogenes_1639_v99	Listeria_monocytogenes_1639_v118	0.9634
Listeria_monocytogenes_1639_v98	Listeria_monocytogenes_1639_v118	0.9289
Listeria_monocytogenes_1639_v97	Listeria_monocytogenes_1639_v118	0.9688
Listeria_monocytogenes_1639_v96	Listeria_monocytogenes_1639_v118	0.9627
Listeria_monocytogenes_1639_v95	Listeria_monocytogenes_1639_v118	0.9627
Listeria_monocytogenes_1639_v94	Listeria_monocytogenes_1639_v118	0.9627
Listeria_monocytogenes_1639_v93	Listeria_monocytogenes_1639_v118	0.9294
Listeria_monocytogenes_1639_v92	Listeria_monocytogenes_1639_v118	0.9631
Listeria_monocytogenes_1639_v91	Listeria_monocytogenes_1639_v118	0.9585
Listeria_monocytogenes_1639_v90	Listeria_monocytogenes_1639_v118	0.9289
Listeria_monocytogenes_1639_v8	Listeria_monocytogenes_1639_v118	0.9154
Listeria_monocytogenes_1639_v89	Listeria_monocytogenes_1639_v118	0.9706
Listeria_monocytogenes_1639_v88	Listeria_monocytogenes_1639_v118	0.9341
Listeria_monocytogenes_1639_v87	Listeria_monocytogenes_1639_v118	0.9298
Listeria_monocytogenes_1639_v86	Listeria_monocytogenes_1639_v118	0.9291
Listeria_monocytogenes_1639_v85	Listeria_monocytogenes_1639_v118	0.9611
Listeria_monocytogenes_1639_v84	Listeria_monocytogenes_1639_v118	0.9369
Listeria_monocytogenes_1639_v83	Listeria_monocytogenes_1639_v118	0.9613
Listeria_monocytogenes_1639_v82	Listeria_monocytogenes_1639_v118	0.9606
Listeria_monocytogenes_1639_v81	Listeria_monocytogenes_1639_v118	0.934
Listeria_monocytogenes_1639_v80	Listeria_monocytogenes_1639_v118	0.9533
Listeria_monocytogenes_1639_v7	Listeria_monocytogenes_1639_v118	0.9154
Listeria_monocytogenes_1639_v79	Listeria_monocytogenes_1639_v118	0.9319
Listeria_monocytogenes_1639_v78	Listeria_monocytogenes_1639_v118	0.9333
Listeria_monocytogenes_1639_v77	Listeria_monocytogenes_1639_v118	0.9331
Listeria_monocytogenes_1639_v76	Listeria_monocytogenes_1639_v118	0.9259
Listeria_monocytogenes_1639_v75	Listeria_monocytogenes_1639_v118	0.91
Listeria_monocytogenes_1639_v74	Listeria_monocytogenes_1639_v118	0.9287
Listeria_monocytogenes_1639_v73	Listeria_monocytogenes_1639_v118	0.9276
Listeria_monocytogenes_1639_v72	Listeria_monocytogenes_1639_v118	0.9601
Listeria_monocytogenes_1639_v71	Listeria_monocytogenes_1639_v118	0.9172
Listeria_monocytogenes_1639_v70	Listeria_monocytogenes_1639_v118	0.9303
Listeria_monocytogenes_1639_v6	Listeria_monocytogenes_1639_v118	0.9148
Listeria_monocytogenes_1639_v69	Listeria_monocytogenes_1639_v118	0.9633
Listeria_monocytogenes_1639_v68	Listeria_monocytogenes_1639_v118	0.9359
Listeria_monocytogenes_1639_v67	Listeria_monocytogenes_1639_v118	0.9515
Listeria_monocytogenes_1639_v66	Listeria_monocytogenes_1639_v118	0.9375
Listeria_monocytogenes_1639_v65	Listeria_monocytogenes_1639_v118	0.9296
Listeria_monocytogenes_1639_v64	Listeria_monocytogenes_1639_v118	0.93
Listeria_monocytogenes_1639_v63	Listeria_monocytogenes_1639_v118	0.9472
Listeria_monocytogenes_1639_v62	Listeria_monocytogenes_1639_v118	0.9298
Listeria_monocytogenes_1639_v61	Listeria_monocytogenes_1639_v118	0.9292
Listeria_monocytogenes_1639_v60	Listeria_monocytogenes_1639_v118	0.9336
Listeria_monocytogenes_1639_v5	Listeria_monocytogenes_1639_v118	0.913
Listeria_monocytogenes_1639_v59	Listeria_monocytogenes_1639_v118	0.962
Listeria_monocytogenes_1639_v58	Listeria_monocytogenes_1639_v118	0.9655
Listeria_monocytogenes_1639_v57	Listeria_monocytogenes_1639_v118	0.9595
Listeria_monocytogenes_1639_v56	Listeria_monocytogenes_1639_v118	0.9617
Listeria_monocytogenes_1639_v55	Listeria_monocytogenes_1639_v118	0.9551
Listeria_monocytogenes_1639_v54	Listeria_monocytogenes_1639_v118	0.9286
Listeria_monocytogenes_1639_v53	Listeria_monocytogenes_1639_v118	0.9333
Listeria_monocytogenes_1639_v52	Listeria_monocytogenes_1639_v118	0.958
Listeria_monocytogenes_1639_v51	Listeria_monocytogenes_1639_v118	0.9559
Listeria_monocytogenes_1639_v50	Listeria_monocytogenes_1639_v118	0.9256
Listeria_monocytogenes_1639_v4	Listeria_monocytogenes_1639_v118	0.9257
Listeria_monocytogenes_1639_v49	Listeria_monocytogenes_1639_v118	0.961
Listeria_monocytogenes_1639_v48	Listeria_monocytogenes_1639_v118	0.9282
Listeria_monocytogenes_1639_v47	Listeria_monocytogenes_1639_v118	0.9261
Listeria_monocytogenes_1639_v46	Listeria_monocytogenes_1639_v118	0.9352
Listeria_monocytogenes_1639_v45	Listeria_monocytogenes_1639_v118	0.9168
Listeria_monocytogenes_1639_v44	Listeria_monocytogenes_1639_v118	0.9264
Listeria_monocytogenes_1639_v43	Listeria_monocytogenes_1639_v118	0.9264
Listeria_monocytogenes_1639_v42	Listeria_monocytogenes_1639_v118	0.9175
Listeria_monocytogenes_1639_v41	Listeria_monocytogenes_1639_v118	0.9074

Listeria_monocytogenes_1639_v40	Listeria_monocytogenes_1639_v118	0.9328
Listeria_monocytogenes_1639_v3	Listeria_monocytogenes_1639_v118	0.9261
Listeria_monocytogenes_1639_v39	Listeria_monocytogenes_1639_v118	0.9483
Listeria_monocytogenes_1639_v38	Listeria_monocytogenes_1639_v118	0.93
Listeria_monocytogenes_1639_v37	Listeria_monocytogenes_1639_v118	0.9259
Listeria_monocytogenes_1639_v36	Listeria_monocytogenes_1639_v118	0.9223
Listeria_monocytogenes_1639_v35	Listeria_monocytogenes_1639_v118	0.9335
Listeria_monocytogenes_1639_v34	Listeria_monocytogenes_1639_v118	0.9278
Listeria_monocytogenes_1639_v33	Listeria_monocytogenes_1639_v118	0.9462
Listeria_monocytogenes_1639_v32	Listeria_monocytogenes_1639_v118	0.9248
Listeria_monocytogenes_1639_v31	Listeria_monocytogenes_1639_v118	0.9282
Listeria_monocytogenes_1639_v30	Listeria_monocytogenes_1639_v118	0.9498
Listeria_monocytogenes_1639_v2	Listeria_monocytogenes_1639_v118	0.9101
Listeria_monocytogenes_1639_v29	Listeria_monocytogenes_1639_v118	0.9193
Listeria_monocytogenes_1639_v28	Listeria_monocytogenes_1639_v118	0.9237
Listeria_monocytogenes_1639_v27	Listeria_monocytogenes_1639_v118	0.9145
Listeria_monocytogenes_1639_v26	Listeria_monocytogenes_1639_v118	0.9145
Listeria_monocytogenes_1639_v25	Listeria_monocytogenes_1639_v118	0.9142
Listeria_monocytogenes_1639_v24	Listeria_monocytogenes_1639_v118	0.9523
Listeria_monocytogenes_1639_v23	Listeria_monocytogenes_1639_v118	0.9523
Listeria_monocytogenes_1639_v22	Listeria_monocytogenes_1639_v118	0.9491
Listeria_monocytogenes_1639_v21	Listeria_monocytogenes_1639_v118	0.9519
Listeria_monocytogenes_1639_v20	Listeria_monocytogenes_1639_v118	0.9398
Listeria_monocytogenes_1639_v1	Listeria_monocytogenes_1639_v118	0.9096
Listeria_monocytogenes_1639_v19	Listeria_monocytogenes_1639_v118	0.9394
Listeria_monocytogenes_1639_v198	Listeria_monocytogenes_1639_v118	0.8999
Listeria_monocytogenes_1639_v197	Listeria_monocytogenes_1639_v118	0.9299
Listeria_monocytogenes_1639_v196	Listeria_monocytogenes_1639_v118	0.9245
Listeria_monocytogenes_1639_v195	Listeria_monocytogenes_1639_v118	0.9473
Listeria_monocytogenes_1639_v194	Listeria_monocytogenes_1639_v118	0.947
Listeria_monocytogenes_1639_v193	Listeria_monocytogenes_1639_v118	0.947
Listeria_monocytogenes_1639_v192	Listeria_monocytogenes_1639_v118	0.9126
Listeria_monocytogenes_1639_v191	Listeria_monocytogenes_1639_v118	0.9534
Listeria_monocytogenes_1639_v190	Listeria_monocytogenes_1639_v118	0.9535
Listeria_monocytogenes_1639_v18	Listeria_monocytogenes_1639_v118	0.9534
Listeria_monocytogenes_1639_v189	Listeria_monocytogenes_1639_v118	0.9527
Listeria_monocytogenes_1639_v188	Listeria_monocytogenes_1639_v118	0.9534
Listeria_monocytogenes_1639_v187	Listeria_monocytogenes_1639_v118	0.9531
Listeria_monocytogenes_1639_v186	Listeria_monocytogenes_1639_v118	0.9539
Listeria_monocytogenes_1639_v185	Listeria_monocytogenes_1639_v118	0.9535
Listeria_monocytogenes_1639_v184	Listeria_monocytogenes_1639_v118	0.9531
Listeria_monocytogenes_1639_v183	Listeria_monocytogenes_1639_v118	0.9481
Listeria_monocytogenes_1639_v182	Listeria_monocytogenes_1639_v118	0.9503
Listeria_monocytogenes_1639_v181	Listeria_monocytogenes_1639_v118	0.9337
Listeria_monocytogenes_1639_v180	Listeria_monocytogenes_1639_v118	0.9337
Listeria_monocytogenes_1639_v17	Listeria_monocytogenes_1639_v118	0.9534
Listeria_monocytogenes_1639_v179	Listeria_monocytogenes_1639_v118	0.9382
Listeria_monocytogenes_1639_v178	Listeria_monocytogenes_1639_v118	0.955
Listeria_monocytogenes_1639_v177	Listeria_monocytogenes_1639_v118	0.9823
Listeria_monocytogenes_1639_v176	Listeria_monocytogenes_1639_v118	0.9444
Listeria_monocytogenes_1639_v175	Listeria_monocytogenes_1639_v118	0.9424
Listeria_monocytogenes_1639_v174	Listeria_monocytogenes_1639_v118	0.9539
Listeria_monocytogenes_1639_v173	Listeria_monocytogenes_1639_v118	0.9714
Listeria_monocytogenes_1639_v172	Listeria_monocytogenes_1639_v118	0.9426
Listeria_monocytogenes_1639_v171	Listeria_monocytogenes_1639_v118	0.9386
Listeria_monocytogenes_1639_v170	Listeria_monocytogenes_1639_v118	0.9469
Listeria_monocytogenes_1639_v16	Listeria_monocytogenes_1639_v118	0.9127
Listeria_monocytogenes_1639_v169	Listeria_monocytogenes_1639_v118	0.9466
Listeria_monocytogenes_1639_v168	Listeria_monocytogenes_1639_v118	0.9474
Listeria_monocytogenes_1639_v167	Listeria_monocytogenes_1639_v118	0.94
Listeria_monocytogenes_1639_v166	Listeria_monocytogenes_1639_v118	0.9478
Listeria_monocytogenes_1639_v165	Listeria_monocytogenes_1639_v118	0.9466
Listeria_monocytogenes_1639_v164	Listeria_monocytogenes_1639_v118	0.978
Listeria_monocytogenes_1639_v163	Listeria_monocytogenes_1639_v118	0.968
Listeria_monocytogenes_1639_v162	Listeria_monocytogenes_1639_v118	0.978
Listeria_monocytogenes_1639_v161	Listeria_monocytogenes_1639_v118	0.978
Listeria_monocytogenes_1639_v160	Listeria_monocytogenes_1639_v118	0.978
Listeria_monocytogenes_1639_v15	Listeria_monocytogenes_1639_v118	0.9323
Listeria_monocytogenes_1639_v159	Listeria_monocytogenes_1639_v118	0.9827
Listeria_monocytogenes_1639_v158	Listeria_monocytogenes_1639_v118	0.9792
Listeria_monocytogenes_1639_v157	Listeria_monocytogenes_1639_v118	0.9788
Listeria_monocytogenes_1639_v156	Listeria_monocytogenes_1639_v118	0.9308
Listeria_monocytogenes_1639_v155	Listeria_monocytogenes_1639_v118	0.9413
Listeria_monocytogenes_1639_v154	Listeria_monocytogenes_1639_v118	0.9452
Listeria_monocytogenes_1639_v153	Listeria_monocytogenes_1639_v118	0.9421
Listeria_monocytogenes_1639_v152	Listeria_monocytogenes_1639_v118	0.9831
Listeria_monocytogenes_1639_v151	Listeria_monocytogenes_1639_v118	0.9831
Listeria_monocytogenes_1639_v150	Listeria_monocytogenes_1639_v118	0.9433
Listeria_monocytogenes_1639_v14	Listeria_monocytogenes_1639_v118	0.9396
Listeria_monocytogenes_1639_v149	Listeria_monocytogenes_1639_v118	0.9419
Listeria_monocytogenes_1639_v148	Listeria_monocytogenes_1639_v118	0.9816
Listeria_monocytogenes_1639_v147	Listeria_monocytogenes_1639_v118	0.9678
Listeria_monocytogenes_1639_v146	Listeria_monocytogenes_1639_v118	0.9517
Listeria_monocytogenes_1639_v145	Listeria_monocytogenes_1639_v118	0.9464
Listeria_monocytogenes_1639_v144	Listeria_monocytogenes_1639_v118	0.9803
Listeria_monocytogenes_1639_v143	Listeria_monocytogenes_1639_v118	0.9518
Listeria_monocytogenes_1639_v142	Listeria_monocytogenes_1639_v118	0.9807
Listeria_monocytogenes_1639_v141	Listeria_monocytogenes_1639_v118	0.9726
Listeria_monocytogenes_1639_v140	Listeria_monocytogenes_1639_v118	0.9811
Listeria_monocytogenes_1639_v13	Listeria_monocytogenes_1639_v118	0.9322
Listeria_monocytogenes_1639_v139	Listeria_monocytogenes_1639_v118	0.98

Listeria_monocytogenes_1639_v138	Listeria_monocytogenes_1639_v118	0.983
Listeria_monocytogenes_1639_v137	Listeria_monocytogenes_1639_v118	0.9827
Listeria_monocytogenes_1639_v136	Listeria_monocytogenes_1639_v118	0.9416
Listeria_monocytogenes_1639_v135	Listeria_monocytogenes_1639_v118	0.9213
Listeria_monocytogenes_1639_v134	Listeria_monocytogenes_1639_v118	0.9731
Listeria_monocytogenes_1639_v133	Listeria_monocytogenes_1639_v118	0.9387
Listeria_monocytogenes_1639_v132	Listeria_monocytogenes_1639_v118	0.9221
Listeria_monocytogenes_1639_v131	Listeria_monocytogenes_1639_v118	0.9707
Listeria_monocytogenes_1639_v130	Listeria_monocytogenes_1639_v118	0.9339
Listeria_monocytogenes_1639_v12	Listeria_monocytogenes_1639_v118	0.9132
Listeria_monocytogenes_1639_v129	Listeria_monocytogenes_1639_v118	0.9703
Listeria_monocytogenes_1639_v128	Listeria_monocytogenes_1639_v118	0.9681
Listeria_monocytogenes_1639_v127	Listeria_monocytogenes_1639_v118	0.9417
Listeria_monocytogenes_1639_v126	Listeria_monocytogenes_1639_v118	0.9666
Listeria_monocytogenes_1639_v125	Listeria_monocytogenes_1639_v118	0.9387
Listeria_monocytogenes_1639_v124	Listeria_monocytogenes_1639_v118	0.9403
Listeria_monocytogenes_1639_v123	Listeria_monocytogenes_1639_v118	0.9996
Listeria_monocytogenes_1639_v122	Listeria_monocytogenes_1639_v118	1
Listeria_monocytogenes_1639_v121	Listeria_monocytogenes_1639_v118	1
Listeria_monocytogenes_1639_v120	Listeria_monocytogenes_1639_v118	0.9996
Listeria_monocytogenes_1639_v11	Listeria_monocytogenes_1639_v118	0.9126
Listeria_monocytogenes_1639_v119	Listeria_monocytogenes_1639_v118	1
Listeria_monocytogenes_1639_v118	Listeria_monocytogenes_1639_v118	0.0677
Listeria_welshimeri_1643	Listeria_monocytogenes_1639_v119	0.8835
Listeria_welshimeri_serovar_6b_str_SLCC5334_386043	Listeria_monocytogenes_1639_v119	0.8829
Listeria_seeligeri_serovar_1_2b_str_SLCC3954_683837	Listeria_monocytogenes_1639_v119	0.8908
Listeria_seeligeri_FSL_S4-171_702452	Listeria_monocytogenes_1639_v119	0.8311
Listeria_seeligeri_FSL_N1-067_702453	Listeria_monocytogenes_1639_v119	0.8296
Listeria_seeligeri_1640_v1	Listeria_monocytogenes_1639_v119	0.8988
Listeria_seeligeri_1640	Listeria_monocytogenes_1639_v119	0.8869
Listeria_monocytogenes_WSLC1042_1457188	Listeria_monocytogenes_1639_v119	0.9721
Listeria_monocytogenes_WSLC1001_1457187	Listeria_monocytogenes_1639_v119	0.9368
Listeria_monocytogenes_SLCC7179_879090	Listeria_monocytogenes_1639_v119	0.9424
Listeria_monocytogenes_SLCC5850_882096	Listeria_monocytogenes_1639_v119	0.9411
Listeria_monocytogenes_SLCC2755_932919	Listeria_monocytogenes_1639_v119	0.9526
Listeria_monocytogenes_SLCC2540_879089	Listeria_monocytogenes_1639_v119	0.9516
Listeria_monocytogenes_SLCC2479_882020	Listeria_monocytogenes_1639_v119	0.9339
Listeria_monocytogenes_SLCC2378_879088	Listeria_monocytogenes_1639_v119	0.9732
Listeria_monocytogenes_SLCC2376_882097	Listeria_monocytogenes_1639_v119	0.9462
Listeria_monocytogenes_SLCC2372_932920	Listeria_monocytogenes_1639_v119	0.9265
Listeria_monocytogenes_serotype_4b_str_LL195_1230340	Listeria_monocytogenes_1639_v119	0.9788
Listeria_monocytogenes_serotype_4b_str_LIS0087_1134669	Listeria_monocytogenes_1639_v119	0.9171
Listeria_monocytogenes_serotype_4b_str_F2365_265669	Listeria_monocytogenes_1639_v119	0.9788
Listeria_monocytogenes_serotype_4b_str_CLIP_80459_568819	Listeria_monocytogenes_1639_v119	0.9807
Listeria_monocytogenes_serotype_4b_str_81-0861_1196161	Listeria_monocytogenes_1639_v119	0.9618
Listeria_monocytogenes_serotype_4b_str_81-0592_1196160	Listeria_monocytogenes_1639_v119	0.9629
Listeria_monocytogenes_serotype_4b_str_81-0558_1196159	Listeria_monocytogenes_1639_v119	0.9621
Listeria_monocytogenes_serotype_4b_str_10-0809_1196162	Listeria_monocytogenes_1639_v119	0.9629
Listeria_monocytogenes_serotype_4b_str_02-6680_1196175	Listeria_monocytogenes_1639_v119	0.9769
Listeria_monocytogenes_serotype_4b_str_02-6679_1196174	Listeria_monocytogenes_1639_v119	0.9769
Listeria_monocytogenes_serotype_4b_str_02-1792_1196173	Listeria_monocytogenes_1639_v119	0.9633
Listeria_monocytogenes_serotype_4b_str_02-1289_1196172	Listeria_monocytogenes_1639_v119	0.9633
Listeria_monocytogenes_serotype_4b_str_02-1103_1196171	Listeria_monocytogenes_1639_v119	0.9633
Listeria_monocytogenes_serotype_3c_str_10-5027_1196195	Listeria_monocytogenes_1639_v119	0.9331
Listeria_monocytogenes_serotype_1_2c_str_10-5026_1196194	Listeria_monocytogenes_1639_v119	0.9342
Listeria_monocytogenes_serotype_1_2c_str_10-5025_1196193	Listeria_monocytogenes_1639_v119	0.9342
Listeria_monocytogenes_serotype_1_2b_str_10-0811_1196164	Listeria_monocytogenes_1639_v119	0.9462
Listeria_monocytogenes_serotype_1_2b_str_10-0810_1196163	Listeria_monocytogenes_1639_v119	0.9462
Listeria_monocytogenes_serotype_1_2a_str_99-6370_1196179	Listeria_monocytogenes_1639_v119	0.9206
Listeria_monocytogenes_serotype_1_2a_str_98-2035_1196178	Listeria_monocytogenes_1639_v119	0.9206
Listeria_monocytogenes_serotype_1_2a_str_95-0093_1196177	Listeria_monocytogenes_1639_v119	0.9261
Listeria_monocytogenes_serotype_1_2a_str_88-0478_1196176	Listeria_monocytogenes_1639_v119	0.9252
Listeria_monocytogenes_serotype_1_2a_str_10-5024_1196192	Listeria_monocytogenes_1639_v119	0.9264
Listeria_monocytogenes_serotype_1_2a_str_10-4758_1196168	Listeria_monocytogenes_1639_v119	0.9423
Listeria_monocytogenes_serotype_1_2a_str_10-4754_1196167	Listeria_monocytogenes_1639_v119	0.942
Listeria_monocytogenes_serotype_1_2a_str_10-1321_1196191	Listeria_monocytogenes_1639_v119	0.9264
Listeria_monocytogenes_serotype_1_2a_str_10-1047_1196190	Listeria_monocytogenes_1639_v119	0.9267
Listeria_monocytogenes_serotype_1_2a_str_10-1046_1196189	Listeria_monocytogenes_1639_v119	0.9267
Listeria_monocytogenes_serotype_1_2a_str_10-0934_1196170	Listeria_monocytogenes_1639_v119	0.9532
Listeria_monocytogenes_serotype_1_2a_str_10-0933_1196169	Listeria_monocytogenes_1639_v119	0.9532
Listeria_monocytogenes_serotype_1_2a_str_10-0815_1196188	Listeria_monocytogenes_1639_v119	0.9219
Listeria_monocytogenes_serotype_1_2a_str_10-0814_1196187	Listeria_monocytogenes_1639_v119	0.9264
Listeria_monocytogenes_serotype_1_2a_str_10-0813_1196166	Listeria_monocytogenes_1639_v119	0.934
Listeria_monocytogenes_serotype_1_2a_str_10-0812_1196165	Listeria_monocytogenes_1639_v119	0.934
Listeria_monocytogenes_serotype_1_2a_str_08-7669_1196186	Listeria_monocytogenes_1639_v119	0.9355
Listeria_monocytogenes_serotype_1_2a_str_08-7374_1196185	Listeria_monocytogenes_1639_v119	0.9264
Listeria_monocytogenes_serotype_1_2a_str_08-6997_1196184	Listeria_monocytogenes_1639_v119	0.9219
Listeria_monocytogenes_serotype_1_2a_str_08-6569_1196183	Listeria_monocytogenes_1639_v119	0.9219
Listeria_monocytogenes_serotype_1_2a_str_08-6056_1196182	Listeria_monocytogenes_1639_v119	0.9219
Listeria_monocytogenes_serotype_1_2a_str_04-5457_1196181	Listeria_monocytogenes_1639_v119	0.9264
Listeria_monocytogenes_serotype_1_2a_str_02-5993_1196180	Listeria_monocytogenes_1639_v119	0.9261
Listeria_monocytogenes_R479a_1437838	Listeria_monocytogenes_1639_v119	0.9257
Listeria_monocytogenes_N53-1_1234142	Listeria_monocytogenes_1639_v119	0.8972
Listeria_monocytogenes_M7_1030009	Listeria_monocytogenes_1639_v119	0.9093
Listeria_monocytogenes_LO28_393132	Listeria_monocytogenes_1639_v119	0.9325
Listeria_monocytogenes_L99_563174	Listeria_monocytogenes_1639_v119	0.91
Listeria_monocytogenes_L312_882094	Listeria_monocytogenes_1639_v119	0.9811
Listeria_monocytogenes_J1816_930781	Listeria_monocytogenes_1639_v119	0.9619
Listeria_monocytogenes_J1-220_930782	Listeria_monocytogenes_1639_v119	0.9523
Listeria_monocytogenes_J0161_393130	Listeria_monocytogenes_1639_v119	0.9317
Listeria_monocytogenes_HCC23_552536	Listeria_monocytogenes_1639_v119	0.9093

Listeria_monocytogenes_FSL_R2-561_393126	Listeria_monocytogenes_1639_v119	0.9324
Listeria_monocytogenes_FSL_J1-208_393119	Listeria_monocytogenes_1639_v119	0.9212
Listeria_monocytogenes_FSL_J1-175_393118	Listeria_monocytogenes_1639_v119	0.9614
Listeria_monocytogenes_Finland_1998_393127	Listeria_monocytogenes_1639_v119	0.9536
Listeria_monocytogenes_EGD-e_169963_v1	Listeria_monocytogenes_1639_v119	0.9421
Listeria_monocytogenes_EGD-e_169963	Listeria_monocytogenes_1639_v119	0.9424
Listeria_monocytogenes_EGD_1334565	Listeria_monocytogenes_1639_v119	0.9419
Listeria_monocytogenes_CFSAN002349_1299895	Listeria_monocytogenes_1639_v119	0.9996
Listeria_monocytogenes_ATCC_19117_882095_v1	Listeria_monocytogenes_1639_v119	0.9728
Listeria_monocytogenes_ATCC_19117_882095	Listeria_monocytogenes_1639_v119	0.9727
Listeria_monocytogenes_6179_1288295	Listeria_monocytogenes_1639_v119	0.9155
Listeria_monocytogenes_1639_v9	Listeria_monocytogenes_1639_v119	0.9317
Listeria_monocytogenes_1639_v99	Listeria_monocytogenes_1639_v119	0.9634
Listeria_monocytogenes_1639_v98	Listeria_monocytogenes_1639_v119	0.9289
Listeria_monocytogenes_1639_v97	Listeria_monocytogenes_1639_v119	0.9688
Listeria_monocytogenes_1639_v96	Listeria_monocytogenes_1639_v119	0.9627
Listeria_monocytogenes_1639_v95	Listeria_monocytogenes_1639_v119	0.9627
Listeria_monocytogenes_1639_v94	Listeria_monocytogenes_1639_v119	0.9627
Listeria_monocytogenes_1639_v93	Listeria_monocytogenes_1639_v119	0.9294
Listeria_monocytogenes_1639_v92	Listeria_monocytogenes_1639_v119	0.9631
Listeria_monocytogenes_1639_v91	Listeria_monocytogenes_1639_v119	0.9585
Listeria_monocytogenes_1639_v90	Listeria_monocytogenes_1639_v119	0.9289
Listeria_monocytogenes_1639_v8	Listeria_monocytogenes_1639_v119	0.9154
Listeria_monocytogenes_1639_v89	Listeria_monocytogenes_1639_v119	0.9706
Listeria_monocytogenes_1639_v88	Listeria_monocytogenes_1639_v119	0.9341
Listeria_monocytogenes_1639_v87	Listeria_monocytogenes_1639_v119	0.9298
Listeria_monocytogenes_1639_v86	Listeria_monocytogenes_1639_v119	0.9291
Listeria_monocytogenes_1639_v85	Listeria_monocytogenes_1639_v119	0.9611
Listeria_monocytogenes_1639_v84	Listeria_monocytogenes_1639_v119	0.9369
Listeria_monocytogenes_1639_v83	Listeria_monocytogenes_1639_v119	0.9613
Listeria_monocytogenes_1639_v82	Listeria_monocytogenes_1639_v119	0.9606
Listeria_monocytogenes_1639_v81	Listeria_monocytogenes_1639_v119	0.934
Listeria_monocytogenes_1639_v80	Listeria_monocytogenes_1639_v119	0.9533
Listeria_monocytogenes_1639_v7	Listeria_monocytogenes_1639_v119	0.9154
Listeria_monocytogenes_1639_v79	Listeria_monocytogenes_1639_v119	0.9319
Listeria_monocytogenes_1639_v78	Listeria_monocytogenes_1639_v119	0.9333
Listeria_monocytogenes_1639_v77	Listeria_monocytogenes_1639_v119	0.9331
Listeria_monocytogenes_1639_v76	Listeria_monocytogenes_1639_v119	0.9259
Listeria_monocytogenes_1639_v75	Listeria_monocytogenes_1639_v119	0.91
Listeria_monocytogenes_1639_v74	Listeria_monocytogenes_1639_v119	0.9287
Listeria_monocytogenes_1639_v73	Listeria_monocytogenes_1639_v119	0.9276
Listeria_monocytogenes_1639_v72	Listeria_monocytogenes_1639_v119	0.9601
Listeria_monocytogenes_1639_v71	Listeria_monocytogenes_1639_v119	0.9172
Listeria_monocytogenes_1639_v70	Listeria_monocytogenes_1639_v119	0.9303
Listeria_monocytogenes_1639_v6	Listeria_monocytogenes_1639_v119	0.9148
Listeria_monocytogenes_1639_v69	Listeria_monocytogenes_1639_v119	0.9633
Listeria_monocytogenes_1639_v68	Listeria_monocytogenes_1639_v119	0.9359
Listeria_monocytogenes_1639_v67	Listeria_monocytogenes_1639_v119	0.9515
Listeria_monocytogenes_1639_v66	Listeria_monocytogenes_1639_v119	0.9375
Listeria_monocytogenes_1639_v65	Listeria_monocytogenes_1639_v119	0.9296
Listeria_monocytogenes_1639_v64	Listeria_monocytogenes_1639_v119	0.93
Listeria_monocytogenes_1639_v63	Listeria_monocytogenes_1639_v119	0.9472
Listeria_monocytogenes_1639_v62	Listeria_monocytogenes_1639_v119	0.9298
Listeria_monocytogenes_1639_v61	Listeria_monocytogenes_1639_v119	0.9292
Listeria_monocytogenes_1639_v60	Listeria_monocytogenes_1639_v119	0.9336
Listeria_monocytogenes_1639_v5	Listeria_monocytogenes_1639_v119	0.913
Listeria_monocytogenes_1639_v59	Listeria_monocytogenes_1639_v119	0.962
Listeria_monocytogenes_1639_v58	Listeria_monocytogenes_1639_v119	0.9655
Listeria_monocytogenes_1639_v57	Listeria_monocytogenes_1639_v119	0.9595
Listeria_monocytogenes_1639_v56	Listeria_monocytogenes_1639_v119	0.9617
Listeria_monocytogenes_1639_v55	Listeria_monocytogenes_1639_v119	0.9551
Listeria_monocytogenes_1639_v54	Listeria_monocytogenes_1639_v119	0.9286
Listeria_monocytogenes_1639_v53	Listeria_monocytogenes_1639_v119	0.9333
Listeria_monocytogenes_1639_v52	Listeria_monocytogenes_1639_v119	0.958
Listeria_monocytogenes_1639_v51	Listeria_monocytogenes_1639_v119	0.9559
Listeria_monocytogenes_1639_v50	Listeria_monocytogenes_1639_v119	0.9256
Listeria_monocytogenes_1639_v4	Listeria_monocytogenes_1639_v119	0.9257
Listeria_monocytogenes_1639_v49	Listeria_monocytogenes_1639_v119	0.961
Listeria_monocytogenes_1639_v48	Listeria_monocytogenes_1639_v119	0.9282
Listeria_monocytogenes_1639_v47	Listeria_monocytogenes_1639_v119	0.9261
Listeria_monocytogenes_1639_v46	Listeria_monocytogenes_1639_v119	0.9352
Listeria_monocytogenes_1639_v45	Listeria_monocytogenes_1639_v119	0.9168
Listeria_monocytogenes_1639_v44	Listeria_monocytogenes_1639_v119	0.9264
Listeria_monocytogenes_1639_v43	Listeria_monocytogenes_1639_v119	0.9264
Listeria_monocytogenes_1639_v42	Listeria_monocytogenes_1639_v119	0.9175
Listeria_monocytogenes_1639_v41	Listeria_monocytogenes_1639_v119	0.9074
Listeria_monocytogenes_1639_v40	Listeria_monocytogenes_1639_v119	0.9328
Listeria_monocytogenes_1639_v3	Listeria_monocytogenes_1639_v119	0.9261
Listeria_monocytogenes_1639_v39	Listeria_monocytogenes_1639_v119	0.9483
Listeria_monocytogenes_1639_v38	Listeria_monocytogenes_1639_v119	0.93
Listeria_monocytogenes_1639_v37	Listeria_monocytogenes_1639_v119	0.9259
Listeria_monocytogenes_1639_v36	Listeria_monocytogenes_1639_v119	0.9223
Listeria_monocytogenes_1639_v35	Listeria_monocytogenes_1639_v119	0.9335
Listeria_monocytogenes_1639_v34	Listeria_monocytogenes_1639_v119	0.9278
Listeria_monocytogenes_1639_v33	Listeria_monocytogenes_1639_v119	0.9462
Listeria_monocytogenes_1639_v32	Listeria_monocytogenes_1639_v119	0.9248
Listeria_monocytogenes_1639_v31	Listeria_monocytogenes_1639_v119	0.9282
Listeria_monocytogenes_1639_v30	Listeria_monocytogenes_1639_v119	0.9498
Listeria_monocytogenes_1639_v2	Listeria_monocytogenes_1639_v119	0.9101
Listeria_monocytogenes_1639_v29	Listeria_monocytogenes_1639_v119	0.9193
Listeria_monocytogenes_1639_v28	Listeria_monocytogenes_1639_v119	0.9237

Listeria_monocytogenes_1639_v27	Listeria_monocytogenes_1639_v119	0.9145
Listeria_monocytogenes_1639_v26	Listeria_monocytogenes_1639_v119	0.9145
Listeria_monocytogenes_1639_v25	Listeria_monocytogenes_1639_v119	0.9142
Listeria_monocytogenes_1639_v24	Listeria_monocytogenes_1639_v119	0.9523
Listeria_monocytogenes_1639_v23	Listeria_monocytogenes_1639_v119	0.9523
Listeria_monocytogenes_1639_v22	Listeria_monocytogenes_1639_v119	0.9491
Listeria_monocytogenes_1639_v21	Listeria_monocytogenes_1639_v119	0.9519
Listeria_monocytogenes_1639_v20	Listeria_monocytogenes_1639_v119	0.9398
Listeria_monocytogenes_1639_v1	Listeria_monocytogenes_1639_v119	0.9096
Listeria_monocytogenes_1639_v19	Listeria_monocytogenes_1639_v119	0.9394
Listeria_monocytogenes_1639_v198	Listeria_monocytogenes_1639_v119	0.8999
Listeria_monocytogenes_1639_v197	Listeria_monocytogenes_1639_v119	0.9299
Listeria_monocytogenes_1639_v196	Listeria_monocytogenes_1639_v119	0.9245
Listeria_monocytogenes_1639_v195	Listeria_monocytogenes_1639_v119	0.9473
Listeria_monocytogenes_1639_v194	Listeria_monocytogenes_1639_v119	0.947
Listeria_monocytogenes_1639_v193	Listeria_monocytogenes_1639_v119	0.947
Listeria_monocytogenes_1639_v192	Listeria_monocytogenes_1639_v119	0.9126
Listeria_monocytogenes_1639_v191	Listeria_monocytogenes_1639_v119	0.9534
Listeria_monocytogenes_1639_v190	Listeria_monocytogenes_1639_v119	0.9535
Listeria_monocytogenes_1639_v18	Listeria_monocytogenes_1639_v119	0.9534
Listeria_monocytogenes_1639_v189	Listeria_monocytogenes_1639_v119	0.9527
Listeria_monocytogenes_1639_v188	Listeria_monocytogenes_1639_v119	0.9534
Listeria_monocytogenes_1639_v187	Listeria_monocytogenes_1639_v119	0.9531
Listeria_monocytogenes_1639_v186	Listeria_monocytogenes_1639_v119	0.9539
Listeria_monocytogenes_1639_v185	Listeria_monocytogenes_1639_v119	0.9535
Listeria_monocytogenes_1639_v184	Listeria_monocytogenes_1639_v119	0.9531
Listeria_monocytogenes_1639_v183	Listeria_monocytogenes_1639_v119	0.9481
Listeria_monocytogenes_1639_v182	Listeria_monocytogenes_1639_v119	0.9503
Listeria_monocytogenes_1639_v181	Listeria_monocytogenes_1639_v119	0.9337
Listeria_monocytogenes_1639_v180	Listeria_monocytogenes_1639_v119	0.9337
Listeria_monocytogenes_1639_v17	Listeria_monocytogenes_1639_v119	0.9534
Listeria_monocytogenes_1639_v179	Listeria_monocytogenes_1639_v119	0.9382
Listeria_monocytogenes_1639_v178	Listeria_monocytogenes_1639_v119	0.955
Listeria_monocytogenes_1639_v177	Listeria_monocytogenes_1639_v119	0.9823
Listeria_monocytogenes_1639_v176	Listeria_monocytogenes_1639_v119	0.9444
Listeria_monocytogenes_1639_v175	Listeria_monocytogenes_1639_v119	0.9424
Listeria_monocytogenes_1639_v174	Listeria_monocytogenes_1639_v119	0.9539
Listeria_monocytogenes_1639_v173	Listeria_monocytogenes_1639_v119	0.9714
Listeria_monocytogenes_1639_v172	Listeria_monocytogenes_1639_v119	0.9426
Listeria_monocytogenes_1639_v171	Listeria_monocytogenes_1639_v119	0.9386
Listeria_monocytogenes_1639_v170	Listeria_monocytogenes_1639_v119	0.9469
Listeria_monocytogenes_1639_v16	Listeria_monocytogenes_1639_v119	0.9127
Listeria_monocytogenes_1639_v169	Listeria_monocytogenes_1639_v119	0.9466
Listeria_monocytogenes_1639_v168	Listeria_monocytogenes_1639_v119	0.9474
Listeria_monocytogenes_1639_v167	Listeria_monocytogenes_1639_v119	0.94
Listeria_monocytogenes_1639_v166	Listeria_monocytogenes_1639_v119	0.9478
Listeria_monocytogenes_1639_v165	Listeria_monocytogenes_1639_v119	0.9466
Listeria_monocytogenes_1639_v164	Listeria_monocytogenes_1639_v119	0.978
Listeria_monocytogenes_1639_v163	Listeria_monocytogenes_1639_v119	0.968
Listeria_monocytogenes_1639_v162	Listeria_monocytogenes_1639_v119	0.978
Listeria_monocytogenes_1639_v161	Listeria_monocytogenes_1639_v119	0.978
Listeria_monocytogenes_1639_v160	Listeria_monocytogenes_1639_v119	0.978
Listeria_monocytogenes_1639_v15	Listeria_monocytogenes_1639_v119	0.9323
Listeria_monocytogenes_1639_v159	Listeria_monocytogenes_1639_v119	0.9827
Listeria_monocytogenes_1639_v158	Listeria_monocytogenes_1639_v119	0.9792
Listeria_monocytogenes_1639_v157	Listeria_monocytogenes_1639_v119	0.9788
Listeria_monocytogenes_1639_v156	Listeria_monocytogenes_1639_v119	0.9308
Listeria_monocytogenes_1639_v155	Listeria_monocytogenes_1639_v119	0.9413
Listeria_monocytogenes_1639_v154	Listeria_monocytogenes_1639_v119	0.9452
Listeria_monocytogenes_1639_v153	Listeria_monocytogenes_1639_v119	0.9421
Listeria_monocytogenes_1639_v152	Listeria_monocytogenes_1639_v119	0.9831
Listeria_monocytogenes_1639_v151	Listeria_monocytogenes_1639_v119	0.9831
Listeria_monocytogenes_1639_v150	Listeria_monocytogenes_1639_v119	0.9433
Listeria_monocytogenes_1639_v14	Listeria_monocytogenes_1639_v119	0.9396
Listeria_monocytogenes_1639_v149	Listeria_monocytogenes_1639_v119	0.9419
Listeria_monocytogenes_1639_v148	Listeria_monocytogenes_1639_v119	0.9816
Listeria_monocytogenes_1639_v147	Listeria_monocytogenes_1639_v119	0.9678
Listeria_monocytogenes_1639_v146	Listeria_monocytogenes_1639_v119	0.9517
Listeria_monocytogenes_1639_v145	Listeria_monocytogenes_1639_v119	0.9464
Listeria_monocytogenes_1639_v144	Listeria_monocytogenes_1639_v119	0.9803
Listeria_monocytogenes_1639_v143	Listeria_monocytogenes_1639_v119	0.9518
Listeria_monocytogenes_1639_v142	Listeria_monocytogenes_1639_v119	0.9807
Listeria_monocytogenes_1639_v141	Listeria_monocytogenes_1639_v119	0.9726
Listeria_monocytogenes_1639_v140	Listeria_monocytogenes_1639_v119	0.9811
Listeria_monocytogenes_1639_v13	Listeria_monocytogenes_1639_v119	0.9322
Listeria_monocytogenes_1639_v139	Listeria_monocytogenes_1639_v119	0.98
Listeria_monocytogenes_1639_v138	Listeria_monocytogenes_1639_v119	0.983
Listeria_monocytogenes_1639_v137	Listeria_monocytogenes_1639_v119	0.9827
Listeria_monocytogenes_1639_v136	Listeria_monocytogenes_1639_v119	0.9416
Listeria_monocytogenes_1639_v135	Listeria_monocytogenes_1639_v119	0.9213
Listeria_monocytogenes_1639_v134	Listeria_monocytogenes_1639_v119	0.9731
Listeria_monocytogenes_1639_v133	Listeria_monocytogenes_1639_v119	0.9387
Listeria_monocytogenes_1639_v132	Listeria_monocytogenes_1639_v119	0.9221
Listeria_monocytogenes_1639_v131	Listeria_monocytogenes_1639_v119	0.9707
Listeria_monocytogenes_1639_v130	Listeria_monocytogenes_1639_v119	0.9339
Listeria_monocytogenes_1639_v12	Listeria_monocytogenes_1639_v119	0.9132
Listeria_monocytogenes_1639_v129	Listeria_monocytogenes_1639_v119	0.9703
Listeria_monocytogenes_1639_v128	Listeria_monocytogenes_1639_v119	0.9681
Listeria_monocytogenes_1639_v127	Listeria_monocytogenes_1639_v119	0.9417
Listeria_monocytogenes_1639_v126	Listeria_monocytogenes_1639_v119	0.9666
Listeria_monocytogenes_1639_v125	Listeria_monocytogenes_1639_v119	0.9387

Listeria_monocytogenes_1639_v124	Listeria_monocytogenes_1639_v119	0.9403
Listeria_monocytogenes_1639_v123	Listeria_monocytogenes_1639_v119	0.9996
Listeria_monocytogenes_1639_v122	Listeria_monocytogenes_1639_v119	1
Listeria_monocytogenes_1639_v121	Listeria_monocytogenes_1639_v119	1
Listeria_monocytogenes_1639_v120	Listeria_monocytogenes_1639_v119	0.9996
Listeria_monocytogenes_1639_v11	Listeria_monocytogenes_1639_v119	0.9126
Listeria_monocytogenes_1639_v119	Listeria_monocytogenes_1639_v119	0.0677
Listeria_welshimeri_1643	Listeria_monocytogenes_1639_v11	0.8548
Listeria_welshimeri_serovar_6b_str_SLCC5334_386043	Listeria_monocytogenes_1639_v11	0.8542
Listeria_seeligeri_serovar_1_2b_str_SLCC3954_683837	Listeria_monocytogenes_1639_v11	0.8608
Listeria_seeligeri_FSL_S4-171_702452	Listeria_monocytogenes_1639_v11	0.8033
Listeria_seeligeri_FSL_N1-067_702453	Listeria_monocytogenes_1639_v11	0.8304
Listeria_seeligeri_1640_v1	Listeria_monocytogenes_1639_v11	0.8602
Listeria_seeligeri_1640	Listeria_monocytogenes_1639_v11	0.8653
Listeria_monocytogenes_WSLC1042_1457188	Listeria_monocytogenes_1639_v11	0.9043
Listeria_monocytogenes_WSLC1001_1457187	Listeria_monocytogenes_1639_v11	0.9388
Listeria_monocytogenes_SLCC7179_879090	Listeria_monocytogenes_1639_v11	0.9397
Listeria_monocytogenes_SLCC5850_882096	Listeria_monocytogenes_1639_v11	0.9389
Listeria_monocytogenes_SLCC2755_932919	Listeria_monocytogenes_1639_v11	0.916
Listeria_monocytogenes_SLCC2540_879089	Listeria_monocytogenes_1639_v11	0.9233
Listeria_monocytogenes_SLCC2479_882020	Listeria_monocytogenes_1639_v11	0.9417
Listeria_monocytogenes_SLCC2378_879088	Listeria_monocytogenes_1639_v11	0.9056
Listeria_monocytogenes_SLCC2376_882097	Listeria_monocytogenes_1639_v11	0.9108
Listeria_monocytogenes_SLCC2372_932920	Listeria_monocytogenes_1639_v11	0.9436
Listeria_monocytogenes_serotype_4b_str_LL195_1230340	Listeria_monocytogenes_1639_v11	0.911
Listeria_monocytogenes_serotype_4b_str_LIS0087_1134669	Listeria_monocytogenes_1639_v11	0.9272
Listeria_monocytogenes_serotype_4b_str_F2365_265669	Listeria_monocytogenes_1639_v11	0.911
Listeria_monocytogenes_serotype_4b_str_CLIP_80459_568819	Listeria_monocytogenes_1639_v11	0.912
Listeria_monocytogenes_serotype_4b_str_81-0861_1196161	Listeria_monocytogenes_1639_v11	0.9103
Listeria_monocytogenes_serotype_4b_str_81-0592_1196160	Listeria_monocytogenes_1639_v11	0.9113
Listeria_monocytogenes_serotype_4b_str_81-0558_1196159	Listeria_monocytogenes_1639_v11	0.9113
Listeria_monocytogenes_serotype_4b_str_10-0809_1196162	Listeria_monocytogenes_1639_v11	0.9113
Listeria_monocytogenes_serotype_4b_str_02-6680_1196175	Listeria_monocytogenes_1639_v11	0.9103
Listeria_monocytogenes_serotype_4b_str_02-6679_1196174	Listeria_monocytogenes_1639_v11	0.9103
Listeria_monocytogenes_serotype_4b_str_02-1792_1196173	Listeria_monocytogenes_1639_v11	0.9113
Listeria_monocytogenes_serotype_4b_str_02-1289_1196172	Listeria_monocytogenes_1639_v11	0.9113
Listeria_monocytogenes_serotype_4b_str_02-1103_1196171	Listeria_monocytogenes_1639_v11	0.9113
Listeria_monocytogenes_serotype_3c_str_10-5027_1196195	Listeria_monocytogenes_1639_v11	0.9454
Listeria_monocytogenes_serotype_1_2c_str_10-5026_1196194	Listeria_monocytogenes_1639_v11	0.9461
Listeria_monocytogenes_serotype_1_2c_str_10-5025_1196193	Listeria_monocytogenes_1639_v11	0.9461
Listeria_monocytogenes_serotype_1_2b_str_10-0811_1196164	Listeria_monocytogenes_1639_v11	0.9351
Listeria_monocytogenes_serotype_1_2b_str_10-0810_1196163	Listeria_monocytogenes_1639_v11	0.9351
Listeria_monocytogenes_serotype_1_2a_str_99-6370_1196179	Listeria_monocytogenes_1639_v11	0.9425
Listeria_monocytogenes_serotype_1_2a_str_98-2035_1196178	Listeria_monocytogenes_1639_v11	0.9425
Listeria_monocytogenes_serotype_1_2a_str_95-0093_1196177	Listeria_monocytogenes_1639_v11	0.9389
Listeria_monocytogenes_serotype_1_2a_str_88-0478_1196176	Listeria_monocytogenes_1639_v11	0.9388
Listeria_monocytogenes_serotype_1_2a_str_10-5024_1196192	Listeria_monocytogenes_1639_v11	0.9396
Listeria_monocytogenes_serotype_1_2a_str_10-4758_1196168	Listeria_monocytogenes_1639_v11	0.9408
Listeria_monocytogenes_serotype_1_2a_str_10-4754_1196167	Listeria_monocytogenes_1639_v11	0.9408
Listeria_monocytogenes_serotype_1_2a_str_10-1321_1196191	Listeria_monocytogenes_1639_v11	0.9396
Listeria_monocytogenes_serotype_1_2a_str_10-1047_1196190	Listeria_monocytogenes_1639_v11	0.9388
Listeria_monocytogenes_serotype_1_2a_str_10-1046_1196189	Listeria_monocytogenes_1639_v11	0.9388
Listeria_monocytogenes_serotype_1_2a_str_10-0934_1196170	Listeria_monocytogenes_1639_v11	0.9367
Listeria_monocytogenes_serotype_1_2a_str_10-0933_1196169	Listeria_monocytogenes_1639_v11	0.9367
Listeria_monocytogenes_serotype_1_2a_str_10-0815_1196188	Listeria_monocytogenes_1639_v11	0.9424
Listeria_monocytogenes_serotype_1_2a_str_10-0814_1196187	Listeria_monocytogenes_1639_v11	0.9392
Listeria_monocytogenes_serotype_1_2a_str_10-0813_1196166	Listeria_monocytogenes_1639_v11	0.9477
Listeria_monocytogenes_serotype_1_2a_str_10-0812_1196165	Listeria_monocytogenes_1639_v11	0.9477
Listeria_monocytogenes_serotype_1_2a_str_08-7669_1196186	Listeria_monocytogenes_1639_v11	0.9342
Listeria_monocytogenes_serotype_1_2a_str_08-7374_1196185	Listeria_monocytogenes_1639_v11	0.9396
Listeria_monocytogenes_serotype_1_2a_str_08-6997_1196184	Listeria_monocytogenes_1639_v11	0.9424
Listeria_monocytogenes_serotype_1_2a_str_08-6569_1196183	Listeria_monocytogenes_1639_v11	0.9424
Listeria_monocytogenes_serotype_1_2a_str_08-6056_1196182	Listeria_monocytogenes_1639_v11	0.9424
Listeria_monocytogenes_serotype_1_2a_str_04-5457_1196181	Listeria_monocytogenes_1639_v11	0.9396
Listeria_monocytogenes_serotype_1_2a_str_02-5993_1196180	Listeria_monocytogenes_1639_v11	0.9392
Listeria_monocytogenes_R479a_1437838	Listeria_monocytogenes_1639_v11	0.9483
Listeria_monocytogenes_N53-1_1234142	Listeria_monocytogenes_1639_v11	0.8955
Listeria_monocytogenes_M7_1030009	Listeria_monocytogenes_1639_v11	0.9028
Listeria_monocytogenes_LO28_393132	Listeria_monocytogenes_1639_v11	0.944
Listeria_monocytogenes_L99_563174	Listeria_monocytogenes_1639_v11	0.9035
Listeria_monocytogenes_L312_882094	Listeria_monocytogenes_1639_v11	0.912
Listeria_monocytogenes_J1816_930781	Listeria_monocytogenes_1639_v11	0.9091
Listeria_monocytogenes_J1-220_930782	Listeria_monocytogenes_1639_v11	0.9146
Listeria_monocytogenes_J0161_393130	Listeria_monocytogenes_1639_v11	0.9502
Listeria_monocytogenes_HCC23_552536	Listeria_monocytogenes_1639_v11	0.9028
Listeria_monocytogenes_FSL_R2-561_393126	Listeria_monocytogenes_1639_v11	0.9418
Listeria_monocytogenes_FSL_J1-208_393119	Listeria_monocytogenes_1639_v11	0.8825
Listeria_monocytogenes_FSL_J1-175_393118	Listeria_monocytogenes_1639_v11	0.9271
Listeria_monocytogenes_Finland_1998_393127	Listeria_monocytogenes_1639_v11	0.9344
Listeria_monocytogenes_EGD-e_169963_v1	Listeria_monocytogenes_1639_v11	0.9401
Listeria_monocytogenes_EGD-e_169963	Listeria_monocytogenes_1639_v11	0.9401
Listeria_monocytogenes_EGD_1334565	Listeria_monocytogenes_1639_v11	0.9396
Listeria_monocytogenes_CFSAN002349_1299895	Listeria_monocytogenes_1639_v11	0.9127
Listeria_monocytogenes_ATCC_19117_882095_v1	Listeria_monocytogenes_1639_v11	0.9091
Listeria_monocytogenes_ATCC_19117_882095	Listeria_monocytogenes_1639_v11	0.9091
Listeria_monocytogenes_6179_1288295	Listeria_monocytogenes_1639_v11	0.9878
Listeria_monocytogenes_1639_v9	Listeria_monocytogenes_1639_v11	0.9234
Listeria_monocytogenes_1639_v99	Listeria_monocytogenes_1639_v11	0.9164
Listeria_monocytogenes_1639_v98	Listeria_monocytogenes_1639_v11	0.9461
Listeria_monocytogenes_1639_v97	Listeria_monocytogenes_1639_v11	0.918
Listeria_monocytogenes_1639_v96	Listeria_monocytogenes_1639_v11	0.9147

Listeria_monocytogenes_1639_v95	Listeria_monocytogenes_1639_v11	0.9147
Listeria_monocytogenes_1639_v94	Listeria_monocytogenes_1639_v11	0.9147
Listeria_monocytogenes_1639_v93	Listeria_monocytogenes_1639_v11	0.9508
Listeria_monocytogenes_1639_v92	Listeria_monocytogenes_1639_v11	0.9157
Listeria_monocytogenes_1639_v91	Listeria_monocytogenes_1639_v11	0.9163
Listeria_monocytogenes_1639_v90	Listeria_monocytogenes_1639_v11	0.9461
Listeria_monocytogenes_1639_v8	Listeria_monocytogenes_1639_v11	0.9428
Listeria_monocytogenes_1639_v89	Listeria_monocytogenes_1639_v11	0.9176
Listeria_monocytogenes_1639_v88	Listeria_monocytogenes_1639_v11	0.9423
Listeria_monocytogenes_1639_v87	Listeria_monocytogenes_1639_v11	0.9454
Listeria_monocytogenes_1639_v86	Listeria_monocytogenes_1639_v11	0.9447
Listeria_monocytogenes_1639_v85	Listeria_monocytogenes_1639_v11	0.9101
Listeria_monocytogenes_1639_v84	Listeria_monocytogenes_1639_v11	0.9444
Listeria_monocytogenes_1639_v83	Listeria_monocytogenes_1639_v11	0.9123
Listeria_monocytogenes_1639_v82	Listeria_monocytogenes_1639_v11	0.9116
Listeria_monocytogenes_1639_v81	Listeria_monocytogenes_1639_v11	0.9456
Listeria_monocytogenes_1639_v80	Listeria_monocytogenes_1639_v11	0.9294
Listeria_monocytogenes_1639_v7	Listeria_monocytogenes_1639_v11	0.9428
Listeria_monocytogenes_1639_v79	Listeria_monocytogenes_1639_v11	0.9431
Listeria_monocytogenes_1639_v78	Listeria_monocytogenes_1639_v11	0.946
Listeria_monocytogenes_1639_v77	Listeria_monocytogenes_1639_v11	0.9355
Listeria_monocytogenes_1639_v76	Listeria_monocytogenes_1639_v11	0.9457
Listeria_monocytogenes_1639_v75	Listeria_monocytogenes_1639_v11	0.9035
Listeria_monocytogenes_1639_v74	Listeria_monocytogenes_1639_v11	0.9335
Listeria_monocytogenes_1639_v73	Listeria_monocytogenes_1639_v11	0.9461
Listeria_monocytogenes_1639_v72	Listeria_monocytogenes_1639_v11	0.9115
Listeria_monocytogenes_1639_v71	Listeria_monocytogenes_1639_v11	0.9401
Listeria_monocytogenes_1639_v70	Listeria_monocytogenes_1639_v11	0.9441
Listeria_monocytogenes_1639_v6	Listeria_monocytogenes_1639_v11	0.9421
Listeria_monocytogenes_1639_v69	Listeria_monocytogenes_1639_v11	0.9117
Listeria_monocytogenes_1639_v68	Listeria_monocytogenes_1639_v11	0.9415
Listeria_monocytogenes_1639_v67	Listeria_monocytogenes_1639_v11	0.9302
Listeria_monocytogenes_1639_v66	Listeria_monocytogenes_1639_v11	0.9423
Listeria_monocytogenes_1639_v65	Listeria_monocytogenes_1639_v11	0.9499
Listeria_monocytogenes_1639_v64	Listeria_monocytogenes_1639_v11	0.9457
Listeria_monocytogenes_1639_v63	Listeria_monocytogenes_1639_v11	0.9277
Listeria_monocytogenes_1639_v62	Listeria_monocytogenes_1639_v11	0.9509
Listeria_monocytogenes_1639_v61	Listeria_monocytogenes_1639_v11	0.9397
Listeria_monocytogenes_1639_v60	Listeria_monocytogenes_1639_v11	0.9463
Listeria_monocytogenes_1639_v5	Listeria_monocytogenes_1639_v11	0.9066
Listeria_monocytogenes_1639_v59	Listeria_monocytogenes_1639_v11	0.9097
Listeria_monocytogenes_1639_v58	Listeria_monocytogenes_1639_v11	0.9112
Listeria_monocytogenes_1639_v57	Listeria_monocytogenes_1639_v11	0.9081
Listeria_monocytogenes_1639_v56	Listeria_monocytogenes_1639_v11	0.9148
Listeria_monocytogenes_1639_v55	Listeria_monocytogenes_1639_v11	0.9153
Listeria_monocytogenes_1639_v54	Listeria_monocytogenes_1639_v11	0.9051
Listeria_monocytogenes_1639_v53	Listeria_monocytogenes_1639_v11	0.9247
Listeria_monocytogenes_1639_v52	Listeria_monocytogenes_1639_v11	0.918
Listeria_monocytogenes_1639_v51	Listeria_monocytogenes_1639_v11	0.9138
Listeria_monocytogenes_1639_v50	Listeria_monocytogenes_1639_v11	0.9381
Listeria_monocytogenes_1639_v4	Listeria_monocytogenes_1639_v11	0.9142
Listeria_monocytogenes_1639_v49	Listeria_monocytogenes_1639_v11	0.8993
Listeria_monocytogenes_1639_v48	Listeria_monocytogenes_1639_v11	0.9493
Listeria_monocytogenes_1639_v47	Listeria_monocytogenes_1639_v11	0.9381
Listeria_monocytogenes_1639_v46	Listeria_monocytogenes_1639_v11	0.9401
Listeria_monocytogenes_1639_v45	Listeria_monocytogenes_1639_v11	0.9427
Listeria_monocytogenes_1639_v44	Listeria_monocytogenes_1639_v11	0.9396
Listeria_monocytogenes_1639_v43	Listeria_monocytogenes_1639_v11	0.9396
Listeria_monocytogenes_1639_v42	Listeria_monocytogenes_1639_v11	0.9359
Listeria_monocytogenes_1639_v41	Listeria_monocytogenes_1639_v11	0.9342
Listeria_monocytogenes_1639_v40	Listeria_monocytogenes_1639_v11	0.9454
Listeria_monocytogenes_1639_v3	Listeria_monocytogenes_1639_v11	0.9149
Listeria_monocytogenes_1639_v39	Listeria_monocytogenes_1639_v11	0.9295
Listeria_monocytogenes_1639_v38	Listeria_monocytogenes_1639_v11	0.9246
Listeria_monocytogenes_1639_v37	Listeria_monocytogenes_1639_v11	0.9217
Listeria_monocytogenes_1639_v36	Listeria_monocytogenes_1639_v11	0.9395
Listeria_monocytogenes_1639_v35	Listeria_monocytogenes_1639_v11	0.9428
Listeria_monocytogenes_1639_v34	Listeria_monocytogenes_1639_v11	0.9471
Listeria_monocytogenes_1639_v33	Listeria_monocytogenes_1639_v11	0.9351
Listeria_monocytogenes_1639_v32	Listeria_monocytogenes_1639_v11	0.9366
Listeria_monocytogenes_1639_v31	Listeria_monocytogenes_1639_v11	0.9475
Listeria_monocytogenes_1639_v30	Listeria_monocytogenes_1639_v11	0.9164
Listeria_monocytogenes_1639_v2	Listeria_monocytogenes_1639_v11	0.9073
Listeria_monocytogenes_1639_v29	Listeria_monocytogenes_1639_v11	0.9379
Listeria_monocytogenes_1639_v28	Listeria_monocytogenes_1639_v11	0.9387
Listeria_monocytogenes_1639_v27	Listeria_monocytogenes_1639_v11	0.9375
Listeria_monocytogenes_1639_v26	Listeria_monocytogenes_1639_v11	0.9376
Listeria_monocytogenes_1639_v25	Listeria_monocytogenes_1639_v11	0.9372
Listeria_monocytogenes_1639_v24	Listeria_monocytogenes_1639_v11	0.9131
Listeria_monocytogenes_1639_v23	Listeria_monocytogenes_1639_v11	0.9127
Listeria_monocytogenes_1639_v22	Listeria_monocytogenes_1639_v11	0.9364
Listeria_monocytogenes_1639_v21	Listeria_monocytogenes_1639_v11	0.9135
Listeria_monocytogenes_1639_v20	Listeria_monocytogenes_1639_v11	0.9365
Listeria_monocytogenes_1639_v1	Listeria_monocytogenes_1639_v11	0.995
Listeria_monocytogenes_1639_v19	Listeria_monocytogenes_1639_v11	0.9358
Listeria_monocytogenes_1639_v198	Listeria_monocytogenes_1639_v11	0.896
Listeria_monocytogenes_1639_v197	Listeria_monocytogenes_1639_v11	0.9274
Listeria_monocytogenes_1639_v196	Listeria_monocytogenes_1639_v11	0.9229
Listeria_monocytogenes_1639_v195	Listeria_monocytogenes_1639_v11	0.9118
Listeria_monocytogenes_1639_v194	Listeria_monocytogenes_1639_v11	0.9111
Listeria_monocytogenes_1639_v193	Listeria_monocytogenes_1639_v11	0.9115

Listeria_monocytogenes_1639_v192	Listeria_monocytogenes_1639_v11	0.88
Listeria_monocytogenes_1639_v191	Listeria_monocytogenes_1639_v11	0.9402
Listeria_monocytogenes_1639_v190	Listeria_monocytogenes_1639_v11	0.9358
Listeria_monocytogenes_1639_v18	Listeria_monocytogenes_1639_v11	0.9098
Listeria_monocytogenes_1639_v189	Listeria_monocytogenes_1639_v11	0.9351
Listeria_monocytogenes_1639_v188	Listeria_monocytogenes_1639_v11	0.9331
Listeria_monocytogenes_1639_v187	Listeria_monocytogenes_1639_v11	0.9354
Listeria_monocytogenes_1639_v186	Listeria_monocytogenes_1639_v11	0.9362
Listeria_monocytogenes_1639_v185	Listeria_monocytogenes_1639_v11	0.9358
Listeria_monocytogenes_1639_v184	Listeria_monocytogenes_1639_v11	0.9354
Listeria_monocytogenes_1639_v183	Listeria_monocytogenes_1639_v11	0.9325
Listeria_monocytogenes_1639_v182	Listeria_monocytogenes_1639_v11	0.9346
Listeria_monocytogenes_1639_v181	Listeria_monocytogenes_1639_v11	0.8966
Listeria_monocytogenes_1639_v180	Listeria_monocytogenes_1639_v11	0.8966
Listeria_monocytogenes_1639_v17	Listeria_monocytogenes_1639_v11	0.9098
Listeria_monocytogenes_1639_v179	Listeria_monocytogenes_1639_v11	0.9405
Listeria_monocytogenes_1639_v178	Listeria_monocytogenes_1639_v11	0.9388
Listeria_monocytogenes_1639_v177	Listeria_monocytogenes_1639_v11	0.9119
Listeria_monocytogenes_1639_v176	Listeria_monocytogenes_1639_v11	0.909
Listeria_monocytogenes_1639_v175	Listeria_monocytogenes_1639_v11	0.9419
Listeria_monocytogenes_1639_v174	Listeria_monocytogenes_1639_v11	0.9385
Listeria_monocytogenes_1639_v173	Listeria_monocytogenes_1639_v11	0.9214
Listeria_monocytogenes_1639_v172	Listeria_monocytogenes_1639_v11	0.9136
Listeria_monocytogenes_1639_v171	Listeria_monocytogenes_1639_v11	0.9449
Listeria_monocytogenes_1639_v170	Listeria_monocytogenes_1639_v11	0.9429
Listeria_monocytogenes_1639_v16	Listeria_monocytogenes_1639_v11	0.9461
Listeria_monocytogenes_1639_v169	Listeria_monocytogenes_1639_v11	0.9441
Listeria_monocytogenes_1639_v168	Listeria_monocytogenes_1639_v11	0.9338
Listeria_monocytogenes_1639_v167	Listeria_monocytogenes_1639_v11	0.9418
Listeria_monocytogenes_1639_v166	Listeria_monocytogenes_1639_v11	0.9341
Listeria_monocytogenes_1639_v165	Listeria_monocytogenes_1639_v11	0.9438
Listeria_monocytogenes_1639_v164	Listeria_monocytogenes_1639_v11	0.9101
Listeria_monocytogenes_1639_v163	Listeria_monocytogenes_1639_v11	0.9199
Listeria_monocytogenes_1639_v162	Listeria_monocytogenes_1639_v11	0.9101
Listeria_monocytogenes_1639_v161	Listeria_monocytogenes_1639_v11	0.9109
Listeria_monocytogenes_1639_v160	Listeria_monocytogenes_1639_v11	0.9109
Listeria_monocytogenes_1639_v15	Listeria_monocytogenes_1639_v11	0.9215
Listeria_monocytogenes_1639_v159	Listeria_monocytogenes_1639_v11	0.9119
Listeria_monocytogenes_1639_v158	Listeria_monocytogenes_1639_v11	0.911
Listeria_monocytogenes_1639_v157	Listeria_monocytogenes_1639_v11	0.911
Listeria_monocytogenes_1639_v156	Listeria_monocytogenes_1639_v11	0.9344
Listeria_monocytogenes_1639_v155	Listeria_monocytogenes_1639_v11	0.9387
Listeria_monocytogenes_1639_v154	Listeria_monocytogenes_1639_v11	0.9399
Listeria_monocytogenes_1639_v153	Listeria_monocytogenes_1639_v11	0.9405
Listeria_monocytogenes_1639_v152	Listeria_monocytogenes_1639_v11	0.9122
Listeria_monocytogenes_1639_v151	Listeria_monocytogenes_1639_v11	0.9122
Listeria_monocytogenes_1639_v150	Listeria_monocytogenes_1639_v11	0.9391
Listeria_monocytogenes_1639_v14	Listeria_monocytogenes_1639_v11	0.9216
Listeria_monocytogenes_1639_v149	Listeria_monocytogenes_1639_v11	0.9396
Listeria_monocytogenes_1639_v148	Listeria_monocytogenes_1639_v11	0.9108
Listeria_monocytogenes_1639_v147	Listeria_monocytogenes_1639_v11	0.9052
Listeria_monocytogenes_1639_v146	Listeria_monocytogenes_1639_v11	0.9382
Listeria_monocytogenes_1639_v145	Listeria_monocytogenes_1639_v11	0.9347
Listeria_monocytogenes_1639_v144	Listeria_monocytogenes_1639_v11	0.9113
Listeria_monocytogenes_1639_v143	Listeria_monocytogenes_1639_v11	0.9402
Listeria_monocytogenes_1639_v142	Listeria_monocytogenes_1639_v11	0.9117
Listeria_monocytogenes_1639_v141	Listeria_monocytogenes_1639_v11	0.919
Listeria_monocytogenes_1639_v140	Listeria_monocytogenes_1639_v11	0.912
Listeria_monocytogenes_1639_v13	Listeria_monocytogenes_1639_v11	0.9218
Listeria_monocytogenes_1639_v139	Listeria_monocytogenes_1639_v11	0.9088
Listeria_monocytogenes_1639_v138	Listeria_monocytogenes_1639_v11	0.9143
Listeria_monocytogenes_1639_v137	Listeria_monocytogenes_1639_v11	0.9139
Listeria_monocytogenes_1639_v136	Listeria_monocytogenes_1639_v11	0.939
Listeria_monocytogenes_1639_v135	Listeria_monocytogenes_1639_v11	0.9015
Listeria_monocytogenes_1639_v134	Listeria_monocytogenes_1639_v11	0.9054
Listeria_monocytogenes_1639_v133	Listeria_monocytogenes_1639_v11	0.9432
Listeria_monocytogenes_1639_v132	Listeria_monocytogenes_1639_v11	0.9002
Listeria_monocytogenes_1639_v131	Listeria_monocytogenes_1639_v11	0.9142
Listeria_monocytogenes_1639_v130	Listeria_monocytogenes_1639_v11	0.9395
Listeria_monocytogenes_1639_v12	Listeria_monocytogenes_1639_v11	0.9394
Listeria_monocytogenes_1639_v129	Listeria_monocytogenes_1639_v11	0.9183
Listeria_monocytogenes_1639_v128	Listeria_monocytogenes_1639_v11	0.9152
Listeria_monocytogenes_1639_v127	Listeria_monocytogenes_1639_v11	0.9479
Listeria_monocytogenes_1639_v126	Listeria_monocytogenes_1639_v11	0.9164
Listeria_monocytogenes_1639_v125	Listeria_monocytogenes_1639_v11	0.9391
Listeria_monocytogenes_1639_v124	Listeria_monocytogenes_1639_v11	0.8997
Listeria_monocytogenes_1639_v123	Listeria_monocytogenes_1639_v11	0.9127
Listeria_monocytogenes_1639_v122	Listeria_monocytogenes_1639_v11	0.9126
Listeria_monocytogenes_1639_v121	Listeria_monocytogenes_1639_v11	0.9126
Listeria_monocytogenes_1639_v120	Listeria_monocytogenes_1639_v11	0.9123
Listeria_monocytogenes_1639_v11	Listeria_monocytogenes_1639_v11	0.0764
Listeria_welshimeri_1643	Listeria_monocytogenes_1639_v120	0.8836
Listeria_welshimeri_serovar_6b_str_SLCC5334_386043	Listeria_monocytogenes_1639_v120	0.8832
Listeria_seeligeri_serovar_1_2b_str_SLCC3954_683837	Listeria_monocytogenes_1639_v120	0.8912
Listeria_seeligeri_FSL_S4-171_702452	Listeria_monocytogenes_1639_v120	0.8312
Listeria_seeligeri_FSL_N1-067_702453	Listeria_monocytogenes_1639_v120	0.83
Listeria_seeligeri_1640_v1	Listeria_monocytogenes_1639_v120	0.8992
Listeria_seeligeri_1640	Listeria_monocytogenes_1639_v120	0.8869
Listeria_monocytogenes_WSLC1042_1457188	Listeria_monocytogenes_1639_v120	0.9721
Listeria_monocytogenes_WSLC1001_1457187	Listeria_monocytogenes_1639_v120	0.9365
Listeria_monocytogenes_SLCC7179_879090	Listeria_monocytogenes_1639_v120	0.942

Listeria_monocytogenes_SLCC5850_882096	Listeria_monocytogenes_1639_v120	0.9407
Listeria_monocytogenes_SLCC2755_932919	Listeria_monocytogenes_1639_v120	0.9522
Listeria_monocytogenes_SLCC2540_879089	Listeria_monocytogenes_1639_v120	0.9513
Listeria_monocytogenes_SLCC2479_882020	Listeria_monocytogenes_1639_v120	0.9339
Listeria_monocytogenes_SLCC2378_879088	Listeria_monocytogenes_1639_v120	0.9728
Listeria_monocytogenes_SLCC2376_882097	Listeria_monocytogenes_1639_v120	0.9462
Listeria_monocytogenes_SLCC2372_932920	Listeria_monocytogenes_1639_v120	0.9265
Listeria_monocytogenes_serotype_4b_str_LL195_1230340	Listeria_monocytogenes_1639_v120	0.9784
Listeria_monocytogenes_serotype_4b_str_LIS0087_1134669	Listeria_monocytogenes_1639_v120	0.9171
Listeria_monocytogenes_serotype_4b_str_F2365_265669	Listeria_monocytogenes_1639_v120	0.9784
Listeria_monocytogenes_serotype_4b_str_CLIP_80459_568819	Listeria_monocytogenes_1639_v120	0.9803
Listeria_monocytogenes_serotype_4b_str_81-0861_1196161	Listeria_monocytogenes_1639_v120	0.9618
Listeria_monocytogenes_serotype_4b_str_81-0592_1196160	Listeria_monocytogenes_1639_v120	0.9629
Listeria_monocytogenes_serotype_4b_str_81-0558_1196159	Listeria_monocytogenes_1639_v120	0.9617
Listeria_monocytogenes_serotype_4b_str_10-0809_1196162	Listeria_monocytogenes_1639_v120	0.9629
Listeria_monocytogenes_serotype_4b_str_02-6680_1196175	Listeria_monocytogenes_1639_v120	0.9773
Listeria_monocytogenes_serotype_4b_str_02-6679_1196174	Listeria_monocytogenes_1639_v120	0.9773
Listeria_monocytogenes_serotype_4b_str_02-1792_1196173	Listeria_monocytogenes_1639_v120	0.9629
Listeria_monocytogenes_serotype_4b_str_02-1289_1196172	Listeria_monocytogenes_1639_v120	0.9629
Listeria_monocytogenes_serotype_4b_str_02-1103_1196171	Listeria_monocytogenes_1639_v120	0.9629
Listeria_monocytogenes_serotype_3c_str_10-5027_1196195	Listeria_monocytogenes_1639_v120	0.9331
Listeria_monocytogenes_serotype_1_2c_str_10-5026_1196194	Listeria_monocytogenes_1639_v120	0.9339
Listeria_monocytogenes_serotype_1_2c_str_10-5025_1196193	Listeria_monocytogenes_1639_v120	0.9339
Listeria_monocytogenes_serotype_1_2b_str_10-0811_1196164	Listeria_monocytogenes_1639_v120	0.9462
Listeria_monocytogenes_serotype_1_2b_str_10-0810_1196163	Listeria_monocytogenes_1639_v120	0.9462
Listeria_monocytogenes_serotype_1_2a_str_99-6370_1196179	Listeria_monocytogenes_1639_v120	0.9206
Listeria_monocytogenes_serotype_1_2a_str_98-2035_1196178	Listeria_monocytogenes_1639_v120	0.9206
Listeria_monocytogenes_serotype_1_2a_str_95-0093_1196177	Listeria_monocytogenes_1639_v120	0.9261
Listeria_monocytogenes_serotype_1_2a_str_88-0478_1196176	Listeria_monocytogenes_1639_v120	0.9249
Listeria_monocytogenes_serotype_1_2a_str_10-5024_1196192	Listeria_monocytogenes_1639_v120	0.9261
Listeria_monocytogenes_serotype_1_2a_str_10-4758_1196168	Listeria_monocytogenes_1639_v120	0.942
Listeria_monocytogenes_serotype_1_2a_str_10-4754_1196167	Listeria_monocytogenes_1639_v120	0.942
Listeria_monocytogenes_serotype_1_2a_str_10-1321_1196191	Listeria_monocytogenes_1639_v120	0.9261
Listeria_monocytogenes_serotype_1_2a_str_10-1047_1196190	Listeria_monocytogenes_1639_v120	0.9267
Listeria_monocytogenes_serotype_1_2a_str_10-1046_1196189	Listeria_monocytogenes_1639_v120	0.9267
Listeria_monocytogenes_serotype_1_2a_str_10-0934_1196170	Listeria_monocytogenes_1639_v120	0.9532
Listeria_monocytogenes_serotype_1_2a_str_10-0933_1196169	Listeria_monocytogenes_1639_v120	0.9532
Listeria_monocytogenes_serotype_1_2a_str_10-0815_1196188	Listeria_monocytogenes_1639_v120	0.9219
Listeria_monocytogenes_serotype_1_2a_str_10-0814_1196187	Listeria_monocytogenes_1639_v120	0.9264
Listeria_monocytogenes_serotype_1_2a_str_10-0813_1196166	Listeria_monocytogenes_1639_v120	0.934
Listeria_monocytogenes_serotype_1_2a_str_10-0812_1196165	Listeria_monocytogenes_1639_v120	0.934
Listeria_monocytogenes_serotype_1_2a_str_08-7669_1196186	Listeria_monocytogenes_1639_v120	0.9355
Listeria_monocytogenes_serotype_1_2a_str_08-7374_1196185	Listeria_monocytogenes_1639_v120	0.9261
Listeria_monocytogenes_serotype_1_2a_str_08-6997_1196184	Listeria_monocytogenes_1639_v120	0.9219
Listeria_monocytogenes_serotype_1_2a_str_08-6569_1196183	Listeria_monocytogenes_1639_v120	0.9219
Listeria_monocytogenes_serotype_1_2a_str_08-6056_1196182	Listeria_monocytogenes_1639_v120	0.9219
Listeria_monocytogenes_serotype_1_2a_str_04-5457_1196181	Listeria_monocytogenes_1639_v120	0.9261
Listeria_monocytogenes_serotype_1_2a_str_02-5993_1196180	Listeria_monocytogenes_1639_v120	0.9261
Listeria_monocytogenes_R479a_1437838	Listeria_monocytogenes_1639_v120	0.9257
Listeria_monocytogenes_N53-1_1234142	Listeria_monocytogenes_1639_v120	0.8972
Listeria_monocytogenes_M7_1030009	Listeria_monocytogenes_1639_v120	0.9093
Listeria_monocytogenes_LO28_393132	Listeria_monocytogenes_1639_v120	0.9325
Listeria_monocytogenes_L99_563174	Listeria_monocytogenes_1639_v120	0.9096
Listeria_monocytogenes_L312_882094	Listeria_monocytogenes_1639_v120	0.9807
Listeria_monocytogenes_J1816_930781	Listeria_monocytogenes_1639_v120	0.9619
Listeria_monocytogenes_J1-220_930782	Listeria_monocytogenes_1639_v120	0.9523
Listeria_monocytogenes_J0161_393130	Listeria_monocytogenes_1639_v120	0.9313
Listeria_monocytogenes_HCC23_552536	Listeria_monocytogenes_1639_v120	0.9089
Listeria_monocytogenes_FSL_R2-561_393126	Listeria_monocytogenes_1639_v120	0.9324
Listeria_monocytogenes_FSL_J1-208_393119	Listeria_monocytogenes_1639_v120	0.9212
Listeria_monocytogenes_FSL_J1-175_393118	Listeria_monocytogenes_1639_v120	0.9614
Listeria_monocytogenes_Finland_1998_393127	Listeria_monocytogenes_1639_v120	0.9536
Listeria_monocytogenes_EGD-e_169963_v1	Listeria_monocytogenes_1639_v120	0.9421
Listeria_monocytogenes_EGD-e_169963	Listeria_monocytogenes_1639_v120	0.9424
Listeria_monocytogenes_EGD_1334565	Listeria_monocytogenes_1639_v120	0.9415
Listeria_monocytogenes_CFSAN002349_1299895	Listeria_monocytogenes_1639_v120	0.9996
Listeria_monocytogenes_ATCC_19117_882095_v1	Listeria_monocytogenes_1639_v120	0.9728
Listeria_monocytogenes_ATCC_19117_882095	Listeria_monocytogenes_1639_v120	0.9724
Listeria_monocytogenes_6179_1288295	Listeria_monocytogenes_1639_v120	0.9152
Listeria_monocytogenes_1639_v9	Listeria_monocytogenes_1639_v120	0.9317
Listeria_monocytogenes_1639_v99	Listeria_monocytogenes_1639_v120	0.9631
Listeria_monocytogenes_1639_v98	Listeria_monocytogenes_1639_v120	0.9286
Listeria_monocytogenes_1639_v97	Listeria_monocytogenes_1639_v120	0.9688
Listeria_monocytogenes_1639_v96	Listeria_monocytogenes_1639_v120	0.9623
Listeria_monocytogenes_1639_v95	Listeria_monocytogenes_1639_v120	0.9623
Listeria_monocytogenes_1639_v94	Listeria_monocytogenes_1639_v120	0.9627
Listeria_monocytogenes_1639_v93	Listeria_monocytogenes_1639_v120	0.9294
Listeria_monocytogenes_1639_v92	Listeria_monocytogenes_1639_v120	0.9627
Listeria_monocytogenes_1639_v91	Listeria_monocytogenes_1639_v120	0.9581
Listeria_monocytogenes_1639_v90	Listeria_monocytogenes_1639_v120	0.9286
Listeria_monocytogenes_1639_v8	Listeria_monocytogenes_1639_v120	0.9151
Listeria_monocytogenes_1639_v89	Listeria_monocytogenes_1639_v120	0.9706
Listeria_monocytogenes_1639_v88	Listeria_monocytogenes_1639_v120	0.9337
Listeria_monocytogenes_1639_v87	Listeria_monocytogenes_1639_v120	0.9294
Listeria_monocytogenes_1639_v86	Listeria_monocytogenes_1639_v120	0.9291
Listeria_monocytogenes_1639_v85	Listeria_monocytogenes_1639_v120	0.9615
Listeria_monocytogenes_1639_v84	Listeria_monocytogenes_1639_v120	0.9366
Listeria_monocytogenes_1639_v83	Listeria_monocytogenes_1639_v120	0.9613
Listeria_monocytogenes_1639_v82	Listeria_monocytogenes_1639_v120	0.961
Listeria_monocytogenes_1639_v81	Listeria_monocytogenes_1639_v120	0.9337
Listeria_monocytogenes_1639_v80	Listeria_monocytogenes_1639_v120	0.9529

Listeria_monocytogenes_1639_v7	Listeria_monocytogenes_1639_v120	0.9151
Listeria_monocytogenes_1639_v79	Listeria_monocytogenes_1639_v120	0.9319
Listeria_monocytogenes_1639_v78	Listeria_monocytogenes_1639_v120	0.933
Listeria_monocytogenes_1639_v77	Listeria_monocytogenes_1639_v120	0.9331
Listeria_monocytogenes_1639_v76	Listeria_monocytogenes_1639_v120	0.9259
Listeria_monocytogenes_1639_v75	Listeria_monocytogenes_1639_v120	0.9096
Listeria_monocytogenes_1639_v74	Listeria_monocytogenes_1639_v120	0.9287
Listeria_monocytogenes_1639_v73	Listeria_monocytogenes_1639_v120	0.9276
Listeria_monocytogenes_1639_v72	Listeria_monocytogenes_1639_v120	0.9598
Listeria_monocytogenes_1639_v71	Listeria_monocytogenes_1639_v120	0.9172
Listeria_monocytogenes_1639_v70	Listeria_monocytogenes_1639_v120	0.93
Listeria_monocytogenes_1639_v6	Listeria_monocytogenes_1639_v120	0.9148
Listeria_monocytogenes_1639_v69	Listeria_monocytogenes_1639_v120	0.9629
Listeria_monocytogenes_1639_v68	Listeria_monocytogenes_1639_v120	0.9356
Listeria_monocytogenes_1639_v67	Listeria_monocytogenes_1639_v120	0.9511
Listeria_monocytogenes_1639_v66	Listeria_monocytogenes_1639_v120	0.9371
Listeria_monocytogenes_1639_v65	Listeria_monocytogenes_1639_v120	0.9292
Listeria_monocytogenes_1639_v64	Listeria_monocytogenes_1639_v120	0.9296
Listeria_monocytogenes_1639_v63	Listeria_monocytogenes_1639_v120	0.9472
Listeria_monocytogenes_1639_v62	Listeria_monocytogenes_1639_v120	0.9295
Listeria_monocytogenes_1639_v61	Listeria_monocytogenes_1639_v120	0.9288
Listeria_monocytogenes_1639_v60	Listeria_monocytogenes_1639_v120	0.9333
Listeria_monocytogenes_1639_v5	Listeria_monocytogenes_1639_v120	0.9127
Listeria_monocytogenes_1639_v59	Listeria_monocytogenes_1639_v120	0.9617
Listeria_monocytogenes_1639_v58	Listeria_monocytogenes_1639_v120	0.9655
Listeria_monocytogenes_1639_v57	Listeria_monocytogenes_1639_v120	0.9591
Listeria_monocytogenes_1639_v56	Listeria_monocytogenes_1639_v120	0.9617
Listeria_monocytogenes_1639_v55	Listeria_monocytogenes_1639_v120	0.9548
Listeria_monocytogenes_1639_v54	Listeria_monocytogenes_1639_v120	0.9286
Listeria_monocytogenes_1639_v53	Listeria_monocytogenes_1639_v120	0.9333
Listeria_monocytogenes_1639_v52	Listeria_monocytogenes_1639_v120	0.958
Listeria_monocytogenes_1639_v51	Listeria_monocytogenes_1639_v120	0.9555
Listeria_monocytogenes_1639_v50	Listeria_monocytogenes_1639_v120	0.9253
Listeria_monocytogenes_1639_v4	Listeria_monocytogenes_1639_v120	0.9257
Listeria_monocytogenes_1639_v49	Listeria_monocytogenes_1639_v120	0.9607
Listeria_monocytogenes_1639_v48	Listeria_monocytogenes_1639_v120	0.9282
Listeria_monocytogenes_1639_v47	Listeria_monocytogenes_1639_v120	0.9257
Listeria_monocytogenes_1639_v46	Listeria_monocytogenes_1639_v120	0.9348
Listeria_monocytogenes_1639_v45	Listeria_monocytogenes_1639_v120	0.9168
Listeria_monocytogenes_1639_v44	Listeria_monocytogenes_1639_v120	0.9261
Listeria_monocytogenes_1639_v43	Listeria_monocytogenes_1639_v120	0.9261
Listeria_monocytogenes_1639_v42	Listeria_monocytogenes_1639_v120	0.9179
Listeria_monocytogenes_1639_v41	Listeria_monocytogenes_1639_v120	0.9074
Listeria_monocytogenes_1639_v40	Listeria_monocytogenes_1639_v120	0.9328
Listeria_monocytogenes_1639_v3	Listeria_monocytogenes_1639_v120	0.9261
Listeria_monocytogenes_1639_v39	Listeria_monocytogenes_1639_v120	0.9483
Listeria_monocytogenes_1639_v38	Listeria_monocytogenes_1639_v120	0.93
Listeria_monocytogenes_1639_v37	Listeria_monocytogenes_1639_v120	0.9255
Listeria_monocytogenes_1639_v36	Listeria_monocytogenes_1639_v120	0.9223
Listeria_monocytogenes_1639_v35	Listeria_monocytogenes_1639_v120	0.9335
Listeria_monocytogenes_1639_v34	Listeria_monocytogenes_1639_v120	0.9275
Listeria_monocytogenes_1639_v33	Listeria_monocytogenes_1639_v120	0.9462
Listeria_monocytogenes_1639_v32	Listeria_monocytogenes_1639_v120	0.9245
Listeria_monocytogenes_1639_v31	Listeria_monocytogenes_1639_v120	0.9278
Listeria_monocytogenes_1639_v30	Listeria_monocytogenes_1639_v120	0.9495
Listeria_monocytogenes_1639_v2	Listeria_monocytogenes_1639_v120	0.9098
Listeria_monocytogenes_1639_v29	Listeria_monocytogenes_1639_v120	0.9193
Listeria_monocytogenes_1639_v28	Listeria_monocytogenes_1639_v120	0.9237
Listeria_monocytogenes_1639_v27	Listeria_monocytogenes_1639_v120	0.9145
Listeria_monocytogenes_1639_v26	Listeria_monocytogenes_1639_v120	0.9142
Listeria_monocytogenes_1639_v25	Listeria_monocytogenes_1639_v120	0.9142
Listeria_monocytogenes_1639_v24	Listeria_monocytogenes_1639_v120	0.9523
Listeria_monocytogenes_1639_v23	Listeria_monocytogenes_1639_v120	0.9519
Listeria_monocytogenes_1639_v22	Listeria_monocytogenes_1639_v120	0.9488
Listeria_monocytogenes_1639_v21	Listeria_monocytogenes_1639_v120	0.9519
Listeria_monocytogenes_1639_v20	Listeria_monocytogenes_1639_v120	0.9398
Listeria_monocytogenes_1639_v1	Listeria_monocytogenes_1639_v120	0.9096
Listeria_monocytogenes_1639_v19	Listeria_monocytogenes_1639_v120	0.9394
Listeria_monocytogenes_1639_v198	Listeria_monocytogenes_1639_v120	0.8999
Listeria_monocytogenes_1639_v197	Listeria_monocytogenes_1639_v120	0.9296
Listeria_monocytogenes_1639_v196	Listeria_monocytogenes_1639_v120	0.9242
Listeria_monocytogenes_1639_v195	Listeria_monocytogenes_1639_v120	0.9473
Listeria_monocytogenes_1639_v194	Listeria_monocytogenes_1639_v120	0.9467
Listeria_monocytogenes_1639_v193	Listeria_monocytogenes_1639_v120	0.9467
Listeria_monocytogenes_1639_v192	Listeria_monocytogenes_1639_v120	0.9126
Listeria_monocytogenes_1639_v191	Listeria_monocytogenes_1639_v120	0.9535
Listeria_monocytogenes_1639_v190	Listeria_monocytogenes_1639_v120	0.9531
Listeria_monocytogenes_1639_v18	Listeria_monocytogenes_1639_v120	0.953
Listeria_monocytogenes_1639_v189	Listeria_monocytogenes_1639_v120	0.9524
Listeria_monocytogenes_1639_v188	Listeria_monocytogenes_1639_v120	0.953
Listeria_monocytogenes_1639_v187	Listeria_monocytogenes_1639_v120	0.9531
Listeria_monocytogenes_1639_v186	Listeria_monocytogenes_1639_v120	0.9535
Listeria_monocytogenes_1639_v185	Listeria_monocytogenes_1639_v120	0.9535
Listeria_monocytogenes_1639_v184	Listeria_monocytogenes_1639_v120	0.9527
Listeria_monocytogenes_1639_v183	Listeria_monocytogenes_1639_v120	0.9484
Listeria_monocytogenes_1639_v182	Listeria_monocytogenes_1639_v120	0.9503
Listeria_monocytogenes_1639_v181	Listeria_monocytogenes_1639_v120	0.9337
Listeria_monocytogenes_1639_v180	Listeria_monocytogenes_1639_v120	0.9337
Listeria_monocytogenes_1639_v17	Listeria_monocytogenes_1639_v120	0.9534
Listeria_monocytogenes_1639_v179	Listeria_monocytogenes_1639_v120	0.9379
Listeria_monocytogenes_1639_v178	Listeria_monocytogenes_1639_v120	0.9547

Listeria_monocytogenes_1639_v177	Listeria_monocytogenes_1639_v120	0.9827
Listeria_monocytogenes_1639_v176	Listeria_monocytogenes_1639_v120	0.944
Listeria_monocytogenes_1639_v175	Listeria_monocytogenes_1639_v120	0.942
Listeria_monocytogenes_1639_v174	Listeria_monocytogenes_1639_v120	0.9536
Listeria_monocytogenes_1639_v173	Listeria_monocytogenes_1639_v120	0.971
Listeria_monocytogenes_1639_v172	Listeria_monocytogenes_1639_v120	0.9426
Listeria_monocytogenes_1639_v171	Listeria_monocytogenes_1639_v120	0.9386
Listeria_monocytogenes_1639_v170	Listeria_monocytogenes_1639_v120	0.9469
Listeria_monocytogenes_1639_v16	Listeria_monocytogenes_1639_v120	0.9124
Listeria_monocytogenes_1639_v169	Listeria_monocytogenes_1639_v120	0.9466
Listeria_monocytogenes_1639_v168	Listeria_monocytogenes_1639_v120	0.9471
Listeria_monocytogenes_1639_v167	Listeria_monocytogenes_1639_v120	0.9396
Listeria_monocytogenes_1639_v166	Listeria_monocytogenes_1639_v120	0.9474
Listeria_monocytogenes_1639_v165	Listeria_monocytogenes_1639_v120	0.9466
Listeria_monocytogenes_1639_v164	Listeria_monocytogenes_1639_v120	0.978
Listeria_monocytogenes_1639_v163	Listeria_monocytogenes_1639_v120	0.968
Listeria_monocytogenes_1639_v162	Listeria_monocytogenes_1639_v120	0.978
Listeria_monocytogenes_1639_v161	Listeria_monocytogenes_1639_v120	0.978
Listeria_monocytogenes_1639_v160	Listeria_monocytogenes_1639_v120	0.978
Listeria_monocytogenes_1639_v15	Listeria_monocytogenes_1639_v120	0.9323
Listeria_monocytogenes_1639_v159	Listeria_monocytogenes_1639_v120	0.9827
Listeria_monocytogenes_1639_v158	Listeria_monocytogenes_1639_v120	0.9788
Listeria_monocytogenes_1639_v157	Listeria_monocytogenes_1639_v120	0.9784
Listeria_monocytogenes_1639_v156	Listeria_monocytogenes_1639_v120	0.9308
Listeria_monocytogenes_1639_v155	Listeria_monocytogenes_1639_v120	0.941
Listeria_monocytogenes_1639_v154	Listeria_monocytogenes_1639_v120	0.9448
Listeria_monocytogenes_1639_v153	Listeria_monocytogenes_1639_v120	0.9417
Listeria_monocytogenes_1639_v152	Listeria_monocytogenes_1639_v120	0.9827
Listeria_monocytogenes_1639_v151	Listeria_monocytogenes_1639_v120	0.9827
Listeria_monocytogenes_1639_v150	Listeria_monocytogenes_1639_v120	0.9433
Listeria_monocytogenes_1639_v14	Listeria_monocytogenes_1639_v120	0.9392
Listeria_monocytogenes_1639_v149	Listeria_monocytogenes_1639_v120	0.9419
Listeria_monocytogenes_1639_v148	Listeria_monocytogenes_1639_v120	0.9816
Listeria_monocytogenes_1639_v147	Listeria_monocytogenes_1639_v120	0.9678
Listeria_monocytogenes_1639_v146	Listeria_monocytogenes_1639_v120	0.9513
Listeria_monocytogenes_1639_v145	Listeria_monocytogenes_1639_v120	0.9468
Listeria_monocytogenes_1639_v144	Listeria_monocytogenes_1639_v120	0.9803
Listeria_monocytogenes_1639_v143	Listeria_monocytogenes_1639_v120	0.9514
Listeria_monocytogenes_1639_v142	Listeria_monocytogenes_1639_v120	0.9807
Listeria_monocytogenes_1639_v141	Listeria_monocytogenes_1639_v120	0.9722
Listeria_monocytogenes_1639_v140	Listeria_monocytogenes_1639_v120	0.9811
Listeria_monocytogenes_1639_v13	Listeria_monocytogenes_1639_v120	0.9319
Listeria_monocytogenes_1639_v139	Listeria_monocytogenes_1639_v120	0.98
Listeria_monocytogenes_1639_v138	Listeria_monocytogenes_1639_v120	0.9834
Listeria_monocytogenes_1639_v137	Listeria_monocytogenes_1639_v120	0.9827
Listeria_monocytogenes_1639_v136	Listeria_monocytogenes_1639_v120	0.9416
Listeria_monocytogenes_1639_v135	Listeria_monocytogenes_1639_v120	0.9213
Listeria_monocytogenes_1639_v134	Listeria_monocytogenes_1639_v120	0.9728
Listeria_monocytogenes_1639_v133	Listeria_monocytogenes_1639_v120	0.9387
Listeria_monocytogenes_1639_v132	Listeria_monocytogenes_1639_v120	0.9218
Listeria_monocytogenes_1639_v131	Listeria_monocytogenes_1639_v120	0.9703
Listeria_monocytogenes_1639_v130	Listeria_monocytogenes_1639_v120	0.9335
Listeria_monocytogenes_1639_v12	Listeria_monocytogenes_1639_v120	0.9132
Listeria_monocytogenes_1639_v129	Listeria_monocytogenes_1639_v120	0.9703
Listeria_monocytogenes_1639_v128	Listeria_monocytogenes_1639_v120	0.9681
Listeria_monocytogenes_1639_v127	Listeria_monocytogenes_1639_v120	0.9417
Listeria_monocytogenes_1639_v126	Listeria_monocytogenes_1639_v120	0.9662
Listeria_monocytogenes_1639_v125	Listeria_monocytogenes_1639_v120	0.9384
Listeria_monocytogenes_1639_v124	Listeria_monocytogenes_1639_v120	0.9399
Listeria_monocytogenes_1639_v123	Listeria_monocytogenes_1639_v120	0.9996
Listeria_monocytogenes_1639_v122	Listeria_monocytogenes_1639_v120	0.9996
Listeria_monocytogenes_1639_v121	Listeria_monocytogenes_1639_v120	0.9996
Listeria_monocytogenes_1639_v120	Listeria_monocytogenes_1639_v120	0.0676
Listeria_welshimeri_1643	Listeria_monocytogenes_1639_v121	0.8835
Listeria_welshimeri_serovar_6b_str_SLCC5334_386043	Listeria_monocytogenes_1639_v121	0.8829
Listeria_seeligeri_serovar_1_2b_str_SLCC3954_683837	Listeria_monocytogenes_1639_v121	0.8908
Listeria_seeligeri_FSL_S4-171_702452	Listeria_monocytogenes_1639_v121	0.8311
Listeria_seeligeri_FSL_N1-067_702453	Listeria_monocytogenes_1639_v121	0.8296
Listeria_seeligeri_1640_v1	Listeria_monocytogenes_1639_v121	0.8988
Listeria_seeligeri_1640	Listeria_monocytogenes_1639_v121	0.8869
Listeria_monocytogenes_WSLC1042_1457188	Listeria_monocytogenes_1639_v121	0.9721
Listeria_monocytogenes_WSLC1001_1457187	Listeria_monocytogenes_1639_v121	0.9368
Listeria_monocytogenes_SLCC7179_879090	Listeria_monocytogenes_1639_v121	0.9424
Listeria_monocytogenes_SLCC5850_882096	Listeria_monocytogenes_1639_v121	0.9411
Listeria_monocytogenes_SLCC2755_932919	Listeria_monocytogenes_1639_v121	0.9526
Listeria_monocytogenes_SLCC2540_879089	Listeria_monocytogenes_1639_v121	0.9516
Listeria_monocytogenes_SLCC2479_882020	Listeria_monocytogenes_1639_v121	0.9339
Listeria_monocytogenes_SLCC2378_879088	Listeria_monocytogenes_1639_v121	0.9732
Listeria_monocytogenes_SLCC2376_882097	Listeria_monocytogenes_1639_v121	0.9462
Listeria_monocytogenes_SLCC2372_932920	Listeria_monocytogenes_1639_v121	0.9265
Listeria_monocytogenes_serotype_4b_str_LL195_1230340	Listeria_monocytogenes_1639_v121	0.9788
Listeria_monocytogenes_serotype_4b_str_LIS0087_1134669	Listeria_monocytogenes_1639_v121	0.9171
Listeria_monocytogenes_serotype_4b_str_F2365_265669	Listeria_monocytogenes_1639_v121	0.9788
Listeria_monocytogenes_serotype_4b_str_CLIP_80459_568819	Listeria_monocytogenes_1639_v121	0.9807
Listeria_monocytogenes_serotype_4b_str_81-0861_1196161	Listeria_monocytogenes_1639_v121	0.9618
Listeria_monocytogenes_serotype_4b_str_81-0592_1196160	Listeria_monocytogenes_1639_v121	0.9629
Listeria_monocytogenes_serotype_4b_str_81-0558_1196159	Listeria_monocytogenes_1639_v121	0.9621
Listeria_monocytogenes_serotype_4b_str_10-0809_1196162	Listeria_monocytogenes_1639_v121	0.9629
Listeria_monocytogenes_serotype_4b_str_02-6680_1196175	Listeria_monocytogenes_1639_v121	0.9769
Listeria_monocytogenes_serotype_4b_str_02-6679_1196174	Listeria_monocytogenes_1639_v121	0.9769
Listeria_monocytogenes_serotype_4b_str_02-1792_1196173	Listeria_monocytogenes_1639_v121	0.9633

Listeria_monocytogenes_serotype_4b_str_02-1289_1196172	Listeria_monocytogenes_1639_v121	0.9633
Listeria_monocytogenes_serotype_4b_str_02-1103_1196171	Listeria_monocytogenes_1639_v121	0.9633
Listeria_monocytogenes_serotype_3c_str_10-5027_1196195	Listeria_monocytogenes_1639_v121	0.9331
Listeria_monocytogenes_serotype_1_2c_str_10-5026_1196194	Listeria_monocytogenes_1639_v121	0.9342
Listeria_monocytogenes_serotype_1_2c_str_10-5025_1196193	Listeria_monocytogenes_1639_v121	0.9342
Listeria_monocytogenes_serotype_1_2b_str_10-0811_1196164	Listeria_monocytogenes_1639_v121	0.9462
Listeria_monocytogenes_serotype_1_2b_str_10-0810_1196163	Listeria_monocytogenes_1639_v121	0.9462
Listeria_monocytogenes_serotype_1_2a_str_99-6370_1196179	Listeria_monocytogenes_1639_v121	0.9206
Listeria_monocytogenes_serotype_1_2a_str_98-2035_1196178	Listeria_monocytogenes_1639_v121	0.9206
Listeria_monocytogenes_serotype_1_2a_str_95-0093_1196177	Listeria_monocytogenes_1639_v121	0.9261
Listeria_monocytogenes_serotype_1_2a_str_88-0478_1196176	Listeria_monocytogenes_1639_v121	0.9252
Listeria_monocytogenes_serotype_1_2a_str_10-5024_1196192	Listeria_monocytogenes_1639_v121	0.9264
Listeria_monocytogenes_serotype_1_2a_str_10-4758_1196168	Listeria_monocytogenes_1639_v121	0.9423
Listeria_monocytogenes_serotype_1_2a_str_10-4754_1196167	Listeria_monocytogenes_1639_v121	0.942
Listeria_monocytogenes_serotype_1_2a_str_10-1321_1196191	Listeria_monocytogenes_1639_v121	0.9264
Listeria_monocytogenes_serotype_1_2a_str_10-1047_1196190	Listeria_monocytogenes_1639_v121	0.9267
Listeria_monocytogenes_serotype_1_2a_str_10-1046_1196189	Listeria_monocytogenes_1639_v121	0.9267
Listeria_monocytogenes_serotype_1_2a_str_10-0934_1196170	Listeria_monocytogenes_1639_v121	0.9532
Listeria_monocytogenes_serotype_1_2a_str_10-0933_1196169	Listeria_monocytogenes_1639_v121	0.9532
Listeria_monocytogenes_serotype_1_2a_str_10-0815_1196188	Listeria_monocytogenes_1639_v121	0.9219
Listeria_monocytogenes_serotype_1_2a_str_10-0814_1196187	Listeria_monocytogenes_1639_v121	0.9264
Listeria_monocytogenes_serotype_1_2a_str_10-0813_1196166	Listeria_monocytogenes_1639_v121	0.934
Listeria_monocytogenes_serotype_1_2a_str_10-0812_1196165	Listeria_monocytogenes_1639_v121	0.934
Listeria_monocytogenes_serotype_1_2a_str_08-7669_1196186	Listeria_monocytogenes_1639_v121	0.9355
Listeria_monocytogenes_serotype_1_2a_str_08-7374_1196185	Listeria_monocytogenes_1639_v121	0.9264
Listeria_monocytogenes_serotype_1_2a_str_08-6997_1196184	Listeria_monocytogenes_1639_v121	0.9219
Listeria_monocytogenes_serotype_1_2a_str_08-6569_1196183	Listeria_monocytogenes_1639_v121	0.9219
Listeria_monocytogenes_serotype_1_2a_str_08-6056_1196182	Listeria_monocytogenes_1639_v121	0.9219
Listeria_monocytogenes_serotype_1_2a_str_04-5457_1196181	Listeria_monocytogenes_1639_v121	0.9264
Listeria_monocytogenes_serotype_1_2a_str_02-5993_1196180	Listeria_monocytogenes_1639_v121	0.9261
Listeria_monocytogenes_R479a_1437838	Listeria_monocytogenes_1639_v121	0.9257
Listeria_monocytogenes_N53-1_1234142	Listeria_monocytogenes_1639_v121	0.8972
Listeria_monocytogenes_M7_1030009	Listeria_monocytogenes_1639_v121	0.9093
Listeria_monocytogenes_LO28_393132	Listeria_monocytogenes_1639_v121	0.9325
Listeria_monocytogenes_L99_563174	Listeria_monocytogenes_1639_v121	0.91
Listeria_monocytogenes_L312_882094	Listeria_monocytogenes_1639_v121	0.9811
Listeria_monocytogenes_J1816_930781	Listeria_monocytogenes_1639_v121	0.9619
Listeria_monocytogenes_J1-220_930782	Listeria_monocytogenes_1639_v121	0.9523
Listeria_monocytogenes_J0161_393130	Listeria_monocytogenes_1639_v121	0.9317
Listeria_monocytogenes_HCC23_552536	Listeria_monocytogenes_1639_v121	0.9093
Listeria_monocytogenes_FSL_R2-561_393126	Listeria_monocytogenes_1639_v121	0.9324
Listeria_monocytogenes_FSL_J1-208_393119	Listeria_monocytogenes_1639_v121	0.9212
Listeria_monocytogenes_FSL_J1-175_393118	Listeria_monocytogenes_1639_v121	0.9614
Listeria_monocytogenes_Finland_1998_393127	Listeria_monocytogenes_1639_v121	0.9536
Listeria_monocytogenes_EGD-e_169963_v1	Listeria_monocytogenes_1639_v121	0.9421
Listeria_monocytogenes_EGD-e_169963	Listeria_monocytogenes_1639_v121	0.9424
Listeria_monocytogenes_EGD_1334565	Listeria_monocytogenes_1639_v121	0.9419
Listeria_monocytogenes_CFSAN002349_1299895	Listeria_monocytogenes_1639_v121	0.9996
Listeria_monocytogenes_ATCC_19117_882095_v1	Listeria_monocytogenes_1639_v121	0.9728
Listeria_monocytogenes_ATCC_19117_882095	Listeria_monocytogenes_1639_v121	0.9727
Listeria_monocytogenes_6179_1288295	Listeria_monocytogenes_1639_v121	0.9155
Listeria_monocytogenes_1639_v9	Listeria_monocytogenes_1639_v121	0.9317
Listeria_monocytogenes_1639_v99	Listeria_monocytogenes_1639_v121	0.9634
Listeria_monocytogenes_1639_v98	Listeria_monocytogenes_1639_v121	0.9289
Listeria_monocytogenes_1639_v97	Listeria_monocytogenes_1639_v121	0.9688
Listeria_monocytogenes_1639_v96	Listeria_monocytogenes_1639_v121	0.9627
Listeria_monocytogenes_1639_v95	Listeria_monocytogenes_1639_v121	0.9627
Listeria_monocytogenes_1639_v94	Listeria_monocytogenes_1639_v121	0.9627
Listeria_monocytogenes_1639_v93	Listeria_monocytogenes_1639_v121	0.9294
Listeria_monocytogenes_1639_v92	Listeria_monocytogenes_1639_v121	0.9631
Listeria_monocytogenes_1639_v91	Listeria_monocytogenes_1639_v121	0.9585
Listeria_monocytogenes_1639_v90	Listeria_monocytogenes_1639_v121	0.9289
Listeria_monocytogenes_1639_v8	Listeria_monocytogenes_1639_v121	0.9154
Listeria_monocytogenes_1639_v89	Listeria_monocytogenes_1639_v121	0.9706
Listeria_monocytogenes_1639_v88	Listeria_monocytogenes_1639_v121	0.9341
Listeria_monocytogenes_1639_v87	Listeria_monocytogenes_1639_v121	0.9298
Listeria_monocytogenes_1639_v86	Listeria_monocytogenes_1639_v121	0.9291
Listeria_monocytogenes_1639_v85	Listeria_monocytogenes_1639_v121	0.9611
Listeria_monocytogenes_1639_v84	Listeria_monocytogenes_1639_v121	0.9369
Listeria_monocytogenes_1639_v83	Listeria_monocytogenes_1639_v121	0.9613
Listeria_monocytogenes_1639_v82	Listeria_monocytogenes_1639_v121	0.9606
Listeria_monocytogenes_1639_v81	Listeria_monocytogenes_1639_v121	0.934
Listeria_monocytogenes_1639_v80	Listeria_monocytogenes_1639_v121	0.9533
Listeria_monocytogenes_1639_v7	Listeria_monocytogenes_1639_v121	0.9154
Listeria_monocytogenes_1639_v79	Listeria_monocytogenes_1639_v121	0.9319
Listeria_monocytogenes_1639_v78	Listeria_monocytogenes_1639_v121	0.9333
Listeria_monocytogenes_1639_v77	Listeria_monocytogenes_1639_v121	0.9331
Listeria_monocytogenes_1639_v76	Listeria_monocytogenes_1639_v121	0.9259
Listeria_monocytogenes_1639_v75	Listeria_monocytogenes_1639_v121	0.91
Listeria_monocytogenes_1639_v74	Listeria_monocytogenes_1639_v121	0.9287
Listeria_monocytogenes_1639_v73	Listeria_monocytogenes_1639_v121	0.9276
Listeria_monocytogenes_1639_v72	Listeria_monocytogenes_1639_v121	0.9601
Listeria_monocytogenes_1639_v71	Listeria_monocytogenes_1639_v121	0.9172
Listeria_monocytogenes_1639_v70	Listeria_monocytogenes_1639_v121	0.9303
Listeria_monocytogenes_1639_v6	Listeria_monocytogenes_1639_v121	0.9148
Listeria_monocytogenes_1639_v69	Listeria_monocytogenes_1639_v121	0.9633
Listeria_monocytogenes_1639_v68	Listeria_monocytogenes_1639_v121	0.9359
Listeria_monocytogenes_1639_v67	Listeria_monocytogenes_1639_v121	0.9515
Listeria_monocytogenes_1639_v66	Listeria_monocytogenes_1639_v121	0.9375
Listeria_monocytogenes_1639_v65	Listeria_monocytogenes_1639_v121	0.9296
Listeria_monocytogenes_1639_v64	Listeria_monocytogenes_1639_v121	0.93

Listeria_monocytogenes_1639_v63	Listeria_monocytogenes_1639_v121	0.9472
Listeria_monocytogenes_1639_v62	Listeria_monocytogenes_1639_v121	0.9298
Listeria_monocytogenes_1639_v61	Listeria_monocytogenes_1639_v121	0.9292
Listeria_monocytogenes_1639_v60	Listeria_monocytogenes_1639_v121	0.9336
Listeria_monocytogenes_1639_v5	Listeria_monocytogenes_1639_v121	0.913
Listeria_monocytogenes_1639_v59	Listeria_monocytogenes_1639_v121	0.962
Listeria_monocytogenes_1639_v58	Listeria_monocytogenes_1639_v121	0.9655
Listeria_monocytogenes_1639_v57	Listeria_monocytogenes_1639_v121	0.9595
Listeria_monocytogenes_1639_v56	Listeria_monocytogenes_1639_v121	0.9617
Listeria_monocytogenes_1639_v55	Listeria_monocytogenes_1639_v121	0.9551
Listeria_monocytogenes_1639_v54	Listeria_monocytogenes_1639_v121	0.9286
Listeria_monocytogenes_1639_v53	Listeria_monocytogenes_1639_v121	0.9333
Listeria_monocytogenes_1639_v52	Listeria_monocytogenes_1639_v121	0.958
Listeria_monocytogenes_1639_v51	Listeria_monocytogenes_1639_v121	0.9559
Listeria_monocytogenes_1639_v50	Listeria_monocytogenes_1639_v121	0.9256
Listeria_monocytogenes_1639_v4	Listeria_monocytogenes_1639_v121	0.9257
Listeria_monocytogenes_1639_v49	Listeria_monocytogenes_1639_v121	0.961
Listeria_monocytogenes_1639_v48	Listeria_monocytogenes_1639_v121	0.9282
Listeria_monocytogenes_1639_v47	Listeria_monocytogenes_1639_v121	0.9261
Listeria_monocytogenes_1639_v46	Listeria_monocytogenes_1639_v121	0.9352
Listeria_monocytogenes_1639_v45	Listeria_monocytogenes_1639_v121	0.9168
Listeria_monocytogenes_1639_v44	Listeria_monocytogenes_1639_v121	0.9264
Listeria_monocytogenes_1639_v43	Listeria_monocytogenes_1639_v121	0.9264
Listeria_monocytogenes_1639_v42	Listeria_monocytogenes_1639_v121	0.9175
Listeria_monocytogenes_1639_v41	Listeria_monocytogenes_1639_v121	0.9074
Listeria_monocytogenes_1639_v40	Listeria_monocytogenes_1639_v121	0.9328
Listeria_monocytogenes_1639_v3	Listeria_monocytogenes_1639_v121	0.9261
Listeria_monocytogenes_1639_v39	Listeria_monocytogenes_1639_v121	0.9483
Listeria_monocytogenes_1639_v38	Listeria_monocytogenes_1639_v121	0.93
Listeria_monocytogenes_1639_v37	Listeria_monocytogenes_1639_v121	0.9259
Listeria_monocytogenes_1639_v36	Listeria_monocytogenes_1639_v121	0.9223
Listeria_monocytogenes_1639_v35	Listeria_monocytogenes_1639_v121	0.9335
Listeria_monocytogenes_1639_v34	Listeria_monocytogenes_1639_v121	0.9278
Listeria_monocytogenes_1639_v33	Listeria_monocytogenes_1639_v121	0.9462
Listeria_monocytogenes_1639_v32	Listeria_monocytogenes_1639_v121	0.9248
Listeria_monocytogenes_1639_v31	Listeria_monocytogenes_1639_v121	0.9282
Listeria_monocytogenes_1639_v30	Listeria_monocytogenes_1639_v121	0.9498
Listeria_monocytogenes_1639_v2	Listeria_monocytogenes_1639_v121	0.9101
Listeria_monocytogenes_1639_v29	Listeria_monocytogenes_1639_v121	0.9193
Listeria_monocytogenes_1639_v28	Listeria_monocytogenes_1639_v121	0.9237
Listeria_monocytogenes_1639_v27	Listeria_monocytogenes_1639_v121	0.9145
Listeria_monocytogenes_1639_v26	Listeria_monocytogenes_1639_v121	0.9145
Listeria_monocytogenes_1639_v25	Listeria_monocytogenes_1639_v121	0.9142
Listeria_monocytogenes_1639_v24	Listeria_monocytogenes_1639_v121	0.9523
Listeria_monocytogenes_1639_v23	Listeria_monocytogenes_1639_v121	0.9523
Listeria_monocytogenes_1639_v22	Listeria_monocytogenes_1639_v121	0.9491
Listeria_monocytogenes_1639_v21	Listeria_monocytogenes_1639_v121	0.9519
Listeria_monocytogenes_1639_v20	Listeria_monocytogenes_1639_v121	0.9398
Listeria_monocytogenes_1639_v1	Listeria_monocytogenes_1639_v121	0.9096
Listeria_monocytogenes_1639_v19	Listeria_monocytogenes_1639_v121	0.9394
Listeria_monocytogenes_1639_v198	Listeria_monocytogenes_1639_v121	0.8999
Listeria_monocytogenes_1639_v197	Listeria_monocytogenes_1639_v121	0.9299
Listeria_monocytogenes_1639_v196	Listeria_monocytogenes_1639_v121	0.9245
Listeria_monocytogenes_1639_v195	Listeria_monocytogenes_1639_v121	0.9473
Listeria_monocytogenes_1639_v194	Listeria_monocytogenes_1639_v121	0.947
Listeria_monocytogenes_1639_v193	Listeria_monocytogenes_1639_v121	0.947
Listeria_monocytogenes_1639_v192	Listeria_monocytogenes_1639_v121	0.9126
Listeria_monocytogenes_1639_v191	Listeria_monocytogenes_1639_v121	0.9534
Listeria_monocytogenes_1639_v190	Listeria_monocytogenes_1639_v121	0.9535
Listeria_monocytogenes_1639_v18	Listeria_monocytogenes_1639_v121	0.9534
Listeria_monocytogenes_1639_v189	Listeria_monocytogenes_1639_v121	0.9527
Listeria_monocytogenes_1639_v188	Listeria_monocytogenes_1639_v121	0.9534
Listeria_monocytogenes_1639_v187	Listeria_monocytogenes_1639_v121	0.9531
Listeria_monocytogenes_1639_v186	Listeria_monocytogenes_1639_v121	0.9539
Listeria_monocytogenes_1639_v185	Listeria_monocytogenes_1639_v121	0.9535
Listeria_monocytogenes_1639_v184	Listeria_monocytogenes_1639_v121	0.9531
Listeria_monocytogenes_1639_v183	Listeria_monocytogenes_1639_v121	0.9481
Listeria_monocytogenes_1639_v182	Listeria_monocytogenes_1639_v121	0.9503
Listeria_monocytogenes_1639_v181	Listeria_monocytogenes_1639_v121	0.9337
Listeria_monocytogenes_1639_v180	Listeria_monocytogenes_1639_v121	0.9337
Listeria_monocytogenes_1639_v17	Listeria_monocytogenes_1639_v121	0.9534
Listeria_monocytogenes_1639_v179	Listeria_monocytogenes_1639_v121	0.9382
Listeria_monocytogenes_1639_v178	Listeria_monocytogenes_1639_v121	0.955
Listeria_monocytogenes_1639_v177	Listeria_monocytogenes_1639_v121	0.9823
Listeria_monocytogenes_1639_v176	Listeria_monocytogenes_1639_v121	0.9444
Listeria_monocytogenes_1639_v175	Listeria_monocytogenes_1639_v121	0.9424
Listeria_monocytogenes_1639_v174	Listeria_monocytogenes_1639_v121	0.9539
Listeria_monocytogenes_1639_v173	Listeria_monocytogenes_1639_v121	0.9714
Listeria_monocytogenes_1639_v172	Listeria_monocytogenes_1639_v121	0.9426
Listeria_monocytogenes_1639_v171	Listeria_monocytogenes_1639_v121	0.9386
Listeria_monocytogenes_1639_v170	Listeria_monocytogenes_1639_v121	0.9469
Listeria_monocytogenes_1639_v16	Listeria_monocytogenes_1639_v121	0.9127
Listeria_monocytogenes_1639_v169	Listeria_monocytogenes_1639_v121	0.9466
Listeria_monocytogenes_1639_v168	Listeria_monocytogenes_1639_v121	0.9474
Listeria_monocytogenes_1639_v167	Listeria_monocytogenes_1639_v121	0.94
Listeria_monocytogenes_1639_v166	Listeria_monocytogenes_1639_v121	0.9478
Listeria_monocytogenes_1639_v165	Listeria_monocytogenes_1639_v121	0.9466
Listeria_monocytogenes_1639_v164	Listeria_monocytogenes_1639_v121	0.978
Listeria_monocytogenes_1639_v163	Listeria_monocytogenes_1639_v121	0.968
Listeria_monocytogenes_1639_v162	Listeria_monocytogenes_1639_v121	0.978
Listeria_monocytogenes_1639_v161	Listeria_monocytogenes_1639_v121	0.978

Listeria_monocytogenes_1639_v160	Listeria_monocytogenes_1639_v121	0.978
Listeria_monocytogenes_1639_v15	Listeria_monocytogenes_1639_v121	0.9323
Listeria_monocytogenes_1639_v159	Listeria_monocytogenes_1639_v121	0.9827
Listeria_monocytogenes_1639_v158	Listeria_monocytogenes_1639_v121	0.9792
Listeria_monocytogenes_1639_v157	Listeria_monocytogenes_1639_v121	0.9788
Listeria_monocytogenes_1639_v156	Listeria_monocytogenes_1639_v121	0.9308
Listeria_monocytogenes_1639_v155	Listeria_monocytogenes_1639_v121	0.9413
Listeria_monocytogenes_1639_v154	Listeria_monocytogenes_1639_v121	0.9452
Listeria_monocytogenes_1639_v153	Listeria_monocytogenes_1639_v121	0.9421
Listeria_monocytogenes_1639_v152	Listeria_monocytogenes_1639_v121	0.9831
Listeria_monocytogenes_1639_v151	Listeria_monocytogenes_1639_v121	0.9831
Listeria_monocytogenes_1639_v150	Listeria_monocytogenes_1639_v121	0.9433
Listeria_monocytogenes_1639_v14	Listeria_monocytogenes_1639_v121	0.9396
Listeria_monocytogenes_1639_v149	Listeria_monocytogenes_1639_v121	0.9419
Listeria_monocytogenes_1639_v148	Listeria_monocytogenes_1639_v121	0.9816
Listeria_monocytogenes_1639_v147	Listeria_monocytogenes_1639_v121	0.9678
Listeria_monocytogenes_1639_v146	Listeria_monocytogenes_1639_v121	0.9517
Listeria_monocytogenes_1639_v145	Listeria_monocytogenes_1639_v121	0.9464
Listeria_monocytogenes_1639_v144	Listeria_monocytogenes_1639_v121	0.9803
Listeria_monocytogenes_1639_v143	Listeria_monocytogenes_1639_v121	0.9518
Listeria_monocytogenes_1639_v142	Listeria_monocytogenes_1639_v121	0.9807
Listeria_monocytogenes_1639_v141	Listeria_monocytogenes_1639_v121	0.9726
Listeria_monocytogenes_1639_v140	Listeria_monocytogenes_1639_v121	0.9811
Listeria_monocytogenes_1639_v13	Listeria_monocytogenes_1639_v121	0.9322
Listeria_monocytogenes_1639_v139	Listeria_monocytogenes_1639_v121	0.98
Listeria_monocytogenes_1639_v138	Listeria_monocytogenes_1639_v121	0.983
Listeria_monocytogenes_1639_v137	Listeria_monocytogenes_1639_v121	0.9827
Listeria_monocytogenes_1639_v136	Listeria_monocytogenes_1639_v121	0.9416
Listeria_monocytogenes_1639_v135	Listeria_monocytogenes_1639_v121	0.9213
Listeria_monocytogenes_1639_v134	Listeria_monocytogenes_1639_v121	0.9731
Listeria_monocytogenes_1639_v133	Listeria_monocytogenes_1639_v121	0.9387
Listeria_monocytogenes_1639_v132	Listeria_monocytogenes_1639_v121	0.9221
Listeria_monocytogenes_1639_v131	Listeria_monocytogenes_1639_v121	0.9707
Listeria_monocytogenes_1639_v130	Listeria_monocytogenes_1639_v121	0.9339
Listeria_monocytogenes_1639_v12	Listeria_monocytogenes_1639_v121	0.9132
Listeria_monocytogenes_1639_v129	Listeria_monocytogenes_1639_v121	0.9703
Listeria_monocytogenes_1639_v128	Listeria_monocytogenes_1639_v121	0.9681
Listeria_monocytogenes_1639_v127	Listeria_monocytogenes_1639_v121	0.9417
Listeria_monocytogenes_1639_v126	Listeria_monocytogenes_1639_v121	0.9666
Listeria_monocytogenes_1639_v125	Listeria_monocytogenes_1639_v121	0.9387
Listeria_monocytogenes_1639_v124	Listeria_monocytogenes_1639_v121	0.9403
Listeria_monocytogenes_1639_v123	Listeria_monocytogenes_1639_v121	0.9996
Listeria_monocytogenes_1639_v122	Listeria_monocytogenes_1639_v121	1
Listeria_monocytogenes_1639_v121	Listeria_monocytogenes_1639_v121	0.0673
Listeria_welshimeri_1643	Listeria_monocytogenes_1639_v122	0.8835
Listeria_welshimeri_serovar_6b_str_SLCC5334_386043	Listeria_monocytogenes_1639_v122	0.8829
Listeria_seeligeri_serovar_1_2b_str_SLCC3954_683837	Listeria_monocytogenes_1639_v122	0.8908
Listeria_seeligeri_FSL_S4-171_702452	Listeria_monocytogenes_1639_v122	0.8311
Listeria_seeligeri_FSL_N1-067_702453	Listeria_monocytogenes_1639_v122	0.8296
Listeria_seeligeri_1640_v1	Listeria_monocytogenes_1639_v122	0.8988
Listeria_seeligeri_1640	Listeria_monocytogenes_1639_v122	0.8869
Listeria_monocytogenes_WSLC1042_1457188	Listeria_monocytogenes_1639_v122	0.9721
Listeria_monocytogenes_WSLC1001_1457187	Listeria_monocytogenes_1639_v122	0.9368
Listeria_monocytogenes_SLCC7179_879090	Listeria_monocytogenes_1639_v122	0.9424
Listeria_monocytogenes_SLCC5850_882096	Listeria_monocytogenes_1639_v122	0.9411
Listeria_monocytogenes_SLCC2755_932919	Listeria_monocytogenes_1639_v122	0.9526
Listeria_monocytogenes_SLCC2540_879089	Listeria_monocytogenes_1639_v122	0.9516
Listeria_monocytogenes_SLCC2479_882020	Listeria_monocytogenes_1639_v122	0.9339
Listeria_monocytogenes_SLCC2378_879088	Listeria_monocytogenes_1639_v122	0.9732
Listeria_monocytogenes_SLCC2376_882097	Listeria_monocytogenes_1639_v122	0.9462
Listeria_monocytogenes_SLCC2372_932920	Listeria_monocytogenes_1639_v122	0.9265
Listeria_monocytogenes_serotype_4b_str_LL195_1230340	Listeria_monocytogenes_1639_v122	0.9788
Listeria_monocytogenes_serotype_4b_str_LIS0087_1134669	Listeria_monocytogenes_1639_v122	0.9171
Listeria_monocytogenes_serotype_4b_str_F2365_265669	Listeria_monocytogenes_1639_v122	0.9788
Listeria_monocytogenes_serotype_4b_str_CLIP_80459_568819	Listeria_monocytogenes_1639_v122	0.9807
Listeria_monocytogenes_serotype_4b_str_81-0861_1196161	Listeria_monocytogenes_1639_v122	0.9618
Listeria_monocytogenes_serotype_4b_str_81-0592_1196160	Listeria_monocytogenes_1639_v122	0.9629
Listeria_monocytogenes_serotype_4b_str_81-0558_1196159	Listeria_monocytogenes_1639_v122	0.9621
Listeria_monocytogenes_serotype_4b_str_10-0809_1196162	Listeria_monocytogenes_1639_v122	0.9629
Listeria_monocytogenes_serotype_4b_str_02-6680_1196175	Listeria_monocytogenes_1639_v122	0.9769
Listeria_monocytogenes_serotype_4b_str_02-6679_1196174	Listeria_monocytogenes_1639_v122	0.9769
Listeria_monocytogenes_serotype_4b_str_02-1792_1196173	Listeria_monocytogenes_1639_v122	0.9633
Listeria_monocytogenes_serotype_4b_str_02-1289_1196172	Listeria_monocytogenes_1639_v122	0.9633
Listeria_monocytogenes_serotype_4b_str_02-1103_1196171	Listeria_monocytogenes_1639_v122	0.9633
Listeria_monocytogenes_serotype_3c_str_10-5027_1196195	Listeria_monocytogenes_1639_v122	0.9331
Listeria_monocytogenes_serotype_1_2c_str_10-5026_1196194	Listeria_monocytogenes_1639_v122	0.9342
Listeria_monocytogenes_serotype_1_2c_str_10-5025_1196193	Listeria_monocytogenes_1639_v122	0.9342
Listeria_monocytogenes_serotype_1_2b_str_10-0811_1196164	Listeria_monocytogenes_1639_v122	0.9462
Listeria_monocytogenes_serotype_1_2b_str_10-0810_1196163	Listeria_monocytogenes_1639_v122	0.9462
Listeria_monocytogenes_serotype_1_2a_str_99-6370_1196179	Listeria_monocytogenes_1639_v122	0.9206
Listeria_monocytogenes_serotype_1_2a_str_98-2035_1196178	Listeria_monocytogenes_1639_v122	0.9206
Listeria_monocytogenes_serotype_1_2a_str_95-0093_1196177	Listeria_monocytogenes_1639_v122	0.9261
Listeria_monocytogenes_serotype_1_2a_str_88-0478_1196176	Listeria_monocytogenes_1639_v122	0.9252
Listeria_monocytogenes_serotype_1_2a_str_10-5024_1196192	Listeria_monocytogenes_1639_v122	0.9264
Listeria_monocytogenes_serotype_1_2a_str_10-4758_1196168	Listeria_monocytogenes_1639_v122	0.9423
Listeria_monocytogenes_serotype_1_2a_str_10-4754_1196167	Listeria_monocytogenes_1639_v122	0.942
Listeria_monocytogenes_serotype_1_2a_str_10-1321_1196191	Listeria_monocytogenes_1639_v122	0.9264
Listeria_monocytogenes_serotype_1_2a_str_10-1047_1196190	Listeria_monocytogenes_1639_v122	0.9267
Listeria_monocytogenes_serotype_1_2a_str_10-1046_1196189	Listeria_monocytogenes_1639_v122	0.9267
Listeria_monocytogenes_serotype_1_2a_str_10-0934_1196170	Listeria_monocytogenes_1639_v122	0.9532
Listeria_monocytogenes_serotype_1_2a_str_10-0933_1196169	Listeria_monocytogenes_1639_v122	0.9532

Listeria_monocytogenes_serotype_1_2a_str_10-0815_1196188	Listeria_monocytogenes_1639_v122	0.9219
Listeria_monocytogenes_serotype_1_2a_str_10-0814_1196187	Listeria_monocytogenes_1639_v122	0.9264
Listeria_monocytogenes_serotype_1_2a_str_10-0813_1196166	Listeria_monocytogenes_1639_v122	0.934
Listeria_monocytogenes_serotype_1_2a_str_10-0812_1196165	Listeria_monocytogenes_1639_v122	0.934
Listeria_monocytogenes_serotype_1_2a_str_08-7669_1196186	Listeria_monocytogenes_1639_v122	0.9355
Listeria_monocytogenes_serotype_1_2a_str_08-7374_1196185	Listeria_monocytogenes_1639_v122	0.9264
Listeria_monocytogenes_serotype_1_2a_str_08-6997_1196184	Listeria_monocytogenes_1639_v122	0.9219
Listeria_monocytogenes_serotype_1_2a_str_08-6569_1196183	Listeria_monocytogenes_1639_v122	0.9219
Listeria_monocytogenes_serotype_1_2a_str_08-6056_1196182	Listeria_monocytogenes_1639_v122	0.9219
Listeria_monocytogenes_serotype_1_2a_str_04-5457_1196181	Listeria_monocytogenes_1639_v122	0.9264
Listeria_monocytogenes_serotype_1_2a_str_02-5993_1196180	Listeria_monocytogenes_1639_v122	0.9261
Listeria_monocytogenes_R479a_1437838	Listeria_monocytogenes_1639_v122	0.9257
Listeria_monocytogenes_N53-1_1234142	Listeria_monocytogenes_1639_v122	0.8972
Listeria_monocytogenes_M7_1030009	Listeria_monocytogenes_1639_v122	0.9093
Listeria_monocytogenes_LO28_393132	Listeria_monocytogenes_1639_v122	0.9325
Listeria_monocytogenes_L99_563174	Listeria_monocytogenes_1639_v122	0.91
Listeria_monocytogenes_L312_882094	Listeria_monocytogenes_1639_v122	0.9811
Listeria_monocytogenes_J1816_930781	Listeria_monocytogenes_1639_v122	0.9619
Listeria_monocytogenes_J1-220_930782	Listeria_monocytogenes_1639_v122	0.9523
Listeria_monocytogenes_J0161_393130	Listeria_monocytogenes_1639_v122	0.9317
Listeria_monocytogenes_HCC23_552536	Listeria_monocytogenes_1639_v122	0.9093
Listeria_monocytogenes_FSL_R2-561_393126	Listeria_monocytogenes_1639_v122	0.9324
Listeria_monocytogenes_FSL_J1-208_393119	Listeria_monocytogenes_1639_v122	0.9212
Listeria_monocytogenes_FSL_J1-175_393118	Listeria_monocytogenes_1639_v122	0.9614
Listeria_monocytogenes_Finland_1998_393127	Listeria_monocytogenes_1639_v122	0.9536
Listeria_monocytogenes_EGD-e_169963_v1	Listeria_monocytogenes_1639_v122	0.9421
Listeria_monocytogenes_EGD-e_169963	Listeria_monocytogenes_1639_v122	0.9424
Listeria_monocytogenes_EGD_1334565	Listeria_monocytogenes_1639_v122	0.9419
Listeria_monocytogenes_CFSAN002349_1299895	Listeria_monocytogenes_1639_v122	0.9996
Listeria_monocytogenes_ATCC_19117_882095_v1	Listeria_monocytogenes_1639_v122	0.9728
Listeria_monocytogenes_ATCC_19117_882095	Listeria_monocytogenes_1639_v122	0.9727
Listeria_monocytogenes_6179_1288295	Listeria_monocytogenes_1639_v122	0.9155
Listeria_monocytogenes_1639_v9	Listeria_monocytogenes_1639_v122	0.9317
Listeria_monocytogenes_1639_v99	Listeria_monocytogenes_1639_v122	0.9634
Listeria_monocytogenes_1639_v98	Listeria_monocytogenes_1639_v122	0.9289
Listeria_monocytogenes_1639_v97	Listeria_monocytogenes_1639_v122	0.9688
Listeria_monocytogenes_1639_v96	Listeria_monocytogenes_1639_v122	0.9627
Listeria_monocytogenes_1639_v95	Listeria_monocytogenes_1639_v122	0.9627
Listeria_monocytogenes_1639_v94	Listeria_monocytogenes_1639_v122	0.9627
Listeria_monocytogenes_1639_v93	Listeria_monocytogenes_1639_v122	0.9294
Listeria_monocytogenes_1639_v92	Listeria_monocytogenes_1639_v122	0.9631
Listeria_monocytogenes_1639_v91	Listeria_monocytogenes_1639_v122	0.9585
Listeria_monocytogenes_1639_v90	Listeria_monocytogenes_1639_v122	0.9289
Listeria_monocytogenes_1639_v8	Listeria_monocytogenes_1639_v122	0.9154
Listeria_monocytogenes_1639_v89	Listeria_monocytogenes_1639_v122	0.9706
Listeria_monocytogenes_1639_v88	Listeria_monocytogenes_1639_v122	0.9341
Listeria_monocytogenes_1639_v87	Listeria_monocytogenes_1639_v122	0.9298
Listeria_monocytogenes_1639_v86	Listeria_monocytogenes_1639_v122	0.9291
Listeria_monocytogenes_1639_v85	Listeria_monocytogenes_1639_v122	0.9611
Listeria_monocytogenes_1639_v84	Listeria_monocytogenes_1639_v122	0.9369
Listeria_monocytogenes_1639_v83	Listeria_monocytogenes_1639_v122	0.9613
Listeria_monocytogenes_1639_v82	Listeria_monocytogenes_1639_v122	0.9606
Listeria_monocytogenes_1639_v81	Listeria_monocytogenes_1639_v122	0.934
Listeria_monocytogenes_1639_v80	Listeria_monocytogenes_1639_v122	0.9533
Listeria_monocytogenes_1639_v7	Listeria_monocytogenes_1639_v122	0.9154
Listeria_monocytogenes_1639_v79	Listeria_monocytogenes_1639_v122	0.9319
Listeria_monocytogenes_1639_v78	Listeria_monocytogenes_1639_v122	0.9333
Listeria_monocytogenes_1639_v77	Listeria_monocytogenes_1639_v122	0.9331
Listeria_monocytogenes_1639_v76	Listeria_monocytogenes_1639_v122	0.9259
Listeria_monocytogenes_1639_v75	Listeria_monocytogenes_1639_v122	0.91
Listeria_monocytogenes_1639_v74	Listeria_monocytogenes_1639_v122	0.9287
Listeria_monocytogenes_1639_v73	Listeria_monocytogenes_1639_v122	0.9276
Listeria_monocytogenes_1639_v72	Listeria_monocytogenes_1639_v122	0.9601
Listeria_monocytogenes_1639_v71	Listeria_monocytogenes_1639_v122	0.9172
Listeria_monocytogenes_1639_v70	Listeria_monocytogenes_1639_v122	0.9303
Listeria_monocytogenes_1639_v6	Listeria_monocytogenes_1639_v122	0.9148
Listeria_monocytogenes_1639_v69	Listeria_monocytogenes_1639_v122	0.9633
Listeria_monocytogenes_1639_v68	Listeria_monocytogenes_1639_v122	0.9359
Listeria_monocytogenes_1639_v67	Listeria_monocytogenes_1639_v122	0.9515
Listeria_monocytogenes_1639_v66	Listeria_monocytogenes_1639_v122	0.9375
Listeria_monocytogenes_1639_v65	Listeria_monocytogenes_1639_v122	0.9296
Listeria_monocytogenes_1639_v64	Listeria_monocytogenes_1639_v122	0.93
Listeria_monocytogenes_1639_v63	Listeria_monocytogenes_1639_v122	0.9472
Listeria_monocytogenes_1639_v62	Listeria_monocytogenes_1639_v122	0.9298
Listeria_monocytogenes_1639_v61	Listeria_monocytogenes_1639_v122	0.9292
Listeria_monocytogenes_1639_v60	Listeria_monocytogenes_1639_v122	0.9336
Listeria_monocytogenes_1639_v5	Listeria_monocytogenes_1639_v122	0.913
Listeria_monocytogenes_1639_v59	Listeria_monocytogenes_1639_v122	0.962
Listeria_monocytogenes_1639_v58	Listeria_monocytogenes_1639_v122	0.9655
Listeria_monocytogenes_1639_v57	Listeria_monocytogenes_1639_v122	0.9595
Listeria_monocytogenes_1639_v56	Listeria_monocytogenes_1639_v122	0.9617
Listeria_monocytogenes_1639_v55	Listeria_monocytogenes_1639_v122	0.9551
Listeria_monocytogenes_1639_v54	Listeria_monocytogenes_1639_v122	0.9286
Listeria_monocytogenes_1639_v53	Listeria_monocytogenes_1639_v122	0.9333
Listeria_monocytogenes_1639_v52	Listeria_monocytogenes_1639_v122	0.958
Listeria_monocytogenes_1639_v51	Listeria_monocytogenes_1639_v122	0.9559
Listeria_monocytogenes_1639_v50	Listeria_monocytogenes_1639_v122	0.9256
Listeria_monocytogenes_1639_v4	Listeria_monocytogenes_1639_v122	0.9257
Listeria_monocytogenes_1639_v49	Listeria_monocytogenes_1639_v122	0.961
Listeria_monocytogenes_1639_v48	Listeria_monocytogenes_1639_v122	0.9282
Listeria_monocytogenes_1639_v47	Listeria_monocytogenes_1639_v122	0.9261

Listeria_monocytogenes_1639_v46	Listeria_monocytogenes_1639_v122	0.9352
Listeria_monocytogenes_1639_v45	Listeria_monocytogenes_1639_v122	0.9168
Listeria_monocytogenes_1639_v44	Listeria_monocytogenes_1639_v122	0.9264
Listeria_monocytogenes_1639_v43	Listeria_monocytogenes_1639_v122	0.9264
Listeria_monocytogenes_1639_v42	Listeria_monocytogenes_1639_v122	0.9175
Listeria_monocytogenes_1639_v41	Listeria_monocytogenes_1639_v122	0.9074
Listeria_monocytogenes_1639_v40	Listeria_monocytogenes_1639_v122	0.9328
Listeria_monocytogenes_1639_v3	Listeria_monocytogenes_1639_v122	0.9261
Listeria_monocytogenes_1639_v39	Listeria_monocytogenes_1639_v122	0.9483
Listeria_monocytogenes_1639_v38	Listeria_monocytogenes_1639_v122	0.93
Listeria_monocytogenes_1639_v37	Listeria_monocytogenes_1639_v122	0.9259
Listeria_monocytogenes_1639_v36	Listeria_monocytogenes_1639_v122	0.9223
Listeria_monocytogenes_1639_v35	Listeria_monocytogenes_1639_v122	0.9335
Listeria_monocytogenes_1639_v34	Listeria_monocytogenes_1639_v122	0.9278
Listeria_monocytogenes_1639_v33	Listeria_monocytogenes_1639_v122	0.9462
Listeria_monocytogenes_1639_v32	Listeria_monocytogenes_1639_v122	0.9248
Listeria_monocytogenes_1639_v31	Listeria_monocytogenes_1639_v122	0.9282
Listeria_monocytogenes_1639_v30	Listeria_monocytogenes_1639_v122	0.9498
Listeria_monocytogenes_1639_v2	Listeria_monocytogenes_1639_v122	0.9101
Listeria_monocytogenes_1639_v29	Listeria_monocytogenes_1639_v122	0.9193
Listeria_monocytogenes_1639_v28	Listeria_monocytogenes_1639_v122	0.9237
Listeria_monocytogenes_1639_v27	Listeria_monocytogenes_1639_v122	0.9145
Listeria_monocytogenes_1639_v26	Listeria_monocytogenes_1639_v122	0.9145
Listeria_monocytogenes_1639_v25	Listeria_monocytogenes_1639_v122	0.9142
Listeria_monocytogenes_1639_v24	Listeria_monocytogenes_1639_v122	0.9523
Listeria_monocytogenes_1639_v23	Listeria_monocytogenes_1639_v122	0.9523
Listeria_monocytogenes_1639_v22	Listeria_monocytogenes_1639_v122	0.9491
Listeria_monocytogenes_1639_v21	Listeria_monocytogenes_1639_v122	0.9519
Listeria_monocytogenes_1639_v20	Listeria_monocytogenes_1639_v122	0.9398
Listeria_monocytogenes_1639_v1	Listeria_monocytogenes_1639_v122	0.9096
Listeria_monocytogenes_1639_v19	Listeria_monocytogenes_1639_v122	0.9394
Listeria_monocytogenes_1639_v198	Listeria_monocytogenes_1639_v122	0.8999
Listeria_monocytogenes_1639_v197	Listeria_monocytogenes_1639_v122	0.9299
Listeria_monocytogenes_1639_v196	Listeria_monocytogenes_1639_v122	0.9245
Listeria_monocytogenes_1639_v195	Listeria_monocytogenes_1639_v122	0.9473
Listeria_monocytogenes_1639_v194	Listeria_monocytogenes_1639_v122	0.947
Listeria_monocytogenes_1639_v193	Listeria_monocytogenes_1639_v122	0.947
Listeria_monocytogenes_1639_v192	Listeria_monocytogenes_1639_v122	0.9126
Listeria_monocytogenes_1639_v191	Listeria_monocytogenes_1639_v122	0.9534
Listeria_monocytogenes_1639_v190	Listeria_monocytogenes_1639_v122	0.9535
Listeria_monocytogenes_1639_v18	Listeria_monocytogenes_1639_v122	0.9534
Listeria_monocytogenes_1639_v189	Listeria_monocytogenes_1639_v122	0.9527
Listeria_monocytogenes_1639_v188	Listeria_monocytogenes_1639_v122	0.9534
Listeria_monocytogenes_1639_v187	Listeria_monocytogenes_1639_v122	0.9531
Listeria_monocytogenes_1639_v186	Listeria_monocytogenes_1639_v122	0.9539
Listeria_monocytogenes_1639_v185	Listeria_monocytogenes_1639_v122	0.9535
Listeria_monocytogenes_1639_v184	Listeria_monocytogenes_1639_v122	0.9531
Listeria_monocytogenes_1639_v183	Listeria_monocytogenes_1639_v122	0.9481
Listeria_monocytogenes_1639_v182	Listeria_monocytogenes_1639_v122	0.9503
Listeria_monocytogenes_1639_v181	Listeria_monocytogenes_1639_v122	0.9337
Listeria_monocytogenes_1639_v180	Listeria_monocytogenes_1639_v122	0.9337
Listeria_monocytogenes_1639_v17	Listeria_monocytogenes_1639_v122	0.9534
Listeria_monocytogenes_1639_v179	Listeria_monocytogenes_1639_v122	0.9382
Listeria_monocytogenes_1639_v178	Listeria_monocytogenes_1639_v122	0.955
Listeria_monocytogenes_1639_v177	Listeria_monocytogenes_1639_v122	0.9823
Listeria_monocytogenes_1639_v176	Listeria_monocytogenes_1639_v122	0.9444
Listeria_monocytogenes_1639_v175	Listeria_monocytogenes_1639_v122	0.9424
Listeria_monocytogenes_1639_v174	Listeria_monocytogenes_1639_v122	0.9539
Listeria_monocytogenes_1639_v173	Listeria_monocytogenes_1639_v122	0.9714
Listeria_monocytogenes_1639_v172	Listeria_monocytogenes_1639_v122	0.9426
Listeria_monocytogenes_1639_v171	Listeria_monocytogenes_1639_v122	0.9386
Listeria_monocytogenes_1639_v170	Listeria_monocytogenes_1639_v122	0.9469
Listeria_monocytogenes_1639_v16	Listeria_monocytogenes_1639_v122	0.9127
Listeria_monocytogenes_1639_v169	Listeria_monocytogenes_1639_v122	0.9466
Listeria_monocytogenes_1639_v168	Listeria_monocytogenes_1639_v122	0.9474
Listeria_monocytogenes_1639_v167	Listeria_monocytogenes_1639_v122	0.94
Listeria_monocytogenes_1639_v166	Listeria_monocytogenes_1639_v122	0.9478
Listeria_monocytogenes_1639_v165	Listeria_monocytogenes_1639_v122	0.9466
Listeria_monocytogenes_1639_v164	Listeria_monocytogenes_1639_v122	0.978
Listeria_monocytogenes_1639_v163	Listeria_monocytogenes_1639_v122	0.968
Listeria_monocytogenes_1639_v162	Listeria_monocytogenes_1639_v122	0.978
Listeria_monocytogenes_1639_v161	Listeria_monocytogenes_1639_v122	0.978
Listeria_monocytogenes_1639_v160	Listeria_monocytogenes_1639_v122	0.978
Listeria_monocytogenes_1639_v15	Listeria_monocytogenes_1639_v122	0.9323
Listeria_monocytogenes_1639_v159	Listeria_monocytogenes_1639_v122	0.9827
Listeria_monocytogenes_1639_v158	Listeria_monocytogenes_1639_v122	0.9792
Listeria_monocytogenes_1639_v157	Listeria_monocytogenes_1639_v122	0.9788
Listeria_monocytogenes_1639_v156	Listeria_monocytogenes_1639_v122	0.9308
Listeria_monocytogenes_1639_v155	Listeria_monocytogenes_1639_v122	0.9413
Listeria_monocytogenes_1639_v154	Listeria_monocytogenes_1639_v122	0.9452
Listeria_monocytogenes_1639_v153	Listeria_monocytogenes_1639_v122	0.9421
Listeria_monocytogenes_1639_v152	Listeria_monocytogenes_1639_v122	0.9831
Listeria_monocytogenes_1639_v151	Listeria_monocytogenes_1639_v122	0.9831
Listeria_monocytogenes_1639_v150	Listeria_monocytogenes_1639_v122	0.9433
Listeria_monocytogenes_1639_v14	Listeria_monocytogenes_1639_v122	0.9396
Listeria_monocytogenes_1639_v149	Listeria_monocytogenes_1639_v122	0.9419
Listeria_monocytogenes_1639_v148	Listeria_monocytogenes_1639_v122	0.9816
Listeria_monocytogenes_1639_v147	Listeria_monocytogenes_1639_v122	0.9678
Listeria_monocytogenes_1639_v146	Listeria_monocytogenes_1639_v122	0.9517
Listeria_monocytogenes_1639_v145	Listeria_monocytogenes_1639_v122	0.9464
Listeria_monocytogenes_1639_v144	Listeria_monocytogenes_1639_v122	0.9803

Listeria_monocytogenes_1639_v143	Listeria_monocytogenes_1639_v122	0.9518
Listeria_monocytogenes_1639_v142	Listeria_monocytogenes_1639_v122	0.9807
Listeria_monocytogenes_1639_v141	Listeria_monocytogenes_1639_v122	0.9726
Listeria_monocytogenes_1639_v140	Listeria_monocytogenes_1639_v122	0.9811
Listeria_monocytogenes_1639_v13	Listeria_monocytogenes_1639_v122	0.9322
Listeria_monocytogenes_1639_v139	Listeria_monocytogenes_1639_v122	0.98
Listeria_monocytogenes_1639_v138	Listeria_monocytogenes_1639_v122	0.983
Listeria_monocytogenes_1639_v137	Listeria_monocytogenes_1639_v122	0.9827
Listeria_monocytogenes_1639_v136	Listeria_monocytogenes_1639_v122	0.9416
Listeria_monocytogenes_1639_v135	Listeria_monocytogenes_1639_v122	0.9213
Listeria_monocytogenes_1639_v134	Listeria_monocytogenes_1639_v122	0.9731
Listeria_monocytogenes_1639_v133	Listeria_monocytogenes_1639_v122	0.9387
Listeria_monocytogenes_1639_v132	Listeria_monocytogenes_1639_v122	0.9221
Listeria_monocytogenes_1639_v131	Listeria_monocytogenes_1639_v122	0.9707
Listeria_monocytogenes_1639_v130	Listeria_monocytogenes_1639_v122	0.9339
Listeria_monocytogenes_1639_v12	Listeria_monocytogenes_1639_v122	0.9132
Listeria_monocytogenes_1639_v129	Listeria_monocytogenes_1639_v122	0.9703
Listeria_monocytogenes_1639_v128	Listeria_monocytogenes_1639_v122	0.9681
Listeria_monocytogenes_1639_v127	Listeria_monocytogenes_1639_v122	0.9417
Listeria_monocytogenes_1639_v126	Listeria_monocytogenes_1639_v122	0.9666
Listeria_monocytogenes_1639_v125	Listeria_monocytogenes_1639_v122	0.9387
Listeria_monocytogenes_1639_v124	Listeria_monocytogenes_1639_v122	0.9403
Listeria_monocytogenes_1639_v123	Listeria_monocytogenes_1639_v122	0.9996
Listeria_monocytogenes_1639_v122	Listeria_monocytogenes_1639_v122	0.0673
Listeria_welshimeri_1643	Listeria_monocytogenes_1639_v123	0.8832
Listeria_welshimeri_serovar_6b_str_SLCC5334_386043	Listeria_monocytogenes_1639_v123	0.8829
Listeria_seeligeri_serovar_1_2b_str_SLCC3954_683837	Listeria_monocytogenes_1639_v123	0.8908
Listeria_seeligeri_FSL_S4-171_702452	Listeria_monocytogenes_1639_v123	0.8308
Listeria_seeligeri_FSL_N1-067_702453	Listeria_monocytogenes_1639_v123	0.8296
Listeria_seeligeri_1640_v1	Listeria_monocytogenes_1639_v123	0.8992
Listeria_seeligeri_1640	Listeria_monocytogenes_1639_v123	0.8869
Listeria_monocytogenes_WSLC1042_1457188	Listeria_monocytogenes_1639_v123	0.9721
Listeria_monocytogenes_WSLC1001_1457187	Listeria_monocytogenes_1639_v123	0.9368
Listeria_monocytogenes_SLCC7179_879090	Listeria_monocytogenes_1639_v123	0.9424
Listeria_monocytogenes_SLCC5850_882096	Listeria_monocytogenes_1639_v123	0.9411
Listeria_monocytogenes_SLCC2755_932919	Listeria_monocytogenes_1639_v123	0.9522
Listeria_monocytogenes_SLCC2540_879089	Listeria_monocytogenes_1639_v123	0.9513
Listeria_monocytogenes_SLCC2479_882020	Listeria_monocytogenes_1639_v123	0.9342
Listeria_monocytogenes_SLCC2378_879088	Listeria_monocytogenes_1639_v123	0.9728
Listeria_monocytogenes_SLCC2376_882097	Listeria_monocytogenes_1639_v123	0.9462
Listeria_monocytogenes_SLCC2372_932920	Listeria_monocytogenes_1639_v123	0.9269
Listeria_monocytogenes_serotype_4b_str_LL195_1230340	Listeria_monocytogenes_1639_v123	0.9784
Listeria_monocytogenes_serotype_4b_str_LIS0087_1134669	Listeria_monocytogenes_1639_v123	0.9174
Listeria_monocytogenes_serotype_4b_str_F2365_265669	Listeria_monocytogenes_1639_v123	0.9784
Listeria_monocytogenes_serotype_4b_str_CLIP_80459_568819	Listeria_monocytogenes_1639_v123	0.9803
Listeria_monocytogenes_serotype_4b_str_81-0861_1196161	Listeria_monocytogenes_1639_v123	0.9618
Listeria_monocytogenes_serotype_4b_str_81-0592_1196160	Listeria_monocytogenes_1639_v123	0.9629
Listeria_monocytogenes_serotype_4b_str_81-0558_1196159	Listeria_monocytogenes_1639_v123	0.9617
Listeria_monocytogenes_serotype_4b_str_10-0809_1196162	Listeria_monocytogenes_1639_v123	0.9629
Listeria_monocytogenes_serotype_4b_str_02-6680_1196175	Listeria_monocytogenes_1639_v123	0.9769
Listeria_monocytogenes_serotype_4b_str_02-6679_1196174	Listeria_monocytogenes_1639_v123	0.9769
Listeria_monocytogenes_serotype_4b_str_02-1792_1196173	Listeria_monocytogenes_1639_v123	0.9629
Listeria_monocytogenes_serotype_4b_str_02-1289_1196172	Listeria_monocytogenes_1639_v123	0.9629
Listeria_monocytogenes_serotype_4b_str_02-1103_1196171	Listeria_monocytogenes_1639_v123	0.9629
Listeria_monocytogenes_serotype_3c_str_10-5027_1196195	Listeria_monocytogenes_1639_v123	0.9335
Listeria_monocytogenes_serotype_1_2c_str_10-5026_1196194	Listeria_monocytogenes_1639_v123	0.9342
Listeria_monocytogenes_serotype_1_2c_str_10-5025_1196193	Listeria_monocytogenes_1639_v123	0.9342
Listeria_monocytogenes_serotype_1_2b_str_10-0811_1196164	Listeria_monocytogenes_1639_v123	0.9462
Listeria_monocytogenes_serotype_1_2b_str_10-0810_1196163	Listeria_monocytogenes_1639_v123	0.9462
Listeria_monocytogenes_serotype_1_2a_str_99-6370_1196179	Listeria_monocytogenes_1639_v123	0.9209
Listeria_monocytogenes_serotype_1_2a_str_98-2035_1196178	Listeria_monocytogenes_1639_v123	0.9209
Listeria_monocytogenes_serotype_1_2a_str_95-0093_1196177	Listeria_monocytogenes_1639_v123	0.9264
Listeria_monocytogenes_serotype_1_2a_str_88-0478_1196176	Listeria_monocytogenes_1639_v123	0.9253
Listeria_monocytogenes_serotype_1_2a_str_10-5024_1196192	Listeria_monocytogenes_1639_v123	0.9264
Listeria_monocytogenes_serotype_1_2a_str_10-4758_1196168	Listeria_monocytogenes_1639_v123	0.9424
Listeria_monocytogenes_serotype_1_2a_str_10-4754_1196167	Listeria_monocytogenes_1639_v123	0.9423
Listeria_monocytogenes_serotype_1_2a_str_10-1321_1196191	Listeria_monocytogenes_1639_v123	0.9264
Listeria_monocytogenes_serotype_1_2a_str_10-1047_1196190	Listeria_monocytogenes_1639_v123	0.9271
Listeria_monocytogenes_serotype_1_2a_str_10-1046_1196189	Listeria_monocytogenes_1639_v123	0.9271
Listeria_monocytogenes_serotype_1_2a_str_10-0934_1196170	Listeria_monocytogenes_1639_v123	0.9536
Listeria_monocytogenes_serotype_1_2a_str_10-0933_1196169	Listeria_monocytogenes_1639_v123	0.9536
Listeria_monocytogenes_serotype_1_2a_str_10-0815_1196188	Listeria_monocytogenes_1639_v123	0.9223
Listeria_monocytogenes_serotype_1_2a_str_10-0814_1196187	Listeria_monocytogenes_1639_v123	0.9268
Listeria_monocytogenes_serotype_1_2a_str_10-0813_1196166	Listeria_monocytogenes_1639_v123	0.9344
Listeria_monocytogenes_serotype_1_2a_str_10-0812_1196165	Listeria_monocytogenes_1639_v123	0.9344
Listeria_monocytogenes_serotype_1_2a_str_08-7669_1196186	Listeria_monocytogenes_1639_v123	0.9359
Listeria_monocytogenes_serotype_1_2a_str_08-7374_1196185	Listeria_monocytogenes_1639_v123	0.9264
Listeria_monocytogenes_serotype_1_2a_str_08-6997_1196184	Listeria_monocytogenes_1639_v123	0.9223
Listeria_monocytogenes_serotype_1_2a_str_08-6569_1196183	Listeria_monocytogenes_1639_v123	0.9223
Listeria_monocytogenes_serotype_1_2a_str_08-6056_1196182	Listeria_monocytogenes_1639_v123	0.9223
Listeria_monocytogenes_serotype_1_2a_str_04-5457_1196181	Listeria_monocytogenes_1639_v123	0.9264
Listeria_monocytogenes_serotype_1_2a_str_02-5993_1196180	Listeria_monocytogenes_1639_v123	0.9264
Listeria_monocytogenes_R479a_1437838	Listeria_monocytogenes_1639_v123	0.9261
Listeria_monocytogenes_N53-1_1234142	Listeria_monocytogenes_1639_v123	0.8976
Listeria_monocytogenes_M7_1030009	Listeria_monocytogenes_1639_v123	0.9093
Listeria_monocytogenes_LO28_393132	Listeria_monocytogenes_1639_v123	0.9328
Listeria_monocytogenes_L99_563174	Listeria_monocytogenes_1639_v123	0.9096
Listeria_monocytogenes_L312_882094	Listeria_monocytogenes_1639_v123	0.9807
Listeria_monocytogenes_J1816_930781	Listeria_monocytogenes_1639_v123	0.9619
Listeria_monocytogenes_J1-220_930782	Listeria_monocytogenes_1639_v123	0.9523
Listeria_monocytogenes_J0161_393130	Listeria_monocytogenes_1639_v123	0.9317

Listeria_monocytogenes_HCC23_552536	Listeria_monocytogenes_1639_v123	0.9089
Listeria_monocytogenes_FSL_R2-561_393126	Listeria_monocytogenes_1639_v123	0.9328
Listeria_monocytogenes_FSL_J1-208_393119	Listeria_monocytogenes_1639_v123	0.9216
Listeria_monocytogenes_FSL_J1-175_393118	Listeria_monocytogenes_1639_v123	0.9614
Listeria_monocytogenes_Finland_1998_393127	Listeria_monocytogenes_1639_v123	0.9539
Listeria_monocytogenes_EGD-e_169963_v1	Listeria_monocytogenes_1639_v123	0.9424
Listeria_monocytogenes_EGD-e_169963	Listeria_monocytogenes_1639_v123	0.9428
Listeria_monocytogenes_EGD_1334565	Listeria_monocytogenes_1639_v123	0.9419
Listeria_monocytogenes_CFSAN002349_1299895	Listeria_monocytogenes_1639_v123	1
Listeria_monocytogenes_ATCC_19117_882095_v1	Listeria_monocytogenes_1639_v123	0.9728
Listeria_monocytogenes_ATCC_19117_882095	Listeria_monocytogenes_1639_v123	0.9724
Listeria_monocytogenes_6179_1288295	Listeria_monocytogenes_1639_v123	0.9156
Listeria_monocytogenes_1639_v9	Listeria_monocytogenes_1639_v123	0.9317
Listeria_monocytogenes_1639_v99	Listeria_monocytogenes_1639_v123	0.9631
Listeria_monocytogenes_1639_v98	Listeria_monocytogenes_1639_v123	0.929
Listeria_monocytogenes_1639_v97	Listeria_monocytogenes_1639_v123	0.9688
Listeria_monocytogenes_1639_v96	Listeria_monocytogenes_1639_v123	0.9623
Listeria_monocytogenes_1639_v95	Listeria_monocytogenes_1639_v123	0.9623
Listeria_monocytogenes_1639_v94	Listeria_monocytogenes_1639_v123	0.9627
Listeria_monocytogenes_1639_v93	Listeria_monocytogenes_1639_v123	0.9298
Listeria_monocytogenes_1639_v92	Listeria_monocytogenes_1639_v123	0.9627
Listeria_monocytogenes_1639_v91	Listeria_monocytogenes_1639_v123	0.9581
Listeria_monocytogenes_1639_v90	Listeria_monocytogenes_1639_v123	0.929
Listeria_monocytogenes_1639_v8	Listeria_monocytogenes_1639_v123	0.9155
Listeria_monocytogenes_1639_v89	Listeria_monocytogenes_1639_v123	0.9706
Listeria_monocytogenes_1639_v88	Listeria_monocytogenes_1639_v123	0.9341
Listeria_monocytogenes_1639_v87	Listeria_monocytogenes_1639_v123	0.9298
Listeria_monocytogenes_1639_v86	Listeria_monocytogenes_1639_v123	0.9294
Listeria_monocytogenes_1639_v85	Listeria_monocytogenes_1639_v123	0.9611
Listeria_monocytogenes_1639_v84	Listeria_monocytogenes_1639_v123	0.937
Listeria_monocytogenes_1639_v83	Listeria_monocytogenes_1639_v123	0.9613
Listeria_monocytogenes_1639_v82	Listeria_monocytogenes_1639_v123	0.9606
Listeria_monocytogenes_1639_v81	Listeria_monocytogenes_1639_v123	0.9341
Listeria_monocytogenes_1639_v80	Listeria_monocytogenes_1639_v123	0.9529
Listeria_monocytogenes_1639_v7	Listeria_monocytogenes_1639_v123	0.9155
Listeria_monocytogenes_1639_v79	Listeria_monocytogenes_1639_v123	0.9323
Listeria_monocytogenes_1639_v78	Listeria_monocytogenes_1639_v123	0.9334
Listeria_monocytogenes_1639_v77	Listeria_monocytogenes_1639_v123	0.9335
Listeria_monocytogenes_1639_v76	Listeria_monocytogenes_1639_v123	0.9263
Listeria_monocytogenes_1639_v75	Listeria_monocytogenes_1639_v123	0.9096
Listeria_monocytogenes_1639_v74	Listeria_monocytogenes_1639_v123	0.9291
Listeria_monocytogenes_1639_v73	Listeria_monocytogenes_1639_v123	0.9279
Listeria_monocytogenes_1639_v72	Listeria_monocytogenes_1639_v123	0.9598
Listeria_monocytogenes_1639_v71	Listeria_monocytogenes_1639_v123	0.9176
Listeria_monocytogenes_1639_v70	Listeria_monocytogenes_1639_v123	0.9304
Listeria_monocytogenes_1639_v6	Listeria_monocytogenes_1639_v123	0.9151
Listeria_monocytogenes_1639_v69	Listeria_monocytogenes_1639_v123	0.9629
Listeria_monocytogenes_1639_v68	Listeria_monocytogenes_1639_v123	0.9359
Listeria_monocytogenes_1639_v67	Listeria_monocytogenes_1639_v123	0.9511
Listeria_monocytogenes_1639_v66	Listeria_monocytogenes_1639_v123	0.9375
Listeria_monocytogenes_1639_v65	Listeria_monocytogenes_1639_v123	0.9296
Listeria_monocytogenes_1639_v64	Listeria_monocytogenes_1639_v123	0.93
Listeria_monocytogenes_1639_v63	Listeria_monocytogenes_1639_v123	0.9472
Listeria_monocytogenes_1639_v62	Listeria_monocytogenes_1639_v123	0.9298
Listeria_monocytogenes_1639_v61	Listeria_monocytogenes_1639_v123	0.9292
Listeria_monocytogenes_1639_v60	Listeria_monocytogenes_1639_v123	0.9337
Listeria_monocytogenes_1639_v5	Listeria_monocytogenes_1639_v123	0.9127
Listeria_monocytogenes_1639_v59	Listeria_monocytogenes_1639_v123	0.9617
Listeria_monocytogenes_1639_v58	Listeria_monocytogenes_1639_v123	0.9655
Listeria_monocytogenes_1639_v57	Listeria_monocytogenes_1639_v123	0.9591
Listeria_monocytogenes_1639_v56	Listeria_monocytogenes_1639_v123	0.9621
Listeria_monocytogenes_1639_v55	Listeria_monocytogenes_1639_v123	0.9548
Listeria_monocytogenes_1639_v54	Listeria_monocytogenes_1639_v123	0.929
Listeria_monocytogenes_1639_v53	Listeria_monocytogenes_1639_v123	0.9336
Listeria_monocytogenes_1639_v52	Listeria_monocytogenes_1639_v123	0.9584
Listeria_monocytogenes_1639_v51	Listeria_monocytogenes_1639_v123	0.9555
Listeria_monocytogenes_1639_v50	Listeria_monocytogenes_1639_v123	0.9257
Listeria_monocytogenes_1639_v4	Listeria_monocytogenes_1639_v123	0.9257
Listeria_monocytogenes_1639_v49	Listeria_monocytogenes_1639_v123	0.9607
Listeria_monocytogenes_1639_v48	Listeria_monocytogenes_1639_v123	0.9286
Listeria_monocytogenes_1639_v47	Listeria_monocytogenes_1639_v123	0.9261
Listeria_monocytogenes_1639_v46	Listeria_monocytogenes_1639_v123	0.9352
Listeria_monocytogenes_1639_v45	Listeria_monocytogenes_1639_v123	0.9172
Listeria_monocytogenes_1639_v44	Listeria_monocytogenes_1639_v123	0.9264
Listeria_monocytogenes_1639_v43	Listeria_monocytogenes_1639_v123	0.9264
Listeria_monocytogenes_1639_v42	Listeria_monocytogenes_1639_v123	0.9179
Listeria_monocytogenes_1639_v41	Listeria_monocytogenes_1639_v123	0.9074
Listeria_monocytogenes_1639_v40	Listeria_monocytogenes_1639_v123	0.9332
Listeria_monocytogenes_1639_v3	Listeria_monocytogenes_1639_v123	0.9261
Listeria_monocytogenes_1639_v39	Listeria_monocytogenes_1639_v123	0.9483
Listeria_monocytogenes_1639_v38	Listeria_monocytogenes_1639_v123	0.9304
Listeria_monocytogenes_1639_v37	Listeria_monocytogenes_1639_v123	0.9259
Listeria_monocytogenes_1639_v36	Listeria_monocytogenes_1639_v123	0.9226
Listeria_monocytogenes_1639_v35	Listeria_monocytogenes_1639_v123	0.9339
Listeria_monocytogenes_1639_v34	Listeria_monocytogenes_1639_v123	0.9278
Listeria_monocytogenes_1639_v33	Listeria_monocytogenes_1639_v123	0.9462
Listeria_monocytogenes_1639_v32	Listeria_monocytogenes_1639_v123	0.9249
Listeria_monocytogenes_1639_v31	Listeria_monocytogenes_1639_v123	0.9282
Listeria_monocytogenes_1639_v30	Listeria_monocytogenes_1639_v123	0.9495
Listeria_monocytogenes_1639_v2	Listeria_monocytogenes_1639_v123	0.9102
Listeria_monocytogenes_1639_v29	Listeria_monocytogenes_1639_v123	0.9196

Listeria_monocytogenes_1639_v28	Listeria_monocytogenes_1639_v123	0.9241
Listeria_monocytogenes_1639_v27	Listeria_monocytogenes_1639_v123	0.9149
Listeria_monocytogenes_1639_v26	Listeria_monocytogenes_1639_v123	0.9146
Listeria_monocytogenes_1639_v25	Listeria_monocytogenes_1639_v123	0.9146
Listeria_monocytogenes_1639_v24	Listeria_monocytogenes_1639_v123	0.9523
Listeria_monocytogenes_1639_v23	Listeria_monocytogenes_1639_v123	0.9519
Listeria_monocytogenes_1639_v22	Listeria_monocytogenes_1639_v123	0.9488
Listeria_monocytogenes_1639_v21	Listeria_monocytogenes_1639_v123	0.9523
Listeria_monocytogenes_1639_v20	Listeria_monocytogenes_1639_v123	0.9401
Listeria_monocytogenes_1639_v1	Listeria_monocytogenes_1639_v123	0.91
Listeria_monocytogenes_1639_v19	Listeria_monocytogenes_1639_v123	0.9398
Listeria_monocytogenes_1639_v198	Listeria_monocytogenes_1639_v123	0.8999
Listeria_monocytogenes_1639_v197	Listeria_monocytogenes_1639_v123	0.9299
Listeria_monocytogenes_1639_v196	Listeria_monocytogenes_1639_v123	0.9246
Listeria_monocytogenes_1639_v195	Listeria_monocytogenes_1639_v123	0.9473
Listeria_monocytogenes_1639_v194	Listeria_monocytogenes_1639_v123	0.9467
Listeria_monocytogenes_1639_v193	Listeria_monocytogenes_1639_v123	0.9467
Listeria_monocytogenes_1639_v192	Listeria_monocytogenes_1639_v123	0.9129
Listeria_monocytogenes_1639_v191	Listeria_monocytogenes_1639_v123	0.9535
Listeria_monocytogenes_1639_v190	Listeria_monocytogenes_1639_v123	0.9535
Listeria_monocytogenes_1639_v18	Listeria_monocytogenes_1639_v123	0.953
Listeria_monocytogenes_1639_v189	Listeria_monocytogenes_1639_v123	0.9527
Listeria_monocytogenes_1639_v188	Listeria_monocytogenes_1639_v123	0.9534
Listeria_monocytogenes_1639_v187	Listeria_monocytogenes_1639_v123	0.9535
Listeria_monocytogenes_1639_v186	Listeria_monocytogenes_1639_v123	0.9539
Listeria_monocytogenes_1639_v185	Listeria_monocytogenes_1639_v123	0.9539
Listeria_monocytogenes_1639_v184	Listeria_monocytogenes_1639_v123	0.9531
Listeria_monocytogenes_1639_v183	Listeria_monocytogenes_1639_v123	0.9484
Listeria_monocytogenes_1639_v182	Listeria_monocytogenes_1639_v123	0.9507
Listeria_monocytogenes_1639_v181	Listeria_monocytogenes_1639_v123	0.934
Listeria_monocytogenes_1639_v180	Listeria_monocytogenes_1639_v123	0.934
Listeria_monocytogenes_1639_v17	Listeria_monocytogenes_1639_v123	0.9534
Listeria_monocytogenes_1639_v179	Listeria_monocytogenes_1639_v123	0.9382
Listeria_monocytogenes_1639_v178	Listeria_monocytogenes_1639_v123	0.9551
Listeria_monocytogenes_1639_v177	Listeria_monocytogenes_1639_v123	0.9823
Listeria_monocytogenes_1639_v176	Listeria_monocytogenes_1639_v123	0.9444
Listeria_monocytogenes_1639_v175	Listeria_monocytogenes_1639_v123	0.9424
Listeria_monocytogenes_1639_v174	Listeria_monocytogenes_1639_v123	0.954
Listeria_monocytogenes_1639_v173	Listeria_monocytogenes_1639_v123	0.971
Listeria_monocytogenes_1639_v172	Listeria_monocytogenes_1639_v123	0.9426
Listeria_monocytogenes_1639_v171	Listeria_monocytogenes_1639_v123	0.939
Listeria_monocytogenes_1639_v170	Listeria_monocytogenes_1639_v123	0.9473
Listeria_monocytogenes_1639_v16	Listeria_monocytogenes_1639_v123	0.9128
Listeria_monocytogenes_1639_v169	Listeria_monocytogenes_1639_v123	0.947
Listeria_monocytogenes_1639_v168	Listeria_monocytogenes_1639_v123	0.9474
Listeria_monocytogenes_1639_v167	Listeria_monocytogenes_1639_v123	0.94
Listeria_monocytogenes_1639_v166	Listeria_monocytogenes_1639_v123	0.9478
Listeria_monocytogenes_1639_v165	Listeria_monocytogenes_1639_v123	0.947
Listeria_monocytogenes_1639_v164	Listeria_monocytogenes_1639_v123	0.978
Listeria_monocytogenes_1639_v163	Listeria_monocytogenes_1639_v123	0.9677
Listeria_monocytogenes_1639_v162	Listeria_monocytogenes_1639_v123	0.978
Listeria_monocytogenes_1639_v161	Listeria_monocytogenes_1639_v123	0.978
Listeria_monocytogenes_1639_v160	Listeria_monocytogenes_1639_v123	0.9777
Listeria_monocytogenes_1639_v15	Listeria_monocytogenes_1639_v123	0.9323
Listeria_monocytogenes_1639_v159	Listeria_monocytogenes_1639_v123	0.9827
Listeria_monocytogenes_1639_v158	Listeria_monocytogenes_1639_v123	0.9788
Listeria_monocytogenes_1639_v157	Listeria_monocytogenes_1639_v123	0.9784
Listeria_monocytogenes_1639_v156	Listeria_monocytogenes_1639_v123	0.9312
Listeria_monocytogenes_1639_v155	Listeria_monocytogenes_1639_v123	0.9414
Listeria_monocytogenes_1639_v154	Listeria_monocytogenes_1639_v123	0.9452
Listeria_monocytogenes_1639_v153	Listeria_monocytogenes_1639_v123	0.9421
Listeria_monocytogenes_1639_v152	Listeria_monocytogenes_1639_v123	0.9827
Listeria_monocytogenes_1639_v151	Listeria_monocytogenes_1639_v123	0.9827
Listeria_monocytogenes_1639_v150	Listeria_monocytogenes_1639_v123	0.9436
Listeria_monocytogenes_1639_v14	Listeria_monocytogenes_1639_v123	0.9392
Listeria_monocytogenes_1639_v149	Listeria_monocytogenes_1639_v123	0.9422
Listeria_monocytogenes_1639_v148	Listeria_monocytogenes_1639_v123	0.9816
Listeria_monocytogenes_1639_v147	Listeria_monocytogenes_1639_v123	0.9678
Listeria_monocytogenes_1639_v146	Listeria_monocytogenes_1639_v123	0.9517
Listeria_monocytogenes_1639_v145	Listeria_monocytogenes_1639_v123	0.9468
Listeria_monocytogenes_1639_v144	Listeria_monocytogenes_1639_v123	0.98
Listeria_monocytogenes_1639_v143	Listeria_monocytogenes_1639_v123	0.9518
Listeria_monocytogenes_1639_v142	Listeria_monocytogenes_1639_v123	0.9803
Listeria_monocytogenes_1639_v141	Listeria_monocytogenes_1639_v123	0.9722
Listeria_monocytogenes_1639_v140	Listeria_monocytogenes_1639_v123	0.9811
Listeria_monocytogenes_1639_v13	Listeria_monocytogenes_1639_v123	0.9319
Listeria_monocytogenes_1639_v139	Listeria_monocytogenes_1639_v123	0.98
Listeria_monocytogenes_1639_v138	Listeria_monocytogenes_1639_v123	0.983
Listeria_monocytogenes_1639_v137	Listeria_monocytogenes_1639_v123	0.9827
Listeria_monocytogenes_1639_v136	Listeria_monocytogenes_1639_v123	0.942
Listeria_monocytogenes_1639_v135	Listeria_monocytogenes_1639_v123	0.9213
Listeria_monocytogenes_1639_v134	Listeria_monocytogenes_1639_v123	0.9728
Listeria_monocytogenes_1639_v133	Listeria_monocytogenes_1639_v123	0.9391
Listeria_monocytogenes_1639_v132	Listeria_monocytogenes_1639_v123	0.9218
Listeria_monocytogenes_1639_v131	Listeria_monocytogenes_1639_v123	0.9703
Listeria_monocytogenes_1639_v130	Listeria_monocytogenes_1639_v123	0.9339
Listeria_monocytogenes_1639_v12	Listeria_monocytogenes_1639_v123	0.9136
Listeria_monocytogenes_1639_v129	Listeria_monocytogenes_1639_v123	0.9703
Listeria_monocytogenes_1639_v128	Listeria_monocytogenes_1639_v123	0.9681
Listeria_monocytogenes_1639_v127	Listeria_monocytogenes_1639_v123	0.9421
Listeria_monocytogenes_1639_v126	Listeria_monocytogenes_1639_v123	0.9662

Listeria_monocytogenes_1639_v125	Listeria_monocytogenes_1639_v123	0.9387
Listeria_monocytogenes_1639_v124	Listeria_monocytogenes_1639_v123	0.9399
Listeria_monocytogenes_1639_v123	Listeria_monocytogenes_1639_v123	0.0676
Listeria_welshimeri_1643	Listeria_monocytogenes_1639_v124	0.8829
Listeria_welshimeri_serovar_6b_str_SLCC5334_386043	Listeria_monocytogenes_1639_v124	0.8822
Listeria_seeligeri_serovar_1_2b_str_SLCC3954_683837	Listeria_monocytogenes_1639_v124	0.8813
Listeria_seeligeri_FSL_S4-171_702452	Listeria_monocytogenes_1639_v124	0.822
Listeria_seeligeri_FSL_N1-067_702453	Listeria_monocytogenes_1639_v124	0.8239
Listeria_seeligeri_1640_v1	Listeria_monocytogenes_1639_v124	0.8859
Listeria_seeligeri_1640	Listeria_monocytogenes_1639_v124	0.8771
Listeria_monocytogenes_WSLC1042_1457188	Listeria_monocytogenes_1639_v124	0.9433
Listeria_monocytogenes_WSLC1001_1457187	Listeria_monocytogenes_1639_v124	0.9231
Listeria_monocytogenes_SLCC7179_879090	Listeria_monocytogenes_1639_v124	0.9262
Listeria_monocytogenes_SLCC5850_882096	Listeria_monocytogenes_1639_v124	0.9273
Listeria_monocytogenes_SLCC2755_932919	Listeria_monocytogenes_1639_v124	0.9264
Listeria_monocytogenes_SLCC2540_879089	Listeria_monocytogenes_1639_v124	0.9287
Listeria_monocytogenes_SLCC2479_882020	Listeria_monocytogenes_1639_v124	0.9142
Listeria_monocytogenes_SLCC2378_879088	Listeria_monocytogenes_1639_v124	0.9448
Listeria_monocytogenes_SLCC2376_882097	Listeria_monocytogenes_1639_v124	0.9523
Listeria_monocytogenes_SLCC2372_932920	Listeria_monocytogenes_1639_v124	0.9067
Listeria_monocytogenes_serotype_4b_str_LL195_1230340	Listeria_monocytogenes_1639_v124	0.9471
Listeria_monocytogenes_serotype_4b_str_LIS0087_1134669	Listeria_monocytogenes_1639_v124	0.9036
Listeria_monocytogenes_serotype_4b_str_F2365_265669	Listeria_monocytogenes_1639_v124	0.9471
Listeria_monocytogenes_serotype_4b_str_CLIP_80459_568819	Listeria_monocytogenes_1639_v124	0.9424
Listeria_monocytogenes_serotype_4b_str_81-0861_1196161	Listeria_monocytogenes_1639_v124	0.9337
Listeria_monocytogenes_serotype_4b_str_81-0592_1196160	Listeria_monocytogenes_1639_v124	0.9348
Listeria_monocytogenes_serotype_4b_str_81-0558_1196159	Listeria_monocytogenes_1639_v124	0.9355
Listeria_monocytogenes_serotype_4b_str_10-0809_1196162	Listeria_monocytogenes_1639_v124	0.9348
Listeria_monocytogenes_serotype_4b_str_02-6680_1196175	Listeria_monocytogenes_1639_v124	0.9418
Listeria_monocytogenes_serotype_4b_str_02-6679_1196174	Listeria_monocytogenes_1639_v124	0.9418
Listeria_monocytogenes_serotype_4b_str_02-1792_1196173	Listeria_monocytogenes_1639_v124	0.9348
Listeria_monocytogenes_serotype_4b_str_02-1289_1196172	Listeria_monocytogenes_1639_v124	0.9348
Listeria_monocytogenes_serotype_4b_str_02-1103_1196171	Listeria_monocytogenes_1639_v124	0.9348
Listeria_monocytogenes_serotype_3c_str_10-5027_1196195	Listeria_monocytogenes_1639_v124	0.9131
Listeria_monocytogenes_serotype_1_2c_str_10-5026_1196194	Listeria_monocytogenes_1639_v124	0.9142
Listeria_monocytogenes_serotype_1_2c_str_10-5025_1196193	Listeria_monocytogenes_1639_v124	0.9142
Listeria_monocytogenes_serotype_1_2b_str_10-0811_1196164	Listeria_monocytogenes_1639_v124	0.9168
Listeria_monocytogenes_serotype_1_2b_str_10-0810_1196163	Listeria_monocytogenes_1639_v124	0.9168
Listeria_monocytogenes_serotype_1_2a_str_99-6370_1196179	Listeria_monocytogenes_1639_v124	0.8997
Listeria_monocytogenes_serotype_1_2a_str_98-2035_1196178	Listeria_monocytogenes_1639_v124	0.8997
Listeria_monocytogenes_serotype_1_2a_str_95-0093_1196177	Listeria_monocytogenes_1639_v124	0.9055
Listeria_monocytogenes_serotype_1_2a_str_88-0478_1196176	Listeria_monocytogenes_1639_v124	0.9046
Listeria_monocytogenes_serotype_1_2a_str_10-5024_1196192	Listeria_monocytogenes_1639_v124	0.9058
Listeria_monocytogenes_serotype_1_2a_str_10-4758_1196168	Listeria_monocytogenes_1639_v124	0.9236
Listeria_monocytogenes_serotype_1_2a_str_10-4754_1196167	Listeria_monocytogenes_1639_v124	0.9236
Listeria_monocytogenes_serotype_1_2a_str_10-1321_1196191	Listeria_monocytogenes_1639_v124	0.9058
Listeria_monocytogenes_serotype_1_2a_str_10-1047_1196190	Listeria_monocytogenes_1639_v124	0.9061
Listeria_monocytogenes_serotype_1_2a_str_10-1046_1196189	Listeria_monocytogenes_1639_v124	0.9061
Listeria_monocytogenes_serotype_1_2a_str_10-0934_1196170	Listeria_monocytogenes_1639_v124	0.9353
Listeria_monocytogenes_serotype_1_2a_str_10-0933_1196169	Listeria_monocytogenes_1639_v124	0.9353
Listeria_monocytogenes_serotype_1_2a_str_10-0815_1196188	Listeria_monocytogenes_1639_v124	0.9011
Listeria_monocytogenes_serotype_1_2a_str_10-0814_1196187	Listeria_monocytogenes_1639_v124	0.9055
Listeria_monocytogenes_serotype_1_2a_str_10-0813_1196166	Listeria_monocytogenes_1639_v124	0.9199
Listeria_monocytogenes_serotype_1_2a_str_10-0812_1196165	Listeria_monocytogenes_1639_v124	0.9199
Listeria_monocytogenes_serotype_1_2a_str_08-7669_1196186	Listeria_monocytogenes_1639_v124	0.915
Listeria_monocytogenes_serotype_1_2a_str_08-7374_1196185	Listeria_monocytogenes_1639_v124	0.9058
Listeria_monocytogenes_serotype_1_2a_str_08-6997_1196184	Listeria_monocytogenes_1639_v124	0.9011
Listeria_monocytogenes_serotype_1_2a_str_08-6569_1196183	Listeria_monocytogenes_1639_v124	0.9011
Listeria_monocytogenes_serotype_1_2a_str_08-6056_1196182	Listeria_monocytogenes_1639_v124	0.9011
Listeria_monocytogenes_serotype_1_2a_str_04-5457_1196181	Listeria_monocytogenes_1639_v124	0.9058
Listeria_monocytogenes_serotype_1_2a_str_02-5993_1196180	Listeria_monocytogenes_1639_v124	0.9055
Listeria_monocytogenes_R479a_1437838	Listeria_monocytogenes_1639_v124	0.9044
Listeria_monocytogenes_N53-1_1234142	Listeria_monocytogenes_1639_v124	0.8808
Listeria_monocytogenes_M7_1030009	Listeria_monocytogenes_1639_v124	0.9168
Listeria_monocytogenes_LO28_393132	Listeria_monocytogenes_1639_v124	0.9128
Listeria_monocytogenes_L99_563174	Listeria_monocytogenes_1639_v124	0.9175
Listeria_monocytogenes_L312_882094	Listeria_monocytogenes_1639_v124	0.9424
Listeria_monocytogenes_J1816_930781	Listeria_monocytogenes_1639_v124	0.9219
Listeria_monocytogenes_J1-220_930782	Listeria_monocytogenes_1639_v124	0.9248
Listeria_monocytogenes_J0161_393130	Listeria_monocytogenes_1639_v124	0.9188
Listeria_monocytogenes_HCC23_552536	Listeria_monocytogenes_1639_v124	0.9171
Listeria_monocytogenes_FSL_R2-561_393126	Listeria_monocytogenes_1639_v124	0.9124
Listeria_monocytogenes_FSL_J1-208_393119	Listeria_monocytogenes_1639_v124	0.9292
Listeria_monocytogenes_FSL_J1-175_393118	Listeria_monocytogenes_1639_v124	0.9342
Listeria_monocytogenes_Finland_1998_393127	Listeria_monocytogenes_1639_v124	0.9318
Listeria_monocytogenes_EGD-e_169963_v1	Listeria_monocytogenes_1639_v124	0.9275
Listeria_monocytogenes_EGD-e_169963	Listeria_monocytogenes_1639_v124	0.9275
Listeria_monocytogenes_EGD_1334565	Listeria_monocytogenes_1639_v124	0.928
Listeria_monocytogenes_CFSAN002349_1299895	Listeria_monocytogenes_1639_v124	0.9399
Listeria_monocytogenes_ATCC_19117_882095_v1	Listeria_monocytogenes_1639_v124	0.9442
Listeria_monocytogenes_ATCC_19117_882095	Listeria_monocytogenes_1639_v124	0.9442
Listeria_monocytogenes_6179_1288295	Listeria_monocytogenes_1639_v124	0.9033
Listeria_monocytogenes_1639_v9	Listeria_monocytogenes_1639_v124	0.9062
Listeria_monocytogenes_1639_v99	Listeria_monocytogenes_1639_v124	0.9227
Listeria_monocytogenes_1639_v98	Listeria_monocytogenes_1639_v124	0.9142
Listeria_monocytogenes_1639_v97	Listeria_monocytogenes_1639_v124	0.9315
Listeria_monocytogenes_1639_v96	Listeria_monocytogenes_1639_v124	0.9216
Listeria_monocytogenes_1639_v95	Listeria_monocytogenes_1639_v124	0.9216
Listeria_monocytogenes_1639_v94	Listeria_monocytogenes_1639_v124	0.922
Listeria_monocytogenes_1639_v93	Listeria_monocytogenes_1639_v124	0.9106
Listeria_monocytogenes_1639_v92	Listeria_monocytogenes_1639_v124	0.9223

Listeria_monocytogenes_1639_v91	Listeria_monocytogenes_1639_v124	0.9289
Listeria_monocytogenes_1639_v90	Listeria_monocytogenes_1639_v124	0.9142
Listeria_monocytogenes_1639_v8	Listeria_monocytogenes_1639_v124	0.8944
Listeria_monocytogenes_1639_v89	Listeria_monocytogenes_1639_v124	0.9282
Listeria_monocytogenes_1639_v88	Listeria_monocytogenes_1639_v124	0.9159
Listeria_monocytogenes_1639_v87	Listeria_monocytogenes_1639_v124	0.9154
Listeria_monocytogenes_1639_v86	Listeria_monocytogenes_1639_v124	0.9147
Listeria_monocytogenes_1639_v85	Listeria_monocytogenes_1639_v124	0.933
Listeria_monocytogenes_1639_v84	Listeria_monocytogenes_1639_v124	0.9175
Listeria_monocytogenes_1639_v83	Listeria_monocytogenes_1639_v124	0.9332
Listeria_monocytogenes_1639_v82	Listeria_monocytogenes_1639_v124	0.9325
Listeria_monocytogenes_1639_v81	Listeria_monocytogenes_1639_v124	0.9132
Listeria_monocytogenes_1639_v80	Listeria_monocytogenes_1639_v124	0.9221
Listeria_monocytogenes_1639_v7	Listeria_monocytogenes_1639_v124	0.8944
Listeria_monocytogenes_1639_v79	Listeria_monocytogenes_1639_v124	0.9111
Listeria_monocytogenes_1639_v78	Listeria_monocytogenes_1639_v124	0.9125
Listeria_monocytogenes_1639_v77	Listeria_monocytogenes_1639_v124	0.9213
Listeria_monocytogenes_1639_v76	Listeria_monocytogenes_1639_v124	0.9076
Listeria_monocytogenes_1639_v75	Listeria_monocytogenes_1639_v124	0.9175
Listeria_monocytogenes_1639_v74	Listeria_monocytogenes_1639_v124	0.9144
Listeria_monocytogenes_1639_v73	Listeria_monocytogenes_1639_v124	0.9081
Listeria_monocytogenes_1639_v72	Listeria_monocytogenes_1639_v124	0.932
Listeria_monocytogenes_1639_v71	Listeria_monocytogenes_1639_v124	0.9027
Listeria_monocytogenes_1639_v70	Listeria_monocytogenes_1639_v124	0.9108
Listeria_monocytogenes_1639_v6	Listeria_monocytogenes_1639_v124	0.8937
Listeria_monocytogenes_1639_v69	Listeria_monocytogenes_1639_v124	0.9351
Listeria_monocytogenes_1639_v68	Listeria_monocytogenes_1639_v124	0.9237
Listeria_monocytogenes_1639_v67	Listeria_monocytogenes_1639_v124	0.9268
Listeria_monocytogenes_1639_v66	Listeria_monocytogenes_1639_v124	0.9188
Listeria_monocytogenes_1639_v65	Listeria_monocytogenes_1639_v124	0.9081
Listeria_monocytogenes_1639_v64	Listeria_monocytogenes_1639_v124	0.9186
Listeria_monocytogenes_1639_v63	Listeria_monocytogenes_1639_v124	0.9171
Listeria_monocytogenes_1639_v62	Listeria_monocytogenes_1639_v124	0.9087
Listeria_monocytogenes_1639_v61	Listeria_monocytogenes_1639_v124	0.9081
Listeria_monocytogenes_1639_v60	Listeria_monocytogenes_1639_v124	0.9207
Listeria_monocytogenes_1639_v5	Listeria_monocytogenes_1639_v124	0.8831
Listeria_monocytogenes_1639_v59	Listeria_monocytogenes_1639_v124	0.9343
Listeria_monocytogenes_1639_v58	Listeria_monocytogenes_1639_v124	0.935
Listeria_monocytogenes_1639_v57	Listeria_monocytogenes_1639_v124	0.9186
Listeria_monocytogenes_1639_v56	Listeria_monocytogenes_1639_v124	0.9335
Listeria_monocytogenes_1639_v55	Listeria_monocytogenes_1639_v124	0.9148
Listeria_monocytogenes_1639_v54	Listeria_monocytogenes_1639_v124	0.9205
Listeria_monocytogenes_1639_v53	Listeria_monocytogenes_1639_v124	0.92
Listeria_monocytogenes_1639_v52	Listeria_monocytogenes_1639_v124	0.9274
Listeria_monocytogenes_1639_v51	Listeria_monocytogenes_1639_v124	0.9287
Listeria_monocytogenes_1639_v50	Listeria_monocytogenes_1639_v124	0.9047
Listeria_monocytogenes_1639_v4	Listeria_monocytogenes_1639_v124	0.8957
Listeria_monocytogenes_1639_v49	Listeria_monocytogenes_1639_v124	0.9371
Listeria_monocytogenes_1639_v48	Listeria_monocytogenes_1639_v124	0.9084
Listeria_monocytogenes_1639_v47	Listeria_monocytogenes_1639_v124	0.9055
Listeria_monocytogenes_1639_v46	Listeria_monocytogenes_1639_v124	0.923
Listeria_monocytogenes_1639_v45	Listeria_monocytogenes_1639_v124	0.8961
Listeria_monocytogenes_1639_v44	Listeria_monocytogenes_1639_v124	0.9058
Listeria_monocytogenes_1639_v43	Listeria_monocytogenes_1639_v124	0.9058
Listeria_monocytogenes_1639_v42	Listeria_monocytogenes_1639_v124	0.906
Listeria_monocytogenes_1639_v41	Listeria_monocytogenes_1639_v124	0.8909
Listeria_monocytogenes_1639_v40	Listeria_monocytogenes_1639_v124	0.9132
Listeria_monocytogenes_1639_v3	Listeria_monocytogenes_1639_v124	0.896
Listeria_monocytogenes_1639_v39	Listeria_monocytogenes_1639_v124	0.9219
Listeria_monocytogenes_1639_v38	Listeria_monocytogenes_1639_v124	0.9131
Listeria_monocytogenes_1639_v37	Listeria_monocytogenes_1639_v124	0.9057
Listeria_monocytogenes_1639_v36	Listeria_monocytogenes_1639_v124	0.9059
Listeria_monocytogenes_1639_v35	Listeria_monocytogenes_1639_v124	0.9138
Listeria_monocytogenes_1639_v34	Listeria_monocytogenes_1639_v124	0.9158
Listeria_monocytogenes_1639_v33	Listeria_monocytogenes_1639_v124	0.9168
Listeria_monocytogenes_1639_v32	Listeria_monocytogenes_1639_v124	0.9043
Listeria_monocytogenes_1639_v31	Listeria_monocytogenes_1639_v124	0.9162
Listeria_monocytogenes_1639_v30	Listeria_monocytogenes_1639_v124	0.9193
Listeria_monocytogenes_1639_v2	Listeria_monocytogenes_1639_v124	0.8864
Listeria_monocytogenes_1639_v29	Listeria_monocytogenes_1639_v124	0.9
Listeria_monocytogenes_1639_v28	Listeria_monocytogenes_1639_v124	0.9043
Listeria_monocytogenes_1639_v27	Listeria_monocytogenes_1639_v124	0.8954
Listeria_monocytogenes_1639_v26	Listeria_monocytogenes_1639_v124	0.895
Listeria_monocytogenes_1639_v25	Listeria_monocytogenes_1639_v124	0.8947
Listeria_monocytogenes_1639_v24	Listeria_monocytogenes_1639_v124	0.9248
Listeria_monocytogenes_1639_v23	Listeria_monocytogenes_1639_v124	0.9248
Listeria_monocytogenes_1639_v22	Listeria_monocytogenes_1639_v124	0.9178
Listeria_monocytogenes_1639_v21	Listeria_monocytogenes_1639_v124	0.9245
Listeria_monocytogenes_1639_v20	Listeria_monocytogenes_1639_v124	0.914
Listeria_monocytogenes_1639_v1	Listeria_monocytogenes_1639_v124	0.8968
Listeria_monocytogenes_1639_v19	Listeria_monocytogenes_1639_v124	0.9136
Listeria_monocytogenes_1639_v198	Listeria_monocytogenes_1639_v124	0.883
Listeria_monocytogenes_1639_v197	Listeria_monocytogenes_1639_v124	0.9149
Listeria_monocytogenes_1639_v196	Listeria_monocytogenes_1639_v124	0.9113
Listeria_monocytogenes_1639_v195	Listeria_monocytogenes_1639_v124	0.953
Listeria_monocytogenes_1639_v194	Listeria_monocytogenes_1639_v124	0.9535
Listeria_monocytogenes_1639_v193	Listeria_monocytogenes_1639_v124	0.9535
Listeria_monocytogenes_1639_v192	Listeria_monocytogenes_1639_v124	0.9133
Listeria_monocytogenes_1639_v191	Listeria_monocytogenes_1639_v124	0.9359
Listeria_monocytogenes_1639_v190	Listeria_monocytogenes_1639_v124	0.9394
Listeria_monocytogenes_1639_v18	Listeria_monocytogenes_1639_v124	0.9128

Listeria_monocytogenes_1639_v189	Listeria_monocytogenes_1639_v124	0.9387
Listeria_monocytogenes_1639_v188	Listeria_monocytogenes_1639_v124	0.9382
Listeria_monocytogenes_1639_v187	Listeria_monocytogenes_1639_v124	0.939
Listeria_monocytogenes_1639_v186	Listeria_monocytogenes_1639_v124	0.9398
Listeria_monocytogenes_1639_v185	Listeria_monocytogenes_1639_v124	0.9394
Listeria_monocytogenes_1639_v184	Listeria_monocytogenes_1639_v124	0.939
Listeria_monocytogenes_1639_v183	Listeria_monocytogenes_1639_v124	0.9348
Listeria_monocytogenes_1639_v182	Listeria_monocytogenes_1639_v124	0.937
Listeria_monocytogenes_1639_v181	Listeria_monocytogenes_1639_v124	0.9508
Listeria_monocytogenes_1639_v180	Listeria_monocytogenes_1639_v124	0.9508
Listeria_monocytogenes_1639_v17	Listeria_monocytogenes_1639_v124	0.9128
Listeria_monocytogenes_1639_v179	Listeria_monocytogenes_1639_v124	0.9213
Listeria_monocytogenes_1639_v178	Listeria_monocytogenes_1639_v124	0.936
Listeria_monocytogenes_1639_v177	Listeria_monocytogenes_1639_v124	0.9407
Listeria_monocytogenes_1639_v176	Listeria_monocytogenes_1639_v124	0.945
Listeria_monocytogenes_1639_v175	Listeria_monocytogenes_1639_v124	0.9262
Listeria_monocytogenes_1639_v174	Listeria_monocytogenes_1639_v124	0.9368
Listeria_monocytogenes_1639_v173	Listeria_monocytogenes_1639_v124	0.9445
Listeria_monocytogenes_1639_v172	Listeria_monocytogenes_1639_v124	0.9487
Listeria_monocytogenes_1639_v171	Listeria_monocytogenes_1639_v124	0.9248
Listeria_monocytogenes_1639_v170	Listeria_monocytogenes_1639_v124	0.933
Listeria_monocytogenes_1639_v16	Listeria_monocytogenes_1639_v124	0.8921
Listeria_monocytogenes_1639_v169	Listeria_monocytogenes_1639_v124	0.9296
Listeria_monocytogenes_1639_v168	Listeria_monocytogenes_1639_v124	0.928
Listeria_monocytogenes_1639_v167	Listeria_monocytogenes_1639_v124	0.9238
Listeria_monocytogenes_1639_v166	Listeria_monocytogenes_1639_v124	0.9284
Listeria_monocytogenes_1639_v165	Listeria_monocytogenes_1639_v124	0.9293
Listeria_monocytogenes_1639_v164	Listeria_monocytogenes_1639_v124	0.9463
Listeria_monocytogenes_1639_v163	Listeria_monocytogenes_1639_v124	0.9383
Listeria_monocytogenes_1639_v162	Listeria_monocytogenes_1639_v124	0.9463
Listeria_monocytogenes_1639_v161	Listeria_monocytogenes_1639_v124	0.9474
Listeria_monocytogenes_1639_v160	Listeria_monocytogenes_1639_v124	0.9474
Listeria_monocytogenes_1639_v15	Listeria_monocytogenes_1639_v124	0.9016
Listeria_monocytogenes_1639_v159	Listeria_monocytogenes_1639_v124	0.9411
Listeria_monocytogenes_1639_v158	Listeria_monocytogenes_1639_v124	0.9471
Listeria_monocytogenes_1639_v157	Listeria_monocytogenes_1639_v124	0.9471
Listeria_monocytogenes_1639_v156	Listeria_monocytogenes_1639_v124	0.9164
Listeria_monocytogenes_1639_v155	Listeria_monocytogenes_1639_v124	0.9263
Listeria_monocytogenes_1639_v154	Listeria_monocytogenes_1639_v124	0.9237
Listeria_monocytogenes_1639_v153	Listeria_monocytogenes_1639_v124	0.9271
Listeria_monocytogenes_1639_v152	Listeria_monocytogenes_1639_v124	0.9414
Listeria_monocytogenes_1639_v151	Listeria_monocytogenes_1639_v124	0.9414
Listeria_monocytogenes_1639_v150	Listeria_monocytogenes_1639_v124	0.9294
Listeria_monocytogenes_1639_v14	Listeria_monocytogenes_1639_v124	0.9083
Listeria_monocytogenes_1639_v149	Listeria_monocytogenes_1639_v124	0.928
Listeria_monocytogenes_1639_v148	Listeria_monocytogenes_1639_v124	0.94
Listeria_monocytogenes_1639_v147	Listeria_monocytogenes_1639_v124	0.9261
Listeria_monocytogenes_1639_v146	Listeria_monocytogenes_1639_v124	0.9377
Listeria_monocytogenes_1639_v145	Listeria_monocytogenes_1639_v124	0.9344
Listeria_monocytogenes_1639_v144	Listeria_monocytogenes_1639_v124	0.9417
Listeria_monocytogenes_1639_v143	Listeria_monocytogenes_1639_v124	0.9381
Listeria_monocytogenes_1639_v142	Listeria_monocytogenes_1639_v124	0.9421
Listeria_monocytogenes_1639_v141	Listeria_monocytogenes_1639_v124	0.9397
Listeria_monocytogenes_1639_v140	Listeria_monocytogenes_1639_v124	0.9424
Listeria_monocytogenes_1639_v13	Listeria_monocytogenes_1639_v124	0.902
Listeria_monocytogenes_1639_v139	Listeria_monocytogenes_1639_v124	0.9391
Listeria_monocytogenes_1639_v138	Listeria_monocytogenes_1639_v124	0.9432
Listeria_monocytogenes_1639_v137	Listeria_monocytogenes_1639_v124	0.9428
Listeria_monocytogenes_1639_v136	Listeria_monocytogenes_1639_v124	0.9229
Listeria_monocytogenes_1639_v135	Listeria_monocytogenes_1639_v124	0.9308
Listeria_monocytogenes_1639_v134	Listeria_monocytogenes_1639_v124	0.9442
Listeria_monocytogenes_1639_v133	Listeria_monocytogenes_1639_v124	0.9276
Listeria_monocytogenes_1639_v132	Listeria_monocytogenes_1639_v124	0.939
Listeria_monocytogenes_1639_v131	Listeria_monocytogenes_1639_v124	0.9303
Listeria_monocytogenes_1639_v130	Listeria_monocytogenes_1639_v124	0.9138
Listeria_monocytogenes_1639_v12	Listeria_monocytogenes_1639_v124	0.8937
Listeria_monocytogenes_1639_v129	Listeria_monocytogenes_1639_v124	0.9447
Listeria_monocytogenes_1639_v128	Listeria_monocytogenes_1639_v124	0.9419
Listeria_monocytogenes_1639_v127	Listeria_monocytogenes_1639_v124	0.9245
Listeria_monocytogenes_1639_v126	Listeria_monocytogenes_1639_v124	0.9422
Listeria_monocytogenes_1639_v125	Listeria_monocytogenes_1639_v124	0.9196
Listeria_monocytogenes_1639_v124	Listeria_monocytogenes_1639_v124	0.0632
Listeria_welshimeri_1643	Listeria_monocytogenes_1639_v125	0.8755
Listeria_welshimeri_serovar_6b_str_SLCC5334_386043	Listeria_monocytogenes_1639_v125	0.8749
Listeria_seeligeri_serovar_1_2b_str_SLCC3954_683837	Listeria_monocytogenes_1639_v125	0.887
Listeria_seeligeri_FSL_S4-171_702452	Listeria_monocytogenes_1639_v125	0.8255
Listeria_seeligeri_FSL_N1-067_702453	Listeria_monocytogenes_1639_v125	0.8406
Listeria_seeligeri_1640_v1	Listeria_monocytogenes_1639_v125	0.88
Listeria_seeligeri_1640	Listeria_monocytogenes_1639_v125	0.8816
Listeria_monocytogenes_WSLC1042_1457188	Listeria_monocytogenes_1639_v125	0.9292
Listeria_monocytogenes_WSLC1001_1457187	Listeria_monocytogenes_1639_v125	0.9496
Listeria_monocytogenes_SLCC7179_879090	Listeria_monocytogenes_1639_v125	0.9602
Listeria_monocytogenes_SLCC5850_882096	Listeria_monocytogenes_1639_v125	0.9531
Listeria_monocytogenes_SLCC2755_932919	Listeria_monocytogenes_1639_v125	0.9324
Listeria_monocytogenes_SLCC2540_879089	Listeria_monocytogenes_1639_v125	0.9313
Listeria_monocytogenes_SLCC2479_882020	Listeria_monocytogenes_1639_v125	0.9473
Listeria_monocytogenes_SLCC2378_879088	Listeria_monocytogenes_1639_v125	0.9306
Listeria_monocytogenes_SLCC2376_882097	Listeria_monocytogenes_1639_v125	0.9441
Listeria_monocytogenes_SLCC2372_932920	Listeria_monocytogenes_1639_v125	0.9406
Listeria_monocytogenes_serotype_4b_str_LL195_1230340	Listeria_monocytogenes_1639_v125	0.9363
Listeria_monocytogenes_serotype_4b_str_LIS0087_1134669	Listeria_monocytogenes_1639_v125	0.9335

Listeria_monocytogenes_serotype_4b_str_F2365_265669	Listeria_monocytogenes_1639_v125	0.9363
Listeria_monocytogenes_serotype_4b_str_CLIP_80459_568819	Listeria_monocytogenes_1639_v125	0.9408
Listeria_monocytogenes_serotype_4b_str_81-0861_1196161	Listeria_monocytogenes_1639_v125	0.9242
Listeria_monocytogenes_serotype_4b_str_81-0592_1196160	Listeria_monocytogenes_1639_v125	0.9253
Listeria_monocytogenes_serotype_4b_str_81-0558_1196159	Listeria_monocytogenes_1639_v125	0.926
Listeria_monocytogenes_serotype_4b_str_10-0809_1196162	Listeria_monocytogenes_1639_v125	0.9253
Listeria_monocytogenes_serotype_4b_str_02-6680_1196175	Listeria_monocytogenes_1639_v125	0.9394
Listeria_monocytogenes_serotype_4b_str_02-6679_1196174	Listeria_monocytogenes_1639_v125	0.9394
Listeria_monocytogenes_serotype_4b_str_02-1792_1196173	Listeria_monocytogenes_1639_v125	0.9275
Listeria_monocytogenes_serotype_4b_str_02-1289_1196172	Listeria_monocytogenes_1639_v125	0.9275
Listeria_monocytogenes_serotype_4b_str_02-1103_1196171	Listeria_monocytogenes_1639_v125	0.9275
Listeria_monocytogenes_serotype_3c_str_10-5027_1196195	Listeria_monocytogenes_1639_v125	0.9469
Listeria_monocytogenes_serotype_1_2c_str_10-5026_1196194	Listeria_monocytogenes_1639_v125	0.948
Listeria_monocytogenes_serotype_1_2c_str_10-5025_1196193	Listeria_monocytogenes_1639_v125	0.948
Listeria_monocytogenes_serotype_1_2b_str_10-0811_1196164	Listeria_monocytogenes_1639_v125	0.9265
Listeria_monocytogenes_serotype_1_2b_str_10-0810_1196163	Listeria_monocytogenes_1639_v125	0.9265
Listeria_monocytogenes_serotype_1_2a_str_99-6370_1196179	Listeria_monocytogenes_1639_v125	0.9398
Listeria_monocytogenes_serotype_1_2a_str_98-2035_1196178	Listeria_monocytogenes_1639_v125	0.9398
Listeria_monocytogenes_serotype_1_2a_str_95-0093_1196177	Listeria_monocytogenes_1639_v125	0.9458
Listeria_monocytogenes_serotype_1_2a_str_88-0478_1196176	Listeria_monocytogenes_1639_v125	0.9454
Listeria_monocytogenes_serotype_1_2a_str_10-5024_1196192	Listeria_monocytogenes_1639_v125	0.9461
Listeria_monocytogenes_serotype_1_2a_str_10-4758_1196168	Listeria_monocytogenes_1639_v125	0.9513
Listeria_monocytogenes_serotype_1_2a_str_10-4754_1196167	Listeria_monocytogenes_1639_v125	0.9513
Listeria_monocytogenes_serotype_1_2a_str_10-1321_1196191	Listeria_monocytogenes_1639_v125	0.9461
Listeria_monocytogenes_serotype_1_2a_str_10-1047_1196190	Listeria_monocytogenes_1639_v125	0.9465
Listeria_monocytogenes_serotype_1_2a_str_10-1046_1196189	Listeria_monocytogenes_1639_v125	0.9465
Listeria_monocytogenes_serotype_1_2a_str_10-0934_1196170	Listeria_monocytogenes_1639_v125	0.9645
Listeria_monocytogenes_serotype_1_2a_str_10-0933_1196169	Listeria_monocytogenes_1639_v125	0.9645
Listeria_monocytogenes_serotype_1_2a_str_10-0815_1196188	Listeria_monocytogenes_1639_v125	0.9412
Listeria_monocytogenes_serotype_1_2a_str_10-0814_1196187	Listeria_monocytogenes_1639_v125	0.9458
Listeria_monocytogenes_serotype_1_2a_str_10-0813_1196166	Listeria_monocytogenes_1639_v125	0.9512
Listeria_monocytogenes_serotype_1_2a_str_10-0812_1196165	Listeria_monocytogenes_1639_v125	0.9512
Listeria_monocytogenes_serotype_1_2a_str_08-7669_1196186	Listeria_monocytogenes_1639_v125	0.9554
Listeria_monocytogenes_serotype_1_2a_str_08-7374_1196185	Listeria_monocytogenes_1639_v125	0.9461
Listeria_monocytogenes_serotype_1_2a_str_08-6997_1196184	Listeria_monocytogenes_1639_v125	0.9412
Listeria_monocytogenes_serotype_1_2a_str_08-6569_1196183	Listeria_monocytogenes_1639_v125	0.9412
Listeria_monocytogenes_serotype_1_2a_str_08-6056_1196182	Listeria_monocytogenes_1639_v125	0.9412
Listeria_monocytogenes_serotype_1_2a_str_04-5457_1196181	Listeria_monocytogenes_1639_v125	0.9461
Listeria_monocytogenes_serotype_1_2a_str_02-5993_1196180	Listeria_monocytogenes_1639_v125	0.9458
Listeria_monocytogenes_R479a_1437838	Listeria_monocytogenes_1639_v125	0.9432
Listeria_monocytogenes_N53-1_1234142	Listeria_monocytogenes_1639_v125	0.909
Listeria_monocytogenes_M7_1030009	Listeria_monocytogenes_1639_v125	0.9078
Listeria_monocytogenes_LO28_393132	Listeria_monocytogenes_1639_v125	0.9414
Listeria_monocytogenes_L99_563174	Listeria_monocytogenes_1639_v125	0.9084
Listeria_monocytogenes_L312_882094	Listeria_monocytogenes_1639_v125	0.9408
Listeria_monocytogenes_J1816_930781	Listeria_monocytogenes_1639_v125	0.9339
Listeria_monocytogenes_J1-220_930782	Listeria_monocytogenes_1639_v125	0.9218
Listeria_monocytogenes_J0161_393130	Listeria_monocytogenes_1639_v125	0.9624
Listeria_monocytogenes_HCC23_552536	Listeria_monocytogenes_1639_v125	0.9077
Listeria_monocytogenes_FSL_R2-561_393126	Listeria_monocytogenes_1639_v125	0.9451
Listeria_monocytogenes_FSL_J1-208_393119	Listeria_monocytogenes_1639_v125	0.9028
Listeria_monocytogenes_FSL_J1-175_393118	Listeria_monocytogenes_1639_v125	0.9345
Listeria_monocytogenes_Finland_1998_393127	Listeria_monocytogenes_1639_v125	0.9626
Listeria_monocytogenes_EGD-e_169963_v1	Listeria_monocytogenes_1639_v125	0.9518
Listeria_monocytogenes_EGD-e_169963	Listeria_monocytogenes_1639_v125	0.9518
Listeria_monocytogenes_EGD_1334565	Listeria_monocytogenes_1639_v125	0.9539
Listeria_monocytogenes_CFSAN002349_1299895	Listeria_monocytogenes_1639_v125	0.9387
Listeria_monocytogenes_ATCC_19117_882095_v1	Listeria_monocytogenes_1639_v125	0.935
Listeria_monocytogenes_ATCC_19117_882095	Listeria_monocytogenes_1639_v125	0.935
Listeria_monocytogenes_6179_1288295	Listeria_monocytogenes_1639_v125	0.9451
Listeria_monocytogenes_1639_v9	Listeria_monocytogenes_1639_v125	0.9198
Listeria_monocytogenes_1639_v99	Listeria_monocytogenes_1639_v125	0.935
Listeria_monocytogenes_1639_v98	Listeria_monocytogenes_1639_v125	0.9514
Listeria_monocytogenes_1639_v97	Listeria_monocytogenes_1639_v125	0.9402
Listeria_monocytogenes_1639_v96	Listeria_monocytogenes_1639_v125	0.9343
Listeria_monocytogenes_1639_v95	Listeria_monocytogenes_1639_v125	0.9343
Listeria_monocytogenes_1639_v94	Listeria_monocytogenes_1639_v125	0.9347
Listeria_monocytogenes_1639_v93	Listeria_monocytogenes_1639_v125	0.9431
Listeria_monocytogenes_1639_v92	Listeria_monocytogenes_1639_v125	0.9347
Listeria_monocytogenes_1639_v91	Listeria_monocytogenes_1639_v125	0.9248
Listeria_monocytogenes_1639_v90	Listeria_monocytogenes_1639_v125	0.9514
Listeria_monocytogenes_1639_v8	Listeria_monocytogenes_1639_v125	0.9335
Listeria_monocytogenes_1639_v89	Listeria_monocytogenes_1639_v125	0.9354
Listeria_monocytogenes_1639_v88	Listeria_monocytogenes_1639_v125	0.957
Listeria_monocytogenes_1639_v87	Listeria_monocytogenes_1639_v125	0.9455
Listeria_monocytogenes_1639_v86	Listeria_monocytogenes_1639_v125	0.9451
Listeria_monocytogenes_1639_v85	Listeria_monocytogenes_1639_v125	0.9258
Listeria_monocytogenes_1639_v84	Listeria_monocytogenes_1639_v125	0.9606
Listeria_monocytogenes_1639_v83	Listeria_monocytogenes_1639_v125	0.9286
Listeria_monocytogenes_1639_v82	Listeria_monocytogenes_1639_v125	0.9279
Listeria_monocytogenes_1639_v81	Listeria_monocytogenes_1639_v125	0.9554
Listeria_monocytogenes_1639_v80	Listeria_monocytogenes_1639_v125	0.9296
Listeria_monocytogenes_1639_v7	Listeria_monocytogenes_1639_v125	0.9335
Listeria_monocytogenes_1639_v79	Listeria_monocytogenes_1639_v125	0.954
Listeria_monocytogenes_1639_v78	Listeria_monocytogenes_1639_v125	0.9551
Listeria_monocytogenes_1639_v77	Listeria_monocytogenes_1639_v125	0.9606
Listeria_monocytogenes_1639_v76	Listeria_monocytogenes_1639_v125	0.9472
Listeria_monocytogenes_1639_v75	Listeria_monocytogenes_1639_v125	0.9084
Listeria_monocytogenes_1639_v74	Listeria_monocytogenes_1639_v125	0.944
Listeria_monocytogenes_1639_v73	Listeria_monocytogenes_1639_v125	0.9364
Listeria_monocytogenes_1639_v72	Listeria_monocytogenes_1639_v125	0.9281

Listeria_monocytogenes_1639_v71	Listeria_monocytogenes_1639_v125	0.9429
Listeria_monocytogenes_1639_v70	Listeria_monocytogenes_1639_v125	0.9456
Listeria_monocytogenes_1639_v6	Listeria_monocytogenes_1639_v125	0.9328
Listeria_monocytogenes_1639_v69	Listeria_monocytogenes_1639_v125	0.9257
Listeria_monocytogenes_1639_v68	Listeria_monocytogenes_1639_v125	0.9581
Listeria_monocytogenes_1639_v67	Listeria_monocytogenes_1639_v125	0.9373
Listeria_monocytogenes_1639_v66	Listeria_monocytogenes_1639_v125	0.9552
Listeria_monocytogenes_1639_v65	Listeria_monocytogenes_1639_v125	0.9433
Listeria_monocytogenes_1639_v64	Listeria_monocytogenes_1639_v125	0.948
Listeria_monocytogenes_1639_v63	Listeria_monocytogenes_1639_v125	0.9309
Listeria_monocytogenes_1639_v62	Listeria_monocytogenes_1639_v125	0.9515
Listeria_monocytogenes_1639_v61	Listeria_monocytogenes_1639_v125	0.9493
Listeria_monocytogenes_1639_v60	Listeria_monocytogenes_1639_v125	0.9645
Listeria_monocytogenes_1639_v5	Listeria_monocytogenes_1639_v125	0.8931
Listeria_monocytogenes_1639_v59	Listeria_monocytogenes_1639_v125	0.9267
Listeria_monocytogenes_1639_v58	Listeria_monocytogenes_1639_v125	0.9286
Listeria_monocytogenes_1639_v57	Listeria_monocytogenes_1639_v125	0.9312
Listeria_monocytogenes_1639_v56	Listeria_monocytogenes_1639_v125	0.935
Listeria_monocytogenes_1639_v55	Listeria_monocytogenes_1639_v125	0.9285
Listeria_monocytogenes_1639_v54	Listeria_monocytogenes_1639_v125	0.9277
Listeria_monocytogenes_1639_v53	Listeria_monocytogenes_1639_v125	0.9467
Listeria_monocytogenes_1639_v52	Listeria_monocytogenes_1639_v125	0.9281
Listeria_monocytogenes_1639_v51	Listeria_monocytogenes_1639_v125	0.926
Listeria_monocytogenes_1639_v50	Listeria_monocytogenes_1639_v125	0.9446
Listeria_monocytogenes_1639_v4	Listeria_monocytogenes_1639_v125	0.9048
Listeria_monocytogenes_1639_v49	Listeria_monocytogenes_1639_v125	0.9208
Listeria_monocytogenes_1639_v48	Listeria_monocytogenes_1639_v125	0.9431
Listeria_monocytogenes_1639_v47	Listeria_monocytogenes_1639_v125	0.9461
Listeria_monocytogenes_1639_v46	Listeria_monocytogenes_1639_v125	0.9626
Listeria_monocytogenes_1639_v45	Listeria_monocytogenes_1639_v125	0.9363
Listeria_monocytogenes_1639_v44	Listeria_monocytogenes_1639_v125	0.9461
Listeria_monocytogenes_1639_v43	Listeria_monocytogenes_1639_v125	0.9461
Listeria_monocytogenes_1639_v42	Listeria_monocytogenes_1639_v125	0.9457
Listeria_monocytogenes_1639_v41	Listeria_monocytogenes_1639_v125	0.925
Listeria_monocytogenes_1639_v40	Listeria_monocytogenes_1639_v125	0.9402
Listeria_monocytogenes_1639_v3	Listeria_monocytogenes_1639_v125	0.9051
Listeria_monocytogenes_1639_v39	Listeria_monocytogenes_1639_v125	0.9346
Listeria_monocytogenes_1639_v38	Listeria_monocytogenes_1639_v125	0.9355
Listeria_monocytogenes_1639_v37	Listeria_monocytogenes_1639_v125	0.9321
Listeria_monocytogenes_1639_v36	Listeria_monocytogenes_1639_v125	0.9378
Listeria_monocytogenes_1639_v35	Listeria_monocytogenes_1639_v125	0.9409
Listeria_monocytogenes_1639_v34	Listeria_monocytogenes_1639_v125	0.9588
Listeria_monocytogenes_1639_v33	Listeria_monocytogenes_1639_v125	0.9265
Listeria_monocytogenes_1639_v32	Listeria_monocytogenes_1639_v125	0.9348
Listeria_monocytogenes_1639_v31	Listeria_monocytogenes_1639_v125	0.9592
Listeria_monocytogenes_1639_v30	Listeria_monocytogenes_1639_v125	0.926
Listeria_monocytogenes_1639_v2	Listeria_monocytogenes_1639_v125	0.9061
Listeria_monocytogenes_1639_v29	Listeria_monocytogenes_1639_v125	0.9337
Listeria_monocytogenes_1639_v28	Listeria_monocytogenes_1639_v125	0.9408
Listeria_monocytogenes_1639_v27	Listeria_monocytogenes_1639_v125	0.9237
Listeria_monocytogenes_1639_v26	Listeria_monocytogenes_1639_v125	0.9233
Listeria_monocytogenes_1639_v25	Listeria_monocytogenes_1639_v125	0.923
Listeria_monocytogenes_1639_v24	Listeria_monocytogenes_1639_v125	0.921
Listeria_monocytogenes_1639_v23	Listeria_monocytogenes_1639_v125	0.921
Listeria_monocytogenes_1639_v22	Listeria_monocytogenes_1639_v125	0.9354
Listeria_monocytogenes_1639_v21	Listeria_monocytogenes_1639_v125	0.9203
Listeria_monocytogenes_1639_v20	Listeria_monocytogenes_1639_v125	0.9344
Listeria_monocytogenes_1639_v1	Listeria_monocytogenes_1639_v125	0.9364
Listeria_monocytogenes_1639_v19	Listeria_monocytogenes_1639_v125	0.9341
Listeria_monocytogenes_1639_v198	Listeria_monocytogenes_1639_v125	0.911
Listeria_monocytogenes_1639_v197	Listeria_monocytogenes_1639_v125	0.9388
Listeria_monocytogenes_1639_v196	Listeria_monocytogenes_1639_v125	0.9368
Listeria_monocytogenes_1639_v195	Listeria_monocytogenes_1639_v125	0.9441
Listeria_monocytogenes_1639_v194	Listeria_monocytogenes_1639_v125	0.9445
Listeria_monocytogenes_1639_v193	Listeria_monocytogenes_1639_v125	0.9449
Listeria_monocytogenes_1639_v192	Listeria_monocytogenes_1639_v125	0.8982
Listeria_monocytogenes_1639_v191	Listeria_monocytogenes_1639_v125	0.9691
Listeria_monocytogenes_1639_v190	Listeria_monocytogenes_1639_v125	0.9645
Listeria_monocytogenes_1639_v18	Listeria_monocytogenes_1639_v125	0.9264
Listeria_monocytogenes_1639_v189	Listeria_monocytogenes_1639_v125	0.9637
Listeria_monocytogenes_1639_v188	Listeria_monocytogenes_1639_v125	0.9605
Listeria_monocytogenes_1639_v187	Listeria_monocytogenes_1639_v125	0.9641
Listeria_monocytogenes_1639_v186	Listeria_monocytogenes_1639_v125	0.9649
Listeria_monocytogenes_1639_v185	Listeria_monocytogenes_1639_v125	0.9645
Listeria_monocytogenes_1639_v184	Listeria_monocytogenes_1639_v125	0.9641
Listeria_monocytogenes_1639_v183	Listeria_monocytogenes_1639_v125	0.9645
Listeria_monocytogenes_1639_v182	Listeria_monocytogenes_1639_v125	0.9667
Listeria_monocytogenes_1639_v181	Listeria_monocytogenes_1639_v125	0.9186
Listeria_monocytogenes_1639_v180	Listeria_monocytogenes_1639_v125	0.9186
Listeria_monocytogenes_1639_v17	Listeria_monocytogenes_1639_v125	0.9264
Listeria_monocytogenes_1639_v179	Listeria_monocytogenes_1639_v125	0.9706
Listeria_monocytogenes_1639_v178	Listeria_monocytogenes_1639_v125	0.9668
Listeria_monocytogenes_1639_v177	Listeria_monocytogenes_1639_v125	0.9426
Listeria_monocytogenes_1639_v176	Listeria_monocytogenes_1639_v125	0.9423
Listeria_monocytogenes_1639_v175	Listeria_monocytogenes_1639_v125	0.9587
Listeria_monocytogenes_1639_v174	Listeria_monocytogenes_1639_v125	0.9676
Listeria_monocytogenes_1639_v173	Listeria_monocytogenes_1639_v125	0.9437
Listeria_monocytogenes_1639_v172	Listeria_monocytogenes_1639_v125	0.9333
Listeria_monocytogenes_1639_v171	Listeria_monocytogenes_1639_v125	0.9521
Listeria_monocytogenes_1639_v170	Listeria_monocytogenes_1639_v125	0.9594
Listeria_monocytogenes_1639_v16	Listeria_monocytogenes_1639_v125	0.9322

Listeria_monocytogenes_1639_v169	Listeria_monocytogenes_1639_v125	0.9598
Listeria_monocytogenes_1639_v168	Listeria_monocytogenes_1639_v125	0.9888
Listeria_monocytogenes_1639_v167	Listeria_monocytogenes_1639_v125	0.9724
Listeria_monocytogenes_1639_v166	Listeria_monocytogenes_1639_v125	0.9892
Listeria_monocytogenes_1639_v165	Listeria_monocytogenes_1639_v125	0.9594
Listeria_monocytogenes_1639_v164	Listeria_monocytogenes_1639_v125	0.9374
Listeria_monocytogenes_1639_v163	Listeria_monocytogenes_1639_v125	0.9459
Listeria_monocytogenes_1639_v162	Listeria_monocytogenes_1639_v125	0.9374
Listeria_monocytogenes_1639_v161	Listeria_monocytogenes_1639_v125	0.9366
Listeria_monocytogenes_1639_v160	Listeria_monocytogenes_1639_v125	0.9367
Listeria_monocytogenes_1639_v15	Listeria_monocytogenes_1639_v125	0.9122
Listeria_monocytogenes_1639_v159	Listeria_monocytogenes_1639_v125	0.9426
Listeria_monocytogenes_1639_v158	Listeria_monocytogenes_1639_v125	0.9363
Listeria_monocytogenes_1639_v157	Listeria_monocytogenes_1639_v125	0.9363
Listeria_monocytogenes_1639_v156	Listeria_monocytogenes_1639_v125	0.9472
Listeria_monocytogenes_1639_v155	Listeria_monocytogenes_1639_v125	0.9622
Listeria_monocytogenes_1639_v154	Listeria_monocytogenes_1639_v125	0.9531
Listeria_monocytogenes_1639_v153	Listeria_monocytogenes_1639_v125	0.9553
Listeria_monocytogenes_1639_v152	Listeria_monocytogenes_1639_v125	0.9429
Listeria_monocytogenes_1639_v151	Listeria_monocytogenes_1639_v125	0.9429
Listeria_monocytogenes_1639_v150	Listeria_monocytogenes_1639_v125	0.9553
Listeria_monocytogenes_1639_v14	Listeria_monocytogenes_1639_v125	0.9194
Listeria_monocytogenes_1639_v149	Listeria_monocytogenes_1639_v125	0.9539
Listeria_monocytogenes_1639_v148	Listeria_monocytogenes_1639_v125	0.9414
Listeria_monocytogenes_1639_v147	Listeria_monocytogenes_1639_v125	0.9291
Listeria_monocytogenes_1639_v146	Listeria_monocytogenes_1639_v125	0.96
Listeria_monocytogenes_1639_v145	Listeria_monocytogenes_1639_v125	0.962
Listeria_monocytogenes_1639_v144	Listeria_monocytogenes_1639_v125	0.9401
Listeria_monocytogenes_1639_v143	Listeria_monocytogenes_1639_v125	0.9635
Listeria_monocytogenes_1639_v142	Listeria_monocytogenes_1639_v125	0.9405
Listeria_monocytogenes_1639_v141	Listeria_monocytogenes_1639_v125	0.9462
Listeria_monocytogenes_1639_v140	Listeria_monocytogenes_1639_v125	0.9408
Listeria_monocytogenes_1639_v13	Listeria_monocytogenes_1639_v125	0.9126
Listeria_monocytogenes_1639_v139	Listeria_monocytogenes_1639_v125	0.9402
Listeria_monocytogenes_1639_v138	Listeria_monocytogenes_1639_v125	0.9432
Listeria_monocytogenes_1639_v137	Listeria_monocytogenes_1639_v125	0.9428
Listeria_monocytogenes_1639_v136	Listeria_monocytogenes_1639_v125	0.951
Listeria_monocytogenes_1639_v135	Listeria_monocytogenes_1639_v125	0.9193
Listeria_monocytogenes_1639_v134	Listeria_monocytogenes_1639_v125	0.9304
Listeria_monocytogenes_1639_v133	Listeria_monocytogenes_1639_v125	0.9638
Listeria_monocytogenes_1639_v132	Listeria_monocytogenes_1639_v125	0.9168
Listeria_monocytogenes_1639_v131	Listeria_monocytogenes_1639_v125	0.9363
Listeria_monocytogenes_1639_v130	Listeria_monocytogenes_1639_v125	0.9405
Listeria_monocytogenes_1639_v12	Listeria_monocytogenes_1639_v125	0.9275
Listeria_monocytogenes_1639_v129	Listeria_monocytogenes_1639_v125	0.9416
Listeria_monocytogenes_1639_v128	Listeria_monocytogenes_1639_v125	0.9418
Listeria_monocytogenes_1639_v127	Listeria_monocytogenes_1639_v125	0.9601
Listeria_monocytogenes_1639_v126	Listeria_monocytogenes_1639_v125	0.943
Listeria_monocytogenes_1639_v125	Listeria_monocytogenes_1639_v125	0.0679
Listeria_welshimeri_1643	Listeria_monocytogenes_1639_v126	0.8832
Listeria_welshimeri_serovar_6b_str_SLCC5334_386043	Listeria_monocytogenes_1639_v126	0.8825
Listeria_seeligeri_serovar_1_2b_str_SLCC3954_683837	Listeria_monocytogenes_1639_v126	0.8948
Listeria_seeligeri_FSL_S4-171_702452	Listeria_monocytogenes_1639_v126	0.83
Listeria_seeligeri_FSL_N1-067_702453	Listeria_monocytogenes_1639_v126	0.8358
Listeria_seeligeri_1640_v1	Listeria_monocytogenes_1639_v126	0.8977
Listeria_seeligeri_1640	Listeria_monocytogenes_1639_v126	0.8858
Listeria_monocytogenes_WSLC1042_1457188	Listeria_monocytogenes_1639_v126	0.9599
Listeria_monocytogenes_WSLC1001_1457187	Listeria_monocytogenes_1639_v126	0.9422
Listeria_monocytogenes_SLCC7179_879090	Listeria_monocytogenes_1639_v126	0.9485
Listeria_monocytogenes_SLCC5850_882096	Listeria_monocytogenes_1639_v126	0.9461
Listeria_monocytogenes_SLCC2755_932919	Listeria_monocytogenes_1639_v126	0.9538
Listeria_monocytogenes_SLCC2540_879089	Listeria_monocytogenes_1639_v126	0.9604
Listeria_monocytogenes_SLCC2479_882020	Listeria_monocytogenes_1639_v126	0.9392
Listeria_monocytogenes_SLCC2378_879088	Listeria_monocytogenes_1639_v126	0.961
Listeria_monocytogenes_SLCC2376_882097	Listeria_monocytogenes_1639_v126	0.9451
Listeria_monocytogenes_SLCC2372_932920	Listeria_monocytogenes_1639_v126	0.9318
Listeria_monocytogenes_serotype_4b_str_LL195_1230340	Listeria_monocytogenes_1639_v126	0.965
Listeria_monocytogenes_serotype_4b_str_LIS0087_1134669	Listeria_monocytogenes_1639_v126	0.9233
Listeria_monocytogenes_serotype_4b_str_F2365_265669	Listeria_monocytogenes_1639_v126	0.965
Listeria_monocytogenes_serotype_4b_str_CLIP_80459_568819	Listeria_monocytogenes_1639_v126	0.9661
Listeria_monocytogenes_serotype_4b_str_81-0861_1196161	Listeria_monocytogenes_1639_v126	0.9505
Listeria_monocytogenes_serotype_4b_str_81-0592_1196160	Listeria_monocytogenes_1639_v126	0.9516
Listeria_monocytogenes_serotype_4b_str_81-0558_1196159	Listeria_monocytogenes_1639_v126	0.9527
Listeria_monocytogenes_serotype_4b_str_10-0809_1196162	Listeria_monocytogenes_1639_v126	0.9519
Listeria_monocytogenes_serotype_4b_str_02-6680_1196175	Listeria_monocytogenes_1639_v126	0.9635
Listeria_monocytogenes_serotype_4b_str_02-6679_1196174	Listeria_monocytogenes_1639_v126	0.9635
Listeria_monocytogenes_serotype_4b_str_02-1792_1196173	Listeria_monocytogenes_1639_v126	0.9527
Listeria_monocytogenes_serotype_4b_str_02-1289_1196172	Listeria_monocytogenes_1639_v126	0.9527
Listeria_monocytogenes_serotype_4b_str_02-1103_1196171	Listeria_monocytogenes_1639_v126	0.9527
Listeria_monocytogenes_serotype_3c_str_10-5027_1196195	Listeria_monocytogenes_1639_v126	0.9388
Listeria_monocytogenes_serotype_1_2c_str_10-5026_1196194	Listeria_monocytogenes_1639_v126	0.9399
Listeria_monocytogenes_serotype_1_2c_str_10-5025_1196193	Listeria_monocytogenes_1639_v126	0.9399
Listeria_monocytogenes_serotype_1_2b_str_10-0811_1196164	Listeria_monocytogenes_1639_v126	0.9488
Listeria_monocytogenes_serotype_1_2b_str_10-0810_1196163	Listeria_monocytogenes_1639_v126	0.9488
Listeria_monocytogenes_serotype_1_2a_str_99-6370_1196179	Listeria_monocytogenes_1639_v126	0.9288
Listeria_monocytogenes_serotype_1_2a_str_98-2035_1196178	Listeria_monocytogenes_1639_v126	0.9288
Listeria_monocytogenes_serotype_1_2a_str_95-0093_1196177	Listeria_monocytogenes_1639_v126	0.9347
Listeria_monocytogenes_serotype_1_2a_str_88-0478_1196176	Listeria_monocytogenes_1639_v126	0.9339
Listeria_monocytogenes_serotype_1_2a_str_10-5024_1196192	Listeria_monocytogenes_1639_v126	0.9351
Listeria_monocytogenes_serotype_1_2a_str_10-4758_1196168	Listeria_monocytogenes_1639_v126	0.9477
Listeria_monocytogenes_serotype_1_2a_str_10-4754_1196167	Listeria_monocytogenes_1639_v126	0.9473

Listeria_monocytogenes_serotype_1_2a_str_10-1321_1196191	Listeria_monocytogenes_1639_v126	0.9351
Listeria_monocytogenes_serotype_1_2a_str_10-1047_1196190	Listeria_monocytogenes_1639_v126	0.9354
Listeria_monocytogenes_serotype_1_2a_str_10-1046_1196189	Listeria_monocytogenes_1639_v126	0.9354
Listeria_monocytogenes_serotype_1_2a_str_10-0934_1196170	Listeria_monocytogenes_1639_v126	0.9575
Listeria_monocytogenes_serotype_1_2a_str_10-0933_1196169	Listeria_monocytogenes_1639_v126	0.9575
Listeria_monocytogenes_serotype_1_2a_str_10-0815_1196188	Listeria_monocytogenes_1639_v126	0.9302
Listeria_monocytogenes_serotype_1_2a_str_10-0814_1196187	Listeria_monocytogenes_1639_v126	0.9347
Listeria_monocytogenes_serotype_1_2a_str_10-0813_1196166	Listeria_monocytogenes_1639_v126	0.9397
Listeria_monocytogenes_serotype_1_2a_str_10-0812_1196165	Listeria_monocytogenes_1639_v126	0.9397
Listeria_monocytogenes_serotype_1_2a_str_08-7669_1196186	Listeria_monocytogenes_1639_v126	0.9439
Listeria_monocytogenes_serotype_1_2a_str_08-7374_1196185	Listeria_monocytogenes_1639_v126	0.9351
Listeria_monocytogenes_serotype_1_2a_str_08-6997_1196184	Listeria_monocytogenes_1639_v126	0.9302
Listeria_monocytogenes_serotype_1_2a_str_08-6569_1196183	Listeria_monocytogenes_1639_v126	0.9302
Listeria_monocytogenes_serotype_1_2a_str_08-6056_1196182	Listeria_monocytogenes_1639_v126	0.9302
Listeria_monocytogenes_serotype_1_2a_str_04-5457_1196181	Listeria_monocytogenes_1639_v126	0.9351
Listeria_monocytogenes_serotype_1_2a_str_02-5993_1196180	Listeria_monocytogenes_1639_v126	0.9347
Listeria_monocytogenes_R479a_1437838	Listeria_monocytogenes_1639_v126	0.9333
Listeria_monocytogenes_N53-1_1234142	Listeria_monocytogenes_1639_v126	0.9002
Listeria_monocytogenes_M7_1030009	Listeria_monocytogenes_1639_v126	0.9063
Listeria_monocytogenes_LO28_393132	Listeria_monocytogenes_1639_v126	0.9381
Listeria_monocytogenes_L99_563174	Listeria_monocytogenes_1639_v126	0.907
Listeria_monocytogenes_L312_882094	Listeria_monocytogenes_1639_v126	0.9665
Listeria_monocytogenes_J1816_930781	Listeria_monocytogenes_1639_v126	0.9465
Listeria_monocytogenes_J1-220_930782	Listeria_monocytogenes_1639_v126	0.9425
Listeria_monocytogenes_J0161_393130	Listeria_monocytogenes_1639_v126	0.937
Listeria_monocytogenes_HCC23_552536	Listeria_monocytogenes_1639_v126	0.9063
Listeria_monocytogenes_FSL_R2-561_393126	Listeria_monocytogenes_1639_v126	0.9374
Listeria_monocytogenes_FSL_J1-208_393119	Listeria_monocytogenes_1639_v126	0.9155
Listeria_monocytogenes_FSL_J1-175_393118	Listeria_monocytogenes_1639_v126	0.9603
Listeria_monocytogenes_Finland_1998_393127	Listeria_monocytogenes_1639_v126	0.9567
Listeria_monocytogenes_EGD-e_169963_v1	Listeria_monocytogenes_1639_v126	0.952
Listeria_monocytogenes_EGD-e_169963	Listeria_monocytogenes_1639_v126	0.952
Listeria_monocytogenes_EGD_1334565	Listeria_monocytogenes_1639_v126	0.9468
Listeria_monocytogenes_CFSAN002349_1299895	Listeria_monocytogenes_1639_v126	0.9662
Listeria_monocytogenes_ATCC_19117_882095_v1	Listeria_monocytogenes_1639_v126	0.9609
Listeria_monocytogenes_ATCC_19117_882095	Listeria_monocytogenes_1639_v126	0.9609
Listeria_monocytogenes_6179_1288295	Listeria_monocytogenes_1639_v126	0.9211
Listeria_monocytogenes_1639_v9	Listeria_monocytogenes_1639_v126	0.9332
Listeria_monocytogenes_1639_v99	Listeria_monocytogenes_1639_v126	0.9469
Listeria_monocytogenes_1639_v98	Listeria_monocytogenes_1639_v126	0.9339
Listeria_monocytogenes_1639_v97	Listeria_monocytogenes_1639_v126	0.9544
Listeria_monocytogenes_1639_v96	Listeria_monocytogenes_1639_v126	0.9469
Listeria_monocytogenes_1639_v95	Listeria_monocytogenes_1639_v126	0.9465
Listeria_monocytogenes_1639_v94	Listeria_monocytogenes_1639_v126	0.9469
Listeria_monocytogenes_1639_v93	Listeria_monocytogenes_1639_v126	0.9339
Listeria_monocytogenes_1639_v92	Listeria_monocytogenes_1639_v126	0.9472
Listeria_monocytogenes_1639_v91	Listeria_monocytogenes_1639_v126	0.9483
Listeria_monocytogenes_1639_v90	Listeria_monocytogenes_1639_v126	0.9339
Listeria_monocytogenes_1639_v8	Listeria_monocytogenes_1639_v126	0.9229
Listeria_monocytogenes_1639_v89	Listeria_monocytogenes_1639_v126	0.9588
Listeria_monocytogenes_1639_v88	Listeria_monocytogenes_1639_v126	0.9405
Listeria_monocytogenes_1639_v87	Listeria_monocytogenes_1639_v126	0.9362
Listeria_monocytogenes_1639_v86	Listeria_monocytogenes_1639_v126	0.9355
Listeria_monocytogenes_1639_v85	Listeria_monocytogenes_1639_v126	0.9501
Listeria_monocytogenes_1639_v84	Listeria_monocytogenes_1639_v126	0.9423
Listeria_monocytogenes_1639_v83	Listeria_monocytogenes_1639_v126	0.9515
Listeria_monocytogenes_1639_v82	Listeria_monocytogenes_1639_v126	0.9511
Listeria_monocytogenes_1639_v81	Listeria_monocytogenes_1639_v126	0.9397
Listeria_monocytogenes_1639_v80	Listeria_monocytogenes_1639_v126	0.9533
Listeria_monocytogenes_1639_v7	Listeria_monocytogenes_1639_v126	0.9229
Listeria_monocytogenes_1639_v79	Listeria_monocytogenes_1639_v126	0.938
Listeria_monocytogenes_1639_v78	Listeria_monocytogenes_1639_v126	0.939
Listeria_monocytogenes_1639_v77	Listeria_monocytogenes_1639_v126	0.9449
Listeria_monocytogenes_1639_v76	Listeria_monocytogenes_1639_v126	0.932
Listeria_monocytogenes_1639_v75	Listeria_monocytogenes_1639_v126	0.907
Listeria_monocytogenes_1639_v74	Listeria_monocytogenes_1639_v126	0.9329
Listeria_monocytogenes_1639_v73	Listeria_monocytogenes_1639_v126	0.934
Listeria_monocytogenes_1639_v72	Listeria_monocytogenes_1639_v126	0.9495
Listeria_monocytogenes_1639_v71	Listeria_monocytogenes_1639_v126	0.921
Listeria_monocytogenes_1639_v70	Listeria_monocytogenes_1639_v126	0.9356
Listeria_monocytogenes_1639_v6	Listeria_monocytogenes_1639_v126	0.9222
Listeria_monocytogenes_1639_v69	Listeria_monocytogenes_1639_v126	0.9519
Listeria_monocytogenes_1639_v68	Listeria_monocytogenes_1639_v126	0.9439
Listeria_monocytogenes_1639_v67	Listeria_monocytogenes_1639_v126	0.9526
Listeria_monocytogenes_1639_v66	Listeria_monocytogenes_1639_v126	0.9428
Listeria_monocytogenes_1639_v65	Listeria_monocytogenes_1639_v126	0.9375
Listeria_monocytogenes_1639_v64	Listeria_monocytogenes_1639_v126	0.9361
Listeria_monocytogenes_1639_v63	Listeria_monocytogenes_1639_v126	0.9492
Listeria_monocytogenes_1639_v62	Listeria_monocytogenes_1639_v126	0.9366
Listeria_monocytogenes_1639_v61	Listeria_monocytogenes_1639_v126	0.9379
Listeria_monocytogenes_1639_v60	Listeria_monocytogenes_1639_v126	0.9378
Listeria_monocytogenes_1639_v5	Listeria_monocytogenes_1639_v126	0.9131
Listeria_monocytogenes_1639_v59	Listeria_monocytogenes_1639_v126	0.9514
Listeria_monocytogenes_1639_v58	Listeria_monocytogenes_1639_v126	0.9564
Listeria_monocytogenes_1639_v57	Listeria_monocytogenes_1639_v126	0.9426
Listeria_monocytogenes_1639_v56	Listeria_monocytogenes_1639_v126	0.9511
Listeria_monocytogenes_1639_v55	Listeria_monocytogenes_1639_v126	0.9395
Listeria_monocytogenes_1639_v54	Listeria_monocytogenes_1639_v126	0.9252
Listeria_monocytogenes_1639_v53	Listeria_monocytogenes_1639_v126	0.9393
Listeria_monocytogenes_1639_v52	Listeria_monocytogenes_1639_v126	0.9463
Listeria_monocytogenes_1639_v51	Listeria_monocytogenes_1639_v126	0.9461

Listeria_monocytogenes_1639_v50	Listeria_monocytogenes_1639_v126	0.9339
Listeria_monocytogenes_1639_v4	Listeria_monocytogenes_1639_v126	0.9265
Listeria_monocytogenes_1639_v49	Listeria_monocytogenes_1639_v126	0.9546
Listeria_monocytogenes_1639_v48	Listeria_monocytogenes_1639_v126	0.9339
Listeria_monocytogenes_1639_v47	Listeria_monocytogenes_1639_v126	0.9347
Listeria_monocytogenes_1639_v46	Listeria_monocytogenes_1639_v126	0.9428
Listeria_monocytogenes_1639_v45	Listeria_monocytogenes_1639_v126	0.925
Listeria_monocytogenes_1639_v44	Listeria_monocytogenes_1639_v126	0.9351
Listeria_monocytogenes_1639_v43	Listeria_monocytogenes_1639_v126	0.9351
Listeria_monocytogenes_1639_v42	Listeria_monocytogenes_1639_v126	0.9243
Listeria_monocytogenes_1639_v41	Listeria_monocytogenes_1639_v126	0.91
Listeria_monocytogenes_1639_v40	Listeria_monocytogenes_1639_v126	0.9381
Listeria_monocytogenes_1639_v3	Listeria_monocytogenes_1639_v126	0.9265
Listeria_monocytogenes_1639_v39	Listeria_monocytogenes_1639_v126	0.9484
Listeria_monocytogenes_1639_v38	Listeria_monocytogenes_1639_v126	0.9364
Listeria_monocytogenes_1639_v37	Listeria_monocytogenes_1639_v126	0.9308
Listeria_monocytogenes_1639_v36	Listeria_monocytogenes_1639_v126	0.9305
Listeria_monocytogenes_1639_v35	Listeria_monocytogenes_1639_v126	0.9388
Listeria_monocytogenes_1639_v34	Listeria_monocytogenes_1639_v126	0.9357
Listeria_monocytogenes_1639_v33	Listeria_monocytogenes_1639_v126	0.9488
Listeria_monocytogenes_1639_v32	Listeria_monocytogenes_1639_v126	0.9293
Listeria_monocytogenes_1639_v31	Listeria_monocytogenes_1639_v126	0.9361
Listeria_monocytogenes_1639_v30	Listeria_monocytogenes_1639_v126	0.9495
Listeria_monocytogenes_1639_v2	Listeria_monocytogenes_1639_v126	0.9142
Listeria_monocytogenes_1639_v29	Listeria_monocytogenes_1639_v126	0.9249
Listeria_monocytogenes_1639_v28	Listeria_monocytogenes_1639_v126	0.929
Listeria_monocytogenes_1639_v27	Listeria_monocytogenes_1639_v126	0.9197
Listeria_monocytogenes_1639_v26	Listeria_monocytogenes_1639_v126	0.9197
Listeria_monocytogenes_1639_v25	Listeria_monocytogenes_1639_v126	0.9201
Listeria_monocytogenes_1639_v24	Listeria_monocytogenes_1639_v126	0.9433
Listeria_monocytogenes_1639_v23	Listeria_monocytogenes_1639_v126	0.9433
Listeria_monocytogenes_1639_v22	Listeria_monocytogenes_1639_v126	0.9503
Listeria_monocytogenes_1639_v21	Listeria_monocytogenes_1639_v126	0.9426
Listeria_monocytogenes_1639_v20	Listeria_monocytogenes_1639_v126	0.9447
Listeria_monocytogenes_1639_v1	Listeria_monocytogenes_1639_v126	0.9137
Listeria_monocytogenes_1639_v19	Listeria_monocytogenes_1639_v126	0.9443
Listeria_monocytogenes_1639_v198	Listeria_monocytogenes_1639_v126	0.9025
Listeria_monocytogenes_1639_v197	Listeria_monocytogenes_1639_v126	0.939
Listeria_monocytogenes_1639_v196	Listeria_monocytogenes_1639_v126	0.9296
Listeria_monocytogenes_1639_v195	Listeria_monocytogenes_1639_v126	0.9454
Listeria_monocytogenes_1639_v194	Listeria_monocytogenes_1639_v126	0.9455
Listeria_monocytogenes_1639_v193	Listeria_monocytogenes_1639_v126	0.9459
Listeria_monocytogenes_1639_v192	Listeria_monocytogenes_1639_v126	0.9062
Listeria_monocytogenes_1639_v191	Listeria_monocytogenes_1639_v126	0.9554
Listeria_monocytogenes_1639_v190	Listeria_monocytogenes_1639_v126	0.9574
Listeria_monocytogenes_1639_v18	Listeria_monocytogenes_1639_v126	0.9381
Listeria_monocytogenes_1639_v189	Listeria_monocytogenes_1639_v126	0.9566
Listeria_monocytogenes_1639_v188	Listeria_monocytogenes_1639_v126	0.955
Listeria_monocytogenes_1639_v187	Listeria_monocytogenes_1639_v126	0.957
Listeria_monocytogenes_1639_v186	Listeria_monocytogenes_1639_v126	0.9578
Listeria_monocytogenes_1639_v185	Listeria_monocytogenes_1639_v126	0.9574
Listeria_monocytogenes_1639_v184	Listeria_monocytogenes_1639_v126	0.9566
Listeria_monocytogenes_1639_v183	Listeria_monocytogenes_1639_v126	0.9543
Listeria_monocytogenes_1639_v182	Listeria_monocytogenes_1639_v126	0.9565
Listeria_monocytogenes_1639_v181	Listeria_monocytogenes_1639_v126	0.9256
Listeria_monocytogenes_1639_v180	Listeria_monocytogenes_1639_v126	0.9256
Listeria_monocytogenes_1639_v17	Listeria_monocytogenes_1639_v126	0.9381
Listeria_monocytogenes_1639_v179	Listeria_monocytogenes_1639_v126	0.9413
Listeria_monocytogenes_1639_v178	Listeria_monocytogenes_1639_v126	0.9585
Listeria_monocytogenes_1639_v177	Listeria_monocytogenes_1639_v126	0.9639
Listeria_monocytogenes_1639_v176	Listeria_monocytogenes_1639_v126	0.9425
Listeria_monocytogenes_1639_v175	Listeria_monocytogenes_1639_v126	0.9482
Listeria_monocytogenes_1639_v174	Listeria_monocytogenes_1639_v126	0.9605
Listeria_monocytogenes_1639_v173	Listeria_monocytogenes_1639_v126	0.9752
Listeria_monocytogenes_1639_v172	Listeria_monocytogenes_1639_v126	0.9384
Listeria_monocytogenes_1639_v171	Listeria_monocytogenes_1639_v126	0.9424
Listeria_monocytogenes_1639_v170	Listeria_monocytogenes_1639_v126	0.9508
Listeria_monocytogenes_1639_v16	Listeria_monocytogenes_1639_v126	0.9209
Listeria_monocytogenes_1639_v169	Listeria_monocytogenes_1639_v126	0.9516
Listeria_monocytogenes_1639_v168	Listeria_monocytogenes_1639_v126	0.9502
Listeria_monocytogenes_1639_v167	Listeria_monocytogenes_1639_v126	0.9419
Listeria_monocytogenes_1639_v166	Listeria_monocytogenes_1639_v126	0.9505
Listeria_monocytogenes_1639_v165	Listeria_monocytogenes_1639_v126	0.9512
Listeria_monocytogenes_1639_v164	Listeria_monocytogenes_1639_v126	0.9641
Listeria_monocytogenes_1639_v163	Listeria_monocytogenes_1639_v126	0.9715
Listeria_monocytogenes_1639_v162	Listeria_monocytogenes_1639_v126	0.9641
Listeria_monocytogenes_1639_v161	Listeria_monocytogenes_1639_v126	0.9653
Listeria_monocytogenes_1639_v160	Listeria_monocytogenes_1639_v126	0.9653
Listeria_monocytogenes_1639_v15	Listeria_monocytogenes_1639_v126	0.933
Listeria_monocytogenes_1639_v159	Listeria_monocytogenes_1639_v126	0.9643
Listeria_monocytogenes_1639_v158	Listeria_monocytogenes_1639_v126	0.9653
Listeria_monocytogenes_1639_v157	Listeria_monocytogenes_1639_v126	0.965
Listeria_monocytogenes_1639_v156	Listeria_monocytogenes_1639_v126	0.935
Listeria_monocytogenes_1639_v155	Listeria_monocytogenes_1639_v126	0.946
Listeria_monocytogenes_1639_v154	Listeria_monocytogenes_1639_v126	0.9521
Listeria_monocytogenes_1639_v153	Listeria_monocytogenes_1639_v126	0.9452
Listeria_monocytogenes_1639_v152	Listeria_monocytogenes_1639_v126	0.9646
Listeria_monocytogenes_1639_v151	Listeria_monocytogenes_1639_v126	0.9646
Listeria_monocytogenes_1639_v150	Listeria_monocytogenes_1639_v126	0.9483
Listeria_monocytogenes_1639_v14	Listeria_monocytogenes_1639_v126	0.9396
Listeria_monocytogenes_1639_v149	Listeria_monocytogenes_1639_v126	0.9468

Listeria_monocytogenes_1639_v148	Listeria_monocytogenes_1639_v126	0.9639
Listeria_monocytogenes_1639_v147	Listeria_monocytogenes_1639_v126	0.9493
Listeria_monocytogenes_1639_v146	Listeria_monocytogenes_1639_v126	0.9583
Listeria_monocytogenes_1639_v145	Listeria_monocytogenes_1639_v126	0.9503
Listeria_monocytogenes_1639_v144	Listeria_monocytogenes_1639_v126	0.9657
Listeria_monocytogenes_1639_v143	Listeria_monocytogenes_1639_v126	0.9549
Listeria_monocytogenes_1639_v142	Listeria_monocytogenes_1639_v126	0.9661
Listeria_monocytogenes_1639_v141	Listeria_monocytogenes_1639_v126	0.9931
Listeria_monocytogenes_1639_v140	Listeria_monocytogenes_1639_v126	0.9665
Listeria_monocytogenes_1639_v13	Listeria_monocytogenes_1639_v126	0.9334
Listeria_monocytogenes_1639_v139	Listeria_monocytogenes_1639_v126	0.9615
Listeria_monocytogenes_1639_v138	Listeria_monocytogenes_1639_v126	0.9688
Listeria_monocytogenes_1639_v137	Listeria_monocytogenes_1639_v126	0.9688
Listeria_monocytogenes_1639_v136	Listeria_monocytogenes_1639_v126	0.947
Listeria_monocytogenes_1639_v135	Listeria_monocytogenes_1639_v126	0.9179
Listeria_monocytogenes_1639_v134	Listeria_monocytogenes_1639_v126	0.9609
Listeria_monocytogenes_1639_v133	Listeria_monocytogenes_1639_v126	0.946
Listeria_monocytogenes_1639_v132	Listeria_monocytogenes_1639_v126	0.9206
Listeria_monocytogenes_1639_v131	Listeria_monocytogenes_1639_v126	0.9543
Listeria_monocytogenes_1639_v130	Listeria_monocytogenes_1639_v126	0.9388
Listeria_monocytogenes_1639_v12	Listeria_monocytogenes_1639_v126	0.9188
Listeria_monocytogenes_1639_v129	Listeria_monocytogenes_1639_v126	0.9765
Listeria_monocytogenes_1639_v128	Listeria_monocytogenes_1639_v126	0.9969
Listeria_monocytogenes_1639_v127	Listeria_monocytogenes_1639_v126	0.9447
Listeria_monocytogenes_1639_v126	Listeria_monocytogenes_1639_v126	0.0672
Listeria_welshimeri_1643	Listeria_monocytogenes_1639_v127	0.8715
Listeria_welshimeri_serovar_6b_str_SLCC5334_386043	Listeria_monocytogenes_1639_v127	0.8712
Listeria_seeligeri_serovar_1_2b_str_SLCC3954_683837	Listeria_monocytogenes_1639_v127	0.8852
Listeria_seeligeri_FSL_S4-171_702452	Listeria_monocytogenes_1639_v127	0.82
Listeria_seeligeri_FSL_N1-067_702453	Listeria_monocytogenes_1639_v127	0.8314
Listeria_seeligeri_1640_v1	Listeria_monocytogenes_1639_v127	0.8817
Listeria_seeligeri_1640	Listeria_monocytogenes_1639_v127	0.887
Listeria_monocytogenes_WSLC1042_1457188	Listeria_monocytogenes_1639_v127	0.9306
Listeria_monocytogenes_WSLC1001_1457187	Listeria_monocytogenes_1639_v127	0.9502
Listeria_monocytogenes_SLCC7179_879090	Listeria_monocytogenes_1639_v127	0.9676
Listeria_monocytogenes_SLCC5850_882096	Listeria_monocytogenes_1639_v127	0.9534
Listeria_monocytogenes_SLCC2755_932919	Listeria_monocytogenes_1639_v127	0.9331
Listeria_monocytogenes_SLCC2540_879089	Listeria_monocytogenes_1639_v127	0.9358
Listeria_monocytogenes_SLCC2479_882020	Listeria_monocytogenes_1639_v127	0.9569
Listeria_monocytogenes_SLCC2378_879088	Listeria_monocytogenes_1639_v127	0.9317
Listeria_monocytogenes_SLCC2376_882097	Listeria_monocytogenes_1639_v127	0.9393
Listeria_monocytogenes_SLCC2372_932920	Listeria_monocytogenes_1639_v127	0.9498
Listeria_monocytogenes_serotype_4b_str_LL195_1230340	Listeria_monocytogenes_1639_v127	0.9374
Listeria_monocytogenes_serotype_4b_str_LIS0087_1134669	Listeria_monocytogenes_1639_v127	0.9414
Listeria_monocytogenes_serotype_4b_str_F2365_265669	Listeria_monocytogenes_1639_v127	0.9374
Listeria_monocytogenes_serotype_4b_str_CLIP_80459_568819	Listeria_monocytogenes_1639_v127	0.9434
Listeria_monocytogenes_serotype_4b_str_81-0861_1196161	Listeria_monocytogenes_1639_v127	0.9358
Listeria_monocytogenes_serotype_4b_str_81-0592_1196160	Listeria_monocytogenes_1639_v127	0.9369
Listeria_monocytogenes_serotype_4b_str_81-0558_1196159	Listeria_monocytogenes_1639_v127	0.9372
Listeria_monocytogenes_serotype_4b_str_10-0809_1196162	Listeria_monocytogenes_1639_v127	0.9369
Listeria_monocytogenes_serotype_4b_str_02-6680_1196175	Listeria_monocytogenes_1639_v127	0.9385
Listeria_monocytogenes_serotype_4b_str_02-6679_1196174	Listeria_monocytogenes_1639_v127	0.9385
Listeria_monocytogenes_serotype_4b_str_02-1792_1196173	Listeria_monocytogenes_1639_v127	0.9373
Listeria_monocytogenes_serotype_4b_str_02-1289_1196172	Listeria_monocytogenes_1639_v127	0.9373
Listeria_monocytogenes_serotype_4b_str_02-1103_1196171	Listeria_monocytogenes_1639_v127	0.9373
Listeria_monocytogenes_serotype_3c_str_10-5027_1196195	Listeria_monocytogenes_1639_v127	0.9565
Listeria_monocytogenes_serotype_1_2c_str_10-5026_1196194	Listeria_monocytogenes_1639_v127	0.9573
Listeria_monocytogenes_serotype_1_2c_str_10-5025_1196193	Listeria_monocytogenes_1639_v127	0.9573
Listeria_monocytogenes_serotype_1_2b_str_10-0811_1196164	Listeria_monocytogenes_1639_v127	0.9417
Listeria_monocytogenes_serotype_1_2b_str_10-0810_1196163	Listeria_monocytogenes_1639_v127	0.9417
Listeria_monocytogenes_serotype_1_2a_str_99-6370_1196179	Listeria_monocytogenes_1639_v127	0.9367
Listeria_monocytogenes_serotype_1_2a_str_98-2035_1196178	Listeria_monocytogenes_1639_v127	0.9367
Listeria_monocytogenes_serotype_1_2a_str_95-0093_1196177	Listeria_monocytogenes_1639_v127	0.9415
Listeria_monocytogenes_serotype_1_2a_str_88-0478_1196176	Listeria_monocytogenes_1639_v127	0.9411
Listeria_monocytogenes_serotype_1_2a_str_10-5024_1196192	Listeria_monocytogenes_1639_v127	0.9415
Listeria_monocytogenes_serotype_1_2a_str_10-4758_1196168	Listeria_monocytogenes_1639_v127	0.9576
Listeria_monocytogenes_serotype_1_2a_str_10-4754_1196167	Listeria_monocytogenes_1639_v127	0.958
Listeria_monocytogenes_serotype_1_2a_str_10-1321_1196191	Listeria_monocytogenes_1639_v127	0.9415
Listeria_monocytogenes_serotype_1_2a_str_10-1047_1196190	Listeria_monocytogenes_1639_v127	0.9422
Listeria_monocytogenes_serotype_1_2a_str_10-1046_1196189	Listeria_monocytogenes_1639_v127	0.9422
Listeria_monocytogenes_serotype_1_2a_str_10-0934_1196170	Listeria_monocytogenes_1639_v127	0.9662
Listeria_monocytogenes_serotype_1_2a_str_10-0933_1196169	Listeria_monocytogenes_1639_v127	0.9662
Listeria_monocytogenes_serotype_1_2a_str_10-0815_1196188	Listeria_monocytogenes_1639_v127	0.9374
Listeria_monocytogenes_serotype_1_2a_str_10-0814_1196187	Listeria_monocytogenes_1639_v127	0.9419
Listeria_monocytogenes_serotype_1_2a_str_10-0813_1196166	Listeria_monocytogenes_1639_v127	0.962
Listeria_monocytogenes_serotype_1_2a_str_10-0812_1196165	Listeria_monocytogenes_1639_v127	0.962
Listeria_monocytogenes_serotype_1_2a_str_08-7669_1196186	Listeria_monocytogenes_1639_v127	0.9507
Listeria_monocytogenes_serotype_1_2a_str_08-7374_1196185	Listeria_monocytogenes_1639_v127	0.9415
Listeria_monocytogenes_serotype_1_2a_str_08-6997_1196184	Listeria_monocytogenes_1639_v127	0.9374
Listeria_monocytogenes_serotype_1_2a_str_08-6569_1196183	Listeria_monocytogenes_1639_v127	0.9374
Listeria_monocytogenes_serotype_1_2a_str_08-6056_1196182	Listeria_monocytogenes_1639_v127	0.9374
Listeria_monocytogenes_serotype_1_2a_str_04-5457_1196181	Listeria_monocytogenes_1639_v127	0.9415
Listeria_monocytogenes_serotype_1_2a_str_02-5993_1196180	Listeria_monocytogenes_1639_v127	0.9415
Listeria_monocytogenes_R479a_1437838	Listeria_monocytogenes_1639_v127	0.9393
Listeria_monocytogenes_N53-1_1234142	Listeria_monocytogenes_1639_v127	0.917
Listeria_monocytogenes_M7_1030009	Listeria_monocytogenes_1639_v127	0.9171
Listeria_monocytogenes_LO28_393132	Listeria_monocytogenes_1639_v127	0.9633
Listeria_monocytogenes_L99_563174	Listeria_monocytogenes_1639_v127	0.9174
Listeria_monocytogenes_L312_882094	Listeria_monocytogenes_1639_v127	0.9434
Listeria_monocytogenes_J1816_930781	Listeria_monocytogenes_1639_v127	0.9297
Listeria_monocytogenes_J1-220_930782	Listeria_monocytogenes_1639_v127	0.9296

Listeria_monocytogenes_J0161_393130	Listeria_monocytogenes_1639_v127	0.9671
Listeria_monocytogenes_HCC23_552536	Listeria_monocytogenes_1639_v127	0.9171
Listeria_monocytogenes_FSL_R2-561_393126	Listeria_monocytogenes_1639_v127	0.9547
Listeria_monocytogenes_FSL_J1-208_393119	Listeria_monocytogenes_1639_v127	0.9044
Listeria_monocytogenes_FSL_J1-175_393118	Listeria_monocytogenes_1639_v127	0.9412
Listeria_monocytogenes_Finland_1998_393127	Listeria_monocytogenes_1639_v127	0.9639
Listeria_monocytogenes_EGD-e_169963_v1	Listeria_monocytogenes_1639_v127	0.9769
Listeria_monocytogenes_EGD-e_169963	Listeria_monocytogenes_1639_v127	0.9773
Listeria_monocytogenes_EGD_1334565	Listeria_monocytogenes_1639_v127	0.9541
Listeria_monocytogenes_CFSAN002349_1299895	Listeria_monocytogenes_1639_v127	0.9421
Listeria_monocytogenes_ATCC_19117_882095_v1	Listeria_monocytogenes_1639_v127	0.9353
Listeria_monocytogenes_ATCC_19117_882095	Listeria_monocytogenes_1639_v127	0.9349
Listeria_monocytogenes_6179_1288295	Listeria_monocytogenes_1639_v127	0.9513
Listeria_monocytogenes_1639_v9	Listeria_monocytogenes_1639_v127	0.9242
Listeria_monocytogenes_1639_v99	Listeria_monocytogenes_1639_v127	0.9301
Listeria_monocytogenes_1639_v98	Listeria_monocytogenes_1639_v127	0.9576
Listeria_monocytogenes_1639_v97	Listeria_monocytogenes_1639_v127	0.934
Listeria_monocytogenes_1639_v96	Listeria_monocytogenes_1639_v127	0.9301
Listeria_monocytogenes_1639_v95	Listeria_monocytogenes_1639_v127	0.9301
Listeria_monocytogenes_1639_v94	Listeria_monocytogenes_1639_v127	0.9305
Listeria_monocytogenes_1639_v93	Listeria_monocytogenes_1639_v127	0.9445
Listeria_monocytogenes_1639_v92	Listeria_monocytogenes_1639_v127	0.9297
Listeria_monocytogenes_1639_v91	Listeria_monocytogenes_1639_v127	0.9348
Listeria_monocytogenes_1639_v90	Listeria_monocytogenes_1639_v127	0.9576
Listeria_monocytogenes_1639_v8	Listeria_monocytogenes_1639_v127	0.9316
Listeria_monocytogenes_1639_v89	Listeria_monocytogenes_1639_v127	0.9428
Listeria_monocytogenes_1639_v88	Listeria_monocytogenes_1639_v127	0.953
Listeria_monocytogenes_1639_v87	Listeria_monocytogenes_1639_v127	0.9532
Listeria_monocytogenes_1639_v86	Listeria_monocytogenes_1639_v127	0.9532
Listeria_monocytogenes_1639_v85	Listeria_monocytogenes_1639_v127	0.9355
Listeria_monocytogenes_1639_v84	Listeria_monocytogenes_1639_v127	0.954
Listeria_monocytogenes_1639_v83	Listeria_monocytogenes_1639_v127	0.9375
Listeria_monocytogenes_1639_v82	Listeria_monocytogenes_1639_v127	0.9368
Listeria_monocytogenes_1639_v81	Listeria_monocytogenes_1639_v127	0.9508
Listeria_monocytogenes_1639_v80	Listeria_monocytogenes_1639_v127	0.9439
Listeria_monocytogenes_1639_v7	Listeria_monocytogenes_1639_v127	0.9316
Listeria_monocytogenes_1639_v79	Listeria_monocytogenes_1639_v127	0.9497
Listeria_monocytogenes_1639_v78	Listeria_monocytogenes_1639_v127	0.95
Listeria_monocytogenes_1639_v77	Listeria_monocytogenes_1639_v127	0.9521
Listeria_monocytogenes_1639_v76	Listeria_monocytogenes_1639_v127	0.9459
Listeria_monocytogenes_1639_v75	Listeria_monocytogenes_1639_v127	0.9174
Listeria_monocytogenes_1639_v74	Listeria_monocytogenes_1639_v127	0.951
Listeria_monocytogenes_1639_v73	Listeria_monocytogenes_1639_v127	0.9575
Listeria_monocytogenes_1639_v72	Listeria_monocytogenes_1639_v127	0.9341
Listeria_monocytogenes_1639_v71	Listeria_monocytogenes_1639_v127	0.9357
Listeria_monocytogenes_1639_v70	Listeria_monocytogenes_1639_v127	0.9537
Listeria_monocytogenes_1639_v6	Listeria_monocytogenes_1639_v127	0.9312
Listeria_monocytogenes_1639_v69	Listeria_monocytogenes_1639_v127	0.9369
Listeria_monocytogenes_1639_v68	Listeria_monocytogenes_1639_v127	0.9673
Listeria_monocytogenes_1639_v67	Listeria_monocytogenes_1639_v127	0.938
Listeria_monocytogenes_1639_v66	Listeria_monocytogenes_1639_v127	0.9607
Listeria_monocytogenes_1639_v65	Listeria_monocytogenes_1639_v127	0.9526
Listeria_monocytogenes_1639_v64	Listeria_monocytogenes_1639_v127	0.9437
Listeria_monocytogenes_1639_v63	Listeria_monocytogenes_1639_v127	0.9338
Listeria_monocytogenes_1639_v62	Listeria_monocytogenes_1639_v127	0.9487
Listeria_monocytogenes_1639_v61	Listeria_monocytogenes_1639_v127	0.9451
Listeria_monocytogenes_1639_v60	Listeria_monocytogenes_1639_v127	0.9616
Listeria_monocytogenes_1639_v5	Listeria_monocytogenes_1639_v127	0.9065
Listeria_monocytogenes_1639_v59	Listeria_monocytogenes_1639_v127	0.9277
Listeria_monocytogenes_1639_v58	Listeria_monocytogenes_1639_v127	0.9394
Listeria_monocytogenes_1639_v57	Listeria_monocytogenes_1639_v127	0.9252
Listeria_monocytogenes_1639_v56	Listeria_monocytogenes_1639_v127	0.933
Listeria_monocytogenes_1639_v55	Listeria_monocytogenes_1639_v127	0.9292
Listeria_monocytogenes_1639_v54	Listeria_monocytogenes_1639_v127	0.9219
Listeria_monocytogenes_1639_v53	Listeria_monocytogenes_1639_v127	0.9447
Listeria_monocytogenes_1639_v52	Listeria_monocytogenes_1639_v127	0.9355
Listeria_monocytogenes_1639_v51	Listeria_monocytogenes_1639_v127	0.9338
Listeria_monocytogenes_1639_v50	Listeria_monocytogenes_1639_v127	0.9404
Listeria_monocytogenes_1639_v4	Listeria_monocytogenes_1639_v127	0.9194
Listeria_monocytogenes_1639_v49	Listeria_monocytogenes_1639_v127	0.923
Listeria_monocytogenes_1639_v48	Listeria_monocytogenes_1639_v127	0.9523
Listeria_monocytogenes_1639_v47	Listeria_monocytogenes_1639_v127	0.9415
Listeria_monocytogenes_1639_v46	Listeria_monocytogenes_1639_v127	0.9564
Listeria_monocytogenes_1639_v45	Listeria_monocytogenes_1639_v127	0.9322
Listeria_monocytogenes_1639_v44	Listeria_monocytogenes_1639_v127	0.9415
Listeria_monocytogenes_1639_v43	Listeria_monocytogenes_1639_v127	0.9415
Listeria_monocytogenes_1639_v42	Listeria_monocytogenes_1639_v127	0.9393
Listeria_monocytogenes_1639_v41	Listeria_monocytogenes_1639_v127	0.9312
Listeria_monocytogenes_1639_v40	Listeria_monocytogenes_1639_v127	0.9532
Listeria_monocytogenes_1639_v3	Listeria_monocytogenes_1639_v127	0.9197
Listeria_monocytogenes_1639_v39	Listeria_monocytogenes_1639_v127	0.9349
Listeria_monocytogenes_1639_v38	Listeria_monocytogenes_1639_v127	0.9485
Listeria_monocytogenes_1639_v37	Listeria_monocytogenes_1639_v127	0.9439
Listeria_monocytogenes_1639_v36	Listeria_monocytogenes_1639_v127	0.9388
Listeria_monocytogenes_1639_v35	Listeria_monocytogenes_1639_v127	0.9535
Listeria_monocytogenes_1639_v34	Listeria_monocytogenes_1639_v127	0.953
Listeria_monocytogenes_1639_v33	Listeria_monocytogenes_1639_v127	0.9417
Listeria_monocytogenes_1639_v32	Listeria_monocytogenes_1639_v127	0.9536
Listeria_monocytogenes_1639_v31	Listeria_monocytogenes_1639_v127	0.9534
Listeria_monocytogenes_1639_v30	Listeria_monocytogenes_1639_v127	0.9338
Listeria_monocytogenes_1639_v2	Listeria_monocytogenes_1639_v127	0.9091

Listeria_monocytogenes_1639_v29	Listeria_monocytogenes_1639_v127	0.9435
Listeria_monocytogenes_1639_v28	Listeria_monocytogenes_1639_v127	0.9484
Listeria_monocytogenes_1639_v27	Listeria_monocytogenes_1639_v127	0.9442
Listeria_monocytogenes_1639_v26	Listeria_monocytogenes_1639_v127	0.945
Listeria_monocytogenes_1639_v25	Listeria_monocytogenes_1639_v127	0.9446
Listeria_monocytogenes_1639_v24	Listeria_monocytogenes_1639_v127	0.9299
Listeria_monocytogenes_1639_v23	Listeria_monocytogenes_1639_v127	0.9292
Listeria_monocytogenes_1639_v22	Listeria_monocytogenes_1639_v127	0.9443
Listeria_monocytogenes_1639_v21	Listeria_monocytogenes_1639_v127	0.9303
Listeria_monocytogenes_1639_v20	Listeria_monocytogenes_1639_v127	0.9298
Listeria_monocytogenes_1639_v1	Listeria_monocytogenes_1639_v127	0.9447
Listeria_monocytogenes_1639_v19	Listeria_monocytogenes_1639_v127	0.9295
Listeria_monocytogenes_1639_v198	Listeria_monocytogenes_1639_v127	0.9184
Listeria_monocytogenes_1639_v197	Listeria_monocytogenes_1639_v127	0.9634
Listeria_monocytogenes_1639_v196	Listeria_monocytogenes_1639_v127	0.9371
Listeria_monocytogenes_1639_v195	Listeria_monocytogenes_1639_v127	0.9412
Listeria_monocytogenes_1639_v194	Listeria_monocytogenes_1639_v127	0.9394
Listeria_monocytogenes_1639_v193	Listeria_monocytogenes_1639_v127	0.9397
Listeria_monocytogenes_1639_v192	Listeria_monocytogenes_1639_v127	0.8994
Listeria_monocytogenes_1639_v191	Listeria_monocytogenes_1639_v127	0.9677
Listeria_monocytogenes_1639_v190	Listeria_monocytogenes_1639_v127	0.9643
Listeria_monocytogenes_1639_v18	Listeria_monocytogenes_1639_v127	0.9271
Listeria_monocytogenes_1639_v189	Listeria_monocytogenes_1639_v127	0.9635
Listeria_monocytogenes_1639_v188	Listeria_monocytogenes_1639_v127	0.9677
Listeria_monocytogenes_1639_v187	Listeria_monocytogenes_1639_v127	0.9642
Listeria_monocytogenes_1639_v186	Listeria_monocytogenes_1639_v127	0.9646
Listeria_monocytogenes_1639_v185	Listeria_monocytogenes_1639_v127	0.9646
Listeria_monocytogenes_1639_v184	Listeria_monocytogenes_1639_v127	0.9639
Listeria_monocytogenes_1639_v183	Listeria_monocytogenes_1639_v127	0.9673
Listeria_monocytogenes_1639_v182	Listeria_monocytogenes_1639_v127	0.9696
Listeria_monocytogenes_1639_v181	Listeria_monocytogenes_1639_v127	0.9254
Listeria_monocytogenes_1639_v180	Listeria_monocytogenes_1639_v127	0.9254
Listeria_monocytogenes_1639_v17	Listeria_monocytogenes_1639_v127	0.9275
Listeria_monocytogenes_1639_v179	Listeria_monocytogenes_1639_v127	0.9619
Listeria_monocytogenes_1639_v178	Listeria_monocytogenes_1639_v127	0.9654
Listeria_monocytogenes_1639_v177	Listeria_monocytogenes_1639_v127	0.9413
Listeria_monocytogenes_1639_v176	Listeria_monocytogenes_1639_v127	0.9341
Listeria_monocytogenes_1639_v175	Listeria_monocytogenes_1639_v127	0.9581
Listeria_monocytogenes_1639_v174	Listeria_monocytogenes_1639_v127	0.9674
Listeria_monocytogenes_1639_v173	Listeria_monocytogenes_1639_v127	0.9482
Listeria_monocytogenes_1639_v172	Listeria_monocytogenes_1639_v127	0.9401
Listeria_monocytogenes_1639_v171	Listeria_monocytogenes_1639_v127	0.9516
Listeria_monocytogenes_1639_v170	Listeria_monocytogenes_1639_v127	0.9592
Listeria_monocytogenes_1639_v16	Listeria_monocytogenes_1639_v127	0.9277
Listeria_monocytogenes_1639_v169	Listeria_monocytogenes_1639_v127	0.9584
Listeria_monocytogenes_1639_v168	Listeria_monocytogenes_1639_v127	0.9617
Listeria_monocytogenes_1639_v167	Listeria_monocytogenes_1639_v127	0.9615
Listeria_monocytogenes_1639_v166	Listeria_monocytogenes_1639_v127	0.962
Listeria_monocytogenes_1639_v165	Listeria_monocytogenes_1639_v127	0.9585
Listeria_monocytogenes_1639_v164	Listeria_monocytogenes_1639_v127	0.9369
Listeria_monocytogenes_1639_v163	Listeria_monocytogenes_1639_v127	0.9465
Listeria_monocytogenes_1639_v162	Listeria_monocytogenes_1639_v127	0.9369
Listeria_monocytogenes_1639_v161	Listeria_monocytogenes_1639_v127	0.938
Listeria_monocytogenes_1639_v160	Listeria_monocytogenes_1639_v127	0.9377
Listeria_monocytogenes_1639_v15	Listeria_monocytogenes_1639_v127	0.9262
Listeria_monocytogenes_1639_v159	Listeria_monocytogenes_1639_v127	0.9413
Listeria_monocytogenes_1639_v158	Listeria_monocytogenes_1639_v127	0.9374
Listeria_monocytogenes_1639_v157	Listeria_monocytogenes_1639_v127	0.9374
Listeria_monocytogenes_1639_v156	Listeria_monocytogenes_1639_v127	0.9542
Listeria_monocytogenes_1639_v155	Listeria_monocytogenes_1639_v127	0.9624
Listeria_monocytogenes_1639_v154	Listeria_monocytogenes_1639_v127	0.9544
Listeria_monocytogenes_1639_v153	Listeria_monocytogenes_1639_v127	0.9703
Listeria_monocytogenes_1639_v152	Listeria_monocytogenes_1639_v127	0.9413
Listeria_monocytogenes_1639_v151	Listeria_monocytogenes_1639_v127	0.9413
Listeria_monocytogenes_1639_v150	Listeria_monocytogenes_1639_v127	0.9555
Listeria_monocytogenes_1639_v14	Listeria_monocytogenes_1639_v127	0.9334
Listeria_monocytogenes_1639_v149	Listeria_monocytogenes_1639_v127	0.9545
Listeria_monocytogenes_1639_v148	Listeria_monocytogenes_1639_v127	0.9402
Listeria_monocytogenes_1639_v147	Listeria_monocytogenes_1639_v127	0.9271
Listeria_monocytogenes_1639_v146	Listeria_monocytogenes_1639_v127	0.9678
Listeria_monocytogenes_1639_v145	Listeria_monocytogenes_1639_v127	0.9583
Listeria_monocytogenes_1639_v144	Listeria_monocytogenes_1639_v127	0.9427
Listeria_monocytogenes_1639_v143	Listeria_monocytogenes_1639_v127	0.9675
Listeria_monocytogenes_1639_v142	Listeria_monocytogenes_1639_v127	0.943
Listeria_monocytogenes_1639_v141	Listeria_monocytogenes_1639_v127	0.9479
Listeria_monocytogenes_1639_v140	Listeria_monocytogenes_1639_v127	0.9438
Listeria_monocytogenes_1639_v13	Listeria_monocytogenes_1639_v127	0.9262
Listeria_monocytogenes_1639_v139	Listeria_monocytogenes_1639_v127	0.9393
Listeria_monocytogenes_1639_v138	Listeria_monocytogenes_1639_v127	0.9427
Listeria_monocytogenes_1639_v137	Listeria_monocytogenes_1639_v127	0.9423
Listeria_monocytogenes_1639_v136	Listeria_monocytogenes_1639_v127	0.9569
Listeria_monocytogenes_1639_v135	Listeria_monocytogenes_1639_v127	0.928
Listeria_monocytogenes_1639_v134	Listeria_monocytogenes_1639_v127	0.9315
Listeria_monocytogenes_1639_v133	Listeria_monocytogenes_1639_v127	0.9643
Listeria_monocytogenes_1639_v132	Listeria_monocytogenes_1639_v127	0.9235
Listeria_monocytogenes_1639_v131	Listeria_monocytogenes_1639_v127	0.9403
Listeria_monocytogenes_1639_v130	Listeria_monocytogenes_1639_v127	0.9535
Listeria_monocytogenes_1639_v12	Listeria_monocytogenes_1639_v127	0.9373
Listeria_monocytogenes_1639_v129	Listeria_monocytogenes_1639_v127	0.9484
Listeria_monocytogenes_1639_v128	Listeria_monocytogenes_1639_v127	0.944
Listeria_monocytogenes_1639_v127	Listeria_monocytogenes_1639_v127	0.0654

Listeria_welshimeri_1643	Listeria_monocytogenes_1639_v128	0.8836
Listeria_welshimeri_serovar_6b_str_SLCC5334_386043	Listeria_monocytogenes_1639_v128	0.8832
Listeria_seeligeri_serovar_1_2b_str_SLCC3954_683837	Listeria_monocytogenes_1639_v128	0.8951
Listeria_seeligeri_FSL_S4-171_702452	Listeria_monocytogenes_1639_v128	0.8308
Listeria_seeligeri_FSL_N1-067_702453	Listeria_monocytogenes_1639_v128	0.8358
Listeria_seeligeri_1640_v1	Listeria_monocytogenes_1639_v128	0.8985
Listeria_seeligeri_1640	Listeria_monocytogenes_1639_v128	0.8865
Listeria_monocytogenes_WSLC1042_1457188	Listeria_monocytogenes_1639_v128	0.9618
Listeria_monocytogenes_WSLC1001_1457187	Listeria_monocytogenes_1639_v128	0.941
Listeria_monocytogenes_SLCC7179_879090	Listeria_monocytogenes_1639_v128	0.947
Listeria_monocytogenes_SLCC5850_882096	Listeria_monocytogenes_1639_v128	0.945
Listeria_monocytogenes_SLCC2755_932919	Listeria_monocytogenes_1639_v128	0.9553
Listeria_monocytogenes_SLCC2540_879089	Listeria_monocytogenes_1639_v128	0.9619
Listeria_monocytogenes_SLCC2479_882020	Listeria_monocytogenes_1639_v128	0.9395
Listeria_monocytogenes_SLCC2378_879088	Listeria_monocytogenes_1639_v128	0.9625
Listeria_monocytogenes_SLCC2376_882097	Listeria_monocytogenes_1639_v128	0.9459
Listeria_monocytogenes_SLCC2372_932920	Listeria_monocytogenes_1639_v128	0.9321
Listeria_monocytogenes_serotype_4b_str_LL195_1230340	Listeria_monocytogenes_1639_v128	0.9665
Listeria_monocytogenes_serotype_4b_str_LIS0087_1134669	Listeria_monocytogenes_1639_v128	0.9228
Listeria_monocytogenes_serotype_4b_str_F2365_265669	Listeria_monocytogenes_1639_v128	0.9665
Listeria_monocytogenes_serotype_4b_str_CLIP_80459_568819	Listeria_monocytogenes_1639_v128	0.9676
Listeria_monocytogenes_serotype_4b_str_81-0861_1196161	Listeria_monocytogenes_1639_v128	0.9523
Listeria_monocytogenes_serotype_4b_str_81-0592_1196160	Listeria_monocytogenes_1639_v128	0.9534
Listeria_monocytogenes_serotype_4b_str_81-0558_1196159	Listeria_monocytogenes_1639_v128	0.9542
Listeria_monocytogenes_serotype_4b_str_10-0809_1196162	Listeria_monocytogenes_1639_v128	0.9538
Listeria_monocytogenes_serotype_4b_str_02-6680_1196175	Listeria_monocytogenes_1639_v128	0.9654
Listeria_monocytogenes_serotype_4b_str_02-6679_1196174	Listeria_monocytogenes_1639_v128	0.9654
Listeria_monocytogenes_serotype_4b_str_02-1792_1196173	Listeria_monocytogenes_1639_v128	0.9542
Listeria_monocytogenes_serotype_4b_str_02-1289_1196172	Listeria_monocytogenes_1639_v128	0.9542
Listeria_monocytogenes_serotype_4b_str_02-1103_1196171	Listeria_monocytogenes_1639_v128	0.9542
Listeria_monocytogenes_serotype_3c_str_10-5027_1196195	Listeria_monocytogenes_1639_v128	0.9391
Listeria_monocytogenes_serotype_1_2c_str_10-5026_1196194	Listeria_monocytogenes_1639_v128	0.9399
Listeria_monocytogenes_serotype_1_2c_str_10-5025_1196193	Listeria_monocytogenes_1639_v128	0.9399
Listeria_monocytogenes_serotype_1_2b_str_10-0811_1196164	Listeria_monocytogenes_1639_v128	0.9507
Listeria_monocytogenes_serotype_1_2b_str_10-0810_1196163	Listeria_monocytogenes_1639_v128	0.9507
Listeria_monocytogenes_serotype_1_2a_str_99-6370_1196179	Listeria_monocytogenes_1639_v128	0.928
Listeria_monocytogenes_serotype_1_2a_str_98-2035_1196178	Listeria_monocytogenes_1639_v128	0.928
Listeria_monocytogenes_serotype_1_2a_str_95-0093_1196177	Listeria_monocytogenes_1639_v128	0.9336
Listeria_monocytogenes_serotype_1_2a_str_88-0478_1196176	Listeria_monocytogenes_1639_v128	0.9324
Listeria_monocytogenes_serotype_1_2a_str_10-5024_1196192	Listeria_monocytogenes_1639_v128	0.9336
Listeria_monocytogenes_serotype_1_2a_str_10-4758_1196168	Listeria_monocytogenes_1639_v128	0.9462
Listeria_monocytogenes_serotype_1_2a_str_10-4754_1196167	Listeria_monocytogenes_1639_v128	0.9462
Listeria_monocytogenes_serotype_1_2a_str_10-1321_1196191	Listeria_monocytogenes_1639_v128	0.9336
Listeria_monocytogenes_serotype_1_2a_str_10-1047_1196190	Listeria_monocytogenes_1639_v128	0.9343
Listeria_monocytogenes_serotype_1_2a_str_10-1046_1196189	Listeria_monocytogenes_1639_v128	0.9343
Listeria_monocytogenes_serotype_1_2a_str_10-0934_1196170	Listeria_monocytogenes_1639_v128	0.9563
Listeria_monocytogenes_serotype_1_2a_str_10-0933_1196169	Listeria_monocytogenes_1639_v128	0.9563
Listeria_monocytogenes_serotype_1_2a_str_10-0815_1196188	Listeria_monocytogenes_1639_v128	0.9294
Listeria_monocytogenes_serotype_1_2a_str_10-0814_1196187	Listeria_monocytogenes_1639_v128	0.9339
Listeria_monocytogenes_serotype_1_2a_str_10-0813_1196166	Listeria_monocytogenes_1639_v128	0.9393
Listeria_monocytogenes_serotype_1_2a_str_10-0812_1196165	Listeria_monocytogenes_1639_v128	0.9393
Listeria_monocytogenes_serotype_1_2a_str_08-7669_1196186	Listeria_monocytogenes_1639_v128	0.9427
Listeria_monocytogenes_serotype_1_2a_str_08-7374_1196185	Listeria_monocytogenes_1639_v128	0.9336
Listeria_monocytogenes_serotype_1_2a_str_08-6997_1196184	Listeria_monocytogenes_1639_v128	0.9294
Listeria_monocytogenes_serotype_1_2a_str_08-6569_1196183	Listeria_monocytogenes_1639_v128	0.9294
Listeria_monocytogenes_serotype_1_2a_str_08-6056_1196182	Listeria_monocytogenes_1639_v128	0.9294
Listeria_monocytogenes_serotype_1_2a_str_04-5457_1196181	Listeria_monocytogenes_1639_v128	0.9336
Listeria_monocytogenes_serotype_1_2a_str_02-5993_1196180	Listeria_monocytogenes_1639_v128	0.9336
Listeria_monocytogenes_R479a_1437838	Listeria_monocytogenes_1639_v128	0.9321
Listeria_monocytogenes_N53-1_1234142	Listeria_monocytogenes_1639_v128	0.8998
Listeria_monocytogenes_M7_1030009	Listeria_monocytogenes_1639_v128	0.9078
Listeria_monocytogenes_LO28_393132	Listeria_monocytogenes_1639_v128	0.9385
Listeria_monocytogenes_L99_563174	Listeria_monocytogenes_1639_v128	0.9081
Listeria_monocytogenes_L312_882094	Listeria_monocytogenes_1639_v128	0.968
Listeria_monocytogenes_J1816_930781	Listeria_monocytogenes_1639_v128	0.9476
Listeria_monocytogenes_J1-220_930782	Listeria_monocytogenes_1639_v128	0.9444
Listeria_monocytogenes_J0161_393130	Listeria_monocytogenes_1639_v128	0.9355
Listeria_monocytogenes_HCC23_552536	Listeria_monocytogenes_1639_v128	0.9074
Listeria_monocytogenes_FSL_R2-561_393126	Listeria_monocytogenes_1639_v128	0.9377
Listeria_monocytogenes_FSL_J1-208_393119	Listeria_monocytogenes_1639_v128	0.9166
Listeria_monocytogenes_FSL_J1-175_393118	Listeria_monocytogenes_1639_v128	0.9625
Listeria_monocytogenes_Finland_1998_393127	Listeria_monocytogenes_1639_v128	0.9563
Listeria_monocytogenes_EGD-e_169963_v1	Listeria_monocytogenes_1639_v128	0.9523
Listeria_monocytogenes_EGD-e_169963	Listeria_monocytogenes_1639_v128	0.9527
Listeria_monocytogenes_EGD_1334565	Listeria_monocytogenes_1639_v128	0.9457
Listeria_monocytogenes_CFSAN002349_1299895	Listeria_monocytogenes_1639_v128	0.9681
Listeria_monocytogenes_ATCC_19117_882095_v1	Listeria_monocytogenes_1639_v128	0.9628
Listeria_monocytogenes_ATCC_19117_882095	Listeria_monocytogenes_1639_v128	0.9624
Listeria_monocytogenes_6179_1288295	Listeria_monocytogenes_1639_v128	0.92
Listeria_monocytogenes_1639_v9	Listeria_monocytogenes_1639_v128	0.9354
Listeria_monocytogenes_1639_v99	Listeria_monocytogenes_1639_v128	0.9484
Listeria_monocytogenes_1639_v98	Listeria_monocytogenes_1639_v128	0.9339
Listeria_monocytogenes_1639_v97	Listeria_monocytogenes_1639_v128	0.9563
Listeria_monocytogenes_1639_v96	Listeria_monocytogenes_1639_v128	0.948
Listeria_monocytogenes_1639_v95	Listeria_monocytogenes_1639_v128	0.948
Listeria_monocytogenes_1639_v94	Listeria_monocytogenes_1639_v128	0.9488
Listeria_monocytogenes_1639_v93	Listeria_monocytogenes_1639_v128	0.9331
Listeria_monocytogenes_1639_v92	Listeria_monocytogenes_1639_v128	0.9488
Listeria_monocytogenes_1639_v91	Listeria_monocytogenes_1639_v128	0.9498
Listeria_monocytogenes_1639_v90	Listeria_monocytogenes_1639_v128	0.9339
Listeria_monocytogenes_1639_v8	Listeria_monocytogenes_1639_v128	0.9214

Listeria_monocytogenes_1639_v89	Listeria_monocytogenes_1639_v128	0.9607
Listeria_monocytogenes_1639_v88	Listeria_monocytogenes_1639_v128	0.9394
Listeria_monocytogenes_1639_v87	Listeria_monocytogenes_1639_v128	0.9351
Listeria_monocytogenes_1639_v86	Listeria_monocytogenes_1639_v128	0.9347
Listeria_monocytogenes_1639_v85	Listeria_monocytogenes_1639_v128	0.952
Listeria_monocytogenes_1639_v84	Listeria_monocytogenes_1639_v128	0.9411
Listeria_monocytogenes_1639_v83	Listeria_monocytogenes_1639_v128	0.9534
Listeria_monocytogenes_1639_v82	Listeria_monocytogenes_1639_v128	0.953
Listeria_monocytogenes_1639_v81	Listeria_monocytogenes_1639_v128	0.939
Listeria_monocytogenes_1639_v80	Listeria_monocytogenes_1639_v128	0.9548
Listeria_monocytogenes_1639_v7	Listeria_monocytogenes_1639_v128	0.9214
Listeria_monocytogenes_1639_v79	Listeria_monocytogenes_1639_v128	0.9376
Listeria_monocytogenes_1639_v78	Listeria_monocytogenes_1639_v128	0.9383
Listeria_monocytogenes_1639_v77	Listeria_monocytogenes_1639_v128	0.9441
Listeria_monocytogenes_1639_v76	Listeria_monocytogenes_1639_v128	0.9308
Listeria_monocytogenes_1639_v75	Listeria_monocytogenes_1639_v128	0.9081
Listeria_monocytogenes_1639_v74	Listeria_monocytogenes_1639_v128	0.9325
Listeria_monocytogenes_1639_v73	Listeria_monocytogenes_1639_v128	0.9343
Listeria_monocytogenes_1639_v72	Listeria_monocytogenes_1639_v128	0.951
Listeria_monocytogenes_1639_v71	Listeria_monocytogenes_1639_v128	0.9206
Listeria_monocytogenes_1639_v70	Listeria_monocytogenes_1639_v128	0.9356
Listeria_monocytogenes_1639_v6	Listeria_monocytogenes_1639_v128	0.9211
Listeria_monocytogenes_1639_v69	Listeria_monocytogenes_1639_v128	0.9534
Listeria_monocytogenes_1639_v68	Listeria_monocytogenes_1639_v128	0.9431
Listeria_monocytogenes_1639_v67	Listeria_monocytogenes_1639_v128	0.9545
Listeria_monocytogenes_1639_v66	Listeria_monocytogenes_1639_v128	0.9413
Listeria_monocytogenes_1639_v65	Listeria_monocytogenes_1639_v128	0.9364
Listeria_monocytogenes_1639_v64	Listeria_monocytogenes_1639_v128	0.9345
Listeria_monocytogenes_1639_v63	Listeria_monocytogenes_1639_v128	0.9506
Listeria_monocytogenes_1639_v62	Listeria_monocytogenes_1639_v128	0.9359
Listeria_monocytogenes_1639_v61	Listeria_monocytogenes_1639_v128	0.9363
Listeria_monocytogenes_1639_v60	Listeria_monocytogenes_1639_v128	0.9363
Listeria_monocytogenes_1639_v5	Listeria_monocytogenes_1639_v128	0.9145
Listeria_monocytogenes_1639_v59	Listeria_monocytogenes_1639_v128	0.953
Listeria_monocytogenes_1639_v58	Listeria_monocytogenes_1639_v128	0.9583
Listeria_monocytogenes_1639_v57	Listeria_monocytogenes_1639_v128	0.9441
Listeria_monocytogenes_1639_v56	Listeria_monocytogenes_1639_v128	0.953
Listeria_monocytogenes_1639_v55	Listeria_monocytogenes_1639_v128	0.941
Listeria_monocytogenes_1639_v54	Listeria_monocytogenes_1639_v128	0.9255
Listeria_monocytogenes_1639_v53	Listeria_monocytogenes_1639_v128	0.9382
Listeria_monocytogenes_1639_v52	Listeria_monocytogenes_1639_v128	0.9482
Listeria_monocytogenes_1639_v51	Listeria_monocytogenes_1639_v128	0.9476
Listeria_monocytogenes_1639_v50	Listeria_monocytogenes_1639_v128	0.9328
Listeria_monocytogenes_1639_v4	Listeria_monocytogenes_1639_v128	0.9279
Listeria_monocytogenes_1639_v49	Listeria_monocytogenes_1639_v128	0.9561
Listeria_monocytogenes_1639_v48	Listeria_monocytogenes_1639_v128	0.9343
Listeria_monocytogenes_1639_v47	Listeria_monocytogenes_1639_v128	0.9332
Listeria_monocytogenes_1639_v46	Listeria_monocytogenes_1639_v128	0.942
Listeria_monocytogenes_1639_v45	Listeria_monocytogenes_1639_v128	0.9242
Listeria_monocytogenes_1639_v44	Listeria_monocytogenes_1639_v128	0.9336
Listeria_monocytogenes_1639_v43	Listeria_monocytogenes_1639_v128	0.9336
Listeria_monocytogenes_1639_v42	Listeria_monocytogenes_1639_v128	0.9231
Listeria_monocytogenes_1639_v41	Listeria_monocytogenes_1639_v128	0.9093
Listeria_monocytogenes_1639_v40	Listeria_monocytogenes_1639_v128	0.9385
Listeria_monocytogenes_1639_v3	Listeria_monocytogenes_1639_v128	0.9283
Listeria_monocytogenes_1639_v39	Listeria_monocytogenes_1639_v128	0.9506
Listeria_monocytogenes_1639_v38	Listeria_monocytogenes_1639_v128	0.9368
Listeria_monocytogenes_1639_v37	Listeria_monocytogenes_1639_v128	0.9308
Listeria_monocytogenes_1639_v36	Listeria_monocytogenes_1639_v128	0.9309
Listeria_monocytogenes_1639_v35	Listeria_monocytogenes_1639_v128	0.9392
Listeria_monocytogenes_1639_v34	Listeria_monocytogenes_1639_v128	0.935
Listeria_monocytogenes_1639_v33	Listeria_monocytogenes_1639_v128	0.9507
Listeria_monocytogenes_1639_v32	Listeria_monocytogenes_1639_v128	0.9297
Listeria_monocytogenes_1639_v31	Listeria_monocytogenes_1639_v128	0.9354
Listeria_monocytogenes_1639_v30	Listeria_monocytogenes_1639_v128	0.9514
Listeria_monocytogenes_1639_v2	Listeria_monocytogenes_1639_v128	0.9156
Listeria_monocytogenes_1639_v29	Listeria_monocytogenes_1639_v128	0.9252
Listeria_monocytogenes_1639_v28	Listeria_monocytogenes_1639_v128	0.9293
Listeria_monocytogenes_1639_v27	Listeria_monocytogenes_1639_v128	0.9201
Listeria_monocytogenes_1639_v26	Listeria_monocytogenes_1639_v128	0.9201
Listeria_monocytogenes_1639_v25	Listeria_monocytogenes_1639_v128	0.9209
Listeria_monocytogenes_1639_v24	Listeria_monocytogenes_1639_v128	0.9452
Listeria_monocytogenes_1639_v23	Listeria_monocytogenes_1639_v128	0.9448
Listeria_monocytogenes_1639_v22	Listeria_monocytogenes_1639_v128	0.9518
Listeria_monocytogenes_1639_v21	Listeria_monocytogenes_1639_v128	0.9444
Listeria_monocytogenes_1639_v20	Listeria_monocytogenes_1639_v128	0.9465
Listeria_monocytogenes_1639_v1	Listeria_monocytogenes_1639_v128	0.9129
Listeria_monocytogenes_1639_v19	Listeria_monocytogenes_1639_v128	0.9461
Listeria_monocytogenes_1639_v198	Listeria_monocytogenes_1639_v128	0.9018
Listeria_monocytogenes_1639_v197	Listeria_monocytogenes_1639_v128	0.9397
Listeria_monocytogenes_1639_v196	Listeria_monocytogenes_1639_v128	0.9285
Listeria_monocytogenes_1639_v195	Listeria_monocytogenes_1639_v128	0.9466
Listeria_monocytogenes_1639_v194	Listeria_monocytogenes_1639_v128	0.9463
Listeria_monocytogenes_1639_v193	Listeria_monocytogenes_1639_v128	0.9463
Listeria_monocytogenes_1639_v192	Listeria_monocytogenes_1639_v128	0.9076
Listeria_monocytogenes_1639_v191	Listeria_monocytogenes_1639_v128	0.9542
Listeria_monocytogenes_1639_v190	Listeria_monocytogenes_1639_v128	0.9562
Listeria_monocytogenes_1639_v18	Listeria_monocytogenes_1639_v128	0.9396
Listeria_monocytogenes_1639_v189	Listeria_monocytogenes_1639_v128	0.9555
Listeria_monocytogenes_1639_v188	Listeria_monocytogenes_1639_v128	0.955
Listeria_monocytogenes_1639_v187	Listeria_monocytogenes_1639_v128	0.9562

Listeria_monocytogenes_1639_v186	Listeria_monocytogenes_1639_v128	0.9566
Listeria_monocytogenes_1639_v185	Listeria_monocytogenes_1639_v128	0.9566
Listeria_monocytogenes_1639_v184	Listeria_monocytogenes_1639_v128	0.9554
Listeria_monocytogenes_1639_v183	Listeria_monocytogenes_1639_v128	0.9531
Listeria_monocytogenes_1639_v182	Listeria_monocytogenes_1639_v128	0.9553
Listeria_monocytogenes_1639_v181	Listeria_monocytogenes_1639_v128	0.9268
Listeria_monocytogenes_1639_v180	Listeria_monocytogenes_1639_v128	0.9268
Listeria_monocytogenes_1639_v17	Listeria_monocytogenes_1639_v128	0.94
Listeria_monocytogenes_1639_v179	Listeria_monocytogenes_1639_v128	0.9398
Listeria_monocytogenes_1639_v178	Listeria_monocytogenes_1639_v128	0.9574
Listeria_monocytogenes_1639_v177	Listeria_monocytogenes_1639_v128	0.9658
Listeria_monocytogenes_1639_v176	Listeria_monocytogenes_1639_v128	0.9432
Listeria_monocytogenes_1639_v175	Listeria_monocytogenes_1639_v128	0.9466
Listeria_monocytogenes_1639_v174	Listeria_monocytogenes_1639_v128	0.9605
Listeria_monocytogenes_1639_v173	Listeria_monocytogenes_1639_v128	0.9772
Listeria_monocytogenes_1639_v172	Listeria_monocytogenes_1639_v128	0.9376
Listeria_monocytogenes_1639_v171	Listeria_monocytogenes_1639_v128	0.9416
Listeria_monocytogenes_1639_v170	Listeria_monocytogenes_1639_v128	0.95
Listeria_monocytogenes_1639_v16	Listeria_monocytogenes_1639_v128	0.9198
Listeria_monocytogenes_1639_v169	Listeria_monocytogenes_1639_v128	0.9508
Listeria_monocytogenes_1639_v168	Listeria_monocytogenes_1639_v128	0.949
Listeria_monocytogenes_1639_v167	Listeria_monocytogenes_1639_v128	0.9404
Listeria_monocytogenes_1639_v166	Listeria_monocytogenes_1639_v128	0.9494
Listeria_monocytogenes_1639_v165	Listeria_monocytogenes_1639_v128	0.9504
Listeria_monocytogenes_1639_v164	Listeria_monocytogenes_1639_v128	0.9661
Listeria_monocytogenes_1639_v163	Listeria_monocytogenes_1639_v128	0.9734
Listeria_monocytogenes_1639_v162	Listeria_monocytogenes_1639_v128	0.9661
Listeria_monocytogenes_1639_v161	Listeria_monocytogenes_1639_v128	0.9672
Listeria_monocytogenes_1639_v160	Listeria_monocytogenes_1639_v128	0.9669
Listeria_monocytogenes_1639_v15	Listeria_monocytogenes_1639_v128	0.9349
Listeria_monocytogenes_1639_v159	Listeria_monocytogenes_1639_v128	0.9662
Listeria_monocytogenes_1639_v158	Listeria_monocytogenes_1639_v128	0.9669
Listeria_monocytogenes_1639_v157	Listeria_monocytogenes_1639_v128	0.9665
Listeria_monocytogenes_1639_v156	Listeria_monocytogenes_1639_v128	0.9346
Listeria_monocytogenes_1639_v155	Listeria_monocytogenes_1639_v128	0.946
Listeria_monocytogenes_1639_v154	Listeria_monocytogenes_1639_v128	0.9506
Listeria_monocytogenes_1639_v153	Listeria_monocytogenes_1639_v128	0.9452
Listeria_monocytogenes_1639_v152	Listeria_monocytogenes_1639_v128	0.9662
Listeria_monocytogenes_1639_v151	Listeria_monocytogenes_1639_v128	0.9662
Listeria_monocytogenes_1639_v150	Listeria_monocytogenes_1639_v128	0.9475
Listeria_monocytogenes_1639_v14	Listeria_monocytogenes_1639_v128	0.9411
Listeria_monocytogenes_1639_v149	Listeria_monocytogenes_1639_v128	0.9461
Listeria_monocytogenes_1639_v148	Listeria_monocytogenes_1639_v128	0.9654
Listeria_monocytogenes_1639_v147	Listeria_monocytogenes_1639_v128	0.9508
Listeria_monocytogenes_1639_v146	Listeria_monocytogenes_1639_v128	0.9571
Listeria_monocytogenes_1639_v145	Listeria_monocytogenes_1639_v128	0.9491
Listeria_monocytogenes_1639_v144	Listeria_monocytogenes_1639_v128	0.9672
Listeria_monocytogenes_1639_v143	Listeria_monocytogenes_1639_v128	0.9537
Listeria_monocytogenes_1639_v142	Listeria_monocytogenes_1639_v128	0.9676
Listeria_monocytogenes_1639_v141	Listeria_monocytogenes_1639_v128	0.995
Listeria_monocytogenes_1639_v140	Listeria_monocytogenes_1639_v128	0.9684
Listeria_monocytogenes_1639_v13	Listeria_monocytogenes_1639_v128	0.9349
Listeria_monocytogenes_1639_v139	Listeria_monocytogenes_1639_v128	0.9634
Listeria_monocytogenes_1639_v138	Listeria_monocytogenes_1639_v128	0.9707
Listeria_monocytogenes_1639_v137	Listeria_monocytogenes_1639_v128	0.9707
Listeria_monocytogenes_1639_v136	Listeria_monocytogenes_1639_v128	0.9458
Listeria_monocytogenes_1639_v135	Listeria_monocytogenes_1639_v128	0.919
Listeria_monocytogenes_1639_v134	Listeria_monocytogenes_1639_v128	0.9624
Listeria_monocytogenes_1639_v133	Listeria_monocytogenes_1639_v128	0.9448
Listeria_monocytogenes_1639_v132	Listeria_monocytogenes_1639_v128	0.9199
Listeria_monocytogenes_1639_v131	Listeria_monocytogenes_1639_v128	0.9558
Listeria_monocytogenes_1639_v130	Listeria_monocytogenes_1639_v128	0.9388
Listeria_monocytogenes_1639_v12	Listeria_monocytogenes_1639_v128	0.9195
Listeria_monocytogenes_1639_v129	Listeria_monocytogenes_1639_v128	0.9784
Listeria_monocytogenes_1639_v128	Listeria_monocytogenes_1639_v128	0.0657
Listeria_welshimeri_1643	Listeria_monocytogenes_1639_v129	0.8813
Listeria_welshimeri_serovar_6b_str_SLCC5334_386043	Listeria_monocytogenes_1639_v129	0.8809
Listeria_seeligeri_serovar_1_2b_str_SLCC3954_683837	Listeria_monocytogenes_1639_v129	0.8948
Listeria_seeligeri_FSL_S4-171_702452	Listeria_monocytogenes_1639_v129	0.8286
Listeria_seeligeri_FSL_N1-067_702453	Listeria_monocytogenes_1639_v129	0.836
Listeria_seeligeri_1640_v1	Listeria_monocytogenes_1639_v129	0.8978
Listeria_seeligeri_1640	Listeria_monocytogenes_1639_v129	0.8854
Listeria_monocytogenes_WSLC1042_1457188	Listeria_monocytogenes_1639_v129	0.9628
Listeria_monocytogenes_WSLC1001_1457187	Listeria_monocytogenes_1639_v129	0.9408
Listeria_monocytogenes_SLCC7179_879090	Listeria_monocytogenes_1639_v129	0.9488
Listeria_monocytogenes_SLCC5850_882096	Listeria_monocytogenes_1639_v129	0.9456
Listeria_monocytogenes_SLCC2755_932919	Listeria_monocytogenes_1639_v129	0.9521
Listeria_monocytogenes_SLCC2540_879089	Listeria_monocytogenes_1639_v129	0.963
Listeria_monocytogenes_SLCC2479_882020	Listeria_monocytogenes_1639_v129	0.9416
Listeria_monocytogenes_SLCC2378_879088	Listeria_monocytogenes_1639_v129	0.9639
Listeria_monocytogenes_SLCC2376_882097	Listeria_monocytogenes_1639_v129	0.9496
Listeria_monocytogenes_SLCC2372_932920	Listeria_monocytogenes_1639_v129	0.9342
Listeria_monocytogenes_serotype_4b_str_LL195_1230340	Listeria_monocytogenes_1639_v129	0.9679
Listeria_monocytogenes_serotype_4b_str_LIS0087_1134669	Listeria_monocytogenes_1639_v129	0.923
Listeria_monocytogenes_serotype_4b_str_F2365_265669	Listeria_monocytogenes_1639_v129	0.9679
Listeria_monocytogenes_serotype_4b_str_CLIP_80459_568819	Listeria_monocytogenes_1639_v129	0.9687
Listeria_monocytogenes_serotype_4b_str_81-0861_1196161	Listeria_monocytogenes_1639_v129	0.9526
Listeria_monocytogenes_serotype_4b_str_81-0592_1196160	Listeria_monocytogenes_1639_v129	0.9537
Listeria_monocytogenes_serotype_4b_str_81-0558_1196159	Listeria_monocytogenes_1639_v129	0.9529
Listeria_monocytogenes_serotype_4b_str_10-0809_1196162	Listeria_monocytogenes_1639_v129	0.954
Listeria_monocytogenes_serotype_4b_str_02-6680_1196175	Listeria_monocytogenes_1639_v129	0.9641

Listeria_monocytogenes_serotype_4b_str_02-6679_1196174	Listeria_monocytogenes_1639_v129	0.9641
Listeria_monocytogenes_serotype_4b_str_02-1792_1196173	Listeria_monocytogenes_1639_v129	0.9544
Listeria_monocytogenes_serotype_4b_str_02-1289_1196172	Listeria_monocytogenes_1639_v129	0.9544
Listeria_monocytogenes_serotype_4b_str_02-1103_1196171	Listeria_monocytogenes_1639_v129	0.9544
Listeria_monocytogenes_serotype_3c_str_10-5027_1196195	Listeria_monocytogenes_1639_v129	0.9408
Listeria_monocytogenes_serotype_1_2c_str_10-5026_1196194	Listeria_monocytogenes_1639_v129	0.9416
Listeria_monocytogenes_serotype_1_2c_str_10-5025_1196193	Listeria_monocytogenes_1639_v129	0.9416
Listeria_monocytogenes_serotype_1_2b_str_10-0811_1196164	Listeria_monocytogenes_1639_v129	0.9668
Listeria_monocytogenes_serotype_1_2b_str_10-0810_1196163	Listeria_monocytogenes_1639_v129	0.9668
Listeria_monocytogenes_serotype_1_2a_str_99-6370_1196179	Listeria_monocytogenes_1639_v129	0.9267
Listeria_monocytogenes_serotype_1_2a_str_98-2035_1196178	Listeria_monocytogenes_1639_v129	0.9267
Listeria_monocytogenes_serotype_1_2a_str_95-0093_1196177	Listeria_monocytogenes_1639_v129	0.9322
Listeria_monocytogenes_serotype_1_2a_str_88-0478_1196176	Listeria_monocytogenes_1639_v129	0.9311
Listeria_monocytogenes_serotype_1_2a_str_10-5024_1196192	Listeria_monocytogenes_1639_v129	0.9322
Listeria_monocytogenes_serotype_1_2a_str_10-4758_1196168	Listeria_monocytogenes_1639_v129	0.9495
Listeria_monocytogenes_serotype_1_2a_str_10-4754_1196167	Listeria_monocytogenes_1639_v129	0.9498
Listeria_monocytogenes_serotype_1_2a_str_10-1321_1196191	Listeria_monocytogenes_1639_v129	0.9322
Listeria_monocytogenes_serotype_1_2a_str_10-1047_1196190	Listeria_monocytogenes_1639_v129	0.9329
Listeria_monocytogenes_serotype_1_2a_str_10-1046_1196189	Listeria_monocytogenes_1639_v129	0.9329
Listeria_monocytogenes_serotype_1_2a_str_10-0934_1196170	Listeria_monocytogenes_1639_v129	0.9593
Listeria_monocytogenes_serotype_1_2a_str_10-0933_1196169	Listeria_monocytogenes_1639_v129	0.9593
Listeria_monocytogenes_serotype_1_2a_str_10-0815_1196188	Listeria_monocytogenes_1639_v129	0.9281
Listeria_monocytogenes_serotype_1_2a_str_10-0814_1196187	Listeria_monocytogenes_1639_v129	0.9326
Listeria_monocytogenes_serotype_1_2a_str_10-0813_1196166	Listeria_monocytogenes_1639_v129	0.9387
Listeria_monocytogenes_serotype_1_2a_str_10-0812_1196165	Listeria_monocytogenes_1639_v129	0.9387
Listeria_monocytogenes_serotype_1_2a_str_08-7669_1196186	Listeria_monocytogenes_1639_v129	0.9422
Listeria_monocytogenes_serotype_1_2a_str_08-7374_1196185	Listeria_monocytogenes_1639_v129	0.9322
Listeria_monocytogenes_serotype_1_2a_str_08-6997_1196184	Listeria_monocytogenes_1639_v129	0.9281
Listeria_monocytogenes_serotype_1_2a_str_08-6569_1196183	Listeria_monocytogenes_1639_v129	0.9281
Listeria_monocytogenes_serotype_1_2a_str_08-6056_1196182	Listeria_monocytogenes_1639_v129	0.9281
Listeria_monocytogenes_serotype_1_2a_str_04-5457_1196181	Listeria_monocytogenes_1639_v129	0.9322
Listeria_monocytogenes_serotype_1_2a_str_02-5993_1196180	Listeria_monocytogenes_1639_v129	0.9322
Listeria_monocytogenes_R479a_1437838	Listeria_monocytogenes_1639_v129	0.9312
Listeria_monocytogenes_N53-1_1234142	Listeria_monocytogenes_1639_v129	0.9039
Listeria_monocytogenes_M7_1030009	Listeria_monocytogenes_1639_v129	0.9098
Listeria_monocytogenes_LO28_393132	Listeria_monocytogenes_1639_v129	0.9402
Listeria_monocytogenes_L99_563174	Listeria_monocytogenes_1639_v129	0.9101
Listeria_monocytogenes_L312_882094	Listeria_monocytogenes_1639_v129	0.9691
Listeria_monocytogenes_J1816_930781	Listeria_monocytogenes_1639_v129	0.9482
Listeria_monocytogenes_J1-220_930782	Listeria_monocytogenes_1639_v129	0.9454
Listeria_monocytogenes_J0161_393130	Listeria_monocytogenes_1639_v129	0.9383
Listeria_monocytogenes_HCC23_552536	Listeria_monocytogenes_1639_v129	0.9094
Listeria_monocytogenes_FSL_R2-561_393126	Listeria_monocytogenes_1639_v129	0.9402
Listeria_monocytogenes_FSL_J1-208_393119	Listeria_monocytogenes_1639_v129	0.9171
Listeria_monocytogenes_FSL_J1-175_393118	Listeria_monocytogenes_1639_v129	0.9663
Listeria_monocytogenes_Finland_1998_393127	Listeria_monocytogenes_1639_v129	0.9589
Listeria_monocytogenes_EGD-e_169963_v1	Listeria_monocytogenes_1639_v129	0.9556
Listeria_monocytogenes_EGD-e_169963	Listeria_monocytogenes_1639_v129	0.956
Listeria_monocytogenes_EGD_1334565	Listeria_monocytogenes_1639_v129	0.9463
Listeria_monocytogenes_CFSAN002349_1299895	Listeria_monocytogenes_1639_v129	0.9703
Listeria_monocytogenes_ATCC_19117_882095_v1	Listeria_monocytogenes_1639_v129	0.965
Listeria_monocytogenes_ATCC_19117_882095	Listeria_monocytogenes_1639_v129	0.9646
Listeria_monocytogenes_6179_1288295	Listeria_monocytogenes_1639_v129	0.9213
Listeria_monocytogenes_1639_v9	Listeria_monocytogenes_1639_v129	0.9371
Listeria_monocytogenes_1639_v99	Listeria_monocytogenes_1639_v129	0.949
Listeria_monocytogenes_1639_v98	Listeria_monocytogenes_1639_v129	0.9352
Listeria_monocytogenes_1639_v97	Listeria_monocytogenes_1639_v129	0.9543
Listeria_monocytogenes_1639_v96	Listeria_monocytogenes_1639_v129	0.9482
Listeria_monocytogenes_1639_v95	Listeria_monocytogenes_1639_v129	0.9483
Listeria_monocytogenes_1639_v94	Listeria_monocytogenes_1639_v129	0.9486
Listeria_monocytogenes_1639_v93	Listeria_monocytogenes_1639_v129	0.9363
Listeria_monocytogenes_1639_v92	Listeria_monocytogenes_1639_v129	0.949
Listeria_monocytogenes_1639_v91	Listeria_monocytogenes_1639_v129	0.9481
Listeria_monocytogenes_1639_v90	Listeria_monocytogenes_1639_v129	0.9352
Listeria_monocytogenes_1639_v8	Listeria_monocytogenes_1639_v129	0.9204
Listeria_monocytogenes_1639_v89	Listeria_monocytogenes_1639_v129	0.9598
Listeria_monocytogenes_1639_v88	Listeria_monocytogenes_1639_v129	0.9392
Listeria_monocytogenes_1639_v87	Listeria_monocytogenes_1639_v129	0.9345
Listeria_monocytogenes_1639_v86	Listeria_monocytogenes_1639_v129	0.9341
Listeria_monocytogenes_1639_v85	Listeria_monocytogenes_1639_v129	0.9522
Listeria_monocytogenes_1639_v84	Listeria_monocytogenes_1639_v129	0.9398
Listeria_monocytogenes_1639_v83	Listeria_monocytogenes_1639_v129	0.954
Listeria_monocytogenes_1639_v82	Listeria_monocytogenes_1639_v129	0.9536
Listeria_monocytogenes_1639_v81	Listeria_monocytogenes_1639_v129	0.9392
Listeria_monocytogenes_1639_v80	Listeria_monocytogenes_1639_v129	0.9722
Listeria_monocytogenes_1639_v7	Listeria_monocytogenes_1639_v129	0.9204
Listeria_monocytogenes_1639_v79	Listeria_monocytogenes_1639_v129	0.9374
Listeria_monocytogenes_1639_v78	Listeria_monocytogenes_1639_v129	0.9385
Listeria_monocytogenes_1639_v77	Listeria_monocytogenes_1639_v129	0.9436
Listeria_monocytogenes_1639_v76	Listeria_monocytogenes_1639_v129	0.9333
Listeria_monocytogenes_1639_v75	Listeria_monocytogenes_1639_v129	0.9101
Listeria_monocytogenes_1639_v74	Listeria_monocytogenes_1639_v129	0.933
Listeria_monocytogenes_1639_v73	Listeria_monocytogenes_1639_v129	0.9356
Listeria_monocytogenes_1639_v72	Listeria_monocytogenes_1639_v129	0.952
Listeria_monocytogenes_1639_v71	Listeria_monocytogenes_1639_v129	0.9215
Listeria_monocytogenes_1639_v70	Listeria_monocytogenes_1639_v129	0.9377
Listeria_monocytogenes_1639_v6	Listeria_monocytogenes_1639_v129	0.9201
Listeria_monocytogenes_1639_v69	Listeria_monocytogenes_1639_v129	0.9537
Listeria_monocytogenes_1639_v68	Listeria_monocytogenes_1639_v129	0.9433
Listeria_monocytogenes_1639_v67	Listeria_monocytogenes_1639_v129	0.9559
Listeria_monocytogenes_1639_v66	Listeria_monocytogenes_1639_v129	0.943

Listeria_monocytogenes_1639_v65	Listeria_monocytogenes_1639_v129	0.935
Listeria_monocytogenes_1639_v64	Listeria_monocytogenes_1639_v129	0.9374
Listeria_monocytogenes_1639_v63	Listeria_monocytogenes_1639_v129	0.9675
Listeria_monocytogenes_1639_v62	Listeria_monocytogenes_1639_v129	0.9345
Listeria_monocytogenes_1639_v61	Listeria_monocytogenes_1639_v129	0.9346
Listeria_monocytogenes_1639_v60	Listeria_monocytogenes_1639_v129	0.9399
Listeria_monocytogenes_1639_v5	Listeria_monocytogenes_1639_v129	0.9317
Listeria_monocytogenes_1639_v59	Listeria_monocytogenes_1639_v129	0.9543
Listeria_monocytogenes_1639_v58	Listeria_monocytogenes_1639_v129	0.9574
Listeria_monocytogenes_1639_v57	Listeria_monocytogenes_1639_v129	0.9451
Listeria_monocytogenes_1639_v56	Listeria_monocytogenes_1639_v129	0.954
Listeria_monocytogenes_1639_v55	Listeria_monocytogenes_1639_v129	0.9408
Listeria_monocytogenes_1639_v54	Listeria_monocytogenes_1639_v129	0.9272
Listeria_monocytogenes_1639_v53	Listeria_monocytogenes_1639_v129	0.941
Listeria_monocytogenes_1639_v52	Listeria_monocytogenes_1639_v129	0.9492
Listeria_monocytogenes_1639_v51	Listeria_monocytogenes_1639_v129	0.9482
Listeria_monocytogenes_1639_v50	Listeria_monocytogenes_1639_v129	0.9315
Listeria_monocytogenes_1639_v4	Listeria_monocytogenes_1639_v129	0.9454
Listeria_monocytogenes_1639_v49	Listeria_monocytogenes_1639_v129	0.9548
Listeria_monocytogenes_1639_v48	Listeria_monocytogenes_1639_v129	0.9359
Listeria_monocytogenes_1639_v47	Listeria_monocytogenes_1639_v129	0.9319
Listeria_monocytogenes_1639_v46	Listeria_monocytogenes_1639_v129	0.9426
Listeria_monocytogenes_1639_v45	Listeria_monocytogenes_1639_v129	0.9229
Listeria_monocytogenes_1639_v44	Listeria_monocytogenes_1639_v129	0.9322
Listeria_monocytogenes_1639_v43	Listeria_monocytogenes_1639_v129	0.9322
Listeria_monocytogenes_1639_v42	Listeria_monocytogenes_1639_v129	0.9251
Listeria_monocytogenes_1639_v41	Listeria_monocytogenes_1639_v129	0.9086
Listeria_monocytogenes_1639_v40	Listeria_monocytogenes_1639_v129	0.9409
Listeria_monocytogenes_1639_v3	Listeria_monocytogenes_1639_v129	0.9458
Listeria_monocytogenes_1639_v39	Listeria_monocytogenes_1639_v129	0.9535
Listeria_monocytogenes_1639_v38	Listeria_monocytogenes_1639_v129	0.9392
Listeria_monocytogenes_1639_v37	Listeria_monocytogenes_1639_v129	0.9332
Listeria_monocytogenes_1639_v36	Listeria_monocytogenes_1639_v129	0.9269
Listeria_monocytogenes_1639_v35	Listeria_monocytogenes_1639_v129	0.9416
Listeria_monocytogenes_1639_v34	Listeria_monocytogenes_1639_v129	0.9352
Listeria_monocytogenes_1639_v33	Listeria_monocytogenes_1639_v129	0.9668
Listeria_monocytogenes_1639_v32	Listeria_monocytogenes_1639_v129	0.9321
Listeria_monocytogenes_1639_v31	Listeria_monocytogenes_1639_v129	0.9355
Listeria_monocytogenes_1639_v30	Listeria_monocytogenes_1639_v129	0.9599
Listeria_monocytogenes_1639_v2	Listeria_monocytogenes_1639_v129	0.9099
Listeria_monocytogenes_1639_v29	Listeria_monocytogenes_1639_v129	0.9269
Listeria_monocytogenes_1639_v28	Listeria_monocytogenes_1639_v129	0.9314
Listeria_monocytogenes_1639_v27	Listeria_monocytogenes_1639_v129	0.922
Listeria_monocytogenes_1639_v26	Listeria_monocytogenes_1639_v129	0.9217
Listeria_monocytogenes_1639_v25	Listeria_monocytogenes_1639_v129	0.9221
Listeria_monocytogenes_1639_v24	Listeria_monocytogenes_1639_v129	0.9454
Listeria_monocytogenes_1639_v23	Listeria_monocytogenes_1639_v129	0.945
Listeria_monocytogenes_1639_v22	Listeria_monocytogenes_1639_v129	0.9694
Listeria_monocytogenes_1639_v21	Listeria_monocytogenes_1639_v129	0.9446
Listeria_monocytogenes_1639_v20	Listeria_monocytogenes_1639_v129	0.94
Listeria_monocytogenes_1639_v1	Listeria_monocytogenes_1639_v129	0.9156
Listeria_monocytogenes_1639_v19	Listeria_monocytogenes_1639_v129	0.9396
Listeria_monocytogenes_1639_v198	Listeria_monocytogenes_1639_v129	0.9063
Listeria_monocytogenes_1639_v197	Listeria_monocytogenes_1639_v129	0.9426
Listeria_monocytogenes_1639_v196	Listeria_monocytogenes_1639_v129	0.929
Listeria_monocytogenes_1639_v195	Listeria_monocytogenes_1639_v129	0.9507
Listeria_monocytogenes_1639_v194	Listeria_monocytogenes_1639_v129	0.95
Listeria_monocytogenes_1639_v193	Listeria_monocytogenes_1639_v129	0.95
Listeria_monocytogenes_1639_v192	Listeria_monocytogenes_1639_v129	0.9093
Listeria_monocytogenes_1639_v191	Listeria_monocytogenes_1639_v129	0.9572
Listeria_monocytogenes_1639_v190	Listeria_monocytogenes_1639_v129	0.9588
Listeria_monocytogenes_1639_v18	Listeria_monocytogenes_1639_v129	0.9394
Listeria_monocytogenes_1639_v189	Listeria_monocytogenes_1639_v129	0.9577
Listeria_monocytogenes_1639_v188	Listeria_monocytogenes_1639_v129	0.9599
Listeria_monocytogenes_1639_v187	Listeria_monocytogenes_1639_v129	0.9588
Listeria_monocytogenes_1639_v186	Listeria_monocytogenes_1639_v129	0.9588
Listeria_monocytogenes_1639_v185	Listeria_monocytogenes_1639_v129	0.9592
Listeria_monocytogenes_1639_v184	Listeria_monocytogenes_1639_v129	0.958
Listeria_monocytogenes_1639_v183	Listeria_monocytogenes_1639_v129	0.9533
Listeria_monocytogenes_1639_v182	Listeria_monocytogenes_1639_v129	0.9556
Listeria_monocytogenes_1639_v181	Listeria_monocytogenes_1639_v129	0.9304
Listeria_monocytogenes_1639_v180	Listeria_monocytogenes_1639_v129	0.9304
Listeria_monocytogenes_1639_v17	Listeria_monocytogenes_1639_v129	0.9398
Listeria_monocytogenes_1639_v179	Listeria_monocytogenes_1639_v129	0.9404
Listeria_monocytogenes_1639_v178	Listeria_monocytogenes_1639_v129	0.9588
Listeria_monocytogenes_1639_v177	Listeria_monocytogenes_1639_v129	0.968
Listeria_monocytogenes_1639_v176	Listeria_monocytogenes_1639_v129	0.945
Listeria_monocytogenes_1639_v175	Listeria_monocytogenes_1639_v129	0.9484
Listeria_monocytogenes_1639_v174	Listeria_monocytogenes_1639_v129	0.9585
Listeria_monocytogenes_1639_v173	Listeria_monocytogenes_1639_v129	0.9748
Listeria_monocytogenes_1639_v172	Listeria_monocytogenes_1639_v129	0.9448
Listeria_monocytogenes_1639_v171	Listeria_monocytogenes_1639_v129	0.9438
Listeria_monocytogenes_1639_v170	Listeria_monocytogenes_1639_v129	0.9521
Listeria_monocytogenes_1639_v16	Listeria_monocytogenes_1639_v129	0.9184
Listeria_monocytogenes_1639_v169	Listeria_monocytogenes_1639_v129	0.9507
Listeria_monocytogenes_1639_v168	Listeria_monocytogenes_1639_v129	0.9504
Listeria_monocytogenes_1639_v167	Listeria_monocytogenes_1639_v129	0.9425
Listeria_monocytogenes_1639_v166	Listeria_monocytogenes_1639_v129	0.9508
Listeria_monocytogenes_1639_v165	Listeria_monocytogenes_1639_v129	0.9503
Listeria_monocytogenes_1639_v164	Listeria_monocytogenes_1639_v129	0.9675
Listeria_monocytogenes_1639_v163	Listeria_monocytogenes_1639_v129	0.9691

Listeria_monocytogenes_1639_v162	Listeria_monocytogenes_1639_v129	0.9675
Listeria_monocytogenes_1639_v161	Listeria_monocytogenes_1639_v129	0.9675
Listeria_monocytogenes_1639_v160	Listeria_monocytogenes_1639_v129	0.9672
Listeria_monocytogenes_1639_v15	Listeria_monocytogenes_1639_v129	0.9521
Listeria_monocytogenes_1639_v159	Listeria_monocytogenes_1639_v129	0.9684
Listeria_monocytogenes_1639_v158	Listeria_monocytogenes_1639_v129	0.9679
Listeria_monocytogenes_1639_v157	Listeria_monocytogenes_1639_v129	0.9679
Listeria_monocytogenes_1639_v156	Listeria_monocytogenes_1639_v129	0.9351
Listeria_monocytogenes_1639_v155	Listeria_monocytogenes_1639_v129	0.9477
Listeria_monocytogenes_1639_v154	Listeria_monocytogenes_1639_v129	0.9508
Listeria_monocytogenes_1639_v153	Listeria_monocytogenes_1639_v129	0.9488
Listeria_monocytogenes_1639_v152	Listeria_monocytogenes_1639_v129	0.9684
Listeria_monocytogenes_1639_v151	Listeria_monocytogenes_1639_v129	0.9684
Listeria_monocytogenes_1639_v150	Listeria_monocytogenes_1639_v129	0.9481
Listeria_monocytogenes_1639_v14	Listeria_monocytogenes_1639_v129	0.9589
Listeria_monocytogenes_1639_v149	Listeria_monocytogenes_1639_v129	0.9467
Listeria_monocytogenes_1639_v148	Listeria_monocytogenes_1639_v129	0.9676
Listeria_monocytogenes_1639_v147	Listeria_monocytogenes_1639_v129	0.9533
Listeria_monocytogenes_1639_v146	Listeria_monocytogenes_1639_v129	0.9616
Listeria_monocytogenes_1639_v145	Listeria_monocytogenes_1639_v129	0.9544
Listeria_monocytogenes_1639_v144	Listeria_monocytogenes_1639_v129	0.9683
Listeria_monocytogenes_1639_v143	Listeria_monocytogenes_1639_v129	0.9609
Listeria_monocytogenes_1639_v142	Listeria_monocytogenes_1639_v129	0.9687
Listeria_monocytogenes_1639_v141	Listeria_monocytogenes_1639_v129	0.9744
Listeria_monocytogenes_1639_v140	Listeria_monocytogenes_1639_v129	0.9694
Listeria_monocytogenes_1639_v13	Listeria_monocytogenes_1639_v129	0.9517
Listeria_monocytogenes_1639_v139	Listeria_monocytogenes_1639_v129	0.9652
Listeria_monocytogenes_1639_v138	Listeria_monocytogenes_1639_v129	0.9706
Listeria_monocytogenes_1639_v137	Listeria_monocytogenes_1639_v129	0.9706
Listeria_monocytogenes_1639_v136	Listeria_monocytogenes_1639_v129	0.9491
Listeria_monocytogenes_1639_v135	Listeria_monocytogenes_1639_v129	0.923
Listeria_monocytogenes_1639_v134	Listeria_monocytogenes_1639_v129	0.9638
Listeria_monocytogenes_1639_v133	Listeria_monocytogenes_1639_v129	0.9501
Listeria_monocytogenes_1639_v132	Listeria_monocytogenes_1639_v129	0.9212
Listeria_monocytogenes_1639_v131	Listeria_monocytogenes_1639_v129	0.9569
Listeria_monocytogenes_1639_v130	Listeria_monocytogenes_1639_v129	0.9413
Listeria_monocytogenes_1639_v12	Listeria_monocytogenes_1639_v129	0.9211
Listeria_monocytogenes_1639_v129	Listeria_monocytogenes_1639_v129	0.0661
Listeria_welshimeri_1643	Listeria_monocytogenes_1639_v12	0.8533
Listeria_welshimeri_serovar_6b_str_SLCC5334_386043	Listeria_monocytogenes_1639_v12	0.853
Listeria_seeligeri_serovar_1_2b_str_SLCC3954_683837	Listeria_monocytogenes_1639_v12	0.8577
Listeria_seeligeri_FSL_S4-171_702452	Listeria_monocytogenes_1639_v12	0.8011
Listeria_seeligeri_FSL_N1-067_702453	Listeria_monocytogenes_1639_v12	0.8233
Listeria_seeligeri_1640_v1	Listeria_monocytogenes_1639_v12	0.8605
Listeria_seeligeri_1640	Listeria_monocytogenes_1639_v12	0.8532
Listeria_monocytogenes_WSLC1042_1457188	Listeria_monocytogenes_1639_v12	0.9056
Listeria_monocytogenes_WSLC1001_1457187	Listeria_monocytogenes_1639_v12	0.9378
Listeria_monocytogenes_SLCC7179_879090	Listeria_monocytogenes_1639_v12	0.9291
Listeria_monocytogenes_SLCC5850_882096	Listeria_monocytogenes_1639_v12	0.9353
Listeria_monocytogenes_SLCC2755_932919	Listeria_monocytogenes_1639_v12	0.9322
Listeria_monocytogenes_SLCC2540_879089	Listeria_monocytogenes_1639_v12	0.9239
Listeria_monocytogenes_SLCC2479_882020	Listeria_monocytogenes_1639_v12	0.9706
Listeria_monocytogenes_SLCC2378_879088	Listeria_monocytogenes_1639_v12	0.9066
Listeria_monocytogenes_SLCC2376_882097	Listeria_monocytogenes_1639_v12	0.9113
Listeria_monocytogenes_SLCC2372_932920	Listeria_monocytogenes_1639_v12	0.9726
Listeria_monocytogenes_serotype_4b_str_LL195_1230340	Listeria_monocytogenes_1639_v12	0.9104
Listeria_monocytogenes_serotype_4b_str_LIS0087_1134669	Listeria_monocytogenes_1639_v12	0.9155
Listeria_monocytogenes_serotype_4b_str_F2365_265669	Listeria_monocytogenes_1639_v12	0.9104
Listeria_monocytogenes_serotype_4b_str_CLIP_80459_568819	Listeria_monocytogenes_1639_v12	0.9129
Listeria_monocytogenes_serotype_4b_str_81-0861_1196161	Listeria_monocytogenes_1639_v12	0.9021
Listeria_monocytogenes_serotype_4b_str_81-0592_1196160	Listeria_monocytogenes_1639_v12	0.9031
Listeria_monocytogenes_serotype_4b_str_81-0558_1196159	Listeria_monocytogenes_1639_v12	0.9035
Listeria_monocytogenes_serotype_4b_str_10-0809_1196162	Listeria_monocytogenes_1639_v12	0.9031
Listeria_monocytogenes_serotype_4b_str_02-6680_1196175	Listeria_monocytogenes_1639_v12	0.9105
Listeria_monocytogenes_serotype_4b_str_02-6679_1196174	Listeria_monocytogenes_1639_v12	0.9105
Listeria_monocytogenes_serotype_4b_str_02-1792_1196173	Listeria_monocytogenes_1639_v12	0.9053
Listeria_monocytogenes_serotype_4b_str_02-1289_1196172	Listeria_monocytogenes_1639_v12	0.9053
Listeria_monocytogenes_serotype_4b_str_02-1103_1196171	Listeria_monocytogenes_1639_v12	0.9053
Listeria_monocytogenes_serotype_3c_str_10-5027_1196195	Listeria_monocytogenes_1639_v12	0.9669
Listeria_monocytogenes_serotype_1_2c_str_10-5026_1196194	Listeria_monocytogenes_1639_v12	0.968
Listeria_monocytogenes_serotype_1_2c_str_10-5025_1196193	Listeria_monocytogenes_1639_v12	0.968
Listeria_monocytogenes_serotype_1_2b_str_10-0811_1196164	Listeria_monocytogenes_1639_v12	0.9301
Listeria_monocytogenes_serotype_1_2b_str_10-0810_1196163	Listeria_monocytogenes_1639_v12	0.9301
Listeria_monocytogenes_serotype_1_2a_str_99-6370_1196179	Listeria_monocytogenes_1639_v12	0.9397
Listeria_monocytogenes_serotype_1_2a_str_98-2035_1196178	Listeria_monocytogenes_1639_v12	0.9397
Listeria_monocytogenes_serotype_1_2a_str_95-0093_1196177	Listeria_monocytogenes_1639_v12	0.9346
Listeria_monocytogenes_serotype_1_2a_str_88-0478_1196176	Listeria_monocytogenes_1639_v12	0.9368
Listeria_monocytogenes_serotype_1_2a_str_10-5024_1196192	Listeria_monocytogenes_1639_v12	0.935
Listeria_monocytogenes_serotype_1_2a_str_10-4758_1196168	Listeria_monocytogenes_1639_v12	0.938
Listeria_monocytogenes_serotype_1_2a_str_10-4754_1196167	Listeria_monocytogenes_1639_v12	0.9383
Listeria_monocytogenes_serotype_1_2a_str_10-1321_1196191	Listeria_monocytogenes_1639_v12	0.935
Listeria_monocytogenes_serotype_1_2a_str_10-1047_1196190	Listeria_monocytogenes_1639_v12	0.9346
Listeria_monocytogenes_serotype_1_2a_str_10-1046_1196189	Listeria_monocytogenes_1639_v12	0.9346
Listeria_monocytogenes_serotype_1_2a_str_10-0934_1196170	Listeria_monocytogenes_1639_v12	0.9349
Listeria_monocytogenes_serotype_1_2a_str_10-0933_1196169	Listeria_monocytogenes_1639_v12	0.9349
Listeria_monocytogenes_serotype_1_2a_str_10-0815_1196188	Listeria_monocytogenes_1639_v12	0.9385
Listeria_monocytogenes_serotype_1_2a_str_10-0814_1196187	Listeria_monocytogenes_1639_v12	0.9354
Listeria_monocytogenes_serotype_1_2a_str_10-0813_1196166	Listeria_monocytogenes_1639_v12	0.9365
Listeria_monocytogenes_serotype_1_2a_str_10-0812_1196165	Listeria_monocytogenes_1639_v12	0.9365
Listeria_monocytogenes_serotype_1_2a_str_08-7669_1196186	Listeria_monocytogenes_1639_v12	0.9292
Listeria_monocytogenes_serotype_1_2a_str_08-7374_1196185	Listeria_monocytogenes_1639_v12	0.935

Listeria_monocytogenes_serotype_1_2a_str_08-6997_1196184	Listeria_monocytogenes_1639_v12	0.9385
Listeria_monocytogenes_serotype_1_2a_str_08-6569_1196183	Listeria_monocytogenes_1639_v12	0.9385
Listeria_monocytogenes_serotype_1_2a_str_08-6056_1196182	Listeria_monocytogenes_1639_v12	0.9385
Listeria_monocytogenes_serotype_1_2a_str_04-5457_1196181	Listeria_monocytogenes_1639_v12	0.935
Listeria_monocytogenes_serotype_1_2a_str_02-5993_1196180	Listeria_monocytogenes_1639_v12	0.935
Listeria_monocytogenes_R479a_1437838	Listeria_monocytogenes_1639_v12	0.943
Listeria_monocytogenes_N53-1_1234142	Listeria_monocytogenes_1639_v12	0.8872
Listeria_monocytogenes_M7_1030009	Listeria_monocytogenes_1639_v12	0.8947
Listeria_monocytogenes_LO28_393132	Listeria_monocytogenes_1639_v12	0.9626
Listeria_monocytogenes_L99_563174	Listeria_monocytogenes_1639_v12	0.895
Listeria_monocytogenes_L312_882094	Listeria_monocytogenes_1639_v12	0.9129
Listeria_monocytogenes_J1816_930781	Listeria_monocytogenes_1639_v12	0.9061
Listeria_monocytogenes_J1-220_930782	Listeria_monocytogenes_1639_v12	0.9075
Listeria_monocytogenes_J0161_393130	Listeria_monocytogenes_1639_v12	0.926
Listeria_monocytogenes_HCC23_552536	Listeria_monocytogenes_1639_v12	0.8947
Listeria_monocytogenes_FSL_R2-561_393126	Listeria_monocytogenes_1639_v12	0.9677
Listeria_monocytogenes_FSL_J1-208_393119	Listeria_monocytogenes_1639_v12	0.8786
Listeria_monocytogenes_FSL_J1-175_393118	Listeria_monocytogenes_1639_v12	0.9239
Listeria_monocytogenes_Finland_1998_393127	Listeria_monocytogenes_1639_v12	0.9286
Listeria_monocytogenes_EGD-e_169963_v1	Listeria_monocytogenes_1639_v12	0.9494
Listeria_monocytogenes_EGD-e_169963	Listeria_monocytogenes_1639_v12	0.9498
Listeria_monocytogenes_EGD_1334565	Listeria_monocytogenes_1639_v12	0.936
Listeria_monocytogenes_CFSAN002349_1299895	Listeria_monocytogenes_1639_v12	0.9136
Listeria_monocytogenes_ATCC_19117_882095_v1	Listeria_monocytogenes_1639_v12	0.9078
Listeria_monocytogenes_ATCC_19117_882095	Listeria_monocytogenes_1639_v12	0.9074
Listeria_monocytogenes_6179_1288295	Listeria_monocytogenes_1639_v12	0.9334
Listeria_monocytogenes_1639_v9	Listeria_monocytogenes_1639_v12	0.9129
Listeria_monocytogenes_1639_v99	Listeria_monocytogenes_1639_v12	0.9155
Listeria_monocytogenes_1639_v98	Listeria_monocytogenes_1639_v12	0.936
Listeria_monocytogenes_1639_v97	Listeria_monocytogenes_1639_v12	0.9094
Listeria_monocytogenes_1639_v96	Listeria_monocytogenes_1639_v12	0.9152
Listeria_monocytogenes_1639_v95	Listeria_monocytogenes_1639_v12	0.9152
Listeria_monocytogenes_1639_v94	Listeria_monocytogenes_1639_v12	0.9155
Listeria_monocytogenes_1639_v93	Listeria_monocytogenes_1639_v12	0.9324
Listeria_monocytogenes_1639_v92	Listeria_monocytogenes_1639_v12	0.9159
Listeria_monocytogenes_1639_v91	Listeria_monocytogenes_1639_v12	0.9099
Listeria_monocytogenes_1639_v90	Listeria_monocytogenes_1639_v12	0.936
Listeria_monocytogenes_1639_v8	Listeria_monocytogenes_1639_v12	0.9522
Listeria_monocytogenes_1639_v89	Listeria_monocytogenes_1639_v12	0.9108
Listeria_monocytogenes_1639_v88	Listeria_monocytogenes_1639_v12	0.9413
Listeria_monocytogenes_1639_v87	Listeria_monocytogenes_1639_v12	0.9339
Listeria_monocytogenes_1639_v86	Listeria_monocytogenes_1639_v12	0.9336
Listeria_monocytogenes_1639_v85	Listeria_monocytogenes_1639_v12	0.9026
Listeria_monocytogenes_1639_v84	Listeria_monocytogenes_1639_v12	0.9397
Listeria_monocytogenes_1639_v83	Listeria_monocytogenes_1639_v12	0.9037
Listeria_monocytogenes_1639_v82	Listeria_monocytogenes_1639_v12	0.9034
Listeria_monocytogenes_1639_v81	Listeria_monocytogenes_1639_v12	0.9402
Listeria_monocytogenes_1639_v80	Listeria_monocytogenes_1639_v12	0.9237
Listeria_monocytogenes_1639_v7	Listeria_monocytogenes_1639_v12	0.9522
Listeria_monocytogenes_1639_v79	Listeria_monocytogenes_1639_v12	0.9345
Listeria_monocytogenes_1639_v78	Listeria_monocytogenes_1639_v12	0.9392
Listeria_monocytogenes_1639_v77	Listeria_monocytogenes_1639_v12	0.9283
Listeria_monocytogenes_1639_v76	Listeria_monocytogenes_1639_v12	0.9421
Listeria_monocytogenes_1639_v75	Listeria_monocytogenes_1639_v12	0.895
Listeria_monocytogenes_1639_v74	Listeria_monocytogenes_1639_v12	0.9252
Listeria_monocytogenes_1639_v73	Listeria_monocytogenes_1639_v12	0.9711
Listeria_monocytogenes_1639_v72	Listeria_monocytogenes_1639_v12	0.9022
Listeria_monocytogenes_1639_v71	Listeria_monocytogenes_1639_v12	0.9254
Listeria_monocytogenes_1639_v70	Listeria_monocytogenes_1639_v12	0.9725
Listeria_monocytogenes_1639_v6	Listeria_monocytogenes_1639_v12	0.9518
Listeria_monocytogenes_1639_v69	Listeria_monocytogenes_1639_v12	0.9031
Listeria_monocytogenes_1639_v68	Listeria_monocytogenes_1639_v12	0.9256
Listeria_monocytogenes_1639_v67	Listeria_monocytogenes_1639_v12	0.9271
Listeria_monocytogenes_1639_v66	Listeria_monocytogenes_1639_v12	0.938
Listeria_monocytogenes_1639_v65	Listeria_monocytogenes_1639_v12	0.941
Listeria_monocytogenes_1639_v64	Listeria_monocytogenes_1639_v12	0.9396
Listeria_monocytogenes_1639_v63	Listeria_monocytogenes_1639_v12	0.9322
Listeria_monocytogenes_1639_v62	Listeria_monocytogenes_1639_v12	0.9438
Listeria_monocytogenes_1639_v61	Listeria_monocytogenes_1639_v12	0.9366
Listeria_monocytogenes_1639_v60	Listeria_monocytogenes_1639_v12	0.9278
Listeria_monocytogenes_1639_v5	Listeria_monocytogenes_1639_v12	0.9142
Listeria_monocytogenes_1639_v59	Listeria_monocytogenes_1639_v12	0.9088
Listeria_monocytogenes_1639_v58	Listeria_monocytogenes_1639_v12	0.9084
Listeria_monocytogenes_1639_v57	Listeria_monocytogenes_1639_v12	0.904
Listeria_monocytogenes_1639_v56	Listeria_monocytogenes_1639_v12	0.9092
Listeria_monocytogenes_1639_v55	Listeria_monocytogenes_1639_v12	0.909
Listeria_monocytogenes_1639_v54	Listeria_monocytogenes_1639_v12	0.903
Listeria_monocytogenes_1639_v53	Listeria_monocytogenes_1639_v12	0.919
Listeria_monocytogenes_1639_v52	Listeria_monocytogenes_1639_v12	0.908
Listeria_monocytogenes_1639_v51	Listeria_monocytogenes_1639_v12	0.9057
Listeria_monocytogenes_1639_v50	Listeria_monocytogenes_1639_v12	0.9339
Listeria_monocytogenes_1639_v4	Listeria_monocytogenes_1639_v12	0.926
Listeria_monocytogenes_1639_v49	Listeria_monocytogenes_1639_v12	0.9036
Listeria_monocytogenes_1639_v48	Listeria_monocytogenes_1639_v12	0.9707
Listeria_monocytogenes_1639_v47	Listeria_monocytogenes_1639_v12	0.9347
Listeria_monocytogenes_1639_v46	Listeria_monocytogenes_1639_v12	0.9275
Listeria_monocytogenes_1639_v45	Listeria_monocytogenes_1639_v12	0.9346
Listeria_monocytogenes_1639_v44	Listeria_monocytogenes_1639_v12	0.935
Listeria_monocytogenes_1639_v43	Listeria_monocytogenes_1639_v12	0.935
Listeria_monocytogenes_1639_v42	Listeria_monocytogenes_1639_v12	0.9274
Listeria_monocytogenes_1639_v41	Listeria_monocytogenes_1639_v12	0.9254

Listeria_monocytogenes_1639_v40	Listeria_monocytogenes_1639_v12	0.9702
Listeria_monocytogenes_1639_v3	Listeria_monocytogenes_1639_v12	0.9267
Listeria_monocytogenes_1639_v39	Listeria_monocytogenes_1639_v12	0.9264
Listeria_monocytogenes_1639_v38	Listeria_monocytogenes_1639_v12	0.9547
Listeria_monocytogenes_1639_v37	Listeria_monocytogenes_1639_v12	0.9535
Listeria_monocytogenes_1639_v36	Listeria_monocytogenes_1639_v12	0.9471
Listeria_monocytogenes_1639_v35	Listeria_monocytogenes_1639_v12	0.9666
Listeria_monocytogenes_1639_v34	Listeria_monocytogenes_1639_v12	0.9353
Listeria_monocytogenes_1639_v33	Listeria_monocytogenes_1639_v12	0.9301
Listeria_monocytogenes_1639_v32	Listeria_monocytogenes_1639_v12	0.9622
Listeria_monocytogenes_1639_v31	Listeria_monocytogenes_1639_v12	0.9356
Listeria_monocytogenes_1639_v30	Listeria_monocytogenes_1639_v12	0.913
Listeria_monocytogenes_1639_v2	Listeria_monocytogenes_1639_v12	0.9082
Listeria_monocytogenes_1639_v29	Listeria_monocytogenes_1639_v12	0.9875
Listeria_monocytogenes_1639_v28	Listeria_monocytogenes_1639_v12	0.9813
Listeria_monocytogenes_1639_v27	Listeria_monocytogenes_1639_v12	0.9772
Listeria_monocytogenes_1639_v26	Listeria_monocytogenes_1639_v12	0.9761
Listeria_monocytogenes_1639_v25	Listeria_monocytogenes_1639_v12	0.9769
Listeria_monocytogenes_1639_v24	Listeria_monocytogenes_1639_v12	0.906
Listeria_monocytogenes_1639_v23	Listeria_monocytogenes_1639_v12	0.9057
Listeria_monocytogenes_1639_v22	Listeria_monocytogenes_1639_v12	0.9271
Listeria_monocytogenes_1639_v21	Listeria_monocytogenes_1639_v12	0.9057
Listeria_monocytogenes_1639_v20	Listeria_monocytogenes_1639_v12	0.932
Listeria_monocytogenes_1639_v1	Listeria_monocytogenes_1639_v12	0.9371
Listeria_monocytogenes_1639_v19	Listeria_monocytogenes_1639_v12	0.9316
Listeria_monocytogenes_1639_v198	Listeria_monocytogenes_1639_v12	0.8887
Listeria_monocytogenes_1639_v197	Listeria_monocytogenes_1639_v12	0.937
Listeria_monocytogenes_1639_v196	Listeria_monocytogenes_1639_v12	0.9193
Listeria_monocytogenes_1639_v195	Listeria_monocytogenes_1639_v12	0.9138
Listeria_monocytogenes_1639_v194	Listeria_monocytogenes_1639_v12	0.9113
Listeria_monocytogenes_1639_v193	Listeria_monocytogenes_1639_v12	0.9117
Listeria_monocytogenes_1639_v192	Listeria_monocytogenes_1639_v12	0.8744
Listeria_monocytogenes_1639_v191	Listeria_monocytogenes_1639_v12	0.9322
Listeria_monocytogenes_1639_v190	Listeria_monocytogenes_1639_v12	0.9334
Listeria_monocytogenes_1639_v18	Listeria_monocytogenes_1639_v12	0.9006
Listeria_monocytogenes_1639_v189	Listeria_monocytogenes_1639_v12	0.9326
Listeria_monocytogenes_1639_v188	Listeria_monocytogenes_1639_v12	0.9347
Listeria_monocytogenes_1639_v187	Listeria_monocytogenes_1639_v12	0.9333
Listeria_monocytogenes_1639_v186	Listeria_monocytogenes_1639_v12	0.9337
Listeria_monocytogenes_1639_v185	Listeria_monocytogenes_1639_v12	0.9337
Listeria_monocytogenes_1639_v184	Listeria_monocytogenes_1639_v12	0.933
Listeria_monocytogenes_1639_v183	Listeria_monocytogenes_1639_v12	0.9296
Listeria_monocytogenes_1639_v182	Listeria_monocytogenes_1639_v12	0.9317
Listeria_monocytogenes_1639_v181	Listeria_monocytogenes_1639_v12	0.8953
Listeria_monocytogenes_1639_v180	Listeria_monocytogenes_1639_v12	0.8953
Listeria_monocytogenes_1639_v17	Listeria_monocytogenes_1639_v12	0.901
Listeria_monocytogenes_1639_v179	Listeria_monocytogenes_1639_v12	0.9373
Listeria_monocytogenes_1639_v178	Listeria_monocytogenes_1639_v12	0.9308
Listeria_monocytogenes_1639_v177	Listeria_monocytogenes_1639_v12	0.9124
Listeria_monocytogenes_1639_v176	Listeria_monocytogenes_1639_v12	0.9062
Listeria_monocytogenes_1639_v175	Listeria_monocytogenes_1639_v12	0.9395
Listeria_monocytogenes_1639_v174	Listeria_monocytogenes_1639_v12	0.9331
Listeria_monocytogenes_1639_v173	Listeria_monocytogenes_1639_v12	0.9208
Listeria_monocytogenes_1639_v172	Listeria_monocytogenes_1639_v12	0.9086
Listeria_monocytogenes_1639_v171	Listeria_monocytogenes_1639_v12	0.9439
Listeria_monocytogenes_1639_v170	Listeria_monocytogenes_1639_v12	0.9401
Listeria_monocytogenes_1639_v16	Listeria_monocytogenes_1639_v12	0.9377
Listeria_monocytogenes_1639_v169	Listeria_monocytogenes_1639_v12	0.9405
Listeria_monocytogenes_1639_v168	Listeria_monocytogenes_1639_v12	0.9254
Listeria_monocytogenes_1639_v167	Listeria_monocytogenes_1639_v12	0.9312
Listeria_monocytogenes_1639_v166	Listeria_monocytogenes_1639_v12	0.9253
Listeria_monocytogenes_1639_v165	Listeria_monocytogenes_1639_v12	0.9405
Listeria_monocytogenes_1639_v164	Listeria_monocytogenes_1639_v12	0.9099
Listeria_monocytogenes_1639_v163	Listeria_monocytogenes_1639_v12	0.9226
Listeria_monocytogenes_1639_v162	Listeria_monocytogenes_1639_v12	0.9099
Listeria_monocytogenes_1639_v161	Listeria_monocytogenes_1639_v12	0.9111
Listeria_monocytogenes_1639_v160	Listeria_monocytogenes_1639_v12	0.9108
Listeria_monocytogenes_1639_v15	Listeria_monocytogenes_1639_v12	0.9166
Listeria_monocytogenes_1639_v159	Listeria_monocytogenes_1639_v12	0.9124
Listeria_monocytogenes_1639_v158	Listeria_monocytogenes_1639_v12	0.9104
Listeria_monocytogenes_1639_v157	Listeria_monocytogenes_1639_v12	0.9104
Listeria_monocytogenes_1639_v156	Listeria_monocytogenes_1639_v12	0.92
Listeria_monocytogenes_1639_v155	Listeria_monocytogenes_1639_v12	0.937
Listeria_monocytogenes_1639_v154	Listeria_monocytogenes_1639_v12	0.9385
Listeria_monocytogenes_1639_v153	Listeria_monocytogenes_1639_v12	0.934
Listeria_monocytogenes_1639_v152	Listeria_monocytogenes_1639_v12	0.9124
Listeria_monocytogenes_1639_v151	Listeria_monocytogenes_1639_v12	0.9124
Listeria_monocytogenes_1639_v150	Listeria_monocytogenes_1639_v12	0.9362
Listeria_monocytogenes_1639_v14	Listeria_monocytogenes_1639_v12	0.9311
Listeria_monocytogenes_1639_v149	Listeria_monocytogenes_1639_v12	0.9364
Listeria_monocytogenes_1639_v148	Listeria_monocytogenes_1639_v12	0.9117
Listeria_monocytogenes_1639_v147	Listeria_monocytogenes_1639_v12	0.9024
Listeria_monocytogenes_1639_v146	Listeria_monocytogenes_1639_v12	0.935
Listeria_monocytogenes_1639_v145	Listeria_monocytogenes_1639_v12	0.9278
Listeria_monocytogenes_1639_v144	Listeria_monocytogenes_1639_v12	0.9122
Listeria_monocytogenes_1639_v143	Listeria_monocytogenes_1639_v12	0.9333
Listeria_monocytogenes_1639_v142	Listeria_monocytogenes_1639_v12	0.9126
Listeria_monocytogenes_1639_v141	Listeria_monocytogenes_1639_v12	0.9228
Listeria_monocytogenes_1639_v140	Listeria_monocytogenes_1639_v12	0.9133
Listeria_monocytogenes_1639_v13	Listeria_monocytogenes_1639_v12	0.9166
Listeria_monocytogenes_1639_v139	Listeria_monocytogenes_1639_v12	0.9115

Listeria_monocytogenes_1639_v138	Listeria_monocytogenes_1639_v12	0.9148
Listeria_monocytogenes_1639_v137	Listeria_monocytogenes_1639_v12	0.9144
Listeria_monocytogenes_1639_v136	Listeria_monocytogenes_1639_v12	0.9391
Listeria_monocytogenes_1639_v135	Listeria_monocytogenes_1639_v12	0.89
Listeria_monocytogenes_1639_v134	Listeria_monocytogenes_1639_v12	0.9063
Listeria_monocytogenes_1639_v133	Listeria_monocytogenes_1639_v12	0.9341
Listeria_monocytogenes_1639_v132	Listeria_monocytogenes_1639_v12	0.8884
Listeria_monocytogenes_1639_v131	Listeria_monocytogenes_1639_v12	0.9089
Listeria_monocytogenes_1639_v130	Listeria_monocytogenes_1639_v12	0.9666
Listeria_monocytogenes_1639_v12	Listeria_monocytogenes_1639_v12	0.0743
Listeria_welshimeri_1643	Listeria_monocytogenes_1639_v130	0.8681
Listeria_welshimeri_serovar_6b_str_SLCC5334_386043	Listeria_monocytogenes_1639_v130	0.8674
Listeria_seeligeri_serovar_1_2b_str_SLCC3954_683837	Listeria_monocytogenes_1639_v130	0.8747
Listeria_seeligeri_FSL_S4-171_702452	Listeria_monocytogenes_1639_v130	0.8103
Listeria_seeligeri_FSL_N1-067_702453	Listeria_monocytogenes_1639_v130	0.8291
Listeria_seeligeri_1640_v1	Listeria_monocytogenes_1639_v130	0.8805
Listeria_seeligeri_1640	Listeria_monocytogenes_1639_v130	0.8682
Listeria_monocytogenes_WSLC1042_1457188	Listeria_monocytogenes_1639_v130	0.9256
Listeria_monocytogenes_WSLC1001_1457187	Listeria_monocytogenes_1639_v130	0.9615
Listeria_monocytogenes_SLCC7179_879090	Listeria_monocytogenes_1639_v130	0.9479
Listeria_monocytogenes_SLCC5850_882096	Listeria_monocytogenes_1639_v130	0.9545
Listeria_monocytogenes_SLCC2755_932919	Listeria_monocytogenes_1639_v130	0.9389
Listeria_monocytogenes_SLCC2540_879089	Listeria_monocytogenes_1639_v130	0.9427
Listeria_monocytogenes_SLCC2479_882020	Listeria_monocytogenes_1639_v130	0.981
Listeria_monocytogenes_SLCC2378_879088	Listeria_monocytogenes_1639_v130	0.927
Listeria_monocytogenes_SLCC2376_882097	Listeria_monocytogenes_1639_v130	0.9319
Listeria_monocytogenes_SLCC2372_932920	Listeria_monocytogenes_1639_v130	0.9904
Listeria_monocytogenes_serotype_4b_str_LL195_1230340	Listeria_monocytogenes_1639_v130	0.9311
Listeria_monocytogenes_serotype_4b_str_LIS0087_1134669	Listeria_monocytogenes_1639_v130	0.9352
Listeria_monocytogenes_serotype_4b_str_F2365_265669	Listeria_monocytogenes_1639_v130	0.9311
Listeria_monocytogenes_serotype_4b_str_CLIP_80459_568819	Listeria_monocytogenes_1639_v130	0.9337
Listeria_monocytogenes_serotype_4b_str_81-0861_1196161	Listeria_monocytogenes_1639_v130	0.9182
Listeria_monocytogenes_serotype_4b_str_81-0592_1196160	Listeria_monocytogenes_1639_v130	0.9192
Listeria_monocytogenes_serotype_4b_str_81-0558_1196159	Listeria_monocytogenes_1639_v130	0.9199
Listeria_monocytogenes_serotype_4b_str_10-0809_1196162	Listeria_monocytogenes_1639_v130	0.9192
Listeria_monocytogenes_serotype_4b_str_02-6680_1196175	Listeria_monocytogenes_1639_v130	0.9304
Listeria_monocytogenes_serotype_4b_str_02-6679_1196174	Listeria_monocytogenes_1639_v130	0.9304
Listeria_monocytogenes_serotype_4b_str_02-1792_1196173	Listeria_monocytogenes_1639_v130	0.9203
Listeria_monocytogenes_serotype_4b_str_02-1289_1196172	Listeria_monocytogenes_1639_v130	0.9203
Listeria_monocytogenes_serotype_4b_str_02-1103_1196171	Listeria_monocytogenes_1639_v130	0.9203
Listeria_monocytogenes_serotype_3c_str_10-5027_1196195	Listeria_monocytogenes_1639_v130	0.9721
Listeria_monocytogenes_serotype_1_2c_str_10-5026_1196194	Listeria_monocytogenes_1639_v130	0.9732
Listeria_monocytogenes_serotype_1_2c_str_10-5025_1196193	Listeria_monocytogenes_1639_v130	0.9732
Listeria_monocytogenes_serotype_1_2b_str_10-0811_1196164	Listeria_monocytogenes_1639_v130	0.933
Listeria_monocytogenes_serotype_1_2b_str_10-0810_1196163	Listeria_monocytogenes_1639_v130	0.933
Listeria_monocytogenes_serotype_1_2a_str_99-6370_1196179	Listeria_monocytogenes_1639_v130	0.9347
Listeria_monocytogenes_serotype_1_2a_str_98-2035_1196178	Listeria_monocytogenes_1639_v130	0.9347
Listeria_monocytogenes_serotype_1_2a_str_95-0093_1196177	Listeria_monocytogenes_1639_v130	0.9402
Listeria_monocytogenes_serotype_1_2a_str_88-0478_1196176	Listeria_monocytogenes_1639_v130	0.9416
Listeria_monocytogenes_serotype_1_2a_str_10-5024_1196192	Listeria_monocytogenes_1639_v130	0.9405
Listeria_monocytogenes_serotype_1_2a_str_10-4758_1196168	Listeria_monocytogenes_1639_v130	0.9583
Listeria_monocytogenes_serotype_1_2a_str_10-4754_1196167	Listeria_monocytogenes_1639_v130	0.9583
Listeria_monocytogenes_serotype_1_2a_str_10-1321_1196191	Listeria_monocytogenes_1639_v130	0.9405
Listeria_monocytogenes_serotype_1_2a_str_10-1047_1196190	Listeria_monocytogenes_1639_v130	0.9405
Listeria_monocytogenes_serotype_1_2a_str_10-1046_1196189	Listeria_monocytogenes_1639_v130	0.9405
Listeria_monocytogenes_serotype_1_2a_str_10-0934_1196170	Listeria_monocytogenes_1639_v130	0.9559
Listeria_monocytogenes_serotype_1_2a_str_10-0933_1196169	Listeria_monocytogenes_1639_v130	0.9559
Listeria_monocytogenes_serotype_1_2a_str_10-0815_1196188	Listeria_monocytogenes_1639_v130	0.9357
Listeria_monocytogenes_serotype_1_2a_str_10-0814_1196187	Listeria_monocytogenes_1639_v130	0.9402
Listeria_monocytogenes_serotype_1_2a_str_10-0813_1196166	Listeria_monocytogenes_1639_v130	0.9493
Listeria_monocytogenes_serotype_1_2a_str_10-0812_1196165	Listeria_monocytogenes_1639_v130	0.9493
Listeria_monocytogenes_serotype_1_2a_str_08-7669_1196186	Listeria_monocytogenes_1639_v130	0.937
Listeria_monocytogenes_serotype_1_2a_str_08-7374_1196185	Listeria_monocytogenes_1639_v130	0.9405
Listeria_monocytogenes_serotype_1_2a_str_08-6997_1196184	Listeria_monocytogenes_1639_v130	0.9357
Listeria_monocytogenes_serotype_1_2a_str_08-6569_1196183	Listeria_monocytogenes_1639_v130	0.9357
Listeria_monocytogenes_serotype_1_2a_str_08-6056_1196182	Listeria_monocytogenes_1639_v130	0.9357
Listeria_monocytogenes_serotype_1_2a_str_04-5457_1196181	Listeria_monocytogenes_1639_v130	0.9405
Listeria_monocytogenes_serotype_1_2a_str_02-5993_1196180	Listeria_monocytogenes_1639_v130	0.9402
Listeria_monocytogenes_R479a_1437838	Listeria_monocytogenes_1639_v130	0.9465
Listeria_monocytogenes_N53-1_1234142	Listeria_monocytogenes_1639_v130	0.9032
Listeria_monocytogenes_M7_1030009	Listeria_monocytogenes_1639_v130	0.9038
Listeria_monocytogenes_LO28_393132	Listeria_monocytogenes_1639_v130	0.9796
Listeria_monocytogenes_L99_563174	Listeria_monocytogenes_1639_v130	0.9045
Listeria_monocytogenes_L312_882094	Listeria_monocytogenes_1639_v130	0.9337
Listeria_monocytogenes_J1816_930781	Listeria_monocytogenes_1639_v130	0.9162
Listeria_monocytogenes_J1-220_930782	Listeria_monocytogenes_1639_v130	0.9158
Listeria_monocytogenes_J0161_393130	Listeria_monocytogenes_1639_v130	0.9362
Listeria_monocytogenes_HCC23_552536	Listeria_monocytogenes_1639_v130	0.9041
Listeria_monocytogenes_FSL_R2-561_393126	Listeria_monocytogenes_1639_v130	0.9714
Listeria_monocytogenes_FSL_J1-208_393119	Listeria_monocytogenes_1639_v130	0.8977
Listeria_monocytogenes_FSL_J1-175_393118	Listeria_monocytogenes_1639_v130	0.9421
Listeria_monocytogenes_Finland_1998_393127	Listeria_monocytogenes_1639_v130	0.9502
Listeria_monocytogenes_EGD-e_169963_v1	Listeria_monocytogenes_1639_v130	0.9644
Listeria_monocytogenes_EGD-e_169963	Listeria_monocytogenes_1639_v130	0.9644
Listeria_monocytogenes_EGD_1334565	Listeria_monocytogenes_1639_v130	0.9552
Listeria_monocytogenes_CFSAN002349_1299895	Listeria_monocytogenes_1639_v130	0.9339
Listeria_monocytogenes_ATCC_19117_882095_v1	Listeria_monocytogenes_1639_v130	0.9268
Listeria_monocytogenes_ATCC_19117_882095	Listeria_monocytogenes_1639_v130	0.9268
Listeria_monocytogenes_6179_1288295	Listeria_monocytogenes_1639_v130	0.9429
Listeria_monocytogenes_1639_v9	Listeria_monocytogenes_1639_v130	0.9168
Listeria_monocytogenes_1639_v99	Listeria_monocytogenes_1639_v130	0.9258

Listeria_monocytogenes_1639_v98	Listeria_monocytogenes_1639_v130	0.9379
Listeria_monocytogenes_1639_v97	Listeria_monocytogenes_1639_v130	0.9208
Listeria_monocytogenes_1639_v96	Listeria_monocytogenes_1639_v130	0.9259
Listeria_monocytogenes_1639_v95	Listeria_monocytogenes_1639_v130	0.9259
Listeria_monocytogenes_1639_v94	Listeria_monocytogenes_1639_v130	0.9259
Listeria_monocytogenes_1639_v93	Listeria_monocytogenes_1639_v130	0.952
Listeria_monocytogenes_1639_v92	Listeria_monocytogenes_1639_v130	0.9262
Listeria_monocytogenes_1639_v91	Listeria_monocytogenes_1639_v130	0.922
Listeria_monocytogenes_1639_v90	Listeria_monocytogenes_1639_v130	0.9379
Listeria_monocytogenes_1639_v8	Listeria_monocytogenes_1639_v130	0.942
Listeria_monocytogenes_1639_v89	Listeria_monocytogenes_1639_v130	0.9261
Listeria_monocytogenes_1639_v88	Listeria_monocytogenes_1639_v130	0.9538
Listeria_monocytogenes_1639_v87	Listeria_monocytogenes_1639_v130	0.9451
Listeria_monocytogenes_1639_v86	Listeria_monocytogenes_1639_v130	0.9443
Listeria_monocytogenes_1639_v85	Listeria_monocytogenes_1639_v130	0.9171
Listeria_monocytogenes_1639_v84	Listeria_monocytogenes_1639_v130	0.9533
Listeria_monocytogenes_1639_v83	Listeria_monocytogenes_1639_v130	0.918
Listeria_monocytogenes_1639_v82	Listeria_monocytogenes_1639_v130	0.9177
Listeria_monocytogenes_1639_v81	Listeria_monocytogenes_1639_v130	0.9471
Listeria_monocytogenes_1639_v80	Listeria_monocytogenes_1639_v130	0.9376
Listeria_monocytogenes_1639_v7	Listeria_monocytogenes_1639_v130	0.942
Listeria_monocytogenes_1639_v79	Listeria_monocytogenes_1639_v130	0.9476
Listeria_monocytogenes_1639_v78	Listeria_monocytogenes_1639_v130	0.9464
Listeria_monocytogenes_1639_v77	Listeria_monocytogenes_1639_v130	0.9387
Listeria_monocytogenes_1639_v76	Listeria_monocytogenes_1639_v130	0.942
Listeria_monocytogenes_1639_v75	Listeria_monocytogenes_1639_v130	0.9045
Listeria_monocytogenes_1639_v74	Listeria_monocytogenes_1639_v130	0.9362
Listeria_monocytogenes_1639_v73	Listeria_monocytogenes_1639_v130	0.9797
Listeria_monocytogenes_1639_v72	Listeria_monocytogenes_1639_v130	0.9164
Listeria_monocytogenes_1639_v71	Listeria_monocytogenes_1639_v130	0.9344
Listeria_monocytogenes_1639_v70	Listeria_monocytogenes_1639_v130	0.9785
Listeria_monocytogenes_1639_v6	Listeria_monocytogenes_1639_v130	0.9413
Listeria_monocytogenes_1639_v69	Listeria_monocytogenes_1639_v130	0.9196
Listeria_monocytogenes_1639_v68	Listeria_monocytogenes_1639_v130	0.9415
Listeria_monocytogenes_1639_v67	Listeria_monocytogenes_1639_v130	0.9378
Listeria_monocytogenes_1639_v66	Listeria_monocytogenes_1639_v130	0.9569
Listeria_monocytogenes_1639_v65	Listeria_monocytogenes_1639_v130	0.9422
Listeria_monocytogenes_1639_v64	Listeria_monocytogenes_1639_v130	0.9498
Listeria_monocytogenes_1639_v63	Listeria_monocytogenes_1639_v130	0.9441
Listeria_monocytogenes_1639_v62	Listeria_monocytogenes_1639_v130	0.9417
Listeria_monocytogenes_1639_v61	Listeria_monocytogenes_1639_v130	0.9415
Listeria_monocytogenes_1639_v60	Listeria_monocytogenes_1639_v130	0.9381
Listeria_monocytogenes_1639_v5	Listeria_monocytogenes_1639_v130	0.9059
Listeria_monocytogenes_1639_v59	Listeria_monocytogenes_1639_v130	0.9243
Listeria_monocytogenes_1639_v58	Listeria_monocytogenes_1639_v130	0.9239
Listeria_monocytogenes_1639_v57	Listeria_monocytogenes_1639_v130	0.9125
Listeria_monocytogenes_1639_v56	Listeria_monocytogenes_1639_v130	0.9187
Listeria_monocytogenes_1639_v55	Listeria_monocytogenes_1639_v130	0.9191
Listeria_monocytogenes_1639_v54	Listeria_monocytogenes_1639_v130	0.9133
Listeria_monocytogenes_1639_v53	Listeria_monocytogenes_1639_v130	0.9374
Listeria_monocytogenes_1639_v52	Listeria_monocytogenes_1639_v130	0.9201
Listeria_monocytogenes_1639_v51	Listeria_monocytogenes_1639_v130	0.921
Listeria_monocytogenes_1639_v50	Listeria_monocytogenes_1639_v130	0.9391
Listeria_monocytogenes_1639_v4	Listeria_monocytogenes_1639_v130	0.9179
Listeria_monocytogenes_1639_v49	Listeria_monocytogenes_1639_v130	0.9211
Listeria_monocytogenes_1639_v48	Listeria_monocytogenes_1639_v130	0.9671
Listeria_monocytogenes_1639_v47	Listeria_monocytogenes_1639_v130	0.9409
Listeria_monocytogenes_1639_v46	Listeria_monocytogenes_1639_v130	0.9404
Listeria_monocytogenes_1639_v45	Listeria_monocytogenes_1639_v130	0.939
Listeria_monocytogenes_1639_v44	Listeria_monocytogenes_1639_v130	0.9405
Listeria_monocytogenes_1639_v43	Listeria_monocytogenes_1639_v130	0.9405
Listeria_monocytogenes_1639_v42	Listeria_monocytogenes_1639_v130	0.9273
Listeria_monocytogenes_1639_v41	Listeria_monocytogenes_1639_v130	0.9296
Listeria_monocytogenes_1639_v40	Listeria_monocytogenes_1639_v130	0.9796
Listeria_monocytogenes_1639_v3	Listeria_monocytogenes_1639_v130	0.9186
Listeria_monocytogenes_1639_v39	Listeria_monocytogenes_1639_v130	0.937
Listeria_monocytogenes_1639_v38	Listeria_monocytogenes_1639_v130	0.9682
Listeria_monocytogenes_1639_v37	Listeria_monocytogenes_1639_v130	0.9558
Listeria_monocytogenes_1639_v36	Listeria_monocytogenes_1639_v130	0.9566
Listeria_monocytogenes_1639_v35	Listeria_monocytogenes_1639_v130	0.9814
Listeria_monocytogenes_1639_v34	Listeria_monocytogenes_1639_v130	0.946
Listeria_monocytogenes_1639_v33	Listeria_monocytogenes_1639_v130	0.933
Listeria_monocytogenes_1639_v32	Listeria_monocytogenes_1639_v130	0.9592
Listeria_monocytogenes_1639_v31	Listeria_monocytogenes_1639_v130	0.9464
Listeria_monocytogenes_1639_v30	Listeria_monocytogenes_1639_v130	0.924
Listeria_monocytogenes_1639_v2	Listeria_monocytogenes_1639_v130	0.9045
Listeria_monocytogenes_1639_v29	Listeria_monocytogenes_1639_v130	0.9718
Listeria_monocytogenes_1639_v28	Listeria_monocytogenes_1639_v130	0.9665
Listeria_monocytogenes_1639_v27	Listeria_monocytogenes_1639_v130	0.9742
Listeria_monocytogenes_1639_v26	Listeria_monocytogenes_1639_v130	0.9738
Listeria_monocytogenes_1639_v25	Listeria_monocytogenes_1639_v130	0.9738
Listeria_monocytogenes_1639_v24	Listeria_monocytogenes_1639_v130	0.9161
Listeria_monocytogenes_1639_v23	Listeria_monocytogenes_1639_v130	0.9161
Listeria_monocytogenes_1639_v22	Listeria_monocytogenes_1639_v130	0.934
Listeria_monocytogenes_1639_v21	Listeria_monocytogenes_1639_v130	0.9169
Listeria_monocytogenes_1639_v20	Listeria_monocytogenes_1639_v130	0.9422
Listeria_monocytogenes_1639_v1	Listeria_monocytogenes_1639_v130	0.9361
Listeria_monocytogenes_1639_v19	Listeria_monocytogenes_1639_v130	0.9415
Listeria_monocytogenes_1639_v198	Listeria_monocytogenes_1639_v130	0.9055
Listeria_monocytogenes_1639_v197	Listeria_monocytogenes_1639_v130	0.9507
Listeria_monocytogenes_1639_v196	Listeria_monocytogenes_1639_v130	0.9385

Listeria_monocytogenes_1639_v195	Listeria_monocytogenes_1639_v130	0.9345
Listeria_monocytogenes_1639_v194	Listeria_monocytogenes_1639_v130	0.9322
Listeria_monocytogenes_1639_v193	Listeria_monocytogenes_1639_v130	0.9326
Listeria_monocytogenes_1639_v192	Listeria_monocytogenes_1639_v130	0.8913
Listeria_monocytogenes_1639_v191	Listeria_monocytogenes_1639_v130	0.9539
Listeria_monocytogenes_1639_v190	Listeria_monocytogenes_1639_v130	0.9547
Listeria_monocytogenes_1639_v18	Listeria_monocytogenes_1639_v130	0.908
Listeria_monocytogenes_1639_v189	Listeria_monocytogenes_1639_v130	0.9539
Listeria_monocytogenes_1639_v188	Listeria_monocytogenes_1639_v130	0.9565
Listeria_monocytogenes_1639_v187	Listeria_monocytogenes_1639_v130	0.9543
Listeria_monocytogenes_1639_v186	Listeria_monocytogenes_1639_v130	0.955
Listeria_monocytogenes_1639_v185	Listeria_monocytogenes_1639_v130	0.9547
Listeria_monocytogenes_1639_v184	Listeria_monocytogenes_1639_v130	0.9543
Listeria_monocytogenes_1639_v183	Listeria_monocytogenes_1639_v130	0.9509
Listeria_monocytogenes_1639_v182	Listeria_monocytogenes_1639_v130	0.9531
Listeria_monocytogenes_1639_v181	Listeria_monocytogenes_1639_v130	0.9144
Listeria_monocytogenes_1639_v180	Listeria_monocytogenes_1639_v130	0.9144
Listeria_monocytogenes_1639_v17	Listeria_monocytogenes_1639_v130	0.908
Listeria_monocytogenes_1639_v179	Listeria_monocytogenes_1639_v130	0.9438
Listeria_monocytogenes_1639_v178	Listeria_monocytogenes_1639_v130	0.9524
Listeria_monocytogenes_1639_v177	Listeria_monocytogenes_1639_v130	0.9324
Listeria_monocytogenes_1639_v176	Listeria_monocytogenes_1639_v130	0.927
Listeria_monocytogenes_1639_v175	Listeria_monocytogenes_1639_v130	0.9585
Listeria_monocytogenes_1639_v174	Listeria_monocytogenes_1639_v130	0.9551
Listeria_monocytogenes_1639_v173	Listeria_monocytogenes_1639_v130	0.9418
Listeria_monocytogenes_1639_v172	Listeria_monocytogenes_1639_v130	0.9292
Listeria_monocytogenes_1639_v171	Listeria_monocytogenes_1639_v130	0.9591
Listeria_monocytogenes_1639_v170	Listeria_monocytogenes_1639_v130	0.9501
Listeria_monocytogenes_1639_v16	Listeria_monocytogenes_1639_v130	0.9349
Listeria_monocytogenes_1639_v169	Listeria_monocytogenes_1639_v130	0.9607
Listeria_monocytogenes_1639_v168	Listeria_monocytogenes_1639_v130	0.9468
Listeria_monocytogenes_1639_v167	Listeria_monocytogenes_1639_v130	0.944
Listeria_monocytogenes_1639_v166	Listeria_monocytogenes_1639_v130	0.9472
Listeria_monocytogenes_1639_v165	Listeria_monocytogenes_1639_v130	0.9607
Listeria_monocytogenes_1639_v164	Listeria_monocytogenes_1639_v130	0.9303
Listeria_monocytogenes_1639_v163	Listeria_monocytogenes_1639_v130	0.9436
Listeria_monocytogenes_1639_v162	Listeria_monocytogenes_1639_v130	0.9303
Listeria_monocytogenes_1639_v161	Listeria_monocytogenes_1639_v130	0.9314
Listeria_monocytogenes_1639_v160	Listeria_monocytogenes_1639_v130	0.9314
Listeria_monocytogenes_1639_v15	Listeria_monocytogenes_1639_v130	0.9334
Listeria_monocytogenes_1639_v159	Listeria_monocytogenes_1639_v130	0.9324
Listeria_monocytogenes_1639_v158	Listeria_monocytogenes_1639_v130	0.9311
Listeria_monocytogenes_1639_v157	Listeria_monocytogenes_1639_v130	0.9311
Listeria_monocytogenes_1639_v156	Listeria_monocytogenes_1639_v130	0.9357
Listeria_monocytogenes_1639_v155	Listeria_monocytogenes_1639_v130	0.948
Listeria_monocytogenes_1639_v154	Listeria_monocytogenes_1639_v130	0.9563
Listeria_monocytogenes_1639_v153	Listeria_monocytogenes_1639_v130	0.9465
Listeria_monocytogenes_1639_v152	Listeria_monocytogenes_1639_v130	0.9327
Listeria_monocytogenes_1639_v151	Listeria_monocytogenes_1639_v130	0.9327
Listeria_monocytogenes_1639_v150	Listeria_monocytogenes_1639_v130	0.9593
Listeria_monocytogenes_1639_v14	Listeria_monocytogenes_1639_v130	0.92
Listeria_monocytogenes_1639_v149	Listeria_monocytogenes_1639_v130	0.9552
Listeria_monocytogenes_1639_v148	Listeria_monocytogenes_1639_v130	0.9316
Listeria_monocytogenes_1639_v147	Listeria_monocytogenes_1639_v130	0.9215
Listeria_monocytogenes_1639_v146	Listeria_monocytogenes_1639_v130	0.9571
Listeria_monocytogenes_1639_v145	Listeria_monocytogenes_1639_v130	0.9489
Listeria_monocytogenes_1639_v144	Listeria_monocytogenes_1639_v130	0.9329
Listeria_monocytogenes_1639_v143	Listeria_monocytogenes_1639_v130	0.9552
Listeria_monocytogenes_1639_v142	Listeria_monocytogenes_1639_v130	0.9333
Listeria_monocytogenes_1639_v141	Listeria_monocytogenes_1639_v130	0.9427
Listeria_monocytogenes_1639_v140	Listeria_monocytogenes_1639_v130	0.9337
Listeria_monocytogenes_1639_v13	Listeria_monocytogenes_1639_v130	0.9337
Listeria_monocytogenes_1639_v139	Listeria_monocytogenes_1639_v130	0.9319
Listeria_monocytogenes_1639_v138	Listeria_monocytogenes_1639_v130	0.9352
Listeria_monocytogenes_1639_v137	Listeria_monocytogenes_1639_v130	0.9348
Listeria_monocytogenes_1639_v136	Listeria_monocytogenes_1639_v130	0.9591
Listeria_monocytogenes_1639_v135	Listeria_monocytogenes_1639_v130	0.9047
Listeria_monocytogenes_1639_v134	Listeria_monocytogenes_1639_v130	0.9268
Listeria_monocytogenes_1639_v133	Listeria_monocytogenes_1639_v130	0.948
Listeria_monocytogenes_1639_v132	Listeria_monocytogenes_1639_v130	0.9041
Listeria_monocytogenes_1639_v131	Listeria_monocytogenes_1639_v130	0.924
Listeria_monocytogenes_1639_v130	Listeria_monocytogenes_1639_v130	0.068
Listeria_welshimeri_1643	Listeria_monocytogenes_1639_v131	0.8751
Listeria_welshimeri_serovar_6b_str_SLCC5334_386043	Listeria_monocytogenes_1639_v131	0.8744
Listeria_seeligeri_serovar_1_2b_str_SLCC3954_683837	Listeria_monocytogenes_1639_v131	0.8795
Listeria_seeligeri_FSL_S4-171_702452	Listeria_monocytogenes_1639_v131	0.8253
Listeria_seeligeri_FSL_N1-067_702453	Listeria_monocytogenes_1639_v131	0.8312
Listeria_seeligeri_1640_v1	Listeria_monocytogenes_1639_v131	0.886
Listeria_seeligeri_1640	Listeria_monocytogenes_1639_v131	0.8898
Listeria_monocytogenes_WSLC1042_1457188	Listeria_monocytogenes_1639_v131	0.9561
Listeria_monocytogenes_WSLC1001_1457187	Listeria_monocytogenes_1639_v131	0.9299
Listeria_monocytogenes_SLCC7179_879090	Listeria_monocytogenes_1639_v131	0.9399
Listeria_monocytogenes_SLCC5850_882096	Listeria_monocytogenes_1639_v131	0.9341
Listeria_monocytogenes_SLCC2755_932919	Listeria_monocytogenes_1639_v131	0.9399
Listeria_monocytogenes_SLCC2540_879089	Listeria_monocytogenes_1639_v131	0.9419
Listeria_monocytogenes_SLCC2479_882020	Listeria_monocytogenes_1639_v131	0.9285
Listeria_monocytogenes_SLCC2378_879088	Listeria_monocytogenes_1639_v131	0.9575
Listeria_monocytogenes_SLCC2376_882097	Listeria_monocytogenes_1639_v131	0.9363
Listeria_monocytogenes_SLCC2372_932920	Listeria_monocytogenes_1639_v131	0.9212
Listeria_monocytogenes_serotype_4b_str_LL195_1230340	Listeria_monocytogenes_1639_v131	0.9634
Listeria_monocytogenes_serotype_4b_str_LIS0087_1134669	Listeria_monocytogenes_1639_v131	0.919

Listeria_monocytogenes_serotype_4b_str_F2365_265669	Listeria_monocytogenes_1639_v131	0.9634
Listeria_monocytogenes_serotype_4b_str_CLIP_80459_568819	Listeria_monocytogenes_1639_v131	0.9652
Listeria_monocytogenes_serotype_4b_str_81-0861_1196161	Listeria_monocytogenes_1639_v131	0.9576
Listeria_monocytogenes_serotype_4b_str_81-0592_1196160	Listeria_monocytogenes_1639_v131	0.9587
Listeria_monocytogenes_serotype_4b_str_81-0558_1196159	Listeria_monocytogenes_1639_v131	0.9587
Listeria_monocytogenes_serotype_4b_str_10-0809_1196162	Listeria_monocytogenes_1639_v131	0.9591
Listeria_monocytogenes_serotype_4b_str_02-6680_1196175	Listeria_monocytogenes_1639_v131	0.9623
Listeria_monocytogenes_serotype_4b_str_02-6679_1196174	Listeria_monocytogenes_1639_v131	0.9623
Listeria_monocytogenes_serotype_4b_str_02-1792_1196173	Listeria_monocytogenes_1639_v131	0.9606
Listeria_monocytogenes_serotype_4b_str_02-1289_1196172	Listeria_monocytogenes_1639_v131	0.9606
Listeria_monocytogenes_serotype_4b_str_02-1103_1196171	Listeria_monocytogenes_1639_v131	0.9606
Listeria_monocytogenes_serotype_3c_str_10-5027_1196195	Listeria_monocytogenes_1639_v131	0.9273
Listeria_monocytogenes_serotype_1_2c_str_10-5026_1196194	Listeria_monocytogenes_1639_v131	0.9277
Listeria_monocytogenes_serotype_1_2c_str_10-5025_1196193	Listeria_monocytogenes_1639_v131	0.9277
Listeria_monocytogenes_serotype_1_2b_str_10-0811_1196164	Listeria_monocytogenes_1639_v131	0.9463
Listeria_monocytogenes_serotype_1_2b_str_10-0810_1196163	Listeria_monocytogenes_1639_v131	0.9463
Listeria_monocytogenes_serotype_1_2a_str_99-6370_1196179	Listeria_monocytogenes_1639_v131	0.9187
Listeria_monocytogenes_serotype_1_2a_str_98-2035_1196178	Listeria_monocytogenes_1639_v131	0.9187
Listeria_monocytogenes_serotype_1_2a_str_95-0093_1196177	Listeria_monocytogenes_1639_v131	0.923
Listeria_monocytogenes_serotype_1_2a_str_88-0478_1196176	Listeria_monocytogenes_1639_v131	0.9226
Listeria_monocytogenes_serotype_1_2a_str_10-5024_1196192	Listeria_monocytogenes_1639_v131	0.9234
Listeria_monocytogenes_serotype_1_2a_str_10-4758_1196168	Listeria_monocytogenes_1639_v131	0.9338
Listeria_monocytogenes_serotype_1_2a_str_10-4754_1196167	Listeria_monocytogenes_1639_v131	0.9334
Listeria_monocytogenes_serotype_1_2a_str_10-1321_1196191	Listeria_monocytogenes_1639_v131	0.9234
Listeria_monocytogenes_serotype_1_2a_str_10-1047_1196190	Listeria_monocytogenes_1639_v131	0.9237
Listeria_monocytogenes_serotype_1_2a_str_10-1046_1196189	Listeria_monocytogenes_1639_v131	0.9237
Listeria_monocytogenes_serotype_1_2a_str_10-0934_1196170	Listeria_monocytogenes_1639_v131	0.9402
Listeria_monocytogenes_serotype_1_2a_str_10-0933_1196169	Listeria_monocytogenes_1639_v131	0.9402
Listeria_monocytogenes_serotype_1_2a_str_10-0815_1196188	Listeria_monocytogenes_1639_v131	0.9193
Listeria_monocytogenes_serotype_1_2a_str_10-0814_1196187	Listeria_monocytogenes_1639_v131	0.9234
Listeria_monocytogenes_serotype_1_2a_str_10-0813_1196166	Listeria_monocytogenes_1639_v131	0.9357
Listeria_monocytogenes_serotype_1_2a_str_10-0812_1196165	Listeria_monocytogenes_1639_v131	0.9357
Listeria_monocytogenes_serotype_1_2a_str_08-7669_1196186	Listeria_monocytogenes_1639_v131	0.9316
Listeria_monocytogenes_serotype_1_2a_str_08-7374_1196185	Listeria_monocytogenes_1639_v131	0.9234
Listeria_monocytogenes_serotype_1_2a_str_08-6997_1196184	Listeria_monocytogenes_1639_v131	0.9193
Listeria_monocytogenes_serotype_1_2a_str_08-6569_1196183	Listeria_monocytogenes_1639_v131	0.9193
Listeria_monocytogenes_serotype_1_2a_str_08-6056_1196182	Listeria_monocytogenes_1639_v131	0.9193
Listeria_monocytogenes_serotype_1_2a_str_04-5457_1196181	Listeria_monocytogenes_1639_v131	0.9234
Listeria_monocytogenes_serotype_1_2a_str_02-5993_1196180	Listeria_monocytogenes_1639_v131	0.923
Listeria_monocytogenes_R479a_1437838	Listeria_monocytogenes_1639_v131	0.9201
Listeria_monocytogenes_N53-1_1234142	Listeria_monocytogenes_1639_v131	0.8919
Listeria_monocytogenes_M7_1030009	Listeria_monocytogenes_1639_v131	0.9131
Listeria_monocytogenes_LO28_393132	Listeria_monocytogenes_1639_v131	0.932
Listeria_monocytogenes_L99_563174	Listeria_monocytogenes_1639_v131	0.9138
Listeria_monocytogenes_L312_882094	Listeria_monocytogenes_1639_v131	0.9656
Listeria_monocytogenes_J1816_930781	Listeria_monocytogenes_1639_v131	0.9723
Listeria_monocytogenes_J1-220_930782	Listeria_monocytogenes_1639_v131	0.9527
Listeria_monocytogenes_J0161_393130	Listeria_monocytogenes_1639_v131	0.9323
Listeria_monocytogenes_HCC23_552536	Listeria_monocytogenes_1639_v131	0.9131
Listeria_monocytogenes_FSL_R2-561_393126	Listeria_monocytogenes_1639_v131	0.9267
Listeria_monocytogenes_FSL_J1-208_393119	Listeria_monocytogenes_1639_v131	0.9091
Listeria_monocytogenes_FSL_J1-175_393118	Listeria_monocytogenes_1639_v131	0.9477
Listeria_monocytogenes_Finland_1998_393127	Listeria_monocytogenes_1639_v131	0.9414
Listeria_monocytogenes_EGD-e_169963_v1	Listeria_monocytogenes_1639_v131	0.9426
Listeria_monocytogenes_EGD-e_169963	Listeria_monocytogenes_1639_v131	0.943
Listeria_monocytogenes_EGD_1334565	Listeria_monocytogenes_1639_v131	0.9348
Listeria_monocytogenes_CFSAN002349_1299895	Listeria_monocytogenes_1639_v131	0.9703
Listeria_monocytogenes_ATCC_19117_882095_v1	Listeria_monocytogenes_1639_v131	0.9586
Listeria_monocytogenes_ATCC_19117_882095	Listeria_monocytogenes_1639_v131	0.9586
Listeria_monocytogenes_6179_1288295	Listeria_monocytogenes_1639_v131	0.9181
Listeria_monocytogenes_1639_v9	Listeria_monocytogenes_1639_v131	0.9309
Listeria_monocytogenes_1639_v99	Listeria_monocytogenes_1639_v131	0.9772
Listeria_monocytogenes_1639_v98	Listeria_monocytogenes_1639_v131	0.9341
Listeria_monocytogenes_1639_v97	Listeria_monocytogenes_1639_v131	0.962
Listeria_monocytogenes_1639_v96	Listeria_monocytogenes_1639_v131	0.9735
Listeria_monocytogenes_1639_v95	Listeria_monocytogenes_1639_v131	0.9735
Listeria_monocytogenes_1639_v94	Listeria_monocytogenes_1639_v131	0.9739
Listeria_monocytogenes_1639_v93	Listeria_monocytogenes_1639_v131	0.9185
Listeria_monocytogenes_1639_v92	Listeria_monocytogenes_1639_v131	0.9802
Listeria_monocytogenes_1639_v91	Listeria_monocytogenes_1639_v131	0.9581
Listeria_monocytogenes_1639_v90	Listeria_monocytogenes_1639_v131	0.9341
Listeria_monocytogenes_1639_v8	Listeria_monocytogenes_1639_v131	0.91
Listeria_monocytogenes_1639_v89	Listeria_monocytogenes_1639_v131	0.9773
Listeria_monocytogenes_1639_v88	Listeria_monocytogenes_1639_v131	0.9279
Listeria_monocytogenes_1639_v87	Listeria_monocytogenes_1639_v131	0.9312
Listeria_monocytogenes_1639_v86	Listeria_monocytogenes_1639_v131	0.9305
Listeria_monocytogenes_1639_v85	Listeria_monocytogenes_1639_v131	0.9573
Listeria_monocytogenes_1639_v84	Listeria_monocytogenes_1639_v131	0.9338
Listeria_monocytogenes_1639_v83	Listeria_monocytogenes_1639_v131	0.9576
Listeria_monocytogenes_1639_v82	Listeria_monocytogenes_1639_v131	0.9568
Listeria_monocytogenes_1639_v81	Listeria_monocytogenes_1639_v131	0.9302
Listeria_monocytogenes_1639_v80	Listeria_monocytogenes_1639_v131	0.9503
Listeria_monocytogenes_1639_v7	Listeria_monocytogenes_1639_v131	0.91
Listeria_monocytogenes_1639_v79	Listeria_monocytogenes_1639_v131	0.9281
Listeria_monocytogenes_1639_v78	Listeria_monocytogenes_1639_v131	0.9291
Listeria_monocytogenes_1639_v77	Listeria_monocytogenes_1639_v131	0.9284
Listeria_monocytogenes_1639_v76	Listeria_monocytogenes_1639_v131	0.9222
Listeria_monocytogenes_1639_v75	Listeria_monocytogenes_1639_v131	0.9138
Listeria_monocytogenes_1639_v74	Listeria_monocytogenes_1639_v131	0.9275
Listeria_monocytogenes_1639_v73	Listeria_monocytogenes_1639_v131	0.9271
Listeria_monocytogenes_1639_v72	Listeria_monocytogenes_1639_v131	0.9601

Listeria_monocytogenes_1639_v71	Listeria_monocytogenes_1639_v131	0.9109
Listeria_monocytogenes_1639_v70	Listeria_monocytogenes_1639_v131	0.9261
Listeria_monocytogenes_1639_v6	Listeria_monocytogenes_1639_v131	0.9093
Listeria_monocytogenes_1639_v69	Listeria_monocytogenes_1639_v131	0.9591
Listeria_monocytogenes_1639_v68	Listeria_monocytogenes_1639_v131	0.9343
Listeria_monocytogenes_1639_v67	Listeria_monocytogenes_1639_v131	0.9407
Listeria_monocytogenes_1639_v66	Listeria_monocytogenes_1639_v131	0.929
Listeria_monocytogenes_1639_v65	Listeria_monocytogenes_1639_v131	0.9321
Listeria_monocytogenes_1639_v64	Listeria_monocytogenes_1639_v131	0.9201
Listeria_monocytogenes_1639_v63	Listeria_monocytogenes_1639_v131	0.9414
Listeria_monocytogenes_1639_v62	Listeria_monocytogenes_1639_v131	0.9245
Listeria_monocytogenes_1639_v61	Listeria_monocytogenes_1639_v131	0.9265
Listeria_monocytogenes_1639_v60	Listeria_monocytogenes_1639_v131	0.9286
Listeria_monocytogenes_1639_v5	Listeria_monocytogenes_1639_v131	0.9124
Listeria_monocytogenes_1639_v59	Listeria_monocytogenes_1639_v131	0.9511
Listeria_monocytogenes_1639_v58	Listeria_monocytogenes_1639_v131	0.9613
Listeria_monocytogenes_1639_v57	Listeria_monocytogenes_1639_v131	0.9725
Listeria_monocytogenes_1639_v56	Listeria_monocytogenes_1639_v131	0.9538
Listeria_monocytogenes_1639_v55	Listeria_monocytogenes_1639_v131	0.9781
Listeria_monocytogenes_1639_v54	Listeria_monocytogenes_1639_v131	0.9202
Listeria_monocytogenes_1639_v53	Listeria_monocytogenes_1639_v131	0.9234
Listeria_monocytogenes_1639_v52	Listeria_monocytogenes_1639_v131	0.9581
Listeria_monocytogenes_1639_v51	Listeria_monocytogenes_1639_v131	0.9548
Listeria_monocytogenes_1639_v50	Listeria_monocytogenes_1639_v131	0.9226
Listeria_monocytogenes_1639_v4	Listeria_monocytogenes_1639_v131	0.9253
Listeria_monocytogenes_1639_v49	Listeria_monocytogenes_1639_v131	0.949
Listeria_monocytogenes_1639_v48	Listeria_monocytogenes_1639_v131	0.923
Listeria_monocytogenes_1639_v47	Listeria_monocytogenes_1639_v131	0.9234
Listeria_monocytogenes_1639_v46	Listeria_monocytogenes_1639_v131	0.9283
Listeria_monocytogenes_1639_v45	Listeria_monocytogenes_1639_v131	0.9142
Listeria_monocytogenes_1639_v44	Listeria_monocytogenes_1639_v131	0.9234
Listeria_monocytogenes_1639_v43	Listeria_monocytogenes_1639_v131	0.9234
Listeria_monocytogenes_1639_v42	Listeria_monocytogenes_1639_v131	0.9109
Listeria_monocytogenes_1639_v41	Listeria_monocytogenes_1639_v131	0.906
Listeria_monocytogenes_1639_v40	Listeria_monocytogenes_1639_v131	0.9226
Listeria_monocytogenes_1639_v3	Listeria_monocytogenes_1639_v131	0.9257
Listeria_monocytogenes_1639_v39	Listeria_monocytogenes_1639_v131	0.9402
Listeria_monocytogenes_1639_v38	Listeria_monocytogenes_1639_v131	0.9202
Listeria_monocytogenes_1639_v37	Listeria_monocytogenes_1639_v131	0.9177
Listeria_monocytogenes_1639_v36	Listeria_monocytogenes_1639_v131	0.9148
Listeria_monocytogenes_1639_v35	Listeria_monocytogenes_1639_v131	0.9236
Listeria_monocytogenes_1639_v34	Listeria_monocytogenes_1639_v131	0.9214
Listeria_monocytogenes_1639_v33	Listeria_monocytogenes_1639_v131	0.9463
Listeria_monocytogenes_1639_v32	Listeria_monocytogenes_1639_v131	0.9255
Listeria_monocytogenes_1639_v31	Listeria_monocytogenes_1639_v131	0.9218
Listeria_monocytogenes_1639_v30	Listeria_monocytogenes_1639_v131	0.9376
Listeria_monocytogenes_1639_v2	Listeria_monocytogenes_1639_v131	0.9106
Listeria_monocytogenes_1639_v29	Listeria_monocytogenes_1639_v131	0.9134
Listeria_monocytogenes_1639_v28	Listeria_monocytogenes_1639_v131	0.9192
Listeria_monocytogenes_1639_v27	Listeria_monocytogenes_1639_v131	0.9149
Listeria_monocytogenes_1639_v26	Listeria_monocytogenes_1639_v131	0.915
Listeria_monocytogenes_1639_v25	Listeria_monocytogenes_1639_v131	0.9146
Listeria_monocytogenes_1639_v24	Listeria_monocytogenes_1639_v131	0.9524
Listeria_monocytogenes_1639_v23	Listeria_monocytogenes_1639_v131	0.952
Listeria_monocytogenes_1639_v22	Listeria_monocytogenes_1639_v131	0.95
Listeria_monocytogenes_1639_v21	Listeria_monocytogenes_1639_v131	0.9509
Listeria_monocytogenes_1639_v20	Listeria_monocytogenes_1639_v131	0.9329
Listeria_monocytogenes_1639_v1	Listeria_monocytogenes_1639_v131	0.913
Listeria_monocytogenes_1639_v19	Listeria_monocytogenes_1639_v131	0.9326
Listeria_monocytogenes_1639_v198	Listeria_monocytogenes_1639_v131	0.8935
Listeria_monocytogenes_1639_v197	Listeria_monocytogenes_1639_v131	0.9309
Listeria_monocytogenes_1639_v196	Listeria_monocytogenes_1639_v131	0.9176
Listeria_monocytogenes_1639_v195	Listeria_monocytogenes_1639_v131	0.9374
Listeria_monocytogenes_1639_v194	Listeria_monocytogenes_1639_v131	0.9371
Listeria_monocytogenes_1639_v193	Listeria_monocytogenes_1639_v131	0.9371
Listeria_monocytogenes_1639_v192	Listeria_monocytogenes_1639_v131	0.901
Listeria_monocytogenes_1639_v191	Listeria_monocytogenes_1639_v131	0.942
Listeria_monocytogenes_1639_v190	Listeria_monocytogenes_1639_v131	0.9428
Listeria_monocytogenes_1639_v18	Listeria_monocytogenes_1639_v131	0.9716
Listeria_monocytogenes_1639_v189	Listeria_monocytogenes_1639_v131	0.9417
Listeria_monocytogenes_1639_v188	Listeria_monocytogenes_1639_v131	0.9427
Listeria_monocytogenes_1639_v187	Listeria_monocytogenes_1639_v131	0.9424
Listeria_monocytogenes_1639_v186	Listeria_monocytogenes_1639_v131	0.9428
Listeria_monocytogenes_1639_v185	Listeria_monocytogenes_1639_v131	0.9428
Listeria_monocytogenes_1639_v184	Listeria_monocytogenes_1639_v131	0.942
Listeria_monocytogenes_1639_v183	Listeria_monocytogenes_1639_v131	0.9355
Listeria_monocytogenes_1639_v182	Listeria_monocytogenes_1639_v131	0.9377
Listeria_monocytogenes_1639_v181	Listeria_monocytogenes_1639_v131	0.9229
Listeria_monocytogenes_1639_v180	Listeria_monocytogenes_1639_v131	0.9229
Listeria_monocytogenes_1639_v17	Listeria_monocytogenes_1639_v131	0.9716
Listeria_monocytogenes_1639_v179	Listeria_monocytogenes_1639_v131	0.9323
Listeria_monocytogenes_1639_v178	Listeria_monocytogenes_1639_v131	0.9424
Listeria_monocytogenes_1639_v177	Listeria_monocytogenes_1639_v131	0.9871
Listeria_monocytogenes_1639_v176	Listeria_monocytogenes_1639_v131	0.9353
Listeria_monocytogenes_1639_v175	Listeria_monocytogenes_1639_v131	0.9323
Listeria_monocytogenes_1639_v174	Listeria_monocytogenes_1639_v131	0.9429
Listeria_monocytogenes_1639_v173	Listeria_monocytogenes_1639_v131	0.9598
Listeria_monocytogenes_1639_v172	Listeria_monocytogenes_1639_v131	0.9321
Listeria_monocytogenes_1639_v171	Listeria_monocytogenes_1639_v131	0.9282
Listeria_monocytogenes_1639_v170	Listeria_monocytogenes_1639_v131	0.9364
Listeria_monocytogenes_1639_v16	Listeria_monocytogenes_1639_v131	0.9102

Listeria_monocytogenes_1639_v169	Listeria_monocytogenes_1639_v131	0.938
Listeria_monocytogenes_1639_v168	Listeria_monocytogenes_1639_v131	0.9403
Listeria_monocytogenes_1639_v167	Listeria_monocytogenes_1639_v131	0.9314
Listeria_monocytogenes_1639_v166	Listeria_monocytogenes_1639_v131	0.9406
Listeria_monocytogenes_1639_v165	Listeria_monocytogenes_1639_v131	0.9376
Listeria_monocytogenes_1639_v164	Listeria_monocytogenes_1639_v131	0.9626
Listeria_monocytogenes_1639_v163	Listeria_monocytogenes_1639_v131	0.9535
Listeria_monocytogenes_1639_v162	Listeria_monocytogenes_1639_v131	0.9626
Listeria_monocytogenes_1639_v161	Listeria_monocytogenes_1639_v131	0.9633
Listeria_monocytogenes_1639_v160	Listeria_monocytogenes_1639_v131	0.963
Listeria_monocytogenes_1639_v15	Listeria_monocytogenes_1639_v131	0.9325
Listeria_monocytogenes_1639_v159	Listeria_monocytogenes_1639_v131	0.9871
Listeria_monocytogenes_1639_v158	Listeria_monocytogenes_1639_v131	0.9634
Listeria_monocytogenes_1639_v157	Listeria_monocytogenes_1639_v131	0.9634
Listeria_monocytogenes_1639_v156	Listeria_monocytogenes_1639_v131	0.9296
Listeria_monocytogenes_1639_v155	Listeria_monocytogenes_1639_v131	0.9385
Listeria_monocytogenes_1639_v154	Listeria_monocytogenes_1639_v131	0.9362
Listeria_monocytogenes_1639_v153	Listeria_monocytogenes_1639_v131	0.9426
Listeria_monocytogenes_1639_v152	Listeria_monocytogenes_1639_v131	0.9874
Listeria_monocytogenes_1639_v151	Listeria_monocytogenes_1639_v131	0.9874
Listeria_monocytogenes_1639_v150	Listeria_monocytogenes_1639_v131	0.9347
Listeria_monocytogenes_1639_v14	Listeria_monocytogenes_1639_v131	0.9383
Listeria_monocytogenes_1639_v149	Listeria_monocytogenes_1639_v131	0.9348
Listeria_monocytogenes_1639_v148	Listeria_monocytogenes_1639_v131	0.9859
Listeria_monocytogenes_1639_v147	Listeria_monocytogenes_1639_v131	0.9715
Listeria_monocytogenes_1639_v146	Listeria_monocytogenes_1639_v131	0.9384
Listeria_monocytogenes_1639_v145	Listeria_monocytogenes_1639_v131	0.9336
Listeria_monocytogenes_1639_v144	Listeria_monocytogenes_1639_v131	0.9649
Listeria_monocytogenes_1639_v143	Listeria_monocytogenes_1639_v131	0.9404
Listeria_monocytogenes_1639_v142	Listeria_monocytogenes_1639_v131	0.9653
Listeria_monocytogenes_1639_v141	Listeria_monocytogenes_1639_v131	0.9602
Listeria_monocytogenes_1639_v140	Listeria_monocytogenes_1639_v131	0.9656
Listeria_monocytogenes_1639_v13	Listeria_monocytogenes_1639_v131	0.9329
Listeria_monocytogenes_1639_v139	Listeria_monocytogenes_1639_v131	0.9649
Listeria_monocytogenes_1639_v138	Listeria_monocytogenes_1639_v131	0.9679
Listeria_monocytogenes_1639_v137	Listeria_monocytogenes_1639_v131	0.9676
Listeria_monocytogenes_1639_v136	Listeria_monocytogenes_1639_v131	0.9335
Listeria_monocytogenes_1639_v135	Listeria_monocytogenes_1639_v131	0.9239
Listeria_monocytogenes_1639_v134	Listeria_monocytogenes_1639_v131	0.959
Listeria_monocytogenes_1639_v133	Listeria_monocytogenes_1639_v131	0.9325
Listeria_monocytogenes_1639_v132	Listeria_monocytogenes_1639_v131	0.9243
Listeria_monocytogenes_1639_v131	Listeria_monocytogenes_1639_v131	0.0653
Listeria_welshimeri_1643	Listeria_monocytogenes_1639_v132	0.8684
Listeria_welshimeri_serovar_6b_str_SLCC5334_386043	Listeria_monocytogenes_1639_v132	0.8678
Listeria_seeligeri_serovar_1_2b_str_SLCC3954_683837	Listeria_monocytogenes_1639_v132	0.8712
Listeria_seeligeri_FSL_S4-171_702452	Listeria_monocytogenes_1639_v132	0.8192
Listeria_seeligeri_FSL_N1-067_702453	Listeria_monocytogenes_1639_v132	0.8174
Listeria_seeligeri_1640_v1	Listeria_monocytogenes_1639_v132	0.8717
Listeria_seeligeri_1640	Listeria_monocytogenes_1639_v132	0.879
Listeria_monocytogenes_WSLC1042_1457188	Listeria_monocytogenes_1639_v132	0.9166
Listeria_monocytogenes_WSLC1001_1457187	Listeria_monocytogenes_1639_v132	0.9086
Listeria_monocytogenes_SLCC7179_879090	Listeria_monocytogenes_1639_v132	0.9226
Listeria_monocytogenes_SLCC5850_882096	Listeria_monocytogenes_1639_v132	0.9124
Listeria_monocytogenes_SLCC2755_932919	Listeria_monocytogenes_1639_v132	0.9094
Listeria_monocytogenes_SLCC2540_879089	Listeria_monocytogenes_1639_v132	0.9116
Listeria_monocytogenes_SLCC2479_882020	Listeria_monocytogenes_1639_v132	0.9059
Listeria_monocytogenes_SLCC2378_879088	Listeria_monocytogenes_1639_v132	0.918
Listeria_monocytogenes_SLCC2376_882097	Listeria_monocytogenes_1639_v132	0.9383
Listeria_monocytogenes_SLCC2372_932920	Listeria_monocytogenes_1639_v132	0.9
Listeria_monocytogenes_serotype_4b_str_LL195_1230340	Listeria_monocytogenes_1639_v132	0.9231
Listeria_monocytogenes_serotype_4b_str_LIS0087_1134669	Listeria_monocytogenes_1639_v132	0.8968
Listeria_monocytogenes_serotype_4b_str_F2365_265669	Listeria_monocytogenes_1639_v132	0.9231
Listeria_monocytogenes_serotype_4b_str_CLIP_80459_568819	Listeria_monocytogenes_1639_v132	0.9283
Listeria_monocytogenes_serotype_4b_str_81-0861_1196161	Listeria_monocytogenes_1639_v132	0.9207
Listeria_monocytogenes_serotype_4b_str_81-0592_1196160	Listeria_monocytogenes_1639_v132	0.9218
Listeria_monocytogenes_serotype_4b_str_81-0558_1196159	Listeria_monocytogenes_1639_v132	0.9225
Listeria_monocytogenes_serotype_4b_str_10-0809_1196162	Listeria_monocytogenes_1639_v132	0.9218
Listeria_monocytogenes_serotype_4b_str_02-6680_1196175	Listeria_monocytogenes_1639_v132	0.9266
Listeria_monocytogenes_serotype_4b_str_02-6679_1196174	Listeria_monocytogenes_1639_v132	0.9266
Listeria_monocytogenes_serotype_4b_str_02-1792_1196173	Listeria_monocytogenes_1639_v132	0.9225
Listeria_monocytogenes_serotype_4b_str_02-1289_1196172	Listeria_monocytogenes_1639_v132	0.9225
Listeria_monocytogenes_serotype_4b_str_02-1103_1196171	Listeria_monocytogenes_1639_v132	0.9225
Listeria_monocytogenes_serotype_3c_str_10-5027_1196195	Listeria_monocytogenes_1639_v132	0.9059
Listeria_monocytogenes_serotype_1_2c_str_10-5026_1196194	Listeria_monocytogenes_1639_v132	0.907
Listeria_monocytogenes_serotype_1_2c_str_10-5025_1196193	Listeria_monocytogenes_1639_v132	0.907
Listeria_monocytogenes_serotype_1_2b_str_10-0811_1196164	Listeria_monocytogenes_1639_v132	0.9119
Listeria_monocytogenes_serotype_1_2b_str_10-0810_1196163	Listeria_monocytogenes_1639_v132	0.9119
Listeria_monocytogenes_serotype_1_2a_str_99-6370_1196179	Listeria_monocytogenes_1639_v132	0.8939
Listeria_monocytogenes_serotype_1_2a_str_98-2035_1196178	Listeria_monocytogenes_1639_v132	0.8939
Listeria_monocytogenes_serotype_1_2a_str_95-0093_1196177	Listeria_monocytogenes_1639_v132	0.8984
Listeria_monocytogenes_serotype_1_2a_str_88-0478_1196176	Listeria_monocytogenes_1639_v132	0.8987
Listeria_monocytogenes_serotype_1_2a_str_10-5024_1196192	Listeria_monocytogenes_1639_v132	0.8988
Listeria_monocytogenes_serotype_1_2a_str_10-4758_1196168	Listeria_monocytogenes_1639_v132	0.9133
Listeria_monocytogenes_serotype_1_2a_str_10-4754_1196167	Listeria_monocytogenes_1639_v132	0.9133
Listeria_monocytogenes_serotype_1_2a_str_10-1321_1196191	Listeria_monocytogenes_1639_v132	0.8988
Listeria_monocytogenes_serotype_1_2a_str_10-1047_1196190	Listeria_monocytogenes_1639_v132	0.8999
Listeria_monocytogenes_serotype_1_2a_str_10-1046_1196189	Listeria_monocytogenes_1639_v132	0.8999
Listeria_monocytogenes_serotype_1_2a_str_10-0934_1196170	Listeria_monocytogenes_1639_v132	0.9201
Listeria_monocytogenes_serotype_1_2a_str_10-0933_1196169	Listeria_monocytogenes_1639_v132	0.9201
Listeria_monocytogenes_serotype_1_2a_str_10-0815_1196188	Listeria_monocytogenes_1639_v132	0.8945
Listeria_monocytogenes_serotype_1_2a_str_10-0814_1196187	Listeria_monocytogenes_1639_v132	0.8984

Listeria_monocytogenes_serotype_1_2a_str_10-0813_1196166	Listeria_monocytogenes_1639_v132	0.916
Listeria_monocytogenes_serotype_1_2a_str_10-0812_1196165	Listeria_monocytogenes_1639_v132	0.916
Listeria_monocytogenes_serotype_1_2a_str_08-7669_1196186	Listeria_monocytogenes_1639_v132	0.9071
Listeria_monocytogenes_serotype_1_2a_str_08-7374_1196185	Listeria_monocytogenes_1639_v132	0.8988
Listeria_monocytogenes_serotype_1_2a_str_08-6997_1196184	Listeria_monocytogenes_1639_v132	0.8945
Listeria_monocytogenes_serotype_1_2a_str_08-6569_1196183	Listeria_monocytogenes_1639_v132	0.8945
Listeria_monocytogenes_serotype_1_2a_str_08-6056_1196182	Listeria_monocytogenes_1639_v132	0.8945
Listeria_monocytogenes_serotype_1_2a_str_04-5457_1196181	Listeria_monocytogenes_1639_v132	0.8988
Listeria_monocytogenes_serotype_1_2a_str_02-5993_1196180	Listeria_monocytogenes_1639_v132	0.8984
Listeria_monocytogenes_R479a_1437838	Listeria_monocytogenes_1639_v132	0.9004
Listeria_monocytogenes_N53-1_1234142	Listeria_monocytogenes_1639_v132	0.8749
Listeria_monocytogenes_M7_1030009	Listeria_monocytogenes_1639_v132	0.9244
Listeria_monocytogenes_LO28_393132	Listeria_monocytogenes_1639_v132	0.9098
Listeria_monocytogenes_L99_563174	Listeria_monocytogenes_1639_v132	0.9251
Listeria_monocytogenes_L312_882094	Listeria_monocytogenes_1639_v132	0.9283
Listeria_monocytogenes_J1816_930781	Listeria_monocytogenes_1639_v132	0.9106
Listeria_monocytogenes_J1-220_930782	Listeria_monocytogenes_1639_v132	0.9172
Listeria_monocytogenes_J0161_393130	Listeria_monocytogenes_1639_v132	0.9165
Listeria_monocytogenes_HCC23_552536	Listeria_monocytogenes_1639_v132	0.9248
Listeria_monocytogenes_FSL_R2-561_393126	Listeria_monocytogenes_1639_v132	0.9053
Listeria_monocytogenes_FSL_J1-208_393119	Listeria_monocytogenes_1639_v132	0.9142
Listeria_monocytogenes_FSL_J1-175_393118	Listeria_monocytogenes_1639_v132	0.9128
Listeria_monocytogenes_Finland_1998_393127	Listeria_monocytogenes_1639_v132	0.9193
Listeria_monocytogenes_EGD-e_169963_v1	Listeria_monocytogenes_1639_v132	0.922
Listeria_monocytogenes_EGD-e_169963	Listeria_monocytogenes_1639_v132	0.922
Listeria_monocytogenes_EGD_1334565	Listeria_monocytogenes_1639_v132	0.9131
Listeria_monocytogenes_CFSAN002349_1299895	Listeria_monocytogenes_1639_v132	0.9218
Listeria_monocytogenes_ATCC_19117_882095_v1	Listeria_monocytogenes_1639_v132	0.9215
Listeria_monocytogenes_ATCC_19117_882095	Listeria_monocytogenes_1639_v132	0.9215
Listeria_monocytogenes_6179_1288295	Listeria_monocytogenes_1639_v132	0.9044
Listeria_monocytogenes_1639_v9	Listeria_monocytogenes_1639_v132	0.8959
Listeria_monocytogenes_1639_v99	Listeria_monocytogenes_1639_v132	0.9121
Listeria_monocytogenes_1639_v98	Listeria_monocytogenes_1639_v132	0.9141
Listeria_monocytogenes_1639_v97	Listeria_monocytogenes_1639_v132	0.9178
Listeria_monocytogenes_1639_v96	Listeria_monocytogenes_1639_v132	0.9114
Listeria_monocytogenes_1639_v95	Listeria_monocytogenes_1639_v132	0.9114
Listeria_monocytogenes_1639_v94	Listeria_monocytogenes_1639_v132	0.9118
Listeria_monocytogenes_1639_v93	Listeria_monocytogenes_1639_v132	0.9006
Listeria_monocytogenes_1639_v92	Listeria_monocytogenes_1639_v132	0.9132
Listeria_monocytogenes_1639_v91	Listeria_monocytogenes_1639_v132	0.9194
Listeria_monocytogenes_1639_v90	Listeria_monocytogenes_1639_v132	0.9141
Listeria_monocytogenes_1639_v8	Listeria_monocytogenes_1639_v132	0.8909
Listeria_monocytogenes_1639_v89	Listeria_monocytogenes_1639_v132	0.9272
Listeria_monocytogenes_1639_v88	Listeria_monocytogenes_1639_v132	0.9079
Listeria_monocytogenes_1639_v87	Listeria_monocytogenes_1639_v132	0.9116
Listeria_monocytogenes_1639_v86	Listeria_monocytogenes_1639_v132	0.9109
Listeria_monocytogenes_1639_v85	Listeria_monocytogenes_1639_v132	0.9201
Listeria_monocytogenes_1639_v84	Listeria_monocytogenes_1639_v132	0.9103
Listeria_monocytogenes_1639_v83	Listeria_monocytogenes_1639_v132	0.9224
Listeria_monocytogenes_1639_v82	Listeria_monocytogenes_1639_v132	0.9217
Listeria_monocytogenes_1639_v81	Listeria_monocytogenes_1639_v132	0.9079
Listeria_monocytogenes_1639_v80	Listeria_monocytogenes_1639_v132	0.9163
Listeria_monocytogenes_1639_v7	Listeria_monocytogenes_1639_v132	0.8909
Listeria_monocytogenes_1639_v79	Listeria_monocytogenes_1639_v132	0.9059
Listeria_monocytogenes_1639_v78	Listeria_monocytogenes_1639_v132	0.9083
Listeria_monocytogenes_1639_v77	Listeria_monocytogenes_1639_v132	0.9035
Listeria_monocytogenes_1639_v76	Listeria_monocytogenes_1639_v132	0.9027
Listeria_monocytogenes_1639_v75	Listeria_monocytogenes_1639_v132	0.9251
Listeria_monocytogenes_1639_v74	Listeria_monocytogenes_1639_v132	0.9105
Listeria_monocytogenes_1639_v73	Listeria_monocytogenes_1639_v132	0.9055
Listeria_monocytogenes_1639_v72	Listeria_monocytogenes_1639_v132	0.9227
Listeria_monocytogenes_1639_v71	Listeria_monocytogenes_1639_v132	0.8954
Listeria_monocytogenes_1639_v70	Listeria_monocytogenes_1639_v132	0.9048
Listeria_monocytogenes_1639_v6	Listeria_monocytogenes_1639_v132	0.8902
Listeria_monocytogenes_1639_v69	Listeria_monocytogenes_1639_v132	0.9221
Listeria_monocytogenes_1639_v68	Listeria_monocytogenes_1639_v132	0.9191
Listeria_monocytogenes_1639_v67	Listeria_monocytogenes_1639_v132	0.9094
Listeria_monocytogenes_1639_v66	Listeria_monocytogenes_1639_v132	0.9074
Listeria_monocytogenes_1639_v65	Listeria_monocytogenes_1639_v132	0.9059
Listeria_monocytogenes_1639_v64	Listeria_monocytogenes_1639_v132	0.9051
Listeria_monocytogenes_1639_v63	Listeria_monocytogenes_1639_v132	0.907
Listeria_monocytogenes_1639_v62	Listeria_monocytogenes_1639_v132	0.9046
Listeria_monocytogenes_1639_v61	Listeria_monocytogenes_1639_v132	0.901
Listeria_monocytogenes_1639_v60	Listeria_monocytogenes_1639_v132	0.9127
Listeria_monocytogenes_1639_v5	Listeria_monocytogenes_1639_v132	0.8827
Listeria_monocytogenes_1639_v59	Listeria_monocytogenes_1639_v132	0.9156
Listeria_monocytogenes_1639_v58	Listeria_monocytogenes_1639_v132	0.9235
Listeria_monocytogenes_1639_v57	Listeria_monocytogenes_1639_v132	0.9073
Listeria_monocytogenes_1639_v56	Listeria_monocytogenes_1639_v132	0.9183
Listeria_monocytogenes_1639_v55	Listeria_monocytogenes_1639_v132	0.9114
Listeria_monocytogenes_1639_v54	Listeria_monocytogenes_1639_v132	0.9042
Listeria_monocytogenes_1639_v53	Listeria_monocytogenes_1639_v132	0.9071
Listeria_monocytogenes_1639_v52	Listeria_monocytogenes_1639_v132	0.9223
Listeria_monocytogenes_1639_v51	Listeria_monocytogenes_1639_v132	0.9187
Listeria_monocytogenes_1639_v50	Listeria_monocytogenes_1639_v132	0.8977
Listeria_monocytogenes_1639_v4	Listeria_monocytogenes_1639_v132	0.8925
Listeria_monocytogenes_1639_v49	Listeria_monocytogenes_1639_v132	0.9095
Listeria_monocytogenes_1639_v48	Listeria_monocytogenes_1639_v132	0.901
Listeria_monocytogenes_1639_v47	Listeria_monocytogenes_1639_v132	0.8996
Listeria_monocytogenes_1639_v46	Listeria_monocytogenes_1639_v132	0.9113
Listeria_monocytogenes_1639_v45	Listeria_monocytogenes_1639_v132	0.8922

Listeria_monocytogenes_1639_v44	Listeria_monocytogenes_1639_v132	0.8988
Listeria_monocytogenes_1639_v43	Listeria_monocytogenes_1639_v132	0.8988
Listeria_monocytogenes_1639_v42	Listeria_monocytogenes_1639_v132	0.8983
Listeria_monocytogenes_1639_v41	Listeria_monocytogenes_1639_v132	0.89
Listeria_monocytogenes_1639_v40	Listeria_monocytogenes_1639_v132	0.9038
Listeria_monocytogenes_1639_v3	Listeria_monocytogenes_1639_v132	0.8929
Listeria_monocytogenes_1639_v39	Listeria_monocytogenes_1639_v132	0.9091
Listeria_monocytogenes_1639_v38	Listeria_monocytogenes_1639_v132	0.9008
Listeria_monocytogenes_1639_v37	Listeria_monocytogenes_1639_v132	0.8958
Listeria_monocytogenes_1639_v36	Listeria_monocytogenes_1639_v132	0.896
Listeria_monocytogenes_1639_v35	Listeria_monocytogenes_1639_v132	0.9041
Listeria_monocytogenes_1639_v34	Listeria_monocytogenes_1639_v132	0.9075
Listeria_monocytogenes_1639_v33	Listeria_monocytogenes_1639_v132	0.9119
Listeria_monocytogenes_1639_v32	Listeria_monocytogenes_1639_v132	0.9018
Listeria_monocytogenes_1639_v31	Listeria_monocytogenes_1639_v132	0.9079
Listeria_monocytogenes_1639_v30	Listeria_monocytogenes_1639_v132	0.9032
Listeria_monocytogenes_1639_v2	Listeria_monocytogenes_1639_v132	0.8873
Listeria_monocytogenes_1639_v29	Listeria_monocytogenes_1639_v132	0.8931
Listeria_monocytogenes_1639_v28	Listeria_monocytogenes_1639_v132	0.8966
Listeria_monocytogenes_1639_v27	Listeria_monocytogenes_1639_v132	0.8933
Listeria_monocytogenes_1639_v26	Listeria_monocytogenes_1639_v132	0.893
Listeria_monocytogenes_1639_v25	Listeria_monocytogenes_1639_v132	0.8926
Listeria_monocytogenes_1639_v24	Listeria_monocytogenes_1639_v132	0.9154
Listeria_monocytogenes_1639_v23	Listeria_monocytogenes_1639_v132	0.915
Listeria_monocytogenes_1639_v22	Listeria_monocytogenes_1639_v132	0.9147
Listeria_monocytogenes_1639_v21	Listeria_monocytogenes_1639_v132	0.915
Listeria_monocytogenes_1639_v20	Listeria_monocytogenes_1639_v132	0.9065
Listeria_monocytogenes_1639_v1	Listeria_monocytogenes_1639_v132	0.898
Listeria_monocytogenes_1639_v19	Listeria_monocytogenes_1639_v132	0.9062
Listeria_monocytogenes_1639_v198	Listeria_monocytogenes_1639_v132	0.8774
Listeria_monocytogenes_1639_v197	Listeria_monocytogenes_1639_v132	0.9096
Listeria_monocytogenes_1639_v196	Listeria_monocytogenes_1639_v132	0.8964
Listeria_monocytogenes_1639_v195	Listeria_monocytogenes_1639_v132	0.9379
Listeria_monocytogenes_1639_v194	Listeria_monocytogenes_1639_v132	0.9395
Listeria_monocytogenes_1639_v193	Listeria_monocytogenes_1639_v132	0.9395
Listeria_monocytogenes_1639_v192	Listeria_monocytogenes_1639_v132	0.9077
Listeria_monocytogenes_1639_v191	Listeria_monocytogenes_1639_v132	0.9229
Listeria_monocytogenes_1639_v190	Listeria_monocytogenes_1639_v132	0.9215
Listeria_monocytogenes_1639_v18	Listeria_monocytogenes_1639_v132	0.9083
Listeria_monocytogenes_1639_v189	Listeria_monocytogenes_1639_v132	0.9208
Listeria_monocytogenes_1639_v188	Listeria_monocytogenes_1639_v132	0.9248
Listeria_monocytogenes_1639_v187	Listeria_monocytogenes_1639_v132	0.9211
Listeria_monocytogenes_1639_v186	Listeria_monocytogenes_1639_v132	0.9218
Listeria_monocytogenes_1639_v185	Listeria_monocytogenes_1639_v132	0.9215
Listeria_monocytogenes_1639_v184	Listeria_monocytogenes_1639_v132	0.9211
Listeria_monocytogenes_1639_v183	Listeria_monocytogenes_1639_v132	0.923
Listeria_monocytogenes_1639_v182	Listeria_monocytogenes_1639_v132	0.9252
Listeria_monocytogenes_1639_v181	Listeria_monocytogenes_1639_v132	0.9382
Listeria_monocytogenes_1639_v180	Listeria_monocytogenes_1639_v132	0.9382
Listeria_monocytogenes_1639_v17	Listeria_monocytogenes_1639_v132	0.9083
Listeria_monocytogenes_1639_v179	Listeria_monocytogenes_1639_v132	0.9143
Listeria_monocytogenes_1639_v178	Listeria_monocytogenes_1639_v132	0.9238
Listeria_monocytogenes_1639_v177	Listeria_monocytogenes_1639_v132	0.9229
Listeria_monocytogenes_1639_v176	Listeria_monocytogenes_1639_v132	0.9415
Listeria_monocytogenes_1639_v175	Listeria_monocytogenes_1639_v132	0.9143
Listeria_monocytogenes_1639_v174	Listeria_monocytogenes_1639_v132	0.9212
Listeria_monocytogenes_1639_v173	Listeria_monocytogenes_1639_v132	0.9247
Listeria_monocytogenes_1639_v172	Listeria_monocytogenes_1639_v132	0.9322
Listeria_monocytogenes_1639_v171	Listeria_monocytogenes_1639_v132	0.9111
Listeria_monocytogenes_1639_v170	Listeria_monocytogenes_1639_v132	0.9171
Listeria_monocytogenes_1639_v16	Listeria_monocytogenes_1639_v132	0.8883
Listeria_monocytogenes_1639_v169	Listeria_monocytogenes_1639_v132	0.9165
Listeria_monocytogenes_1639_v168	Listeria_monocytogenes_1639_v132	0.9194
Listeria_monocytogenes_1639_v167	Listeria_monocytogenes_1639_v132	0.9175
Listeria_monocytogenes_1639_v166	Listeria_monocytogenes_1639_v132	0.9198
Listeria_monocytogenes_1639_v165	Listeria_monocytogenes_1639_v132	0.9165
Listeria_monocytogenes_1639_v164	Listeria_monocytogenes_1639_v132	0.9238
Listeria_monocytogenes_1639_v163	Listeria_monocytogenes_1639_v132	0.9193
Listeria_monocytogenes_1639_v162	Listeria_monocytogenes_1639_v132	0.9238
Listeria_monocytogenes_1639_v161	Listeria_monocytogenes_1639_v132	0.9234
Listeria_monocytogenes_1639_v160	Listeria_monocytogenes_1639_v132	0.9235
Listeria_monocytogenes_1639_v15	Listeria_monocytogenes_1639_v132	0.8969
Listeria_monocytogenes_1639_v159	Listeria_monocytogenes_1639_v132	0.9229
Listeria_monocytogenes_1639_v158	Listeria_monocytogenes_1639_v132	0.9231
Listeria_monocytogenes_1639_v157	Listeria_monocytogenes_1639_v132	0.9231
Listeria_monocytogenes_1639_v156	Listeria_monocytogenes_1639_v132	0.9122
Listeria_monocytogenes_1639_v155	Listeria_monocytogenes_1639_v132	0.9174
Listeria_monocytogenes_1639_v154	Listeria_monocytogenes_1639_v132	0.9118
Listeria_monocytogenes_1639_v153	Listeria_monocytogenes_1639_v132	0.9264
Listeria_monocytogenes_1639_v152	Listeria_monocytogenes_1639_v132	0.9232
Listeria_monocytogenes_1639_v151	Listeria_monocytogenes_1639_v132	0.9232
Listeria_monocytogenes_1639_v150	Listeria_monocytogenes_1639_v132	0.9137
Listeria_monocytogenes_1639_v14	Listeria_monocytogenes_1639_v132	0.9046
Listeria_monocytogenes_1639_v149	Listeria_monocytogenes_1639_v132	0.9131
Listeria_monocytogenes_1639_v148	Listeria_monocytogenes_1639_v132	0.9222
Listeria_monocytogenes_1639_v147	Listeria_monocytogenes_1639_v132	0.9118
Listeria_monocytogenes_1639_v146	Listeria_monocytogenes_1639_v132	0.9217
Listeria_monocytogenes_1639_v145	Listeria_monocytogenes_1639_v132	0.9174
Listeria_monocytogenes_1639_v144	Listeria_monocytogenes_1639_v132	0.9276
Listeria_monocytogenes_1639_v143	Listeria_monocytogenes_1639_v132	0.9199
Listeria_monocytogenes_1639_v142	Listeria_monocytogenes_1639_v132	0.928

Listeria_monocytogenes_1639_v141	Listeria_monocytogenes_1639_v132	0.9229
Listeria_monocytogenes_1639_v140	Listeria_monocytogenes_1639_v132	0.9283
Listeria_monocytogenes_1639_v13	Listeria_monocytogenes_1639_v132	0.8973
Listeria_monocytogenes_1639_v139	Listeria_monocytogenes_1639_v132	0.9193
Listeria_monocytogenes_1639_v138	Listeria_monocytogenes_1639_v132	0.9276
Listeria_monocytogenes_1639_v137	Listeria_monocytogenes_1639_v132	0.9272
Listeria_monocytogenes_1639_v136	Listeria_monocytogenes_1639_v132	0.9126
Listeria_monocytogenes_1639_v135	Listeria_monocytogenes_1639_v132	0.9372
Listeria_monocytogenes_1639_v134	Listeria_monocytogenes_1639_v132	0.9174
Listeria_monocytogenes_1639_v133	Listeria_monocytogenes_1639_v132	0.9171
Listeria_monocytogenes_1639_v132	Listeria_monocytogenes_1639_v132	0.0617
Listeria_welshimeri_1643	Listeria_monocytogenes_1639_v133	0.8728
Listeria_welshimeri_serovar_6b_str_SLCC5334_386043	Listeria_monocytogenes_1639_v133	0.8725
Listeria_seeligeri_serovar_1_2b_str_SLCC3954_683837	Listeria_monocytogenes_1639_v133	0.8858
Listeria_seeligeri_FSL_S4-171_702452	Listeria_monocytogenes_1639_v133	0.8258
Listeria_seeligeri_FSL_N1-067_702453	Listeria_monocytogenes_1639_v133	0.8386
Listeria_seeligeri_1640_v1	Listeria_monocytogenes_1639_v133	0.8846
Listeria_seeligeri_1640	Listeria_monocytogenes_1639_v133	0.8812
Listeria_monocytogenes_WSLC1042_1457188	Listeria_monocytogenes_1639_v133	0.933
Listeria_monocytogenes_WSLC1001_1457187	Listeria_monocytogenes_1639_v133	0.9534
Listeria_monocytogenes_SLCC7179_879090	Listeria_monocytogenes_1639_v133	0.9598
Listeria_monocytogenes_SLCC5850_882096	Listeria_monocytogenes_1639_v133	0.957
Listeria_monocytogenes_SLCC2755_932919	Listeria_monocytogenes_1639_v133	0.9358
Listeria_monocytogenes_SLCC2540_879089	Listeria_monocytogenes_1639_v133	0.9404
Listeria_monocytogenes_SLCC2479_882020	Listeria_monocytogenes_1639_v133	0.9544
Listeria_monocytogenes_SLCC2378_879088	Listeria_monocytogenes_1639_v133	0.934
Listeria_monocytogenes_SLCC2376_882097	Listeria_monocytogenes_1639_v133	0.9422
Listeria_monocytogenes_SLCC2372_932920	Listeria_monocytogenes_1639_v133	0.9477
Listeria_monocytogenes_serotype_4b_str_LL195_1230340	Listeria_monocytogenes_1639_v133	0.9398
Listeria_monocytogenes_serotype_4b_str_LIS0087_1134669	Listeria_monocytogenes_1639_v133	0.9369
Listeria_monocytogenes_serotype_4b_str_F2365_265669	Listeria_monocytogenes_1639_v133	0.9398
Listeria_monocytogenes_serotype_4b_str_CLIP_80459_568819	Listeria_monocytogenes_1639_v133	0.9435
Listeria_monocytogenes_serotype_4b_str_81-0861_1196161	Listeria_monocytogenes_1639_v133	0.9272
Listeria_monocytogenes_serotype_4b_str_81-0592_1196160	Listeria_monocytogenes_1639_v133	0.9283
Listeria_monocytogenes_serotype_4b_str_81-0558_1196159	Listeria_monocytogenes_1639_v133	0.9279
Listeria_monocytogenes_serotype_4b_str_10-0809_1196162	Listeria_monocytogenes_1639_v133	0.9283
Listeria_monocytogenes_serotype_4b_str_02-6680_1196175	Listeria_monocytogenes_1639_v133	0.939
Listeria_monocytogenes_serotype_4b_str_02-6679_1196174	Listeria_monocytogenes_1639_v133	0.939
Listeria_monocytogenes_serotype_4b_str_02-1792_1196173	Listeria_monocytogenes_1639_v133	0.9298
Listeria_monocytogenes_serotype_4b_str_02-1289_1196172	Listeria_monocytogenes_1639_v133	0.9298
Listeria_monocytogenes_serotype_4b_str_02-1103_1196171	Listeria_monocytogenes_1639_v133	0.9298
Listeria_monocytogenes_serotype_3c_str_10-5027_1196195	Listeria_monocytogenes_1639_v133	0.9563
Listeria_monocytogenes_serotype_1_2c_str_10-5026_1196194	Listeria_monocytogenes_1639_v133	0.9571
Listeria_monocytogenes_serotype_1_2c_str_10-5025_1196193	Listeria_monocytogenes_1639_v133	0.9571
Listeria_monocytogenes_serotype_1_2b_str_10-0811_1196164	Listeria_monocytogenes_1639_v133	0.934
Listeria_monocytogenes_serotype_1_2b_str_10-0810_1196163	Listeria_monocytogenes_1639_v133	0.934
Listeria_monocytogenes_serotype_1_2a_str_99-6370_1196179	Listeria_monocytogenes_1639_v133	0.9431
Listeria_monocytogenes_serotype_1_2a_str_98-2035_1196178	Listeria_monocytogenes_1639_v133	0.9431
Listeria_monocytogenes_serotype_1_2a_str_95-0093_1196177	Listeria_monocytogenes_1639_v133	0.9487
Listeria_monocytogenes_serotype_1_2a_str_88-0478_1196176	Listeria_monocytogenes_1639_v133	0.9484
Listeria_monocytogenes_serotype_1_2a_str_10-5024_1196192	Listeria_monocytogenes_1639_v133	0.9487
Listeria_monocytogenes_serotype_1_2a_str_10-4758_1196168	Listeria_monocytogenes_1639_v133	0.957
Listeria_monocytogenes_serotype_1_2a_str_10-4754_1196167	Listeria_monocytogenes_1639_v133	0.9574
Listeria_monocytogenes_serotype_1_2a_str_10-1321_1196191	Listeria_monocytogenes_1639_v133	0.9487
Listeria_monocytogenes_serotype_1_2a_str_10-1047_1196190	Listeria_monocytogenes_1639_v133	0.9495
Listeria_monocytogenes_serotype_1_2a_str_10-1046_1196189	Listeria_monocytogenes_1639_v133	0.9495
Listeria_monocytogenes_serotype_1_2a_str_10-0934_1196170	Listeria_monocytogenes_1639_v133	0.9657
Listeria_monocytogenes_serotype_1_2a_str_10-0933_1196169	Listeria_monocytogenes_1639_v133	0.9657
Listeria_monocytogenes_serotype_1_2a_str_10-0815_1196188	Listeria_monocytogenes_1639_v133	0.9445
Listeria_monocytogenes_serotype_1_2a_str_10-0814_1196187	Listeria_monocytogenes_1639_v133	0.9487
Listeria_monocytogenes_serotype_1_2a_str_10-0813_1196166	Listeria_monocytogenes_1639_v133	0.9535
Listeria_monocytogenes_serotype_1_2a_str_10-0812_1196165	Listeria_monocytogenes_1639_v133	0.9535
Listeria_monocytogenes_serotype_1_2a_str_08-7669_1196186	Listeria_monocytogenes_1639_v133	0.9577
Listeria_monocytogenes_serotype_1_2a_str_08-7374_1196185	Listeria_monocytogenes_1639_v133	0.9487
Listeria_monocytogenes_serotype_1_2a_str_08-6997_1196184	Listeria_monocytogenes_1639_v133	0.9445
Listeria_monocytogenes_serotype_1_2a_str_08-6569_1196183	Listeria_monocytogenes_1639_v133	0.9445
Listeria_monocytogenes_serotype_1_2a_str_08-6056_1196182	Listeria_monocytogenes_1639_v133	0.9445
Listeria_monocytogenes_serotype_1_2a_str_04-5457_1196181	Listeria_monocytogenes_1639_v133	0.9487
Listeria_monocytogenes_serotype_1_2a_str_02-5993_1196180	Listeria_monocytogenes_1639_v133	0.9487
Listeria_monocytogenes_R479a_1437838	Listeria_monocytogenes_1639_v133	0.9495
Listeria_monocytogenes_N53-1_1234142	Listeria_monocytogenes_1639_v133	0.9104
Listeria_monocytogenes_M7_1030009	Listeria_monocytogenes_1639_v133	0.9062
Listeria_monocytogenes_LO28_393132	Listeria_monocytogenes_1639_v133	0.9504
Listeria_monocytogenes_L99_563174	Listeria_monocytogenes_1639_v133	0.9066
Listeria_monocytogenes_L312_882094	Listeria_monocytogenes_1639_v133	0.9435
Listeria_monocytogenes_J1816_930781	Listeria_monocytogenes_1639_v133	0.932
Listeria_monocytogenes_J1-220_930782	Listeria_monocytogenes_1639_v133	0.9214
Listeria_monocytogenes_J0161_393130	Listeria_monocytogenes_1639_v133	0.9616
Listeria_monocytogenes_HCC23_552536	Listeria_monocytogenes_1639_v133	0.9059
Listeria_monocytogenes_FSL_R2-561_393126	Listeria_monocytogenes_1639_v133	0.9537
Listeria_monocytogenes_FSL_J1-208_393119	Listeria_monocytogenes_1639_v133	0.9085
Listeria_monocytogenes_FSL_J1-175_393118	Listeria_monocytogenes_1639_v133	0.9467
Listeria_monocytogenes_Finland_1998_393127	Listeria_monocytogenes_1639_v133	0.9661
Listeria_monocytogenes_EGD-e_169963_v1	Listeria_monocytogenes_1639_v133	0.9571
Listeria_monocytogenes_EGD-e_169963	Listeria_monocytogenes_1639_v133	0.9571
Listeria_monocytogenes_EGD_1334565	Listeria_monocytogenes_1639_v133	0.9577
Listeria_monocytogenes_CFSAN002349_1299895	Listeria_monocytogenes_1639_v133	0.9391
Listeria_monocytogenes_ATCC_19117_882095_v1	Listeria_monocytogenes_1639_v133	0.9361
Listeria_monocytogenes_ATCC_19117_882095	Listeria_monocytogenes_1639_v133	0.9358
Listeria_monocytogenes_6179_1288295	Listeria_monocytogenes_1639_v133	0.947
Listeria_monocytogenes_1639_v9	Listeria_monocytogenes_1639_v133	0.9312

Listeria_monocytogenes_1639_v99	Listeria_monocytogenes_1639_v133	0.932
Listeria_monocytogenes_1639_v98	Listeria_monocytogenes_1639_v133	0.9571
Listeria_monocytogenes_1639_v97	Listeria_monocytogenes_1639_v133	0.9383
Listeria_monocytogenes_1639_v96	Listeria_monocytogenes_1639_v133	0.9321
Listeria_monocytogenes_1639_v95	Listeria_monocytogenes_1639_v133	0.9321
Listeria_monocytogenes_1639_v94	Listeria_monocytogenes_1639_v133	0.9324
Listeria_monocytogenes_1639_v93	Listeria_monocytogenes_1639_v133	0.945
Listeria_monocytogenes_1639_v92	Listeria_monocytogenes_1639_v133	0.932
Listeria_monocytogenes_1639_v91	Listeria_monocytogenes_1639_v133	0.9259
Listeria_monocytogenes_1639_v90	Listeria_monocytogenes_1639_v133	0.9571
Listeria_monocytogenes_1639_v8	Listeria_monocytogenes_1639_v133	0.9379
Listeria_monocytogenes_1639_v89	Listeria_monocytogenes_1639_v133	0.9372
Listeria_monocytogenes_1639_v88	Listeria_monocytogenes_1639_v133	0.9573
Listeria_monocytogenes_1639_v87	Listeria_monocytogenes_1639_v133	0.9489
Listeria_monocytogenes_1639_v86	Listeria_monocytogenes_1639_v133	0.9489
Listeria_monocytogenes_1639_v85	Listeria_monocytogenes_1639_v133	0.9281
Listeria_monocytogenes_1639_v84	Listeria_monocytogenes_1639_v133	0.9595
Listeria_monocytogenes_1639_v83	Listeria_monocytogenes_1639_v133	0.9305
Listeria_monocytogenes_1639_v82	Listeria_monocytogenes_1639_v133	0.9301
Listeria_monocytogenes_1639_v81	Listeria_monocytogenes_1639_v133	0.9573
Listeria_monocytogenes_1639_v80	Listeria_monocytogenes_1639_v133	0.9398
Listeria_monocytogenes_1639_v7	Listeria_monocytogenes_1639_v133	0.9379
Listeria_monocytogenes_1639_v79	Listeria_monocytogenes_1639_v133	0.9567
Listeria_monocytogenes_1639_v78	Listeria_monocytogenes_1639_v133	0.957
Listeria_monocytogenes_1639_v77	Listeria_monocytogenes_1639_v133	0.9595
Listeria_monocytogenes_1639_v76	Listeria_monocytogenes_1639_v133	0.9562
Listeria_monocytogenes_1639_v75	Listeria_monocytogenes_1639_v133	0.9066
Listeria_monocytogenes_1639_v74	Listeria_monocytogenes_1639_v133	0.9455
Listeria_monocytogenes_1639_v73	Listeria_monocytogenes_1639_v133	0.945
Listeria_monocytogenes_1639_v72	Listeria_monocytogenes_1639_v133	0.9274
Listeria_monocytogenes_1639_v71	Listeria_monocytogenes_1639_v133	0.944
Listeria_monocytogenes_1639_v70	Listeria_monocytogenes_1639_v133	0.952
Listeria_monocytogenes_1639_v6	Listeria_monocytogenes_1639_v133	0.9376
Listeria_monocytogenes_1639_v69	Listeria_monocytogenes_1639_v133	0.9283
Listeria_monocytogenes_1639_v68	Listeria_monocytogenes_1639_v133	0.9528
Listeria_monocytogenes_1639_v67	Listeria_monocytogenes_1639_v133	0.9471
Listeria_monocytogenes_1639_v66	Listeria_monocytogenes_1639_v133	0.9567
Listeria_monocytogenes_1639_v65	Listeria_monocytogenes_1639_v133	0.9463
Listeria_monocytogenes_1639_v64	Listeria_monocytogenes_1639_v133	0.9491
Listeria_monocytogenes_1639_v63	Listeria_monocytogenes_1639_v133	0.9399
Listeria_monocytogenes_1639_v62	Listeria_monocytogenes_1639_v133	0.9534
Listeria_monocytogenes_1639_v61	Listeria_monocytogenes_1639_v133	0.9516
Listeria_monocytogenes_1639_v60	Listeria_monocytogenes_1639_v133	0.9622
Listeria_monocytogenes_1639_v5	Listeria_monocytogenes_1639_v133	0.9011
Listeria_monocytogenes_1639_v59	Listeria_monocytogenes_1639_v133	0.927
Listeria_monocytogenes_1639_v58	Listeria_monocytogenes_1639_v133	0.9301
Listeria_monocytogenes_1639_v57	Listeria_monocytogenes_1639_v133	0.9275
Listeria_monocytogenes_1639_v56	Listeria_monocytogenes_1639_v133	0.9346
Listeria_monocytogenes_1639_v55	Listeria_monocytogenes_1639_v133	0.9255
Listeria_monocytogenes_1639_v54	Listeria_monocytogenes_1639_v133	0.9303
Listeria_monocytogenes_1639_v53	Listeria_monocytogenes_1639_v133	0.9471
Listeria_monocytogenes_1639_v52	Listeria_monocytogenes_1639_v133	0.9277
Listeria_monocytogenes_1639_v51	Listeria_monocytogenes_1639_v133	0.9256
Listeria_monocytogenes_1639_v50	Listeria_monocytogenes_1639_v133	0.9473
Listeria_monocytogenes_1639_v4	Listeria_monocytogenes_1639_v133	0.9143
Listeria_monocytogenes_1639_v49	Listeria_monocytogenes_1639_v133	0.9242
Listeria_monocytogenes_1639_v48	Listeria_monocytogenes_1639_v133	0.9525
Listeria_monocytogenes_1639_v47	Listeria_monocytogenes_1639_v133	0.9484
Listeria_monocytogenes_1639_v46	Listeria_monocytogenes_1639_v133	0.9589
Listeria_monocytogenes_1639_v45	Listeria_monocytogenes_1639_v133	0.9393
Listeria_monocytogenes_1639_v44	Listeria_monocytogenes_1639_v133	0.9487
Listeria_monocytogenes_1639_v43	Listeria_monocytogenes_1639_v133	0.9487
Listeria_monocytogenes_1639_v42	Listeria_monocytogenes_1639_v133	0.9479
Listeria_monocytogenes_1639_v41	Listeria_monocytogenes_1639_v133	0.9232
Listeria_monocytogenes_1639_v40	Listeria_monocytogenes_1639_v133	0.9473
Listeria_monocytogenes_1639_v3	Listeria_monocytogenes_1639_v133	0.9146
Listeria_monocytogenes_1639_v39	Listeria_monocytogenes_1639_v133	0.9451
Listeria_monocytogenes_1639_v38	Listeria_monocytogenes_1639_v133	0.9441
Listeria_monocytogenes_1639_v37	Listeria_monocytogenes_1639_v133	0.9388
Listeria_monocytogenes_1639_v36	Listeria_monocytogenes_1639_v133	0.9393
Listeria_monocytogenes_1639_v35	Listeria_monocytogenes_1639_v133	0.948
Listeria_monocytogenes_1639_v34	Listeria_monocytogenes_1639_v133	0.9521
Listeria_monocytogenes_1639_v33	Listeria_monocytogenes_1639_v133	0.934
Listeria_monocytogenes_1639_v32	Listeria_monocytogenes_1639_v133	0.9393
Listeria_monocytogenes_1639_v31	Listeria_monocytogenes_1639_v133	0.9524
Listeria_monocytogenes_1639_v30	Listeria_monocytogenes_1639_v133	0.9354
Listeria_monocytogenes_1639_v2	Listeria_monocytogenes_1639_v133	0.9094
Listeria_monocytogenes_1639_v29	Listeria_monocytogenes_1639_v133	0.9425
Listeria_monocytogenes_1639_v28	Listeria_monocytogenes_1639_v133	0.9471
Listeria_monocytogenes_1639_v27	Listeria_monocytogenes_1639_v133	0.9314
Listeria_monocytogenes_1639_v26	Listeria_monocytogenes_1639_v133	0.9311
Listeria_monocytogenes_1639_v25	Listeria_monocytogenes_1639_v133	0.9314
Listeria_monocytogenes_1639_v24	Listeria_monocytogenes_1639_v133	0.9221
Listeria_monocytogenes_1639_v23	Listeria_monocytogenes_1639_v133	0.9218
Listeria_monocytogenes_1639_v22	Listeria_monocytogenes_1639_v133	0.9452
Listeria_monocytogenes_1639_v21	Listeria_monocytogenes_1639_v133	0.9233
Listeria_monocytogenes_1639_v20	Listeria_monocytogenes_1639_v133	0.9403
Listeria_monocytogenes_1639_v1	Listeria_monocytogenes_1639_v133	0.9415
Listeria_monocytogenes_1639_v19	Listeria_monocytogenes_1639_v133	0.94
Listeria_monocytogenes_1639_v198	Listeria_monocytogenes_1639_v133	0.9117
Listeria_monocytogenes_1639_v197	Listeria_monocytogenes_1639_v133	0.9429

Listeria_monocytogenes_1639_v196	Listeria_monocytogenes_1639_v133	0.9407
Listeria_monocytogenes_1639_v195	Listeria_monocytogenes_1639_v133	0.9436
Listeria_monocytogenes_1639_v194	Listeria_monocytogenes_1639_v133	0.9422
Listeria_monocytogenes_1639_v193	Listeria_monocytogenes_1639_v133	0.9426
Listeria_monocytogenes_1639_v192	Listeria_monocytogenes_1639_v133	0.9
Listeria_monocytogenes_1639_v191	Listeria_monocytogenes_1639_v133	0.9656
Listeria_monocytogenes_1639_v190	Listeria_monocytogenes_1639_v133	0.9668
Listeria_monocytogenes_1639_v18	Listeria_monocytogenes_1639_v133	0.9227
Listeria_monocytogenes_1639_v189	Listeria_monocytogenes_1639_v133	0.9657
Listeria_monocytogenes_1639_v188	Listeria_monocytogenes_1639_v133	0.9656
Listeria_monocytogenes_1639_v187	Listeria_monocytogenes_1639_v133	0.9668
Listeria_monocytogenes_1639_v186	Listeria_monocytogenes_1639_v133	0.9668
Listeria_monocytogenes_1639_v185	Listeria_monocytogenes_1639_v133	0.9672
Listeria_monocytogenes_1639_v184	Listeria_monocytogenes_1639_v133	0.9664
Listeria_monocytogenes_1639_v183	Listeria_monocytogenes_1639_v133	0.9672
Listeria_monocytogenes_1639_v182	Listeria_monocytogenes_1639_v133	0.9694
Listeria_monocytogenes_1639_v181	Listeria_monocytogenes_1639_v133	0.9205
Listeria_monocytogenes_1639_v180	Listeria_monocytogenes_1639_v133	0.9205
Listeria_monocytogenes_1639_v17	Listeria_monocytogenes_1639_v133	0.9231
Listeria_monocytogenes_1639_v179	Listeria_monocytogenes_1639_v133	0.9652
Listeria_monocytogenes_1639_v178	Listeria_monocytogenes_1639_v133	0.9653
Listeria_monocytogenes_1639_v177	Listeria_monocytogenes_1639_v133	0.9398
Listeria_monocytogenes_1639_v176	Listeria_monocytogenes_1639_v133	0.9372
Listeria_monocytogenes_1639_v175	Listeria_monocytogenes_1639_v133	0.9591
Listeria_monocytogenes_1639_v174	Listeria_monocytogenes_1639_v133	0.9676
Listeria_monocytogenes_1639_v173	Listeria_monocytogenes_1639_v133	0.9534
Listeria_monocytogenes_1639_v172	Listeria_monocytogenes_1639_v133	0.939
Listeria_monocytogenes_1639_v171	Listeria_monocytogenes_1639_v133	0.9552
Listeria_monocytogenes_1639_v170	Listeria_monocytogenes_1639_v133	0.9624
Listeria_monocytogenes_1639_v16	Listeria_monocytogenes_1639_v133	0.9352
Listeria_monocytogenes_1639_v169	Listeria_monocytogenes_1639_v133	0.9628
Listeria_monocytogenes_1639_v168	Listeria_monocytogenes_1639_v133	0.9592
Listeria_monocytogenes_1639_v167	Listeria_monocytogenes_1639_v133	0.9674
Listeria_monocytogenes_1639_v166	Listeria_monocytogenes_1639_v133	0.9596
Listeria_monocytogenes_1639_v165	Listeria_monocytogenes_1639_v133	0.9625
Listeria_monocytogenes_1639_v164	Listeria_monocytogenes_1639_v133	0.9393
Listeria_monocytogenes_1639_v163	Listeria_monocytogenes_1639_v133	0.9478
Listeria_monocytogenes_1639_v162	Listeria_monocytogenes_1639_v133	0.9393
Listeria_monocytogenes_1639_v161	Listeria_monocytogenes_1639_v133	0.9401
Listeria_monocytogenes_1639_v160	Listeria_monocytogenes_1639_v133	0.9397
Listeria_monocytogenes_1639_v15	Listeria_monocytogenes_1639_v133	0.9225
Listeria_monocytogenes_1639_v159	Listeria_monocytogenes_1639_v133	0.9398
Listeria_monocytogenes_1639_v158	Listeria_monocytogenes_1639_v133	0.9398
Listeria_monocytogenes_1639_v157	Listeria_monocytogenes_1639_v133	0.9398
Listeria_monocytogenes_1639_v156	Listeria_monocytogenes_1639_v133	0.9469
Listeria_monocytogenes_1639_v155	Listeria_monocytogenes_1639_v133	0.9672
Listeria_monocytogenes_1639_v154	Listeria_monocytogenes_1639_v133	0.9588
Listeria_monocytogenes_1639_v153	Listeria_monocytogenes_1639_v133	0.9583
Listeria_monocytogenes_1639_v152	Listeria_monocytogenes_1639_v133	0.9398
Listeria_monocytogenes_1639_v151	Listeria_monocytogenes_1639_v133	0.9398
Listeria_monocytogenes_1639_v150	Listeria_monocytogenes_1639_v133	0.9599
Listeria_monocytogenes_1639_v14	Listeria_monocytogenes_1639_v133	0.9234
Listeria_monocytogenes_1639_v149	Listeria_monocytogenes_1639_v133	0.9581
Listeria_monocytogenes_1639_v148	Listeria_monocytogenes_1639_v133	0.9391
Listeria_monocytogenes_1639_v147	Listeria_monocytogenes_1639_v133	0.9253
Listeria_monocytogenes_1639_v146	Listeria_monocytogenes_1639_v133	0.9707
Listeria_monocytogenes_1639_v145	Listeria_monocytogenes_1639_v133	0.9616
Listeria_monocytogenes_1639_v144	Listeria_monocytogenes_1639_v133	0.9428
Listeria_monocytogenes_1639_v143	Listeria_monocytogenes_1639_v133	0.9742
Listeria_monocytogenes_1639_v142	Listeria_monocytogenes_1639_v133	0.9432
Listeria_monocytogenes_1639_v141	Listeria_monocytogenes_1639_v133	0.9485
Listeria_monocytogenes_1639_v140	Listeria_monocytogenes_1639_v133	0.9439
Listeria_monocytogenes_1639_v13	Listeria_monocytogenes_1639_v133	0.9225
Listeria_monocytogenes_1639_v139	Listeria_monocytogenes_1639_v133	0.9367
Listeria_monocytogenes_1639_v138	Listeria_monocytogenes_1639_v133	0.9432
Listeria_monocytogenes_1639_v137	Listeria_monocytogenes_1639_v133	0.9428
Listeria_monocytogenes_1639_v136	Listeria_monocytogenes_1639_v133	0.9578
Listeria_monocytogenes_1639_v135	Listeria_monocytogenes_1639_v133	0.9182
Listeria_monocytogenes_1639_v134	Listeria_monocytogenes_1639_v133	0.9339
Listeria_monocytogenes_1639_v133	Listeria_monocytogenes_1639_v133	0.066
Listeria_welshimeri_1643	Listeria_monocytogenes_1639_v134	0.8876
Listeria_welshimeri_serovar_6b_str_SLCC5334_386043	Listeria_monocytogenes_1639_v134	0.8869
Listeria_seeligeri_serovar_1_2b_str_SLCC3954_683837	Listeria_monocytogenes_1639_v134	0.8825
Listeria_seeligeri_FSL_S4-171_702452	Listeria_monocytogenes_1639_v134	0.8243
Listeria_seeligeri_FSL_N1-067_702453	Listeria_monocytogenes_1639_v134	0.828
Listeria_seeligeri_1640_v1	Listeria_monocytogenes_1639_v134	0.8936
Listeria_seeligeri_1640	Listeria_monocytogenes_1639_v134	0.8814
Listeria_monocytogenes_WSLC1042_1457188	Listeria_monocytogenes_1639_v134	0.995
Listeria_monocytogenes_WSLC1001_1457187	Listeria_monocytogenes_1639_v134	0.9255
Listeria_monocytogenes_SLCC7179_879090	Listeria_monocytogenes_1639_v134	0.936
Listeria_monocytogenes_SLCC5850_882096	Listeria_monocytogenes_1639_v134	0.9302
Listeria_monocytogenes_SLCC2755_932919	Listeria_monocytogenes_1639_v134	0.9455
Listeria_monocytogenes_SLCC2540_879089	Listeria_monocytogenes_1639_v134	0.9502
Listeria_monocytogenes_SLCC2479_882020	Listeria_monocytogenes_1639_v134	0.9264
Listeria_monocytogenes_SLCC2378_879088	Listeria_monocytogenes_1639_v134	0.9966
Listeria_monocytogenes_SLCC2376_882097	Listeria_monocytogenes_1639_v134	0.9397
Listeria_monocytogenes_SLCC2372_932920	Listeria_monocytogenes_1639_v134	0.9192
Listeria_monocytogenes_serotype_4b_str_LL195_1230340	Listeria_monocytogenes_1639_v134	0.9927
Listeria_monocytogenes_serotype_4b_str_LIS0087_1134669	Listeria_monocytogenes_1639_v134	0.9057
Listeria_monocytogenes_serotype_4b_str_F2365_265669	Listeria_monocytogenes_1639_v134	0.9927
Listeria_monocytogenes_serotype_4b_str_CLIP_80459_568819	Listeria_monocytogenes_1639_v134	0.9727

Listeria_monocytogenes_serotype_4b_str_81-0861_1196161	Listeria_monocytogenes_1639_v134	0.9845
Listeria_monocytogenes_serotype_4b_str_81-0592_1196160	Listeria_monocytogenes_1639_v134	0.9856
Listeria_monocytogenes_serotype_4b_str_81-0558_1196159	Listeria_monocytogenes_1639_v134	0.9852
Listeria_monocytogenes_serotype_4b_str_10-0809_1196162	Listeria_monocytogenes_1639_v134	0.986
Listeria_monocytogenes_serotype_4b_str_02-6680_1196175	Listeria_monocytogenes_1639_v134	0.9727
Listeria_monocytogenes_serotype_4b_str_02-6679_1196174	Listeria_monocytogenes_1639_v134	0.9727
Listeria_monocytogenes_serotype_4b_str_02-1792_1196173	Listeria_monocytogenes_1639_v134	0.9864
Listeria_monocytogenes_serotype_4b_str_02-1289_1196172	Listeria_monocytogenes_1639_v134	0.9864
Listeria_monocytogenes_serotype_4b_str_02-1103_1196171	Listeria_monocytogenes_1639_v134	0.9864
Listeria_monocytogenes_serotype_3c_str_10-5027_1196195	Listeria_monocytogenes_1639_v134	0.9257
Listeria_monocytogenes_serotype_1_2c_str_10-5026_1196194	Listeria_monocytogenes_1639_v134	0.9268
Listeria_monocytogenes_serotype_1_2c_str_10-5025_1196193	Listeria_monocytogenes_1639_v134	0.9268
Listeria_monocytogenes_serotype_1_2b_str_10-0811_1196164	Listeria_monocytogenes_1639_v134	0.938
Listeria_monocytogenes_serotype_1_2b_str_10-0810_1196163	Listeria_monocytogenes_1639_v134	0.938
Listeria_monocytogenes_serotype_1_2a_str_99-6370_1196179	Listeria_monocytogenes_1639_v134	0.9118
Listeria_monocytogenes_serotype_1_2a_str_98-2035_1196178	Listeria_monocytogenes_1639_v134	0.9118
Listeria_monocytogenes_serotype_1_2a_str_95-0093_1196177	Listeria_monocytogenes_1639_v134	0.9172
Listeria_monocytogenes_serotype_1_2a_str_88-0478_1196176	Listeria_monocytogenes_1639_v134	0.9164
Listeria_monocytogenes_serotype_1_2a_str_10-5024_1196192	Listeria_monocytogenes_1639_v134	0.9175
Listeria_monocytogenes_serotype_1_2a_str_10-4758_1196168	Listeria_monocytogenes_1639_v134	0.9356
Listeria_monocytogenes_serotype_1_2a_str_10-4754_1196167	Listeria_monocytogenes_1639_v134	0.9356
Listeria_monocytogenes_serotype_1_2a_str_10-1321_1196191	Listeria_monocytogenes_1639_v134	0.9175
Listeria_monocytogenes_serotype_1_2a_str_10-1047_1196190	Listeria_monocytogenes_1639_v134	0.9179
Listeria_monocytogenes_serotype_1_2a_str_10-1046_1196189	Listeria_monocytogenes_1639_v134	0.9179
Listeria_monocytogenes_serotype_1_2a_str_10-0934_1196170	Listeria_monocytogenes_1639_v134	0.9429
Listeria_monocytogenes_serotype_1_2a_str_10-0933_1196169	Listeria_monocytogenes_1639_v134	0.9429
Listeria_monocytogenes_serotype_1_2a_str_10-0815_1196188	Listeria_monocytogenes_1639_v134	0.9131
Listeria_monocytogenes_serotype_1_2a_str_10-0814_1196187	Listeria_monocytogenes_1639_v134	0.9176
Listeria_monocytogenes_serotype_1_2a_str_10-0813_1196166	Listeria_monocytogenes_1639_v134	0.9228
Listeria_monocytogenes_serotype_1_2a_str_10-0812_1196165	Listeria_monocytogenes_1639_v134	0.9228
Listeria_monocytogenes_serotype_1_2a_str_08-7669_1196186	Listeria_monocytogenes_1639_v134	0.9265
Listeria_monocytogenes_serotype_1_2a_str_08-7374_1196185	Listeria_monocytogenes_1639_v134	0.9175
Listeria_monocytogenes_serotype_1_2a_str_08-6997_1196184	Listeria_monocytogenes_1639_v134	0.9131
Listeria_monocytogenes_serotype_1_2a_str_08-6569_1196183	Listeria_monocytogenes_1639_v134	0.9131
Listeria_monocytogenes_serotype_1_2a_str_08-6056_1196182	Listeria_monocytogenes_1639_v134	0.9131
Listeria_monocytogenes_serotype_1_2a_str_04-5457_1196181	Listeria_monocytogenes_1639_v134	0.9175
Listeria_monocytogenes_serotype_1_2a_str_02-5993_1196180	Listeria_monocytogenes_1639_v134	0.9172
Listeria_monocytogenes_R479a_1437838	Listeria_monocytogenes_1639_v134	0.9165
Listeria_monocytogenes_N53-1_1234142	Listeria_monocytogenes_1639_v134	0.8908
Listeria_monocytogenes_M7_1030009	Listeria_monocytogenes_1639_v134	0.9046
Listeria_monocytogenes_LO28_393132	Listeria_monocytogenes_1639_v134	0.925
Listeria_monocytogenes_L99_563174	Listeria_monocytogenes_1639_v134	0.9053
Listeria_monocytogenes_L312_882094	Listeria_monocytogenes_1639_v134	0.9731
Listeria_monocytogenes_J1816_930781	Listeria_monocytogenes_1639_v134	0.9492
Listeria_monocytogenes_J1-220_930782	Listeria_monocytogenes_1639_v134	0.9606
Listeria_monocytogenes_J0161_393130	Listeria_monocytogenes_1639_v134	0.9235
Listeria_monocytogenes_HCC23_552536	Listeria_monocytogenes_1639_v134	0.9046
Listeria_monocytogenes_FSL_R2-561_393126	Listeria_monocytogenes_1639_v134	0.925
Listeria_monocytogenes_FSL_J1-208_393119	Listeria_monocytogenes_1639_v134	0.9191
Listeria_monocytogenes_FSL_J1-175_393118	Listeria_monocytogenes_1639_v134	0.9535
Listeria_monocytogenes_Finland_1998_393127	Listeria_monocytogenes_1639_v134	0.9424
Listeria_monocytogenes_EGD-e_169963_v1	Listeria_monocytogenes_1639_v134	0.9349
Listeria_monocytogenes_EGD-e_169963	Listeria_monocytogenes_1639_v134	0.9353
Listeria_monocytogenes_EGD_1334565	Listeria_monocytogenes_1639_v134	0.9309
Listeria_monocytogenes_CFSAN002349_1299895	Listeria_monocytogenes_1639_v134	0.9728
Listeria_monocytogenes_ATCC_19117_882095_v1	Listeria_monocytogenes_1639_v134	0.9812
Listeria_monocytogenes_ATCC_19117_882095	Listeria_monocytogenes_1639_v134	0.9812
Listeria_monocytogenes_6179_1288295	Listeria_monocytogenes_1639_v134	0.9086
Listeria_monocytogenes_1639_v9	Listeria_monocytogenes_1639_v134	0.9244
Listeria_monocytogenes_1639_v99	Listeria_monocytogenes_1639_v134	0.9503
Listeria_monocytogenes_1639_v98	Listeria_monocytogenes_1639_v134	0.92
Listeria_monocytogenes_1639_v97	Listeria_monocytogenes_1639_v134	0.9643
Listeria_monocytogenes_1639_v96	Listeria_monocytogenes_1639_v134	0.9496
Listeria_monocytogenes_1639_v95	Listeria_monocytogenes_1639_v134	0.9496
Listeria_monocytogenes_1639_v94	Listeria_monocytogenes_1639_v134	0.9496
Listeria_monocytogenes_1639_v93	Listeria_monocytogenes_1639_v134	0.9228
Listeria_monocytogenes_1639_v92	Listeria_monocytogenes_1639_v134	0.95
Listeria_monocytogenes_1639_v91	Listeria_monocytogenes_1639_v134	0.9725
Listeria_monocytogenes_1639_v90	Listeria_monocytogenes_1639_v134	0.92
Listeria_monocytogenes_1639_v8	Listeria_monocytogenes_1639_v134	0.9078
Listeria_monocytogenes_1639_v89	Listeria_monocytogenes_1639_v134	0.96
Listeria_monocytogenes_1639_v88	Listeria_monocytogenes_1639_v134	0.924
Listeria_monocytogenes_1639_v87	Listeria_monocytogenes_1639_v134	0.919
Listeria_monocytogenes_1639_v86	Listeria_monocytogenes_1639_v134	0.9183
Listeria_monocytogenes_1639_v85	Listeria_monocytogenes_1639_v134	0.9838
Listeria_monocytogenes_1639_v84	Listeria_monocytogenes_1639_v134	0.9272
Listeria_monocytogenes_1639_v83	Listeria_monocytogenes_1639_v134	0.9697
Listeria_monocytogenes_1639_v82	Listeria_monocytogenes_1639_v134	0.969
Listeria_monocytogenes_1639_v81	Listeria_monocytogenes_1639_v134	0.9232
Listeria_monocytogenes_1639_v80	Listeria_monocytogenes_1639_v134	0.9458
Listeria_monocytogenes_1639_v7	Listeria_monocytogenes_1639_v134	0.9078
Listeria_monocytogenes_1639_v79	Listeria_monocytogenes_1639_v134	0.9211
Listeria_monocytogenes_1639_v78	Listeria_monocytogenes_1639_v134	0.9225
Listeria_monocytogenes_1639_v77	Listeria_monocytogenes_1639_v134	0.9294
Listeria_monocytogenes_1639_v76	Listeria_monocytogenes_1639_v134	0.9201
Listeria_monocytogenes_1639_v75	Listeria_monocytogenes_1639_v134	0.9053
Listeria_monocytogenes_1639_v74	Listeria_monocytogenes_1639_v134	0.9175
Listeria_monocytogenes_1639_v73	Listeria_monocytogenes_1639_v134	0.9206
Listeria_monocytogenes_1639_v72	Listeria_monocytogenes_1639_v134	0.9693
Listeria_monocytogenes_1639_v71	Listeria_monocytogenes_1639_v134	0.9065
Listeria_monocytogenes_1639_v70	Listeria_monocytogenes_1639_v134	0.923

Listeria_monocytogenes_1639_v6	Listeria_monocytogenes_1639_v134	0.9071
Listeria_monocytogenes_1639_v69	Listeria_monocytogenes_1639_v134	0.986
Listeria_monocytogenes_1639_v68	Listeria_monocytogenes_1639_v134	0.9372
Listeria_monocytogenes_1639_v67	Listeria_monocytogenes_1639_v134	0.9436
Listeria_monocytogenes_1639_v66	Listeria_monocytogenes_1639_v134	0.9315
Listeria_monocytogenes_1639_v65	Listeria_monocytogenes_1639_v134	0.9203
Listeria_monocytogenes_1639_v64	Listeria_monocytogenes_1639_v134	0.9267
Listeria_monocytogenes_1639_v63	Listeria_monocytogenes_1639_v134	0.9395
Listeria_monocytogenes_1639_v62	Listeria_monocytogenes_1639_v134	0.9186
Listeria_monocytogenes_1639_v61	Listeria_monocytogenes_1639_v134	0.9203
Listeria_monocytogenes_1639_v60	Listeria_monocytogenes_1639_v134	0.9254
Listeria_monocytogenes_1639_v5	Listeria_monocytogenes_1639_v134	0.9052
Listeria_monocytogenes_1639_v59	Listeria_monocytogenes_1639_v134	0.9705
Listeria_monocytogenes_1639_v58	Listeria_monocytogenes_1639_v134	0.9765
Listeria_monocytogenes_1639_v57	Listeria_monocytogenes_1639_v134	0.9468
Listeria_monocytogenes_1639_v56	Listeria_monocytogenes_1639_v134	0.9701
Listeria_monocytogenes_1639_v55	Listeria_monocytogenes_1639_v134	0.9425
Listeria_monocytogenes_1639_v54	Listeria_monocytogenes_1639_v134	0.9226
Listeria_monocytogenes_1639_v53	Listeria_monocytogenes_1639_v134	0.9292
Listeria_monocytogenes_1639_v52	Listeria_monocytogenes_1639_v134	0.9574
Listeria_monocytogenes_1639_v51	Listeria_monocytogenes_1639_v134	0.9642
Listeria_monocytogenes_1639_v50	Listeria_monocytogenes_1639_v134	0.9168
Listeria_monocytogenes_1639_v4	Listeria_monocytogenes_1639_v134	0.9182
Listeria_monocytogenes_1639_v49	Listeria_monocytogenes_1639_v134	0.9841
Listeria_monocytogenes_1639_v48	Listeria_monocytogenes_1639_v134	0.9209
Listeria_monocytogenes_1639_v47	Listeria_monocytogenes_1639_v134	0.9172
Listeria_monocytogenes_1639_v46	Listeria_monocytogenes_1639_v134	0.9365
Listeria_monocytogenes_1639_v45	Listeria_monocytogenes_1639_v134	0.9084
Listeria_monocytogenes_1639_v44	Listeria_monocytogenes_1639_v134	0.9175
Listeria_monocytogenes_1639_v43	Listeria_monocytogenes_1639_v134	0.9175
Listeria_monocytogenes_1639_v42	Listeria_monocytogenes_1639_v134	0.9143
Listeria_monocytogenes_1639_v41	Listeria_monocytogenes_1639_v134	0.8987
Listeria_monocytogenes_1639_v40	Listeria_monocytogenes_1639_v134	0.9254
Listeria_monocytogenes_1639_v3	Listeria_monocytogenes_1639_v134	0.9185
Listeria_monocytogenes_1639_v39	Listeria_monocytogenes_1639_v134	0.942
Listeria_monocytogenes_1639_v38	Listeria_monocytogenes_1639_v134	0.9305
Listeria_monocytogenes_1639_v37	Listeria_monocytogenes_1639_v134	0.9167
Listeria_monocytogenes_1639_v36	Listeria_monocytogenes_1639_v134	0.915
Listeria_monocytogenes_1639_v35	Listeria_monocytogenes_1639_v134	0.9261
Listeria_monocytogenes_1639_v34	Listeria_monocytogenes_1639_v134	0.9291
Listeria_monocytogenes_1639_v33	Listeria_monocytogenes_1639_v134	0.938
Listeria_monocytogenes_1639_v32	Listeria_monocytogenes_1639_v134	0.9156
Listeria_monocytogenes_1639_v31	Listeria_monocytogenes_1639_v134	0.9295
Listeria_monocytogenes_1639_v30	Listeria_monocytogenes_1639_v134	0.9409
Listeria_monocytogenes_1639_v2	Listeria_monocytogenes_1639_v134	0.9034
Listeria_monocytogenes_1639_v29	Listeria_monocytogenes_1639_v134	0.9124
Listeria_monocytogenes_1639_v28	Listeria_monocytogenes_1639_v134	0.9164
Listeria_monocytogenes_1639_v27	Listeria_monocytogenes_1639_v134	0.9076
Listeria_monocytogenes_1639_v26	Listeria_monocytogenes_1639_v134	0.9077
Listeria_monocytogenes_1639_v25	Listeria_monocytogenes_1639_v134	0.9073
Listeria_monocytogenes_1639_v24	Listeria_monocytogenes_1639_v134	0.9606
Listeria_monocytogenes_1639_v23	Listeria_monocytogenes_1639_v134	0.9606
Listeria_monocytogenes_1639_v22	Listeria_monocytogenes_1639_v134	0.9409
Listeria_monocytogenes_1639_v21	Listeria_monocytogenes_1639_v134	0.9603
Listeria_monocytogenes_1639_v20	Listeria_monocytogenes_1639_v134	0.9332
Listeria_monocytogenes_1639_v1	Listeria_monocytogenes_1639_v134	0.9024
Listeria_monocytogenes_1639_v19	Listeria_monocytogenes_1639_v134	0.9328
Listeria_monocytogenes_1639_v198	Listeria_monocytogenes_1639_v134	0.8938
Listeria_monocytogenes_1639_v197	Listeria_monocytogenes_1639_v134	0.9233
Listeria_monocytogenes_1639_v196	Listeria_monocytogenes_1639_v134	0.9134
Listeria_monocytogenes_1639_v195	Listeria_monocytogenes_1639_v134	0.9424
Listeria_monocytogenes_1639_v194	Listeria_monocytogenes_1639_v134	0.9405
Listeria_monocytogenes_1639_v193	Listeria_monocytogenes_1639_v134	0.9405
Listeria_monocytogenes_1639_v192	Listeria_monocytogenes_1639_v134	0.9093
Listeria_monocytogenes_1639_v191	Listeria_monocytogenes_1639_v134	0.9458
Listeria_monocytogenes_1639_v190	Listeria_monocytogenes_1639_v134	0.9424
Listeria_monocytogenes_1639_v18	Listeria_monocytogenes_1639_v134	0.9408
Listeria_monocytogenes_1639_v189	Listeria_monocytogenes_1639_v134	0.9416
Listeria_monocytogenes_1639_v188	Listeria_monocytogenes_1639_v134	0.9442
Listeria_monocytogenes_1639_v187	Listeria_monocytogenes_1639_v134	0.942
Listeria_monocytogenes_1639_v186	Listeria_monocytogenes_1639_v134	0.9427
Listeria_monocytogenes_1639_v185	Listeria_monocytogenes_1639_v134	0.9424
Listeria_monocytogenes_1639_v184	Listeria_monocytogenes_1639_v134	0.9416
Listeria_monocytogenes_1639_v183	Listeria_monocytogenes_1639_v134	0.9416
Listeria_monocytogenes_1639_v182	Listeria_monocytogenes_1639_v134	0.9438
Listeria_monocytogenes_1639_v181	Listeria_monocytogenes_1639_v134	0.9326
Listeria_monocytogenes_1639_v180	Listeria_monocytogenes_1639_v134	0.9326
Listeria_monocytogenes_1639_v17	Listeria_monocytogenes_1639_v134	0.9408
Listeria_monocytogenes_1639_v179	Listeria_monocytogenes_1639_v134	0.9284
Listeria_monocytogenes_1639_v178	Listeria_monocytogenes_1639_v134	0.9447
Listeria_monocytogenes_1639_v177	Listeria_monocytogenes_1639_v134	0.9689
Listeria_monocytogenes_1639_v176	Listeria_monocytogenes_1639_v134	0.9387
Listeria_monocytogenes_1639_v175	Listeria_monocytogenes_1639_v134	0.9356
Listeria_monocytogenes_1639_v174	Listeria_monocytogenes_1639_v134	0.9455
Listeria_monocytogenes_1639_v173	Listeria_monocytogenes_1639_v134	0.9672
Listeria_monocytogenes_1639_v172	Listeria_monocytogenes_1639_v134	0.9405
Listeria_monocytogenes_1639_v171	Listeria_monocytogenes_1639_v134	0.9277
Listeria_monocytogenes_1639_v170	Listeria_monocytogenes_1639_v134	0.9355
Listeria_monocytogenes_1639_v16	Listeria_monocytogenes_1639_v134	0.9044
Listeria_monocytogenes_1639_v169	Listeria_monocytogenes_1639_v134	0.9375
Listeria_monocytogenes_1639_v168	Listeria_monocytogenes_1639_v134	0.9387

Listeria_monocytogenes_1639_v167	Listeria_monocytogenes_1639_v134	0.9308
Listeria_monocytogenes_1639_v166	Listeria_monocytogenes_1639_v134	0.939
Listeria_monocytogenes_1639_v165	Listeria_monocytogenes_1639_v134	0.9371
Listeria_monocytogenes_1639_v164	Listeria_monocytogenes_1639_v134	0.9919
Listeria_monocytogenes_1639_v163	Listeria_monocytogenes_1639_v134	0.9585
Listeria_monocytogenes_1639_v162	Listeria_monocytogenes_1639_v134	0.9919
Listeria_monocytogenes_1639_v161	Listeria_monocytogenes_1639_v134	0.9923
Listeria_monocytogenes_1639_v160	Listeria_monocytogenes_1639_v134	0.9919
Listeria_monocytogenes_1639_v15	Listeria_monocytogenes_1639_v134	0.9243
Listeria_monocytogenes_1639_v159	Listeria_monocytogenes_1639_v134	0.9693
Listeria_monocytogenes_1639_v158	Listeria_monocytogenes_1639_v134	0.9927
Listeria_monocytogenes_1639_v157	Listeria_monocytogenes_1639_v134	0.9927
Listeria_monocytogenes_1639_v156	Listeria_monocytogenes_1639_v134	0.9192
Listeria_monocytogenes_1639_v155	Listeria_monocytogenes_1639_v134	0.9319
Listeria_monocytogenes_1639_v154	Listeria_monocytogenes_1639_v134	0.9358
Listeria_monocytogenes_1639_v153	Listeria_monocytogenes_1639_v134	0.9326
Listeria_monocytogenes_1639_v152	Listeria_monocytogenes_1639_v134	0.9697
Listeria_monocytogenes_1639_v151	Listeria_monocytogenes_1639_v134	0.9697
Listeria_monocytogenes_1639_v150	Listeria_monocytogenes_1639_v134	0.9319
Listeria_monocytogenes_1639_v14	Listeria_monocytogenes_1639_v134	0.9311
Listeria_monocytogenes_1639_v149	Listeria_monocytogenes_1639_v134	0.9309
Listeria_monocytogenes_1639_v148	Listeria_monocytogenes_1639_v134	0.9682
Listeria_monocytogenes_1639_v147	Listeria_monocytogenes_1639_v134	0.9547
Listeria_monocytogenes_1639_v146	Listeria_monocytogenes_1639_v134	0.9468
Listeria_monocytogenes_1639_v145	Listeria_monocytogenes_1639_v134	0.9431
Listeria_monocytogenes_1639_v144	Listeria_monocytogenes_1639_v134	0.9723
Listeria_monocytogenes_1639_v143	Listeria_monocytogenes_1639_v134	0.9484
Listeria_monocytogenes_1639_v142	Listeria_monocytogenes_1639_v134	0.9727
Listeria_monocytogenes_1639_v141	Listeria_monocytogenes_1639_v134	0.9649
Listeria_monocytogenes_1639_v140	Listeria_monocytogenes_1639_v134	0.9731
Listeria_monocytogenes_1639_v13	Listeria_monocytogenes_1639_v134	0.9242
Listeria_monocytogenes_1639_v139	Listeria_monocytogenes_1639_v134	0.9689
Listeria_monocytogenes_1639_v138	Listeria_monocytogenes_1639_v134	0.9754
Listeria_monocytogenes_1639_v137	Listeria_monocytogenes_1639_v134	0.9754
Listeria_monocytogenes_1639_v136	Listeria_monocytogenes_1639_v134	0.9349
Listeria_monocytogenes_1639_v135	Listeria_monocytogenes_1639_v134	0.9188
Listeria_monocytogenes_1639_v134	Listeria_monocytogenes_1639_v134	0.067
Listeria_welshimeri_1643	Listeria_monocytogenes_1639_v135	0.8738
Listeria_welshimeri_serovar_6b_str_SLCC5334_386043	Listeria_monocytogenes_1639_v135	0.8735
Listeria_seeligeri_serovar_1_2b_str_SLCC3954_683837	Listeria_monocytogenes_1639_v135	0.8682
Listeria_seeligeri_FSL_S4-171_702452	Listeria_monocytogenes_1639_v135	0.8167
Listeria_seeligeri_FSL_N1-067_702453	Listeria_monocytogenes_1639_v135	0.8137
Listeria_seeligeri_1640_v1	Listeria_monocytogenes_1639_v135	0.8641
Listeria_seeligeri_1640	Listeria_monocytogenes_1639_v135	0.8746
Listeria_monocytogenes_WSLC1042_1457188	Listeria_monocytogenes_1639_v135	0.9184
Listeria_monocytogenes_WSLC1001_1457187	Listeria_monocytogenes_1639_v135	0.9082
Listeria_monocytogenes_SLCC7179_879090	Listeria_monocytogenes_1639_v135	0.9206
Listeria_monocytogenes_SLCC5850_882096	Listeria_monocytogenes_1639_v135	0.9119
Listeria_monocytogenes_SLCC2755_932919	Listeria_monocytogenes_1639_v135	0.9036
Listeria_monocytogenes_SLCC2540_879089	Listeria_monocytogenes_1639_v135	0.9142
Listeria_monocytogenes_SLCC2479_882020	Listeria_monocytogenes_1639_v135	0.9088
Listeria_monocytogenes_SLCC2378_879088	Listeria_monocytogenes_1639_v135	0.9194
Listeria_monocytogenes_SLCC2376_882097	Listeria_monocytogenes_1639_v135	0.9418
Listeria_monocytogenes_SLCC2372_932920	Listeria_monocytogenes_1639_v135	0.9021
Listeria_monocytogenes_serotype_4b_str_LL195_1230340	Listeria_monocytogenes_1639_v135	0.9219
Listeria_monocytogenes_serotype_4b_str_LIS0087_1134669	Listeria_monocytogenes_1639_v135	0.8985
Listeria_monocytogenes_serotype_4b_str_F2365_265669	Listeria_monocytogenes_1639_v135	0.9219
Listeria_monocytogenes_serotype_4b_str_CLIP_80459_568819	Listeria_monocytogenes_1639_v135	0.9272
Listeria_monocytogenes_serotype_4b_str_81-0861_1196161	Listeria_monocytogenes_1639_v135	0.9218
Listeria_monocytogenes_serotype_4b_str_81-0592_1196160	Listeria_monocytogenes_1639_v135	0.9228
Listeria_monocytogenes_serotype_4b_str_81-0558_1196159	Listeria_monocytogenes_1639_v135	0.9232
Listeria_monocytogenes_serotype_4b_str_10-0809_1196162	Listeria_monocytogenes_1639_v135	0.9228
Listeria_monocytogenes_serotype_4b_str_02-6680_1196175	Listeria_monocytogenes_1639_v135	0.9216
Listeria_monocytogenes_serotype_4b_str_02-6679_1196174	Listeria_monocytogenes_1639_v135	0.9216
Listeria_monocytogenes_serotype_4b_str_02-1792_1196173	Listeria_monocytogenes_1639_v135	0.9236
Listeria_monocytogenes_serotype_4b_str_02-1289_1196172	Listeria_monocytogenes_1639_v135	0.9236
Listeria_monocytogenes_serotype_4b_str_02-1103_1196171	Listeria_monocytogenes_1639_v135	0.9236
Listeria_monocytogenes_serotype_3c_str_10-5027_1196195	Listeria_monocytogenes_1639_v135	0.9065
Listeria_monocytogenes_serotype_1_2c_str_10-5026_1196194	Listeria_monocytogenes_1639_v135	0.9069
Listeria_monocytogenes_serotype_1_2c_str_10-5025_1196193	Listeria_monocytogenes_1639_v135	0.9069
Listeria_monocytogenes_serotype_1_2b_str_10-0811_1196164	Listeria_monocytogenes_1639_v135	0.9144
Listeria_monocytogenes_serotype_1_2b_str_10-0810_1196163	Listeria_monocytogenes_1639_v135	0.9144
Listeria_monocytogenes_serotype_1_2a_str_99-6370_1196179	Listeria_monocytogenes_1639_v135	0.8937
Listeria_monocytogenes_serotype_1_2a_str_98-2035_1196178	Listeria_monocytogenes_1639_v135	0.8937
Listeria_monocytogenes_serotype_1_2a_str_95-0093_1196177	Listeria_monocytogenes_1639_v135	0.8975
Listeria_monocytogenes_serotype_1_2a_str_88-0478_1196176	Listeria_monocytogenes_1639_v135	0.8967
Listeria_monocytogenes_serotype_1_2a_str_10-5024_1196192	Listeria_monocytogenes_1639_v135	0.8975
Listeria_monocytogenes_serotype_1_2a_str_10-4758_1196168	Listeria_monocytogenes_1639_v135	0.9139
Listeria_monocytogenes_serotype_1_2a_str_10-4754_1196167	Listeria_monocytogenes_1639_v135	0.9143
Listeria_monocytogenes_serotype_1_2a_str_10-1321_1196191	Listeria_monocytogenes_1639_v135	0.8975
Listeria_monocytogenes_serotype_1_2a_str_10-1047_1196190	Listeria_monocytogenes_1639_v135	0.8986
Listeria_monocytogenes_serotype_1_2a_str_10-1046_1196189	Listeria_monocytogenes_1639_v135	0.8986
Listeria_monocytogenes_serotype_1_2a_str_10-0934_1196170	Listeria_monocytogenes_1639_v135	0.9204
Listeria_monocytogenes_serotype_1_2a_str_10-0933_1196169	Listeria_monocytogenes_1639_v135	0.9204
Listeria_monocytogenes_serotype_1_2a_str_10-0815_1196188	Listeria_monocytogenes_1639_v135	0.8939
Listeria_monocytogenes_serotype_1_2a_str_10-0814_1196187	Listeria_monocytogenes_1639_v135	0.8975
Listeria_monocytogenes_serotype_1_2a_str_10-0813_1196166	Listeria_monocytogenes_1639_v135	0.9144
Listeria_monocytogenes_serotype_1_2a_str_10-0812_1196165	Listeria_monocytogenes_1639_v135	0.9144
Listeria_monocytogenes_serotype_1_2a_str_08-7669_1196186	Listeria_monocytogenes_1639_v135	0.9066
Listeria_monocytogenes_serotype_1_2a_str_08-7374_1196185	Listeria_monocytogenes_1639_v135	0.8975
Listeria_monocytogenes_serotype_1_2a_str_08-6997_1196184	Listeria_monocytogenes_1639_v135	0.8939

Listeria_monocytogenes_serotype_1_2a_str_08-6569_1196183	Listeria_monocytogenes_1639_v135	0.8939
Listeria_monocytogenes_serotype_1_2a_str_08-6056_1196182	Listeria_monocytogenes_1639_v135	0.8939
Listeria_monocytogenes_serotype_1_2a_str_04-5457_1196181	Listeria_monocytogenes_1639_v135	0.8975
Listeria_monocytogenes_serotype_1_2a_str_02-5993_1196180	Listeria_monocytogenes_1639_v135	0.8975
Listeria_monocytogenes_R479a_1437838	Listeria_monocytogenes_1639_v135	0.8965
Listeria_monocytogenes_N53-1_1234142	Listeria_monocytogenes_1639_v135	0.8782
Listeria_monocytogenes_M7_1030009	Listeria_monocytogenes_1639_v135	0.9748
Listeria_monocytogenes_LO28_393132	Listeria_monocytogenes_1639_v135	0.913
Listeria_monocytogenes_L99_563174	Listeria_monocytogenes_1639_v135	0.9751
Listeria_monocytogenes_L312_882094	Listeria_monocytogenes_1639_v135	0.9272
Listeria_monocytogenes_J1816_930781	Listeria_monocytogenes_1639_v135	0.9109
Listeria_monocytogenes_J1-220_930782	Listeria_monocytogenes_1639_v135	0.9156
Listeria_monocytogenes_J0161_393130	Listeria_monocytogenes_1639_v135	0.9201
Listeria_monocytogenes_HCC23_552536	Listeria_monocytogenes_1639_v135	0.9744
Listeria_monocytogenes_FSL_R2-561_393126	Listeria_monocytogenes_1639_v135	0.9059
Listeria_monocytogenes_FSL_J1-208_393119	Listeria_monocytogenes_1639_v135	0.9088
Listeria_monocytogenes_FSL_J1-175_393118	Listeria_monocytogenes_1639_v135	0.9123
Listeria_monocytogenes_Finland_1998_393127	Listeria_monocytogenes_1639_v135	0.9235
Listeria_monocytogenes_EGD-e_169963_v1	Listeria_monocytogenes_1639_v135	0.9253
Listeria_monocytogenes_EGD-e_169963	Listeria_monocytogenes_1639_v135	0.9253
Listeria_monocytogenes_EGD_1334565	Listeria_monocytogenes_1639_v135	0.9126
Listeria_monocytogenes_CFSAN002349_1299895	Listeria_monocytogenes_1639_v135	0.9213
Listeria_monocytogenes_ATCC_19117_882095_v1	Listeria_monocytogenes_1639_v135	0.9211
Listeria_monocytogenes_ATCC_19117_882095	Listeria_monocytogenes_1639_v135	0.9207
Listeria_monocytogenes_6179_1288295	Listeria_monocytogenes_1639_v135	0.9076
Listeria_monocytogenes_1639_v9	Listeria_monocytogenes_1639_v135	0.8954
Listeria_monocytogenes_1639_v99	Listeria_monocytogenes_1639_v135	0.9139
Listeria_monocytogenes_1639_v98	Listeria_monocytogenes_1639_v135	0.9182
Listeria_monocytogenes_1639_v97	Listeria_monocytogenes_1639_v135	0.9169
Listeria_monocytogenes_1639_v96	Listeria_monocytogenes_1639_v135	0.9117
Listeria_monocytogenes_1639_v95	Listeria_monocytogenes_1639_v135	0.9117
Listeria_monocytogenes_1639_v94	Listeria_monocytogenes_1639_v135	0.9124
Listeria_monocytogenes_1639_v93	Listeria_monocytogenes_1639_v135	0.8993
Listeria_monocytogenes_1639_v92	Listeria_monocytogenes_1639_v135	0.9139
Listeria_monocytogenes_1639_v91	Listeria_monocytogenes_1639_v135	0.9167
Listeria_monocytogenes_1639_v90	Listeria_monocytogenes_1639_v135	0.9182
Listeria_monocytogenes_1639_v8	Listeria_monocytogenes_1639_v135	0.8896
Listeria_monocytogenes_1639_v89	Listeria_monocytogenes_1639_v135	0.9241
Listeria_monocytogenes_1639_v88	Listeria_monocytogenes_1639_v135	0.9082
Listeria_monocytogenes_1639_v87	Listeria_monocytogenes_1639_v135	0.9112
Listeria_monocytogenes_1639_v86	Listeria_monocytogenes_1639_v135	0.9112
Listeria_monocytogenes_1639_v85	Listeria_monocytogenes_1639_v135	0.923
Listeria_monocytogenes_1639_v84	Listeria_monocytogenes_1639_v135	0.9136
Listeria_monocytogenes_1639_v83	Listeria_monocytogenes_1639_v135	0.9227
Listeria_monocytogenes_1639_v82	Listeria_monocytogenes_1639_v135	0.922
Listeria_monocytogenes_1639_v81	Listeria_monocytogenes_1639_v135	0.9108
Listeria_monocytogenes_1639_v80	Listeria_monocytogenes_1639_v135	0.9166
Listeria_monocytogenes_1639_v7	Listeria_monocytogenes_1639_v135	0.8896
Listeria_monocytogenes_1639_v79	Listeria_monocytogenes_1639_v135	0.9091
Listeria_monocytogenes_1639_v78	Listeria_monocytogenes_1639_v135	0.9094
Listeria_monocytogenes_1639_v77	Listeria_monocytogenes_1639_v135	0.9082
Listeria_monocytogenes_1639_v76	Listeria_monocytogenes_1639_v135	0.9033
Listeria_monocytogenes_1639_v75	Listeria_monocytogenes_1639_v135	0.9751
Listeria_monocytogenes_1639_v74	Listeria_monocytogenes_1639_v135	0.9085
Listeria_monocytogenes_1639_v73	Listeria_monocytogenes_1639_v135	0.9079
Listeria_monocytogenes_1639_v72	Listeria_monocytogenes_1639_v135	0.9208
Listeria_monocytogenes_1639_v71	Listeria_monocytogenes_1639_v135	0.8959
Listeria_monocytogenes_1639_v70	Listeria_monocytogenes_1639_v135	0.9043
Listeria_monocytogenes_1639_v6	Listeria_monocytogenes_1639_v135	0.8893
Listeria_monocytogenes_1639_v69	Listeria_monocytogenes_1639_v135	0.9228
Listeria_monocytogenes_1639_v68	Listeria_monocytogenes_1639_v135	0.9243
Listeria_monocytogenes_1639_v67	Listeria_monocytogenes_1639_v135	0.9078
Listeria_monocytogenes_1639_v66	Listeria_monocytogenes_1639_v135	0.9099
Listeria_monocytogenes_1639_v65	Listeria_monocytogenes_1639_v135	0.908
Listeria_monocytogenes_1639_v64	Listeria_monocytogenes_1639_v135	0.9143
Listeria_monocytogenes_1639_v63	Listeria_monocytogenes_1639_v135	0.9053
Listeria_monocytogenes_1639_v62	Listeria_monocytogenes_1639_v135	0.9067
Listeria_monocytogenes_1639_v61	Listeria_monocytogenes_1639_v135	0.9013
Listeria_monocytogenes_1639_v60	Listeria_monocytogenes_1639_v135	0.9153
Listeria_monocytogenes_1639_v5	Listeria_monocytogenes_1639_v135	0.8814
Listeria_monocytogenes_1639_v59	Listeria_monocytogenes_1639_v135	0.9126
Listeria_monocytogenes_1639_v58	Listeria_monocytogenes_1639_v135	0.9235
Listeria_monocytogenes_1639_v57	Listeria_monocytogenes_1639_v135	0.9091
Listeria_monocytogenes_1639_v56	Listeria_monocytogenes_1639_v135	0.9167
Listeria_monocytogenes_1639_v55	Listeria_monocytogenes_1639_v135	0.9139
Listeria_monocytogenes_1639_v54	Listeria_monocytogenes_1639_v135	0.904
Listeria_monocytogenes_1639_v53	Listeria_monocytogenes_1639_v135	0.9149
Listeria_monocytogenes_1639_v52	Listeria_monocytogenes_1639_v135	0.9185
Listeria_monocytogenes_1639_v51	Listeria_monocytogenes_1639_v135	0.9198
Listeria_monocytogenes_1639_v50	Listeria_monocytogenes_1639_v135	0.8964
Listeria_monocytogenes_1639_v4	Listeria_monocytogenes_1639_v135	0.8931
Listeria_monocytogenes_1639_v49	Listeria_monocytogenes_1639_v135	0.9121
Listeria_monocytogenes_1639_v48	Listeria_monocytogenes_1639_v135	0.903
Listeria_monocytogenes_1639_v47	Listeria_monocytogenes_1639_v135	0.8976
Listeria_monocytogenes_1639_v46	Listeria_monocytogenes_1639_v135	0.9161
Listeria_monocytogenes_1639_v45	Listeria_monocytogenes_1639_v135	0.8894
Listeria_monocytogenes_1639_v44	Listeria_monocytogenes_1639_v135	0.8975
Listeria_monocytogenes_1639_v43	Listeria_monocytogenes_1639_v135	0.8975
Listeria_monocytogenes_1639_v42	Listeria_monocytogenes_1639_v135	0.9073
Listeria_monocytogenes_1639_v41	Listeria_monocytogenes_1639_v135	0.8909
Listeria_monocytogenes_1639_v40	Listeria_monocytogenes_1639_v135	0.9066

Listeria_monocytogenes_1639_v3	Listeria_monocytogenes_1639_v135	0.8934
Listeria_monocytogenes_1639_v39	Listeria_monocytogenes_1639_v135	0.9034
Listeria_monocytogenes_1639_v38	Listeria_monocytogenes_1639_v135	0.9039
Listeria_monocytogenes_1639_v37	Listeria_monocytogenes_1639_v135	0.8967
Listeria_monocytogenes_1639_v36	Listeria_monocytogenes_1639_v135	0.8998
Listeria_monocytogenes_1639_v35	Listeria_monocytogenes_1639_v135	0.9077
Listeria_monocytogenes_1639_v34	Listeria_monocytogenes_1639_v135	0.9097
Listeria_monocytogenes_1639_v33	Listeria_monocytogenes_1639_v135	0.9144
Listeria_monocytogenes_1639_v32	Listeria_monocytogenes_1639_v135	0.9046
Listeria_monocytogenes_1639_v31	Listeria_monocytogenes_1639_v135	0.91
Listeria_monocytogenes_1639_v30	Listeria_monocytogenes_1639_v135	0.9075
Listeria_monocytogenes_1639_v2	Listeria_monocytogenes_1639_v135	0.8796
Listeria_monocytogenes_1639_v29	Listeria_monocytogenes_1639_v135	0.8947
Listeria_monocytogenes_1639_v28	Listeria_monocytogenes_1639_v135	0.899
Listeria_monocytogenes_1639_v27	Listeria_monocytogenes_1639_v135	0.8964
Listeria_monocytogenes_1639_v26	Listeria_monocytogenes_1639_v135	0.8957
Listeria_monocytogenes_1639_v25	Listeria_monocytogenes_1639_v135	0.8957
Listeria_monocytogenes_1639_v24	Listeria_monocytogenes_1639_v135	0.9179
Listeria_monocytogenes_1639_v23	Listeria_monocytogenes_1639_v135	0.9172
Listeria_monocytogenes_1639_v22	Listeria_monocytogenes_1639_v135	0.9143
Listeria_monocytogenes_1639_v21	Listeria_monocytogenes_1639_v135	0.9153
Listeria_monocytogenes_1639_v20	Listeria_monocytogenes_1639_v135	0.8975
Listeria_monocytogenes_1639_v1	Listeria_monocytogenes_1639_v135	0.8996
Listeria_monocytogenes_1639_v19	Listeria_monocytogenes_1639_v135	0.8972
Listeria_monocytogenes_1639_v198	Listeria_monocytogenes_1639_v135	0.8794
Listeria_monocytogenes_1639_v197	Listeria_monocytogenes_1639_v135	0.9124
Listeria_monocytogenes_1639_v196	Listeria_monocytogenes_1639_v135	0.8962
Listeria_monocytogenes_1639_v195	Listeria_monocytogenes_1639_v135	0.9391
Listeria_monocytogenes_1639_v194	Listeria_monocytogenes_1639_v135	0.9422
Listeria_monocytogenes_1639_v193	Listeria_monocytogenes_1639_v135	0.9422
Listeria_monocytogenes_1639_v192	Listeria_monocytogenes_1639_v135	0.9034
Listeria_monocytogenes_1639_v191	Listeria_monocytogenes_1639_v135	0.9264
Listeria_monocytogenes_1639_v190	Listeria_monocytogenes_1639_v135	0.9211
Listeria_monocytogenes_1639_v18	Listeria_monocytogenes_1639_v135	0.9108
Listeria_monocytogenes_1639_v189	Listeria_monocytogenes_1639_v135	0.9207
Listeria_monocytogenes_1639_v188	Listeria_monocytogenes_1639_v135	0.9255
Listeria_monocytogenes_1639_v187	Listeria_monocytogenes_1639_v135	0.9214
Listeria_monocytogenes_1639_v186	Listeria_monocytogenes_1639_v135	0.9214
Listeria_monocytogenes_1639_v185	Listeria_monocytogenes_1639_v135	0.9214
Listeria_monocytogenes_1639_v184	Listeria_monocytogenes_1639_v135	0.921
Listeria_monocytogenes_1639_v183	Listeria_monocytogenes_1639_v135	0.9237
Listeria_monocytogenes_1639_v182	Listeria_monocytogenes_1639_v135	0.9259
Listeria_monocytogenes_1639_v181	Listeria_monocytogenes_1639_v135	0.9462
Listeria_monocytogenes_1639_v180	Listeria_monocytogenes_1639_v135	0.9462
Listeria_monocytogenes_1639_v17	Listeria_monocytogenes_1639_v135	0.9111
Listeria_monocytogenes_1639_v179	Listeria_monocytogenes_1639_v135	0.9173
Listeria_monocytogenes_1639_v178	Listeria_monocytogenes_1639_v135	0.9241
Listeria_monocytogenes_1639_v177	Listeria_monocytogenes_1639_v135	0.9228
Listeria_monocytogenes_1639_v176	Listeria_monocytogenes_1639_v135	0.9408
Listeria_monocytogenes_1639_v175	Listeria_monocytogenes_1639_v135	0.9115
Listeria_monocytogenes_1639_v174	Listeria_monocytogenes_1639_v135	0.9262
Listeria_monocytogenes_1639_v173	Listeria_monocytogenes_1639_v135	0.9197
Listeria_monocytogenes_1639_v172	Listeria_monocytogenes_1639_v135	0.9357
Listeria_monocytogenes_1639_v171	Listeria_monocytogenes_1639_v135	0.9083
Listeria_monocytogenes_1639_v170	Listeria_monocytogenes_1639_v135	0.9155
Listeria_monocytogenes_1639_v16	Listeria_monocytogenes_1639_v135	0.8855
Listeria_monocytogenes_1639_v169	Listeria_monocytogenes_1639_v135	0.9179
Listeria_monocytogenes_1639_v168	Listeria_monocytogenes_1639_v135	0.9224
Listeria_monocytogenes_1639_v167	Listeria_monocytogenes_1639_v135	0.9178
Listeria_monocytogenes_1639_v166	Listeria_monocytogenes_1639_v135	0.9228
Listeria_monocytogenes_1639_v165	Listeria_monocytogenes_1639_v135	0.9176
Listeria_monocytogenes_1639_v164	Listeria_monocytogenes_1639_v135	0.923
Listeria_monocytogenes_1639_v163	Listeria_monocytogenes_1639_v135	0.9204
Listeria_monocytogenes_1639_v162	Listeria_monocytogenes_1639_v135	0.923
Listeria_monocytogenes_1639_v161	Listeria_monocytogenes_1639_v135	0.9226
Listeria_monocytogenes_1639_v160	Listeria_monocytogenes_1639_v135	0.9223
Listeria_monocytogenes_1639_v15	Listeria_monocytogenes_1639_v135	0.9004
Listeria_monocytogenes_1639_v159	Listeria_monocytogenes_1639_v135	0.9232
Listeria_monocytogenes_1639_v158	Listeria_monocytogenes_1639_v135	0.9219
Listeria_monocytogenes_1639_v157	Listeria_monocytogenes_1639_v135	0.9219
Listeria_monocytogenes_1639_v156	Listeria_monocytogenes_1639_v135	0.9113
Listeria_monocytogenes_1639_v155	Listeria_monocytogenes_1639_v135	0.9204
Listeria_monocytogenes_1639_v154	Listeria_monocytogenes_1639_v135	0.9106
Listeria_monocytogenes_1639_v153	Listeria_monocytogenes_1639_v135	0.9279
Listeria_monocytogenes_1639_v152	Listeria_monocytogenes_1639_v135	0.9232
Listeria_monocytogenes_1639_v151	Listeria_monocytogenes_1639_v135	0.9232
Listeria_monocytogenes_1639_v150	Listeria_monocytogenes_1639_v135	0.9135
Listeria_monocytogenes_1639_v14	Listeria_monocytogenes_1639_v135	0.9052
Listeria_monocytogenes_1639_v149	Listeria_monocytogenes_1639_v135	0.913
Listeria_monocytogenes_1639_v148	Listeria_monocytogenes_1639_v135	0.9221
Listeria_monocytogenes_1639_v147	Listeria_monocytogenes_1639_v135	0.9101
Listeria_monocytogenes_1639_v146	Listeria_monocytogenes_1639_v135	0.9213
Listeria_monocytogenes_1639_v145	Listeria_monocytogenes_1639_v135	0.9276
Listeria_monocytogenes_1639_v144	Listeria_monocytogenes_1639_v135	0.9264
Listeria_monocytogenes_1639_v143	Listeria_monocytogenes_1639_v135	0.9241
Listeria_monocytogenes_1639_v142	Listeria_monocytogenes_1639_v135	0.9268
Listeria_monocytogenes_1639_v141	Listeria_monocytogenes_1639_v135	0.9202
Listeria_monocytogenes_1639_v140	Listeria_monocytogenes_1639_v135	0.9275
Listeria_monocytogenes_1639_v13	Listeria_monocytogenes_1639_v135	0.9004
Listeria_monocytogenes_1639_v139	Listeria_monocytogenes_1639_v135	0.9174
Listeria_monocytogenes_1639_v138	Listeria_monocytogenes_1639_v135	0.9253

Listeria_monocytogenes_1639_v137	Listeria_monocytogenes_1639_v135	0.9249
Listeria_monocytogenes_1639_v136	Listeria_monocytogenes_1639_v135	0.9136
Listeria_monocytogenes_1639_v135	Listeria_monocytogenes_1639_v135	0.0617
Listeria_welshimeri_1643	Listeria_monocytogenes_1639_v136	0.876
Listeria_welshimeri_serovar_6b_str_SLCC5334_386043	Listeria_monocytogenes_1639_v136	0.8757
Listeria_seeligeri_serovar_1_2b_str_SLCC3954_683837	Listeria_monocytogenes_1639_v136	0.8796
Listeria_seeligeri_FSL_S4-171_702452	Listeria_monocytogenes_1639_v136	0.8128
Listeria_seeligeri_FSL_N1-067_702453	Listeria_monocytogenes_1639_v136	0.8286
Listeria_seeligeri_1640_v1	Listeria_monocytogenes_1639_v136	0.8819
Listeria_seeligeri_1640	Listeria_monocytogenes_1639_v136	0.873
Listeria_monocytogenes_WSLC1042_1457188	Listeria_monocytogenes_1639_v136	0.934
Listeria_monocytogenes_WSLC1001_1457187	Listeria_monocytogenes_1639_v136	0.9637
Listeria_monocytogenes_SLCC7179_879090	Listeria_monocytogenes_1639_v136	0.9569
Listeria_monocytogenes_SLCC5850_882096	Listeria_monocytogenes_1639_v136	0.9647
Listeria_monocytogenes_SLCC2755_932919	Listeria_monocytogenes_1639_v136	0.9289
Listeria_monocytogenes_SLCC2540_879089	Listeria_monocytogenes_1639_v136	0.9535
Listeria_monocytogenes_SLCC2479_882020	Listeria_monocytogenes_1639_v136	0.9591
Listeria_monocytogenes_SLCC2378_879088	Listeria_monocytogenes_1639_v136	0.9351
Listeria_monocytogenes_SLCC2376_882097	Listeria_monocytogenes_1639_v136	0.9362
Listeria_monocytogenes_SLCC2372_932920	Listeria_monocytogenes_1639_v136	0.952
Listeria_monocytogenes_serotype_4b_str_LL195_1230340	Listeria_monocytogenes_1639_v136	0.94
Listeria_monocytogenes_serotype_4b_str_LIS0087_1134669	Listeria_monocytogenes_1639_v136	0.9429
Listeria_monocytogenes_serotype_4b_str_F2365_265669	Listeria_monocytogenes_1639_v136	0.94
Listeria_monocytogenes_serotype_4b_str_CLIP_80459_568819	Listeria_monocytogenes_1639_v136	0.9434
Listeria_monocytogenes_serotype_4b_str_81-0861_1196161	Listeria_monocytogenes_1639_v136	0.9256
Listeria_monocytogenes_serotype_4b_str_81-0592_1196160	Listeria_monocytogenes_1639_v136	0.9267
Listeria_monocytogenes_serotype_4b_str_81-0558_1196159	Listeria_monocytogenes_1639_v136	0.9263
Listeria_monocytogenes_serotype_4b_str_10-0809_1196162	Listeria_monocytogenes_1639_v136	0.9267
Listeria_monocytogenes_serotype_4b_str_02-6680_1196175	Listeria_monocytogenes_1639_v136	0.9419
Listeria_monocytogenes_serotype_4b_str_02-6679_1196174	Listeria_monocytogenes_1639_v136	0.9419
Listeria_monocytogenes_serotype_4b_str_02-1792_1196173	Listeria_monocytogenes_1639_v136	0.9286
Listeria_monocytogenes_serotype_4b_str_02-1289_1196172	Listeria_monocytogenes_1639_v136	0.9286
Listeria_monocytogenes_serotype_4b_str_02-1103_1196171	Listeria_monocytogenes_1639_v136	0.9286
Listeria_monocytogenes_serotype_3c_str_10-5027_1196195	Listeria_monocytogenes_1639_v136	0.9591
Listeria_monocytogenes_serotype_1_2c_str_10-5026_1196194	Listeria_monocytogenes_1639_v136	0.9595
Listeria_monocytogenes_serotype_1_2c_str_10-5025_1196193	Listeria_monocytogenes_1639_v136	0.9595
Listeria_monocytogenes_serotype_1_2b_str_10-0811_1196164	Listeria_monocytogenes_1639_v136	0.9421
Listeria_monocytogenes_serotype_1_2b_str_10-0810_1196163	Listeria_monocytogenes_1639_v136	0.9421
Listeria_monocytogenes_serotype_1_2a_str_99-6370_1196179	Listeria_monocytogenes_1639_v136	0.9459
Listeria_monocytogenes_serotype_1_2a_str_98-2035_1196178	Listeria_monocytogenes_1639_v136	0.9459
Listeria_monocytogenes_serotype_1_2a_str_95-0093_1196177	Listeria_monocytogenes_1639_v136	0.9512
Listeria_monocytogenes_serotype_1_2a_str_88-0478_1196176	Listeria_monocytogenes_1639_v136	0.9497
Listeria_monocytogenes_serotype_1_2a_str_10-5024_1196192	Listeria_monocytogenes_1639_v136	0.9515
Listeria_monocytogenes_serotype_1_2a_str_10-4758_1196168	Listeria_monocytogenes_1639_v136	0.9932
Listeria_monocytogenes_serotype_1_2a_str_10-4754_1196167	Listeria_monocytogenes_1639_v136	0.9932
Listeria_monocytogenes_serotype_1_2a_str_10-1321_1196191	Listeria_monocytogenes_1639_v136	0.9515
Listeria_monocytogenes_serotype_1_2a_str_10-1047_1196190	Listeria_monocytogenes_1639_v136	0.9515
Listeria_monocytogenes_serotype_1_2a_str_10-1046_1196189	Listeria_monocytogenes_1639_v136	0.9515
Listeria_monocytogenes_serotype_1_2a_str_10-0934_1196170	Listeria_monocytogenes_1639_v136	0.9662
Listeria_monocytogenes_serotype_1_2a_str_10-0933_1196169	Listeria_monocytogenes_1639_v136	0.9662
Listeria_monocytogenes_serotype_1_2a_str_10-0815_1196188	Listeria_monocytogenes_1639_v136	0.947
Listeria_monocytogenes_serotype_1_2a_str_10-0814_1196187	Listeria_monocytogenes_1639_v136	0.9515
Listeria_monocytogenes_serotype_1_2a_str_10-0813_1196166	Listeria_monocytogenes_1639_v136	0.9593
Listeria_monocytogenes_serotype_1_2a_str_10-0812_1196165	Listeria_monocytogenes_1639_v136	0.9593
Listeria_monocytogenes_serotype_1_2a_str_08-7669_1196186	Listeria_monocytogenes_1639_v136	0.9469
Listeria_monocytogenes_serotype_1_2a_str_08-7374_1196185	Listeria_monocytogenes_1639_v136	0.9515
Listeria_monocytogenes_serotype_1_2a_str_08-6997_1196184	Listeria_monocytogenes_1639_v136	0.947
Listeria_monocytogenes_serotype_1_2a_str_08-6569_1196183	Listeria_monocytogenes_1639_v136	0.947
Listeria_monocytogenes_serotype_1_2a_str_08-6056_1196182	Listeria_monocytogenes_1639_v136	0.947
Listeria_monocytogenes_serotype_1_2a_str_04-5457_1196181	Listeria_monocytogenes_1639_v136	0.9515
Listeria_monocytogenes_serotype_1_2a_str_02-5993_1196180	Listeria_monocytogenes_1639_v136	0.9515
Listeria_monocytogenes_R479a_1437838	Listeria_monocytogenes_1639_v136	0.9486
Listeria_monocytogenes_N53-1_1234142	Listeria_monocytogenes_1639_v136	0.9074
Listeria_monocytogenes_M7_1030009	Listeria_monocytogenes_1639_v136	0.9129
Listeria_monocytogenes_LO28_393132	Listeria_monocytogenes_1639_v136	0.9577
Listeria_monocytogenes_L99_563174	Listeria_monocytogenes_1639_v136	0.9133
Listeria_monocytogenes_L312_882094	Listeria_monocytogenes_1639_v136	0.9434
Listeria_monocytogenes_J1816_930781	Listeria_monocytogenes_1639_v136	0.9247
Listeria_monocytogenes_J1-220_930782	Listeria_monocytogenes_1639_v136	0.9295
Listeria_monocytogenes_J0161_393130	Listeria_monocytogenes_1639_v136	0.9453
Listeria_monocytogenes_HCC23_552536	Listeria_monocytogenes_1639_v136	0.9129
Listeria_monocytogenes_FSL_R2-561_393126	Listeria_monocytogenes_1639_v136	0.958
Listeria_monocytogenes_FSL_J1-208_393119	Listeria_monocytogenes_1639_v136	0.9047
Listeria_monocytogenes_FSL_J1-175_393118	Listeria_monocytogenes_1639_v136	0.9518
Listeria_monocytogenes_Finland_1998_393127	Listeria_monocytogenes_1639_v136	0.9627
Listeria_monocytogenes_EGD-e_169963_v1	Listeria_monocytogenes_1639_v136	0.9553
Listeria_monocytogenes_EGD-e_169963	Listeria_monocytogenes_1639_v136	0.9553
Listeria_monocytogenes_EGD_1334565	Listeria_monocytogenes_1639_v136	0.9655
Listeria_monocytogenes_CFSAN002349_1299895	Listeria_monocytogenes_1639_v136	0.942
Listeria_monocytogenes_ATCC_19117_882095_v1	Listeria_monocytogenes_1639_v136	0.939
Listeria_monocytogenes_ATCC_19117_882095	Listeria_monocytogenes_1639_v136	0.9387
Listeria_monocytogenes_6179_1288295	Listeria_monocytogenes_1639_v136	0.9442
Listeria_monocytogenes_1639_v9	Listeria_monocytogenes_1639_v136	0.9252
Listeria_monocytogenes_1639_v99	Listeria_monocytogenes_1639_v136	0.9248
Listeria_monocytogenes_1639_v98	Listeria_monocytogenes_1639_v136	0.9437
Listeria_monocytogenes_1639_v97	Listeria_monocytogenes_1639_v136	0.9343
Listeria_monocytogenes_1639_v96	Listeria_monocytogenes_1639_v136	0.9245
Listeria_monocytogenes_1639_v95	Listeria_monocytogenes_1639_v136	0.9245
Listeria_monocytogenes_1639_v94	Listeria_monocytogenes_1639_v136	0.9248
Listeria_monocytogenes_1639_v93	Listeria_monocytogenes_1639_v136	0.9549
Listeria_monocytogenes_1639_v92	Listeria_monocytogenes_1639_v136	0.9259

Listeria_monocytogenes_1639_v91	Listeria_monocytogenes_1639_v136	0.9318
Listeria_monocytogenes_1639_v90	Listeria_monocytogenes_1639_v136	0.9437
Listeria_monocytogenes_1639_v8	Listeria_monocytogenes_1639_v136	0.9378
Listeria_monocytogenes_1639_v89	Listeria_monocytogenes_1639_v136	0.9375
Listeria_monocytogenes_1639_v88	Listeria_monocytogenes_1639_v136	0.9594
Listeria_monocytogenes_1639_v87	Listeria_monocytogenes_1639_v136	0.9547
Listeria_monocytogenes_1639_v86	Listeria_monocytogenes_1639_v136	0.9547
Listeria_monocytogenes_1639_v85	Listeria_monocytogenes_1639_v136	0.9261
Listeria_monocytogenes_1639_v84	Listeria_monocytogenes_1639_v136	0.9642
Listeria_monocytogenes_1639_v83	Listeria_monocytogenes_1639_v136	0.9303
Listeria_monocytogenes_1639_v82	Listeria_monocytogenes_1639_v136	0.9296
Listeria_monocytogenes_1639_v81	Listeria_monocytogenes_1639_v136	0.9594
Listeria_monocytogenes_1639_v80	Listeria_monocytogenes_1639_v136	0.9483
Listeria_monocytogenes_1639_v7	Listeria_monocytogenes_1639_v136	0.9378
Listeria_monocytogenes_1639_v79	Listeria_monocytogenes_1639_v136	0.9557
Listeria_monocytogenes_1639_v78	Listeria_monocytogenes_1639_v136	0.959
Listeria_monocytogenes_1639_v77	Listeria_monocytogenes_1639_v136	0.9479
Listeria_monocytogenes_1639_v76	Listeria_monocytogenes_1639_v136	0.978
Listeria_monocytogenes_1639_v75	Listeria_monocytogenes_1639_v136	0.9133
Listeria_monocytogenes_1639_v74	Listeria_monocytogenes_1639_v136	0.9397
Listeria_monocytogenes_1639_v73	Listeria_monocytogenes_1639_v136	0.953
Listeria_monocytogenes_1639_v72	Listeria_monocytogenes_1639_v136	0.9288
Listeria_monocytogenes_1639_v71	Listeria_monocytogenes_1639_v136	0.9409
Listeria_monocytogenes_1639_v70	Listeria_monocytogenes_1639_v136	0.9563
Listeria_monocytogenes_1639_v6	Listeria_monocytogenes_1639_v136	0.9374
Listeria_monocytogenes_1639_v69	Listeria_monocytogenes_1639_v136	0.9267
Listeria_monocytogenes_1639_v68	Listeria_monocytogenes_1639_v136	0.9481
Listeria_monocytogenes_1639_v67	Listeria_monocytogenes_1639_v136	0.9409
Listeria_monocytogenes_1639_v66	Listeria_monocytogenes_1639_v136	0.9663
Listeria_monocytogenes_1639_v65	Listeria_monocytogenes_1639_v136	0.9529
Listeria_monocytogenes_1639_v64	Listeria_monocytogenes_1639_v136	0.9576
Listeria_monocytogenes_1639_v63	Listeria_monocytogenes_1639_v136	0.9431
Listeria_monocytogenes_1639_v62	Listeria_monocytogenes_1639_v136	0.9536
Listeria_monocytogenes_1639_v61	Listeria_monocytogenes_1639_v136	0.9533
Listeria_monocytogenes_1639_v60	Listeria_monocytogenes_1639_v136	0.9469
Listeria_monocytogenes_1639_v5	Listeria_monocytogenes_1639_v136	0.8964
Listeria_monocytogenes_1639_v59	Listeria_monocytogenes_1639_v136	0.9379
Listeria_monocytogenes_1639_v58	Listeria_monocytogenes_1639_v136	0.9292
Listeria_monocytogenes_1639_v57	Listeria_monocytogenes_1639_v136	0.9222
Listeria_monocytogenes_1639_v56	Listeria_monocytogenes_1639_v136	0.9307
Listeria_monocytogenes_1639_v55	Listeria_monocytogenes_1639_v136	0.9191
Listeria_monocytogenes_1639_v54	Listeria_monocytogenes_1639_v136	0.9185
Listeria_monocytogenes_1639_v53	Listeria_monocytogenes_1639_v136	0.9435
Listeria_monocytogenes_1639_v52	Listeria_monocytogenes_1639_v136	0.9354
Listeria_monocytogenes_1639_v51	Listeria_monocytogenes_1639_v136	0.9251
Listeria_monocytogenes_1639_v50	Listeria_monocytogenes_1639_v136	0.9501
Listeria_monocytogenes_1639_v4	Listeria_monocytogenes_1639_v136	0.9095
Listeria_monocytogenes_1639_v49	Listeria_monocytogenes_1639_v136	0.9286
Listeria_monocytogenes_1639_v48	Listeria_monocytogenes_1639_v136	0.9541
Listeria_monocytogenes_1639_v47	Listeria_monocytogenes_1639_v136	0.9512
Listeria_monocytogenes_1639_v46	Listeria_monocytogenes_1639_v136	0.9477
Listeria_monocytogenes_1639_v45	Listeria_monocytogenes_1639_v136	0.9417
Listeria_monocytogenes_1639_v44	Listeria_monocytogenes_1639_v136	0.9515
Listeria_monocytogenes_1639_v43	Listeria_monocytogenes_1639_v136	0.9515
Listeria_monocytogenes_1639_v42	Listeria_monocytogenes_1639_v136	0.934
Listeria_monocytogenes_1639_v41	Listeria_monocytogenes_1639_v136	0.93
Listeria_monocytogenes_1639_v40	Listeria_monocytogenes_1639_v136	0.9584
Listeria_monocytogenes_1639_v3	Listeria_monocytogenes_1639_v136	0.9098
Listeria_monocytogenes_1639_v39	Listeria_monocytogenes_1639_v136	0.9397
Listeria_monocytogenes_1639_v38	Listeria_monocytogenes_1639_v136	0.9555
Listeria_monocytogenes_1639_v37	Listeria_monocytogenes_1639_v136	0.9469
Listeria_monocytogenes_1639_v36	Listeria_monocytogenes_1639_v136	0.9451
Listeria_monocytogenes_1639_v35	Listeria_monocytogenes_1639_v136	0.9583
Listeria_monocytogenes_1639_v34	Listeria_monocytogenes_1639_v136	0.9541
Listeria_monocytogenes_1639_v33	Listeria_monocytogenes_1639_v136	0.9421
Listeria_monocytogenes_1639_v32	Listeria_monocytogenes_1639_v136	0.9499
Listeria_monocytogenes_1639_v31	Listeria_monocytogenes_1639_v136	0.9545
Listeria_monocytogenes_1639_v30	Listeria_monocytogenes_1639_v136	0.9319
Listeria_monocytogenes_1639_v2	Listeria_monocytogenes_1639_v136	0.895
Listeria_monocytogenes_1639_v29	Listeria_monocytogenes_1639_v136	0.9457
Listeria_monocytogenes_1639_v28	Listeria_monocytogenes_1639_v136	0.9495
Listeria_monocytogenes_1639_v27	Listeria_monocytogenes_1639_v136	0.9405
Listeria_monocytogenes_1639_v26	Listeria_monocytogenes_1639_v136	0.9398
Listeria_monocytogenes_1639_v25	Listeria_monocytogenes_1639_v136	0.9398
Listeria_monocytogenes_1639_v24	Listeria_monocytogenes_1639_v136	0.931
Listeria_monocytogenes_1639_v23	Listeria_monocytogenes_1639_v136	0.9306
Listeria_monocytogenes_1639_v22	Listeria_monocytogenes_1639_v136	0.9428
Listeria_monocytogenes_1639_v21	Listeria_monocytogenes_1639_v136	0.931
Listeria_monocytogenes_1639_v20	Listeria_monocytogenes_1639_v136	0.9328
Listeria_monocytogenes_1639_v1	Listeria_monocytogenes_1639_v136	0.9373
Listeria_monocytogenes_1639_v19	Listeria_monocytogenes_1639_v136	0.9324
Listeria_monocytogenes_1639_v198	Listeria_monocytogenes_1639_v136	0.9095
Listeria_monocytogenes_1639_v197	Listeria_monocytogenes_1639_v136	0.9417
Listeria_monocytogenes_1639_v196	Listeria_monocytogenes_1639_v136	0.9491
Listeria_monocytogenes_1639_v195	Listeria_monocytogenes_1639_v136	0.9392
Listeria_monocytogenes_1639_v194	Listeria_monocytogenes_1639_v136	0.9362
Listeria_monocytogenes_1639_v193	Listeria_monocytogenes_1639_v136	0.9366
Listeria_monocytogenes_1639_v192	Listeria_monocytogenes_1639_v136	0.8978
Listeria_monocytogenes_1639_v191	Listeria_monocytogenes_1639_v136	0.9684
Listeria_monocytogenes_1639_v190	Listeria_monocytogenes_1639_v136	0.9635
Listeria_monocytogenes_1639_v18	Listeria_monocytogenes_1639_v136	0.9171

Listeria_monocytogenes_1639_v189	Listeria_monocytogenes_1639_v136	0.9627
Listeria_monocytogenes_1639_v188	Listeria_monocytogenes_1639_v136	0.9622
Listeria_monocytogenes_1639_v187	Listeria_monocytogenes_1639_v136	0.9638
Listeria_monocytogenes_1639_v186	Listeria_monocytogenes_1639_v136	0.9634
Listeria_monocytogenes_1639_v185	Listeria_monocytogenes_1639_v136	0.9638
Listeria_monocytogenes_1639_v184	Listeria_monocytogenes_1639_v136	0.9634
Listeria_monocytogenes_1639_v183	Listeria_monocytogenes_1639_v136	0.9611
Listeria_monocytogenes_1639_v182	Listeria_monocytogenes_1639_v136	0.9634
Listeria_monocytogenes_1639_v181	Listeria_monocytogenes_1639_v136	0.9201
Listeria_monocytogenes_1639_v180	Listeria_monocytogenes_1639_v136	0.9201
Listeria_monocytogenes_1639_v17	Listeria_monocytogenes_1639_v136	0.9174
Listeria_monocytogenes_1639_v179	Listeria_monocytogenes_1639_v136	0.9513
Listeria_monocytogenes_1639_v178	Listeria_monocytogenes_1639_v136	0.9631
Listeria_monocytogenes_1639_v177	Listeria_monocytogenes_1639_v136	0.9416
Listeria_monocytogenes_1639_v176	Listeria_monocytogenes_1639_v136	0.9336
Listeria_monocytogenes_1639_v175	Listeria_monocytogenes_1639_v136	0.9699
Listeria_monocytogenes_1639_v174	Listeria_monocytogenes_1639_v136	0.9624
Listeria_monocytogenes_1639_v173	Listeria_monocytogenes_1639_v136	0.9497
Listeria_monocytogenes_1639_v172	Listeria_monocytogenes_1639_v136	0.9354
Listeria_monocytogenes_1639_v171	Listeria_monocytogenes_1639_v136	0.9512
Listeria_monocytogenes_1639_v170	Listeria_monocytogenes_1639_v136	0.9595
Listeria_monocytogenes_1639_v16	Listeria_monocytogenes_1639_v136	0.9372
Listeria_monocytogenes_1639_v169	Listeria_monocytogenes_1639_v136	0.9844
Listeria_monocytogenes_1639_v168	Listeria_monocytogenes_1639_v136	0.957
Listeria_monocytogenes_1639_v167	Listeria_monocytogenes_1639_v136	0.9534
Listeria_monocytogenes_1639_v166	Listeria_monocytogenes_1639_v136	0.9574
Listeria_monocytogenes_1639_v165	Listeria_monocytogenes_1639_v136	0.9844
Listeria_monocytogenes_1639_v164	Listeria_monocytogenes_1639_v136	0.9395
Listeria_monocytogenes_1639_v163	Listeria_monocytogenes_1639_v136	0.9423
Listeria_monocytogenes_1639_v162	Listeria_monocytogenes_1639_v136	0.9395
Listeria_monocytogenes_1639_v161	Listeria_monocytogenes_1639_v136	0.9403
Listeria_monocytogenes_1639_v160	Listeria_monocytogenes_1639_v136	0.94
Listeria_monocytogenes_1639_v15	Listeria_monocytogenes_1639_v136	0.9276
Listeria_monocytogenes_1639_v159	Listeria_monocytogenes_1639_v136	0.9416
Listeria_monocytogenes_1639_v158	Listeria_monocytogenes_1639_v136	0.94
Listeria_monocytogenes_1639_v157	Listeria_monocytogenes_1639_v136	0.94
Listeria_monocytogenes_1639_v156	Listeria_monocytogenes_1639_v136	0.9418
Listeria_monocytogenes_1639_v155	Listeria_monocytogenes_1639_v136	0.954
Listeria_monocytogenes_1639_v154	Listeria_monocytogenes_1639_v136	0.9681
Listeria_monocytogenes_1639_v153	Listeria_monocytogenes_1639_v136	0.9513
Listeria_monocytogenes_1639_v152	Listeria_monocytogenes_1639_v136	0.9416
Listeria_monocytogenes_1639_v151	Listeria_monocytogenes_1639_v136	0.9416
Listeria_monocytogenes_1639_v150	Listeria_monocytogenes_1639_v136	0.9673
Listeria_monocytogenes_1639_v14	Listeria_monocytogenes_1639_v136	0.9215
Listeria_monocytogenes_1639_v149	Listeria_monocytogenes_1639_v136	0.9658
Listeria_monocytogenes_1639_v148	Listeria_monocytogenes_1639_v136	0.9405
Listeria_monocytogenes_1639_v147	Listeria_monocytogenes_1639_v136	0.9271
Listeria_monocytogenes_1639_v146	Listeria_monocytogenes_1639_v136	0.9617
Listeria_monocytogenes_1639_v145	Listeria_monocytogenes_1639_v136	0.9572
Listeria_monocytogenes_1639_v144	Listeria_monocytogenes_1639_v136	0.9426
Listeria_monocytogenes_1639_v143	Listeria_monocytogenes_1639_v136	0.9682
Listeria_monocytogenes_1639_v142	Listeria_monocytogenes_1639_v136	0.943
Listeria_monocytogenes_1639_v141	Listeria_monocytogenes_1639_v136	0.949
Listeria_monocytogenes_1639_v140	Listeria_monocytogenes_1639_v136	0.9437
Listeria_monocytogenes_1639_v13	Listeria_monocytogenes_1639_v136	0.9276
Listeria_monocytogenes_1639_v139	Listeria_monocytogenes_1639_v136	0.9385
Listeria_monocytogenes_1639_v138	Listeria_monocytogenes_1639_v136	0.9449
Listeria_monocytogenes_1639_v137	Listeria_monocytogenes_1639_v136	0.9445
Listeria_monocytogenes_1639_v136	Listeria_monocytogenes_1639_v136	0.0648
Listeria_welshimeri_1643	Listeria_monocytogenes_1639_v137	0.8875
Listeria_welshimeri_serovar_6b_str_SLCC5334_386043	Listeria_monocytogenes_1639_v137	0.8872
Listeria_seeligeri_serovar_1_2b_str_SLCC3954_683837	Listeria_monocytogenes_1639_v137	0.8921
Listeria_seeligeri_FSL_S4-171_702452	Listeria_monocytogenes_1639_v137	0.8323
Listeria_seeligeri_FSL_N1-067_702453	Listeria_monocytogenes_1639_v137	0.8322
Listeria_seeligeri_1640_v1	Listeria_monocytogenes_1639_v137	0.8986
Listeria_seeligeri_1640	Listeria_monocytogenes_1639_v137	0.8897
Listeria_monocytogenes_WSLC1042_1457188	Listeria_monocytogenes_1639_v137	0.9743
Listeria_monocytogenes_WSLC1001_1457187	Listeria_monocytogenes_1639_v137	0.9405
Listeria_monocytogenes_SLCC7179_879090	Listeria_monocytogenes_1639_v137	0.9453
Listeria_monocytogenes_SLCC5850_882096	Listeria_monocytogenes_1639_v137	0.9452
Listeria_monocytogenes_SLCC2755_932919	Listeria_monocytogenes_1639_v137	0.9559
Listeria_monocytogenes_SLCC2540_879089	Listeria_monocytogenes_1639_v137	0.9542
Listeria_monocytogenes_SLCC2479_882020	Listeria_monocytogenes_1639_v137	0.9352
Listeria_monocytogenes_SLCC2378_879088	Listeria_monocytogenes_1639_v137	0.9755
Listeria_monocytogenes_SLCC2376_882097	Listeria_monocytogenes_1639_v137	0.9504
Listeria_monocytogenes_SLCC2372_932920	Listeria_monocytogenes_1639_v137	0.9278
Listeria_monocytogenes_serotype_4b_str_LL195_1230340	Listeria_monocytogenes_1639_v137	0.9819
Listeria_monocytogenes_serotype_4b_str_LIS0087_1134669	Listeria_monocytogenes_1639_v137	0.9211
Listeria_monocytogenes_serotype_4b_str_F2365_265669	Listeria_monocytogenes_1639_v137	0.9819
Listeria_monocytogenes_serotype_4b_str_CLIP_80459_568819	Listeria_monocytogenes_1639_v137	0.995
Listeria_monocytogenes_serotype_4b_str_81-0861_1196161	Listeria_monocytogenes_1639_v137	0.964
Listeria_monocytogenes_serotype_4b_str_81-0592_1196160	Listeria_monocytogenes_1639_v137	0.9651
Listeria_monocytogenes_serotype_4b_str_81-0558_1196159	Listeria_monocytogenes_1639_v137	0.9658
Listeria_monocytogenes_serotype_4b_str_10-0809_1196162	Listeria_monocytogenes_1639_v137	0.9655
Listeria_monocytogenes_serotype_4b_str_02-6680_1196175	Listeria_monocytogenes_1639_v137	0.983
Listeria_monocytogenes_serotype_4b_str_02-6679_1196174	Listeria_monocytogenes_1639_v137	0.983
Listeria_monocytogenes_serotype_4b_str_02-1792_1196173	Listeria_monocytogenes_1639_v137	0.9655
Listeria_monocytogenes_serotype_4b_str_02-1289_1196172	Listeria_monocytogenes_1639_v137	0.9655
Listeria_monocytogenes_serotype_4b_str_02-1103_1196171	Listeria_monocytogenes_1639_v137	0.9655
Listeria_monocytogenes_serotype_3c_str_10-5027_1196195	Listeria_monocytogenes_1639_v137	0.9344
Listeria_monocytogenes_serotype_1_2c_str_10-5026_1196194	Listeria_monocytogenes_1639_v137	0.9352

Listeria_monocytogenes_serotype_1_2c_str_10-5025_1196193	Listeria_monocytogenes_1639_v137	0.9352
Listeria_monocytogenes_serotype_1_2b_str_10-0811_1196164	Listeria_monocytogenes_1639_v137	0.9479
Listeria_monocytogenes_serotype_1_2b_str_10-0810_1196163	Listeria_monocytogenes_1639_v137	0.9479
Listeria_monocytogenes_serotype_1_2a_str_99-6370_1196179	Listeria_monocytogenes_1639_v137	0.926
Listeria_monocytogenes_serotype_1_2a_str_98-2035_1196178	Listeria_monocytogenes_1639_v137	0.926
Listeria_monocytogenes_serotype_1_2a_str_95-0093_1196177	Listeria_monocytogenes_1639_v137	0.9315
Listeria_monocytogenes_serotype_1_2a_str_88-0478_1196176	Listeria_monocytogenes_1639_v137	0.9303
Listeria_monocytogenes_serotype_1_2a_str_10-5024_1196192	Listeria_monocytogenes_1639_v137	0.9315
Listeria_monocytogenes_serotype_1_2a_str_10-4758_1196168	Listeria_monocytogenes_1639_v137	0.9449
Listeria_monocytogenes_serotype_1_2a_str_10-4754_1196167	Listeria_monocytogenes_1639_v137	0.9449
Listeria_monocytogenes_serotype_1_2a_str_10-1321_1196191	Listeria_monocytogenes_1639_v137	0.9315
Listeria_monocytogenes_serotype_1_2a_str_10-1047_1196190	Listeria_monocytogenes_1639_v137	0.9322
Listeria_monocytogenes_serotype_1_2a_str_10-1046_1196189	Listeria_monocytogenes_1639_v137	0.9322
Listeria_monocytogenes_serotype_1_2a_str_10-0934_1196170	Listeria_monocytogenes_1639_v137	0.9558
Listeria_monocytogenes_serotype_1_2a_str_10-0933_1196169	Listeria_monocytogenes_1639_v137	0.9558
Listeria_monocytogenes_serotype_1_2a_str_10-0815_1196188	Listeria_monocytogenes_1639_v137	0.9273
Listeria_monocytogenes_serotype_1_2a_str_10-0814_1196187	Listeria_monocytogenes_1639_v137	0.9319
Listeria_monocytogenes_serotype_1_2a_str_10-0813_1196166	Listeria_monocytogenes_1639_v137	0.938
Listeria_monocytogenes_serotype_1_2a_str_10-0812_1196165	Listeria_monocytogenes_1639_v137	0.938
Listeria_monocytogenes_serotype_1_2a_str_08-7669_1196186	Listeria_monocytogenes_1639_v137	0.941
Listeria_monocytogenes_serotype_1_2a_str_08-7374_1196185	Listeria_monocytogenes_1639_v137	0.9315
Listeria_monocytogenes_serotype_1_2a_str_08-6997_1196184	Listeria_monocytogenes_1639_v137	0.9273
Listeria_monocytogenes_serotype_1_2a_str_08-6569_1196183	Listeria_monocytogenes_1639_v137	0.9273
Listeria_monocytogenes_serotype_1_2a_str_08-6056_1196182	Listeria_monocytogenes_1639_v137	0.9273
Listeria_monocytogenes_serotype_1_2a_str_04-5457_1196181	Listeria_monocytogenes_1639_v137	0.9315
Listeria_monocytogenes_serotype_1_2a_str_02-5993_1196180	Listeria_monocytogenes_1639_v137	0.9315
Listeria_monocytogenes_R479a_1437838	Listeria_monocytogenes_1639_v137	0.9308
Listeria_monocytogenes_N53-1_1234142	Listeria_monocytogenes_1639_v137	0.8999
Listeria_monocytogenes_M7_1030009	Listeria_monocytogenes_1639_v137	0.9132
Listeria_monocytogenes_LO28_393132	Listeria_monocytogenes_1639_v137	0.9338
Listeria_monocytogenes_L99_563174	Listeria_monocytogenes_1639_v137	0.9135
Listeria_monocytogenes_L312_882094	Listeria_monocytogenes_1639_v137	0.9954
Listeria_monocytogenes_J1816_930781	Listeria_monocytogenes_1639_v137	0.9588
Listeria_monocytogenes_J1-220_930782	Listeria_monocytogenes_1639_v137	0.9548
Listeria_monocytogenes_J0161_393130	Listeria_monocytogenes_1639_v137	0.9353
Listeria_monocytogenes_HCC23_552536	Listeria_monocytogenes_1639_v137	0.9128
Listeria_monocytogenes_FSL_R2-561_393126	Listeria_monocytogenes_1639_v137	0.9338
Listeria_monocytogenes_FSL_J1-208_393119	Listeria_monocytogenes_1639_v137	0.9237
Listeria_monocytogenes_FSL_J1-175_393118	Listeria_monocytogenes_1639_v137	0.9598
Listeria_monocytogenes_Finland_1998_393127	Listeria_monocytogenes_1639_v137	0.9554
Listeria_monocytogenes_EGD-e_169963_v1	Listeria_monocytogenes_1639_v137	0.9427
Listeria_monocytogenes_EGD-e_169963	Listeria_monocytogenes_1639_v137	0.943
Listeria_monocytogenes_EGD_1334565	Listeria_monocytogenes_1639_v137	0.9459
Listeria_monocytogenes_CFSAN002349_1299895	Listeria_monocytogenes_1639_v137	0.9827
Listeria_monocytogenes_ATCC_19117_882095_v1	Listeria_monocytogenes_1639_v137	0.975
Listeria_monocytogenes_ATCC_19117_882095	Listeria_monocytogenes_1639_v137	0.9746
Listeria_monocytogenes_6179_1288295	Listeria_monocytogenes_1639_v137	0.9176
Listeria_monocytogenes_1639_v9	Listeria_monocytogenes_1639_v137	0.9304
Listeria_monocytogenes_1639_v99	Listeria_monocytogenes_1639_v137	0.9603
Listeria_monocytogenes_1639_v98	Listeria_monocytogenes_1639_v137	0.9326
Listeria_monocytogenes_1639_v97	Listeria_monocytogenes_1639_v137	0.9699
Listeria_monocytogenes_1639_v96	Listeria_monocytogenes_1639_v137	0.9592
Listeria_monocytogenes_1639_v95	Listeria_monocytogenes_1639_v137	0.9592
Listeria_monocytogenes_1639_v94	Listeria_monocytogenes_1639_v137	0.96
Listeria_monocytogenes_1639_v93	Listeria_monocytogenes_1639_v137	0.9303
Listeria_monocytogenes_1639_v92	Listeria_monocytogenes_1639_v137	0.96
Listeria_monocytogenes_1639_v91	Listeria_monocytogenes_1639_v137	0.9629
Listeria_monocytogenes_1639_v90	Listeria_monocytogenes_1639_v137	0.9326
Listeria_monocytogenes_1639_v8	Listeria_monocytogenes_1639_v137	0.9201
Listeria_monocytogenes_1639_v89	Listeria_monocytogenes_1639_v137	0.9694
Listeria_monocytogenes_1639_v88	Listeria_monocytogenes_1639_v137	0.9362
Listeria_monocytogenes_1639_v87	Listeria_monocytogenes_1639_v137	0.9334
Listeria_monocytogenes_1639_v86	Listeria_monocytogenes_1639_v137	0.933
Listeria_monocytogenes_1639_v85	Listeria_monocytogenes_1639_v137	0.9632
Listeria_monocytogenes_1639_v84	Listeria_monocytogenes_1639_v137	0.9414
Listeria_monocytogenes_1639_v83	Listeria_monocytogenes_1639_v137	0.9635
Listeria_monocytogenes_1639_v82	Listeria_monocytogenes_1639_v137	0.9628
Listeria_monocytogenes_1639_v81	Listeria_monocytogenes_1639_v137	0.9354
Listeria_monocytogenes_1639_v80	Listeria_monocytogenes_1639_v137	0.9524
Listeria_monocytogenes_1639_v7	Listeria_monocytogenes_1639_v137	0.9201
Listeria_monocytogenes_1639_v79	Listeria_monocytogenes_1639_v137	0.9336
Listeria_monocytogenes_1639_v78	Listeria_monocytogenes_1639_v137	0.9347
Listeria_monocytogenes_1639_v77	Listeria_monocytogenes_1639_v137	0.9344
Listeria_monocytogenes_1639_v76	Listeria_monocytogenes_1639_v137	0.9288
Listeria_monocytogenes_1639_v75	Listeria_monocytogenes_1639_v137	0.9135
Listeria_monocytogenes_1639_v74	Listeria_monocytogenes_1639_v137	0.9319
Listeria_monocytogenes_1639_v73	Listeria_monocytogenes_1639_v137	0.9289
Listeria_monocytogenes_1639_v72	Listeria_monocytogenes_1639_v137	0.9627
Listeria_monocytogenes_1639_v71	Listeria_monocytogenes_1639_v137	0.92
Listeria_monocytogenes_1639_v70	Listeria_monocytogenes_1639_v137	0.9313
Listeria_monocytogenes_1639_v6	Listeria_monocytogenes_1639_v137	0.9197
Listeria_monocytogenes_1639_v69	Listeria_monocytogenes_1639_v137	0.9651
Listeria_monocytogenes_1639_v68	Listeria_monocytogenes_1639_v137	0.9392
Listeria_monocytogenes_1639_v67	Listeria_monocytogenes_1639_v137	0.9517
Listeria_monocytogenes_1639_v66	Listeria_monocytogenes_1639_v137	0.9404
Listeria_monocytogenes_1639_v65	Listeria_monocytogenes_1639_v137	0.9347
Listeria_monocytogenes_1639_v64	Listeria_monocytogenes_1639_v137	0.9347
Listeria_monocytogenes_1639_v63	Listeria_monocytogenes_1639_v137	0.9486
Listeria_monocytogenes_1639_v62	Listeria_monocytogenes_1639_v137	0.9312
Listeria_monocytogenes_1639_v61	Listeria_monocytogenes_1639_v137	0.9343
Listeria_monocytogenes_1639_v60	Listeria_monocytogenes_1639_v137	0.9373

Listeria_monocytogenes_1639_v5	Listeria_monocytogenes_1639_v137	0.9129
Listeria_monocytogenes_1639_v59	Listeria_monocytogenes_1639_v137	0.9642
Listeria_monocytogenes_1639_v58	Listeria_monocytogenes_1639_v137	0.9639
Listeria_monocytogenes_1639_v57	Listeria_monocytogenes_1639_v137	0.956
Listeria_monocytogenes_1639_v56	Listeria_monocytogenes_1639_v137	0.9639
Listeria_monocytogenes_1639_v55	Listeria_monocytogenes_1639_v137	0.952
Listeria_monocytogenes_1639_v54	Listeria_monocytogenes_1639_v137	0.9295
Listeria_monocytogenes_1639_v53	Listeria_monocytogenes_1639_v137	0.9365
Listeria_monocytogenes_1639_v52	Listeria_monocytogenes_1639_v137	0.961
Listeria_monocytogenes_1639_v51	Listeria_monocytogenes_1639_v137	0.9577
Listeria_monocytogenes_1639_v50	Listeria_monocytogenes_1639_v137	0.9307
Listeria_monocytogenes_1639_v4	Listeria_monocytogenes_1639_v137	0.9259
Listeria_monocytogenes_1639_v49	Listeria_monocytogenes_1639_v137	0.9647
Listeria_monocytogenes_1639_v48	Listeria_monocytogenes_1639_v137	0.9296
Listeria_monocytogenes_1639_v47	Listeria_monocytogenes_1639_v137	0.9312
Listeria_monocytogenes_1639_v46	Listeria_monocytogenes_1639_v137	0.9385
Listeria_monocytogenes_1639_v45	Listeria_monocytogenes_1639_v137	0.9222
Listeria_monocytogenes_1639_v44	Listeria_monocytogenes_1639_v137	0.9315
Listeria_monocytogenes_1639_v43	Listeria_monocytogenes_1639_v137	0.9315
Listeria_monocytogenes_1639_v42	Listeria_monocytogenes_1639_v137	0.9225
Listeria_monocytogenes_1639_v41	Listeria_monocytogenes_1639_v137	0.9098
Listeria_monocytogenes_1639_v40	Listeria_monocytogenes_1639_v137	0.9341
Listeria_monocytogenes_1639_v3	Listeria_monocytogenes_1639_v137	0.9263
Listeria_monocytogenes_1639_v39	Listeria_monocytogenes_1639_v137	0.9471
Listeria_monocytogenes_1639_v38	Listeria_monocytogenes_1639_v137	0.9306
Listeria_monocytogenes_1639_v37	Listeria_monocytogenes_1639_v137	0.9265
Listeria_monocytogenes_1639_v36	Listeria_monocytogenes_1639_v137	0.9258
Listeria_monocytogenes_1639_v35	Listeria_monocytogenes_1639_v137	0.9348
Listeria_monocytogenes_1639_v34	Listeria_monocytogenes_1639_v137	0.9314
Listeria_monocytogenes_1639_v33	Listeria_monocytogenes_1639_v137	0.9479
Listeria_monocytogenes_1639_v32	Listeria_monocytogenes_1639_v137	0.9254
Listeria_monocytogenes_1639_v31	Listeria_monocytogenes_1639_v137	0.9318
Listeria_monocytogenes_1639_v30	Listeria_monocytogenes_1639_v137	0.9471
Listeria_monocytogenes_1639_v2	Listeria_monocytogenes_1639_v137	0.9132
Listeria_monocytogenes_1639_v29	Listeria_monocytogenes_1639_v137	0.9206
Listeria_monocytogenes_1639_v28	Listeria_monocytogenes_1639_v137	0.925
Listeria_monocytogenes_1639_v27	Listeria_monocytogenes_1639_v137	0.9158
Listeria_monocytogenes_1639_v26	Listeria_monocytogenes_1639_v137	0.9155
Listeria_monocytogenes_1639_v25	Listeria_monocytogenes_1639_v137	0.9155
Listeria_monocytogenes_1639_v24	Listeria_monocytogenes_1639_v137	0.9548
Listeria_monocytogenes_1639_v23	Listeria_monocytogenes_1639_v137	0.9544
Listeria_monocytogenes_1639_v22	Listeria_monocytogenes_1639_v137	0.949
Listeria_monocytogenes_1639_v21	Listeria_monocytogenes_1639_v137	0.9544
Listeria_monocytogenes_1639_v20	Listeria_monocytogenes_1639_v137	0.9437
Listeria_monocytogenes_1639_v1	Listeria_monocytogenes_1639_v137	0.9112
Listeria_monocytogenes_1639_v19	Listeria_monocytogenes_1639_v137	0.9434
Listeria_monocytogenes_1639_v198	Listeria_monocytogenes_1639_v137	0.9026
Listeria_monocytogenes_1639_v197	Listeria_monocytogenes_1639_v137	0.9305
Listeria_monocytogenes_1639_v196	Listeria_monocytogenes_1639_v137	0.9287
Listeria_monocytogenes_1639_v195	Listeria_monocytogenes_1639_v137	0.9507
Listeria_monocytogenes_1639_v194	Listeria_monocytogenes_1639_v137	0.9508
Listeria_monocytogenes_1639_v193	Listeria_monocytogenes_1639_v137	0.9508
Listeria_monocytogenes_1639_v192	Listeria_monocytogenes_1639_v137	0.9154
Listeria_monocytogenes_1639_v191	Listeria_monocytogenes_1639_v137	0.958
Listeria_monocytogenes_1639_v190	Listeria_monocytogenes_1639_v137	0.9573
Listeria_monocytogenes_1639_v18	Listeria_monocytogenes_1639_v137	0.9499
Listeria_monocytogenes_1639_v189	Listeria_monocytogenes_1639_v137	0.9565
Listeria_monocytogenes_1639_v188	Listeria_monocytogenes_1639_v137	0.9568
Listeria_monocytogenes_1639_v187	Listeria_monocytogenes_1639_v137	0.9572
Listeria_monocytogenes_1639_v186	Listeria_monocytogenes_1639_v137	0.9576
Listeria_monocytogenes_1639_v185	Listeria_monocytogenes_1639_v137	0.9576
Listeria_monocytogenes_1639_v184	Listeria_monocytogenes_1639_v137	0.9565
Listeria_monocytogenes_1639_v183	Listeria_monocytogenes_1639_v137	0.9518
Listeria_monocytogenes_1639_v182	Listeria_monocytogenes_1639_v137	0.954
Listeria_monocytogenes_1639_v181	Listeria_monocytogenes_1639_v137	0.9385
Listeria_monocytogenes_1639_v180	Listeria_monocytogenes_1639_v137	0.9385
Listeria_monocytogenes_1639_v17	Listeria_monocytogenes_1639_v137	0.9503
Listeria_monocytogenes_1639_v179	Listeria_monocytogenes_1639_v137	0.9404
Listeria_monocytogenes_1639_v178	Listeria_monocytogenes_1639_v137	0.9557
Listeria_monocytogenes_1639_v177	Listeria_monocytogenes_1639_v137	0.9796
Listeria_monocytogenes_1639_v176	Listeria_monocytogenes_1639_v137	0.9481
Listeria_monocytogenes_1639_v175	Listeria_monocytogenes_1639_v137	0.9457
Listeria_monocytogenes_1639_v174	Listeria_monocytogenes_1639_v137	0.9577
Listeria_monocytogenes_1639_v173	Listeria_monocytogenes_1639_v137	0.9728
Listeria_monocytogenes_1639_v172	Listeria_monocytogenes_1639_v137	0.9464
Listeria_monocytogenes_1639_v171	Listeria_monocytogenes_1639_v137	0.9426
Listeria_monocytogenes_1639_v170	Listeria_monocytogenes_1639_v137	0.951
Listeria_monocytogenes_1639_v16	Listeria_monocytogenes_1639_v137	0.9177
Listeria_monocytogenes_1639_v169	Listeria_monocytogenes_1639_v137	0.9518
Listeria_monocytogenes_1639_v168	Listeria_monocytogenes_1639_v137	0.9516
Listeria_monocytogenes_1639_v167	Listeria_monocytogenes_1639_v137	0.9421
Listeria_monocytogenes_1639_v166	Listeria_monocytogenes_1639_v137	0.9519
Listeria_monocytogenes_1639_v165	Listeria_monocytogenes_1639_v137	0.9518
Listeria_monocytogenes_1639_v164	Listeria_monocytogenes_1639_v137	0.9814
Listeria_monocytogenes_1639_v163	Listeria_monocytogenes_1639_v137	0.9695
Listeria_monocytogenes_1639_v162	Listeria_monocytogenes_1639_v137	0.9814
Listeria_monocytogenes_1639_v161	Listeria_monocytogenes_1639_v137	0.9826
Listeria_monocytogenes_1639_v160	Listeria_monocytogenes_1639_v137	0.9822
Listeria_monocytogenes_1639_v15	Listeria_monocytogenes_1639_v137	0.9325
Listeria_monocytogenes_1639_v159	Listeria_monocytogenes_1639_v137	0.98
Listeria_monocytogenes_1639_v158	Listeria_monocytogenes_1639_v137	0.9819

Listeria_monocytogenes_1639_v157	Listeria_monocytogenes_1639_v137	0.9819
Listeria_monocytogenes_1639_v156	Listeria_monocytogenes_1639_v137	0.934
Listeria_monocytogenes_1639_v155	Listeria_monocytogenes_1639_v137	0.9447
Listeria_monocytogenes_1639_v154	Listeria_monocytogenes_1639_v137	0.9505
Listeria_monocytogenes_1639_v153	Listeria_monocytogenes_1639_v137	0.9454
Listeria_monocytogenes_1639_v152	Listeria_monocytogenes_1639_v137	0.98
Listeria_monocytogenes_1639_v151	Listeria_monocytogenes_1639_v137	0.98
Listeria_monocytogenes_1639_v150	Listeria_monocytogenes_1639_v137	0.9473
Listeria_monocytogenes_1639_v14	Listeria_monocytogenes_1639_v137	0.9391
Listeria_monocytogenes_1639_v149	Listeria_monocytogenes_1639_v137	0.9463
Listeria_monocytogenes_1639_v148	Listeria_monocytogenes_1639_v137	0.9788
Listeria_monocytogenes_1639_v147	Listeria_monocytogenes_1639_v137	0.9643
Listeria_monocytogenes_1639_v146	Listeria_monocytogenes_1639_v137	0.9555
Listeria_monocytogenes_1639_v145	Listeria_monocytogenes_1639_v137	0.9513
Listeria_monocytogenes_1639_v144	Listeria_monocytogenes_1639_v137	0.9946
Listeria_monocytogenes_1639_v143	Listeria_monocytogenes_1639_v137	0.9547
Listeria_monocytogenes_1639_v142	Listeria_monocytogenes_1639_v137	0.995
Listeria_monocytogenes_1639_v141	Listeria_monocytogenes_1639_v137	0.9745
Listeria_monocytogenes_1639_v140	Listeria_monocytogenes_1639_v137	0.9957
Listeria_monocytogenes_1639_v13	Listeria_monocytogenes_1639_v137	0.9325
Listeria_monocytogenes_1639_v139	Listeria_monocytogenes_1639_v137	0.9799
Listeria_monocytogenes_1639_v138	Listeria_monocytogenes_1639_v137	0.9992
Listeria_monocytogenes_1639_v137	Listeria_monocytogenes_1639_v137	0.0653
Listeria_welshimeri_1643	Listeria_monocytogenes_1639_v138	0.8883
Listeria_welshimeri_serovar_6b_str_SLCC5334_386043	Listeria_monocytogenes_1639_v138	0.8879
Listeria_seeligeri_serovar_1_2b_str_SLCC3954_683837	Listeria_monocytogenes_1639_v138	0.8929
Listeria_seeligeri_FSL_S4-171_702452	Listeria_monocytogenes_1639_v138	0.8331
Listeria_seeligeri_FSL_N1-067_702453	Listeria_monocytogenes_1639_v138	0.833
Listeria_seeligeri_1640_v1	Listeria_monocytogenes_1639_v138	0.8993
Listeria_seeligeri_1640	Listeria_monocytogenes_1639_v138	0.8904
Listeria_monocytogenes_WSLC1042_1457188	Listeria_monocytogenes_1639_v138	0.9743
Listeria_monocytogenes_WSLC1001_1457187	Listeria_monocytogenes_1639_v138	0.9409
Listeria_monocytogenes_SLCC7179_879090	Listeria_monocytogenes_1639_v138	0.9457
Listeria_monocytogenes_SLCC5850_882096	Listeria_monocytogenes_1639_v138	0.9456
Listeria_monocytogenes_SLCC2755_932919	Listeria_monocytogenes_1639_v138	0.9559
Listeria_monocytogenes_SLCC2540_879089	Listeria_monocytogenes_1639_v138	0.9542
Listeria_monocytogenes_SLCC2479_882020	Listeria_monocytogenes_1639_v138	0.9356
Listeria_monocytogenes_SLCC2378_879088	Listeria_monocytogenes_1639_v138	0.9755
Listeria_monocytogenes_SLCC2376_882097	Listeria_monocytogenes_1639_v138	0.9508
Listeria_monocytogenes_SLCC2372_932920	Listeria_monocytogenes_1639_v138	0.9282
Listeria_monocytogenes_serotype_4b_str_LL195_1230340	Listeria_monocytogenes_1639_v138	0.9819
Listeria_monocytogenes_serotype_4b_str_LIS0087_1134669	Listeria_monocytogenes_1639_v138	0.9215
Listeria_monocytogenes_serotype_4b_str_F2365_265669	Listeria_monocytogenes_1639_v138	0.9819
Listeria_monocytogenes_serotype_4b_str_CLIP_80459_568819	Listeria_monocytogenes_1639_v138	0.995
Listeria_monocytogenes_serotype_4b_str_81-0861_1196161	Listeria_monocytogenes_1639_v138	0.964
Listeria_monocytogenes_serotype_4b_str_81-0592_1196160	Listeria_monocytogenes_1639_v138	0.9651
Listeria_monocytogenes_serotype_4b_str_81-0558_1196159	Listeria_monocytogenes_1639_v138	0.9658
Listeria_monocytogenes_serotype_4b_str_10-0809_1196162	Listeria_monocytogenes_1639_v138	0.9655
Listeria_monocytogenes_serotype_4b_str_02-6680_1196175	Listeria_monocytogenes_1639_v138	0.9834
Listeria_monocytogenes_serotype_4b_str_02-6679_1196174	Listeria_monocytogenes_1639_v138	0.9834
Listeria_monocytogenes_serotype_4b_str_02-1792_1196173	Listeria_monocytogenes_1639_v138	0.9655
Listeria_monocytogenes_serotype_4b_str_02-1289_1196172	Listeria_monocytogenes_1639_v138	0.9655
Listeria_monocytogenes_serotype_4b_str_02-1103_1196171	Listeria_monocytogenes_1639_v138	0.9655
Listeria_monocytogenes_serotype_3c_str_10-5027_1196195	Listeria_monocytogenes_1639_v138	0.9348
Listeria_monocytogenes_serotype_1_2c_str_10-5026_1196194	Listeria_monocytogenes_1639_v138	0.9356
Listeria_monocytogenes_serotype_1_2c_str_10-5025_1196193	Listeria_monocytogenes_1639_v138	0.9356
Listeria_monocytogenes_serotype_1_2b_str_10-0811_1196164	Listeria_monocytogenes_1639_v138	0.9479
Listeria_monocytogenes_serotype_1_2b_str_10-0810_1196163	Listeria_monocytogenes_1639_v138	0.9479
Listeria_monocytogenes_serotype_1_2a_str_99-6370_1196179	Listeria_monocytogenes_1639_v138	0.9263
Listeria_monocytogenes_serotype_1_2a_str_98-2035_1196178	Listeria_monocytogenes_1639_v138	0.9263
Listeria_monocytogenes_serotype_1_2a_str_95-0093_1196177	Listeria_monocytogenes_1639_v138	0.9319
Listeria_monocytogenes_serotype_1_2a_str_88-0478_1196176	Listeria_monocytogenes_1639_v138	0.9307
Listeria_monocytogenes_serotype_1_2a_str_10-5024_1196192	Listeria_monocytogenes_1639_v138	0.9319
Listeria_monocytogenes_serotype_1_2a_str_10-4758_1196168	Listeria_monocytogenes_1639_v138	0.9453
Listeria_monocytogenes_serotype_1_2a_str_10-4754_1196167	Listeria_monocytogenes_1639_v138	0.9453
Listeria_monocytogenes_serotype_1_2a_str_10-1321_1196191	Listeria_monocytogenes_1639_v138	0.9319
Listeria_monocytogenes_serotype_1_2a_str_10-1047_1196190	Listeria_monocytogenes_1639_v138	0.9326
Listeria_monocytogenes_serotype_1_2a_str_10-1046_1196189	Listeria_monocytogenes_1639_v138	0.9326
Listeria_monocytogenes_serotype_1_2a_str_10-0934_1196170	Listeria_monocytogenes_1639_v138	0.9562
Listeria_monocytogenes_serotype_1_2a_str_10-0933_1196169	Listeria_monocytogenes_1639_v138	0.9562
Listeria_monocytogenes_serotype_1_2a_str_10-0815_1196188	Listeria_monocytogenes_1639_v138	0.9277
Listeria_monocytogenes_serotype_1_2a_str_10-0814_1196187	Listeria_monocytogenes_1639_v138	0.9323
Listeria_monocytogenes_serotype_1_2a_str_10-0813_1196166	Listeria_monocytogenes_1639_v138	0.9384
Listeria_monocytogenes_serotype_1_2a_str_10-0812_1196165	Listeria_monocytogenes_1639_v138	0.9384
Listeria_monocytogenes_serotype_1_2a_str_08-7669_1196186	Listeria_monocytogenes_1639_v138	0.9414
Listeria_monocytogenes_serotype_1_2a_str_08-7374_1196185	Listeria_monocytogenes_1639_v138	0.9319
Listeria_monocytogenes_serotype_1_2a_str_08-6997_1196184	Listeria_monocytogenes_1639_v138	0.9277
Listeria_monocytogenes_serotype_1_2a_str_08-6569_1196183	Listeria_monocytogenes_1639_v138	0.9277
Listeria_monocytogenes_serotype_1_2a_str_08-6056_1196182	Listeria_monocytogenes_1639_v138	0.9277
Listeria_monocytogenes_serotype_1_2a_str_04-5457_1196181	Listeria_monocytogenes_1639_v138	0.9319
Listeria_monocytogenes_serotype_1_2a_str_02-5993_1196180	Listeria_monocytogenes_1639_v138	0.9319
Listeria_monocytogenes_R479a_1437838	Listeria_monocytogenes_1639_v138	0.9312
Listeria_monocytogenes_N53-1_1234142	Listeria_monocytogenes_1639_v138	0.9003
Listeria_monocytogenes_M7_1030009	Listeria_monocytogenes_1639_v138	0.9136
Listeria_monocytogenes_LO28_393132	Listeria_monocytogenes_1639_v138	0.9342
Listeria_monocytogenes_L99_563174	Listeria_monocytogenes_1639_v138	0.9139
Listeria_monocytogenes_L312_882094	Listeria_monocytogenes_1639_v138	0.9954
Listeria_monocytogenes_J1816_930781	Listeria_monocytogenes_1639_v138	0.9595
Listeria_monocytogenes_J1-220_930782	Listeria_monocytogenes_1639_v138	0.9548
Listeria_monocytogenes_J0161_393130	Listeria_monocytogenes_1639_v138	0.9357
Listeria_monocytogenes_HCC23_552536	Listeria_monocytogenes_1639_v138	0.9132

Listeria_monocytogenes_FSL_R2-561_393126	Listeria_monocytogenes_1639_v138	0.9341
Listeria_monocytogenes_FSL_J1-208_393119	Listeria_monocytogenes_1639_v138	0.9241
Listeria_monocytogenes_FSL_J1-175_393118	Listeria_monocytogenes_1639_v138	0.9598
Listeria_monocytogenes_Finland_1998_393127	Listeria_monocytogenes_1639_v138	0.9558
Listeria_monocytogenes_EGD-e_169963_v1	Listeria_monocytogenes_1639_v138	0.943
Listeria_monocytogenes_EGD-e_169963	Listeria_monocytogenes_1639_v138	0.9434
Listeria_monocytogenes_EGD_1334565	Listeria_monocytogenes_1639_v138	0.9463
Listeria_monocytogenes_CFSAN002349_1299895	Listeria_monocytogenes_1639_v138	0.983
Listeria_monocytogenes_ATCC_19117_882095_v1	Listeria_monocytogenes_1639_v138	0.975
Listeria_monocytogenes_ATCC_19117_882095	Listeria_monocytogenes_1639_v138	0.9746
Listeria_monocytogenes_6179_1288295	Listeria_monocytogenes_1639_v138	0.9179
Listeria_monocytogenes_1639_v9	Listeria_monocytogenes_1639_v138	0.9304
Listeria_monocytogenes_1639_v99	Listeria_monocytogenes_1639_v138	0.9607
Listeria_monocytogenes_1639_v98	Listeria_monocytogenes_1639_v138	0.933
Listeria_monocytogenes_1639_v97	Listeria_monocytogenes_1639_v138	0.9699
Listeria_monocytogenes_1639_v96	Listeria_monocytogenes_1639_v138	0.9596
Listeria_monocytogenes_1639_v95	Listeria_monocytogenes_1639_v138	0.9596
Listeria_monocytogenes_1639_v94	Listeria_monocytogenes_1639_v138	0.9603
Listeria_monocytogenes_1639_v93	Listeria_monocytogenes_1639_v138	0.9307
Listeria_monocytogenes_1639_v92	Listeria_monocytogenes_1639_v138	0.9603
Listeria_monocytogenes_1639_v91	Listeria_monocytogenes_1639_v138	0.9629
Listeria_monocytogenes_1639_v90	Listeria_monocytogenes_1639_v138	0.933
Listeria_monocytogenes_1639_v8	Listeria_monocytogenes_1639_v138	0.9204
Listeria_monocytogenes_1639_v89	Listeria_monocytogenes_1639_v138	0.9694
Listeria_monocytogenes_1639_v88	Listeria_monocytogenes_1639_v138	0.9366
Listeria_monocytogenes_1639_v87	Listeria_monocytogenes_1639_v138	0.9338
Listeria_monocytogenes_1639_v86	Listeria_monocytogenes_1639_v138	0.9334
Listeria_monocytogenes_1639_v85	Listeria_monocytogenes_1639_v138	0.9636
Listeria_monocytogenes_1639_v84	Listeria_monocytogenes_1639_v138	0.9417
Listeria_monocytogenes_1639_v83	Listeria_monocytogenes_1639_v138	0.9635
Listeria_monocytogenes_1639_v82	Listeria_monocytogenes_1639_v138	0.9631
Listeria_monocytogenes_1639_v81	Listeria_monocytogenes_1639_v138	0.9358
Listeria_monocytogenes_1639_v80	Listeria_monocytogenes_1639_v138	0.9528
Listeria_monocytogenes_1639_v7	Listeria_monocytogenes_1639_v138	0.9204
Listeria_monocytogenes_1639_v79	Listeria_monocytogenes_1639_v138	0.934
Listeria_monocytogenes_1639_v78	Listeria_monocytogenes_1639_v138	0.9351
Listeria_monocytogenes_1639_v77	Listeria_monocytogenes_1639_v138	0.9348
Listeria_monocytogenes_1639_v76	Listeria_monocytogenes_1639_v138	0.9291
Listeria_monocytogenes_1639_v75	Listeria_monocytogenes_1639_v138	0.9139
Listeria_monocytogenes_1639_v74	Listeria_monocytogenes_1639_v138	0.9323
Listeria_monocytogenes_1639_v73	Listeria_monocytogenes_1639_v138	0.9292
Listeria_monocytogenes_1639_v72	Listeria_monocytogenes_1639_v138	0.9623
Listeria_monocytogenes_1639_v71	Listeria_monocytogenes_1639_v138	0.9204
Listeria_monocytogenes_1639_v70	Listeria_monocytogenes_1639_v138	0.9317
Listeria_monocytogenes_1639_v6	Listeria_monocytogenes_1639_v138	0.9201
Listeria_monocytogenes_1639_v69	Listeria_monocytogenes_1639_v138	0.9651
Listeria_monocytogenes_1639_v68	Listeria_monocytogenes_1639_v138	0.9396
Listeria_monocytogenes_1639_v67	Listeria_monocytogenes_1639_v138	0.9517
Listeria_monocytogenes_1639_v66	Listeria_monocytogenes_1639_v138	0.9407
Listeria_monocytogenes_1639_v65	Listeria_monocytogenes_1639_v138	0.9351
Listeria_monocytogenes_1639_v64	Listeria_monocytogenes_1639_v138	0.9351
Listeria_monocytogenes_1639_v63	Listeria_monocytogenes_1639_v138	0.9486
Listeria_monocytogenes_1639_v62	Listeria_monocytogenes_1639_v138	0.9315
Listeria_monocytogenes_1639_v61	Listeria_monocytogenes_1639_v138	0.9347
Listeria_monocytogenes_1639_v60	Listeria_monocytogenes_1639_v138	0.9377
Listeria_monocytogenes_1639_v5	Listeria_monocytogenes_1639_v138	0.9125
Listeria_monocytogenes_1639_v59	Listeria_monocytogenes_1639_v138	0.9642
Listeria_monocytogenes_1639_v58	Listeria_monocytogenes_1639_v138	0.9639
Listeria_monocytogenes_1639_v57	Listeria_monocytogenes_1639_v138	0.9564
Listeria_monocytogenes_1639_v56	Listeria_monocytogenes_1639_v138	0.9639
Listeria_monocytogenes_1639_v55	Listeria_monocytogenes_1639_v138	0.9524
Listeria_monocytogenes_1639_v54	Listeria_monocytogenes_1639_v138	0.9299
Listeria_monocytogenes_1639_v53	Listeria_monocytogenes_1639_v138	0.9369
Listeria_monocytogenes_1639_v52	Listeria_monocytogenes_1639_v138	0.961
Listeria_monocytogenes_1639_v51	Listeria_monocytogenes_1639_v138	0.9577
Listeria_monocytogenes_1639_v50	Listeria_monocytogenes_1639_v138	0.9311
Listeria_monocytogenes_1639_v4	Listeria_monocytogenes_1639_v138	0.9259
Listeria_monocytogenes_1639_v49	Listeria_monocytogenes_1639_v138	0.9647
Listeria_monocytogenes_1639_v48	Listeria_monocytogenes_1639_v138	0.9299
Listeria_monocytogenes_1639_v47	Listeria_monocytogenes_1639_v138	0.9315
Listeria_monocytogenes_1639_v46	Listeria_monocytogenes_1639_v138	0.9389
Listeria_monocytogenes_1639_v45	Listeria_monocytogenes_1639_v138	0.9225
Listeria_monocytogenes_1639_v44	Listeria_monocytogenes_1639_v138	0.9319
Listeria_monocytogenes_1639_v43	Listeria_monocytogenes_1639_v138	0.9319
Listeria_monocytogenes_1639_v42	Listeria_monocytogenes_1639_v138	0.9233
Listeria_monocytogenes_1639_v41	Listeria_monocytogenes_1639_v138	0.9105
Listeria_monocytogenes_1639_v40	Listeria_monocytogenes_1639_v138	0.9345
Listeria_monocytogenes_1639_v3	Listeria_monocytogenes_1639_v138	0.9263
Listeria_monocytogenes_1639_v39	Listeria_monocytogenes_1639_v138	0.9471
Listeria_monocytogenes_1639_v38	Listeria_monocytogenes_1639_v138	0.9309
Listeria_monocytogenes_1639_v37	Listeria_monocytogenes_1639_v138	0.9268
Listeria_monocytogenes_1639_v36	Listeria_monocytogenes_1639_v138	0.9262
Listeria_monocytogenes_1639_v35	Listeria_monocytogenes_1639_v138	0.9352
Listeria_monocytogenes_1639_v34	Listeria_monocytogenes_1639_v138	0.9318
Listeria_monocytogenes_1639_v33	Listeria_monocytogenes_1639_v138	0.9479
Listeria_monocytogenes_1639_v32	Listeria_monocytogenes_1639_v138	0.9258
Listeria_monocytogenes_1639_v31	Listeria_monocytogenes_1639_v138	0.9322
Listeria_monocytogenes_1639_v30	Listeria_monocytogenes_1639_v138	0.9471
Listeria_monocytogenes_1639_v2	Listeria_monocytogenes_1639_v138	0.9132
Listeria_monocytogenes_1639_v29	Listeria_monocytogenes_1639_v138	0.9209
Listeria_monocytogenes_1639_v28	Listeria_monocytogenes_1639_v138	0.9254

Listeria_monocytogenes_1639_v27	Listeria_monocytogenes_1639_v138	0.9161
Listeria_monocytogenes_1639_v26	Listeria_monocytogenes_1639_v138	0.9158
Listeria_monocytogenes_1639_v25	Listeria_monocytogenes_1639_v138	0.9158
Listeria_monocytogenes_1639_v24	Listeria_monocytogenes_1639_v138	0.9548
Listeria_monocytogenes_1639_v23	Listeria_monocytogenes_1639_v138	0.9544
Listeria_monocytogenes_1639_v22	Listeria_monocytogenes_1639_v138	0.949
Listeria_monocytogenes_1639_v21	Listeria_monocytogenes_1639_v138	0.9544
Listeria_monocytogenes_1639_v20	Listeria_monocytogenes_1639_v138	0.9437
Listeria_monocytogenes_1639_v1	Listeria_monocytogenes_1639_v138	0.9116
Listeria_monocytogenes_1639_v19	Listeria_monocytogenes_1639_v138	0.9434
Listeria_monocytogenes_1639_v198	Listeria_monocytogenes_1639_v138	0.9034
Listeria_monocytogenes_1639_v197	Listeria_monocytogenes_1639_v138	0.9305
Listeria_monocytogenes_1639_v196	Listeria_monocytogenes_1639_v138	0.929
Listeria_monocytogenes_1639_v195	Listeria_monocytogenes_1639_v138	0.9511
Listeria_monocytogenes_1639_v194	Listeria_monocytogenes_1639_v138	0.9512
Listeria_monocytogenes_1639_v193	Listeria_monocytogenes_1639_v138	0.9512
Listeria_monocytogenes_1639_v192	Listeria_monocytogenes_1639_v138	0.9157
Listeria_monocytogenes_1639_v191	Listeria_monocytogenes_1639_v138	0.9588
Listeria_monocytogenes_1639_v190	Listeria_monocytogenes_1639_v138	0.9577
Listeria_monocytogenes_1639_v18	Listeria_monocytogenes_1639_v138	0.9503
Listeria_monocytogenes_1639_v189	Listeria_monocytogenes_1639_v138	0.9569
Listeria_monocytogenes_1639_v188	Listeria_monocytogenes_1639_v138	0.9572
Listeria_monocytogenes_1639_v187	Listeria_monocytogenes_1639_v138	0.9576
Listeria_monocytogenes_1639_v186	Listeria_monocytogenes_1639_v138	0.958
Listeria_monocytogenes_1639_v185	Listeria_monocytogenes_1639_v138	0.958
Listeria_monocytogenes_1639_v184	Listeria_monocytogenes_1639_v138	0.9569
Listeria_monocytogenes_1639_v183	Listeria_monocytogenes_1639_v138	0.9526
Listeria_monocytogenes_1639_v182	Listeria_monocytogenes_1639_v138	0.9544
Listeria_monocytogenes_1639_v181	Listeria_monocytogenes_1639_v138	0.9389
Listeria_monocytogenes_1639_v180	Listeria_monocytogenes_1639_v138	0.9389
Listeria_monocytogenes_1639_v17	Listeria_monocytogenes_1639_v138	0.9507
Listeria_monocytogenes_1639_v179	Listeria_monocytogenes_1639_v138	0.9408
Listeria_monocytogenes_1639_v178	Listeria_monocytogenes_1639_v138	0.9561
Listeria_monocytogenes_1639_v177	Listeria_monocytogenes_1639_v138	0.9803
Listeria_monocytogenes_1639_v176	Listeria_monocytogenes_1639_v138	0.9485
Listeria_monocytogenes_1639_v175	Listeria_monocytogenes_1639_v138	0.9461
Listeria_monocytogenes_1639_v174	Listeria_monocytogenes_1639_v138	0.9581
Listeria_monocytogenes_1639_v173	Listeria_monocytogenes_1639_v138	0.9732
Listeria_monocytogenes_1639_v172	Listeria_monocytogenes_1639_v138	0.9467
Listeria_monocytogenes_1639_v171	Listeria_monocytogenes_1639_v138	0.943
Listeria_monocytogenes_1639_v170	Listeria_monocytogenes_1639_v138	0.9514
Listeria_monocytogenes_1639_v16	Listeria_monocytogenes_1639_v138	0.9181
Listeria_monocytogenes_1639_v169	Listeria_monocytogenes_1639_v138	0.9522
Listeria_monocytogenes_1639_v168	Listeria_monocytogenes_1639_v138	0.952
Listeria_monocytogenes_1639_v167	Listeria_monocytogenes_1639_v138	0.9425
Listeria_monocytogenes_1639_v166	Listeria_monocytogenes_1639_v138	0.9523
Listeria_monocytogenes_1639_v165	Listeria_monocytogenes_1639_v138	0.9522
Listeria_monocytogenes_1639_v164	Listeria_monocytogenes_1639_v138	0.9814
Listeria_monocytogenes_1639_v163	Listeria_monocytogenes_1639_v138	0.9699
Listeria_monocytogenes_1639_v162	Listeria_monocytogenes_1639_v138	0.9814
Listeria_monocytogenes_1639_v161	Listeria_monocytogenes_1639_v138	0.9826
Listeria_monocytogenes_1639_v160	Listeria_monocytogenes_1639_v138	0.9826
Listeria_monocytogenes_1639_v15	Listeria_monocytogenes_1639_v138	0.9325
Listeria_monocytogenes_1639_v159	Listeria_monocytogenes_1639_v138	0.9803
Listeria_monocytogenes_1639_v158	Listeria_monocytogenes_1639_v138	0.9819
Listeria_monocytogenes_1639_v157	Listeria_monocytogenes_1639_v138	0.9819
Listeria_monocytogenes_1639_v156	Listeria_monocytogenes_1639_v138	0.9344
Listeria_monocytogenes_1639_v155	Listeria_monocytogenes_1639_v138	0.945
Listeria_monocytogenes_1639_v154	Listeria_monocytogenes_1639_v138	0.9508
Listeria_monocytogenes_1639_v153	Listeria_monocytogenes_1639_v138	0.9458
Listeria_monocytogenes_1639_v152	Listeria_monocytogenes_1639_v138	0.9803
Listeria_monocytogenes_1639_v151	Listeria_monocytogenes_1639_v138	0.9803
Listeria_monocytogenes_1639_v150	Listeria_monocytogenes_1639_v138	0.9477
Listeria_monocytogenes_1639_v14	Listeria_monocytogenes_1639_v138	0.9391
Listeria_monocytogenes_1639_v149	Listeria_monocytogenes_1639_v138	0.9467
Listeria_monocytogenes_1639_v148	Listeria_monocytogenes_1639_v138	0.9792
Listeria_monocytogenes_1639_v147	Listeria_monocytogenes_1639_v138	0.9647
Listeria_monocytogenes_1639_v146	Listeria_monocytogenes_1639_v138	0.9558
Listeria_monocytogenes_1639_v145	Listeria_monocytogenes_1639_v138	0.9521
Listeria_monocytogenes_1639_v144	Listeria_monocytogenes_1639_v138	0.995
Listeria_monocytogenes_1639_v143	Listeria_monocytogenes_1639_v138	0.9551
Listeria_monocytogenes_1639_v142	Listeria_monocytogenes_1639_v138	0.9954
Listeria_monocytogenes_1639_v141	Listeria_monocytogenes_1639_v138	0.9745
Listeria_monocytogenes_1639_v140	Listeria_monocytogenes_1639_v138	0.9957
Listeria_monocytogenes_1639_v13	Listeria_monocytogenes_1639_v138	0.9325
Listeria_monocytogenes_1639_v139	Listeria_monocytogenes_1639_v138	0.9803
Listeria_monocytogenes_1639_v138	Listeria_monocytogenes_1639_v138	0.0653
Listeria_welshimeri_1643	Listeria_monocytogenes_1639_v139	0.8841
Listeria_welshimeri_serovar_6b_str_SLCC5334_386043	Listeria_monocytogenes_1639_v139	0.8842
Listeria_seeligeri_serovar_1_2b_str_SLCC3954_683837	Listeria_monocytogenes_1639_v139	0.8867
Listeria_seeligeri_FSL_S4-171_702452	Listeria_monocytogenes_1639_v139	0.832
Listeria_seeligeri_FSL_N1-067_702453	Listeria_monocytogenes_1639_v139	0.8305
Listeria_seeligeri_1640_v1	Listeria_monocytogenes_1639_v139	0.8948
Listeria_seeligeri_1640	Listeria_monocytogenes_1639_v139	0.8874
Listeria_monocytogenes_WSLC1042_1457188	Listeria_monocytogenes_1639_v139	0.9667
Listeria_monocytogenes_WSLC1001_1457187	Listeria_monocytogenes_1639_v139	0.9363
Listeria_monocytogenes_SLCC7179_879090	Listeria_monocytogenes_1639_v139	0.9407
Listeria_monocytogenes_SLCC5850_882096	Listeria_monocytogenes_1639_v139	0.9406
Listeria_monocytogenes_SLCC2755_932919	Listeria_monocytogenes_1639_v139	0.948
Listeria_monocytogenes_SLCC2540_879089	Listeria_monocytogenes_1639_v139	0.947
Listeria_monocytogenes_SLCC2479_882020	Listeria_monocytogenes_1639_v139	0.9318

Listeria_monocytogenes_SLCC2378_879088	Listeria_monocytogenes_1639_v139	0.9674
Listeria_monocytogenes_SLCC2376_882097	Listeria_monocytogenes_1639_v139	0.9434
Listeria_monocytogenes_SLCC2372_932920	Listeria_monocytogenes_1639_v139	0.9249
Listeria_monocytogenes_serotype_4b_str_LL195_1230340	Listeria_monocytogenes_1639_v139	0.9738
Listeria_monocytogenes_serotype_4b_str_LIS0087_1134669	Listeria_monocytogenes_1639_v139	0.9169
Listeria_monocytogenes_serotype_4b_str_F2365_265669	Listeria_monocytogenes_1639_v139	0.9738
Listeria_monocytogenes_serotype_4b_str_CLIP_80459_568819	Listeria_monocytogenes_1639_v139	0.9776
Listeria_monocytogenes_serotype_4b_str_81-0861_1196161	Listeria_monocytogenes_1639_v139	0.9564
Listeria_monocytogenes_serotype_4b_str_81-0592_1196160	Listeria_monocytogenes_1639_v139	0.9575
Listeria_monocytogenes_serotype_4b_str_81-0558_1196159	Listeria_monocytogenes_1639_v139	0.9579
Listeria_monocytogenes_serotype_4b_str_10-0809_1196162	Listeria_monocytogenes_1639_v139	0.9575
Listeria_monocytogenes_serotype_4b_str_02-6680_1196175	Listeria_monocytogenes_1639_v139	0.9823
Listeria_monocytogenes_serotype_4b_str_02-6679_1196174	Listeria_monocytogenes_1639_v139	0.9823
Listeria_monocytogenes_serotype_4b_str_02-1792_1196173	Listeria_monocytogenes_1639_v139	0.9575
Listeria_monocytogenes_serotype_4b_str_02-1289_1196172	Listeria_monocytogenes_1639_v139	0.9575
Listeria_monocytogenes_serotype_4b_str_02-1103_1196171	Listeria_monocytogenes_1639_v139	0.9575
Listeria_monocytogenes_serotype_3c_str_10-5027_1196195	Listeria_monocytogenes_1639_v139	0.9311
Listeria_monocytogenes_serotype_1_2c_str_10-5026_1196194	Listeria_monocytogenes_1639_v139	0.9318
Listeria_monocytogenes_serotype_1_2c_str_10-5025_1196193	Listeria_monocytogenes_1639_v139	0.9318
Listeria_monocytogenes_serotype_1_2b_str_10-0811_1196164	Listeria_monocytogenes_1639_v139	0.9427
Listeria_monocytogenes_serotype_1_2b_str_10-0810_1196163	Listeria_monocytogenes_1639_v139	0.9427
Listeria_monocytogenes_serotype_1_2a_str_99-6370_1196179	Listeria_monocytogenes_1639_v139	0.9178
Listeria_monocytogenes_serotype_1_2a_str_98-2035_1196178	Listeria_monocytogenes_1639_v139	0.9178
Listeria_monocytogenes_serotype_1_2a_str_95-0093_1196177	Listeria_monocytogenes_1639_v139	0.9233
Listeria_monocytogenes_serotype_1_2a_str_88-0478_1196176	Listeria_monocytogenes_1639_v139	0.9221
Listeria_monocytogenes_serotype_1_2a_str_10-5024_1196192	Listeria_monocytogenes_1639_v139	0.9233
Listeria_monocytogenes_serotype_1_2a_str_10-4758_1196168	Listeria_monocytogenes_1639_v139	0.9388
Listeria_monocytogenes_serotype_1_2a_str_10-4754_1196167	Listeria_monocytogenes_1639_v139	0.9392
Listeria_monocytogenes_serotype_1_2a_str_10-1321_1196191	Listeria_monocytogenes_1639_v139	0.9233
Listeria_monocytogenes_serotype_1_2a_str_10-1047_1196190	Listeria_monocytogenes_1639_v139	0.924
Listeria_monocytogenes_serotype_1_2a_str_10-1046_1196189	Listeria_monocytogenes_1639_v139	0.924
Listeria_monocytogenes_serotype_1_2a_str_10-0934_1196170	Listeria_monocytogenes_1639_v139	0.9496
Listeria_monocytogenes_serotype_1_2a_str_10-0933_1196169	Listeria_monocytogenes_1639_v139	0.9496
Listeria_monocytogenes_serotype_1_2a_str_10-0815_1196188	Listeria_monocytogenes_1639_v139	0.9192
Listeria_monocytogenes_serotype_1_2a_str_10-0814_1196187	Listeria_monocytogenes_1639_v139	0.9237
Listeria_monocytogenes_serotype_1_2a_str_10-0813_1196166	Listeria_monocytogenes_1639_v139	0.9339
Listeria_monocytogenes_serotype_1_2a_str_10-0812_1196165	Listeria_monocytogenes_1639_v139	0.9339
Listeria_monocytogenes_serotype_1_2a_str_08-7669_1196186	Listeria_monocytogenes_1639_v139	0.9327
Listeria_monocytogenes_serotype_1_2a_str_08-7374_1196185	Listeria_monocytogenes_1639_v139	0.9233
Listeria_monocytogenes_serotype_1_2a_str_08-6997_1196184	Listeria_monocytogenes_1639_v139	0.9192
Listeria_monocytogenes_serotype_1_2a_str_08-6569_1196183	Listeria_monocytogenes_1639_v139	0.9192
Listeria_monocytogenes_serotype_1_2a_str_08-6056_1196182	Listeria_monocytogenes_1639_v139	0.9192
Listeria_monocytogenes_serotype_1_2a_str_04-5457_1196181	Listeria_monocytogenes_1639_v139	0.9233
Listeria_monocytogenes_serotype_1_2a_str_02-5993_1196180	Listeria_monocytogenes_1639_v139	0.9233
Listeria_monocytogenes_R479a_1437838	Listeria_monocytogenes_1639_v139	0.923
Listeria_monocytogenes_N53-1_1234142	Listeria_monocytogenes_1639_v139	0.8952
Listeria_monocytogenes_M7_1030009	Listeria_monocytogenes_1639_v139	0.9054
Listeria_monocytogenes_LO28_393132	Listeria_monocytogenes_1639_v139	0.9304
Listeria_monocytogenes_L99_563174	Listeria_monocytogenes_1639_v139	0.9057
Listeria_monocytogenes_L312_882094	Listeria_monocytogenes_1639_v139	0.978
Listeria_monocytogenes_J1816_930781	Listeria_monocytogenes_1639_v139	0.9558
Listeria_monocytogenes_J1-220_930782	Listeria_monocytogenes_1639_v139	0.953
Listeria_monocytogenes_J0161_393130	Listeria_monocytogenes_1639_v139	0.9301
Listeria_monocytogenes_HCC23_552536	Listeria_monocytogenes_1639_v139	0.9054
Listeria_monocytogenes_FSL_R2-561_393126	Listeria_monocytogenes_1639_v139	0.9304
Listeria_monocytogenes_FSL_J1-208_393119	Listeria_monocytogenes_1639_v139	0.9214
Listeria_monocytogenes_FSL_J1-175_393118	Listeria_monocytogenes_1639_v139	0.9567
Listeria_monocytogenes_Finland_1998_393127	Listeria_monocytogenes_1639_v139	0.9511
Listeria_monocytogenes_EGD-e_169963_v1	Listeria_monocytogenes_1639_v139	0.9396
Listeria_monocytogenes_EGD-e_169963	Listeria_monocytogenes_1639_v139	0.94
Listeria_monocytogenes_EGD_1334565	Listeria_monocytogenes_1639_v139	0.9414
Listeria_monocytogenes_CFSAN002349_1299895	Listeria_monocytogenes_1639_v139	0.98
Listeria_monocytogenes_ATCC_19117_882095_v1	Listeria_monocytogenes_1639_v139	0.9735
Listeria_monocytogenes_ATCC_19117_882095	Listeria_monocytogenes_1639_v139	0.9735
Listeria_monocytogenes_6179_1288295	Listeria_monocytogenes_1639_v139	0.9124
Listeria_monocytogenes_1639_v9	Listeria_monocytogenes_1639_v139	0.9279
Listeria_monocytogenes_1639_v99	Listeria_monocytogenes_1639_v139	0.9573
Listeria_monocytogenes_1639_v98	Listeria_monocytogenes_1639_v139	0.9277
Listeria_monocytogenes_1639_v97	Listeria_monocytogenes_1639_v139	0.9627
Listeria_monocytogenes_1639_v96	Listeria_monocytogenes_1639_v139	0.9562
Listeria_monocytogenes_1639_v95	Listeria_monocytogenes_1639_v139	0.9562
Listeria_monocytogenes_1639_v94	Listeria_monocytogenes_1639_v139	0.957
Listeria_monocytogenes_1639_v93	Listeria_monocytogenes_1639_v139	0.9251
Listeria_monocytogenes_1639_v92	Listeria_monocytogenes_1639_v139	0.957
Listeria_monocytogenes_1639_v91	Listeria_monocytogenes_1639_v139	0.9546
Listeria_monocytogenes_1639_v90	Listeria_monocytogenes_1639_v139	0.9277
Listeria_monocytogenes_1639_v8	Listeria_monocytogenes_1639_v139	0.9123
Listeria_monocytogenes_1639_v89	Listeria_monocytogenes_1639_v139	0.9633
Listeria_monocytogenes_1639_v88	Listeria_monocytogenes_1639_v139	0.9302
Listeria_monocytogenes_1639_v87	Listeria_monocytogenes_1639_v139	0.9293
Listeria_monocytogenes_1639_v86	Listeria_monocytogenes_1639_v139	0.9289
Listeria_monocytogenes_1639_v85	Listeria_monocytogenes_1639_v139	0.9557
Listeria_monocytogenes_1639_v84	Listeria_monocytogenes_1639_v139	0.9346
Listeria_monocytogenes_1639_v83	Listeria_monocytogenes_1639_v139	0.962
Listeria_monocytogenes_1639_v82	Listeria_monocytogenes_1639_v139	0.9613
Listeria_monocytogenes_1639_v81	Listeria_monocytogenes_1639_v139	0.9313
Listeria_monocytogenes_1639_v80	Listeria_monocytogenes_1639_v139	0.9502
Listeria_monocytogenes_1639_v7	Listeria_monocytogenes_1639_v139	0.9123
Listeria_monocytogenes_1639_v79	Listeria_monocytogenes_1639_v139	0.9295
Listeria_monocytogenes_1639_v78	Listeria_monocytogenes_1639_v139	0.9306
Listeria_monocytogenes_1639_v77	Listeria_monocytogenes_1639_v139	0.9276

Listeria_monocytogenes_1639_v76	Listeria_monocytogenes_1639_v139	0.9228
Listeria_monocytogenes_1639_v75	Listeria_monocytogenes_1639_v139	0.9057
Listeria_monocytogenes_1639_v74	Listeria_monocytogenes_1639_v139	0.9282
Listeria_monocytogenes_1639_v73	Listeria_monocytogenes_1639_v139	0.9259
Listeria_monocytogenes_1639_v72	Listeria_monocytogenes_1639_v139	0.9616
Listeria_monocytogenes_1639_v71	Listeria_monocytogenes_1639_v139	0.9159
Listeria_monocytogenes_1639_v70	Listeria_monocytogenes_1639_v139	0.928
Listeria_monocytogenes_1639_v6	Listeria_monocytogenes_1639_v139	0.912
Listeria_monocytogenes_1639_v69	Listeria_monocytogenes_1639_v139	0.9575
Listeria_monocytogenes_1639_v68	Listeria_monocytogenes_1639_v139	0.9335
Listeria_monocytogenes_1639_v67	Listeria_monocytogenes_1639_v139	0.9484
Listeria_monocytogenes_1639_v66	Listeria_monocytogenes_1639_v139	0.9339
Listeria_monocytogenes_1639_v65	Listeria_monocytogenes_1639_v139	0.9264
Listeria_monocytogenes_1639_v64	Listeria_monocytogenes_1639_v139	0.9284
Listeria_monocytogenes_1639_v63	Listeria_monocytogenes_1639_v139	0.9438
Listeria_monocytogenes_1639_v62	Listeria_monocytogenes_1639_v139	0.9271
Listeria_monocytogenes_1639_v61	Listeria_monocytogenes_1639_v139	0.926
Listeria_monocytogenes_1639_v60	Listeria_monocytogenes_1639_v139	0.932
Listeria_monocytogenes_1639_v5	Listeria_monocytogenes_1639_v139	0.9078
Listeria_monocytogenes_1639_v59	Listeria_monocytogenes_1639_v139	0.9624
Listeria_monocytogenes_1639_v58	Listeria_monocytogenes_1639_v139	0.9666
Listeria_monocytogenes_1639_v57	Listeria_monocytogenes_1639_v139	0.9526
Listeria_monocytogenes_1639_v56	Listeria_monocytogenes_1639_v139	0.9624
Listeria_monocytogenes_1639_v55	Listeria_monocytogenes_1639_v139	0.9491
Listeria_monocytogenes_1639_v54	Listeria_monocytogenes_1639_v139	0.9265
Listeria_monocytogenes_1639_v53	Listeria_monocytogenes_1639_v139	0.932
Listeria_monocytogenes_1639_v52	Listeria_monocytogenes_1639_v139	0.9591
Listeria_monocytogenes_1639_v51	Listeria_monocytogenes_1639_v139	0.9562
Listeria_monocytogenes_1639_v50	Listeria_monocytogenes_1639_v139	0.9229
Listeria_monocytogenes_1639_v4	Listeria_monocytogenes_1639_v139	0.9212
Listeria_monocytogenes_1639_v49	Listeria_monocytogenes_1639_v139	0.9568
Listeria_monocytogenes_1639_v48	Listeria_monocytogenes_1639_v139	0.9262
Listeria_monocytogenes_1639_v47	Listeria_monocytogenes_1639_v139	0.9229
Listeria_monocytogenes_1639_v46	Listeria_monocytogenes_1639_v139	0.9328
Listeria_monocytogenes_1639_v45	Listeria_monocytogenes_1639_v139	0.9144
Listeria_monocytogenes_1639_v44	Listeria_monocytogenes_1639_v139	0.9233
Listeria_monocytogenes_1639_v43	Listeria_monocytogenes_1639_v139	0.9233
Listeria_monocytogenes_1639_v42	Listeria_monocytogenes_1639_v139	0.9162
Listeria_monocytogenes_1639_v41	Listeria_monocytogenes_1639_v139	0.9035
Listeria_monocytogenes_1639_v40	Listeria_monocytogenes_1639_v139	0.9308
Listeria_monocytogenes_1639_v3	Listeria_monocytogenes_1639_v139	0.9215
Listeria_monocytogenes_1639_v39	Listeria_monocytogenes_1639_v139	0.9437
Listeria_monocytogenes_1639_v38	Listeria_monocytogenes_1639_v139	0.9276
Listeria_monocytogenes_1639_v37	Listeria_monocytogenes_1639_v139	0.9235
Listeria_monocytogenes_1639_v36	Listeria_monocytogenes_1639_v139	0.9203
Listeria_monocytogenes_1639_v35	Listeria_monocytogenes_1639_v139	0.9315
Listeria_monocytogenes_1639_v34	Listeria_monocytogenes_1639_v139	0.9255
Listeria_monocytogenes_1639_v33	Listeria_monocytogenes_1639_v139	0.9427
Listeria_monocytogenes_1639_v32	Listeria_monocytogenes_1639_v139	0.9225
Listeria_monocytogenes_1639_v31	Listeria_monocytogenes_1639_v139	0.9258
Listeria_monocytogenes_1639_v30	Listeria_monocytogenes_1639_v139	0.9452
Listeria_monocytogenes_1639_v2	Listeria_monocytogenes_1639_v139	0.9053
Listeria_monocytogenes_1639_v29	Listeria_monocytogenes_1639_v139	0.9176
Listeria_monocytogenes_1639_v28	Listeria_monocytogenes_1639_v139	0.9217
Listeria_monocytogenes_1639_v27	Listeria_monocytogenes_1639_v139	0.9129
Listeria_monocytogenes_1639_v26	Listeria_monocytogenes_1639_v139	0.9126
Listeria_monocytogenes_1639_v25	Listeria_monocytogenes_1639_v139	0.9126
Listeria_monocytogenes_1639_v24	Listeria_monocytogenes_1639_v139	0.953
Listeria_monocytogenes_1639_v23	Listeria_monocytogenes_1639_v139	0.9526
Listeria_monocytogenes_1639_v22	Listeria_monocytogenes_1639_v139	0.9438
Listeria_monocytogenes_1639_v21	Listeria_monocytogenes_1639_v139	0.9526
Listeria_monocytogenes_1639_v20	Listeria_monocytogenes_1639_v139	0.9355
Listeria_monocytogenes_1639_v1	Listeria_monocytogenes_1639_v139	0.9061
Listeria_monocytogenes_1639_v19	Listeria_monocytogenes_1639_v139	0.9352
Listeria_monocytogenes_1639_v198	Listeria_monocytogenes_1639_v139	0.8971
Listeria_monocytogenes_1639_v197	Listeria_monocytogenes_1639_v139	0.9272
Listeria_monocytogenes_1639_v196	Listeria_monocytogenes_1639_v139	0.924
Listeria_monocytogenes_1639_v195	Listeria_monocytogenes_1639_v139	0.9445
Listeria_monocytogenes_1639_v194	Listeria_monocytogenes_1639_v139	0.9438
Listeria_monocytogenes_1639_v193	Listeria_monocytogenes_1639_v139	0.9438
Listeria_monocytogenes_1639_v192	Listeria_monocytogenes_1639_v139	0.9128
Listeria_monocytogenes_1639_v191	Listeria_monocytogenes_1639_v139	0.9518
Listeria_monocytogenes_1639_v190	Listeria_monocytogenes_1639_v139	0.953
Listeria_monocytogenes_1639_v18	Listeria_monocytogenes_1639_v139	0.9466
Listeria_monocytogenes_1639_v189	Listeria_monocytogenes_1639_v139	0.9523
Listeria_monocytogenes_1639_v188	Listeria_monocytogenes_1639_v139	0.9522
Listeria_monocytogenes_1639_v187	Listeria_monocytogenes_1639_v139	0.953
Listeria_monocytogenes_1639_v186	Listeria_monocytogenes_1639_v139	0.9534
Listeria_monocytogenes_1639_v185	Listeria_monocytogenes_1639_v139	0.9534
Listeria_monocytogenes_1639_v184	Listeria_monocytogenes_1639_v139	0.9526
Listeria_monocytogenes_1639_v183	Listeria_monocytogenes_1639_v139	0.9468
Listeria_monocytogenes_1639_v182	Listeria_monocytogenes_1639_v139	0.949
Listeria_monocytogenes_1639_v181	Listeria_monocytogenes_1639_v139	0.9335
Listeria_monocytogenes_1639_v180	Listeria_monocytogenes_1639_v139	0.9335
Listeria_monocytogenes_1639_v17	Listeria_monocytogenes_1639_v139	0.947
Listeria_monocytogenes_1639_v179	Listeria_monocytogenes_1639_v139	0.9331
Listeria_monocytogenes_1639_v178	Listeria_monocytogenes_1639_v139	0.9511
Listeria_monocytogenes_1639_v177	Listeria_monocytogenes_1639_v139	0.9761
Listeria_monocytogenes_1639_v176	Listeria_monocytogenes_1639_v139	0.9408
Listeria_monocytogenes_1639_v175	Listeria_monocytogenes_1639_v139	0.9408
Listeria_monocytogenes_1639_v174	Listeria_monocytogenes_1639_v139	0.9512

Listeria_monocytogenes_1639_v173	Listeria_monocytogenes_1639_v139	0.9667
Listeria_monocytogenes_1639_v172	Listeria_monocytogenes_1639_v139	0.9441
Listeria_monocytogenes_1639_v171	Listeria_monocytogenes_1639_v139	0.9389
Listeria_monocytogenes_1639_v170	Listeria_monocytogenes_1639_v139	0.9468
Listeria_monocytogenes_1639_v16	Listeria_monocytogenes_1639_v139	0.91
Listeria_monocytogenes_1639_v169	Listeria_monocytogenes_1639_v139	0.9453
Listeria_monocytogenes_1639_v168	Listeria_monocytogenes_1639_v139	0.9489
Listeria_monocytogenes_1639_v167	Listeria_monocytogenes_1639_v139	0.9352
Listeria_monocytogenes_1639_v166	Listeria_monocytogenes_1639_v139	0.9493
Listeria_monocytogenes_1639_v165	Listeria_monocytogenes_1639_v139	0.945
Listeria_monocytogenes_1639_v164	Listeria_monocytogenes_1639_v139	0.9733
Listeria_monocytogenes_1639_v163	Listeria_monocytogenes_1639_v139	0.9672
Listeria_monocytogenes_1639_v162	Listeria_monocytogenes_1639_v139	0.9733
Listeria_monocytogenes_1639_v161	Listeria_monocytogenes_1639_v139	0.9745
Listeria_monocytogenes_1639_v160	Listeria_monocytogenes_1639_v139	0.9741
Listeria_monocytogenes_1639_v15	Listeria_monocytogenes_1639_v139	0.9273
Listeria_monocytogenes_1639_v159	Listeria_monocytogenes_1639_v139	0.9765
Listeria_monocytogenes_1639_v158	Listeria_monocytogenes_1639_v139	0.9742
Listeria_monocytogenes_1639_v157	Listeria_monocytogenes_1639_v139	0.9738
Listeria_monocytogenes_1639_v156	Listeria_monocytogenes_1639_v139	0.9303
Listeria_monocytogenes_1639_v155	Listeria_monocytogenes_1639_v139	0.9401
Listeria_monocytogenes_1639_v154	Listeria_monocytogenes_1639_v139	0.9421
Listeria_monocytogenes_1639_v153	Listeria_monocytogenes_1639_v139	0.9408
Listeria_monocytogenes_1639_v152	Listeria_monocytogenes_1639_v139	0.9765
Listeria_monocytogenes_1639_v151	Listeria_monocytogenes_1639_v139	0.9765
Listeria_monocytogenes_1639_v150	Listeria_monocytogenes_1639_v139	0.9431
Listeria_monocytogenes_1639_v14	Listeria_monocytogenes_1639_v139	0.9339
Listeria_monocytogenes_1639_v149	Listeria_monocytogenes_1639_v139	0.9417
Listeria_monocytogenes_1639_v148	Listeria_monocytogenes_1639_v139	0.9754
Listeria_monocytogenes_1639_v147	Listeria_monocytogenes_1639_v139	0.9609
Listeria_monocytogenes_1639_v146	Listeria_monocytogenes_1639_v139	0.9497
Listeria_monocytogenes_1639_v145	Listeria_monocytogenes_1639_v139	0.9452
Listeria_monocytogenes_1639_v144	Listeria_monocytogenes_1639_v139	0.9772
Listeria_monocytogenes_1639_v143	Listeria_monocytogenes_1639_v139	0.949
Listeria_monocytogenes_1639_v142	Listeria_monocytogenes_1639_v139	0.9776
Listeria_monocytogenes_1639_v141	Listeria_monocytogenes_1639_v139	0.9675
Listeria_monocytogenes_1639_v140	Listeria_monocytogenes_1639_v139	0.9784
Listeria_monocytogenes_1639_v13	Listeria_monocytogenes_1639_v139	0.9273
Listeria_monocytogenes_1639_v139	Listeria_monocytogenes_1639_v139	0.0683
Listeria_welshimeri_1643	Listeria_monocytogenes_1639_v13	0.8541
Listeria_welshimeri_serovar_6b_str_SLCC5334_386043	Listeria_monocytogenes_1639_v13	0.8534
Listeria_seeligeri_serovar_1_2b_str_SLCC3954_683837	Listeria_monocytogenes_1639_v13	0.8604
Listeria_seeligeri_FSL_S4-171_702452	Listeria_monocytogenes_1639_v13	0.8014
Listeria_seeligeri_FSL_N1-067_702453	Listeria_monocytogenes_1639_v13	0.819
Listeria_seeligeri_1640_v1	Listeria_monocytogenes_1639_v13	0.8639
Listeria_seeligeri_1640	Listeria_monocytogenes_1639_v13	0.8804
Listeria_monocytogenes_WSLC1042_1457188	Listeria_monocytogenes_1639_v13	0.9231
Listeria_monocytogenes_WSLC1001_1457187	Listeria_monocytogenes_1639_v13	0.9226
Listeria_monocytogenes_SLCC7179_879090	Listeria_monocytogenes_1639_v13	0.9249
Listeria_monocytogenes_SLCC5850_882096	Listeria_monocytogenes_1639_v13	0.9241
Listeria_monocytogenes_SLCC2755_932919	Listeria_monocytogenes_1639_v13	0.9313
Listeria_monocytogenes_SLCC2540_879089	Listeria_monocytogenes_1639_v13	0.9409
Listeria_monocytogenes_SLCC2479_882020	Listeria_monocytogenes_1639_v13	0.9232
Listeria_monocytogenes_SLCC2378_879088	Listeria_monocytogenes_1639_v13	0.9244
Listeria_monocytogenes_SLCC2376_882097	Listeria_monocytogenes_1639_v13	0.9115
Listeria_monocytogenes_SLCC2372_932920	Listeria_monocytogenes_1639_v13	0.9306
Listeria_monocytogenes_serotype_4b_str_LL195_1230340	Listeria_monocytogenes_1639_v13	0.9299
Listeria_monocytogenes_serotype_4b_str_LIS0087_1134669	Listeria_monocytogenes_1639_v13	0.9134
Listeria_monocytogenes_serotype_4b_str_F2365_265669	Listeria_monocytogenes_1639_v13	0.9299
Listeria_monocytogenes_serotype_4b_str_CLIP_80459_568819	Listeria_monocytogenes_1639_v13	0.9309
Listeria_monocytogenes_serotype_4b_str_81-0861_1196161	Listeria_monocytogenes_1639_v13	0.9278
Listeria_monocytogenes_serotype_4b_str_81-0592_1196160	Listeria_monocytogenes_1639_v13	0.9289
Listeria_monocytogenes_serotype_4b_str_81-0558_1196159	Listeria_monocytogenes_1639_v13	0.9288
Listeria_monocytogenes_serotype_4b_str_10-0809_1196162	Listeria_monocytogenes_1639_v13	0.9292
Listeria_monocytogenes_serotype_4b_str_02-6680_1196175	Listeria_monocytogenes_1639_v13	0.9263
Listeria_monocytogenes_serotype_4b_str_02-6679_1196174	Listeria_monocytogenes_1639_v13	0.9263
Listeria_monocytogenes_serotype_4b_str_02-1792_1196173	Listeria_monocytogenes_1639_v13	0.9314
Listeria_monocytogenes_serotype_4b_str_02-1289_1196172	Listeria_monocytogenes_1639_v13	0.9314
Listeria_monocytogenes_serotype_4b_str_02-1103_1196171	Listeria_monocytogenes_1639_v13	0.9314
Listeria_monocytogenes_serotype_3c_str_10-5027_1196195	Listeria_monocytogenes_1639_v13	0.9279
Listeria_monocytogenes_serotype_1_2c_str_10-5026_1196194	Listeria_monocytogenes_1639_v13	0.9286
Listeria_monocytogenes_serotype_1_2c_str_10-5025_1196193	Listeria_monocytogenes_1639_v13	0.9286
Listeria_monocytogenes_serotype_1_2b_str_10-0811_1196164	Listeria_monocytogenes_1639_v13	0.9645
Listeria_monocytogenes_serotype_1_2b_str_10-0810_1196163	Listeria_monocytogenes_1639_v13	0.9645
Listeria_monocytogenes_serotype_1_2a_str_99-6370_1196179	Listeria_monocytogenes_1639_v13	0.9155
Listeria_monocytogenes_serotype_1_2a_str_98-2035_1196178	Listeria_monocytogenes_1639_v13	0.9155
Listeria_monocytogenes_serotype_1_2a_str_95-0093_1196177	Listeria_monocytogenes_1639_v13	0.9193
Listeria_monocytogenes_serotype_1_2a_str_88-0478_1196176	Listeria_monocytogenes_1639_v13	0.9164
Listeria_monocytogenes_serotype_1_2a_str_10-5024_1196192	Listeria_monocytogenes_1639_v13	0.9197
Listeria_monocytogenes_serotype_1_2a_str_10-4758_1196168	Listeria_monocytogenes_1639_v13	0.9264
Listeria_monocytogenes_serotype_1_2a_str_10-4754_1196167	Listeria_monocytogenes_1639_v13	0.9264
Listeria_monocytogenes_serotype_1_2a_str_10-1321_1196191	Listeria_monocytogenes_1639_v13	0.9197
Listeria_monocytogenes_serotype_1_2a_str_10-1047_1196190	Listeria_monocytogenes_1639_v13	0.9185
Listeria_monocytogenes_serotype_1_2a_str_10-1046_1196189	Listeria_monocytogenes_1639_v13	0.9185
Listeria_monocytogenes_serotype_1_2a_str_10-0934_1196170	Listeria_monocytogenes_1639_v13	0.9203
Listeria_monocytogenes_serotype_1_2a_str_10-0933_1196169	Listeria_monocytogenes_1639_v13	0.9203
Listeria_monocytogenes_serotype_1_2a_str_10-0815_1196188	Listeria_monocytogenes_1639_v13	0.9161
Listeria_monocytogenes_serotype_1_2a_str_10-0814_1196187	Listeria_monocytogenes_1639_v13	0.9197
Listeria_monocytogenes_serotype_1_2a_str_10-0813_1196166	Listeria_monocytogenes_1639_v13	0.9306
Listeria_monocytogenes_serotype_1_2a_str_10-0812_1196165	Listeria_monocytogenes_1639_v13	0.9306
Listeria_monocytogenes_serotype_1_2a_str_08-7669_1196186	Listeria_monocytogenes_1639_v13	0.9126

Listeria_monocytogenes_serotype_1_2a_str_08-7374_1196185	Listeria_monocytogenes_1639_v13	0.9197
Listeria_monocytogenes_serotype_1_2a_str_08-6997_1196184	Listeria_monocytogenes_1639_v13	0.9161
Listeria_monocytogenes_serotype_1_2a_str_08-6569_1196183	Listeria_monocytogenes_1639_v13	0.9161
Listeria_monocytogenes_serotype_1_2a_str_08-6056_1196182	Listeria_monocytogenes_1639_v13	0.9161
Listeria_monocytogenes_serotype_1_2a_str_04-5457_1196181	Listeria_monocytogenes_1639_v13	0.9197
Listeria_monocytogenes_serotype_1_2a_str_02-5993_1196180	Listeria_monocytogenes_1639_v13	0.9193
Listeria_monocytogenes_R479a_1437838	Listeria_monocytogenes_1639_v13	0.9248
Listeria_monocytogenes_N53-1_1234142	Listeria_monocytogenes_1639_v13	0.8774
Listeria_monocytogenes_M7_1030009	Listeria_monocytogenes_1639_v13	0.9017
Listeria_monocytogenes_LO28_393132	Listeria_monocytogenes_1639_v13	0.9309
Listeria_monocytogenes_L99_563174	Listeria_monocytogenes_1639_v13	0.9024
Listeria_monocytogenes_L312_882094	Listeria_monocytogenes_1639_v13	0.9313
Listeria_monocytogenes_J1816_930781	Listeria_monocytogenes_1639_v13	0.9218
Listeria_monocytogenes_J1-220_930782	Listeria_monocytogenes_1639_v13	0.9298
Listeria_monocytogenes_J0161_393130	Listeria_monocytogenes_1639_v13	0.9229
Listeria_monocytogenes_HCC23_552536	Listeria_monocytogenes_1639_v13	0.9017
Listeria_monocytogenes_FSL_R2-561_393126	Listeria_monocytogenes_1639_v13	0.9232
Listeria_monocytogenes_FSL_J1-208_393119	Listeria_monocytogenes_1639_v13	0.8821
Listeria_monocytogenes_FSL_J1-175_393118	Listeria_monocytogenes_1639_v13	0.9418
Listeria_monocytogenes_Finland_1998_393127	Listeria_monocytogenes_1639_v13	0.9214
Listeria_monocytogenes_EGD-e_169963_v1	Listeria_monocytogenes_1639_v13	0.9266
Listeria_monocytogenes_EGD-e_169963	Listeria_monocytogenes_1639_v13	0.9269
Listeria_monocytogenes_EGD_1334565	Listeria_monocytogenes_1639_v13	0.9248
Listeria_monocytogenes_CFSAN002349_1299895	Listeria_monocytogenes_1639_v13	0.9319
Listeria_monocytogenes_ATCC_19117_882095_v1	Listeria_monocytogenes_1639_v13	0.9254
Listeria_monocytogenes_ATCC_19117_882095	Listeria_monocytogenes_1639_v13	0.9253
Listeria_monocytogenes_6179_1288295	Listeria_monocytogenes_1639_v13	0.9254
Listeria_monocytogenes_1639_v9	Listeria_monocytogenes_1639_v13	0.9538
Listeria_monocytogenes_1639_v99	Listeria_monocytogenes_1639_v13	0.9306
Listeria_monocytogenes_1639_v98	Listeria_monocytogenes_1639_v13	0.9202
Listeria_monocytogenes_1639_v97	Listeria_monocytogenes_1639_v13	0.9231
Listeria_monocytogenes_1639_v96	Listeria_monocytogenes_1639_v13	0.931
Listeria_monocytogenes_1639_v95	Listeria_monocytogenes_1639_v13	0.931
Listeria_monocytogenes_1639_v94	Listeria_monocytogenes_1639_v13	0.931
Listeria_monocytogenes_1639_v93	Listeria_monocytogenes_1639_v13	0.9258
Listeria_monocytogenes_1639_v92	Listeria_monocytogenes_1639_v13	0.9313
Listeria_monocytogenes_1639_v91	Listeria_monocytogenes_1639_v13	0.9341
Listeria_monocytogenes_1639_v90	Listeria_monocytogenes_1639_v13	0.9202
Listeria_monocytogenes_1639_v8	Listeria_monocytogenes_1639_v13	0.9149
Listeria_monocytogenes_1639_v89	Listeria_monocytogenes_1639_v13	0.9357
Listeria_monocytogenes_1639_v88	Listeria_monocytogenes_1639_v13	0.923
Listeria_monocytogenes_1639_v87	Listeria_monocytogenes_1639_v13	0.9269
Listeria_monocytogenes_1639_v86	Listeria_monocytogenes_1639_v13	0.9262
Listeria_monocytogenes_1639_v85	Listeria_monocytogenes_1639_v13	0.929
Listeria_monocytogenes_1639_v84	Listeria_monocytogenes_1639_v13	0.9235
Listeria_monocytogenes_1639_v83	Listeria_monocytogenes_1639_v13	0.932
Listeria_monocytogenes_1639_v82	Listeria_monocytogenes_1639_v13	0.9317
Listeria_monocytogenes_1639_v81	Listeria_monocytogenes_1639_v13	0.9233
Listeria_monocytogenes_1639_v80	Listeria_monocytogenes_1639_v13	0.9645
Listeria_monocytogenes_1639_v7	Listeria_monocytogenes_1639_v13	0.9149
Listeria_monocytogenes_1639_v79	Listeria_monocytogenes_1639_v13	0.9198
Listeria_monocytogenes_1639_v78	Listeria_monocytogenes_1639_v13	0.9241
Listeria_monocytogenes_1639_v77	Listeria_monocytogenes_1639_v13	0.9106
Listeria_monocytogenes_1639_v76	Listeria_monocytogenes_1639_v13	0.9196
Listeria_monocytogenes_1639_v75	Listeria_monocytogenes_1639_v13	0.9024
Listeria_monocytogenes_1639_v74	Listeria_monocytogenes_1639_v13	0.9149
Listeria_monocytogenes_1639_v73	Listeria_monocytogenes_1639_v13	0.9367
Listeria_monocytogenes_1639_v72	Listeria_monocytogenes_1639_v13	0.9261
Listeria_monocytogenes_1639_v71	Listeria_monocytogenes_1639_v13	0.913
Listeria_monocytogenes_1639_v70	Listeria_monocytogenes_1639_v13	0.9271
Listeria_monocytogenes_1639_v6	Listeria_monocytogenes_1639_v13	0.9142
Listeria_monocytogenes_1639_v69	Listeria_monocytogenes_1639_v13	0.9292
Listeria_monocytogenes_1639_v68	Listeria_monocytogenes_1639_v13	0.9196
Listeria_monocytogenes_1639_v67	Listeria_monocytogenes_1639_v13	0.9394
Listeria_monocytogenes_1639_v66	Listeria_monocytogenes_1639_v13	0.9236
Listeria_monocytogenes_1639_v65	Listeria_monocytogenes_1639_v13	0.9307
Listeria_monocytogenes_1639_v64	Listeria_monocytogenes_1639_v13	0.9179
Listeria_monocytogenes_1639_v63	Listeria_monocytogenes_1639_v13	0.9593
Listeria_monocytogenes_1639_v62	Listeria_monocytogenes_1639_v13	0.9218
Listeria_monocytogenes_1639_v61	Listeria_monocytogenes_1639_v13	0.9212
Listeria_monocytogenes_1639_v60	Listeria_monocytogenes_1639_v13	0.916
Listeria_monocytogenes_1639_v5	Listeria_monocytogenes_1639_v13	0.9559
Listeria_monocytogenes_1639_v59	Listeria_monocytogenes_1639_v13	0.9269
Listeria_monocytogenes_1639_v58	Listeria_monocytogenes_1639_v13	0.9313
Listeria_monocytogenes_1639_v57	Listeria_monocytogenes_1639_v13	0.9378
Listeria_monocytogenes_1639_v56	Listeria_monocytogenes_1639_v13	0.9225
Listeria_monocytogenes_1639_v55	Listeria_monocytogenes_1639_v13	0.9319
Listeria_monocytogenes_1639_v54	Listeria_monocytogenes_1639_v13	0.8974
Listeria_monocytogenes_1639_v53	Listeria_monocytogenes_1639_v13	0.9083
Listeria_monocytogenes_1639_v52	Listeria_monocytogenes_1639_v13	0.9341
Listeria_monocytogenes_1639_v51	Listeria_monocytogenes_1639_v13	0.9335
Listeria_monocytogenes_1639_v50	Listeria_monocytogenes_1639_v13	0.9186
Listeria_monocytogenes_1639_v4	Listeria_monocytogenes_1639_v13	0.9691
Listeria_monocytogenes_1639_v49	Listeria_monocytogenes_1639_v13	0.919
Listeria_monocytogenes_1639_v48	Listeria_monocytogenes_1639_v13	0.9251
Listeria_monocytogenes_1639_v47	Listeria_monocytogenes_1639_v13	0.9175
Listeria_monocytogenes_1639_v46	Listeria_monocytogenes_1639_v13	0.912
Listeria_monocytogenes_1639_v45	Listeria_monocytogenes_1639_v13	0.9173
Listeria_monocytogenes_1639_v44	Listeria_monocytogenes_1639_v13	0.9197
Listeria_monocytogenes_1639_v43	Listeria_monocytogenes_1639_v13	0.9197
Listeria_monocytogenes_1639_v42	Listeria_monocytogenes_1639_v13	0.9014

Listeria_monocytogenes_1639_v41	Listeria_monocytogenes_1639_v13	0.9125
Listeria_monocytogenes_1639_v40	Listeria_monocytogenes_1639_v13	0.9262
Listeria_monocytogenes_1639_v3	Listeria_monocytogenes_1639_v13	0.9699
Listeria_monocytogenes_1639_v39	Listeria_monocytogenes_1639_v13	0.9371
Listeria_monocytogenes_1639_v38	Listeria_monocytogenes_1639_v13	0.9155
Listeria_monocytogenes_1639_v37	Listeria_monocytogenes_1639_v13	0.9314
Listeria_monocytogenes_1639_v36	Listeria_monocytogenes_1639_v13	0.9207
Listeria_monocytogenes_1639_v35	Listeria_monocytogenes_1639_v13	0.9206
Listeria_monocytogenes_1639_v34	Listeria_monocytogenes_1639_v13	0.9167
Listeria_monocytogenes_1639_v33	Listeria_monocytogenes_1639_v13	0.9645
Listeria_monocytogenes_1639_v32	Listeria_monocytogenes_1639_v13	0.9254
Listeria_monocytogenes_1639_v31	Listeria_monocytogenes_1639_v13	0.917
Listeria_monocytogenes_1639_v30	Listeria_monocytogenes_1639_v13	0.9444
Listeria_monocytogenes_1639_v2	Listeria_monocytogenes_1639_v13	0.9315
Listeria_monocytogenes_1639_v29	Listeria_monocytogenes_1639_v13	0.9225
Listeria_monocytogenes_1639_v28	Listeria_monocytogenes_1639_v13	0.9153
Listeria_monocytogenes_1639_v27	Listeria_monocytogenes_1639_v13	0.9318
Listeria_monocytogenes_1639_v26	Listeria_monocytogenes_1639_v13	0.9329
Listeria_monocytogenes_1639_v25	Listeria_monocytogenes_1639_v13	0.933
Listeria_monocytogenes_1639_v24	Listeria_monocytogenes_1639_v13	0.9308
Listeria_monocytogenes_1639_v23	Listeria_monocytogenes_1639_v13	0.9305
Listeria_monocytogenes_1639_v22	Listeria_monocytogenes_1639_v13	0.9637
Listeria_monocytogenes_1639_v21	Listeria_monocytogenes_1639_v13	0.932
Listeria_monocytogenes_1639_v20	Listeria_monocytogenes_1639_v13	0.9445
Listeria_monocytogenes_1639_v1	Listeria_monocytogenes_1639_v13	0.9206
Listeria_monocytogenes_1639_v19	Listeria_monocytogenes_1639_v13	0.9438
Listeria_monocytogenes_1639_v198	Listeria_monocytogenes_1639_v13	0.8792
Listeria_monocytogenes_1639_v197	Listeria_monocytogenes_1639_v13	0.915
Listeria_monocytogenes_1639_v196	Listeria_monocytogenes_1639_v13	0.9079
Listeria_monocytogenes_1639_v195	Listeria_monocytogenes_1639_v13	0.9125
Listeria_monocytogenes_1639_v194	Listeria_monocytogenes_1639_v13	0.9126
Listeria_monocytogenes_1639_v193	Listeria_monocytogenes_1639_v13	0.9123
Listeria_monocytogenes_1639_v192	Listeria_monocytogenes_1639_v13	0.8775
Listeria_monocytogenes_1639_v191	Listeria_monocytogenes_1639_v13	0.9197
Listeria_monocytogenes_1639_v190	Listeria_monocytogenes_1639_v13	0.9209
Listeria_monocytogenes_1639_v18	Listeria_monocytogenes_1639_v13	0.9403
Listeria_monocytogenes_1639_v189	Listeria_monocytogenes_1639_v13	0.9198
Listeria_monocytogenes_1639_v188	Listeria_monocytogenes_1639_v13	0.9219
Listeria_monocytogenes_1639_v187	Listeria_monocytogenes_1639_v13	0.9205
Listeria_monocytogenes_1639_v186	Listeria_monocytogenes_1639_v13	0.9209
Listeria_monocytogenes_1639_v185	Listeria_monocytogenes_1639_v13	0.9209
Listeria_monocytogenes_1639_v184	Listeria_monocytogenes_1639_v13	0.9201
Listeria_monocytogenes_1639_v183	Listeria_monocytogenes_1639_v13	0.9153
Listeria_monocytogenes_1639_v182	Listeria_monocytogenes_1639_v13	0.9174
Listeria_monocytogenes_1639_v181	Listeria_monocytogenes_1639_v13	0.8936
Listeria_monocytogenes_1639_v180	Listeria_monocytogenes_1639_v13	0.8936
Listeria_monocytogenes_1639_v17	Listeria_monocytogenes_1639_v13	0.9403
Listeria_monocytogenes_1639_v179	Listeria_monocytogenes_1639_v13	0.9109
Listeria_monocytogenes_1639_v178	Listeria_monocytogenes_1639_v13	0.9209
Listeria_monocytogenes_1639_v177	Listeria_monocytogenes_1639_v13	0.9304
Listeria_monocytogenes_1639_v176	Listeria_monocytogenes_1639_v13	0.9083
Listeria_monocytogenes_1639_v175	Listeria_monocytogenes_1639_v13	0.9275
Listeria_monocytogenes_1639_v174	Listeria_monocytogenes_1639_v13	0.921
Listeria_monocytogenes_1639_v173	Listeria_monocytogenes_1639_v13	0.9363
Listeria_monocytogenes_1639_v172	Listeria_monocytogenes_1639_v13	0.9077
Listeria_monocytogenes_1639_v171	Listeria_monocytogenes_1639_v13	0.9243
Listeria_monocytogenes_1639_v170	Listeria_monocytogenes_1639_v13	0.917
Listeria_monocytogenes_1639_v16	Listeria_monocytogenes_1639_v13	0.9141
Listeria_monocytogenes_1639_v169	Listeria_monocytogenes_1639_v13	0.9293
Listeria_monocytogenes_1639_v168	Listeria_monocytogenes_1639_v13	0.9137
Listeria_monocytogenes_1639_v167	Listeria_monocytogenes_1639_v13	0.9144
Listeria_monocytogenes_1639_v166	Listeria_monocytogenes_1639_v13	0.914
Listeria_monocytogenes_1639_v165	Listeria_monocytogenes_1639_v13	0.929
Listeria_monocytogenes_1639_v164	Listeria_monocytogenes_1639_v13	0.9291
Listeria_monocytogenes_1639_v163	Listeria_monocytogenes_1639_v13	0.9366
Listeria_monocytogenes_1639_v162	Listeria_monocytogenes_1639_v13	0.9291
Listeria_monocytogenes_1639_v161	Listeria_monocytogenes_1639_v13	0.9295
Listeria_monocytogenes_1639_v160	Listeria_monocytogenes_1639_v13	0.9295
Listeria_monocytogenes_1639_v15	Listeria_monocytogenes_1639_v13	0.9993
Listeria_monocytogenes_1639_v159	Listeria_monocytogenes_1639_v13	0.9304
Listeria_monocytogenes_1639_v158	Listeria_monocytogenes_1639_v13	0.9299
Listeria_monocytogenes_1639_v157	Listeria_monocytogenes_1639_v13	0.9299
Listeria_monocytogenes_1639_v156	Listeria_monocytogenes_1639_v13	0.9176
Listeria_monocytogenes_1639_v155	Listeria_monocytogenes_1639_v13	0.9199
Listeria_monocytogenes_1639_v154	Listeria_monocytogenes_1639_v13	0.9314
Listeria_monocytogenes_1639_v153	Listeria_monocytogenes_1639_v13	0.9269
Listeria_monocytogenes_1639_v152	Listeria_monocytogenes_1639_v13	0.9307
Listeria_monocytogenes_1639_v151	Listeria_monocytogenes_1639_v13	0.9307
Listeria_monocytogenes_1639_v150	Listeria_monocytogenes_1639_v13	0.9239
Listeria_monocytogenes_1639_v14	Listeria_monocytogenes_1639_v13	0.945
Listeria_monocytogenes_1639_v149	Listeria_monocytogenes_1639_v13	0.9248
Listeria_monocytogenes_1639_v148	Listeria_monocytogenes_1639_v13	0.9297
Listeria_monocytogenes_1639_v147	Listeria_monocytogenes_1639_v13	0.9209
Listeria_monocytogenes_1639_v146	Listeria_monocytogenes_1639_v13	0.9237
Listeria_monocytogenes_1639_v145	Listeria_monocytogenes_1639_v13	0.9154
Listeria_monocytogenes_1639_v144	Listeria_monocytogenes_1639_v13	0.9306
Listeria_monocytogenes_1639_v143	Listeria_monocytogenes_1639_v13	0.9238
Listeria_monocytogenes_1639_v142	Listeria_monocytogenes_1639_v13	0.931
Listeria_monocytogenes_1639_v141	Listeria_monocytogenes_1639_v13	0.9387
Listeria_monocytogenes_1639_v140	Listeria_monocytogenes_1639_v13	0.9313
Listeria_monocytogenes_1639_v13	Listeria_monocytogenes_1639_v13	0.071

Listeria_welshimeri_1643	Listeria_monocytogenes_1639_v140	0.8863
Listeria_welshimeri_serovar_6b_str_SLCC5334_386043	Listeria_monocytogenes_1639_v140	0.8859
Listeria_seeligeri_serovar_1_2b_str_SLCC3954_683837	Listeria_monocytogenes_1639_v140	0.8908
Listeria_seeligeri_FSL_S4-171_702452	Listeria_monocytogenes_1639_v140	0.8329
Listeria_seeligeri_FSL_N1-067_702453	Listeria_monocytogenes_1639_v140	0.8313
Listeria_seeligeri_1640_v1	Listeria_monocytogenes_1639_v140	0.8969
Listeria_seeligeri_1640	Listeria_monocytogenes_1639_v140	0.8888
Listeria_monocytogenes_WSLC1042_1457188	Listeria_monocytogenes_1639_v140	0.9724
Listeria_monocytogenes_WSLC1001_1457187	Listeria_monocytogenes_1639_v140	0.9385
Listeria_monocytogenes_SLCC7179_879090	Listeria_monocytogenes_1639_v140	0.9434
Listeria_monocytogenes_SLCC5850_882096	Listeria_monocytogenes_1639_v140	0.9432
Listeria_monocytogenes_SLCC2755_932919	Listeria_monocytogenes_1639_v140	0.9536
Listeria_monocytogenes_SLCC2540_879089	Listeria_monocytogenes_1639_v140	0.9538
Listeria_monocytogenes_SLCC2479_882020	Listeria_monocytogenes_1639_v140	0.934
Listeria_monocytogenes_SLCC2378_879088	Listeria_monocytogenes_1639_v140	0.9731
Listeria_monocytogenes_SLCC2376_882097	Listeria_monocytogenes_1639_v140	0.9511
Listeria_monocytogenes_SLCC2372_932920	Listeria_monocytogenes_1639_v140	0.9266
Listeria_monocytogenes_serotype_4b_str_LL195_1230340	Listeria_monocytogenes_1639_v140	0.9795
Listeria_monocytogenes_serotype_4b_str_LIS0087_1134669	Listeria_monocytogenes_1639_v140	0.9191
Listeria_monocytogenes_serotype_4b_str_F2365_265669	Listeria_monocytogenes_1639_v140	0.9795
Listeria_monocytogenes_serotype_4b_str_CLIP_80459_568819	Listeria_monocytogenes_1639_v140	0.9992
Listeria_monocytogenes_serotype_4b_str_81-0861_1196161	Listeria_monocytogenes_1639_v140	0.9617
Listeria_monocytogenes_serotype_4b_str_81-0592_1196160	Listeria_monocytogenes_1639_v140	0.9628
Listeria_monocytogenes_serotype_4b_str_81-0558_1196159	Listeria_monocytogenes_1639_v140	0.9635
Listeria_monocytogenes_serotype_4b_str_10-0809_1196162	Listeria_monocytogenes_1639_v140	0.9631
Listeria_monocytogenes_serotype_4b_str_02-6680_1196175	Listeria_monocytogenes_1639_v140	0.9819
Listeria_monocytogenes_serotype_4b_str_02-6679_1196174	Listeria_monocytogenes_1639_v140	0.9819
Listeria_monocytogenes_serotype_4b_str_02-1792_1196173	Listeria_monocytogenes_1639_v140	0.9631
Listeria_monocytogenes_serotype_4b_str_02-1289_1196172	Listeria_monocytogenes_1639_v140	0.9631
Listeria_monocytogenes_serotype_4b_str_02-1103_1196171	Listeria_monocytogenes_1639_v140	0.9631
Listeria_monocytogenes_serotype_3c_str_10-5027_1196195	Listeria_monocytogenes_1639_v140	0.9332
Listeria_monocytogenes_serotype_1_2c_str_10-5026_1196194	Listeria_monocytogenes_1639_v140	0.934
Listeria_monocytogenes_serotype_1_2c_str_10-5025_1196193	Listeria_monocytogenes_1639_v140	0.934
Listeria_monocytogenes_serotype_1_2b_str_10-0811_1196164	Listeria_monocytogenes_1639_v140	0.9468
Listeria_monocytogenes_serotype_1_2b_str_10-0810_1196163	Listeria_monocytogenes_1639_v140	0.9468
Listeria_monocytogenes_serotype_1_2a_str_99-6370_1196179	Listeria_monocytogenes_1639_v140	0.924
Listeria_monocytogenes_serotype_1_2a_str_98-2035_1196178	Listeria_monocytogenes_1639_v140	0.924
Listeria_monocytogenes_serotype_1_2a_str_95-0093_1196177	Listeria_monocytogenes_1639_v140	0.9296
Listeria_monocytogenes_serotype_1_2a_str_88-0478_1196176	Listeria_monocytogenes_1639_v140	0.9284
Listeria_monocytogenes_serotype_1_2a_str_10-5024_1196192	Listeria_monocytogenes_1639_v140	0.9296
Listeria_monocytogenes_serotype_1_2a_str_10-4758_1196168	Listeria_monocytogenes_1639_v140	0.9441
Listeria_monocytogenes_serotype_1_2a_str_10-4754_1196167	Listeria_monocytogenes_1639_v140	0.9441
Listeria_monocytogenes_serotype_1_2a_str_10-1321_1196191	Listeria_monocytogenes_1639_v140	0.9296
Listeria_monocytogenes_serotype_1_2a_str_10-1047_1196190	Listeria_monocytogenes_1639_v140	0.9303
Listeria_monocytogenes_serotype_1_2a_str_10-1046_1196189	Listeria_monocytogenes_1639_v140	0.9303
Listeria_monocytogenes_serotype_1_2a_str_10-0934_1196170	Listeria_monocytogenes_1639_v140	0.9538
Listeria_monocytogenes_serotype_1_2a_str_10-0933_1196169	Listeria_monocytogenes_1639_v140	0.9538
Listeria_monocytogenes_serotype_1_2a_str_10-0815_1196188	Listeria_monocytogenes_1639_v140	0.9254
Listeria_monocytogenes_serotype_1_2a_str_10-0814_1196187	Listeria_monocytogenes_1639_v140	0.9299
Listeria_monocytogenes_serotype_1_2a_str_10-0813_1196166	Listeria_monocytogenes_1639_v140	0.936
Listeria_monocytogenes_serotype_1_2a_str_10-0812_1196165	Listeria_monocytogenes_1639_v140	0.936
Listeria_monocytogenes_serotype_1_2a_str_08-7669_1196186	Listeria_monocytogenes_1639_v140	0.9391
Listeria_monocytogenes_serotype_1_2a_str_08-7374_1196185	Listeria_monocytogenes_1639_v140	0.9296
Listeria_monocytogenes_serotype_1_2a_str_08-6997_1196184	Listeria_monocytogenes_1639_v140	0.9254
Listeria_monocytogenes_serotype_1_2a_str_08-6569_1196183	Listeria_monocytogenes_1639_v140	0.9254
Listeria_monocytogenes_serotype_1_2a_str_08-6056_1196182	Listeria_monocytogenes_1639_v140	0.9254
Listeria_monocytogenes_serotype_1_2a_str_04-5457_1196181	Listeria_monocytogenes_1639_v140	0.9296
Listeria_monocytogenes_serotype_1_2a_str_02-5993_1196180	Listeria_monocytogenes_1639_v140	0.9296
Listeria_monocytogenes_R479a_1437838	Listeria_monocytogenes_1639_v140	0.9289
Listeria_monocytogenes_N53-1_1234142	Listeria_monocytogenes_1639_v140	0.8987
Listeria_monocytogenes_M7_1030009	Listeria_monocytogenes_1639_v140	0.9158
Listeria_monocytogenes_LO28_393132	Listeria_monocytogenes_1639_v140	0.9326
Listeria_monocytogenes_L99_563174	Listeria_monocytogenes_1639_v140	0.9161
Listeria_monocytogenes_L312_882094	Listeria_monocytogenes_1639_v140	0.9996
Listeria_monocytogenes_J1816_930781	Listeria_monocytogenes_1639_v140	0.9569
Listeria_monocytogenes_J1-220_930782	Listeria_monocytogenes_1639_v140	0.9533
Listeria_monocytogenes_J0161_393130	Listeria_monocytogenes_1639_v140	0.936
Listeria_monocytogenes_HCC23_552536	Listeria_monocytogenes_1639_v140	0.9154
Listeria_monocytogenes_FSL_R2-561_393126	Listeria_monocytogenes_1639_v140	0.9326
Listeria_monocytogenes_FSL_J1-208_393119	Listeria_monocytogenes_1639_v140	0.9232
Listeria_monocytogenes_FSL_J1-175_393118	Listeria_monocytogenes_1639_v140	0.9574
Listeria_monocytogenes_Finland_1998_393127	Listeria_monocytogenes_1639_v140	0.9546
Listeria_monocytogenes_EGD-e_169963_v1	Listeria_monocytogenes_1639_v140	0.9419
Listeria_monocytogenes_EGD-e_169963	Listeria_monocytogenes_1639_v140	0.9422
Listeria_monocytogenes_EGD_1334565	Listeria_monocytogenes_1639_v140	0.944
Listeria_monocytogenes_CFSAN002349_1299895	Listeria_monocytogenes_1639_v140	0.9811
Listeria_monocytogenes_ATCC_19117_882095_v1	Listeria_monocytogenes_1639_v140	0.9734
Listeria_monocytogenes_ATCC_19117_882095	Listeria_monocytogenes_1639_v140	0.973
Listeria_monocytogenes_6179_1288295	Listeria_monocytogenes_1639_v140	0.9156
Listeria_monocytogenes_1639_v9	Listeria_monocytogenes_1639_v140	0.9281
Listeria_monocytogenes_1639_v99	Listeria_monocytogenes_1639_v140	0.9584
Listeria_monocytogenes_1639_v98	Listeria_monocytogenes_1639_v140	0.934
Listeria_monocytogenes_1639_v97	Listeria_monocytogenes_1639_v140	0.968
Listeria_monocytogenes_1639_v96	Listeria_monocytogenes_1639_v140	0.9573
Listeria_monocytogenes_1639_v95	Listeria_monocytogenes_1639_v140	0.9573
Listeria_monocytogenes_1639_v94	Listeria_monocytogenes_1639_v140	0.958
Listeria_monocytogenes_1639_v93	Listeria_monocytogenes_1639_v140	0.9284
Listeria_monocytogenes_1639_v92	Listeria_monocytogenes_1639_v140	0.958
Listeria_monocytogenes_1639_v91	Listeria_monocytogenes_1639_v140	0.9606
Listeria_monocytogenes_1639_v90	Listeria_monocytogenes_1639_v140	0.934
Listeria_monocytogenes_1639_v8	Listeria_monocytogenes_1639_v140	0.9182

Listeria_monocytogenes_1639_v89	Listeria_monocytogenes_1639_v140	0.9682
Listeria_monocytogenes_1639_v88	Listeria_monocytogenes_1639_v140	0.9342
Listeria_monocytogenes_1639_v87	Listeria_monocytogenes_1639_v140	0.9315
Listeria_monocytogenes_1639_v86	Listeria_monocytogenes_1639_v140	0.9311
Listeria_monocytogenes_1639_v85	Listeria_monocytogenes_1639_v140	0.9609
Listeria_monocytogenes_1639_v84	Listeria_monocytogenes_1639_v140	0.9409
Listeria_monocytogenes_1639_v83	Listeria_monocytogenes_1639_v140	0.9619
Listeria_monocytogenes_1639_v82	Listeria_monocytogenes_1639_v140	0.9612
Listeria_monocytogenes_1639_v81	Listeria_monocytogenes_1639_v140	0.9346
Listeria_monocytogenes_1639_v80	Listeria_monocytogenes_1639_v140	0.9512
Listeria_monocytogenes_1639_v7	Listeria_monocytogenes_1639_v140	0.9182
Listeria_monocytogenes_1639_v79	Listeria_monocytogenes_1639_v140	0.9328
Listeria_monocytogenes_1639_v78	Listeria_monocytogenes_1639_v140	0.9339
Listeria_monocytogenes_1639_v77	Listeria_monocytogenes_1639_v140	0.9325
Listeria_monocytogenes_1639_v76	Listeria_monocytogenes_1639_v140	0.928
Listeria_monocytogenes_1639_v75	Listeria_monocytogenes_1639_v140	0.9161
Listeria_monocytogenes_1639_v74	Listeria_monocytogenes_1639_v140	0.93
Listeria_monocytogenes_1639_v73	Listeria_monocytogenes_1639_v140	0.9277
Listeria_monocytogenes_1639_v72	Listeria_monocytogenes_1639_v140	0.9608
Listeria_monocytogenes_1639_v71	Listeria_monocytogenes_1639_v140	0.9196
Listeria_monocytogenes_1639_v70	Listeria_monocytogenes_1639_v140	0.9301
Listeria_monocytogenes_1639_v6	Listeria_monocytogenes_1639_v140	0.9178
Listeria_monocytogenes_1639_v69	Listeria_monocytogenes_1639_v140	0.9628
Listeria_monocytogenes_1639_v68	Listeria_monocytogenes_1639_v140	0.9399
Listeria_monocytogenes_1639_v67	Listeria_monocytogenes_1639_v140	0.9494
Listeria_monocytogenes_1639_v66	Listeria_monocytogenes_1639_v140	0.9384
Listeria_monocytogenes_1639_v65	Listeria_monocytogenes_1639_v140	0.9328
Listeria_monocytogenes_1639_v64	Listeria_monocytogenes_1639_v140	0.9339
Listeria_monocytogenes_1639_v63	Listeria_monocytogenes_1639_v140	0.9474
Listeria_monocytogenes_1639_v62	Listeria_monocytogenes_1639_v140	0.9304
Listeria_monocytogenes_1639_v61	Listeria_monocytogenes_1639_v140	0.9324
Listeria_monocytogenes_1639_v60	Listeria_monocytogenes_1639_v140	0.938
Listeria_monocytogenes_1639_v5	Listeria_monocytogenes_1639_v140	0.9113
Listeria_monocytogenes_1639_v59	Listeria_monocytogenes_1639_v140	0.9627
Listeria_monocytogenes_1639_v58	Listeria_monocytogenes_1639_v140	0.9616
Listeria_monocytogenes_1639_v57	Listeria_monocytogenes_1639_v140	0.9541
Listeria_monocytogenes_1639_v56	Listeria_monocytogenes_1639_v140	0.9623
Listeria_monocytogenes_1639_v55	Listeria_monocytogenes_1639_v140	0.9501
Listeria_monocytogenes_1639_v54	Listeria_monocytogenes_1639_v140	0.9272
Listeria_monocytogenes_1639_v53	Listeria_monocytogenes_1639_v140	0.9357
Listeria_monocytogenes_1639_v52	Listeria_monocytogenes_1639_v140	0.9594
Listeria_monocytogenes_1639_v51	Listeria_monocytogenes_1639_v140	0.9561
Listeria_monocytogenes_1639_v50	Listeria_monocytogenes_1639_v140	0.9288
Listeria_monocytogenes_1639_v4	Listeria_monocytogenes_1639_v140	0.9248
Listeria_monocytogenes_1639_v49	Listeria_monocytogenes_1639_v140	0.9624
Listeria_monocytogenes_1639_v48	Listeria_monocytogenes_1639_v140	0.9284
Listeria_monocytogenes_1639_v47	Listeria_monocytogenes_1639_v140	0.9292
Listeria_monocytogenes_1639_v46	Listeria_monocytogenes_1639_v140	0.9392
Listeria_monocytogenes_1639_v45	Listeria_monocytogenes_1639_v140	0.9202
Listeria_monocytogenes_1639_v44	Listeria_monocytogenes_1639_v140	0.9296
Listeria_monocytogenes_1639_v43	Listeria_monocytogenes_1639_v140	0.9296
Listeria_monocytogenes_1639_v42	Listeria_monocytogenes_1639_v140	0.9217
Listeria_monocytogenes_1639_v41	Listeria_monocytogenes_1639_v140	0.9093
Listeria_monocytogenes_1639_v40	Listeria_monocytogenes_1639_v140	0.933
Listeria_monocytogenes_1639_v3	Listeria_monocytogenes_1639_v140	0.9251
Listeria_monocytogenes_1639_v39	Listeria_monocytogenes_1639_v140	0.9448
Listeria_monocytogenes_1639_v38	Listeria_monocytogenes_1639_v140	0.9298
Listeria_monocytogenes_1639_v37	Listeria_monocytogenes_1639_v140	0.9249
Listeria_monocytogenes_1639_v36	Listeria_monocytogenes_1639_v140	0.9239
Listeria_monocytogenes_1639_v35	Listeria_monocytogenes_1639_v140	0.9336
Listeria_monocytogenes_1639_v34	Listeria_monocytogenes_1639_v140	0.9321
Listeria_monocytogenes_1639_v33	Listeria_monocytogenes_1639_v140	0.9468
Listeria_monocytogenes_1639_v32	Listeria_monocytogenes_1639_v140	0.9239
Listeria_monocytogenes_1639_v31	Listeria_monocytogenes_1639_v140	0.9325
Listeria_monocytogenes_1639_v30	Listeria_monocytogenes_1639_v140	0.9455
Listeria_monocytogenes_1639_v2	Listeria_monocytogenes_1639_v140	0.911
Listeria_monocytogenes_1639_v29	Listeria_monocytogenes_1639_v140	0.9194
Listeria_monocytogenes_1639_v28	Listeria_monocytogenes_1639_v140	0.9238
Listeria_monocytogenes_1639_v27	Listeria_monocytogenes_1639_v140	0.9146
Listeria_monocytogenes_1639_v26	Listeria_monocytogenes_1639_v140	0.9143
Listeria_monocytogenes_1639_v25	Listeria_monocytogenes_1639_v140	0.9143
Listeria_monocytogenes_1639_v24	Listeria_monocytogenes_1639_v140	0.9533
Listeria_monocytogenes_1639_v23	Listeria_monocytogenes_1639_v140	0.9529
Listeria_monocytogenes_1639_v22	Listeria_monocytogenes_1639_v140	0.9478
Listeria_monocytogenes_1639_v21	Listeria_monocytogenes_1639_v140	0.9529
Listeria_monocytogenes_1639_v20	Listeria_monocytogenes_1639_v140	0.9414
Listeria_monocytogenes_1639_v1	Listeria_monocytogenes_1639_v140	0.9093
Listeria_monocytogenes_1639_v19	Listeria_monocytogenes_1639_v140	0.9411
Listeria_monocytogenes_1639_v198	Listeria_monocytogenes_1639_v140	0.9015
Listeria_monocytogenes_1639_v197	Listeria_monocytogenes_1639_v140	0.9293
Listeria_monocytogenes_1639_v196	Listeria_monocytogenes_1639_v140	0.9266
Listeria_monocytogenes_1639_v195	Listeria_monocytogenes_1639_v140	0.9515
Listeria_monocytogenes_1639_v194	Listeria_monocytogenes_1639_v140	0.9515
Listeria_monocytogenes_1639_v193	Listeria_monocytogenes_1639_v140	0.9515
Listeria_monocytogenes_1639_v192	Listeria_monocytogenes_1639_v140	0.9157
Listeria_monocytogenes_1639_v191	Listeria_monocytogenes_1639_v140	0.9568
Listeria_monocytogenes_1639_v190	Listeria_monocytogenes_1639_v140	0.9553
Listeria_monocytogenes_1639_v18	Listeria_monocytogenes_1639_v140	0.948
Listeria_monocytogenes_1639_v189	Listeria_monocytogenes_1639_v140	0.9545
Listeria_monocytogenes_1639_v188	Listeria_monocytogenes_1639_v140	0.9583
Listeria_monocytogenes_1639_v187	Listeria_monocytogenes_1639_v140	0.9553

Listeria_monocytogenes_1639_v186	Listeria_monocytogenes_1639_v140	0.9557
Listeria_monocytogenes_1639_v185	Listeria_monocytogenes_1639_v140	0.9557
Listeria_monocytogenes_1639_v184	Listeria_monocytogenes_1639_v140	0.9545
Listeria_monocytogenes_1639_v183	Listeria_monocytogenes_1639_v140	0.9529
Listeria_monocytogenes_1639_v182	Listeria_monocytogenes_1639_v140	0.9552
Listeria_monocytogenes_1639_v181	Listeria_monocytogenes_1639_v140	0.9411
Listeria_monocytogenes_1639_v180	Listeria_monocytogenes_1639_v140	0.9411
Listeria_monocytogenes_1639_v17	Listeria_monocytogenes_1639_v140	0.9484
Listeria_monocytogenes_1639_v179	Listeria_monocytogenes_1639_v140	0.94
Listeria_monocytogenes_1639_v178	Listeria_monocytogenes_1639_v140	0.9537
Listeria_monocytogenes_1639_v177	Listeria_monocytogenes_1639_v140	0.9776
Listeria_monocytogenes_1639_v176	Listeria_monocytogenes_1639_v140	0.9485
Listeria_monocytogenes_1639_v175	Listeria_monocytogenes_1639_v140	0.9438
Listeria_monocytogenes_1639_v174	Listeria_monocytogenes_1639_v140	0.9557
Listeria_monocytogenes_1639_v173	Listeria_monocytogenes_1639_v140	0.9713
Listeria_monocytogenes_1639_v172	Listeria_monocytogenes_1639_v140	0.9463
Listeria_monocytogenes_1639_v171	Listeria_monocytogenes_1639_v140	0.9407
Listeria_monocytogenes_1639_v170	Listeria_monocytogenes_1639_v140	0.949
Listeria_monocytogenes_1639_v16	Listeria_monocytogenes_1639_v140	0.9158
Listeria_monocytogenes_1639_v169	Listeria_monocytogenes_1639_v140	0.9514
Listeria_monocytogenes_1639_v168	Listeria_monocytogenes_1639_v140	0.9496
Listeria_monocytogenes_1639_v167	Listeria_monocytogenes_1639_v140	0.9417
Listeria_monocytogenes_1639_v166	Listeria_monocytogenes_1639_v140	0.95
Listeria_monocytogenes_1639_v165	Listeria_monocytogenes_1639_v140	0.9514
Listeria_monocytogenes_1639_v164	Listeria_monocytogenes_1639_v140	0.9791
Listeria_monocytogenes_1639_v163	Listeria_monocytogenes_1639_v140	0.9675
Listeria_monocytogenes_1639_v162	Listeria_monocytogenes_1639_v140	0.9791
Listeria_monocytogenes_1639_v161	Listeria_monocytogenes_1639_v140	0.9803
Listeria_monocytogenes_1639_v160	Listeria_monocytogenes_1639_v140	0.9799
Listeria_monocytogenes_1639_v15	Listeria_monocytogenes_1639_v140	0.9313
Listeria_monocytogenes_1639_v159	Listeria_monocytogenes_1639_v140	0.978
Listeria_monocytogenes_1639_v158	Listeria_monocytogenes_1639_v140	0.9799
Listeria_monocytogenes_1639_v157	Listeria_monocytogenes_1639_v140	0.9795
Listeria_monocytogenes_1639_v156	Listeria_monocytogenes_1639_v140	0.9321
Listeria_monocytogenes_1639_v155	Listeria_monocytogenes_1639_v140	0.9462
Listeria_monocytogenes_1639_v154	Listeria_monocytogenes_1639_v140	0.9485
Listeria_monocytogenes_1639_v153	Listeria_monocytogenes_1639_v140	0.9469
Listeria_monocytogenes_1639_v152	Listeria_monocytogenes_1639_v140	0.978
Listeria_monocytogenes_1639_v151	Listeria_monocytogenes_1639_v140	0.978
Listeria_monocytogenes_1639_v150	Listeria_monocytogenes_1639_v140	0.9454
Listeria_monocytogenes_1639_v14	Listeria_monocytogenes_1639_v140	0.9379
Listeria_monocytogenes_1639_v149	Listeria_monocytogenes_1639_v140	0.9443
Listeria_monocytogenes_1639_v148	Listeria_monocytogenes_1639_v140	0.9769
Listeria_monocytogenes_1639_v147	Listeria_monocytogenes_1639_v140	0.9624
Listeria_monocytogenes_1639_v146	Listeria_monocytogenes_1639_v140	0.9566
Listeria_monocytogenes_1639_v145	Listeria_monocytogenes_1639_v140	0.9505
Listeria_monocytogenes_1639_v144	Listeria_monocytogenes_1639_v140	0.9988
Listeria_monocytogenes_1639_v143	Listeria_monocytogenes_1639_v140	0.9535
Listeria_monocytogenes_1639_v142	Listeria_monocytogenes_1639_v140	0.9992
Listeria_monocytogenes_1639_v141	Listeria_monocytogenes_1639_v140	0.9721
Listeria_monocytogenes_1639_v140	Listeria_monocytogenes_1639_v140	0.0662
Listeria_welshimeri_1643	Listeria_monocytogenes_1639_v141	0.8854
Listeria_welshimeri_serovar_6b_str_SLCC5334_386043	Listeria_monocytogenes_1639_v141	0.8847
Listeria_seeligeri_serovar_1_2b_str_SLCC3954_683837	Listeria_monocytogenes_1639_v141	0.8982
Listeria_seeligeri_FSL_S4-171_702452	Listeria_monocytogenes_1639_v141	0.8335
Listeria_seeligeri_FSL_N1-067_702453	Listeria_monocytogenes_1639_v141	0.8385
Listeria_seeligeri_1640_v1	Listeria_monocytogenes_1639_v141	0.9015
Listeria_seeligeri_1640	Listeria_monocytogenes_1639_v141	0.8894
Listeria_monocytogenes_WSLC1042_1457188	Listeria_monocytogenes_1639_v141	0.9639
Listeria_monocytogenes_WSLC1001_1457187	Listeria_monocytogenes_1639_v141	0.945
Listeria_monocytogenes_SLCC7179_879090	Listeria_monocytogenes_1639_v141	0.9514
Listeria_monocytogenes_SLCC5850_882096	Listeria_monocytogenes_1639_v141	0.9489
Listeria_monocytogenes_SLCC2755_932919	Listeria_monocytogenes_1639_v141	0.9596
Listeria_monocytogenes_SLCC2540_879089	Listeria_monocytogenes_1639_v141	0.9644
Listeria_monocytogenes_SLCC2479_882020	Listeria_monocytogenes_1639_v141	0.9431
Listeria_monocytogenes_SLCC2378_879088	Listeria_monocytogenes_1639_v141	0.965
Listeria_monocytogenes_SLCC2376_882097	Listeria_monocytogenes_1639_v141	0.9499
Listeria_monocytogenes_SLCC2372_932920	Listeria_monocytogenes_1639_v141	0.9356
Listeria_monocytogenes_serotype_4b_str_LL195_1230340	Listeria_monocytogenes_1639_v141	0.971
Listeria_monocytogenes_serotype_4b_str_LIS0087_1134669	Listeria_monocytogenes_1639_v141	0.9264
Listeria_monocytogenes_serotype_4b_str_F2365_265669	Listeria_monocytogenes_1639_v141	0.971
Listeria_monocytogenes_serotype_4b_str_CLIP_80459_568819	Listeria_monocytogenes_1639_v141	0.9717
Listeria_monocytogenes_serotype_4b_str_81-0861_1196161	Listeria_monocytogenes_1639_v141	0.9544
Listeria_monocytogenes_serotype_4b_str_81-0592_1196160	Listeria_monocytogenes_1639_v141	0.9555
Listeria_monocytogenes_serotype_4b_str_81-0558_1196159	Listeria_monocytogenes_1639_v141	0.9566
Listeria_monocytogenes_serotype_4b_str_10-0809_1196162	Listeria_monocytogenes_1639_v141	0.9559
Listeria_monocytogenes_serotype_4b_str_02-6680_1196175	Listeria_monocytogenes_1639_v141	0.9695
Listeria_monocytogenes_serotype_4b_str_02-6679_1196174	Listeria_monocytogenes_1639_v141	0.9695
Listeria_monocytogenes_serotype_4b_str_02-1792_1196173	Listeria_monocytogenes_1639_v141	0.9566
Listeria_monocytogenes_serotype_4b_str_02-1289_1196172	Listeria_monocytogenes_1639_v141	0.9566
Listeria_monocytogenes_serotype_4b_str_02-1103_1196171	Listeria_monocytogenes_1639_v141	0.9566
Listeria_monocytogenes_serotype_3c_str_10-5027_1196195	Listeria_monocytogenes_1639_v141	0.9427
Listeria_monocytogenes_serotype_1_2c_str_10-5026_1196194	Listeria_monocytogenes_1639_v141	0.9438
Listeria_monocytogenes_serotype_1_2c_str_10-5025_1196193	Listeria_monocytogenes_1639_v141	0.9438
Listeria_monocytogenes_serotype_1_2b_str_10-0811_1196164	Listeria_monocytogenes_1639_v141	0.9543
Listeria_monocytogenes_serotype_1_2b_str_10-0810_1196163	Listeria_monocytogenes_1639_v141	0.9543
Listeria_monocytogenes_serotype_1_2a_str_99-6370_1196179	Listeria_monocytogenes_1639_v141	0.9307
Listeria_monocytogenes_serotype_1_2a_str_98-2035_1196178	Listeria_monocytogenes_1639_v141	0.9307
Listeria_monocytogenes_serotype_1_2a_str_95-0093_1196177	Listeria_monocytogenes_1639_v141	0.9363
Listeria_monocytogenes_serotype_1_2a_str_88-0478_1196176	Listeria_monocytogenes_1639_v141	0.9355
Listeria_monocytogenes_serotype_1_2a_str_10-5024_1196192	Listeria_monocytogenes_1639_v141	0.9367

Listeria_monocytogenes_serotype_1_2a_str_10-4758_1196168	Listeria_monocytogenes_1639_v141	0.9498
Listeria_monocytogenes_serotype_1_2a_str_10-4754_1196167	Listeria_monocytogenes_1639_v141	0.9494
Listeria_monocytogenes_serotype_1_2a_str_10-1321_1196191	Listeria_monocytogenes_1639_v141	0.9367
Listeria_monocytogenes_serotype_1_2a_str_10-1047_1196190	Listeria_monocytogenes_1639_v141	0.937
Listeria_monocytogenes_serotype_1_2a_str_10-1046_1196189	Listeria_monocytogenes_1639_v141	0.937
Listeria_monocytogenes_serotype_1_2a_str_10-0934_1196170	Listeria_monocytogenes_1639_v141	0.9592
Listeria_monocytogenes_serotype_1_2a_str_10-0933_1196169	Listeria_monocytogenes_1639_v141	0.9592
Listeria_monocytogenes_serotype_1_2a_str_10-0815_1196188	Listeria_monocytogenes_1639_v141	0.9321
Listeria_monocytogenes_serotype_1_2a_str_10-0814_1196187	Listeria_monocytogenes_1639_v141	0.9367
Listeria_monocytogenes_serotype_1_2a_str_10-0813_1196166	Listeria_monocytogenes_1639_v141	0.9429
Listeria_monocytogenes_serotype_1_2a_str_10-0812_1196165	Listeria_monocytogenes_1639_v141	0.9429
Listeria_monocytogenes_serotype_1_2a_str_08-7669_1196186	Listeria_monocytogenes_1639_v141	0.9455
Listeria_monocytogenes_serotype_1_2a_str_08-7374_1196185	Listeria_monocytogenes_1639_v141	0.9367
Listeria_monocytogenes_serotype_1_2a_str_08-6997_1196184	Listeria_monocytogenes_1639_v141	0.9321
Listeria_monocytogenes_serotype_1_2a_str_08-6569_1196183	Listeria_monocytogenes_1639_v141	0.9321
Listeria_monocytogenes_serotype_1_2a_str_08-6056_1196182	Listeria_monocytogenes_1639_v141	0.9321
Listeria_monocytogenes_serotype_1_2a_str_04-5457_1196181	Listeria_monocytogenes_1639_v141	0.9367
Listeria_monocytogenes_serotype_1_2a_str_02-5993_1196180	Listeria_monocytogenes_1639_v141	0.9363
Listeria_monocytogenes_R479a_1437838	Listeria_monocytogenes_1639_v141	0.9352
Listeria_monocytogenes_N53-1_1234142	Listeria_monocytogenes_1639_v141	0.9034
Listeria_monocytogenes_M7_1030009	Listeria_monocytogenes_1639_v141	0.9104
Listeria_monocytogenes_LO28_393132	Listeria_monocytogenes_1639_v141	0.942
Listeria_monocytogenes_L99_563174	Listeria_monocytogenes_1639_v141	0.9111
Listeria_monocytogenes_L312_882094	Listeria_monocytogenes_1639_v141	0.9721
Listeria_monocytogenes_J1816_930781	Listeria_monocytogenes_1639_v141	0.9519
Listeria_monocytogenes_J1-220_930782	Listeria_monocytogenes_1639_v141	0.9461
Listeria_monocytogenes_J0161_393130	Listeria_monocytogenes_1639_v141	0.9393
Listeria_monocytogenes_HCC23_552536	Listeria_monocytogenes_1639_v141	0.9104
Listeria_monocytogenes_FSL_R2-561_393126	Listeria_monocytogenes_1639_v141	0.9412
Listeria_monocytogenes_FSL_J1-208_393119	Listeria_monocytogenes_1639_v141	0.9189
Listeria_monocytogenes_FSL_J1-175_393118	Listeria_monocytogenes_1639_v141	0.9662
Listeria_monocytogenes_Finland_1998_393127	Listeria_monocytogenes_1639_v141	0.9604
Listeria_monocytogenes_EGD-e_169963_v1	Listeria_monocytogenes_1639_v141	0.9502
Listeria_monocytogenes_EGD-e_169963	Listeria_monocytogenes_1639_v141	0.9506
Listeria_monocytogenes_EGD_1334565	Listeria_monocytogenes_1639_v141	0.9497
Listeria_monocytogenes_CFSAN002349_1299895	Listeria_monocytogenes_1639_v141	0.9722
Listeria_monocytogenes_ATCC_19117_882095_v1	Listeria_monocytogenes_1639_v141	0.9649
Listeria_monocytogenes_ATCC_19117_882095	Listeria_monocytogenes_1639_v141	0.9649
Listeria_monocytogenes_6179_1288295	Listeria_monocytogenes_1639_v141	0.9238
Listeria_monocytogenes_1639_v9	Listeria_monocytogenes_1639_v141	0.9382
Listeria_monocytogenes_1639_v99	Listeria_monocytogenes_1639_v141	0.9527
Listeria_monocytogenes_1639_v98	Listeria_monocytogenes_1639_v141	0.9378
Listeria_monocytogenes_1639_v97	Listeria_monocytogenes_1639_v141	0.9603
Listeria_monocytogenes_1639_v96	Listeria_monocytogenes_1639_v141	0.9523
Listeria_monocytogenes_1639_v95	Listeria_monocytogenes_1639_v141	0.9523
Listeria_monocytogenes_1639_v94	Listeria_monocytogenes_1639_v141	0.9527
Listeria_monocytogenes_1639_v93	Listeria_monocytogenes_1639_v141	0.9366
Listeria_monocytogenes_1639_v92	Listeria_monocytogenes_1639_v141	0.9531
Listeria_monocytogenes_1639_v91	Listeria_monocytogenes_1639_v141	0.9537
Listeria_monocytogenes_1639_v90	Listeria_monocytogenes_1639_v141	0.9378
Listeria_monocytogenes_1639_v8	Listeria_monocytogenes_1639_v141	0.9248
Listeria_monocytogenes_1639_v89	Listeria_monocytogenes_1639_v141	0.9647
Listeria_monocytogenes_1639_v88	Listeria_monocytogenes_1639_v141	0.9425
Listeria_monocytogenes_1639_v87	Listeria_monocytogenes_1639_v141	0.939
Listeria_monocytogenes_1639_v86	Listeria_monocytogenes_1639_v141	0.9382
Listeria_monocytogenes_1639_v85	Listeria_monocytogenes_1639_v141	0.9541
Listeria_monocytogenes_1639_v84	Listeria_monocytogenes_1639_v141	0.9451
Listeria_monocytogenes_1639_v83	Listeria_monocytogenes_1639_v141	0.9554
Listeria_monocytogenes_1639_v82	Listeria_monocytogenes_1639_v141	0.9551
Listeria_monocytogenes_1639_v81	Listeria_monocytogenes_1639_v141	0.9425
Listeria_monocytogenes_1639_v80	Listeria_monocytogenes_1639_v141	0.9588
Listeria_monocytogenes_1639_v7	Listeria_monocytogenes_1639_v141	0.9248
Listeria_monocytogenes_1639_v79	Listeria_monocytogenes_1639_v141	0.9408
Listeria_monocytogenes_1639_v78	Listeria_monocytogenes_1639_v141	0.9418
Listeria_monocytogenes_1639_v77	Listeria_monocytogenes_1639_v141	0.9431
Listeria_monocytogenes_1639_v76	Listeria_monocytogenes_1639_v141	0.9339
Listeria_monocytogenes_1639_v75	Listeria_monocytogenes_1639_v141	0.9111
Listeria_monocytogenes_1639_v74	Listeria_monocytogenes_1639_v141	0.9363
Listeria_monocytogenes_1639_v73	Listeria_monocytogenes_1639_v141	0.9378
Listeria_monocytogenes_1639_v72	Listeria_monocytogenes_1639_v141	0.9535
Listeria_monocytogenes_1639_v71	Listeria_monocytogenes_1639_v141	0.924
Listeria_monocytogenes_1639_v70	Listeria_monocytogenes_1639_v141	0.9395
Listeria_monocytogenes_1639_v6	Listeria_monocytogenes_1639_v141	0.9241
Listeria_monocytogenes_1639_v69	Listeria_monocytogenes_1639_v141	0.9559
Listeria_monocytogenes_1639_v68	Listeria_monocytogenes_1639_v141	0.9452
Listeria_monocytogenes_1639_v67	Listeria_monocytogenes_1639_v141	0.9581
Listeria_monocytogenes_1639_v66	Listeria_monocytogenes_1639_v141	0.9456
Listeria_monocytogenes_1639_v65	Listeria_monocytogenes_1639_v141	0.9399
Listeria_monocytogenes_1639_v64	Listeria_monocytogenes_1639_v141	0.9365
Listeria_monocytogenes_1639_v63	Listeria_monocytogenes_1639_v141	0.9542
Listeria_monocytogenes_1639_v62	Listeria_monocytogenes_1639_v141	0.9397
Listeria_monocytogenes_1639_v61	Listeria_monocytogenes_1639_v141	0.9398
Listeria_monocytogenes_1639_v60	Listeria_monocytogenes_1639_v141	0.9402
Listeria_monocytogenes_1639_v5	Listeria_monocytogenes_1639_v141	0.9182
Listeria_monocytogenes_1639_v59	Listeria_monocytogenes_1639_v141	0.955
Listeria_monocytogenes_1639_v58	Listeria_monocytogenes_1639_v141	0.9604
Listeria_monocytogenes_1639_v57	Listeria_monocytogenes_1639_v141	0.9484
Listeria_monocytogenes_1639_v56	Listeria_monocytogenes_1639_v141	0.955
Listeria_monocytogenes_1639_v55	Listeria_monocytogenes_1639_v141	0.9452
Listeria_monocytogenes_1639_v54	Listeria_monocytogenes_1639_v141	0.9287
Listeria_monocytogenes_1639_v53	Listeria_monocytogenes_1639_v141	0.9402

Listeria_monocytogenes_1639_v52	Listeria_monocytogenes_1639_v141	0.9518
Listeria_monocytogenes_1639_v51	Listeria_monocytogenes_1639_v141	0.95
Listeria_monocytogenes_1639_v50	Listeria_monocytogenes_1639_v141	0.9359
Listeria_monocytogenes_1639_v4	Listeria_monocytogenes_1639_v141	0.9313
Listeria_monocytogenes_1639_v49	Listeria_monocytogenes_1639_v141	0.9555
Listeria_monocytogenes_1639_v48	Listeria_monocytogenes_1639_v141	0.9378
Listeria_monocytogenes_1639_v47	Listeria_monocytogenes_1639_v141	0.9363
Listeria_monocytogenes_1639_v46	Listeria_monocytogenes_1639_v141	0.9441
Listeria_monocytogenes_1639_v45	Listeria_monocytogenes_1639_v141	0.9269
Listeria_monocytogenes_1639_v44	Listeria_monocytogenes_1639_v141	0.9367
Listeria_monocytogenes_1639_v43	Listeria_monocytogenes_1639_v141	0.9367
Listeria_monocytogenes_1639_v42	Listeria_monocytogenes_1639_v141	0.9247
Listeria_monocytogenes_1639_v41	Listeria_monocytogenes_1639_v141	0.9129
Listeria_monocytogenes_1639_v40	Listeria_monocytogenes_1639_v141	0.9416
Listeria_monocytogenes_1639_v3	Listeria_monocytogenes_1639_v141	0.9317
Listeria_monocytogenes_1639_v39	Listeria_monocytogenes_1639_v141	0.9538
Listeria_monocytogenes_1639_v38	Listeria_monocytogenes_1639_v141	0.9384
Listeria_monocytogenes_1639_v37	Listeria_monocytogenes_1639_v141	0.9346
Listeria_monocytogenes_1639_v36	Listeria_monocytogenes_1639_v141	0.9343
Listeria_monocytogenes_1639_v35	Listeria_monocytogenes_1639_v141	0.9423
Listeria_monocytogenes_1639_v34	Listeria_monocytogenes_1639_v141	0.9366
Listeria_monocytogenes_1639_v33	Listeria_monocytogenes_1639_v141	0.9543
Listeria_monocytogenes_1639_v32	Listeria_monocytogenes_1639_v141	0.9335
Listeria_monocytogenes_1639_v31	Listeria_monocytogenes_1639_v141	0.937
Listeria_monocytogenes_1639_v30	Listeria_monocytogenes_1639_v141	0.9534
Listeria_monocytogenes_1639_v2	Listeria_monocytogenes_1639_v141	0.9192
Listeria_monocytogenes_1639_v29	Listeria_monocytogenes_1639_v141	0.9287
Listeria_monocytogenes_1639_v28	Listeria_monocytogenes_1639_v141	0.9328
Listeria_monocytogenes_1639_v27	Listeria_monocytogenes_1639_v141	0.9234
Listeria_monocytogenes_1639_v26	Listeria_monocytogenes_1639_v141	0.9238
Listeria_monocytogenes_1639_v25	Listeria_monocytogenes_1639_v141	0.9242
Listeria_monocytogenes_1639_v24	Listeria_monocytogenes_1639_v141	0.9468
Listeria_monocytogenes_1639_v23	Listeria_monocytogenes_1639_v141	0.9468
Listeria_monocytogenes_1639_v22	Listeria_monocytogenes_1639_v141	0.9557
Listeria_monocytogenes_1639_v21	Listeria_monocytogenes_1639_v141	0.9461
Listeria_monocytogenes_1639_v20	Listeria_monocytogenes_1639_v141	0.95
Listeria_monocytogenes_1639_v1	Listeria_monocytogenes_1639_v141	0.9163
Listeria_monocytogenes_1639_v19	Listeria_monocytogenes_1639_v141	0.9497
Listeria_monocytogenes_1639_v198	Listeria_monocytogenes_1639_v141	0.9058
Listeria_monocytogenes_1639_v197	Listeria_monocytogenes_1639_v141	0.9376
Listeria_monocytogenes_1639_v196	Listeria_monocytogenes_1639_v141	0.9324
Listeria_monocytogenes_1639_v195	Listeria_monocytogenes_1639_v141	0.9506
Listeria_monocytogenes_1639_v194	Listeria_monocytogenes_1639_v141	0.9507
Listeria_monocytogenes_1639_v193	Listeria_monocytogenes_1639_v141	0.9507
Listeria_monocytogenes_1639_v192	Listeria_monocytogenes_1639_v141	0.9103
Listeria_monocytogenes_1639_v191	Listeria_monocytogenes_1639_v141	0.9587
Listeria_monocytogenes_1639_v190	Listeria_monocytogenes_1639_v141	0.9607
Listeria_monocytogenes_1639_v18	Listeria_monocytogenes_1639_v141	0.9438
Listeria_monocytogenes_1639_v189	Listeria_monocytogenes_1639_v141	0.9599
Listeria_monocytogenes_1639_v188	Listeria_monocytogenes_1639_v141	0.9594
Listeria_monocytogenes_1639_v187	Listeria_monocytogenes_1639_v141	0.9603
Listeria_monocytogenes_1639_v186	Listeria_monocytogenes_1639_v141	0.9611
Listeria_monocytogenes_1639_v185	Listeria_monocytogenes_1639_v141	0.9607
Listeria_monocytogenes_1639_v184	Listeria_monocytogenes_1639_v141	0.9599
Listeria_monocytogenes_1639_v183	Listeria_monocytogenes_1639_v141	0.9572
Listeria_monocytogenes_1639_v182	Listeria_monocytogenes_1639_v141	0.9594
Listeria_monocytogenes_1639_v181	Listeria_monocytogenes_1639_v141	0.9299
Listeria_monocytogenes_1639_v180	Listeria_monocytogenes_1639_v141	0.9299
Listeria_monocytogenes_1639_v17	Listeria_monocytogenes_1639_v141	0.9438
Listeria_monocytogenes_1639_v179	Listeria_monocytogenes_1639_v141	0.9441
Listeria_monocytogenes_1639_v178	Listeria_monocytogenes_1639_v141	0.9619
Listeria_monocytogenes_1639_v177	Listeria_monocytogenes_1639_v141	0.9699
Listeria_monocytogenes_1639_v176	Listeria_monocytogenes_1639_v141	0.9477
Listeria_monocytogenes_1639_v175	Listeria_monocytogenes_1639_v141	0.9506
Listeria_monocytogenes_1639_v174	Listeria_monocytogenes_1639_v141	0.965
Listeria_monocytogenes_1639_v173	Listeria_monocytogenes_1639_v141	0.9813
Listeria_monocytogenes_1639_v172	Listeria_monocytogenes_1639_v141	0.9416
Listeria_monocytogenes_1639_v171	Listeria_monocytogenes_1639_v141	0.9452
Listeria_monocytogenes_1639_v170	Listeria_monocytogenes_1639_v141	0.9536
Listeria_monocytogenes_1639_v16	Listeria_monocytogenes_1639_v141	0.9228
Listeria_monocytogenes_1639_v169	Listeria_monocytogenes_1639_v141	0.9545
Listeria_monocytogenes_1639_v168	Listeria_monocytogenes_1639_v141	0.9534
Listeria_monocytogenes_1639_v167	Listeria_monocytogenes_1639_v141	0.9447
Listeria_monocytogenes_1639_v166	Listeria_monocytogenes_1639_v141	0.9538
Listeria_monocytogenes_1639_v165	Listeria_monocytogenes_1639_v141	0.9541
Listeria_monocytogenes_1639_v164	Listeria_monocytogenes_1639_v141	0.9702
Listeria_monocytogenes_1639_v163	Listeria_monocytogenes_1639_v141	0.9779
Listeria_monocytogenes_1639_v162	Listeria_monocytogenes_1639_v141	0.9702
Listeria_monocytogenes_1639_v161	Listeria_monocytogenes_1639_v141	0.9714
Listeria_monocytogenes_1639_v160	Listeria_monocytogenes_1639_v141	0.9714
Listeria_monocytogenes_1639_v15	Listeria_monocytogenes_1639_v141	0.9383
Listeria_monocytogenes_1639_v159	Listeria_monocytogenes_1639_v141	0.9703
Listeria_monocytogenes_1639_v158	Listeria_monocytogenes_1639_v141	0.9714
Listeria_monocytogenes_1639_v157	Listeria_monocytogenes_1639_v141	0.971
Listeria_monocytogenes_1639_v156	Listeria_monocytogenes_1639_v141	0.9385
Listeria_monocytogenes_1639_v155	Listeria_monocytogenes_1639_v141	0.9503
Listeria_monocytogenes_1639_v154	Listeria_monocytogenes_1639_v141	0.9542
Listeria_monocytogenes_1639_v153	Listeria_monocytogenes_1639_v141	0.9495
Listeria_monocytogenes_1639_v152	Listeria_monocytogenes_1639_v141	0.9706
Listeria_monocytogenes_1639_v151	Listeria_monocytogenes_1639_v141	0.9706
Listeria_monocytogenes_1639_v150	Listeria_monocytogenes_1639_v141	0.9511

Listeria_monocytogenes_1639_v14	Listeria_monocytogenes_1639_v141	0.9449
Listeria_monocytogenes_1639_v149	Listeria_monocytogenes_1639_v141	0.9497
Listeria_monocytogenes_1639_v148	Listeria_monocytogenes_1639_v141	0.9695
Listeria_monocytogenes_1639_v147	Listeria_monocytogenes_1639_v141	0.9548
Listeria_monocytogenes_1639_v146	Listeria_monocytogenes_1639_v141	0.9616
Listeria_monocytogenes_1639_v145	Listeria_monocytogenes_1639_v141	0.9512
Listeria_monocytogenes_1639_v144	Listeria_monocytogenes_1639_v141	0.9714
Listeria_monocytogenes_1639_v143	Listeria_monocytogenes_1639_v141	0.9581
Listeria_monocytogenes_1639_v142	Listeria_monocytogenes_1639_v141	0.9717
Listeria_monocytogenes_1639_v141	Listeria_monocytogenes_1639_v141	0.0659
Listeria_welshimeri_1643	Listeria_monocytogenes_1639_v142	0.8863
Listeria_welshimeri_serovar_6b_str_SLCC5334_386043	Listeria_monocytogenes_1639_v142	0.8856
Listeria_seeligeri_serovar_1_2b_str_SLCC3954_683837	Listeria_monocytogenes_1639_v142	0.8905
Listeria_seeligeri_FSL_S4-171_702452	Listeria_monocytogenes_1639_v142	0.833
Listeria_seeligeri_FSL_N1-067_702453	Listeria_monocytogenes_1639_v142	0.8311
Listeria_seeligeri_1640_v1	Listeria_monocytogenes_1639_v142	0.8966
Listeria_seeligeri_1640	Listeria_monocytogenes_1639_v142	0.8888
Listeria_monocytogenes_WSLC1042_1457188	Listeria_monocytogenes_1639_v142	0.9716
Listeria_monocytogenes_WSLC1001_1457187	Listeria_monocytogenes_1639_v142	0.9382
Listeria_monocytogenes_SLCC7179_879090	Listeria_monocytogenes_1639_v142	0.943
Listeria_monocytogenes_SLCC5850_882096	Listeria_monocytogenes_1639_v142	0.9429
Listeria_monocytogenes_SLCC2755_932919	Listeria_monocytogenes_1639_v142	0.9532
Listeria_monocytogenes_SLCC2540_879089	Listeria_monocytogenes_1639_v142	0.9534
Listeria_monocytogenes_SLCC2479_882020	Listeria_monocytogenes_1639_v142	0.9333
Listeria_monocytogenes_SLCC2378_879088	Listeria_monocytogenes_1639_v142	0.9728
Listeria_monocytogenes_SLCC2376_882097	Listeria_monocytogenes_1639_v142	0.9504
Listeria_monocytogenes_SLCC2372_932920	Listeria_monocytogenes_1639_v142	0.9259
Listeria_monocytogenes_serotype_4b_str_LL195_1230340	Listeria_monocytogenes_1639_v142	0.9791
Listeria_monocytogenes_serotype_4b_str_LIS0087_1134669	Listeria_monocytogenes_1639_v142	0.9184
Listeria_monocytogenes_serotype_4b_str_F2365_265669	Listeria_monocytogenes_1639_v142	0.9791
Listeria_monocytogenes_serotype_4b_str_CLIP_80459_568819	Listeria_monocytogenes_1639_v142	0.9988
Listeria_monocytogenes_serotype_4b_str_81-0861_1196161	Listeria_monocytogenes_1639_v142	0.9609
Listeria_monocytogenes_serotype_4b_str_81-0592_1196160	Listeria_monocytogenes_1639_v142	0.962
Listeria_monocytogenes_serotype_4b_str_81-0558_1196159	Listeria_monocytogenes_1639_v142	0.9631
Listeria_monocytogenes_serotype_4b_str_10-0809_1196162	Listeria_monocytogenes_1639_v142	0.9624
Listeria_monocytogenes_serotype_4b_str_02-6680_1196175	Listeria_monocytogenes_1639_v142	0.9815
Listeria_monocytogenes_serotype_4b_str_02-6679_1196174	Listeria_monocytogenes_1639_v142	0.9815
Listeria_monocytogenes_serotype_4b_str_02-1792_1196173	Listeria_monocytogenes_1639_v142	0.9628
Listeria_monocytogenes_serotype_4b_str_02-1289_1196172	Listeria_monocytogenes_1639_v142	0.9628
Listeria_monocytogenes_serotype_4b_str_02-1103_1196171	Listeria_monocytogenes_1639_v142	0.9628
Listeria_monocytogenes_serotype_3c_str_10-5027_1196195	Listeria_monocytogenes_1639_v142	0.9325
Listeria_monocytogenes_serotype_1_2c_str_10-5026_1196194	Listeria_monocytogenes_1639_v142	0.9337
Listeria_monocytogenes_serotype_1_2c_str_10-5025_1196193	Listeria_monocytogenes_1639_v142	0.9337
Listeria_monocytogenes_serotype_1_2b_str_10-0811_1196164	Listeria_monocytogenes_1639_v142	0.946
Listeria_monocytogenes_serotype_1_2b_str_10-0810_1196163	Listeria_monocytogenes_1639_v142	0.946
Listeria_monocytogenes_serotype_1_2a_str_99-6370_1196179	Listeria_monocytogenes_1639_v142	0.9233
Listeria_monocytogenes_serotype_1_2a_str_98-2035_1196178	Listeria_monocytogenes_1639_v142	0.9233
Listeria_monocytogenes_serotype_1_2a_str_95-0093_1196177	Listeria_monocytogenes_1639_v142	0.9289
Listeria_monocytogenes_serotype_1_2a_str_88-0478_1196176	Listeria_monocytogenes_1639_v142	0.9281
Listeria_monocytogenes_serotype_1_2a_str_10-5024_1196192	Listeria_monocytogenes_1639_v142	0.9292
Listeria_monocytogenes_serotype_1_2a_str_10-4758_1196168	Listeria_monocytogenes_1639_v142	0.9437
Listeria_monocytogenes_serotype_1_2a_str_10-4754_1196167	Listeria_monocytogenes_1639_v142	0.9433
Listeria_monocytogenes_serotype_1_2a_str_10-1321_1196191	Listeria_monocytogenes_1639_v142	0.9292
Listeria_monocytogenes_serotype_1_2a_str_10-1047_1196190	Listeria_monocytogenes_1639_v142	0.9296
Listeria_monocytogenes_serotype_1_2a_str_10-1046_1196189	Listeria_monocytogenes_1639_v142	0.9296
Listeria_monocytogenes_serotype_1_2a_str_10-0934_1196170	Listeria_monocytogenes_1639_v142	0.9531
Listeria_monocytogenes_serotype_1_2a_str_10-0933_1196169	Listeria_monocytogenes_1639_v142	0.9531
Listeria_monocytogenes_serotype_1_2a_str_10-0815_1196188	Listeria_monocytogenes_1639_v142	0.9247
Listeria_monocytogenes_serotype_1_2a_str_10-0814_1196187	Listeria_monocytogenes_1639_v142	0.9292
Listeria_monocytogenes_serotype_1_2a_str_10-0813_1196166	Listeria_monocytogenes_1639_v142	0.9353
Listeria_monocytogenes_serotype_1_2a_str_10-0812_1196165	Listeria_monocytogenes_1639_v142	0.9353
Listeria_monocytogenes_serotype_1_2a_str_08-7669_1196186	Listeria_monocytogenes_1639_v142	0.9384
Listeria_monocytogenes_serotype_1_2a_str_08-7374_1196185	Listeria_monocytogenes_1639_v142	0.9292
Listeria_monocytogenes_serotype_1_2a_str_08-6997_1196184	Listeria_monocytogenes_1639_v142	0.9247
Listeria_monocytogenes_serotype_1_2a_str_08-6569_1196183	Listeria_monocytogenes_1639_v142	0.9247
Listeria_monocytogenes_serotype_1_2a_str_08-6056_1196182	Listeria_monocytogenes_1639_v142	0.9247
Listeria_monocytogenes_serotype_1_2a_str_04-5457_1196181	Listeria_monocytogenes_1639_v142	0.9292
Listeria_monocytogenes_serotype_1_2a_str_02-5993_1196180	Listeria_monocytogenes_1639_v142	0.9289
Listeria_monocytogenes_R479a_1437838	Listeria_monocytogenes_1639_v142	0.9282
Listeria_monocytogenes_N53-1_1234142	Listeria_monocytogenes_1639_v142	0.898
Listeria_monocytogenes_M7_1030009	Listeria_monocytogenes_1639_v142	0.9151
Listeria_monocytogenes_LO28_393132	Listeria_monocytogenes_1639_v142	0.9319
Listeria_monocytogenes_L99_563174	Listeria_monocytogenes_1639_v142	0.9158
Listeria_monocytogenes_L312_882094	Listeria_monocytogenes_1639_v142	0.9992
Listeria_monocytogenes_J1816_930781	Listeria_monocytogenes_1639_v142	0.9569
Listeria_monocytogenes_J1-220_930782	Listeria_monocytogenes_1639_v142	0.9525
Listeria_monocytogenes_J0161_393130	Listeria_monocytogenes_1639_v142	0.9357
Listeria_monocytogenes_HCC23_552536	Listeria_monocytogenes_1639_v142	0.915
Listeria_monocytogenes_FSL_R2-561_393126	Listeria_monocytogenes_1639_v142	0.9319
Listeria_monocytogenes_FSL_J1-208_393119	Listeria_monocytogenes_1639_v142	0.9225
Listeria_monocytogenes_FSL_J1-175_393118	Listeria_monocytogenes_1639_v142	0.9567
Listeria_monocytogenes_Finland_1998_393127	Listeria_monocytogenes_1639_v142	0.9538
Listeria_monocytogenes_EGD-e_169963_v1	Listeria_monocytogenes_1639_v142	0.9411
Listeria_monocytogenes_EGD-e_169963	Listeria_monocytogenes_1639_v142	0.9415
Listeria_monocytogenes_EGD_1334565	Listeria_monocytogenes_1639_v142	0.9436
Listeria_monocytogenes_CFSAN002349_1299895	Listeria_monocytogenes_1639_v142	0.9803
Listeria_monocytogenes_ATCC_19117_882095_v1	Listeria_monocytogenes_1639_v142	0.9727
Listeria_monocytogenes_ATCC_19117_882095	Listeria_monocytogenes_1639_v142	0.9727
Listeria_monocytogenes_6179_1288295	Listeria_monocytogenes_1639_v142	0.9153
Listeria_monocytogenes_1639_v9	Listeria_monocytogenes_1639_v142	0.9275
Listeria_monocytogenes_1639_v99	Listeria_monocytogenes_1639_v142	0.958

Listeria_monocytogenes_1639_v98	Listeria_monocytogenes_1639_v142	0.9337
Listeria_monocytogenes_1639_v97	Listeria_monocytogenes_1639_v142	0.9672
Listeria_monocytogenes_1639_v96	Listeria_monocytogenes_1639_v142	0.9569
Listeria_monocytogenes_1639_v95	Listeria_monocytogenes_1639_v142	0.9569
Listeria_monocytogenes_1639_v94	Listeria_monocytogenes_1639_v142	0.9573
Listeria_monocytogenes_1639_v93	Listeria_monocytogenes_1639_v142	0.9277
Listeria_monocytogenes_1639_v92	Listeria_monocytogenes_1639_v142	0.9577
Listeria_monocytogenes_1639_v91	Listeria_monocytogenes_1639_v142	0.9602
Listeria_monocytogenes_1639_v90	Listeria_monocytogenes_1639_v142	0.9337
Listeria_monocytogenes_1639_v8	Listeria_monocytogenes_1639_v142	0.9178
Listeria_monocytogenes_1639_v89	Listeria_monocytogenes_1639_v142	0.9675
Listeria_monocytogenes_1639_v88	Listeria_monocytogenes_1639_v142	0.9339
Listeria_monocytogenes_1639_v87	Listeria_monocytogenes_1639_v142	0.9311
Listeria_monocytogenes_1639_v86	Listeria_monocytogenes_1639_v142	0.9304
Listeria_monocytogenes_1639_v85	Listeria_monocytogenes_1639_v142	0.9606
Listeria_monocytogenes_1639_v84	Listeria_monocytogenes_1639_v142	0.9406
Listeria_monocytogenes_1639_v83	Listeria_monocytogenes_1639_v142	0.9612
Listeria_monocytogenes_1639_v82	Listeria_monocytogenes_1639_v142	0.9609
Listeria_monocytogenes_1639_v81	Listeria_monocytogenes_1639_v142	0.9342
Listeria_monocytogenes_1639_v80	Listeria_monocytogenes_1639_v142	0.9509
Listeria_monocytogenes_1639_v7	Listeria_monocytogenes_1639_v142	0.9178
Listeria_monocytogenes_1639_v79	Listeria_monocytogenes_1639_v142	0.9321
Listeria_monocytogenes_1639_v78	Listeria_monocytogenes_1639_v142	0.9335
Listeria_monocytogenes_1639_v77	Listeria_monocytogenes_1639_v142	0.9318
Listeria_monocytogenes_1639_v76	Listeria_monocytogenes_1639_v142	0.9273
Listeria_monocytogenes_1639_v75	Listeria_monocytogenes_1639_v142	0.9158
Listeria_monocytogenes_1639_v74	Listeria_monocytogenes_1639_v142	0.9292
Listeria_monocytogenes_1639_v73	Listeria_monocytogenes_1639_v142	0.927
Listeria_monocytogenes_1639_v72	Listeria_monocytogenes_1639_v142	0.9604
Listeria_monocytogenes_1639_v71	Listeria_monocytogenes_1639_v142	0.9189
Listeria_monocytogenes_1639_v70	Listeria_monocytogenes_1639_v142	0.9298
Listeria_monocytogenes_1639_v6	Listeria_monocytogenes_1639_v142	0.9171
Listeria_monocytogenes_1639_v69	Listeria_monocytogenes_1639_v142	0.9624
Listeria_monocytogenes_1639_v68	Listeria_monocytogenes_1639_v142	0.9395
Listeria_monocytogenes_1639_v67	Listeria_monocytogenes_1639_v142	0.9491
Listeria_monocytogenes_1639_v66	Listeria_monocytogenes_1639_v142	0.938
Listeria_monocytogenes_1639_v65	Listeria_monocytogenes_1639_v142	0.9324
Listeria_monocytogenes_1639_v64	Listeria_monocytogenes_1639_v142	0.9336
Listeria_monocytogenes_1639_v63	Listeria_monocytogenes_1639_v142	0.9467
Listeria_monocytogenes_1639_v62	Listeria_monocytogenes_1639_v142	0.93
Listeria_monocytogenes_1639_v61	Listeria_monocytogenes_1639_v142	0.932
Listeria_monocytogenes_1639_v60	Listeria_monocytogenes_1639_v142	0.9376
Listeria_monocytogenes_1639_v5	Listeria_monocytogenes_1639_v142	0.911
Listeria_monocytogenes_1639_v59	Listeria_monocytogenes_1639_v142	0.9623
Listeria_monocytogenes_1639_v58	Listeria_monocytogenes_1639_v142	0.9608
Listeria_monocytogenes_1639_v57	Listeria_monocytogenes_1639_v142	0.9537
Listeria_monocytogenes_1639_v56	Listeria_monocytogenes_1639_v142	0.9616
Listeria_monocytogenes_1639_v55	Listeria_monocytogenes_1639_v142	0.9498
Listeria_monocytogenes_1639_v54	Listeria_monocytogenes_1639_v142	0.9268
Listeria_monocytogenes_1639_v53	Listeria_monocytogenes_1639_v142	0.935
Listeria_monocytogenes_1639_v52	Listeria_monocytogenes_1639_v142	0.9587
Listeria_monocytogenes_1639_v51	Listeria_monocytogenes_1639_v142	0.9557
Listeria_monocytogenes_1639_v50	Listeria_monocytogenes_1639_v142	0.9285
Listeria_monocytogenes_1639_v4	Listeria_monocytogenes_1639_v142	0.9241
Listeria_monocytogenes_1639_v49	Listeria_monocytogenes_1639_v142	0.962
Listeria_monocytogenes_1639_v48	Listeria_monocytogenes_1639_v142	0.9277
Listeria_monocytogenes_1639_v47	Listeria_monocytogenes_1639_v142	0.9289
Listeria_monocytogenes_1639_v46	Listeria_monocytogenes_1639_v142	0.9388
Listeria_monocytogenes_1639_v45	Listeria_monocytogenes_1639_v142	0.9196
Listeria_monocytogenes_1639_v44	Listeria_monocytogenes_1639_v142	0.9292
Listeria_monocytogenes_1639_v43	Listeria_monocytogenes_1639_v142	0.9292
Listeria_monocytogenes_1639_v42	Listeria_monocytogenes_1639_v142	0.9214
Listeria_monocytogenes_1639_v41	Listeria_monocytogenes_1639_v142	0.9094
Listeria_monocytogenes_1639_v40	Listeria_monocytogenes_1639_v142	0.9322
Listeria_monocytogenes_1639_v3	Listeria_monocytogenes_1639_v142	0.9244
Listeria_monocytogenes_1639_v39	Listeria_monocytogenes_1639_v142	0.944
Listeria_monocytogenes_1639_v38	Listeria_monocytogenes_1639_v142	0.9294
Listeria_monocytogenes_1639_v37	Listeria_monocytogenes_1639_v142	0.9245
Listeria_monocytogenes_1639_v36	Listeria_monocytogenes_1639_v142	0.9232
Listeria_monocytogenes_1639_v35	Listeria_monocytogenes_1639_v142	0.9329
Listeria_monocytogenes_1639_v34	Listeria_monocytogenes_1639_v142	0.9318
Listeria_monocytogenes_1639_v33	Listeria_monocytogenes_1639_v142	0.946
Listeria_monocytogenes_1639_v32	Listeria_monocytogenes_1639_v142	0.9235
Listeria_monocytogenes_1639_v31	Listeria_monocytogenes_1639_v142	0.9322
Listeria_monocytogenes_1639_v30	Listeria_monocytogenes_1639_v142	0.9452
Listeria_monocytogenes_1639_v2	Listeria_monocytogenes_1639_v142	0.9106
Listeria_monocytogenes_1639_v29	Listeria_monocytogenes_1639_v142	0.9187
Listeria_monocytogenes_1639_v28	Listeria_monocytogenes_1639_v142	0.9231
Listeria_monocytogenes_1639_v27	Listeria_monocytogenes_1639_v142	0.9139
Listeria_monocytogenes_1639_v26	Listeria_monocytogenes_1639_v142	0.9139
Listeria_monocytogenes_1639_v25	Listeria_monocytogenes_1639_v142	0.9136
Listeria_monocytogenes_1639_v24	Listeria_monocytogenes_1639_v142	0.9525
Listeria_monocytogenes_1639_v23	Listeria_monocytogenes_1639_v142	0.9525
Listeria_monocytogenes_1639_v22	Listeria_monocytogenes_1639_v142	0.9475
Listeria_monocytogenes_1639_v21	Listeria_monocytogenes_1639_v142	0.9522
Listeria_monocytogenes_1639_v20	Listeria_monocytogenes_1639_v142	0.9407
Listeria_monocytogenes_1639_v1	Listeria_monocytogenes_1639_v142	0.9086
Listeria_monocytogenes_1639_v19	Listeria_monocytogenes_1639_v142	0.9404
Listeria_monocytogenes_1639_v198	Listeria_monocytogenes_1639_v142	0.9015
Listeria_monocytogenes_1639_v197	Listeria_monocytogenes_1639_v142	0.9294
Listeria_monocytogenes_1639_v196	Listeria_monocytogenes_1639_v142	0.9263

Listeria_monocytogenes_1639_v195	Listeria_monocytogenes_1639_v142	0.9507
Listeria_monocytogenes_1639_v194	Listeria_monocytogenes_1639_v142	0.9512
Listeria_monocytogenes_1639_v193	Listeria_monocytogenes_1639_v142	0.9512
Listeria_monocytogenes_1639_v192	Listeria_monocytogenes_1639_v142	0.915
Listeria_monocytogenes_1639_v191	Listeria_monocytogenes_1639_v142	0.9568
Listeria_monocytogenes_1639_v190	Listeria_monocytogenes_1639_v142	0.9549
Listeria_monocytogenes_1639_v18	Listeria_monocytogenes_1639_v142	0.9477
Listeria_monocytogenes_1639_v189	Listeria_monocytogenes_1639_v142	0.9542
Listeria_monocytogenes_1639_v188	Listeria_monocytogenes_1639_v142	0.9579
Listeria_monocytogenes_1639_v187	Listeria_monocytogenes_1639_v142	0.9545
Listeria_monocytogenes_1639_v186	Listeria_monocytogenes_1639_v142	0.9553
Listeria_monocytogenes_1639_v185	Listeria_monocytogenes_1639_v142	0.9549
Listeria_monocytogenes_1639_v184	Listeria_monocytogenes_1639_v142	0.9541
Listeria_monocytogenes_1639_v183	Listeria_monocytogenes_1639_v142	0.9526
Listeria_monocytogenes_1639_v182	Listeria_monocytogenes_1639_v142	0.9544
Listeria_monocytogenes_1639_v181	Listeria_monocytogenes_1639_v142	0.9404
Listeria_monocytogenes_1639_v180	Listeria_monocytogenes_1639_v142	0.9404
Listeria_monocytogenes_1639_v17	Listeria_monocytogenes_1639_v142	0.9477
Listeria_monocytogenes_1639_v179	Listeria_monocytogenes_1639_v142	0.9396
Listeria_monocytogenes_1639_v178	Listeria_monocytogenes_1639_v142	0.9534
Listeria_monocytogenes_1639_v177	Listeria_monocytogenes_1639_v142	0.9773
Listeria_monocytogenes_1639_v176	Listeria_monocytogenes_1639_v142	0.9481
Listeria_monocytogenes_1639_v175	Listeria_monocytogenes_1639_v142	0.9434
Listeria_monocytogenes_1639_v174	Listeria_monocytogenes_1639_v142	0.9554
Listeria_monocytogenes_1639_v173	Listeria_monocytogenes_1639_v142	0.9709
Listeria_monocytogenes_1639_v172	Listeria_monocytogenes_1639_v142	0.9456
Listeria_monocytogenes_1639_v171	Listeria_monocytogenes_1639_v142	0.94
Listeria_monocytogenes_1639_v170	Listeria_monocytogenes_1639_v142	0.9483
Listeria_monocytogenes_1639_v16	Listeria_monocytogenes_1639_v142	0.9155
Listeria_monocytogenes_1639_v169	Listeria_monocytogenes_1639_v142	0.9507
Listeria_monocytogenes_1639_v168	Listeria_monocytogenes_1639_v142	0.9492
Listeria_monocytogenes_1639_v167	Listeria_monocytogenes_1639_v142	0.9413
Listeria_monocytogenes_1639_v166	Listeria_monocytogenes_1639_v142	0.9496
Listeria_monocytogenes_1639_v165	Listeria_monocytogenes_1639_v142	0.9507
Listeria_monocytogenes_1639_v164	Listeria_monocytogenes_1639_v142	0.9783
Listeria_monocytogenes_1639_v163	Listeria_monocytogenes_1639_v142	0.9676
Listeria_monocytogenes_1639_v162	Listeria_monocytogenes_1639_v142	0.9783
Listeria_monocytogenes_1639_v161	Listeria_monocytogenes_1639_v142	0.9795
Listeria_monocytogenes_1639_v160	Listeria_monocytogenes_1639_v142	0.9799
Listeria_monocytogenes_1639_v15	Listeria_monocytogenes_1639_v142	0.9306
Listeria_monocytogenes_1639_v159	Listeria_monocytogenes_1639_v142	0.9773
Listeria_monocytogenes_1639_v158	Listeria_monocytogenes_1639_v142	0.9795
Listeria_monocytogenes_1639_v157	Listeria_monocytogenes_1639_v142	0.9791
Listeria_monocytogenes_1639_v156	Listeria_monocytogenes_1639_v142	0.9314
Listeria_monocytogenes_1639_v155	Listeria_monocytogenes_1639_v142	0.9458
Listeria_monocytogenes_1639_v154	Listeria_monocytogenes_1639_v142	0.9481
Listeria_monocytogenes_1639_v153	Listeria_monocytogenes_1639_v142	0.9465
Listeria_monocytogenes_1639_v152	Listeria_monocytogenes_1639_v142	0.9776
Listeria_monocytogenes_1639_v151	Listeria_monocytogenes_1639_v142	0.9776
Listeria_monocytogenes_1639_v150	Listeria_monocytogenes_1639_v142	0.9446
Listeria_monocytogenes_1639_v14	Listeria_monocytogenes_1639_v142	0.9375
Listeria_monocytogenes_1639_v149	Listeria_monocytogenes_1639_v142	0.9436
Listeria_monocytogenes_1639_v148	Listeria_monocytogenes_1639_v142	0.9761
Listeria_monocytogenes_1639_v147	Listeria_monocytogenes_1639_v142	0.9617
Listeria_monocytogenes_1639_v146	Listeria_monocytogenes_1639_v142	0.9562
Listeria_monocytogenes_1639_v145	Listeria_monocytogenes_1639_v142	0.9502
Listeria_monocytogenes_1639_v144	Listeria_monocytogenes_1639_v142	0.9988
Listeria_monocytogenes_1639_v143	Listeria_monocytogenes_1639_v142	0.9532
Listeria_monocytogenes_1639_v142	Listeria_monocytogenes_1639_v142	0.0653
Listeria_welshimeri_1643	Listeria_monocytogenes_1639_v143	0.878
Listeria_welshimeri_serovar_6b_str_SLCC5334_386043	Listeria_monocytogenes_1639_v143	0.8774
Listeria_seeligeri_serovar_1_2b_str_SLCC3954_683837	Listeria_monocytogenes_1639_v143	0.8908
Listeria_seeligeri_FSL_S4-171_702452	Listeria_monocytogenes_1639_v143	0.8213
Listeria_seeligeri_FSL_N1-067_702453	Listeria_monocytogenes_1639_v143	0.8363
Listeria_seeligeri_1640_v1	Listeria_monocytogenes_1639_v143	0.8946
Listeria_seeligeri_1640	Listeria_monocytogenes_1639_v143	0.8837
Listeria_monocytogenes_WSLC1042_1457188	Listeria_monocytogenes_1639_v143	0.9471
Listeria_monocytogenes_WSLC1001_1457187	Listeria_monocytogenes_1639_v143	0.9564
Listeria_monocytogenes_SLCC7179_879090	Listeria_monocytogenes_1639_v143	0.9672
Listeria_monocytogenes_SLCC5850_882096	Listeria_monocytogenes_1639_v143	0.9608
Listeria_monocytogenes_SLCC2755_932919	Listeria_monocytogenes_1639_v143	0.9448
Listeria_monocytogenes_SLCC2540_879089	Listeria_monocytogenes_1639_v143	0.9472
Listeria_monocytogenes_SLCC2479_882020	Listeria_monocytogenes_1639_v143	0.9545
Listeria_monocytogenes_SLCC2378_879088	Listeria_monocytogenes_1639_v143	0.9485
Listeria_monocytogenes_SLCC2376_882097	Listeria_monocytogenes_1639_v143	0.948
Listeria_monocytogenes_SLCC2372_932920	Listeria_monocytogenes_1639_v143	0.9477
Listeria_monocytogenes_serotype_4b_str_LL195_1230340	Listeria_monocytogenes_1639_v143	0.9544
Listeria_monocytogenes_serotype_4b_str_LIS0087_1134669	Listeria_monocytogenes_1639_v143	0.9373
Listeria_monocytogenes_serotype_4b_str_F2365_265669	Listeria_monocytogenes_1639_v143	0.9544
Listeria_monocytogenes_serotype_4b_str_CLIP_80459_568819	Listeria_monocytogenes_1639_v143	0.9535
Listeria_monocytogenes_serotype_4b_str_81-0861_1196161	Listeria_monocytogenes_1639_v143	0.9373
Listeria_monocytogenes_serotype_4b_str_81-0592_1196160	Listeria_monocytogenes_1639_v143	0.9384
Listeria_monocytogenes_serotype_4b_str_81-0558_1196159	Listeria_monocytogenes_1639_v143	0.9384
Listeria_monocytogenes_serotype_4b_str_10-0809_1196162	Listeria_monocytogenes_1639_v143	0.9384
Listeria_monocytogenes_serotype_4b_str_02-6680_1196175	Listeria_monocytogenes_1639_v143	0.9494
Listeria_monocytogenes_serotype_4b_str_02-6679_1196174	Listeria_monocytogenes_1639_v143	0.9494
Listeria_monocytogenes_serotype_4b_str_02-1792_1196173	Listeria_monocytogenes_1639_v143	0.9388
Listeria_monocytogenes_serotype_4b_str_02-1289_1196172	Listeria_monocytogenes_1639_v143	0.9388
Listeria_monocytogenes_serotype_4b_str_02-1103_1196171	Listeria_monocytogenes_1639_v143	0.9388
Listeria_monocytogenes_serotype_3c_str_10-5027_1196195	Listeria_monocytogenes_1639_v143	0.9537
Listeria_monocytogenes_serotype_1_2c_str_10-5026_1196194	Listeria_monocytogenes_1639_v143	0.9548

Listeria_monocytogenes_serotype_1_2c_str_10-5025_1196193	Listeria_monocytogenes_1639_v143	0.9548
Listeria_monocytogenes_serotype_1_2b_str_10-0811_1196164	Listeria_monocytogenes_1639_v143	0.9373
Listeria_monocytogenes_serotype_1_2b_str_10-0810_1196163	Listeria_monocytogenes_1639_v143	0.9373
Listeria_monocytogenes_serotype_1_2a_str_99-6370_1196179	Listeria_monocytogenes_1639_v143	0.9371
Listeria_monocytogenes_serotype_1_2a_str_98-2035_1196178	Listeria_monocytogenes_1639_v143	0.9371
Listeria_monocytogenes_serotype_1_2a_str_95-0093_1196177	Listeria_monocytogenes_1639_v143	0.9431
Listeria_monocytogenes_serotype_1_2a_str_88-0478_1196176	Listeria_monocytogenes_1639_v143	0.9423
Listeria_monocytogenes_serotype_1_2a_str_10-5024_1196192	Listeria_monocytogenes_1639_v143	0.9435
Listeria_monocytogenes_serotype_1_2a_str_10-4758_1196168	Listeria_monocytogenes_1639_v143	0.969
Listeria_monocytogenes_serotype_1_2a_str_10-4754_1196167	Listeria_monocytogenes_1639_v143	0.969
Listeria_monocytogenes_serotype_1_2a_str_10-1321_1196191	Listeria_monocytogenes_1639_v143	0.9435
Listeria_monocytogenes_serotype_1_2a_str_10-1047_1196190	Listeria_monocytogenes_1639_v143	0.9438
Listeria_monocytogenes_serotype_1_2a_str_10-1046_1196189	Listeria_monocytogenes_1639_v143	0.9438
Listeria_monocytogenes_serotype_1_2a_str_10-0934_1196170	Listeria_monocytogenes_1639_v143	0.9748
Listeria_monocytogenes_serotype_1_2a_str_10-0933_1196169	Listeria_monocytogenes_1639_v143	0.9748
Listeria_monocytogenes_serotype_1_2a_str_10-0815_1196188	Listeria_monocytogenes_1639_v143	0.9385
Listeria_monocytogenes_serotype_1_2a_str_10-0814_1196187	Listeria_monocytogenes_1639_v143	0.9431
Listeria_monocytogenes_serotype_1_2a_str_10-0813_1196166	Listeria_monocytogenes_1639_v143	0.9535
Listeria_monocytogenes_serotype_1_2a_str_10-0812_1196165	Listeria_monocytogenes_1639_v143	0.9535
Listeria_monocytogenes_serotype_1_2a_str_08-7669_1196186	Listeria_monocytogenes_1639_v143	0.9532
Listeria_monocytogenes_serotype_1_2a_str_08-7374_1196185	Listeria_monocytogenes_1639_v143	0.9435
Listeria_monocytogenes_serotype_1_2a_str_08-6997_1196184	Listeria_monocytogenes_1639_v143	0.9385
Listeria_monocytogenes_serotype_1_2a_str_08-6569_1196183	Listeria_monocytogenes_1639_v143	0.9385
Listeria_monocytogenes_serotype_1_2a_str_08-6056_1196182	Listeria_monocytogenes_1639_v143	0.9385
Listeria_monocytogenes_serotype_1_2a_str_04-5457_1196181	Listeria_monocytogenes_1639_v143	0.9435
Listeria_monocytogenes_serotype_1_2a_str_02-5993_1196180	Listeria_monocytogenes_1639_v143	0.9431
Listeria_monocytogenes_R479a_1437838	Listeria_monocytogenes_1639_v143	0.9428
Listeria_monocytogenes_N53-1_1234142	Listeria_monocytogenes_1639_v143	0.9172
Listeria_monocytogenes_M7_1030009	Listeria_monocytogenes_1639_v143	0.9116
Listeria_monocytogenes_LO28_393132	Listeria_monocytogenes_1639_v143	0.953
Listeria_monocytogenes_L99_563174	Listeria_monocytogenes_1639_v143	0.9123
Listeria_monocytogenes_L312_882094	Listeria_monocytogenes_1639_v143	0.9535
Listeria_monocytogenes_J1816_930781	Listeria_monocytogenes_1639_v143	0.9322
Listeria_monocytogenes_J1-220_930782	Listeria_monocytogenes_1639_v143	0.928
Listeria_monocytogenes_J0161_393130	Listeria_monocytogenes_1639_v143	0.9568
Listeria_monocytogenes_HCC23_552536	Listeria_monocytogenes_1639_v143	0.9119
Listeria_monocytogenes_FSL_R2-561_393126	Listeria_monocytogenes_1639_v143	0.953
Listeria_monocytogenes_FSL_J1-208_393119	Listeria_monocytogenes_1639_v143	0.9159
Listeria_monocytogenes_FSL_J1-175_393118	Listeria_monocytogenes_1639_v143	0.9528
Listeria_monocytogenes_Finland_1998_393127	Listeria_monocytogenes_1639_v143	0.9736
Listeria_monocytogenes_EGD-e_169963_v1	Listeria_monocytogenes_1639_v143	0.9629
Listeria_monocytogenes_EGD-e_169963	Listeria_monocytogenes_1639_v143	0.9629
Listeria_monocytogenes_EGD_1334565	Listeria_monocytogenes_1639_v143	0.9616
Listeria_monocytogenes_CFSAN002349_1299895	Listeria_monocytogenes_1639_v143	0.9518
Listeria_monocytogenes_ATCC_19117_882095_v1	Listeria_monocytogenes_1639_v143	0.948
Listeria_monocytogenes_ATCC_19117_882095	Listeria_monocytogenes_1639_v143	0.948
Listeria_monocytogenes_6179_1288295	Listeria_monocytogenes_1639_v143	0.9436
Listeria_monocytogenes_1639_v9	Listeria_monocytogenes_1639_v143	0.9236
Listeria_monocytogenes_1639_v99	Listeria_monocytogenes_1639_v143	0.933
Listeria_monocytogenes_1639_v98	Listeria_monocytogenes_1639_v143	0.9465
Listeria_monocytogenes_1639_v97	Listeria_monocytogenes_1639_v143	0.9428
Listeria_monocytogenes_1639_v96	Listeria_monocytogenes_1639_v143	0.9323
Listeria_monocytogenes_1639_v95	Listeria_monocytogenes_1639_v143	0.9323
Listeria_monocytogenes_1639_v94	Listeria_monocytogenes_1639_v143	0.9323
Listeria_monocytogenes_1639_v93	Listeria_monocytogenes_1639_v143	0.9555
Listeria_monocytogenes_1639_v92	Listeria_monocytogenes_1639_v143	0.9327
Listeria_monocytogenes_1639_v91	Listeria_monocytogenes_1639_v143	0.9352
Listeria_monocytogenes_1639_v90	Listeria_monocytogenes_1639_v143	0.9465
Listeria_monocytogenes_1639_v8	Listeria_monocytogenes_1639_v143	0.9322
Listeria_monocytogenes_1639_v89	Listeria_monocytogenes_1639_v143	0.9425
Listeria_monocytogenes_1639_v88	Listeria_monocytogenes_1639_v143	0.9544
Listeria_monocytogenes_1639_v87	Listeria_monocytogenes_1639_v143	0.9488
Listeria_monocytogenes_1639_v86	Listeria_monocytogenes_1639_v143	0.9485
Listeria_monocytogenes_1639_v85	Listeria_monocytogenes_1639_v143	0.9366
Listeria_monocytogenes_1639_v84	Listeria_monocytogenes_1639_v143	0.9588
Listeria_monocytogenes_1639_v83	Listeria_monocytogenes_1639_v143	0.9368
Listeria_monocytogenes_1639_v82	Listeria_monocytogenes_1639_v143	0.9361
Listeria_monocytogenes_1639_v81	Listeria_monocytogenes_1639_v143	0.9536
Listeria_monocytogenes_1639_v80	Listeria_monocytogenes_1639_v143	0.9432
Listeria_monocytogenes_1639_v7	Listeria_monocytogenes_1639_v143	0.9322
Listeria_monocytogenes_1639_v79	Listeria_monocytogenes_1639_v143	0.9514
Listeria_monocytogenes_1639_v78	Listeria_monocytogenes_1639_v143	0.9529
Listeria_monocytogenes_1639_v77	Listeria_monocytogenes_1639_v143	0.958
Listeria_monocytogenes_1639_v76	Listeria_monocytogenes_1639_v143	0.9517
Listeria_monocytogenes_1639_v75	Listeria_monocytogenes_1639_v143	0.9123
Listeria_monocytogenes_1639_v74	Listeria_monocytogenes_1639_v143	0.9481
Listeria_monocytogenes_1639_v73	Listeria_monocytogenes_1639_v143	0.9484
Listeria_monocytogenes_1639_v72	Listeria_monocytogenes_1639_v143	0.9356
Listeria_monocytogenes_1639_v71	Listeria_monocytogenes_1639_v143	0.9406
Listeria_monocytogenes_1639_v70	Listeria_monocytogenes_1639_v143	0.9509
Listeria_monocytogenes_1639_v6	Listeria_monocytogenes_1639_v143	0.9315
Listeria_monocytogenes_1639_v69	Listeria_monocytogenes_1639_v143	0.9388
Listeria_monocytogenes_1639_v68	Listeria_monocytogenes_1639_v143	0.9577
Listeria_monocytogenes_1639_v67	Listeria_monocytogenes_1639_v143	0.9429
Listeria_monocytogenes_1639_v66	Listeria_monocytogenes_1639_v143	0.964
Listeria_monocytogenes_1639_v65	Listeria_monocytogenes_1639_v143	0.9463
Listeria_monocytogenes_1639_v64	Listeria_monocytogenes_1639_v143	0.9571
Listeria_monocytogenes_1639_v63	Listeria_monocytogenes_1639_v143	0.938
Listeria_monocytogenes_1639_v62	Listeria_monocytogenes_1639_v143	0.9492
Listeria_monocytogenes_1639_v61	Listeria_monocytogenes_1639_v143	0.9463
Listeria_monocytogenes_1639_v60	Listeria_monocytogenes_1639_v143	0.9589

Listeria_monocytogenes_1639_v5	Listeria_monocytogenes_1639_v143	0.904
Listeria_monocytogenes_1639_v59	Listeria_monocytogenes_1639_v143	0.9375
Listeria_monocytogenes_1639_v58	Listeria_monocytogenes_1639_v143	0.9383
Listeria_monocytogenes_1639_v57	Listeria_monocytogenes_1639_v143	0.9288
Listeria_monocytogenes_1639_v56	Listeria_monocytogenes_1639_v143	0.9375
Listeria_monocytogenes_1639_v55	Listeria_monocytogenes_1639_v143	0.925
Listeria_monocytogenes_1639_v54	Listeria_monocytogenes_1639_v143	0.9302
Listeria_monocytogenes_1639_v53	Listeria_monocytogenes_1639_v143	0.9589
Listeria_monocytogenes_1639_v52	Listeria_monocytogenes_1639_v143	0.9343
Listeria_monocytogenes_1639_v51	Listeria_monocytogenes_1639_v143	0.9322
Listeria_monocytogenes_1639_v50	Listeria_monocytogenes_1639_v143	0.942
Listeria_monocytogenes_1639_v4	Listeria_monocytogenes_1639_v143	0.917
Listeria_monocytogenes_1639_v49	Listeria_monocytogenes_1639_v143	0.9373
Listeria_monocytogenes_1639_v48	Listeria_monocytogenes_1639_v143	0.9487
Listeria_monocytogenes_1639_v47	Listeria_monocytogenes_1639_v143	0.9431
Listeria_monocytogenes_1639_v46	Listeria_monocytogenes_1639_v143	0.957
Listeria_monocytogenes_1639_v45	Listeria_monocytogenes_1639_v143	0.9336
Listeria_monocytogenes_1639_v44	Listeria_monocytogenes_1639_v143	0.9435
Listeria_monocytogenes_1639_v43	Listeria_monocytogenes_1639_v143	0.9435
Listeria_monocytogenes_1639_v42	Listeria_monocytogenes_1639_v143	0.9442
Listeria_monocytogenes_1639_v41	Listeria_monocytogenes_1639_v143	0.9252
Listeria_monocytogenes_1639_v40	Listeria_monocytogenes_1639_v143	0.9534
Listeria_monocytogenes_1639_v3	Listeria_monocytogenes_1639_v143	0.9173
Listeria_monocytogenes_1639_v39	Listeria_monocytogenes_1639_v143	0.9398
Listeria_monocytogenes_1639_v38	Listeria_monocytogenes_1639_v143	0.9501
Listeria_monocytogenes_1639_v37	Listeria_monocytogenes_1639_v143	0.9459
Listeria_monocytogenes_1639_v36	Listeria_monocytogenes_1639_v143	0.9434
Listeria_monocytogenes_1639_v35	Listeria_monocytogenes_1639_v143	0.9537
Listeria_monocytogenes_1639_v34	Listeria_monocytogenes_1639_v143	0.9491
Listeria_monocytogenes_1639_v33	Listeria_monocytogenes_1639_v143	0.9373
Listeria_monocytogenes_1639_v32	Listeria_monocytogenes_1639_v143	0.9444
Listeria_monocytogenes_1639_v31	Listeria_monocytogenes_1639_v143	0.9494
Listeria_monocytogenes_1639_v30	Listeria_monocytogenes_1639_v143	0.9417
Listeria_monocytogenes_1639_v2	Listeria_monocytogenes_1639_v143	0.9029
Listeria_monocytogenes_1639_v29	Listeria_monocytogenes_1639_v143	0.9399
Listeria_monocytogenes_1639_v28	Listeria_monocytogenes_1639_v143	0.9441
Listeria_monocytogenes_1639_v27	Listeria_monocytogenes_1639_v143	0.9354
Listeria_monocytogenes_1639_v26	Listeria_monocytogenes_1639_v143	0.935
Listeria_monocytogenes_1639_v25	Listeria_monocytogenes_1639_v143	0.9347
Listeria_monocytogenes_1639_v24	Listeria_monocytogenes_1639_v143	0.928
Listeria_monocytogenes_1639_v23	Listeria_monocytogenes_1639_v143	0.928
Listeria_monocytogenes_1639_v22	Listeria_monocytogenes_1639_v143	0.9395
Listeria_monocytogenes_1639_v21	Listeria_monocytogenes_1639_v143	0.928
Listeria_monocytogenes_1639_v20	Listeria_monocytogenes_1639_v143	0.9324
Listeria_monocytogenes_1639_v1	Listeria_monocytogenes_1639_v143	0.937
Listeria_monocytogenes_1639_v19	Listeria_monocytogenes_1639_v143	0.9321
Listeria_monocytogenes_1639_v198	Listeria_monocytogenes_1639_v143	0.9196
Listeria_monocytogenes_1639_v197	Listeria_monocytogenes_1639_v143	0.9501
Listeria_monocytogenes_1639_v196	Listeria_monocytogenes_1639_v143	0.9446
Listeria_monocytogenes_1639_v195	Listeria_monocytogenes_1639_v143	0.9507
Listeria_monocytogenes_1639_v194	Listeria_monocytogenes_1639_v143	0.9484
Listeria_monocytogenes_1639_v193	Listeria_monocytogenes_1639_v143	0.9488
Listeria_monocytogenes_1639_v192	Listeria_monocytogenes_1639_v143	0.9069
Listeria_monocytogenes_1639_v191	Listeria_monocytogenes_1639_v143	0.979
Listeria_monocytogenes_1639_v190	Listeria_monocytogenes_1639_v143	0.9747
Listeria_monocytogenes_1639_v18	Listeria_monocytogenes_1639_v143	0.9233
Listeria_monocytogenes_1639_v189	Listeria_monocytogenes_1639_v143	0.9736
Listeria_monocytogenes_1639_v188	Listeria_monocytogenes_1639_v143	0.9723
Listeria_monocytogenes_1639_v187	Listeria_monocytogenes_1639_v143	0.9743
Listeria_monocytogenes_1639_v186	Listeria_monocytogenes_1639_v143	0.9747
Listeria_monocytogenes_1639_v185	Listeria_monocytogenes_1639_v143	0.9747
Listeria_monocytogenes_1639_v184	Listeria_monocytogenes_1639_v143	0.9743
Listeria_monocytogenes_1639_v183	Listeria_monocytogenes_1639_v143	0.9763
Listeria_monocytogenes_1639_v182	Listeria_monocytogenes_1639_v143	0.9786
Listeria_monocytogenes_1639_v181	Listeria_monocytogenes_1639_v143	0.9303
Listeria_monocytogenes_1639_v180	Listeria_monocytogenes_1639_v143	0.9303
Listeria_monocytogenes_1639_v17	Listeria_monocytogenes_1639_v143	0.9233
Listeria_monocytogenes_1639_v179	Listeria_monocytogenes_1639_v143	0.9634
Listeria_monocytogenes_1639_v178	Listeria_monocytogenes_1639_v143	0.9786
Listeria_monocytogenes_1639_v177	Listeria_monocytogenes_1639_v143	0.951
Listeria_monocytogenes_1639_v176	Listeria_monocytogenes_1639_v143	0.9453
Listeria_monocytogenes_1639_v175	Listeria_monocytogenes_1639_v143	0.9665
Listeria_monocytogenes_1639_v174	Listeria_monocytogenes_1639_v143	0.9767
Listeria_monocytogenes_1639_v173	Listeria_monocytogenes_1639_v143	0.9643
Listeria_monocytogenes_1639_v172	Listeria_monocytogenes_1639_v143	0.9494
Listeria_monocytogenes_1639_v171	Listeria_monocytogenes_1639_v143	0.9598
Listeria_monocytogenes_1639_v170	Listeria_monocytogenes_1639_v143	0.9676
Listeria_monocytogenes_1639_v16	Listeria_monocytogenes_1639_v143	0.9295
Listeria_monocytogenes_1639_v169	Listeria_monocytogenes_1639_v143	0.9715
Listeria_monocytogenes_1639_v168	Listeria_monocytogenes_1639_v143	0.9724
Listeria_monocytogenes_1639_v167	Listeria_monocytogenes_1639_v143	0.9633
Listeria_monocytogenes_1639_v166	Listeria_monocytogenes_1639_v143	0.9728
Listeria_monocytogenes_1639_v165	Listeria_monocytogenes_1639_v143	0.9715
Listeria_monocytogenes_1639_v164	Listeria_monocytogenes_1639_v143	0.9535
Listeria_monocytogenes_1639_v163	Listeria_monocytogenes_1639_v143	0.9594
Listeria_monocytogenes_1639_v162	Listeria_monocytogenes_1639_v143	0.9535
Listeria_monocytogenes_1639_v161	Listeria_monocytogenes_1639_v143	0.9543
Listeria_monocytogenes_1639_v160	Listeria_monocytogenes_1639_v143	0.9544
Listeria_monocytogenes_1639_v15	Listeria_monocytogenes_1639_v143	0.9235
Listeria_monocytogenes_1639_v159	Listeria_monocytogenes_1639_v143	0.9514
Listeria_monocytogenes_1639_v158	Listeria_monocytogenes_1639_v143	0.9544

Listeria_monocytogenes_1639_v157	Listeria_monocytogenes_1639_v143	0.9544
Listeria_monocytogenes_1639_v156	Listeria_monocytogenes_1639_v143	0.9503
Listeria_monocytogenes_1639_v155	Listeria_monocytogenes_1639_v143	0.96
Listeria_monocytogenes_1639_v154	Listeria_monocytogenes_1639_v143	0.9627
Listeria_monocytogenes_1639_v153	Listeria_monocytogenes_1639_v143	0.9607
Listeria_monocytogenes_1639_v152	Listeria_monocytogenes_1639_v143	0.9517
Listeria_monocytogenes_1639_v151	Listeria_monocytogenes_1639_v143	0.9517
Listeria_monocytogenes_1639_v150	Listeria_monocytogenes_1639_v143	0.9634
Listeria_monocytogenes_1639_v14	Listeria_monocytogenes_1639_v143	0.9292
Listeria_monocytogenes_1639_v149	Listeria_monocytogenes_1639_v143	0.9616
Listeria_monocytogenes_1639_v148	Listeria_monocytogenes_1639_v143	0.9503
Listeria_monocytogenes_1639_v147	Listeria_monocytogenes_1639_v143	0.9366
Listeria_monocytogenes_1639_v146	Listeria_monocytogenes_1639_v143	0.9779
Listeria_monocytogenes_1639_v145	Listeria_monocytogenes_1639_v143	0.9745
Listeria_monocytogenes_1639_v144	Listeria_monocytogenes_1639_v143	0.9528
Listeria_monocytogenes_1639_v143	Listeria_monocytogenes_1639_v143	0.0694
Listeria_welshimeri_1643	Listeria_monocytogenes_1639_v144	0.8859
Listeria_welshimeri_serovar_6b_str_SLCC5334_386043	Listeria_monocytogenes_1639_v144	0.8852
Listeria_seeligeri_serovar_1_2b_str_SLCC3954_683837	Listeria_monocytogenes_1639_v144	0.8901
Listeria_seeligeri_FSL_S4-171_702452	Listeria_monocytogenes_1639_v144	0.833
Listeria_seeligeri_FSL_N1-067_702453	Listeria_monocytogenes_1639_v144	0.8311
Listeria_seeligeri_1640_v1	Listeria_monocytogenes_1639_v144	0.8962
Listeria_seeligeri_1640	Listeria_monocytogenes_1639_v144	0.8885
Listeria_monocytogenes_WSLC1042_1457188	Listeria_monocytogenes_1639_v144	0.9713
Listeria_monocytogenes_WSLC1001_1457187	Listeria_monocytogenes_1639_v144	0.9378
Listeria_monocytogenes_SLCC7179_879090	Listeria_monocytogenes_1639_v144	0.9426
Listeria_monocytogenes_SLCC5850_882096	Listeria_monocytogenes_1639_v144	0.9425
Listeria_monocytogenes_SLCC2755_932919	Listeria_monocytogenes_1639_v144	0.9529
Listeria_monocytogenes_SLCC2540_879089	Listeria_monocytogenes_1639_v144	0.953
Listeria_monocytogenes_SLCC2479_882020	Listeria_monocytogenes_1639_v144	0.9329
Listeria_monocytogenes_SLCC2378_879088	Listeria_monocytogenes_1639_v144	0.9724
Listeria_monocytogenes_SLCC2376_882097	Listeria_monocytogenes_1639_v144	0.95
Listeria_monocytogenes_SLCC2372_932920	Listeria_monocytogenes_1639_v144	0.9256
Listeria_monocytogenes_serotype_4b_str_LL195_1230340	Listeria_monocytogenes_1639_v144	0.9788
Listeria_monocytogenes_serotype_4b_str_LIS0087_1134669	Listeria_monocytogenes_1639_v144	0.918
Listeria_monocytogenes_serotype_4b_str_F2365_265669	Listeria_monocytogenes_1639_v144	0.9788
Listeria_monocytogenes_serotype_4b_str_CLIP_80459_568819	Listeria_monocytogenes_1639_v144	0.9985
Listeria_monocytogenes_serotype_4b_str_81-0861_1196161	Listeria_monocytogenes_1639_v144	0.9605
Listeria_monocytogenes_serotype_4b_str_81-0592_1196160	Listeria_monocytogenes_1639_v144	0.9617
Listeria_monocytogenes_serotype_4b_str_81-0558_1196159	Listeria_monocytogenes_1639_v144	0.9628
Listeria_monocytogenes_serotype_4b_str_10-0809_1196162	Listeria_monocytogenes_1639_v144	0.962
Listeria_monocytogenes_serotype_4b_str_02-6680_1196175	Listeria_monocytogenes_1639_v144	0.9811
Listeria_monocytogenes_serotype_4b_str_02-6679_1196174	Listeria_monocytogenes_1639_v144	0.9811
Listeria_monocytogenes_serotype_4b_str_02-1792_1196173	Listeria_monocytogenes_1639_v144	0.9624
Listeria_monocytogenes_serotype_4b_str_02-1289_1196172	Listeria_monocytogenes_1639_v144	0.9624
Listeria_monocytogenes_serotype_4b_str_02-1103_1196171	Listeria_monocytogenes_1639_v144	0.9624
Listeria_monocytogenes_serotype_3c_str_10-5027_1196195	Listeria_monocytogenes_1639_v144	0.9321
Listeria_monocytogenes_serotype_1_2c_str_10-5026_1196194	Listeria_monocytogenes_1639_v144	0.9333
Listeria_monocytogenes_serotype_1_2c_str_10-5025_1196193	Listeria_monocytogenes_1639_v144	0.9333
Listeria_monocytogenes_serotype_1_2b_str_10-0811_1196164	Listeria_monocytogenes_1639_v144	0.9457
Listeria_monocytogenes_serotype_1_2b_str_10-0810_1196163	Listeria_monocytogenes_1639_v144	0.9457
Listeria_monocytogenes_serotype_1_2a_str_99-6370_1196179	Listeria_monocytogenes_1639_v144	0.9233
Listeria_monocytogenes_serotype_1_2a_str_98-2035_1196178	Listeria_monocytogenes_1639_v144	0.9233
Listeria_monocytogenes_serotype_1_2a_str_95-0093_1196177	Listeria_monocytogenes_1639_v144	0.9289
Listeria_monocytogenes_serotype_1_2a_str_88-0478_1196176	Listeria_monocytogenes_1639_v144	0.9281
Listeria_monocytogenes_serotype_1_2a_str_10-5024_1196192	Listeria_monocytogenes_1639_v144	0.9292
Listeria_monocytogenes_serotype_1_2a_str_10-4758_1196168	Listeria_monocytogenes_1639_v144	0.9433
Listeria_monocytogenes_serotype_1_2a_str_10-4754_1196167	Listeria_monocytogenes_1639_v144	0.943
Listeria_monocytogenes_serotype_1_2a_str_10-1321_1196191	Listeria_monocytogenes_1639_v144	0.9292
Listeria_monocytogenes_serotype_1_2a_str_10-1047_1196190	Listeria_monocytogenes_1639_v144	0.9296
Listeria_monocytogenes_serotype_1_2a_str_10-1046_1196189	Listeria_monocytogenes_1639_v144	0.9296
Listeria_monocytogenes_serotype_1_2a_str_10-0934_1196170	Listeria_monocytogenes_1639_v144	0.9527
Listeria_monocytogenes_serotype_1_2a_str_10-0933_1196169	Listeria_monocytogenes_1639_v144	0.9527
Listeria_monocytogenes_serotype_1_2a_str_10-0815_1196188	Listeria_monocytogenes_1639_v144	0.9247
Listeria_monocytogenes_serotype_1_2a_str_10-0814_1196187	Listeria_monocytogenes_1639_v144	0.9292
Listeria_monocytogenes_serotype_1_2a_str_10-0813_1196166	Listeria_monocytogenes_1639_v144	0.935
Listeria_monocytogenes_serotype_1_2a_str_10-0812_1196165	Listeria_monocytogenes_1639_v144	0.935
Listeria_monocytogenes_serotype_1_2a_str_08-7669_1196186	Listeria_monocytogenes_1639_v144	0.9384
Listeria_monocytogenes_serotype_1_2a_str_08-7374_1196185	Listeria_monocytogenes_1639_v144	0.9292
Listeria_monocytogenes_serotype_1_2a_str_08-6997_1196184	Listeria_monocytogenes_1639_v144	0.9247
Listeria_monocytogenes_serotype_1_2a_str_08-6569_1196183	Listeria_monocytogenes_1639_v144	0.9247
Listeria_monocytogenes_serotype_1_2a_str_08-6056_1196182	Listeria_monocytogenes_1639_v144	0.9247
Listeria_monocytogenes_serotype_1_2a_str_04-5457_1196181	Listeria_monocytogenes_1639_v144	0.9292
Listeria_monocytogenes_serotype_1_2a_str_02-5993_1196180	Listeria_monocytogenes_1639_v144	0.9289
Listeria_monocytogenes_R479a_1437838	Listeria_monocytogenes_1639_v144	0.9282
Listeria_monocytogenes_N53-1_1234142	Listeria_monocytogenes_1639_v144	0.8984
Listeria_monocytogenes_M7_1030009	Listeria_monocytogenes_1639_v144	0.9147
Listeria_monocytogenes_LO28_393132	Listeria_monocytogenes_1639_v144	0.9315
Listeria_monocytogenes_L99_563174	Listeria_monocytogenes_1639_v144	0.9154
Listeria_monocytogenes_L312_882094	Listeria_monocytogenes_1639_v144	0.9988
Listeria_monocytogenes_J1816_930781	Listeria_monocytogenes_1639_v144	0.9565
Listeria_monocytogenes_J1-220_930782	Listeria_monocytogenes_1639_v144	0.9522
Listeria_monocytogenes_J0161_393130	Listeria_monocytogenes_1639_v144	0.9353
Listeria_monocytogenes_HCC23_552536	Listeria_monocytogenes_1639_v144	0.9147
Listeria_monocytogenes_FSL_R2-561_393126	Listeria_monocytogenes_1639_v144	0.9315
Listeria_monocytogenes_FSL_J1-208_393119	Listeria_monocytogenes_1639_v144	0.9221
Listeria_monocytogenes_FSL_J1-175_393118	Listeria_monocytogenes_1639_v144	0.9563
Listeria_monocytogenes_Finland_1998_393127	Listeria_monocytogenes_1639_v144	0.9534
Listeria_monocytogenes_EGD-e_169963_v1	Listeria_monocytogenes_1639_v144	0.9407
Listeria_monocytogenes_EGD-e_169963	Listeria_monocytogenes_1639_v144	0.9411
Listeria_monocytogenes_EGD_1334565	Listeria_monocytogenes_1639_v144	0.9432

Listeria_monocytogenes_CFSAN002349_1299895	Listeria_monocytogenes_1639_v144	0.98
Listeria_monocytogenes_ATCC_19117_882095_v1	Listeria_monocytogenes_1639_v144	0.9723
Listeria_monocytogenes_ATCC_19117_882095	Listeria_monocytogenes_1639_v144	0.9723
Listeria_monocytogenes_6179_1288295	Listeria_monocytogenes_1639_v144	0.9149
Listeria_monocytogenes_1639_v9	Listeria_monocytogenes_1639_v144	0.9271
Listeria_monocytogenes_1639_v99	Listeria_monocytogenes_1639_v144	0.9577
Listeria_monocytogenes_1639_v98	Listeria_monocytogenes_1639_v144	0.9333
Listeria_monocytogenes_1639_v97	Listeria_monocytogenes_1639_v144	0.9668
Listeria_monocytogenes_1639_v96	Listeria_monocytogenes_1639_v144	0.9566
Listeria_monocytogenes_1639_v95	Listeria_monocytogenes_1639_v144	0.9566
Listeria_monocytogenes_1639_v94	Listeria_monocytogenes_1639_v144	0.9569
Listeria_monocytogenes_1639_v93	Listeria_monocytogenes_1639_v144	0.9273
Listeria_monocytogenes_1639_v92	Listeria_monocytogenes_1639_v144	0.9573
Listeria_monocytogenes_1639_v91	Listeria_monocytogenes_1639_v144	0.9599
Listeria_monocytogenes_1639_v90	Listeria_monocytogenes_1639_v144	0.9333
Listeria_monocytogenes_1639_v8	Listeria_monocytogenes_1639_v144	0.9178
Listeria_monocytogenes_1639_v89	Listeria_monocytogenes_1639_v144	0.9671
Listeria_monocytogenes_1639_v88	Listeria_monocytogenes_1639_v144	0.9335
Listeria_monocytogenes_1639_v87	Listeria_monocytogenes_1639_v144	0.9307
Listeria_monocytogenes_1639_v86	Listeria_monocytogenes_1639_v144	0.93
Listeria_monocytogenes_1639_v85	Listeria_monocytogenes_1639_v144	0.9602
Listeria_monocytogenes_1639_v84	Listeria_monocytogenes_1639_v144	0.9402
Listeria_monocytogenes_1639_v83	Listeria_monocytogenes_1639_v144	0.9608
Listeria_monocytogenes_1639_v82	Listeria_monocytogenes_1639_v144	0.9605
Listeria_monocytogenes_1639_v81	Listeria_monocytogenes_1639_v144	0.9339
Listeria_monocytogenes_1639_v80	Listeria_monocytogenes_1639_v144	0.9505
Listeria_monocytogenes_1639_v7	Listeria_monocytogenes_1639_v144	0.9178
Listeria_monocytogenes_1639_v79	Listeria_monocytogenes_1639_v144	0.9317
Listeria_monocytogenes_1639_v78	Listeria_monocytogenes_1639_v144	0.9332
Listeria_monocytogenes_1639_v77	Listeria_monocytogenes_1639_v144	0.9314
Listeria_monocytogenes_1639_v76	Listeria_monocytogenes_1639_v144	0.9269
Listeria_monocytogenes_1639_v75	Listeria_monocytogenes_1639_v144	0.9154
Listeria_monocytogenes_1639_v74	Listeria_monocytogenes_1639_v144	0.9289
Listeria_monocytogenes_1639_v73	Listeria_monocytogenes_1639_v144	0.9266
Listeria_monocytogenes_1639_v72	Listeria_monocytogenes_1639_v144	0.96
Listeria_monocytogenes_1639_v71	Listeria_monocytogenes_1639_v144	0.9185
Listeria_monocytogenes_1639_v70	Listeria_monocytogenes_1639_v144	0.9294
Listeria_monocytogenes_1639_v6	Listeria_monocytogenes_1639_v144	0.9171
Listeria_monocytogenes_1639_v69	Listeria_monocytogenes_1639_v144	0.962
Listeria_monocytogenes_1639_v68	Listeria_monocytogenes_1639_v144	0.9392
Listeria_monocytogenes_1639_v67	Listeria_monocytogenes_1639_v144	0.9487
Listeria_monocytogenes_1639_v66	Listeria_monocytogenes_1639_v144	0.9377
Listeria_monocytogenes_1639_v65	Listeria_monocytogenes_1639_v144	0.9324
Listeria_monocytogenes_1639_v64	Listeria_monocytogenes_1639_v144	0.9332
Listeria_monocytogenes_1639_v63	Listeria_monocytogenes_1639_v144	0.9464
Listeria_monocytogenes_1639_v62	Listeria_monocytogenes_1639_v144	0.9296
Listeria_monocytogenes_1639_v61	Listeria_monocytogenes_1639_v144	0.932
Listeria_monocytogenes_1639_v60	Listeria_monocytogenes_1639_v144	0.9373
Listeria_monocytogenes_1639_v5	Listeria_monocytogenes_1639_v144	0.9106
Listeria_monocytogenes_1639_v59	Listeria_monocytogenes_1639_v144	0.9619
Listeria_monocytogenes_1639_v58	Listeria_monocytogenes_1639_v144	0.9604
Listeria_monocytogenes_1639_v57	Listeria_monocytogenes_1639_v144	0.9533
Listeria_monocytogenes_1639_v56	Listeria_monocytogenes_1639_v144	0.9612
Listeria_monocytogenes_1639_v55	Listeria_monocytogenes_1639_v144	0.9494
Listeria_monocytogenes_1639_v54	Listeria_monocytogenes_1639_v144	0.9261
Listeria_monocytogenes_1639_v53	Listeria_monocytogenes_1639_v144	0.9346
Listeria_monocytogenes_1639_v52	Listeria_monocytogenes_1639_v144	0.9583
Listeria_monocytogenes_1639_v51	Listeria_monocytogenes_1639_v144	0.9554
Listeria_monocytogenes_1639_v50	Listeria_monocytogenes_1639_v144	0.9285
Listeria_monocytogenes_1639_v4	Listeria_monocytogenes_1639_v144	0.9237
Listeria_monocytogenes_1639_v49	Listeria_monocytogenes_1639_v144	0.9617
Listeria_monocytogenes_1639_v48	Listeria_monocytogenes_1639_v144	0.9273
Listeria_monocytogenes_1639_v47	Listeria_monocytogenes_1639_v144	0.9289
Listeria_monocytogenes_1639_v46	Listeria_monocytogenes_1639_v144	0.9384
Listeria_monocytogenes_1639_v45	Listeria_monocytogenes_1639_v144	0.9196
Listeria_monocytogenes_1639_v44	Listeria_monocytogenes_1639_v144	0.9292
Listeria_monocytogenes_1639_v43	Listeria_monocytogenes_1639_v144	0.9292
Listeria_monocytogenes_1639_v42	Listeria_monocytogenes_1639_v144	0.921
Listeria_monocytogenes_1639_v41	Listeria_monocytogenes_1639_v144	0.909
Listeria_monocytogenes_1639_v40	Listeria_monocytogenes_1639_v144	0.9319
Listeria_monocytogenes_1639_v3	Listeria_monocytogenes_1639_v144	0.9241
Listeria_monocytogenes_1639_v39	Listeria_monocytogenes_1639_v144	0.9437
Listeria_monocytogenes_1639_v38	Listeria_monocytogenes_1639_v144	0.9287
Listeria_monocytogenes_1639_v37	Listeria_monocytogenes_1639_v144	0.9242
Listeria_monocytogenes_1639_v36	Listeria_monocytogenes_1639_v144	0.9228
Listeria_monocytogenes_1639_v35	Listeria_monocytogenes_1639_v144	0.9326
Listeria_monocytogenes_1639_v34	Listeria_monocytogenes_1639_v144	0.9314
Listeria_monocytogenes_1639_v33	Listeria_monocytogenes_1639_v144	0.9457
Listeria_monocytogenes_1639_v32	Listeria_monocytogenes_1639_v144	0.9231
Listeria_monocytogenes_1639_v31	Listeria_monocytogenes_1639_v144	0.9318
Listeria_monocytogenes_1639_v30	Listeria_monocytogenes_1639_v144	0.9448
Listeria_monocytogenes_1639_v2	Listeria_monocytogenes_1639_v144	0.9103
Listeria_monocytogenes_1639_v29	Listeria_monocytogenes_1639_v144	0.9183
Listeria_monocytogenes_1639_v28	Listeria_monocytogenes_1639_v144	0.9228
Listeria_monocytogenes_1639_v27	Listeria_monocytogenes_1639_v144	0.9135
Listeria_monocytogenes_1639_v26	Listeria_monocytogenes_1639_v144	0.9136
Listeria_monocytogenes_1639_v25	Listeria_monocytogenes_1639_v144	0.9132
Listeria_monocytogenes_1639_v24	Listeria_monocytogenes_1639_v144	0.9522
Listeria_monocytogenes_1639_v23	Listeria_monocytogenes_1639_v144	0.9522
Listeria_monocytogenes_1639_v22	Listeria_monocytogenes_1639_v144	0.9471
Listeria_monocytogenes_1639_v21	Listeria_monocytogenes_1639_v144	0.9518

Listeria_monocytogenes_1639_v20	Listeria_monocytogenes_1639_v144	0.9404
Listeria_monocytogenes_1639_v1	Listeria_monocytogenes_1639_v144	0.9083
Listeria_monocytogenes_1639_v19	Listeria_monocytogenes_1639_v144	0.94
Listeria_monocytogenes_1639_v198	Listeria_monocytogenes_1639_v144	0.9019
Listeria_monocytogenes_1639_v197	Listeria_monocytogenes_1639_v144	0.9286
Listeria_monocytogenes_1639_v196	Listeria_monocytogenes_1639_v144	0.9259
Listeria_monocytogenes_1639_v195	Listeria_monocytogenes_1639_v144	0.9503
Listeria_monocytogenes_1639_v194	Listeria_monocytogenes_1639_v144	0.9508
Listeria_monocytogenes_1639_v193	Listeria_monocytogenes_1639_v144	0.9508
Listeria_monocytogenes_1639_v192	Listeria_monocytogenes_1639_v144	0.9146
Listeria_monocytogenes_1639_v191	Listeria_monocytogenes_1639_v144	0.9564
Listeria_monocytogenes_1639_v190	Listeria_monocytogenes_1639_v144	0.9545
Listeria_monocytogenes_1639_v18	Listeria_monocytogenes_1639_v144	0.9473
Listeria_monocytogenes_1639_v189	Listeria_monocytogenes_1639_v144	0.9538
Listeria_monocytogenes_1639_v188	Listeria_monocytogenes_1639_v144	0.9576
Listeria_monocytogenes_1639_v187	Listeria_monocytogenes_1639_v144	0.9541
Listeria_monocytogenes_1639_v186	Listeria_monocytogenes_1639_v144	0.9549
Listeria_monocytogenes_1639_v185	Listeria_monocytogenes_1639_v144	0.9545
Listeria_monocytogenes_1639_v184	Listeria_monocytogenes_1639_v144	0.9537
Listeria_monocytogenes_1639_v183	Listeria_monocytogenes_1639_v144	0.9522
Listeria_monocytogenes_1639_v182	Listeria_monocytogenes_1639_v144	0.954
Listeria_monocytogenes_1639_v181	Listeria_monocytogenes_1639_v144	0.94
Listeria_monocytogenes_1639_v180	Listeria_monocytogenes_1639_v144	0.94
Listeria_monocytogenes_1639_v17	Listeria_monocytogenes_1639_v144	0.9473
Listeria_monocytogenes_1639_v179	Listeria_monocytogenes_1639_v144	0.9392
Listeria_monocytogenes_1639_v178	Listeria_monocytogenes_1639_v144	0.953
Listeria_monocytogenes_1639_v177	Listeria_monocytogenes_1639_v144	0.9769
Listeria_monocytogenes_1639_v176	Listeria_monocytogenes_1639_v144	0.9477
Listeria_monocytogenes_1639_v175	Listeria_monocytogenes_1639_v144	0.943
Listeria_monocytogenes_1639_v174	Listeria_monocytogenes_1639_v144	0.955
Listeria_monocytogenes_1639_v173	Listeria_monocytogenes_1639_v144	0.9705
Listeria_monocytogenes_1639_v172	Listeria_monocytogenes_1639_v144	0.9452
Listeria_monocytogenes_1639_v171	Listeria_monocytogenes_1639_v144	0.9396
Listeria_monocytogenes_1639_v170	Listeria_monocytogenes_1639_v144	0.9479
Listeria_monocytogenes_1639_v16	Listeria_monocytogenes_1639_v144	0.9155
Listeria_monocytogenes_1639_v169	Listeria_monocytogenes_1639_v144	0.9503
Listeria_monocytogenes_1639_v168	Listeria_monocytogenes_1639_v144	0.9488
Listeria_monocytogenes_1639_v167	Listeria_monocytogenes_1639_v144	0.9409
Listeria_monocytogenes_1639_v166	Listeria_monocytogenes_1639_v144	0.9492
Listeria_monocytogenes_1639_v165	Listeria_monocytogenes_1639_v144	0.9503
Listeria_monocytogenes_1639_v164	Listeria_monocytogenes_1639_v144	0.978
Listeria_monocytogenes_1639_v163	Listeria_monocytogenes_1639_v144	0.9672
Listeria_monocytogenes_1639_v162	Listeria_monocytogenes_1639_v144	0.978
Listeria_monocytogenes_1639_v161	Listeria_monocytogenes_1639_v144	0.9791
Listeria_monocytogenes_1639_v160	Listeria_monocytogenes_1639_v144	0.9795
Listeria_monocytogenes_1639_v15	Listeria_monocytogenes_1639_v144	0.9302
Listeria_monocytogenes_1639_v159	Listeria_monocytogenes_1639_v144	0.9769
Listeria_monocytogenes_1639_v158	Listeria_monocytogenes_1639_v144	0.9791
Listeria_monocytogenes_1639_v157	Listeria_monocytogenes_1639_v144	0.9788
Listeria_monocytogenes_1639_v156	Listeria_monocytogenes_1639_v144	0.931
Listeria_monocytogenes_1639_v155	Listeria_monocytogenes_1639_v144	0.9454
Listeria_monocytogenes_1639_v154	Listeria_monocytogenes_1639_v144	0.9481
Listeria_monocytogenes_1639_v153	Listeria_monocytogenes_1639_v144	0.9462
Listeria_monocytogenes_1639_v152	Listeria_monocytogenes_1639_v144	0.9772
Listeria_monocytogenes_1639_v151	Listeria_monocytogenes_1639_v144	0.9772
Listeria_monocytogenes_1639_v150	Listeria_monocytogenes_1639_v144	0.9443
Listeria_monocytogenes_1639_v14	Listeria_monocytogenes_1639_v144	0.9372
Listeria_monocytogenes_1639_v149	Listeria_monocytogenes_1639_v144	0.9432
Listeria_monocytogenes_1639_v148	Listeria_monocytogenes_1639_v144	0.9757
Listeria_monocytogenes_1639_v147	Listeria_monocytogenes_1639_v144	0.9613
Listeria_monocytogenes_1639_v146	Listeria_monocytogenes_1639_v144	0.9558
Listeria_monocytogenes_1639_v145	Listeria_monocytogenes_1639_v144	0.9498
Listeria_monocytogenes_1639_v144	Listeria_monocytogenes_1639_v144	0.0653
Listeria_welshimeri_1643	Listeria_monocytogenes_1639_v145	0.8794
Listeria_welshimeri_serovar_6b_str_SLCC5334_386043	Listeria_monocytogenes_1639_v145	0.8791
Listeria_seeligeri_serovar_1_2b_str_SLCC3954_683837	Listeria_monocytogenes_1639_v145	0.8874
Listeria_seeligeri_FSL_S4-171_702452	Listeria_monocytogenes_1639_v145	0.8224
Listeria_seeligeri_FSL_N1-067_702453	Listeria_monocytogenes_1639_v145	0.8322
Listeria_seeligeri_1640_v1	Listeria_monocytogenes_1639_v145	0.8873
Listeria_seeligeri_1640	Listeria_monocytogenes_1639_v145	0.8782
Listeria_monocytogenes_WSLC1042_1457188	Listeria_monocytogenes_1639_v145	0.9421
Listeria_monocytogenes_WSLC1001_1457187	Listeria_monocytogenes_1639_v145	0.9538
Listeria_monocytogenes_SLCC7179_879090	Listeria_monocytogenes_1639_v145	0.9634
Listeria_monocytogenes_SLCC5850_882096	Listeria_monocytogenes_1639_v145	0.9582
Listeria_monocytogenes_SLCC2755_932919	Listeria_monocytogenes_1639_v145	0.9377
Listeria_monocytogenes_SLCC2540_879089	Listeria_monocytogenes_1639_v145	0.9439
Listeria_monocytogenes_SLCC2479_882020	Listeria_monocytogenes_1639_v145	0.9488
Listeria_monocytogenes_SLCC2378_879088	Listeria_monocytogenes_1639_v145	0.9432
Listeria_monocytogenes_SLCC2376_882097	Listeria_monocytogenes_1639_v145	0.9477
Listeria_monocytogenes_SLCC2372_932920	Listeria_monocytogenes_1639_v145	0.9417
Listeria_monocytogenes_serotype_4b_str_LL195_1230340	Listeria_monocytogenes_1639_v145	0.9471
Listeria_monocytogenes_serotype_4b_str_LIS0087_1134669	Listeria_monocytogenes_1639_v145	0.935
Listeria_monocytogenes_serotype_4b_str_F2365_265669	Listeria_monocytogenes_1639_v145	0.9471
Listeria_monocytogenes_serotype_4b_str_CLIP_80459_568819	Listeria_monocytogenes_1639_v145	0.9505
Listeria_monocytogenes_serotype_4b_str_81-0861_1196161	Listeria_monocytogenes_1639_v145	0.9325
Listeria_monocytogenes_serotype_4b_str_81-0592_1196160	Listeria_monocytogenes_1639_v145	0.9336
Listeria_monocytogenes_serotype_4b_str_81-0558_1196159	Listeria_monocytogenes_1639_v145	0.9339
Listeria_monocytogenes_serotype_4b_str_10-0809_1196162	Listeria_monocytogenes_1639_v145	0.9336
Listeria_monocytogenes_serotype_4b_str_02-6680_1196175	Listeria_monocytogenes_1639_v145	0.9456
Listeria_monocytogenes_serotype_4b_str_02-6679_1196174	Listeria_monocytogenes_1639_v145	0.9456
Listeria_monocytogenes_serotype_4b_str_02-1792_1196173	Listeria_monocytogenes_1639_v145	0.9336

Listeria_monocytogenes_serotype_4b_str_02-1289_1196172	Listeria_monocytogenes_1639_v145	0.9336
Listeria_monocytogenes_serotype_4b_str_02-1103_1196171	Listeria_monocytogenes_1639_v145	0.9336
Listeria_monocytogenes_serotype_3c_str_10-5027_1196195	Listeria_monocytogenes_1639_v145	0.9481
Listeria_monocytogenes_serotype_1_2c_str_10-5026_1196194	Listeria_monocytogenes_1639_v145	0.9488
Listeria_monocytogenes_serotype_1_2c_str_10-5025_1196193	Listeria_monocytogenes_1639_v145	0.9488
Listeria_monocytogenes_serotype_1_2b_str_10-0811_1196164	Listeria_monocytogenes_1639_v145	0.9298
Listeria_monocytogenes_serotype_1_2b_str_10-0810_1196163	Listeria_monocytogenes_1639_v145	0.9298
Listeria_monocytogenes_serotype_1_2a_str_99-6370_1196179	Listeria_monocytogenes_1639_v145	0.9342
Listeria_monocytogenes_serotype_1_2a_str_98-2035_1196178	Listeria_monocytogenes_1639_v145	0.9342
Listeria_monocytogenes_serotype_1_2a_str_95-0093_1196177	Listeria_monocytogenes_1639_v145	0.9402
Listeria_monocytogenes_serotype_1_2a_str_88-0478_1196176	Listeria_monocytogenes_1639_v145	0.939
Listeria_monocytogenes_serotype_1_2a_str_10-5024_1196192	Listeria_monocytogenes_1639_v145	0.9402
Listeria_monocytogenes_serotype_1_2a_str_10-4758_1196168	Listeria_monocytogenes_1639_v145	0.9575
Listeria_monocytogenes_serotype_1_2a_str_10-4754_1196167	Listeria_monocytogenes_1639_v145	0.9579
Listeria_monocytogenes_serotype_1_2a_str_10-1321_1196191	Listeria_monocytogenes_1639_v145	0.9402
Listeria_monocytogenes_serotype_1_2a_str_10-1047_1196190	Listeria_monocytogenes_1639_v145	0.9409
Listeria_monocytogenes_serotype_1_2a_str_10-1046_1196189	Listeria_monocytogenes_1639_v145	0.9409
Listeria_monocytogenes_serotype_1_2a_str_10-0934_1196170	Listeria_monocytogenes_1639_v145	0.9709
Listeria_monocytogenes_serotype_1_2a_str_10-0933_1196169	Listeria_monocytogenes_1639_v145	0.9709
Listeria_monocytogenes_serotype_1_2a_str_10-0815_1196188	Listeria_monocytogenes_1639_v145	0.9356
Listeria_monocytogenes_serotype_1_2a_str_10-0814_1196187	Listeria_monocytogenes_1639_v145	0.9402
Listeria_monocytogenes_serotype_1_2a_str_10-0813_1196166	Listeria_monocytogenes_1639_v145	0.9513
Listeria_monocytogenes_serotype_1_2a_str_10-0812_1196165	Listeria_monocytogenes_1639_v145	0.9513
Listeria_monocytogenes_serotype_1_2a_str_08-7669_1196186	Listeria_monocytogenes_1639_v145	0.9502
Listeria_monocytogenes_serotype_1_2a_str_08-7374_1196185	Listeria_monocytogenes_1639_v145	0.9402
Listeria_monocytogenes_serotype_1_2a_str_08-6997_1196184	Listeria_monocytogenes_1639_v145	0.9356
Listeria_monocytogenes_serotype_1_2a_str_08-6569_1196183	Listeria_monocytogenes_1639_v145	0.9356
Listeria_monocytogenes_serotype_1_2a_str_08-6056_1196182	Listeria_monocytogenes_1639_v145	0.9356
Listeria_monocytogenes_serotype_1_2a_str_04-5457_1196181	Listeria_monocytogenes_1639_v145	0.9402
Listeria_monocytogenes_serotype_1_2a_str_02-5993_1196180	Listeria_monocytogenes_1639_v145	0.9402
Listeria_monocytogenes_R479a_1437838	Listeria_monocytogenes_1639_v145	0.941
Listeria_monocytogenes_N53-1_1234142	Listeria_monocytogenes_1639_v145	0.9154
Listeria_monocytogenes_M7_1030009	Listeria_monocytogenes_1639_v145	0.9094
Listeria_monocytogenes_LO28_393132	Listeria_monocytogenes_1639_v145	0.9474
Listeria_monocytogenes_L99_563174	Listeria_monocytogenes_1639_v145	0.9098
Listeria_monocytogenes_L312_882094	Listeria_monocytogenes_1639_v145	0.9501
Listeria_monocytogenes_J1816_930781	Listeria_monocytogenes_1639_v145	0.9255
Listeria_monocytogenes_J1-220_930782	Listeria_monocytogenes_1639_v145	0.9243
Listeria_monocytogenes_J0161_393130	Listeria_monocytogenes_1639_v145	0.958
Listeria_monocytogenes_HCC23_552536	Listeria_monocytogenes_1639_v145	0.9094
Listeria_monocytogenes_FSL_R2-561_393126	Listeria_monocytogenes_1639_v145	0.9474
Listeria_monocytogenes_FSL_J1-208_393119	Listeria_monocytogenes_1639_v145	0.9148
Listeria_monocytogenes_FSL_J1-175_393118	Listeria_monocytogenes_1639_v145	0.9452
Listeria_monocytogenes_Finland_1998_393127	Listeria_monocytogenes_1639_v145	0.9705
Listeria_monocytogenes_EGD-e_169963_v1	Listeria_monocytogenes_1639_v145	0.9591
Listeria_monocytogenes_EGD-e_169963	Listeria_monocytogenes_1639_v145	0.9591
Listeria_monocytogenes_EGD_1334565	Listeria_monocytogenes_1639_v145	0.959
Listeria_monocytogenes_CFSAN002349_1299895	Listeria_monocytogenes_1639_v145	0.9468
Listeria_monocytogenes_ATCC_19117_882095_v1	Listeria_monocytogenes_1639_v145	0.9442
Listeria_monocytogenes_ATCC_19117_882095	Listeria_monocytogenes_1639_v145	0.9438
Listeria_monocytogenes_6179_1288295	Listeria_monocytogenes_1639_v145	0.9385
Listeria_monocytogenes_1639_v9	Listeria_monocytogenes_1639_v145	0.9159
Listeria_monocytogenes_1639_v99	Listeria_monocytogenes_1639_v145	0.9267
Listeria_monocytogenes_1639_v98	Listeria_monocytogenes_1639_v145	0.9428
Listeria_monocytogenes_1639_v97	Listeria_monocytogenes_1639_v145	0.9356
Listeria_monocytogenes_1639_v96	Listeria_monocytogenes_1639_v145	0.9256
Listeria_monocytogenes_1639_v95	Listeria_monocytogenes_1639_v145	0.9256
Listeria_monocytogenes_1639_v94	Listeria_monocytogenes_1639_v145	0.9263
Listeria_monocytogenes_1639_v93	Listeria_monocytogenes_1639_v145	0.9454
Listeria_monocytogenes_1639_v92	Listeria_monocytogenes_1639_v145	0.9263
Listeria_monocytogenes_1639_v91	Listeria_monocytogenes_1639_v145	0.9285
Listeria_monocytogenes_1639_v90	Listeria_monocytogenes_1639_v145	0.9428
Listeria_monocytogenes_1639_v8	Listeria_monocytogenes_1639_v145	0.9297
Listeria_monocytogenes_1639_v89	Listeria_monocytogenes_1639_v145	0.9361
Listeria_monocytogenes_1639_v88	Listeria_monocytogenes_1639_v145	0.9502
Listeria_monocytogenes_1639_v87	Listeria_monocytogenes_1639_v145	0.9459
Listeria_monocytogenes_1639_v86	Listeria_monocytogenes_1639_v145	0.9463
Listeria_monocytogenes_1639_v85	Listeria_monocytogenes_1639_v145	0.9322
Listeria_monocytogenes_1639_v84	Listeria_monocytogenes_1639_v145	0.9524
Listeria_monocytogenes_1639_v83	Listeria_monocytogenes_1639_v145	0.9331
Listeria_monocytogenes_1639_v82	Listeria_monocytogenes_1639_v145	0.9328
Listeria_monocytogenes_1639_v81	Listeria_monocytogenes_1639_v145	0.9506
Listeria_monocytogenes_1639_v80	Listeria_monocytogenes_1639_v145	0.9345
Listeria_monocytogenes_1639_v7	Listeria_monocytogenes_1639_v145	0.9297
Listeria_monocytogenes_1639_v79	Listeria_monocytogenes_1639_v145	0.9488
Listeria_monocytogenes_1639_v78	Listeria_monocytogenes_1639_v145	0.9499
Listeria_monocytogenes_1639_v77	Listeria_monocytogenes_1639_v145	0.9565
Listeria_monocytogenes_1639_v76	Listeria_monocytogenes_1639_v145	0.9412
Listeria_monocytogenes_1639_v75	Listeria_monocytogenes_1639_v145	0.9098
Listeria_monocytogenes_1639_v74	Listeria_monocytogenes_1639_v145	0.9482
Listeria_monocytogenes_1639_v73	Listeria_monocytogenes_1639_v145	0.9428
Listeria_monocytogenes_1639_v72	Listeria_monocytogenes_1639_v145	0.9315
Listeria_monocytogenes_1639_v71	Listeria_monocytogenes_1639_v145	0.9399
Listeria_monocytogenes_1639_v70	Listeria_monocytogenes_1639_v145	0.9449
Listeria_monocytogenes_1639_v6	Listeria_monocytogenes_1639_v145	0.9294
Listeria_monocytogenes_1639_v69	Listeria_monocytogenes_1639_v145	0.9336
Listeria_monocytogenes_1639_v68	Listeria_monocytogenes_1639_v145	0.959
Listeria_monocytogenes_1639_v67	Listeria_monocytogenes_1639_v145	0.9369
Listeria_monocytogenes_1639_v66	Listeria_monocytogenes_1639_v145	0.9572
Listeria_monocytogenes_1639_v65	Listeria_monocytogenes_1639_v145	0.943
Listeria_monocytogenes_1639_v64	Listeria_monocytogenes_1639_v145	0.9803

Listeria_monocytogenes_1639_v63	Listeria_monocytogenes_1639_v145	0.9309
Listeria_monocytogenes_1639_v62	Listeria_monocytogenes_1639_v145	0.9459
Listeria_monocytogenes_1639_v61	Listeria_monocytogenes_1639_v145	0.943
Listeria_monocytogenes_1639_v60	Listeria_monocytogenes_1639_v145	0.9601
Listeria_monocytogenes_1639_v5	Listeria_monocytogenes_1639_v145	0.8969
Listeria_monocytogenes_1639_v59	Listeria_monocytogenes_1639_v145	0.9334
Listeria_monocytogenes_1639_v58	Listeria_monocytogenes_1639_v145	0.9342
Listeria_monocytogenes_1639_v57	Listeria_monocytogenes_1639_v145	0.9218
Listeria_monocytogenes_1639_v56	Listeria_monocytogenes_1639_v145	0.9338
Listeria_monocytogenes_1639_v55	Listeria_monocytogenes_1639_v145	0.9187
Listeria_monocytogenes_1639_v54	Listeria_monocytogenes_1639_v145	0.928
Listeria_monocytogenes_1639_v53	Listeria_monocytogenes_1639_v145	0.9795
Listeria_monocytogenes_1639_v52	Listeria_monocytogenes_1639_v145	0.9284
Listeria_monocytogenes_1639_v51	Listeria_monocytogenes_1639_v145	0.9285
Listeria_monocytogenes_1639_v50	Listeria_monocytogenes_1639_v145	0.9387
Listeria_monocytogenes_1639_v4	Listeria_monocytogenes_1639_v145	0.9086
Listeria_monocytogenes_1639_v49	Listeria_monocytogenes_1639_v145	0.9332
Listeria_monocytogenes_1639_v48	Listeria_monocytogenes_1639_v145	0.9431
Listeria_monocytogenes_1639_v47	Listeria_monocytogenes_1639_v145	0.9398
Listeria_monocytogenes_1639_v46	Listeria_monocytogenes_1639_v145	0.9582
Listeria_monocytogenes_1639_v45	Listeria_monocytogenes_1639_v145	0.9315
Listeria_monocytogenes_1639_v44	Listeria_monocytogenes_1639_v145	0.9402
Listeria_monocytogenes_1639_v43	Listeria_monocytogenes_1639_v145	0.9402
Listeria_monocytogenes_1639_v42	Listeria_monocytogenes_1639_v145	0.9678
Listeria_monocytogenes_1639_v41	Listeria_monocytogenes_1639_v145	0.926
Listeria_monocytogenes_1639_v40	Listeria_monocytogenes_1639_v145	0.9481
Listeria_monocytogenes_1639_v3	Listeria_monocytogenes_1639_v145	0.909
Listeria_monocytogenes_1639_v39	Listeria_monocytogenes_1639_v145	0.9323
Listeria_monocytogenes_1639_v38	Listeria_monocytogenes_1639_v145	0.9468
Listeria_monocytogenes_1639_v37	Listeria_monocytogenes_1639_v145	0.9392
Listeria_monocytogenes_1639_v36	Listeria_monocytogenes_1639_v145	0.9371
Listeria_monocytogenes_1639_v35	Listeria_monocytogenes_1639_v145	0.9488
Listeria_monocytogenes_1639_v34	Listeria_monocytogenes_1639_v145	0.9503
Listeria_monocytogenes_1639_v33	Listeria_monocytogenes_1639_v145	0.9298
Listeria_monocytogenes_1639_v32	Listeria_monocytogenes_1639_v145	0.9378
Listeria_monocytogenes_1639_v31	Listeria_monocytogenes_1639_v145	0.9506
Listeria_monocytogenes_1639_v30	Listeria_monocytogenes_1639_v145	0.9376
Listeria_monocytogenes_1639_v2	Listeria_monocytogenes_1639_v145	0.8976
Listeria_monocytogenes_1639_v29	Listeria_monocytogenes_1639_v145	0.9344
Listeria_monocytogenes_1639_v28	Listeria_monocytogenes_1639_v145	0.9385
Listeria_monocytogenes_1639_v27	Listeria_monocytogenes_1639_v145	0.9295
Listeria_monocytogenes_1639_v26	Listeria_monocytogenes_1639_v145	0.9288
Listeria_monocytogenes_1639_v25	Listeria_monocytogenes_1639_v145	0.9288
Listeria_monocytogenes_1639_v24	Listeria_monocytogenes_1639_v145	0.9243
Listeria_monocytogenes_1639_v23	Listeria_monocytogenes_1639_v145	0.9239
Listeria_monocytogenes_1639_v22	Listeria_monocytogenes_1639_v145	0.9309
Listeria_monocytogenes_1639_v21	Listeria_monocytogenes_1639_v145	0.9243
Listeria_monocytogenes_1639_v20	Listeria_monocytogenes_1639_v145	0.9257
Listeria_monocytogenes_1639_v1	Listeria_monocytogenes_1639_v145	0.9319
Listeria_monocytogenes_1639_v19	Listeria_monocytogenes_1639_v145	0.9254
Listeria_monocytogenes_1639_v198	Listeria_monocytogenes_1639_v145	0.9175
Listeria_monocytogenes_1639_v197	Listeria_monocytogenes_1639_v145	0.9456
Listeria_monocytogenes_1639_v196	Listeria_monocytogenes_1639_v145	0.9423
Listeria_monocytogenes_1639_v195	Listeria_monocytogenes_1639_v145	0.9492
Listeria_monocytogenes_1639_v194	Listeria_monocytogenes_1639_v145	0.9477
Listeria_monocytogenes_1639_v193	Listeria_monocytogenes_1639_v145	0.9481
Listeria_monocytogenes_1639_v192	Listeria_monocytogenes_1639_v145	0.9059
Listeria_monocytogenes_1639_v191	Listeria_monocytogenes_1639_v145	0.9763
Listeria_monocytogenes_1639_v190	Listeria_monocytogenes_1639_v145	0.9716
Listeria_monocytogenes_1639_v18	Listeria_monocytogenes_1639_v145	0.9159
Listeria_monocytogenes_1639_v189	Listeria_monocytogenes_1639_v145	0.9713
Listeria_monocytogenes_1639_v188	Listeria_monocytogenes_1639_v145	0.9685
Listeria_monocytogenes_1639_v187	Listeria_monocytogenes_1639_v145	0.972
Listeria_monocytogenes_1639_v186	Listeria_monocytogenes_1639_v145	0.972
Listeria_monocytogenes_1639_v185	Listeria_monocytogenes_1639_v145	0.972
Listeria_monocytogenes_1639_v184	Listeria_monocytogenes_1639_v145	0.9716
Listeria_monocytogenes_1639_v183	Listeria_monocytogenes_1639_v145	0.9709
Listeria_monocytogenes_1639_v182	Listeria_monocytogenes_1639_v145	0.9727
Listeria_monocytogenes_1639_v181	Listeria_monocytogenes_1639_v145	0.93
Listeria_monocytogenes_1639_v180	Listeria_monocytogenes_1639_v145	0.93
Listeria_monocytogenes_1639_v17	Listeria_monocytogenes_1639_v145	0.9163
Listeria_monocytogenes_1639_v179	Listeria_monocytogenes_1639_v145	0.9562
Listeria_monocytogenes_1639_v178	Listeria_monocytogenes_1639_v145	0.974
Listeria_monocytogenes_1639_v177	Listeria_monocytogenes_1639_v145	0.9449
Listeria_monocytogenes_1639_v176	Listeria_monocytogenes_1639_v145	0.9489
Listeria_monocytogenes_1639_v175	Listeria_monocytogenes_1639_v145	0.9634
Listeria_monocytogenes_1639_v174	Listeria_monocytogenes_1639_v145	0.9697
Listeria_monocytogenes_1639_v173	Listeria_monocytogenes_1639_v145	0.9546
Listeria_monocytogenes_1639_v172	Listeria_monocytogenes_1639_v145	0.9433
Listeria_monocytogenes_1639_v171	Listeria_monocytogenes_1639_v145	0.9568
Listeria_monocytogenes_1639_v170	Listeria_monocytogenes_1639_v145	0.9649
Listeria_monocytogenes_1639_v16	Listeria_monocytogenes_1639_v145	0.927
Listeria_monocytogenes_1639_v169	Listeria_monocytogenes_1639_v145	0.9653
Listeria_monocytogenes_1639_v168	Listeria_monocytogenes_1639_v145	0.9709
Listeria_monocytogenes_1639_v167	Listeria_monocytogenes_1639_v145	0.9591
Listeria_monocytogenes_1639_v166	Listeria_monocytogenes_1639_v145	0.9713
Listeria_monocytogenes_1639_v165	Listeria_monocytogenes_1639_v145	0.9649
Listeria_monocytogenes_1639_v164	Listeria_monocytogenes_1639_v145	0.9466
Listeria_monocytogenes_1639_v163	Listeria_monocytogenes_1639_v145	0.9529
Listeria_monocytogenes_1639_v162	Listeria_monocytogenes_1639_v145	0.9466
Listeria_monocytogenes_1639_v161	Listeria_monocytogenes_1639_v145	0.9478

Listeria_monocytogenes_1639_v160	Listeria_monocytogenes_1639_v145	0.9479
Listeria_monocytogenes_1639_v15	Listeria_monocytogenes_1639_v145	0.9154
Listeria_monocytogenes_1639_v159	Listeria_monocytogenes_1639_v145	0.9449
Listeria_monocytogenes_1639_v158	Listeria_monocytogenes_1639_v145	0.9471
Listeria_monocytogenes_1639_v157	Listeria_monocytogenes_1639_v145	0.9471
Listeria_monocytogenes_1639_v156	Listeria_monocytogenes_1639_v145	0.9503
Listeria_monocytogenes_1639_v155	Listeria_monocytogenes_1639_v145	0.9562
Listeria_monocytogenes_1639_v154	Listeria_monocytogenes_1639_v145	0.9593
Listeria_monocytogenes_1639_v153	Listeria_monocytogenes_1639_v145	0.9569
Listeria_monocytogenes_1639_v152	Listeria_monocytogenes_1639_v145	0.9449
Listeria_monocytogenes_1639_v151	Listeria_monocytogenes_1639_v145	0.9449
Listeria_monocytogenes_1639_v150	Listeria_monocytogenes_1639_v145	0.9616
Listeria_monocytogenes_1639_v14	Listeria_monocytogenes_1639_v145	0.9222
Listeria_monocytogenes_1639_v149	Listeria_monocytogenes_1639_v145	0.9593
Listeria_monocytogenes_1639_v148	Listeria_monocytogenes_1639_v145	0.9438
Listeria_monocytogenes_1639_v147	Listeria_monocytogenes_1639_v145	0.9317
Listeria_monocytogenes_1639_v146	Listeria_monocytogenes_1639_v145	0.9702
Listeria_monocytogenes_1639_v145	Listeria_monocytogenes_1639_v145	0.0672
Listeria_welshimeri_1643	Listeria_monocytogenes_1639_v146	0.8822
Listeria_welshimeri_serovar_6b_str_SLCC5334_386043	Listeria_monocytogenes_1639_v146	0.8815
Listeria_seeligeri_serovar_1_2b_str_SLCC3954_683837	Listeria_monocytogenes_1639_v146	0.8934
Listeria_seeligeri_FSL_S4-171_702452	Listeria_monocytogenes_1639_v146	0.8266
Listeria_seeligeri_FSL_N1-067_702453	Listeria_monocytogenes_1639_v146	0.8376
Listeria_seeligeri_1640_v1	Listeria_monocytogenes_1639_v146	0.8964
Listeria_seeligeri_1640	Listeria_monocytogenes_1639_v146	0.8836
Listeria_monocytogenes_WSLC1042_1457188	Listeria_monocytogenes_1639_v146	0.9455
Listeria_monocytogenes_WSLC1001_1457187	Listeria_monocytogenes_1639_v146	0.9575
Listeria_monocytogenes_SLCC7179_879090	Listeria_monocytogenes_1639_v146	0.9653
Listeria_monocytogenes_SLCC5850_882096	Listeria_monocytogenes_1639_v146	0.9623
Listeria_monocytogenes_SLCC2755_932919	Listeria_monocytogenes_1639_v146	0.9463
Listeria_monocytogenes_SLCC2540_879089	Listeria_monocytogenes_1639_v146	0.9506
Listeria_monocytogenes_SLCC2479_882020	Listeria_monocytogenes_1639_v146	0.9567
Listeria_monocytogenes_SLCC2378_879088	Listeria_monocytogenes_1639_v146	0.9469
Listeria_monocytogenes_SLCC2376_882097	Listeria_monocytogenes_1639_v146	0.9518
Listeria_monocytogenes_SLCC2372_932920	Listeria_monocytogenes_1639_v146	0.9495
Listeria_monocytogenes_serotype_4b_str_LL195_1230340	Listeria_monocytogenes_1639_v146	0.9532
Listeria_monocytogenes_serotype_4b_str_LIS0087_1134669	Listeria_monocytogenes_1639_v146	0.938
Listeria_monocytogenes_serotype_4b_str_F2365_265669	Listeria_monocytogenes_1639_v146	0.9532
Listeria_monocytogenes_serotype_4b_str_CLIP_80459_568819	Listeria_monocytogenes_1639_v146	0.9566
Listeria_monocytogenes_serotype_4b_str_81-0861_1196161	Listeria_monocytogenes_1639_v146	0.9357
Listeria_monocytogenes_serotype_4b_str_81-0592_1196160	Listeria_monocytogenes_1639_v146	0.9368
Listeria_monocytogenes_serotype_4b_str_81-0558_1196159	Listeria_monocytogenes_1639_v146	0.9375
Listeria_monocytogenes_serotype_4b_str_10-0809_1196162	Listeria_monocytogenes_1639_v146	0.9368
Listeria_monocytogenes_serotype_4b_str_02-6680_1196175	Listeria_monocytogenes_1639_v146	0.9517
Listeria_monocytogenes_serotype_4b_str_02-6679_1196174	Listeria_monocytogenes_1639_v146	0.9517
Listeria_monocytogenes_serotype_4b_str_02-1792_1196173	Listeria_monocytogenes_1639_v146	0.9372
Listeria_monocytogenes_serotype_4b_str_02-1289_1196172	Listeria_monocytogenes_1639_v146	0.9372
Listeria_monocytogenes_serotype_4b_str_02-1103_1196171	Listeria_monocytogenes_1639_v146	0.9372
Listeria_monocytogenes_serotype_3c_str_10-5027_1196195	Listeria_monocytogenes_1639_v146	0.9559
Listeria_monocytogenes_serotype_1_2c_str_10-5026_1196194	Listeria_monocytogenes_1639_v146	0.9571
Listeria_monocytogenes_serotype_1_2c_str_10-5025_1196193	Listeria_monocytogenes_1639_v146	0.9571
Listeria_monocytogenes_serotype_1_2b_str_10-0811_1196164	Listeria_monocytogenes_1639_v146	0.9387
Listeria_monocytogenes_serotype_1_2b_str_10-0810_1196163	Listeria_monocytogenes_1639_v146	0.9387
Listeria_monocytogenes_serotype_1_2a_str_99-6370_1196179	Listeria_monocytogenes_1639_v146	0.94
Listeria_monocytogenes_serotype_1_2a_str_98-2035_1196178	Listeria_monocytogenes_1639_v146	0.94
Listeria_monocytogenes_serotype_1_2a_str_95-0093_1196177	Listeria_monocytogenes_1639_v146	0.9461
Listeria_monocytogenes_serotype_1_2a_str_88-0478_1196176	Listeria_monocytogenes_1639_v146	0.9453
Listeria_monocytogenes_serotype_1_2a_str_10-5024_1196192	Listeria_monocytogenes_1639_v146	0.9464
Listeria_monocytogenes_serotype_1_2a_str_10-4758_1196168	Listeria_monocytogenes_1639_v146	0.9624
Listeria_monocytogenes_serotype_1_2a_str_10-4754_1196167	Listeria_monocytogenes_1639_v146	0.9624
Listeria_monocytogenes_serotype_1_2a_str_10-1321_1196191	Listeria_monocytogenes_1639_v146	0.9464
Listeria_monocytogenes_serotype_1_2a_str_10-1047_1196190	Listeria_monocytogenes_1639_v146	0.9468
Listeria_monocytogenes_serotype_1_2a_str_10-1046_1196189	Listeria_monocytogenes_1639_v146	0.9468
Listeria_monocytogenes_serotype_1_2a_str_10-0934_1196170	Listeria_monocytogenes_1639_v146	0.9759
Listeria_monocytogenes_serotype_1_2a_str_10-0933_1196169	Listeria_monocytogenes_1639_v146	0.9759
Listeria_monocytogenes_serotype_1_2a_str_10-0815_1196188	Listeria_monocytogenes_1639_v146	0.9414
Listeria_monocytogenes_serotype_1_2a_str_10-0814_1196187	Listeria_monocytogenes_1639_v146	0.9461
Listeria_monocytogenes_serotype_1_2a_str_10-0813_1196166	Listeria_monocytogenes_1639_v146	0.955
Listeria_monocytogenes_serotype_1_2a_str_10-0812_1196165	Listeria_monocytogenes_1639_v146	0.955
Listeria_monocytogenes_serotype_1_2a_str_08-7669_1196186	Listeria_monocytogenes_1639_v146	0.9562
Listeria_monocytogenes_serotype_1_2a_str_08-7374_1196185	Listeria_monocytogenes_1639_v146	0.9464
Listeria_monocytogenes_serotype_1_2a_str_08-6997_1196184	Listeria_monocytogenes_1639_v146	0.9414
Listeria_monocytogenes_serotype_1_2a_str_08-6569_1196183	Listeria_monocytogenes_1639_v146	0.9414
Listeria_monocytogenes_serotype_1_2a_str_08-6056_1196182	Listeria_monocytogenes_1639_v146	0.9414
Listeria_monocytogenes_serotype_1_2a_str_04-5457_1196181	Listeria_monocytogenes_1639_v146	0.9464
Listeria_monocytogenes_serotype_1_2a_str_02-5993_1196180	Listeria_monocytogenes_1639_v146	0.9461
Listeria_monocytogenes_R479a_1437838	Listeria_monocytogenes_1639_v146	0.945
Listeria_monocytogenes_N53-1_1234142	Listeria_monocytogenes_1639_v146	0.9185
Listeria_monocytogenes_M7_1030009	Listeria_monocytogenes_1639_v146	0.9089
Listeria_monocytogenes_LO28_393132	Listeria_monocytogenes_1639_v146	0.9553
Listeria_monocytogenes_L99_563174	Listeria_monocytogenes_1639_v146	0.9095
Listeria_monocytogenes_L312_882094	Listeria_monocytogenes_1639_v146	0.9566
Listeria_monocytogenes_J1816_930781	Listeria_monocytogenes_1639_v146	0.9303
Listeria_monocytogenes_J1-220_930782	Listeria_monocytogenes_1639_v146	0.9275
Listeria_monocytogenes_J0161_393130	Listeria_monocytogenes_1639_v146	0.9583
Listeria_monocytogenes_HCC23_552536	Listeria_monocytogenes_1639_v146	0.9092
Listeria_monocytogenes_FSL_R2-561_393126	Listeria_monocytogenes_1639_v146	0.9552
Listeria_monocytogenes_FSL_J1-208_393119	Listeria_monocytogenes_1639_v146	0.9162
Listeria_monocytogenes_FSL_J1-175_393118	Listeria_monocytogenes_1639_v146	0.9547
Listeria_monocytogenes_Finland_1998_393127	Listeria_monocytogenes_1639_v146	0.9712
Listeria_monocytogenes_EGD-e_169963_v1	Listeria_monocytogenes_1639_v146	0.9655

Listeria_monocytogenes_EGD-e_169963	Listeria_monocytogenes_1639_v146	0.9655
Listeria_monocytogenes_EGD_1334565	Listeria_monocytogenes_1639_v146	0.9627
Listeria_monocytogenes_CFSAN002349_1299895	Listeria_monocytogenes_1639_v146	0.9517
Listeria_monocytogenes_ATCC_19117_882095_v1	Listeria_monocytogenes_1639_v146	0.9476
Listeria_monocytogenes_ATCC_19117_882095	Listeria_monocytogenes_1639_v146	0.9475
Listeria_monocytogenes_6179_1288295	Listeria_monocytogenes_1639_v146	0.942
Listeria_monocytogenes_1639_v9	Listeria_monocytogenes_1639_v146	0.9254
Listeria_monocytogenes_1639_v99	Listeria_monocytogenes_1639_v146	0.931
Listeria_monocytogenes_1639_v98	Listeria_monocytogenes_1639_v146	0.9533
Listeria_monocytogenes_1639_v97	Listeria_monocytogenes_1639_v146	0.9412
Listeria_monocytogenes_1639_v96	Listeria_monocytogenes_1639_v146	0.9303
Listeria_monocytogenes_1639_v95	Listeria_monocytogenes_1639_v146	0.9304
Listeria_monocytogenes_1639_v94	Listeria_monocytogenes_1639_v146	0.9303
Listeria_monocytogenes_1639_v93	Listeria_monocytogenes_1639_v146	0.949
Listeria_monocytogenes_1639_v92	Listeria_monocytogenes_1639_v146	0.9307
Listeria_monocytogenes_1639_v91	Listeria_monocytogenes_1639_v146	0.9347
Listeria_monocytogenes_1639_v90	Listeria_monocytogenes_1639_v146	0.9533
Listeria_monocytogenes_1639_v8	Listeria_monocytogenes_1639_v146	0.9348
Listeria_monocytogenes_1639_v89	Listeria_monocytogenes_1639_v146	0.9455
Listeria_monocytogenes_1639_v88	Listeria_monocytogenes_1639_v146	0.9558
Listeria_monocytogenes_1639_v87	Listeria_monocytogenes_1639_v146	0.9495
Listeria_monocytogenes_1639_v86	Listeria_monocytogenes_1639_v146	0.9492
Listeria_monocytogenes_1639_v85	Listeria_monocytogenes_1639_v146	0.9351
Listeria_monocytogenes_1639_v84	Listeria_monocytogenes_1639_v146	0.9565
Listeria_monocytogenes_1639_v83	Listeria_monocytogenes_1639_v146	0.9364
Listeria_monocytogenes_1639_v82	Listeria_monocytogenes_1639_v146	0.936
Listeria_monocytogenes_1639_v81	Listeria_monocytogenes_1639_v146	0.9512
Listeria_monocytogenes_1639_v80	Listeria_monocytogenes_1639_v146	0.9439
Listeria_monocytogenes_1639_v7	Listeria_monocytogenes_1639_v146	0.9348
Listeria_monocytogenes_1639_v79	Listeria_monocytogenes_1639_v146	0.9491
Listeria_monocytogenes_1639_v78	Listeria_monocytogenes_1639_v146	0.9505
Listeria_monocytogenes_1639_v77	Listeria_monocytogenes_1639_v146	0.9568
Listeria_monocytogenes_1639_v76	Listeria_monocytogenes_1639_v146	0.9452
Listeria_monocytogenes_1639_v75	Listeria_monocytogenes_1639_v146	0.9095
Listeria_monocytogenes_1639_v74	Listeria_monocytogenes_1639_v146	0.9492
Listeria_monocytogenes_1639_v73	Listeria_monocytogenes_1639_v146	0.9502
Listeria_monocytogenes_1639_v72	Listeria_monocytogenes_1639_v146	0.9351
Listeria_monocytogenes_1639_v71	Listeria_monocytogenes_1639_v146	0.9405
Listeria_monocytogenes_1639_v70	Listeria_monocytogenes_1639_v146	0.9531
Listeria_monocytogenes_1639_v6	Listeria_monocytogenes_1639_v146	0.9341
Listeria_monocytogenes_1639_v69	Listeria_monocytogenes_1639_v146	0.9372
Listeria_monocytogenes_1639_v68	Listeria_monocytogenes_1639_v146	0.9634
Listeria_monocytogenes_1639_v67	Listeria_monocytogenes_1639_v146	0.9463
Listeria_monocytogenes_1639_v66	Listeria_monocytogenes_1639_v146	0.9613
Listeria_monocytogenes_1639_v65	Listeria_monocytogenes_1639_v146	0.9489
Listeria_monocytogenes_1639_v64	Listeria_monocytogenes_1639_v146	0.9532
Listeria_monocytogenes_1639_v63	Listeria_monocytogenes_1639_v146	0.9394
Listeria_monocytogenes_1639_v62	Listeria_monocytogenes_1639_v146	0.9469
Listeria_monocytogenes_1639_v61	Listeria_monocytogenes_1639_v146	0.9489
Listeria_monocytogenes_1639_v60	Listeria_monocytogenes_1639_v146	0.9607
Listeria_monocytogenes_1639_v5	Listeria_monocytogenes_1639_v146	0.9043
Listeria_monocytogenes_1639_v59	Listeria_monocytogenes_1639_v146	0.9375
Listeria_monocytogenes_1639_v58	Listeria_monocytogenes_1639_v146	0.9398
Listeria_monocytogenes_1639_v57	Listeria_monocytogenes_1639_v146	0.9272
Listeria_monocytogenes_1639_v56	Listeria_monocytogenes_1639_v146	0.9371
Listeria_monocytogenes_1639_v55	Listeria_monocytogenes_1639_v146	0.923
Listeria_monocytogenes_1639_v54	Listeria_monocytogenes_1639_v146	0.9328
Listeria_monocytogenes_1639_v53	Listeria_monocytogenes_1639_v146	0.9566
Listeria_monocytogenes_1639_v52	Listeria_monocytogenes_1639_v146	0.9343
Listeria_monocytogenes_1639_v51	Listeria_monocytogenes_1639_v146	0.9318
Listeria_monocytogenes_1639_v50	Listeria_monocytogenes_1639_v146	0.9449
Listeria_monocytogenes_1639_v4	Listeria_monocytogenes_1639_v146	0.9169
Listeria_monocytogenes_1639_v49	Listeria_monocytogenes_1639_v146	0.9365
Listeria_monocytogenes_1639_v48	Listeria_monocytogenes_1639_v146	0.9509
Listeria_monocytogenes_1639_v47	Listeria_monocytogenes_1639_v146	0.9461
Listeria_monocytogenes_1639_v46	Listeria_monocytogenes_1639_v146	0.9627
Listeria_monocytogenes_1639_v45	Listeria_monocytogenes_1639_v146	0.9362
Listeria_monocytogenes_1639_v44	Listeria_monocytogenes_1639_v146	0.9464
Listeria_monocytogenes_1639_v43	Listeria_monocytogenes_1639_v146	0.9464
Listeria_monocytogenes_1639_v42	Listeria_monocytogenes_1639_v146	0.9404
Listeria_monocytogenes_1639_v41	Listeria_monocytogenes_1639_v146	0.9258
Listeria_monocytogenes_1639_v40	Listeria_monocytogenes_1639_v146	0.956
Listeria_monocytogenes_1639_v3	Listeria_monocytogenes_1639_v146	0.9173
Listeria_monocytogenes_1639_v39	Listeria_monocytogenes_1639_v146	0.9416
Listeria_monocytogenes_1639_v38	Listeria_monocytogenes_1639_v146	0.9523
Listeria_monocytogenes_1639_v37	Listeria_monocytogenes_1639_v146	0.9477
Listeria_monocytogenes_1639_v36	Listeria_monocytogenes_1639_v146	0.9403
Listeria_monocytogenes_1639_v35	Listeria_monocytogenes_1639_v146	0.9567
Listeria_monocytogenes_1639_v34	Listeria_monocytogenes_1639_v146	0.9547
Listeria_monocytogenes_1639_v33	Listeria_monocytogenes_1639_v146	0.9387
Listeria_monocytogenes_1639_v32	Listeria_monocytogenes_1639_v146	0.9463
Listeria_monocytogenes_1639_v31	Listeria_monocytogenes_1639_v146	0.955
Listeria_monocytogenes_1639_v30	Listeria_monocytogenes_1639_v146	0.9413
Listeria_monocytogenes_1639_v2	Listeria_monocytogenes_1639_v146	0.9046
Listeria_monocytogenes_1639_v29	Listeria_monocytogenes_1639_v146	0.9417
Listeria_monocytogenes_1639_v28	Listeria_monocytogenes_1639_v146	0.9463
Listeria_monocytogenes_1639_v27	Listeria_monocytogenes_1639_v146	0.9368
Listeria_monocytogenes_1639_v26	Listeria_monocytogenes_1639_v146	0.9364
Listeria_monocytogenes_1639_v25	Listeria_monocytogenes_1639_v146	0.9365
Listeria_monocytogenes_1639_v24	Listeria_monocytogenes_1639_v146	0.9279
Listeria_monocytogenes_1639_v23	Listeria_monocytogenes_1639_v146	0.9279

Listeria_monocytogenes_1639_v22	Listeria_monocytogenes_1639_v146	0.9402
Listeria_monocytogenes_1639_v21	Listeria_monocytogenes_1639_v146	0.9279
Listeria_monocytogenes_1639_v20	Listeria_monocytogenes_1639_v146	0.9338
Listeria_monocytogenes_1639_v1	Listeria_monocytogenes_1639_v146	0.9351
Listeria_monocytogenes_1639_v19	Listeria_monocytogenes_1639_v146	0.9335
Listeria_monocytogenes_1639_v198	Listeria_monocytogenes_1639_v146	0.9214
Listeria_monocytogenes_1639_v197	Listeria_monocytogenes_1639_v146	0.9515
Listeria_monocytogenes_1639_v196	Listeria_monocytogenes_1639_v146	0.9457
Listeria_monocytogenes_1639_v195	Listeria_monocytogenes_1639_v146	0.9541
Listeria_monocytogenes_1639_v194	Listeria_monocytogenes_1639_v146	0.9522
Listeria_monocytogenes_1639_v193	Listeria_monocytogenes_1639_v146	0.9526
Listeria_monocytogenes_1639_v192	Listeria_monocytogenes_1639_v146	0.9114
Listeria_monocytogenes_1639_v191	Listeria_monocytogenes_1639_v146	0.9766
Listeria_monocytogenes_1639_v190	Listeria_monocytogenes_1639_v146	0.9755
Listeria_monocytogenes_1639_v18	Listeria_monocytogenes_1639_v146	0.9213
Listeria_monocytogenes_1639_v189	Listeria_monocytogenes_1639_v146	0.9747
Listeria_monocytogenes_1639_v188	Listeria_monocytogenes_1639_v146	0.9793
Listeria_monocytogenes_1639_v187	Listeria_monocytogenes_1639_v146	0.9751
Listeria_monocytogenes_1639_v186	Listeria_monocytogenes_1639_v146	0.9758
Listeria_monocytogenes_1639_v185	Listeria_monocytogenes_1639_v146	0.9755
Listeria_monocytogenes_1639_v184	Listeria_monocytogenes_1639_v146	0.9751
Listeria_monocytogenes_1639_v183	Listeria_monocytogenes_1639_v146	0.9762
Listeria_monocytogenes_1639_v182	Listeria_monocytogenes_1639_v146	0.9785
Listeria_monocytogenes_1639_v181	Listeria_monocytogenes_1639_v146	0.9329
Listeria_monocytogenes_1639_v180	Listeria_monocytogenes_1639_v146	0.9329
Listeria_monocytogenes_1639_v17	Listeria_monocytogenes_1639_v146	0.9213
Listeria_monocytogenes_1639_v179	Listeria_monocytogenes_1639_v146	0.9618
Listeria_monocytogenes_1639_v178	Listeria_monocytogenes_1639_v146	0.9797
Listeria_monocytogenes_1639_v177	Listeria_monocytogenes_1639_v146	0.949
Listeria_monocytogenes_1639_v176	Listeria_monocytogenes_1639_v146	0.9504
Listeria_monocytogenes_1639_v175	Listeria_monocytogenes_1639_v146	0.9649
Listeria_monocytogenes_1639_v174	Listeria_monocytogenes_1639_v146	0.9739
Listeria_monocytogenes_1639_v173	Listeria_monocytogenes_1639_v146	0.9673
Listeria_monocytogenes_1639_v172	Listeria_monocytogenes_1639_v146	0.9501
Listeria_monocytogenes_1639_v171	Listeria_monocytogenes_1639_v146	0.9598
Listeria_monocytogenes_1639_v170	Listeria_monocytogenes_1639_v146	0.9679
Listeria_monocytogenes_1639_v16	Listeria_monocytogenes_1639_v146	0.932
Listeria_monocytogenes_1639_v169	Listeria_monocytogenes_1639_v146	0.9695
Listeria_monocytogenes_1639_v168	Listeria_monocytogenes_1639_v146	0.9693
Listeria_monocytogenes_1639_v167	Listeria_monocytogenes_1639_v146	0.9648
Listeria_monocytogenes_1639_v166	Listeria_monocytogenes_1639_v146	0.9697
Listeria_monocytogenes_1639_v165	Listeria_monocytogenes_1639_v146	0.9691
Listeria_monocytogenes_1639_v164	Listeria_monocytogenes_1639_v146	0.9523
Listeria_monocytogenes_1639_v163	Listeria_monocytogenes_1639_v146	0.9582
Listeria_monocytogenes_1639_v162	Listeria_monocytogenes_1639_v146	0.9523
Listeria_monocytogenes_1639_v161	Listeria_monocytogenes_1639_v146	0.9535
Listeria_monocytogenes_1639_v160	Listeria_monocytogenes_1639_v146	0.9535
Listeria_monocytogenes_1639_v15	Listeria_monocytogenes_1639_v146	0.9234
Listeria_monocytogenes_1639_v159	Listeria_monocytogenes_1639_v146	0.9494
Listeria_monocytogenes_1639_v158	Listeria_monocytogenes_1639_v146	0.9532
Listeria_monocytogenes_1639_v157	Listeria_monocytogenes_1639_v146	0.9532
Listeria_monocytogenes_1639_v156	Listeria_monocytogenes_1639_v146	0.9517
Listeria_monocytogenes_1639_v155	Listeria_monocytogenes_1639_v146	0.9669
Listeria_monocytogenes_1639_v154	Listeria_monocytogenes_1639_v146	0.9653
Listeria_monocytogenes_1639_v153	Listeria_monocytogenes_1639_v146	0.9676
Listeria_monocytogenes_1639_v152	Listeria_monocytogenes_1639_v146	0.9497
Listeria_monocytogenes_1639_v151	Listeria_monocytogenes_1639_v146	0.9497
Listeria_monocytogenes_1639_v150	Listeria_monocytogenes_1639_v146	0.9642
Listeria_monocytogenes_1639_v14	Listeria_monocytogenes_1639_v146	0.9303
Listeria_monocytogenes_1639_v149	Listeria_monocytogenes_1639_v146	0.9627
Listeria_monocytogenes_1639_v148	Listeria_monocytogenes_1639_v146	0.9486
Listeria_monocytogenes_1639_v147	Listeria_monocytogenes_1639_v146	0.935
Listeria_monocytogenes_1639_v146	Listeria_monocytogenes_1639_v146	0.0664
Listeria_welshimeri_1643	Listeria_monocytogenes_1639_v147	0.8686
Listeria_welshimeri_serovar_6b_str_SLCC5334_386043	Listeria_monocytogenes_1639_v147	0.8683
Listeria_seeligeri_serovar_1_2b_str_SLCC3954_683837	Listeria_monocytogenes_1639_v147	0.8764
Listeria_seeligeri_FSL_S4-171_702452	Listeria_monocytogenes_1639_v147	0.8174
Listeria_seeligeri_FSL_N1-067_702453	Listeria_monocytogenes_1639_v147	0.8469
Listeria_seeligeri_1640_v1	Listeria_monocytogenes_1639_v147	0.8867
Listeria_seeligeri_1640	Listeria_monocytogenes_1639_v147	0.8766
Listeria_monocytogenes_WSLC1042_1457188	Listeria_monocytogenes_1639_v147	0.9537
Listeria_monocytogenes_WSLC1001_1457187	Listeria_monocytogenes_1639_v147	0.9217
Listeria_monocytogenes_SLCC7179_879090	Listeria_monocytogenes_1639_v147	0.927
Listeria_monocytogenes_SLCC5850_882096	Listeria_monocytogenes_1639_v147	0.9262
Listeria_monocytogenes_SLCC2755_932919	Listeria_monocytogenes_1639_v147	0.9391
Listeria_monocytogenes_SLCC2540_879089	Listeria_monocytogenes_1639_v147	0.9363
Listeria_monocytogenes_SLCC2479_882020	Listeria_monocytogenes_1639_v147	0.9188
Listeria_monocytogenes_SLCC2378_879088	Listeria_monocytogenes_1639_v147	0.9548
Listeria_monocytogenes_SLCC2376_882097	Listeria_monocytogenes_1639_v147	0.9333
Listeria_monocytogenes_SLCC2372_932920	Listeria_monocytogenes_1639_v147	0.9139
Listeria_monocytogenes_serotype_4b_str_LL195_1230340	Listeria_monocytogenes_1639_v147	0.9602
Listeria_monocytogenes_serotype_4b_str_LIS0087_1134669	Listeria_monocytogenes_1639_v147	0.9035
Listeria_monocytogenes_serotype_4b_str_F2365_265669	Listeria_monocytogenes_1639_v147	0.9602
Listeria_monocytogenes_serotype_4b_str_CLIP_80459_568819	Listeria_monocytogenes_1639_v147	0.9617
Listeria_monocytogenes_serotype_4b_str_81-0861_1196161	Listeria_monocytogenes_1639_v147	0.9441
Listeria_monocytogenes_serotype_4b_str_81-0592_1196160	Listeria_monocytogenes_1639_v147	0.9452
Listeria_monocytogenes_serotype_4b_str_81-0558_1196159	Listeria_monocytogenes_1639_v147	0.9448
Listeria_monocytogenes_serotype_4b_str_10-0809_1196162	Listeria_monocytogenes_1639_v147	0.9455
Listeria_monocytogenes_serotype_4b_str_02-6680_1196175	Listeria_monocytogenes_1639_v147	0.9591
Listeria_monocytogenes_serotype_4b_str_02-6679_1196174	Listeria_monocytogenes_1639_v147	0.9591
Listeria_monocytogenes_serotype_4b_str_02-1792_1196173	Listeria_monocytogenes_1639_v147	0.9452

Listeria_monocytogenes_serotype_4b_str_02-1289_1196172	Listeria_monocytogenes_1639_v147	0.9452
Listeria_monocytogenes_serotype_4b_str_02-1103_1196171	Listeria_monocytogenes_1639_v147	0.9452
Listeria_monocytogenes_serotype_3c_str_10-5027_1196195	Listeria_monocytogenes_1639_v147	0.9181
Listeria_monocytogenes_serotype_1_2c_str_10-5026_1196194	Listeria_monocytogenes_1639_v147	0.9188
Listeria_monocytogenes_serotype_1_2c_str_10-5025_1196193	Listeria_monocytogenes_1639_v147	0.9188
Listeria_monocytogenes_serotype_1_2b_str_10-0811_1196164	Listeria_monocytogenes_1639_v147	0.9307
Listeria_monocytogenes_serotype_1_2b_str_10-0810_1196163	Listeria_monocytogenes_1639_v147	0.9307
Listeria_monocytogenes_serotype_1_2a_str_99-6370_1196179	Listeria_monocytogenes_1639_v147	0.9085
Listeria_monocytogenes_serotype_1_2a_str_98-2035_1196178	Listeria_monocytogenes_1639_v147	0.9085
Listeria_monocytogenes_serotype_1_2a_str_95-0093_1196177	Listeria_monocytogenes_1639_v147	0.9135
Listeria_monocytogenes_serotype_1_2a_str_88-0478_1196176	Listeria_monocytogenes_1639_v147	0.9123
Listeria_monocytogenes_serotype_1_2a_str_10-5024_1196192	Listeria_monocytogenes_1639_v147	0.9135
Listeria_monocytogenes_serotype_1_2a_str_10-4758_1196168	Listeria_monocytogenes_1639_v147	0.9274
Listeria_monocytogenes_serotype_1_2a_str_10-4754_1196167	Listeria_monocytogenes_1639_v147	0.9274
Listeria_monocytogenes_serotype_1_2a_str_10-1321_1196191	Listeria_monocytogenes_1639_v147	0.9135
Listeria_monocytogenes_serotype_1_2a_str_10-1047_1196190	Listeria_monocytogenes_1639_v147	0.9141
Listeria_monocytogenes_serotype_1_2a_str_10-1046_1196189	Listeria_monocytogenes_1639_v147	0.9141
Listeria_monocytogenes_serotype_1_2a_str_10-0934_1196170	Listeria_monocytogenes_1639_v147	0.9371
Listeria_monocytogenes_serotype_1_2a_str_10-0933_1196169	Listeria_monocytogenes_1639_v147	0.9371
Listeria_monocytogenes_serotype_1_2a_str_10-0815_1196188	Listeria_monocytogenes_1639_v147	0.9098
Listeria_monocytogenes_serotype_1_2a_str_10-0814_1196187	Listeria_monocytogenes_1639_v147	0.9138
Listeria_monocytogenes_serotype_1_2a_str_10-0813_1196166	Listeria_monocytogenes_1639_v147	0.9193
Listeria_monocytogenes_serotype_1_2a_str_10-0812_1196165	Listeria_monocytogenes_1639_v147	0.9193
Listeria_monocytogenes_serotype_1_2a_str_08-7669_1196186	Listeria_monocytogenes_1639_v147	0.9226
Listeria_monocytogenes_serotype_1_2a_str_08-7374_1196185	Listeria_monocytogenes_1639_v147	0.9135
Listeria_monocytogenes_serotype_1_2a_str_08-6997_1196184	Listeria_monocytogenes_1639_v147	0.9098
Listeria_monocytogenes_serotype_1_2a_str_08-6569_1196183	Listeria_monocytogenes_1639_v147	0.9098
Listeria_monocytogenes_serotype_1_2a_str_08-6056_1196182	Listeria_monocytogenes_1639_v147	0.9098
Listeria_monocytogenes_serotype_1_2a_str_04-5457_1196181	Listeria_monocytogenes_1639_v147	0.9135
Listeria_monocytogenes_serotype_1_2a_str_02-5993_1196180	Listeria_monocytogenes_1639_v147	0.9135
Listeria_monocytogenes_R479a_1437838	Listeria_monocytogenes_1639_v147	0.9202
Listeria_monocytogenes_N53-1_1234142	Listeria_monocytogenes_1639_v147	0.8838
Listeria_monocytogenes_M7_1030009	Listeria_monocytogenes_1639_v147	0.8984
Listeria_monocytogenes_LO28_393132	Listeria_monocytogenes_1639_v147	0.9175
Listeria_monocytogenes_L99_563174	Listeria_monocytogenes_1639_v147	0.8988
Listeria_monocytogenes_L312_882094	Listeria_monocytogenes_1639_v147	0.962
Listeria_monocytogenes_J1816_930781	Listeria_monocytogenes_1639_v147	0.9629
Listeria_monocytogenes_J1-220_930782	Listeria_monocytogenes_1639_v147	0.9367
Listeria_monocytogenes_J0161_393130	Listeria_monocytogenes_1639_v147	0.9164
Listeria_monocytogenes_HCC23_552536	Listeria_monocytogenes_1639_v147	0.8981
Listeria_monocytogenes_FSL_R2-561_393126	Listeria_monocytogenes_1639_v147	0.9174
Listeria_monocytogenes_FSL_J1-208_393119	Listeria_monocytogenes_1639_v147	0.9084
Listeria_monocytogenes_FSL_J1-175_393118	Listeria_monocytogenes_1639_v147	0.9424
Listeria_monocytogenes_Finland_1998_393127	Listeria_monocytogenes_1639_v147	0.9375
Listeria_monocytogenes_EGD-e_169963_v1	Listeria_monocytogenes_1639_v147	0.9264
Listeria_monocytogenes_EGD-e_169963	Listeria_monocytogenes_1639_v147	0.9268
Listeria_monocytogenes_EGD_1334565	Listeria_monocytogenes_1639_v147	0.9269
Listeria_monocytogenes_CFSAN002349_1299895	Listeria_monocytogenes_1639_v147	0.9678
Listeria_monocytogenes_ATCC_19117_882095_v1	Listeria_monocytogenes_1639_v147	0.9562
Listeria_monocytogenes_ATCC_19117_882095	Listeria_monocytogenes_1639_v147	0.9558
Listeria_monocytogenes_6179_1288295	Listeria_monocytogenes_1639_v147	0.9076
Listeria_monocytogenes_1639_v9	Listeria_monocytogenes_1639_v147	0.9141
Listeria_monocytogenes_1639_v99	Listeria_monocytogenes_1639_v147	0.9681
Listeria_monocytogenes_1639_v98	Listeria_monocytogenes_1639_v147	0.9151
Listeria_monocytogenes_1639_v97	Listeria_monocytogenes_1639_v147	0.9525
Listeria_monocytogenes_1639_v96	Listeria_monocytogenes_1639_v147	0.9677
Listeria_monocytogenes_1639_v95	Listeria_monocytogenes_1639_v147	0.9674
Listeria_monocytogenes_1639_v94	Listeria_monocytogenes_1639_v147	0.9677
Listeria_monocytogenes_1639_v93	Listeria_monocytogenes_1639_v147	0.9208
Listeria_monocytogenes_1639_v92	Listeria_monocytogenes_1639_v147	0.9677
Listeria_monocytogenes_1639_v91	Listeria_monocytogenes_1639_v147	0.9409
Listeria_monocytogenes_1639_v90	Listeria_monocytogenes_1639_v147	0.9151
Listeria_monocytogenes_1639_v8	Listeria_monocytogenes_1639_v147	0.9109
Listeria_monocytogenes_1639_v89	Listeria_monocytogenes_1639_v147	0.9523
Listeria_monocytogenes_1639_v88	Listeria_monocytogenes_1639_v147	0.9183
Listeria_monocytogenes_1639_v87	Listeria_monocytogenes_1639_v147	0.9152
Listeria_monocytogenes_1639_v86	Listeria_monocytogenes_1639_v147	0.9149
Listeria_monocytogenes_1639_v85	Listeria_monocytogenes_1639_v147	0.943
Listeria_monocytogenes_1639_v84	Listeria_monocytogenes_1639_v147	0.9214
Listeria_monocytogenes_1639_v83	Listeria_monocytogenes_1639_v147	0.9451
Listeria_monocytogenes_1639_v82	Listeria_monocytogenes_1639_v147	0.9444
Listeria_monocytogenes_1639_v81	Listeria_monocytogenes_1639_v147	0.9183
Listeria_monocytogenes_1639_v80	Listeria_monocytogenes_1639_v147	0.936
Listeria_monocytogenes_1639_v7	Listeria_monocytogenes_1639_v147	0.9109
Listeria_monocytogenes_1639_v79	Listeria_monocytogenes_1639_v147	0.9165
Listeria_monocytogenes_1639_v78	Listeria_monocytogenes_1639_v147	0.9176
Listeria_monocytogenes_1639_v77	Listeria_monocytogenes_1639_v147	0.9184
Listeria_monocytogenes_1639_v76	Listeria_monocytogenes_1639_v147	0.9122
Listeria_monocytogenes_1639_v75	Listeria_monocytogenes_1639_v147	0.8988
Listeria_monocytogenes_1639_v74	Listeria_monocytogenes_1639_v147	0.9439
Listeria_monocytogenes_1639_v73	Listeria_monocytogenes_1639_v147	0.9153
Listeria_monocytogenes_1639_v72	Listeria_monocytogenes_1639_v147	0.9436
Listeria_monocytogenes_1639_v71	Listeria_monocytogenes_1639_v147	0.931
Listeria_monocytogenes_1639_v70	Listeria_monocytogenes_1639_v147	0.9162
Listeria_monocytogenes_1639_v6	Listeria_monocytogenes_1639_v147	0.9105
Listeria_monocytogenes_1639_v69	Listeria_monocytogenes_1639_v147	0.9452
Listeria_monocytogenes_1639_v68	Listeria_monocytogenes_1639_v147	0.9216
Listeria_monocytogenes_1639_v67	Listeria_monocytogenes_1639_v147	0.9317
Listeria_monocytogenes_1639_v66	Listeria_monocytogenes_1639_v147	0.923
Listeria_monocytogenes_1639_v65	Listeria_monocytogenes_1639_v147	0.9154
Listeria_monocytogenes_1639_v64	Listeria_monocytogenes_1639_v147	0.9158

Listeria_monocytogenes_1639_v63	Listeria_monocytogenes_1639_v147	0.9373
Listeria_monocytogenes_1639_v62	Listeria_monocytogenes_1639_v147	0.9142
Listeria_monocytogenes_1639_v61	Listeria_monocytogenes_1639_v147	0.9147
Listeria_monocytogenes_1639_v60	Listeria_monocytogenes_1639_v147	0.9182
Listeria_monocytogenes_1639_v5	Listeria_monocytogenes_1639_v147	0.9268
Listeria_monocytogenes_1639_v59	Listeria_monocytogenes_1639_v147	0.9454
Listeria_monocytogenes_1639_v58	Listeria_monocytogenes_1639_v147	0.9473
Listeria_monocytogenes_1639_v57	Listeria_monocytogenes_1639_v147	0.9616
Listeria_monocytogenes_1639_v56	Listeria_monocytogenes_1639_v147	0.9454
Listeria_monocytogenes_1639_v55	Listeria_monocytogenes_1639_v147	0.9599
Listeria_monocytogenes_1639_v54	Listeria_monocytogenes_1639_v147	0.9135
Listeria_monocytogenes_1639_v53	Listeria_monocytogenes_1639_v147	0.9197
Listeria_monocytogenes_1639_v52	Listeria_monocytogenes_1639_v147	0.9423
Listeria_monocytogenes_1639_v51	Listeria_monocytogenes_1639_v147	0.9409
Listeria_monocytogenes_1639_v50	Listeria_monocytogenes_1639_v147	0.9127
Listeria_monocytogenes_1639_v4	Listeria_monocytogenes_1639_v147	0.9167
Listeria_monocytogenes_1639_v49	Listeria_monocytogenes_1639_v147	0.9433
Listeria_monocytogenes_1639_v48	Listeria_monocytogenes_1639_v147	0.9138
Listeria_monocytogenes_1639_v47	Listeria_monocytogenes_1639_v147	0.9131
Listeria_monocytogenes_1639_v46	Listeria_monocytogenes_1639_v147	0.9209
Listeria_monocytogenes_1639_v45	Listeria_monocytogenes_1639_v147	0.9118
Listeria_monocytogenes_1639_v44	Listeria_monocytogenes_1639_v147	0.9135
Listeria_monocytogenes_1639_v43	Listeria_monocytogenes_1639_v147	0.9135
Listeria_monocytogenes_1639_v42	Listeria_monocytogenes_1639_v147	0.904
Listeria_monocytogenes_1639_v41	Listeria_monocytogenes_1639_v147	0.8989
Listeria_monocytogenes_1639_v40	Listeria_monocytogenes_1639_v147	0.9197
Listeria_monocytogenes_1639_v3	Listeria_monocytogenes_1639_v147	0.9167
Listeria_monocytogenes_1639_v39	Listeria_monocytogenes_1639_v147	0.9298
Listeria_monocytogenes_1639_v38	Listeria_monocytogenes_1639_v147	0.9158
Listeria_monocytogenes_1639_v37	Listeria_monocytogenes_1639_v147	0.9119
Listeria_monocytogenes_1639_v36	Listeria_monocytogenes_1639_v147	0.9124
Listeria_monocytogenes_1639_v35	Listeria_monocytogenes_1639_v147	0.9196
Listeria_monocytogenes_1639_v34	Listeria_monocytogenes_1639_v147	0.9137
Listeria_monocytogenes_1639_v33	Listeria_monocytogenes_1639_v147	0.9307
Listeria_monocytogenes_1639_v32	Listeria_monocytogenes_1639_v147	0.9101
Listeria_monocytogenes_1639_v31	Listeria_monocytogenes_1639_v147	0.9141
Listeria_monocytogenes_1639_v30	Listeria_monocytogenes_1639_v147	0.9335
Listeria_monocytogenes_1639_v2	Listeria_monocytogenes_1639_v147	0.9236
Listeria_monocytogenes_1639_v29	Listeria_monocytogenes_1639_v147	0.908
Listeria_monocytogenes_1639_v28	Listeria_monocytogenes_1639_v147	0.9097
Listeria_monocytogenes_1639_v27	Listeria_monocytogenes_1639_v147	0.9052
Listeria_monocytogenes_1639_v26	Listeria_monocytogenes_1639_v147	0.9049
Listeria_monocytogenes_1639_v25	Listeria_monocytogenes_1639_v147	0.9049
Listeria_monocytogenes_1639_v24	Listeria_monocytogenes_1639_v147	0.9367
Listeria_monocytogenes_1639_v23	Listeria_monocytogenes_1639_v147	0.9364
Listeria_monocytogenes_1639_v22	Listeria_monocytogenes_1639_v147	0.9324
Listeria_monocytogenes_1639_v21	Listeria_monocytogenes_1639_v147	0.9364
Listeria_monocytogenes_1639_v20	Listeria_monocytogenes_1639_v147	0.9263
Listeria_monocytogenes_1639_v1	Listeria_monocytogenes_1639_v147	0.9025
Listeria_monocytogenes_1639_v19	Listeria_monocytogenes_1639_v147	0.9259
Listeria_monocytogenes_1639_v198	Listeria_monocytogenes_1639_v147	0.8857
Listeria_monocytogenes_1639_v197	Listeria_monocytogenes_1639_v147	0.9143
Listeria_monocytogenes_1639_v196	Listeria_monocytogenes_1639_v147	0.9096
Listeria_monocytogenes_1639_v195	Listeria_monocytogenes_1639_v147	0.9339
Listeria_monocytogenes_1639_v194	Listeria_monocytogenes_1639_v147	0.9337
Listeria_monocytogenes_1639_v193	Listeria_monocytogenes_1639_v147	0.9337
Listeria_monocytogenes_1639_v192	Listeria_monocytogenes_1639_v147	0.9008
Listeria_monocytogenes_1639_v191	Listeria_monocytogenes_1639_v147	0.9381
Listeria_monocytogenes_1639_v190	Listeria_monocytogenes_1639_v147	0.9386
Listeria_monocytogenes_1639_v18	Listeria_monocytogenes_1639_v147	0.9552
Listeria_monocytogenes_1639_v189	Listeria_monocytogenes_1639_v147	0.9375
Listeria_monocytogenes_1639_v188	Listeria_monocytogenes_1639_v147	0.9385
Listeria_monocytogenes_1639_v187	Listeria_monocytogenes_1639_v147	0.9385
Listeria_monocytogenes_1639_v186	Listeria_monocytogenes_1639_v147	0.9385
Listeria_monocytogenes_1639_v185	Listeria_monocytogenes_1639_v147	0.9389
Listeria_monocytogenes_1639_v184	Listeria_monocytogenes_1639_v147	0.9378
Listeria_monocytogenes_1639_v183	Listeria_monocytogenes_1639_v147	0.9317
Listeria_monocytogenes_1639_v182	Listeria_monocytogenes_1639_v147	0.9338
Listeria_monocytogenes_1639_v181	Listeria_monocytogenes_1639_v147	0.9207
Listeria_monocytogenes_1639_v180	Listeria_monocytogenes_1639_v147	0.9207
Listeria_monocytogenes_1639_v17	Listeria_monocytogenes_1639_v147	0.9555
Listeria_monocytogenes_1639_v179	Listeria_monocytogenes_1639_v147	0.9233
Listeria_monocytogenes_1639_v178	Listeria_monocytogenes_1639_v147	0.9382
Listeria_monocytogenes_1639_v177	Listeria_monocytogenes_1639_v147	0.9833
Listeria_monocytogenes_1639_v176	Listeria_monocytogenes_1639_v147	0.9322
Listeria_monocytogenes_1639_v175	Listeria_monocytogenes_1639_v147	0.927
Listeria_monocytogenes_1639_v174	Listeria_monocytogenes_1639_v147	0.9387
Listeria_monocytogenes_1639_v173	Listeria_monocytogenes_1639_v147	0.9558
Listeria_monocytogenes_1639_v172	Listeria_monocytogenes_1639_v147	0.9299
Listeria_monocytogenes_1639_v171	Listeria_monocytogenes_1639_v147	0.9275
Listeria_monocytogenes_1639_v170	Listeria_monocytogenes_1639_v147	0.9326
Listeria_monocytogenes_1639_v16	Listeria_monocytogenes_1639_v147	0.9078
Listeria_monocytogenes_1639_v169	Listeria_monocytogenes_1639_v147	0.9315
Listeria_monocytogenes_1639_v168	Listeria_monocytogenes_1639_v147	0.9376
Listeria_monocytogenes_1639_v167	Listeria_monocytogenes_1639_v147	0.925
Listeria_monocytogenes_1639_v166	Listeria_monocytogenes_1639_v147	0.938
Listeria_monocytogenes_1639_v165	Listeria_monocytogenes_1639_v147	0.9311
Listeria_monocytogenes_1639_v164	Listeria_monocytogenes_1639_v147	0.9597
Listeria_monocytogenes_1639_v163	Listeria_monocytogenes_1639_v147	0.953
Listeria_monocytogenes_1639_v162	Listeria_monocytogenes_1639_v147	0.9597
Listeria_monocytogenes_1639_v161	Listeria_monocytogenes_1639_v147	0.9601

Listeria_monocytogenes_1639_v160	Listeria_monocytogenes_1639_v147	0.9598
Listeria_monocytogenes_1639_v15	Listeria_monocytogenes_1639_v147	0.9209
Listeria_monocytogenes_1639_v159	Listeria_monocytogenes_1639_v147	0.9837
Listeria_monocytogenes_1639_v158	Listeria_monocytogenes_1639_v147	0.9602
Listeria_monocytogenes_1639_v157	Listeria_monocytogenes_1639_v147	0.9602
Listeria_monocytogenes_1639_v156	Listeria_monocytogenes_1639_v147	0.9441
Listeria_monocytogenes_1639_v155	Listeria_monocytogenes_1639_v147	0.9268
Listeria_monocytogenes_1639_v154	Listeria_monocytogenes_1639_v147	0.9302
Listeria_monocytogenes_1639_v153	Listeria_monocytogenes_1639_v147	0.9275
Listeria_monocytogenes_1639_v152	Listeria_monocytogenes_1639_v147	0.9837
Listeria_monocytogenes_1639_v151	Listeria_monocytogenes_1639_v147	0.9837
Listeria_monocytogenes_1639_v150	Listeria_monocytogenes_1639_v147	0.9282
Listeria_monocytogenes_1639_v14	Listeria_monocytogenes_1639_v147	0.9298
Listeria_monocytogenes_1639_v149	Listeria_monocytogenes_1639_v147	0.9273
Listeria_monocytogenes_1639_v148	Listeria_monocytogenes_1639_v147	0.983
Listeria_monocytogenes_1639_v147	Listeria_monocytogenes_1639_v147	0.0652
Listeria_welshimeri_1643	Listeria_monocytogenes_1639_v148	0.8805
Listeria_welshimeri_serovar_6b_str_SLCC5334_386043	Listeria_monocytogenes_1639_v148	0.8802
Listeria_seeligeri_serovar_1_2b_str_SLCC3954_683837	Listeria_monocytogenes_1639_v148	0.8889
Listeria_seeligeri_FSL_S4-171_702452	Listeria_monocytogenes_1639_v148	0.8292
Listeria_seeligeri_FSL_N1-067_702453	Listeria_monocytogenes_1639_v148	0.8335
Listeria_seeligeri_1640_v1	Listeria_monocytogenes_1639_v148	0.8966
Listeria_seeligeri_1640	Listeria_monocytogenes_1639_v148	0.8888
Listeria_monocytogenes_WSLC1042_1457188	Listeria_monocytogenes_1639_v148	0.9672
Listeria_monocytogenes_WSLC1001_1457187	Listeria_monocytogenes_1639_v148	0.9357
Listeria_monocytogenes_SLCC7179_879090	Listeria_monocytogenes_1639_v148	0.9409
Listeria_monocytogenes_SLCC5850_882096	Listeria_monocytogenes_1639_v148	0.9408
Listeria_monocytogenes_SLCC2755_932919	Listeria_monocytogenes_1639_v148	0.95
Listeria_monocytogenes_SLCC2540_879089	Listeria_monocytogenes_1639_v148	0.949
Listeria_monocytogenes_SLCC2479_882020	Listeria_monocytogenes_1639_v148	0.932
Listeria_monocytogenes_SLCC2378_879088	Listeria_monocytogenes_1639_v148	0.9683
Listeria_monocytogenes_SLCC2376_882097	Listeria_monocytogenes_1639_v148	0.947
Listeria_monocytogenes_SLCC2372_932920	Listeria_monocytogenes_1639_v148	0.9247
Listeria_monocytogenes_serotype_4b_str_LL195_1230340	Listeria_monocytogenes_1639_v148	0.9742
Listeria_monocytogenes_serotype_4b_str_LIS0087_1134669	Listeria_monocytogenes_1639_v148	0.9175
Listeria_monocytogenes_serotype_4b_str_F2365_265669	Listeria_monocytogenes_1639_v148	0.9742
Listeria_monocytogenes_serotype_4b_str_CLIP_80459_568819	Listeria_monocytogenes_1639_v148	0.9761
Listeria_monocytogenes_serotype_4b_str_81-0861_1196161	Listeria_monocytogenes_1639_v148	0.9569
Listeria_monocytogenes_serotype_4b_str_81-0592_1196160	Listeria_monocytogenes_1639_v148	0.958
Listeria_monocytogenes_serotype_4b_str_81-0558_1196159	Listeria_monocytogenes_1639_v148	0.9576
Listeria_monocytogenes_serotype_4b_str_10-0809_1196162	Listeria_monocytogenes_1639_v148	0.9584
Listeria_monocytogenes_serotype_4b_str_02-6680_1196175	Listeria_monocytogenes_1639_v148	0.9735
Listeria_monocytogenes_serotype_4b_str_02-6679_1196174	Listeria_monocytogenes_1639_v148	0.9735
Listeria_monocytogenes_serotype_4b_str_02-1792_1196173	Listeria_monocytogenes_1639_v148	0.9584
Listeria_monocytogenes_serotype_4b_str_02-1289_1196172	Listeria_monocytogenes_1639_v148	0.9584
Listeria_monocytogenes_serotype_4b_str_02-1103_1196171	Listeria_monocytogenes_1639_v148	0.9584
Listeria_monocytogenes_serotype_3c_str_10-5027_1196195	Listeria_monocytogenes_1639_v148	0.9312
Listeria_monocytogenes_serotype_1_2c_str_10-5026_1196194	Listeria_monocytogenes_1639_v148	0.932
Listeria_monocytogenes_serotype_1_2c_str_10-5025_1196193	Listeria_monocytogenes_1639_v148	0.932
Listeria_monocytogenes_serotype_1_2b_str_10-0811_1196164	Listeria_monocytogenes_1639_v148	0.9439
Listeria_monocytogenes_serotype_1_2b_str_10-0810_1196163	Listeria_monocytogenes_1639_v148	0.9439
Listeria_monocytogenes_serotype_1_2a_str_99-6370_1196179	Listeria_monocytogenes_1639_v148	0.9198
Listeria_monocytogenes_serotype_1_2a_str_98-2035_1196178	Listeria_monocytogenes_1639_v148	0.9198
Listeria_monocytogenes_serotype_1_2a_str_95-0093_1196177	Listeria_monocytogenes_1639_v148	0.9253
Listeria_monocytogenes_serotype_1_2a_str_88-0478_1196176	Listeria_monocytogenes_1639_v148	0.9242
Listeria_monocytogenes_serotype_1_2a_str_10-5024_1196192	Listeria_monocytogenes_1639_v148	0.9253
Listeria_monocytogenes_serotype_1_2a_str_10-4758_1196168	Listeria_monocytogenes_1639_v148	0.9409
Listeria_monocytogenes_serotype_1_2a_str_10-4754_1196167	Listeria_monocytogenes_1639_v148	0.9408
Listeria_monocytogenes_serotype_1_2a_str_10-1321_1196191	Listeria_monocytogenes_1639_v148	0.9253
Listeria_monocytogenes_serotype_1_2a_str_10-1047_1196190	Listeria_monocytogenes_1639_v148	0.926
Listeria_monocytogenes_serotype_1_2a_str_10-1046_1196189	Listeria_monocytogenes_1639_v148	0.926
Listeria_monocytogenes_serotype_1_2a_str_10-0934_1196170	Listeria_monocytogenes_1639_v148	0.9505
Listeria_monocytogenes_serotype_1_2a_str_10-0933_1196169	Listeria_monocytogenes_1639_v148	0.9505
Listeria_monocytogenes_serotype_1_2a_str_10-0815_1196188	Listeria_monocytogenes_1639_v148	0.9212
Listeria_monocytogenes_serotype_1_2a_str_10-0814_1196187	Listeria_monocytogenes_1639_v148	0.9257
Listeria_monocytogenes_serotype_1_2a_str_10-0813_1196166	Listeria_monocytogenes_1639_v148	0.9333
Listeria_monocytogenes_serotype_1_2a_str_10-0812_1196165	Listeria_monocytogenes_1639_v148	0.9333
Listeria_monocytogenes_serotype_1_2a_str_08-7669_1196186	Listeria_monocytogenes_1639_v148	0.9348
Listeria_monocytogenes_serotype_1_2a_str_08-7374_1196185	Listeria_monocytogenes_1639_v148	0.9253
Listeria_monocytogenes_serotype_1_2a_str_08-6997_1196184	Listeria_monocytogenes_1639_v148	0.9212
Listeria_monocytogenes_serotype_1_2a_str_08-6569_1196183	Listeria_monocytogenes_1639_v148	0.9212
Listeria_monocytogenes_serotype_1_2a_str_08-6056_1196182	Listeria_monocytogenes_1639_v148	0.9212
Listeria_monocytogenes_serotype_1_2a_str_04-5457_1196181	Listeria_monocytogenes_1639_v148	0.9253
Listeria_monocytogenes_serotype_1_2a_str_02-5993_1196180	Listeria_monocytogenes_1639_v148	0.9253
Listeria_monocytogenes_R479a_1437838	Listeria_monocytogenes_1639_v148	0.9246
Listeria_monocytogenes_N53-1_1234142	Listeria_monocytogenes_1639_v148	0.8958
Listeria_monocytogenes_M7_1030009	Listeria_monocytogenes_1639_v148	0.9101
Listeria_monocytogenes_LO28_393132	Listeria_monocytogenes_1639_v148	0.9306
Listeria_monocytogenes_L99_563174	Listeria_monocytogenes_1639_v148	0.9104
Listeria_monocytogenes_L312_882094	Listeria_monocytogenes_1639_v148	0.9765
Listeria_monocytogenes_J1816_930781	Listeria_monocytogenes_1639_v148	0.9766
Listeria_monocytogenes_J1-220_930782	Listeria_monocytogenes_1639_v148	0.9493
Listeria_monocytogenes_J0161_393130	Listeria_monocytogenes_1639_v148	0.9291
Listeria_monocytogenes_HCC23_552536	Listeria_monocytogenes_1639_v148	0.9097
Listeria_monocytogenes_FSL_R2-561_393126	Listeria_monocytogenes_1639_v148	0.9306
Listeria_monocytogenes_FSL_J1-208_393119	Listeria_monocytogenes_1639_v148	0.9185
Listeria_monocytogenes_FSL_J1-175_393118	Listeria_monocytogenes_1639_v148	0.9557
Listeria_monocytogenes_Finland_1998_393127	Listeria_monocytogenes_1639_v148	0.952
Listeria_monocytogenes_EGD-e_169963_v1	Listeria_monocytogenes_1639_v148	0.9398
Listeria_monocytogenes_EGD-e_169963	Listeria_monocytogenes_1639_v148	0.9402
Listeria_monocytogenes_EGD_1334565	Listeria_monocytogenes_1639_v148	0.9411

Listeria_monocytogenes_CFSAN002349_1299895	Listeria_monocytogenes_1639_v148	0.9816
Listeria_monocytogenes_ATCC_19117_882095_v1	Listeria_monocytogenes_1639_v148	0.9697
Listeria_monocytogenes_ATCC_19117_882095	Listeria_monocytogenes_1639_v148	0.9693
Listeria_monocytogenes_6179_1288295	Listeria_monocytogenes_1639_v148	0.9137
Listeria_monocytogenes_1639_v9	Listeria_monocytogenes_1639_v148	0.9266
Listeria_monocytogenes_1639_v99	Listeria_monocytogenes_1639_v148	0.9785
Listeria_monocytogenes_1639_v98	Listeria_monocytogenes_1639_v148	0.9282
Listeria_monocytogenes_1639_v97	Listeria_monocytogenes_1639_v148	0.9662
Listeria_monocytogenes_1639_v96	Listeria_monocytogenes_1639_v148	0.9778
Listeria_monocytogenes_1639_v95	Listeria_monocytogenes_1639_v148	0.9774
Listeria_monocytogenes_1639_v94	Listeria_monocytogenes_1639_v148	0.9782
Listeria_monocytogenes_1639_v93	Listeria_monocytogenes_1639_v148	0.9271
Listeria_monocytogenes_1639_v92	Listeria_monocytogenes_1639_v148	0.9782
Listeria_monocytogenes_1639_v91	Listeria_monocytogenes_1639_v148	0.9543
Listeria_monocytogenes_1639_v90	Listeria_monocytogenes_1639_v148	0.9282
Listeria_monocytogenes_1639_v8	Listeria_monocytogenes_1639_v148	0.9144
Listeria_monocytogenes_1639_v89	Listeria_monocytogenes_1639_v148	0.9664
Listeria_monocytogenes_1639_v88	Listeria_monocytogenes_1639_v148	0.9311
Listeria_monocytogenes_1639_v87	Listeria_monocytogenes_1639_v148	0.9291
Listeria_monocytogenes_1639_v86	Listeria_monocytogenes_1639_v148	0.9287
Listeria_monocytogenes_1639_v85	Listeria_monocytogenes_1639_v148	0.9562
Listeria_monocytogenes_1639_v84	Listeria_monocytogenes_1639_v148	0.9347
Listeria_monocytogenes_1639_v83	Listeria_monocytogenes_1639_v148	0.9583
Listeria_monocytogenes_1639_v82	Listeria_monocytogenes_1639_v148	0.958
Listeria_monocytogenes_1639_v81	Listeria_monocytogenes_1639_v148	0.9322
Listeria_monocytogenes_1639_v80	Listeria_monocytogenes_1639_v148	0.9499
Listeria_monocytogenes_1639_v7	Listeria_monocytogenes_1639_v148	0.9144
Listeria_monocytogenes_1639_v79	Listeria_monocytogenes_1639_v148	0.9304
Listeria_monocytogenes_1639_v78	Listeria_monocytogenes_1639_v148	0.9315
Listeria_monocytogenes_1639_v77	Listeria_monocytogenes_1639_v148	0.932
Listeria_monocytogenes_1639_v76	Listeria_monocytogenes_1639_v148	0.9248
Listeria_monocytogenes_1639_v75	Listeria_monocytogenes_1639_v148	0.9104
Listeria_monocytogenes_1639_v74	Listeria_monocytogenes_1639_v148	0.9276
Listeria_monocytogenes_1639_v73	Listeria_monocytogenes_1639_v148	0.9257
Listeria_monocytogenes_1639_v72	Listeria_monocytogenes_1639_v148	0.9568
Listeria_monocytogenes_1639_v71	Listeria_monocytogenes_1639_v148	0.9161
Listeria_monocytogenes_1639_v70	Listeria_monocytogenes_1639_v148	0.9281
Listeria_monocytogenes_1639_v6	Listeria_monocytogenes_1639_v148	0.914
Listeria_monocytogenes_1639_v69	Listeria_monocytogenes_1639_v148	0.958
Listeria_monocytogenes_1639_v68	Listeria_monocytogenes_1639_v148	0.9341
Listeria_monocytogenes_1639_v67	Listeria_monocytogenes_1639_v148	0.9451
Listeria_monocytogenes_1639_v66	Listeria_monocytogenes_1639_v148	0.936
Listeria_monocytogenes_1639_v65	Listeria_monocytogenes_1639_v148	0.9285
Listeria_monocytogenes_1639_v64	Listeria_monocytogenes_1639_v148	0.927
Listeria_monocytogenes_1639_v63	Listeria_monocytogenes_1639_v148	0.945
Listeria_monocytogenes_1639_v62	Listeria_monocytogenes_1639_v148	0.9276
Listeria_monocytogenes_1639_v61	Listeria_monocytogenes_1639_v148	0.9281
Listeria_monocytogenes_1639_v60	Listeria_monocytogenes_1639_v148	0.931
Listeria_monocytogenes_1639_v5	Listeria_monocytogenes_1639_v148	0.9102
Listeria_monocytogenes_1639_v59	Listeria_monocytogenes_1639_v148	0.9587
Listeria_monocytogenes_1639_v58	Listeria_monocytogenes_1639_v148	0.9602
Listeria_monocytogenes_1639_v57	Listeria_monocytogenes_1639_v148	0.9741
Listeria_monocytogenes_1639_v56	Listeria_monocytogenes_1639_v148	0.9587
Listeria_monocytogenes_1639_v55	Listeria_monocytogenes_1639_v148	0.9701
Listeria_monocytogenes_1639_v54	Listeria_monocytogenes_1639_v148	0.9259
Listeria_monocytogenes_1639_v53	Listeria_monocytogenes_1639_v148	0.9314
Listeria_monocytogenes_1639_v52	Listeria_monocytogenes_1639_v148	0.9554
Listeria_monocytogenes_1639_v51	Listeria_monocytogenes_1639_v148	0.9525
Listeria_monocytogenes_1639_v50	Listeria_monocytogenes_1639_v148	0.9246
Listeria_monocytogenes_1639_v4	Listeria_monocytogenes_1639_v148	0.9239
Listeria_monocytogenes_1639_v49	Listeria_monocytogenes_1639_v148	0.9565
Listeria_monocytogenes_1639_v48	Listeria_monocytogenes_1639_v148	0.9264
Listeria_monocytogenes_1639_v47	Listeria_monocytogenes_1639_v148	0.925
Listeria_monocytogenes_1639_v46	Listeria_monocytogenes_1639_v148	0.9334
Listeria_monocytogenes_1639_v45	Listeria_monocytogenes_1639_v148	0.9161
Listeria_monocytogenes_1639_v44	Listeria_monocytogenes_1639_v148	0.9253
Listeria_monocytogenes_1639_v43	Listeria_monocytogenes_1639_v148	0.9253
Listeria_monocytogenes_1639_v42	Listeria_monocytogenes_1639_v148	0.9149
Listeria_monocytogenes_1639_v41	Listeria_monocytogenes_1639_v148	0.9038
Listeria_monocytogenes_1639_v40	Listeria_monocytogenes_1639_v148	0.9309
Listeria_monocytogenes_1639_v3	Listeria_monocytogenes_1639_v148	0.9239
Listeria_monocytogenes_1639_v39	Listeria_monocytogenes_1639_v148	0.9431
Listeria_monocytogenes_1639_v38	Listeria_monocytogenes_1639_v148	0.9281
Listeria_monocytogenes_1639_v37	Listeria_monocytogenes_1639_v148	0.9237
Listeria_monocytogenes_1639_v36	Listeria_monocytogenes_1639_v148	0.9223
Listeria_monocytogenes_1639_v35	Listeria_monocytogenes_1639_v148	0.9316
Listeria_monocytogenes_1639_v34	Listeria_monocytogenes_1639_v148	0.926
Listeria_monocytogenes_1639_v33	Listeria_monocytogenes_1639_v148	0.9439
Listeria_monocytogenes_1639_v32	Listeria_monocytogenes_1639_v148	0.9226
Listeria_monocytogenes_1639_v31	Listeria_monocytogenes_1639_v148	0.9264
Listeria_monocytogenes_1639_v30	Listeria_monocytogenes_1639_v148	0.945
Listeria_monocytogenes_1639_v2	Listeria_monocytogenes_1639_v148	0.9073
Listeria_monocytogenes_1639_v29	Listeria_monocytogenes_1639_v148	0.9178
Listeria_monocytogenes_1639_v28	Listeria_monocytogenes_1639_v148	0.9222
Listeria_monocytogenes_1639_v27	Listeria_monocytogenes_1639_v148	0.9131
Listeria_monocytogenes_1639_v26	Listeria_monocytogenes_1639_v148	0.9128
Listeria_monocytogenes_1639_v25	Listeria_monocytogenes_1639_v148	0.9128
Listeria_monocytogenes_1639_v24	Listeria_monocytogenes_1639_v148	0.9493
Listeria_monocytogenes_1639_v23	Listeria_monocytogenes_1639_v148	0.9489
Listeria_monocytogenes_1639_v22	Listeria_monocytogenes_1639_v148	0.9461
Listeria_monocytogenes_1639_v21	Listeria_monocytogenes_1639_v148	0.9489

Listeria_monocytogenes_1639_v20	Listeria_monocytogenes_1639_v148	0.9372
Listeria_monocytogenes_1639_v1	Listeria_monocytogenes_1639_v148	0.9082
Listeria_monocytogenes_1639_v19	Listeria_monocytogenes_1639_v148	0.9368
Listeria_monocytogenes_1639_v198	Listeria_monocytogenes_1639_v148	0.8977
Listeria_monocytogenes_1639_v197	Listeria_monocytogenes_1639_v148	0.9273
Listeria_monocytogenes_1639_v196	Listeria_monocytogenes_1639_v148	0.9238
Listeria_monocytogenes_1639_v195	Listeria_monocytogenes_1639_v148	0.9481
Listeria_monocytogenes_1639_v194	Listeria_monocytogenes_1639_v148	0.9475
Listeria_monocytogenes_1639_v193	Listeria_monocytogenes_1639_v148	0.9475
Listeria_monocytogenes_1639_v192	Listeria_monocytogenes_1639_v148	0.9103
Listeria_monocytogenes_1639_v191	Listeria_monocytogenes_1639_v148	0.9519
Listeria_monocytogenes_1639_v190	Listeria_monocytogenes_1639_v148	0.9531
Listeria_monocytogenes_1639_v18	Listeria_monocytogenes_1639_v148	0.968
Listeria_monocytogenes_1639_v189	Listeria_monocytogenes_1639_v148	0.952
Listeria_monocytogenes_1639_v188	Listeria_monocytogenes_1639_v148	0.9527
Listeria_monocytogenes_1639_v187	Listeria_monocytogenes_1639_v148	0.9531
Listeria_monocytogenes_1639_v186	Listeria_monocytogenes_1639_v148	0.9531
Listeria_monocytogenes_1639_v185	Listeria_monocytogenes_1639_v148	0.9535
Listeria_monocytogenes_1639_v184	Listeria_monocytogenes_1639_v148	0.9523
Listeria_monocytogenes_1639_v183	Listeria_monocytogenes_1639_v148	0.9458
Listeria_monocytogenes_1639_v182	Listeria_monocytogenes_1639_v148	0.948
Listeria_monocytogenes_1639_v181	Listeria_monocytogenes_1639_v148	0.9337
Listeria_monocytogenes_1639_v180	Listeria_monocytogenes_1639_v148	0.9337
Listeria_monocytogenes_1639_v17	Listeria_monocytogenes_1639_v148	0.9683
Listeria_monocytogenes_1639_v179	Listeria_monocytogenes_1639_v148	0.9367
Listeria_monocytogenes_1639_v178	Listeria_monocytogenes_1639_v148	0.9524
Listeria_monocytogenes_1639_v177	Listeria_monocytogenes_1639_v148	0.9981
Listeria_monocytogenes_1639_v176	Listeria_monocytogenes_1639_v148	0.9452
Listeria_monocytogenes_1639_v175	Listeria_monocytogenes_1639_v148	0.9405
Listeria_monocytogenes_1639_v174	Listeria_monocytogenes_1639_v148	0.9532
Listeria_monocytogenes_1639_v173	Listeria_monocytogenes_1639_v148	0.9702
Listeria_monocytogenes_1639_v172	Listeria_monocytogenes_1639_v148	0.9431
Listeria_monocytogenes_1639_v171	Listeria_monocytogenes_1639_v148	0.9382
Listeria_monocytogenes_1639_v170	Listeria_monocytogenes_1639_v148	0.9465
Listeria_monocytogenes_1639_v16	Listeria_monocytogenes_1639_v148	0.9117
Listeria_monocytogenes_1639_v169	Listeria_monocytogenes_1639_v148	0.9451
Listeria_monocytogenes_1639_v168	Listeria_monocytogenes_1639_v148	0.9502
Listeria_monocytogenes_1639_v167	Listeria_monocytogenes_1639_v148	0.9385
Listeria_monocytogenes_1639_v166	Listeria_monocytogenes_1639_v148	0.9505
Listeria_monocytogenes_1639_v165	Listeria_monocytogenes_1639_v148	0.9447
Listeria_monocytogenes_1639_v164	Listeria_monocytogenes_1639_v148	0.9738
Listeria_monocytogenes_1639_v163	Listeria_monocytogenes_1639_v148	0.9634
Listeria_monocytogenes_1639_v162	Listeria_monocytogenes_1639_v148	0.9738
Listeria_monocytogenes_1639_v161	Listeria_monocytogenes_1639_v148	0.9742
Listeria_monocytogenes_1639_v160	Listeria_monocytogenes_1639_v148	0.9738
Listeria_monocytogenes_1639_v15	Listeria_monocytogenes_1639_v148	0.9297
Listeria_monocytogenes_1639_v159	Listeria_monocytogenes_1639_v148	0.9985
Listeria_monocytogenes_1639_v158	Listeria_monocytogenes_1639_v148	0.9742
Listeria_monocytogenes_1639_v157	Listeria_monocytogenes_1639_v148	0.9742
Listeria_monocytogenes_1639_v156	Listeria_monocytogenes_1639_v148	0.9297
Listeria_monocytogenes_1639_v155	Listeria_monocytogenes_1639_v148	0.9406
Listeria_monocytogenes_1639_v154	Listeria_monocytogenes_1639_v148	0.9441
Listeria_monocytogenes_1639_v153	Listeria_monocytogenes_1639_v148	0.9414
Listeria_monocytogenes_1639_v152	Listeria_monocytogenes_1639_v148	0.9985
Listeria_monocytogenes_1639_v151	Listeria_monocytogenes_1639_v148	0.9985
Listeria_monocytogenes_1639_v150	Listeria_monocytogenes_1639_v148	0.9425
Listeria_monocytogenes_1639_v14	Listeria_monocytogenes_1639_v148	0.9366
Listeria_monocytogenes_1639_v149	Listeria_monocytogenes_1639_v148	0.9415
Listeria_monocytogenes_1639_v148	Listeria_monocytogenes_1639_v148	0.0649
Listeria_welshimeri_1643	Listeria_monocytogenes_1639_v149	0.8807
Listeria_welshimeri_serovar_6b_str_SLCC5334_386043	Listeria_monocytogenes_1639_v149	0.8803
Listeria_seeligeri_serovar_1_2b_str_SLCC3954_683837	Listeria_monocytogenes_1639_v149	0.8844
Listeria_seeligeri_FSL_S4-171_702452	Listeria_monocytogenes_1639_v149	0.819
Listeria_seeligeri_FSL_N1-067_702453	Listeria_monocytogenes_1639_v149	0.8346
Listeria_seeligeri_1640_v1	Listeria_monocytogenes_1639_v149	0.8824
Listeria_seeligeri_1640	Listeria_monocytogenes_1639_v149	0.8772
Listeria_monocytogenes_WSLC1042_1457188	Listeria_monocytogenes_1639_v149	0.93
Listeria_monocytogenes_WSLC1001_1457187	Listeria_monocytogenes_1639_v149	0.9936
Listeria_monocytogenes_SLCC7179_879090	Listeria_monocytogenes_1639_v149	0.9595
Listeria_monocytogenes_SLCC5850_882096	Listeria_monocytogenes_1639_v149	0.9989
Listeria_monocytogenes_SLCC2755_932919	Listeria_monocytogenes_1639_v149	0.9347
Listeria_monocytogenes_SLCC2540_879089	Listeria_monocytogenes_1639_v149	0.9481
Listeria_monocytogenes_SLCC2479_882020	Listeria_monocytogenes_1639_v149	0.9556
Listeria_monocytogenes_SLCC2378_879088	Listeria_monocytogenes_1639_v149	0.9311
Listeria_monocytogenes_SLCC2376_882097	Listeria_monocytogenes_1639_v149	0.9438
Listeria_monocytogenes_SLCC2372_932920	Listeria_monocytogenes_1639_v149	0.9481
Listeria_monocytogenes_serotype_4b_str_LL195_1230340	Listeria_monocytogenes_1639_v149	0.9371
Listeria_monocytogenes_serotype_4b_str_LIS0087_1134669	Listeria_monocytogenes_1639_v149	0.9647
Listeria_monocytogenes_serotype_4b_str_F2365_265669	Listeria_monocytogenes_1639_v149	0.9371
Listeria_monocytogenes_serotype_4b_str_CLIP_80459_568819	Listeria_monocytogenes_1639_v149	0.944
Listeria_monocytogenes_serotype_4b_str_81-0861_1196161	Listeria_monocytogenes_1639_v149	0.9228
Listeria_monocytogenes_serotype_4b_str_81-0592_1196160	Listeria_monocytogenes_1639_v149	0.9239
Listeria_monocytogenes_serotype_4b_str_81-0558_1196159	Listeria_monocytogenes_1639_v149	0.9246
Listeria_monocytogenes_serotype_4b_str_10-0809_1196162	Listeria_monocytogenes_1639_v149	0.9242
Listeria_monocytogenes_serotype_4b_str_02-6680_1196175	Listeria_monocytogenes_1639_v149	0.9426
Listeria_monocytogenes_serotype_4b_str_02-6679_1196174	Listeria_monocytogenes_1639_v149	0.9426
Listeria_monocytogenes_serotype_4b_str_02-1792_1196173	Listeria_monocytogenes_1639_v149	0.9239
Listeria_monocytogenes_serotype_4b_str_02-1289_1196172	Listeria_monocytogenes_1639_v149	0.9239
Listeria_monocytogenes_serotype_4b_str_02-1103_1196171	Listeria_monocytogenes_1639_v149	0.9239
Listeria_monocytogenes_serotype_3c_str_10-5027_1196195	Listeria_monocytogenes_1639_v149	0.9567
Listeria_monocytogenes_serotype_1_2c_str_10-5026_1196194	Listeria_monocytogenes_1639_v149	0.9571

Listeria_monocytogenes_serotype_1_2c_str_10-5025_1196193	Listeria_monocytogenes_1639_v149	0.9571
Listeria_monocytogenes_serotype_1_2b_str_10-0811_1196164	Listeria_monocytogenes_1639_v149	0.9401
Listeria_monocytogenes_serotype_1_2b_str_10-0810_1196163	Listeria_monocytogenes_1639_v149	0.9401
Listeria_monocytogenes_serotype_1_2a_str_99-6370_1196179	Listeria_monocytogenes_1639_v149	0.948
Listeria_monocytogenes_serotype_1_2a_str_98-2035_1196178	Listeria_monocytogenes_1639_v149	0.948
Listeria_monocytogenes_serotype_1_2a_str_95-0093_1196177	Listeria_monocytogenes_1639_v149	0.9525
Listeria_monocytogenes_serotype_1_2a_str_88-0478_1196176	Listeria_monocytogenes_1639_v149	0.9525
Listeria_monocytogenes_serotype_1_2a_str_10-5024_1196192	Listeria_monocytogenes_1639_v149	0.9529
Listeria_monocytogenes_serotype_1_2a_str_10-4758_1196168	Listeria_monocytogenes_1639_v149	0.9681
Listeria_monocytogenes_serotype_1_2a_str_10-4754_1196167	Listeria_monocytogenes_1639_v149	0.9685
Listeria_monocytogenes_serotype_1_2a_str_10-1321_1196191	Listeria_monocytogenes_1639_v149	0.9529
Listeria_monocytogenes_serotype_1_2a_str_10-1047_1196190	Listeria_monocytogenes_1639_v149	0.9536
Listeria_monocytogenes_serotype_1_2a_str_10-1046_1196189	Listeria_monocytogenes_1639_v149	0.9536
Listeria_monocytogenes_serotype_1_2a_str_10-0934_1196170	Listeria_monocytogenes_1639_v149	0.9738
Listeria_monocytogenes_serotype_1_2a_str_10-0933_1196169	Listeria_monocytogenes_1639_v149	0.9738
Listeria_monocytogenes_serotype_1_2a_str_10-0815_1196188	Listeria_monocytogenes_1639_v149	0.9491
Listeria_monocytogenes_serotype_1_2a_str_10-0814_1196187	Listeria_monocytogenes_1639_v149	0.9529
Listeria_monocytogenes_serotype_1_2a_str_10-0813_1196166	Listeria_monocytogenes_1639_v149	0.9796
Listeria_monocytogenes_serotype_1_2a_str_10-0812_1196165	Listeria_monocytogenes_1639_v149	0.9796
Listeria_monocytogenes_serotype_1_2a_str_08-7669_1196186	Listeria_monocytogenes_1639_v149	0.9513
Listeria_monocytogenes_serotype_1_2a_str_08-7374_1196185	Listeria_monocytogenes_1639_v149	0.9529
Listeria_monocytogenes_serotype_1_2a_str_08-6997_1196184	Listeria_monocytogenes_1639_v149	0.9491
Listeria_monocytogenes_serotype_1_2a_str_08-6569_1196183	Listeria_monocytogenes_1639_v149	0.9491
Listeria_monocytogenes_serotype_1_2a_str_08-6056_1196182	Listeria_monocytogenes_1639_v149	0.9491
Listeria_monocytogenes_serotype_1_2a_str_04-5457_1196181	Listeria_monocytogenes_1639_v149	0.9529
Listeria_monocytogenes_serotype_1_2a_str_02-5993_1196180	Listeria_monocytogenes_1639_v149	0.9529
Listeria_monocytogenes_R479a_1437838	Listeria_monocytogenes_1639_v149	0.9507
Listeria_monocytogenes_N53-1_1234142	Listeria_monocytogenes_1639_v149	0.9087
Listeria_monocytogenes_M7_1030009	Listeria_monocytogenes_1639_v149	0.9138
Listeria_monocytogenes_LO28_393132	Listeria_monocytogenes_1639_v149	0.9542
Listeria_monocytogenes_L99_563174	Listeria_monocytogenes_1639_v149	0.9141
Listeria_monocytogenes_L312_882094	Listeria_monocytogenes_1639_v149	0.944
Listeria_monocytogenes_J1816_930781	Listeria_monocytogenes_1639_v149	0.9253
Listeria_monocytogenes_J1-220_930782	Listeria_monocytogenes_1639_v149	0.9282
Listeria_monocytogenes_J0161_393130	Listeria_monocytogenes_1639_v149	0.9455
Listeria_monocytogenes_HCC23_552536	Listeria_monocytogenes_1639_v149	0.9138
Listeria_monocytogenes_FSL_R2-561_393126	Listeria_monocytogenes_1639_v149	0.9553
Listeria_monocytogenes_FSL_J1-208_393119	Listeria_monocytogenes_1639_v149	0.9086
Listeria_monocytogenes_FSL_J1-175_393118	Listeria_monocytogenes_1639_v149	0.9521
Listeria_monocytogenes_Finland_1998_393127	Listeria_monocytogenes_1639_v149	0.9619
Listeria_monocytogenes_EGD-e_169963_v1	Listeria_monocytogenes_1639_v149	0.9541
Listeria_monocytogenes_EGD-e_169963	Listeria_monocytogenes_1639_v149	0.9541
Listeria_monocytogenes_EGD_1334565	Listeria_monocytogenes_1639_v149	0.9996
Listeria_monocytogenes_CFSAN002349_1299895	Listeria_monocytogenes_1639_v149	0.9422
Listeria_monocytogenes_ATCC_19117_882095_v1	Listeria_monocytogenes_1639_v149	0.9374
Listeria_monocytogenes_ATCC_19117_882095	Listeria_monocytogenes_1639_v149	0.937
Listeria_monocytogenes_6179_1288295	Listeria_monocytogenes_1639_v149	0.9441
Listeria_monocytogenes_1639_v9	Listeria_monocytogenes_1639_v149	0.925
Listeria_monocytogenes_1639_v99	Listeria_monocytogenes_1639_v149	0.9276
Listeria_monocytogenes_1639_v98	Listeria_monocytogenes_1639_v149	0.9466
Listeria_monocytogenes_1639_v97	Listeria_monocytogenes_1639_v149	0.933
Listeria_monocytogenes_1639_v96	Listeria_monocytogenes_1639_v149	0.925
Listeria_monocytogenes_1639_v95	Listeria_monocytogenes_1639_v149	0.925
Listeria_monocytogenes_1639_v94	Listeria_monocytogenes_1639_v149	0.9257
Listeria_monocytogenes_1639_v93	Listeria_monocytogenes_1639_v149	0.9518
Listeria_monocytogenes_1639_v92	Listeria_monocytogenes_1639_v149	0.9269
Listeria_monocytogenes_1639_v91	Listeria_monocytogenes_1639_v149	0.9301
Listeria_monocytogenes_1639_v90	Listeria_monocytogenes_1639_v149	0.9466
Listeria_monocytogenes_1639_v8	Listeria_monocytogenes_1639_v149	0.9398
Listeria_monocytogenes_1639_v89	Listeria_monocytogenes_1639_v149	0.9381
Listeria_monocytogenes_1639_v88	Listeria_monocytogenes_1639_v149	0.9664
Listeria_monocytogenes_1639_v87	Listeria_monocytogenes_1639_v149	0.9767
Listeria_monocytogenes_1639_v86	Listeria_monocytogenes_1639_v149	0.9763
Listeria_monocytogenes_1639_v85	Listeria_monocytogenes_1639_v149	0.9225
Listeria_monocytogenes_1639_v84	Listeria_monocytogenes_1639_v149	0.9618
Listeria_monocytogenes_1639_v83	Listeria_monocytogenes_1639_v149	0.9287
Listeria_monocytogenes_1639_v82	Listeria_monocytogenes_1639_v149	0.9283
Listeria_monocytogenes_1639_v81	Listeria_monocytogenes_1639_v149	0.9562
Listeria_monocytogenes_1639_v80	Listeria_monocytogenes_1639_v149	0.9444
Listeria_monocytogenes_1639_v7	Listeria_monocytogenes_1639_v149	0.9398
Listeria_monocytogenes_1639_v79	Listeria_monocytogenes_1639_v149	0.9529
Listeria_monocytogenes_1639_v78	Listeria_monocytogenes_1639_v149	0.9555
Listeria_monocytogenes_1639_v77	Listeria_monocytogenes_1639_v149	0.9489
Listeria_monocytogenes_1639_v76	Listeria_monocytogenes_1639_v149	0.9536
Listeria_monocytogenes_1639_v75	Listeria_monocytogenes_1639_v149	0.9141
Listeria_monocytogenes_1639_v74	Listeria_monocytogenes_1639_v149	0.9609
Listeria_monocytogenes_1639_v73	Listeria_monocytogenes_1639_v149	0.9507
Listeria_monocytogenes_1639_v72	Listeria_monocytogenes_1639_v149	0.9271
Listeria_monocytogenes_1639_v71	Listeria_monocytogenes_1639_v149	0.9463
Listeria_monocytogenes_1639_v70	Listeria_monocytogenes_1639_v149	0.9532
Listeria_monocytogenes_1639_v6	Listeria_monocytogenes_1639_v149	0.9395
Listeria_monocytogenes_1639_v69	Listeria_monocytogenes_1639_v149	0.9239
Listeria_monocytogenes_1639_v68	Listeria_monocytogenes_1639_v149	0.9464
Listeria_monocytogenes_1639_v67	Listeria_monocytogenes_1639_v149	0.9419
Listeria_monocytogenes_1639_v66	Listeria_monocytogenes_1639_v149	0.9666
Listeria_monocytogenes_1639_v65	Listeria_monocytogenes_1639_v149	0.955
Listeria_monocytogenes_1639_v64	Listeria_monocytogenes_1639_v149	0.9593
Listeria_monocytogenes_1639_v63	Listeria_monocytogenes_1639_v149	0.9407
Listeria_monocytogenes_1639_v62	Listeria_monocytogenes_1639_v149	0.9512
Listeria_monocytogenes_1639_v61	Listeria_monocytogenes_1639_v149	0.9554
Listeria_monocytogenes_1639_v60	Listeria_monocytogenes_1639_v149	0.9487

Listeria_monocytogenes_1639_v5	Listeria_monocytogenes_1639_v149	0.8965
Listeria_monocytogenes_1639_v59	Listeria_monocytogenes_1639_v149	0.9366
Listeria_monocytogenes_1639_v58	Listeria_monocytogenes_1639_v149	0.9298
Listeria_monocytogenes_1639_v57	Listeria_monocytogenes_1639_v149	0.9231
Listeria_monocytogenes_1639_v56	Listeria_monocytogenes_1639_v149	0.9301
Listeria_monocytogenes_1639_v55	Listeria_monocytogenes_1639_v149	0.92
Listeria_monocytogenes_1639_v54	Listeria_monocytogenes_1639_v149	0.9236
Listeria_monocytogenes_1639_v53	Listeria_monocytogenes_1639_v149	0.946
Listeria_monocytogenes_1639_v52	Listeria_monocytogenes_1639_v149	0.9345
Listeria_monocytogenes_1639_v51	Listeria_monocytogenes_1639_v149	0.9249
Listeria_monocytogenes_1639_v50	Listeria_monocytogenes_1639_v149	0.9514
Listeria_monocytogenes_1639_v4	Listeria_monocytogenes_1639_v149	0.9096
Listeria_monocytogenes_1639_v49	Listeria_monocytogenes_1639_v149	0.9254
Listeria_monocytogenes_1639_v48	Listeria_monocytogenes_1639_v149	0.9525
Listeria_monocytogenes_1639_v47	Listeria_monocytogenes_1639_v149	0.9533
Listeria_monocytogenes_1639_v46	Listeria_monocytogenes_1639_v149	0.9483
Listeria_monocytogenes_1639_v45	Listeria_monocytogenes_1639_v149	0.943
Listeria_monocytogenes_1639_v44	Listeria_monocytogenes_1639_v149	0.9529
Listeria_monocytogenes_1639_v43	Listeria_monocytogenes_1639_v149	0.9529
Listeria_monocytogenes_1639_v42	Listeria_monocytogenes_1639_v149	0.9376
Listeria_monocytogenes_1639_v41	Listeria_monocytogenes_1639_v149	0.9357
Listeria_monocytogenes_1639_v40	Listeria_monocytogenes_1639_v149	0.9564
Listeria_monocytogenes_1639_v3	Listeria_monocytogenes_1639_v149	0.91
Listeria_monocytogenes_1639_v39	Listeria_monocytogenes_1639_v149	0.9377
Listeria_monocytogenes_1639_v38	Listeria_monocytogenes_1639_v149	0.9517
Listeria_monocytogenes_1639_v37	Listeria_monocytogenes_1639_v149	0.9475
Listeria_monocytogenes_1639_v36	Listeria_monocytogenes_1639_v149	0.9509
Listeria_monocytogenes_1639_v35	Listeria_monocytogenes_1639_v149	0.9556
Listeria_monocytogenes_1639_v34	Listeria_monocytogenes_1639_v149	0.9521
Listeria_monocytogenes_1639_v33	Listeria_monocytogenes_1639_v149	0.9401
Listeria_monocytogenes_1639_v32	Listeria_monocytogenes_1639_v149	0.9475
Listeria_monocytogenes_1639_v31	Listeria_monocytogenes_1639_v149	0.9525
Listeria_monocytogenes_1639_v30	Listeria_monocytogenes_1639_v149	0.9317
Listeria_monocytogenes_1639_v2	Listeria_monocytogenes_1639_v149	0.9005
Listeria_monocytogenes_1639_v29	Listeria_monocytogenes_1639_v149	0.9426
Listeria_monocytogenes_1639_v28	Listeria_monocytogenes_1639_v149	0.9475
Listeria_monocytogenes_1639_v27	Listeria_monocytogenes_1639_v149	0.9377
Listeria_monocytogenes_1639_v26	Listeria_monocytogenes_1639_v149	0.937
Listeria_monocytogenes_1639_v25	Listeria_monocytogenes_1639_v149	0.937
Listeria_monocytogenes_1639_v24	Listeria_monocytogenes_1639_v149	0.9293
Listeria_monocytogenes_1639_v23	Listeria_monocytogenes_1639_v149	0.929
Listeria_monocytogenes_1639_v22	Listeria_monocytogenes_1639_v149	0.9407
Listeria_monocytogenes_1639_v21	Listeria_monocytogenes_1639_v149	0.9293
Listeria_monocytogenes_1639_v20	Listeria_monocytogenes_1639_v149	0.9371
Listeria_monocytogenes_1639_v1	Listeria_monocytogenes_1639_v149	0.9383
Listeria_monocytogenes_1639_v19	Listeria_monocytogenes_1639_v149	0.9367
Listeria_monocytogenes_1639_v198	Listeria_monocytogenes_1639_v149	0.9103
Listeria_monocytogenes_1639_v197	Listeria_monocytogenes_1639_v149	0.9411
Listeria_monocytogenes_1639_v196	Listeria_monocytogenes_1639_v149	0.9834
Listeria_monocytogenes_1639_v195	Listeria_monocytogenes_1639_v149	0.946
Listeria_monocytogenes_1639_v194	Listeria_monocytogenes_1639_v149	0.9438
Listeria_monocytogenes_1639_v193	Listeria_monocytogenes_1639_v149	0.9442
Listeria_monocytogenes_1639_v192	Listeria_monocytogenes_1639_v149	0.902
Listeria_monocytogenes_1639_v191	Listeria_monocytogenes_1639_v149	0.9695
Listeria_monocytogenes_1639_v190	Listeria_monocytogenes_1639_v149	0.9861
Listeria_monocytogenes_1639_v18	Listeria_monocytogenes_1639_v149	0.9176
Listeria_monocytogenes_1639_v189	Listeria_monocytogenes_1639_v149	0.9869
Listeria_monocytogenes_1639_v188	Listeria_monocytogenes_1639_v149	0.9645
Listeria_monocytogenes_1639_v187	Listeria_monocytogenes_1639_v149	0.9861
Listeria_monocytogenes_1639_v186	Listeria_monocytogenes_1639_v149	0.9865
Listeria_monocytogenes_1639_v185	Listeria_monocytogenes_1639_v149	0.9865
Listeria_monocytogenes_1639_v184	Listeria_monocytogenes_1639_v149	0.9853
Listeria_monocytogenes_1639_v183	Listeria_monocytogenes_1639_v149	0.9614
Listeria_monocytogenes_1639_v182	Listeria_monocytogenes_1639_v149	0.9637
Listeria_monocytogenes_1639_v181	Listeria_monocytogenes_1639_v149	0.9195
Listeria_monocytogenes_1639_v180	Listeria_monocytogenes_1639_v149	0.9195
Listeria_monocytogenes_1639_v17	Listeria_monocytogenes_1639_v149	0.918
Listeria_monocytogenes_1639_v179	Listeria_monocytogenes_1639_v149	0.9549
Listeria_monocytogenes_1639_v178	Listeria_monocytogenes_1639_v149	0.9664
Listeria_monocytogenes_1639_v177	Listeria_monocytogenes_1639_v149	0.9422
Listeria_monocytogenes_1639_v176	Listeria_monocytogenes_1639_v149	0.9346
Listeria_monocytogenes_1639_v175	Listeria_monocytogenes_1639_v149	0.971
Listeria_monocytogenes_1639_v174	Listeria_monocytogenes_1639_v149	0.97
Listeria_monocytogenes_1639_v173	Listeria_monocytogenes_1639_v149	0.9511
Listeria_monocytogenes_1639_v172	Listeria_monocytogenes_1639_v149	0.9352
Listeria_monocytogenes_1639_v171	Listeria_monocytogenes_1639_v149	0.9727
Listeria_monocytogenes_1639_v170	Listeria_monocytogenes_1639_v149	0.9809
Listeria_monocytogenes_1639_v16	Listeria_monocytogenes_1639_v149	0.9393
Listeria_monocytogenes_1639_v169	Listeria_monocytogenes_1639_v149	0.9736
Listeria_monocytogenes_1639_v168	Listeria_monocytogenes_1639_v149	0.9612
Listeria_monocytogenes_1639_v167	Listeria_monocytogenes_1639_v149	0.9575
Listeria_monocytogenes_1639_v166	Listeria_monocytogenes_1639_v149	0.9615
Listeria_monocytogenes_1639_v165	Listeria_monocytogenes_1639_v149	0.9732
Listeria_monocytogenes_1639_v164	Listeria_monocytogenes_1639_v149	0.9367
Listeria_monocytogenes_1639_v163	Listeria_monocytogenes_1639_v149	0.946
Listeria_monocytogenes_1639_v162	Listeria_monocytogenes_1639_v149	0.9367
Listeria_monocytogenes_1639_v161	Listeria_monocytogenes_1639_v149	0.9378
Listeria_monocytogenes_1639_v160	Listeria_monocytogenes_1639_v149	0.9375
Listeria_monocytogenes_1639_v15	Listeria_monocytogenes_1639_v149	0.9248
Listeria_monocytogenes_1639_v159	Listeria_monocytogenes_1639_v149	0.9422
Listeria_monocytogenes_1639_v158	Listeria_monocytogenes_1639_v149	0.9371

Listeria_monocytogenes_1639_v157	Listeria_monocytogenes_1639_v149	0.9371
Listeria_monocytogenes_1639_v156	Listeria_monocytogenes_1639_v149	0.9642
Listeria_monocytogenes_1639_v155	Listeria_monocytogenes_1639_v149	0.9565
Listeria_monocytogenes_1639_v154	Listeria_monocytogenes_1639_v149	0.9706
Listeria_monocytogenes_1639_v153	Listeria_monocytogenes_1639_v149	0.9542
Listeria_monocytogenes_1639_v152	Listeria_monocytogenes_1639_v149	0.9422
Listeria_monocytogenes_1639_v151	Listeria_monocytogenes_1639_v149	0.9422
Listeria_monocytogenes_1639_v150	Listeria_monocytogenes_1639_v149	0.9893
Listeria_monocytogenes_1639_v14	Listeria_monocytogenes_1639_v149	0.9206
Listeria_monocytogenes_1639_v149	Listeria_monocytogenes_1639_v149	0.0682
Listeria_welshimeri_1643	Listeria_monocytogenes_1639_v14	0.8556
Listeria_welshimeri_serovar_6b_str_SLCC5334_386043	Listeria_monocytogenes_1639_v14	0.855
Listeria_seeligeri_serovar_1_2b_str_SLCC3954_683837	Listeria_monocytogenes_1639_v14	0.8669
Listeria_seeligeri_FSL_S4-171_702452	Listeria_monocytogenes_1639_v14	0.8079
Listeria_seeligeri_FSL_N1-067_702453	Listeria_monocytogenes_1639_v14	0.8213
Listeria_seeligeri_1640_v1	Listeria_monocytogenes_1639_v14	0.87
Listeria_seeligeri_1640	Listeria_monocytogenes_1639_v14	0.8695
Listeria_monocytogenes_WSLC1042_1457188	Listeria_monocytogenes_1639_v14	0.9299
Listeria_monocytogenes_WSLC1001_1457187	Listeria_monocytogenes_1639_v14	0.9169
Listeria_monocytogenes_SLCC7179_879090	Listeria_monocytogenes_1639_v14	0.9299
Listeria_monocytogenes_SLCC5850_882096	Listeria_monocytogenes_1639_v14	0.9199
Listeria_monocytogenes_SLCC2755_932919	Listeria_monocytogenes_1639_v14	0.9455
Listeria_monocytogenes_SLCC2540_879089	Listeria_monocytogenes_1639_v14	0.9346
Listeria_monocytogenes_SLCC2479_882020	Listeria_monocytogenes_1639_v14	0.9219
Listeria_monocytogenes_SLCC2378_879088	Listeria_monocytogenes_1639_v14	0.9313
Listeria_monocytogenes_SLCC2376_882097	Listeria_monocytogenes_1639_v14	0.9188
Listeria_monocytogenes_SLCC2372_932920	Listeria_monocytogenes_1639_v14	0.9188
Listeria_monocytogenes_serotype_4b_str_LL195_1230340	Listeria_monocytogenes_1639_v14	0.9368
Listeria_monocytogenes_serotype_4b_str_LIS0087_1134669	Listeria_monocytogenes_1639_v14	0.9079
Listeria_monocytogenes_serotype_4b_str_F2365_265669	Listeria_monocytogenes_1639_v14	0.9368
Listeria_monocytogenes_serotype_4b_str_CLIP_80459_568819	Listeria_monocytogenes_1639_v14	0.9375
Listeria_monocytogenes_serotype_4b_str_81-0861_1196161	Listeria_monocytogenes_1639_v14	0.9346
Listeria_monocytogenes_serotype_4b_str_81-0592_1196160	Listeria_monocytogenes_1639_v14	0.9357
Listeria_monocytogenes_serotype_4b_str_81-0558_1196159	Listeria_monocytogenes_1639_v14	0.9353
Listeria_monocytogenes_serotype_4b_str_10-0809_1196162	Listeria_monocytogenes_1639_v14	0.9361
Listeria_monocytogenes_serotype_4b_str_02-6680_1196175	Listeria_monocytogenes_1639_v14	0.9332
Listeria_monocytogenes_serotype_4b_str_02-6679_1196174	Listeria_monocytogenes_1639_v14	0.9332
Listeria_monocytogenes_serotype_4b_str_02-1792_1196173	Listeria_monocytogenes_1639_v14	0.9368
Listeria_monocytogenes_serotype_4b_str_02-1289_1196172	Listeria_monocytogenes_1639_v14	0.9368
Listeria_monocytogenes_serotype_4b_str_02-1103_1196171	Listeria_monocytogenes_1639_v14	0.9368
Listeria_monocytogenes_serotype_3c_str_10-5027_1196195	Listeria_monocytogenes_1639_v14	0.9218
Listeria_monocytogenes_serotype_1_2c_str_10-5026_1196194	Listeria_monocytogenes_1639_v14	0.9229
Listeria_monocytogenes_serotype_1_2c_str_10-5025_1196193	Listeria_monocytogenes_1639_v14	0.9229
Listeria_monocytogenes_serotype_1_2b_str_10-0811_1196164	Listeria_monocytogenes_1639_v14	0.9642
Listeria_monocytogenes_serotype_1_2b_str_10-0810_1196163	Listeria_monocytogenes_1639_v14	0.9642
Listeria_monocytogenes_serotype_1_2a_str_99-6370_1196179	Listeria_monocytogenes_1639_v14	0.9192
Listeria_monocytogenes_serotype_1_2a_str_98-2035_1196178	Listeria_monocytogenes_1639_v14	0.9192
Listeria_monocytogenes_serotype_1_2a_str_95-0093_1196177	Listeria_monocytogenes_1639_v14	0.9155
Listeria_monocytogenes_serotype_1_2a_str_88-0478_1196176	Listeria_monocytogenes_1639_v14	0.9139
Listeria_monocytogenes_serotype_1_2a_str_10-5024_1196192	Listeria_monocytogenes_1639_v14	0.9158
Listeria_monocytogenes_serotype_1_2a_str_10-4758_1196168	Listeria_monocytogenes_1639_v14	0.9215
Listeria_monocytogenes_serotype_1_2a_str_10-4754_1196167	Listeria_monocytogenes_1639_v14	0.9215
Listeria_monocytogenes_serotype_1_2a_str_10-1321_1196191	Listeria_monocytogenes_1639_v14	0.9158
Listeria_monocytogenes_serotype_1_2a_str_10-1047_1196190	Listeria_monocytogenes_1639_v14	0.9158
Listeria_monocytogenes_serotype_1_2a_str_10-1046_1196189	Listeria_monocytogenes_1639_v14	0.9158
Listeria_monocytogenes_serotype_1_2a_str_10-0934_1196170	Listeria_monocytogenes_1639_v14	0.9268
Listeria_monocytogenes_serotype_1_2a_str_10-0933_1196169	Listeria_monocytogenes_1639_v14	0.9268
Listeria_monocytogenes_serotype_1_2a_str_10-0815_1196188	Listeria_monocytogenes_1639_v14	0.9198
Listeria_monocytogenes_serotype_1_2a_str_10-0814_1196187	Listeria_monocytogenes_1639_v14	0.9158
Listeria_monocytogenes_serotype_1_2a_str_10-0813_1196166	Listeria_monocytogenes_1639_v14	0.926
Listeria_monocytogenes_serotype_1_2a_str_10-0812_1196165	Listeria_monocytogenes_1639_v14	0.926
Listeria_monocytogenes_serotype_1_2a_str_08-7669_1196186	Listeria_monocytogenes_1639_v14	0.9219
Listeria_monocytogenes_serotype_1_2a_str_08-7374_1196185	Listeria_monocytogenes_1639_v14	0.9158
Listeria_monocytogenes_serotype_1_2a_str_08-6997_1196184	Listeria_monocytogenes_1639_v14	0.9198
Listeria_monocytogenes_serotype_1_2a_str_08-6569_1196183	Listeria_monocytogenes_1639_v14	0.9198
Listeria_monocytogenes_serotype_1_2a_str_08-6056_1196182	Listeria_monocytogenes_1639_v14	0.9198
Listeria_monocytogenes_serotype_1_2a_str_04-5457_1196181	Listeria_monocytogenes_1639_v14	0.9158
Listeria_monocytogenes_serotype_1_2a_str_02-5993_1196180	Listeria_monocytogenes_1639_v14	0.9155
Listeria_monocytogenes_R479a_1437838	Listeria_monocytogenes_1639_v14	0.9133
Listeria_monocytogenes_N53-1_1234142	Listeria_monocytogenes_1639_v14	0.8848
Listeria_monocytogenes_M7_1030009	Listeria_monocytogenes_1639_v14	0.8988
Listeria_monocytogenes_LO28_393132	Listeria_monocytogenes_1639_v14	0.9264
Listeria_monocytogenes_L99_563174	Listeria_monocytogenes_1639_v14	0.8995
Listeria_monocytogenes_L312_882094	Listeria_monocytogenes_1639_v14	0.9379
Listeria_monocytogenes_J1816_930781	Listeria_monocytogenes_1639_v14	0.9275
Listeria_monocytogenes_J1-220_930782	Listeria_monocytogenes_1639_v14	0.9307
Listeria_monocytogenes_J0161_393130	Listeria_monocytogenes_1639_v14	0.9252
Listeria_monocytogenes_HCC23_552536	Listeria_monocytogenes_1639_v14	0.8988
Listeria_monocytogenes_FSL_R2-561_393126	Listeria_monocytogenes_1639_v14	0.9205
Listeria_monocytogenes_FSL_J1-208_393119	Listeria_monocytogenes_1639_v14	0.8879
Listeria_monocytogenes_FSL_J1-175_393118	Listeria_monocytogenes_1639_v14	0.9373
Listeria_monocytogenes_Finland_1998_393127	Listeria_monocytogenes_1639_v14	0.9272
Listeria_monocytogenes_EGD-e_169963_v1	Listeria_monocytogenes_1639_v14	0.9338
Listeria_monocytogenes_EGD-e_169963	Listeria_monocytogenes_1639_v14	0.9341
Listeria_monocytogenes_EGD_1334565	Listeria_monocytogenes_1639_v14	0.9206
Listeria_monocytogenes_CFSAN002349_1299895	Listeria_monocytogenes_1639_v14	0.9392
Listeria_monocytogenes_ATCC_19117_882095_v1	Listeria_monocytogenes_1639_v14	0.933
Listeria_monocytogenes_ATCC_19117_882095	Listeria_monocytogenes_1639_v14	0.933
Listeria_monocytogenes_6179_1288295	Listeria_monocytogenes_1639_v14	0.9162
Listeria_monocytogenes_1639_v9	Listeria_monocytogenes_1639_v14	0.9243
Listeria_monocytogenes_1639_v99	Listeria_monocytogenes_1639_v14	0.9352

Listeria_monocytogenes_1639_v98	Listeria_monocytogenes_1639_v14	0.9314
Listeria_monocytogenes_1639_v97	Listeria_monocytogenes_1639_v14	0.9303
Listeria_monocytogenes_1639_v96	Listeria_monocytogenes_1639_v14	0.9349
Listeria_monocytogenes_1639_v95	Listeria_monocytogenes_1639_v14	0.9349
Listeria_monocytogenes_1639_v94	Listeria_monocytogenes_1639_v14	0.9349
Listeria_monocytogenes_1639_v93	Listeria_monocytogenes_1639_v14	0.9147
Listeria_monocytogenes_1639_v92	Listeria_monocytogenes_1639_v14	0.936
Listeria_monocytogenes_1639_v91	Listeria_monocytogenes_1639_v14	0.9344
Listeria_monocytogenes_1639_v90	Listeria_monocytogenes_1639_v14	0.9314
Listeria_monocytogenes_1639_v8	Listeria_monocytogenes_1639_v14	0.9254
Listeria_monocytogenes_1639_v89	Listeria_monocytogenes_1639_v14	0.9404
Listeria_monocytogenes_1639_v88	Listeria_monocytogenes_1639_v14	0.9209
Listeria_monocytogenes_1639_v87	Listeria_monocytogenes_1639_v14	0.923
Listeria_monocytogenes_1639_v86	Listeria_monocytogenes_1639_v14	0.9223
Listeria_monocytogenes_1639_v85	Listeria_monocytogenes_1639_v14	0.9347
Listeria_monocytogenes_1639_v84	Listeria_monocytogenes_1639_v14	0.9196
Listeria_monocytogenes_1639_v83	Listeria_monocytogenes_1639_v14	0.9356
Listeria_monocytogenes_1639_v82	Listeria_monocytogenes_1639_v14	0.9353
Listeria_monocytogenes_1639_v81	Listeria_monocytogenes_1639_v14	0.9194
Listeria_monocytogenes_1639_v80	Listeria_monocytogenes_1639_v14	0.9569
Listeria_monocytogenes_1639_v7	Listeria_monocytogenes_1639_v14	0.9254
Listeria_monocytogenes_1639_v79	Listeria_monocytogenes_1639_v14	0.9178
Listeria_monocytogenes_1639_v78	Listeria_monocytogenes_1639_v14	0.9195
Listeria_monocytogenes_1639_v77	Listeria_monocytogenes_1639_v14	0.917
Listeria_monocytogenes_1639_v76	Listeria_monocytogenes_1639_v14	0.9197
Listeria_monocytogenes_1639_v75	Listeria_monocytogenes_1639_v14	0.8995
Listeria_monocytogenes_1639_v74	Listeria_monocytogenes_1639_v14	0.922
Listeria_monocytogenes_1639_v73	Listeria_monocytogenes_1639_v14	0.9242
Listeria_monocytogenes_1639_v72	Listeria_monocytogenes_1639_v14	0.9329
Listeria_monocytogenes_1639_v71	Listeria_monocytogenes_1639_v14	0.9069
Listeria_monocytogenes_1639_v70	Listeria_monocytogenes_1639_v14	0.9181
Listeria_monocytogenes_1639_v6	Listeria_monocytogenes_1639_v14	0.9248
Listeria_monocytogenes_1639_v69	Listeria_monocytogenes_1639_v14	0.936
Listeria_monocytogenes_1639_v68	Listeria_monocytogenes_1639_v14	0.9234
Listeria_monocytogenes_1639_v67	Listeria_monocytogenes_1639_v14	0.9352
Listeria_monocytogenes_1639_v66	Listeria_monocytogenes_1639_v14	0.9171
Listeria_monocytogenes_1639_v65	Listeria_monocytogenes_1639_v14	0.9327
Listeria_monocytogenes_1639_v64	Listeria_monocytogenes_1639_v14	0.9203
Listeria_monocytogenes_1639_v63	Listeria_monocytogenes_1639_v14	0.9481
Listeria_monocytogenes_1639_v62	Listeria_monocytogenes_1639_v14	0.9256
Listeria_monocytogenes_1639_v61	Listeria_monocytogenes_1639_v14	0.9192
Listeria_monocytogenes_1639_v60	Listeria_monocytogenes_1639_v14	0.9234
Listeria_monocytogenes_1639_v5	Listeria_monocytogenes_1639_v14	0.957
Listeria_monocytogenes_1639_v59	Listeria_monocytogenes_1639_v14	0.9282
Listeria_monocytogenes_1639_v58	Listeria_monocytogenes_1639_v14	0.9378
Listeria_monocytogenes_1639_v57	Listeria_monocytogenes_1639_v14	0.9239
Listeria_monocytogenes_1639_v56	Listeria_monocytogenes_1639_v14	0.9308
Listeria_monocytogenes_1639_v55	Listeria_monocytogenes_1639_v14	0.9347
Listeria_monocytogenes_1639_v54	Listeria_monocytogenes_1639_v14	0.9033
Listeria_monocytogenes_1639_v53	Listeria_monocytogenes_1639_v14	0.9146
Listeria_monocytogenes_1639_v52	Listeria_monocytogenes_1639_v14	0.9354
Listeria_monocytogenes_1639_v51	Listeria_monocytogenes_1639_v14	0.9337
Listeria_monocytogenes_1639_v50	Listeria_monocytogenes_1639_v14	0.9151
Listeria_monocytogenes_1639_v4	Listeria_monocytogenes_1639_v14	0.9661
Listeria_monocytogenes_1639_v49	Listeria_monocytogenes_1639_v14	0.9251
Listeria_monocytogenes_1639_v48	Listeria_monocytogenes_1639_v14	0.926
Listeria_monocytogenes_1639_v47	Listeria_monocytogenes_1639_v14	0.9155
Listeria_monocytogenes_1639_v46	Listeria_monocytogenes_1639_v14	0.9147
Listeria_monocytogenes_1639_v45	Listeria_monocytogenes_1639_v14	0.9084
Listeria_monocytogenes_1639_v44	Listeria_monocytogenes_1639_v14	0.9158
Listeria_monocytogenes_1639_v43	Listeria_monocytogenes_1639_v14	0.9158
Listeria_monocytogenes_1639_v42	Listeria_monocytogenes_1639_v14	0.9159
Listeria_monocytogenes_1639_v41	Listeria_monocytogenes_1639_v14	0.9122
Listeria_monocytogenes_1639_v40	Listeria_monocytogenes_1639_v14	0.9263
Listeria_monocytogenes_1639_v3	Listeria_monocytogenes_1639_v14	0.9664
Listeria_monocytogenes_1639_v39	Listeria_monocytogenes_1639_v14	0.933
Listeria_monocytogenes_1639_v38	Listeria_monocytogenes_1639_v14	0.9193
Listeria_monocytogenes_1639_v37	Listeria_monocytogenes_1639_v14	0.9251
Listeria_monocytogenes_1639_v36	Listeria_monocytogenes_1639_v14	0.9231
Listeria_monocytogenes_1639_v35	Listeria_monocytogenes_1639_v14	0.9259
Listeria_monocytogenes_1639_v34	Listeria_monocytogenes_1639_v14	0.9146
Listeria_monocytogenes_1639_v33	Listeria_monocytogenes_1639_v14	0.9642
Listeria_monocytogenes_1639_v32	Listeria_monocytogenes_1639_v14	0.9346
Listeria_monocytogenes_1639_v31	Listeria_monocytogenes_1639_v14	0.915
Listeria_monocytogenes_1639_v30	Listeria_monocytogenes_1639_v14	0.9403
Listeria_monocytogenes_1639_v2	Listeria_monocytogenes_1639_v14	0.9271
Listeria_monocytogenes_1639_v29	Listeria_monocytogenes_1639_v14	0.9288
Listeria_monocytogenes_1639_v28	Listeria_monocytogenes_1639_v14	0.9317
Listeria_monocytogenes_1639_v27	Listeria_monocytogenes_1639_v14	0.9299
Listeria_monocytogenes_1639_v26	Listeria_monocytogenes_1639_v14	0.931
Listeria_monocytogenes_1639_v25	Listeria_monocytogenes_1639_v14	0.9314
Listeria_monocytogenes_1639_v24	Listeria_monocytogenes_1639_v14	0.9303
Listeria_monocytogenes_1639_v23	Listeria_monocytogenes_1639_v14	0.93
Listeria_monocytogenes_1639_v22	Listeria_monocytogenes_1639_v14	0.9572
Listeria_monocytogenes_1639_v21	Listeria_monocytogenes_1639_v14	0.9296
Listeria_monocytogenes_1639_v20	Listeria_monocytogenes_1639_v14	0.9263
Listeria_monocytogenes_1639_v1	Listeria_monocytogenes_1639_v14	0.9186
Listeria_monocytogenes_1639_v19	Listeria_monocytogenes_1639_v14	0.9259
Listeria_monocytogenes_1639_v198	Listeria_monocytogenes_1639_v14	0.8858
Listeria_monocytogenes_1639_v197	Listeria_monocytogenes_1639_v14	0.9221
Listeria_monocytogenes_1639_v196	Listeria_monocytogenes_1639_v14	0.9034

Listeria_monocytogenes_1639_v195	Listeria_monocytogenes_1639_v14	0.9194
Listeria_monocytogenes_1639_v194	Listeria_monocytogenes_1639_v14	0.9195
Listeria_monocytogenes_1639_v193	Listeria_monocytogenes_1639_v14	0.9195
Listeria_monocytogenes_1639_v192	Listeria_monocytogenes_1639_v14	0.8843
Listeria_monocytogenes_1639_v191	Listeria_monocytogenes_1639_v14	0.9259
Listeria_monocytogenes_1639_v190	Listeria_monocytogenes_1639_v14	0.9274
Listeria_monocytogenes_1639_v18	Listeria_monocytogenes_1639_v14	0.9261
Listeria_monocytogenes_1639_v189	Listeria_monocytogenes_1639_v14	0.9263
Listeria_monocytogenes_1639_v188	Listeria_monocytogenes_1639_v14	0.9284
Listeria_monocytogenes_1639_v187	Listeria_monocytogenes_1639_v14	0.9271
Listeria_monocytogenes_1639_v186	Listeria_monocytogenes_1639_v14	0.9274
Listeria_monocytogenes_1639_v185	Listeria_monocytogenes_1639_v14	0.9274
Listeria_monocytogenes_1639_v184	Listeria_monocytogenes_1639_v14	0.9267
Listeria_monocytogenes_1639_v183	Listeria_monocytogenes_1639_v14	0.9214
Listeria_monocytogenes_1639_v182	Listeria_monocytogenes_1639_v14	0.9235
Listeria_monocytogenes_1639_v181	Listeria_monocytogenes_1639_v14	0.9003
Listeria_monocytogenes_1639_v180	Listeria_monocytogenes_1639_v14	0.9003
Listeria_monocytogenes_1639_v17	Listeria_monocytogenes_1639_v14	0.9261
Listeria_monocytogenes_1639_v179	Listeria_monocytogenes_1639_v14	0.9151
Listeria_monocytogenes_1639_v178	Listeria_monocytogenes_1639_v14	0.9271
Listeria_monocytogenes_1639_v177	Listeria_monocytogenes_1639_v14	0.9373
Listeria_monocytogenes_1639_v176	Listeria_monocytogenes_1639_v14	0.9151
Listeria_monocytogenes_1639_v175	Listeria_monocytogenes_1639_v14	0.921
Listeria_monocytogenes_1639_v174	Listeria_monocytogenes_1639_v14	0.9268
Listeria_monocytogenes_1639_v173	Listeria_monocytogenes_1639_v14	0.9429
Listeria_monocytogenes_1639_v172	Listeria_monocytogenes_1639_v14	0.9141
Listeria_monocytogenes_1639_v171	Listeria_monocytogenes_1639_v14	0.9286
Listeria_monocytogenes_1639_v170	Listeria_monocytogenes_1639_v14	0.9324
Listeria_monocytogenes_1639_v16	Listeria_monocytogenes_1639_v14	0.9127
Listeria_monocytogenes_1639_v169	Listeria_monocytogenes_1639_v14	0.9236
Listeria_monocytogenes_1639_v168	Listeria_monocytogenes_1639_v14	0.9205
Listeria_monocytogenes_1639_v167	Listeria_monocytogenes_1639_v14	0.9182
Listeria_monocytogenes_1639_v166	Listeria_monocytogenes_1639_v14	0.9209
Listeria_monocytogenes_1639_v165	Listeria_monocytogenes_1639_v14	0.9232
Listeria_monocytogenes_1639_v164	Listeria_monocytogenes_1639_v14	0.936
Listeria_monocytogenes_1639_v163	Listeria_monocytogenes_1639_v14	0.941
Listeria_monocytogenes_1639_v162	Listeria_monocytogenes_1639_v14	0.936
Listeria_monocytogenes_1639_v161	Listeria_monocytogenes_1639_v14	0.9361
Listeria_monocytogenes_1639_v160	Listeria_monocytogenes_1639_v14	0.9361
Listeria_monocytogenes_1639_v15	Listeria_monocytogenes_1639_v14	0.945
Listeria_monocytogenes_1639_v159	Listeria_monocytogenes_1639_v14	0.9373
Listeria_monocytogenes_1639_v158	Listeria_monocytogenes_1639_v14	0.9368
Listeria_monocytogenes_1639_v157	Listeria_monocytogenes_1639_v14	0.9368
Listeria_monocytogenes_1639_v156	Listeria_monocytogenes_1639_v14	0.9251
Listeria_monocytogenes_1639_v155	Listeria_monocytogenes_1639_v14	0.9256
Listeria_monocytogenes_1639_v154	Listeria_monocytogenes_1639_v14	0.9249
Listeria_monocytogenes_1639_v153	Listeria_monocytogenes_1639_v14	0.9326
Listeria_monocytogenes_1639_v152	Listeria_monocytogenes_1639_v14	0.9377
Listeria_monocytogenes_1639_v151	Listeria_monocytogenes_1639_v14	0.9377
Listeria_monocytogenes_1639_v150	Listeria_monocytogenes_1639_v14	0.92
Listeria_monocytogenes_1639_v14	Listeria_monocytogenes_1639_v14	0.0757
Listeria_welshimeri_1643	Listeria_monocytogenes_1639_v150	0.8777
Listeria_welshimeri_serovar_6b_str_SLCC5334_386043	Listeria_monocytogenes_1639_v150	0.8774
Listeria_seeligeri_serovar_1_2b_str_SLCC3954_683837	Listeria_monocytogenes_1639_v150	0.8856
Listeria_seeligeri_FSL_S4-171_702452	Listeria_monocytogenes_1639_v150	0.8193
Listeria_seeligeri_FSL_N1-067_702453	Listeria_monocytogenes_1639_v150	0.8327
Listeria_seeligeri_1640_v1	Listeria_monocytogenes_1639_v150	0.8837
Listeria_seeligeri_1640	Listeria_monocytogenes_1639_v150	0.8765
Listeria_monocytogenes_WSLC1042_1457188	Listeria_monocytogenes_1639_v150	0.931
Listeria_monocytogenes_WSLC1001_1457187	Listeria_monocytogenes_1639_v150	0.9879
Listeria_monocytogenes_SLCC7179_879090	Listeria_monocytogenes_1639_v150	0.9605
Listeria_monocytogenes_SLCC5850_882096	Listeria_monocytogenes_1639_v150	0.9882
Listeria_monocytogenes_SLCC2755_932919	Listeria_monocytogenes_1639_v150	0.9361
Listeria_monocytogenes_SLCC2540_879089	Listeria_monocytogenes_1639_v150	0.948
Listeria_monocytogenes_SLCC2479_882020	Listeria_monocytogenes_1639_v150	0.9593
Listeria_monocytogenes_SLCC2378_879088	Listeria_monocytogenes_1639_v150	0.9321
Listeria_monocytogenes_SLCC2376_882097	Listeria_monocytogenes_1639_v150	0.9452
Listeria_monocytogenes_SLCC2372_932920	Listeria_monocytogenes_1639_v150	0.9521
Listeria_monocytogenes_serotype_4b_str_LL195_1230340	Listeria_monocytogenes_1639_v150	0.9381
Listeria_monocytogenes_serotype_4b_str_LIS0087_1134669	Listeria_monocytogenes_1639_v150	0.9727
Listeria_monocytogenes_serotype_4b_str_F2365_265669	Listeria_monocytogenes_1639_v150	0.9381
Listeria_monocytogenes_serotype_4b_str_CLIP_80459_568819	Listeria_monocytogenes_1639_v150	0.945
Listeria_monocytogenes_serotype_4b_str_81-0861_1196161	Listeria_monocytogenes_1639_v150	0.923
Listeria_monocytogenes_serotype_4b_str_81-0592_1196160	Listeria_monocytogenes_1639_v150	0.9241
Listeria_monocytogenes_serotype_4b_str_81-0558_1196159	Listeria_monocytogenes_1639_v150	0.9244
Listeria_monocytogenes_serotype_4b_str_10-0809_1196162	Listeria_monocytogenes_1639_v150	0.9241
Listeria_monocytogenes_serotype_4b_str_02-6680_1196175	Listeria_monocytogenes_1639_v150	0.9436
Listeria_monocytogenes_serotype_4b_str_02-6679_1196174	Listeria_monocytogenes_1639_v150	0.9436
Listeria_monocytogenes_serotype_4b_str_02-1792_1196173	Listeria_monocytogenes_1639_v150	0.9241
Listeria_monocytogenes_serotype_4b_str_02-1289_1196172	Listeria_monocytogenes_1639_v150	0.9241
Listeria_monocytogenes_serotype_4b_str_02-1103_1196171	Listeria_monocytogenes_1639_v150	0.9241
Listeria_monocytogenes_serotype_3c_str_10-5027_1196195	Listeria_monocytogenes_1639_v150	0.9559
Listeria_monocytogenes_serotype_1_2c_str_10-5026_1196194	Listeria_monocytogenes_1639_v150	0.957
Listeria_monocytogenes_serotype_1_2c_str_10-5025_1196193	Listeria_monocytogenes_1639_v150	0.957
Listeria_monocytogenes_serotype_1_2b_str_10-0811_1196164	Listeria_monocytogenes_1639_v150	0.9384
Listeria_monocytogenes_serotype_1_2b_str_10-0810_1196163	Listeria_monocytogenes_1639_v150	0.9384
Listeria_monocytogenes_serotype_1_2a_str_99-6370_1196179	Listeria_monocytogenes_1639_v150	0.9468
Listeria_monocytogenes_serotype_1_2a_str_98-2035_1196178	Listeria_monocytogenes_1639_v150	0.9468
Listeria_monocytogenes_serotype_1_2a_str_95-0093_1196177	Listeria_monocytogenes_1639_v150	0.9521
Listeria_monocytogenes_serotype_1_2a_str_88-0478_1196176	Listeria_monocytogenes_1639_v150	0.9513
Listeria_monocytogenes_serotype_1_2a_str_10-5024_1196192	Listeria_monocytogenes_1639_v150	0.9524

Listeria_monocytogenes_serotype_1_2a_str_10-4758_1196168	Listeria_monocytogenes_1639_v150	0.9692
Listeria_monocytogenes_serotype_1_2a_str_10-4754_1196167	Listeria_monocytogenes_1639_v150	0.9695
Listeria_monocytogenes_serotype_1_2a_str_10-1321_1196191	Listeria_monocytogenes_1639_v150	0.9524
Listeria_monocytogenes_serotype_1_2a_str_10-1047_1196190	Listeria_monocytogenes_1639_v150	0.9528
Listeria_monocytogenes_serotype_1_2a_str_10-1046_1196189	Listeria_monocytogenes_1639_v150	0.9528
Listeria_monocytogenes_serotype_1_2a_str_10-0934_1196170	Listeria_monocytogenes_1639_v150	0.9757
Listeria_monocytogenes_serotype_1_2a_str_10-0933_1196169	Listeria_monocytogenes_1639_v150	0.9757
Listeria_monocytogenes_serotype_1_2a_str_10-0815_1196188	Listeria_monocytogenes_1639_v150	0.9482
Listeria_monocytogenes_serotype_1_2a_str_10-0814_1196187	Listeria_monocytogenes_1639_v150	0.9524
Listeria_monocytogenes_serotype_1_2a_str_10-0813_1196166	Listeria_monocytogenes_1639_v150	0.9796
Listeria_monocytogenes_serotype_1_2a_str_10-0812_1196165	Listeria_monocytogenes_1639_v150	0.9796
Listeria_monocytogenes_serotype_1_2a_str_08-7669_1196186	Listeria_monocytogenes_1639_v150	0.9519
Listeria_monocytogenes_serotype_1_2a_str_08-7374_1196185	Listeria_monocytogenes_1639_v150	0.9524
Listeria_monocytogenes_serotype_1_2a_str_08-6997_1196184	Listeria_monocytogenes_1639_v150	0.9482
Listeria_monocytogenes_serotype_1_2a_str_08-6569_1196183	Listeria_monocytogenes_1639_v150	0.9482
Listeria_monocytogenes_serotype_1_2a_str_08-6056_1196182	Listeria_monocytogenes_1639_v150	0.9482
Listeria_monocytogenes_serotype_1_2a_str_04-5457_1196181	Listeria_monocytogenes_1639_v150	0.9524
Listeria_monocytogenes_serotype_1_2a_str_02-5993_1196180	Listeria_monocytogenes_1639_v150	0.9524
Listeria_monocytogenes_R479a_1437838	Listeria_monocytogenes_1639_v150	0.951
Listeria_monocytogenes_N53-1_1234142	Listeria_monocytogenes_1639_v150	0.9092
Listeria_monocytogenes_M7_1030009	Listeria_monocytogenes_1639_v150	0.9117
Listeria_monocytogenes_LO28_393132	Listeria_monocytogenes_1639_v150	0.9582
Listeria_monocytogenes_L99_563174	Listeria_monocytogenes_1639_v150	0.9121
Listeria_monocytogenes_L312_882094	Listeria_monocytogenes_1639_v150	0.945
Listeria_monocytogenes_J1816_930781	Listeria_monocytogenes_1639_v150	0.9255
Listeria_monocytogenes_J1-220_930782	Listeria_monocytogenes_1639_v150	0.9258
Listeria_monocytogenes_J0161_393130	Listeria_monocytogenes_1639_v150	0.9469
Listeria_monocytogenes_HCC23_552536	Listeria_monocytogenes_1639_v150	0.9117
Listeria_monocytogenes_FSL_R2-561_393126	Listeria_monocytogenes_1639_v150	0.9548
Listeria_monocytogenes_FSL_J1-208_393119	Listeria_monocytogenes_1639_v150	0.9103
Listeria_monocytogenes_FSL_J1-175_393118	Listeria_monocytogenes_1639_v150	0.9516
Listeria_monocytogenes_Finland_1998_393127	Listeria_monocytogenes_1639_v150	0.9633
Listeria_monocytogenes_EGD-e_169963_v1	Listeria_monocytogenes_1639_v150	0.9547
Listeria_monocytogenes_EGD-e_169963	Listeria_monocytogenes_1639_v150	0.9547
Listeria_monocytogenes_EGD_1334565	Listeria_monocytogenes_1639_v150	0.9889
Listeria_monocytogenes_CFSAN002349_1299895	Listeria_monocytogenes_1639_v150	0.9436
Listeria_monocytogenes_ATCC_19117_882095_v1	Listeria_monocytogenes_1639_v150	0.9387
Listeria_monocytogenes_ATCC_19117_882095	Listeria_monocytogenes_1639_v150	0.9384
Listeria_monocytogenes_6179_1288295	Listeria_monocytogenes_1639_v150	0.9421
Listeria_monocytogenes_1639_v9	Listeria_monocytogenes_1639_v150	0.9245
Listeria_monocytogenes_1639_v99	Listeria_monocytogenes_1639_v150	0.9274
Listeria_monocytogenes_1639_v98	Listeria_monocytogenes_1639_v150	0.9469
Listeria_monocytogenes_1639_v97	Listeria_monocytogenes_1639_v150	0.9329
Listeria_monocytogenes_1639_v96	Listeria_monocytogenes_1639_v150	0.9252
Listeria_monocytogenes_1639_v95	Listeria_monocytogenes_1639_v150	0.9252
Listeria_monocytogenes_1639_v94	Listeria_monocytogenes_1639_v150	0.9259
Listeria_monocytogenes_1639_v93	Listeria_monocytogenes_1639_v150	0.9513
Listeria_monocytogenes_1639_v92	Listeria_monocytogenes_1639_v150	0.9267
Listeria_monocytogenes_1639_v91	Listeria_monocytogenes_1639_v150	0.9269
Listeria_monocytogenes_1639_v90	Listeria_monocytogenes_1639_v150	0.9469
Listeria_monocytogenes_1639_v8	Listeria_monocytogenes_1639_v150	0.9397
Listeria_monocytogenes_1639_v89	Listeria_monocytogenes_1639_v150	0.9383
Listeria_monocytogenes_1639_v88	Listeria_monocytogenes_1639_v150	0.9709
Listeria_monocytogenes_1639_v87	Listeria_monocytogenes_1639_v150	0.9767
Listeria_monocytogenes_1639_v86	Listeria_monocytogenes_1639_v150	0.9767
Listeria_monocytogenes_1639_v85	Listeria_monocytogenes_1639_v150	0.9219
Listeria_monocytogenes_1639_v84	Listeria_monocytogenes_1639_v150	0.9648
Listeria_monocytogenes_1639_v83	Listeria_monocytogenes_1639_v150	0.9292
Listeria_monocytogenes_1639_v82	Listeria_monocytogenes_1639_v150	0.9289
Listeria_monocytogenes_1639_v81	Listeria_monocytogenes_1639_v150	0.9558
Listeria_monocytogenes_1639_v80	Listeria_monocytogenes_1639_v150	0.9443
Listeria_monocytogenes_1639_v7	Listeria_monocytogenes_1639_v150	0.9397
Listeria_monocytogenes_1639_v79	Listeria_monocytogenes_1639_v150	0.9566
Listeria_monocytogenes_1639_v78	Listeria_monocytogenes_1639_v150	0.9547
Listeria_monocytogenes_1639_v77	Listeria_monocytogenes_1639_v150	0.9492
Listeria_monocytogenes_1639_v76	Listeria_monocytogenes_1639_v150	0.9535
Listeria_monocytogenes_1639_v75	Listeria_monocytogenes_1639_v150	0.9121
Listeria_monocytogenes_1639_v74	Listeria_monocytogenes_1639_v150	0.9631
Listeria_monocytogenes_1639_v73	Listeria_monocytogenes_1639_v150	0.9506
Listeria_monocytogenes_1639_v72	Listeria_monocytogenes_1639_v150	0.9273
Listeria_monocytogenes_1639_v71	Listeria_monocytogenes_1639_v150	0.9447
Listeria_monocytogenes_1639_v70	Listeria_monocytogenes_1639_v150	0.9531
Listeria_monocytogenes_1639_v6	Listeria_monocytogenes_1639_v150	0.9393
Listeria_monocytogenes_1639_v69	Listeria_monocytogenes_1639_v150	0.9241
Listeria_monocytogenes_1639_v68	Listeria_monocytogenes_1639_v150	0.9474
Listeria_monocytogenes_1639_v67	Listeria_monocytogenes_1639_v150	0.9456
Listeria_monocytogenes_1639_v66	Listeria_monocytogenes_1639_v150	0.9646
Listeria_monocytogenes_1639_v65	Listeria_monocytogenes_1639_v150	0.9527
Listeria_monocytogenes_1639_v64	Listeria_monocytogenes_1639_v150	0.9577
Listeria_monocytogenes_1639_v63	Listeria_monocytogenes_1639_v150	0.9402
Listeria_monocytogenes_1639_v62	Listeria_monocytogenes_1639_v150	0.9503
Listeria_monocytogenes_1639_v61	Listeria_monocytogenes_1639_v150	0.9538
Listeria_monocytogenes_1639_v60	Listeria_monocytogenes_1639_v150	0.9486
Listeria_monocytogenes_1639_v5	Listeria_monocytogenes_1639_v150	0.8963
Listeria_monocytogenes_1639_v59	Listeria_monocytogenes_1639_v150	0.9353
Listeria_monocytogenes_1639_v58	Listeria_monocytogenes_1639_v150	0.9288
Listeria_monocytogenes_1639_v57	Listeria_monocytogenes_1639_v150	0.9233
Listeria_monocytogenes_1639_v56	Listeria_monocytogenes_1639_v150	0.9296
Listeria_monocytogenes_1639_v55	Listeria_monocytogenes_1639_v150	0.9198
Listeria_monocytogenes_1639_v54	Listeria_monocytogenes_1639_v150	0.9245
Listeria_monocytogenes_1639_v53	Listeria_monocytogenes_1639_v150	0.9467

Listeria_monocytogenes_1639_v52	Listeria_monocytogenes_1639_v150	0.9321
Listeria_monocytogenes_1639_v51	Listeria_monocytogenes_1639_v150	0.9244
Listeria_monocytogenes_1639_v50	Listeria_monocytogenes_1639_v150	0.9509
Listeria_monocytogenes_1639_v4	Listeria_monocytogenes_1639_v150	0.9094
Listeria_monocytogenes_1639_v49	Listeria_monocytogenes_1639_v150	0.926
Listeria_monocytogenes_1639_v48	Listeria_monocytogenes_1639_v150	0.9509
Listeria_monocytogenes_1639_v47	Listeria_monocytogenes_1639_v150	0.9532
Listeria_monocytogenes_1639_v46	Listeria_monocytogenes_1639_v150	0.9482
Listeria_monocytogenes_1639_v45	Listeria_monocytogenes_1639_v150	0.9429
Listeria_monocytogenes_1639_v44	Listeria_monocytogenes_1639_v150	0.9524
Listeria_monocytogenes_1639_v43	Listeria_monocytogenes_1639_v150	0.9524
Listeria_monocytogenes_1639_v42	Listeria_monocytogenes_1639_v150	0.9378
Listeria_monocytogenes_1639_v41	Listeria_monocytogenes_1639_v150	0.9338
Listeria_monocytogenes_1639_v40	Listeria_monocytogenes_1639_v150	0.9559
Listeria_monocytogenes_1639_v3	Listeria_monocytogenes_1639_v150	0.9098
Listeria_monocytogenes_1639_v39	Listeria_monocytogenes_1639_v150	0.9413
Listeria_monocytogenes_1639_v38	Listeria_monocytogenes_1639_v150	0.9564
Listeria_monocytogenes_1639_v37	Listeria_monocytogenes_1639_v150	0.9477
Listeria_monocytogenes_1639_v36	Listeria_monocytogenes_1639_v150	0.9545
Listeria_monocytogenes_1639_v35	Listeria_monocytogenes_1639_v150	0.9597
Listeria_monocytogenes_1639_v34	Listeria_monocytogenes_1639_v150	0.9509
Listeria_monocytogenes_1639_v33	Listeria_monocytogenes_1639_v150	0.9384
Listeria_monocytogenes_1639_v32	Listeria_monocytogenes_1639_v150	0.9478
Listeria_monocytogenes_1639_v31	Listeria_monocytogenes_1639_v150	0.9513
Listeria_monocytogenes_1639_v30	Listeria_monocytogenes_1639_v150	0.9323
Listeria_monocytogenes_1639_v2	Listeria_monocytogenes_1639_v150	0.8999
Listeria_monocytogenes_1639_v29	Listeria_monocytogenes_1639_v150	0.9432
Listeria_monocytogenes_1639_v28	Listeria_monocytogenes_1639_v150	0.9474
Listeria_monocytogenes_1639_v27	Listeria_monocytogenes_1639_v150	0.9383
Listeria_monocytogenes_1639_v26	Listeria_monocytogenes_1639_v150	0.9376
Listeria_monocytogenes_1639_v25	Listeria_monocytogenes_1639_v150	0.9376
Listeria_monocytogenes_1639_v24	Listeria_monocytogenes_1639_v150	0.9273
Listeria_monocytogenes_1639_v23	Listeria_monocytogenes_1639_v150	0.9269
Listeria_monocytogenes_1639_v22	Listeria_monocytogenes_1639_v150	0.9398
Listeria_monocytogenes_1639_v21	Listeria_monocytogenes_1639_v150	0.9269
Listeria_monocytogenes_1639_v20	Listeria_monocytogenes_1639_v150	0.9373
Listeria_monocytogenes_1639_v1	Listeria_monocytogenes_1639_v150	0.937
Listeria_monocytogenes_1639_v19	Listeria_monocytogenes_1639_v150	0.9369
Listeria_monocytogenes_1639_v198	Listeria_monocytogenes_1639_v150	0.9109
Listeria_monocytogenes_1639_v197	Listeria_monocytogenes_1639_v150	0.941
Listeria_monocytogenes_1639_v196	Listeria_monocytogenes_1639_v150	0.9728
Listeria_monocytogenes_1639_v195	Listeria_monocytogenes_1639_v150	0.9475
Listeria_monocytogenes_1639_v194	Listeria_monocytogenes_1639_v150	0.9452
Listeria_monocytogenes_1639_v193	Listeria_monocytogenes_1639_v150	0.9456
Listeria_monocytogenes_1639_v192	Listeria_monocytogenes_1639_v150	0.9037
Listeria_monocytogenes_1639_v191	Listeria_monocytogenes_1639_v150	0.971
Listeria_monocytogenes_1639_v190	Listeria_monocytogenes_1639_v150	0.9872
Listeria_monocytogenes_1639_v18	Listeria_monocytogenes_1639_v150	0.9178
Listeria_monocytogenes_1639_v189	Listeria_monocytogenes_1639_v150	0.9869
Listeria_monocytogenes_1639_v188	Listeria_monocytogenes_1639_v150	0.9667
Listeria_monocytogenes_1639_v187	Listeria_monocytogenes_1639_v150	0.9876
Listeria_monocytogenes_1639_v186	Listeria_monocytogenes_1639_v150	0.9876
Listeria_monocytogenes_1639_v185	Listeria_monocytogenes_1639_v150	0.9876
Listeria_monocytogenes_1639_v184	Listeria_monocytogenes_1639_v150	0.9872
Listeria_monocytogenes_1639_v183	Listeria_monocytogenes_1639_v150	0.9633
Listeria_monocytogenes_1639_v182	Listeria_monocytogenes_1639_v150	0.9655
Listeria_monocytogenes_1639_v181	Listeria_monocytogenes_1639_v150	0.9208
Listeria_monocytogenes_1639_v180	Listeria_monocytogenes_1639_v150	0.9208
Listeria_monocytogenes_1639_v17	Listeria_monocytogenes_1639_v150	0.9182
Listeria_monocytogenes_1639_v179	Listeria_monocytogenes_1639_v150	0.9568
Listeria_monocytogenes_1639_v178	Listeria_monocytogenes_1639_v150	0.9687
Listeria_monocytogenes_1639_v177	Listeria_monocytogenes_1639_v150	0.9428
Listeria_monocytogenes_1639_v176	Listeria_monocytogenes_1639_v150	0.936
Listeria_monocytogenes_1639_v175	Listeria_monocytogenes_1639_v150	0.9736
Listeria_monocytogenes_1639_v174	Listeria_monocytogenes_1639_v150	0.9718
Listeria_monocytogenes_1639_v173	Listeria_monocytogenes_1639_v150	0.9521
Listeria_monocytogenes_1639_v172	Listeria_monocytogenes_1639_v150	0.9366
Listeria_monocytogenes_1639_v171	Listeria_monocytogenes_1639_v150	0.9745
Listeria_monocytogenes_1639_v170	Listeria_monocytogenes_1639_v150	0.9831
Listeria_monocytogenes_1639_v16	Listeria_monocytogenes_1639_v150	0.9384
Listeria_monocytogenes_1639_v169	Listeria_monocytogenes_1639_v150	0.9747
Listeria_monocytogenes_1639_v168	Listeria_monocytogenes_1639_v150	0.9626
Listeria_monocytogenes_1639_v167	Listeria_monocytogenes_1639_v150	0.9582
Listeria_monocytogenes_1639_v166	Listeria_monocytogenes_1639_v150	0.963
Listeria_monocytogenes_1639_v165	Listeria_monocytogenes_1639_v150	0.9743
Listeria_monocytogenes_1639_v164	Listeria_monocytogenes_1639_v150	0.9377
Listeria_monocytogenes_1639_v163	Listeria_monocytogenes_1639_v150	0.9474
Listeria_monocytogenes_1639_v162	Listeria_monocytogenes_1639_v150	0.9377
Listeria_monocytogenes_1639_v161	Listeria_monocytogenes_1639_v150	0.9388
Listeria_monocytogenes_1639_v160	Listeria_monocytogenes_1639_v150	0.9385
Listeria_monocytogenes_1639_v15	Listeria_monocytogenes_1639_v150	0.9239
Listeria_monocytogenes_1639_v159	Listeria_monocytogenes_1639_v150	0.9432
Listeria_monocytogenes_1639_v158	Listeria_monocytogenes_1639_v150	0.9381
Listeria_monocytogenes_1639_v157	Listeria_monocytogenes_1639_v150	0.9381
Listeria_monocytogenes_1639_v156	Listeria_monocytogenes_1639_v150	0.9657
Listeria_monocytogenes_1639_v155	Listeria_monocytogenes_1639_v150	0.9576
Listeria_monocytogenes_1639_v154	Listeria_monocytogenes_1639_v150	0.9694
Listeria_monocytogenes_1639_v153	Listeria_monocytogenes_1639_v150	0.9556
Listeria_monocytogenes_1639_v152	Listeria_monocytogenes_1639_v150	0.9432
Listeria_monocytogenes_1639_v151	Listeria_monocytogenes_1639_v150	0.9432
Listeria_monocytogenes_1639_v150	Listeria_monocytogenes_1639_v150	0.0662

Listeria_welshimeri_1643	Listeria_monocytogenes_1639_v151	0.8819
Listeria_welshimeri_serovar_6b_str_SLCC5334_386043	Listeria_monocytogenes_1639_v151	0.8813
Listeria_seeligeri_serovar_1_2b_str_SLCC3954_683837	Listeria_monocytogenes_1639_v151	0.89
Listeria_seeligeri_FSL_S4-171_702452	Listeria_monocytogenes_1639_v151	0.8306
Listeria_seeligeri_FSL_N1-067_702453	Listeria_monocytogenes_1639_v151	0.8345
Listeria_seeligeri_1640_v1	Listeria_monocytogenes_1639_v151	0.8976
Listeria_seeligeri_1640	Listeria_monocytogenes_1639_v151	0.8902
Listeria_monocytogenes_WSLC1042_1457188	Listeria_monocytogenes_1639_v151	0.9683
Listeria_monocytogenes_WSLC1001_1457187	Listeria_monocytogenes_1639_v151	0.9368
Listeria_monocytogenes_SLCC7179_879090	Listeria_monocytogenes_1639_v151	0.942
Listeria_monocytogenes_SLCC5850_882096	Listeria_monocytogenes_1639_v151	0.9415
Listeria_monocytogenes_SLCC2755_932919	Listeria_monocytogenes_1639_v151	0.9511
Listeria_monocytogenes_SLCC2540_879089	Listeria_monocytogenes_1639_v151	0.9505
Listeria_monocytogenes_SLCC2479_882020	Listeria_monocytogenes_1639_v151	0.9327
Listeria_monocytogenes_SLCC2378_879088	Listeria_monocytogenes_1639_v151	0.9698
Listeria_monocytogenes_SLCC2376_882097	Listeria_monocytogenes_1639_v151	0.9478
Listeria_monocytogenes_SLCC2372_932920	Listeria_monocytogenes_1639_v151	0.9254
Listeria_monocytogenes_serotype_4b_str_LL195_1230340	Listeria_monocytogenes_1639_v151	0.9757
Listeria_monocytogenes_serotype_4b_str_LIS0087_1134669	Listeria_monocytogenes_1639_v151	0.9182
Listeria_monocytogenes_serotype_4b_str_F2365_265669	Listeria_monocytogenes_1639_v151	0.9757
Listeria_monocytogenes_serotype_4b_str_CLIP_80459_568819	Listeria_monocytogenes_1639_v151	0.9776
Listeria_monocytogenes_serotype_4b_str_81-0861_1196161	Listeria_monocytogenes_1639_v151	0.958
Listeria_monocytogenes_serotype_4b_str_81-0592_1196160	Listeria_monocytogenes_1639_v151	0.9591
Listeria_monocytogenes_serotype_4b_str_81-0558_1196159	Listeria_monocytogenes_1639_v151	0.9591
Listeria_monocytogenes_serotype_4b_str_10-0809_1196162	Listeria_monocytogenes_1639_v151	0.9595
Listeria_monocytogenes_serotype_4b_str_02-6680_1196175	Listeria_monocytogenes_1639_v151	0.9746
Listeria_monocytogenes_serotype_4b_str_02-6679_1196174	Listeria_monocytogenes_1639_v151	0.9746
Listeria_monocytogenes_serotype_4b_str_02-1792_1196173	Listeria_monocytogenes_1639_v151	0.9598
Listeria_monocytogenes_serotype_4b_str_02-1289_1196172	Listeria_monocytogenes_1639_v151	0.9598
Listeria_monocytogenes_serotype_4b_str_02-1103_1196171	Listeria_monocytogenes_1639_v151	0.9598
Listeria_monocytogenes_serotype_3c_str_10-5027_1196195	Listeria_monocytogenes_1639_v151	0.9319
Listeria_monocytogenes_serotype_1_2c_str_10-5026_1196194	Listeria_monocytogenes_1639_v151	0.9331
Listeria_monocytogenes_serotype_1_2c_str_10-5025_1196193	Listeria_monocytogenes_1639_v151	0.9331
Listeria_monocytogenes_serotype_1_2b_str_10-0811_1196164	Listeria_monocytogenes_1639_v151	0.9447
Listeria_monocytogenes_serotype_1_2b_str_10-0810_1196163	Listeria_monocytogenes_1639_v151	0.9447
Listeria_monocytogenes_serotype_1_2a_str_99-6370_1196179	Listeria_monocytogenes_1639_v151	0.9205
Listeria_monocytogenes_serotype_1_2a_str_98-2035_1196178	Listeria_monocytogenes_1639_v151	0.9205
Listeria_monocytogenes_serotype_1_2a_str_95-0093_1196177	Listeria_monocytogenes_1639_v151	0.926
Listeria_monocytogenes_serotype_1_2a_str_88-0478_1196176	Listeria_monocytogenes_1639_v151	0.9252
Listeria_monocytogenes_serotype_1_2a_str_10-5024_1196192	Listeria_monocytogenes_1639_v151	0.9264
Listeria_monocytogenes_serotype_1_2a_str_10-4758_1196168	Listeria_monocytogenes_1639_v151	0.9423
Listeria_monocytogenes_serotype_1_2a_str_10-4754_1196167	Listeria_monocytogenes_1639_v151	0.9419
Listeria_monocytogenes_serotype_1_2a_str_10-1321_1196191	Listeria_monocytogenes_1639_v151	0.9264
Listeria_monocytogenes_serotype_1_2a_str_10-1047_1196190	Listeria_monocytogenes_1639_v151	0.9267
Listeria_monocytogenes_serotype_1_2a_str_10-1046_1196189	Listeria_monocytogenes_1639_v151	0.9267
Listeria_monocytogenes_serotype_1_2a_str_10-0934_1196170	Listeria_monocytogenes_1639_v151	0.9516
Listeria_monocytogenes_serotype_1_2a_str_10-0933_1196169	Listeria_monocytogenes_1639_v151	0.9516
Listeria_monocytogenes_serotype_1_2a_str_10-0815_1196188	Listeria_monocytogenes_1639_v151	0.9219
Listeria_monocytogenes_serotype_1_2a_str_10-0814_1196187	Listeria_monocytogenes_1639_v151	0.9264
Listeria_monocytogenes_serotype_1_2a_str_10-0813_1196166	Listeria_monocytogenes_1639_v151	0.934
Listeria_monocytogenes_serotype_1_2a_str_10-0812_1196165	Listeria_monocytogenes_1639_v151	0.934
Listeria_monocytogenes_serotype_1_2a_str_08-7669_1196186	Listeria_monocytogenes_1639_v151	0.9355
Listeria_monocytogenes_serotype_1_2a_str_08-7374_1196185	Listeria_monocytogenes_1639_v151	0.9264
Listeria_monocytogenes_serotype_1_2a_str_08-6997_1196184	Listeria_monocytogenes_1639_v151	0.9219
Listeria_monocytogenes_serotype_1_2a_str_08-6569_1196183	Listeria_monocytogenes_1639_v151	0.9219
Listeria_monocytogenes_serotype_1_2a_str_08-6056_1196182	Listeria_monocytogenes_1639_v151	0.9219
Listeria_monocytogenes_serotype_1_2a_str_04-5457_1196181	Listeria_monocytogenes_1639_v151	0.9264
Listeria_monocytogenes_serotype_1_2a_str_02-5993_1196180	Listeria_monocytogenes_1639_v151	0.926
Listeria_monocytogenes_R479a_1437838	Listeria_monocytogenes_1639_v151	0.9253
Listeria_monocytogenes_N53-1_1234142	Listeria_monocytogenes_1639_v151	0.8968
Listeria_monocytogenes_M7_1030009	Listeria_monocytogenes_1639_v151	0.9111
Listeria_monocytogenes_LO28_393132	Listeria_monocytogenes_1639_v151	0.9313
Listeria_monocytogenes_L99_563174	Listeria_monocytogenes_1639_v151	0.9118
Listeria_monocytogenes_L312_882094	Listeria_monocytogenes_1639_v151	0.978
Listeria_monocytogenes_J1816_930781	Listeria_monocytogenes_1639_v151	0.9777
Listeria_monocytogenes_J1-220_930782	Listeria_monocytogenes_1639_v151	0.9504
Listeria_monocytogenes_J0161_393130	Listeria_monocytogenes_1639_v151	0.9305
Listeria_monocytogenes_HCC23_552536	Listeria_monocytogenes_1639_v151	0.9111
Listeria_monocytogenes_FSL_R2-561_393126	Listeria_monocytogenes_1639_v151	0.9313
Listeria_monocytogenes_FSL_J1-208_393119	Listeria_monocytogenes_1639_v151	0.9196
Listeria_monocytogenes_FSL_J1-175_393118	Listeria_monocytogenes_1639_v151	0.9568
Listeria_monocytogenes_Finland_1998_393127	Listeria_monocytogenes_1639_v151	0.9528
Listeria_monocytogenes_EGD-e_169963_v1	Listeria_monocytogenes_1639_v151	0.9405
Listeria_monocytogenes_EGD-e_169963	Listeria_monocytogenes_1639_v151	0.9409
Listeria_monocytogenes_EGD_1334565	Listeria_monocytogenes_1639_v151	0.9422
Listeria_monocytogenes_CFSAN002349_1299895	Listeria_monocytogenes_1639_v151	0.9827
Listeria_monocytogenes_ATCC_19117_882095_v1	Listeria_monocytogenes_1639_v151	0.9708
Listeria_monocytogenes_ATCC_19117_882095	Listeria_monocytogenes_1639_v151	0.9708
Listeria_monocytogenes_6179_1288295	Listeria_monocytogenes_1639_v151	0.9151
Listeria_monocytogenes_1639_v9	Listeria_monocytogenes_1639_v151	0.9272
Listeria_monocytogenes_1639_v99	Listeria_monocytogenes_1639_v151	0.98
Listeria_monocytogenes_1639_v98	Listeria_monocytogenes_1639_v151	0.9293
Listeria_monocytogenes_1639_v97	Listeria_monocytogenes_1639_v151	0.9673
Listeria_monocytogenes_1639_v96	Listeria_monocytogenes_1639_v151	0.9789
Listeria_monocytogenes_1639_v95	Listeria_monocytogenes_1639_v151	0.9789
Listeria_monocytogenes_1639_v94	Listeria_monocytogenes_1639_v151	0.9793
Listeria_monocytogenes_1639_v93	Listeria_monocytogenes_1639_v151	0.9278
Listeria_monocytogenes_1639_v92	Listeria_monocytogenes_1639_v151	0.9796
Listeria_monocytogenes_1639_v91	Listeria_monocytogenes_1639_v151	0.9558
Listeria_monocytogenes_1639_v90	Listeria_monocytogenes_1639_v151	0.9293
Listeria_monocytogenes_1639_v8	Listeria_monocytogenes_1639_v151	0.9154

Listeria_monocytogenes_1639_v89	Listeria_monocytogenes_1639_v151	0.9672
Listeria_monocytogenes_1639_v88	Listeria_monocytogenes_1639_v151	0.9325
Listeria_monocytogenes_1639_v87	Listeria_monocytogenes_1639_v151	0.9301
Listeria_monocytogenes_1639_v86	Listeria_monocytogenes_1639_v151	0.9294
Listeria_monocytogenes_1639_v85	Listeria_monocytogenes_1639_v151	0.9573
Listeria_monocytogenes_1639_v84	Listeria_monocytogenes_1639_v151	0.9361
Listeria_monocytogenes_1639_v83	Listeria_monocytogenes_1639_v151	0.9594
Listeria_monocytogenes_1639_v82	Listeria_monocytogenes_1639_v151	0.9587
Listeria_monocytogenes_1639_v81	Listeria_monocytogenes_1639_v151	0.9333
Listeria_monocytogenes_1639_v80	Listeria_monocytogenes_1639_v151	0.951
Listeria_monocytogenes_1639_v7	Listeria_monocytogenes_1639_v151	0.9154
Listeria_monocytogenes_1639_v79	Listeria_monocytogenes_1639_v151	0.9311
Listeria_monocytogenes_1639_v78	Listeria_monocytogenes_1639_v151	0.9326
Listeria_monocytogenes_1639_v77	Listeria_monocytogenes_1639_v151	0.9327
Listeria_monocytogenes_1639_v76	Listeria_monocytogenes_1639_v151	0.9259
Listeria_monocytogenes_1639_v75	Listeria_monocytogenes_1639_v151	0.9118
Listeria_monocytogenes_1639_v74	Listeria_monocytogenes_1639_v151	0.9283
Listeria_monocytogenes_1639_v73	Listeria_monocytogenes_1639_v151	0.9264
Listeria_monocytogenes_1639_v72	Listeria_monocytogenes_1639_v151	0.9582
Listeria_monocytogenes_1639_v71	Listeria_monocytogenes_1639_v151	0.9168
Listeria_monocytogenes_1639_v70	Listeria_monocytogenes_1639_v151	0.9292
Listeria_monocytogenes_1639_v6	Listeria_monocytogenes_1639_v151	0.9147
Listeria_monocytogenes_1639_v69	Listeria_monocytogenes_1639_v151	0.9595
Listeria_monocytogenes_1639_v68	Listeria_monocytogenes_1639_v151	0.9355
Listeria_monocytogenes_1639_v67	Listeria_monocytogenes_1639_v151	0.9465
Listeria_monocytogenes_1639_v66	Listeria_monocytogenes_1639_v151	0.937
Listeria_monocytogenes_1639_v65	Listeria_monocytogenes_1639_v151	0.9295
Listeria_monocytogenes_1639_v64	Listeria_monocytogenes_1639_v151	0.9284
Listeria_monocytogenes_1639_v63	Listeria_monocytogenes_1639_v151	0.9453
Listeria_monocytogenes_1639_v62	Listeria_monocytogenes_1639_v151	0.9287
Listeria_monocytogenes_1639_v61	Listeria_monocytogenes_1639_v151	0.9291
Listeria_monocytogenes_1639_v60	Listeria_monocytogenes_1639_v151	0.9325
Listeria_monocytogenes_1639_v5	Listeria_monocytogenes_1639_v151	0.9112
Listeria_monocytogenes_1639_v59	Listeria_monocytogenes_1639_v151	0.9601
Listeria_monocytogenes_1639_v58	Listeria_monocytogenes_1639_v151	0.9613
Listeria_monocytogenes_1639_v57	Listeria_monocytogenes_1639_v151	0.9756
Listeria_monocytogenes_1639_v56	Listeria_monocytogenes_1639_v151	0.9598
Listeria_monocytogenes_1639_v55	Listeria_monocytogenes_1639_v151	0.9716
Listeria_monocytogenes_1639_v54	Listeria_monocytogenes_1639_v151	0.927
Listeria_monocytogenes_1639_v53	Listeria_monocytogenes_1639_v151	0.9325
Listeria_monocytogenes_1639_v52	Listeria_monocytogenes_1639_v151	0.9565
Listeria_monocytogenes_1639_v51	Listeria_monocytogenes_1639_v151	0.954
Listeria_monocytogenes_1639_v50	Listeria_monocytogenes_1639_v151	0.9256
Listeria_monocytogenes_1639_v4	Listeria_monocytogenes_1639_v151	0.9242
Listeria_monocytogenes_1639_v49	Listeria_monocytogenes_1639_v151	0.958
Listeria_monocytogenes_1639_v48	Listeria_monocytogenes_1639_v151	0.9271
Listeria_monocytogenes_1639_v47	Listeria_monocytogenes_1639_v151	0.926
Listeria_monocytogenes_1639_v46	Listeria_monocytogenes_1639_v151	0.9348
Listeria_monocytogenes_1639_v45	Listeria_monocytogenes_1639_v151	0.9168
Listeria_monocytogenes_1639_v44	Listeria_monocytogenes_1639_v151	0.9264
Listeria_monocytogenes_1639_v43	Listeria_monocytogenes_1639_v151	0.9264
Listeria_monocytogenes_1639_v42	Listeria_monocytogenes_1639_v151	0.916
Listeria_monocytogenes_1639_v41	Listeria_monocytogenes_1639_v151	0.9051
Listeria_monocytogenes_1639_v40	Listeria_monocytogenes_1639_v151	0.9316
Listeria_monocytogenes_1639_v3	Listeria_monocytogenes_1639_v151	0.9246
Listeria_monocytogenes_1639_v39	Listeria_monocytogenes_1639_v151	0.9442
Listeria_monocytogenes_1639_v38	Listeria_monocytogenes_1639_v151	0.9288
Listeria_monocytogenes_1639_v37	Listeria_monocytogenes_1639_v151	0.9247
Listeria_monocytogenes_1639_v36	Listeria_monocytogenes_1639_v151	0.923
Listeria_monocytogenes_1639_v35	Listeria_monocytogenes_1639_v151	0.9323
Listeria_monocytogenes_1639_v34	Listeria_monocytogenes_1639_v151	0.9274
Listeria_monocytogenes_1639_v33	Listeria_monocytogenes_1639_v151	0.9447
Listeria_monocytogenes_1639_v32	Listeria_monocytogenes_1639_v151	0.9237
Listeria_monocytogenes_1639_v31	Listeria_monocytogenes_1639_v151	0.9278
Listeria_monocytogenes_1639_v30	Listeria_monocytogenes_1639_v151	0.9464
Listeria_monocytogenes_1639_v2	Listeria_monocytogenes_1639_v151	0.9083
Listeria_monocytogenes_1639_v29	Listeria_monocytogenes_1639_v151	0.9185
Listeria_monocytogenes_1639_v28	Listeria_monocytogenes_1639_v151	0.9229
Listeria_monocytogenes_1639_v27	Listeria_monocytogenes_1639_v151	0.9137
Listeria_monocytogenes_1639_v26	Listeria_monocytogenes_1639_v151	0.9138
Listeria_monocytogenes_1639_v25	Listeria_monocytogenes_1639_v151	0.9134
Listeria_monocytogenes_1639_v24	Listeria_monocytogenes_1639_v151	0.9504
Listeria_monocytogenes_1639_v23	Listeria_monocytogenes_1639_v151	0.9504
Listeria_monocytogenes_1639_v22	Listeria_monocytogenes_1639_v151	0.9472
Listeria_monocytogenes_1639_v21	Listeria_monocytogenes_1639_v151	0.95
Listeria_monocytogenes_1639_v20	Listeria_monocytogenes_1639_v151	0.9379
Listeria_monocytogenes_1639_v1	Listeria_monocytogenes_1639_v151	0.9092
Listeria_monocytogenes_1639_v19	Listeria_monocytogenes_1639_v151	0.9375
Listeria_monocytogenes_1639_v198	Listeria_monocytogenes_1639_v151	0.8991
Listeria_monocytogenes_1639_v197	Listeria_monocytogenes_1639_v151	0.9284
Listeria_monocytogenes_1639_v196	Listeria_monocytogenes_1639_v151	0.9249
Listeria_monocytogenes_1639_v195	Listeria_monocytogenes_1639_v151	0.9489
Listeria_monocytogenes_1639_v194	Listeria_monocytogenes_1639_v151	0.9486
Listeria_monocytogenes_1639_v193	Listeria_monocytogenes_1639_v151	0.9486
Listeria_monocytogenes_1639_v192	Listeria_monocytogenes_1639_v151	0.9114
Listeria_monocytogenes_1639_v191	Listeria_monocytogenes_1639_v151	0.9534
Listeria_monocytogenes_1639_v190	Listeria_monocytogenes_1639_v151	0.9542
Listeria_monocytogenes_1639_v18	Listeria_monocytogenes_1639_v151	0.9694
Listeria_monocytogenes_1639_v189	Listeria_monocytogenes_1639_v151	0.9531
Listeria_monocytogenes_1639_v188	Listeria_monocytogenes_1639_v151	0.9538
Listeria_monocytogenes_1639_v187	Listeria_monocytogenes_1639_v151	0.9539

Listeria_monocytogenes_1639_v186	Listeria_monocytogenes_1639_v151	0.9542
Listeria_monocytogenes_1639_v185	Listeria_monocytogenes_1639_v151	0.9542
Listeria_monocytogenes_1639_v184	Listeria_monocytogenes_1639_v151	0.9535
Listeria_monocytogenes_1639_v183	Listeria_monocytogenes_1639_v151	0.9469
Listeria_monocytogenes_1639_v182	Listeria_monocytogenes_1639_v151	0.9491
Listeria_monocytogenes_1639_v181	Listeria_monocytogenes_1639_v151	0.9348
Listeria_monocytogenes_1639_v180	Listeria_monocytogenes_1639_v151	0.9348
Listeria_monocytogenes_1639_v17	Listeria_monocytogenes_1639_v151	0.9694
Listeria_monocytogenes_1639_v179	Listeria_monocytogenes_1639_v151	0.9382
Listeria_monocytogenes_1639_v178	Listeria_monocytogenes_1639_v151	0.9539
Listeria_monocytogenes_1639_v177	Listeria_monocytogenes_1639_v151	0.9992
Listeria_monocytogenes_1639_v176	Listeria_monocytogenes_1639_v151	0.9467
Listeria_monocytogenes_1639_v175	Listeria_monocytogenes_1639_v151	0.942
Listeria_monocytogenes_1639_v174	Listeria_monocytogenes_1639_v151	0.9543
Listeria_monocytogenes_1639_v173	Listeria_monocytogenes_1639_v151	0.9714
Listeria_monocytogenes_1639_v172	Listeria_monocytogenes_1639_v151	0.9442
Listeria_monocytogenes_1639_v171	Listeria_monocytogenes_1639_v151	0.939
Listeria_monocytogenes_1639_v170	Listeria_monocytogenes_1639_v151	0.9473
Listeria_monocytogenes_1639_v16	Listeria_monocytogenes_1639_v151	0.9127
Listeria_monocytogenes_1639_v169	Listeria_monocytogenes_1639_v151	0.9462
Listeria_monocytogenes_1639_v168	Listeria_monocytogenes_1639_v151	0.9517
Listeria_monocytogenes_1639_v167	Listeria_monocytogenes_1639_v151	0.9399
Listeria_monocytogenes_1639_v166	Listeria_monocytogenes_1639_v151	0.952
Listeria_monocytogenes_1639_v165	Listeria_monocytogenes_1639_v151	0.9458
Listeria_monocytogenes_1639_v164	Listeria_monocytogenes_1639_v151	0.9749
Listeria_monocytogenes_1639_v163	Listeria_monocytogenes_1639_v151	0.9649
Listeria_monocytogenes_1639_v162	Listeria_monocytogenes_1639_v151	0.9749
Listeria_monocytogenes_1639_v161	Listeria_monocytogenes_1639_v151	0.9753
Listeria_monocytogenes_1639_v160	Listeria_monocytogenes_1639_v151	0.9753
Listeria_monocytogenes_1639_v15	Listeria_monocytogenes_1639_v151	0.9304
Listeria_monocytogenes_1639_v159	Listeria_monocytogenes_1639_v151	0.9996
Listeria_monocytogenes_1639_v158	Listeria_monocytogenes_1639_v151	0.9757
Listeria_monocytogenes_1639_v157	Listeria_monocytogenes_1639_v151	0.9757
Listeria_monocytogenes_1639_v156	Listeria_monocytogenes_1639_v151	0.9304
Listeria_monocytogenes_1639_v155	Listeria_monocytogenes_1639_v151	0.9417
Listeria_monocytogenes_1639_v154	Listeria_monocytogenes_1639_v151	0.9452
Listeria_monocytogenes_1639_v153	Listeria_monocytogenes_1639_v151	0.9424
Listeria_monocytogenes_1639_v152	Listeria_monocytogenes_1639_v151	1
Listeria_monocytogenes_1639_v151	Listeria_monocytogenes_1639_v151	0.0643
Listeria_welshimeri_1643	Listeria_monocytogenes_1639_v152	0.8819
Listeria_welshimeri_serovar_6b_str_SLCC5334_386043	Listeria_monocytogenes_1639_v152	0.8813
Listeria_seeligeri_serovar_1_2b_str_SLCC3954_683837	Listeria_monocytogenes_1639_v152	0.89
Listeria_seeligeri_FSL_S4-171_702452	Listeria_monocytogenes_1639_v152	0.8306
Listeria_seeligeri_FSL_N1-067_702453	Listeria_monocytogenes_1639_v152	0.8345
Listeria_seeligeri_1640_v1	Listeria_monocytogenes_1639_v152	0.8976
Listeria_seeligeri_1640	Listeria_monocytogenes_1639_v152	0.8902
Listeria_monocytogenes_WSLC1042_1457188	Listeria_monocytogenes_1639_v152	0.9683
Listeria_monocytogenes_WSLC1001_1457187	Listeria_monocytogenes_1639_v152	0.9368
Listeria_monocytogenes_SLCC7179_879090	Listeria_monocytogenes_1639_v152	0.942
Listeria_monocytogenes_SLCC5850_882096	Listeria_monocytogenes_1639_v152	0.9415
Listeria_monocytogenes_SLCC2755_932919	Listeria_monocytogenes_1639_v152	0.9511
Listeria_monocytogenes_SLCC2540_879089	Listeria_monocytogenes_1639_v152	0.9505
Listeria_monocytogenes_SLCC2479_882020	Listeria_monocytogenes_1639_v152	0.9327
Listeria_monocytogenes_SLCC2378_879088	Listeria_monocytogenes_1639_v152	0.9698
Listeria_monocytogenes_SLCC2376_882097	Listeria_monocytogenes_1639_v152	0.9478
Listeria_monocytogenes_SLCC2372_932920	Listeria_monocytogenes_1639_v152	0.9254
Listeria_monocytogenes_serotype_4b_str_LL195_1230340	Listeria_monocytogenes_1639_v152	0.9757
Listeria_monocytogenes_serotype_4b_str_LIS0087_1134669	Listeria_monocytogenes_1639_v152	0.9182
Listeria_monocytogenes_serotype_4b_str_F2365_265669	Listeria_monocytogenes_1639_v152	0.9757
Listeria_monocytogenes_serotype_4b_str_CLIP_80459_568819	Listeria_monocytogenes_1639_v152	0.9776
Listeria_monocytogenes_serotype_4b_str_81-0861_1196161	Listeria_monocytogenes_1639_v152	0.958
Listeria_monocytogenes_serotype_4b_str_81-0592_1196160	Listeria_monocytogenes_1639_v152	0.9591
Listeria_monocytogenes_serotype_4b_str_81-0558_1196159	Listeria_monocytogenes_1639_v152	0.9591
Listeria_monocytogenes_serotype_4b_str_10-0809_1196162	Listeria_monocytogenes_1639_v152	0.9595
Listeria_monocytogenes_serotype_4b_str_02-6680_1196175	Listeria_monocytogenes_1639_v152	0.9746
Listeria_monocytogenes_serotype_4b_str_02-6679_1196174	Listeria_monocytogenes_1639_v152	0.9746
Listeria_monocytogenes_serotype_4b_str_02-1792_1196173	Listeria_monocytogenes_1639_v152	0.9598
Listeria_monocytogenes_serotype_4b_str_02-1289_1196172	Listeria_monocytogenes_1639_v152	0.9598
Listeria_monocytogenes_serotype_4b_str_02-1103_1196171	Listeria_monocytogenes_1639_v152	0.9598
Listeria_monocytogenes_serotype_3c_str_10-5027_1196195	Listeria_monocytogenes_1639_v152	0.9319
Listeria_monocytogenes_serotype_1_2c_str_10-5026_1196194	Listeria_monocytogenes_1639_v152	0.9331
Listeria_monocytogenes_serotype_1_2c_str_10-5025_1196193	Listeria_monocytogenes_1639_v152	0.9331
Listeria_monocytogenes_serotype_1_2b_str_10-0811_1196164	Listeria_monocytogenes_1639_v152	0.9447
Listeria_monocytogenes_serotype_1_2b_str_10-0810_1196163	Listeria_monocytogenes_1639_v152	0.9447
Listeria_monocytogenes_serotype_1_2a_str_99-6370_1196179	Listeria_monocytogenes_1639_v152	0.9205
Listeria_monocytogenes_serotype_1_2a_str_98-2035_1196178	Listeria_monocytogenes_1639_v152	0.9205
Listeria_monocytogenes_serotype_1_2a_str_95-0093_1196177	Listeria_monocytogenes_1639_v152	0.926
Listeria_monocytogenes_serotype_1_2a_str_88-0478_1196176	Listeria_monocytogenes_1639_v152	0.9252
Listeria_monocytogenes_serotype_1_2a_str_10-5024_1196192	Listeria_monocytogenes_1639_v152	0.9264
Listeria_monocytogenes_serotype_1_2a_str_10-4758_1196168	Listeria_monocytogenes_1639_v152	0.9423
Listeria_monocytogenes_serotype_1_2a_str_10-4754_1196167	Listeria_monocytogenes_1639_v152	0.9419
Listeria_monocytogenes_serotype_1_2a_str_10-1321_1196191	Listeria_monocytogenes_1639_v152	0.9264
Listeria_monocytogenes_serotype_1_2a_str_10-1047_1196190	Listeria_monocytogenes_1639_v152	0.9267
Listeria_monocytogenes_serotype_1_2a_str_10-1046_1196189	Listeria_monocytogenes_1639_v152	0.9267
Listeria_monocytogenes_serotype_1_2a_str_10-0934_1196170	Listeria_monocytogenes_1639_v152	0.9516
Listeria_monocytogenes_serotype_1_2a_str_10-0933_1196169	Listeria_monocytogenes_1639_v152	0.9516
Listeria_monocytogenes_serotype_1_2a_str_10-0815_1196188	Listeria_monocytogenes_1639_v152	0.9219
Listeria_monocytogenes_serotype_1_2a_str_10-0814_1196187	Listeria_monocytogenes_1639_v152	0.9264
Listeria_monocytogenes_serotype_1_2a_str_10-0813_1196166	Listeria_monocytogenes_1639_v152	0.934
Listeria_monocytogenes_serotype_1_2a_str_10-0812_1196165	Listeria_monocytogenes_1639_v152	0.934
Listeria_monocytogenes_serotype_1_2a_str_08-7669_1196186	Listeria_monocytogenes_1639_v152	0.9355

Listeria_monocytogenes_serotype_1_2a_str_08-7374_1196185	Listeria_monocytogenes_1639_v152	0.9264
Listeria_monocytogenes_serotype_1_2a_str_08-6997_1196184	Listeria_monocytogenes_1639_v152	0.9219
Listeria_monocytogenes_serotype_1_2a_str_08-6569_1196183	Listeria_monocytogenes_1639_v152	0.9219
Listeria_monocytogenes_serotype_1_2a_str_08-6056_1196182	Listeria_monocytogenes_1639_v152	0.9219
Listeria_monocytogenes_serotype_1_2a_str_04-5457_1196181	Listeria_monocytogenes_1639_v152	0.9264
Listeria_monocytogenes_serotype_1_2a_str_02-5993_1196180	Listeria_monocytogenes_1639_v152	0.926
Listeria_monocytogenes_R479a_1437838	Listeria_monocytogenes_1639_v152	0.9253
Listeria_monocytogenes_N53-1_1234142	Listeria_monocytogenes_1639_v152	0.8968
Listeria_monocytogenes_M7_1030009	Listeria_monocytogenes_1639_v152	0.9111
Listeria_monocytogenes_LO28_393132	Listeria_monocytogenes_1639_v152	0.9313
Listeria_monocytogenes_L99_563174	Listeria_monocytogenes_1639_v152	0.9118
Listeria_monocytogenes_L312_882094	Listeria_monocytogenes_1639_v152	0.978
Listeria_monocytogenes_J1816_930781	Listeria_monocytogenes_1639_v152	0.9777
Listeria_monocytogenes_J1-220_930782	Listeria_monocytogenes_1639_v152	0.9504
Listeria_monocytogenes_J0161_393130	Listeria_monocytogenes_1639_v152	0.9305
Listeria_monocytogenes_HCC23_552536	Listeria_monocytogenes_1639_v152	0.9111
Listeria_monocytogenes_FSL_R2-561_393126	Listeria_monocytogenes_1639_v152	0.9313
Listeria_monocytogenes_FSL_J1-208_393119	Listeria_monocytogenes_1639_v152	0.9196
Listeria_monocytogenes_FSL_J1-175_393118	Listeria_monocytogenes_1639_v152	0.9568
Listeria_monocytogenes_Finland_1998_393127	Listeria_monocytogenes_1639_v152	0.9528
Listeria_monocytogenes_EGD-e_169963_v1	Listeria_monocytogenes_1639_v152	0.9405
Listeria_monocytogenes_EGD-e_169963	Listeria_monocytogenes_1639_v152	0.9409
Listeria_monocytogenes_EGD_1334565	Listeria_monocytogenes_1639_v152	0.9422
Listeria_monocytogenes_CFSAN002349_1299895	Listeria_monocytogenes_1639_v152	0.9827
Listeria_monocytogenes_ATCC_19117_882095_v1	Listeria_monocytogenes_1639_v152	0.9708
Listeria_monocytogenes_ATCC_19117_882095	Listeria_monocytogenes_1639_v152	0.9708
Listeria_monocytogenes_6179_1288295	Listeria_monocytogenes_1639_v152	0.9151
Listeria_monocytogenes_1639_v9	Listeria_monocytogenes_1639_v152	0.9272
Listeria_monocytogenes_1639_v99	Listeria_monocytogenes_1639_v152	0.98
Listeria_monocytogenes_1639_v98	Listeria_monocytogenes_1639_v152	0.9293
Listeria_monocytogenes_1639_v97	Listeria_monocytogenes_1639_v152	0.9673
Listeria_monocytogenes_1639_v96	Listeria_monocytogenes_1639_v152	0.9789
Listeria_monocytogenes_1639_v95	Listeria_monocytogenes_1639_v152	0.9789
Listeria_monocytogenes_1639_v94	Listeria_monocytogenes_1639_v152	0.9793
Listeria_monocytogenes_1639_v93	Listeria_monocytogenes_1639_v152	0.9278
Listeria_monocytogenes_1639_v92	Listeria_monocytogenes_1639_v152	0.9796
Listeria_monocytogenes_1639_v91	Listeria_monocytogenes_1639_v152	0.9558
Listeria_monocytogenes_1639_v90	Listeria_monocytogenes_1639_v152	0.9293
Listeria_monocytogenes_1639_v8	Listeria_monocytogenes_1639_v152	0.9154
Listeria_monocytogenes_1639_v89	Listeria_monocytogenes_1639_v152	0.9672
Listeria_monocytogenes_1639_v88	Listeria_monocytogenes_1639_v152	0.9325
Listeria_monocytogenes_1639_v87	Listeria_monocytogenes_1639_v152	0.9301
Listeria_monocytogenes_1639_v86	Listeria_monocytogenes_1639_v152	0.9294
Listeria_monocytogenes_1639_v85	Listeria_monocytogenes_1639_v152	0.9573
Listeria_monocytogenes_1639_v84	Listeria_monocytogenes_1639_v152	0.9361
Listeria_monocytogenes_1639_v83	Listeria_monocytogenes_1639_v152	0.9594
Listeria_monocytogenes_1639_v82	Listeria_monocytogenes_1639_v152	0.9587
Listeria_monocytogenes_1639_v81	Listeria_monocytogenes_1639_v152	0.9333
Listeria_monocytogenes_1639_v80	Listeria_monocytogenes_1639_v152	0.951
Listeria_monocytogenes_1639_v7	Listeria_monocytogenes_1639_v152	0.9154
Listeria_monocytogenes_1639_v79	Listeria_monocytogenes_1639_v152	0.9311
Listeria_monocytogenes_1639_v78	Listeria_monocytogenes_1639_v152	0.9326
Listeria_monocytogenes_1639_v77	Listeria_monocytogenes_1639_v152	0.9327
Listeria_monocytogenes_1639_v76	Listeria_monocytogenes_1639_v152	0.9259
Listeria_monocytogenes_1639_v75	Listeria_monocytogenes_1639_v152	0.9118
Listeria_monocytogenes_1639_v74	Listeria_monocytogenes_1639_v152	0.9283
Listeria_monocytogenes_1639_v73	Listeria_monocytogenes_1639_v152	0.9264
Listeria_monocytogenes_1639_v72	Listeria_monocytogenes_1639_v152	0.9582
Listeria_monocytogenes_1639_v71	Listeria_monocytogenes_1639_v152	0.9168
Listeria_monocytogenes_1639_v70	Listeria_monocytogenes_1639_v152	0.9292
Listeria_monocytogenes_1639_v6	Listeria_monocytogenes_1639_v152	0.9147
Listeria_monocytogenes_1639_v69	Listeria_monocytogenes_1639_v152	0.9595
Listeria_monocytogenes_1639_v68	Listeria_monocytogenes_1639_v152	0.9355
Listeria_monocytogenes_1639_v67	Listeria_monocytogenes_1639_v152	0.9465
Listeria_monocytogenes_1639_v66	Listeria_monocytogenes_1639_v152	0.937
Listeria_monocytogenes_1639_v65	Listeria_monocytogenes_1639_v152	0.9295
Listeria_monocytogenes_1639_v64	Listeria_monocytogenes_1639_v152	0.9284
Listeria_monocytogenes_1639_v63	Listeria_monocytogenes_1639_v152	0.9453
Listeria_monocytogenes_1639_v62	Listeria_monocytogenes_1639_v152	0.9287
Listeria_monocytogenes_1639_v61	Listeria_monocytogenes_1639_v152	0.9291
Listeria_monocytogenes_1639_v60	Listeria_monocytogenes_1639_v152	0.9325
Listeria_monocytogenes_1639_v5	Listeria_monocytogenes_1639_v152	0.9112
Listeria_monocytogenes_1639_v59	Listeria_monocytogenes_1639_v152	0.9601
Listeria_monocytogenes_1639_v58	Listeria_monocytogenes_1639_v152	0.9613
Listeria_monocytogenes_1639_v57	Listeria_monocytogenes_1639_v152	0.9756
Listeria_monocytogenes_1639_v56	Listeria_monocytogenes_1639_v152	0.9598
Listeria_monocytogenes_1639_v55	Listeria_monocytogenes_1639_v152	0.9716
Listeria_monocytogenes_1639_v54	Listeria_monocytogenes_1639_v152	0.927
Listeria_monocytogenes_1639_v53	Listeria_monocytogenes_1639_v152	0.9325
Listeria_monocytogenes_1639_v52	Listeria_monocytogenes_1639_v152	0.9565
Listeria_monocytogenes_1639_v51	Listeria_monocytogenes_1639_v152	0.954
Listeria_monocytogenes_1639_v50	Listeria_monocytogenes_1639_v152	0.9256
Listeria_monocytogenes_1639_v4	Listeria_monocytogenes_1639_v152	0.9242
Listeria_monocytogenes_1639_v49	Listeria_monocytogenes_1639_v152	0.958
Listeria_monocytogenes_1639_v48	Listeria_monocytogenes_1639_v152	0.9271
Listeria_monocytogenes_1639_v47	Listeria_monocytogenes_1639_v152	0.926
Listeria_monocytogenes_1639_v46	Listeria_monocytogenes_1639_v152	0.9348
Listeria_monocytogenes_1639_v45	Listeria_monocytogenes_1639_v152	0.9168
Listeria_monocytogenes_1639_v44	Listeria_monocytogenes_1639_v152	0.9264
Listeria_monocytogenes_1639_v43	Listeria_monocytogenes_1639_v152	0.9264
Listeria_monocytogenes_1639_v42	Listeria_monocytogenes_1639_v152	0.916

Listeria_monocytogenes_1639_v41	Listeria_monocytogenes_1639_v152	0.9051
Listeria_monocytogenes_1639_v40	Listeria_monocytogenes_1639_v152	0.9316
Listeria_monocytogenes_1639_v3	Listeria_monocytogenes_1639_v152	0.9246
Listeria_monocytogenes_1639_v39	Listeria_monocytogenes_1639_v152	0.9442
Listeria_monocytogenes_1639_v38	Listeria_monocytogenes_1639_v152	0.9288
Listeria_monocytogenes_1639_v37	Listeria_monocytogenes_1639_v152	0.9247
Listeria_monocytogenes_1639_v36	Listeria_monocytogenes_1639_v152	0.923
Listeria_monocytogenes_1639_v35	Listeria_monocytogenes_1639_v152	0.9323
Listeria_monocytogenes_1639_v34	Listeria_monocytogenes_1639_v152	0.9274
Listeria_monocytogenes_1639_v33	Listeria_monocytogenes_1639_v152	0.9447
Listeria_monocytogenes_1639_v32	Listeria_monocytogenes_1639_v152	0.9237
Listeria_monocytogenes_1639_v31	Listeria_monocytogenes_1639_v152	0.9278
Listeria_monocytogenes_1639_v30	Listeria_monocytogenes_1639_v152	0.9464
Listeria_monocytogenes_1639_v2	Listeria_monocytogenes_1639_v152	0.9083
Listeria_monocytogenes_1639_v29	Listeria_monocytogenes_1639_v152	0.9185
Listeria_monocytogenes_1639_v28	Listeria_monocytogenes_1639_v152	0.9229
Listeria_monocytogenes_1639_v27	Listeria_monocytogenes_1639_v152	0.9137
Listeria_monocytogenes_1639_v26	Listeria_monocytogenes_1639_v152	0.9138
Listeria_monocytogenes_1639_v25	Listeria_monocytogenes_1639_v152	0.9134
Listeria_monocytogenes_1639_v24	Listeria_monocytogenes_1639_v152	0.9504
Listeria_monocytogenes_1639_v23	Listeria_monocytogenes_1639_v152	0.9504
Listeria_monocytogenes_1639_v22	Listeria_monocytogenes_1639_v152	0.9472
Listeria_monocytogenes_1639_v21	Listeria_monocytogenes_1639_v152	0.95
Listeria_monocytogenes_1639_v20	Listeria_monocytogenes_1639_v152	0.9379
Listeria_monocytogenes_1639_v1	Listeria_monocytogenes_1639_v152	0.9092
Listeria_monocytogenes_1639_v19	Listeria_monocytogenes_1639_v152	0.9375
Listeria_monocytogenes_1639_v198	Listeria_monocytogenes_1639_v152	0.8991
Listeria_monocytogenes_1639_v197	Listeria_monocytogenes_1639_v152	0.9284
Listeria_monocytogenes_1639_v196	Listeria_monocytogenes_1639_v152	0.9249
Listeria_monocytogenes_1639_v195	Listeria_monocytogenes_1639_v152	0.9489
Listeria_monocytogenes_1639_v194	Listeria_monocytogenes_1639_v152	0.9486
Listeria_monocytogenes_1639_v193	Listeria_monocytogenes_1639_v152	0.9486
Listeria_monocytogenes_1639_v192	Listeria_monocytogenes_1639_v152	0.9114
Listeria_monocytogenes_1639_v191	Listeria_monocytogenes_1639_v152	0.9534
Listeria_monocytogenes_1639_v190	Listeria_monocytogenes_1639_v152	0.9542
Listeria_monocytogenes_1639_v18	Listeria_monocytogenes_1639_v152	0.9694
Listeria_monocytogenes_1639_v189	Listeria_monocytogenes_1639_v152	0.9531
Listeria_monocytogenes_1639_v188	Listeria_monocytogenes_1639_v152	0.9538
Listeria_monocytogenes_1639_v187	Listeria_monocytogenes_1639_v152	0.9539
Listeria_monocytogenes_1639_v186	Listeria_monocytogenes_1639_v152	0.9542
Listeria_monocytogenes_1639_v185	Listeria_monocytogenes_1639_v152	0.9542
Listeria_monocytogenes_1639_v184	Listeria_monocytogenes_1639_v152	0.9535
Listeria_monocytogenes_1639_v183	Listeria_monocytogenes_1639_v152	0.9469
Listeria_monocytogenes_1639_v182	Listeria_monocytogenes_1639_v152	0.9491
Listeria_monocytogenes_1639_v181	Listeria_monocytogenes_1639_v152	0.9348
Listeria_monocytogenes_1639_v180	Listeria_monocytogenes_1639_v152	0.9348
Listeria_monocytogenes_1639_v17	Listeria_monocytogenes_1639_v152	0.9694
Listeria_monocytogenes_1639_v179	Listeria_monocytogenes_1639_v152	0.9382
Listeria_monocytogenes_1639_v178	Listeria_monocytogenes_1639_v152	0.9539
Listeria_monocytogenes_1639_v177	Listeria_monocytogenes_1639_v152	0.9992
Listeria_monocytogenes_1639_v176	Listeria_monocytogenes_1639_v152	0.9467
Listeria_monocytogenes_1639_v175	Listeria_monocytogenes_1639_v152	0.942
Listeria_monocytogenes_1639_v174	Listeria_monocytogenes_1639_v152	0.9543
Listeria_monocytogenes_1639_v173	Listeria_monocytogenes_1639_v152	0.9714
Listeria_monocytogenes_1639_v172	Listeria_monocytogenes_1639_v152	0.9442
Listeria_monocytogenes_1639_v171	Listeria_monocytogenes_1639_v152	0.939
Listeria_monocytogenes_1639_v170	Listeria_monocytogenes_1639_v152	0.9473
Listeria_monocytogenes_1639_v16	Listeria_monocytogenes_1639_v152	0.9127
Listeria_monocytogenes_1639_v169	Listeria_monocytogenes_1639_v152	0.9462
Listeria_monocytogenes_1639_v168	Listeria_monocytogenes_1639_v152	0.9517
Listeria_monocytogenes_1639_v167	Listeria_monocytogenes_1639_v152	0.9399
Listeria_monocytogenes_1639_v166	Listeria_monocytogenes_1639_v152	0.952
Listeria_monocytogenes_1639_v165	Listeria_monocytogenes_1639_v152	0.9458
Listeria_monocytogenes_1639_v164	Listeria_monocytogenes_1639_v152	0.9749
Listeria_monocytogenes_1639_v163	Listeria_monocytogenes_1639_v152	0.9649
Listeria_monocytogenes_1639_v162	Listeria_monocytogenes_1639_v152	0.9749
Listeria_monocytogenes_1639_v161	Listeria_monocytogenes_1639_v152	0.9753
Listeria_monocytogenes_1639_v160	Listeria_monocytogenes_1639_v152	0.9753
Listeria_monocytogenes_1639_v15	Listeria_monocytogenes_1639_v152	0.9304
Listeria_monocytogenes_1639_v159	Listeria_monocytogenes_1639_v152	0.9996
Listeria_monocytogenes_1639_v158	Listeria_monocytogenes_1639_v152	0.9757
Listeria_monocytogenes_1639_v157	Listeria_monocytogenes_1639_v152	0.9757
Listeria_monocytogenes_1639_v156	Listeria_monocytogenes_1639_v152	0.9304
Listeria_monocytogenes_1639_v155	Listeria_monocytogenes_1639_v152	0.9417
Listeria_monocytogenes_1639_v154	Listeria_monocytogenes_1639_v152	0.9452
Listeria_monocytogenes_1639_v153	Listeria_monocytogenes_1639_v152	0.9424
Listeria_monocytogenes_1639_v152	Listeria_monocytogenes_1639_v152	0.0643
Listeria_welshimeri_1643	Listeria_monocytogenes_1639_v153	0.8692
Listeria_welshimeri_serovar_6b_str_SLCC5334_386043	Listeria_monocytogenes_1639_v153	0.8685
Listeria_seeligeri_serovar_1_2b_str_SLCC3954_683837	Listeria_monocytogenes_1639_v153	0.8849
Listeria_seeligeri_FSL_S4-171_702452	Listeria_monocytogenes_1639_v153	0.8236
Listeria_seeligeri_FSL_N1-067_702453	Listeria_monocytogenes_1639_v153	0.8335
Listeria_seeligeri_1640_v1	Listeria_monocytogenes_1639_v153	0.881
Listeria_seeligeri_1640	Listeria_monocytogenes_1639_v153	0.8867
Listeria_monocytogenes_WSLC1042_1457188	Listeria_monocytogenes_1639_v153	0.9314
Listeria_monocytogenes_WSLC1001_1457187	Listeria_monocytogenes_1639_v153	0.9491
Listeria_monocytogenes_SLCC7179_879090	Listeria_monocytogenes_1639_v153	0.9667
Listeria_monocytogenes_SLCC5850_882096	Listeria_monocytogenes_1639_v153	0.9535
Listeria_monocytogenes_SLCC2755_932919	Listeria_monocytogenes_1639_v153	0.9342
Listeria_monocytogenes_SLCC2540_879089	Listeria_monocytogenes_1639_v153	0.9358
Listeria_monocytogenes_SLCC2479_882020	Listeria_monocytogenes_1639_v153	0.9532

Listeria_monocytogenes_SLCC2378_879088	Listeria_monocytogenes_1639_v153	0.9328
Listeria_monocytogenes_SLCC2376_882097	Listeria_monocytogenes_1639_v153	0.9456
Listeria_monocytogenes_SLCC2372_932920	Listeria_monocytogenes_1639_v153	0.9458
Listeria_monocytogenes_serotype_4b_str_LL195_1230340	Listeria_monocytogenes_1639_v153	0.9389
Listeria_monocytogenes_serotype_4b_str_LIS0087_1134669	Listeria_monocytogenes_1639_v153	0.9411
Listeria_monocytogenes_serotype_4b_str_F2365_265669	Listeria_monocytogenes_1639_v153	0.9389
Listeria_monocytogenes_serotype_4b_str_CLIP_80459_568819	Listeria_monocytogenes_1639_v153	0.9469
Listeria_monocytogenes_serotype_4b_str_81-0861_1196161	Listeria_monocytogenes_1639_v153	0.9351
Listeria_monocytogenes_serotype_4b_str_81-0592_1196160	Listeria_monocytogenes_1639_v153	0.9362
Listeria_monocytogenes_serotype_4b_str_81-0558_1196159	Listeria_monocytogenes_1639_v153	0.9369
Listeria_monocytogenes_serotype_4b_str_10-0809_1196162	Listeria_monocytogenes_1639_v153	0.9362
Listeria_monocytogenes_serotype_4b_str_02-6680_1196175	Listeria_monocytogenes_1639_v153	0.9424
Listeria_monocytogenes_serotype_4b_str_02-6679_1196174	Listeria_monocytogenes_1639_v153	0.9424
Listeria_monocytogenes_serotype_4b_str_02-1792_1196173	Listeria_monocytogenes_1639_v153	0.9369
Listeria_monocytogenes_serotype_4b_str_02-1289_1196172	Listeria_monocytogenes_1639_v153	0.9369
Listeria_monocytogenes_serotype_4b_str_02-1103_1196171	Listeria_monocytogenes_1639_v153	0.9369
Listeria_monocytogenes_serotype_3c_str_10-5027_1196195	Listeria_monocytogenes_1639_v153	0.9528
Listeria_monocytogenes_serotype_1_2c_str_10-5026_1196194	Listeria_monocytogenes_1639_v153	0.954
Listeria_monocytogenes_serotype_1_2c_str_10-5025_1196193	Listeria_monocytogenes_1639_v153	0.954
Listeria_monocytogenes_serotype_1_2b_str_10-0811_1196164	Listeria_monocytogenes_1639_v153	0.9429
Listeria_monocytogenes_serotype_1_2b_str_10-0810_1196163	Listeria_monocytogenes_1639_v153	0.9429
Listeria_monocytogenes_serotype_1_2a_str_99-6370_1196179	Listeria_monocytogenes_1639_v153	0.9371
Listeria_monocytogenes_serotype_1_2a_str_98-2035_1196178	Listeria_monocytogenes_1639_v153	0.9371
Listeria_monocytogenes_serotype_1_2a_str_95-0093_1196177	Listeria_monocytogenes_1639_v153	0.9427
Listeria_monocytogenes_serotype_1_2a_str_88-0478_1196176	Listeria_monocytogenes_1639_v153	0.9419
Listeria_monocytogenes_serotype_1_2a_str_10-5024_1196192	Listeria_monocytogenes_1639_v153	0.943
Listeria_monocytogenes_serotype_1_2a_str_10-4758_1196168	Listeria_monocytogenes_1639_v153	0.9524
Listeria_monocytogenes_serotype_1_2a_str_10-4754_1196167	Listeria_monocytogenes_1639_v153	0.9524
Listeria_monocytogenes_serotype_1_2a_str_10-1321_1196191	Listeria_monocytogenes_1639_v153	0.943
Listeria_monocytogenes_serotype_1_2a_str_10-1047_1196190	Listeria_monocytogenes_1639_v153	0.943
Listeria_monocytogenes_serotype_1_2a_str_10-1046_1196189	Listeria_monocytogenes_1639_v153	0.943
Listeria_monocytogenes_serotype_1_2a_str_10-0934_1196170	Listeria_monocytogenes_1639_v153	0.9629
Listeria_monocytogenes_serotype_1_2a_str_10-0933_1196169	Listeria_monocytogenes_1639_v153	0.9629
Listeria_monocytogenes_serotype_1_2a_str_10-0815_1196188	Listeria_monocytogenes_1639_v153	0.9385
Listeria_monocytogenes_serotype_1_2a_str_10-0814_1196187	Listeria_monocytogenes_1639_v153	0.9427
Listeria_monocytogenes_serotype_1_2a_str_10-0813_1196166	Listeria_monocytogenes_1639_v153	0.9595
Listeria_monocytogenes_serotype_1_2a_str_10-0812_1196165	Listeria_monocytogenes_1639_v153	0.9595
Listeria_monocytogenes_serotype_1_2a_str_08-7669_1196186	Listeria_monocytogenes_1639_v153	0.9519
Listeria_monocytogenes_serotype_1_2a_str_08-7374_1196185	Listeria_monocytogenes_1639_v153	0.943
Listeria_monocytogenes_serotype_1_2a_str_08-6997_1196184	Listeria_monocytogenes_1639_v153	0.9385
Listeria_monocytogenes_serotype_1_2a_str_08-6569_1196183	Listeria_monocytogenes_1639_v153	0.9385
Listeria_monocytogenes_serotype_1_2a_str_08-6056_1196182	Listeria_monocytogenes_1639_v153	0.9385
Listeria_monocytogenes_serotype_1_2a_str_04-5457_1196181	Listeria_monocytogenes_1639_v153	0.943
Listeria_monocytogenes_serotype_1_2a_str_02-5993_1196180	Listeria_monocytogenes_1639_v153	0.9427
Listeria_monocytogenes_R479a_1437838	Listeria_monocytogenes_1639_v153	0.9412
Listeria_monocytogenes_N53-1_1234142	Listeria_monocytogenes_1639_v153	0.9158
Listeria_monocytogenes_M7_1030009	Listeria_monocytogenes_1639_v153	0.9155
Listeria_monocytogenes_LO28_393132	Listeria_monocytogenes_1639_v153	0.959
Listeria_monocytogenes_L99_563174	Listeria_monocytogenes_1639_v153	0.9162
Listeria_monocytogenes_L312_882094	Listeria_monocytogenes_1639_v153	0.9469
Listeria_monocytogenes_J1816_930781	Listeria_monocytogenes_1639_v153	0.9307
Listeria_monocytogenes_J1-220_930782	Listeria_monocytogenes_1639_v153	0.9344
Listeria_monocytogenes_J0161_393130	Listeria_monocytogenes_1639_v153	0.9616
Listeria_monocytogenes_HCC23_552536	Listeria_monocytogenes_1639_v153	0.9158
Listeria_monocytogenes_FSL_R2-561_393126	Listeria_monocytogenes_1639_v153	0.951
Listeria_monocytogenes_FSL_J1-208_393119	Listeria_monocytogenes_1639_v153	0.9088
Listeria_monocytogenes_FSL_J1-175_393118	Listeria_monocytogenes_1639_v153	0.9367
Listeria_monocytogenes_Finland_1998_393127	Listeria_monocytogenes_1639_v153	0.9629
Listeria_monocytogenes_EGD-e_169963_v1	Listeria_monocytogenes_1639_v153	0.968
Listeria_monocytogenes_EGD-e_169963	Listeria_monocytogenes_1639_v153	0.968
Listeria_monocytogenes_EGD_1334565	Listeria_monocytogenes_1639_v153	0.9542
Listeria_monocytogenes_CFSAN002349_1299895	Listeria_monocytogenes_1639_v153	0.9421
Listeria_monocytogenes_ATCC_19117_882095_v1	Listeria_monocytogenes_1639_v153	0.9387
Listeria_monocytogenes_ATCC_19117_882095	Listeria_monocytogenes_1639_v153	0.9387
Listeria_monocytogenes_6179_1288295	Listeria_monocytogenes_1639_v153	0.9428
Listeria_monocytogenes_1639_v9	Listeria_monocytogenes_1639_v153	0.9226
Listeria_monocytogenes_1639_v99	Listeria_monocytogenes_1639_v153	0.9327
Listeria_monocytogenes_1639_v98	Listeria_monocytogenes_1639_v153	0.9774
Listeria_monocytogenes_1639_v97	Listeria_monocytogenes_1639_v153	0.9382
Listeria_monocytogenes_1639_v96	Listeria_monocytogenes_1639_v153	0.9312
Listeria_monocytogenes_1639_v95	Listeria_monocytogenes_1639_v153	0.9312
Listeria_monocytogenes_1639_v94	Listeria_monocytogenes_1639_v153	0.9316
Listeria_monocytogenes_1639_v93	Listeria_monocytogenes_1639_v153	0.9367
Listeria_monocytogenes_1639_v92	Listeria_monocytogenes_1639_v153	0.9319
Listeria_monocytogenes_1639_v91	Listeria_monocytogenes_1639_v153	0.9337
Listeria_monocytogenes_1639_v90	Listeria_monocytogenes_1639_v153	0.9774
Listeria_monocytogenes_1639_v8	Listeria_monocytogenes_1639_v153	0.9289
Listeria_monocytogenes_1639_v89	Listeria_monocytogenes_1639_v153	0.9463
Listeria_monocytogenes_1639_v88	Listeria_monocytogenes_1639_v153	0.9516
Listeria_monocytogenes_1639_v87	Listeria_monocytogenes_1639_v153	0.9563
Listeria_monocytogenes_1639_v86	Listeria_monocytogenes_1639_v153	0.9563
Listeria_monocytogenes_1639_v85	Listeria_monocytogenes_1639_v153	0.9351
Listeria_monocytogenes_1639_v84	Listeria_monocytogenes_1639_v153	0.9545
Listeria_monocytogenes_1639_v83	Listeria_monocytogenes_1639_v153	0.9402
Listeria_monocytogenes_1639_v82	Listeria_monocytogenes_1639_v153	0.9399
Listeria_monocytogenes_1639_v81	Listeria_monocytogenes_1639_v153	0.952
Listeria_monocytogenes_1639_v80	Listeria_monocytogenes_1639_v153	0.945
Listeria_monocytogenes_1639_v7	Listeria_monocytogenes_1639_v153	0.9289
Listeria_monocytogenes_1639_v79	Listeria_monocytogenes_1639_v153	0.9513
Listeria_monocytogenes_1639_v78	Listeria_monocytogenes_1639_v153	0.9516
Listeria_monocytogenes_1639_v77	Listeria_monocytogenes_1639_v153	0.9499

Listeria_monocytogenes_1639_v76	Listeria_monocytogenes_1639_v153	0.9396
Listeria_monocytogenes_1639_v75	Listeria_monocytogenes_1639_v153	0.9162
Listeria_monocytogenes_1639_v74	Listeria_monocytogenes_1639_v153	0.9556
Listeria_monocytogenes_1639_v73	Listeria_monocytogenes_1639_v153	0.9543
Listeria_monocytogenes_1639_v72	Listeria_monocytogenes_1639_v153	0.9402
Listeria_monocytogenes_1639_v71	Listeria_monocytogenes_1639_v153	0.9331
Listeria_monocytogenes_1639_v70	Listeria_monocytogenes_1639_v153	0.9515
Listeria_monocytogenes_1639_v6	Listeria_monocytogenes_1639_v153	0.9283
Listeria_monocytogenes_1639_v69	Listeria_monocytogenes_1639_v153	0.9365
Listeria_monocytogenes_1639_v68	Listeria_monocytogenes_1639_v153	0.9644
Listeria_monocytogenes_1639_v67	Listeria_monocytogenes_1639_v153	0.9353
Listeria_monocytogenes_1639_v66	Listeria_monocytogenes_1639_v153	0.9498
Listeria_monocytogenes_1639_v65	Listeria_monocytogenes_1639_v153	0.9523
Listeria_monocytogenes_1639_v64	Listeria_monocytogenes_1639_v153	0.9415
Listeria_monocytogenes_1639_v63	Listeria_monocytogenes_1639_v153	0.9323
Listeria_monocytogenes_1639_v62	Listeria_monocytogenes_1639_v153	0.9469
Listeria_monocytogenes_1639_v61	Listeria_monocytogenes_1639_v153	0.947
Listeria_monocytogenes_1639_v60	Listeria_monocytogenes_1639_v153	0.9565
Listeria_monocytogenes_1639_v5	Listeria_monocytogenes_1639_v153	0.9071
Listeria_monocytogenes_1639_v59	Listeria_monocytogenes_1639_v153	0.93
Listeria_monocytogenes_1639_v58	Listeria_monocytogenes_1639_v153	0.9368
Listeria_monocytogenes_1639_v57	Listeria_monocytogenes_1639_v153	0.9266
Listeria_monocytogenes_1639_v56	Listeria_monocytogenes_1639_v153	0.9353
Listeria_monocytogenes_1639_v55	Listeria_monocytogenes_1639_v153	0.931
Listeria_monocytogenes_1639_v54	Listeria_monocytogenes_1639_v153	0.9276
Listeria_monocytogenes_1639_v53	Listeria_monocytogenes_1639_v153	0.9467
Listeria_monocytogenes_1639_v52	Listeria_monocytogenes_1639_v153	0.9374
Listeria_monocytogenes_1639_v51	Listeria_monocytogenes_1639_v153	0.9387
Listeria_monocytogenes_1639_v50	Listeria_monocytogenes_1639_v153	0.9416
Listeria_monocytogenes_1639_v4	Listeria_monocytogenes_1639_v153	0.9197
Listeria_monocytogenes_1639_v49	Listeria_monocytogenes_1639_v153	0.9226
Listeria_monocytogenes_1639_v48	Listeria_monocytogenes_1639_v153	0.9487
Listeria_monocytogenes_1639_v47	Listeria_monocytogenes_1639_v153	0.9427
Listeria_monocytogenes_1639_v46	Listeria_monocytogenes_1639_v153	0.9554
Listeria_monocytogenes_1639_v45	Listeria_monocytogenes_1639_v153	0.9333
Listeria_monocytogenes_1639_v44	Listeria_monocytogenes_1639_v153	0.943
Listeria_monocytogenes_1639_v43	Listeria_monocytogenes_1639_v153	0.943
Listeria_monocytogenes_1639_v42	Listeria_monocytogenes_1639_v153	0.9337
Listeria_monocytogenes_1639_v41	Listeria_monocytogenes_1639_v153	0.93
Listeria_monocytogenes_1639_v40	Listeria_monocytogenes_1639_v153	0.9465
Listeria_monocytogenes_1639_v3	Listeria_monocytogenes_1639_v153	0.92
Listeria_monocytogenes_1639_v39	Listeria_monocytogenes_1639_v153	0.9342
Listeria_monocytogenes_1639_v38	Listeria_monocytogenes_1639_v153	0.9429
Listeria_monocytogenes_1639_v37	Listeria_monocytogenes_1639_v153	0.9384
Listeria_monocytogenes_1639_v36	Listeria_monocytogenes_1639_v153	0.9351
Listeria_monocytogenes_1639_v35	Listeria_monocytogenes_1639_v153	0.9468
Listeria_monocytogenes_1639_v34	Listeria_monocytogenes_1639_v153	0.9479
Listeria_monocytogenes_1639_v33	Listeria_monocytogenes_1639_v153	0.9429
Listeria_monocytogenes_1639_v32	Listeria_monocytogenes_1639_v153	0.9507
Listeria_monocytogenes_1639_v31	Listeria_monocytogenes_1639_v153	0.9482
Listeria_monocytogenes_1639_v30	Listeria_monocytogenes_1639_v153	0.9289
Listeria_monocytogenes_1639_v2	Listeria_monocytogenes_1639_v153	0.9067
Listeria_monocytogenes_1639_v29	Listeria_monocytogenes_1639_v153	0.9384
Listeria_monocytogenes_1639_v28	Listeria_monocytogenes_1639_v153	0.944
Listeria_monocytogenes_1639_v27	Listeria_monocytogenes_1639_v153	0.9402
Listeria_monocytogenes_1639_v26	Listeria_monocytogenes_1639_v153	0.9402
Listeria_monocytogenes_1639_v25	Listeria_monocytogenes_1639_v153	0.9402
Listeria_monocytogenes_1639_v24	Listeria_monocytogenes_1639_v153	0.9337
Listeria_monocytogenes_1639_v23	Listeria_monocytogenes_1639_v153	0.9333
Listeria_monocytogenes_1639_v22	Listeria_monocytogenes_1639_v153	0.9451
Listeria_monocytogenes_1639_v21	Listeria_monocytogenes_1639_v153	0.9307
Listeria_monocytogenes_1639_v20	Listeria_monocytogenes_1639_v153	0.9298
Listeria_monocytogenes_1639_v1	Listeria_monocytogenes_1639_v153	0.9378
Listeria_monocytogenes_1639_v19	Listeria_monocytogenes_1639_v153	0.9295
Listeria_monocytogenes_1639_v198	Listeria_monocytogenes_1639_v153	0.9171
Listeria_monocytogenes_1639_v197	Listeria_monocytogenes_1639_v153	0.9545
Listeria_monocytogenes_1639_v196	Listeria_monocytogenes_1639_v153	0.9375
Listeria_monocytogenes_1639_v195	Listeria_monocytogenes_1639_v153	0.9482
Listeria_monocytogenes_1639_v194	Listeria_monocytogenes_1639_v153	0.946
Listeria_monocytogenes_1639_v193	Listeria_monocytogenes_1639_v153	0.9464
Listeria_monocytogenes_1639_v192	Listeria_monocytogenes_1639_v153	0.9037
Listeria_monocytogenes_1639_v191	Listeria_monocytogenes_1639_v153	0.9659
Listeria_monocytogenes_1639_v190	Listeria_monocytogenes_1639_v153	0.9656
Listeria_monocytogenes_1639_v18	Listeria_monocytogenes_1639_v153	0.9278
Listeria_monocytogenes_1639_v189	Listeria_monocytogenes_1639_v153	0.9652
Listeria_monocytogenes_1639_v188	Listeria_monocytogenes_1639_v153	0.9868
Listeria_monocytogenes_1639_v187	Listeria_monocytogenes_1639_v153	0.9656
Listeria_monocytogenes_1639_v186	Listeria_monocytogenes_1639_v153	0.966
Listeria_monocytogenes_1639_v185	Listeria_monocytogenes_1639_v153	0.9656
Listeria_monocytogenes_1639_v184	Listeria_monocytogenes_1639_v153	0.9656
Listeria_monocytogenes_1639_v183	Listeria_monocytogenes_1639_v153	0.9629
Listeria_monocytogenes_1639_v182	Listeria_monocytogenes_1639_v153	0.9651
Listeria_monocytogenes_1639_v181	Listeria_monocytogenes_1639_v153	0.9254
Listeria_monocytogenes_1639_v180	Listeria_monocytogenes_1639_v153	0.9254
Listeria_monocytogenes_1639_v17	Listeria_monocytogenes_1639_v153	0.9278
Listeria_monocytogenes_1639_v179	Listeria_monocytogenes_1639_v153	0.9644
Listeria_monocytogenes_1639_v178	Listeria_monocytogenes_1639_v153	0.9629
Listeria_monocytogenes_1639_v177	Listeria_monocytogenes_1639_v153	0.9421
Listeria_monocytogenes_1639_v176	Listeria_monocytogenes_1639_v153	0.9426
Listeria_monocytogenes_1639_v175	Listeria_monocytogenes_1639_v153	0.9575
Listeria_monocytogenes_1639_v174	Listeria_monocytogenes_1639_v153	0.9656

Listeria_monocytogenes_1639_v173	Listeria_monocytogenes_1639_v153	0.951
Listeria_monocytogenes_1639_v172	Listeria_monocytogenes_1639_v153	0.9397
Listeria_monocytogenes_1639_v171	Listeria_monocytogenes_1639_v153	0.9521
Listeria_monocytogenes_1639_v170	Listeria_monocytogenes_1639_v153	0.9601
Listeria_monocytogenes_1639_v16	Listeria_monocytogenes_1639_v153	0.9292
Listeria_monocytogenes_1639_v169	Listeria_monocytogenes_1639_v153	0.9567
Listeria_monocytogenes_1639_v168	Listeria_monocytogenes_1639_v153	0.958
Listeria_monocytogenes_1639_v167	Listeria_monocytogenes_1639_v153	0.9601
Listeria_monocytogenes_1639_v166	Listeria_monocytogenes_1639_v153	0.9583
Listeria_monocytogenes_1639_v165	Listeria_monocytogenes_1639_v153	0.9563
Listeria_monocytogenes_1639_v164	Listeria_monocytogenes_1639_v153	0.9381
Listeria_monocytogenes_1639_v163	Listeria_monocytogenes_1639_v153	0.9458
Listeria_monocytogenes_1639_v162	Listeria_monocytogenes_1639_v153	0.9381
Listeria_monocytogenes_1639_v161	Listeria_monocytogenes_1639_v153	0.9392
Listeria_monocytogenes_1639_v160	Listeria_monocytogenes_1639_v153	0.9393
Listeria_monocytogenes_1639_v15	Listeria_monocytogenes_1639_v153	0.9265
Listeria_monocytogenes_1639_v159	Listeria_monocytogenes_1639_v153	0.9421
Listeria_monocytogenes_1639_v158	Listeria_monocytogenes_1639_v153	0.9389
Listeria_monocytogenes_1639_v157	Listeria_monocytogenes_1639_v153	0.9389
Listeria_monocytogenes_1639_v156	Listeria_monocytogenes_1639_v153	0.9543
Listeria_monocytogenes_1639_v155	Listeria_monocytogenes_1639_v153	0.9824
Listeria_monocytogenes_1639_v154	Listeria_monocytogenes_1639_v153	0.9534
Listeria_monocytogenes_1639_v153	Listeria_monocytogenes_1639_v153	0.0677
Listeria_welshimeri_1643	Listeria_monocytogenes_1639_v154	0.8816
Listeria_welshimeri_serovar_6b_str_SLCC5334_386043	Listeria_monocytogenes_1639_v154	0.881
Listeria_seeligeri_serovar_1_2b_str_SLCC3954_683837	Listeria_monocytogenes_1639_v154	0.8862
Listeria_seeligeri_FSL_S4-171_702452	Listeria_monocytogenes_1639_v154	0.8183
Listeria_seeligeri_FSL_N1-067_702453	Listeria_monocytogenes_1639_v154	0.8367
Listeria_seeligeri_1640_v1	Listeria_monocytogenes_1639_v154	0.8842
Listeria_seeligeri_1640	Listeria_monocytogenes_1639_v154	0.8793
Listeria_monocytogenes_WSLC1042_1457188	Listeria_monocytogenes_1639_v154	0.9345
Listeria_monocytogenes_WSLC1001_1457187	Listeria_monocytogenes_1639_v154	0.9678
Listeria_monocytogenes_SLCC7179_879090	Listeria_monocytogenes_1639_v154	0.9625
Listeria_monocytogenes_SLCC5850_882096	Listeria_monocytogenes_1639_v154	0.9699
Listeria_monocytogenes_SLCC2755_932919	Listeria_monocytogenes_1639_v154	0.9339
Listeria_monocytogenes_SLCC2540_879089	Listeria_monocytogenes_1639_v154	0.9507
Listeria_monocytogenes_SLCC2479_882020	Listeria_monocytogenes_1639_v154	0.9574
Listeria_monocytogenes_SLCC2378_879088	Listeria_monocytogenes_1639_v154	0.9359
Listeria_monocytogenes_SLCC2376_882097	Listeria_monocytogenes_1639_v154	0.9406
Listeria_monocytogenes_SLCC2372_932920	Listeria_monocytogenes_1639_v154	0.9499
Listeria_monocytogenes_serotype_4b_str_LL195_1230340	Listeria_monocytogenes_1639_v154	0.9417
Listeria_monocytogenes_serotype_4b_str_LIS0087_1134669	Listeria_monocytogenes_1639_v154	0.9446
Listeria_monocytogenes_serotype_4b_str_F2365_265669	Listeria_monocytogenes_1639_v154	0.9417
Listeria_monocytogenes_serotype_4b_str_CLIP_80459_568819	Listeria_monocytogenes_1639_v154	0.9485
Listeria_monocytogenes_serotype_4b_str_81-0861_1196161	Listeria_monocytogenes_1639_v154	0.9265
Listeria_monocytogenes_serotype_4b_str_81-0592_1196160	Listeria_monocytogenes_1639_v154	0.9275
Listeria_monocytogenes_serotype_4b_str_81-0558_1196159	Listeria_monocytogenes_1639_v154	0.9282
Listeria_monocytogenes_serotype_4b_str_10-0809_1196162	Listeria_monocytogenes_1639_v154	0.9275
Listeria_monocytogenes_serotype_4b_str_02-6680_1196175	Listeria_monocytogenes_1639_v154	0.9448
Listeria_monocytogenes_serotype_4b_str_02-6679_1196174	Listeria_monocytogenes_1639_v154	0.9448
Listeria_monocytogenes_serotype_4b_str_02-1792_1196173	Listeria_monocytogenes_1639_v154	0.929
Listeria_monocytogenes_serotype_4b_str_02-1289_1196172	Listeria_monocytogenes_1639_v154	0.929
Listeria_monocytogenes_serotype_4b_str_02-1103_1196171	Listeria_monocytogenes_1639_v154	0.929
Listeria_monocytogenes_serotype_3c_str_10-5027_1196195	Listeria_monocytogenes_1639_v154	0.9623
Listeria_monocytogenes_serotype_1_2c_str_10-5026_1196194	Listeria_monocytogenes_1639_v154	0.9623
Listeria_monocytogenes_serotype_1_2c_str_10-5025_1196193	Listeria_monocytogenes_1639_v154	0.9623
Listeria_monocytogenes_serotype_1_2b_str_10-0811_1196164	Listeria_monocytogenes_1639_v154	0.946
Listeria_monocytogenes_serotype_1_2b_str_10-0810_1196163	Listeria_monocytogenes_1639_v154	0.946
Listeria_monocytogenes_serotype_1_2a_str_99-6370_1196179	Listeria_monocytogenes_1639_v154	0.9688
Listeria_monocytogenes_serotype_1_2a_str_98-2035_1196178	Listeria_monocytogenes_1639_v154	0.9688
Listeria_monocytogenes_serotype_1_2a_str_95-0093_1196177	Listeria_monocytogenes_1639_v154	0.9746
Listeria_monocytogenes_serotype_1_2a_str_88-0478_1196176	Listeria_monocytogenes_1639_v154	0.9723
Listeria_monocytogenes_serotype_1_2a_str_10-5024_1196192	Listeria_monocytogenes_1639_v154	0.9749
Listeria_monocytogenes_serotype_1_2a_str_10-4758_1196168	Listeria_monocytogenes_1639_v154	0.968
Listeria_monocytogenes_serotype_1_2a_str_10-4754_1196167	Listeria_monocytogenes_1639_v154	0.968
Listeria_monocytogenes_serotype_1_2a_str_10-1321_1196191	Listeria_monocytogenes_1639_v154	0.9749
Listeria_monocytogenes_serotype_1_2a_str_10-1047_1196190	Listeria_monocytogenes_1639_v154	0.9753
Listeria_monocytogenes_serotype_1_2a_str_10-1046_1196189	Listeria_monocytogenes_1639_v154	0.9753
Listeria_monocytogenes_serotype_1_2a_str_10-0934_1196170	Listeria_monocytogenes_1639_v154	0.9688
Listeria_monocytogenes_serotype_1_2a_str_10-0933_1196169	Listeria_monocytogenes_1639_v154	0.9688
Listeria_monocytogenes_serotype_1_2a_str_10-0815_1196188	Listeria_monocytogenes_1639_v154	0.9698
Listeria_monocytogenes_serotype_1_2a_str_10-0814_1196187	Listeria_monocytogenes_1639_v154	0.9746
Listeria_monocytogenes_serotype_1_2a_str_10-0813_1196166	Listeria_monocytogenes_1639_v154	0.9664
Listeria_monocytogenes_serotype_1_2a_str_10-0812_1196165	Listeria_monocytogenes_1639_v154	0.9664
Listeria_monocytogenes_serotype_1_2a_str_08-7669_1196186	Listeria_monocytogenes_1639_v154	0.969
Listeria_monocytogenes_serotype_1_2a_str_08-7374_1196185	Listeria_monocytogenes_1639_v154	0.9749
Listeria_monocytogenes_serotype_1_2a_str_08-6997_1196184	Listeria_monocytogenes_1639_v154	0.9698
Listeria_monocytogenes_serotype_1_2a_str_08-6569_1196183	Listeria_monocytogenes_1639_v154	0.9698
Listeria_monocytogenes_serotype_1_2a_str_08-6056_1196182	Listeria_monocytogenes_1639_v154	0.9698
Listeria_monocytogenes_serotype_1_2a_str_04-5457_1196181	Listeria_monocytogenes_1639_v154	0.9749
Listeria_monocytogenes_serotype_1_2a_str_02-5993_1196180	Listeria_monocytogenes_1639_v154	0.9746
Listeria_monocytogenes_R479a_1437838	Listeria_monocytogenes_1639_v154	0.9761
Listeria_monocytogenes_N53-1_1234142	Listeria_monocytogenes_1639_v154	0.906
Listeria_monocytogenes_M7_1030009	Listeria_monocytogenes_1639_v154	0.9107
Listeria_monocytogenes_LO28_393132	Listeria_monocytogenes_1639_v154	0.9568
Listeria_monocytogenes_L99_563174	Listeria_monocytogenes_1639_v154	0.9114
Listeria_monocytogenes_L312_882094	Listeria_monocytogenes_1639_v154	0.9485
Listeria_monocytogenes_J1816_930781	Listeria_monocytogenes_1639_v154	0.9278
Listeria_monocytogenes_J1-220_930782	Listeria_monocytogenes_1639_v154	0.9315
Listeria_monocytogenes_J0161_393130	Listeria_monocytogenes_1639_v154	0.9477
Listeria_monocytogenes_HCC23_552536	Listeria_monocytogenes_1639_v154	0.9107

Listeria_monocytogenes_FSL_R2-561_393126	Listeria_monocytogenes_1639_v154	0.9609
Listeria_monocytogenes_FSL_J1-208_393119	Listeria_monocytogenes_1639_v154	0.9043
Listeria_monocytogenes_FSL_J1-175_393118	Listeria_monocytogenes_1639_v154	0.952
Listeria_monocytogenes_Finland_1998_393127	Listeria_monocytogenes_1639_v154	0.9691
Listeria_monocytogenes_EGD-e_169963_v1	Listeria_monocytogenes_1639_v154	0.9536
Listeria_monocytogenes_EGD-e_169963	Listeria_monocytogenes_1639_v154	0.9536
Listeria_monocytogenes_EGD_1334565	Listeria_monocytogenes_1639_v154	0.9706
Listeria_monocytogenes_CFSAN002349_1299895	Listeria_monocytogenes_1639_v154	0.9452
Listeria_monocytogenes_ATCC_19117_882095_v1	Listeria_monocytogenes_1639_v154	0.9396
Listeria_monocytogenes_ATCC_19117_882095	Listeria_monocytogenes_1639_v154	0.9396
Listeria_monocytogenes_6179_1288295	Listeria_monocytogenes_1639_v154	0.9455
Listeria_monocytogenes_1639_v9	Listeria_monocytogenes_1639_v154	0.926
Listeria_monocytogenes_1639_v99	Listeria_monocytogenes_1639_v154	0.9278
Listeria_monocytogenes_1639_v98	Listeria_monocytogenes_1639_v154	0.9435
Listeria_monocytogenes_1639_v97	Listeria_monocytogenes_1639_v154	0.933
Listeria_monocytogenes_1639_v96	Listeria_monocytogenes_1639_v154	0.9275
[truncated: 2,156,027 more chars]
