# Supplementary material for: Comparative Genomics Reveal the Utilization Ability of Variable Carbohydrates as Key Genetic Features of Listeria Pathogens in Their Pathogenic Lifestyles
Source: Pathogens. 2022 Nov 28;11(12):1430. doi: 10.3390/pathogens11121430 (PMC9784484; doi:10.3390/pathogens11121430)
Supplement: Supplementary file 1 [file pathogens-11-01430-s001.zip › pathogens-1990707-supplementary/Supplymentary Figures S1.pdf]

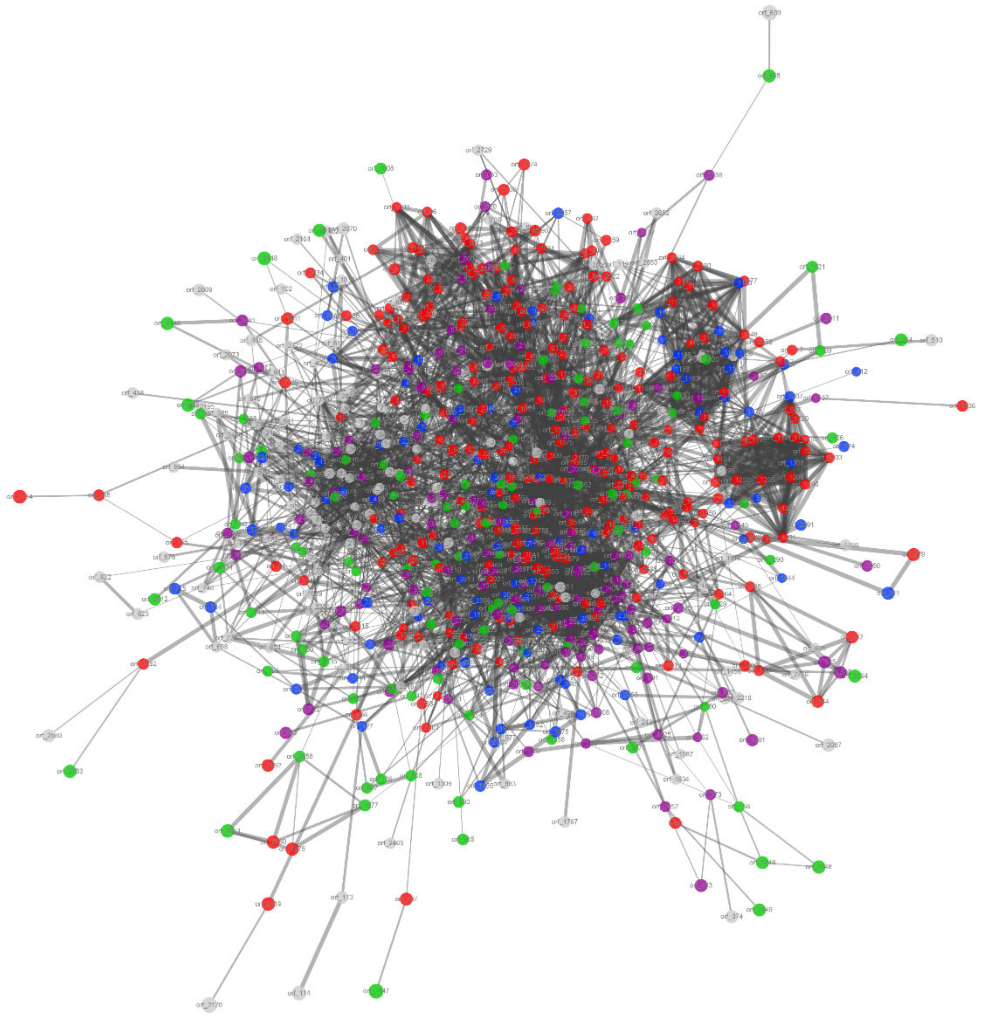

Figure S1. The protein-protein interaction network analysis of UGLP. The PPI network of UGLP referenced with *L. monocytogenes* EGD-e 169963 and the names of nodes corresponded to Table S6. Only those networks with number of nodes greater than 3 were shown in the graph. The edges of network represented the relationship of protein-protein interaction, and their line thickness represented the confidence of the relationship between nodes. Different colors of nodes reflected the different protein function categories: the red represented the basic substance transport and metabolism; the purple represented the genetic information processing, including replication, transcription, and translation; the blue represented the cellular processes, including cell wall/membrane/envelope biogenesis and cell motility; the green represented the bacteria-environment interaction, including signal transduction, extracellular structures, and defense mechanism; the grey represented the protein without meaningful function annotation.
