# Supplementary material for: Comparative Genomics Reveal the Utilization Ability of Variable Carbohydrates as Key Genetic Features of Listeria Pathogens in Their Pathogenic Lifestyles
Source: Pathogens. 2022 Nov 28;11(12):1430. doi: 10.3390/pathogens11121430 (PMC9784484; doi:10.3390/pathogens11121430)
Supplement: Supplementary file 1 [file pathogens-11-01430-s001.zip › pathogens-1990707-supplementary/Supplymentary Tables S1.pdf]

Table S1. Genomic details of *Listeria* strains used in the present study.

Sample_accession	species	Organism	Accession_number	Genome size(bp)	GC content(%)	# of scaffold	# of_plasmid	CDS	rRNA	tRNA	geographic_location	collection_date	isolation_source	host
SAMEA3138260	L.welshimeri	L.welshimeri_serovar_6b_str_SLCC5334_386043	AM263198.1	2,814,130	36.35	1	0	2,781	18	66	NA	NA	NA	NA
SAMEA4534872	L.welshimeri	L.welshimeri_1643	LT906444.1	2,814,137	36.35	1	0	2,778	18	66	USA	1900	Decaying vegetation	Food
SAMEA2272547	L.seeligeri	L.seeligeri_serovar_1_2b_str_SLCC3954_683837	FN557490.1	2,797,636	37.38	1	0	2,688	18	67	NA	NA	NA	NA
SAMN01909079	L.seeligeri	L.seeligeri_FSL_S4_171_702452	CM001052.1	2,972,337	35.17	1	0	2,590	1	24	USA	2001	urban Environment	Environment
SAMN01909078	L.seeligeri	L.seeligeri_FSL_N1_067_702453	CM001051.1	3,140,155	35.48	1	0	2,779	2	25	USA	NA	Food processing plant	Environment
SAMN16409656	L.seeligeri	L.seeligeri_1640_str1	CP063071.1	2,927,941	37.32	1	1	2,846	18	67	Poland	2017	soil sample from dry pine forest	Environment
SAMN10662805	L.seeligeri	L.seeligeri_1640	CP034772.1	2,872,774	37.3	1	0	2,834	8	63	NA	2018	NA	NA
SAMN02743817	L.monocytogenes	L.monocytogenes_WSLC1042_1457188	CP007210.1	2,942,168	37.99	1	0	2,857	18	67	Germany	NA	NA	NA
SAMN02743815	L.monocytogenes	L.monocytogenes_WSLC1001_1457187	CP007160.1	2,951,235	37.96	1	0	2,912	18	67	Germany	NA	NA	NA
SAMEA2272571	L.monocytogenes	L.monocytogenes_SLCC7179_879090	FR733650.1	2,882,234	38.04	1	0	2,818	18	68	NA	NA	NA	NA
SAMEA2272672	L.monocytogenes	L.monocytogenes_SLCC5850_882096	FR733647.1	2,907,142	38.01	1	0	2,852	18	67	NA	NA	NA	NA
SAMEA2271969	L.monocytogenes	L.monocytogenes_SLCC2755_932919	FR733646.1	3,023,926	38.04	1	1	2,916	18	67	NA	NA	NA	NA
SAMEA2272785	L.monocytogenes	L.monocytogenes_SLCC2540_879089	FR733645.1	2,976,958	37.93	1	0	2,879	18	67	NA	NA	NA	NA
SAMEA2272506	L.monocytogenes	L.monocytogenes_SLCC2479_882020	FR733649.1	2,972,172	37.95	1	0	2,924	18	65	NA	NA	NA	NA
SAMEA2272689	L.monocytogenes	L.monocytogenes_SLCC2378_879088	FR733644.1	2,941,360	37.99	1	0	2,845	18	67	NA	NA	NA	NA
SAMEA2272177	L.monocytogenes	L.monocytogenes_SLCC2376_882097	FR733651.1	2,840,185	38.26	1	0	2,733	18	67	NA	NA	NA	NA
SAMEA2272627	L.monocytogenes	L.monocytogenes_SLCC2372_932920	FR733648.1	3,022,910	37.91	1	1	2,980	18	67	NA	NA	NA	NA
SAMEA2272544	L.monocytogenes	L.monocytogenes_serotype_4b_str_LL195_1230340	HF558398.1	2,904,662	38.04	1	0	2,808	18	67	NA	NA	NA	NA
SAMN01816209	L.monocytogenes	L.monocytogenes_serotype_4b_str_LIS0087_1134669	CP044429.1	2,843,285	37.95	1	0	2,804	15	66	USA	2011	Environment swab	Environment
SAMN02603980	L.monocytogenes	L.monocytogenes_serotype_4b_str_F2365_265669	AE017262.2	2,905,187	38.04	1	0	2,808	18	67	NA	NA	NA	NA
SAMEA2272134	L.monocytogenes	L.monocytogenes_serotype_4b_str_CLIP_80459_568819	FM242711.1	2,912,690	38.06	1	0	2,802	18	67	NA	NA	NA	NA
SAMN05771116	L.monocytogenes	L.monocytogenes_serotype_4b_str_81_0861_1196161	CP006874.1	2,974,403	37.96	1	0	2,912	15	58	Canada	1981	coleslaw	Food
SAMN02713778	L.monocytogenes	L.monocytogenes_serotype_4b_str_81_0592_1196160	CP007526.1	2,975,864	37.96	1	0	2,905	15	58	Canada	1981	fetal blood	Homo sapiens
SAMN02713777	L.monocytogenes	L.monocytogenes_serotype_4b_str_81_0558_1196159	CP007525.1	2,971,543	37.96	1	0	2,901	15	58	Canada	1981	CSF	Homo sapiens
SAMN07124730	L.monocytogenes	L.monocytogenes_serotype_4b_str_10_0809_1196162	CP007167.1	2,975,853	37.96	1	0	2,906	15	58	Canada	1981	stool	Homo sapiens
SAMN07124742	L.monocytogenes	L.monocytogenes_serotype_4b_str_02_6680_1196175	CP007462.1	2,908,004	38	1	0	2,813	15	58	Canada	2002	cheese	Environment
SAMN02944848	L.monocytogenes	L.monocytogenes_serotype_4b_str_02_6679_1196174	CP008821.1	2,908,004	38	1	0	2,813	15	58	Canada	2002	stool	Homo sapiens
SAMN07124741	L.monocytogenes	L.monocytogenes_serotype_4b_str_02_1792_1196173	CP007461.1	2,979,105	37.95	1	0	2,910	15	58	Canada	2002	cheese	Environment
SAMN07124740	L.monocytogenes	L.monocytogenes_serotype_4b_str_02_1289_1196172	CP007460.1	2,979,105	37.95	1	0	2,910	15	58	Canada	2002	stool	Homo sapiens
SAMN07124739	L.monocytogenes	L.monocytogenes_serotype_4b_str_02_1103_1196171	CP007459.1	2,979,105	37.95	1	0	2,910	15	58	Canada	2002	CSF	Homo sapiens
SAMN07124755	L.monocytogenes	L.monocytogenes_serotype_3c_str_10_5027_1196195	CP007196.1	2,965,327	37.94	1	0	2,931	15	58	Canada	2009	food processing	Environment
SAMN07124754	L.monocytogenes	L.monocytogenes_serotype_1_2c_str_10_5026_1196194	CP007195.1	2,967,496	37.94	1	0	2,927	15	58	Canada	2009	food processing	Environment
SAMN07124753	L.monocytogenes	L.monocytogenes_serotype_1_2c_str_10_5025_1196193	CP007194.1	2,967,523	37.94	1	0	2,927	15	58	Canada	2009	bacon flakes	Environment
SAMN07124732	L.monocytogenes	L.monocytogenes_serotype_1_2b_str_10_0811_1196164	CP007169.1	3,017,944	37.96	1	0	2,968	15	58	Canada	1996	imitation crab	Environment
SAMN07124731	L.monocytogenes	L.monocytogenes_serotype_1_2b_str_10_0810_1196163	CP007168.1	3,017,944	37.96	1	0	2,968	15	58	Canada	1996	stool	Homo sapiens
SAMN07124745	L.monocytogenes	L.monocytogenes_serotype_1_2a_str_99_6370_1196179	CP007021.1	3,032,209	37.96	1	0	3,006	15	58	Canada	1999	blood	Homo sapiens
SAMN07124744	L.monocytogenes	L.monocytogenes_serotype_1_2a_str_98_2035_1196178	CP007020.1	3,032,209	37.96	1	0	3,006	15	58	Canada	1998	blood	Homo sapiens
SAMN07124743	L.monocytogenes	L.monocytogenes_serotype_1_2a_str_95_0093_1196177	CP007019.1	3,000,273	37.96	1	0	2,960	15	58	Canada	1995	blood	Homo sapiens
SAMN05771118	L.monocytogenes	L.monocytogenes_serotype_1_2a_str_88_0478_1196176	CP006862.1	2,994,351	37.97	1	0	2,957	15	58	Canada	1988	blood	Homo sapiens
SAMN02725209	L.monocytogenes	L.monocytogenes_serotype_1_2a_str_10_5024_1196192	CP008837.1	2,999,326	37.97	1	0	2,959	15	58	Canada	2010	food	Food
SAMN07124736	L.monocytogenes	L.monocytogenes_serotype_1_2a_str_10_4758_1196168	CP007198.1	2,910,921	37.98	1	0	2,853	15	58	Canada	2002	cheese	Environment
SAMN07124735	L.monocytogenes	L.monocytogenes_serotype_1_2a_str_10_4754_1196167	CP007197.1	2,910,922	37.98	1	0	2,852	15	58	Canada	2002	CSF	Homo sapiens
SAMN07124752	L.monocytogenes	L.monocytogenes_serotype_1_2a_str_10_1321_1196191	CP007018.1	2,999,326	37.97	1	0	2,959	15	58	Canada	2010	blood	Homo sapiens
SAMN05771119	L.monocytogenes	L.monocytogenes_serotype_1_2a_str_10_1047_1196190	CP006861.1	2,998,206	37.97	1	0	2,956	15	58	Canada	2010	CSF	Homo sapiens
SAMN07124751	L.monocytogenes	L.monocytogenes_serotype_1_2a_str_10_1046_1196189	CP007017.1	2,998,206	37.97	1	0	2,956	15	58	Canada	2010	blood	Homo sapiens
SAMN07124738	L.monocytogenes	L.monocytogenes_serotype_1_2a_str_10_0934_1196170	CP007200.1	2,870,277	38.02	1	0	2,785	15	58	Canada	2008	cheese	Environment
SAMN07124737	L.monocytogenes	L.monocytogenes_serotype_1_2a_str_10_0933_1196169	CP007199.1	2,870,277	38.02	1	0	2,785	15	58	Canada	2008	blood	Homo sapiens
SAMN05771120	L.monocytogenes	L.monocytogenes_serotype_1_2a_str_10_0815_1196188	CP006860.1	3,032,624	37.96	1	0	3,005	15	58	Canada	2008	meat	Meat
SAMN02873948	L.monocytogenes	L.monocytogenes_serotype_1_2a_str_10_0814_1196187	CP008836.1	2,999,305	37.97	1	0	2,960	15	58	Canada	2010	meat	Meat
SAMN07124734	L.monocytogenes	L.monocytogenes_serotype_1_2a_str_10_0813_1196166	CP007171.1	2,942,234	37.96	1	0	2,895	15	58	Canada	2000	blood	Homo sapiens
SAMN07124733	L.monocytogenes	L.monocytogenes_serotype_1_2a_str_10_0812_1196165	CP007170.1	2,942,234	37.96	1	0	2,895	15	58	Canada	2000	Whipping cream	Environment
SAMN07124750	L.monocytogenes	L.monocytogenes_serotype_1_2a_str_08_7669_1196186	CP007011.1	2,954,577	38.01	1	0	2,893	15	58	Canada	2008	blood	Homo sapiens
SAMN07124749	L.monocytogenes	L.monocytogenes_serotype_1_2a_str_08_7374_1196185	CP007010.1	2,999,326	37.97	1	0	2,959	15	58	Canada	2008	meat	Environment
SAMN05771121	L.monocytogenes	L.monocytogenes_serotype_1_2a_str_08_6997_1196184	CP006859.1	3,032,624	37.96	1	0	3,005	15	58	Canada	2008	blood	Homo sapiens
SAMN05771122	L.monocytogenes	L.monocytogenes_serotype_1_2a_str_08_6569_1196183	CP006858.1	3,032,624	37.96	1	0	3,005	15	58	Canada	2008	food processing	Environment
SAMN07124748	L.monocytogenes	L.monocytogenes_serotype_1_2a_str_08_6056_1196182	CP007009.1	3,032,624	37.96	1	0	3,005	15	58	Canada	2008	turkey meat	Environment
SAMN07124747	L.monocytogenes	L.monocytogenes_serotype_1_2a_str_04_5457_1196181	CP007008.1	2,999,326	37.97	1	0	2,959	15	58	Canada	2004	blood	Homo sapiens
SAMN07124746	L.monocytogenes	L.monocytogenes_serotype_1_2a_str_02_5993_1196180	CP007007.1	2,999,248	37.97	1	0	2,960	15	58	Canada	2002	blood	Homo sapiens
SAMEA3138991	L.monocytogenes	L.monocytogenes_R479a_1437838	HG813247.1	3,031,650	37.91	1	1	2,984	15	58	NA	NA	NA	NA
SAMEA2271966	L.monocytogenes	L.monocytogenes_N53_1_1234142	HE999705.1	2,776,847	38.1	1	0	3,323	18	67	NA	NA	NA	NA
SAMN02604342	L.monocytogenes	L.monocytogenes_M7_1030009	CP002816.1	2,976,163	38.19	1	0	2,959	18	67	NA	NA	NA	NA
SAMN16205177	L.monocytogenes	L.monocytogenes_LO28_393132	CP061814.1	2,975,254	37.96	1	0	2,933	18	67	Netherlands	2004	feces of a pregnant carrier	Homo sapiens
SAMEA2272406	L.monocytogenes	L.monocytogenes_L99_563174	FM211688.1	2,979,198	38.19	1	0	2,954	18	67	NA	NA	NA	NA
SAMEA2272305	L.monocytogenes	L.monocytogenes_L312_882094	FR733642.2	2,912,346	38.06	1	0	2,802	18	67	NA	NA	NA	NA
SAMN01813900	L.monocytogenes	L.monocytogenes_J1816_930781	CP006047.2	2,947,460	37.97	1	0	2,956	15	58	USA	NA	food processing plant	Environment
SAMN01816124	L.monocytogenes	L.monocytogenes_J1_220_930782	CP006046.4	3,032,269	37.94	1	0	2,977	18	67	USA	NA	NA	Homo sapiens
SAMN00769971	L.monocytogenes	L.monocytogenes_J0161_393130	CP002001.1	3,000,464	37.86	1	0	2,947	15	59	NA	NA	NA	NA
SAMN02603154	L.monocytogenes	L.monocytogenes_HCC23_552536	CP001175.1	2,976,212	38.19	1	0	2,961	18	67	NA	NA	NA	NA
SAMN00013319	L.monocytogenes	L.monocytogenes_FSL_R2_561_393126	CP002003.1	2,973,801	37.96	1	0	2,939	18	67	NA	NA	NA	NA
SAMN16231353	L.monocytogenes	L.monocytogenes_FSL_J1_208_393119	CP062127.1	2,859,299	37.9	1	1	2,799	18	67	USA	1998	NA	Goat
SAMN16231352	L.monocytogenes	L.monocytogenes_FSL_J1_175_393118	CP062129.1	2,957,228	38.01	1	0	2,860	18	67	NA	NA	water	Environment
SAMN00012880	L.monocytogenes	L.monocytogenes_Finland_1998_393127	CP002004.1	2,874,431	38.05	1	0	2,786	18	67	NA	NA	NA	NA
SAMN07763702	L.monocytogenes	L.monocytogenes_EGD_e_169963_str1	CP023861.1	2,944,523	37.98	1	0	2,877	18	67	United Kingdom	1924	rabbit tissue	Rabbit
SAMEA3138329	L.monocytogenes	L.monocytogenes_EGD_e_169963	AL591824.1	2,944,528	37.98	1	0	2,875	18	67	NA	NA	NA	NA
SAMEA3138952	L.monocytogenes	L.monocytogenes_EGD_1334565	HG421741.1	2,907,193	38	1	0	2,848	18	67	NA	NA	NA	NA
SAMN01939119	L.monocytogenes	L.monocytogenes_CFSAN002349_1299895	CP045751.1	2,942,451	37.97	1	0	2,846	19	63	USA	2011	organic baby spinach	Food
SAMN04286631	L.monocytogenes	L.monocytogenes_ATCC_19117_882095_str1	CP013288.1	2,950,820	37.99	1	0	2,844	18	67	USA	1961	USA	Sheep
SAMEA2271997	L.monocytogenes	L.monocytogenes_ATCC_19117_882095	FR733643.1	2,951,805	37.99	1	0	2,844	18	67	NA	NA	NA	NA
SAMEA3138937	L.monocytogenes	L.monocytogenes_6179_1288295	HG813249.1	3,072,826	37.86	1	1	3,041	12	49	NA	NA	NA	NA
SAMN06297509	L.monocytogenes	L.monocytogenes_1639_str9	CP019615.1	3,072,093	37.91	1	0	3,080	17	66	NA	2007	Sprouts	Food
SAMN14928102	L.monocytogenes	L.monocytogenes_1639_str99	CP053632.1	3,009,249	37.94	1	1	2,953	18	67	USA	2005	food	Food
SAMN14261396	L.monocytogenes	L.monocytogenes_1639_str98	CP075876.1	2,953,613	37.9	1	0	2,948	6	63	Norway	2007	Environment	Environment
SAMN14896270	L.monocytogenes	L.monocytogenes_1639_str97	CP053357.1	2,953,624	37.94	1	0	2,891	15	57	Italy	2017	radicchio	Food
SAMN02203130	L.monocytogenes	L.monocytogenes_1639_str96	CP006600.1	3,009,511	37.94	1	1	2,956	18	67	USA	2002	food	Food
SAMN02203129	L.monocytogenes	L.monocytogenes_1639_str95	CP006599.1	3,009,520	37.94	1	1	2,957	18	67	USA	2002	Environment	Environment
SAMN02203125	L.monocytogenes	L.monocytogenes_1639_str94	CP006598.1	3,009,523	37.94	1	1	2,955	18	67	USA	2002	NA	Homo sapiens
SAMN02990220	L.monocytogenes	L.monocytogenes_1639_str93	CP020828.1	3,020,953	37.89	1	1	2,985	18	67	USA	2014	barbecue pork	Meat
SAMN14928089	L.monocytogenes	L.monocytogenes_1639_str92	CP053630.1	3,009,759	37.94	1	1	2,950	18	67	USA	2004	food	Food
SAMN08177723	L.monocytogenes	L.monocytogenes_1639_str91	CP031674.1	2,954,075	37.87	1	0	2,934	8	56	India	2016	Vaginal swab of Female	Homo sapiens
SAMN14261395	L.monocytogenes	L.monocytogenes_1639_str90	CP075875.1	2,954,315	37.9	1	0	2,948	5	63	Norway	2007	Environment	Environment
SAMN14260851	L.monocytogenes	L.monocytogenes_1639_str8	CP050029.1	3,080,194	37.87	1	0	3,044	4	65	Norway	2015	Environment	Environment
SAMN25653830	L.monocytogenes	L.monocytogenes_1639_str89	CP092059.1	2,957,755	38.01	1	0	2,868	18	67	Canada	2017	Sweet Kale Veg Salad Kit	NA
SAMN02203124	L.monocytogenes	L.monocytogenes_1639_str88	CP006593.1	2,958,908	37.97	1	0	2,916	18	67	USA	1996	NA	Homo sapiens
SAMN14260762	L.monocytogenes	L.monocytogenes_1639_str87	CP050129.1	2,959,738	37.85	1	0	2,966	7	65	Norway	2015	Environment	Environment
SAMN14260728	L.monocytogenes	L.monocytogenes_1639_str86	CP050026.1	2,959,878	37.85	1	0	2,968	7	65	Norway	2015	Environment	Environment
SAMN14260686	L.monocytogenes	L.monocytogenes_1639_str85	CP050023.1	2,960,280	37.87	1	0	2,917	7	65	Norway	2015	Environment	Environment
SAMN16357249	L.monocytogenes	L.monocytogenes_1639_str84	CP068150.1	2,963,718	37.99	1	0	2,932	18	67	Germany	NA	NA	NA
SAMN14260726	L.monocytogenes	L.monocytogenes_1639_str83	CP050025.1	2,964,050	37.87	1	0	2,907	5	65	Norway	2015	Environment	Environment
SAMN14260707	L.monocytogenes	L.monocytogenes_1639_str82	CP050024.1	2,964,472	37.87	1	0	2,911	6	65	Norway	2015	Environment	Environment
SAMN13191637	L.monocytogenes	L.monocytogenes_1639_str81	CP045969.1	2,964,538	37.98	1	0	2,904	18	67	Australia	2013	NA	Homo sapiens
SAMN06297604	L.monocytogenes	L.monocytogenes_1639_str80	CP019624.1	2,965,658	37.97	1	0	2,903	15	68	NA	2009	fermented meat	Meat
SAMN14260850	L.monocytogenes	L.monocytogenes_1639_str7	CP050028.1	3,081,000	37.87	1	0	3,046	6	65	Norway	2015	Environment	Environment
SAMN06625636	L.monocytogenes	L.monocytogenes_1639_str79	CP021174.1	2,965,849	37.97	1	0	2,915	18	67	South Korea	2012	Tuna Kimchi Gimbap (a type of Korean food)	Food
SAMN03285109	L.monocytogenes	L.monocytogenes_1639_str78	CP020827.1	2,966,603	37.96	1	0	2,904	18	67	USA	2014	Environment	Environment
SAMN06297526	L.monocytogenes	L.monocytogenes_1639_str77	CP019621.1	2,966,818	37.85	1	0	2,899	16	67	NA	2007	Baby spinach	Food
SAMN06297508	L.monocytogenes	L.monocytogenes_1639_str76	CP019614.1	2,972,553	37.95	1	0	2,953	16	66	NA	2006	chicken breast	Meat
SAMN13973456	L.monocytogenes	L.monocytogenes_1639_str75	CP048401.1	2,973,626	38.17	1	0	2,955	15	58	Russia	1953	NA	Pig
SAMN14260764	L.monocytogenes	L.monocytogenes_1639_str74	CP050027.1	2,973,786	37.92	1	0	2,933	5	63	Norway	2007	Environment	Environment
SAMN08056486	L.monocytogenes	L.monocytogenes_1639_str73	CP025082.1	3,032,635	37.94	1	1	2,984	18	67	Norway	2012	meat processing facility	Environment
SAMN06297524	L.monocytogenes	L.monocytogenes_1639_str72	CP019619.1	2,974,450	37.98	1	0	2,906	11	58	NA	2006	Environment swab	Environment
SAMN03354355	L.monocytogenes	L.monocytogenes_1639_str71	CP020833.1	3,057,928	37.92	1	1	2,993	18	68	USA	NA	wiener sausage	Food
SAMN08125516	L.monocytogenes	L.monocytogenes_1639_str70	CP025567.1	2,994,824	37.94	1	1	2,951	18	67	NA	NA	NA	NA
SAMN14260765	L.monocytogenes	L.monocytogenes_1639_str6	CP075872.1	3,081,615	37.87	1	0	3,046	5	65	Norway	2015	Environment	Environment
SAMN02203123	L.monocytogenes	L.monocytogenes_1639_str69	CP006596.2	2,981,886	37.98	1	0	2,908	18	67	Canada	1981	coleslaw	NA
SAMN14260853	L.monocytogenes	L.monocytogenes_1639_str68	CP050030.1	2,982,026	37.78	1	0	2,914	6	65	Norway	2015	Environment	Environment
SAMN04884549	L.monocytogenes	L.monocytogenes_1639_str67	CP015593.1	2,982,685	38	1	0	2,910	18	67	China	2008	blood	Homo sapiens
SAMN11311853	L.monocytogenes	L.monocytogenes_1639_str66	CP038642.1	2,991,072	37.93	1	1	2,906	18	67	Switzerland	2012	blood	Homo sapiens
SAMEA104485072	L.monocytogenes	L.monocytogenes_1639_str65	CP063381.1	2,986,724	37.95	1	0	2,951	18	67	Germany	2012	human listeriosis	Homo sapiens
SAMN08125518	L.monocytogenes	L.monocytogenes_1639_str64	CP025221.1	2,987,434	37.93	1	0	2,920	18	67	NA	NA	NA	NA
SAMN02666871	L.monocytogenes	L.monocytogenes_1639_str63	CP014250.2	3,043,081	37.97	1	1	2,968	18	67	USA	2014	cheese	Food
SAMN02203127	L.monocytogenes	L.monocytogenes_1639_str62	CP006591.1	2,988,947	37.97	1	0	2,936	18	67	USA	1999	food	Food
SAMN03003922	L.monocytogenes	L.monocytogenes_1639_str61	CP009258.1	2,989,591	38	1	0	2,943	18	67	Switzerland	2006	blood	Homo sapiens
SAMN06297521	L.monocytogenes	L.monocytogenes_1639_str60	CP019617.1	2,989,685	37.86	1	0	2,953	15	58	NA	2008	Environment swab	Environment
SAMN02923161	L.monocytogenes	L.monocytogenes_1639_str5	CP022020.1	3,238,513	38	1	1	3,238	18	67	USA	2011	Environment swab	Environment
SAMN05291441	L.monocytogenes	L.monocytogenes_1639_str59	CP018149.2	2,989,866	37.95	1	0	2,903	18	67	Russia	2005	Aborted fetus	Homo sapiens
SAMN10346771	L.monocytogenes	L.monocytogenes_1639_str58	CP041014.1	2,992,056	37.95	1	0	2,904	18	67	USA	NA	Sheep isolate, USA	Sheep
SAMEA5041142	L.monocytogenes	L.monocytogenes_1639_str57	CP063383.1	2,996,964	37.98	1	1	2,961	18	67	Germany	2018	human listeriosis	Homo sapiens
SAMN08663440	L.monocytogenes	L.monocytogenes_1639_str56	CP028333.1	2,994,410	37.97	1	0	2,906	18	67	Denmark	2015	smoked fish	NA
SAMN14928072	L.monocytogenes	L.monocytogenes_1639_str55	CP053628.1	3,050,429	37.92	1	1	3,022	18	67	USA	2003	food	Food
SAMN02730097	L.monocytogenes	L.monocytogenes_1639_str54	CP007583.1	2,994,702	37.95	1	0	2,921	18	67	China	2011	NA	Goat
SAMN08125517	L.monocytogenes	L.monocytogenes_1639_str53	CP025222.1	2,995,531	37.95	1	0	2,918	18	67	NA	NA	NA	NA
SAMEA104485140	L.monocytogenes	L.monocytogenes_1639_str52	CP063382.1	2,995,602	37.98	1	0	2,935	18	67	Germany	2015	human listeriosis	Homo sapiens
SAMN08125519	L.monocytogenes	L.monocytogenes_1639_str51	CP025565.1	3,024,914	37.95	1	1	2,946	18	67	NA	NA	NA	NA
SAMN06297522	L.monocytogenes	L.monocytogenes_1639_str50	CP019618.1	2,996,793	37.99	1	0	2,959	13	66	NA	2002	Environment sponge	Environment
SAMN02923159	L.monocytogenes	L.monocytogenes_1639_str4	CP023052.1	3,148,164	38.01	1	1	3,105	18	67	USA	2011	Environment swab	Environment
SAMN10434278	L.monocytogenes	L.monocytogenes_1639_str49	CP071154.1	2,998,940	38.04	1	0	2,912	18	67	China	2012	food	Food
SAMN06920120	L.monocytogenes	L.monocytogenes_1639_str48	CP021325.1	3,002,491	37.92	1	0	2,973	15	58	China	2007	market	NA
SAMN02867555	L.monocytogenes	L.monocytogenes_1639_str47	CP019164.1	3,003,994	37.99	1	0	2,963	18	67	Canada	1994	Food Processing Facility	Environment
SAMN13191636	L.monocytogenes	L.monocytogenes_1639_str46	CP045970.1	3,007,802	37.88	1	1	2,913	18	67	Australia	2009	NA	Homo sapiens
SAMN02867583	L.monocytogenes	L.monocytogenes_1639_str45	CP019165.1	3,082,433	37.96	1	1	3,035	18	67	Canada	2008	Beef Deli Slices	Meat
SAMN04277002	L.monocytogenes	L.monocytogenes_1639_str44	CP019170.1	3,005,348	37.99	1	0	2,959	18	67	Canada	2008	blood	Homo sapiens
SAMN03160826	L.monocytogenes	L.monocytogenes_1639_str43	CP018685.1	3,005,352	37.99	1	0	2,959	18	67	NA	NA	food	Food
SAMN14261962	L.monocytogenes	L.monocytogenes_1639_str42	CP075877.1	3,005,957	37.86	1	0	2,999	6	63	Norway	2007	Environment	Environment
SAMN23386451	L.monocytogenes	L.monocytogenes_1639_str41	CP090052.1	3,070,976	37.95	1	1	3,102	18	68	USA	1998	Salmon processing facility	Environment
SAMN08056483	L.monocytogenes	L.monocytogenes_1639_str40	CP025443.1	3,035,873	37.95	1	1	2,967	18	67	Norway	2012	meat processing facility	Environment
SAMN06339459	L.monocytogenes	L.monocytogenes_1639_str3	CP023050.1	3,148,183	38.01	1	1	3,104	18	67	USA	2017	Environment swab	Environment
SAMN13397725	L.monocytogenes	L.monocytogenes_1639_str39	CP046478.1	3,011,126	37.97	1	0	2,946	18	67	China	2015	NA	Meat
SAMN18537091	L.monocytogenes	L.monocytogenes_1639_str38	CP074104.1	3,011,469	37.9	1	0	2,946	18	67	South Korea	2017	tissue	Goat
SAMN14364188	L.monocytogenes	L.monocytogenes_1639_str37	CP058256.1	3,013,751	38.04	1	0	2,979	18	67	South Africa	2018	food	NA
SAMN08125520	L.monocytogenes	L.monocytogenes_1639_str36	CP025560.1	3,072,784	37.89	1	1	3,001	18	67	NA	NA	NA	Food
SAMN08056484	L.monocytogenes	L.monocytogenes_1639_str35	CP025442.1	3,016,037	37.95	1	0	2,946	18	67	Norway	2012	meat processing facility	Environment
SAMN14260901	L.monocytogenes	L.monocytogenes_1639_str34	CP075874.1	3,017,780	37.74	1	0	2,963	4	65	Norway	2007	Environment	Environment
SAMEA5851829	L.monocytogenes	L.monocytogenes_1639_str33	LR698978.1	3,017,944	37.96	1	0	2,968	15	58	NA	NA	human gut	Homo sapiens
SAMN03579634	L.monocytogenes	L.monocytogenes_1639_str32	CP011345.1	3,018,390	38.04	1	0	2,988	18	67	NA	NA	NA	NA
SAMN14260852	L.monocytogenes	L.monocytogenes_1639_str31	CP075873.1	3,019,084	37.75	1	0	2,964	6	65	Norway	2007	Environment	Environment
SAMN10148433	L.monocytogenes	L.monocytogenes_1639_str30	CP032671.1	3,019,461	37.96	1	0	2,944	18	67	USA	2014	cervicoNAvaginal swab	Cattle
SAMN02203128	L.monocytogenes	L.monocytogenes_1639_str2	CP006597.1	3,243,301	37.97	1	1	3,222	18	67	USA	NA	Environment	Environment
SAMN08125515	L.monocytogenes	L.monocytogenes_1639_str29	CP025568.1	3,085,336	37.99	1	1	3,038	18	67	NA	NA	NA	NA
SAMN07718436	L.monocytogenes	L.monocytogenes_1639_str28	CP023754.1	3,027,995	38.02	1	0	2,984	18	67	Finland	1998	Equipment	Environment
SAMN08056487	L.monocytogenes	L.monocytogenes_1639_str27	CP025438.1	3,093,572	37.98	1	1	3,061	18	67	Norway	2012	meat processing facility	Environment
SAMN08056488	L.monocytogenes	L.monocytogenes_1639_str26	CP025440.1	3,093,763	37.98	1	1	3,065	18	67	Norway	2012	meat processing facility	Environment
SAMN08056485	L.monocytogenes	L.monocytogenes_1639_str25	CP025259.1	3,093,884	37.98	1	1	3,064	18	67	Norway	2012	meat processing facility	Environment
SAMN07763686	L.monocytogenes	L.monocytogenes_1639_str24	CP023862.1	3,030,813	37.93	1	0	2,971	18	67	USA	1983	Massachusetts listeriosis outbreak	Homo sapiens
SAMN02743864	L.monocytogenes	L.monocytogenes_1639_str23	CP020022.1	3,030,817	37.93	1	0	2,973	18	67	USA	1983	clinical	NA
SAMN03386937	L.monocytogenes	L.monocytogenes_1639_str22	CP016213.2	3,030,827	37.96	1	0	2,983	18	67	USA	2015	distribution center	Environment
SAMN08125521	L.monocytogenes	L.monocytogenes_1639_str21	CP025220.1	3,031,937	37.94	1	0	2,972	18	67	NA	NA	NA	NA
SAMN09692929	L.monocytogenes	L.monocytogenes_1639_str20	CP041213.1	3,091,571	37.89	1	1	3,030	18	67	USA	2018	chocolate milk	Food
SAMN14261963	L.monocytogenes	L.monocytogenes_1639_str1	CP075878.1	3,100,316	37.8	1	0	3,085	4	65	Norway	2007	Environment	Environment
SAMN02203126	L.monocytogenes	L.monocytogenes_1639_str19	CP006594.1	3,091,600	37.89	1	1	3,031	18	67	USA	1994	food	Food
SAMEA2272573	L.monocytogenes	L.monocytogenes_1639_str198	HE999704.1	2,776,517	38.11	1	0	3,302	18	67	NA	NA	NA	NA
SAMN08138490	L.monocytogenes	L.monocytogenes_1639_str197	CP025201.1	2,944,137	37.97	1	0	3,011	18	67	NA	NA	NA	NA
SAMEA26404168	L.monocytogenes	L.monocytogenes_1639_str196	LR134398.1	2,824,968	38.05	1	0	2,755	18	67	NA	1941	Vein blood	Homo sapiens
SAMN10163247	L.monocytogenes	L.monocytogenes_1639_str195	CP033738.1	2,836,861	38.29	1	0	2,730	18	67	NA	NA	clinical isolate	Homo sapiens
SAMN04286629	L.monocytogenes	L.monocytogenes_1639_str194	CP013286.1	2,839,870	38.25	1	0	2,738	17	58	United Kingdom	1961	England	Chicken
SAMN04286630	L.monocytogenes	L.monocytogenes_1639_str193	CP013287.1	2,842,039	38.26	1	0	2,737	18	67	USA	1931	animal tissue	NA
SAMN23386453	L.monocytogenes	L.monocytogenes_1639_str192	CP090057.1	2,926,991	37.8	1	1	2,871	18	67	USA	1997	Sheep outbreak NA vaginal swab	Sheep
SAMN10163248	L.monocytogenes	L.monocytogenes_1639_str191	CP033737.1	2,852,069	38.09	1	0	2,759	18	67	NA	NA	clinical isolate	Homo sapiens
SAMN04452088	L.monocytogenes	L.monocytogenes_1639_str190	CP014261.1	2,864,291	38.05	1	0	2,773	18	67	Italy	2014	blood	Homo sapiens
SAMEA7887387	L.monocytogenes	L.monocytogenes_1639_str18	LR999860.1	3,034,113	37.96	1	0	3,021	18	67	United Kingdom	2015	hip replacement	Homo sapiens
SAMN16231354	L.monocytogenes	L.monocytogenes_1639_str189	CP062126.1	2,864,720	38.05	1	0	2,780	18	67	NA	NA	NA	NA
SAMN19292164	L.monocytogenes	L.monocytogenes_1639_str188	CP076125.1	2,865,238	38.08	1	0	2,765	18	67	South Korea	2013	tissue	Goat
SAMN14074482	L.monocytogenes	L.monocytogenes_1639_str187	CP060432.1	2,865,294	38.06	1	0	2,772	18	67	Russia	2019	food	Food
SAMN25653831	L.monocytogenes	L.monocytogenes_1639_str186	CP092058.1	2,865,342	38.06	1	0	2,770	18	67	Canada	2018	Microgreen Mix	Food
SAMN14074485	L.monocytogenes	L.monocytogenes_1639_str185	CP060429.1	2,865,353	38.06	1	0	2,773	18	67	Russia	1971	necrotizing tonsils	Homo sapiens
SAMN14074483	L.monocytogenes	L.monocytogenes_1639_str184	CP060431.1	2,865,554	38.06	1	0	2,773	18	67	Russia	1952	internal organs	Microtus arvalis
SAMN18537090	L.monocytogenes	L.monocytogenes_1639_str183	CP076625.1	2,871,309	38.02	1	0	2,785	18	67	South Korea	2013	tissue	Goat
SAMN17320318	L.monocytogenes	L.monocytogenes_1639_str182	CP068392.1	2,871,334	38.02	1	0	2,771	18	67	South Korea	2017	tissue	Goat
SAMN10148445	L.monocytogenes	L.monocytogenes_1639_str181	CP032673.1	2,871,995	38.15	1	0	2,813	18	67	USA	2014	water	Environment
SAMN10148434	L.monocytogenes	L.monocytogenes_1639_str180	CP032670.1	2,871,995	38.15	1	0	2,813	18	67	USA	2014	placenta	Cattle
SAMEA7887386	L.monocytogenes	L.monocytogenes_1639_str17	LR999861.1	3,034,314	37.96	1	0	3,020	18	67	United Kingdom	2015	Hip replacement	Homo sapiens

SAMN14261987	L.monocytogenes	L.monocytogenes_1639_str179	CP076051.1	2,875,161	37.9	1	0	2,831	6	66	Norway	2007	Environment	Environment
SAMN25653832	L.monocytogenes	L.monocytogenes_1639_str178	CP092057.1	2,879,234	38.01	1	0	2,777	18	67	Canada	2018	Sunflower Microgreens	Food
SAMN14260727	L.monocytogenes	L.monocytogenes_1639_str177	CP075871.1	2,884,293	37.91	1	0	2,815	6	66	Norway	2007	Environment	Environment
SAMN16822871	L.monocytogenes	L.monocytogenes_1639_str176	CP065028.1	2,885,142	38.24	1	0	2,751	18	67	USA	2014	brain	Sheep
SAMN17320315	L.monocytogenes	L.monocytogenes_1639_str175	CP068599.1	2,886,896	38.02	1	0	2,815	18	67	South Korea	2018	NA	Goat
SAMN25653833	L.monocytogenes	L.monocytogenes_1639_str174	CP092056.1	2,890,028	38.01	1	0	2,776	18	67	Canada	2018	Spring Pea Microgreens	Food
SAMN06681248	L.monocytogenes	L.monocytogenes_1639_str173	CP020774.1	2,892,474	38.05	1	0	2,794	18	67	Uruguay	2016	blood and lung culture	Homo sapiens
SAMN19669079	L.monocytogenes	L.monocytogenes_1639_str172	CP076644.1	2,893,649	38.2	1	0	2,799	18	67	USA	2014	liver	Cow
SAMN04452089	L.monocytogenes	L.monocytogenes_1639_str171	CP014790.1	2,952,246	38	1	1	2,873	18	67	Italy	2015	blood	Homo sapiens
SAMN09197734	L.monocytogenes	L.monocytogenes_1639_str170	CP029372.1	2,894,782	38.04	1	0	2,813	18	67	Italy	2018	blood	Homo sapiens
SAMN02839425	L.monocytogenes	L.monocytogenes_1639_str16	CP019167.1	3,115,752	37.95	1	1	3,079	18	67	Canada	2008	blood	NA
SAMN02713648	L.monocytogenes	L.monocytogenes_1639_str169	CP007687.1	2,896,586	38.05	1	0	2,817	18	67	USA	2011	human clinical isolate	Homo sapiens
SAMN13341946	L.monocytogenes	L.monocytogenes_1639_str168	CP046362.1	2,896,666	37.95	1	0	2,803	18	67	Switzerland	2015	septic hip prosthetic joint fluid	Homo sapiens
SAMN02713647	L.monocytogenes	L.monocytogenes_1639_str167	CP007689.1	2,897,190	38.02	1	0	2,815	18	67	USA	2010	Human clinical case	Homo sapiens
SAMN13341854	L.monocytogenes	L.monocytogenes_1639_str166	CP046361.1	2,897,260	37.96	1	0	2,804	18	67	Switzerland	2010	Hip prosthetic joint fluid	Homo sapiens
SAMN02347435	L.monocytogenes	L.monocytogenes_1639_str165	CP020832.1	2,897,335	38.05	1	0	2,820	18	67	USA	NA	NA	NA
SAMN10391004	L.monocytogenes	L.monocytogenes_1639_str164	CP033612.1	2,899,315	38.04	1	0	2,797	18	67	Switzerland	2007	brain	Cattle
SAMN03255437	L.monocytogenes	L.monocytogenes_1639_str163	CP030102.1	2,935,398	38	1	1	2,826	18	67	USA	2002	water	Environment
SAMEA104569033	L.monocytogenes	L.monocytogenes_1639_str162	LT985474.1	2,900,890	38.04	1	0	2,798	18	67	NA	NA	NA	NA
SAMN17320316	L.monocytogenes	L.monocytogenes_1639_str161	CP068600.1	2,901,393	38.04	1	0	2,798	18	67	South Korea	2018	NA	Bos taurus
SAMN19267648	L.monocytogenes	L.monocytogenes_1639_str160	CP076127.1	2,901,641	38.04	1	0	2,802	18	67	South Korea	2017	tissue	Goat
SAMN02689388	L.monocytogenes	L.monocytogenes_1639_str15	CP011398.2	3,108,102	37.91	1	1	3,091	18	67	USA	2014	green chili	NA
SAMN03073546	L.monocytogenes	L.monocytogenes_1639_str159	CP010346.1	2,904,228	38.03	1	0	2,814	18	65	Italy	2009	cephalorachidian fluid	Food
SAMN03177401	L.monocytogenes	L.monocytogenes_1639_str158	CP009897.1	2,904,500	38.04	1	0	2,806	18	67	China	2011	NA	Sheep
SAMN02689320	L.monocytogenes	L.monocytogenes_1639_str157	CP007492.1	2,904,662	38.04	1	0	2,808	18	67	USA	2014	food	Food
SAMN09692985	L.monocytogenes	L.monocytogenes_1639_str156	CP041211.1	2,986,335	38.02	1	1	2,918	18	67	USA	2018	NA	NA
SAMN04348332	L.monocytogenes	L.monocytogenes_1639_str155	CP013723.1	2,905,347	38.04	1	0	2,844	18	67	Switzerland	2005	blood	Homo sapiens
SAMN04387840	L.monocytogenes	L.monocytogenes_1639_str154	CP013919.1	2,905,533	37.96	1	0	2,841	15	58	Italy	2008	blood	Homo sapiens
SAMN02769705	L.monocytogenes	L.monocytogenes_1639_str153	CP020830.1	2,905,907	38.05	1	0	2,839	18	67	USA	1988	blood	Homo sapiens
SAMN02318985	L.monocytogenes	L.monocytogenes_1639_str152	CP007600.2	2,906,670	38.03	1	0	2,813	18	67	USA	2013	cheese	Food
SAMN03255438	L.monocytogenes	L.monocytogenes_1639_str151	CP026043.1	2,906,671	38.03	1	0	2,813	18	67	USA	2013	cheese	Environment
SAMN02713641	L.monocytogenes	L.monocytogenes_1639_str150	CP007684.1	2,907,486	38	1	0	2,831	18	67	USA	2011	human host	Homo sapiens
SAMN03002779	L.monocytogenes	L.monocytogenes_1639_str14	CP014252.2	3,105,523	38.02	1	1	3,037	18	67	USA	2014	fire pearl white nectarine	NA
SAMEA26403418	L.monocytogenes	L.monocytogenes_1639_str149	LR134397.1	2,908,293	38.01	1	0	2,849	18	67	United Kingdom	1924	Mesenteric lymph gland	Pig
SAMN10745353	L.monocytogenes	L.monocytogenes_1639_str148	CP035187.1	2,908,718	38.03	1	0	2,823	18	67	Switzerland	2016	blood	Homo sapiens
SAMN15862383	L.monocytogenes	L.monocytogenes_1639_str147	CP060526.1	2,999,149	38.01	1	1	2,912	18	67	USA	2008	NA	NA
SAMN25653828	L.monocytogenes	L.monocytogenes_1639_str146	CP092061.1	2,910,079	37.98	1	0	2,797	18	67	Canada	2014	Bean Sprouts	Food
SAMN08939842	L.monocytogenes	L.monocytogenes_1639_str145	CP029175.1	2,911,466	37.98	1	0	2,811	18	67	South Korea	2012	blood	Homo sapiens
SAMN03372633	L.monocytogenes	L.monocytogenes_1639_str144	CP011004.1	2,911,639	38.06	1	0	2,805	18	67	Switzerland	2014	blood	Homo sapiens
SAMN02351401	L.monocytogenes	L.monocytogenes_1639_str143	CP020831.1	2,912,199	38	1	0	2,812	18	67	USA	2013	blood	NA
SAMN19267651	L.monocytogenes	L.monocytogenes_1639_str142	CP076626.1	2,912,948	38.06	1	0	2,806	18	67	South Korea	2019	tissue	Goat
SAMN17036357	L.monocytogenes	L.monocytogenes_1639_str141	CP067362.1	2,913,085	38.01	1	0	2,799	18	67	South Korea	2016	NA	Goat
SAMEA104314690	L.monocytogenes	L.monocytogenes_1639_str140	LT985475.1	2,913,696	38.06	1	0	2,806	18	67	NA	NA	NA	NA
SAMN02689388	L.monocytogenes	L.monocytogenes_1639_str13	CP045749.1	3,094,088	37.93	1	1	3,065	18	67	USA	2014	green chili	Food
SAMN06297510	L.monocytogenes	L.monocytogenes_1639_str139	CP019616.1	2,913,947	37.99	1	0	2,823	16	67	Canada	2009	Baby spinach	Food
SAMN19267647	L.monocytogenes	L.monocytogenes_1639_str138	CP076126.1	2,915,057	38.07	1	0	2,806	18	67	South Korea	2016	tissue	Goat
SAMN10148429	L.monocytogenes	L.monocytogenes_1639_str137	CP032672.1	2,915,525	38.07	1	0	2,806	18	67	USA	2014	cervico-vaginal swab	Cattle
SAMN02569973	L.monocytogenes	L.monocytogenes_1639_str136	CP011397.1	2,918,170	38	1	0	2,857	18	67	NA	NA	NA	NA
SAMN02997429	L.monocytogenes	L.monocytogenes_1639_str135	CP009242.1	2,918,502	38.15	1	0	2,861	20	72	Australia	1985	NA	Ovis aries
SAMN06297525	L.monocytogenes	L.monocytogenes_1639_str134	CP019620.1	2,922,011	37.99	1	0	2,835	17	66	NA	2006	Environment swab	Environment
SAMN04348333	L.monocytogenes	L.monocytogenes_1639_str133	CP013722.1	2,927,751	38	1	0	2,841	18	67	Switzerland	2005	blood	Homo sapiens
SAMN19696832	L.monocytogenes	L.monocytogenes_1639_str132	CP076669.1	2,983,560	38.04	1	1	2,900	18	67	USA	2014	liver	Horse
SAMN06297605	L.monocytogenes	L.monocytogenes_1639_str131	CP019625.1	2,929,632	38.01	1	0	2,872	17	67	NA	2002	Environment sponge	Environment
SAMN13191635	L.monocytogenes	L.monocytogenes_1639_str130	CP045972.1	2,989,366	37.93	1	1	2,927	18	67	Australia	2009	NA	Homo sapiens
SAMN07718437	L.monocytogenes	L.monocytogenes_1639_str12	CP023752.1	3,116,331	37.99	1	1	3,085	18	67	Finland	1995	food product	Environment
SAMN02713668	L.monocytogenes	L.monocytogenes_1639_str129	CP007686.1	2,932,501	38.04	1	0	2,808	18	67	USA	2011	human clinical isolate	Homo sapiens
SAMN05363481	L.monocytogenes	L.monocytogenes_1639_str128	CP016629.1	2,935,033	38	1	0	2,823	18	67	South Korea	2014	Lettuce	Food
SAMN10148451	L.monocytogenes	L.monocytogenes_1639_str127	CP032669.1	2,935,335	37.98	1	0	2,870	18	67	USA	NA	abortion case	Cattle
SAMN08939835	L.monocytogenes	L.monocytogenes_1639_str126	CP031141.1	2,935,813	38	1	0	2,828	18	67	South Korea	2017	blood	Homo sapiens
SAMEA104314691	L.monocytogenes	L.monocytogenes_1639_str125	LT985476.1	2,935,970	37.93	1	0	2,853	18	68	NA	NA	NA	NA
SAMN10148454	L.monocytogenes	L.monocytogenes_1639_str124	CP032668.1	2,936,760	38.06	1	0	2,839	18	67	USA	NA	abortion case	Cattle
SAMN03002784	L.monocytogenes	L.monocytogenes_1639_str123	CP045748.1	2,939,705	37.98	1	0	2,841	18	66	USA	2014	peach	Food
SAMN03002785	L.monocytogenes	L.monocytogenes_1639_str122	CP045747.1	2,939,729	37.98	1	0	2,839	18	67	USA	2014	peach	Food
SAMN03002789	L.monocytogenes	L.monocytogenes_1639_str121	CP045745.1	2,939,732	37.98	1	0	2,839	18	67	USA	2014	peach	Food
SAMN03002788	L.monocytogenes	L.monocytogenes_1639_str120	CP045746.1	2,939,733	37.98	1	0	2,841	18	66	USA	2014	peach	Food
SAMN08663437	L.monocytogenes	L.monocytogenes_1639_str11	CP028183.1	3,124,949	37.91	1	1	3,083	18	67	Denmark	2015	smoked fish	Meat
SAMN03002783	L.monocytogenes	L.monocytogenes_1639_str119	CP012021.2	2,939,733	37.98	1	0	2,840	18	67	USA	2014	peach	Food
SAMN25653829	L.monocytogenes	L.monocytogenes_1639_str118	CP092060.1	2,939,920	37.98	1	0	2,840	18	67	Canada	2015	Chopped Romaine Lettuce	Food
SAMN06297603	L.monocytogenes	L.monocytogenes_1639_str117	CP019623.1	2,940,913	37.91	1	0	2,870	16	58	NA	2007	chicken breast	Meat
SAMN07612368	L.monocytogenes	L.monocytogenes_1639_str116	CP023321.1	2,941,368	37.99	1	0	2,850	18	67	India	2016	blood	Homo sapiens
SAMN04286628	L.monocytogenes	L.monocytogenes_1639_str115	CP013285.1	2,942,510	37.99	1	0	2,849	18	67	United Kingdom	1961	England	Chicken
SAMN13973245	L.monocytogenes	L.monocytogenes_1639_str114	CP048400.1	2,942,932	37.98	1	0	2,893	18	67	USSR	1952	Kazakhstan	Tick
SAMN02203122	L.monocytogenes	L.monocytogenes_1639_str113	CP006592.1	2,943,218	37.99	1	0	2,845	14	58	USA	1989	animal	NA
SAMN17320319	L.monocytogenes	L.monocytogenes_1639_str112	CP068601.1	2,945,237	38.01	1	0	2,870	18	67	South Korea	2019	NA	Goat
SAMN16231355	L.monocytogenes	L.monocytogenes_1639_str111	CP062124.1	2,996,205	37.97	1	1	2,888	18	67	USA	1983	NA	NA
SAMN02713644	L.monocytogenes	L.monocytogenes_1639_str110	CP007685.1	2,947,293	37.98	1	0	2,898	18	67	USA	2011	human clinical case	Homo sapiens
SAMN06297602	L.monocytogenes	L.monocytogenes_1639_str10	CP019622.1	3,070,031	38.03	1	0	3,046	17	68	NA	2007	Ground beef	Meat
SAMN02712416	L.monocytogenes	L.monocytogenes_1639_str109	CP007688.1	2,947,477	37.98	1	0	2,898	18	67	USA	2009	human clinical case	Homo sapiens
SAMN14927919	L.monocytogenes	L.monocytogenes_1639_str108	CP053478.1	2,949,231	38.01	1	0	2,884	18	67	USA	2002	food	Food
SAMN04286632	L.monocytogenes	L.monocytogenes_1639_str107	CP013289.1	2,950,749	37.99	1	0	2,852	18	67	NA	NA	NA	NA
SAMN05291439	L.monocytogenes	L.monocytogenes_1639_str106	CP018148.2	2,950,780	37.99	1	0	2,844	18	67	Russia	2005	Myodes rufocanus	NA
SAMN11056493	L.monocytogenes	L.monocytogenes_1639_str105	CP040988.1	2,950,983	37.99	1	0	2,844	18	67	USA	NA	ATCC Isolate	NA
SAMN09760372	L.monocytogenes	L.monocytogenes_1639_str104	CP031476.1	2,950,983	37.99	1	0	2,843	18	67	NA	NA	NA	Homo sapiens
SAMN08125522	L.monocytogenes	L.monocytogenes_1639_str103	CP025219.1	2,950,984	37.99	1	0	2,842	18	67	NA	NA	NA	NA
SAMEA7376280	L.monocytogenes	L.monocytogenes_1639_str102	CP063240.1	2,951,169	37.96	1	0	2,902	18	67	Germany	2019	clinical isolate	Homo sapiens
SAMEA6798783	L.monocytogenes	L.monocytogenes_1639_str101	CP064843.1	3,038,549	37.93	1	1	2,989	18	67	Germany	2018	human listeriosis	Homo sapiens
SAMN04348334	L.monocytogenes	L.monocytogenes_1639_str100	CP013724.1	3,039,224	37.93	1	1	2,995	18	67	Switzerland	2011	blood	Homo sapiens
SAMEA44515918	L.monocytogenes	L.monocytogenes_1639	LT906436.1	2,864,663	38.06	1	0	2,777	18	67	NA	1924	NA	Rabbit
SAMN02641382	L.monocytogenes	L.monocytogenes_10403S_393133	CP002002.1	2,903,106	38.03	1	0	2,824	18	67	NA	NA	NA	NA
SAMN02603721	L.monocytogenes	L.monocytogenes_08_5923_637381	CP001604.1	2,999,054	37.96	1	0	2,959	15	58	NA	NA	NA	NA
SAMN02603776	L.monocytogenes	L.monocytogenes_08_5578_653938	CP001602.2	3,109,678	37.92	1	1	3,080	15	58	NA	NA	NA	NA
SAMN02604190	L.monocytogenes	L.monocytogenes_07PF0776_1126011	CP003414.1	2,901,562	38.04	1	0	2,814	18	67	NA	NA	myocardial abscess	NA
SAMN01909075	L.marthii	L.marthii_FSL_S4_120_702457	CM001047.1	2,884,551	36.79	1	0	2,597	2	28	USA	2001	forest soil	Environment
SAMN21217737	L.marthii	L.marthii_529731_str1	CP089089.1	2,947,729	38.62	1	0	2,863	18	67	USA	2021	soil	Environment
SAMN21217731	L.marthii	L.marthii_529731	CP089090.1	2,840,389	38.74	1	0	2,759	18	67	USA	2021	soil	NA
SAMN02743816	L.ivanovii	L.ivanovii_WSLC3009_1457190	CP007172.1	2,919,538	37.15	1	0	2,810	18	67	Germany	NA	NA	NA
SAMN02953499	L.ivanovii	L.ivanovii_subsp_londoniensis_202752_str2	CP009575.1	2,993,492	37.04	1	0	2,843	18	67	NA	NA	NA	NA
SAMN02953498	L.ivanovii	L.ivanovii_subsp_londoniensis_202752_str1	CP009576.1	3,045,035	37.09	1	0	2,924	18	67	NA	NA	NA	NA
SAMEA26389918	L.ivanovii	L.ivanovii_subsp_londoniensis_202752	LR134394.1	3,007,116	37.02	1	0	2,871	18	67	NA	1900	NA	NA
SAMEA3138408	L.ivanovii	L.ivanovii_subsp_ivanovii_PAM_55_881621	FR687253.1	2,928,879	37.13	1	0	2,828	18	67	NA	NA	NA	NA
SAMN02953497	L.ivanovii	L.ivanovii_subsp_ivanovii_202751_str2	CP009577.1	2,919,548	37.15	1	0	2,805	18	67	NA	NA	NA	NA
SAMEA4362421	L.ivanovii	L.ivanovii_subsp_ivanovii_202751_str1	LT906478.1	2,919,550	37.15	1	0	2,805	18	67	NA	1900	NA	Sheep
SAMEA4475701	L.ivanovii	L.ivanovii_subsp_ivanovii_202751	LT906466.1	2,919,553	37.15	1	0	2,806	18	67	NA	1900	NA	NA
SAMN01908947	L.ivanovii	L.ivanovii_FSL_F6_596_702454	CM001050.1	3,133,385	34.59	1	0	2,682	1	27	France	NA	food	Food
SAMN10662806	L.ivanovii	L.ivanovii_1638	CP034771.1	2,893,311	37.02	1	0	2,806	7	52	NA	2018	NA	NA
SAMN01909076	L.innocua	L.innocua_FSL_S4_378_702456	CM001048.1	3,115,943	34.65	1	0	2,649	0	24	USA	2002	puddle of water	Environment
SAMN01909077	L.innocua	L.innocua_FSL_J1_023_702455	CM001049.1	2,914,007	35.85	1	0	2,622	3	25	NA	NA	NA	NA
SAMEA3138320	L.innocua	L.innocua_Clip11262_272626	AL592022.1	3,093,113	37.39	1	1	3,130	18	67	NA	NA	NA	NA
SAMN18080006	L.innocua	L.innocua_1642_str4	CP071179.1	3,073,568	37.32	1	1	3,059	18	67	China	2015	food	Food
SAMN18080009	L.innocua	L.innocua_1642_str3	CP071157.1	3,023,427	37.39	1	1	2,990	18	67	China	2016	food	Food
SAMN18079989	L.innocua	L.innocua_1642_str2	CP071155.1	3,015,983	37.37	1	1	2,976	18	67	China	2015	food	Food
SAMN04417899	L.innocua	L.innocua_1642_str1	CP045743.1	2,922,148	37.48	1	1	2,879	18	66	Italy	2005	minced meat	Meat
SAMN08138574	L.innocua	L.innocua_1642	CP025202.1	2,889,727	37.46	1	0	2,892	18	67	NA	NA	NA	NA
SAMN00001446	L.grayi	L.grayi_DSM_20601_525367	GL538352.1	2,598,321	41.47	3	1	2,561	7	52	NA	NA	NA	Homo sapiens
SAMEA103980423	L.grayi	L.grayi_1641_str2	LR134483.1	2,751,104	41.57	1	0	2,657	18	65	USA	1900	Cornstalks and leaves	Plant
SAMEA4556066	L.grayi	L.grayi_1641_str1	UGPG01000002.1	2,602,779	41.73	2	1	2,982	18	65	USA	1900	Cornstalks and leaves	Plant
SAMEA5850801	L.grayi	L.grayi_1641	CABKNG010000001.1	2,598,321	41.47	4	1	2,561	7	52	NA	NA	human gut	Homo sapiens
