# Supplementary material for: Comparative Genomics Reveal the Utilization Ability of Variable Carbohydrates as Key Genetic Features of Listeria Pathogens in Their Pathogenic Lifestyles
Source: Pathogens. 2022 Nov 28;11(12):1430. doi: 10.3390/pathogens11121430 (PMC9784484; doi:10.3390/pathogens11121430)
Supplement: Supplementary file 1 [file pathogens-11-01430-s001.zip › pathogens-1990707-supplementary/Supplymentary Tables S3.pdf]

Table S3. Unique genes of LMO referenced to *L.monocytogenes* EGD-e_169963

ORF_id	Sequence_id	Group_id	start	end	strand	COG_id	COG_annotation	VFDB_annotation	Uniprot_annotation
orf_10	AL591824.1	group_1348	12918	13886	+	COG1577	I;Mevalonate kinase;ERG12;Isoprenoid bio	-	Mevalonate kinase OS=Pyrococcus abyssi (strain GE5 / Orsay) OX=272844 GN=mvk PE=3 SV=1
orf_19	AL591824.1	group_1915	23132	23953	-	COG4990	R;Predicted cysteine peptidase, C39 family;	-	Uncharacterized protein YvpB OS=Bacillus subtilis (strain 168) OX=224308 GN=yvpB PE=3 SV=1
orf_20	AL591824.1	group_2301	24194	24934	+	COG2188	K;DNA-binding transcriptional regulator, Gr	-	Probable fructoselysine utilization operon transcriptional repressor OS=Escherichia coli O157:H7 OX=83334 GN=frlR PE=3 SV=2
orf_21	AL591824.1	group_2287	24950	25351	+	COG2893	G;Phosphotransferase system, mannose/fr	-	PTS system sorbose-specific EIIA component OS=Klebsiella pneumoniae OX=573 GN=sorF PE=1 SV=1
orf_23	AL591824.1	group_2292	25862	26665	+	COG3715	G;Phosphotransferase system, mannose/fr	-	N-acetylgalactosamine permease IIC component 1 OS=Escherichia coli (strain K12) OX=83333 GN=agaC PE=1 SV=1
orf_25	AL591824.1	group_2294	27495	28199	+	COG4821	R;Uncharacterized conserved protein, cont	-	UPF0309 protein lmo0025 OS=Listeria monocytogenes serovar 1/2a (strain ATCC BAA-679 / EGD-e) OX=169963 GN=lmo0025 PE=3 SV=1
orf_26	AL591824.1	group_2297	28254	28895	+	COG3142	P;Copper homeostasis protein CutC;CutC;	-	PF03932 family protein CutC OS=Enterococcus faecalis (strain ATCC 700802 / V583) OX=226185 GN=cutC PE=2 SV=1
orf_28	AL591824.1	group_1836	31088	31963	+	COG1619	M;Muramoyltetrapeptide carboxypeptidas	-	Probable murein peptide carboxypeptidase OS=Bacillus subtilis (strain 168) OX=224308 GN=ykfA PE=2 SV=2
orf_52	AL591824.1	group_1808	54839	56812	+	COG3887	T;Cyclic di-AMP phosphodiesterase GdpP, c	-	Cyclic-di-AMP phosphodiesterase PdeA OS=Listeria monocytogenes serotype 1/2a (strain 10403S) OX=393133 GN=pdeA PE=3 SV=1
orf_78	AL591824.1	group_33	82959	84437	+	-	-	-	Toxin BC_0920 OS=Bacillus cereus (strain ATCC 14579 / DSM 31 / CCUG 7414 / JCM 2152 / NBRC 15305 / NCIMB 9373 / NCTC 2599 / NRRL B-3711) OX=226900 GN=BC_0920 PE=1 SV=1
orf_85	AL591824.1	group_602	88888	94767	+	-	-	-	-
orf_87	AL591824.1	group_2206	97127	97369	+	COG0636	C;FoF1-type ATP synthase, membrane subu	-	ATP synthase subunit c, chloroplastic OS=Pisum sativum OX=3888 GN=atpH PE=1 SV=1
orf_94	AL591824.1	group_219	103204	103836	+	-	-	-	-
orf_107	AL591824.1	group_2220	116972	117805	+	COG2207	K;AraC-type DNA-binding domain and AraC	-	HTH-type transcriptional activator RhaS OS=Escherichia fergusonii (strain ATCC 35469 / DSM 13698 / CCUG 18766 / IAM 14443 / JCM 21226 / LMG 7866 / NBRC 102419 / NCTC 12128 / CDC 0568-73) OX=585054 GN=rhaS PE=3 SV=1
orf_129	AL591824.1	group_2250	133961	134710	+	COG2200	T;EAL domain, c-di-GMP-specific phosphod	-	Signaling protein YkoW OS=Bacillus subtilis (strain 168) OX=224308 GN=ykoW PE=3 SV=2
orf_135	AL591824.1	group_1804	139960	140853	+	COG1173	EP;ABC-type dipeptide/oligopeptide/nickel	-	Oligopeptide transport system permease protein AppC OS=Bacillus subtilis (strain 168) OX=224308 GN=appC PE=3 SV=1
orf_137	AL591824.1	group_180	141337	141705	+	-	-	-	-
orf_138	AL591824.1	group_33	141702	143006	+	-	-	-	Toxin BC_0920 OS=Bacillus cereus (strain ATCC 14579 / DSM 31 / CCUG 7414 / JCM 2152 / NBRC 15305 / NCIMB 9373 / NCTC 2599 / NRRL B-3711) OX=226900 GN=BC_0920 PE=1 SV=1
orf_152	AL591824.1	group_1788	151839	152645	+	COG1108	P;ABC-type Mn2+/Zn2+ transport system, p	-	High-affinity zinc uptake system membrane protein ZnuB OS=Bacillus subtilis (strain 168) OX=224308 GN=znuB PE=1 SV=1
orf_167	AL591824.1	group_170	168011	169270	+	COG5361	X;Uncharacterized conserved protein;;	-	-
orf_174	AL591824.1	group_1800	175763	177760	+	COG0143	J;Methionyl-tRNA synthetase;MetG;Amino	-	Methionine--tRNA ligase OS=Listeria monocytogenes serovar 1/2a (strain ATCC BAA-679 / EGD-e) OX=169963 GN=metG PE=3 SV=1
orf_185	AL591824.1	group_1412	192586	193473	+	COG0030	J;16S rRNA A1518 and A1519 N6-dimethylt	-	Ribosomal RNA small subunit methyltransferase A OS=Listeria monocytogenes serovar 1/2a (strain ATCC BAA-679 / EGD-e) OX=169963 GN=rsmA PE=3 SV=1
orf_197	AL591824.1	group_2342	203640	204353	-	COG0664	T;cAMP-binding domain of CRP or a regulat	(prfA) listeriolysin positive regulatory protein [PrfA (VF0062) - Regulation (VFC	Listeriolysin regulatory protein OS=Listeria monocytogenes serovar 1/2a (strain ATCC BAA-679 / EGD-e) OX=169963 GN=prfA PE=1 SV=2
orf_201	AL591824.1	group_2382	209470	211389	+	-	-	(actA) actin-assembly inducing protein precursor [ActA (VF0066) - Motility (VF	Actin assembly-inducing protein OS=Listeria monocytogenes serovar 1/2a (strain ATCC BAA-679 / EGD-e) OX=169963 GN=actA PE=1 SV=1
orf_203	AL591824.1	group_2379	212366	212689	+	-	-	-	Uncharacterized protein Lmo0206 OS=Listeria monocytogenes serovar 1/2a (strain ATCC BAA-679 / EGD-e) OX=169963 GN=lmo0206 PE=3 SV=1
orf_204	AL591824.1	group_2335	212824	213285	+	COG4808	S;Uncharacterized lipoprotein YehR, DUF13	-	Uncharacterized lipoprotein Lmo0207 OS=Listeria monocytogenes serovar 1/2a (strain ATCC BAA-679 / EGD-e) OX=169963 GN=lmo0207 PE=3 SV=1
orf_216	AL591824.1	group_1763	224562	226508	+	COG0634	F;Hypoxanthine-guanine phosphoribosyltra	-	Bifunctional protein TilS/HprT OS=Listeria monocytogenes serovar 1/2a (strain ATCC BAA-679 / EGD-e) OX=169963 GN=tilS/hprT PE=3 SV=1
orf_219	AL591824.1	group_772	229840	230724	+	COG1281	O;Redox-regulated molecular chaperone, H	-	33 kDa chaperonin OS=Listeria monocytogenes serovar 1/2a (strain ATCC BAA-679 / EGD-e) OX=169963 GN=hslO PE=3 SV=1
orf_233	AL591824.1	group_2322	256491	256964	+	COG0245	I;2C-methyl-D-erythritol 2,4-cyclodiphosph	-	2-C-methyl-D-erythritol 2,4-cyclodiphosphate synthase OS=Listeria monocytogenes serovar 1/2a (strain ATCC BAA-679 / EGD-e) OX=169963 GN=ispF PE=3 SV=1
orf_237	AL591824.1	group_1649	260896	261306	+	COG1939	J;23S rRNA maturation mini-RNase III;MrnC	-	Mini-ribonuclease 3 OS=Listeria monocytogenes serovar 1/2a (strain ATCC BAA-679 / EGD-e) OX=169963 GN=mrnC PE=3 SV=1
orf_255	AL591824.1	group_1726	276728	280333	+	COG0086	K;DNA-directed RNA polymerase, beta' sub	-	DNA-directed RNA polymerase subunit beta' OS=Listeria monocytogenes serovar 1/2a (strain ATCC BAA-679 / EGD-e) OX=169963 GN=rpoC PE=3 SV=1
orf_267	AL591824.1	group_8	293845	295281	-	COG2723	G;Beta-glucosidase/6-phospho-beta-glucos	-	Aryl-phospho-beta-D-glucosidase BglC OS=Bacillus subtilis (strain 168) OX=224308 GN=bglC PE=1 SV=1
orf_293	AL591824.1	group_2318	322134	324005	-	COG3711	K;Transcriptional antiterminator;BglG;	-	Probable licABCH operon regulator OS=Bacillus subtilis (strain 168) OX=224308 GN=licR PE=1 SV=1
orf_296	AL591824.1	group_8	325729	327120	-	COG2723	G;Beta-glucosidase/6-phospho-beta-glucos	-	6-phospho-beta-galactosidase OS=Clostridium acetobutylicum (strain ATCC 824 / DSM 792 / JCM 1419 / LMG 5710 / VKM B-1787) OX=272562 GN=lacG PE=3 SV=1
orf_297	AL591824.1	group_2314	327113	327454	-	COG1447	G;Phosphotransferase system cellobiose-sp	-	PTS system oligo-beta-mannoside-specific EIIA component OS=Bacillus subtilis (strain 168) OX=224308 GN=gmuA PE=1 SV=2
orf_301	AL591824.1	group_2244	329923	330999	+	COG2008	E;Threonine aldolase;GLY1;	-	Low-specificity L-threonine aldolase 1 OS=Arabidopsis thaliana OX=3702 GN=THA1 PE=1 SV=1
orf_311	AL591824.1	group_2027	340278	340952	+	COG0819	H;Aminopyrimidine aminohydrolase TenA (	-	Aminopyrimidine aminohydrolase OS=Bacillus subtilis (strain 168) OX=224308 GN=tenA PE=1 SV=1
orf_312	AL591824.1	group_2115	340945	341754	+	COG2145	H;Hydroxyethylthiazole kinase, sugar kinas	-	Hydroxyethylthiazole kinase OS=Listeria monocytogenes serovar 1/2a (strain ATCC BAA-679 / EGD-e) OX=169963 GN=thiM PE=3 SV=1
orf_314	AL591824.1	group_2226	342551	343195	+	COG0352	H;Thiamine monophosphate synthase;ThiE	-	Thiamine-phosphate synthase OS=Listeria monocytogenes serovar 1/2a (strain ATCC BAA-679 / EGD-e) OX=169963 GN=thiE PE=3 SV=1
orf_317	AL591824.1	group_2249	346377	347036	+	-	-	-	-
orf_321	AL591824.1	group_23	349326	350228	+	-	-	-	HTH-type transcriptional regulator rgg OS=Streptococcus gordonii (strain Challis / ATCC 35105 / BCRC 15272 / CH1 / DL1 / V288) OX=467705 GN=rgg PE=4 SV=1
orf_336	AL591824.1	group_2334	370446	370634	+	-	-	-	-
orf_340	AL591824.1	group_2243	373832	374596	-	COG1028	I;NAD(P)-dependent dehydrogenase, short	-	NAD-dependent glycerol dehydrogenase OS=Listeria innocua serovar 6a (strain ATCC BAA-680 / CLIP 11262) OX=272626 GN=golD PE=1 SV=1
orf_341	AL591824.1	group_2295	374621	375067	-	COG0698	G;Ribose 5-phosphate isomerase RpiB;RpiB	-	Ribose-5-P isomerase B OS=Listeria innocua serovar 6a (strain ATCC BAA-680 / CLIP 11262) OX=272626 GN=rpiB PE=1 SV=1
orf_343	AL591824.1	group_2299	375842	376492	-	COG2376	G;Dihydroxyacetone kinase;DAK1;Isopreno	-	PEP-dependent dihydroxyacetone kinase 2, ADP-binding subunit DhaL OS=Listeria innocua serovar 6a (strain ATCC BAA-680 / CLIP 11262) OX=272626 GN=dhaL-2 PE=1 SV=1
orf_345	AL591824.1	group_2302	377531	377872	-	-	-	-	Putative glycerol transporter Lin0367 OS=Listeria innocua serovar 6a (strain ATCC BAA-680 / CLIP 11262) OX=272626 GN=lin0367 PE=2 SV=1
orf_346	AL591824.1	group_2293	377888	378319	-	-	-	-	Putative glycerol transporter Lin0368 OS=Listeria innocua serovar 6a (strain ATCC BAA-680 / CLIP 11262) OX=272626 GN=lin0368 PE=2 SV=1
orf_347	AL591824.1	group_2300	378404	378781	-	COG3412	T;Phosphotransferase subunit DhaM of the	-	PEP-dependent dihydroxyacetone kinase 2, phosphoryl donor subunit DhaM OS=Listeria innocua serovar 6a (strain ATCC BAA-680 / CLIP 11262) OX=272626 GN=dhaM-2 PE=1 SV=1
orf_348	AL591824.1	group_2217	378964	379728	+	COG1349	KG;DNA-binding transcriptional regulator o	-	HTH-type transcriptional regulator GolR OS=Listeria innocua serovar 6a (strain ATCC BAA-680 / CLIP 11262) OX=272626 GN=golR PE=4 SV=1
orf_349	AL591824.1	group_2353	379794	380207	+	COG0456	J;Ribosomal protein S18 acetylase RimI and	-	Uncharacterized N-acetyltransferase ycf52 OS=Neopyropia yezoensis OX=2788 GN=ycf52 PE=3 SV=1
orf_386	AL591824.1	group_2214	412457	412825	+	-	-	-	-
orf_396	AL591824.1	group_2341	419544	420002	+	COG1762	GT;Phosphotransferase system mannitol/fr	-	PTS system 2-O-alpha-mannosyl-D-glycerate-specific EIIABC component OS=Escherichia coli (strain K12) OX=83333 GN=mngA PE=1 SV=1
orf_398	AL591824.1	group_26	420335	421447	+	COG1299	G;Phosphotransferase system, fructose-spe	-	PTS system fructose-specific EIIB'BC component OS=Vibrio cholerae serotype O1 (strain ATCC 39315 / El Tor Inaba N16961) OX=243277 GN=fruA PE=2 SV=1
orf_399	AL591824.1	group_22	421469	424096	+	COG0383	G;Alpha-mannosidase;MngB;	-	Mannosylglycerate hydrolase OS=Escherichia coli (strain K12) OX=83333 GN=mngB PE=1 SV=2
orf_403	AL591824.1	group_904	427104	428111	+	COG0306	P;Phosphate/sulfate permease;PitA;	-	Probable low-affinity inorganic phosphate transporter OS=Bacillus subtilis (strain 168) OX=224308 GN=pit PE=3 SV=2
orf_423	AL591824.1	group_2288	445049	447010	+	COG3711	K;Transcriptional antiterminator;BglG;	-	Probable licABCH operon regulator OS=Bacillus subtilis (strain 168) OX=224308 GN=licR PE=1 SV=1
orf_426	AL591824.1	group_26	447804	448910	+	COG1299	G;Phosphotransferase system, fructose-spe	-	PTS system fructose-specific EIIB'BC component OS=Rhodobacter capsulatus OX=1061 GN=fruA PE=3 SV=1
orf_427	AL591824.1	group_22	448926	451508	+	COG0383	G;Alpha-mannosidase;MngB;	-	Mannosylglycerate hydrolase OS=Escherichia coli (strain K12) OX=83333 GN=mngB PE=1 SV=2
orf_435	AL591824.1	group_2257	466517	467362	+	COG0702	R;Uncharacterized conserved protein YbjT,	-	Quinone oxidoreductase 2 OS=Escherichia coli (strain K12) OX=83333 GN=qorB PE=1 SV=1
orf_440	AL591824.1	group_2077	472330	472644	+	-	-	-	-
orf_442	AL591824.1	group_169	473936	476716	+	COG1511	S;Uncharacterized membrane protein YhgE	-	Uncharacterized protein YhgE OS=Bacillus subtilis (strain 168) OX=224308 GN=yhgE PE=4 SV=2
orf_449	AL591824.1	group_2352	483934	484599	-	COG3619	S;Uncharacterized membrane protein YoaK	-	UPF0700 transmembrane protein YoaK OS=Bacillus subtilis (strain 168) OX=224308 GN=yoaK PE=3 SV=1
orf_450	AL591824.1	group_2343	484620	485375	-	-	-	-	-
orf_456	AL591824.1	group_2222	492918	494018	+	COG3535	S;Uncharacterized conserved protein, DUF	-	-
orf_457	AL591824.1	group_195	494011	495561	+	COG0145	EQ;N-methylhydantoinase A/oxoprolinase/	-	Putative D-/L-hydantoinase subunit A OS=Pseudomonas sp. (strain NS671) OX=29441 GN=hyuA PE=3 SV=1
orf_463	AL591824.1	group_19	501693	501959	+	COG2963	X;Transposase InsE and inactivated derivati	-	-
orf_480	AL591824.1	group_2235	516076	516636	+	COG1309	K;DNA-binding protein, AcrR family, include	-	HTH-type dhaKLM operon transcriptional activator DhaS OS=Lactococcus lactis subsp. lactis (strain IL1403) OX=272623 GN=dhaS PE=1 SV=1
orf_481	AL591824.1	group_1802	516746	518446	+	COG4716	S;Myosin-crossreactive antigen (function u	-	Oleate hydratase OS=Elizabethkingia meningoseptica OX=238 GN=ohyA PE=1 SV=1
orf_495	AL591824.1	group_2346	531294	532253	+	COG0697	GER;Permease of the drug/metabolite tran	-	Uncharacterized transporter YoaV OS=Bacillus subtilis (strain 168) OX=224308 GN=yoaV PE=3 SV=1
orf_506	AL591824.1	group_133	540421	541467	+	COG1063	ER;Threonine dehydrogenase or related Zn	-	Galactitol 1-phosphate 5-dehydrogenase OS=Escherichia coli (strain K12) OX=83333 GN=gatD PE=1 SV=1
orf_511	AL591824.1	group_1745	545609	546289	+	COG0518	F;GMP synthase, glutamine amidotransfera	-	Putative glutamine amidotransferase-like protein YfeJ OS=Salmonella typhimurium (strain LT2 / SGSC1412 / ATCC 700720) OX=99287 GN=yfeJ PE=4 SV=2
orf_516	AL591824.1	group_2363	549916	551346	-	COG2843	M;Poly-gamma-glutamate biosynthesis pro	-	Capsule biosynthesis protein CapA OS=Bacillus anthracis OX=1392 GN=capA PE=2 SV=2
orf_525	AL591824.1	group_170	560443	561774	+	COG5361	X;Uncharacterized conserved protein;;	-	-
orf_528	AL591824.1	group_2225	564257	565753	+	COG5298	R;Predicted metal-dependent carbohydrate	-	-
orf_529	AL591824.1	group_2221	565764	567014	+	COG1215	N;Glycosyltransferase, catalytic subunit of	-	Uncharacterized glycosyltransferase YdaM OS=Bacillus subtilis (strain 168) OX=224308 GN=ydaM PE=3 SV=1
orf_530	AL591824.1	group_2128	567030	569081	+	-	-	-	Uncharacterized protein YdaN OS=Bacillus subtilis (strain 168) OX=224308 GN=ydaN PE=3 SV=1
orf_531	AL591824.1	group_2218	569094	569948	+	COG2199	T;GGDEF domain, diguanylate cyclase (c-di-	-	-
orf_568	AL591824.1	group_1967	605401	606042	-	COG0040	E;ATP phosphoribosyltransferase;HisG;Hist	-	ATP phosphoribosyltransferase OS=Listeria monocytogenes serovar 1/2a (strain ATCC BAA-679 / EGD-e) OX=169963 GN=hisG PE=3 SV=1
orf_594	AL591824.1	group_1783	635317	636423	-	COG2021	E;Homoserine O-acetyltransferase;MET2;M	-	Homoserine O-acetyltransferase OS=Listeria monocytogenes serovar 1/2a (strain ATCC BAA-679 / EGD-e) OX=169963 GN=metXA PE=3 SV=1
orf_597	AL591824.1	group_1605	638781	639482	-	COG0664	T;cAMP-binding domain of CRP or a regulat	-	-
orf_627	AL591824.1	group_2320	664242	668990	+	-	-	-	Collagen adhesin OS=Staphylococcus aureus OX=1280 GN=cna PE=1 SV=1
orf_628	AL591824.1	group_2324	669086	669361	+	-	-	-	-
orf_629	AL591824.1	group_2254	669423	669950	+	COG1335	HR;Nicotinamidase-related amidase;PncA;F	-	Uncharacterized isochorismatase family protein HVO_2328 OS=Haloferax volcanii (strain ATCC 29605 / DSM 3757 / JCM 8879 / NBRC 14742 / NCIMB 2012 / VKM B-1768 / DS2) OX=309800 GN=HVO_2328 PE=1 SV=1
orf_632	AL591824.1	group_26	672794	673855	+	COG1299	G;Phosphotransferase system, fructose-spe	-	Fructose-like permease IIC component 2 OS=Escherichia coli (strain K12) OX=83333 GN=frwC PE=3 SV=1
orf_660	AL591824.1	group_2344	698785	699420	+	COG2231	L;3-Methyladenine DNA glycosylase, HhH-G	-	Putative DNA repair glycosylase MJ1434 OS=Methanocaldococcus jannaschii (strain ATCC 43067 / DSM 2661 / JAL-1 / JCM 10045 / NBRC 100440) OX=243232 GN=MJ1434 PE=1 SV=1
orf_661	AL591824.1	group_23	699410	700306	-	-	-	-	HTH-type transcriptional regulator rgg OS=Streptococcus gordonii (strain Challis / ATCC 35105 / BCRC 15272 / CH1 / DL1 / V288) OX=467705 GN=rgg PE=4 SV=1
orf_662	AL591824.1	group_19	700537	700821	+	COG2963	X;Transposase InsE and inactivated derivati	-	-
orf_663	AL591824.1	group_2191	700876	701184	-	COG0599	R;Uncharacterized conserved protein YurZ,	-	-
orf_672	AL591824.1	group_2038	706411	707292	+	COG1028	I;NAD(P)-dependent dehydrogenase, short	-	Uncharacterized oxidoreductase YhxD OS=Bacillus subtilis (strain 168) OX=224308 GN=yhxD PE=3 SV=2
orf_705	AL591824.1	group_2165	735996	737162	+	-	-	-	-
orf_708	AL591824.1	group_1697	738191	739711	+	COG1256	N;Flagellar hook-associated protein FlgK;Fl	-	Flagellar hook-associated protein 1 OS=Escherichia coli (strain K12) OX=83333 GN=flgK PE=1 SV=3
orf_720	AL591824.1	group_1613	748537	749205	+	COG0741	M;Soluble lytic murein transglycosylase or	-	Putative murein lytic transglycosylase YjbJ OS=Bacillus subtilis (strain 168) OX=224308 GN=yjbJ PE=3 SV=1
orf_725	AL591824.1	group_1663	751598	753328	+	COG0028	EH;Acetolactate synthase large subunit or	-	Putative thiamine pyrophosphate-containing protein YdaP OS=Bacillus subtilis (strain 168) OX=224308 GN=ydaP PE=2 SV=1
orf_732	AL591824.1	group_2291	759479	759958	+	-	-	-	-
orf_742	AL591824.1	group_8	769639	771012	+	COG2723	G;Beta-glucosidase/6-phospho-beta-glucos	-	6-phospho-beta-glucosidase OS=Clostridium longisporum OX=1523 GN=abgA PE=3 SV=1
orf_747	AL591824.1	group_2269	773715	775715	+	COG4591	M;ABC-type transport system involved in li	-	Macrolide export ATP-binding/permease protein MacB 2 OS=Aeromonas hydrophila subsp. hydrophila (strain ATCC 7966 / DSM 30187 / BCRC 13018 / CCUG 14551 / JCM 1027 / KCTC 2358 / NCIMB 9240 / NCTC 8049) OX=380703 GN=macB2 PE=3 SV=1
orf_758	AL591824.1	group_2251	782794	783774	+	COG1277	O;ABC-type transport system involved in m	-	Uncharacterized protein YhcI OS=Bacillus subtilis (strain 168) OX=224308 GN=yhcI PE=4 SV=1
orf_773	AL591824.1	group_1358	798141	798788	+	COG1802	K;DNA-binding transcriptional regulator, Gr	-	Uncharacterized HTH-type transcriptional regulator YdhC OS=Bacillus subtilis (strain 168) OX=224308 GN=ydhC PE=4 SV=1
orf_823	AL591824.1	group_1674	848804	849193	-	COG0789	K;DNA-binding transcriptional regulator, M	-	Uncharacterized HTH-type transcriptional regulator HI_0186 OS=Haemophilus influenzae (strain ATCC 51907 / DSM 11121 / KW20 / Rd) OX=71421 GN=HI_0186 PE=1 SV=1
orf_833	AL591824.1	group_19	863322	863606	+	COG2963	X;Transposase InsE and inactivated derivati	-	-
orf_834	AL591824.1	group_23	863798	864688	+	-	-	-	HTH-type transcriptional regulator rgg OS=Streptococcus gordonii (strain Challis / ATCC 35105 / BCRC 15272 / CH1 / DL1 / V288) OX=467705 GN=rgg PE=4 SV=1
orf_843	AL591824.1	group_157	875721	881855	+	-	-	-	-
orf_849	AL591824.1	group_408	886875	887603	+	COG1126	E;ABC-type polar amino acid transport syst	-	Arginine transport ATP-binding protein ArtM OS=Bacillus subtilis (strain 168) OX=224308 GN=artM PE=1 SV=1
orf_850	AL591824.1	group_2284	887652	889502	-	COG0154	J;Asp-tRNAAsn/Glu-tRNAGln amidotransfer	-	Probable amidase At4g34880 OS=Arabidopsis thaliana OX=3702 GN=At4g34880 PE=2 SV=1
orf_862	AL591824.1	group_2176	899454	900287	+	COG0395	G;ABC-type glycerol-3-phosphate transport	-	Arabinooligosaccharides transport system permease protein AraQ OS=Bacillus subtilis (strain 168) OX=224308 GN=araQ PE=1 SV=2
orf_865	AL591824.1	group_2154	902773	903840	+	COG2152	G;Predicted glycosyl hydrolase, GH43/DUF	-	Beta-1,2-mannobiose phosphorylase OS=Listeria innocua serovar 6a (strain ATCC BAA-680 / CLIP 11262) OX=272626 GN=lin0857 PE=1 SV=1
orf_873	AL591824.1	group_1597	910998	912164	+	COG2814	G;Predicted arabinose efflux permease Ara	-	Uncharacterized MFS-type transporter YtbD OS=Bacillus subtilis (strain 168) OX=224308 GN=ytbD PE=3 SV=1
orf_881	AL591824.1	group_327	919020	920408	+	COG4932	M;Clumping factor A-related surface protei	-	Cell wall protein Lmo0880 OS=Listeria monocytogenes serovar 1/2a (strain ATCC BAA-679 / EGD-e) OX=169963 GN=lmo0880 PE=1 SV=1
orf_933	AL591824.1	group_154	969549	970496	-	COG0463	M;Glycosyltransferase involved in cell wall	-	Uncharacterized glycosyltransferase YkcC OS=Bacillus subtilis (strain 168) OX=224308 GN=ykcC PE=3 SV=2
orf_944	AL591824.1	group_1710	979762	980064	+	COG4841	S;Uncharacterized conserved protein YneR,	-	Uncharacterized protein YneR OS=Bacillus subtilis (strain 168) OX=224308 GN=yneR PE=3 SV=1
orf_958	AL591824.1	group_137	990968	991690	+	COG2188	K;DNA-binding transcriptional regulator, Gr	-	HTH-type transcriptional repressor NagR OS=Bacillus subtilis (strain 168) OX=224308 GN=nagR PE=1 SV=1
orf_973	AL591824.1	group_1425	1003855	1005039	-	COG1696	M;D-alanyl-lipoteichoic acid acyltransferas	-	Teichoic acid D-alanyltransferase OS=Bacillus subtilis (strain 168) OX=224308 GN=dltB PE=1 SV=1
orf_985	AL591824.1	group_1610	1015153	1015608	+	COG3279	KT;DNA-binding response regulator, LytR/A	-	Sensory transduction protein LytT OS=Enterococcus faecalis (strain ATCC 700802 / V583) OX=226185 GN=lytT PE=4 SV=1
orf_1046	AL591824.1	group_1888	1073552	1073800	+	COG1977	H;Molybdopterin synthase sulfur carrier su	-	Molybdopterin synthase sulfur carrier subunit OS=Dictyostelium discoideum OX=44689 GN=mocs2s PE=3 SV=1
orf_1077	AL591824.1	group_201	1106041	1107759	+	COG1705	MN;Flagellum-specific peptidoglycan hydrc	(aut) autolysin [Auto (VF0348) - Invasion (VFC0083)] [Listeria monocytogenes	Autolysin OS=Enterococcus faecalis (strain ATCC 700802 / V583) OX=226185 GN=EF_0799 PE=1 SV=2
orf_1129	AL591824.1	group_2348	1160778	1161230	+	COG0456	J;Ribosomal protein S18 acetylase RimI and	-	Peptidyl-lysine N-acetyltransferase YjaB OS=Escherichia coli (strain K12) OX=83333 GN=yjaB PE=1 SV=1
orf_1132	AL591824.1	group_2296	1162666	1163280	-	COG0456	J;Ribosomal protein S18 acetylase RimI and	-	-
orf_1133	AL591824.1	group_238	1163277	1163996	-	COG0789	K;DNA-binding transcriptional regulator, M	-	HTH-type transcriptional activator TipA OS=Streptomyces lividans OX=1916 GN=tipA PE=1 SV=1
orf_1138	AL591824.1	group_2366	1168390	1169541	+	-	-	-	-
orf_1172	AL591824.1	group_2039	1196097	1197182	+	COG0079	E;Histidinol-phosphate/aromatic aminotrar	-	Threonine-phosphate decarboxylase OS=Salmonella typhi OX=90370 GN=cobD PE=3 SV=1
orf_1181	AL591824.1	group_2055	1206590	1207111	+	COG4577	QC;Carboxysome shell and ethanolamine u	-	Bacterial microcompartment protein homohexamer OS=Haliangium ochraceum (strain DSM 14365 / JCM 11303 / SMP-2) OX=502025 GN=Hoch_5815 PE=1 SV=1
orf_1186	AL591824.1	group_2054	1210459	1211031	+	-	-	-	-
orf_1212	AL591824.1	group_2198	1232272	1232838	+	COG2096	H;Cob(II)alamin adenosyltransferase;PduO;	-	Corrinoid adenosyltransferase OS=Bacillus subtilis (strain 168) OX=224308 GN=yvqK PE=1 SV=2
orf_1284	AL591824.1	group_1793	1303027	1304436	+	COG1220	O;ATP-dependent protease HslVU (ClpYQ),	-	ATP-dependent protease ATPase subunit HslU OS=Listeria monocytogenes serovar 1/2a (strain ATCC BAA-679 / EGD-e) OX=169963 GN=hslU PE=3 SV=1
orf_1311	AL591824.1	group_1734	1334028	1334267	+	COG3763	S;Uncharacterized conserved protein YneF,	-	UPF0154 protein lin1344 OS=Listeria innocua serovar 6a (strain ATCC BAA-680 / CLIP 11262) OX=272626 GN=lin1344 PE=3 SV=1
orf_1333	AL591824.1	group_1617	1356769	1357683	+	COG0130	J;tRNA U55 pseudouridine synthase TruB, r	-	tRNA pseudouridine synthase B OS=Listeria monocytogenes serovar 1/2a (strain ATCC BAA-679 / EGD-e) OX=169963 GN=truB PE=3 SV=1
orf_1348	AL591824.1	group_2305	1369412	1369696	-	COG2165	NUW;Type II secretory pathway, pseudopil	-	-
orf_1393	AL591824.1	group_2141	1412455	1413294	-	COG0345	E;Pyrroline-5-carboxylate reductase;ProC;P	-	Pyrroline-5-carboxylate reductase 3 OS=Bacillus subtilis (strain 168) OX=224308 GN=proG PE=3 SV=2
orf_1573	AL591824.1	group_1698	1605895	1607016	-	COG0372	C;Citrate synthase;GltA;TCA cycle	-	Citrate synthase 2 OS=Bacillus subtilis (strain 168) OX=224308 GN=citZ PE=1 SV=2
orf_1607	AL591824.1	group_1083	1644106	1645191	-	COG2876	E;3-deoxy-D-arabino-heptulosonate 7-phos	-	Protein AroA(G) OS=Bacillus subtilis (strain 168) OX=224308 GN=aroA PE=1 SV=1
orf_1670	AL591824.1	group_1935	1716858	1717160	+	-	-	-	-
orf_1679	AL591824.1	group_1587	1727824	1728651	-	COG0596	HR;2-succinyl-6-hydroxy-2,4-cyclohexadien	-	Putative 2-succinyl-6-hydroxy-2,4-cyclohexadiene-1-carboxylate synthase OS=Bacillus subtilis (strain 168) OX=224308 GN=menH PE=3 SV=2
orf_1683	AL591824.1	group_1628	1732926	1734779	-	COG0685	E;5,10-methylenetetrahydrofolate reducta	-	Bifunctional homocysteine S-methyltransferase/5,10-methylenetetrahydrofolate reductase OS=Bacillus subtilis (strain 168) OX=224308 GN=yitJ PE=2 SV=1
orf_1782	AL591824.1	group_1622	1854077	1854682	-	COG1418	JR;HD superfamily phosphodieasterase, incl	-	Uncharacterized protein YpgQ OS=Bacillus subtilis (strain 168) OX=224308 GN=ypgQ PE=4 SV=1
orf_1784	AL591824.1	group_2149	1855571	1855933	+	-	-	-	-
orf_1794	AL591824.1	group_2357	1862423	1862893	+	COG0789	K;DNA-binding transcriptional regulator, M	-	Mercuric resistance operon regulatory protein (Fragment) OS=Acidithiobacillus ferrooxidans OX=920 GN=merR PE=4 SV=1
orf_1795	AL591824.1	group_2362	1862893	1863480	+	COG2249	R;Putative NADPH-quinone reductase (moc	-	NAD(P)H dehydrogenase [quinone] 1 OS=Cavia porcellus OX=10141 GN=NQO1 PE=2 SV=1
orf_1866	AL591824.1	group_265	1934765	1935298	-	COG0225	O;Peptide methionine sulfoxide reductase	-	Peptide methionine sulfoxide reductase MsrA OS=Listeria monocytogenes serovar 1/2a (strain ATCC BAA-679 / EGD-e) OX=169963 GN=msrA PE=3 SV=1
orf_1871	AL591824.1	group_2201	1938538	1939155	+	COG2905	T;Signal-transduction protein containing cA	-	Transcriptional repressor CcpN OS=Bacillus subtilis (strain 168) OX=224308 GN=ccpN PE=1 SV=1
orf_1873	AL591824.1	group_458	1939999	1942638	+	COG0574	G;Phosphoenolpyruvate synthase/pyruvate	-	Pyruvate, phosphate dikinase OS=Clostridium symbiosum OX=1512 GN=ppdK PE=1 SV=5
orf_1895	AL591824.1	group_2013	1962225	1962566	-	COG3599	D;Cell division septum initiation protein Div	-	Cell cycle protein GpsB OS=Listeria monocytogenes serovar 1/2a (strain ATCC BAA-679 / EGD-e) OX=169963 GN=gpsB PE=1 SV=1
orf_1919	AL591824.1	group_2196	1985218	1986345	+	COG2199	T;GGDEF domain, diguanylate cyclase (c-di-	-	Diguanylate cyclase A OS=Treponema denticola (strain ATCC 35405 / DSM 14222 / CIP 103919 / JCM 8153 / KCTC 15104) OX=243275 GN=dgcA PE=1 SV=1
orf_1920	AL591824.1	group_2188	1986342	1987346	+	-	-	-	Putative lipoprotein YdaJ OS=Bacillus subtilis (strain 168) OX=224308 GN=ydaJ PE=3 SV=1
orf_1938	AL591824.1	group_1727	2005953	2006666	-	COG2226	H;Ubiquinone/menaquinone biosynthesis C	-	Demethylmenaquinone methyltransferase OS=Listeria monocytogenes serovar 1/2a (strain ATCC BAA-679 / EGD-e) OX=169963 GN=menG PE=3 SV=1
orf_1946	AL591824.1	group_1655	2014086	2014760	-	COG0283	F;Cytidylate kinase;Cmk;	-	Cytidylate kinase OS=Listeria monocytogenes serovar 1/2a (strain ATCC BAA-679 / EGD-e) OX=169963 GN=cmk PE=3 SV=1
orf_1948	AL591824.1	group_2189	2015819	2016538	-	-	-	-	-
orf_1969	AL591824.1	group_2150	2036521	2037141	-	COG1309	K;DNA-binding protein, AcrR family, include	-	HTH-type transcriptional regulator RutR OS=Shigella flexneri OX=623 GN=rutR PE=3 SV=1
orf_1970	AL591824.1	group_2215	2037141	2037956	-	COG1277	O;ABC-type transport system involved in m	-	-
orf_1978	AL591824.1	group_163	2044481	2045749	-	COG3037	G;Ascorbate-specific PTS system IIC compo	-	Ascorbate-specific PTS system EIIC component OS=Salmonella typhimurium (strain LT2 / SGSC1412 / ATCC 700720) OX=99287 GN=ulaA PE=3 SV=2
orf_1979	AL591824.1	group_161	2045765	2046046	-	COG3414	G;Phosphotransferase system, galactitol-sp	-	Ascorbate-specific PTS system EIIB component OS=Mycoplasma pneumoniae (strain ATCC 29342 / M129) OX=272634 GN=ulaB PE=1 SV=1
orf_2006	AL591824.1	group_2045	2072205	2073269	-	COG0449	M;Glucosamine 6-phosphate synthetase, c	-	Glutamine--fructose-6-phosphate aminotransferase [isomerizing] OS=Encephalitozoon cuniculi (strain GB-M1) OX=284813 GN=GFA1 PE=1 SV=1
orf_2025	AL591824.1	group_1599	2096075	2097064	-	COG0253	E;Diaminopimelate epimerase;DapF;Lysine	-	Diaminopimelate epimerase OS=Listeria monocytogenes serovar 1/2a (strain ATCC BAA-679 / EGD-e) OX=169963 GN=dapF PE=3 SV=1
orf_2036	AL591824.1	group_1795	2110909	2111199	-	COG0762	O;Cytochrome b6 maturation protein CCB3	-	Uncharacterized membrane protein YlmG OS=Bacillus subtilis (strain 168) OX=224308 GN=ylmG PE=3 SV=1
orf_2090	AL591824.1	group_2246	2160538	2161164	-	COG4832	VT;Cyclopropanoid cyclopropyl hydrolase,	-	-
orf_2091	AL591824.1	group_216	2161161	2162054	-	COG3173	R;Predicted kinase, aminoglycoside phosph	-	-
orf_2093	AL591824.1	group_1588	2164106	2165377	-	COG1396	K;Transcriptional regulator, contains XRE-fa	-	-
orf_2152	AL591824.1	group_137	2229193	2229927	-	COG2188	K;DNA-binding transcriptional regulator, Gr	-	HTH-type transcriptional repressor NagR OS=Bacillus subtilis (strain 168) OX=224308 GN=nagR PE=1 SV=1
orf_2182	AL591824.1	group_2247	2256507	2257580	-	COG2199	T;GGDEF domain, diguanylate cyclase (c-di-	-	Uncharacterized protein YhcK OS=Bacillus subtilis (strain 168) OX=224308 GN=yhcK PE=4 SV=1
orf_2183	AL591824.1	group_2200	2257754	2258515	-	COG1028	I;NAD(P)-dependent dehydrogenase, short	-	3beta-hydroxysteroid dehydrogenase 2 OS=Eggerthella lenta (strain ATCC 25559 / DSM 2243 / CCUG 17323 / JCM 9979 / KCTC 3265 / NCTC 11813 / VPI 0255 / 1899 B) OX=479437 GN=Elen_1325 PE=1 SV=1
orf_2184	AL591824.1	group_220	2258697	2259260	+	COG1309	K;DNA-binding protein, AcrR family, include	-	Uncharacterized HTH-type transcriptional regulator YxbF OS=Bacillus subtilis (strain 168) OX=224308 GN=yxbF PE=4 SV=1
orf_2187	AL591824.1	group_2356	2264772	2268230	-	-	-	-	Internalin J OS=Listeria monocytogenes serotype 4b (strain F2365) OX=265669 GN=inlJ PE=3 SV=1
orf_2228	AL591824.1	group_1681	2307755	2308741	-	COG3481	J;3'-5' exoribonuclease YhaM, can participa	-	3'-5' exoribonuclease YhaM OS=Listeria monocytogenes serovar 1/2a (strain ATCC BAA-679 / EGD-e) OX=169963 GN=yhaM PE=3 SV=1
orf_2242	AL591824.1	group_2163	2324554	2325408	+	COG1082	G;Sugar phosphate isomerase/epimerase;Y	-	Inosose isomerase OS=Bacillus velezensis (strain DSM 23117 / BGSC 10A6 / LMG 26770 / FZB42) OX=326423 GN=iolI PE=3 SV=1
orf_2248	AL591824.1	group_2261	2332020	2332898	-	COG4152	R;ABC-type uncharacterized transport syste	-	ABC transporter ATP-binding protein YtrB OS=Bacillus subtilis (strain 168) OX=224308 GN=ytrB PE=2 SV=1
orf_2249	AL591824.1	group_2263	2332888	2333283	-	COG1725	K;DNA-binding transcriptional regulator Yh	-	HTH-type transcriptional repressor YtrA OS=Bacillus subtilis (strain 168) OX=224308 GN=ytrA PE=2 SV=1
orf_2355	AL591824.1	group_2308	2411900	2413222	-	COG2141	HR;Flavin-dependent oxidoreductase, lucif	-	Putative monooxygenase MoxC OS=Bacillus subtilis (strain 168) OX=224308 GN=moxC PE=1 SV=1
orf_2356	AL591824.1	group_2321	2413219	2413485	-	COG0695	O;Glutaredoxin;GrxC;	-	Putative glutaredoxin YtnI OS=Bacillus subtilis (strain 168) OX=224308 GN=ytnI PE=3 SV=1
orf_2357	AL591824.1	group_2319	2413478	2414473	-	COG2141	HR;Flavin-dependent oxidoreductase, lucif	-	Uncharacterized protein YtmO OS=Bacillus subtilis (strain 168) OX=224308 GN=ytmO PE=4 SV=1
orf_2359	AL591824.1	group_2332	2415246	2415953	-	COG0765	E;ABC-type amino acid transport system, p	-	L-cystine transport system permease protein TcyM OS=Bacillus subtilis (strain 168) OX=224308 GN=tcyM PE=1 SV=1
orf_2360	AL591824.1	group_2315	2415967	2416683	-	COG0765	E;ABC-type amino acid transport system, p	-	L-cystine transport system permease protein TcyL OS=Bacillus subtilis (strain 168) OX=224308 GN=tcyL PE=1 SV=1
orf_2361	AL591824.1	group_2290	2416705	2417514	-	COG0834	ET;ABC-type amino acid transport/signal tr	-	L-cystine-binding protein TcyK OS=Bacillus subtilis (strain 168) OX=224308 GN=tcyK PE=1 SV=1
orf_2362	AL591824.1	group_2327	2417531	2418067	-	COG1246	E;N-acetylglutamate synthase or related ac	-	Uncharacterized N-acetyltransferase YtmI OS=Bacillus subtilis (strain 168) OX=224308 GN=ytmI PE=1 SV=1
orf_2363	AL591824.1	group_2304	2418083	2418628	-	COG0431	C;NAD(P)H-dependent FMN reductase;SsuE	-	FMN reductase (NADPH) OS=Escherichia coli (strain K12) OX=83333 GN=ssuE PE=1 SV=2
orf_2364	AL591824.1	group_2326	2418871	2419770	+	COG0583	K;DNA-binding transcriptional regulator, Ly	-	HTH-type transcriptional regulator YtlI OS=Bacillus subtilis (strain 168) OX=224308 GN=ytlI PE=3 SV=1
orf_2372	AL591824.1	group_169	2428010	2430793	-	COG1511	S;Uncharacterized membrane protein YhgE	-	Uncharacterized protein YhgE OS=Bacillus subtilis (strain 168) OX=224308 GN=yhgE PE=4 SV=2
orf_2395	AL591824.1	group_1732	2459819	2460106	+	COG2212	P;Multisubunit Na+/H+ antiporter, MnhF su	-	Na(+)/H(+) antiporter subunit F OS=Bacillus subtilis (strain 168) OX=224308 GN=mrpF PE=1 SV=2
orf_2461	AL591824.1	group_1658	2522830	2523576	-	COG1647	Q;Esterase/lipase;YvaK;	-	Carboxylesterase OS=Bacillus subtilis (strain 168) OX=224308 GN=est PE=2 SV=2
orf_2478	AL591824.1	group_2187	2540421	2541857	-	COG3979	G;Chitodextrinase;;	-	GlcNAc-binding protein A OS=Shewanella oneidensis (strain MR-1) OX=211586 GN=gbpA PE=3 SV=2
orf_2491	AL591824.1	group_1606	2556013	2556510	-	COG0110	R;Acetyltransferase, isoleucine patch super	-	Putative acetyltransferase YvoF OS=Bacillus subtilis (strain 168) OX=224308 GN=yvoF PE=3 SV=1
orf_2497	AL591824.1	group_1685	2559841	2561052	-	COG3595	S;Uncharacterized conserved protein YvlB,	-	Uncharacterized protein YvlB OS=Bacillus subtilis (strain 168) OX=224308 GN=yvlB PE=4 SV=1
orf_2507	AL591824.1	group_1713	2571156	2571974	-	COG1117	P;ABC-type phosphate transport system, A	-	Phosphate import ATP-binding protein PstB 2 OS=Listeria monocytogenes serovar 1/2a (strain ATCC BAA-679 / EGD-e) OX=169963 GN=pstB2 PE=3 SV=1
orf_2540	AL591824.1	group_1718	2605697	2606101	-	COG0355	C;FoF1-type ATP synthase, epsilon subunit;	-	ATP synthase epsilon chain OS=Listeria monocytogenes serovar 1/2a (strain ATCC BAA-679 / EGD-e) OX=169963 GN=atpC PE=3 SV=1
orf_2562	AL591824.1	group_154	2625414	2626361	-	COG0463	M;Glycosyltransferase involved in cell wall	-	Uncharacterized glycosyltransferase sll0501 OS=Synechocystis sp. (strain PCC 6803 / Kazusa) OX=1111708 GN=sll0501 PE=1 SV=1
orf_2584	AL591824.1	group_1657	2649598	2650230	-	COG1335	HR;Nicotinamidase-related amidase;PncA;F	-	Nicotinamidase OS=Saccharomyces cerevisiae (strain ATCC 204508 / S288c) OX=559292 GN=PNC1 PE=1 SV=1
orf_2596	AL591824.1	group_2137	2663619	2664296	-	COG0745	TK;DNA-binding response regulator, OmpR	-	Heme response regulator HssR OS=Staphylococcus aureus (strain bovine RF122 / ET3-1) OX=273036 GN=hssR PE=3 SV=1
orf_2599	AL591824.1	group_1934	2665666	2668653	-	COG3383	R;Predicted molibdopterin-dependent oxid	-	Putative formate dehydrogenase YrhE OS=Bacillus subtilis (strain 168) OX=224308 GN=yrhE PE=3 SV=1
orf_2617	AL591824.1	group_2153	2690371	2691465	-	-	-	-	-
orf_2628	AL591824.1	group_2114	2697463	2697966	-	COG0098	J;Ribosomal protein S5;RpsE;Ribosome 30S	-	30S ribosomal protein S5 OS=Listeria welshimeri serovar 6b (strain ATCC 35897 / DSM 20650 / CIP 8149 / NCTC 11857 / SLCC 5334 / V8) OX=386043 GN=rpsE PE=3 SV=1
orf_2632	AL591824.1	group_1825	2699383	2699568	-	COG0199	J;Ribosomal protein S14;RpsN;Ribosome 3(	-	30S ribosomal protein S14 type Z OS=Listeria welshimeri serovar 6b (strain ATCC 35897 / DSM 20650 / CIP 8149 / NCTC 11857 / SLCC 5334 / V8) OX=386043 GN=rpsZ PE=3 SV=1
orf_2640	AL591824.1	group_1824	2702532	2702888	-	COG0091	J;Ribosomal protein L22;RplV;Ribosome 50	-	50S ribosomal protein L22 OS=Listeria welshimeri serovar 6b (strain ATCC 35897 / DSM 20650 / CIP 8149 / NCTC 11857 / SLCC 5334 / V8) OX=386043 GN=rplV PE=3 SV=1
orf_2645	AL591824.1	group_2112	2705082	2705711	-	COG0087	J;Ribosomal protein L3;RplC;Ribosome 50S	-	50S ribosomal protein L3 OS=Listeria monocytogenes serovar 1/2a (strain ATCC BAA-679 / EGD-e) OX=169963 GN=rplC PE=1 SV=1
orf_2662	AL591824.1	group_163	2721559	2722857	-	COG3037	G;Ascorbate-specific PTS system IIC compo	-	Ascorbate-specific PTS system EIIC component OS=Shigella flexneri OX=623 GN=ulaA PE=3 SV=2
orf_2663	AL591824.1	group_161	2722884	2723156	-	COG3414	G;Phosphotransferase system, galactitol-sp	-	Ascorbate-specific PTS system EIIB component OS=Mycoplasma pneumoniae (strain ATCC 29342 / M129) OX=272634 GN=ulaB PE=1 SV=1
orf_2664	AL591824.1	group_139	2723159	2723593	-	COG1762	GT;Phosphotransferase system mannitol/fr	-	Ascorbate-specific PTS system EIIA component OS=Escherichia coli (strain K12) OX=83333 GN=ulaC PE=1 SV=1
orf_2671	AL591824.1	group_1592	2731976	2732455	-	COG0454	KR;N-acetyltransferase, GNAT superfamily	-	N-acetyltransferase ats1 OS=Schizosaccharomyces pombe (strain 972 / ATCC 24843) OX=284812 GN=ats1 PE=2 SV=1
orf_2677	AL591824.1	group_133	2737630	2738682	-	COG1063	ER;Threonine dehydrogenase or related Zn	-	Galactitol 1-phosphate 5-dehydrogenase OS=Escherichia coli (strain K12) OX=83333 GN=gatD PE=1 SV=1
orf_2690	AL591824.1	group_2211	2748685	2749497	-	COG0596	HR;2-succinyl-6-hydroxy-2,4-cyclohexadien	-	Uncharacterized hydrolase YugF OS=Bacillus subtilis (strain 168) OX=224308 GN=yugF PE=3 SV=1
orf_2695	AL591824.1	group_1974	2755580	2757265	-	COG2060	P;K+-transporting ATPase, KdpA subunit;Kd	-	Potassium-transporting ATPase potassium-binding subunit OS=Listeria monocytogenes serovar 1/2a (strain ATCC BAA-679 / EGD-e) OX=169963 GN=kdpA PE=3 SV=1
orf_2702	AL591824.1	group_1854	2761951	2763081	-	COG0772	D;Peptodoglycan polymerase FtsW/RodA/S	-	Probable peptidoglycan glycosyltransferase FtsW OS=Bacillus subtilis (strain 168) OX=224308 GN=ftsW PE=1 SV=1
orf_2706	AL591824.1	group_262	2766935	2768707	+	COG1705	MN;Flagellum-specific peptidoglycan hydrc	-	Muramidase-2 OS=Enterococcus hirae (strain ATCC 9790 / DSM 20160 / JCM 8729 / LMG 6399 / NBRC 3181 / NCIMB 6459 / NCDO 1258 / NCTC 12367 / WDCM 00089 / R) OX=768486 GN=EHR_05900 PE=1 SV=1
orf_2725	AL591824.1	group_1782	2783466	2784107	-	-	-	-	-
orf_2726	AL591824.1	group_2282	2784652	2784747	-	-	-	-	-
orf_2749	AL591824.1	group_22	2807459	2810107	+	COG0383	G;Alpha-mannosidase;MngB;	-	Mannosylglycerate hydrolase OS=Escherichia coli (strain K12) OX=83333 GN=mngB PE=1 SV=2
orf_2753	AL591824.1	group_1643	2813949	2815283	-	COG1253	R;Hemolysin-related protein, contains CBS	-	UPF0053 protein YhdP OS=Bacillus subtilis (strain 168) OX=224308 GN=yhdP PE=3 SV=1
orf_2756	AL591824.1	group_1391	2816363	2817571	-	COG2814	G;Predicted arabinose efflux permease Ara	-	Multidrug resistance protein MdtG OS=Erwinia billingiae (strain Eb661) OX=634500 GN=mdtG PE=3 SV=1
orf_2760	AL591824.1	group_843	2819860	2821587	+	COG1132	V;ABC-type multidrug transport system, AT	-	Multidrug resistance ABC transporter ATP-binding/permease protein BmrA OS=Bacillus subtilis (strain 168) OX=224308 GN=bmrA PE=1 SV=1
orf_2761	AL591824.1	group_2133	2821641	2821892	-	COG3070	K;Transcriptional regulator of competence	-	DNA transformation protein TfoX2 OS=Aliivibrio fischeri (strain ATCC 700601 / ES114) OX=312309 GN=tfoX2 PE=3 SV=1
orf_2771	AL591824.1	group_233	2833834	2835987	-	COG0551	L;DNA topoisomerase I, ssDNA-binding Zn-f	-	DNA topoisomerase 3 OS=Bacillus thuringiensis subsp. konkukian (strain 97-27) OX=281309 GN=topB PE=3 SV=1
orf_2772	AL591824.1	group_1781	2836011	2837783	-	COG0514	L;Superfamily II DNA helicase RecQ;RecQ;	-	Probable ATP-dependent DNA helicase RecQ OS=Bacillus subtilis (strain 168) OX=224308 GN=recQ PE=3 SV=1
orf_2777	AL591824.1	group_8	2842413	2843867	+	COG2723	G;Beta-glucosidase/6-phospho-beta-glucos	-	Aryl-phospho-beta-D-glucosidase BglC OS=Bacillus subtilis (strain 168) OX=224308 GN=bglC PE=1 SV=1
orf_2794	AL591824.1	group_2285	2861424	2862614	+	COG2814	G;Predicted arabinose efflux permease Ara	-	Uncharacterized transporter RF_0358 OS=Rickettsia felis (strain ATCC VR-1525 / URRWXCal2) OX=315456 GN=RF_0358 PE=3 SV=1
orf_2796	AL591824.1	group_1111	2863561	2864661	-	COG0012	J;Ribosome-binding ATPase YchF, GTP1/OB	-	Ribosome-binding ATPase YchF OS=Bacillus subtilis (strain 168) OX=224308 GN=ychF PE=2 SV=1
orf_2807	AL591824.1	group_1944	2877160	2878011	-	COG1475	D;Chromosome segregation protein Spo0J,	-	Stage 0 sporulation protein J OS=Bacillus subtilis (strain 168) OX=224308 GN=spo0J PE=1 SV=2
orf_2813	AL591824.1	group_2311	2882033	2882938	-	COG1940	GK;Sugar kinase of the NBD/HSP70 family,	-	Uncharacterized protein CPE0188 OS=Clostridium perfringens (strain 13 / Type A) OX=195102 GN=CPE0188 PE=3 SV=2
orf_2814	AL591824.1	group_139	2882963	2883409	-	COG1762	GT;Phosphotransferase system mannitol/fr	-	Putative phosphotransferase IIA component SgcA OS=Escherichia coli (strain K12) OX=83333 GN=sgcA PE=3 SV=1
orf_2815	AL591824.1	group_2337	2883422	2884078	-	COG0637	G;Beta-phosphoglucomutase, HAD superfa	-	Putative phosphatase YhcW OS=Bacillus subtilis (strain 168) OX=224308 GN=yhcW PE=3 SV=1
