# Supplementary material for: Comparative Genomics Reveal the Utilization Ability of Variable Carbohydrates as Key Genetic Features of Listeria Pathogens in Their Pathogenic Lifestyles
Source: Pathogens. 2022 Nov 28;11(12):1430. doi: 10.3390/pathogens11121430 (PMC9784484; doi:10.3390/pathogens11121430)
Supplement: Supplementary file 1 [file pathogens-11-01430-s001.zip › pathogens-1990707-supplementary/Supplymentary Tables S4.pdf]

Table S4. Unique genes of LMO referenced to *L.ivanovii* 1638

ORF_id	Sequence_id	Group_id	start	end	strand	COG_id	COG_annotation	VFDB_annotation	Uniprot_annotation
orf_22	CP034771.1	group_1724	23762	24055	-	COG0360	J;Ribosomal protein S6;RpsF;Ribosome 30S subunit	-	30S ribosomal protein S6 OS=Listeria welshimeri serovar 6b (strain ATCC 35897 / DSM 20650 / CIP 8149 / NCTC 11857 / SLCC 5334 / V8) OX=386043 GN=rpsF PE=3 SV=1
orf_24	CP034771.1	group_1801	25646	26305	-	COG0586	M;Membrane integrity protein DedA, putative transporter, DedA/T	-	Protein DedA OS=Escherichia coli (strain K12) OX=83333 GN=dedA PE=1 SV=1
orf_25	CP034771.1	group_2402	26407	27186	-	COG1737	K;DNA-binding transcriptional regulator, MurR/RpiR family, contair	-	Uncharacterized HTH-type transcriptional regulator HI_0143 OS=Haemophilus influenzae (strain ATCC 51907 / DSM 11121 / KW20 / Rd) OX=71421 GN=HI_0143 PE=4 SV=2
orf_26	CP034771.1	group_143	27204	28313	-	COG2957	E;Agmatine/peptidylarginine deiminase;AguA;	-	Putative agmatine deiminase 2 OS=Listeria monocytogenes serotype 4b (strain F2365) OX=265669 GN=aguA2 PE=3 SV=1
orf_27	CP034771.1	group_2391	28417	29361	-	COG0549	E;Carbamate kinase;ArcC;	-	Carbamate kinase OS=Latilactobacillus sakei OX=1599 GN=arcC PE=3 SV=1
orf_28	CP034771.1	group_143	29371	30465	-	COG2957	E;Agmatine/peptidylarginine deiminase;AguA;	-	Putative agmatine deiminase 1 OS=Listeria monocytogenes serotype 4b (strain F2365) OX=265669 GN=aguA1 PE=3 SV=1
orf_29	CP034771.1	group_2397	30462	31835	-	COG0531	E;Serine transporter YbeC, amino acid:H+ symporter family;PotE;	-	Probable agmatine/putrescine antiporter AguD OS=Lactococcus lactis subsp. lactis (strain IL1403) OX=272623 GN=aguD PE=3 SV=1
orf_70	CP034771.1	group_2663	75484	81312	-	-	-	-	Internalin I OS=Listeria monocytogenes serotype 4b (strain F2365) OX=265669 GN=inlI PE=3 SV=1
orf_85	CP034771.1	group_2071	98192	98545	+	-	-	-	-
orf_86	CP034771.1	group_2231	98648	99070	+	COG1846	K;DNA-binding transcriptional regulator, MarR family;MarR;	-	HTH-type transcriptional regulator MhqR OS=Bacillus subtilis (strain 168) OX=224308 GN=mhqR PE=2 SV=1
orf_93	CP034771.1	group_2663	107156	109816	-	-	-	-	Pesticidal crystal protein Cry22Aa OS=Bacillus thuringiensis OX=1428 GN=cry22Aa PE=2 SV=1
orf_116	CP034771.1	group_1897	129433	129636	+	COG4481	S;Uncharacterized conserved protein, DUF951 family	-	Uncharacterized protein YyzM OS=Bacillus subtilis (strain 168) OX=224308 GN=yyzM PE=4 SV=1
orf_131	CP034771.1	group_2113	143282	144634	-	COG1455	G;Phosphotransferase system cellobiose-specific component IIC;Ce	-	PTS system N,N'-diacetylchitobiose-specific EIIC component OS=Escherichia coli (strain K12) OX=83333 GN=chbC PE=1 SV=2
orf_134	CP034771.1	group_2441	146703	146813	+	-	-	-	-
orf_157	CP034771.1	group_312	174795	175751	+	COG1609	K;DNA-binding transcriptional regulator, LacI/PurR family;PurR;	-	Catabolite control protein B OS=Bacillus subtilis (strain 168) OX=224308 GN=ccpB PE=1 SV=1
orf_184	CP034771.1	group_3706	198731	199204	-	-	-	-	-
orf_185	CP034771.1	group_2058	199355	199519	-	-	-	-	-
orf_197	CP034771.1	group_284	208364	210631	+	-	-	-	Alpha-xylosidase OS=Emericella nidulans (strain FGSC A4 / ATCC 38163 / CBS 112.46 / NRRL 194 / M139) OX=227321 GN=agdD PE=1 SV=1
orf_202	CP034771.1	group_2683	214067	214507	+	COG0494	V;8-oxo-dGTP pyrophosphatase MutT and related house-cleaning N	-	-
orf_241	CP034771.1	group_1876	255606	256709	+	COG0772	D;Peptodoglycan polymerase FtsW/RodA/SpoVE;FtsW;	-	Peptidoglycan glycosyltransferase RodA OS=Bacillus subtilis (strain 168) OX=224308 GN=rodA PE=1 SV=1
orf_257	CP034771.1	group_2384	271553	271921	+	-	-	-	-
orf_301	CP034771.1	group_1642	316258	317337	+	COG1477	HO;FAD:protein FMN transferase ApbE;ApbE;	-	FAD:protein FMN transferase OS=Klebsiella pneumoniae (strain 342) OX=507522 GN=apbE2 PE=3 SV=1
orf_344	CP034771.1	group_3446	344306	344671	-	-	-	-	-
orf_345	CP034771.1	group_2131	345077	346603	-	COG1705	MN;Flagellum-specific peptidoglycan hydrolase FlgJ;FlgJ;	-	Protein Ami OS=Listeria monocytogenes serotype 1/2a (strain EGD / Mackaness) OX=1334565 GN=ami PE=3 SV=1
orf_346	CP034771.1	group_2140	347011	348039	+	COG0489	D;Fe-S cluster carrier ATPase, Mrp/ApbC/NBP35 family;Mrp;	-	Iron-sulfur cluster carrier protein OS=Bacillus subtilis (strain 168) OX=224308 GN=salA PE=1 SV=1
orf_347	CP034771.1	group_3720	348840	349781	+	COG4886	K;Leucine-rich repeat (LRR) protein;LRR;	-	Internalin C OS=Listeria monocytogenes serotype 1/2a (strain EGD / Mackaness) OX=1334565 GN=inlC PE=1 SV=1
orf_348	CP034771.1	group_2370	350212	351102	+	COG4886	K;Leucine-rich repeat (LRR) protein;LRR;	(inlC) internalin C [InlC (VF0438) - Immune modulation (VFC0258)] [Listeria monocytogene	Internalin C OS=Listeria monocytogenes serotype 1/2a (strain EGD / Mackaness) OX=1334565 GN=inlC PE=1 SV=1
orf_349	CP034771.1	group_3529	351503	351775	+	-	-	-	-
orf_356	CP034771.1	group_3606	360032	360385	-	-	-	-	Uncharacterized HTH-type transcriptional regulator YdzF OS=Bacillus subtilis (strain 168) OX=224308 GN=ydzF PE=4 SV=1
orf_362	CP034771.1	group_1593	364892	365158	+	-	-	-	-
orf_388	CP034771.1	group_2117	389953	390387	+	COG1917	R;Cupin domain protein related to quercetin dioxygenase;QdoI;	-	-
orf_389	CP034771.1	group_3532	390384	390707	+	COG0599	R;Uncharacterized conserved protein YurZ, alkylhydroperoxidase/c	-	4-carboxymuconolactone decarboxylase OS=Acinetobacter baylyi (strain ATCC 33305 / BD413 / ADP1) OX=62977 GN=pcaC PE=4 SV=2
orf_390	CP034771.1	group_3482	390734	391903	+	-	-	-	-
orf_475	CP034771.1	group_2240	479464	479799	-	COG4269	S;Uncharacterized membrane protein YjgN, DUF898 family;YjgN	-	-
orf_495	CP034771.1	group_62	501116	504391	+	COG1501	G;Alpha-glucosidase/xylosidase, GH31 family;YicI;	-	1,3-alpha-isomaltosidase OS=Kribbella flavida (strain DSM 17836 / JCM 10339 / NBRC 14399) OX=479435 GN=Kfla_1895 PE=1 SV=1
orf_496	CP034771.1	group_2313	504450	505394	+	-	-	-	Internalin C OS=Listeria monocytogenes serotype 1/2a (strain EGD / Mackaness) OX=1334565 GN=inlC PE=1 SV=1
orf_497	CP034771.1	group_305	505407	507026	+	COG1501	G;Alpha-glucosidase/xylosidase, GH31 family;YicI;	-	Alpha-xylosidase OS=Saccharolobus solfataricus (strain ATCC 35092 / DSM 1617 / JCM 11322 / P2) OX=273057 GN=xylS PE=1 SV=1
orf_498	CP034771.1	group_305	507036	509333	+	COG1501	G;Alpha-glucosidase/xylosidase, GH31 family;YicI;	-	Alpha-glucosidase 2 OS=Bacillus thermoamyloliquefaciens OX=1425 PE=3 SV=1
orf_509	CP034771.1	group_2164	517802	517966	-	-	-	-	-
orf_534	CP034771.1	group_1805	539718	540083	-	COG4272	S;Uncharacterized membrane protein	-	-
orf_544	CP034771.1	group_1693	547103	547336	+	COG4844	S;Uncharacterized conserved protein YuzB, UPF0349 family;YuzB	-	UPF0349 protein lwe2340 OS=Listeria welshimeri serovar 6b (strain ATCC 35897 / DSM 20650 / CIP 8149 / NCTC 11857 / SLCC 5334 / V8) OX=386043 GN=lwe2340 PE=3 SV=1
orf_549	CP034771.1	group_2586	551841	552470	+	-	-	-	-
orf_582	CP034771.1	group_2035	587994	589598	+	COG1574	R;Predicted amidohydrolase YtcJ;YtcJ;	-	Putative amidohydrolase YtcJ OS=Bacillus subtilis (strain 168) OX=224308 GN=ytcJ PE=3 SV=1
orf_599	CP034771.1	group_1812	610541	611443	+	COG1284	S;Uncharacterized membrane-anchored protein YitT, contains DUF	-	UPF0750 membrane protein YitT OS=Bacillus subtilis (strain 168) OX=224308 GN=yitT PE=3 SV=2
orf_603	CP034771.1	group_1845	613688	614563	+	COG2153	R;Predicted N-acyltransferase, GNAT family;ElaA;	-	-
orf_606	CP034771.1	group_1809	615744	616265	+	COG0693	V;Protein/nucleotide deglycase, PfpI/YajL/DJ-1 family (repair of me	-	Uncharacterized protein AF_1281 OS=Archaeoglobus fulgidus (strain ATCC 49558 / DSM 4304 / JCM 9628 / NBRC 100126 / VC-16) OX=224325 GN=AF_1281 PE=3 SV=1
orf_609	CP034771.1	group_2145	618149	618778	+	COG0637	G;Beta-phosphoglucomutase, HAD superfamily;YcjU;	-	Putative phosphatase YhcW OS=Bacillus subtilis (strain 168) OX=224308 GN=yhcW PE=3 SV=1
orf_622	CP034771.1	group_2185	629307	629861	-	COG2169	L;Methylphosphotriester-DNA--protein-cysteine methyltransferase	-	Bifunctional transcriptional activator/DNA repair enzyme AdaA OS=Bacillus subtilis (strain 168) OX=224308 GN=adaA PE=1 SV=1
orf_664	CP034771.1	group_1694	672963	673958	-	COG0180	J;Tryptophanyl-tRNA synthetase;TrpS;Aminoacyl-tRNA synthetases	-	Tryptophan--tRNA ligase OS=Listeria innocua serovar 6a (strain ATCC BAA-680 / CLIP 11262) OX=272626 GN=trpS PE=3 SV=1
orf_692	CP034771.1	group_2242	715119	715277	-	-	-	-	-
orf_712	CP034771.1	group_1797	730285	730926	-	COG4330	S;Uncharacterized membrane protein, DUF1361 domain	-	-
orf_724	CP034771.1	group_2146	741693	742547	+	COG0191	G;Fructose/tagatose bisphosphate aldolase;Fba;Glycolysis	-	D-tagatose-1,6-bisphosphate aldolase subunit GatY OS=Escherichia coli (strain K12) OX=83333 GN=gatY PE=1 SV=1
orf_754	CP034771.1	group_2513	776707	777282	+	COG1136	M;ABC-type lipoprotein export system, ATPase component;LolD;	-	Uncharacterized ABC transporter ATP-binding protein YvrO OS=Bacillus subtilis (strain 168) OX=224308 GN=yvrO PE=3 SV=2
orf_768	CP034771.1	group_1937	790982	791449	+	COG1762	GT;Phosphotransferase system mannitol/fructose-specific IIA dom	-	PTS system galactitol-specific EIIA component OS=Escherichia coli (strain K12) OX=83333 GN=gatA PE=1 SV=1
orf_771	CP034771.1	group_2079	793125	794057	+	COG1105	G;1-phosphofructokinase or 6-phosphofructokinase II;FruK;	-	Tagatose-6-phosphate kinase OS=Staphylococcus epidermidis (strain ATCC 35984 / RP62A) OX=176279 GN=lacC PE=3 SV=1
orf_778	CP034771.1	group_3562	800654	801178	-	COG3177	K;Fic family protein;;	-	-
orf_811	CP034771.1	group_2349	831733	833637	+	COG2015	Q;Alkyl sulfatase BDS1 and related hydrolases, metallo-beta-lactam	-	Linear primary-alkylsulfatase OS=Pseudomonas sp. OX=306 GN=sdsAP PE=1 SV=1
orf_812	CP034771.1	group_2586	833891	834106	+	-	-	-	-
orf_901	CP034771.1	group_3491	931669	933315	+	-	-	-	Arylsulfate sulfotransferase AssT OS=Escherichia coli O6:H1 (strain CFT073 / ATCC 700928 / UPEC) OX=199310 GN=assT PE=1 SV=1
orf_906	CP034771.1	group_1760	937523	938410	+	COG1131	V;ABC-type multidrug transport system, ATPase component;CcmA;	-	Multidrug efflux system ATP-binding protein Rv1218c OS=Mycobacterium tuberculosis (strain ATCC 25618 / H37Rv) OX=83332 GN=Rv1218c PE=1 SV=1
orf_978	CP034771.1	group_3705	1009307	1009840	+	COG4912	L;3-methyladenine DNA glycosylase AlkD;AlkD;	-	-
orf_981	CP034771.1	group_1684	1011422	1011910	+	-	-	-	-
orf_982	CP034771.1	group_2438	1012002	1012739	-	COG0778	C;Nitroreductase;NfnB;Pyrimidine degradation	-	CR(VI) reductase OS=Pseudomonas sp. (strain G-1) OX=79677 GN=chrR PE=3 SV=1
orf_983	CP034771.1	group_2437	1012959	1013822	+	COG0583	K;DNA-binding transcriptional regulator, LysR family;LysR;	-	HTH-type transcriptional regulator CzcR OS=Bacillus subtilis (strain 168) OX=224308 GN=czcR PE=3 SV=2
orf_986	CP034771.1	group_1707	1017310	1017675	+	-	-	-	Uncharacterized protein YppE OS=Bacillus subtilis (strain 168) OX=224308 GN=yppE PE=1 SV=1
orf_1005	CP034771.1	group_2447	1036313	1037674	+	-	-	-	Glutamyl endopeptidase OS=Bacillus licheniformis (strain ATCC 14580 / DSM 13 / JCM 2505 / CCUG 7422 / NBRC 12200 / NCIMB 9375 / NCTC 10341 / NRRL NRS-1264 / Gibson 46) OX=279010 GN=blaSE PE=1 SV=2
orf_1102	CP034771.1	group_1641	1128849	1129973	+	COG0026	F;Phosphoribosylaminoimidazole carboxylase (NCAIR synthetase);F	-	N5-carboxyaminoimidazole ribonucleotide synthase OS=Bacillus subtilis (strain 168) OX=224308 GN=purK PE=3 SV=2
orf_1115	CP034771.1	group_2158	1141850	1143205	-	COG0733	R;Na+-dependent transporter, SNF family;YocR;	-	Uncharacterized sodium-dependent transporter YocR OS=Bacillus subtilis (strain 168) OX=224308 GN=yocR PE=3 SV=1
orf_1116	CP034771.1	group_1640	1143411	1144088	+	COG1646	I;Glycerol-1-phosphate heptaprenyltransferase;PcrB;	-	Heptaprenylglyceryl phosphate synthase OS=Listeria innocua serovar 6a (strain ATCC BAA-680 / CLIP 11262) OX=272626 GN=pcrB PE=3 SV=1
orf_1118	CP034771.1	group_1661	1146372	1148387	+	COG0272	L;NAD-dependent DNA ligase;Lig;	-	DNA ligase OS=Listeria monocytogenes serotype 4a (strain HCC23) OX=552536 GN=ligA PE=3 SV=1
orf_1122	CP034771.1	group_1811	1151405	1152835	+	COG0064	J;Asp-tRNAAsn/Glu-tRNAGln amidotransferase B subunit;GatB;Ami	-	Aspartyl/glutamyl-tRNA(Asn/Gln) amidotransferase subunit B OS=Listeria welshimeri serovar 6b (strain ATCC 35897 / DSM 20650 / CIP 8149 / NCTC 11857 / SLCC 5334 / V8) OX=386043 GN=gatB PE=3 SV=1
orf_1124	CP034771.1	group_1883	1153954	1154814	+	-	-	-	-
orf_1126	CP034771.1	group_2052	1156549	1157103	+	COG4884	S;Uncharacterized conserved protein YfeS, contains WGR domain;Y	-	Uncharacterized protein YfeS OS=Escherichia coli (strain K12) OX=83333 GN=yfeS PE=4 SV=1
orf_1127	CP034771.1	group_1639	1157131	1157661	-	COG0703	E;Shikimate kinase;AroK;Aromatic amino acid biosynthesis	-	Shikimate kinase OS=Enterococcus faecalis (strain ATCC 700802 / V583) OX=226185 GN=aroK PE=3 SV=1
orf_1128	CP034771.1	group_1633	1157840	1158343	+	-	-	-	-
orf_1129	CP034771.1	group_2422	1158367	1159254	+	COG0388	C;Omega-amidase YafV/Nit2, hydrolyzes alpha-ketoglutaramate;Ni	-	N-carbamoylputrescine amidase OS=Arabidopsis thaliana OX=3702 GN=CPA PE=1 SV=2
orf_1130	CP034771.1	group_2098	1159408	1160175	+	COG1136	M;ABC-type lipoprotein export system, ATPase component;LolD;	-	Bacitracin export ATP-binding protein BceA OS=Bacillus subtilis (strain 168) OX=224308 GN=bceA PE=1 SV=1
orf_1132	CP034771.1	group_1728	1162206	1162883	+	COG0745	TK;DNA-binding response regulator, OmpR family, contains REC an	-	Sensory transduction protein BceR OS=Alkalihalobacillus halodurans (strain ATCC BAA-125 / DSM 18197 / FERM 7344 / JCM 9153 / C-125) OX=272558 GN=bceR PE=3 SV=1
orf_1136	CP034771.1	group_1668	1166086	1167126	+	COG0642	T;Signal transduction histidine kinase;BaeS;	-	Sensor protein BceS OS=Alkalihalobacillus halodurans (strain ATCC BAA-125 / DSM 18197 / FERM 7344 / JCM 9153 / C-125) OX=272558 GN=bceS PE=3 SV=1
orf_1137	CP034771.1	group_1695	1167522	1168163	+	COG0765	E;ABC-type amino acid transport system, permease component;His	-	Probable amino-acid ABC transporter permease protein YckA OS=Bacillus subtilis (strain 168) OX=224308 GN=yckA PE=3 SV=1
orf_1138	CP034771.1	group_299	1168173	1168820	+	COG1126	E;ABC-type polar amino acid transport system, ATPase component	-	Glutamine transport ATP-binding protein GlnQ OS=Escherichia coli (strain K12) OX=83333 GN=glnQ PE=1 SV=1
orf_1139	CP034771.1	group_1777	1168821	1169636	+	COG0834	ET;ABC-type amino acid transport/signal transduction system, peri	-	L-cystine-binding protein TcyJ OS=Escherichia coli (strain K12) OX=83333 GN=tcyJ PE=1 SV=1
orf_1141	CP034771.1	group_2127	1170837	1171355	+	COG3153	R;Predicted N-acetyltransferase YhbS;yhbS;	-	Uncharacterized N-acetyltransferase YjhQ OS=Escherichia coli (strain K12) OX=83333 GN=yjhQ PE=3 SV=1
orf_1146	CP034771.1	group_1701	1179407	1180300	-	COG1175	G;ABC-type sugar transport system, permease component;UgpA;	-	Probable ABC transporter permease protein YurN OS=Bacillus subtilis (strain 168) OX=224308 GN=yurN PE=3 SV=1
orf_1153	CP034771.1	group_2455	1190002	1190682	+	COG1131	V;ABC-type multidrug transport system, ATPase component;CcmA;	-	Uncharacterized ABC transporter ATP-binding protein YhcG OS=Bacillus subtilis (strain 168) OX=224308 GN=yhcG PE=3 SV=1
orf_1154	CP034771.1	group_2454	1190675	1191448	+	-	-	-	-
orf_1162	CP034771.1	group_3496	1201253	1202593	+	COG4886	K;Leucine-rich repeat (LRR) protein;LRR;	-	Internalin C OS=Listeria monocytogenes serotype 1/2a (strain EGD / Mackaness) OX=1334565 GN=inlC PE=1 SV=1
orf_1164	CP034771.1	group_1408	1204386	1205615	-	COG3104	E;Dipeptide/tripeptide permease;PTR2;	-	Na(+), Li(+), K(+)/H(+) antiporter OS=Planococcus maritimus OX=192421 GN=mdrP PE=1 SV=1
orf_1174	CP034771.1	group_2209	1213260	1213667	+	COG0346	Q;Catechol 2,3-dioxygenase or related enzyme, vicinal oxygen chel	-	Fosfomycin resistance protein FosX OS=Listeria monocytogenes serovar 1/2a (strain ATCC BAA-679 / EGD-e) OX=169963 GN=fosX PE=1 SV=1
orf_1176	CP034771.1	group_2105	1214086	1214292	-	-	-	-	-
orf_1177	CP034771.1	group_1798	1214322	1215224	-	COG0840	T;Methyl-accepting chemotaxis protein (MCP);Tar;	-	Biofilm dispersion protein BdlA OS=Pseudomonas aeruginosa (strain ATCC 15692 / DSM 22644 / CIP 104116 / JCM 14847 / LMG 12228 / 1C / PRS 101 / PAO1) OX=208964 GN=bdlA PE=1 SV=1
orf_1180	CP034771.1	group_1779	1217188	1217862	+	COG4767	V;Glycopeptide antibiotics resistance protein;VanZ	-	-
orf_1187	CP034771.1	group_1986	1223506	1224486	-	COG1988	R;Membrane-bound metal-dependent hydrolase YbcI, DUF457 fam	-	Uncharacterized protein YfhP OS=Bacillus subtilis (strain 168) OX=224308 GN=yfhP PE=2 SV=1
orf_1189	CP034771.1	group_1659	1225792	1226538	+	COG1028	I;NAD(P)-dependent dehydrogenase, short-chain alcohol dehydrog	-	Enoyl-[acyl-carrier-protein] reductase [NADPH] FabL OS=Bacillus subtilis (strain 168) OX=224308 GN=fabL PE=1 SV=1
orf_1221	CP034771.1	group_2591	1263556	1264440	-	COG2103	M;N-acetylmuramic acid 6-phosphate (MurNAc-6-P) etherase;Mur	-	N-acetylmuramic acid 6-phosphate etherase OS=Listeria monocytogenes serotype 4b (strain F2365) OX=265669 GN=murQ PE=3 SV=1
orf_1240	CP034771.1	group_2061	1281757	1282881	+	COG0420	L;DNA repair exonuclease SbcCD nuclease subunit;SbcD;	-	Nuclease SbcCD subunit D OS=Staphylococcus epidermidis (strain ATCC 12228 / FDA PCI 1200) OX=176280 GN=sbcD PE=3 SV=1
orf_1246	CP034771.1	group_2192	1293447	1293680	+	-	-	-	-
orf_1316	CP034771.1	group_3659	1365221	1366924	-	COG2272	I;Carboxylesterase type B;PnbA;	-	Para-nitrobenzyl esterase OS=Bacillus subtilis (strain 168) OX=224308 GN=pnbA PE=1 SV=2
orf_1324	CP034771.1	group_1938	1377061	1377945	+	COG0777	I;Acetyl-CoA carboxylase beta subunit;AccD;Fatty acid biosynthesis	-	Acetyl-coenzyme A carboxylase carboxyl transferase subunit beta OS=Listeria welshimeri serovar 6b (strain ATCC 35897 / DSM 20650 / CIP 8149 / NCTC 11857 / SLCC 5334 / V8) OX=386043 GN=accD PE=3 SV=1
orf_1345	CP034771.1	group_1665	1401478	1404126	+	COG0525	J;Valyl-tRNA synthetase;ValS;Aminoacyl-tRNA synthetases	-	Valine--tRNA ligase OS=Listeria innocua serovar 6a (strain ATCC BAA-680 / CLIP 11262) OX=272626 GN=valS PE=3 SV=1
orf_1408	CP034771.1	group_789	1467994	1468815	+	COG0169	E;Shikimate 5-dehydrogenase;AroE;Aromatic amino acid biosynthe	-	Shikimate dehydrogenase (NADP(+)) OS=Bacillus cereus (strain AH820) OX=405535 GN=aroE PE=3 SV=1
orf_1420	CP034771.1	group_2355	1478666	1478899	+	COG0451	M;Nucleoside-diphosphate-sugar epimerase;WcaG;	-	-
orf_1469	CP034771.1	group_1711	1528381	1529037	+	COG1174	E;ABC-type proline/glycine betaine transport system, permease co	-	Carnitine transport permease protein OpuCB OS=Listeria monocytogenes OX=1639 GN=opuCB PE=1 SV=1
orf_1470	CP034771.1	group_1813	1529039	1529965	+	COG1732	M;Periplasmic glycine betaine/choline-binding (lipo)protein of an A	-	Carnitine transport binding protein OpuCC OS=Listeria monocytogenes OX=1639 GN=opuCC PE=1 SV=1
orf_1473	CP034771.1	group_1794	1532247	1533077	+	-	-	-	-
orf_1478	CP034771.1	group_2121	1537861	1539129	+	COG1524	T;c-di-AMP phosphodiesterase AtaC or nucleotide pyrophosphatas	-	Ectonucleotide pyrophosphatase/phosphodiesterase family member 1 OS=Homo sapiens OX=9606 GN=ENPP1 PE=1 SV=2
orf_1479	CP034771.1	group_2081	1539149	1540393	+	COG2270	R;MFS-type transporter involved in bile tolerance, Atg22 family;Btl	-	Uncharacterized MFS-type transporter YxiO OS=Bacillus subtilis (strain 168) OX=224308 GN=yxiO PE=3 SV=1
orf_1480	CP034771.1	group_2067	1540463	1541062	+	COG4767	V;Glycopeptide antibiotics resistance protein;VanZ	-	-
orf_1484	CP034771.1	group_2663	1545218	1546681	+	-	-	-	-
orf_1485	CP034771.1	group_2663	1546669	1551294	+	-	-	-	Internalin I OS=Listeria monocytogenes serovar 1/2a (strain ATCC BAA-679 / EGD-e) OX=169963 GN=inlI PE=2 SV=1
orf_1486	CP034771.1	group_2360	1551453	1552163	+	-	-	-	-
orf_1487	CP034771.1	group_3537	1552194	1552754	+	COG1611	F;Nucleotide monophosphate nucleosidase PpnN/YdgH, Lonely Gu	-	Cytokinin riboside 5'-monophosphate phosphoribohydrolase OS=Pseudomonas aeruginosa (strain ATCC 15692 / DSM 22644 / CIP 104116 / JCM 14847 / LMG 12228 / 1C / PRS 101 / PAO1) OX=208964 GN=PA4923 PE=1 SV=2
orf_1497	CP034771.1	group_2053	1556347	1556553	-	COG3655	K;DNA-binding transcriptional regulator, XRE family;YozG;	-	Uncharacterized HTH-type transcriptional regulator YozG OS=Bacillus subtilis (strain 168) OX=224308 GN=yozG PE=4 SV=1
orf_1527	CP034771.1	group_1799	1591666	1591947	+	COG1254	C;Acylphosphatase;AcyP;	-	Acylphosphatase OS=Listeria monocytogenes serovar 1/2a (strain ATCC BAA-679 / EGD-e) OX=169963 GN=acyP PE=3 SV=1
orf_1648	CP034771.1	group_2049	1710848	1711783	-	COG2267	I;Lysophospholipase, alpha-beta hydrolase superfamily;PldB;	-	Monoacylglycerol lipase OS=Mycolicibacterium smegmatis (strain ATCC 700084 / mc(2)155) OX=246196 GN=MSMEG_0220 PE=1 SV=1
orf_1650	CP034771.1	group_2034	1712474	1712935	-	COG1051	F;ADP-ribose pyrophosphatase YjhB, NUDIX family;YjhB;	-	-
orf_1651	CP034771.1	group_335	1713051	1714535	+	COG1264	G;Phosphotransferase system IIB components;PtsG2;	-	PTS system trehalose-specific EIIBC component OS=Escherichia coli (strain K12) OX=83333 GN=treB PE=1 SV=4
orf_1653	CP034771.1	group_685	1716231	1716944	-	COG2188	K;DNA-binding transcriptional regulator, GntR family;MngR;	-	HTH-type transcriptional regulator TreR OS=Bacillus subtilis (strain 168) OX=224308 GN=treR PE=1 SV=2
orf_1655	CP034771.1	group_2643	1717256	1717363	+	-	-	-	-
orf_1658	CP034771.1	group_1696	1718711	1719370	-	COG0664	T;cAMP-binding domain of CRP or a regulatory subunit of cAMP-de	-	Lactate racemization regulatory protein OS=Lactiplantibacillus plantarum (strain ATCC BAA-793 / NCIMB 8826 / WCFS1) OX=220668 GN=larR PE=1 SV=1
orf_1661	CP034771.1	group_2219	1721299	1721715	-	COG0789	K;DNA-binding transcriptional regulator, MerR family;SoxR;	-	Uncharacterized HTH-type transcriptional regulator HI_0186 OS=Haemophilus influenzae (strain ATCC 51907 / DSM 11121 / KW20 / Rd) OX=71421 GN=HI_0186 PE=1 SV=1
orf_1665	CP034771.1	group_1647	1724363	1724626	-	-	-	-	-
orf_1668	CP034771.1	group_2028	1726493	1727104	-	COG0406	G;Broad specificity phosphatase PhoE;PhoE;	-	2,3-bisphosphoglycerate-dependent phosphoglycerate mutase OS=Methylorubrum populi (strain ATCC BAA-705 / NCIMB 13946 / BJ001) OX=441620 GN=gpmA PE=3 SV=1
orf_1669	CP034771.1	group_2086	1727269	1727676	+	COG2764	C;Zn-dependent glyoxalase, PhnB family;PhnB;	-	Protein YjdN OS=Escherichia coli (strain K12) OX=83333 GN=yjdN PE=4 SV=1
orf_1672	CP034771.1	group_2662	1728814	1729329	-	-	-	-	-
orf_1673	CP034771.1	group_3304	1730253	1730948	-	COG4886	K;Leucine-rich repeat (LRR) protein;LRR;	-	Internalin C OS=Listeria monocytogenes serotype 1/2a (strain EGD / Mackaness) OX=1334565 GN=inlC PE=1 SV=1
orf_1675	CP034771.1	group_3648	1732676	1733683	+	COG3568	R;Metal-dependent hydrolase, endonuclease/exonuclease/phosph	(smcL) sphingomyelinase-c [SMase (VF0061) - Exoenzyme (VFC0251)] [Listeria ivanovii str.	Sphingomyelinase C OS=Listeria ivanovii OX=1638 GN=smcL PE=1 SV=1
orf_1676	CP034771.1	group_3717	1734088	1735935	-	COG4886	K;Leucine-rich repeat (LRR) protein;LRR;	-	Internalin C OS=Listeria monocytogenes serotype 1/2a (strain EGD / Mackaness) OX=1334565 GN=inlC PE=1 SV=1
orf_1678	CP034771.1	group_3304	1737779	1738471	-	COG4886	K;Leucine-rich repeat (LRR) protein;LRR;	-	Internalin C OS=Listeria monocytogenes serotype 1/2a (strain EGD / Mackaness) OX=1334565 GN=inlC PE=1 SV=1
orf_1681	CP034771.1	group_3649	1744294	1745232	-	COG4886	K;Leucine-rich repeat (LRR) protein;LRR;	-	Internalin C OS=Listeria monocytogenes serotype 1/2a (strain EGD / Mackaness) OX=1334565 GN=inlC PE=1 SV=1
orf_1682	CP034771.1	group_3721	1746183	1746968	-	COG4886	K;Leucine-rich repeat (LRR) protein;LRR;	-	Internalin C OS=Listeria monocytogenes serotype 1/2a (strain EGD / Mackaness) OX=1334565 GN=inlC PE=1 SV=1
orf_1705	CP034771.1	group_3724	1777173	1777340	-	-	-	-	-
orf_1706	CP034771.1	group_3658	1777607	1778134	-	-	-	-	-
orf_1717	CP034771.1	group_2336	1787666	1788211	-	-	-	-	-
orf_1718	CP034771.1	group_2328	1788214	1788525	-	COG1695	K;DNA-binding transcriptional regulator, PadR family;PadR;	-	Uncharacterized 37.1 kDa protein in transposon TN4556 OS=Streptomyces fradiae OX=1906 PE=3 SV=1
orf_1789	CP034771.1	group_3360	1847190	1848098	-	COG1028	I;NAD(P)-dependent dehydrogenase, short-chain alcohol dehydrog	-	WW domain-containing oxidoreductase OS=Gallus gallus OX=9031 GN=WWOX PE=2 SV=2
orf_1790	CP034771.1	group_3488	1848238	1848843	+	-	-	-	Probable HTH-type transcriptional regulator YttP OS=Bacillus subtilis (strain 168) OX=224308 GN=yttP PE=1 SV=1
orf_1795	CP034771.1	group_3237	1851050	1851790	-	COG1472	G;Periplasmic beta-glucosidase and related glycosidases;BglX;	-	Probable beta-glucosidase C OS=Aspergillus flavus (strain ATCC 200026 / FGSC A1120 / IAM 13836 / NRRL 3357 / JCM 12722 / SRRC 167) OX=332952 GN=bglC PE=3 SV=1
orf_1796	CP034771.1	group_3237	1851783	1853270	-	COG1472	G;Periplasmic beta-glucosidase and related glycosidases;BglX;	-	Periplasmic beta-glucosidase/beta-xylosidase OS=Dickeya chrysanthemi OX=556 GN=bgxA PE=3 SV=1
orf_1797	CP034771.1	group_2505	1854065	1854337	-	COG0221	C;Inorganic pyrophosphatase;Ppa;	-	-
orf_1813	CP034771.1	group_2383	1869594	1870040	-	COG4815	S;Uncharacterized conserved protein;;	-	-
orf_1828	CP034771.1	group_2134	1880585	1880989	-	COG1440	G;Phosphotransferase system cellobiose-specific component IIB;Ce	-	-
orf_1834	CP034771.1	group_2607	1885426	1886580	-	COG1887	MI;CDP-glycerol glycerophosphotransferase, TagB/SpsB family;Tag	-	Teichoic acid poly(glycerol phosphate) polymerase OS=Bacillus subtilis (strain 168) OX=224308 GN=tagF PE=1 SV=1
orf_1835	CP034771.1	group_2621	1886602	1887975	-	COG0463	M;Glycosyltransferase involved in cell wall bisynthesis;WcaA;	-	Minor teichoic acid biosynthesis protein GgaA OS=Bacillus subtilis (strain 168) OX=224308 GN=ggaA PE=3 SV=1
orf_1840	CP034771.1	group_2696	1893400	1894347	-	COG0463	M;Glycosyltransferase involved in cell wall bisynthesis;WcaA;	-	Uncharacterized protein SF2094/S2216 OS=Shigella flexneri OX=623 GN=SF2094 PE=4 SV=1
orf_1841	CP034771.1	group_2693	1894358	1896211	-	-	-	-	-
orf_1842	CP034771.1	group_3480	1896336	1898624	-	-	-	-	Beta-N-acetylglucosaminidase OS=Bacillus subtilis (strain 168) OX=224308 GN=lytD PE=1 SV=1
orf_1857	CP034771.1	group_3573	1919162	1919668	-	-	-	-	-
orf_1858	CP034771.1	group_3536	1919712	1920515	-	-	-	-	-
orf_1859	CP034771.1	group_3544	1920527	1921804	-	-	-	-	-
orf_1883	CP034771.1	group_2399	1940814	1942079	-	-	-	-	-
orf_1886	CP034771.1	group_2392	1945414	1946370	-	COG3958	G;Transketolase, C-terminal subunit;TktA2	-	Apulose-4-phosphate transketolase subunit B OS=Phocaeicola vulgatus (strain ATCC 8482 / DSM 1447 / JCM 5826 / CCUG 4940 / NBRC 14291 / NCTC 11154) OX=435590 GN=aptB PE=1 SV=1
orf_1888	CP034771.1	group_2404	1947203	1948606	-	COG2407	G;L-fucose isomerase or related protein;FucI;	-	-
orf_1889	CP034771.1	group_2400	1948871	1949899	+	COG1609	K;DNA-binding transcriptional regulator, LacI/PurR family;PurR;	-	HTH-type transcriptional repressor PurR OS=Vibrio atlanticus (strain LGP32) OX=575788 GN=purR PE=3 SV=1
orf_1894	CP034771.1	group_1654	1954221	1955072	-	COG1289	S;Uncharacterized membrane protein YccC;YccC	-	-
orf_1895	CP034771.1	group_2142	1955069	1955989	-	-	-	-	-
orf_1896	CP034771.1	group_1823	1956145	1956804	-	COG0569	PT;Trk/Ktr K+ transport system regulatory component TrkA/KtrA/K	-	Ktr system potassium uptake protein C OS=Bacillus subtilis (strain 168) OX=224308 GN=ktrC PE=1 SV=1
orf_1898	CP034771.1	group_1716	1957457	1958515	-	COG4585	T;Signal transduction histidine kinase ComP;ComP;	-	Sensor histidine kinase LiaS OS=Bacillus subtilis (strain 168) OX=224308 GN=liaS PE=2 SV=1
orf_1902	CP034771.1	group_313	1961132	1961623	-	COG2190	G;Phosphotransferase system IIA component;NagE;	-	Putative phosphotransferase enzyme IIA component YpqE OS=Bacillus subtilis (strain 168) OX=224308 GN=ypqE PE=3 SV=1
orf_1905	CP034771.1	group_1703	1963508	1964701	-	COG4175	E;ABC-type proline/glycine betaine transport system, ATPase comp	-	Glycine betaine/carnitine transport ATP-binding protein GbuA OS=Listeria monocytogenes serotype 1/2a (strain 10403S) OX=393133 GN=gbuA PE=1 SV=1
orf_1907	CP034771.1	group_1770	1965964	1967082	-	COG1473	R;Metal-dependent amidase/aminoacylase/carboxypeptidase;AbgI	-	N-acetyldiaminopimelate deacetylase OS=Listeria ivanovii OX=1638 PE=3 SV=1
orf_1911	CP034771.1	group_1733	1969441	1969674	-	COG4703	S;Uncharacterized conserved protein YkuJ, DUF1797 family;YkuJ;	-	Uncharacterized protein YkuJ OS=Bacillus subtilis (strain 168) OX=224308 GN=ykuJ PE=1 SV=1
orf_1912	CP034771.1	group_1963	1969978	1970199	-	-	-	-	-
orf_1913	CP034771.1	group_1366	1970419	1971564	+	COG0436	E;Aspartate/methionine/tyrosine aminotransferase;AspB;Isoleucin	-	Probable N-acetyl-LL-diaminopimelate aminotransferase OS=Bacillus subtilis (strain 168) OX=224308 GN=dapX PE=1 SV=3
orf_1919	CP034771.1	group_1338	1976330	1977796	+	COG1233	Q;Phytoene dehydrogenase-related protein;;	-	4,4'-diapophytoene desaturase (4,4'-diapolycopene-forming) OS=Methylomonas sp. OX=418 GN=crtN PE=1 SV=1
orf_1920	CP034771.1	group_1690	1977800	1978591	-	-	-	-	-
orf_1921	CP034771.1	group_1377	1978737	1979348	+	COG1266	O;Membrane protease YdiL, CAAX protease family;YdiL	-	-
orf_1964	CP034771.1	group_1831	2018599	2020059	-	-	-	-	-
orf_1965	CP034771.1	group_3326	2020231	2020797	-	COG0431	C;NAD(P)H-dependent FMN reductase;SsuE;	-	NAD(P)H-dependent FAD/FMN reductase GTNG_3158 OS=Geobacillus thermodenitrificans (strain NG80-2) OX=420246 GN=GTNG_3158 PE=1 SV=1
orf_1966	CP034771.1	group_3334	2020813	2021868	-	COG2141	HR;Flavin-dependent oxidoreductase, luciferase family (includes all	-	Uncharacterized protein y4vJ OS=Sinorhizobium fredii (strain NBRC 101917 / NGR234) OX=394 GN=NGR_a01140 PE=3 SV=2
orf_1997	CP034771.1	group_3497	2048935	2049354	-	COG0346	Q;Catechol 2,3-dioxygenase or related enzyme, vicinal oxygen chel	-	Uncharacterized protein Sfri_3691 OS=Shewanella frigidimarina (strain NCIMB 400) OX=318167 GN=Sfri_3691 PE=4 SV=2
orf_2007	CP034771.1	group_2169	2060364	2060714	-	COG1447	G;Phosphotransferase system cellobiose-specific component IIA;Ce	-	PTS system lactose-specific EIIA component OS=Lactobacillus casei OX=1582 GN=lacF PE=1 SV=1
orf_2034	CP034771.1	group_1497	2084501	2085337	-	COG1366	T;Anti-anti-sigma regulatory factor (antagonist of anti-sigma factor	-	RsbT co-antagonist protein RsbRA OS=Bacillus subtilis (strain 168) OX=224308 GN=rsbRA PE=1 SV=1
orf_2042	CP034771.1	group_2130	2091498	2091974	+	-	-	-	-
orf_2048	CP034771.1	group_2011	2097470	2097805	-	COG1440	G;Phosphotransferase system cellobiose-specific component IIB;Ce	-	PTS system cellobiose-specific EIIB component OS=Geobacillus stearothermophilus OX=1422 GN=celA PE=1 SV=1
orf_2049	CP034771.1	group_2223	2097806	2098153	-	COG1447	G;Phosphotransferase system cellobiose-specific component IIA;Ce	-	PTS system lactose-specific EIIA component OS=Staphylococcus epidermidis (strain ATCC 12228 / FDA PCI 1200) OX=176280 GN=lacF PE=3 SV=1
orf_2053	CP034771.1	group_3698	2101233	2101733	-	-	-	-	-
orf_2056	CP034771.1	group_2385	2102964	2103275	-	COG4367	S;Uncharacterized conserved protein, DUF2316 domain	-	-
orf_2079	CP034771.1	group_3732	2129503	2130828	-	-	-	-	-
orf_2089	CP034771.1	group_3361	2143005	2143973	-	COG0697	GER;Permease of the drug/metabolite transporter (DMT) superfar	-	Uncharacterized transporter CKL_3017 OS=Clostridium kluyveri (strain ATCC 8527 / DSM 555 / NCIMB 10680) OX=431943 GN=CKL_3017 PE=3 SV=1
orf_2090	CP034771.1	group_3543	2144090	2144968	+	-	-	-	Uncharacterized HTH-type transcriptional regulator YybE OS=Bacillus subtilis (strain 168) OX=224308 GN=yybE PE=3 SV=2
orf_2093	CP034771.1	group_2276	2147008	2148381	-	COG2271	G;Sugar phosphate permease;UhpC;	(hpt) hexose phosphate transport protein [Hpt (VF0264) - Nutritional/Metabolic factor (VF	Hexose-6-phosphate:phosphate antiporter OS=Shigella flexneri OX=623 GN=uhpT PE=3 SV=1
orf_2114	CP034771.1	group_3533	2177181	2177879	-	-	-	-	-
orf_2123	CP034771.1	group_2525	2185068	2185793	-	-	-	-	-
orf_2125	CP034771.1	group_3656	2186979	2188265	-	-	-	-	Putative permease IIC component YwbA OS=Bacillus subtilis (strain 168) OX=224308 GN=ywbA PE=3 SV=1
orf_2181	CP034771.1	group_2409	2243387	2243998	-	-	-	-	Uncharacterized protein AN9274 OS=Emericella nidulans (strain FGSC A4 / ATCC 38163 / CBS 112.46 / NRRL 194 / M139) OX=227321 GN=AN9274 PE=3 SV=1
orf_2182	CP034771.1	group_2331	2244084	2244767	-	COG0664	T;cAMP-binding domain of CRP or a regulatory subunit of cAMP-de	-	Probable transcriptional regulator flp OS=Lactobacillus casei OX=1582 GN=flp PE=4 SV=1
orf_2183	CP034771.1	group_2408	2244886	2245725	-	COG0596	HR;2-succinyl-6-hydroxy-2,4-cyclohexadiene-1-carboxylate synthas	-	Uncharacterized hydrolase SAOUHSC_02900 OS=Staphylococcus aureus (strain NCTC 8325 / PS 47) OX=93061 GN=SAOUHSC_02900 PE=3 SV=1
orf_2193	CP034771.1	group_2525	2255719	2256651	-	-	-	-	-
orf_2196	CP034771.1	group_2530	2257981	2258982	+	COG1609	K;DNA-binding transcriptional regulator, LacI/PurR family;PurR;	-	Ribose operon repressor OS=Alkalihalobacillus halodurans (strain ATCC BAA-125 / DSM 18197 / FERM 7344 / JCM 9153 / C-125) OX=272558 GN=rbsR PE=3 SV=1
orf_2197	CP034771.1	group_1644	2259090	2259488	-	-	-	-	-
orf_2210	CP034771.1	group_3500	2271235	2273580	-	COG0474	P;Magnesium-transporting ATPase (P-type);MgtA;	-	Calcium-transporting ATPase CtpE OS=Mycolicibacterium smegmatis (strain ATCC 700084 / mc(2)155) OX=246196 GN=ctpE PE=1 SV=1
orf_2265	CP034771.1	group_2662	2320864	2321385	-	-	-	-	-
orf_2290	CP034771.1	group_2442	2345124	2346392	-	COG4573	G;Tagatose-1,6-bisphosphate aldolase non-catalytic subunit AgaZ/(	-	D-tagatose-1,6-bisphosphate aldolase subunit GatZ OS=Shigella flexneri OX=623 GN=gatZ PE=3 SV=1
orf_2294	CP034771.1	group_2445	2348240	2350288	-	COG3711	K;Transcriptional antiterminator;BglG;	-	Transcriptional regulator ManR OS=Bacillus subtilis (strain 168) OX=224308 GN=manR PE=1 SV=2
orf_2321	CP034771.1	group_2107	2384440	2384703	-	-	-	-	-
orf_2345	CP034771.1	group_2700	2403824	2405122	+	-	-	-	-
orf_2360	CP034771.1	group_1284	2423509	2425077	+	COG3103	R;Uncharacterized conserved protein YgiM, contains N-terminal SH	-	Probable endopeptidase p60 OS=Listeria ivanovii OX=1638 GN=iap PE=3 SV=1
orf_2366	CP034771.1	group_3278	2428707	2430203	-	COG1070	G;Sugar (pentulose or hexulose) kinase;XylB;Non-phosphorylated E	-	Xylulose kinase OS=Lactococcus lactis subsp. lactis (strain IL1403) OX=272623 GN=xylB PE=3 SV=1
orf_2367	CP034771.1	group_3409	2430222	2431535	-	COG2115	G;Xylose isomerase;XylA;	-	Xylose isomerase OS=Enterococcus faecalis (strain ATCC 700802 / V583) OX=226185 GN=xylA PE=3 SV=1
orf_2368	CP034771.1	group_3421	2431697	2432860	-	COG1940	GK;Sugar kinase of the NBD/HSP70 family, may contain an N-termi	-	Xylose repressor OS=Lactiplantibacillus pentosus OX=1589 GN=xylR PE=3 SV=2
orf_2390	CP034771.1	group_2662	2452342	2452902	-	-	-	-	-
orf_2395	CP034771.1	group_2069	2457808	2458650	-	-	-	-	-
orf_2410	CP034771.1	group_3276	2473303	2474493	-	COG0786	E;Na+/glutamate symporter;GltS	-	-
orf_2421	CP034771.1	group_1291	2487151	2487891	+	COG0789	K;DNA-binding transcriptional regulator, MerR family;SoxR;	-	HTH-type transcriptional activator mta OS=Bacillus subtilis (strain 168) OX=224308 GN=mta PE=1 SV=1
orf_2429	CP034771.1	group_2270	2495726	2497474	-	COG1482	G;Mannose-6-phosphate isomerase, class I;ManA;	-	Mannose-6-phosphate isomerase ManA OS=Bacillus subtilis (strain 168) OX=224308 GN=manA PE=2 SV=1
orf_2430	CP034771.1	group_3494	2497596	2498468	-	-	-	-	Uncharacterized HTH-type transcriptional regulator TTE0211 OS=Caldanaerobacter subterraneus subsp. tengcongensis (strain DSM 15242 / JCM 11007 / NBRC 100824 / MB4) OX=273068 GN=TTE0211 PE=4 SV=1
orf_2431	CP034771.1	group_3484	2498567	2499985	-	COG3538	S;Meiotically up-regulated gene 157 (Mug157) protein (function ur	-	Meiotically up-regulated gene 157 protein OS=Schizosaccharomyces pombe (strain 972 / ATCC 24843) OX=284812 GN=mug157 PE=1 SV=1
orf_2433	CP034771.1	group_3489	2500858	2503287	-	-	-	-	-
orf_2434	CP034771.1	group_3329	2503303	2504139	-	COG0395	G;ABC-type glycerol-3-phosphate transport system, permease com	-	Probable ABC transporter permease protein PH1216 OS=Pyrococcus horikoshii (strain ATCC 700860 / DSM 12428 / JCM 9974 / NBRC 100139 / OT-3) OX=70601 GN=PH1216 PE=3 SV=1
orf_2435	CP034771.1	group_3328	2504142	2505080	-	COG1175	G;ABC-type sugar transport system, permease component;UgpA;	-	Probable starch degradation products transport system permease protein AmyD OS=Thermoanaerobacterium thermosulfurigenes OX=33950 GN=amyD PE=3 SV=1
orf_2436	CP034771.1	group_3492	2505111	2506355	-	-	-	-	Uncharacterized ABC transporter extracellular-binding protein PH1039 OS=Pyrococcus horikoshii (strain ATCC 700860 / DSM 12428 / JCM 9974 / NBRC 100139 / OT-3) OX=70601 GN=PH1039 PE=3 SV=1
orf_2484	CP034771.1	group_2273	2548218	2548610	-	-	-	-	Uncharacterized protein YjqA OS=Bacillus subtilis (strain 168) OX=224308 GN=yjqA PE=4 SV=1
orf_2487	CP034771.1	group_2365	2551350	2552336	+	COG1078	R;HD superfamily phosphohydrolase;YdhJ;	-	Uncharacterized protein YdhJ OS=Bacillus subtilis (strain 168) OX=224308 GN=ydhJ PE=4 SV=1
orf_2489	CP034771.1	group_156	2553133	2554005	-	-	-	-	-
orf_2491	CP034771.1	group_175	2554550	2554918	-	-	-	-	-

orf_2492	CP034771.1	group_177	2554919	2555203	-	-	-	-	-
orf_2507	CP034771.1	group_2878	2574558	2578127	-	COG4886	K;Leucine-rich repeat (LRR) protein;LRR;	-	Internalin A OS=Listeria monocytogenes serovar 1/2a (strain ATCC BAA-679 / EGD-e) OX=169963 GN=inlA PE=1 SV=1
orf_2510	CP034771.1	group_3722	2584590	2586137	-	-	-	-	Internalin H OS=Listeria monocytogenes serovar 1/2a (strain ATCC BAA-679 / EGD-e) OX=169963 GN=inlH PE=1 SV=1
orf_2512	CP034771.1	group_3645	2587057	2587779	-	COG1028	I;NAD(P)-dependent dehydrogenase, short-chain alcohol dehydrog	-	Short chain dehydrogenase andC OS=Emericella variicolor OX=1549217 GN=andC PE=3 SV=1
orf_2528	CP034771.1	group_2557	2600643	2600786	-	-	-	-	-
orf_2534	CP034771.1	group_178	2605417	2605518	-	-	-	-	-
orf_2535	CP034771.1	group_178	2605557	2605658	-	-	-	-	-
orf_2539	CP034771.1	group_3397	2608282	2608794	+	-	-	-	-
orf_2540	CP034771.1	group_3417	2608791	2610098	+	COG1215	N;Glycosyltransferase, catalytic subunit of cellulose synthase and p	-	Poly-beta-1,6-N-acetyl-D-glucosamine synthase OS=Escherichia coli (strain K12) OX=83333 GN=pgaC PE=1 SV=1
orf_2542	CP034771.1	group_2499	2611062	2611565	+	COG1595	K;DNA-directed RNA polymerase specialized sigma subunit, sigma2	-	RNA polymerase sigma factor SigV OS=Bacillus subtilis (strain 168) OX=224308 GN=sigV PE=1 SV=1
orf_2543	CP034771.1	group_3650	2611555	2612406	+	-	-	-	Anti-sigma-V factor RsiV OS=Bacillus subtilis (strain 168) OX=224308 GN=rsiV PE=1 SV=1
orf_2544	CP034771.1	group_3568	2612420	2613262	+	-	-	-	Putative polysaccharide deacetylase YheN OS=Bacillus subtilis (strain 168) OX=224308 GN=yheN PE=3 SV=1
orf_2552	CP034771.1	group_3731	2618392	2619507	+	COG4292	S;Low temperature requirement protein LtrA (function unknown);L	-	Uncharacterized protein RA0937 OS=Rhizobium meliloti (strain 1021) OX=266834 GN=RA0937 PE=4 SV=2
orf_2557	CP034771.1	group_141	2625040	2625243	+	COG1476	K;DNA-binding transcriptional regulator, XRE-family HTH domain;XI	(cylR2) cytolysin regulator R2 [Cytolysin (VF0356) - Exotoxin (VFC0235)]	Uncharacterized HTH-type transcriptional regulator MJ0272 OS=Methanocaldococcus jannaschii (strain ATCC 43067 / DSM 2661 / JAL-1 / JCM 10045 / NBRC 100440) OX=243232 GN=MJ0272 PE=4 SV=2
orf_2579	CP034771.1	group_3668	2644996	2645181	-	-	-	-	-
orf_2593	CP034771.1	group_3604	2652326	2652451	-	-	-	-	-
orf_2632	CP034771.1	group_2468	2699165	2700607	-	COG0366	G;Glycosidase/amylase (phosphorylase);AmyA;	-	Sucrose phosphorylase OS=Streptococcus mutans serotype c (strain ATCC 700610 / UA159) OX=210007 GN=gtfA PE=1 SV=4
orf_2634	CP034771.1	group_2469	2702320	2703150	-	COG3833	G;ABC-type maltose transport system, permease component MalG	-	Galactooligosaccharides transport system permease protein GanQ OS=Bacillus subtilis (strain 168) OX=224308 GN=ganQ PE=1 SV=1
orf_2635	CP034771.1	group_2467	2703152	2704489	-	COG1175	G;ABC-type sugar transport system, permease component;UgpA;	-	Galactooligosaccharides transport system permease protein GanP OS=Bacillus subtilis (strain 168) OX=224308 GN=ganP PE=1 SV=1
orf_2636	CP034771.1	group_2462	2704507	2705733	-	COG2182	G;Maltose-binding periplasmic protein MalE;MalE;	-	Galactooligosaccharide-binding protein OS=Bacillus subtilis (strain 168) OX=224308 GN=ganS PE=1 SV=1
orf_2637	CP034771.1	group_2466	2705871	2706866	-	COG1609	K;DNA-binding transcriptional regulator, LacI/PurR family;PurR;	-	NTD biosynthesis operon regulator NtdR OS=Bacillus subtilis (strain 168) OX=224308 GN=ntdR PE=4 SV=1
orf_2689	CP034771.1	group_2042	2766242	2766916	+	-	-	-	Uncharacterized protein Lin0241 OS=Listeria innocua serovar 6a (strain ATCC BAA-680 / CLIP 11262) OX=272626 GN=lin0241 PE=4 SV=1
orf_2691	CP034771.1	group_3438	2767360	2768154	-	-	-	-	Uncharacterized protein MJ0912 OS=Methanocaldococcus jannaschii (strain ATCC 43067 / DSM 2661 / JAL-1 / JCM 10045 / NBRC 100440) OX=243232 GN=MJ0912 PE=4 SV=1
orf_2694	CP034771.1	group_3603	2769642	2772566	-	-	-	-	-
orf_2705	CP034771.1	group_1679	2784659	2785336	-	COG0845	MV;Multidrug efflux pump subunit AcrA (membrane-fusion protein	-	-
orf_2706	CP034771.1	group_1554	2785495	2786313	-	COG0503	F;Adenine/guanine phosphoribosyltransferase or related PRPP-binc	-	Pur operon repressor OS=Bacillus subtilis (strain 168) OX=224308 GN=purR PE=1 SV=1
orf_2707	CP034771.1	group_846	2786481	2787218	-	COG3394	G;Chitooligosaccharide deacetylase ChbG, YdjC/CelG family;ChbG;	-	Carbohydrate deacetylase OS=Listeria welshimeri serovar 6b (strain ATCC 35897 / DSM 20650 / CIP 8149 / NCTC 11857 / SLCC 5334 / V8) OX=386043 GN=lwe0160 PE=3 SV=1
orf_2715	CP034771.1	group_284	2794229	2796520	-	COG1501	G;Alpha-glucosidase/xylosidase, GH31 family;YicI;	-	Alpha-glucosidase 2 OS=Bacillus thermoamyloliquefaciens OX=1425 PE=3 SV=1
orf_2716	CP034771.1	group_62	2796523	2799825	-	COG1501	G;Alpha-glucosidase/xylosidase, GH31 family;YicI;	-	1,3-alpha-isomaltosidase OS=Kribbella flavida (strain DSM 17836 / JCM 10339 / NBRC 14399) OX=479435 GN=Kfla_1895 PE=1 SV=1
orf_2717	CP034771.1	group_1980	2799920	2801176	-	COG1653	G;ABC-type glycerol-3-phosphate transport system, periplasmic co	-	Putative binding protein BRA0748/BS1330_II0741 OS=Brucella suis biovar 1 (strain 1330) OX=204722 GN=BRA0748 PE=3 SV=1
orf_2719	CP034771.1	group_147	2802052	2802930	-	COG1175	G;ABC-type sugar transport system, permease component;UgpA;	-	Probable ABC transporter permease protein YesP OS=Bacillus subtilis (strain 168) OX=224308 GN=yesP PE=3 SV=1
orf_2720	CP034771.1	group_227	2802967	2804181	-	COG1940	GK;Sugar kinase of the NBD/HSP70 family, may contain an N-termi	-	Xylose repressor OS=Caldicellulosiruptor bescii (strain ATCC BAA-1888 / DSM 6725 / Z-1320) OX=521460 GN=xylR PE=3 SV=1
orf_2736	CP034771.1	group_3118	2818885	2819703	-	COG2362	E;D-aminopeptidase;DppA;	-	D-aminopeptidase OS=Bacillus subtilis (strain 168) OX=224308 GN=dppA PE=1 SV=3
orf_2738	CP034771.1	group_3105	2821381	2822160	-	COG2367	V;Beta-lactamase class A;PenP;	-	-
orf_2748	CP034771.1	group_156	2833038	2833637	-	-	-	-	-
orf_2773	CP034771.1	group_2575	2858082	2858369	+	COG0640	K;DNA-binding transcriptional regulator, ArsR family;ArsR;	-	Uncharacterized HTH-type transcriptional regulator YczG OS=Bacillus subtilis (strain 168) OX=224308 GN=yczG PE=4 SV=1
orf_2775	CP034771.1	group_3712	2859861	2861261	-	COG2190	G;Phosphotransferase system IIA component;NagE;	-	PTS system beta-glucoside-specific EIIBCA component OS=Bacillus subtilis (strain 168) OX=224308 GN=bglP PE=3 SV=2
orf_2776	CP034771.1	group_2329	2861401	2862393	+	-	-	-	Regulatory protein PocR OS=Salmonella typhimurium (strain LT2 / SGSC1412 / ATCC 700720) OX=99287 GN=pocR PE=1 SV=2
orf_2788	CP034771.1	group_3566	2872695	2873387	-	COG0235	EG;5-methylthioribulose/5-deoxyribulose/Fuculose 1-phosphate al	-	L-fuculose phosphate aldolase OS=Methanococcus maripaludis (strain C7 / ATCC BAA-1331) OX=426368 GN=fucA PE=3 SV=1
orf_2796	CP034771.1	group_2370	2882991	2883893	-	COG4886	K;Leucine-rich repeat (LRR) protein;LRR;	-	Internalin C OS=Listeria monocytogenes serotype 1/2a (strain EGD / Mackaness) OX=1334565 GN=inlC PE=1 SV=1
orf_2799	CP034771.1	group_3280	2886378	2886899	-	-	-	-	-
orf_2803	CP034771.1	group_2179	2890010	2890783	-	COG2513	G;2-Methylisocitrate lyase and related enzymes, PEP mutase family	-	Uncharacterized protein Rv1998c OS=Mycobacterium tuberculosis (strain ATCC 25618 / H37Rv) OX=83332 GN=Rv1998c PE=1 SV=1
