# Supplementary material for: Comparative Genomics Reveal the Utilization Ability of Variable Carbohydrates as Key Genetic Features of Listeria Pathogens in Their Pathogenic Lifestyles
Source: Pathogens. 2022 Nov 28;11(12):1430. doi: 10.3390/pathogens11121430 (PMC9784484; doi:10.3390/pathogens11121430)
Supplement: Supplementary file 1 [file pathogens-11-01430-s001.zip › pathogens-1990707-supplementary/Supplymentary Tables S5.pdf]

Table S5. Unique genes of Listeria pathogens (UGLP) referenced to L.ivanovii 1638

ORF_id	Sequence_id	Group_id	start	end	strand	COG_id	COG_annotation	VFDB_annotation	Uniprot_annotation
orf_5	CP034771.1	group_223	2999	7495	-	COG1674	D;DNA segregation ATPase FtsK/SpoIIIE or related protein;FtsK;	-	Type VII secretion system protein EssC OS=Staphylococcus aureus (strain N315) OX=158879 GN=essC PE=3 SV=1
orf_6	CP034771.1	group_264	7509	7937	-	COG4499	U;ESX protein secretion system component YukC;YukC;	-	Uncharacterized protein CA_C3710 OS=Clostridium acetobutylicum (strain ATCC 824 / DSM 792 / JCM 1419 / LMG 5710 / VKM B-1787) OX=272562 GN=CA_C3710 PE=4 SV=1
orf_7	CP034771.1	group_264	8045	8587	-	COG4499	U;ESX protein secretion system component YukC;YukC;	-	Type VII secretion system protein EssB OS=Staphylococcus aureus (strain MRSA252) OX=282458 GN=essB PE=3 SV=1
orf_31	CP034771.1	group_2236	33169	34254	-	COG0449	M;Glucosamine 6-phosphate synthetase, contains amidotransferase and phos	-	Glutamine--fructose-6-phosphate aminotransferase [isomerizing] OS=Pyrococcus furiosus (strain ATCC 43587 / DSM 3638 / JCM 8422 / Vc1) OX=186497 GN=glmS PE=3 SV=3
orf_33	CP034771.1	group_2212	35627	38029	-	-	-	-	-
orf_34	CP034771.1	group_2260	38026	38991	-	COG1940	GK;Sugar kinase of the NBD/HSP70 family, may contain an N-terminal HTH do	-	Glucokinase OS=Alkalihalobacillus halodurans (strain ATCC BAA-125 / DSM 18197 / FERM 7344 / JCM 9153 / C-125) OX=272558 GN=glcK PE=3 SV=1
orf_35	CP034771.1	group_2241	39194	40252	+	COG1609	K;DNA-binding transcriptional regulator, LacI/PurR family;PurR;	-	HTH-type transcriptional repressor PurR OS=Haemophilus influenzae (strain PittEE) OX=374930 GN=purR PE=3 SV=1
orf_45	CP034771.1	group_9	46706	48613	-	COG2190	G;Phosphotransferase system IIA component;NagE;	-	PTS system beta-glucoside-specific EIIBCA component OS=Alkalihalobacillus halodurans (strain ATCC BAA-125 / DSM 18197 / FERM 7344 / JCM 9153 / C-125) OX=272558 GN=bglP PE=3 SV=1
orf_48	CP034771.1	group_1520	51509	51841	-	COG3125	C;Heme/copper-type cytochrome/quinol oxidase, subunit 4;CyoD	-	Quinol oxidase subunit 4 OS=Bacillus spizizenii (strain ATCC 23059 / NRRL B-14472 / W23) OX=655816 GN=qoxD PE=1 SV=1
orf_49	CP034771.1	group_1819	51843	52454	-	COG1845	C;Heme/copper-type cytochrome/quinol oxidase, subunit 3;CyoC;	-	Quinol oxidase subunit 3 OS=Bacillus subtilis (strain 168) OX=224308 GN=qoxC PE=1 SV=1
orf_51	CP034771.1	group_537	54439	55545	-	COG1622	C;Heme/copper-type cytochrome/quinol oxidase, subunit 2;CyoA;	-	Probable quinol oxidase subunit 2 OS=Staphylococcus epidermidis (strain ATCC 12228 / FDA PCI 1200) OX=176280 GN=qoxA PE=3 SV=1
orf_53	CP034771.1	group_1364	56949	57920	-	COG3407	I;Mevalonate pyrophosphate decarboxylase;MVD1;Isoprenoid biosynthesis	-	Diphosphomevalonate decarboxylase OS=Dictyostelium discoideum OX=44689 GN=mvd PE=3 SV=1
orf_55	CP034771.1	group_1722	58989	59507	-	COG1670	JO;Protein N-acetyltransferase, RimJ/RimL family;RimL;	-	Spermidine N(1)-acetyltransferase OS=Wigglesworthia glossinidia brevipalpis OX=36870 GN=speG PE=3 SV=1
orf_56	CP034771.1	group_331	59523	61037	-	COG1502	I;Phosphatidylserine/phosphatidylglycerophosphate/cardiolipin synthase;Cls;F	-	Cardiolipin synthase 1 OS=Bacillus anthracis OX=1392 GN=cls1 PE=3 SV=1
orf_59	CP034771.1	group_1688	65780	66892	-	COG1195	L;Recombinational DNA repair ATPase RecF;RecF;	-	DNA replication and repair protein RecF OS=Listeria innocua serovar 6a (strain ATCC BAA-680 / CLIP 11262) OX=272626 GN=recF PE=3 SV=1
orf_64	CP034771.1	group_1960	71944	72414	+	-	-	-	-
orf_65	CP034771.1	group_2024	72582	72716	+	COG0230	J;Ribosomal protein L34;RpmH;Ribosome 50S subunit	-	50S ribosomal protein L34 OS=Listeria welshimeri serovar 6b (strain ATCC 35897 / DSM 20650 / CIP 8149 / NCTC 11857 / SLCC 5334 / V8) OX=386043 GN=rpmH PE=3 SV=1
orf_69	CP034771.1	group_1319	74741	75202	+	COG4405	R;Predicted RNA-binding protein YhfF, contains PUA-like ASCH domain;YhfF	-	-
orf_71	CP034771.1	group_285	81795	83789	+	COG1305	O;Transglutaminase-like enzyme, putative cysteine protease;YebA;	-	-
orf_72	CP034771.1	group_2160	83938	84963	-	COG1609	K;DNA-binding transcriptional regulator, LacI/PurR family;PurR;	-	Uncharacterized HTH-type transcriptional regulator YcjW OS=Escherichia coli (strain K12) OX=83333 GN=ycjW PE=4 SV=1
orf_73	CP034771.1	group_136	85189	86889	+	COG0366	G;Glycosidase/amylase (phosphorylase);AmyA;	-	Glucosylglycerate phosphorylase OS=Escherichia coli (strain K12) OX=83333 GN=ycjM PE=1 SV=2
orf_74	CP034771.1	group_2202	86886	88184	+	COG1653	G;ABC-type glycerol-3-phosphate transport system, periplasmic component;U	-	Putative ABC transporter periplasmic-binding protein YcjN OS=Escherichia coli (strain K12) OX=83333 GN=ycjN PE=3 SV=1
orf_75	CP034771.1	group_2204	88259	89140	+	COG1175	G;ABC-type sugar transport system, permease component;UgpA;	-	Inner membrane ABC transporter permease protein YcjO OS=Shigella flexneri OX=623 GN=ycjO PE=3 SV=1
orf_76	CP034771.1	group_2177	89127	89978	+	COG0395	G;ABC-type glycerol-3-phosphate transport system, permease component;Ug	-	Inner membrane ABC transporter permease protein YcjP OS=Escherichia coli (strain K12) OX=83333 GN=ycjP PE=1 SV=1
orf_77	CP034771.1	group_1769	89996	91048	+	COG1063	ER;Threonine dehydrogenase or related Zn-dependent dehydrogenase;Tdh;Nc	-	D-guloside 3-dehydrogenase OS=Escherichia coli (strain K12) OX=83333 GN=ycjQ PE=1 SV=1
orf_78	CP034771.1	group_2216	91058	91855	+	COG1082	G;Sugar phosphate isomerase/epimerase;YcjR;	-	3-dehydro-D-guloside 4-epimerase OS=Escherichia coli (strain K12) OX=83333 GN=ycjR PE=1 SV=2
orf_79	CP034771.1	group_1874	91912	92943	+	COG0673	R;Predicted dehydrogenase;MviM;	-	D-glucoside 3-dehydrogenase OS=Escherichia coli (strain K12) OX=83333 GN=ycjS PE=1 SV=1
orf_83	CP034771.1	group_1451	97021	97341	+	COG3118	O;Chaperedoxin CnoX, contains thioredoxin-like and TPR-like domains, YbbN/	-	Thioredoxin 1 OS=Chlorobaculum tepidum (strain ATCC 49652 / DSM 12025 / NBRC 103806 / TLS) OX=194439 GN=trx1 PE=3 SV=1
orf_84	CP034771.1	group_1689	97400	97999	-	COG3560	R;Fatty acid repression mutant protein (predicted oxidoreductase);FMR2;	-	Putative nitroreductase HBN1 OS=Saccharomyces cerevisiae (strain ATCC 204508 / S288c) OX=559292 GN=HBN1 PE=1 SV=1
orf_95	CP034771.1	group_538	111588	113477	+	COG0445	J;tRNA U34 5-carboxymethylaminomethyl modifying enzyme MnmG/GidA;Mn	-	tRNA uridine 5-carboxymethylaminomethyl modification enzyme MnmG OS=Listeria monocytogenes serovar 1/2a (strain ATCC BAA-679 / EGD-e) OX=169963 GN=mnmG PE=3 SV=1
orf_103	CP034771.1	group_1625	118543	118842	+	-	-	-	-
orf_113	CP034771.1	group_1374	126840	127598	+	-	-	-	-
orf_119	CP034771.1	group_1287	132673	133170	+	-	-	-	-
orf_120	CP034771.1	group_1335	133220	135550	-	COG2918	H;Gamma-glutamylcysteine synthetase;GshA;	-	Glutathione biosynthesis bifunctional protein GshAB OS=Listeria welshimeri serovar 6b (strain ATCC 35897 / DSM 20650 / CIP 8149 / NCTC 11857 / SLCC 5334 / V8) OX=386043 GN=gshAB PE=3 SV=1
orf_123	CP034771.1	group_1780	137826	138500	+	COG4858	S;Uncharacterized membrane-anchored protein	-	-
orf_136	CP034771.1	group_1841	148651	149181	+	COG2110	J;O-acetyl-ADP-ribose deacetylase (regulator of RNase III), contains Macro dor	-	Macro domain-containing protein lmo2759 OS=Listeria monocytogenes serovar 1/2a (strain ATCC BAA-679 / EGD-e) OX=169963 GN=lmo2759 PE=4 SV=1
orf_137	CP034771.1	group_550	149464	150930	+	COG0517	T;CBS domain;CBS;	-	Inosine-5'-monophosphate dehydrogenase OS=Bacillus subtilis (strain 168) OX=224308 GN=guaB PE=1 SV=2
orf_140	CP034771.1	group_1741	155337	157004	+	COG2936	R;Predicted acyl esterase;;	-	Cocaine esterase OS=Rhodococcus sp. (strain MB1 Bresler) OX=104109 GN=cocE PE=1 SV=1
orf_143	CP034771.1	group_87	160441	162162	-	COG1132	V;ABC-type multidrug transport system, ATPase and permease component;Mc	-	Uncharacterized ABC transporter ATP-binding protein YfiB OS=Bacillus subtilis (strain 168) OX=224308 GN=yfiB PE=3 SV=1
orf_146	CP034771.1	group_2159	164728	165147	+	COG3871	S;General stress protein 26 (function unknown);YzzA;	-	General stress protein 26 OS=Bacillus subtilis (strain 168) OX=224308 GN=ydaG PE=1 SV=2
orf_147	CP034771.1	group_551	165483	166769	+	COG0172	J;Seryl-tRNA synthetase;SerS;Aminoacyl-tRNA synthetases	-	Serine--tRNA ligase OS=Listeria monocytogenes serotype 4a (strain HCC23) OX=552536 GN=serS PE=3 SV=1
orf_150	CP034771.1	group_1979	169148	169837	+	COG0664	T;cAMP-binding domain of CRP or a regulatory subunit of cAMP-dependent pr	-	-
orf_152	CP034771.1	group_1629	170642	170989	+	-	-	-	-
orf_154	CP034771.1	group_1829	172303	172584	+	-	-	-	Mitomycin resistance protein McrB OS=Streptomyces lavendulae OX=1914 GN=mcrB PE=4 SV=1
orf_155	CP034771.1	group_1600	172587	173276	+	COG0846	O;NAD-dependent protein deacetylase, SIR2 family;SIR2;	-	NAD-dependent protein deacetylase OS=Listeria monocytogenes serotype 4b (strain F2365) OX=265669 GN=cobB PE=3 SV=1
orf_159	CP034771.1	group_1474	176221	176832	+	COG0546	C;Phosphoglycolate phosphatase, HAD superfamily;Gph;	-	Putative pyrophosphatase PpaX OS=Caldanaerobacter subterraneus subsp. tengcongensis (strain DSM 15242 / JCM 11007 / NBRC 100824 / MB4) OX=273068 GN=ppaX PE=3 SV=2
orf_162	CP034771.1	group_2157	178429	178878	+	COG1846	K;DNA-binding transcriptional regulator, MarR family;MarR;	-	Uncharacterized HTH-type transcriptional regulator YybA OS=Bacillus subtilis (strain 168) OX=224308 GN=yybA PE=4 SV=1
orf_163	CP034771.1	group_1990	178875	180227	+	COG0534	V;Na+-driven multidrug efflux pump, DinF/NorM/MATE family;NorM;	-	Multidrug export protein MepA OS=Staphylococcus haemolyticus (strain JCSC1435) OX=279808 GN=mepA PE=3 SV=2
orf_164	CP034771.1	group_2138	180290	180733	+	COG2764	C;Zn-dependent glyoxalase, PhnB family;PhnB;	-	Protein YjdN OS=Escherichia coli (strain K12) OX=83333 GN=yjdN PE=4 SV=1
orf_171	CP034771.1	group_1867	183096	183542	-	COG2153	R;Predicted N-acyltransferase, GNAT family;ElaA;	-	Uncharacterized N-acetyltransferase YitI OS=Bacillus subtilis (strain 168) OX=224308 GN=yitI PE=3 SV=3
orf_172	CP034771.1	group_2120	183595	184329	-	COG0789	K;DNA-binding transcriptional regulator, MerR family;SoxR;	-	Multidrug-efflux transporter 1 regulator OS=Bacillus subtilis (strain 168) OX=224308 GN=bmrR PE=1 SV=3
orf_173	CP034771.1	group_384	184410	185129	+	COG0363	G;6-phosphogluconolactonase/Glucosamine-6-phosphate isomerase/deamina	-	Uncharacterized protein YieK OS=Escherichia coli (strain K12) OX=83333 GN=yieK PE=3 SV=3
orf_175	CP034771.1	group_701	186941	187411	+	COG0590	J;tRNA(Arg) A34 adenosine deaminase TadA;TadA;Pyrimidine salvagetRNA mo	-	tRNA-specific adenosine deaminase OS=Bacillus subtilis (strain 168) OX=224308 GN=tadA PE=1 SV=1
orf_176	CP034771.1	group_97	187789	189195	+	COG1271	C;Cytochrome bd-type quinol oxidase, subunit 1;AppC;	-	Cytochrome bd ubiquinol oxidase subunit 1 OS=Bacillus subtilis (strain 168) OX=224308 GN=cydA PE=3 SV=1
orf_177	CP034771.1	group_97	189182	190195	+	COG1294	C;Cytochrome bd-type quinol oxidase, subunit 2;AppB;	-	Cytochrome bd ubiquinol oxidase subunit 2 OS=Bacillus subtilis (strain 168) OX=224308 GN=cydB PE=3 SV=1
orf_178	CP034771.1	group_1310	190195	191919	+	COG4988	CO;ABC-type transport system involved in cytochrome bd biosynthesis, ATPas	-	ATP-binding/permease protein CydC OS=Bacillus subtilis (strain 168) OX=224308 GN=cydC PE=3 SV=1
orf_180	CP034771.1	group_1630	193798	194736	+	-	-	-	Cell wall protein Lmo2714 OS=Listeria monocytogenes serovar 1/2a (strain ATCC BAA-679 / EGD-e) OX=169963 GN=lmo2714 PE=1 SV=1
orf_186	CP034771.1	group_1496	199965	201329	+	COG1455	G;Phosphotransferase system cellobiose-specific component IIC;CelB;	-	PTS system cellobiose-specific EIIC component OS=Lactococcus lactis subsp. lactis (strain IL1403) OX=272623 GN=celB PE=2 SV=1
orf_187	CP034771.1	group_1521	201463	201684	+	-	-	-	-
orf_189	CP034771.1	group_1475	201887	202351	+	-	-	-	-
orf_191	CP034771.1	group_1715	204418	204735	+	COG0718	K;DNA-binding nucleoid-associated protein YbaB/EfbC;YbaB;	-	Nucleoid-associated protein lwe2654 OS=Listeria welshimeri serovar 6b (strain ATCC 35897 / DSM 20650 / CIP 8149 / NCTC 11857 / SLCC 5334 / V8) OX=386043 GN=lwe2654 PE=3 SV=1
orf_192	CP034771.1	group_1721	204844	205440	+	COG0353	L;Recombinational DNA repair protein RecR;RecR;	-	Recombination protein RecR OS=Listeria monocytogenes serovar 1/2a (strain ATCC BAA-679 / EGD-e) OX=169963 GN=recR PE=3 SV=1
orf_193	CP034771.1	group_1476	205455	205697	+	-	-	-	Uncharacterized protein YaaL OS=Bacillus subtilis (strain 168) OX=224308 GN=yaaL PE=4 SV=1
orf_221	CP034771.1	group_2032	236078	236917	-	COG0561	HR;Hydroxymethylpyrimidine pyrophosphatase and other HAD family phosph	-	Sugar phosphatase YidA OS=Shigella flexneri OX=623 GN=yidA PE=3 SV=1
orf_227	CP034771.1	group_1646	240756	241130	-	COG3412	T;Phosphotransferase subunit DhaM of the dihydroxyacetone kinase DhaKLM	-	PEP-dependent dihydroxyacetone kinase 1, phosphoryl donor subunit DhaM OS=Listeria innocua serovar 6a (strain ATCC BAA-680 / CLIP 11262) OX=272626 GN=dhaM-1 PE=3 SV=1
orf_238	CP034771.1	group_1670	250790	251356	+	COG1309	K;DNA-binding protein, AcrR family, includes nucleoid occlusion protein SlmA;	-	Uncharacterized HTH-type transcriptional regulator YvkB OS=Bacillus subtilis (strain 168) OX=224308 GN=yvkB PE=4 SV=1
orf_239	CP034771.1	group_463	251908	254478	+	COG0474	P;Magnesium-transporting ATPase (P-type);MgtA;	(mgtB) Mg2+ transport protein [MgtBC (VF0106)	Magnesium-transporting ATPase, P-type 1 OS=Salmonella typhimurium (strain LT2 / SGSC1412 / ATCC 700720) OX=99287 GN=mgtB PE=1 SV=3
orf_242	CP034771.1	group_35	256811	257113	-	COG1447	G;Phosphotransferase system cellobiose-specific component IIA;CelC;	-	Lichenan-specific phosphotransferase enzyme IIA component OS=Bacillus subtilis (strain 168) OX=224308 GN=licA PE=2 SV=1
orf_247	CP034771.1	group_1442	260937	262982	+	COG2216	P;K+ transport ATPase, ATPase subunit KdpB;KdpB;	-	Potassium-transporting ATPase ATP-binding subunit OS=Listeria monocytogenes serovar 1/2a (strain ATCC BAA-679 / EGD-e) OX=169963 GN=kdpB PE=3 SV=1
orf_248	CP034771.1	group_1290	262997	263554	+	COG2156	P;K+-transporting ATPase, KdpC subunit;KdpC;	-	Potassium-transporting ATPase KdpC subunit OS=Listeria welshimeri serovar 6b (strain ATCC 35897 / DSM 20650 / CIP 8149 / NCTC 11857 / SLCC 5334 / V8) OX=386043 GN=kdpC PE=3 SV=1
orf_249	CP034771.1	group_769	263574	266270	+	COG2205	T;K+-sensing histidine kinase KdpD;KdpD;	-	Sensor protein KdpD OS=Clostridium acetobutylicum (strain ATCC 824 / DSM 792 / JCM 1419 / LMG 5710 / VKM B-1787) OX=272562 GN=kdpD PE=3 SV=2
orf_250	CP034771.1	group_2100	266260	266955	+	COG0745	TK;DNA-binding response regulator, OmpR family, contains REC and winged-h	-	Transcriptional regulatory protein KdpE OS=Mycobacterium tuberculosis (strain ATCC 25618 / H37Rv) OX=83332 GN=kdpE PE=1 SV=1
orf_254	CP034771.1	group_7	269612	270067	-	COG0698	G;Ribose 5-phosphate isomerase RpiB;RpiB;Pentose phosphate pathway	-	Putative sugar phosphate isomerase YwlF OS=Bacillus subtilis (strain 168) OX=224308 GN=ywlF PE=2 SV=1
orf_256	CP034771.1	group_2132	270701	271513	+	COG4977	K;Transcriptional regulator GlxA, contains an amidase domain and an AraC-typ	-	-
orf_258	CP034771.1	group_1900	271918	272286	+	COG2315	K;Predicted DNA-binding protein with double-wing structural motif, MmcQ/YjI	-	Uncharacterized protein YyaQ OS=Bacillus subtilis (strain 168) OX=224308 GN=yyaQ PE=4 SV=1
orf_259	CP034771.1	group_2076	272363	273175	+	COG4905	S;Uncharacterized membrane protein;;	-	-
orf_265	CP034771.1	group_324	278874	279905	+	COG1063	ER;Threonine dehydrogenase or related Zn-dependent dehydrogenase;Tdh;Nc	-	D-arabitol-phosphate dehydrogenase OS=Enterococcus avium OX=33945 PE=1 SV=1
orf_266	CP034771.1	group_7	280100	280540	+	COG0698	G;Ribose 5-phosphate isomerase RpiB;RpiB;Pentose phosphate pathway	-	Ribose-5-phosphate isomerase B OS=Escherichia coli (strain K12) OX=83333 GN=rpiB PE=1 SV=2
orf_267	CP034771.1	group_132	280549	281223	+	COG0036	G;Pentose-5-phosphate-3-epimerase;Rpe;Pentose phosphate pathway	-	Ribulose-phosphate 3-epimerase OS=Bacillus subtilis (strain 168) OX=224308 GN=rpe PE=3 SV=1
orf_275	CP034771.1	group_1581	291211	291624	+	COG0048	J;Ribosomal protein S12;RpsL;Ribosome 30S subunit	-	30S ribosomal protein S12 OS=Listeria welshimeri serovar 6b (strain ATCC 35897 / DSM 20650 / CIP 8149 / NCTC 11857 / SLCC 5334 / V8) OX=386043 GN=rpsL PE=3 SV=1
orf_277	CP034771.1	group_258	292191	294278	+	COG0480	J;Translation elongation factor EF-G, a GTPase;FusA;Translation factors	-	Elongation factor G OS=Listeria monocytogenes serovar 1/2a (strain ATCC BAA-679 / EGD-e) OX=169963 GN=fusA PE=3 SV=1
orf_278	CP034771.1	group_1149	294388	295575	+	COG0050	J;Translation elongation factor EF-Tu, a GTPase;TufA;Translation factors	(tufA) elongation factor Tu [EF-Tu (VF0460) - Adherence (VFC0001)	Elongation factor Tu OS=Listeria monocytogenes serotype 4a (strain HCC23) OX=552536 GN=tuf PE=3 SV=1
orf_289	CP034771.1	group_165	304116	305108	+	COG1735	R;Predicted metal-dependent hydrolase, phosphotriesterase family;Php;	-	Phospho-furanose lactonase OS=Mycoplasmopsis synoviae (strain 53) OX=262723 GN=MS53_0025 PE=1 SV=1
orf_290	CP034771.1	group_162	305121	305870	+	COG1402	H;Creatinine amidohydrolase/Fe(II)-dependent FAPy formamide hydrolase (rib	-	Mycofactocin precursor peptide peptidase OS=Mycobacterium tuberculosis (strain ATCC 25618 / H37Rv) OX=83332 GN=mftE PE=1 SV=1
orf_292	CP034771.1	group_1926	306550	307293	+	COG4821	R;Uncharacterized conserved protein, contains SIS (Sugar ISomerase) phospho	-	UPF0309 protein lin2794 OS=Listeria innocua serovar 6a (strain ATCC BAA-680 / CLIP 11262) OX=272626 GN=lin2794 PE=3 SV=1
orf_295	CP034771.1	group_1925	309257	310594	-	COG1409	T;3',5'-cyclic AMP phosphodiesterase CpdA;CpdA;	-	Probable cyclic nucleotide phosphodiesterase Psyc_2036 OS=Psychrobacter arcticus (strain DSM 17307 / VKM B-2377 / 273-4) OX=259536 GN=Psyc_2036 PE=3 SV=1
orf_297	CP034771.1	group_1073	311676	312221	-	COG4769	S;Uncharacterized membrane protein	-	-
orf_298	CP034771.1	group_1946	312254	312688	-	COG5341	S;Uncharacterized conserved protein;;	-	-
orf_306	CP034771.1	group_1725	320564	321187	+	COG0088	J;Ribosomal protein L4;RplD;Ribosome 50S subunit	-	50S ribosomal protein L4 OS=Listeria monocytogenes serovar 1/2a (strain ATCC BAA-679 / EGD-e) OX=169963 GN=rplD PE=1 SV=1
orf_307	CP034771.1	group_1573	321187	321471	+	COG0089	J;Ribosomal protein L23;RplW;Ribosome 50S subunit	-	50S ribosomal protein L23 OS=Listeria welshimeri serovar 6b (strain ATCC 35897 / DSM 20650 / CIP 8149 / NCTC 11857 / SLCC 5334 / V8) OX=386043 GN=rplW PE=3 SV=1
orf_308	CP034771.1	group_1820	321512	322345	+	COG0090	J;Ribosomal protein L2;RplB;Ribosome 50S subunit	-	50S ribosomal protein L2 OS=Listeria welshimeri serovar 6b (strain ATCC 35897 / DSM 20650 / CIP 8149 / NCTC 11857 / SLCC 5334 / V8) OX=386043 GN=rplB PE=3 SV=1
orf_309	CP034771.1	group_1546	322391	322711	+	COG0185	J;Ribosomal protein S19;RpsS;Ribosome 30S subunit	-	30S ribosomal protein S19 OS=Listeria welshimeri serovar 6b (strain ATCC 35897 / DSM 20650 / CIP 8149 / NCTC 11857 / SLCC 5334 / V8) OX=386043 GN=rpsS PE=3 SV=1
orf_311	CP034771.1	group_1562	323092	323748	+	COG0092	J;Ribosomal protein S3;RpsC;Ribosome 30S subunit	-	30S ribosomal protein S3 OS=Listeria welshimeri serovar 6b (strain ATCC 35897 / DSM 20650 / CIP 8149 / NCTC 11857 / SLCC 5334 / V8) OX=386043 GN=rpsC PE=3 SV=1
orf_313	CP034771.1	group_1582	324175	324366	+	COG0255	J;Ribosomal protein L29;RpmC;Ribosome 50S subunit	-	50S ribosomal protein L29 OS=Listeria welshimeri serovar 6b (strain ATCC 35897 / DSM 20650 / CIP 8149 / NCTC 11857 / SLCC 5334 / V8) OX=386043 GN=rpmC PE=3 SV=1
orf_323	CP034771.1	group_1583	328174	328353	+	COG1841	J;Ribosomal protein L30/L7E;RpmD;Ribosome 50S subunit	-	50S ribosomal protein L30 OS=Listeria monocytogenes serovar 1/2a (strain ATCC BAA-679 / EGD-e) OX=169963 GN=rpmD PE=1 SV=1
orf_328	CP034771.1	group_1736	331561	331674	+	COG0257	J;Ribosomal protein L36;RpmJ;Ribosome 50S subunit	-	50S ribosomal protein L36 OS=Listeria welshimeri serovar 6b (strain ATCC 35897 / DSM 20650 / CIP 8149 / NCTC 11857 / SLCC 5334 / V8) OX=386043 GN=rpmJ PE=3 SV=1
orf_330	CP034771.1	group_1563	332081	332470	+	COG0100	J;Ribosomal protein S11;RpsK;Ribosome 30S subunit	-	30S ribosomal protein S11 OS=Listeria welshimeri serovar 6b (strain ATCC 35897 / DSM 20650 / CIP 8149 / NCTC 11857 / SLCC 5334 / V8) OX=386043 GN=rpsK PE=3 SV=1
orf_334	CP034771.1	group_1910	335315	336214	-	COG2421	C;Acetamidase/formamidase;FmdA;	-	-
orf_335	CP034771.1	group_1702	336334	336996	-	COG1285	P;Magnesium uptake protein YhiD/SapB, involved in acid resistance;SapB;	-	Putative magnesium transporter YhiD OS=Escherichia coli (strain K12) OX=83333 GN=yhiD PE=3 SV=1
orf_354	CP034771.1	group_1924	358666	359142	+	COG2427	S;Uncharacterized conserved protein YjgD, DUF1641 family;YjgD	-	Uncharacterized protein YrhD OS=Bacillus subtilis (strain 168) OX=224308 GN=yrhD PE=4 SV=1
orf_355	CP034771.1	group_2059	359209	359994	+	COG1526	C;Formate dehydrogenase assembly factor FdhD, a sulfurtransferase;FdhD;	-	Sulfur carrier protein FdhD OS=Listeria innocua serovar 6a (strain ATCC BAA-680 / CLIP 11262) OX=272626 GN=fdhD PE=3 SV=1
orf_358	CP034771.1	group_2181	361677	361967	-	COG1359	C;Quinol monooxygenase YgiN;YgiN;	-	Putative monooxygenase YcnE OS=Bacillus subtilis (strain 168) OX=224308 GN=ycnE PE=1 SV=1
orf_359	CP034771.1	group_1843	361980	362858	-	COG4814	S;Uncharacterized conserved protein with an alpha/beta hydrolase fold;;	-	-
orf_366	CP034771.1	group_1708	367430	368044	+	COG5658	V;Bacterial immunity/signal transduction membrane protein SdpI, SdpI/YbgB/	-	-
orf_367	CP034771.1	group_1444	368182	369843	+	COG4166	E;ABC-type oligopeptide transport system, periplasmic component;OppA;	-	Dipeptide-binding protein OS=Lactococcus lactis subsp. cremoris (strain MG1363) OX=416870 GN=dppA PE=1 SV=1
orf_369	CP034771.1	group_1950	370345	370545	+	-	-	-	-
orf_377	CP034771.1	group_1530	377444	379042	+	COG0504	F;CTP synthase (UTP-ammonia lyase);PyrG;Pyrimidine biosynthesis	-	CTP synthase OS=Listeria monocytogenes serovar 1/2a (strain ATCC BAA-679 / EGD-e) OX=169963 GN=pyrG PE=3 SV=1
orf_378	CP034771.1	group_2074	379207	380127	+	COG1597	IR;Phosphatidylglycerol kinase, diacylglycerol kinase family;LCB5;	-	Diacylglycerol kinase OS=Bacillus subtilis (strain 168) OX=224308 GN=dagK PE=1 SV=1
orf_382	CP034771.1	group_880	383783	384862	+	COG0392	M;Predicted membrane flippase AglD2/YbhN, UPF0104 family;AglD2;	-	UPF0104 membrane protein AF_2231 OS=Archaeoglobus fulgidus (strain ATCC 49558 / DSM 4304 / JCM 9628 / NBRC 100126 / VC-16) OX=224325 GN=AF_2231 PE=3 SV=1
orf_383	CP034771.1	group_735	384978	386249	+	COG0766	M;UDP-N-acetylglucosamine enolpyruvyl transferase;MurA;Mureine biosynth	-	UDP-N-acetylglucosamine 1-carboxyvinyltransferase 2 OS=Listeria innocua serovar 6a (strain ATCC BAA-680 / CLIP 11262) OX=272626 GN=murA2 PE=3 SV=1
orf_384	CP034771.1	group_1517	386354	387625	+	COG1158	K;Transcription termination factor Rho;Rho;	-	Transcription termination factor Rho OS=Bacillus subtilis (strain 168) OX=224308 GN=rho PE=3 SV=3
orf_385	CP034771.1	group_2156	387662	388099	+	COG2246	I;Putative flippase GtrA (transmembrane translocase of bactoprenol-linked glu	(gtcA) wall teichoic acid glycosylation protein GtcA [GtcA (VF0607)	Cell wall teichoic acid glycosylation protein GtcA OS=Listeria innocua serovar 6a (strain ATCC BAA-680 / CLIP 11262) OX=272626 GN=gtcA PE=3 SV=1
orf_387	CP034771.1	group_237	388556	389437	+	COG0583	K;DNA-binding transcriptional regulator, LysR family;LysR;	-	Uncharacterized HTH-type transcriptional regulator YwbI OS=Bacillus subtilis (strain 168) OX=224308 GN=ywbI PE=3 SV=1
orf_396	CP034771.1	group_1273	397476	398327	+	COG2890	J;Methylase of polypeptide chain release factors;HemK;	-	Release factor glutamine methyltransferase OS=Listeria monocytogenes serovar 1/2a (strain ATCC BAA-679 / EGD-e) OX=169963 GN=prmC PE=3 SV=1
orf_398	CP034771.1	group_1594	399663	400070	+	COG0394	T;Protein-tyrosine-phosphatase;Wzb;	-	Protein-arginine-phosphatase OS=Geobacillus stearothermophilus OX=1422 GN=ywle PE=1 SV=1
orf_399	CP034771.1	group_546	400206	401447	+	COG0112	E;Glycine/serine hydroxymethyltransferase;GlyA;Serine biosynthesis	-	Serine hydroxymethyltransferase OS=Listeria monocytogenes serovar 1/2a (strain ATCC BAA-679 / EGD-e) OX=169963 GN=glyA PE=3 SV=1
orf_401	CP034771.1	group_1343	402339	403478	+	COG0381	M;UDP-N-acetylglucosamine 2-epimerase;WecB;	(cps4I) capsular polysaccharide biosynthesis protein	UDP-N-acetylglucosamine 2-epimerase OS=Bacillus subtilis (strain 168) OX=224308 GN=mnaA PE=1 SV=1
orf_403	CP034771.1	group_1712	404708	405424	+	COG0356	C;FoF1-type ATP synthase, membrane subunit a;AtpB;FoF1-type ATP synthase	-	ATP synthase subunit a OS=Listeria monocytogenes serovar 1/2a (strain ATCC BAA-679 / EGD-e) OX=169963 GN=atpB PE=3 SV=1
orf_404	CP034771.1	group_1729	405495	405713	+	COG0636	C;FoF1-type ATP synthase, membrane subunit c/Archaeal/vacuolar-type H+-A	-	ATP synthase subunit c OS=Bacillus mycoides (strain KBAB4) OX=315730 GN=atpE PE=3 SV=1
orf_405	CP034771.1	group_1518	405797	406309	+	COG0711	C;FoF1-type ATP synthase, membrane subunit b or b';AtpF;FoF1-type ATP synt	-	ATP synthase subunit b OS=Listeria welshimeri serovar 6b (strain ATCC 35897 / DSM 20650 / CIP 8149 / NCTC 11857 / SLCC 5334 / V8) OX=386043 GN=atpF PE=3 SV=1
orf_406	CP034771.1	group_1403	406306	406845	+	COG0712	C;FoF1-type ATP synthase, delta subunit;AtpH;FoF1-type ATP synthase	-	ATP synthase subunit delta OS=Listeria welshimeri serovar 6b (strain ATCC 35897 / DSM 20650 / CIP 8149 / NCTC 11857 / SLCC 5334 / V8) OX=386043 GN=atpH PE=3 SV=1
orf_411	CP034771.1	group_1784	411325	411561	+	COG4836	S;Uncharacterized membrane protein YwzB;YwzB	-	Uncharacterized membrane protein YwzB OS=Bacillus subtilis (strain 168) OX=224308 GN=ywzB PE=4 SV=1
orf_412	CP034771.1	group_1542	411741	413033	+	COG0766	M;UDP-N-acetylglucosamine enolpyruvyl transferase;MurA;Mureine biosynth	-	UDP-N-acetylglucosamine 1-carboxyvinyltransferase 1 OS=Listeria innocua serovar 6a (strain ATCC BAA-680 / CLIP 11262) OX=272626 GN=murA1 PE=3 SV=1
orf_426	CP034771.1	group_1307	425428	426084	+	COG1040	R;DNA utilization protein ComFC/GntX, contains phosphoribosyltransferase dc	-	ComF operon protein 3 OS=Bacillus subtilis (strain 168) OX=224308 GN=comFC PE=3 SV=1
orf_432	CP034771.1	group_1492	432805	433689	+	COG2177	D;Cell division protein FtsX;FtsX	-	Cell division protein FtsX OS=Bacillus anthracis OX=1392 GN=ftsX PE=1 SV=1
orf_433	CP034771.1	group_508	433760	434983	+	COG3883	S;Uncharacterized N-terminal coiled-coil domain of peptidoglycan hydrolase C	-	Probable endopeptidase p60 OS=Listeria innocua serovar 6a (strain ATCC BAA-680 / CLIP 11262) OX=272626 GN=iap PE=3 SV=2
orf_436	CP034771.1	group_1359	438047	439222	+	COG0265	O;Periplasmic serine protease, S1-C subfamily, contain C-terminal PDZ domain	-	Cell division topological determinant MinJ OS=Bacillus subtilis (strain 168) OX=224308 GN=minJ PE=1 SV=1
orf_445	CP034771.1	group_176	447505	447810	+	COG0640	K;DNA-binding transcriptional regulator, ArsR family;ArsR;	-	HTH-type transcriptional repressor CzrA OS=Bacillus subtilis (strain 168) OX=224308 GN=czrA PE=1 SV=1
orf_450	CP034771.1	group_45	451470	454340	+	COG0178	L;Excinuclease UvrABC ATPase subunit;UvrA;	-	UvrABC system protein A OS=Listeria monocytogenes serovar 1/2a (strain ATCC BAA-679 / EGD-e) OX=169963 GN=uvrA PE=3 SV=1
orf_453	CP034771.1	group_1315	457124	457324	+	COG1983	KT;Phage shock protein PspC (stress-responsive transcriptional regulator);Psp(	-	Uncharacterized membrane protein YvlC OS=Bacillus subtilis (strain 168) OX=224308 GN=yvlC PE=4 SV=1
orf_459	CP034771.1	group_181	461411	461869	+	-	-	-	-
orf_461	CP034771.1	group_387	462366	463841	+	COG5010	UW;Flp pilus assembly protein TadD, contains TPR repeats;TadD;	-	TPR repeat-containing protein YvcD OS=Bacillus subtilis (strain 168) OX=224308 GN=yvcD PE=4 SV=1
orf_462	CP034771.1	group_1493	463916	464878	+	COG0492	O;Thioredoxin reductase;TrxB;	-	Thioredoxin reductase OS=Listeria monocytogenes serovar 1/2a (strain ATCC BAA-679 / EGD-e) OX=169963 GN=trxB PE=3 SV=1
orf_474	CP034771.1	group_2	477847	479331	+	COG4886	K;Leucine-rich repeat (LRR) protein;LRR;	(inlF) internalin F [InlF (VF0447) - Adherence (VFC0001)]	Internalin A OS=Listeria monocytogenes serotype 4b (strain F2365) OX=265669 GN=inlA PE=3 SV=1
orf_478	CP034771.1	group_1789	481206	483368	+	COG2409	R;Predicted lipid transporter YdfJ, MMPL/SSD domain, RND superfamily;YdfJ;	-	Apo-petrobactin exporter OS=Bacillus anthracis OX=1392 GN=apeX PE=1 SV=1
orf_482	CP034771.1	group_320	487398	488408	+	COG0057	G;Glyceraldehyde-3-phosphate dehydrogenase/erythrose-4-phosphate dehyd	-	Glyceraldehyde-3-phosphate dehydrogenase OS=Clostridium pasteurianum OX=1501 GN=gap PE=1 SV=1
orf_487	CP034771.1	group_2094	493592	493759	+	-	-	-	-
orf_488	CP034771.1	group_2095	493901	494620	+	COG0596	HR;2-succinyl-6-hydroxy-2,4-cyclohexadiene-1-carboxylate synthase MenH an	-	Soluble epoxide hydrolase OS=Corynebacterium sp. (strain C12) OX=268954 PE=1 SV=3
orf_492	CP034771.1	group_1360	496909	499290	+	COG0557	K;Exoribonuclease R;VacB;	-	Ribonuclease R OS=Bacillus subtilis (strain 168) OX=224308 GN=rnr PE=1 SV=1
orf_494	CP034771.1	group_196	499828	500739	-	-	-	-	-
orf_499	CP034771.1	group_1834	510477	511046	+	COG1434	M;Lipid carrier protein ElyC involved in cell wall biogenesis, DUF218 family;Ely	-	-
orf_500	CP034771.1	group_2033	511091	511987	+	COG2378	K;Predicted DNA-binding transcriptional regulator YobV, contains HTH and WY	-	Uncharacterized HTH-type transcriptional regulator YobV OS=Bacillus subtilis (strain 168) OX=224308 GN=yobV PE=4 SV=1
orf_503	CP034771.1	group_1899	512920	513429	+	COG4283	R;Uncharacterized conserved protein DfsB/IRC4, DUF1706 (PF08020) domain;	-	Uncharacterized protein IRC4 OS=Saccharomyces cerevisiae (strain ATCC 204508 / S288c) OX=559292 GN=IRC4 PE=1 SV=1
orf_504	CP034771.1	group_2155	513450	513827	-	COG3607	R;Lactoylglutathione lyase-related enzyme, vicinal oxygen chelate (VOC) famil	-	-
orf_506	CP034771.1	group_2072	515041	515349	-	COG5658	V;Bacterial immunity/signal transduction membrane protein SdpI, SdpI/YbgB/	-	Uncharacterized protein YfhL OS=Bacillus subtilis (strain 168) OX=224308 GN=yfhL PE=4 SV=1
orf_513	CP034771.1	group_1968	521035	522204	+	COG0772	D;Peptodoglycan polymerase FtsW/RodA/SpoVE;FtsW;	-	Peptidoglycan glycosyltransferase RodA OS=Bacillus subtilis (strain 168) OX=224308 GN=rodA PE=1 SV=1
orf_521	CP034771.1	group_1851	527802	527978	+	-	-	-	-
orf_528	CP034771.1	group_394	534477	535703	+	COG0520	E;Selenocysteine lyase/Cysteine desulfurase;CsdA;	-	Probable cysteine desulfurase OS=Alkalihalobacillus halodurans (strain ATCC BAA-125 / DSM 18197 / FERM 7344 / JCM 9153 / C-125) OX=272558 GN=csd PE=3 SV=2
orf_529	CP034771.1	group_1570	535700	536143	+	COG0822	O;Fe-S cluster assembly scaffold protein IscU, NifU family;IscU;	-	Zinc-dependent sulfurtransferase SufU OS=Bacillus subtilis (strain 168) OX=224308 GN=sufU PE=1 SV=1
orf_539	CP034771.1	group_1942	543556	543999	+	COG1670	JO;Protein N-acetyltransferase, RimJ/RimL family;RimL;	-	Probable spermine N(1)-acetyltransferase OS=Bacillus subtilis subsp. natto (strain BEST195) OX=645657 GN=bltD PE=1 SV=1
orf_548	CP034771.1	group_1923	550845	551663	+	COG3878	S;Uncharacterized conserved protein YwqG, DUF1963 family;YwqG;	-	Uncharacterized protein YwqG OS=Bacillus subtilis (strain 168) OX=224308 GN=ywqG PE=1 SV=1
orf_550	CP034771.1	group_2289	552510	553739	-	COG0038	P;H+/Cl- antiporter ClcA;ClcA;	-	Chloride channel protein CLC-f OS=Arabidopsis thaliana OX=3702 GN=CLC-F PE=2 SV=2
orf_553	CP034771.1	group_1467	555576	555965	+	COG2050	Q;Acyl-CoA thioesterase PaaI, contains HGG motif;PaaI;	-	Putative esterase ComA2 OS=Bacillus subtilis (strain 168) OX=224308 GN=yuxO PE=3 SV=2
orf_559	CP034771.1	group_1560	559160	559585	-	COG2111	P;Multisubunit Na+/H+ antiporter, MnhB subunit;MnhB	-	Na(+)/H(+) antiporter subunit B OS=Bacillus subtilis (strain 168) OX=224308 GN=mrpB PE=1 SV=1
orf_560	CP034771.1	group_315	559569	561977	-	COG2111	P;Multisubunit Na+/H+ antiporter, MnhB subunit;MnhB	-	Na(+)/H(+) antiporter subunit A OS=Bacillus subtilis (strain 168) OX=224308 GN=mrpA PE=1 SV=2
orf_562	CP034771.1	group_1571	563774	564358	+	COG0652	O;Peptidyl-prolyl cis-trans isomerase (rotamase) - cyclophilin family;PpiB;	-	Putative peptidyl-prolyl cis-trans isomerase OS=Staphylococcus epidermidis (strain ATCC 12228 / FDA PCI 1200) OX=176280 GN=SE_0648 PE=3 SV=1
orf_567	CP034771.1	group_1289	568301	569467	-	COG1168	ER;Bifunctional PLP-dependent enzyme with beta-cystathionase and maltose r	-	Cystathionine beta-lyase PatB OS=Bacillus subtilis (strain 168) OX=224308 GN=patB PE=1 SV=1
orf_571	CP034771.1	group_134	572086	572844	+	COG1349	KG;DNA-binding transcriptional regulator of sugar metabolism, DeoR/GlpR fan	-	Uncharacterized HTH-type transcriptional regulator YulB OS=Bacillus subtilis (strain 168) OX=224308 GN=yulB PE=4 SV=1
orf_572	CP034771.1	group_243	575421	576938	+	COG3711	K;Transcriptional antiterminator;BglG;	-	-
orf_574	CP034771.1	group_171	578354	579877	-	COG0531	E;Serine transporter YbeC, amino acid:H+ symporter family;PotE;	-	Glutamate/gamma-aminobutyrate antiporter OS=Lactococcus lactis subsp. lactis (strain IL1403) OX=272623 GN=gadC PE=3 SV=1
orf_577	CP034771.1	group_1898	583635	584480	+	COG0561	HR;Hydroxymethylpyrimidine pyrophosphatase and other HAD family phosph	-	Putative phosphatase YkrA OS=Bacillus subtilis (strain 168) OX=224308 GN=ykrA PE=3 SV=1
orf_578	CP034771.1	group_1962	584538	585269	+	COG0363	G;6-phosphogluconolactonase/Glucosamine-6-phosphate isomerase/deamina	-	Glucosamine-6-phosphate deaminase OS=Bacillus thuringiensis subsp. konkukian (strain 97-27) OX=281309 GN=nagB PE=3 SV=1
orf_579	CP034771.1	group_1931	585284	585814	+	COG3247	R;Acid resistance membrane protein HdeD, DUF308 family;HdeD;	-	-
orf_580	CP034771.1	group_1922	585955	586551	+	-	-	-	-
orf_581	CP034771.1	group_1993	586750	587991	+	COG2814	G;Predicted arabinose efflux permease AraJ, MFS family;AraJ;	-	Uncharacterized MFS-type transporter YttB OS=Bacillus subtilis (strain 168) OX=224308 GN=yttB PE=3 SV=1
orf_585	CP034771.1	group_2101	592649	593767	+	COG2771	K;DNA-binding transcriptional regulator, CsgD family;CsgD	-	Uncharacterized sugar kinase YeiI OS=Escherichia coli (strain K12) OX=83333 GN=yeiI PE=3 SV=2
orf_586	CP034771.1	group_2175	593764	594675	+	COG2313	F;Pseudouridine-5'-phosphate glycosidase (pseudoU degradation);PsuG;	-	Pseudouridine-5'-phosphate glycosidase OS=Lysinibacillus sphaericus (strain C3-41) OX=444177 GN=psuG PE=3 SV=1
orf_587	CP034771.1	group_1870	594755	595189	+	COG1683	S;Uncharacterized conserved protein YbbK, DUF523 family;YbbK	-	Uncharacterized protein YbbK OS=Bacillus subtilis (strain 168) OX=224308 GN=ybbK PE=4 SV=2
orf_590	CP034771.1	group_812	597476	598399	+	COG1105	G;1-phosphofructokinase or 6-phosphofructokinase II;FruK;	-	1-phosphofructokinase OS=Bacillus subtilis (strain 168) OX=224308 GN=fruK PE=3 SV=1
orf_611	CP034771.1	group_135	620054	620779	-	COG1126	E;ABC-type polar amino acid transport system, ATPase component;GlnQ;	-	Arginine transport ATP-binding protein ArtM OS=Bacillus subtilis (strain 168) OX=224308 GN=artM PE=1 SV=1
orf_616	CP034771.1	group_1717	624453	625451	-	COG0306	P;Phosphate/sulfate permease;PitA;	-	Probable low-affinity inorganic phosphate transporter OS=Bacillus subtilis (strain 168) OX=224308 GN=pit PE=3 SV=2
orf_619	CP034771.1	group_1869	627350	628006	+	COG4912	L;3-methyladenine DNA glycosylase AlkD;AlkD;	-	-
orf_620	CP034771.1	group_1598	628008	628400	+	COG0346	Q;Catechol 2,3-dioxygenase or related enzyme, vicinal oxygen chelate (VOC) f	-	Uncharacterized protein YqjT OS=Bacillus subtilis (strain 168) OX=224308 GN=yqjT PE=4 SV=1
orf_623	CP034771.1	group_2036	630007	630483	+	COG0350	L;DNA repair enzyme Ada (O6-methylguanine-DNA--protein-cysteine methyltr	-	Methylated-DNA--protein-cysteine methyltransferase, inducible OS=Bacillus subtilis (strain 168) OX=224308 GN=adaB PE=1 SV=1
orf_624	CP034771.1	group_1959	630505	631698	-	COG2271	G;Sugar phosphate permease;UhpC;	-	Inner membrane transport protein YdiM OS=Escherichia coli (strain K12) OX=83333 GN=ydiM PE=1 SV=1
orf_625	CP034771.1	group_2009	631854	633062	-	-	-	-	Inner membrane transport protein YdiM OS=Escherichia coli (strain K12) OX=83333 GN=ydiM PE=1 SV=1
orf_626	CP034771.1	group_94	633096	633965	-	COG0169	E;Shikimate 5-dehydrogenase;AroE;Aromatic amino acid biosynthesis	-	Shikimate dehydrogenase (NADP(+)) OS=Listeria monocytogenes serovar 1/2a (strain ATCC BAA-679 / EGD-e) OX=169963 GN=aroE PE=1 SV=1
orf_627	CP034771.1	group_105	634025	635950	-	COG1902	C;2,4-dienoyl-CoA reductase or related NADH-dependent reductase, Old Yello	-	7-beta-hydroxy-3-oxochol-24-oyl-CoA 4-desaturase OS=Clostridium scindens (strain JCM 10418 / VPI 12708) OX=29347 GN=baiH PE=1 SV=1
orf_629	CP034771.1	group_1987	637173	638051	+	COG0583	K;DNA-binding transcriptional regulator, LysR family;LysR;	-	Uncharacterized HTH-type transcriptional regulator YybE OS=Bacillus subtilis (strain 168) OX=224308 GN=yybE PE=3 SV=2
orf_631	CP034771.1	group_1969	639646	640515	-	COG0053	P;Divalent metal cation (Fe/Co/Zn/Cd) efflux pump;FieF;	-	Manganese efflux system protein MneS OS=Bacillus subtilis (strain 168) OX=224308 GN=mneS PE=2 SV=2
orf_633	CP034771.1	group_526	641324	643462	+	COG0744	M;Penicillin-binding protein 1B/1F, peptidoglycan transglycosylase/transpept	-	Penicillin-binding protein 2a OS=Streptococcus pneumoniae (strain ATCC BAA-255 / R6) OX=171101 GN=pbp2a PE=1 SV=1
orf_634	CP034771.1	group_1921	643571	643768	+	-	-	-	Negative regulatory protein YxlE OS=Bacillus subtilis (strain 168) OX=224308 GN=yxlE PE=1 SV=1
orf_635	CP034771.1	group_2063	643768	644673	+	COG1131	V;ABC-type multidrug transport system, ATPase component;CcmA;	-	Uncharacterized ABC transporter ATP-binding protein YxlF OS=Bacillus subtilis (strain 168) OX=224308 GN=yxlF PE=2 SV=1
orf_636	CP034771.1	group_1884	644670	645464	+	-	-	-	Uncharacterized transmembrane protein YxlG OS=Bacillus subtilis (strain 168) OX=224308 GN=yxlG PE=2 SV=2
orf_638	CP034771.1	group_1871	647005	648138	+	COG4399	S;Uncharacterized membrane protein YheB, UPF0754 family;YheB	-	UPF0754 membrane protein LMHCC_0318 OS=Listeria monocytogenes serotype 4a (strain HCC23) OX=552536 GN=LMHCC_0318 PE=3 SV=1
orf_643	CP034771.1	group_111	653703	654590	-	COG0760	O;Peptidyl-prolyl isomerase, parvulin family;SurA;	(prsA2) post translocation chaperone PrsA2 [PrsA2 (VF0449) - Post	Foldase protein PrsA 2 OS=Listeria innocua serovar 6a (strain ATCC BAA-680 / CLIP 11262) OX=272626 GN=prsA2 PE=3 SV=1
orf_646	CP034771.1	group_1537	655809	656231	-	COG0537	FR;Purine nucleoside phosphoramidase/Ap4A hydrolase, histidine triade (HIT)	-	Protein hit OS=Bacillus subtilis (strain 168) OX=224308 GN=hit PE=1 SV=1
orf_651	CP034771.1	group_1417	660209	661138	+	COG0276	H;Protoheme ferro-lyase (ferrochelatase);HemH;Heme biosynthesis	-	Coproporphyrin III ferrochelatase OS=Listeria innocua serovar 6a (strain ATCC BAA-680 / CLIP 11262) OX=272626 GN=cpfC PE=3 SV=1
orf_652	CP034771.1	group_1437	661294	661638	+	-	-	-	-
orf_659	CP034771.1	group_1892	668325	669425	+	COG1705	MN;Flagellum-specific peptidoglycan hydrolase FlgJ;FlgJ;	-	Exo-glucosaminidase LytG OS=Bacillus subtilis (strain 168) OX=224308 GN=lytG PE=1 SV=1
orf_675	CP034771.1	group_2151	686225	686497	+	-	-	-	-
orf_676	CP034771.1	group_1858	686692	687315	+	COG5386	P;Heme-binding NEAT domain;NEAT;	(hbp1/svpA) Haemoglobin binding protein 1 [SvpA (VF0263) - Nutri	Hemin/hemoglobin-binding protein 1 OS=Listeria monocytogenes serovar 1/2a (strain ATCC BAA-679 / EGD-e) OX=169963 GN=hbp1 PE=1 SV=1
orf_677	CP034771.1	group_1906	687328	689037	+	COG5386	P;Heme-binding NEAT domain;NEAT;	(hbp2) hypothetical protein [SvpA (VF0263) - Nutritional/Metabolic	Hemin/hemoglobin-binding protein 2 OS=Listeria monocytogenes serovar 1/2a (strain ATCC BAA-679 / EGD-e) OX=169963 GN=hbp2 PE=1 SV=1
orf_678	CP034771.1	group_1862	689126	689998	+	COG0614	P;ABC-type Fe3+-hydroxamate transport system, periplasmic component;Fep	(isdE) iron-regulated surface determinant protein E	High-affinity heme uptake system protein IsdE OS=Staphylococcus aureus (strain bovine RF122 / ET3-1) OX=273036 GN=isdE PE=3 SV=1
orf_680	CP034771.1	group_1325	690958	691737	+	COG1120	PH;ABC-type cobalamin/Fe3+-siderophores transport system, ATPase compon	-	Petrobactin import ATP-binding protein FpuC OS=Bacillus anthracis OX=1392 GN=fpuC PE=1 SV=1
orf_681	CP034771.1	group_1920	691742	692482	+	COG4509	S;Uncharacterized conserved protein	-	Sortase B OS=Listeria monocytogenes serovar 1/2a (strain ATCC BAA-679 / EGD-e) OX=169963 GN=srtB PE=1 SV=1
orf_682	CP034771.1	group_153	692546	693022	+	-	-	-	-
orf_683	CP034771.1	group_40	693484	697797	+	COG4932	M;Clumping factor A-related surface protein, MSCRAMM (microbial surface cc	-	Internalin J OS=Listeria monocytogenes serovar 1/2a (strain ATCC BAA-679 / EGD-e) OX=169963 GN=inlJ PE=1 SV=1
orf_684	CP034771.1	group_40	698176	701742	+	COG4932	M;Clumping factor A-related surface protein, MSCRAMM (microbial surface cc	-	-
orf_685	CP034771.1	group_40	701769	703166	+	COG4932	M;Clumping factor A-related surface protein, MSCRAMM (microbial surface cc	-	Internalin J OS=Listeria monocytogenes serovar 1/2a (strain ATCC BAA-679 / EGD-e) OX=169963 GN=inlJ PE=1 SV=1
orf_686	CP034771.1	group_2167	703208	703549	-	-	-	-	Uncharacterized protein YvlA OS=Bacillus subtilis (strain 168) OX=224308 GN=yvlA PE=4 SV=1
orf_687	CP034771.1	group_40	703913	709297	+	COG4932	M;Clumping factor A-related surface protein, MSCRAMM (microbial surface cc	-	Internalin J OS=Listeria monocytogenes serotype 4b (strain F2365) OX=265669 GN=inlJ PE=3 SV=1
orf_688	CP034771.1	group_2278	709724	711091	+	COG3829	KT;RocR-type transcriptional regulator, contains PAS, AAA-type ATPase, and D	-	Transcriptional regulatory protein FlbD OS=Caulobacter vibrioides (strain ATCC 19089 / CB15) OX=190650 GN=flbD PE=4 SV=2
orf_689	CP034771.1	group_2252	711320	712903	+	COG4670	I;Acyl CoA:acetate/3-ketoacid CoA transferase;YdiF;	-	Caffeate CoA-transferase OS=Acetobacterium woodii OX=33952 GN=carA PE=1 SV=1
orf_690	CP034771.1	group_2265	712900	714123	+	COG2223	P;Nitrate/nitrite transporter NarK;NarK;	-	L-lactate transporter OS=Syntrophobacter fumaroxidans (strain DSM 10017 / MPOB) OX=335543 GN=Sfum_3364 PE=1 SV=1
orf_691	CP034771.1	group_120	714146	715075	+	COG2070	R;NAD(P)H-dependent flavin oxidoreductase YrpB, nitropropane dioxygenase	-	Probable nitronate monooxygenase OS=Staphylococcus saprophyticus subsp. saprophyticus (strain ATCC 15305 / DSM 20229 / NCIMB 8711 / NCTC 7292 / S-41) OX=342451 GN=SSP1854 PE=3 SV=1
orf_695	CP034771.1	group_1939	716485	717156	+	COG0664	T;cAMP-binding domain of CRP or a regulatory subunit of cAMP-dependent pr	-	-
orf_696	CP034771.1	group_2253	717406	718101	+	COG0664	T;cAMP-binding domain of CRP or a regulatory subunit of cAMP-dependent pr	-	-
orf_697	CP034771.1	group_1970	718173	719051	+	COG2207	K;AraC-type DNA-binding domain and AraC-containing proteins;AraC;	-	Uncharacterized HTH-type transcriptional regulator YdeC OS=Bacillus subtilis (strain 168) OX=224308 GN=ydeC PE=4 SV=1
orf_698	CP034771.1	group_1786	719125	720201	+	COG0673	R;Predicted dehydrogenase;MviM;	-	D-glucoside 3-dehydrogenase OS=Escherichia coli (strain K12) OX=83333 GN=ycjS PE=1 SV=1
orf_699	CP034771.1	group_1878	720217	720957	+	COG1082	G;Sugar phosphate isomerase/epimerase;YcjR;	-	Rhizopine catabolism protein MocC OS=Rhizobium meliloti OX=382 GN=mocC PE=3 SV=1
orf_700	CP034771.1	group_2082	720959	721681	+	COG4813	G;Trehalose utilization protein;ThuA;	-	-
orf_701	CP034771.1	group_2102	721683	722651	+	COG1082	G;Sugar phosphate isomerase/epimerase;YcjR;	-	Uncharacterized protein YfiH OS=Bacillus subtilis (strain 168) OX=224308 GN=yfiH PE=4 SV=2
orf_702	CP034771.1	group_1913	722672	723721	+	COG0673	R;Predicted dehydrogenase;MviM;	-	Uncharacterized oxidoreductase C26H5.09c OS=Schizosaccharomyces pombe (strain 972 / ATCC 24843) OX=284812 GN=SPAC26H5.09c PE=2 SV=3
orf_703	CP034771.1	group_1544	723816	724001	+	COG3237	S;Uncharacterized conserved protein YjbJ, UPF0337 family;yjbJ;	-	UPF0337 protein lin2261 OS=Listeria innocua serovar 6a (strain ATCC BAA-680 / CLIP 11262) OX=272626 GN=lin2261 PE=3 SV=1
orf_704	CP034771.1	group_1955	724081	724422	-	-	-	-	-
