# Supplementary material for: Comparative Genomics Reveal the Utilization Ability of Variable Carbohydrates as Key Genetic Features of Listeria Pathogens in Their Pathogenic Lifestyles
Source: Pathogens. 2022 Nov 28;11(12):1430. doi: 10.3390/pathogens11121430 (PMC9784484; doi:10.3390/pathogens11121430)
Supplement: Supplementary file 1 [file pathogens-11-01430-s001.zip › pathogens-1990707-supplementary/Supplymentary Tables S6.pdf]

Table S6. Unique genes of Listeria pathogens (UGLP) referenced to L.monocytogenes EGD-e_169963

ORF_id	Sequence_id	Group_id	start	end	strand	COG_id	COG_annotation	VFDB_annotation	Uniprot_annotation
orf_5	AL591824.1	group_1688	4869	5981	+	COG1195	L;Recombinational DNA repair ATPase RecF;RecF;	-	DNA replication and repair protein RecF OS=Listeria monocytogenes serovar 1/2a (strain ATCC BAA-679 / EGD-e) OX=169963 GN=recF PE=3 SV=1
orf_8	AL591824.1	group_331	10728	12242	+	COG1502	I;Phosphatidylserine/phosphatidylglycerophosphate/cardiolipin synt	-	Cardiolipin synthase 1 OS=Bacillus cereus (strain ATCC 14579 / DSM 31 / CCUG 7414 / JCM 2152 / NBRC 15305 / NCIMB 9373 / NCTC 2599 / NRRL B-3711) OX=226900 GN=cls1 PE=3 SV=1
orf_9	AL591824.1	group_1722	12258	12776	+	COG1670	JO;Protein N-acetyltransferase, RimJ/RimL family;RimL;	-	Spermidine N(1)-acetyltransferase OS=Wigglesworthia glossinidia brevipalpis OX=36870 GN=speG PE=3 SV=1
orf_11	AL591824.1	group_1364	13843	14814	+	COG3407	I;Mevalonate pyrophosphate decarboxylase;MVD1;Isoprenoid biosy	-	-
orf_13	AL591824.1	group_537	16219	17325	+	COG1622	C;Heme/copper-type cytochrome/quinol oxidase, subunit 2;CyoA;	-	Probable quinol oxidase subunit 2 OS=Staphylococcus epidermidis (strain ATCC 12228 / FDA PCI 1200) OX=176280 GN=qoxA PE=3 SV=1
orf_15	AL591824.1	group_1819	19311	19922	+	COG1845	C;Heme/copper-type cytochrome/quinol oxidase, subunit 3;CyoC;	-	Quinol oxidase subunit 3 OS=Bacillus subtilis (strain 168) OX=224308 GN=qoxC PE=1 SV=1
orf_16	AL591824.1	group_1520	19924	20256	+	COG3125	C;Heme/copper-type cytochrome/quinol oxidase, subunit 4;CyoD	-	Quinol oxidase subunit 4 OS=Bacillus spizizenii (strain ATCC 23059 / NRRL B-14472 / W23) OX=655816 GN=qoxD PE=1 SV=1
orf_22	AL591824.1	group_2197	25351	25839	+	COG3444	G;Phosphotransferase system, mannose/fructose/N-acetylgalactosa	-	PTS system sorbose-specific EIIB component OS=Lactobacillus casei OX=1582 GN=sorB PE=1 SV=1
orf_24	AL591824.1	group_20	26646	27467	+	COG3716	G;Phosphotransferase system, mannose/fructose/N-acetylgalactosa	-	-
orf_27	AL591824.1	group_9	29100	31004	+	COG2190	G;Phosphotransferase system IIA component;NagE;	-	PTS system beta-glucoside-specific EIIBCA component OS=Alkalihalobacillus halodurans (strain ATCC BAA-125 / DSM 18197 / FERM 7344 / JCM 9153 / C-125) OX=272558 GN=bglP PE=3 SV=1
orf_31	AL591824.1	group_2241	33397	34452	-	COG1609	K;DNA-binding transcriptional regulator, LacI/PurR family;PurR;	-	-
orf_32	AL591824.1	group_2260	34654	35619	+	COG1940	GK;Sugar kinase of the NBD/HSP70 family, may contain an N-termina	-	-
orf_33	AL591824.1	group_2212	35616	38018	+	-	-	-	-
orf_35	AL591824.1	group_2236	39385	40470	+	COG0449	M;Glucosamine 6-phosphate synthetase, contains amidotransferase	-	-
orf_60	AL591824.1	group_264	64950	66146	+	COG4499	U;ESX protein secretion system component YukC;YukC;	-	-
orf_61	AL591824.1	group_223	66160	70656	+	COG1674	D;DNA segregation ATPase FtsK/SpoIIIE or related protein;FtsK;	-	Type VII secretion system protein EssC OS=Staphylococcus aureus (strain N315) OX=158879 GN=essC PE=3 SV=1
orf_75	AL591824.1	group_1268	79817	80869	+	COG2169	L;Methylphosphotriester-DNA--protein-cysteine methyltransferase (	-	Methylated-DNA--protein-cysteine methyltransferase OS=Shigella flexneri OX=623 GN=ogt PE=3 SV=1
orf_76	AL591824.1	group_2078	80892	81530	-	COG1357	S;Uncharacterized conserved protein YjbI, contains pentapeptide rep	-	-
orf_77	AL591824.1	group_450	81661	82617	+	COG1052	CHR;Lactate dehydrogenase or related 2-hydroxyacid dehydrogenase	-	Putative 2-hydroxyacid dehydrogenase SH0752 OS=Staphylococcus haemolyticus (strain JCSC1435) OX=279808 GN=SH0752 PE=3 SV=1
orf_82	AL591824.1	group_42	86323	86691	-	COG0789	K;DNA-binding transcriptional regulator, MerR family;SoxR;	-	HTH-type transcriptional regulator HmrR OS=Rhizobium leguminosarum bv. viciae OX=387 GN=hmrR PE=4 SV=1
orf_84	AL591824.1	group_2070	88121	88810	+	COG0664	T;cAMP-binding domain of CRP or a regulatory subunit of cAMP-dep	-	-
orf_86	AL591824.1	group_1611	94764	97070	+	-	-	-	-
orf_88	AL591824.1	group_1918	97366	98412	+	-	-	-	-
orf_90	AL591824.1	group_2084	99902	100771	+	COG0224	C;FoF1-type ATP synthase, gamma subunit;AtpG;FoF1-type ATP synt	-	-
orf_92	AL591824.1	group_1947	102155	102478	+	COG0355	C;FoF1-type ATP synthase, epsilon subunit;AtpC;FoF1-type ATP synt	-	-
orf_93	AL591824.1	group_1919	102468	103028	+	-	-	-	-
orf_96	AL591824.1	group_114	105123	105929	+	COG3715	G;Phosphotransferase system, mannose/fructose/N-acetylgalactosa	-	PTS system sorbose-specific EIIC component OS=Klebsiella pneumoniae OX=573 GN=sorA PE=2 SV=1
orf_99	AL591824.1	group_2097	107499	107849	+	COG4898	S;Uncharacterized conserved protein	-	-
orf_102	AL591824.1	group_2046	109349	109720	+	-	-	-	-
orf_103	AL591824.1	group_1371	110013	112283	+	COG3979	G;Chitodextrinase;;	-	-
orf_106	AL591824.1	group_2083	115087	116829	-	COG1132	V;ABC-type multidrug transport system, ATPase and permease comp	-	-
orf_108	AL591824.1	group_2262	117841	118956	+	COG0657	I;Acetyl esterase/lipase;Aes;	-	-
orf_109	AL591824.1	group_1984	119060	119770	+	COG2200	T;EAL domain, c-di-GMP-specific phosphodiesterase class I (or its en	-	-
orf_113	AL591824.1	group_185	121664	122092	+	-	-	-	-
orf_114	AL591824.1	group_144	122104	122520	+	-	-	-	-
orf_126	AL591824.1	group_221	130249	130671	+	COG4824	X;Phage-related holin (Lysis protein)	-	Uncharacterized protein Lmo0128 OS=Listeria monocytogenes serovar 1/2a (strain ATCC BAA-679 / EGD-e) OX=169963 GN=lmo0128 PE=3 SV=1
orf_127	AL591824.1	group_1849	130652	131380	+	COG0860	M;N-acetylmuramoyl-L-alanine amidase;AmiC;	-	Sporulation-specific N-acetylmuramoyl-L-alanine amidase OS=Bacillus subtilis (strain 168) OX=224308 GN=cwlC PE=1 SV=1
orf_128	AL591824.1	group_1295	131419	133767	-	COG0737	FV;2',3'-cyclic-nucleotide 2'-phosphodiesterase/5'- or 3'-nucleotidase	-	Cell wall protein Lmo0130 OS=Listeria monocytogenes serovar 1/2a (strain ATCC BAA-679 / EGD-e) OX=169963 GN=lmo0130 PE=1 SV=1
orf_130	AL591824.1	group_562	134782	136290	-	COG4109	K;Predicted transcriptional regulator containing CBS domains;YtoI	-	Inosine-5'-monophosphate dehydrogenase OS=Tritrichomonas foetus OX=56690 GN=IMPDH PE=1 SV=1
orf_132	AL591824.1	group_1596	136714	136992	+	COG2388	R;Predicted acetyltransferase, GNAT superfamily;YidJ;	-	Uncharacterized protein YjdJ OS=Escherichia coli (strain K12) OX=83333 GN=yjdJ PE=1 SV=1
orf_133	AL591824.1	group_1692	137323	138897	+	COG0747	E;ABC-type transport system, periplasmic component;DdpA;	-	-
orf_153	AL591824.1	group_2238	152648	153328	-	COG0664	T;cAMP-binding domain of CRP or a regulatory subunit of cAMP-dep	-	-
orf_154	AL591824.1	group_1863	153608	155947	+	COG1199	L;Rad3-related DNA helicase DinG;DinG;	-	-
orf_155	AL591824.1	group_81	155993	156805	+	COG0561	HR;Hydroxymethylpyrimidine pyrophosphatase and other HAD famil	-	Putative phosphatase YxeH OS=Bacillus subtilis (strain 168) OX=224308 GN=yxeH PE=3 SV=1
orf_156	AL591824.1	group_125	157089	159470	+	COG4932	M;Clumping factor A-related surface protein, MSCRAMM (microbial	-	-
orf_157	AL591824.1	group_125	159663	161378	+	COG4932	M;Clumping factor A-related surface protein, MSCRAMM (microbial	-	-
orf_158	AL591824.1	group_1449	161420	162250	-	COG1366	T;Anti-anti-sigma regulatory factor (antagonist of anti-sigma factor);	-	-
orf_160	AL591824.1	group_1817	163465	164298	+	COG1774	T;Cell fate regulator YaaT, PSP1 superfamily (controls sporulation, co	-	Stage 0 sporulation protein YaaT OS=Bacillus subtilis (strain 168) OX=224308 GN=yaaT PE=4 SV=1
orf_162	AL591824.1	group_1328	164735	165508	+	COG4123	J;tRNA1(Val) A37 N6-methylase TrmN6;TrmN6;tRNA modification	-	Probable RNA methyltransferase YabB OS=Bacillus subtilis (strain 168) OX=224308 GN=yabB PE=3 SV=1
orf_163	AL591824.1	group_1334	165492	165764	+	COG2827	L;Predicted endonuclease, GIY-YIG superfamily;YhbQ;	-	UPF0213 protein lmo0166 OS=Listeria monocytogenes serovar 1/2a (strain ATCC BAA-679 / EGD-e) OX=169963 GN=lmo0166 PE=3 SV=1
orf_192	AL591824.1	group_160	198201	199409	+	COG0577	V;ABC-type antimicrobial peptide transport system, permease comp	-	Macrolide export ATP-binding/permease protein MacB OS=Bdellovibrio bacteriovorus (strain ATCC 15356 / DSM 50701 / NCIMB 9529 / HD100) OX=264462 GN=macB PE=3 SV=1
orf_196	AL591824.1	group_1719	202641	203597	+	COG0462	F;Phosphoribosylpyrophosphate synthetase;PrsA;Histidine biosynthe	-	Ribose-phosphate pyrophosphokinase 1 OS=Listeria monocytogenes serovar 1/2a (strain ATCC BAA-679 / EGD-e) OX=169963 GN=prs1 PE=3 SV=1
orf_198	AL591824.1	group_2339	204624	205577	-	-	-	(plcA) phosphatidylinositol-specific phospholipase c [PlcA (VF0063) - Exot	1-phosphatidylinositol phosphodiesterase OS=Listeria monocytogenes serovar 1/2a (strain ATCC BAA-679 / EGD-e) OX=169963 GN=plcA PE=1 SV=1
orf_199	AL591824.1	group_2330	205819	207408	+	-	-	(hly) listeriolysin O precursor [LLO (VF0064) - Exotoxin (VFC0235)] [Listeri	Listeriolysin O OS=Listeria monocytogenes serovar 1/2a (strain ATCC BAA-679 / EGD-e) OX=169963 GN=hly PE=1 SV=1
orf_200	AL591824.1	group_2323	207739	209271	+	COG3227	O;Zn-dependent metalloprotease (Neutral protease B);LasB;	(mpl) Zinc metalloproteinase precursor [Mpl (VF0065) - Exoenzyme (VFC	Zinc metalloproteinase OS=Listeria monocytogenes serovar 1/2a (strain ATCC BAA-679 / EGD-e) OX=169963 GN=mpl PE=2 SV=1
orf_202	AL591824.1	group_2325	211425	212294	+	-	-	(plcB) phospholipase C [PlcB (VF0067) - Exotoxin (VFC0235)] [Listeria mon	Phospholipase C OS=Listeria monocytogenes serovar 1/2a (strain ATCC BAA-679 / EGD-e) OX=169963 GN=plcB PE=1 SV=1
orf_208	AL591824.1	group_1543	215721	216344	+	COG1825	J;Ribosomal protein L25 (general stress protein Ctc);RplY;Ribosome	-	50S ribosomal protein L25 OS=Listeria monocytogenes serovar 1/2a (strain ATCC BAA-679 / EGD-e) OX=169963 GN=rplY PE=3 SV=1
orf_209	AL591824.1	group_1276	216434	217018	+	COG0456	J;Ribosomal protein S18 acetylase RimI and related acetyltransferase	-	-
orf_212	AL591824.1	group_484	221370	222959	+	COG5841	D;Stage V sporulation protein SpoVB/SpoIIIF, required for spore cort	-	-
orf_213	AL591824.1	group_1495	222983	223261	+	COG1188	J;Ribosomal 50S subunit-recycling heat shock protein, contains S4 do	-	Uncharacterized protein Lmo0216 OS=Listeria monocytogenes serovar 1/2a (strain ATCC BAA-679 / EGD-e) OX=169963 GN=lmo0216 PE=4 SV=2
orf_217	AL591824.1	group_240	226854	228929	+	COG0465	O;ATP-dependent Zn proteases;HflB;	-	ATP-dependent zinc metalloprotease FtsH OS=Bacillus subtilis (strain 168) OX=224308 GN=ftsH PE=1 SV=1
orf_220	AL591824.1	group_365	230840	231766	+	COG0031	E;Cysteine synthase;CysK;Cysteine biosynthesis	-	Cysteine synthase OS=Staphylococcus epidermidis (strain ATCC 12228 / FDA PCI 1200) OX=176280 GN=cysK PE=3 SV=1
orf_221	AL591824.1	group_1333	232692	233477	+	COG0294	H;Dihydropteroate synthase;FolP;Folate biosynthesis	-	Dihydropteroate synthase OS=Bacillus subtilis (strain 168) OX=224308 GN=sul PE=3 SV=2
orf_223	AL591824.1	group_1603	233858	234337	+	COG0801	H;7,8-dihydro-6-hydroxymethylpterin pyrophosphokinase (folate bio	-	2-amino-4-hydroxy-6-hydroxymethyldihydropteridine pyrophosphokinase OS=Bacillus subtilis (strain 168) OX=224308 GN=folK PE=3 SV=2
orf_224	AL591824.1	group_1687	234414	235409	+	COG0042	J;tRNA-dihydrouridine synthase;DusA;tRNA modification	-	Probable tRNA-dihydrouridine synthase 1 OS=Bacillus subtilis (strain 168) OX=224308 GN=dus1 PE=3 SV=1
orf_225	AL591824.1	group_1471	235524	237020	+	COG1190	J;Lysyl-tRNA synthetase (class II);LysU;Aminoacyl-tRNA synthetases	-	Lysine--tRNA ligase OS=Listeria monocytogenes serovar 1/2a (strain ATCC BAA-679 / EGD-e) OX=169963 GN=lysS PE=3 SV=1
orf_232	AL591824.1	group_1422	255794	256498	+	COG1211	I;2-C-methyl-D-erythritol 4-phosphate cytidylyltransferase;IspD;Isop	-	2-C-methyl-D-erythritol 4-phosphate cytidylyltransferase OS=Listeria monocytogenes serovar 1/2a (strain ATCC BAA-679 / EGD-e) OX=169963 GN=ispD PE=3 SV=1
orf_238	AL591824.1	group_949	261306	262061	+	COG0566	J;tRNA G18 (ribose-2'-O)-methylase SpoU;SpoU;tRNA modification	-	Putative TrmH family tRNA/rRNA methyltransferase YacO OS=Bacillus subtilis (strain 168) OX=224308 GN=yacO PE=3 SV=2
orf_240	AL591824.1	group_1675	262657	263262	+	COG1595	K;DNA-directed RNA polymerase specialized sigma subunit, sigma24	-	RNA polymerase sigma-H factor OS=Bacillus licheniformis OX=1402 GN=sigH PE=3 SV=2
orf_241	AL591824.1	group_1527	263535	263714	+	COG0690	U;Preprotein translocase subunit SecE;SecE;	-	Protein translocase subunit SecE OS=Bacillus licheniformis OX=1402 GN=secE PE=3 SV=1
orf_243	AL591824.1	group_1830	264432	264902	+	-	-	-	-
orf_244	AL591824.1	group_1731	265029	265454	+	COG0080	J;Ribosomal protein L11;RplK;Ribosome 50S subunit	-	50S ribosomal protein L11 OS=Listeria welshimeri serovar 6b (strain ATCC 35897 / DSM 20650 / CIP 8149 / NCTC 11857 / SLCC 5334 / V8) OX=386043 GN=rplK PE=3 SV=1
orf_252	AL591824.1	group_1331	270372	270977	+	COG2813	J;16S rRNA G1207 methylase RsmC;RsmC;16S rRNA modification	-	Uncharacterized protein YbxB OS=Bacillus subtilis (strain 168) OX=224308 GN=ybxB PE=3 SV=1
orf_256	AL591824.1	group_2118	280410	280955	+	COG1011	H;FMN and 5-amino-6-(5-phospho-D-ribitylamino)uracil phosphatase	-	-
orf_258	AL591824.1	group_2	282755	284227	+	COG4886	K;Leucine-rich repeat (LRR) protein;LRR;	-	Internalin H OS=Listeria monocytogenes serovar 1/2a (strain ATCC BAA-679 / EGD-e) OX=169963 GN=inlH PE=1 SV=1
orf_259	AL591824.1	group_2	284365	286011	+	COG4886	K;Leucine-rich repeat (LRR) protein;LRR;	-	Internalin H OS=Listeria monocytogenes serovar 1/2a (strain ATCC BAA-679 / EGD-e) OX=169963 GN=inlH PE=1 SV=1
orf_260	AL591824.1	group_2	286219	287718	+	COG4886	K;Leucine-rich repeat (LRR) protein;LRR;	-	Internalin H OS=Listeria monocytogenes serovar 1/2a (strain ATCC BAA-679 / EGD-e) OX=169963 GN=inlH PE=1 SV=1
orf_261	AL591824.1	group_1840	287853	288992	+	COG0624	E;Acetylornithine deacetylase/Succinyl-diaminopimelate desuccinyla	-	Probable succinyl-diaminopimelate desuccinylase OS=Staphylococcus aureus (strain MRSA252) OX=282458 GN=dapE PE=3 SV=1
orf_264	AL591824.1	group_488	290593	291228	+	COG0406	G;Broad specificity phosphatase PhoE;PhoE;	-	-
orf_268	AL591824.1	group_81	295401	296213	-	COG0561	HR;Hydroxymethylpyrimidine pyrophosphatase and other HAD famil	-	Putative phosphatase YxeH OS=Bacillus subtilis (strain 168) OX=224308 GN=yxeH PE=3 SV=1
orf_269	AL591824.1	group_1299	296349	296852	+	-	-	-	-
orf_270	AL591824.1	group_2237	296889	297551	-	COG0664	T;cAMP-binding domain of CRP or a regulatory subunit of cAMP-dep	-	-
orf_271	AL591824.1	group_2062	297810	298652	-	COG2333	U;DNA uptake channel protein ComEC C-terminal domain, metallo-b	-	-
orf_273	AL591824.1	group_2048	299604	300578	+	COG0673	R;Predicted dehydrogenase;MviM;	-	-
orf_274	AL591824.1	group_1516	300617	301717	-	COG3839	G;ABC-type sugar transport system, ATPase component MalK;MalK;	-	Oligosaccharides import ATP-binding protein MsmX OS=Bacillus subtilis (strain 168) OX=224308 GN=msmX PE=1 SV=1
orf_276	AL591824.1	group_489	304133	304684	+	COG0602	H;Organic radical activating enzyme NrdG/QueE;QueE;tRNA modifica	-	Anaerobic ribonucleoside-triphosphate reductase-activating protein OS=Salmonella typhi OX=90370 GN=nrdG PE=3 SV=1
orf_277	AL591824.1	group_1893	304942	305613	-	COG0664	T;cAMP-binding domain of CRP or a regulatory subunit of cAMP-dep	-	-
orf_279	AL591824.1	group_571	306644	307306	-	COG2011	E;ABC-type methionine transport system, permease component;Me	-	Probable D-methionine transport system permease protein MetI OS=Vibrio cholerae serotype O1 (strain ATCC 39315 / El Tor Inaba N16961) OX=243277 GN=metI PE=3 SV=1
orf_280	AL591824.1	group_1490	307303	308319	-	COG1135	E;ABC-type methionine transport system, ATPase component;AbcC;	-	Methionine import ATP-binding protein MetN 1 OS=Listeria monocytogenes serovar 1/2a (strain ATCC BAA-679 / EGD-e) OX=169963 GN=metN1 PE=3 SV=1
orf_281	AL591824.1	group_1505	308334	309155	-	COG1464	P;ABC-type metal ion transport system, periplasmic component/surf	-	D-methionine-binding lipoprotein MetQ OS=Salmonella typhi OX=90370 GN=metQ PE=3 SV=1
orf_285	AL591824.1	group_530	313659	314981	+	COG4863	T;YycH protein, regulator of the WalKR two-component signal transd	-	-
orf_287	AL591824.1	group_222	315945	316775	+	COG1235	P;Phosphoribosyl 1,2-cyclic phosphate phosphodiesterase;PhnP;	-	Putative metallo-hydrolase YycJ OS=Bacillus subtilis (strain 168) OX=224308 GN=yycJ PE=2 SV=1
orf_288	AL591824.1	group_1404	316873	318375	+	COG0265	O;Periplasmic serine protease, S1-C subfamily, contain C-terminal PD	-	Uncharacterized serine protease YyxA OS=Bacillus subtilis (strain 168) OX=224308 GN=yyxA PE=3 SV=2
orf_292	AL591824.1	group_1595	321490	322128	+	-	-	-	-
orf_329	AL591824.1	group_28	360936	366272	+	COG4886	K;Leucine-rich repeat (LRR) protein;LRR;	-	Internalin I OS=Listeria monocytogenes serovar 1/2a (strain ATCC BAA-679 / EGD-e) OX=169963 GN=inlI PE=2 SV=1
orf_352	AL591824.1	group_806	383710	384726	+	COG0673	R;Predicted dehydrogenase;MviM;	-	Uncharacterized oxidoreductase YhhX OS=Escherichia coli (strain K12) OX=83333 GN=yhhX PE=1 SV=1
orf_368	AL591824.1	group_1483	397340	398053	-	COG2188	K;DNA-binding transcriptional regulator, GntR family;MngR;	-	-
orf_372	AL591824.1	group_2017	401386	401580	+	-	-	-	-
orf_373	AL591824.1	group_229	401619	402539	-	COG2378	K;Predicted DNA-binding transcriptional regulator YobV, contains HT	-	-
orf_374	AL591824.1	group_1842	402641	403060	+	-	-	-	-
orf_380	AL591824.1	group_134	405680	406441	-	COG1349	KG;DNA-binding transcriptional regulator of sugar metabolism, DeoR	-	HTH-type transcriptional regulator IolR OS=Bacillus subtilis (strain 168) OX=224308 GN=iolR PE=4 SV=1
orf_382	AL591824.1	group_2227	408116	408937	+	COG3718	G;5-deoxy-D-glucuronate isomerase;IolB;	-	5-deoxy-glucuronate isomerase OS=Listeria monocytogenes serovar 1/2a (strain ATCC BAA-679 / EGD-e) OX=169963 GN=iolB PE=3 SV=1
orf_383	AL591824.1	group_1861	408954	409931	+	COG0524	G;Sugar or nucleoside kinase, ribokinase family;RbsK;Glycolysis	-	5-dehydro-2-deoxygluconokinase OS=Listeria monocytogenes serovar 1/2a (strain ATCC BAA-679 / EGD-e) OX=169963 GN=iolC PE=3 SV=1
orf_384	AL591824.1	group_2182	409941	411860	+	COG3962	G;TPP-dependent trihydroxycyclohexane-1,2-dione (THcHDO) dehyd	-	3D-(3,5/4)-trihydroxycyclohexane-1,2-dione hydrolase OS=Listeria monocytogenes serovar 1/2a (strain ATCC BAA-679 / EGD-e) OX=169963 GN=iolD PE=3 SV=1
orf_385	AL591824.1	group_1901	411992	412363	+	COG5646	OP;Iron-binding protein Fra/YdhG, frataxin family (Fe-S cluster biosy	-	Intracellular iron chaperone frataxin OS=Bacillus subtilis (strain 168) OX=224308 GN=fra PE=1 SV=1
orf_392	AL591824.1	group_1631	416796	417479	+	COG3103	R;Uncharacterized conserved protein YgiM, contains N-terminal SH3	-	-
orf_393	AL591824.1	group_461	417535	417960	+	COG0456	J;Ribosomal protein S18 acetylase RimI and related acetyltransferase	-	-
orf_395	AL591824.1	group_1864	418891	419373	+	COG3832	I;Chalcone/flavanone-binding protein YndB, AHSA1/START/SRPBCC d	-	-
orf_397	AL591824.1	group_6	419999	420331	+	COG1445	G;Phosphotransferase system fructose-specific component IIB;FrwB;	-	PTS system fructose-specific EIIB component OS=Haloferax volcanii (strain ATCC 29605 / DSM 3757 / JCM 8879 / NBRC 14742 / NCIMB 2012 / VKM B-1768 / DS2) OX=309800 GN=ptfB PE=2 SV=1
orf_401	AL591824.1	group_339	426190	426606	+	-	-	-	-
orf_402	AL591824.1	group_2193	426597	426995	+	-	-	-	-
orf_404	AL591824.1	group_1875	428127	428507	+	COG0346	Q;Catechol 2,3-dioxygenase or related enzyme, vicinal oxygen chelat	-	Uncharacterized protein YyaH OS=Bacillus subtilis (strain 168) OX=224308 GN=yyaH PE=4 SV=1
orf_405	AL591824.1	group_1975	428576	428968	+	COG4835	S;Uncharacterized conserved protein;;	-	-
orf_407	AL591824.1	group_2	429630	432095	+	COG4886	K;Leucine-rich repeat (LRR) protein;LRR;	(inlF) internalin F [InlF (VF0447) - Adherence (VFC0001)] [Listeria monocy	Internalin A OS=Listeria monocytogenes serotype 4b (strain F2365) OX=265669 GN=inlA PE=3 SV=1
orf_408	AL591824.1	group_297	432143	433171	-	COG0574	G;Phosphoenolpyruvate synthase/pyruvate phosphate dikinase;Pps	-	-
orf_409	AL591824.1	group_297	433153	434745	-	COG0574	G;Phosphoenolpyruvate synthase/pyruvate phosphate dikinase;Pps	-	Putative phosphoenolpyruvate synthase OS=Bacillus subtilis (strain 168) OX=224308 GN=pps PE=3 SV=1
orf_410	AL591824.1	group_326	434945	435817	-	COG3064	M;Membrane protein TolA involved in colicin uptake;TolA;	-	-
orf_412	AL591824.1	group_1994	436379	437188	+	COG0428	P;Zinc transporter ZupT;ZupT;	-	Zinc transporter ZIP11 OS=Rattus norvegicus OX=10116 GN=Slc39a11 PE=2 SV=1
orf_415	AL591824.1	group_181	439214	439624	+	-	-	-	-
orf_416	AL591824.1	group_1976	439677	439976	-	COG1974	KT;SOS-response transcriptional repressor LexA (RecA-mediated aut	-	-
orf_425	AL591824.1	group_6	447471	447791	+	COG1445	G;Phosphotransferase system fructose-specific component IIB;FrwB;	-	PTS system fructose-like EIIB component 2 OS=Shigella flexneri OX=623 GN=frwB PE=3 SV=1
orf_428	AL591824.1	group_237	451531	452406	-	COG0583	K;DNA-binding transcriptional regulator, LysR family;LysR;	-	-
orf_429	AL591824.1	group_1271	452524	453093	+	COG0110	R;Acetyltransferase, isoleucine patch superfamily;WbbJ	-	Putative acetyltransferase SH0499 OS=Staphylococcus haemolyticus (strain JCSC1435) OX=279808 GN=SH0499 PE=3 SV=1
orf_430	AL591824.1	group_378	453107	453853	+	COG4221	C;NADP-dependent 3-hydroxy acid dehydrogenase YdfG;YdfG;Pyrimi	-	Uncharacterized oxidoreductase Lmo0432 OS=Listeria monocytogenes serovar 1/2a (strain ATCC BAA-679 / EGD-e) OX=169963 GN=lmo0432 PE=3 SV=2
orf_431	AL591824.1	group_2	454534	456936	+	COG4886	K;Leucine-rich repeat (LRR) protein;LRR;	(inlA) Internalin A [InlA (VF0069) - Invasion (VFC0083)] [Listeria monocyto	Internalin A OS=Listeria monocytogenes serovar 1/2a (strain ATCC BAA-679 / EGD-e) OX=169963 GN=inlA PE=1 SV=1
orf_432	AL591824.1	group_39	457021	458913	+	COG5632	M;N-acetylmuramoyl-L-alanine amidase CwlA;CwlA	(inlB) Internalin B [InlB (VF0070) - Invasion (VFC0083)] [Listeria monocyto	Internalin B OS=Listeria monocytogenes serovar 1/2a (strain ATCC BAA-679 / EGD-e) OX=169963 GN=inlB PE=1 SV=1
orf_437	AL591824.1	group_1739	468169	469437	-	COG4908	R;Uncharacterized conserved protein, contains a NRPS condensation	-	-
orf_438	AL591824.1	group_395	469575	470078	+	-	-	-	-
orf_439	AL591824.1	group_595	470122	472158	-	COG0768	DM;Cell division protein FtsI, peptidoglycan transpeptidase (Penicilli	-	-
orf_446	AL591824.1	group_171	481250	482701	+	COG0531	E;Serine transporter YbeC, amino acid:H+ symporter family;PotE;	-	Glutamate/gamma-aminobutyrate antiporter OS=Lactococcus lactis subsp. cremoris (strain MG1363) OX=416870 GN=gadC PE=2 SV=1
orf_448	AL591824.1	group_117	483166	483891	+	-	-	-	-
orf_454	AL591824.1	group_2068	489905	491500	+	COG2508	K;DNA-binding transcriptional regulator, PucR/PutR family;PucR;	-	-
orf_455	AL591824.1	group_2203	491634	492917	+	COG1457	F;Purine-cytosine permease or related protein;CodB	-	-
orf_483	AL591824.1	group_1832	519789	520268	+	COG3708	K;Predicted transcriptional regulator YdeE, contains AraC-type DNA-	-	-
orf_488	AL591824.1	group_1936	522628	523521	-	COG0583	K;DNA-binding transcriptional regulator, LysR family;LysR;	-	-
orf_489	AL591824.1	group_105	523719	525713	+	COG1902	C;2,4-dienoyl-CoA reductase or related NADH-dependent reductase,	-	-
orf_490	AL591824.1	group_94	525813	526688	+	COG0169	E;Shikimate 5-dehydrogenase;AroE;Aromatic amino acid biosynthesi	-	Shikimate dehydrogenase (NADP(+)) OS=Listeria monocytogenes serovar 1/2a (strain ATCC BAA-679 / EGD-e) OX=169963 GN=aroE PE=1 SV=1
orf_491	AL591824.1	group_1296	526754	527512	+	COG0710	E;3-dehydroquinate dehydratase;AroD;Aromatic amino acid biosynt	-	3-dehydroquinate dehydratase OS=Listeria monocytogenes serovar 1/2a (strain ATCC BAA-679 / EGD-e) OX=169963 GN=aroD PE=3 SV=1
orf_494	AL591824.1	group_2043	530515	531108	+	COG2755	DI;Lysophospholipase L1 or related esterase. Includes spore coat pro	-	-
orf_496	AL591824.1	group_107	532328	532552	+	COG4224	S;Uncharacterized conserved protein YnzC, UPF0291/DUF896 family;	-	UPF0291 protein LMOf2365_0526 OS=Listeria monocytogenes serotype 4b (strain F2365) OX=265669 GN=LMOf2365_0526 PE=3 SV=1
orf_498	AL591824.1	group_7	534307	534756	+	COG0698	G;Ribose 5-phosphate isomerase RpiB;RpiB;Pentose phosphate path	-	Ribose-5-phosphate isomerase B OS=Escherichia coli (strain K12) OX=83333 GN=rpiB PE=1 SV=2
orf_499	AL591824.1	group_132	534753	535421	+	COG0036	G;Pentose-5-phosphate-3-epimerase;Rpe;Pentose phosphate pathw	-	-
orf_500	AL591824.1	group_130	535428	536078	+	COG0176	G;Transaldolase/fructose-6-phosphate aldolase;TalA;Pentose phosp	-	-
orf_510	AL591824.1	group_260	544923	545501	+	-	-	-	-
orf_517	AL591824.1	group_2089	551467	552282	-	COG0406	G;Broad specificity phosphatase PhoE;PhoE;	-	-
orf_520	AL591824.1	group_1890	554624	555628	+	COG1940	GK;Sugar kinase of the NBD/HSP70 family, may contain an N-termina	-	-
orf_523	AL591824.1	group_1879	558037	558480	+	-	-	-	-
orf_524	AL591824.1	group_501	558526	560187	-	COG0659	P;Sulfate permease or related transporter, MFS superfamily;SUL1;	-	-
orf_532	AL591824.1	group_1835	570008	570838	+	COG2226	H;Ubiquinone/menaquinone biosynthesis C-methylase UbiE/MenG;	-	-
orf_537	AL591824.1	group_2051	575166	576437	+	COG0624	E;Acetylornithine deacetylase/Succinyl-diaminopimelate desuccinyla	-	N-carbamoyl-L-amino-acid hydrolase OS=Paenarthrobacter aurescens OX=43663 GN=hyuC PE=1 SV=1
orf_540	AL591824.1	group_1621	578886	580079	+	COG1680	V;CubicO group peptidase, beta-lactamase class C family;AmpC;	-	Putative penicillin-binding protein PbpX OS=Bacillus subtilis (strain 168) OX=224308 GN=pbpX PE=2 SV=1
orf_542	AL591824.1	group_2275	581150	581500	-	COG3731	G;Phosphotransferase system sorbitol-specific IIA component;SrlB;	-	-
orf_543	AL591824.1	group_2279	581519	582505	-	COG3732	G;Phosphotransferase system sorbitol-specific IIB component;SrlE	-	PTS system glucitol/sorbitol-specific EIIB component OS=Clostridium beijerinckii (strain ATCC 51743 / NCIMB 8052) OX=290402 GN=srlE PE=2 SV=1
orf_544	AL591824.1	group_2280	582526	583047	-	COG3730	G;Phosphotransferase system sorbitol-specific IIC component;SrlA	-	PTS system glucitol/sorbitol-specific EIIC component OS=Clostridium beijerinckii (strain ATCC 51743 / NCIMB 8052) OX=290402 GN=srlA PE=2 SV=1
orf_545	AL591824.1	group_2277	583072	583452	-	COG4578	K;DNA-binding transcriptional regulator of glucitol operon;GutM	-	-
orf_546	AL591824.1	group_2281	583507	584757	-	COG4091	E;Predicted homoserine dehydrogenase, contains C-terminal SAF do	-	-
orf_547	AL591824.1	group_2266	585015	585962	-	COG2390	K;DNA-binding transcriptional regulator LsrR, DeoR family;DeoR;	-	-
orf_557	AL591824.1	group_1337	595843	596538	+	COG0406	G;Broad specificity phosphatase PhoE;PhoE;	-	-
orf_559	AL591824.1	group_280	598020	598925	+	COG0598	P;Mg2+ and Co2+ transporter CorA;CorA;	-	-
orf_560	AL591824.1	group_1485	598968	600344	-	COG0334	E;Glutamate dehydrogenase/leucine dehydrogenase;GdhA;Glutamin	-	NADP-specific glutamate dehydrogenase OS=Corynebacterium glutamicum (strain ATCC 13032 / DSM 20300 / BCRC 11384 / JCM 1318 / LMG 3730 / NCIMB 10025) OX=196627 GN=gdh PE=1 SV=2
orf_562	AL591824.1	group_1369	601149	601466	-	COG0139	E;Phosphoribosyl-AMP cyclohydrolase;HisI1;Histidine biosynthesis	-	Phosphoribosyl-AMP cyclohydrolase OS=Listeria monocytogenes serovar 1/2a (strain ATCC BAA-679 / EGD-e) OX=169963 GN=hisI PE=3 SV=1
orf_565	AL591824.1	group_1297	602909	603535	-	COG0118	E;Imidazoleglycerol phosphate synthase glutamine amidotransferase	-	Imidazole glycerol phosphate synthase subunit HisH OS=Listeria monocytogenes serovar 1/2a (strain ATCC BAA-679 / EGD-e) OX=169963 GN=hisH PE=3 SV=1
orf_572	AL591824.1	group_2000	608557	609588	+	-	-	-	-
orf_577	AL591824.1	group_517	615353	615811	-	COG3610	S;Uncharacterized membrane protein YjjB, DUF3815 family;YjjB	-	-
orf_580	AL591824.1	group_1912	616967	617629	+	COG0400	R;Predicted esterase;YpfH	-	-
orf_587	AL591824.1	group_1833	626506	628971	+	-	-	-	-
orf_589	AL591824.1	group_1767	630629	631042	+	COG4852	S;Uncharacterized membrane protein	-	-
orf_590	AL591824.1	group_2040	631042	632811	+	COG1461	R;Predicted kinase related to dihydroxyacetone kinase;YloV	-	-
orf_591	AL591824.1	group_1873	632808	633581	+	COG3752	R;Steroid 5-alpha reductase family enzyme	-	-
orf_592	AL591824.1	group_1995	633709	634251	+	-	-	-	-
orf_593	AL591824.1	group_1551	634479	635279	+	COG2116	P;Formate/nitrite transporter FocA, FNT family;FocA;	-	-
orf_595	AL591824.1	group_1686	636440	637717	-	COG2873	E;O-acetylhomoserine/O-acetylserine sulfhydrylase, pyridoxal phosp	-	O-acetyl-L-homoserine sulfhydrylase OS=Thermotoga maritima (strain ATCC 43589 / DSM 3109 / JCM 10099 / NBRC 100826 / MSB8) OX=243274 GN=TM_0882 PE=1 SV=1
orf_596	AL591824.1	group_1773	638165	638692	+	COG3247	R;Acid resistance membrane protein HdeD, DUF308 family;HdeD;	-	-
orf_601	AL591824.1	group_1612	641208	642308	+	COG3595	S;Uncharacterized conserved protein YvlB, contains DUF4097 and D	-	-
orf_604	AL591824.1	group_2166	643400	643744	-	-	-	-	-
orf_607	AL591824.1	group_87	645866	647590	+	COG1132	V;ABC-type multidrug transport system, ATPase and permease comp	-	Uncharacterized ABC transporter ATP-binding protein Mb1304c OS=Mycobacterium bovis (strain ATCC BAA-935 / AF2122/97) OX=233413 GN=BQ2027_MB1304C PE=3 SV=1
orf_608	AL591824.1	group_1438	647587	649404	+	COG1132	V;ABC-type multidrug transport system, ATPase and permease comp	-	Uncharacterized ABC transporter ATP-binding protein TM_0288 OS=Thermotoga maritima (strain ATCC 43589 / DSM 3109 / JCM 10099 / NBRC 100826 / MSB8) OX=243274 GN=TM_0288 PE=1 SV=1
orf_612	AL591824.1	group_1847	652673	653044	+	COG1846	K;DNA-binding transcriptional regulator, MarR family;MarR;	-	-
orf_615	AL591824.1	group_2041	654608	654850	-	-	-	-	-
orf_616	AL591824.1	group_1865	654957	656708	+	COG4781	I;Membrane-anchored glycerophosphoryl diester phosphodiesterase	-	-
orf_617	AL591824.1	group_1761	656749	657243	-	-	-	-	-
orf_618	AL591824.1	group_1902	657323	658465	-	COG0515	T;Serine/threonine protein kinase;SPS1;	-	-
orf_620	AL591824.1	group_2123	659083	659475	+	COG5562	X;Prophage-encoded protein YbcV, DUF1398 family;YbcV	-	-
orf_622	AL591824.1	group_1927	660398	660676	+	-	-	-	-
orf_623	AL591824.1	group_277	660692	660958	+	-	-	-	-
orf_625	AL591824.1	group_1951	661470	662171	-	COG2755	DI;Lysophospholipase L1 or related esterase. Includes spore coat pro	-	-
orf_626	AL591824.1	group_1911	662257	663936	-	-	-	-	Uncharacterized membrane protein YkoS OS=Bacillus subtilis (strain 168) OX=224308 GN=ykoS PE=4 SV=1
orf_633	AL591824.1	group_6	673870	674178	+	COG1445	G;Phosphotransferase system fructose-specific component IIB;FrwB;	-	PTS system fructose-like EIIB component 2 OS=Shigella flexneri OX=623 GN=frwB PE=3 SV=1
orf_635	AL591824.1	group_1746	675563	676267	+	COG1011	H;FMN and 5-amino-6-(5-phospho-D-ribitylamino)uracil phosphatase	-	-
orf_641	AL591824.1	group_1743	679763	680296	+	-	-	-	-
orf_644	AL591824.1	group_117	683519	684247	+	-	-	-	-
orf_645	AL591824.1	group_130	684390	685040	+	COG0176	G;Transaldolase/fructose-6-phosphate aldolase;TalA;Pentose phosp	-	-
orf_649	AL591824.1	group_1928	689541	689825	-	-	-	-	-
orf_653	AL591824.1	group_1350	694317	694964	+	COG1802	K;DNA-binding transcriptional regulator, GntR family;GntR;	-	-
orf_654	AL591824.1	group_2122	694973	695509	-	-	-	-	-
orf_655	AL591824.1	group_374	695496	696419	-	COG5523	S;Uncharacterized membrane protein	-	-
orf_658	AL591824.1	group_1453	697634	698197	-	COG2322	R;Predicted membrane metallochaperone YozB (3H, Sco1 fusion), DU	-	Uncharacterized membrane protein YozB OS=Bacillus subtilis (strain 168) OX=224308 GN=yozB PE=4 SV=1
orf_659	AL591824.1	group_1896	698317	698733	+	-	-	-	-
orf_666	AL591824.1	group_1431	703128	703691	+	COG0110	R;Acetyltransferase, isoleucine patch superfamily;WbbJ	-	Maltose O-acetyltransferase OS=Escherichia coli (strain K12) OX=83333 GN=maa PE=1 SV=3
orf_674	AL591824.1	group_215	707612	708220	+	-	-	-	-
orf_675	AL591824.1	group_261	709134	709562	+	COG3152	S;Uncharacterized membrane protein YhaH, DUF805 family;yhaH	-	-
orf_678	AL591824.1	group_1432	711133	711435	+	-	-	-	-
orf_684	AL591824.1	group_1510	716450	717673	+	COG1419	N;Flagellar biosynthesis GTPase FlhF;FlhF;	-	-
orf_698	AL591824.1	group_1848	729026	730072	+	-	-	-	-
orf_714	AL591824.1	group_1576	742991	743401	+	COG1558	N;Flagellar basal body rod protein FlgC;FlgC	-	Flagellar basal-body rod protein FlgC OS=Bacillus subtilis (strain 168) OX=224308 GN=flgC PE=3 SV=1
orf_715	AL591824.1	group_1454	743418	743714	+	COG1677	N;Flagellar hook-basal body complex protein FliE;FliE	-	Flagellar hook-basal body complex protein FliE OS=Listeria monocytogenes serovar 1/2a (strain ATCC BAA-679 / EGD-e) OX=169963 GN=fliE PE=3 SV=1
orf_722	AL591824.1	group_2012	749985	750311	+	COG1695	K;DNA-binding transcriptional regulator, PadR family;PadR;	-	-
orf_723	AL591824.1	group_1957	750311	750634	+	COG4817	L;DNA-binding ferritin-like protein (Dps family);GINS;	-	-
orf_724	AL591824.1	group_1318	750680	751327	-	-	-	-	-
orf_728	AL591824.1	group_2057	756079	756291	-	-	-	-	-
orf_733	AL591824.1	group_2195	759972	760310	+	-	-	-	-
orf_735	AL591824.1	group_1590	761552	763468	+	COG4886	K;Leucine-rich repeat (LRR) protein;LRR;	-	-
orf_739	AL591824.1	group_7	766351	766797	+	COG0698	G;Ribose 5-phosphate isomerase RpiB;RpiB;Pentose phosphate path	-	Ribose-5-phosphate isomerase B OS=Escherichia coli (strain K12) OX=83333 GN=rpiB PE=1 SV=2
orf_741	AL591824.1	group_9	767766	769619	+	COG2190	G;Phosphotransferase system IIA component;NagE;	-	PTS system beta-glucoside-specific EIIBCA component OS=Alkalihalobacillus halodurans (strain ATCC BAA-125 / DSM 18197 / FERM 7344 / JCM 9153 / C-125) OX=272558 GN=bglP PE=3 SV=1
orf_743	AL591824.1	group_2044	771026	771685	-	COG0664	T;cAMP-binding domain of CRP or a regulatory subunit of cAMP-dep	-	-
orf_744	AL591824.1	group_148	771949	772326	+	COG1725	K;DNA-binding transcriptional regulator YhcF, GntR family;YhcF;	-	-
