# Supplementary material for: Comparative Genomics Reveal the Utilization Ability of Variable Carbohydrates as Key Genetic Features of Listeria Pathogens in Their Pathogenic Lifestyles
Source: Pathogens. 2022 Nov 28;11(12):1430. doi: 10.3390/pathogens11121430 (PMC9784484; doi:10.3390/pathogens11121430)
Supplement: Supplementary file 1 [file pathogens-11-01430-s001.zip › pathogens-1990707-supplementary/Supplymentary Tables S7.pdf]

Table S7. Characteristics of plasmids of Listeria strains.

Organism	Species	Accession number	length(bp)	GC content(%)	Note of plasmidFinder
Listeria_grayi_DSM_20601_525367	Listeria_grayi	GL538355.1	68061	37.15%	repA(pLGUG1)
Listeria_innocua_1642_str1	Listeria_innocua	CP045744.1	57553	36.04%	pLM330006(pLM33)
Listeria_innocua_1642_str2	Listeria_innocua	CP071156.1	85554	34.64%	M640p00130(J1776plasmid)
Listeria_innocua_1642_str3	Listeria_innocua	CP071158.1	83202	34.77%	M640p00130(J1776plasmid)
Listeria_innocua_Clip11262_272626	Listeria_innocua	AL592102.1	81905	35.52%	pli0070(pLI100)
Listeria_monocytogenes_08-5578_653938	Listeria_monocytogenes	CP001603.1	77054	36.59%	repA(pLM5578)
Listeria_monocytogenes_1639_str100	Listeria_monocytogenes	CP013725.1	86616	36.98%	repA(pLGUG1)
Listeria_monocytogenes_1639_str101	Listeria_monocytogenes	CP064844.1	86630	36.98%	repA(pLGUG1)
Listeria_monocytogenes_1639_str111	Listeria_monocytogenes	CP062125.1	50101	35.48%	pLM330006(pLM33)
Listeria_monocytogenes_1639_str11	Listeria_monocytogenes	CP028184.1	62214	36.53%	repA(pLM5578)
Listeria_monocytogenes_1639_str12	Listeria_monocytogenes	CP023753.1	58523	36.65%	pLM330006(pLM33)
Listeria_monocytogenes_1639_str130	Listeria_monocytogenes	CP045973.1	57553	36.04%	pLM330006(pLM33)
Listeria_monocytogenes_1639_str132	Listeria_monocytogenes	CP076670.1	53953	31.96%	pLMUKDL7
Listeria_monocytogenes_1639_str13	Listeria_monocytogenes	CP045750.1	51115	35.98%	pLM330006(pLM33)
Listeria_monocytogenes_1639_str147	Listeria_monocytogenes	CP060527.1	90421	37.57%	M643p00680(N1011Aplasmid)
Listeria_monocytogenes_1639_str14	Listeria_monocytogenes	CP014254.1	52687	35.05%	repA(pLGUG1)
Listeria_monocytogenes_1639_str156	Listeria_monocytogenes	CP041212.1	81588	37.57%	M643p00680(N1011Aplasmid)
Listeria_monocytogenes_1639_str15	Listeria_monocytogenes	CP011399.1	68224	35.67%	pLM330006(pLM33)
Listeria_monocytogenes_1639_str163	Listeria_monocytogenes	CP030102.1	35885	34.84%	pLM330006(pLM33)
Listeria_monocytogenes_1639_str16	Listeria_monocytogenes	CP019168.1	77105	36.59%	repA(pLM5578)
Listeria_monocytogenes_1639_str171	Listeria_monocytogenes	CP015985.1	57530	36.04%	pLM330006(pLM33)
Listeria_monocytogenes_1639_str192	Listeria_monocytogenes	CP090058.1	76010	34.09%	pLMIV
Listeria_monocytogenes_1639_str19	Listeria_monocytogenes	CP006595.1	57557	36.03%	pLM330006(pLM33)
Listeria_monocytogenes_1639_str20	Listeria_monocytogenes	CP041214.1	57553	36.04%	pLM330006(pLM33)
Listeria_monocytogenes_1639_str25	Listeria_monocytogenes	CP025260.1	63182	36.15%	pLM330006(pLM33)
Listeria_monocytogenes_1639_str26	Listeria_monocytogenes	CP025441.1	63182	36.15%	pLM330006(pLM33)
Listeria_monocytogenes_1639_str27	Listeria_monocytogenes	CP025439.1	63151	36.15%	pLM330006(pLM33)
Listeria_monocytogenes_1639_str29	Listeria_monocytogenes	CP025569.1	58517	36.65%	pLM330006(pLM33)
Listeria_monocytogenes_1639_str2	Listeria_monocytogenes	CP006611.1	148959	37.05%	pLM330006(pLM33)
Listeria_monocytogenes_1639_str36	Listeria_monocytogenes	CP025561.1	57553	36.03%	pLM330006(pLM33)
Listeria_monocytogenes_1639_str3	Listeria_monocytogenes	CP023051.1	61922	36.10%	pLM330006(pLM33)
Listeria_monocytogenes_1639_str40	Listeria_monocytogenes	CP025444.1	25550	36.53%	pLM330006(pLM33)
Listeria_monocytogenes_1639_str45	Listeria_monocytogenes	CP019166.1	77105	36.59%	repA(pLM5578)
Listeria_monocytogenes_1639_str46	Listeria_monocytogenes	CP045971.1	2776	36.82%	pLMIV
Listeria_monocytogenes_1639_str4	Listeria_monocytogenes	CP023053.1	61919	36.10%	pLM330006(pLM33)
Listeria_monocytogenes_1639_str51	Listeria_monocytogenes	CP025564.1	16073	35.16%	pLM330006(pLM33)
Listeria_monocytogenes_1639_str55	Listeria_monocytogenes	CP053629.1	55801	34.92%	M640p00130(J1776plasmid)
Listeria_monocytogenes_1639_str57	Listeria_monocytogenes	CP063384.1	4265	36.76%	pLIS3
Listeria_monocytogenes_1639_str5	Listeria_monocytogenes	CP022021.1	152337	36.98%	pLM330006(pLM33)
Listeria_monocytogenes_1639_str63	Listeria_monocytogenes	CP014251.1	55521	36.70%	pLM330006(pLM33)
Listeria_monocytogenes_1639_str66	Listeria_monocytogenes	CP038643.1	4392	37.41%	pLIS3
Listeria_monocytogenes_1639_str70	Listeria_monocytogenes	CP025566.1	15792	36.26%	pLM330006(pLM33)
Listeria_monocytogenes_1639_str71	Listeria_monocytogenes	CP020834.1	81588	37.57%	M643p00680(N1011Aplasmid)
Listeria_monocytogenes_1639_str73	Listeria_monocytogenes	CP025083.1	58524	36.64%	pLM330006(pLM33)
Listeria_monocytogenes_1639_str92	Listeria_monocytogenes	CP053631.1	55803	34.93%	M640p00130(J1776plasmid)
Listeria_monocytogenes_1639_str93	Listeria_monocytogenes	CP020829.1	67033	36.43%	repA(pLM5578)
Listeria_monocytogenes_1639_str94	Listeria_monocytogenes	CP006612.1	55804	34.92%	M640p00130(J1776plasmid)
Listeria_monocytogenes_1639_str95	Listeria_monocytogenes	CP006613.1	55804	34.92%	M640p00130(J1776plasmid)
Listeria_monocytogenes_1639_str96	Listeria_monocytogenes	CP006614.1	55803	34.92%	M640p00130(J1776plasmid)
Listeria_monocytogenes_1639_str99	Listeria_monocytogenes	CP053633.1	55798	34.92%	M640p00130(J1776plasmid)
Listeria_monocytogenes_6179_1288295	Listeria_monocytogenes	HG813250.1	62206	36.53%	repA(pLM5578)
Listeria_monocytogenes_FSL_J1-208_393119	Listeria_monocytogenes	CP062128.1	77825	34.02%	LMIVRS16815(pLMIV)
Listeria_monocytogenes_R479a_1437838	Listeria_monocytogenes	HG813248.1	86652	36.98%	repA(pLGUG1)
Listeria_monocytogenes_SLCC2372_932920	Listeria_monocytogenes	FR667691.1	50100	35.48%	pLM330006(pLM33)
Listeria_monocytogenes_SLCC2755_932919	Listeria_monocytogenes	FR667692.1	57780	36.04%	pLM330006(pLM33)
Listeria_seeligeri_1640_str1	Listeria_seeligeri	CP063072.1	60659	34.09%	pLIS5
Listeria_grayi_1641	Listeria_grayi	CABKNG010000004.1	68061	37.15%	repA(pLGUG1)
Listeria_innocua_1642_str4	Listeria_innocua	CP071180.1	93516	35.99%	pLM330006(pLM33)
Listeria_monocytogenes_1639_str41	Listeria_monocytogenes	CP090053.1	63873	37.23%	pLM330006(pLM33)
Listeria_grayi_1641_str1	Listeria_grayi	UGPG01000002.1	47088	35.46%	repA(pLGUG1)
